# Advanced liquid crystal-based switchable optical devices for light protection applications: principles and strategies

**DOI:** 10.1038/s41377-022-01032-y

**Published:** 2023-01-03

**Authors:** Ruicong Zhang, Zhibo Zhang, Jiecai Han, Lei Yang, Jiajun li, Zicheng Song, Tianyu Wang, Jiaqi Zhu

**Affiliations:** 1 National Key Laboratory of Science and Technology on Advanced Composites in Special Environments, Harbin Institute of Technology, Harbin, 150080 China; 2 Research Center of Analysis and Measurement, Harbin Institute of Technology, Harbin, 150080 China; 3 School of Energy Science & Engineering, Harbin Institute of Technology, Harbin, 150001 China; 4 Key Laboratory of Micro-systems and Micro-structures Manufacturing, Ministry of Education, Harbin, 150080 China

**Keywords:** Liquid crystals, Optoelectronic devices and components

## Abstract

With the development of optical technologies, transparent materials that provide protection from light have received considerable attention from scholars. As important channels for external light, windows play a vital role in the regulation of light in buildings, vehicles, and aircrafts. There is a need for windows with switchable optical properties to prevent or attenuate damage or interference to the human eye and light-sensitive instruments by inappropriate optical radiation. In this context, liquid crystals (LCs), owing to their rich responsiveness and unique optical properties, have been considered among the best candidates for advanced light protection materials. In this review, we provide an overview of advances in research on LC-based methods for protection against light. First, we introduce the characteristics of different light sources and their protection requirements. Second, we introduce several classes of light modulation principles based on liquid crystal materials and demonstrate the feasibility of using them for light protection. In addition, we discuss current light protection strategies based on liquid crystal materials for different applications. Finally, we discuss the problems and shortcomings of current strategies. We propose several suggestions for the development of liquid crystal materials in the field of light protection.

## Abstract (web summary)

With the development of optical technology, LCs with intelligent light protection function become more and more important. This paper discusses the research progress and challenges of LCs for light protection.

## Graphical abstract

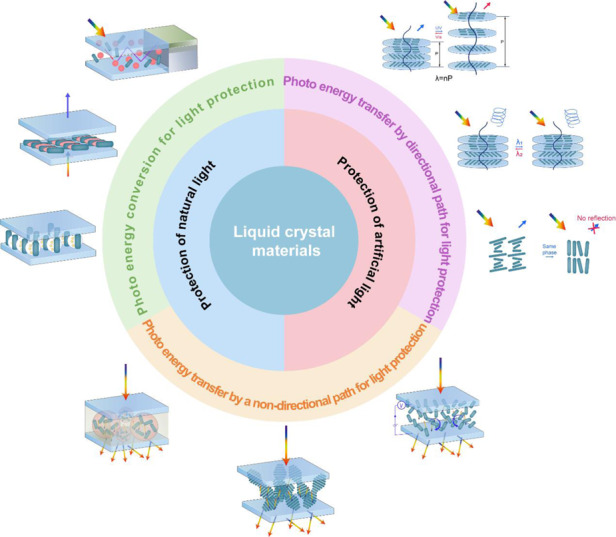

## Introduction

Human life is inextricably bound to light. Developments in science and technology have increased the popularity of artificial light and diversified the use of sunlight. Applications of artificial lighting are increasing at a rate of 6% per year^1–3^. Simultaneously, laser technology has developed rapidly over the last three decades, and its range of applicability has expanded^4^. However, inappropriate light radiation can be harmful to human health and wellbeing^5–12^. High-energy light can penetrate the lens of the human eye and reach the retina where it causes the retinal pigment epithelium to decline and thus affects vision. Additionally, for shorter wavelengths of visible light, the focal point does not fall at the center of the retina but rather slightly farther away. This creates a long-lasting state of tension within the eyeball, causing visual fatigue^13^. Furthermore, an increasing number of traffic accidents are caused by temporary “loss of vision” by drivers due to glare. In the United States alone, nearly 20 accidents occurred each day due to “laser dizziness” in 2017^7^. Considering that windows are the only means that allow outside light into a building or a vehicle, they play an important role in protection from light^14^. Consequently, it is important to design light protection for windows.

Throughout this review, the term “light protection” refers to means of preventing or weakening damage, discomfort or interference to people and photosensitive instruments by inappropriate light radiation. This can be accomplished by changing the intensity, energy, direction, and other characteristics of light passing through optical windows.

**Fig. 1:**
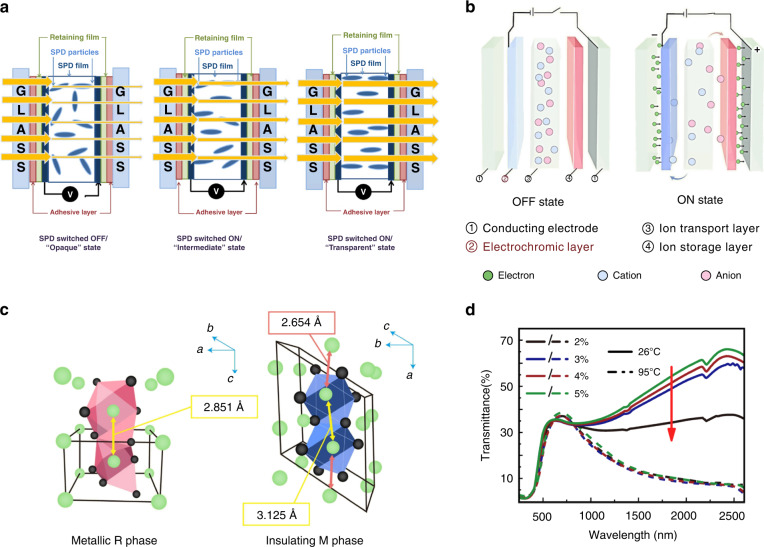
Diagram of the principles of different types of light protection technology. **a** Schematic of the dimming principle of SPD smart windows that require power to maintain the transparent state. Reproduced with permission from ref. ^208^. Copyright2019, Elsevier. **b** Schematic of the relationship between the morphology of EC materials and response time. **c** Schematic of the phase transition of VO_2_ crystals from a high-temperature metallic tetragonal phase R to a low-temperature insulating monoclinic phase M^209^. **d** Transmittance spectra of VO_2_ films prepared with different oxygen flow ratios. **c, d** Reproduced with permission from ref. ^210^. Copyright 2018, Elsevier

Several types of light protection technologies have been investigated in recent years, including suspended particle devices (SPDs)^15,16^, liquid crystal (LC) material systems^17–25^, electrochromic devices (EDs)^26–29^, photochromic devices^30–32^ and thermochromic devices^33–37^. As shown in Fig. 1, each technology has different characteristics in terms of working principles, operating conditions, and performance. SPD films contain suspended needle-like or rod-like halide particles that align perpendicular to the substrate and allow light to pass through when an AC current is applied; they orient randomly and absorb or reflect light due to Brownian motion when unenergized. Thus, SPD films have fast switching speed but require electricity to maintain transparency^16^. Current EC materials can be divided into inorganic materials (functional transition metal oxides), organic materials (including small organic molecules and conductive polymers), and metal complexes. Their color change is achieved through redox reactions. When a suitable voltage is applied, an EC material captures injected electrons and is reduced and ionized, and its optical properties are changed. This color change consumes small amounts of energy, but the rate of color change is limited^38,39^. When exposed to ultraviolet (UV) light, photochromic windows exhibit weak color switching for several minutes^31^. Most thermochromic devices are based on metal oxides, where the lattice structure of the material changes when the temperature reaches the phase transition temperature, and thus, the optical properties change. The high energy consumption of phase changing metal oxides as well as the limited visibility of thermochromic pigments restrict their widespread use^40^.

**Fig. 2:**
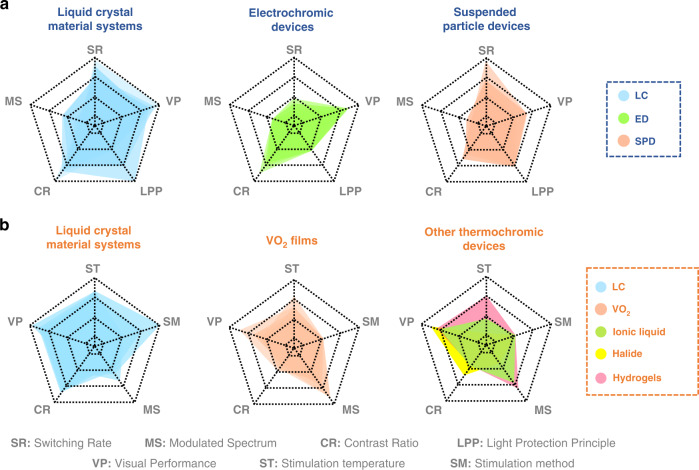
Comparison of the performance of light protection techniques for different stimulus types. **a** Optical performance and response time of different electronically controlled optical devices, where different colors indicate different material types^15,19,171,195–197,200,203,204,207,211–215^. **b** Optical performance and stimulus temperature of different temperature-controlled optical devices, where different colors indicate different material types^171,178,180–182,184,216–227^

As shown in Fig. 2, compared to conventional electronically controlled color-changing materials, LC materials exhibit superiority in terms of response time and protective bandwidth. Compared to conventional thermochromic materials, LC materials have great advantages in terms of stimulation temperature and visible light transmittance.

The LC phase is between crystalline solid and isotropic liquid states. This fascinating thermodynamically stable form of matter exhibits both the ordered nature of crystals and the fluidity of liquids. There are two types of LCs: thermotropic and lyotropic. LC phases form in thermotropic LCs because of temperature changes. Thermotropic LC phases are observed in rod-like, disc-like, and bent-core compounds. Rod-like thermotropic LCs are one of the most widely used materials in commercial applications^41,42^. The use of LCs in photo-regulation applications is assisted by their unique mix of reactivity to their environmental and stunning optical features, which allow immediate viewing of their response^41,43^. Unless otherwise noted, throughout this paper, the term “LC” refers to rod-like thermotropic LCs.

The characteristics of light sources and their protection requirements in different light environments are discussed in the second part of this review. In the third part, principles of light protection using liquid crystal materials are described in detail. In the Section ‘Strategies for LC-based light protection’, light protection strategies based on liquid crystal materials that are suitable for individualized protection requirements in different light environments are discussed. Finally, opportunities for the development of light protection by liquid crystal materials are proposed.

## Specific light environment confronted by LC-based light protection

External and internal light sources create an interior light environment. Natural and artificial light enter a room through windows. Light sources vary in frequency, energy, and irradiation area. They also pose different risks^44^. Thus, it is necessary to discuss their characteristics to determine the best protection.

### Protection against natural light

Natural light refers to light that is present in nature, such as sunlight, fire, and lightning flashes. Sunlight is the predominant source of natural light, and therefore, this section primarily discusses the light environment created by sunlight and the associated protection requirements.

**Fig. 3:**
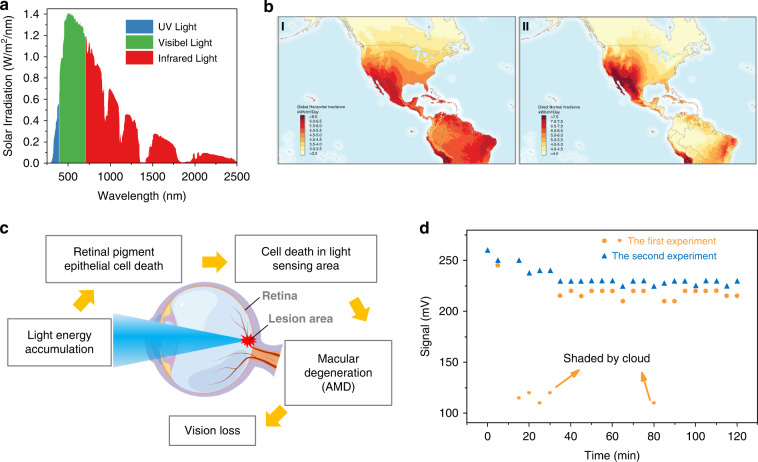
Characteristics of sunlight and its hazards. **a** Wavelength distribution of sunlight. Reproduced with permission from ref. ^228^. Copyright 2017, Wiley Online Library. **b** Global horizontal irradiance (I) and direct normal irradiance (II) for the US and Central America. Reproduced with permission from ref. ^48^. Copyright 2018, Elsevier. **c** Possible effects of short-wavelength, high-energy light on vision. **d** Changes in the detection signal before and after exposure to sunlight

As shown in Fig. 3a, the wavelength range of sunlight reaching Earth is from 250 to 2500 nm, which essentially covers from the UV to the near-infrared band. In sunlight, more than 90% of the energy is concentrated in the visible and infrared bands^45^. Earth receives approximately 1373 W/m^2^ of solar radiation, and the intensity of solar radiation at its surface depends on the altitude and angle of the Sun, distance between the Sun and Earth, and duration of sunshine^46^.

Due to the rotation and revolution of the Earth, the duration of sunshine differs regionally^47^. The radiation from the Sun on the ground plane consists of direct radiation and diffuse radiation. Direct radiation refers to radiation that comes directly from the Sun and does not change direction, whereas diffuse radiation refers to solar radiation that changes direction after being reflected and scattered by the atmosphere^44^. Diffuse reflection changes not only the direction but also the intensity and spectral distribution of radiation. Solar irradiance varies sinusoidally throughout the day and fluctuates irregularly under the influence of clouds^48^. In summary, the irradiation of sunlight has the characteristics of wide spectral range, large variation in irradiation angle, and wide area of irradiation.

The absorption of sunlight provides significant amounts of energy used by industries and daily life; however, it also has negative effects. Due to the conversion of heat by photoenergy, light exposure can cause an increase in the temperature of biological tissues. At increase of at least 10 °C in temperature induces denaturation of many proteins in the retina, which results in photothermal damage^49^. In addition, certain specific wavelengths of light radiation can cause chemical reactions in organisms, and photochemical damage can occur. Photochemical damage occurs when the retina is exposed to incident radiation in the high-energy portion of the visible spectrum^50^. Several recent studies showed that photochemical damage occurred when the retina was exposed to incident radiation at high-energy wavelengths in the visible spectrum, which resulted in impaired vision and potentially serious ocular disease^13,50^. At high latitudes or at sunrise and sunset, the altitude angle of the sun decreases, and consequently, it becomes easier for sunlight to enter the human eye obliquely, thus increasing the risks of accidents^51^. In addition, due to the wide irradiation area and various diffuse reflections, strong sunlight can affect the normal operation of light protection devices. That is, sunlight produces a large amount of noise and overwhelms the measurement signal, leading to device failure^52–56^.

The wide distribution of sunlight necessitates special protection. Because insolation intensity varies over time, protective material must be adjustable^57^. The changing angle of incident sunlight throughout the day necessitates wide angle protective materials. Additionally, simultaneous protection from visible light and infrared radiation is required with broadband shielding over the 390–2500 nm range.

Additionally, protective materials must function throughout the day. The accumulation of solar energy raises the temperature of protective materials. This poses a serious threat to the properties of the materials. Additionally, for use during many hours of operation, protective materials must be energy efficient.

### Protection from artificial light

Artificial light sources have aided human scientific and technological advancements. With the widespread use of artificial light sources, their effects on human life and health have increased. Lasers and other high-intensity lights are common sources of concern.

**Fig. 4:**
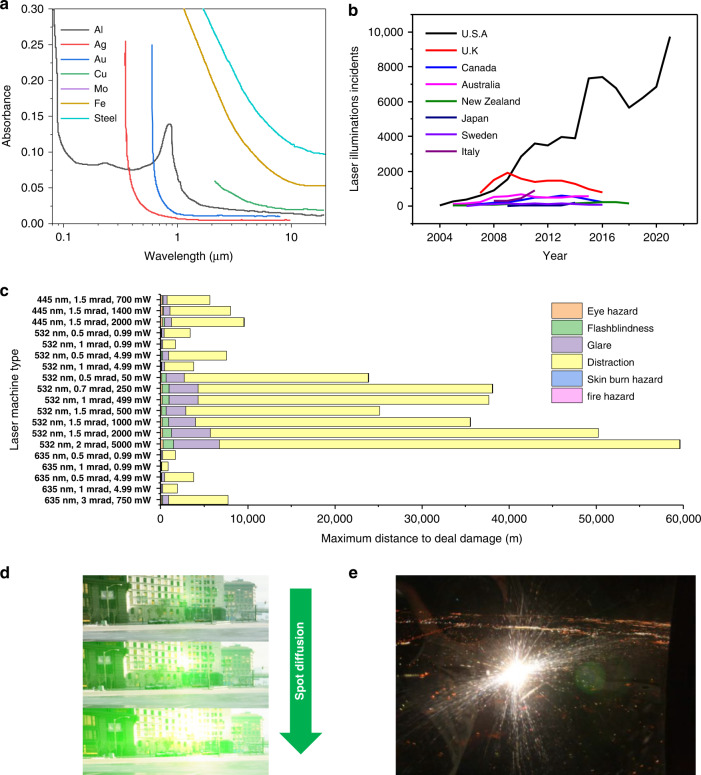
Characteristics of artificial light and its hazards. **a** Common lasers and their luminous bands. **b** Laser illuminations of airplanes on a global and yearly basis. **c** Relationship between the damage caused by different types of lasers and distance. **d** Visual disturbance caused by glare fields of different radii. **e** High-intensity lighting technology that creates dazzling light. Reproduced with permission from ref. ^63^. Copyright 2009, TNO

Lasers have played a groundbreaking role in many scientific and technological fields^58,59^. Laser light is produced by transitions of electrons from excited to ground states^60^. Instruments have been developed using different laser mediums, which lead to different wavelength distributions. Lasers primarily include single-frequency and multifrequency lasers^61^, as shown in Fig. 4a.

However, potential threats resulting from the use of lasers have increasingly attracted attention. Liu et al.^5^ presented the pathological effects of lasers at different wavelengths. The power of a laser can be as great as millions of watts per second, which can severely affect human vision (cause blindness) and destroy optical sensors. As a result, the use of lasers in weapons is frequently reported. Aside from irreversible eye damage, certain types of laser illumination may create transitory visual effects, such as distraction, confusion, or discomfort, and create potentially dangerous conditions for pilots of airplanes or drivers of vehicles^62,63^. According to statistics from the Federal Aviation Administration (FAA), pilots in the United States reported observing laser light 9,723 times in 2021, as shown in Fig. 4b. This was a 42% increase over the 6,852 complaints received in 2020^62^.

As shown in Fig. 4c, several researchers systematically studied and calculated the possible forms of harm to people caused by different types of lasers (wavelength, beam propagation, and power) at various distances. Based on the likelihood of causing injury, various organizations, such as the American National Standards Institute, the Center for Devices and Radiological Health and the International Electrotechnical Commission, have classified lasers into four hazard classes^64–66^. These data show that in the range of visible wavelengths, green lasers cause the strongest interference in the visual field, while with increasing distance, laser damage to people mainly produces transient visual effects and causes distraction, i.e., the first three levels of laser hazards. The use of optical protection from high power lasers that may even cause fires is beyond the scope of this paper and therefore is not discussed further. As the light spot expands, the interference to the visual field of a driver gradually increases, as shown in Fig. 4d.

Owing to the high power, narrow band, and fast response of lasers, researchers have proposed different methods of protection from different types of lasers^67^. As laser protection theory has continuously improved, requirements for protective material systems have become more specific. First, the protective band must cover the preset laser band range. Second, the protective effect should be adequately strong to provide protection from high-energy lasers. Finally, the materials must respond quickly to manage exposure to bursts of laser light.

In addition, high power lasers can increase the temperature of protective materials, which is a crucial factor in their failure. High-intensity light sources, such as sky lanterns and spotlights, are increasingly used in daily life, which results in a series of problems^63^. Currently, the technologies that are primarily used for high-intensity lighting are carbon arc lamps, enclosed arc lamps, and high-intensity discharge lamps. The lighting characteristics of these technologies are listed in Table 1.

**Table 1:** Lighting characteristics/parameters of various high-intensity lights/technologies^63^

Searchlight	Diameter searchlight (m)	Illuminance at 100 m (lx)	Luminous intensity (cd)	Luminance (cd/m²)	Beam width (deg)
Arclight	1.524	5200	5.2 × 10^7^	2.87 × 10^7^	0.93
Dominator	1.524	14000	1.4 × 10^8^	7.72 × 10^7^	2.93
Prolight	0.406	727	7.27 × 10^6^	5.60 × 10^7^	1.68
Xenon	0.406	7013	7.01 × 10^7^	5.41 × 10^8^	1.72
Skytracker	0.406	3320	3.32 × 10^7^	2.56 × 10^8^	2.67
Carbon arc	1.524	12900	1.29 × 10^8^	4.11 × 10^9^	2.85

The most common problem is dazzling by high-intensity lighting, as shown in Fig. 4e. Dazzling refers to visual conditions that can reduce the visibility of objects owing to an inappropriate brightness distribution or extreme brightness contrast in space or time. Dazzling originates from several sources, and therefore, its wavelength covers a wide spectral range. In addition, the duration of dazzling is short, and the intensity is relatively high intensity, which causes discomfort to personnel in buildings or vehicles and results in severe consequences.

The primary requirement for protection against dazzling by light is fast response speed that is comparable to that of lasers. Additionally, high-intensity lighting has a wider spectral range than lasers, including the entire visible light band, and this requires protective equipment that has a wider range of spectral adjustment.

Currently, there are several types of light protection devices based on LC materials, and each has different effects. Smart optical devices using LC materials can realize multiband protection, light intensity regulation, and fast responses to multiple stimuli, among other functions. Consequently, LC materials^68^ have been widely used in light protection^69^. The next section discusses the application of LCs in the field of light protection from the perspective of principles of protection.

## Principles of LC-based light protection

LC-based materials for light protection should correspond to the spectral distribution, intensity, and angle of incidence for specific surroundings. In this section, LC-based materials are classified based on different light protection mechanisms. We focus primarily on the conversion and transfer path of photoenergy. In Section ‘Absorption-based light protection’, we introduce LC-based materials with light energy conversion as the working mechanism. These materials apply the principle of light absorption to provide protection. In Section ‘Reflection-based light protection’, we demonstrate photoenergy transfer by the directional path of LC-based materials for light protection. Here, the focus is on reflection by LC-based materials. In Section ‘Scattering-based light protection’, we discuss protection by photoenergy transfer by nondirectional paths in LC-based materials based on the scattering characteristics of LC-based materials. Finally, we summarize the properties of LC-based materials based on different light protection mechanisms in complex environments.

### Absorption-based light protection

**Fig. 5:**
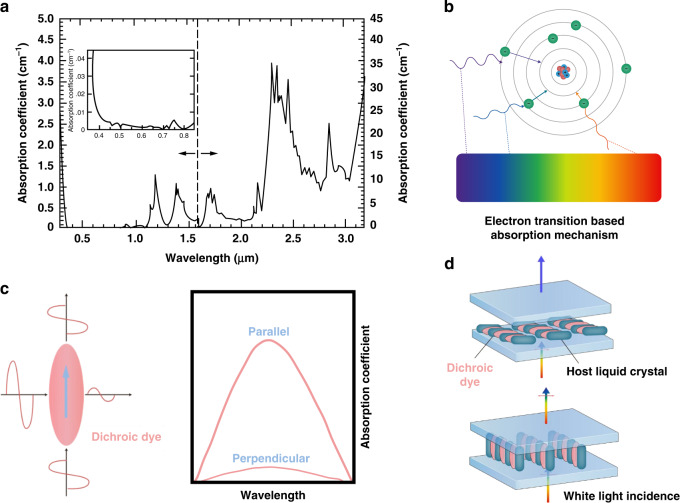
Absorption based on electron transition. **a** Absorption spectra of NLC 5CB. Reproduced with permission from ref. ^229^. Copyright 1998, AIP Publishing. **b** Electron transition absorption mechanism of molecules. **c** Optical properties of positive (p-type) dichroic dyes. **d** Schematic of the guest-host effect

The active form of photoenergy conversion involves converting photoenergy into heat energy, chemical energy, and/or other forms of energy through absorption by LC-based materials. As shown in Fig. 5a, the absorption bands of the thermally induced rod-like LC molecules are concentrated in ultraviolet (<380 nm) and long wavelength infrared light (>3 µm)^70^. In recent years, good light protection was obtained by mixing functional materials with visible and near-infrared absorption properties and LCs.

#### Electron transition-based absorption

Each molecule has its own spectral transitions. Light radiation is absorbed by a molecule when it resonates with electronic transitions, as shown in Fig. 5b. The transitions of valence electrons in several organic molecules can absorb light in the wavelength range of 200–1000 nm, which lies in the UV–visible region^71^. Therefore, the most straightforward approach for obtaining light absorption is to dope the host LC with organic dyes.

Light absorption by a dye molecule is directional or dichroic. There are two types of dichroic dyes, those with positive and negative absorption characteristics. Positive (p-type) dichroic molecules absorb polarized light best along the long axis and almost no light polarized perpendicular to the long axis, as shown in Fig. 5c. Negative (n-type) dichroic dyes have the opposite behavior^72^.

Meanwhile, dichroic dye molecules have rod-like shapes that are similar to those of LC molecules. When dichroic dyes are dissolved in an LC host, the LCs act not only as solvents but also as agents of structural alignment. By adjusting the LCs, the orientation of the dye molecules can be changed, as shown in Fig. 5d; this is known as the guest-host effect. Since the first LC display based on the guest-host effect was reported by Heilmeier et al. in 1968, this type of device attracted widespread attention and was gradually incorporated into light protection devices by researchers^73^.

**Fig. 6:**
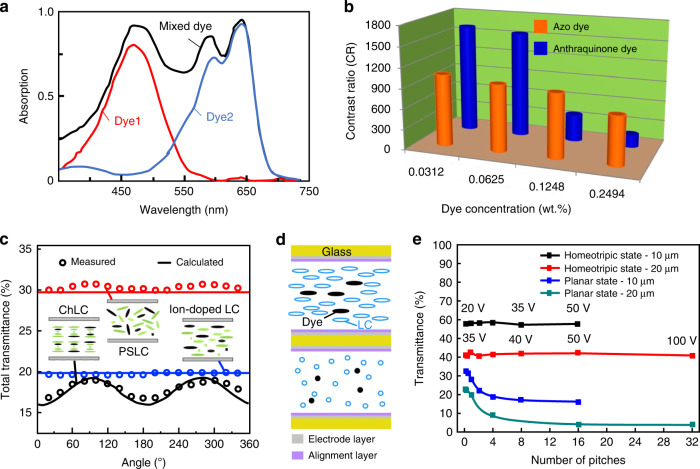
Switchable optical devices doped with dichroic dyes. **a** Absorption spectra of mixed and single dyes. **b** Switching contrast coefficient (CR) of PDLC doped with different concentrations of azo/anthraquinone dichroic dyes. Reproduced with permission from ref. ^82^. Copyright 2018, Elsevier. **c** Cross-polarization structure of double-layer cells. Reproduced with permission from ref. ^86^. Copyright 2018, Elsevier. **d** Transmittance of dye-doped LC devices with different alignments where doping had the strongest light absorption properties in Ch-LC. Adapted with permission from ref. ^87^. Copyright 2019, Optica Publishing Group. **e** The measured and calculated transmittances in the planar state vs. the number of pitches. Reproduced with permission from ref. ^88^. Copyright 2013, Optica Publishing Group

While many dichroic dyes were developed, none were synthesized with uniform absorption across the entire visible light spectrum, which limited the use of the guest-host effect of LC materials in light protection applications. Thus, multiple dichroic dyes were mixed to formulate guest-host LCs with broad absorption bands, as shown in Fig. 6a, and thereby achieve absorption across almost the entire visible range of the spectrum^74–77^. In addition, the solubility of dichroic dyes in LCs and the order parameter (S) greatly affect the performance of guest-host light protection devices. Both factors determine the absorbance and contrast of the device^78–85^, as shown in Fig. 6b. However, this type of research primarily involves the structural design of dye molecules, which is not discussed in this paper.

Additionally, dye molecules absorb only polarized light, which makes them inefficient light absorbers. Light is strongly absorbed when its polarization is parallel to the long axis of a dye molecule. When light is oriented perpendicular to the long axis of a dye molecule, it is weakly absorbed^82^. One method to improve the absorption efficiency is to design a double-layer structure wherein the LC molecules of the upper and lower layers are arranged perpendicular to each other, as shown in Fig. 6c. LC molecules in this arrangement absorb light with all polarizations^86^. Another method is to change the alignment of liquid crystal molecules. Huh^87^ et al. investigated the angular dependence of dye transmittance in planar Ch-LC, PSLC, and ion-doped LCs, as shown in Fig. 6d. A twisted structure in the planar state of the Ch-LC cell resulted in the least transmittance. Further studies found that considering the wave-guide effect, the transmission in the planar state decreases as the number of pitches in the Ch-LC cells increased for the same cell gap^88^, as shown in Fig. 6e. However, this required the addition of more chiral dopants, which led to an increase in the driving voltage.

#### Surface plasmon resonance-based absorption

**Fig. 7:**
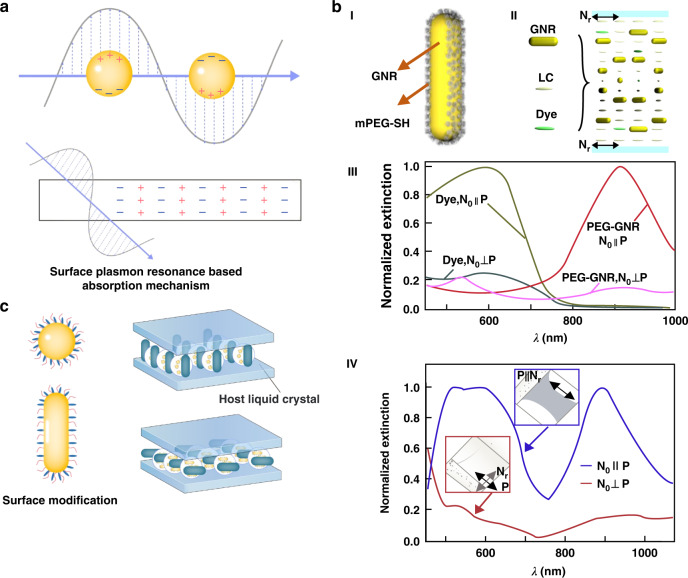
Switchable optical devices doped with nanoparticles. **a** Absorption mechanism of surface plasmon resonance. **b** Through surface modification, the alignment of gold nanorods and liquid crystals was induced to achieve broadband absorption modulation: (I) surface modification of GNRs, (II) gold nanorods and dichroic dyes aligned with liquid crystals, (III) gold nanorods and dichroism absorption spectra of dyes, and (IV) absorption spectra of liquid crystal devices codoped with gold nanorods and dichroic dyes. Reproduced with permission from ref. ^103^. Copyright 2018, Optica Publishing Group. **c** Surface modification of nanoparticles with different shapes and their applications in LC materials

As shown in Fig. 7a, nanoparticles (NPs) exhibit surface plasmon resonance at specific wavelengths with strong absorption effects because an evanescent wave forms in the optically sparse medium and a specific plasma wave forms in the medium when light is totally reflected by the surfaces of the NPs. When two waves meet, resonance occurs. When the evanescent wave resonates with the surface plasmon wave, photons gain energy. The surface plasmon wave absorbs most of the incident light energy, which reduces the reflected light energy^89–91^. The idea of doping functional particles has been a hot topic in liquid crystal research since 1970, when F. Brochard and PG de Gennes modified the properties of liquid crystals by mixing them with submicron magnetic particles^92^. In recent years, with the continuous development of nanotechnology, liquid crystal light protection devices doped with nanoparticles have attracted attention.

The effect of nanoparticles on LCs were found to depend on several factors, such as material composition, shape, size, concentration, modification form and liquid crystal type^93^. Inorganic semiconductor NPs displayed excellent UV and infrared shielding properties due to their localized surface plasmon resonance (LSPR). This suggested new possibilities for plasmonic light manipulation by altering the material composition of doped semiconductors^94–96^. In addition, anisotropic gold nanorods (GNRs), important nanostructures with remarkable optical properties, were investigated^97^. The surface plasmon resonance of anisotropic GNRs enabled them to concentrate and manipulate light based on their size, shape, and proximity. Similar to the guest–host effect described above, thiol molecules were introduced on the surface of GNRs so that they could be arranged with LCs, as shown in Fig. 7b^98–101^. Electrical switching of both visible and infrared light transmission using an LC film containing a dichroic dye and GNRs was demonstrated^102,103^. As shown in Fig. 7b, doped dye molecules and GNRs spontaneously aligned along or across the director field of the host LC. The composite film absorbed light from visible to near-infrared and switched quickly at low voltage.

However, the LC/monomer mixture is usually viewed as a solvent with high polarity. This drastic change in the polarity of the medium destabilizes the dispersion and leads to severe aggregation. In studies, this not only resulted in the loss of transmittance in the visible region but also detrimentally affected the near-infrared shielding ability of NPs owing to the significant reduction in the surface electron density in large aggregates^104–106^. As shown in Fig. 7c, modification of NPs by functionalization or addition of solvents prevented aggregation effects^93^. For example, ITO NPs were encapsulated with an insulating silica barrier to form a core/shell structure to prevent them from aggregating^21^ (Fig. 7d). However, this approach had an impact on the absorption spectra of NPs due to the surface chemistry-dependent modulation of the LSPR of NPs^107^. Additionally, the dispersion of NPs in LCs significantly affected the various physical and electrical properties of the host LC material^108^. Therefore, for devices that rely on the electro-optical response of liquid crystal molecules, it is necessary to consider the multiple effects of NP doping.

The advantage of the photoenergy conversion method of LC-based light protection materials is that they can completely consume light by converting photoenergy into other forms of energy. Therefore, this method provides a good light protection effect; however, new forms of energy may cause a series of problems and failure of the material.

### Reflection-based light protection

**Fig. 8:**
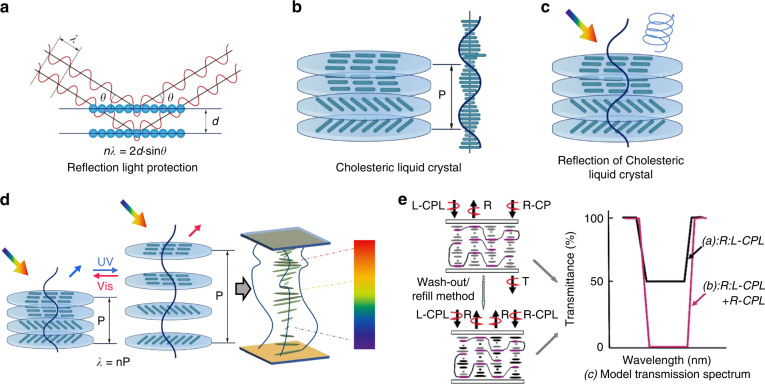
Photoenergy transfer by the reflected path of Ch-LCs. **a** Bragg reflection principle. **b** Helical superstructure of CLC molecules. **c** Selective reflection of Ch-LCs. **d** Construction of helical gradients with polymer networks to broaden the reflection bands. **e** Schematic of the method of breaking the reflection limit. Reproduced with permission from ref. ^127^. Copyright 2009, American Chemical Society

Cholesteryl liquid crystals (Ch-LCs), the most typical reflective material, were discovered in 1888 in the form of cholesteryl esters^109,110^. They spontaneously exhibit helical superstructure and can selectively reflect up to 50% of unpolarized natural light. They are generally developed by introducing chiral molecules into achiral nematic LCs. Chiral dopants generate LC organization, wherein successive layers of nematic LCs are displaced by a small rotation in the molecular director with respect to neighboring layers. The resulting “twist” may be either right- or left-handed. In the cholesteric phase, the molecules possess a helical distribution with a periodic helical structure. According to Bragg’s law shown in Fig. 8a, the central reflection band of Ch-LC is determined by the pitch (P), as shown in Fig. 8b, average refractive index (n_avg_) of the material, and incident angle of the light. The pitch is defined as the distance over which the director field rotates by one full turn (360°). The pitch (P) of the Ch-LC depends on the concentration (C) and helical twisting power (HTP) of the chiral dopants (Eq. 1).

1 $$P = \frac{1}{{C \times {\rm{HTP}}}}$$

The bandwidth of the light reflected by the Ch-LC is determined by the difference between the extraordinary (*n*_*e*_) and ordinary (*n*_*o*_) refractive indices as well as the pitch of the host LC (Eq. 2), which is normally limited to between approximately 75 and 100 nm^111^.

2 $${\Delta}\lambda = \left( {n_e - n_o} \right) \times P$$

The maximum reflection produced by the cholesteric reflector layer is limited to 50% of the unpolarized natural light because Ch-LCs can only reflect circularly polarized light whose polarization matches that of the helix. For example, right-handed cholesteric light reflects only right-circularly polarized light, as shown in Fig. 8c. Both left-circularly polarized light and light outside the cholesteric reflection bandwidth are unaffected by the LC matrix and are transmitted normally^112^. It is necessary to overcome these limitations in the fabrication of polarization-independent devices, such as light protection devices^105^.

To broaden the bandwidth around the central reflected wavelength, the most straightforward approach is to stack single Ch-LC layers with different pitch lengths. Both Hideo Takezoe et al. and Shin-Tson Wu et al. constructed multi-CLC systems and introduced phase-modulated materials to overcome the reflection limit^113,114^. In addition to this multilayer stacking approach, Mitov^115–118^ et al. used polymer-based composites to construct pitch gradients in a single Ch-LC layer, as shown in Fig. 8d. They coated two glass plates with cholesteric films of different pitches and used thermal diffusion between the layers to create a lateral concentration gradient of the chiral agent in the LC unit, which resulted in a gradient of spacing in the Ch-LC layer and an expanded reflection band. In addition, several studies used UV-induced reactive polymer monomers to generate UV intensity gradients in films by adding UV-absorbing dyes to blends of raw materials to alter the polymerization rate within the films and thus construct spacing gradients in the Ch-LC layers^119–125^. Zhou^126^ et al. added a DC bias during the photopolymerization process. This bandwidth broadening was attributed to a frozen pitch gradient that resulted from an in situ electric-field-assisted dynamic ion-dragging effect, which led to the formation of a pitch gradient along the direction of the electrical field.

To break the reflection limit, Guo^127^ et al. employed a “wash-out/refill” technique to use polymers to stabilize LC films with two-handed circularly polarized reflection bands, as shown in Fig. 8e. In contrast, Mitov^117^ et al. used thermally induced helicity-inverted Ch-LCs to prepare LC films stabilized with polymers; the maximum reflectivity of the films was 72%.

In conclusion, by using polymer-liquid crystal (P-LC) materials, the inherent defects of Ch-LC can be greatly improved, and the application of this approach in the field of light protection can be expanded.

According to previous research, photoenergy transfer by the reflected path of LC-based materials adjusted the propagation path of light through reflection to provide light protection. This method had no secondary negative consequences of energy conversion, and the light attenuation rate was high. However, the reflective property depended on the structure of the material, had inherent angular dependence and required films with high dimensional accuracy.

### Scattering-based light protection

**Fig. 9:**
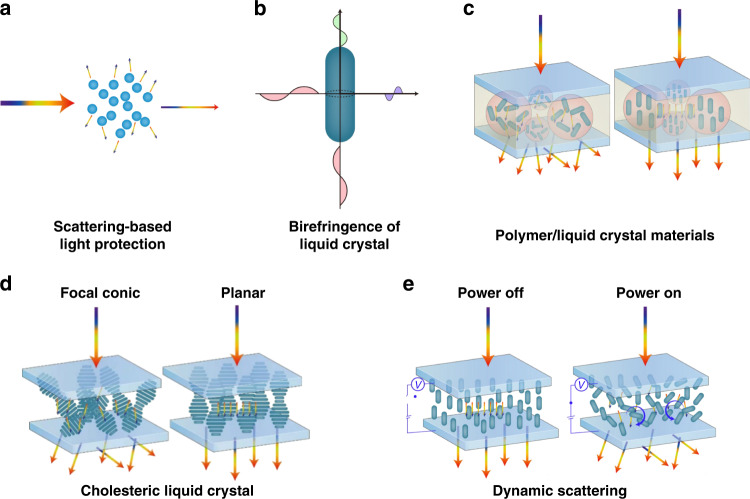
Scattering-based light protection principles. **a** Scattering principle for light protection. **b** Birefringence of liquid crystal molecules. **c** Scattering properties of polymer-liquid crystals (P-LCs). **d** Scattering properties of CLC. **e** Dynamic LC scattering properties

As shown in Fig. 9a, propagation of light in materials with refractive index inhomogeneities results in scattering. Multiple scattering events cause photoenergy to deviate from its original propagation direction and spread out for transmission. As shown in Fig. 9b, LCs with controllable birefringence may provide an effective means for adjusting the refractive index of the medium. Depending on the refractive index adjustment method, protective materials can be divided into two categories: static and dynamic scattering materials.

#### Static scattering

Polymer-liquid crystal (P-LC) materials were used for fabricating commonly commercialized haze-switchable LC smart windows^128^. The P-LC protective system was divided into polymer-dispersed liquid crystal (PDLC) and polymer-stabilized liquid crystal (PSLC) systems depending on the polymer content in the system^128^. In the PDLC system with high polymer content, the LC was dispersed within the polymer matrix in the form of small microdroplets. The LC molecules within the microdroplets were freely oriented. Their refractive indices did not match the refractive indices of the matrix and presented a scattering state. The direction of the optical axis of the liquid crystal microdroplet was adjusted by applying an electric field. When the refractive indices of both matched, a transparent state formed, as shown in Fig. 9c. In the 1980s, J. W. Doane et al. first prepared PDLC films by a phase separation method^129^. This technique was extensively utilized in both academic research and industrial development of polymer-liquid crystal (P-LC) materials. For PSLC systems with low polymer contents, the polymer bound some of the LC molecules, and its optical axis direction was different from that of the LC molecules in other regions, which resulted in a refractive index mismatch and a scattering state^130^. In addition, cholesteric LCs exhibited metastable scattering textures and were transformed into a focal conical domain by external stimuli, as shown in Fig. 9d^130,131^. Therefore, Ch-LCs are often used in P-LC systems to enhance this effect.

**Fig. 10:**
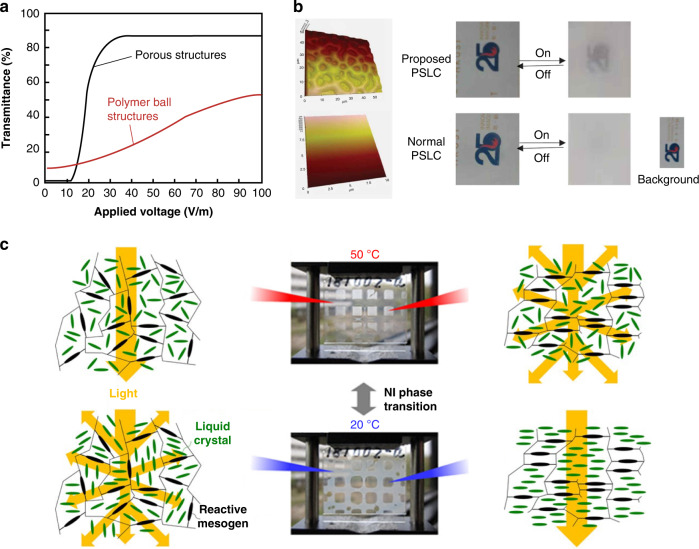
Switchable optical devices based on polymer-liquid crystal materials. **a** The regulation of electro-optical properties by the morphologies of polymer networks in PDLCs. **b** Effects of power on haze states PSLC. The results in the upper and lower rows were from inhomogeneous and smooth films with vertical alignment, respectively. Reproduced with permission from ref. ^142^. Copyright 2019, Taylor & Francis Group. **c** Demonstration of the optical clarity of a grid-patterned sample with normal- and reverse-mode thermoresponsive switching ability. Reproduced with permission from ref. ^144^. Copyright 2019, American Chemical Society

Optimizing the performance of LC/polymer materials has been the focus of research in recent years. Several key aspects used to evaluate the electro-optical performance of LC/polymer materials were the driving performance (threshold voltage V_th_ and saturation voltage V_sat_), the response time (rise time τ_on_ and decay time τ_off_) and the optical contrast (ratio of transmittance in the on-state and off-state of the film). Theoretically, the driving performance of PDLC films and the response time can be predicted according to Eqs. 3–6^132–136^.

3 $$V_{{\rm{th}}} \approx \frac{d}{R} \times \left[ {\frac{{K(\omega ^2 - 1)}}{{\varepsilon _0{\Delta}\varepsilon }}} \right]^{\frac{1}{2}}$$

4 $$V_{{\rm{sat}}} \approx \frac{d}{R} \times \left( {\omega ^2 - 1} \right)^{\frac{1}{2}} \times \frac{{4\pi K}}{{{\Delta}\varepsilon }}$$

5 $$\tau _{{\rm{on}}} \approx \frac{\gamma }{{{\Delta}\varepsilon V^2 - \frac{{K\left( {l^2 - 1} \right)}}{{R^2}}}}$$

6 $$\tau _{{{{\mathrm{off}}}}} \approx \frac{{R^2\gamma }}{{K\left( {l^2 - 1} \right)}}$$

where *V* is the applied electric field, while *d, R, K, ω, ε*_0_, Δ*ε, l*, and *γ* represent the film gap, droplet radius of the LC, elastic constant, aspect ratio, vacuum dielectric constant, dielectric anisotropy, shape anisotropy, and rotational viscosity constant, respectively. The addition of NPs changed the refractive index of the dielectric, the dielectric constant, and the anchoring properties of the liquid crystal, and thus, effectively improved the electro-optical properties of LC/polymer materials^130,137^. For example, SO_2_ NPs changed the anchoring energy at the LC-polymer interface, and inorganic NPs such as ZnO and MgO changed the dielectric constant of the dielectric, both of which effectively reduced the driving voltage. Due to the surface plasmon excitation at the metal-LC interface, which enhanced the local electric field, films doped with metal NPs had low driving voltages and high CR values^130,138–140^. However, as mentioned above, the dispersion stability of NPs in liquid crystals is still a major challenge. In addition to the inherent properties of the liquid crystal molecules, the polymer morphology affects these properties. For example, Li et al.^136^ modified polymer microstructure and altered the electro-optical properties of films by adjusting the content of epoxy resin monomer, as shown in Fig. 10a. Reducing the LC droplet size shortened the response time but it also increased the driving voltage.

For “normal mode” P-LC films, the films are opaque in the absence of an applied field (off-state) and become transparent in the presence of an applied field (on-state). Yao-Dong Ma^141^ et al. proposed the opposite operation of P-LC films, called the reverse mode, where films were transparent in the off-state and changed to opaque in the ON state when a small electric field was applied. This type of film helped to broaden the range of applications of P-LC materials and attracted the attention of many scholars. In recent years, Meng et al. proposed a reverse-mode PSLC (R-PSLC) smart window that used a substrate with an uneven surface, as shown in Fig. 10b; it significantly increased the haze by 42% compared to that for a substrate with a smooth surface^142^. Deng et al. prepared a trans-PDLC smart window using a carbon nanotube as the alignment layer^143^. The optical performance of these smart windows was adjusted using near-infrared irradiation to fabricate highly efficient responsive PSLC smart windows without additional materials for orientation. This improved the durability of the device.

Thermally responsive P-LC materials were prepared based on the liquid crystal phase transition. Hiroshi Kakiuchida^144^ et al. developed a nonuniform irradiation method for photopolymerization-induced phase separation. The transparent and scattering states of LC/polymer films were controlled by temperature-induced phase separation of nuclei, and thermally responsive polymer network liquid crystal films in normal mode and reverse mode were developed, as shown in Fig. 10c.

#### Dynamic scattering

As early as the late 1960s, researchers found that when low-frequency electric fields were applied to some nematic liquid crystals, the LCs exhibited significant hydrodynamic instabilities. When the voltage exceeded a threshold value, as shown in (Fig. 9e), turbulence developed within the liquid crystal layer and produced strong scattering of light^145,146^. One of the main problems with the electrical characteristics of the dynamic light scattering mode was the high operating voltage;^147^ the predicted threshold voltage is given by Eq. 7^148^,

7 $$V_0^2 = \frac{{\pi ^2\left( {1 + \omega ^2\tau ^2} \right)k_{33}\sigma _\parallel ^2q^2}}{{\varepsilon _0^3\varepsilon _\parallel \varepsilon _ \bot \varepsilon _\alpha \left( {\omega ^2 - {{{\mathrm{C}}}}} \right)}}$$

where

8 $$C = \frac{{\sigma _\parallel }}{{\varepsilon _0^2\varepsilon _ \bot }}\left[ {\frac{{ - \alpha _2}}{{\eta _1}}\left( {\frac{{\sigma _\parallel }}{{\varepsilon _\parallel }} - \frac{{\sigma _\alpha }}{{\varepsilon _\alpha }}} \right) - \frac{{\sigma _ \bot }}{{\varepsilon _\parallel }}} \right]$$

ω is the angular frequency of the applied voltage, τ is the charge relaxation, k_33_ is the bend elastic constant, q is the wavenumber (approximately π/d), σ_⊥_and σ_∥_ are the perpendicular and parallel components of the conductivity, σ_a_ is the conductivity anisotropy (σ_α_ = σ_∥_−σ_⊥_), ε_⊥_and ε_∥_ are the perpendicular and parallel components of the dielectric constants, ε_α_ is the dielectric anisotropy (ε_α_ = ε_∥_−ε_⊥_), and η1and α_2_ are shear viscosities.

**Fig. 11:**
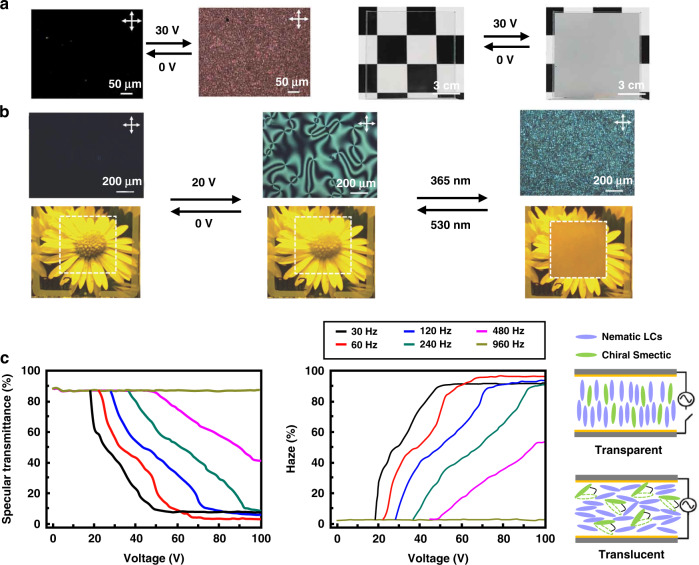
Switchable optical devices based on dynamic scattering principle. **a** smart window fabricated using the EHDI effect. Reproduced with permission from ref. ^149^. Copyright 2020, Taylor & Francis Group. **b** Light-induced EHDI devices obtained by doping LCs with a photochromic dye. Reproduced with permission from ref. ^150^. Copyright 2018, Wiley Online Library. **c** An LC smart window device based on the EHDI effect without ion doping

Increasing the overall conductivity by adding dopants is an effective strategy to lower the threshold value. Zhan^149^ et al. doped different ions in one LC material and discovered that the positive charge of the organic part in the salt was the primary cause of turbulence in the LC. Furthermore, Zhan et al. fabricated light-induced EHDI devices by doping LCs with a photochromic dye, spiropyran, as shown in Fig. 11b. With UV irradiation, the closed spiropyran ring opened to form open cyanocyanine (365 nm, purple flash). Ring closure was achieved with visible light (565 nm, green light). Tens of seconds of exposure to UV and green light activated or eliminated the EHDI effect^150^. However, this technology was limited by long-term stability, heating, and high power consumption due to the accumulation of ionic charges at the interface between the LC and alignment layers or the movement of ions in the cell^151,152^. To address these issues, Yoon^153^ et al. proposed an LC smart window device based on the EHDI effect without ion doping. As shown in Fig. 11c, N-LC was used as the host material, and SmC*-LC was used as the guest material. The dielectric properties of the two materials were opposite, and incident light was strongly scattered by randomly oriented LC molecules when an electric field was applied, which led to a very hazy translucent state.

The scattering process can adjust the propagation path of light to produce light protection. The advantage of this method is that it can provide a wide viewing angle and yet it exhibits the protective effect for wideband light sources. A disadvantage is that the attenuation rate of light is low, and most light passes through the material surface via a scattering path.

These three types of traditional light protection mechanisms have their own characteristics and advantages. Devices prepared by these mechanisms were utilized to provide protection from different light sources in the past few decades and achieved good effects. The properties of LC materials based on different light protection mechanisms are summarized in Table 2.

**Table 2:** Categories of light protection mechanisms based on LCs

Mechanism	Stimulation (range, amplitude, action time)	Light modulation type	Optical modulation effect (range, action effect)	Recovery	Full recovery	Ref
Absorption-based light protection	ES^a^ (AC^b^, 34 V, 100 ms)	Amplitude adjustment	800−2500 nm (S%^i^ > 9 5%), 380–780 nm (T%^j^ 83%−2%)	V^l^ Off	**√**	^176^
	ES^a^ (AC^b^, 30 V, 100 ms)	Amplitude adjustment	800–5500 nm (Δ T%^j^ 20%), 380–780 nm (T%^j^ 20%−78.5%)	V^l^ Off, 300 ms	**√**	^177^
	TS^d^ (~42 °C)	Amplitude adjustment	380–780 nm (T%^j^ 80%−1%)	RT^m^	**√**	^181^
	TS^d^ (27~35 °C)	Amplitude adjustment	380–780 nm (T%^j^ 40%−0%)	RT^m^	**√**	^182^
	TS^d^ (20~50 °C)	Amplitude adjustment	380–780 nm (T%^j^ 83.6%−0) & Infrared band (Δ T%^j^ 20%)	20 °C	**√**	^179^
Reflection -based light protection	TS^d^ (25~75 °C)	Band adjustment	2× RBs^k^	RT^m^	**√**	^195^
	TS^d^ (30~75 °C)	Band adjustment	RB:^k^ 450 nm−660 nm	RT^m^, 3 h	**√**	^196^
	TS^d^ (25~48 °C, 120 s)	Band adjustment	RB:^k^ 440 nm−660 nm	-	**-**	^171^
	TS^d^ (30~75 °C)	Band adjustment	RB:^k^ 450 nm−640 nm RB:^k^ 575 nm-650 nm	RT^m^, 3 h	**√**	^196^
	TS^d^ (17~130 °C)	Band adjustment	RB:^k^ 940 nm−1250 nm	RT^m^	**×**	^196^
	LS^e^ (365 nm, 120 s)	Band adjustment	RB:^k^ 440 nm−660 nm	LS^e^ (450 nm)	**√**	^171^
	LS^e^ (530nm, 60s)	Polarization adjustment	Reflected → Transparent	LS^e^ (440 nm)	**×**	^197^
	LS^e^ (530 nm, 60 s)	Polarization adjustment	Reflected → Transparent	LS^e^ (530 nm)	**×**	^197^
	LS^e^ (360 nm, 2~10 min)	Band adjustment	RB:^k^ 400–570 nm	Light Off, 24 h	**√**	^169^
	LS^e^ (365 nm, 7~43 s)	Band adjustment	RB:^k^ 400 nm−740 nm	LS^e^ (520 nm),12 s	**√**	^170^
	LS^e^ (365nm, 60s)	Band & Polarization adjustment	RB:^k^ 300~420 nm −420~550 nm Right-handed-circularly → Left-handed-circularly	LS^e^ (380–780)/Light Off/TS^d^	**√**	^198^
	LS^e^ (980 nm, 120s)	Band adjustment	RB:^k^ 400 nm−740 nm	LS^e^ (980 nm), 120 s	**√**	^199^
	ES^a^ (AC^b^, 8.6 V µm^−1^)	Polarization adjustment	700–1400 nm Reflected → Transparent	V Off	**√**	^19^
	ES^a^ (DC^c^, 0~1.2 V µm^−1^)	Band adjustment	RB:^k^ 120 nm−1100 nm	V Off	**√**	^200^
	HS^f^ (RH^g^ > 45%)	Band adjustment	RB:^k^ 1150 nm−1570 nm	TS^d^ (>30 °C, 5 min)	**√**	^201^
	PHS^h^ (PH = 7~9, 3~5 min)	Band adjustment	RB:^k^ 450 nm−550 nm	–	**–**	^202^
	PHS^h^ (acidic, >200 s)	Band adjustment	RB:^k^ 600 nm−770 nm	–	**–**	^203^
	PHS^h^ (PH = 9)	Band adjustment	RB:^k^ 520 nm−720 nm	PHS^h^ (PH 3)	**×**	^204^
	TS^d^ (>60 °C)	Band adjustment	RB:^k^ 648 nm−460 nm	RT^m^, 3 days	**√**	^205^
	TS^d^ (>75 °C)	Band adjustment	RB:^k^ 780 nm−540 nm	RT^m^	**√**	^195^
Scattering-based light protection	ES^a^ (AC^b^, 100 Hz, 40 V)	Amplitude adjustment	T%^j^ 64%−10%	ES^a^ (AC^b^,1000 Hz,15 V)	**√**	^180^
	ES^a^ (AC^b^, ~20v, 84 ms)	Amplitude adjustment	T%^j^ 60.8%−1.3%	V^l^ Off	**√**	^175^
	ES^a^ (AC^b^, 0.85~1.45 V/μm)	Amplitude adjustment	T%^j^ 0.41%−98.98%	V^l^ Off	**√**	^167^
	ES^a^ (AC^b^,1 kHz, ~3.8 V/ μm, 10~100 ms)	Amplitude adjustment	T%^j^ 90%−20%	V^l^ Off ES^a^ (AC^b^,1000 Hz, <2.8 V/μm)	**√**	^166^
	ES^a^ (AC^b^, 55 V, 55 ms)	Amplitude adjustment	T%^j^ 79%−1%	V^l^ Off, 55 ms	**√**	^184^
	ES^a^ (AC^b^, 24 V, <5 ms)	Amplitude adjustment	400 nm−800 nm (T%^j^ ~0%−70%)	V^l^ Off, 1000 ms	**√**	^206^
	ES^a^ (AC^b^, 70 V, 40 ms)	Amplitude adjustment	Scattering → Transparent	V^l^ Off, 200 ms	**√**	^164^
	ES^a^ (AC^b^, 40 V)	Amplitude adjustment	Scattering → Transparent	V^l^ Off	**–**	^207^
	LS^e^ (UV, 2 mW·cm^−2^,30 s)	Amplitude adjustment	T%^j^ 79%–1%	Light Off	**√**	^180^
	TS^d^ (37.8 °C)	Amplitude adjustment	T%^j^ 64%–16.7%	TS^d^ (25°C)	**√**	^184^
	TS^d^ (>30 °C)	Amplitude adjustment	380–780 nm (ΔT%^j^ 10%)	RT^m^	**√**	^178^
	LS^e^ (980 nm, ~10 s)	Amplitude adjustment	Transparent → Scattering	Light Off, ~30 s	**√**	^25^
	LS^e^ (UV, 30 s)	Amplitude adjustment	Scattering → Transparent	Light Off	**√**	^207^
	TS^d^ (29 °C)	Amplitude adjustment	T%^j^ 64%–17.1%	TS^d^ (21.2 °C)	**√**	^180^

^a^ES Electrical Stimulation, ^b^AC Alternating Current, ^c^DC Direct Current, ^d^TS Temperature stimulation, ^e^LS Light stimulation, ^f^HS Humidity stimulation, ^g^RH Relative humidity, ^h^PHS Potential of hydrogen stimulation, ^i^S% Shielding rate, ^j^T% Transmittance, ^k^RB Reflection band, ^l^V Voltage, ^m^RT Room temperature

However, these traditional mechanisms for light protection have inherent defects. With the increasing complexity of the light environment, we need to make comprehensive use of these mechanisms and combine their advantages to meet more demanding application requirements.

## Strategies for LC-based light protection

**Fig. 12:**
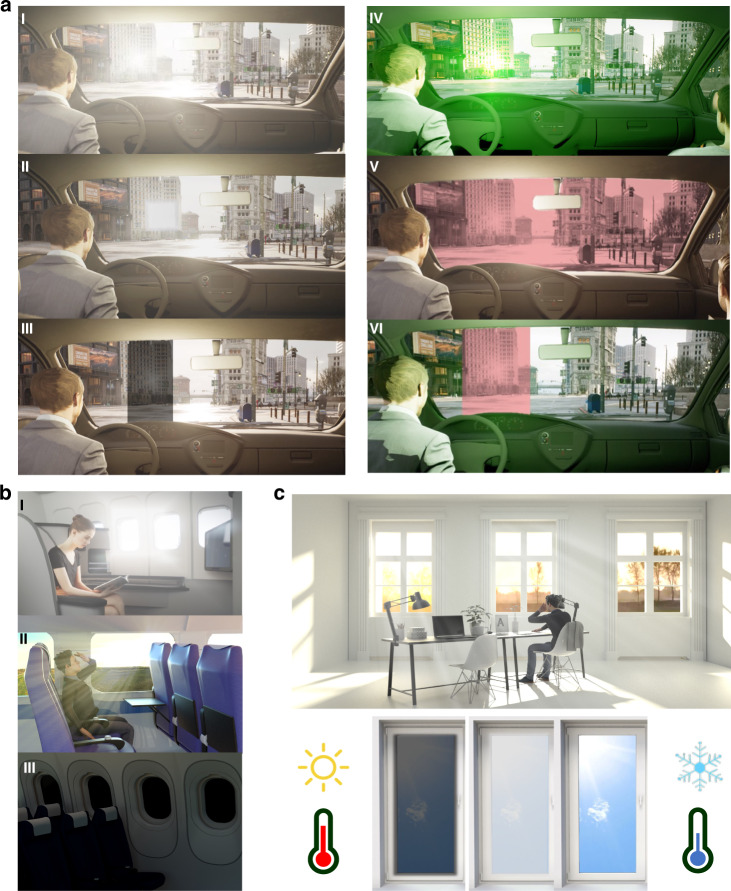
Different light protection environment. **a** Windshields with rectifying protective members made of transparent material placed in front of the driver’s seat: (I–III) Effects of daylight and protection strategies; (IV–VI) Effects of artificial light on windshields and protection strategies. **b** Other optical windows on vehicles and their light protection strategies. **c** Optical windows in buildings and their light protection strategies

Thus far, we have covered the characteristics of various light sources and LC-based light protection mechanisms. However, the requirements for light protection vary according to the light environment. In most cases, comprehensive protection requires multiple coupled mechanisms to meet requirements, such as fast response, high contrast, large-area fabrication, and energy efficiency. In various light protection scenarios, LC-based light protection strategies are different. As shown in Fig. 12a,b, transient light protection is often needed for vehicles. Buildings require devices that provide long-term light protection (Fig. 12c).

### Transient light protection strategies

Traffic injuries are a significant cause of death in the world^154^. The most common situations occur when driving while facing light, as when sunlight shines directly through the windshield into the eyes of the driver (pilot) and affects their vision. The conventional protection strategy is to use sun visors. However, as shown in Section ‘Protection against natural light’ above, the irradiation angle of sunlight varies greatly, and the use of visors in fixed positions greatly limits their protective effect and is a safety hazard that could contribute to collisions. A better protection strategy is to use switchable light-shielding materials in local areas of the windshield to change the propagation path of incident light energy and reduce the damage caused by direct sunlight entering the eyes of drivers (pilots), as shown in Fig. 12a. Additionally, it is necessary for the windshield, as the main channel through which the driver (pilot) sees the outside world, to have a good visual field (high transmittance, low haze) and fast switching speed of light protection.

In Section ‘Scattering-based light protection’, P-LC materials based on the scattering principle exhibited protection from wideband light sources, which was consistent with the characteristics of the broad-spectrum radiation of sunlight. Therefore, these materials are potential candidates for windshield light protection. However, the reported switching speeds of commercial P-LC materials were typically within hundreds of milliseconds^128,130,131,136,142–144,155–162^, and for important windows such as windshields, faster switching speeds are often sought.

**Fig. 13:**
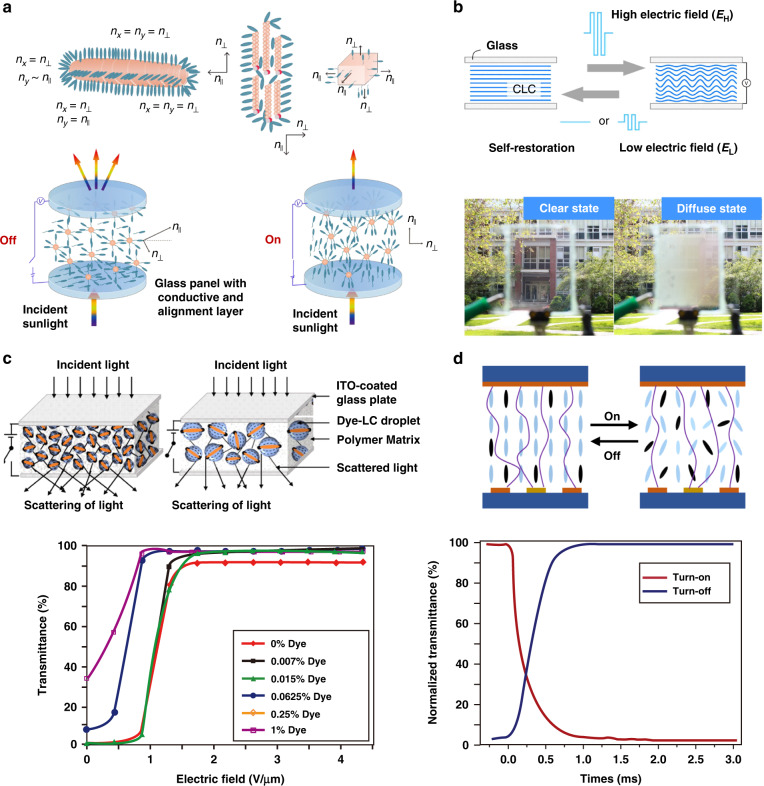
Light protection strategies for windshields. **a** Light protection device based on doping LCs with low concentrations of nanoporous microparticles. The images above are three types of nanoporous microparticles. The picture below is a schematic diagram of nanoporous particles inducing molecular orientation of liquid crystals. **b** Light protection device based on CLC. Reproduced with permission from ref. ^166^. Copyright 2018, Optica Publishing Group. The effect diagram of the device is shown above, and the schematic diagram is shown below. **c** The plot of transmittance vs. electric field for PDLC with different dye concentrations and the scattering effect of PDLC thin films for LC droplets with different diameters. Reproduced with permission from ref. ^167^. Copyright 2022, Elsevier. **d** Graph of transmittance vs. time, electrode design of dye-doped PSLC, and schematic diagram of the scattering principle

The new protocol based on the doping of LC with a low concentration of nanoporous microparticles presented by the group of I. Abdulhalim created windows that rapidly switched between transparent and strong scattering states. In the OFF state, the average refractive index of these particles was highly mismatched with the refractive index of the surrounding LC region due to the random orientations of the particles, which produced strong scattering. In the ON state, the molecules oriented in the direction of the electric field, which increased the transparency of the system. As shown in Fig. 13a, researchers used three types of nanoscale and microscale particles to demonstrate this concept: (1) cochleate particles^163^, (2) colloids of decanol^164^, and (3) nanoporous Si microparticles^165^. In the off and on states, the rise and fall times of these windows were approximately a few milliseconds and tens of milliseconds, respectively. For security reasons, a windshield should be completely transparent during an emergency. However, all the aforementioned methods were opaque at zero electric field; thus, they are unsuitable for this application.

Chun-Wei Chen and colleagues reported the development of a light protection window based on cholesteric LCs with negative dielectric anisotropy, as shown in Fig. 13b. The transparent state exhibited well-aligned planar cholesteric texture and was stable in the absence of an electric field, whereas the scattering state turned on when a field above the fluctuating instability threshold was applied. The material had 86% transmittance in the transparent state and could switch to the scattering state within 10 to 100 ms, where the specular transmittance decreased to 11%^166^.

Scattering-based equipment causes radiation to be scattered in the forward direction^163,164,166^, and protection is not effective under strong sunlight. The light protection method of multi-mechanism integration combines traditional light energy transformation, reflected transfer, and scattering transfer, and it was widely applied in recent years.

Many scholars recently conducted research on doping dye molecules into P-LC materials to combine traditional light energy transformation and scattering transfer mechanisms to enhance the protection of the materials. Anuja Katariya-Jain^167^ et al. investigated the effects of doping LC materials with dyes on the properties of the resulting PDLC films, as shown in Fig. 13c; the dopants changed the specular transmittance of the films from 0.41% to 98.98% at a low voltage (1.45 V/μm). A dye-doped R-PSLC smart window with a special electrode pattern was designed by Tae-Hoon Yoon^168^ et al. As shown in Fig. 13d, the visible transmittance of this device changed from 67.0% (in the OFF state) to 3.2% (in the ON state) with a fast response time (<1 ms) and low operating voltage (10 V).

Artificial light sources, such as lasers and other forms of high-intensity lighting, also pose risks. As shown in V in Fig. 12a, traditional laser protection strategies are achieved by selectively blocking specific wavelengths of laser light. However, this causes difficulty for the driver (pilot) in distinguishing certain colors and affects visual performance. Additionally, since the glare interference source is uncontrollable, the material should react automatically with a passive protection strategy so that it is not necessary for the driver (pilot) to actively adjust the optical properties of the windshield. Currently, there are no proven protection technologies that provide this capability.

**Fig. 14:**
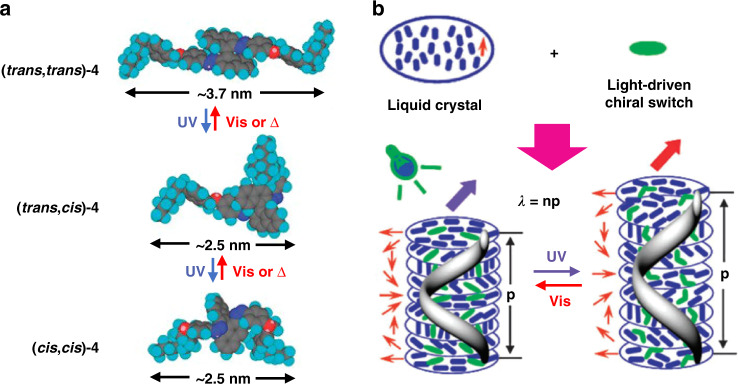
Light-driven switchable optical devices. **a** Photo-driven cis-trans isomerization process of chiral molecular switches. **b** Schematic diagram of the mechanism by which light is reversibly and dynamically tuned to the reflected wavelength. **a, b** Reproduced with permission from ref. ^170^. Copyright 2010, Royal Society of Chemistry

However, one potential method involves using light-driven chiral molecular switches to induce Ch-LCs to achieve reversible selectivity over the entire visible spectral range, a method known as reflective color tuning (shown in Fig. 14a)^169,170^. The HTP value of the chiral molecules changes dramatically after photoisomerization. To achieve light protection when the device is exposed to a certain wavelength band (450 nm), the center of the reflection wavelength of the device shifts to 440 nm^171^.

In addition to windshields, optical windows on vehicles include aircraft portholes, vehicle side windows, sunroofs and side windows on ships. Unlike windshields, these windows primarily enhance the passenger experience and do not require windshield-level visibility and rapid switching speed for light protection. Due to the need for passengers to read and rest, it is necessary for these windows to have good thermal and light insulation properties, as shown in Fig. 12b. Because these windows may be in an opaque state for a long time, low energy consumption is also an issue that should be considered.

**Fig. 15:**
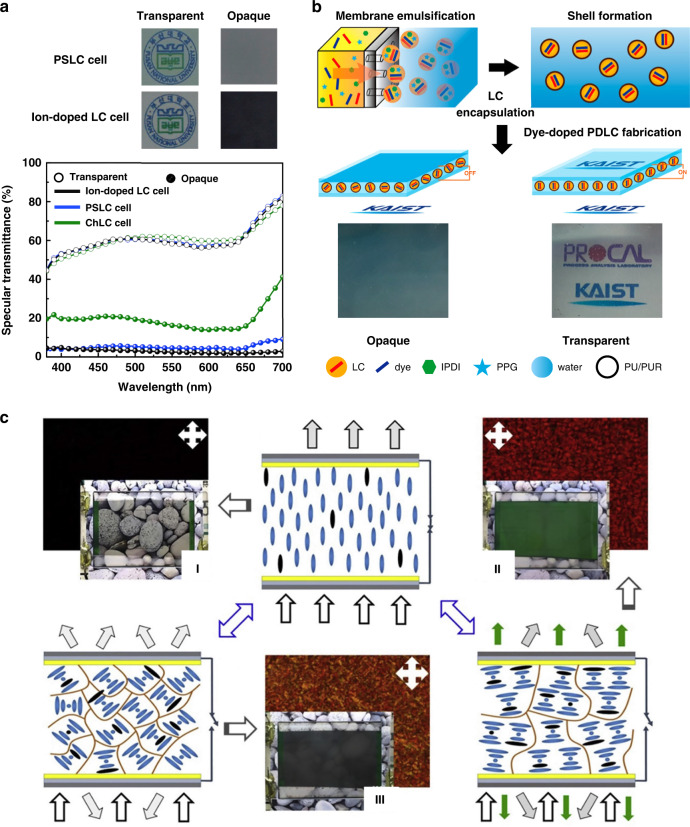
Light protection strategies for other optical windows on vehicles. **a** Comparison of the transmission characteristics of PSLC and ion-doped LC cells. Reproduced with permission from ref. ^86^. Copyright 2018, Elsevier. **b** PDLC preparation process to avoid dye contamination. Adapted with permission from ref. ^172^. Copyright 2015, American Chemical Society. **c** When an electric field was applied, the liquid crystals arranged vertically, and the device was in a transparent state (I). After the voltage was turned off, the CLCs were distributed in a plane, and the device reflected green light with a width of approximately 57 nm centered at 536 nm (II). In another case, the CLCs were arranged in a focal conic state, and the device was in a black fog state (III). Reproduced with permission from ref. ^173^. Copyright 2019, Elsevier

As mentioned above, dye-doped P-LC materials were capable of both light scattering and absorption and had good dimming properties. However, there was a trade-off between light scattering and absorption. Tae-Hoon Yoon^86^ et al. used a negative LC with dye, and electrohydrodynamic effects to provide an LC cell with a very low overall transmittance, as shown in Fig. 15a. At 40 V, the specular transmittance changed from 60.1% to 3% with strong scattering. In addition, in dye-doped P-LC materials, the dye dissolved in the polymer matrix, which limited the contrast. Kim et al. reported encapsulating dye in monodisperse capsules to solve dye-related issues. A contrast ratio of 120 or greater was achieved after encapsulation, as shown in Fig. 15b^172^. When preparing dye-doped PSCLC films, some studies combined traditional light energy conversion, reflected transfer, and scattering transfer^173^. The device shown in Fig. 15c switched between reflected green light, black fog, and transparent states with a response time of hundreds of milliseconds.

**Fig. 16:**
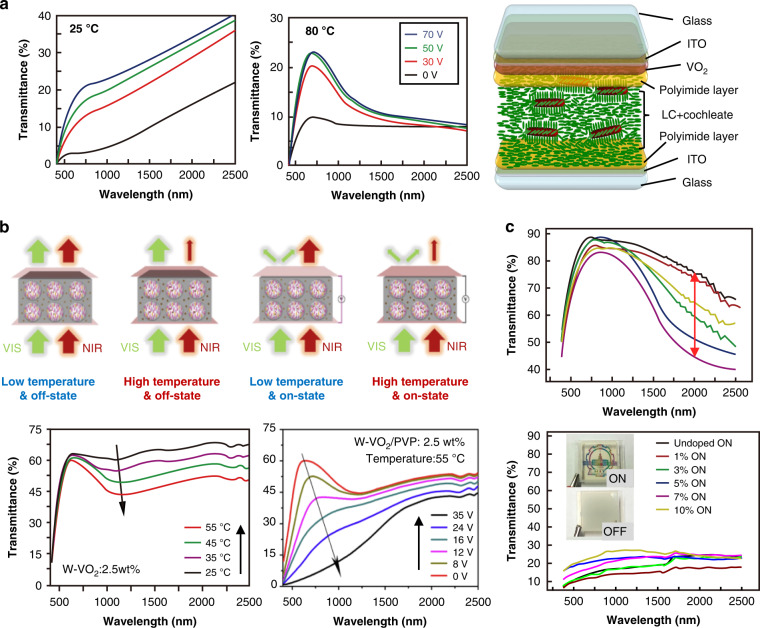
Switchable optical devices with IR shielding performance. **a** Schematic illustration of the VO_2_-based LC-cochleate device and transmission spectra of the device plotted as a function of wavelength at different temperatures. Reproduced with permission from ref. ^174^. Copyright 2021, Optica Publishing Group. **b** Four optical modulation modes realized in a hybrid microscale/nanoscale composite film with W-VO_2_ and variations in the vis/NIR transmittance spectra. Reproduced with permission from ref. ^175^. Copyright 2017, American Chemical Society. **c** Schematic of ATO NPs -doped in a PSLC smart film with on/off states and light protection control. Reproduced with permission from ref. ^177^. Copyright 2021, Royal Society of Chemistry

Unlike visible wavelengths, light transmission in the infrared wavelengths brings heat radiation, so it is essential to manufacture windows that possess IR shielding properties along with good transparency. A hybrid window made of nanoscale and microscale LCs and a layer of thermochromic material (VO_2_) was demonstrated recently to fulfill this purpose, as shown in Fig. 16a^174^. Furthermore, many researchers tried doping NPs into P-LC materials to expand the modulation band while ensuring the fast response of light protection devices. Liang^175^ et al. reported the development of an LC light protection device with controllable visible and near-infrared light transmittance, which contained microscale LC domains with a negative dielectric constant and tungsten-doped vanadium dioxide (W-VO_2_) nanocrystals. Due to the combination of scattering with absorption and reflection, the transmittance of near-infrared light was reversibly and passively modulated between 59.4 and 41.2% by temperature, as shown in Fig. 16b. In addition, tungsten bronze (Cs_x_WO_3_) NRs were doped into this LC system to obtain a multi-response soft matter composite smart film; more than 95% of the NIR irradiation from 800 to 2500 nm was screened^176^. Furthermore, Zhang^177^ et al. prepared multiscale smart windows by mixing ATO NPs into PSLC films. An optical modulator with wavelength range from 380-nm to 5500-nm was created using PSLC visible light scattering and semiconductor nanoparticle absorption. In the visible region, the transmittance was changed from highly transparent (78.5%) to strong light scattering (10%), while up to 80.75% of infrared invisible light was effectively shielded, as shown in Fig. 16c.

**Fig. 17:**
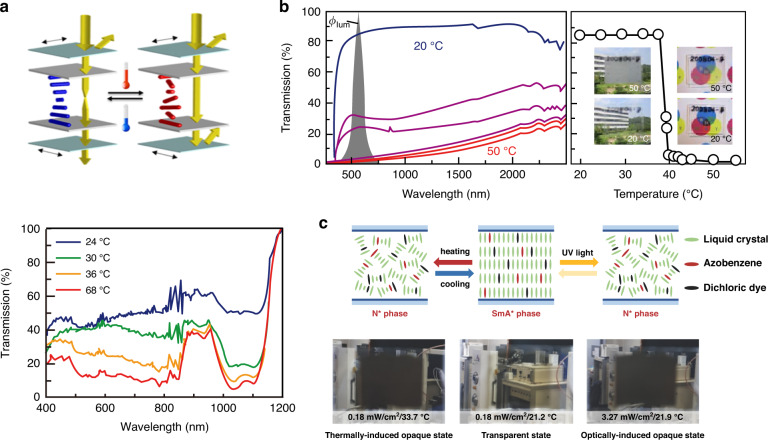
Non-electrically switchable optical devices. **a** The relationship between transmittance and wavelength at different temperatures and the schematic diagram of device adjustment. Reproduced with permission from ref. ^178^. Copyright 2021, Wiley Online Library. **b** Relationship between transmittance and wavelength at different temperatures and sample effect diagram. Reproduced with permission from ref. ^179^. Copyright 2021, American Chemical Society. **c** Schematic diagram of the regulation principle of light and heat dual stimulation and the sample effect diagram. Reproduced with permission from ref. ^180^. Copyright 2018, Wiley Online Library

All of the above examples used electronic equipment to provide light protection. Researchers studied thermally controlled light protection equipment to save even more energy. Kragt^178^ et al. reported a novel LC polymer-based device for light protection that reversibly increased reflectivity upon heating, as shown in Fig. 17a. Kakiuchida^179^ et al. developed a polymer network liquid crystal (PNLC) whose scattering intensity varied with temperature. As shown in Fig. 17b, from 20 °C to 50 °C, the direct transmittance of the PNLC decreased from 83.6% to 0.7%, and the transmittance in the infrared band changed by approximately 20%^179^.

Researchers also developed light protection devices that responded to electricity, heat, and light^180^. UV irradiation, temperature increase, or an electric field caused them to change them from a very transparent to a black fog state (Fig. 17c). Notably, unlike the above devices, it was metastable, meaning that an electric field was not required to maintain the black fog state; this further reduced energy consumption.

### Long-term light protection strategies

Unlike optical windows used on vehicles, windows in buildings are in fixed positions and exposed to sunlight for extended periods of time. Moreover, the areas of windows in buildings are often much larger than those of optical windows in vehicles. These different usage scenarios put forward new requirements for light protection devices for buildings: (1) low-cost and large-area fabrication, (2) long-term operation with low energy consumption, and (3) capability for infrared adjustment.

**Fig. 18:**
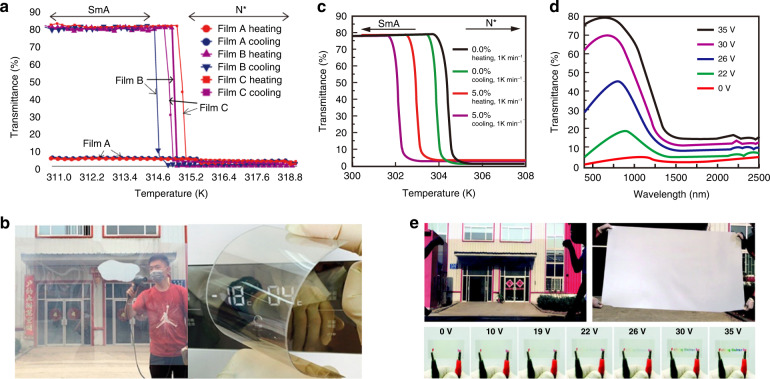
Light protection strategies for buildings. **a** Graph of the transmittance of the sample as a function of temperature. **b** The prepared large-area films and their bending properties. **a, b** Reproduced with permission from ref. ^181^. Copyright 2017, American Chemical Society. **c** Graph of the transmittance of the sample as a function of temperature. **d** Graph of the transmittance of the sample as a function of wavelength for different voltages. **e** The prepared large-area films and their effects at different voltages. Reproduced with permission from ref. ^21^. Copyright 2017, Royal Society of Chemistry

The commercial P-LC materials mentioned in Section ‘Scattering-based light protection’ are the preferred materials. For low-cost large-area fabrication, some researchers have studied the use of flexible substrates (e.g., polyethylene terephthalate or PET) instead of rigid substrates (glass) to prepare P-LC materials. Yang^181^ et al. reported a coexistence system of polymer-dispersed and polymer-stabilized liquid crystals (PD&SLCs) by sandwiching the LC mixture into two pieces ITO-coated flexible substrates. This system can be used in large-area manufacture (with a size of 1.8 m × 1.1 m) of the smart films. In addition, the film automatically adjusted its transparency according to temperature without additional energy input. The LC droplets in the film underwent a phase transition at approximately 42 °C, the specular transmittance of visible light decreased from 80% to 1% without the need for electric field maintenance; the film was transparent at room temperature (Fig. 18a,b). Meanwhile, large-area PD&SLC films were prepared in this study; they displayed great potential for applications. In addition, PDLC films with tITO NCs on flexible substrates were investigated. Due to the localized surface plasmon resonance of ITO NCs, more than 85% of the infrared rays (750–2500 nm) were effectively shielded. The specular transmittance of visible light by the film decreased from 78% to 1.5% (Fig. 18c,d) as the result of temperature stimulation or application of an electric field^21^. Furthermore, large-area films with dimensions of 1.8 m × 1.1 m (Fig. 18e) were prepared in this study.

**Fig. 19:**
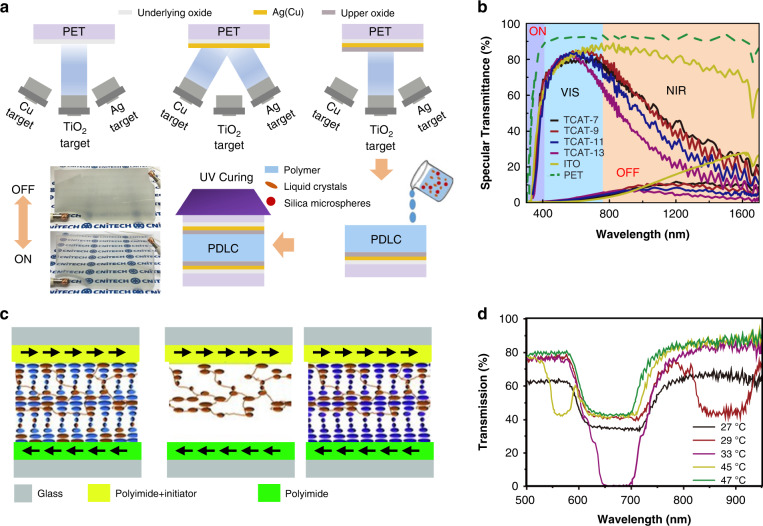
Switchable optical devices with good temperature management. **a** TCAT substrate preparation process and sample effect. **b** Optical properties of PDLC films prepared on different substrates. **a, b** Reproduced with permission from ref. ^230^. Copyright 2019, Elsevier. **c** Principle of STPN-based composite film fabrication. **d** The reflectance of composite films with different hazes vs. wavelength. **c, d** Reproduced with permission from ref. ^182^. Copyright 2011, Wiley Online Library

Huang^153^ et al. applied their expertize in flexible electrodes to design and optimize a TiO_2_/Ag(Cu)/TiO_2_ (TCAT) electrode to simultaneously tune visible light transmission and near-infrared heat shielding (Fig. 19a,b). After applying an electric field, the specular transmittance of the TCAT-based PDLC device increased from approximately 1% to 84.9%. Additionally, TCAT-based devices provided better thermal insulation than ITO-based devices and good modulation performance in the 400–1600 nm band. In addition to utilizing light energy transformation and scattering transfer methods for light protection, the method of reflected transfer of light energy was used for light protection in the architectural field. Michael^182^ et al. presented a method for fabricating surface-tethered polymer networks (STPN) composed of helical polymer structures, where STPN acted as a structurally chiral scaffold that forced the swollen LC mixture to assume the same handedness as the polymeric material and reflect light according to the as-fabricated pitch (Fig. 19c,d). Backfilling an STPN cell with a thermally tunable SmA* mixture allowed for temperature-induced and nearly complete reflection.

**Fig. 20:**
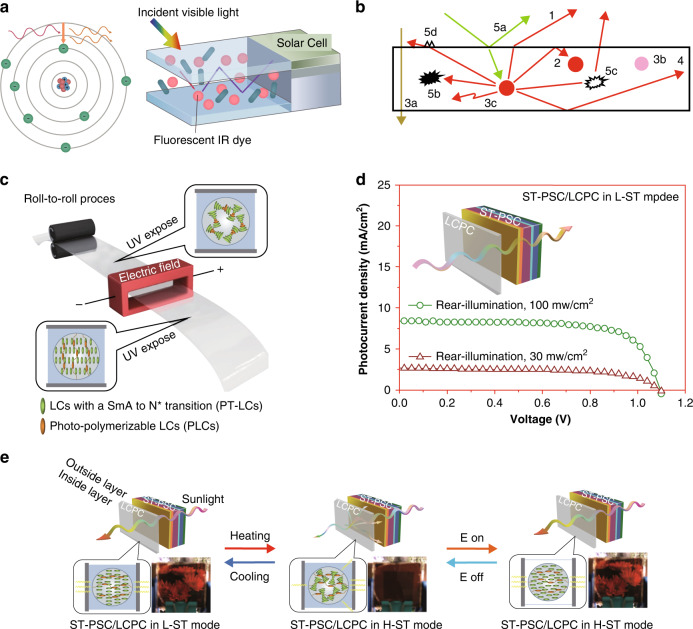
Self-powered switchable optical devices. **a** Schematic diagram of the working principle of the luminescent solar concentrator (LSC). **b** Loss mechanisms in LSCs: (1) Light emitted outside the capture cone; (2) Reabsorption of emitted light by another luminophore; (3a) Input light unabsorbed by the luminophore, (3b) Limited luminophore stability, (3c) Internal quantum efficiency of the luminophore is not unity; (4) Solar cell losses; (5a) Reflection from the waveguide surface, (5b) Absorption of emitted light by the waveguide, (5c) Internal waveguide scattering, and (5d) Surface scattering. Reproduced with permission from ref. ^183^. Copyright 2012, Wiley Online Library. **c** Preparation of rolls of multiresponse LCPC films Schematic diagram of the roll-to-roll process. **d** J–V curves of the back-illuminance of ST-PSC/LCPC under different incident light intensities. **e** Schematic diagram of the working principle in three modes after combining LCPC films with ST-PSCs. **c-e** Reproduced with permission from ref. ^184^. Copyright 2019, Wiley Online Library

A limitation of conventional light protection systems for light energy conversion is that most of the absorbed energy is eventually rereleased as localized heat, thereby causing a temperature rise, which is not good for equipment that operates for long periods of time and can cause damage indoors. A more advanced light energy conversion concept is the luminescent solar concentrator (LSC). LSCs use dyes embedded in polymer or glass plates that function as windows. These dyes absorb near-infrared light and subsequently fluoresce at longer wavelengths (Fig. 20a). A fraction of this re-emitted light is trapped in a higher refractive index polymer or glass panel, which acts as a light guide. The trapped light is transported via total internal reflection and only exits at the sides of the window, where it may be converted to electricity via attached photovoltaic cells^177,181^. LSCs have not yet been widely commercialized, primarily owing to their modest efficiencies. The first loss in the LSC originates from the light emitted by the luminophore at an angle such that it is refracted out of the waveguide through an escape cone rather than reflected internally (Fig. 20b)^183^. Organic luminophores are often dichroic in terms of absorption and transmission. Aligning organic dyes perpendicular to the plane of the waveguide surface (homeotropically) using a host LC leads to emissions in the plane of the waveguide, which results in a sharp decrease in the surface loss to less than 10%. Yu Xia^184^ et al. fabricated a very transparent (79%) light protection device by coupling a multiresponse liquid crystal/polymer composite (LCPC) and planar semitransparent perovskite solar cell (ST-PSC) capable of responding (Fig. 20c–e). The device can respond to temperature or electric field stimuli and generate photoelectric conversion, and it has potential for large-area fabrication.

## Conclusions and perspectives

Due to the unique thermodynamic properties of LC materials, optical technologies based on liquid crystal materials have developed rapidly over the past several decades. In this review, we provide a comprehensive evaluation of light protection applications for switchable optics based on LCs. In this context, “light protection” refers to the prevention or weakening of damage, discomfort or interference caused by inappropriate light radiation on people and light-sensitive instruments. According to the source, light radiation can be classified as natural light or artificial light. In this review, we discuss their characteristics, such as frequency, energy and irradiation area, and summarize the characteristics of light protection. From the perspective of light energy, there are three types of photoprotection principles for LCs: (1) conversion of incident light energy to other forms of energy; (2) transfer of incident light energy through reflected paths; and (3) transfer of incident light energy through scattering paths. We discuss the characteristics, advantages and applications of each of these three types of light protection principles for light protection devices. However, in the presence of complex optical environments, comprehensive protection often requires that multiple mechanisms are coupled. We propose recommendations for light protection strategies in different light environments. When applying light protection devices to vehicles, it is necessary to focus on transient light protection strategies, while for buildings, long-term light protection strategies are appropriate.

Although several types of light protection devices for LCs were studied, those for practical applications are still very limited. Challenges and opportunities coexist in the course of accelerating the maturation of this exiting field. First, it is challenging to develop LC-based light protection devices that adapt to different external environments during long-term stable operation. Prolonged exposure to UV light makes polar groups in LC molecules susceptible to oxidation and molecular structures fracture. It also induces the fracture of organic polymers in P-LC materials, cracks in devices and photodegradation of dichroic dye molecules^185–187^. However, LCs are highly susceptible to ion production under electrical stimulation, and these ions affect the performance of the devices in different ways and interfere with the dispersion stability of NPs in LCs, which greatly limits the lifetime of relevant devices^188–193^. Therefore, mature light protection devices for LCs should have high UV radiation tolerance and electrochemical stability. Second, in current commercial installations, there is a lack of LC-based light protection devices that flexibly adapt to protective requirements for different external environments. Increasingly complex optical environments require a new generation of light protection devices that can operate at multiple wavelengths and react quickly, especially for passive protection. Currently, common external stimuli that cause changes in the optical performance of smart windows include electric fields, temperature, and light. Electrical stimuli correspond to active light protection devices, while thermal and optical stimuli correspond to passive light protection devices. However, the slow response to thermal stimuli and the limited wavelengths of light response limit the application of these passive light protection devices. As near-infrared light is an important component of sunlight and an important source of thermal radiation, further research can focus on developing light stimulation response devices at this range of wavelengths. Additionally, intelligent passive light protection devices should dynamically sense the light environment and adjust their optical properties according to the external light intensity. Third, these technologies face the challenge of ensuring good stability and homogeneity for extensive commercial applications. For example, optically and chemically pure materials are essential for the proper operation of Ch-LC-based devices, as any impurity can adversely affect the self-assembly of molecules^194^. In addition, many commercially available optical windows are irregularly curved rather than planar. There are challenges in the preparation of such conformal light-proof devices. In addition, there is a broad commercial market for retrofitting conventional (traditional) windows with new windows with light protection features. Therefore, the development of deformable, flexible light protection devices will be ideal for commercial applications in the future.

There is no doubt that with the development of lighting technology, the need for light-protection devices is becoming increasingly urgent. There is strong commercial interest from related companies, such as Merck Chemicals, BASF, Dainippon Ink and other leaders in the chemical industry, where researchers are currently working on the development of light-protection devices. We expect that thorough research on a new generation of intelligent optical limiting materials will address the above issues. Programmable flexibility and self-adaptation will be critical in realizing optical materials for new demands. This system will not only provide protection from multiple wavelength bands but also respond quickly in complex environments, protect across the entire UV–visible–infrared spectrum, supply high-strength protection in some bands, and have long service life. The system should work well provided that the device is stable under high-power incident light and consumes small amounts of energy in the long-term protective state.

## Supplementary information

Fig 20 copyright promotion

Fig 1 copyright promotion

Fig 3 copyright promotion

Fig 4 copyright promotion

Fig 5 copyright promotion

Fig 6 copyright promotion

Fig 7 copyright promotion

Fig 8 copyright promotion

Fig 10 copyright promotion

Fig 11 copyright promotion

Fig 13 copyright promotion

Fig 14 copyright promotion

Fig 15 copyright promotion

Fig 16 copyright promotion

Fig 17 copyright promotion

Fig 18 copyright promotion

Fig 19 copyright promotion
