# Supplementary material for: Advanced liquid crystal-based switchable optical devices for light protection applications: principles and strategies
Source: Light Sci Appl. 2023 Jan 3;12:11. doi: 10.1038/s41377-022-01032-y (PMC9807646; doi:10.1038/s41377-022-01032-y)
Supplement: Supplementary file 1 — Fig 20 copyright promotion [file 41377_2022_1032_MOESM1_ESM.pdf]

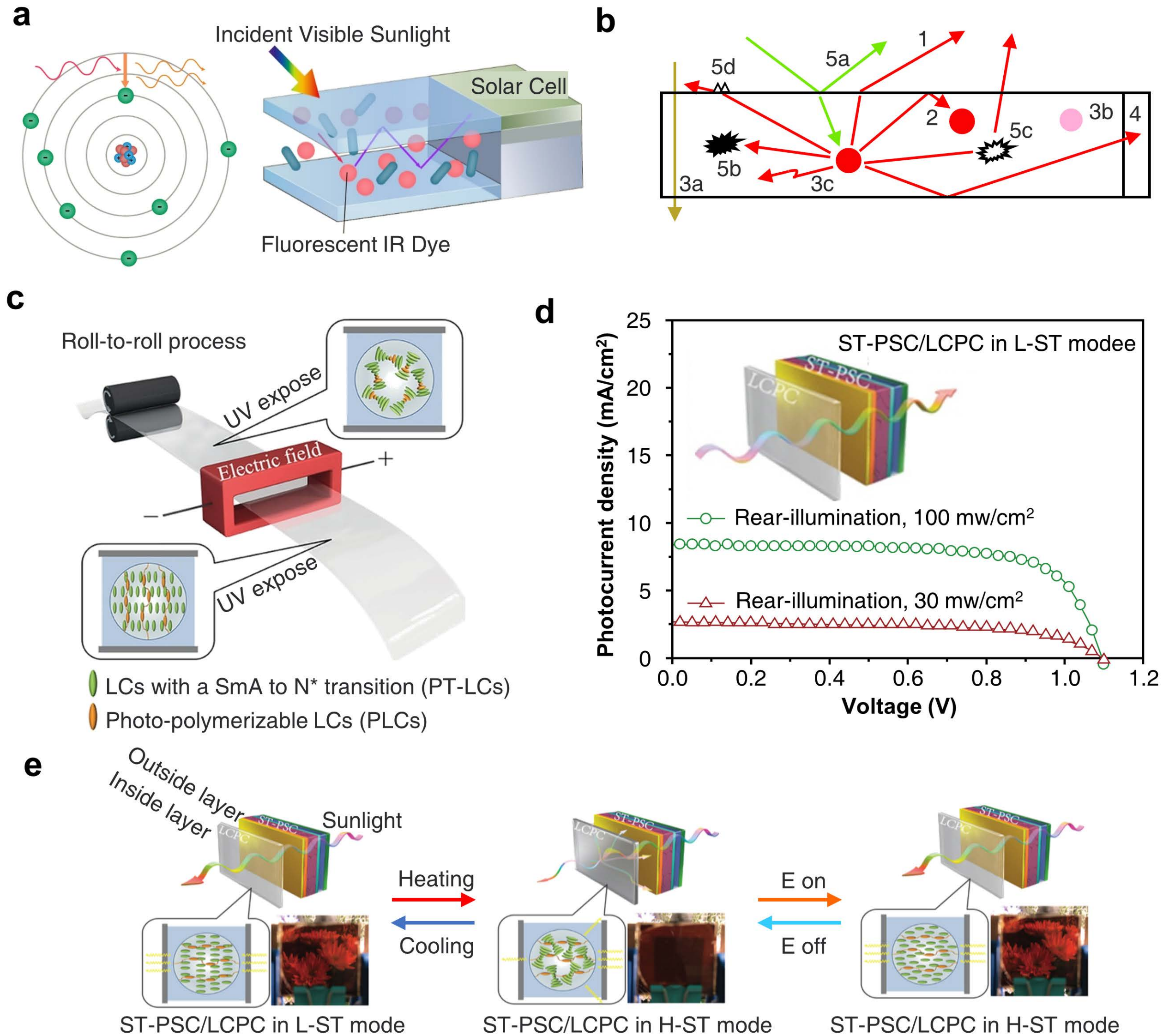

a
Incident Visible Sunlight
Solar Cell
Fluorescent IR Dye
b
1
2
3a
3b
3c
4
5a
5b
5c
5d
c
Roll-to-roll process
UV expose
Electric field
+
−
UV expose
LCs with a SmA to N* transition (PT-LCs)
Photo-polymerizable LCs (PLCs)
d
ST-PSC/LCPC in L-ST modee
ST-PSC
LCPC
Rear-illumination, 100 mw/cm2
Rear-illumination, 30 mw/cm2
Photocurrent density (mA/cm2)
Voltage (V)
0
5
10
15
20
25
0.0
0.2
0.4
0.6
0.8
1.0
1.2
e
Outside layer
Inside layer
Sunlight
ST-PSC
LCPC
Heating
Cooling
E on
E off
ST-PSC/LCPC in L-ST mode
ST-PSC/LCPC in H-ST mode
ST-PSC/LCPC in H-ST mode

JOHN WILEY AND SONS LICENSE TERMS AND CONDITIONS

Sep 19, 2022

This Agreement between Harbin Institute of Technology -- Ruicong Zhang ("You") and John Wiley and Sons ("John Wiley and Sons") consists of your license details and the terms and conditions provided by John Wiley and Sons and Copyright Clearance Center.

License Number	5392430738880
License date	Sep 19, 2022
Licensed Content Publisher	John Wiley and Sons
Licensed Content Publication	Advanced Energy Materials
Licensed Content Title	Thirty Years of Luminescent Solar Concentrator Research: Solar Energy for the Built Environment
Licensed Content Author	Paul P. C. Verbunt, Michael G. Debije
Licensed Content Date	Dec 12, 2011
Licensed Content Volume	2
Licensed Content Issue	1
Licensed Content Pages	24
Type of Use	Journal/Magazine
Requestor type	University/Academic
Is the reuse sponsored by or associated with a pharmaceutical or medical products company?	no
Format	Print and electronic
Portion	Figure/table
Number of figures/tables	1
Will you be translating?	No
Circulation	200 - 499
Title of new article	Advanced liquid crystal-based switchable optical devices for light protection applications: principles and strategies
Lead author	Ruicong Zhang, Zhibo Zhang, Jiecai Han, Lei Yang, Jiajun li, Zicheng Song Tianyu Wang, Jiaqi Zhu
Title of targeted journal	Light: Science & Applications
Publisher	Springer Nature
Expected publication date	Nov 2022
Portions	Figure 4
Requestor Location	Harbin Institute of Technology<br>No. 92, Xidazhi Street, Nangang District<br><br>Harbin, 150080<br>China<br>Attn: Harbin Institute of Technology
Publisher Tax ID	EU826007151
Total	**0.00 USD**
Terms and Conditions	

TERMS AND CONDITIONS

This copyrighted material is owned by or exclusively licensed to John Wiley & Sons, Inc. or one of its group companies (each a"Wiley Company") or handled on behalf of a society with which a Wiley Company has exclusive publishing rights in relation to a particular work (collectively "WILEY"). By clicking "accept" in connection with completing this licensing transaction, you agree that the following terms and conditions apply to this transaction (along with the billing and payment terms and conditions

established by the Copyright Clearance Center Inc., ("CCC's Billing and Payment terms and conditions"), at the time that you opened your RightsLink account (these are available at any time at http://myaccount.copyright.com).

Terms and Conditions

- The materials you have requested permission to reproduce or reuse (the "Wiley Materials") are protected by copyright.

- You are hereby granted a personal, non-exclusive, non-sub licensable (on a stand-alone basis), non-transferable, worldwide, limited license to reproduce the Wiley Materials for the purpose specified in the licensing process. This license, **and any CONTENT (PDF or image file) purchased as part of your order,** is for a one-time use only and limited to any maximum distribution number specified in the license. The first instance of republication or reuse granted by this license must be completed within two years of the date of the grant of this license (although copies prepared before the end date may be distributed thereafter). The Wiley Materials shall not be used in any other manner or for any other purpose, beyond what is granted in the license. Permission is granted subject to an appropriate acknowledgement given to the author, title of the material/book/journal and the publisher. You shall also duplicate the copyright notice that appears in the Wiley publication in your use of the Wiley Material. Permission is also granted on the understanding that nowhere in the text is a previously published source acknowledged for all or part of this Wiley Material. Any third party content is expressly excluded from this permission.

- With respect to the Wiley Materials, all rights are reserved. Except as expressly granted by the terms of the license, no part of the Wiley Materials may be copied, modified, adapted (except for minor reformatting required by the new Publication), translated, reproduced, transferred or distributed, in any form or by any means, and no derivative works may be made based on the Wiley Materials without the prior permission of the respective copyright owner.**For STM Signatory Publishers clearing permission under the terms of the STM Permissions Guidelines only, the terms of the license are extended to include subsequent editions and for editions in other languages, provided such editions are for the work as a whole in situ and does not involve the separate exploitation of the permitted figures or extracts,** You may not alter, remove or suppress in any manner any copyright, trademark or other notices displayed by the Wiley Materials. You may not license, rent, sell, loan, lease, pledge, offer as security, transfer or assign the Wiley Materials on a stand-alone basis, or any of the rights granted to you hereunder to any other person.

- The Wiley Materials and all of the intellectual property rights therein shall at all times remain the exclusive property of John Wiley & Sons Inc, the Wiley Companies, or their respective licensors, and your interest therein is only that of having possession of and the right to reproduce the Wiley Materials pursuant to Section 2 herein during the continuance of this Agreement. You agree that you own no right, title or interest in or to the Wiley Materials or any of the intellectual property rights therein. You shall have no rights hereunder other than the license as provided for above in Section 2. No right, license or interest to any trademark, trade name, service mark or other branding ("Marks") of WILEY or its licensors is granted hereunder, and you agree that you shall not assert any such right, license or interest with respect thereto

- NEITHER WILEY NOR ITS LICENSORS MAKES ANY WARRANTY OR REPRESENTATION OF ANY KIND TO YOU OR ANY THIRD PARTY, EXPRESS, IMPLIED OR STATUTORY, WITH RESPECT TO THE MATERIALS OR THE ACCURACY OF ANY INFORMATION CONTAINED IN THE MATERIALS, INCLUDING, WITHOUT LIMITATION, ANY IMPLIED WARRANTY OF MERCHANTABILITY, ACCURACY, SATISFACTORY QUALITY, FITNESS FOR A PARTICULAR PURPOSE, USABILITY, INTEGRATION OR NON-INFRINGEMENT AND ALL SUCH WARRANTIES ARE HEREBY EXCLUDED BY WILEY AND ITS LICENSORS AND WAIVED BY YOU.

- WILEY shall have the right to terminate this Agreement immediately upon breach of this Agreement by you.

- You shall indemnify, defend and hold harmless WILEY, its Licensors and their respective directors, officers, agents and employees, from and against any actual or threatened claims, demands, causes of action or proceedings arising from any breach of this Agreement by you.

- IN NO EVENT SHALL WILEY OR ITS LICENSORS BE LIABLE TO YOU OR ANY OTHER PARTY OR ANY OTHER PERSON OR ENTITY FOR ANY SPECIAL, CONSEQUENTIAL, INCIDENTAL, INDIRECT, EXEMPLARY OR PUNITIVE DAMAGES, HOWEVER CAUSED, ARISING OUT OF OR IN CONNECTION WITH THE DOWNLOADING, PROVISIONING, VIEWING OR USE OF THE MATERIALS REGARDLESS OF THE FORM OF ACTION, WHETHER FOR BREACH OF CONTRACT, BREACH OF WARRANTY, TORT, NEGLIGENCE, INFRINGEMENT OR OTHERWISE (INCLUDING, WITHOUT LIMITATION, DAMAGES BASED ON LOSS OF PROFITS, DATA, FILES, USE, BUSINESS OPPORTUNITY OR CLAIMS OF THIRD PARTIES), AND WHETHER OR NOT THE PARTY HAS BEEN ADVISED OF THE POSSIBILITY OF SUCH DAMAGES. THIS LIMITATION SHALL APPLY NOTWITHSTANDING ANY FAILURE OF ESSENTIAL PURPOSE OF ANY LIMITED REMEDY PROVIDED HEREIN.

- Should any provision of this Agreement be held by a court of competent jurisdiction to be illegal, invalid, or unenforceable, that provision shall be deemed amended to achieve as nearly as possible the same economic effect as the original provision, and the legality, validity and enforceability of the remaining provisions of this Agreement shall not

be affected or impaired thereby.

- The failure of either party to enforce any term or condition of this Agreement shall not constitute a waiver of either party's right to enforce each and every term and condition of this Agreement. No breach under this agreement shall be deemed waived or excused by either party unless such waiver or consent is in writing signed by the party granting such waiver or consent. The waiver by or consent of a party to a breach of any provision of this Agreement shall not operate or be construed as a waiver of or consent to any other or subsequent breach by such other party.

- This Agreement may not be assigned (including by operation of law or otherwise) by you without WILEY's prior written consent.

- Any fee required for this permission shall be non-refundable after thirty (30) days from receipt by the CCC.

- These terms and conditions together with CCC's Billing and Payment terms and conditions (which are incorporated herein) form the entire agreement between you and WILEY concerning this licensing transaction and (in the absence of fraud) supersedes all prior agreements and representations of the parties, oral or written. This Agreement may not be amended except in writing signed by both parties. This Agreement shall be binding upon and inure to the benefit of the parties' successors, legal representatives, and authorized assigns.

- In the event of any conflict between your obligations established by these terms and conditions and those established by CCC's Billing and Payment terms and conditions, these terms and conditions shall prevail.

- WILEY expressly reserves all rights not specifically granted in the combination of (i) the license details provided by you and accepted in the course of this licensing transaction, (ii) these terms and conditions and (iii) CCC's Billing and Payment terms and conditions.

- This Agreement will be void if the Type of Use, Format, Circulation, or Requestor Type was misrepresented during the licensing process.

- This Agreement shall be governed by and construed in accordance with the laws of the State of New York, USA, without regards to such state's conflict of law rules. Any legal action, suit or proceeding arising out of or relating to these Terms and Conditions or the breach thereof shall be instituted in a court of competent jurisdiction in New York County in the State of New York in the United States of America and each party hereby consents and submits to the personal jurisdiction of such court, waives any objection to venue in such court and consents to service of process by registered or certified mail, return receipt requested, at the last known address of such party.

WILEY OPEN ACCESS TERMS AND CONDITIONS

Wiley Publishes Open Access Articles in fully Open Access Journals and in Subscription journals offering Online Open. Although most of the fully Open Access journals publish open access articles under the terms of the Creative Commons Attribution (CC BY) License only, the subscription journals and a few of the Open Access Journals offer a choice of Creative Commons Licenses. The license type is clearly identified on the article.

The Creative Commons Attribution License

The Creative Commons Attribution License (CC-BY) allows users to copy, distribute and transmit an article, adapt the article and make commercial use of the article. The CC-BY license permits commercial and non-

Creative Commons Attribution Non-Commercial License

The Creative Commons Attribution Non-Commercial (CC-BY-NC)License permits use, distribution and reproduction in any medium, provided the original work is properly cited and is not used for commercial purposes.(see below)

Creative Commons Attribution-Non-Commercial-NoDerivs License

The Creative Commons Attribution Non-Commercial-NoDerivs License (CC-BY-NC-ND) permits use, distribution and reproduction in any medium, provided the original work is properly cited, is not used for commercial purposes and no modifications or adaptations are made. (see below)

Use by commercial "for-profit" organizations

Use of Wiley Open Access articles for commercial, promotional, or marketing purposes requires further explicit permission from Wiley and will be subject to a fee.

Further details can be found on Wiley Online Library http://olabout.wiley.com/WileyCDA/Section/id-410895.html

Other Terms and Conditions:

v1.10 Last updated September 2015

Questions? customercare@copyright.com or +1-855-239-3415 (toll free in the US) or +1-978-646-2777.

Thermo Fisher
SCIENTIFIC

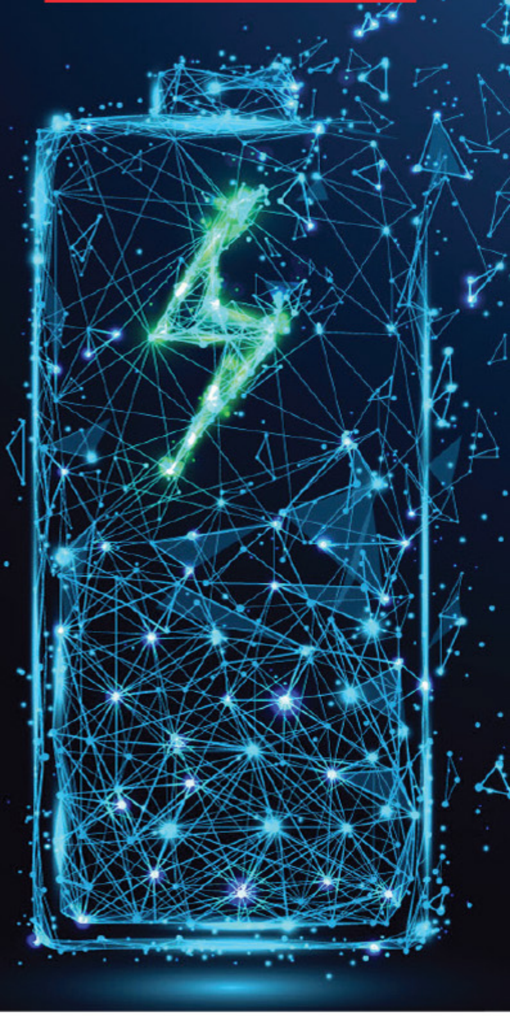

Lithium-ion battery

PowerUp webinars

Analytical solutions for lithium-ion battery material analysis and testing

PowerUp your battery material analysis - Join our educational webinars

Heading toward zero emission goals, lithium-ion battery are expected to generate an unprecedented demand for battery raw material in the upcoming decade. At the same time battery research, production, and quality control will need to keep up with the accelerated demand for electric storage capacities. Battery manufacturers must deliver consistently high quality throughout the entire battery value chain. Analysis and testing of batteries raw material and components requires therefore a variety of analytical methods that provide insights of quality and properties at various scales.

In this webinar session we will highlight the benefits of several analytical techniques and applications, as chromatography, mass spectrometry and electron microscopy, in battery material and structural analysis.

Free education webinars
October 5 and 6, 2022 | 10:00 BST | 11:00 CEST
Part 1: Analysis of cathode materials
Part 2: Analysis of battery electrolytes

No time for live session? Register anyway and get access to the on-demand recording after the live broadcast.

Key learnings

- Learn about analytical solutions for lithium-ion battery material and structural analysis
- Discover how IC, GC-MS, ICP-OES, and SEM support battery value chain from raw materials to recycling
- Get deeper insights based-on selected applications and performance examples

Learn more at **thermofisher.com/battery-webinars**

thermo scientific

For Research Use Only. Not for use in diagnostic procedures. © 2022 Thermo Fisher Scientific Inc. All rights reserved. All trademarks are the property of Thermo Fisher Scientific and its subsidiaries unless otherwise specified. **fl-001396-em-en**

Thirty Years of Luminescent Solar Concentrator Research: Solar Energy for the Built Environment

Michael G. Debije and Paul P. C. Verbunt*

Research on the luminescent solar concentrator (LSC) over the past thirty-odd years is reviewed. The LSC is a simple device at its heart, employing a polymeric or glass waveguide and luminescent molecules to generate electricity from sunlight when attached to a photovoltaic cell. The LSC has the potential to find extended use in an area traditionally difficult for effective use of regular photovoltaic panels: the built environment. The LSC is a device very flexible in its design, with a variety of possible shapes and colors. The primary challenge faced by the devices is increasing their photon-to-electron conversion efficiencies. A number of laboratories are working to improve the efficiency and lifetime of the LSC device, with the ultimate goal of commercializing the devices within a few years. The topics covered here relate to the efforts for reducing losses in these devices. These include studies of novel luminophores, including organic fluorescent dyes, inorganic phosphors, and quantum dots. Ways to limit the surface and internal losses are also discussed, including using organic and inorganic-based selective mirrors which allow sunlight in but reflect luminophore-emitted light, plasmonic structures to enhance emissions, novel photovoltaics, alignment of the luminophores to manipulate the path of the emitted light, and patterning of the dye layer to improve emission efficiency. Finally, some possible 'glimpses of the future' are offered, with additional research paths that could result in a device that makes solar energy a ubiquitous part of the urban setting, finding use as sound barriers, bus-stop roofs, awnings, windows, paving, or siding tiles.

1. Introduction

With the realities of dwindling oil reserves now affecting much of everyday life, world governments have increasingly turned towards renewable energy technologies in an attempt to fill the ever-increasing gap. In particular, there is an increasing interest in bringing solar energy systems to the built environment. Buildings account for about 40% of total energy use, including 70% of total electricity use and 40% of emissions in more developed countries,[1] and about 25% of all energy use globally,[2] much of this related to our inability to control sunlight: tremendous energy resources are used to both heat and cool buildings and provide artificial lighting systems.[1] The scene of an office with shuttered windows and burning lights in the daytime is familiar to everyone.

The European Committee has decreed that all new buildings be near-zero energy by 2020.[2] This demands that architects and the building industry integrate energy saving and energy generation into the design of new buildings. This puts considerable pressure on the architect wishing to meet these requirements: how to create buildings both pleasing to the eye, yet energetically neutral? To give the maximum freedom in their designs, the devices incorporated to save and/or generate energy have to be easily implemented into the design and adaptable to the situation. Adaptability could also make retrofitting of existing structures during renovations easier and cheaper. One energy source that is readily available in a built environment is the sun, which is a clean, safe, inexhaustible, and reliable energy source.

Adapting traditional silicon-based photovoltaics (PVs) for urban integration has proven difficult. Some examples are shown in **Figure 1**a. PV cells remain relatively expensive.[3] Often the modules are heavy and limited in coloration (usually black or dark blue) and shape (rectangular): variability in both size and shape are generally lacking in the marketplace.

Several techniques have been adopted to add color to photovoltaic cells, including adding fluorescent dyes to the encapsulation layer,[4] adjusted color-reflecting films,[5] use of organic photovoltaics[6] and other techniques, all with their own advantages, but also carrying disadvantages. For example, color-reflecting films can reduce performance[5] and organic cells still suffer from reduced efficiencies and lifetimes, although progress is being made.[7–9] If transparency is desired for a window fitting, for example, this capability is limited in traditional solar panels, and requires use of thin-film cells, standard solar cells arranged with physical spaces between them (see Figure 1b), or cells with holes drilled through them. Furthermore, PV cells respond optimally to direct sunlight, while in the built environment much of the sunlight is diffuse due to scattering and reflections by other objects such as trees, other buildings, and even clouds, and shading is common, which reduces the performance of

Dr. M. G. Debije, P. P. C. Verbunt
Functional Organic Materials & Devices
Department of Chemical Engineering and Chemistry
Eindhoven University of Technology
5600 MB Eindhoven, The Netherlands
E-mail: m.g.debije@tue.nl

DOI: 10.1002/aenm.201100554

© 2012 WILEY-VCH Verlag GmbH & Co. KGaA, Weinheim

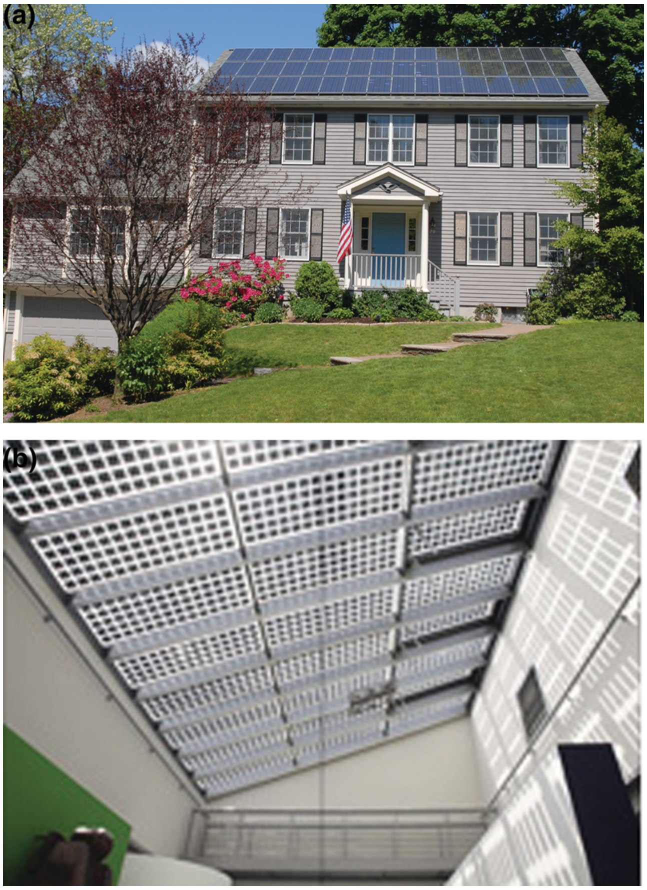

Figure 1. a) Photograph of silicon-based panels mounted on a rooftop (photo by Gray Watson). b) Photograph of an atrium element using semi-transparent silicon-based solar panels (photo courtesy Sapa Building Systems).

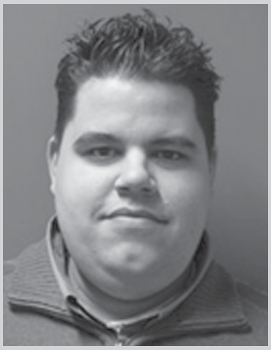

Paul P. C. Verbunt received a Bachelor of Applied Sciences degree in Chemistry from the Fontys Hogeschool Technische Natuurwetenschappen Eindhoven, in 2002. After a couple of years in industry he received a M.Sc. degree in Chemical Engineering and Chemistry from the Eindhoven University of Technology in 2008. He is currently active as a PhD student in the Functional Organic Materials and Devices group of the Chemical Engineering and Chemistry department at the Eindhoven University of Technology, lead by Prof. Dirk Broer. His current research interests are focused on luminescent solar concentrators.

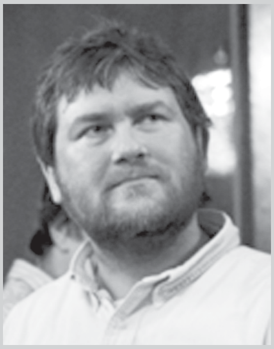

Michael G. Debije received a M.Sc. in High Energy Physics from Iowa State University, Ames, Iowa in 1994 and a Ph.D. in Biophysics from the University of Rochester in Rochester, NY, USA in 2000. After completing a postdoc at the Interfaculty Reactor Institute at the Delft University of Technology in the group of John Warman in 2003, he joined the staff of the Functional Organic Materials and Devices group under Prof. Dirk Broer at the Eindhoven University of Technology, and is responsible for the Energy cluster within the program.

an extended PV system significantly.[10,11] Even many of the new thin-film technologies will be a challenge to adapt to the demands of the architect and society.

In order to combat the high costs associated with solar-energy generation, a multitude of designs have been introduced to concentrate sunlight from large areas onto smaller, more efficient PV cells, thereby reducing the surface coverage of the expensive PV cells.[12] There are predominantly two variations on the concentrator system, those relying on light focusing, and those with associated reflectors for concentrating the sunlight. Both concentrating systems demonstrate high degrees of light concentration and a concurrent reduction in the price of the generated solar electricity.[13]

There are many utilization limitations imposed by these concentrator systems. For one, they often need to be installed with either single- or double-directional tracking systems to trace the path of the sun across the sky, which adds to their expense and complexity. They tend to perform less well in indirect sunlight, and thus are restricted then to areas with minimal cloud cover and shading. Often the installations are quite extensive, require a large area of land, suffer from the 'not in my back yard' effect, and are not easily adaptable to a built-up area. Alternative concentrators using nonimaging, nontracking, flatter devices suitable for building integration tend to be restricted to lower concentration ratios.[14]

1.1. Introduction to the Luminescent Solar Concentrator

The desire, then, is to take advantage of a reflective or focusing solar concentrator (collecting light from a larger area and focusing it onto a smaller area) while avoiding the disadvantages (the need for direct sunlight, tracking, and often awkward dish-like shapes and large sizes) for integration in urban environments. An alternative to traditional semiconductor devices that takes the advantages of concentrator systems but which could still be used in the built environment could be the luminescent solar concentrator (LSC) (**Figure 2**), originally introduced more than three decades ago. The earliest reference to an LSC type device architecture appeared as a grant proposal in 1973 by Lerner,[15] soon followed by papers in the general literature.[16–18] There was a great flurry of activity in the field,

 © 2012 WILEY-VCH Verlag GmbH & Co. KGaA, Weinheim

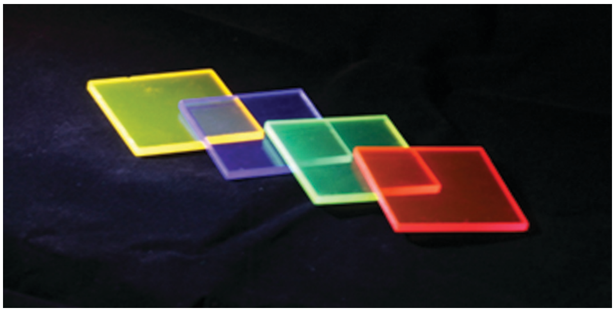

Figure 2. Photograph of four luminescent solar concentrators illuminated by ultraviolet light.

leading to a plethora of patents. The drop in oil prices in the 1980s led to a near-abandonment of the research. Recent surges in oil prices and increased awareness of the effects of power generation from traditional fuels on the global environment have revitalized research on the LSC, and it has again become the interest of many groups around the world.

The past five years have demonstrated a considerable reawakening of interest in the LSC. This has been due to a number of factors, including the increasing awareness by the general public of the dwindling fossil-fuel availability, limitations on the performance and thus capability of deployment in urban areas for traditional PV systems and the desire from the architectural and building sectors for more freedom in their design choices.

The LSC promises potential low costs of production.[19,20] They could provide adaptability to the needs of the architect in that they can be made in a variety of colors, shapes, and transparencies, could be made flexible, and should weigh less than silicon PV panels, for example, which could make LSCs more viable for mounting to the side of a building.[21] In addition, the LSC could function well in both direct and diffuse light,[22,23] of particular interest for countries with frequent cloud coverage or areas of persistent shady conditions, such as are typical in cities, for example. If the photon in/photon out efficiency is high enough, the cost of electricity generated by the LSC could be competitive.[19] While the efficiency of an LSC will be lower than an equivalent area of a silicon PV due to decreased spectral usage, the reduced cost and tremendous flexibility in design could make them a viable alternative for the urban area where it would be too expensive, inappropriate, or impractical to use standard solutions.

1.2. Working Principle of the Luminescent Solar Concentrator

The basic LSC design allows sunlight to penetrate the top surface of an inexpensive plastic or glass waveguide. This light is absorbed by luminescent molecules (which could be organic dyes, inorganic phosphors, or quantum dots, as we describe later in this review), which are embedded in the waveguide, applied in a separate layer on the top or bottom of the waveguide, or contained in a liquid solution between two glass plates.[17,24–26] The absorbed light is re-emitted at a longer wavelength and a fraction of the re-emitted light is trapped in the waveguide by total internal reflection, becoming concentrated along the edges of the plate. Small PV cells can be attached to the edges of the waveguide to collect the light and convert it to electricity. **Figure 3** summarizes the functionality of the device.

Other flat-plate concentrating systems that do not rely on luminescence have been proposed. These systems have a variety of forms, for example, relying on diffractive elements,[27] light trapping,[28] special backside mirrors,[29] or refractive-index variations.[30] However, each of these designs presents their own challenges to produce electricity efficiently enough to be economically feasible. This paper will confine itself to describing research on systems incorporating luminescent materials into the waveguide.

The portion of the emitted light that is trapped inside the waveguide depicted in Figure 3 is determined by the refractive index of this waveguide. According to Snell's law, all photons approaching an interface between a material and air at an angle higher than the critical angle will be totally reflected. This critical angle is defined as

$$\theta_c = \sin^{-1}\left(\frac{1}{n}\right) \tag{1}$$

where n is the refractive index of the waveguide. This means that for an isotropic emitter and a waveguide with a refractive index of 1.5–1.6, approximately 75% of all emitted photons will be internally reflected. These photons will waveguide towards the side of the LSC where a significant fraction is coupled out of the waveguide by a photovoltaic cell which transforms the photons in to electrons. However, the efficiency of an LSC is not only dependent on the trapping efficiency of the waveguide. One description of the optical efficiency, η_{opt}, of an LSC is

$$\eta_{opt} = (1 - R)\, P_{TIR} \cdot \eta_{abs} \cdot \eta_{PLQY} \cdot \eta_{Stokes} \cdot \eta_{host} \cdot \eta_{TIR} \cdot \eta_{self} \tag{2}$$

where R is the reflection of solar light from the waveguide surface, P_{TIR} is the total internal reflection efficiency, η_{abs} is the fraction of solar light that is absorbed by the dye, η_{PLQY} is the photoluminescent quantum yield of the used luminophore(s), η_{Stokes} is the energy lost due to the heat generation during the absorption and emission event, η_{host} is the transport efficiency of the waveguided photons through the waveguide, η_{TIR}

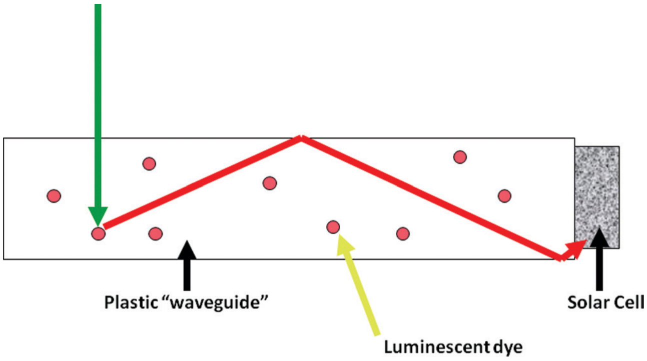

Figure 3. The luminescent solar concentrator. Incoming sunlight (green arrow) enters the waveguide and is absorbed by a luminophore. The light is re-emitted at a longer wavelength (red arrow) and a fraction becomes trapped by total internal reflection. Attached to the edge(s) of the waveguide are photovoltaic cells for conversion of the light into electricity.

© 2012 WILEY-VCH Verlag GmbH & Co. KGaA, Weinheim

is the reflection efficiency of the waveguide determined by the smoothness of the waveguide surface, and η_{self} is the transport efficiency of the waveguided photons related to re-absorption of the emitted photons by another luminophore.[17] The losses which influence all these efficiencies will be described in the second part of this review as well as the solutions proposed by several research groups over the past thirty years.

Thermodynamics limit conventional geometric concentrators, which consequently are inefficient at concentrating diffuse light.[31,32] These devices demonstrate maximum concentration at close to the square of the refractive index of the material in which the concentrator is embedded. Therefore, these concentrators are not very useful in a built environment, where the sunlight is very diffuse due to scattering from buildings, cars, or trees.

The absorption and re-emission of photons in LSCs changes the entropy of the system; the maximum concentration of an LSC is therefore dependent on the heat generation during the absorption and emission event, and thus from the Stokes shift of the luminescent molecules. The maximum concentration can be approximated by:

$$C \approx \frac{e_2^3}{e_1^3} \exp \frac{e_1 - e_2}{kT_0} \tag{3}$$

where e_2 and e_1 are the photon energies of the emitted and absorbed photons, respectively, k is Boltzmann's constant, and T_0 is the ambient temperature.[31,32] For a luminophore with a Stokes shift of 0.2 eV, the maximum concentration is approximately 2000 times.

Considerable effort has been put forth developing a wide range of models using many different approaches to describe the results of the existing devices, as well as to predict optimal, higher-efficiency LSC designs. Random-walk theory has been used to illustrate re-absorption,[33] and Monte Carlo simulations have been used to investigate the performance of single- and double-film stacked LSCs.[23,34] Different thermodynamic models have examined waveguide losses[32,35] which report results in very good agreement with experiments on test modules. Recently, Meyer et al. have compared the re-absorption probability model reported by Weber et al. to ray-tracing results of luminophore-impregnated thin-film and liquid-concentrator systems.[36] Other work has used ray tracing to predict the performance of LSCs, including filled[37–40] and thin-film[41] waveguides.

Each of the theoretical approaches has their advantages and disadvantages. The thermodynamic modeling requires minimal input and respond with swift answers but is often limited to simpler geometries and limited luminescent species.[40] Ray tracing allows much more freedom in device design and number of luminophores and other details, but is quite computationally demanding. Potentially high performance levels for the LSC have been predicted by, for example, detailed balance theory of a single stage fluorescent collector using a high-efficiency luminophore and wavelength-selective surface reflectors. Such a device was predicted to demonstrate efficiencies of up to 90% that of a directly illuminated cell of equivalent size.[42]

There have also been efforts to use the concentrated light from an LSC to generate high temperature thermal heat. In these experiments, LSC plates were attached to either side of a surface oxidized copper pipe inside an evacuated glass tube which acted as the collector. When exposed to external conditions of 980 W m^{-2} total illumination, a stagnation temperature of 535 °C was reached, and on a cloudy day (ambient temperature of 26 °C) the temperature of the absorber reached in excess of 250 °C.[43]

2. Losses of Luminescent Solar Concentrators and Proposed Solutions

The LSC has not yet been extensively commercialized, primarily owing to their modest efficiencies.[44,45] A diagram depicting a number of identified loss mechanisms for LSCs taking into account the factors described in Equation (2) above may be graphically depicted in **Figure 4**.

The first loss of the LSC is light emitted by the luminophore under an angle which is refracted out of the waveguide through an 'escape cone' rather than being reflected internally (Figure 4, 1). The second loss is due to the re-absorption of emitted photons in the waveguiding mode by subsequent luminophores due to an overlap of the emission and absorption bands of the luminophores (limited Stokes shifts, Figure 4, 2). The third loss of an LSC is a combination of losses related to the luminophore used: one is that the luminescent molecules have limited spectral absorption bands which lead to incomplete incident light absorption (Figure 4, 3a). This light simply passes through the waveguide and is lost through the bottom surface. Another loss related to the luminophore is the absorption of high energy UV photons that leads to either direct photodegradation of the molecules (Figure 4, 3b) such that in time a fraction of the molecules will be broken down and total emission of the LSC will decrease, or degradation of other molecular species within the vicinity of the luminophore that subsequently react with it and degrade the luminophore performance. Some of the absorbed photons are not re-emitted by the luminophores due to limited emission quantum yield, but instead lost as heat and vibrations (Figure 4, 3c).

The PV cell at the waveguide edge has a nonuniform spectral response, with a fraction of incident photons being lost due to the finite conversion efficiency of the PV cell leading to a

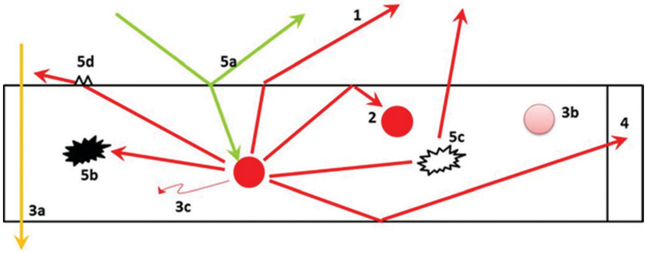

Figure 4. Loss mechanisms in luminescent solar concentrators: 1) Light emitted outside capture cone; 2) re-absorption of emitted light by another luminophore; 3a) input light is not absorbed by the luminophore; 3b) limited luminophore stability; 3c) internal quantum efficiency of the luminophore is not unity; 4) solar cell losses; 5a) reflection from the waveguide surface; 5b) absorption of emitted light by the waveguide; 5c) internal waveguide scattering; 5d) surface scattering.

© 2012 WILEY-VCH Verlag GmbH & Co. KGaA, Weinheim

fourth loss (Figure 4, 4). Even optically perfect waveguides can suffer from losses; a small part of the input light is lost through reflection from the surface of the waveguide, due to Fresnel reflections (Figure 4, 5a). Waveguides used for LSCs can cause losses through parasitic absorption, especially in the near infrared (Figure 4, 5b). Imperfections of the waveguide surface can cause photons in the waveguide mode to leave the surface and be lost (Figure 4, 5d), while imperfections in the waveguide bulk also scatter waveguided photons (Figure 4, 5c), leading to additional surface losses.

In the next section of this paper we give an overview of research done on LSCs towards reducing these losses since their discovery in the 1970s.

2.1. Surface Loss

Photons emitted by luminescent molecules inside the escape cone will be lost through the surfaces (loss 1 in Figure 4). Measurements suggest that 40–55% of all absorbed energy is lost in this way (this translates into a 50–70% loss of photons) in a 5 cm × 5 cm device;[46] these high loss results have been supported by simulations.[40,47] Surface loss is key in LSCs, and in the last few years multiple groups have done research on minimizing them by two main processes: aligning the luminophores and the application of selective mirrors.

2.1.1. Aligned Luminophores

Organic luminophores are often dichroic in absorption and transmission,[48–52] which opens new possibilities in controlling the spatial distribution of emitted light, provided that the physical ordering of the dyes is macroscopically controlled. The alignment of dichroic dyes in liquid crystalline (nematic; LC) materials has been previously investigated[53–56] and it has been shown that macroscopic alignment of the dichroic dyes in the LC host resulted in anisotropy and dichroism in both absorption and emission. There are a number of possible ways of aligning the luminophores, such as using self-aligning fluorescent nanorods,[57] incorporating the materials in a polymeric sheet which can be physically stretched to impose alignment,[58] or mixture in a host that itself may be spontaneously aligned, such as a liquid crystal.[59] See **Figure 5** for a depiction of some of the dye orientations possible using liquid crystals.

Aligning organic dyes perpendicular to the plane of the waveguide surface (homeotropically, Figure 5b) using a host (photopolymerizable) liquid crystal leads to an emission primarily in the plane of the waveguide, resulting in a sharp decrease of surface loss to less than 10%,[60,61] confirmed by simulations using collimated light.[47] However, in this configuration the luminophores have quite a low absorption, as their transition dipole for absorption is parallel to the direction of the incoming light. Lower absorption naturally leads to lower edge emission, although the trapping efficiency of emitted photons increases from ≈65% to over 80% when vertically aligned luminophores in LSCs are excited by an isotropic light source.[62,63] The efficiency of transmission of the emitted photons to the edge of the waveguide can increase with close-to homeotropic alignment, as the path length through the luminophore-containing region of the thin absorbing/emitting layer is minimized.[64] Completely homeotropic alignment, however, could be detrimental, as most of the emitted light would be directed within the thin luminophore layer, and would result in excessive re-absorption events.

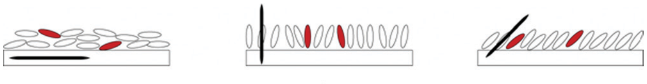

Figure 5. Schematic depiction of red luminophores aligned in nematic liquid crystals on top of a waveguide. The black arrow depicts the average alignment direction: a) planar alignment, b) homeotropic alignment, and c) tilted alignment at 45°.

Liquid crystals have also been used to align the luminophores parallel to the waveguide surface, also known as planar alignment (Figure 5a). In this configuration, one can relatively easily determine the degree of alignment of the luminophores by determining the dichroism parameter in absorption, R_a, for the embedded luminophores through use of the equation:

$$R_a = \frac{A_{par} - A_{perp}}{A_{par} + 2A_{perp}} \tag{4}$$

where A_{par} and A_{perp} are the peak absorbance of the luminophore measured using incident light polarized parallel and perpendicular to the alignment direction of the liquid crystals, respectively. A system in perfect alignment would demonstrate an R_a of 1, while a completely isotropic system would have an R_a of 0.

When fluorescent organic dyes exhibit a high degree of alignment in absorption (that is, $R_a > 0.5$), emission is directed such that >30% more energy is issued from the two edges parallel to the alignment direction of the dye molecules when compared to the edge output from any side of an LSC using no dye alignment (isotropic).[59] The total emission from all four waveguide edges is the same for both planar and isotropic devices, suggesting the surface losses have not been reduced. An LSC configured with this planar alignment could be used as an energy harvesting polarizer[63] for use in display applications, for example. Additionally, if light is emitted primarily towards just two edges, the number of PV cells on the LSC can be reduced to two or even one, reducing the cost of the LSC still further. Surface losses, as one might expect, are not reduced in this configuration.[60]

An obvious next step is to consider combining the reduced surface losses of the homeotropic orientation and the enhanced emission directions of the planar samples and apply the dyes in a tilted alignment (Figure 5c).[60,65] There are a number of potential ways to achieve tilted alignments in liquid-crystal systems. One technique is to use modified alignment layers that can be specifically tuned by rubbing pressure or processing temperature to achieve the desired angular tilt[66] or by using liquid-crystal phases that themselves have a natural tilt, such as the Smectic C phase. However, to date these approaches have proven difficult to produce sufficiently defect-free alignment over an extended area: work on this continues.

© 2012 WILEY-VCH Verlag GmbH & Co. KGaA, Weinheim

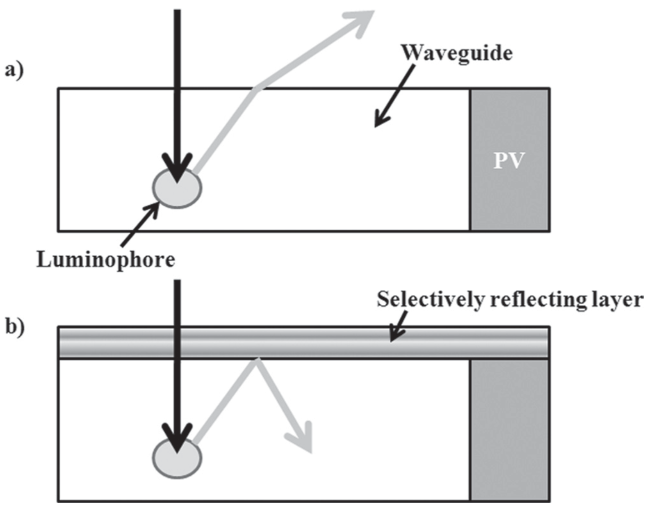

Figure 6. a) Standard LSC. Input light (black arrow) is absorbed and emitted by the luminophore outside the 'capture cone' of the waveguide (gray arrow) and is lost through the surface. b) The same LSC with application of the selective reflector. Incident light (black arrow) is absorbed by the luminophore. The longer-wavelength emitted light (gray arrow) is directed outside the 'capture cone', but is reflected by the selective reflection layer.

2.1.2. Selective Mirrors

A second way to reduce surface loss is by the application of wavelength-selective mirrors, the purpose of which is to allow incoming light to enter the waveguide and be absorbed, but to be reflective for wavelengths emitted by the luminophore[40,42,45,67–76] (see **Figure 6** for the illustrated concept). Simulations have suggested that, in some cases, an ideal top-surface reflector could increase edge output more than 50%.[40] Wavelength selective mirrors so far employed have been made from chiral nematic (cholesteric) liquid crystals,[75] inorganic dichroic Bragg reflectors[40,67] and Rugate filters.[73]

A cholesteric liquid crystal is a planar nematic liquid crystal that has been doped with a chiral molecule. The chiral molecule induces a 'twist' between adjacent layers of the liquid crystal, the twist direction (either right- or left-handed) being dependent on the nature of the chiral dopant. This results in the formation of a type of helical structure through the depth of the layer which is capable of reflecting a narrow bandwidth of light of the same polarization handedness as the helix (that is, right-handed cholesterics will reflect right-circularly polarized light). The wavelength of light reflected is defined by the 'pitch' of the helix, given by

$$\lambda = n_{av} p \sin\theta \qquad (5)$$

where λ is the central reflection wavelength, n_{av} is the average refractive index of the liquid crystal host, p is the pitch, or the distance it takes for the liquid crystal director of a plane of molecules in the helix to undergo a 360° twist, and θ is the incidence angle of the light with respect to the normal.[77] The reflection bandwidth of the cholesteric material will be given by

$$\Delta\lambda = (n_e - n_o)\, p \qquad (6)$$

where n_e and n_o are the extraordinary and ordinary refractive index of the host LC.[78] For most standard LCs, the bandwidth is on the order of 50–75 nm in the visible part of the spectrum. By addition of photo-responsive end groups, it is possible to crosslink the liquid crystals to form solid films. See **Figure 7** for a depiction of the cholesteric liquid crystal.

Naturally, a single cholesteric layer would only be able to reflect half of the incident sunlight (which is equally divided between right- and left-circular polarizations), so to get complete reflection of incoming light a second cholesteric layer employing a left-handed helical structure would need to be added. An advantage of these cholesteric systems is their ease of application: they may be cast or sprayed from solution and self-assemble into the reflective state.

By applying full, narrow band reflecting cholesterics to the top surface of Lumogen F Red 305-containing waveguides (a commercial dye from BASF), up to 30% of the light that had previously escaped the surface was turned into edge emission, translating into a 12% LSC output improvement using the organic reflectors.[75] In these experiments, the reflectors were separated from the waveguide by an air gap. The reflection band which produced the highest enhancement of the edge output was considerably red-shifted with respect to the emission wavelength of the dye. It was shown the fractional increase in emission was higher for waveguides containing a lower dye concentration.

Similar enhancements to LSC edge outputs were determined using inorganic reflectors.[45,73] In this work, waveguides containing the dye BA241 were topped by a Rugate filter, which is a photonic band stop filter with a sinusoidal varying refractive index, separated by an air gap from the top of the waveguide. The reflection bandwidth of the filter used was on the order of 150 nm. By applying the filter to the waveguide, the relative efficiency increased by 20% (see **Figure 8**).

An advantage to using the organic reflectors is that they are cast from solution and spontaneously form the reflective layer: this is generally a much simpler and less expensive process than the application of multilayer inorganic Bragg reflectors. However, as mentioned earlier, the reflection bandwidth of standard cholesteric liquid crystals is limited. In general, the emission spectra of the luminophores is often much broader than the 75 nm reflection band generally present in cholesteric LCs. One obvious way to increase performance of these organic reflectors is by broadening the reflection band.[76] The reflection bandwidth of a cholesteric liquid crystal is determined in part by the difference between the extraordinary and ordinary refractive indices of the liquid crystal used, which is generally limited. Thus, one way to increase bandwidth is to use host liquid crystals with a higher Δn (birefringence), but the bandwidth is still limited to perhaps 150 nm by the birefringence of the host LCs, generally not extending beyond $\Delta n = 0.4$.

Another option is to layer like-handed cholesterics, each offset from its neighbor by 75 nm, creating a stack. Of course, given the single-handedness of the cholesteric, this would necessitate at least four layers (two stacked left-handed and two stacked right-handed) to achieve a 150 nm reflection band. A third option is to employ gradient-pitch cholesterics.[78] In

© 2012 WILEY-VCH Verlag GmbH & Co. KGaA, Weinheim

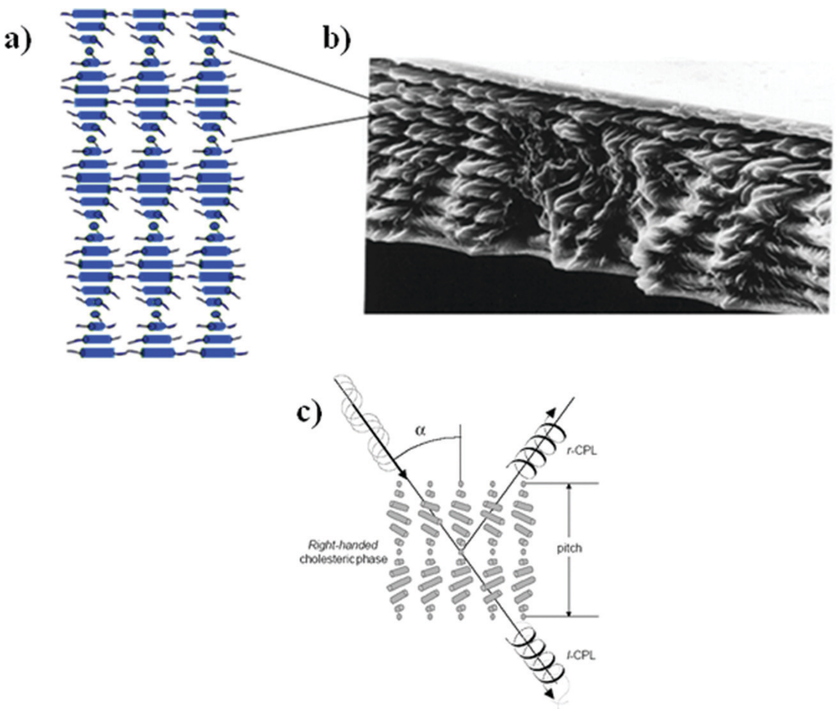

Figure 7. Cholesteric wavelength selective mirror. a) Schematic depiction of the liquid crystal alignment, b) scanning electron microscope image of the cross-section of a cholesteric film, c) depiction of the functionality of the cholesteric layer.

these cholesterics, there is a continually varying pitch through the thickness of the cholesteric layer allowing reflection bandwidths of hundreds of nanometers. The primary challenge in making use of gradient pitch cholesterics is that they are generally created by filling a glass cell with the LC and exposing to ultraviolet light, and relying on a gradient dose of UV through the sample (achieved by adding a UV absorber to the LC) and using a mix of monoacrylate and diacrylate monomers for the host LC and chiral dopant. This introduces a glass top layer that is generally undesirable. Measurements of broadband reflectors made of multiple narrow band structures would be more easily produced.

A challenge facing the use all of these filters is that there is a pronounced angular dependence on the reflection band of the filter. This is very visible for the cholesteric liquid crystal system via Equation (6) above. At angles of incidence deviating from the normal, the reflection band shifts to the blue.[77,79] This becomes a problem for luminophores with restricted Stokes shifts, as the blue-shift may result in rejection of incoming light. Evaluation, therefore, of the LSC using selective reflectors must be careful to also consider the effect of incident light angle on the system performance. In general, the reflection band placement will be shifted to the red of the actual emission peak of the luminophore used.[40,75,76]

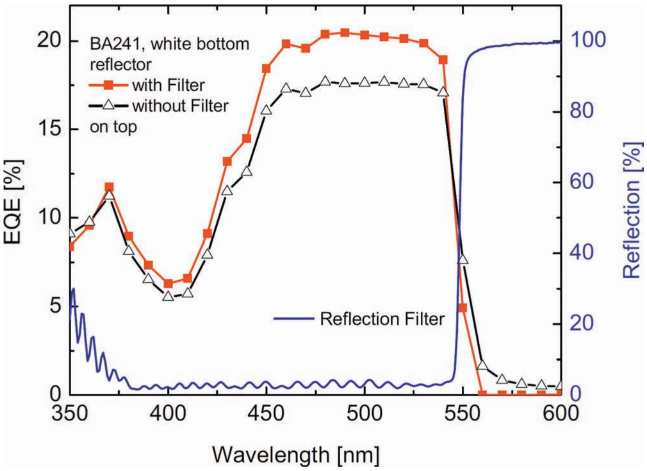

Figure 8. The measured external quantum efficiency (EQE) of a polymeric waveguide containing a dye before (black symbols) and after (red symbols) application of a Rugate filter. Reproduced with permission.[73]

2.2. Re-Absorption of Emitted Photons by Other Luminophores

The Stokes shift of a luminophore is described as the wavelength separation between the peak of the most red-shifted absorption band and the most blue-shifted emission band. Most organic fluorophores used in LSCs have quite small Stokes shifts, leading to relatively large overlaps between the absorption and the emission spectrum.[80] As a consequence, fluorophore-emitted photons can be re-absorbed by subsequent fluorophores encountered during transportation through the waveguide. These re-absorptions are not necessarily a loss themselves, but become a loss if the reabsorbing fluorophore does not again emit a photon (due to nonradiative relaxation pathways) or if it does emit, but in a direction within the escape cone of the waveguide.[33,81–84]

One manner in which the problem of re-absorption events has been approached is in the design of new luminophores with larger Stokes shifts. By reducing the overlap in the absorption and emission spectra of the luminophores, the re-absorption losses are minimized. A number of different molecular species with increased Stokes shift have been employed in the LSC, including lanthanides,[85] phosphors,[86] bipyridyls,[25] and quantum dots.[87–91] These classes of luminophore bring additional challenges to the production of LSCs. Inorganics often suffer from low solubility in organic matrices, and often also display a lower absorption. More on luminophore research is described in Section 2.3.

Another technique to reduce the amount of re-absorption is to introduce a polar and highly mobile material (in this case, thionin) as a dopant while forming the luminophore-containing polymer waveguide plates. In this work it was postulated that the dopant assisted by increasing the separation of the absorption and fluorescence bands of the molecules by altering the electronic states around the luminescent molecules in the

© 2012 WILEY-VCH Verlag GmbH & Co. KGaA, Weinheim

polymeric plate, thereby increasing the Stokes shift and reducing the overlap between the absorption and emission spectra.[92]

Another option to reduce the re-absorption losses in devices using more than one luminophore is to physically stack a number of waveguides, each containing a luminophore that absorbs in a different part of the spectrum, on top of one another.[17,20,93] This is particularly useful when one wishes to include one of the luminophores with absorption approaching the infrared, which to date have generally demonstrated lower fluorescent quantum yields. In this way, by stacking the more efficient luminophores on top, the light is collected, converted and transported to the attached cell without ever encountering the reduced-efficiency luminophore. The added benefit is that luminescence emitted through the escape cone could be captured by a luminophore in an adjacent waveguide rather than being lost.[93]

To reduce encounters of emitted light with the luminophores, one may attach the luminophores in a thin layer (from submicrometer thickness to hundreds of micrometers) to the surface of the waveguide rather than filling the luminophore within the bulk of the waveguide,[20,26,40,41,48,49,94–98] or as multiple thin layers stacked on one another.[99] In this way, emission light may be transported predominantly in the clear host material, and only encounters the luminescent layer with every second internal reflection. Theoretically, the thin layers should perform as well as the filled waveguides.[100] In practice, care must be exercised as the limited solubility of the luminophores often results in an underperformance of the thin layers due to aggregation of the luminophores, creating nonemitting absorption centers.[41]

An advantage in utilizing the thin luminophore-filled layer on top of a blank waveguide is in production: one could envisage the large-area, inexpensive application of thin layers on either glass or polymer hosts using a variety of techniques (including spin coating,[41] bar coating, casting,[40] sol–gel techniques,[101] doctor blading, spraying, Langmuir–Blodgett techniques,[26] or printing). The thin layers could consist of luminophores in acrylates,[102] cellulosetriacetate,[40] polymerized liquid crystals,[59] or a host of other materials.

It is also possible to reduce the losses due to the Stokes shift by using more than one luminophore, and employing Förster resonance energy transfer (FRET). FRET is the direct exchange of energy from an excited molecule to another nearby molecule without the emission of a photon. FRET is very sensitive to distance and other factors: the probability of a transfer being related to the orientations of the interacting molecules as well as the distance (with a sensitivity related to $1/R^6$ where R is the distance between the molecules).

By mixing a number of luminophores at high concentrations it was possible, through a chain of virtual emissions and absorptions, to transfer short-wavelength input light into long-wavelength output light at relatively high efficiency.[20,103] However, the high luminophore concentrations necessary for the FRET effect are often not achievable unless the molecules are brought closer together by other means.[104,105] Another material design taking advantage of FRET use ordered dye–nanochannel antennae (based on zeolites), which could allow enhanced directionality of light emission as well as reduced re-absorption losses.[106,107]

Another option to try to reduce re-absorption was to physically space thin luminophore layers with stretches of empty waveguide using spatially separated patterns of luminophores, so that the number of re-encounters that emitted light could have with other molecules was reduced: a depiction of the functionality of such a device is shown in **Figure 9**.[108] The transport efficiency of the photons through the LSC increased as the overall fluorescent-dye coverage decreased from 100% to 20%, and was relatively independent of the shape of the dye pattern. However, reduction in total light absorption due to the physical gaps between absorption regions resulted in a total system decrease in output, as one might expect.

In order to increase the amount of light absorbed in a patterned system, a lens array on top of the LSC is being developed that would increase the system output.[109] The lenses would focus incoming light from a wide area onto small, patterned regions of dye on the waveguide surface, leading to improved dye absorption. Current lens designs have demonstrated relatively efficient focusing of light over a range of ±30°. By using illumination through the lenses themselves to induce crosslinking of the dye-containing acrylate layer, one would be able to tailor the dye pattern to the expected solar light positions and so effectively minimize the area coverage requirement, as well as eliminate the difficulty of correctly aligning a separately produced lens array with a dye pattern. While this device demonstrates increased efficiency under direct lighting, measures still need to be taken to improve performance in diffuse light.

A very recent approach has used resonance shifting, where spacially confined emission from a bilayer cavities interacts off-resonance with the luminophore layer upon subsequent interactions. This is accomplished by careful variation of the luminophore thin-film thickness across the area of the waveguide. The thickness variation is on the order of a few tens of nanometers and is most appropriate for samples with luminophore layers on the order of a micrometer in thickness. In this way, initial work appears to allow light propagation with practically no

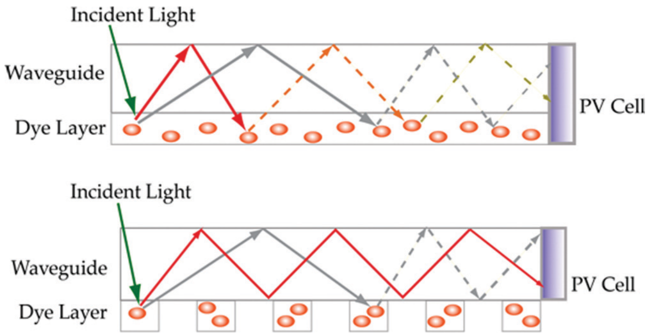

Figure 9. Schematic demonstrating the functionality of the patterned fluorescent dye waveguide. Top: Function of the standard thin-layer LSC system. Incident light (green) is absorbed by a fluorophore and emitted at a longer wavelength (red, gray solid lines) and has a significant probability of re-encountering a subsequent dye molecule and being reabsorbed, and possibly again re-emitted (dotted red, gray lines). Bottom: With the patterned dye layer, some of the emission light (red) will travel along the waveguide and never re-encounter the dye layer, leading to enhanced probability of avoiding re-absorption.

© 2012 WILEY-VCH Verlag GmbH & Co. KGaA, Weinheim

re-absorption losses, and as such promises potentially significant performance improvements if the design may be simply and reproducibly applied.[110]

2.3. Luminophores: Limited Absorption, Stability, and Luminescence Efficiency

Luminophores are the driving force for concentration in luminescent solar concentrators. Due to their absorption and re-emission, luminophores decouple the incoming sunlight from the emission light, overcoming the limitations of geometrical optics in trapping light within waveguiding modes. The absorption and re-emission of the luminophores alter the entropy of the LSC, so higher light concentrations can be reached, in accordance with thermodynamic laws (étendue).[31,32] According to the laws governing étendue (Equation (3)) the maximum concentration achievable by the waveguide is determined by the Stokes shift of the luminophore used. Besides the maximum concentration of an LSC, the luminophore is the most determining factor of light transport efficiency. In Goetzberger's equation (Equation (2)) for LSC efficiency, the fraction of solar light absorbed, the photoluminescent quantum yield, the energy lost due to the heat generation during the absorption and emission events, the transport efficiency of the waveguided photons related to re-absorption of the emitted photons, and the spectrum of the photons reaching the PV cell are all determined by the characteristics of the luminophore. Combining the needs for maximum concentration, maximum efficiency, and maximum lifetime, the luminophore is the single most important component in the device. An effective luminophore must meet all the following requirements:

- Broad spectral absorption
- High absorption efficiency over the whole absorption spectrum
- Large Stokes shift (no or low overlap in absorption and emission spectra)
- High luminescent efficiency (quantum yield)
- Matching the emitted photons to the spectral response of the PV-cell (≈1.14 eV for silicon)
- Solubility in the host matrix material

A wide variety of luminophores have been studied in an effort to meet these requirements. What follows is an overview of the major efforts in these areas, broken down into the following categories: 1) organic dyes, 2) quantum dots, and 3) rare earth ions.

2.3.1. Organic Dyes

Organic dyes are π–conjugated organic molecules, where the core of the molecule is planar with all atoms of the conjugated chain lying in a common plane and linked by σ-bonds. The π-electrons form a cloud above and below this plane along the conjugated chain. Absorption bands of these organic dyes are the promotion of these π-electrons from a ground energy state to an excited higher energy state.[111] The transition moment of these absorption events is mostly parallel to the conjugated plane, mostly the molecular axis of the dye, but some transitions are perpendicular to the molecular axis. These perpendicular transition moments are observed at short wavelength absorptions. The main absorption wavelength (λ_{abs}) can be calculated by

$$\lambda_{abs} = \frac{8mC_0}{h}\frac{L^2}{N+1} \tag{7}$$

where m is the electron mass, C_0 is the velocity of light in a vacuum, L is the chain length of the π–conjugated plane, h is Planck's constant, and N is the number of π electrons. The absorption band of organic dyes is mainly determined by the chain length and the number of π electrons in the conjugated plane of the molecule.[111]

Since the first papers on LSCs, organic dyes have been investigated as luminophores of choice due to their solubility, high fluorescence yields, and large absorption coefficiencts. The dyes investigated as possible components of LSCs belong to the following classes of molecule: bipyridines,[25] coumarins,[40,48,49,112–116] dicarbocyanine iodides,[48] dicyano methylenes,[20,48,49,113,117] lactones,[114] naphtalimides,[114,116,118] oxazines,[48] perylenes and perylenebisimides,[40,46,93,96,114–116,118–123] perylenebisimidazoles,[93] phtalocyanines,[124] phycobilisomes,[125] porphyrins,[20,124] pyrromethenes,[114]rhodamines,[16,18,48,49,111–114]sulforhodamines,[48,121] tertiary amine derivates of tetra-cyano-p-quinodimethane,[126] thioxanthenes,[114] (iso)violanthrones,[119] and some unspecified dyes including BASF K1,[111,121,127] BASF K27,[127] and BASF Lpero.[127] Characteristic examples of these dyes are depicted in **Figure 10**.

As can be seen, there have been a wide variety of dyes explored for use in LSCs over the past few decades, and we will go into more detail on many of these molecules in the following paragraphs.

The most commonly used dye types for LSCs have been the rhodamines, coumarins, and perylene(bisimides) derivatives. Rhodamines, like Rhodamine 6G, and coumarins, like Coumarin 6 (also known as Coumarin 540), belong to the group of dyes mentioned in the earliest stages of LSC research,[16,112] while the perylenes and perylenebisimides are mostly mentioned in the more recent papers on LSCs, and were first described for use in LSCs in the late eighties.[96,119] Rhodamines are known for their high quantum yields and high molar-extinction coefficients, but also for their small Stokes shifts. These small Stokes shifts lead to much re-absorption and consequently to increased LSC losses, both surface and quantum (nonradiative) losses. For Rhodamine 6G, the luminescence was reduced when incorporated in poly(methyl methacrylate) (PMMA) in comparison to the solution luminescence, but no suggestion has been made to explain this behavior as this is contrary to common performance of organic fluorophores in a polymer matrix.[113] In other types of dyes, like Coumarin 540 and dicyanomethylene (DCM), the luminescence increases when they are incorporated in a solid PMMA matrix, probably caused by an increased molecular rigidity, limiting nonradiative relaxation of the molecule in the excited state[113] or isolation from radicals or other impurities.[128] Rhodamines 590, 575, 6G and B show very limited photostability[111,114] in comparison to other types of dye molecules like perylenes and some coumarins.

 © 2012 WILEY-VCH Verlag GmbH & Co. KGaA, Weinheim

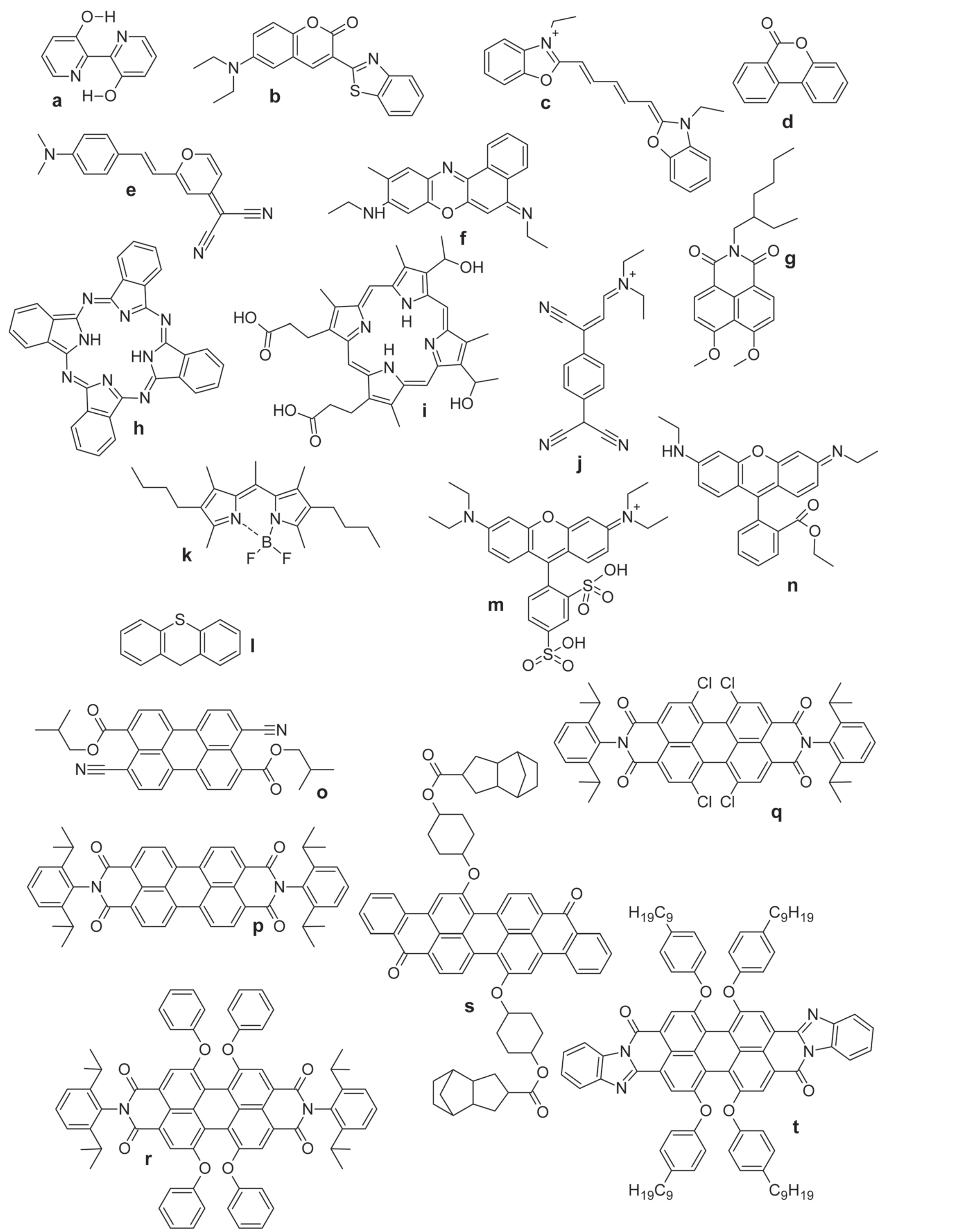

Figure 10. Examples of organic dyes used in LSCs: a) a bipyridine derivate, b) coumarin 6, c) a dicarbocyanine derivate (DODCI), d) a lactone, e) DCM (4-dicyanomethylene-2-methyl-6-p-dimethylaminostyryl-4H-pyran), f) oxazine 720, g) a naphtalimide derivate, h) phtalocyanine, i) hematoporphyrin, j) a tertiary amine derivate of tetra-cyano-p-quinodimethane (DEMI), k) pyrromethene 580, l) thioxanthene, m) sulfoRhodamine B, n) Rhodamine 6G, o) 3,9-diisopropionicacid-4,10-dicyanoperylene, p) perylene-3,4,9,10 tetracarboxylic acid-bis-(2'-6'diisopropylanilide) (Perylenebisimide), q) perylene-1,7,8,12-tetrachloro-3,4,9,10 tetracarboxylic acid-bis-(2'-6'diisopropylanilide) (Perylenebisimide), r) perylene-1,7,8,12-tetraphenoxy-3,4,9,10 tetracarboxylic acid-bis-(2'-6'diisopropylanilide) (Perylenebisimide), s) a derivate of an (iso)violanthrone, and t) a derivate of perylenebisimidazole.[119]

© 2012 WILEY-VCH Verlag GmbH & Co. KGaA, Weinheim

Coumarins can have a larger Stokes shift compared to Rhodamines.[48] The overlap factor, f_a, of the absorption and emission spectra of Coumarin 540A is 0.12, while for Rhodamine 6G this overlap factor of the absorption and emission spectra is 0.48.[113] There have been coumarins tested with moderate to very good quantum yields: Coumarin Red G has a quantum yield of over 80%[116] (87% was also reported[40]), while the quantum yield of coumarin 540A and CRS040 have been reported as nearly unity (98%).[40,114] Even though the photostability of coumarins have proven to be better than of rhodamines, they still have been reported to have reduced stability in comparison to perylene-based dyes.[40]

Perylene dyes and their derivates, like perylene bisimides, perylenebisimidazoles, and (iso)violanthrones, are known for their intense fluorescence and good photostability.[119] Perylene itself has low photostability, due to easy electrophylic substitution. To improve the properties of this dye, new side groups were added: 3,4,9,10-tetracyanoperylene has been synthesized, the cyano groups are electron acceptors and when added at these positions around the perylene core they improve the photostability. The solubility of this tetracyanoperylene is moderate, therefore 3,9-diispropionicacid-4,10-dicyanoperylene (Figure 10o) with a quantum efficiency of 91% and good photostability was synthesized.[119]

Perylene bisimides are known for their intense fluorescence and good photostability, but generally exhibit low solubility. To improve the solubility of these perylene bisimides, ortho-alkylated aromatic bulky groups are added to the bisimides (Figure 10p). These groups are twisted 90° with respect to the perylene core due to steric hindrance, which increases the solubility of the dyes. Addition of large side groups on the perylene core (1,7,8,12 positions; Figure 10q shows a chlorinated perylene core) cause the two sides of the perylene core to twist by 42° with respect to each other. This twist bathochromically shifts the absorption and emission bands of the perylene dye. Furthermore, the tertraphenoxy-substituted perylene bisimides (Figure 10r) have good solubility in organic matrices due to the phenoxy side groups, especially in comparison to the chlorinated perylene bisimides (Figure 10q). Component 10r has an absorption band peaking at 578 nm and an emission band peaking at 613 nm with a quantum yield of 96% with both good solubility[129] and photostability.

The violanthrones[119] and perylene-bisimidazoles (Figure 10t)[93] have more red-shifted absorption and emission bands compared to component 10r, with reasonable quantum yields of, respectively, 55% and 80%. If the conjugated core of the perylene is shortened to a naphthalene core, the absorption and emission bands are blue-shifted. An example of this type of dye is the BASF Lumogen F Violet 084, which is a naphtalimide.[118] This work demonstrates that by changing the side groups or changing the length of the core of an organic dye, a perylene in this case, one can alter all important properties of a dye, allowing for engineering of more suitable and robust molecules.

As described earlier, perylene based dyes are known for their high quantum efficiency[118] and for their large spread in spectral absorption and emission. Combining multiple perylene dyes in one single device could thus lead to both broad spectral absorption (the whole visible spectrum) and reasonably high quantum yields.[118]

Two different types of dicyano methylenes have been used in LSCs, DCM (4-(dicyanomethylene)-2-methyl-6-(p-dimethylaminostyryl)-4H-pyran)[48,49,113,130] and DCJTB (4-dicyanomethylene)2-t-butyl-6-(1,1,7,7-tetramethyljulolidyl-9-enyl)-4H-pyran).[20] DCM has a broad absorption spectrum and a large Stokes shift with a reasonable quantum yield (≈80% in PMMA),[48,113] but the photostability is limited. DCJTB has been used in combination with a platinum–porphyrin derivate, whereby DCJTB transfers its absorbed energy to the platinum complex. This combination is calculated to form an LSC with a 6.8% power conversion if paired with GaAs PV cells.[20]

Other dyes have also been investigated in LSCs, as mentioned before, but the published research on these dyes is more limited. Bipyridines[25] have a large Stokes shift, due to excited-state intramolecular proton transfer and twisted intramolecular charge transfer, but the quantum yield is low (up to 3.6% in butanol). Tertiary amine derivates of tetracyano-*p*-quinodimethane like DEMI (4-[1-cyano-3-(diethylamino)-2-propenylidene]-2,5-cyclohexadiene-1-ylidenepropane dinitril) are red-light absorbing dyes, but their photostability is low. Nature-based luminophores, like phtalocyanines,[124] hematoporphyrin,[124] and phycobilisomes[125] have also been proposed for LSCs. The phtalocyanines and hematoporphyrin have intense absorptions and good thermal stabilities, but the quantum yields are 20% and 8% respectively. The quantum yield of the phycobilisomes is below 50%.

Sulforhodamines, like sulforhodamine-B and sulforhodamine-101 have, similar to the rhodamines, small Stokes shifts,[48] resulting in many re-absorption events. Oxazine 720, oxazine 750, DODCI (a dicarbocyanine iodide: 3,3'-diethyloxadicarbocyanine iodide) and DOTCI (a dicarbocyanine iodide: 3,3′-diethyloxatricarbocyanine iodide) suffer from similar small Stokes shifts.[48] Thioxanthene dye D 315 orange, Lactone dye D 838 yellow, and Pyrromethene dye 580 have been shown to be relatively unstable towards illumination with visible light.[40,114] IR-144 (a dicarbocyanine) is only mentioned in the paper of Batchelder;[48] it appears to have a reasonable Stokes shift and absorption coefficient, but no further information on the performance of this dye in LSCs is available.

One of the disadvantages of organic dyes in general is the limited breadth of their spectral absorptions. To increase this breadth a combination of several dyes can be used. These dyes can be used in a stack of LSCs with one dye in or on each waveguide[20,49,93,113] or all dyes in one LSC.[40,48,49,103,112,118,120] In the first case each plate will act as an LSC itself. Light emitted in the escape cone by one LSC can penetrate another LSC where it can be re-absorbed (if the light is in the absorption band of the dye of the second LSC) and partly re-emitted in the waveguide mode. This is described in more detail in the part of this review focusing on the re-absorption problem of the LSC. In the case of all dyes in one LSC, all dyes will absorb energy and all this absorbed energy is transferred to the dye with the smallest bandgap, via nonradiative or radiative transfer. For FRET the dye molecules have to be very close to each other, typically less than 10 nm, so this only happens in LSCs with very high dye concentrations.

© 2012 WILEY-VCH Verlag GmbH & Co. KGaA, Weinheim

Numerous studies have been performed on the photostability of these organic dyes:[40,49,96,111,114,116,119–121,126–128,131] much depends on the processing conditions and polymeric environment of the fluorophore. Photodegradation of dyes in polymers can occur in two ways: 1) by direct interaction of the dye molecule with the sunlight, leading to decomposition, or 2) by the attack on a dye molecule by an active species formed due to photodecomposition of a residual molecule in the polymer matrix or by singlet oxygen.[114] These residual molecules are mostly materials used during polymerization or processing of the matrix material. Two examples of reduced luminescence in LSCs caused by other molecules present in the polymer matrix under solar illumination are photoreduction, where the dye in its excited state takes an electron from an electron donor, forming a nonradiative stable anion, and photo-oxidation where the dye in its excited state donates an electron to an acceptor, forming a nonradiative cation.[119] Direct interaction of the dye molecule with the solar light can lead to molecular changes, caused by high-energy photons. Photochemical decomposition of the dye molecule can lead to a reduction in absorption and thus also a reduction in fluorescence or to a blue-shift in absorption and emission.[111] An extensive comparison of the photostability between several types of dyes, including rhodamines, coumarins, perylenes, a naphtalimide, a thioxanthene, a lactone, and a pyrromethene, showed that the perylenes from the Lumogen Series of BASF had the best photostability, both under illumination of UV light as well as visible light.[114]

There are several options beyond the engineering of more robust dyes towards extending the photostability of the LSC device. Introducing UV absorbers and hindered amine light stabilizer (HALS) molecules could reduce the photodegradation of the dyes under UV illumination. Using copolymers of PMMA has also been shown to increase the photostability of the dyes.[131] Decomposition of the dye molecules is much faster in a singlet oxygen environment than in a nitrogen environment, with a small recovery of luminescence occurs after some time in the dark.[119,43] Thus, it may be that during dark periods during the night could recover some of the luminescence lost due to photodegradation in the daytime.

2.3.2. Quantum Dots

Quantum dots (QDs) are nanostructures from semiconducting materials with dimensions in the order of 10–100 nanometers. The size of the dots is in the order of the de Broglie wavelength of the electron. As a consequence of their restrictive size, excited electrons are confined in the semiconductor, which exhibits optical and electrical properties similar to those of atoms.

QDs promise several advantages over organic dyes if used in luminescent solar concentrators. The absorption threshold of the QDs may be tuned by judicious choice of the particle diameter (see **Figure 11**). Colloidal InP QDs, for example, have been shown to be capable of absorbing the whole visible spectrum.[133] QDs may also have large Stokes shifts, which are determined by the spread in dot size.[134,135] Their crystalline semiconductor composition should make them more stable than organic-based dyes.[88] Quantum dots as luminophores in luminescent solar concentrators were first suggested and modeled by the group of Barnham,[35,87,88] which predicted a concentrator efficiency up to 20% when type III–V solar cells were used in combination to high quantum efficiency (QE = 1) QDs.

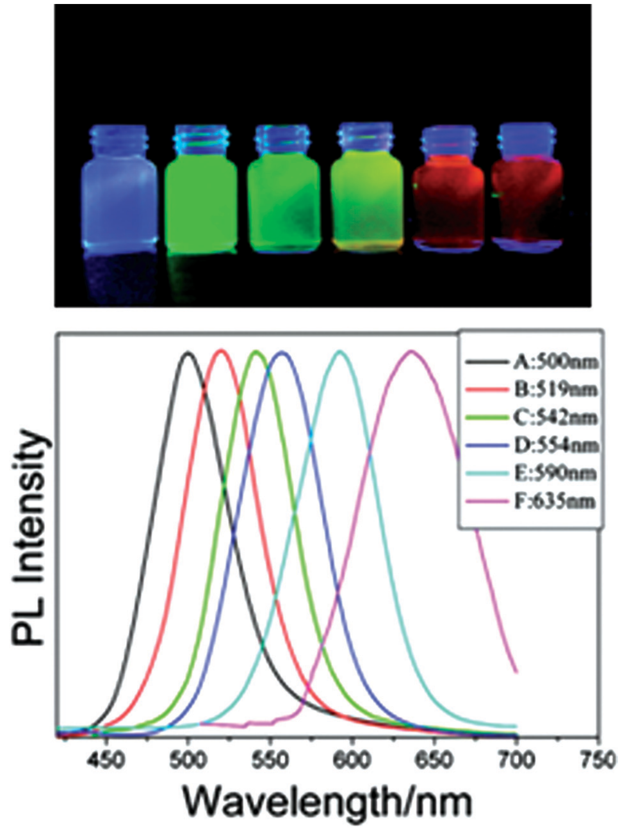

Figure 11. Top: Fluorescence of CdSe with different emissions (λ_{ex} = 365 nm). Bottom: Emission spectra of the CdSe quantum dots at different growth temperatures and at the same reaction time of 10 h (A, 45 °C; B, 60 °C; C, 80 °C; D, 100 °C; E, 120 °C; F, 150 °C). Reproduced with permission.[132] Copyright 2008, American Chemical Society.

As mentioned above, QDs promise good photostability, but outside a solid matrix they are quite sensitive to oxygen and light,[38,136–138] which becomes a challenge for module engineering. Several research groups claimed that the incorporation of QDs into a solid matrix can lead to a blue-shift in both their absorption and emission, caused by surface oxidation of the QD during the manufacturing process.[89,136,139] In addition to a blue-shift in emission wavelength, the emission intensity in QD solar concentrators also is dependent on the nature of the host matrix. For example, CdSe/ZnS QDs in a solid matrix lose 22.5–96% of the emission intensity they exhibited in solution.[89] Several factors can cause this loss, including increased scattering due to particle clustering owing to decreased solubility, and matrix absorption of the emission light.

The spectral emission of QDs may be tuned by adjusting the QD size. With increasing QD size, the bandgap will decrease. The cluster size distribution of the dots can be tuned in situ during the production of the QD concentrator. By increasing the annealing temperature of CdS quantum dots in silicon dioxide films, the photoluminescence spectra red shifted, attributed to an increasing CdS dot cluster size.[140] These results were supported by calculations and direct X-ray diffraction

© 2012 WILEY-VCH Verlag GmbH & Co. KGaA, Weinheim

(XRD) measurements of the QDs, determining that the cluster size grew at increased annealing temperature.[139] Schuler also showed that CdS-rich siliconoxide layers show an absorption band close to the bandgap of bulk CdS, while low concentration layers had bandgaps of higher energy, corresponding to finite-well and tight-bonding calculations.[141,142] Naturally, by altering QD materials one may also define alternative bandgaps. PdS used as a QD material, for example, has an absorption band up to wavelengths in the IR part of the spectrum.[91] In addition to a larger spectral absorption, these QDs also have a larger Stokes shift, leading to less overlap between the absorption and emission band. Where the CdSe/Zns ($\lambda_{abs} \approx 600$ nm) QDs used by Shcherbatyuk[91] have a Stokes shift of 23 nm and an absorption of 0.68 (normalized to the peak absorption) at the peak emission, the PbS ($\lambda_{abs} \approx 750$ nm) QDs had a Stokes shift of 122 nm and a normalized absorption of 0.24 at the emission peak. A disadvantage of these PbS QDs is that the molar extinction coefficient is one order of magnitude lower than that of the used CdSe/ZnS QD (2 × 104 L mol^{-1} cm^{-1} for PbS and 3×10^5 L mol^{-1} cm^{-1} for CdSe/ZnS). Red-absorbing cadmium-based QDs also show a decrease in absorption coefficient,[143] but because the spectral absorption is higher for these materials the total absorption efficiency is still higher than of cadmium-based QDs absorbing only up to the blue or yellow part of the spectrum. Kennedy et al. showed that the optical absorption efficiency for NIR, orange, and green-emitting Cd-based QDs was, respectively, 23.1%, 21.7%, and 11.6%.

The increase in the Stokes shift for the lower bandgap QDs leads to less re-absorption of waveguiding photons, which in turn may also reduce the surface losses of the QD solar concentrator (QDSC). NIR-emitting QD solar concentrators were modeled to have 43 ± 1% surface loss in comparison to the green-emitting QD solar concentrators, which had 58 ± 5% surface loss. For 60 mm × 60 mm × 3 mm QD concentrators, the reduced re-absorption translated into a modeled optical efficiency of 13.2% for the NIR-emitting device, in comparison to 5.0% for one that was green-emitting, assuming similar quantum yields of unity in each device.[143] PbS QDs can also show similar results. The absorption efficiency of the PbS QDs is 40% in comparison to 22% for the CdSe/ZnS QD used, but the luminescent quantum yield for the PbS QDs is lower (30%) than that of the CdSe/ZnS QDs (50%). As a result, the resultant optical efficiency for the PbS QD solar concentrator was set at 12.6%.[91] The emission spectra of the QDs used in these experiments demonstrated that increased pathlength of the emitted light through the waveguide resulted in a shape change, with the short wavelength peak decreasing with respect to the longer wavelength peak, demonstrating that re-absorption still plays an important role in these QD concentrators.[91,140]

QD-based concentrators have been compared directly with organic dye containing LSC via both modeling and experimental studies. One such work compared CdSe/ZnS QD and Lumogen Red F 300 containing concentrators.[89] The best QD concentrator described in this paper reached to 58% of the performance of the device containing Red 300 containing, mainly a result of the lower quantum yields (QY 0.1–0.6) of the dots compared to that of the organic dye (fluorescence quantum yield FQY ≈ 1). Another work has compared the optical efficiency of CdSe/ZnS QD containing concentrators with several other organic dye containing concentrators, including Rhodamine B, a red fluorine (Red F), and several other laser dyes (LDS698 and LDS821).[90] Simulations and experiments described in this work showed the optical efficiency of the QD concentrator was within 10% of the optical efficiency of the Red F- and Rhodamine B-containing LSCs. The optical efficiencies of the LSCs containing the LDS series dyes were comparable to that of the QD concentrator. These relatively low optical device efficiencies reported were again attributed to the relatively low quantum yield of the QDs. Increasing the quantum yield of the dots closer to that of Rhodamine B (stated to be ≈95%) would lead to increased optical efficiencies of the device: as it was, the QD-based LSC performed at 60% of the optical efficiency of the Rhodamine B-containing LSC. Calculations done on PbS containing QD concentrators predict such devices could achieve power-conversion efficiencies (PCEs) more than double the PCE of a Rhodamine-containing LSC, at 3.2% and 1.3%, respectively.[91]

It should be noted there is some concern about the use of the QD LSC due to the potentially toxic nature of many of the materials used in the QD.[144–146] There appear to remain considerable uncertainties as to the true toxicity of these materials, and the level of toxicity often is related to not only chemical composition, but also processing conditions, environmental factors, and a number of other details. Nevertheless, there is continued research aimed at reduction of the potential harmful effects of QDs, such as the use of 'jelly dots'[147] or on QDs based on more benign materials, like silicon.[148,149]

2.3.3. Rare Earth Ions

Rare earth ions (sometimes complexed with ligands) are investigated as luminophores for usage in LSCs primarily because of their promise of high photostability and their large Stokes shift, although the presence of organic ligands may compromise the effective lifetime of the molecules. Levitt and Weber[18] already described in 1977 the use of neodymium (Nd^{3+})-doped glasses as materials for LSCs. Neodymium mainly absorbs around 580 nm, but also absorbs photons at longer wavelengths. Emitted photons have wavelengths around 880 nm ($^4F_{3/2} \rightarrow {}^4I_{9/2}$) and 1060 nm ($^4F_{3/2} \rightarrow {}^4I_{11/2}$). The photons emitted at 880 nm can be reabsorbed ($^4I_{9/2} \rightarrow {}^4F_{3/2}$) and the energy of the photons emitted at 1060 nm is slightly lower than the bandgap of silicon. These characteristics of neodymium lead to low efficiencies of Nd^{3+}-doped glasses as LSCs.[150]

To increase the efficiency of neodymium-doped LSCs, codoping with ytterbium (Yb^{3+}) has been discussed.[150] Energy absorbed by the neodymium ions is transferred to the ytterbium ions, this accomplishes two goals: it will circumvent the limitations due to self-absorption, and the neodymium emission at 1060 nm will decrease due to depopulation of the $^4F_{3/2}$ state of neodymium by the $^2F5_{/2}$ state of ytterbium. Emission from ytterbium ions is around 970 nm ($^2F_{5/2} \rightarrow {}^2F_{7/2}$), which is slightly higher in energy than the bandgap of silicon, but the response of silicon to photons with this wavelength is still high. The energy transfer from neodymium to ytterbium depends on the type of glass: in borate tellurite and germanite glasses the energy transfer efficiency can reach up to 90%.[150,151] Ytterbium itself can also be used as a luminophore in LSCs, but the solar

© 2012 WILEY-VCH Verlag GmbH & Co. KGaA, Weinheim

absorption of these ions only occurs in the NIR ($^2F_{7/2} \rightarrow {}^2F_{5/2}$ in $4f^{13}$ configuration) or in the UV ($4f^{12}$ configuration), so large sunlight absorption is only possible if ytterbium-doped glasses are codoped.[152] In the case of neodymium, the neodymium ions are codopants for the ytterbium. A disadvantage of neodymium and ytterbium is their low absorption efficiency.[153]

Uranyl ions (UO_2^{2+}) have been reported[153] in LSCs because of their high absorption efficiency. Uranyl ions have five orders of magnitude higher absorption efficiency than neodymium ions, but they absorb only in the blue part of the spectrum (maximum absorption around 430 nm). The fluorescence of uranyl ions is maximum at 500–530 nm,[153] and the quantum yield is 0.67.[154] Uranyl ions have also been used as codopants in combination with neodymium,[155] and ytterbium and neodymium.[156]

Chromium(III) ions have a large spectral absorption with peaks at 450 nm ($^4A_2 \rightarrow {}^4T_1$) and 650 nm ($^4A_2 \rightarrow {}^4T_2$) and so could be useful for LSCs, but a major drawback is the limited quantum efficiency (up to 25%).[156] Higher quantum yields have been found in quartz-like (FQY = 50%), pentalite-like (FQY = 75%), and gahnite (FQY = 100%) glass ceramics.[157,158] Chromium(III) ions can be used for codoping neodymium- and ytterbium-doped glasses, as they increase the absorption range of the glass and then transfer their energy to the high-quantum-yield neodymium and/or ytterbium ions. The energy-transfer efficiencies from chromium(III) to neodymium and ytterbium have been determined at 92% and 88% respectively in lithium lanthanum phosphate (LPP) glasses.[156] Other rare earth ions can also be used in LSCs.[154,159]

To increase the absorption of rare earth ions, organic ligands coordinating to the ions have been proposed.[85,156,160] In such a complex, the ligands absorb energy and the electrons go from their S_0 to their S_1 state. From the single S_1 state, energy is transferred to the triplet state of the ligand (T_1) via intersystem crossing (ISC). From this triplet state, energy is transferred to the rare earth ion. Due to all the energy transfers, these complexes have a very high Stokes shift (>200 nm). Moudam et al.[160] synthesized a Eu^{3+} complex (Eu(hexafluoroacetylacetonate)$_3$(*bis*(2-(diphenylphosphino)phenyl)ether oxide). This complex absorbs light in the UV region (<350 nm) and emits it at 613 nm with an FQY of 86% in PMMA. In 2011, Katsagounos[161] showed four europium-based complexes with increased luminescence compared to the single europium ion and using these dyes in LSC-type downconverters increased the efficiency of multicrystalline-silicon solar cells up by 17%, for the complex with a pyridine derivate as a ligand.

The spectral absorption of Eu-complexes is not very broad because low-energy photons create a triplet state lacking sufficient energy for transfer to the europium ion, but may have a large Stokes shift (see **Figure 12**).[85] Other rare earth ions like neodymium and ytterbium can be excited by lower-energy triplet states, creating NIR-emitting complexes with broad absorption in the visible region of the solar spectrum. A problem occurring with these ions is that they can be deactivated by surrounding vibrational states of O–H, N–H, and C–H bonds. Therefore the ligand has to been fluorinated or deuterated, because C–F and C–D absorptions occur at lower energies. The emission of this fluorinated ligand and ytterbium complex peaks at 970 nm when excited at 320 nm. In perfluoromethylcyclohexane, the luminescent QY of the ytterbium complex with the fluorinated ligand was determined to be 2.6%; not high enough for LSC applications.

2.3.4. Enhanced Absorption by Scattering Back Layers

To aid in luminophore absorption, it is standard practice to apply a rear layer to an LSC to act as a reflector. The reflecting back layer effectively doubles the path length of incident light through the dye layer for enhanced absorption. Some of the initial experiments used a silver mirror,[17] but such a mirror absorbs in the visible range and can result in significant absorptive losses for longer transmission distances. To avoid absorptive losses, most recent work has employed a white scatterer.[44,45,103,130,162–165] The effectiveness of the scatterer depends on both the size of the waveguide (the scatterers have been shown to be beneficial for waveguides tens of centimeters long) and the concentration of dye within the waveguide.[165] The scatterer also may direct that fraction of incident light that cannot be absorbed by the dye directly at the PV cell, allowing it to generate electricity. The separation of the scatterer from the waveguide by a low refractive index layer is quite important, as one desire to maintain waveguided light in the trapping modes of the waveguide: every encounter with the attached scattering layer redistributes the light, and a significant fraction of this redirected light will be outside the waveguide modes of the system.

It was demonstrated that the effectiveness of the scatterer is greatest near the edges of the device, where direct scattering of nonabsorbed light into the solar cell directly is prevalent. However, over long distances the direct scattering component is less important, and the primary advantage given by the scattering layer is the return of unabsorbed light back into the waveguide for possible absorption on the second pass, but even this effect can be minimized in highly dye-doped waveguides.[165] This suggests a possible transparent LSC design with only the close edges using a scattering layer.

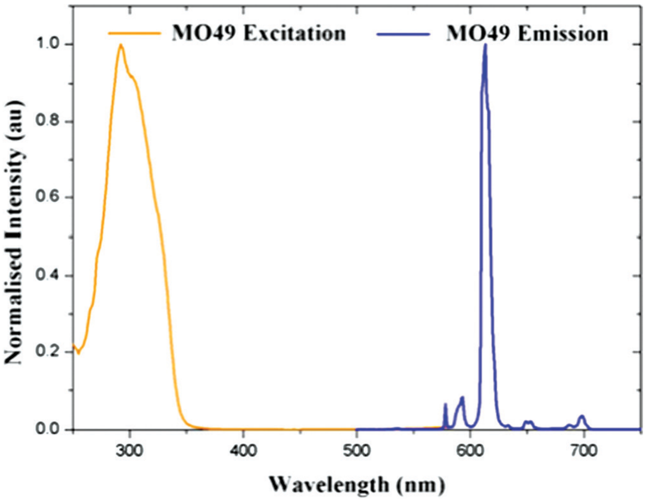

Figure 12. Absorption and emission spectra of Eu^{3+} complex (EuhD) with the structure Eu(hfac)$_3$(DPEPO) where hfac = hexafluoroacetylacetonate and DPEPO = *bis* (2-(diphenylphosphino)phenyl)ether oxide.[85]

© 2012 WILEY-VCH Verlag GmbH & Co. KGaA, Weinheim

The functionality of the rear layer may be enhanced by adding a luminophore to the scattering layer. This can potentially improve performance by converting light otherwise lost due to lack of absorption into light of longer wavelengths that can be absorbed by the luminophores within the waveguide[163,166] while still maintaining the benefits of scattering described earlier.

The use of a scatterer rather than a reflector becomes particularly important in the case one uses a top wavelength-selective mirror, as described in Section 2.1.2 above. As light that is reflected by the selective reflector is not in the waveguiding mode, this means it will encounter the back layer of the waveguide every bounce. If this rear reflector is a silver mirror, the absorption losses from the silver would dramatically reduce the amount of energy that can be emitted from the waveguide edge, and so use of a metallic back mirror should be avoided in this case. Because of the relative contribution of the scattered light is high when compared to dye-emitted light for smaller samples,[40,165] the relative contribution of scattered light somewhat obscures the contribution from the selective reflector. Thus, the true enhancement of performance expected from the selective reflectors will become more obvious for larger (that is, greater than 25 cm^2) waveguides.[40]

2.3.5. Enhanced Fluorescence by Metal Nanoparticles (Plasmonics)

A newer research area for LSCs is in the field of using plasmonics to enhance the system response.[98] It has been shown in many research publications that when a fluorescent molecule is brought within close proximity of a small metallic nanoparticle, such as silver, there may be a considerable enhancement of the fluorescence.[167–171] There has been previous application of surface plasmonics in PVs to enhance device performance.[172–178]

Very recently, experiments have coupled such plasmonic photovoltaic cells to LSCs, with predicted large enhancements of the efficiency.[179] The introduction to plasmonic structures within the LSC waveguide could open up the use of a considerable range of dyes that had heretofore been rejected on the basis of low quantum efficiencies or photostability, as both of these important dye characteristics could be improved by the application of some type of plasmonic-based system.

2.3.6. Enhanced Absorption and Emission by Confinement Effects

Other designs may be contemplated that will reduce the limitations of the dye materials through confinement effects. Recently, a slot waveguide using a nanometer-sized low-refractive-index slot sandwiched by two high-refractive-index regions was calculated to enhance emission by the luminophore due to the Purcell effect, to increase the effective absorption length of luminescent centers and improve their fluorescence quantum yield.[180] The physical requirements of this device, however, are quite extreme, with a 10 nm slot demanding use of materials with refractive indices >2. While immediate use of such an architecture is not likely, the design does provide an interesting framework to build upon.

2.3.7. So, Which Luminophore to Choose?

At this point in our examination of the history of luminophore research in LSCs, we have emphasized mainly the performance characteristics of the dyes in terms of their ability to generate the maximum light output of an LSC. However, the flexibility of the device allows for a broader outlook than simply maximum performance. When one considers the importance of aesthetics of the LSC, one realizes there should not be a search for the single, all-purpose luminophore, but there should be a refinement of an array of high-performance molecules displaying a broad range of characteristics. For instance, for artistic reasons it is of interest to have materials of good performance in a variety of colors/shades. As shown in **Figure 13**, being able to work with a variety of colors adds exponentially to the applicability of the LSC.

To this point, there is no clear 'winner' among the various luminophores, nor even has a class of luminophores been identified as the obvious choice. Research continues apace on organics, quantum dots and phosphors. There may be instances as well where choices will be made based on the specific criteria of the application: does one prefer high fluorescence yield, pleasant aesthetics, or enhanced photostability? Obviously, there is still plenty of room for innovation in this area.

2.4. Photovoltaic Losses

The standard silicon-based PV has a bandgap corresponding to a photon of around 1100 nm (≈1.1 eV). Photons with energies above this threshold may still be processed by the solar cell, of course, but the excess energy of the photon is wasted, and converted most often into heat, and there is a reduction in the response of the cell for these shorter wavelengths. Energies lower than this bandgap are not sufficient to generate any current.

However, an LSC does not emit a spectrum remotely similar to the solar spectrum. Rather, it emits a narrow range of

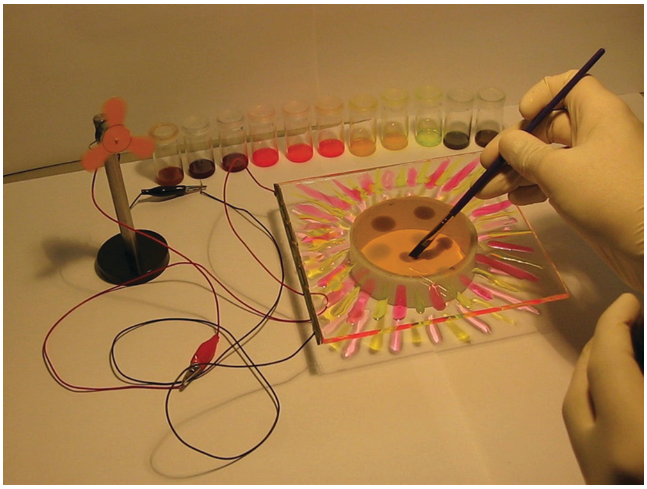

Figure 13. Video still depicting the 'painting' of fluorescent dyes on a clear PMMA plate forming an LSC. The full video may be found as Supporting Information (courtesy Eindhoven University of Technology and Peer+).

© 2012 WILEY-VCH Verlag GmbH & Co. KGaA, Weinheim

wavelengths, most often centered at red and NIR wavelengths (630–720 nm at the maximum). This gives the advantage that the LSC should remain relatively cool under standard operating conditions, as they do not absorb much of the infrared light. In consequence, the light incident on the edge mounted photovoltaic will contain minimal infrared components, preserving the high performance[22] as generally photovoltaics perform less well when heated.[181]

In addition to reduced heat loads on the cell, by matching the spectral response of the solar cell to the output spectra of the waveguides it may be possible to obtain enhanced performance, although the overall effect may be limited due to the generally broad response of many cells to light over the common wavelengths emitted by luminophores.[130] However, this tuning could find application in amorphous silicon[182,183] and various type III–V cells. Using larger-bandgap solar cells would deliver similar currents, but at larger V_{oc}.[40]

Downconversion, which is the process of converting a high energy photon into a lower energy photon with a wavelength more readily utilized by the photovoltaic, has been studied using luminophores attached directly to the surface of the PV cells[184–187] or within the encapsulation layers of the PVs.[4] The goal of these layers is generally to shift the incoming 'blue' portion of the spectrum to longer wavelengths better suited to the PV. In other cases, colors are applied to the photovoltaic surface for more aesthetic reasons, to add alternative colors to the PV panels.[188] A similar application of this type, but this time using small, sliver silicon-based photovoltaics located within the waveguide itself, promise improved performance.[117]

To better exploit the emission spectrum in the LSC, researchers have used type III–V PV cells based on GaAs and InGaP[44] and obtained record-setting efficiencies. If these cells could be produced economically,[189] it could hold promise for widespread adoption of the LSC in future. Another option could be the use of organic-based PV cells, which often have a 'sweet spot' in the spectral range where the LSC emits.[190]

Another option to enhance the response of photovoltaic cells is to use individual dye-filled LSC waveguides, where each waveguide is separately attached to a solar cell specific for that emission wavelength. In the so-called luminescent spectral splitter (LSS) concept[191] illustrated in **Figure 14**, the incoming light is collected over a large area and funneled into a smaller region where it is absorbed by a stack of what are essentially LSCs, each attached to a separate photovoltaic cell tuned to the specific wavelengths emitted by the LSC. In this concept, the LSCs themselves do not need to transport the light over extended distances. Yet another option is to employ an LSC with edge-mounted photovoltaic directly on top of a second photovoltaic: in this configuration, the light that passes through the LSC due to its limited absorption range will be collected by the underlying cell.[192]

2.5. Waveguide Losses

Around 4% of incoming sunlight is reflected from each of the waveguide surface (the refractive indices of poly(methyl methacrylate) (PMMA) and poly(carbonate) (PC) being between about 1.49 and 1.58) and never enter the waveguide, and could thus be considered a loss (Figure 4, loss 5a). While antireflection coatings are very common in PV cells, they have not yet been applied to LSCs. As the LSC relies on total internal reflection from two smooth surfaces, textured systems as used in many antireflective coatings may not be a viable option.[193] Rather, coatings utilizing different refractive indices can reduce these reflective losses and can be applied to polymeric materials.[194] It remains to be seen how these coatings affect LSC performance.

Naturally, it would be a great advantage to be able to produce the LSC panels on the size of square meters in many applications, as this could allow for a more 'seamless' look, and reduces the amount of wiring that needs to be done thus

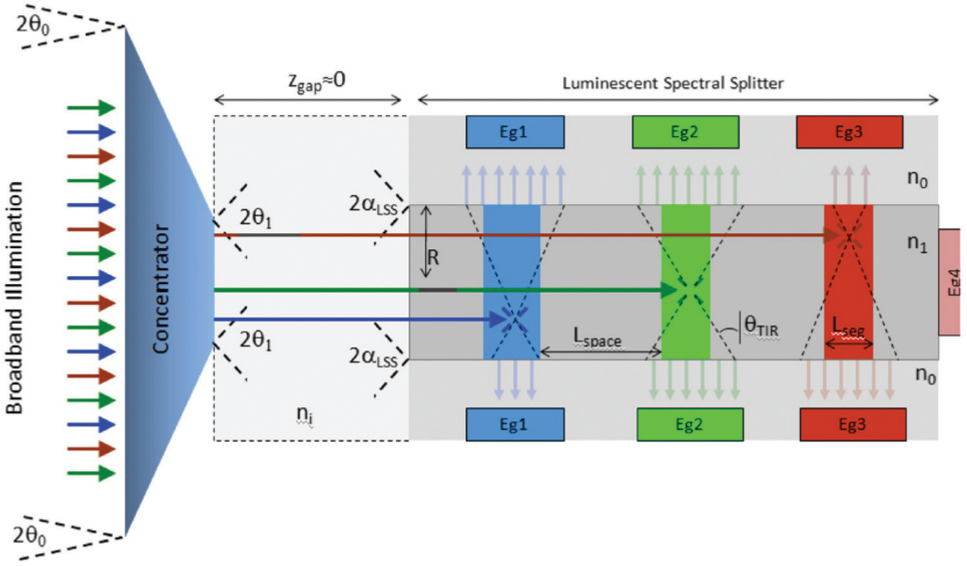

Figure 14. Luminescent spectral splitter. Reproduced with permission.[191] Copyright 2011, Elsevier.

lowering installation costs, among other things. However, there are all the losses described in this section that must be accounted for which places severe limitations on the maximal size for the device. The drop-off of edge output with distance has been reported.[35,164,165] These transport losses are generally the result of two main loss mechanisms: re-absorption of emission light by subsequent dye molecules (described earlier, Figure 4 loss 2) and parasitic absorption of the emission light by the host waveguide material (Figure 4, loss 5b). Other features are certainly at play as well. For example, imperfections in the surface of the LSC waveguide will also lead to losses (Figure 4, loss 5d), and the presence of dust or minor imperfections can result in the scattering of light outside of the waveguiding mode and lost to the environment.[195] Generally, the devices should be kept as scratch free as possible, suggesting the use of hardcoats or the like for particularly soft waveguide materials. There is also potential for a significant degree of scatter from unwanted scattering centers located within the waveguide (Figure 4, loss 5c).[195] These scattering and absorption events dominate for large waveguides: re-absorption by the dyes is primarily a short-range effect,[196] although the absorption 'tail' of the luminophore may have quite a large impact over long distances.[83,164]

One desires to extend the dye absorption as far to the infrared as possible. However, this is complicated in that the polymeric waveguides, which are predominantly made of PMMA or polycarbonate, become parasitic, and absorb strongly at wavelengths beyond 880 nm.[164,197–199] Often the additives designed to improve various characteristics of the host matrix in the solid state (such as altering coloration, stability, or hardness) or the presence of unreacted monomers in the host material can have a large impact on the device's performance.[43,102,116] For example, an additive that shows only a small absorption when measured through the width of the waveguide can have a severe impact on the edge output of the same object, for the pathlength is magnified many tenfold.[102,130] In addition, as the waveguides age due to exposure to the elements and in particular ultraviolet light, generates brittleness, opacity, and a host of reaction species that generate a yellow tint to the plate and act as absorptive light 'traps'.[200] To combat these loss mechanisms, research into copolymer systems has demonstrated enhanced photostability over single component systems.[131] During the transition from the excited state to the ground state of the dye molecule, electron transfer takes place from the dye to the main copolymer (here polystyrene-*co*-methylmethacrylate) chain, which may increase the photostability of the dye.

In general, the waveguide materials generally used for LSC work tend to be chosen with economy, rather than absolute best performance, in mind. However, much optically clearer materials are available in the marketplace, and could be considered for use in the LSC. For example, PMMA and perfluorinated-polymer-based optical fibers may demonstrate considerably enhanced transmission characteristics.[201]

An obvious way of decreasing the waveguide losses is application of waveguides using higher refractive index materials. The most prevalent materials used for the LSC have been PMMA and glass with refractive indexes on the order of 1.49. Higher refractive index materials used or considered have included polycarbonate ($n \approx 1.59$),[102] and special glasses (n = 1.5–1.8).[20,38,102] Glass samples allow for potentially less absorption in the emission regions of the dye than polymeric waveguides,[38] but many of the professed advantages of the LSC (for example, reduced weight and ease of handling) would be nullified.

Another waveguide material that has been considered for its flexibility has been polysiloxane.[94,202] While the durability of such materials might be a question, a range of novel applications could be considered with the addition of flexibility to the LSC range of characteristics. The emission of such polysiloxane-based systems has been comparable to that of filled polymer waveguides of a certain dye concentration (see **Figure 15**). This suggests that if the dyes were more soluble, such rubbery waveguides could be a viable option.

An additional aspect of waveguide design that needs to be considered is that edge emission from rectangular waveguide edges is nonuniform over the exit surface. Intensity of the emission can vary 20% between the center and corner of the waveguide edge.[73,203] This variation may also cause additional losses, as this means illumination of the attached photovoltaic is not uniform, and nonuniform illumination of a photovoltaic cell will result in decreased performance of the cell.[204–206] In order to improve the light concentration at the edges and reduce the size of the attached solar cells, tapering of the waveguide edges has been suggested.[207]

As one of the main goals of the LSC is to reduce the surface area of photovoltaic cells, it would be preferable to reduce the number of edges with solar cells attached from four to fewer. Configurations using two-edge coverage (both opposite and orthogonal cell placement) and single-edge arrangements are common. When reducing the edge coverage of solar cells, reflective mirrors are often added to the PV-less edges, and a fraction of this reflected light reaches the photovoltaic cell.[38,40]

For both performance and aesthetic reasons, alternative shapes of the waveguide have been studied. The shapes of these waveguides also influence the edge-emitted-light distribution.[22,40,81,203,208] While the circular LSC could provide the

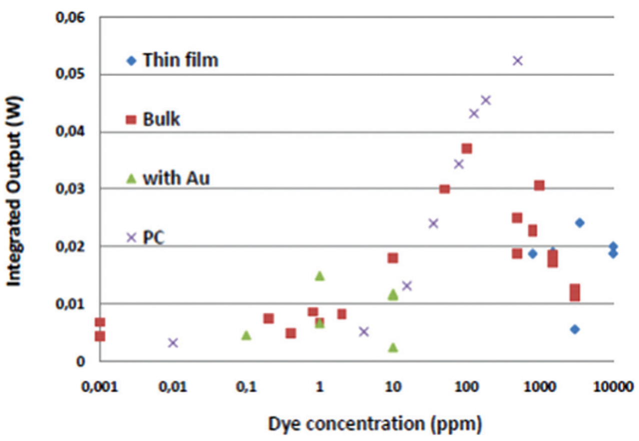

Figure 15. Edge outputs of LSCs made from flexible polysiloxane waveguides compared to filled polycarbonate LSCs (purple crosses) with the dye distributed throughout the bulk of the polysiloxane waveguide with (green triangles) and without (red squares) added gold atoms, and in with the dye cast as thin layers atop a clear polysiloxane waveguide (blue diamonds).[202]

 © 2012 WILEY-VCH Verlag GmbH & Co. KGaA, Weinheim

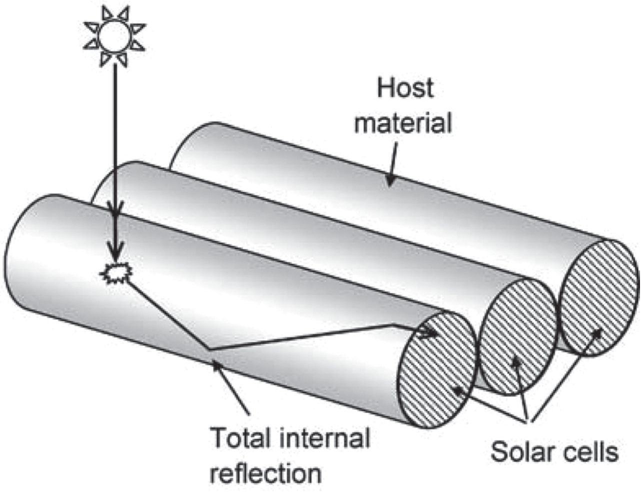

Figure 16. Array of cylindrical LSCs. Reproduced with permission.[210] Copyright 2007, Springer.

greatest edge output,[203] the packing limitations of such an array would be inappropriate. Rather, the hexagonal device was calculated to be a superior design to the common rectangular device as far as light emission,[40,203] but altering the geometry necessitates a different area of solar-cell attachment and material usage for the waveguides, which have an effect on the price of the module. Calculations concluded that varying the geometry type did not reduce costs significantly enough to be an economically viable plan: rather, it was the overall size of the object was a critical feature.[40]

By adding a third dimension to the standard device one can also influence emission efficiencies. Including curvature to the surface of a hexagonal waveguide was claimed to be effective in reducing losses through the escape cone of the waveguide.[209] Expanding upon this, a multicylindrical array of LSC tubes was shown to both increase the degree of light concentration and reduce the surface reflections (see **Figure 16**).[210] An LSC fiber has also been suggested which could be bundled with other fibers and the emission light made incident on a single photovoltaic,[211] and recently fibers with built-in focusing elements have been made.[212]

3. Alternative Applications

The standard application foreseen for the LSC has been as an electricity generating, static element in some larger object. However, this is not the only possible application of the device. In this section we present work on a couple other potential uses of the LSC.

3.1. Switchable 'Smart' Windows

Another option for making use of dye alignment in an LSC is to not add a rear layer at all, instead allowing the LSCs to remain transparent. In this form, the device could be used as a window while simultaneously generating electrical current. While a static LSC has been introduced that could be used as a window,[213] it has the disadvantage common to thin-film transparent photovoltaics, in that they may generate electrical current but not alter their transparency.[214–216] By the same token, responsive systems that can change their transparency, such as photo- and thermochromic windows[217–220] or standard blinds[221] generate no electricity.

An alternative LSC design uses, rather than a waveguide with embedded, inflexible dyes, two glass plates coated with a conductor, and the space between the plates is filled by a liquid-crystal (LC) host containing a fluorescent dye species.[64] The liquid-crystal host can be continually switched between two states, planar and homeotropic, and all angular configurations in between (see **Figure 17** for a graphic depicting the performance of the windows).

The dichroic dye molecules mirror the alignment of the LC host. In the planar alignment, which is the position of maximal absorption for the dye, the dye may emit light which partially may be trapped in the glass panes making up the 'window' and generate electrical current, with preferential emission from the edges parallel to the LC alignment. This constitutes the 'dark' state of the device, with maximum absorption (and consequently minimum transmission through the 'window') and maximum emission, and thus maximum electrical generation with minimal power input.

By applying of a voltage across the plates, the LCs may be made to attain an angle with respect to the glass plate, reducing the absorption by the dye, and allowing more light into the space beyond. In this 'light' state, while the output of the window necessarily drops due to reduced light absorption, it still produces current, and due to the dye alignment, the optical efficiency is actually increased. This design still needs work to achieve the desired transmissive properties and coloration, but could potentially provide considerable advantages over alternative 'smart' windows. It is also possible to create a third state, a scattering 'privacy glass' condition: this work is still in progress.

3.2. Daylighting

Not all applications convert the emitted light into another state. The LSC has also found potential use as a daylighting device, rather than as an electricity generator. In this configuration, an LSC consisting of a stack of dye-doped waveguides of different colors is located on a rooftop, and the emission light from the waveguide edges is collected in a clear polymer or glass cable and transported a distance into the interior of a building, for example. At the end of the light pipe, the emission light is mixed, and passes through a diffuser into the room beyond. See **Figure 18** for a graphical depiction of the device. The LSC daylighting element displays advantages over alternative daylighting systems in that they require no tracking or astigmatic correction.[164,222–224] A key element in this manifestation of the device is the necessity of long transport distances of luminophore-emitted light, and the color mixing of the light at the emission site.[164] In particular, the blue portion of the spectrum is lacking due to the unavailability of a blue emitter that is sufficiently photostable and, to compensate, current designs have used blue light emitting diodes to provide the short wavelengths and obtain the desired white-light emission.

© 2012 WILEY-VCH Verlag GmbH & Co. KGaA, Weinheim

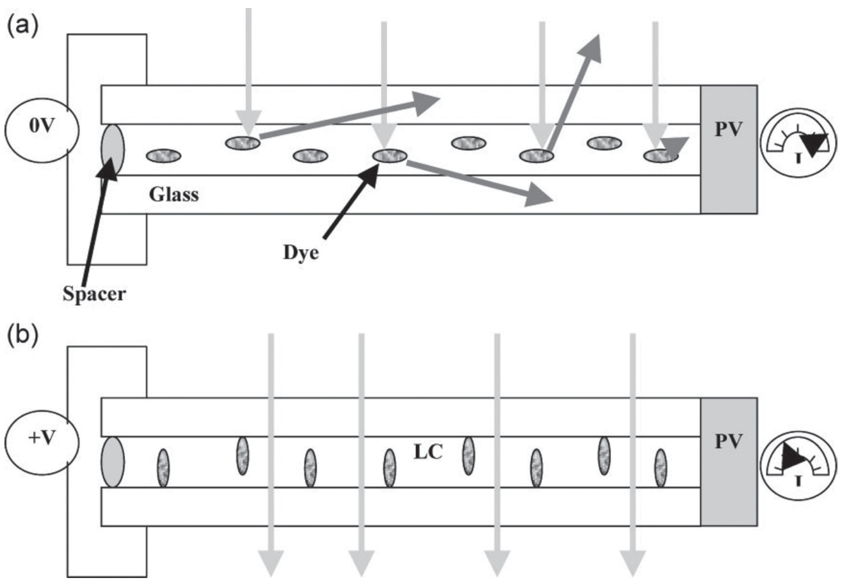

Figure 17. Two states of the LSC 'smart' window. a) At rest, the host liquid-crystal material lies in a planar state, and the associated dyes absorb at maximum, producing a 'dark' window. Emission light is waveguided through the glass panels and emitted from the edge where PV cells are placed to convert the light to electrical current. b) When a voltage is applied, the liquid crystals reorient, as do the dye molecules. Absorption is reduced, allowing light to enter the room, producing a 'light' state. Naturally, the current generated is less, although the efficiency of generating current increases. Reproduced with permission.[64]

4. Current Efficiency Achievements and Conclusion

One challenge the LSC has to confront in order to position itself in the global solar-energy generation space is the misunderstanding of its functionality, and difficulty in describing the device with reference to other solar-energy generating systems. Despite its coloration and composition, the LSC should not be viewed as another type of organic photovoltaic (OPV) or, for that matter, as a solar cell at all. As such, direct comparisons of electron-generation efficiencies commonly reported for photovoltaic materials become close to meaningless. While it is possible to tabulate the reported results for efficiency measurements of luminescent solar concentrators over the years (such an attempt is to be seen in **Table 1**) it becomes obvious the confusion generated in making these comparisons.

The efficiencies reported in the table are entirely dependent on the nature of the attached solar cell. The reported net efficiency tells little or nothing about the features or level of performance of the waveguide itself, the most important component of the device. Thus, it becomes increasingly apparent to these authors another method for describing the performance of these devices be adopted, perhaps some manner of 'total photon-in/photon-out' fraction, weighted in some way to account for the standard response for a variety of solar cells. The final line of the table refers to an as-yet unpublished result stemming from a collaboration between Philips Research and the Eindhoven University of Technology. This latter device consists of two waveguides, each filled with a different fluorescent dye (Red305 on top and a perylenebisimidazole, Figure 10t, as the bottom waveguide) with silicon solar cells attached to two orthogonal edges.

This paper has been written with the intent of providing a complete overview of the research done on the luminescent solar concentrator over the past thirty-five years. Almost all of this work has been carried out in fits and starts by a number of labs working independently around the globe. The authors of this overview believe it is precisely this fragmentation that has prevented more rapid performance improvement of the devices during that time. For the true leap in performance, a coordinated effort bringing together and combining the many talents of these research teams would go far towards making LSCs a viable option for the marketplace.

There are a great number of improvements that can and need to be made on the LSC to make it a more viable option for use in the urban environment. One aspect the authors find particularly intriguing is to provide an opportunity for the use of OPVs. One of the greatest challenges for the OPV has been the ability for the organic molecules that make up the absorbing/transport layers efficient at utilizing the UV portion of the spectrum, as well as survive the high energies of UV light which causes premature degradation of OPVs through destruction of the dye materials. However, the LSC does not illuminate the attached solar cell with a solar spectrum, but with a much more narrow band of light, generally in the NIR. It is in precisely this range of wavelengths that OPVs perform their best. Coupled with the lack of exposure to UV light, this could provide the OPV with the first real niche application where it could excel.

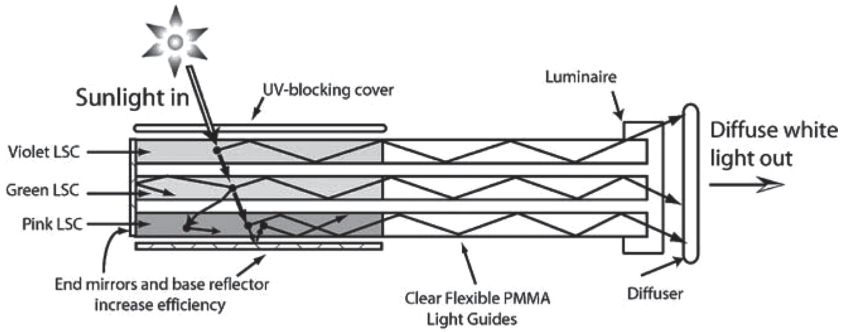

Figure 18. Schematic of a three-color LSC stack connected to light guides and luminaire. Reproduced with permission.[222] Copyright 2004, Elsevier.

The single most important improvement before the LSC may come into general use still needs to be the luminophore, even after more than three decades of research. There is still no luminophore with a broad

© 2012 WILEY-VCH Verlag GmbH & Co. KGaA, Weinheim

Table 1. Reported efficiencies of luminescent solar concentrator devices.

Luminophore	Cell type [# edges]	LSC size [cm]	Efficiency [%]	Reference
Coumarin, Rhodamine(?)	Si	?	1.9	[48]
Not stated	Si	?	2.5	[48]
DCM	Si	120 × 100 × 0.4	1.3	[48]
Coumarin, Rhodamine	Si	120 × 100 × 0.4	1.3	[48]
Not stated	Si	40 × 40 × 0.3	2.1	[43]
Not stated	GaAs	?	2.5	[43]
Not stated, 2 plates	Si	40 × 40 × 0.6	3.0	[43]
Not stated, 2 plates	GaAs	40 × 40 × 0.6	4.0	[43]
Coumarin, Rhodamine	Si	140 × 140 × 3	3.2	[225]
CdSe/CdS QDs	GaAs	140 × 140 × 3	4.5	[225]
CdSe/CdS QD	Si	5 × 5 × 0.3 Perspex	2.1	[89]
Red305	Si	5 × 5 × 0.3 Perspex	3.3	[89]
Red305	Si	5 × 5 × 0.3	2.4	[40]
Red305/CRS040	Si	5 × 5 × 0.3	2.7	[40]
Red305,CRS040	mc-Si (1)	5 × 5 × 0.5	2.7	[44]
Red305,CRS040	GaAs (1)	5 × 5 × 0.5	4.6	[44]
Red305,CRS040	GaAs (4)	5 × 5 × 0.5	7.1	[44]
BA241	GaInP (4)	2 × 2 × 0.3	5.1	[45]
BA241,BA856	GaInP (4)	2 LSCs at 2 × 2 × 0.3	6.7	[45]
BA241	GaInP (1)	5 × 10 × 0.5	2.6	[45]
Not detailed	a-Si	5 × 5 × 0.5 Lucite	≈0.7	[183]
CdSe core/multishell QDs	Si	4.95 × 3.1 × 0.4	2.8	[226]
Red305, Perylene perinone	Si(2)	2 LSCs at 5 × 5 × 0.5	4.3	[227]

spectral absorption, high absorption efficiency over the whole absorption spectrum, a large Stokes Shift, a high luminescent efficiency (quantum yield), a matching spectrum of the emitted photons to the best spectral response of the PV cell (≈1.14 eV for silicon), a high photostability, and good solubility in the host-matrix material. Organic dyes still have a small spectral absorption width, a relatively low Stokes shift, and moderate photostability, while quantum dots lack the quantum yield in combination with a large Stokes shift and solubility, and for rare Earth materials the absorption coefficient and quantum yields are still too low. Phosphors[76] are alternative materials that have relatively high quantum yields, good absorption properties, high photostability, and a high Stokes shift, but the solubility in an organic matrix is still a problem.

There are also still photons in the solar spectrum that can hardly be used by the mentioned luminophores, such as photons with lower energy than the bandgap of the PV cell. To use these types of photons upconverting systems could be an option, combining multiple low-energy photons into a single, higher-energy photon that could be used by the cell.[228,229] Another option is to include materials that may absorb a single higher-energy photon well above the photovoltaic bandgap and emit multiple photons closer to the cell bandgap, known as quantum-cutting.[230,231]

Another obstacle that must be overcome is the misrepresentation of the capabilities of the device: for years it has been espoused as a future replacement of PV panels for rooftops. We believe this is a mistake. On South-facing rooftops with direct lighting (free from shading), where space and efficiency are at a premium, standard silicon-based photovoltaics would seem

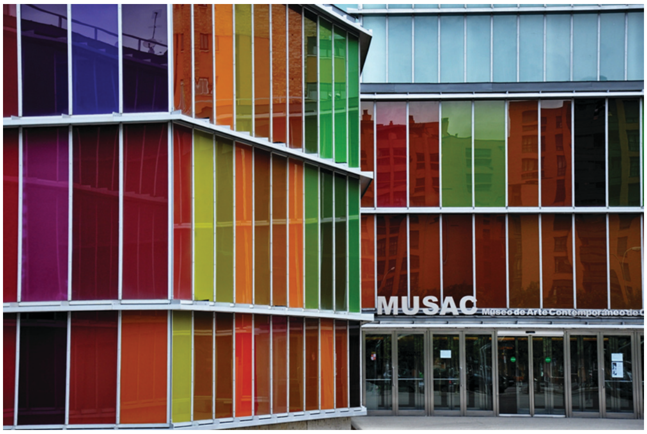

Figure 19. Photograph of the Musac museum in Léon, Spain (photo courtesy Eduardo Sentchordi).

the best option. However, there are huge areas that do not fall into these categories that could be prime locations for the use of LSCs. There are a large number of examples of luminescent objects being incorporated in the environment already, even though the coloration performs no additional function except for visual impact: for an example, consider the Musac museum in Léon, Spain (**Figure 19**). These types of structures exist in our environment anyway, and it would not appear to be such a great stretch to add energy-generating functionality. What is necessary is to bring the architectural and building industries into the conversation as these devices are developed. The tremendous design freedom afforded by the devices could no doubt be well-exploited by the visions of these industries.

It is thus the opinion of the authors that the role that the LSC has to play in a future urban renewable energy plan should be redefined. Given the decrease in the costs associated with traditional silicon-based PV, it would seem folly to attempt to compete directly with the well-established, traditional PV panel on a rooftop. Rather, the LSC could best find its role as a complement to silicon PV rather than a competitor, positioning itself in areas not normally accessible, such as areas with increased fractions of diffuse light. The LSC is designed to be brought directly into the public eye, given its flexibility in shape and color, rather than 'hidden away' as most silicon-based PV panels. By addition of molecular species to make the device 'smart'—responsive to its environment—and working alongside urban planners, the LSC could be used as a large area, low-cost source of solar electricity with an aesthetic advantage. Applications could include sound barriers beside roadways, telephone poles, bus stop roofing, atrium panels, and the like.

Supporting Information

Supporting Information is available from the Wiley Online Library or from the author.

Acknowledgements

M. Debije acknowledges the support of the Stichting voor Technische Wetenschappen (STW) VIDI grant 7940.

Received: September 14, 2011
Published online: December 12, 2011

[1] R. Judkoff, *MRS Bulletin* **2008**, *33*, 449.
[2] Directive 2010/31/EU of the European parliament and of the council of 19 May 2010 on the energy performance of buildings.
[3] For example, see www.Solarbuzz.com, which provides a regular survey of photovoltaic module and solar electricity generation costs,. Accessed: November, **2011**.
[4] E. Klampaftis, B. S. Richards, *Prog. Photovolt: Res. Appl.* **2011**, *19*, 345.
[5] H.-J. Yang, C.-H. Chen, W.-C. Lai, C.-L. Wu, C.-F. Huang, Y.-H. Chen, J.-C. Hwang, *J. Electrochem. Soc.* **2011**, *158*, H851.
[6] P. G. Nicholson, F. A. Castro, *Nanotechnology* **2010**, *21*, 492001.
[7] X. Zhan, D. Zhu, *Polym. Chem.* **2010**, *1*, 409.
[8] C. Deibel, V. Dyakonov, *Rep. Prog. Phys.* **2010**, *73*, 096401.
[9] R. F. Service, *Science* **2011**, *332*, 293.
[10] A. Kovach, J. Schmid, *Sol. Energ.* **1996**, *57*, 117.
[11] M. C. Alonso-García, J. M. Ruiz, F. Chenlo, *Sol. Energ. Mater. Sol. C.* **2006**, *90*, 329.
[12] R. M. Swanson, *Prog. Photovoltaics* **2000**, *8*, 93.
[13] V. C. Coffey, *Optics & Photonics News* **2011**, *22*, 23.
[14] A. Zacharopoulos, P. C. Eames, D. McLarnon, B. Norton, *Sol. Energ.* **2000**, *68*, 439.
[15] J. S. Batchelder, A. H. Zewail, T. Cole, *Appl. Optics* **1979**, *18*, 3090.
[16] W. H. Weber, J. Lambe, *Appl. Optics* **1976**, *15*, 2299.
[17] A. Goetzberger, W. Greubel, *Appl. Phys.* **1977**, *14*, 123.
[18] J. A. Levitt, W. H. Weber, *Appl. Optics* **1977**, *16*, 2684.
[19] E. E. Bende, L. H. Slooff, A. R. Burgers, W. G. J. H. M. van Sark, M. Kennedy, in Proc. 23rd Euro. Photovolt. Sol. Energ. Conf.,Valencia, Spain, WIP-Munich, **2008**, p.461.
[20] M. J. Currie, J. K. Mapel, T. D. Heidel, S. Goffri, M. A. Baldo, *Science* **2008**, *321*, 226.
[21] D. Chemisana, *Renew. Sust. Energ. Rev.* **2011**, *15*, 603.
[22] A. Goetzberger, *Appl. Phys.* **1978**, *16*, 399.
[23] M. Carrascosa, S. Unamuno, F. Agullo-Lopez, *Appl. Optics* **1983**, *22*, 3236.
[24] R. Kondepudi, S. Srinivasan, *Sol. Energ. Mater.* **1990**, *20*, 257.
[25] F. Vollmer, W. Rettig, *J. Photochem. Photobiol. A* **1996**, *95*, 143.
[26] L. Danos, P. Kittidachachan, T. J. J. Meyer, R. Greef, T. Markvart, in Proc. 21st Euro. Photovolt. Sol. Energ. Conf.,Dresden, Germany, WIP-Munich, **2006**.
[27] T. M. De Jong, D. K. G. De Boer, C. W. M. Bastiaansen, *Opt. Express* **2011**, *19*, 15127.
[28] A. Goetzberger, J. C. Goldschmidt, M. Peters, P. Löper, *Sol. Energ. Mater. Sol. C.* **2008**, *92*, 1570.
[29] L. H. Slooff, E. E. Bende, T. Budel, in Proc. 24th Euro. Photovolt. Sol. Energ. Conf.,Hamburg, Germany, WIP-Munich, **2009**, p.336.
[30] R. Tremblay, Patent US4505264 **1985**.
[31] E. Yablonovitch, *J. Opt. Soc. Am.* **1980**, *70*, 1362.
[32] G. P. Smestad, H. Ries, R. Winston, E. Yablonovitch, *Sol. Energ. Mater.* **1990**, *21*, 99.
[33] R. W. Olson, R. F. Loring, M. D. Fayer, *Appl. Optics* **1981**, *20*, 2934.
[34] V. Wittwer, A. Goetzberger, K. Heidler, in Proc. 4th E. C. Photovolt. Sol. Energ. Conf., Stresa, Italy, WIP-Munich, **1982**, p.682.
[35] A. J. Chatten, K. W. J. Barnham, B. F. Buxton, N. J. Ekins-Daukes, M. A. Malik, *Semiconductors* **2004**, *38*, 909.
[36] T. J. J. Meyer, J. Hlavaty, L. Smith, E. R. Freniere, T. Markvart, Proc. SPIE 7211, **2009**, DOI:10.117/12.810922.
[37] B. S. Richards, K. R. McIntosh, in Proc. 21st Euro. Photovolt. Sol. Energ. Conf., Dresden, Germany, WIP-Munich, **2006**, 185.
[38] S. J. Gallagher, P. C. Eames, B. Norton, *Int. J. Ambient Energy* **2004**, *25*, 47.
[39] A. R. Burgers, L. H. Slooff, R. Kinderman, J. A. M. van Roosmalen, in Proc. 20th Euro. Photovolt. Sol. Energ. Conf., Munich, Germany, WIP-Munich, **2005**, 394.
[40] W. G. J. H. M. van Sark, K. W. J. Barnham, L. H. Slooff, A. J. Chatten, A. Büchtemann, A. Meyer, S. J. McCormack, R. Koole, D. J. Farrell, R. Bose, E. E. Bende, A. R. Burgers, T. Budel, J. Quilitz, M. Kennedy, T. Meyer, C. d. M. Donega, A. Meijerink, D. Vanmaekelbergh, *Opt. Express* **2008**, *16*, 21773.
[41] S. Tsoi, P. P. C. Verbunt, L. Xue, D. J. Broer, C. W. M. Bastiaansen, M. G. Debije, unpublished.
[42] T. Markvart, *J. Appl. Phys.* **2006**, *99*, 026101.
[43] V. Wittwer, W. Stahl, A. Goetzberger, *Sol. Energ. Mater.* **1984**, *11*, 187.
[44] L. H. Slooff, E. E. Bende, A. R. Burgers, T. Budel, M. Pravettoni, R. P. Kenny, E. D. Dunlop, A. Büchtemann, *Phys. Status Solidi—R* **2008**, *2*, 257.

© 2012 WILEY-VCH Verlag GmbH & Co. KGaA, Weinheim

[45] J. C. Goldschmidt, M. Peters, A. Bösch, H. Helmers, F. Dimroth, S. W. Glunz, G. P. Willeke, *Sol. Energ. Mater. Sol. C.* **2009**, *93*, 176.
[46] M. G. Debije, P. P. C. Verbunt, B. C. Rowan, B. S. Richards, T. Hoeks, *Appl. Optics* **2008**, *47*, 6763.
[47] S. McDowall, B. L. Johnson, D. L. Patrick, *J. Appl. Phys.* **2010**, *108*, 053508.
[48] J. S. Batchelder, A. H. Zewail, T. Cole, *Appl. Optics* **1981**, *20*, 3733.
[49] A. M. Hermann, *Sol. Energ.* **1982**, *29*, 323.
[50] M. van Gurp, Y. K. Levine, *J. Chem. Phys.* **1989**, *90*, 4095.
[51] M. van Gurp, T. van Heijnsbergen, G. van Ginkel, Y. K. Levine, *J. Chem. Phys.* **1989**, *90*, 4103.
[52] C. Sánchez, B. Villacampa, R. Cases, R. Alcalá, C. Martínez, L. Oriol, M. Piñol, *J. Appl. Phys.* **2000**, *87*, 274.
[53] G. H. Heilmeier, L. A. Zanoni, *Appl. Phys. Lett.* **1968**, *13*, 91.
[54] R. L. van Ewyk, I. O'Connor, A. Mosley, A. Cuddy, C. Hilsm, C. Blackburn, J. Griffiths, F. Jones, *Displays* **1986**, *7*, 155.
[55] H. W. Schmidt, *Adv. Mater.* **1989**, *7*, 218.
[56] R. Piñol, J. Lub, M. P. García, E. Peeters, J. L. Serrano, D. J. Broer, T. Sierra, *Chem. Mater.* **2008**, *20*, 6076.
[57] C. Nobile, L. Carbone, A. Fiore, R. Cingolani, L. Manna, R. Krahne, *J. Phys.—Condens. Mat.* **2009**, *21*, 264013.
[58] A. Montali, C. W. M. Bastiaansen, P. Smith, C. Weder, *Nature* **1998**, *392*, 261.
[59] P. P. C. Verbunt, A. Kaiser, K. Hermans, C. W. M. Bastiaansen, D. J. Broer, M. G. Debije, *Adv. Funct. Mater.* **2009**, *19*, 2714.
[60] P. P. C. Verbunt, C. Bastiaansen, D. J. Broer, M. G. Debije, in Proc. 24th Euro. Photovolt. Sol. Energ. Conf., Hamburg, Germany, WIP-Munich, **2009**, 381.
[61] R. W. MacQueen, Y. Y. Cheng, R. G. C. R. Clady, T. W. Schmidt, *Opt. Express* **2010**, *18*, A161.
[62] C. L. Mulder, P. D. Reusswig, A. M. Velázquez, H. Kim, C. Rotschild, M. A. Baldo, *Opt. Express* **2010**, *18*, A79.
[63] C. L. Mulder, P. D. Reusswig, A. P. Beyler, H. Kim, C. Rotschild, M. A. Baldo, *Opt. Express* **2010**, *18*, A91.
[64] M. G. Debije, *Adv. Funct. Mater.* **2010**, *20*, 1498.
[65] M. G. Debije, C. W. M. Bastiaansen, D. J. Broer, M. J. Escuti, C. Sanchez, Patent WO 2006/088370 **2006**.
[66] K. E. Vaughn, M. Sousa, D. Kang, C. Rosenblatt, *Appl. Phys. Lett.* **2007**, *90*, 194102.
[67] B. S. Richards, A. Shalav, R. P. Corkish, in Proc. 19th Euro. Photovolt. Sol. Energ. Conf., Paris, France, WIP-Munich,**2004**.
[68] U. Rau, F. Einsele, G. C. Glaeser, *Appl. Phys. Lett.* **2005**, *87*, 171101.
[69] M. G. Debije, R. H. L. van der Blom, D. J. Broer, C. W. M. Bastiaansen, in Proc. WREC IX, Florence, Italy, **2006**.
[70] J. C. Goldschmidt, S. W. Glunz, A. Gombert, G. P. Willeke, in Proc. 21st Euro. Photovolt. Sol. Energ. Conf., Dresden, Germany, WIP-Munich, **2006**, p.107.
[71] A. J. Chatten, D. J. Farrell, R. Bose, M. G. Debije, A. Büchtemann, K. W. J. Barnham, in Proc. 22nd Euro. Photovolt. Sol. Energ. Conf., Milan, Italy, WIP-Munich, **2007**, p.349.
[72] M. Peters, J. C. Goldschmidt, P. Looper, B. Bläsi, G. P. Willeke, in Proc. 23rd Euro. Photovolt. Sol. Energ. Conf., Valencia, Spain, WIP-Munich, **2008**.
[73] J. C. Goldschmidt, M. Peters, L. Prönneke, L. Steidl, R. Zentel, B. Bläsi, A. Gombert, S. W. Glunz, G. P. Willeke, U. Rau, *Phys. Stat. Sol.—A* **2008**, *205*, 2811.
[74] M. G. Debije, M.-P. Van, P. P. C. Verbunt, D. J. Broer, C. W. M. Bastiaansen, in Proc. 24th Euro. Photovolt. Sol. Energ. Conf., Hamburg, Germany, WIP-Munich, **2009**, p.373.
[75] M. G. Debije, M.-P. Van, P. P. C. Verbunt, M. J. Kastelijn, R. H. L. van der Blom, D. J. Broer, C. W. M. Bastiaansen, *Appl. Optics* **2010**, *49*, 745.
[76] D. K. G. De Boer, C.-W. Lin, M. P. Giesbers, H. J. Cornelissen, M. G. Debije, P. P. C. Verbunt, D. J. Broer, *Appl. Phys. Lett.* **2011**, *98*, 021111.
[77] J. Watanabe, W. R. Krigbaum, *J. Polym. Sci. Pol.Phys.* **1987**, *25*, 173.
[78] D. J. Broer, J. Lub, G. N. Mol, *Nature* **1995**, *378*, 467.
[79] G. M. Gajiev, V. G. Golubev, D. A. Kurdyukov, A. V. Medvedev, A. B. Pevtsov, A. V. Sel'kin, V. V. Travnikov, *Phys. Rev. B* **2005**, *72*, 205115.
[80] S. A. El-Daly, S. Hirayama, *J. Photochem. Photobiol. A* **1997**, *110*, 59.
[81] R. Sotí, E. Farkas, M. Hilbert, Z. Farkas, I. Ketskemety, *J. Luminescence* **1996**, *68*, 105.
[82] K. Geetha, M. Rajesh, V. P. N. Nampoori, C. P. G. Vallabhan, P. Radhakrishnan, *J. Opt. A: Pure Appl. Opt.* **2004**, *6*, 379.
[83] L. R. Wilson, B. C. Rowan, N. Robertson, O. Moudam, A. C. Jones, B. S. Richards, *Appl. Optics* **2010**, *49*, 1651.
[84] A. A. Earp, J. B. Franklin, G. B. Smith, *Sol. Energ. Mater. Sol. C.* **2011**, *95*, 1157.
[85] B. C. Rowan, L. Wilson, B. S. Richards, in Proc. 24th Euro. Photovolt. Sol. Energ. Conf., Hamburg, Germany, WIP-Munich, **2009**, p.346.
[86] D. K. G. De Boer, C. R. Ronda, W. Keur, A. Meijerink, Proc. SPIE 8108, **2011**, DOI: 10.11712.893902.
[87] K. Barnham, J. L. Marques, J. Hassard, *Appl. Phys. Lett.* **2000**, *76*, 1197.
[88] A. J. Chatten, K. W. J. Barnham, B. F. Buxton, N. J. Ekins-Daukes, M. A. Malik, *Sol. Energ. Mater. Sol. C.* **2003**, *75*, 363.
[89] S. J. Gallagher, B. Norton, P. C. Eames, *Sol. Energy* **2007**, *81*, 813.
[90] V. Sholin, J. D. Olson, S. A. Carter, *J. Appl. Phys.* **2007**, *101*, 123114.
[91] G. V. Shcherbatyuk, R. H. Inman, C. Wang, R. Winstron, S. Ghosh, *Appl. Phys. Lett.* **2010**, *96*, 191901.
[92] A. M. Taleb, *Renew. Energ.* **2002**, *26*, 137.
[93] M. G. Debije, P. P. C. Verbunt, P. J. Nadkarni, S. Velate, K. Bhaumik, S. Nedumbamana, B. C. Rowan, B. S. Richards, T. Hoeks, *Appl. Optics* **2011**, *50*, 163.
[94] C. F. Rapp, N. L. Boling, Patent US 7915210 **1979**.
[95] W. Viehmann, R. L. Frost, *Nucl. Instr. Meth.* **1979**, *167*, 405.
[96] R. Reisfeld, M. Eyal, V. Chernyak, R. Zusman, *Sol. Energ. Mater.* **1988**, *17*, 439.
[97] T. Dienel, C. Bauer, I. Dolamic, D. Brühwiler, *Sol. Energy* **2010**, *84*, 1366.
[98] R. Reisfeld, *Opt. Mater.* **2010**, *32*, 850.
[99] W. R. L. Thomas, Patent US 4488047 (A), **1984**.
[100] A. J. Chatten, R. Bose, D. J. Farrell, Y. Xiao, N. L. A. Chan, L. Manna, A. Büchtemann, J. Quilitz, M. G. Debije, K. W. J. Barnham, in *Nanotechnology for Photovoltaics* (Ed: L. Tsakalakos), Taylor and Francis, London **2010**, p.323.
[101] R. Reisfeld, *J. Fluoresc.* **2002**, *12*, 317.
[102] M. J. Kastelijn, C. W. M. Bastiaansen, M. G. Debije, *Opt. Mater.* **2009**, *31*, 1720.
[103] S. T. Bailey, G. E. Lokey, M. S. Hanes, J. D. M. Shearer, J. B. McLafferty, G. T. Beaumont, T. T. Baseler, J. M. Layhue, D. R. Broussard, Y.-Z. Zhang, B. P. Wittmershaus, *Sol. Energ. Mater. Sol. C.* **2007**, *91*, 67.
[104] R. Bose, M. Gonzalez, P. Jenkins, R. Walters, J. P. Morseman, M. W. Moss, C. E. McLain, P. Linsert, A. Büchtemann, A. J. Chatten, K. W. J. Barnham, in Proc. (PVSC), Honalulu, USA, IEEE **2010**, p.467.
[105] O. Altan Bozdemir, S. Erbas-Cakmak, O. O. Ekiz, A. Dana, E. U. Akkaya, *Angew. Chem. Int. Ed.* **2011**, DOI: 10.1002/anie.201104846.

© 2012 WILEY-VCH Verlag GmbH & Co. KGaA, Weinheim

[106] D. Bruhwiler, G. Calzaferri, T. Torres, J. H. Ramm, N. Gartmann, L.-Q. Dieu, I. Lopez-Duarte, M. V. Martinez-Diaz, *J. Mater. Chem.* **2009**, *19*, 8040.
[107] G. Calzaferri, R. Méallet-Renault, D. Brühwiler, R. Pansu, I. Dolamic, T. Dienel, P. Adler, H. Li, A. Kunzmann, *Chem PhysChem* **2011**, *12*, 580.
[108] S. Tsoi, D. J. Broer, C. W. M. Bastiaansen, M. G. Debije, *Opt. Express* **2010**, *18*, A536.
[109] S. Tsoi, C. W. M. Bastiaansen, M. G. Debije, in Proc. 24th Euro. Photovolt. Sol. Energ. Conf., Hamburg, Germany, WIP-Munich, **2009**, p.377.
[110] N. C. Giebink, G. P. Wiederrecht, M. R. Wasielewski, *Nat. Photonics* **2011**, DOI: 10.1038/nphoton.2011.236.
[111] M. A. El-Shahawy, A. F. Mansour, *J. Mater. Sci. Mater. Elect.* **1996**, *7*, 171.
[112] B. A. Swartz, T. Cole, A. H. Zewail, *Opt. Lett.* **1977**, *1*, 73.
[113] J. M. Drake, M. L. Lesiecki, J. Sansregret, W. R. L. Thomas, *Appl. Optics* **1982**, *21*, 2945.
[114] I. Baumberg, O. Berezin, A. Drabkin, B. Gorelik, L. Kogan, M. Voskobojnik, M. Zaidman, *Polym. Degrad. Stabil.* **2001**, *73*, 403.
[115] A. F. Mansour, H. M. A. Killa, S. Abd El-Wanees, M. Y. El-Sayed, *Polym. Test.* **2005**, *24*, 519.
[116] R. Kinderman, L. H. Slooff, A. R. Burgers, N. J. Bakker, A. Büchtemann, R. Danz, J. A. M. van Roosmalen, *J. Sol. E* **2007**, *129*, 277.
[117] J. Yoon, L. Li, A. V. Semichaevsky, J. H. Ryu, H. T. Johnson, R. G. Nuzzo, J. A. Rogers, *Nature Comm.* **2011**, *2*, 343.
[118] L. R. Wilson, B. S. Richards, *Appl. Optics* **2009**, *48*, 212.
[119] G. Seybold, G. Wagenblast, *Dyes Pigm.* **1989**, *11*, 303.
[120] R. Reisfeld, D. Shamrakov, C. Jorgensen, *Sol. Energ. Mater. Sol. C.* **1994**, *33*, 417.
[121] A. F. Mansour, *Polym. Test.* **1998**, *17*, 333.
[122] A. F. Mansour, M. G. El-Shaarawy, S. M. El-Bashir, M. K. El-Mansy, M. Hammam, *Polym. Int.* **2002**, *51*, 393.
[123] F. Castiglione, G. Lanzani, A. Mele, A. Monguzzi, M. Passarello, A. Ruggirello, F. Scotognella, V. T. Liveri, *J. Mater. Sci.* **2011**, *46*, 6402.
[124] S. M. Reda, *Sol. Energ.* **2007**, *81*, 755.
[125] C. L. Mulder, L. Theogarajan, M. Currie, J. K. Mapel, M. A. Baldo, M. Vaughn, P. Willard, B. D. Bruce, M. W. Moss, C. E. McLain, J. P. Morseman, *Adv. Mater.* **2009**, *21*, 3181.
[126] Y. Ren, M. Szablewski, G. H. Cross, *Appl. Optics* **2000**, *39*, 2499.
[127] N. A. Bakr, A. F. Mansour, M. Hammam, *J. Appl. Polym. Sci.* **1999**, *74*, 3316.
[128] A. Dubois, M. Canva, A. Brun, F. Chaput, J.-P. Boilot, *Appl. Optics* **1996**, *35*, 3193.
[129] R. O. Al-Kaysi, T. Sang Ahn, A. M. Muller, C. J. Bardeen, *Phys. Chem. Chem. Phys.* **2006**, *8*, 3453.
[130] J. Mugnier, Y. Dordet, J. Pouget, B. Valeur, *Revue Phys. Appl.* **1987**, *22*, 89.
[131] A. F. Mansour, *Polym. Test.* **2004**, *23*, 247.
[132] L. Liu, Q. Peng, Y. Li, *Inorg. Chem.* **2008**, *47*, 5022.
[133] O. I. Micic, H. M. Cheong, H. Fu, A. Zunger, J. R. Sprague, A. Mascarenhas, A. J. Nozik, *J. Phys. Chem. B* **1997**, *101*, 4904.
[134] O. I. Micic, C. J. Curtis, K. M. Jones, J. R. Sprague, A. J. Nozik, *J. Phys. Chem.* **1994**, *98*, 4966.
[135] D. J. Norris, M. G. Bawendi, *Phys. Rev. B,* **1996**, *53*, 16338.
[136] X. Wang, J. Zhang, A. Nazzal, M. Xiao, *Appl. Phys. Lett.* **2003**, *83*, 162.
[137] M. Lomascolo, A. Cretì, G. Leo, L. Vasanelli, L. Manna, *Appl. Phys. Lett.* **2003**, *82*, 418.
[138] C. de Mello Donega, S. G. Hickey, S. F. Wuister, D. Vanmaekelbergh, A. Meijerink, *J. Phys. Chem. B* **2002**, *107*, 489.
[139] S. M. Reda, *Acta Mater.* **2008**, *56*, 259.
[140] A. Schüler, M. Python, M. V. del Olmo, E. de Chambrier, *Solar Energy CISBAT 2005* **2007**, *81*, 1159.
[141] Y. Nosaka, *J. Phys. Chem.* **1991**, *95*, 5054.
[142] P. E. Lippens, M. Lannoo, *Phys. Rev. B,* **1989**, *39*, 10935.
[143] M. Kennedy, S. J. McCormack, J. Doran, B. Norton, *Sol. Energ.* **2009**, *83*, 978.
[144] R. Hardman, *Environ. Health Perspect.* **2006**, *114*.
[145] J. L. Pelley, A. S. Daar, M. A. Saner, *Toxicol. Sci.* **2009**, *112*, 276.
[146] M. Bottrill, M. Green, *Chem. Commun.* **2011**, *47*, 7039.
[147] S. J. Byrne, Y. Williams, A. Davies, S. A. Corr, A. Rakovich, Y. K. Gun'ko, Y. P. Rakovich, J. F. Donegan, Y. Volkov, *Small* **2007**, *3*, 1152.
[148] F. Erogbogbo, K.-T. Yong, I. Roy, G. Xu, P. N. Prasad, M. T. Swihart, *ACS Nano* **2008**, *2*, 873.
[149] K. Fujioka, M. Hiruoka, K. Sato, N. Manabe, R. Miyasaka, S. Hanada, A. Hoshino, R. D. Tilley, Y. Manome, K. Hirakuri, K. Yamamoto, *Nanotechnology* **2008**, *19*, 415102.
[150] R. Reisfeld, Y. Kalisky, *Chem. Phys. Lett.* **1981**, *80*, 178.
[151] C. Lurin, C. Parent, G. Le Flem, P. Hagenmuller, *J. Phys. Chem. Solids* **1985**, *46*, 1083.
[152] C. Parent, C. Lurin, G. Le Flem, P. Hagenmuller, *J. Luminescence* **1986**, *36*, 49.
[153] R. Reisfeld, S. Neuman, *Nature* **1978**, *274*, 144.
[154] N. Neuroth, R. Haspel, *Sol. Energ. Mater.* **1987**, *16*, 235.
[155] R. Reisfeld, Y. Kalisky, *Nature* **1980**, *283*, 281.
[156] R. Reisfeld, *J. Less-Common Met.* **1983**, *93*, 243.
[157] R. Reisfeld, *Proc. Int. Symp. Eng. Ceremics* **1985**, *71*, 375.
[158] A. Buch, M. Ish-Shalom, R. Reisfeld, A. Kisilev, E. Greenberg, *Proc. Int. Symp. Eng. Ceremics* **1985**, *71*, 383.
[159] R. Reisfeld, *Inorg. Chim. Acta* **1984**, *95*, 69.
[160] O. Moudam, B. C. Rowan, M. Alamiry, P. Richardson, B. S. Richards, A. C. Jones, N. Robertson, *Chem. Commun.* **2009**, 6649.
[161] G. Katsagounos, E. Stathatos, N. B. Arabatzis, A. D. Keramidas, *J. Luminescence* **2011**, *131*, 1776.
[162] K. Heidler, *Appl. Optics* **1981**, *20*, 773.
[163] J. Roncali, F. Garnier, *Sol. Cells* **1984**, *13*, 133.
[164] A. A. Earp, G. B. Smith, P. D. Swift, J. Franklin, *Sol. Energ.* **2004**, *76*, 655.
[165] M. G. Debije, J.-P. Teunissen, M. J. Kastelijn, P. P. C. Verbunt, C. W. M. Bastiaansen, *Sol. Energ. Mater. Sol. C.* **2009**, *93*, 1345.
[166] M. G. Debije, W. Dekkers, unpublished.
[167] W. R. Holland, D. G. Hall, *Opt. Lett.* **1985**, *10*, 414.
[168] H. R. Wilson, *Sol. Energ. Mater.* **1987**, *16*, 223.
[169] K. Aslan, R. Badugu, J. R. Lakowicz, C. D. Geddes, *J. Fluor.* **2005**, *15*, 99.
[170] R. Reisfeld, M. Pietraszkiewicz, T. Saraidarov, V. Levchenko, *J. Rare Earth* **2009**, *27*, 544.
[171] T. K. Sau, A. L. Rogach, F. Jäckel, T. A. Klar, J. Feldmann, *Adv. Mater.* **2010**, *22*, 1805.
[172] V. E. Ferry, J. N. Munday, H. A. Atwater, *Adv. Mater.* **2010**, *22*, 4794.
[173] B. P. Rand, P. Peumans, S. R. Forrest, *J. Appl. Phys.* **2004**, *96*, 7519.
[174] K. R. Catchpole, A. Polman, *Opt. Express* **2008**, *16*, 21793.
[175] D. Duche, P. Torchio, L. Escoubas, F. Monestier, J.-J. Simon, F. Flory, G. Mathian, *Sol. Energ. Mater. Sol. C* **2009**, *93*, 1377.
[176] A. J. Morfa, K. L. Rowlen, I. T. H. Reilly, M. J. Romero, J. v. d. Lagemaat, *Appl. Phys. Lett.* **2008**, *92*, 013504.
[177] F.-C. Chen, J.-L. Wu, C.-L. Lee, Y. Hong, C.-H. Kuo, M. H. Huang, *Appl. Phys. Lett.* **2009**, *95*, 013305.
[178] J. H. Lee, J. H. Park, J. S. Kim, D. Y. Lee, K. Cho, *Org. Electron.* **2009**, *10*, 416.

© 2012 WILEY-VCH Verlag GmbH & Co. KGaA, Weinheim

[179] S.-Y. Wang, D.-A. Borca-Tasciuc, D. A. Kaminski, *J. Appl. Phys.* **2011**, *109*, 074910.
[180] Y.-F. Xiao, C.-L. Zou, Y.-W. Hu, Y. Li, L. Xiao, F.-W. Sun, Q. Gong, *IEEE J. Quantum Elect.* **2011**, *47*, 1171.
[181] A. Royne, C. J. Dey, D. R. Mills, *Sol. Energ. Mater. Sol. C* **2005**, *86*, 451.
[182] J. S. C. Prentice, *Sol. Energ. Mater. Sol. C* **2001**, *69*, 303.
[183] D. J. Farrell, W. G. J. H. M. v. Sark, S. T. Velthuijsen, R. E. I. Schropp, *Phys. Stat. Solid. C* **2010**, *7*, 1045.
[184] H. J. Hovel, R. T. Hodgson, J. M. Woodall, *Sol. Energ. Mater.* **1979**, *2*, 19.
[185] N. Yamada, L. N. Anh, T. Kambayashi, *Sol. Energ. Mater. Sol. C.*. **2010**, *94*, 413.
[186] W. Ding, R. Jia, D. Wu, C. Chen, H. Li, X. Liu, T. Ye, *J. Appl. Phys.* **2011**, *109*, 054312.
[187] J. Zhou, Y. Teng, S. Ye, X. Liu, J. Qiu, *Opt. Mater.* **2010**, *33*, 153.
[188] D. Geyer, H.-D. Mohring, S. Schröder, J. Springer, S. Rexroth, R. Schäffler, in Proc. 20th Euro. Photovolt. Sol. Energ. Conf., Barcelona, Spain, WIP-Munich, **2005**, p.2514.
[189] J. J. Schermer, G. J. Bauhuis, P. Mulder, E. J. Haverkamp, P. K. Larsen, *Thin Solid Films* **2006**, *511–512*, 645.
[190] A. W. Haines, Z. Liang, M. A. Woohouse, B. A. Gregg, *Chem. Rev.* **2010**, *110*, 6689.
[191] B. Fisher, J. Biddle, *Sol. Energ. Mater. Sol. C.* **2011**, *95*, 1741.
[192] R. Koeppe, *Appl. Phys. Lett.* **2007**, *90*, 181126.
[193] S. Chattopadhyay, Y. F. Huang, Y. J. Jen, A. Ganguly, H. H. Chen, L. C. Chen, *Mater. Sci. Eng.* **2010**, *69*, 1.
[194] U. Schulz, *Appl. Phys.* **2006**, *45*, 1608.
[195] W. R. L. Thomas, J. M. Drake, M. L. Lesiecki, *Appl. Optics* **1983**, *22*, 3440.
[196] J. Sansregret, J. M. Drake, W. R. L. Thomas, M. L. Lesiecki, *Appl. Optics* **1983**, *22*, 573.
[197] G. W. Chantry, J. W. Fleming, P. M. Smith, M. Cudby, H. A. Willis, *Chem. Phys. Lett.* **1971**, *10*, 473.
[198] H. Ma, A. K.-Y. Jen, L. R. Dalton, *Adv. Mater.* **2002**, *14*, 1339.
[199] J. Zubia, J. Arrue, *Opt. Fiber Technol.* **2001**, *7*, 101.
[200] J. R. White, *CR Chim.* **2006**, *9*, 1396.
[201] P. Polishuk, *IEEE Communications Magazine* **2006**, *44*, 140.
[202] M. Buffa, S. Carturan, M. G. Debije, P. P. C. Verbunt, A. Quaranta, G. Maggioni, in Proc. E-MRS,**2011**.
[203] M. Sidrach de Cardona, M. Carrasscosa, F. Meseguer, F. Cusso, F. Jaque, *Sol. Cells* **1985**, *15*, 225.
[204] A. Fantoni, M. Vierira, J. Cruz, R. Schwarz, R. Martins, *J. Phys. D: Appl. Phys.* **1996**, *29*, 3154.
[205] Z. H. Lu, Q. Song, S. Q. Li, Q. Yao, A. Othman, in Proc ISES Solar World Congress 2007, **2007**, 1445.
[206] A. Vishnoi, R. Gopal, R. Dwivedi, S. K. Srivatava, *Int. J. Electronics* **1989**, *66*, 755.
[207] A. Goetzberger, O. Schirmer, *Appl. Phys.* **1979**, *19*, 53.
[208] E. Loh Jr., D. J. Scalapino, *Appl. Optics* **1986**, *25*, 1901.
[209] A. H. Zewail, J. S. Batchelder, Patent US 4227939, **1980**.
[210] K. R. McIntosh, N. Yamada, B. S. Richards, *Appl. Phys. B* **2007**, *88*, 285.
[211] C. Auronet, H. Blumenfeld, M. Bourdinaud, J. Calvet, J.-C. Cavan, J. Meyer, J.-C. Thevenin, Patent US4812013, **1989**.
[212] As presented by A. F. Abouraddy, E. Banaei, at TechConnect World **2011**, USA.
[213] A. J. Chatten, D. J. Farrell, R. Bose, A. Dixon, C. Poelking, K. C. Gödel, M. Mazzer, K. W. J. Barnham, in Proc.TechConnect World, Boston, USA, **2011**, p.669.
[214] J. Yoon, A. J. Baca, S.-I. Park, P. Elvikis, J. B. Geddes, L. Li, R. H. Kim, J. Xiao, S. Wang, T.-H. Kim, M. J. Motala, B. Y. Ahn, E. B. Duoss, J. A. Lewis, R. G. Nuzzo, P. M. Ferreira, Y. Huang, A. Rockett, J. A. Rogers, *Nature Mater.* **2008**, *7*, 907.
[215] P. Fath, H. Nussbaumer, R. Brukhardt, *Sol. Energ. Mater. Sol. C* **2002**, *74*, 127.
[216] T. Nakada, Y. Hirabayashi, T. Tokado, D. Ohmori, T. Mise, *Sol. Energ.* **2004**, *77*, 739.
[217] S. Bao, K. Tajima, Y. Yamada, M. Okada, K. Yoshimura, *Appl. Phys. A* **2007**, *87*, 621.
[218] C. G. Granqvist, *Sol. Energ. Mater. Sol. C* **2008**, *92*, 203.
[219] C. Bechinger, B. A. Gregg, *Sol. Energ. Mater. Sol. C* **1998**, *54*, 405.
[220] G. A. Niklasson, C. G. Granqvist, *J. Mater. Chem.* **2007**, *17*, 127.
[221] M-C. Dubois, *Solar Shading and Building Energy Use*, Lund Institute of Technology, Lund, Sweden **1997**, p.100.
[222] A. A. Earp, G. B. Smith, J. Franklin, P. D. Swift, *Sol. Energ. Mater. Sol. C* **2004**, *84*, 411.
[223] A. Zastrow, V. Wittwer, *SPIE* **1986**, *653*, 93.
[224] G. B. Smith, J. B. Franklin, Patent US 6059438 **2000**.
[225] P. S. Friedman, C. R. Parent, *Luminescent Solar Concentrator Development - Final Subcontract Report*, Contract DE-AC02-83CH10093, US Department of Energy, **1984.**
[226] J. Bomm, A. Büchtemann, A. J. Chatten, R. Bose, D. J. Farrell, N. L. A. Chan, Y. Xiao, L. H. Slooff, T. Meyer, A. Meyer, W. G. J. H. M. van Sark, R. Koole, *Sol. Energ. Mater. Sol. C* **2011**, *95*, 2087.
[227] L. Desmet, D. K. G. de Boer, A.J.M. Ras, M. G. Debije, P. P. C. Verbunt, LSC measurement a result of joint project between Philips Research and the Eindhoven University of Technology, **2011**.
[228] H.-Q. Wang, M. Batentschuk, A. Osvet, L. Pinna, C. J. Brabec, *Adv. Mater.* **2011**, *23*, 2675.
[229] J. de Wild, A. Meijerink, J. K. Rath, W. G. J. H. M. van Sark, R. E. I. Schropp, *Energy Environ. Sci.* **2011**, DOI: 10.1039/C1EE01659H.
[230] C. Ronda, *J. Luminescence* **2002**, *100*, 301.
[231] B. M. van der Ende, L. Aarts, A. Meijerink, *Adv. Mater.* **2009**, *21*, 3073.

© 2012 WILEY-VCH Verlag GmbH & Co. KGaA, Weinheim

Thermo Fisher
SCIENTIFIC

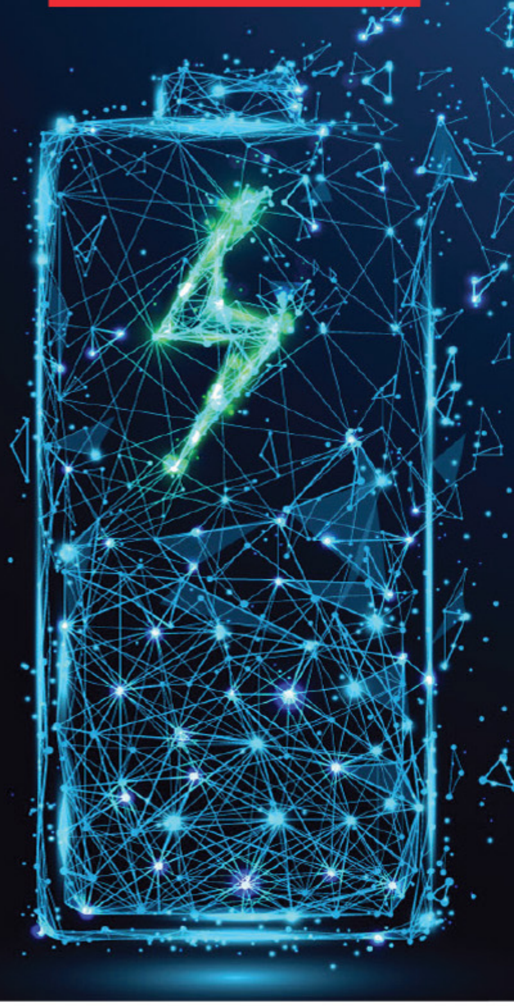

Lithium-ion battery

PowerUp webinars

Analytical solutions for lithium-ion battery material analysis and testing

PowerUp your battery material analysis - Join our educational webinars

Heading toward zero emission goals, lithium-ion battery are expected to generate an unprecedented demand for battery raw material in the upcoming decade. At the same time battery research, production, and quality control will need to keep up with the accelerated demand for electric storage capacities. Battery manufacturers must deliver consistently high quality throughout the entire battery value chain. Analysis and testing of batteries raw material and components requires therefore a variety of analytical methods that provide insights of quality and properties at various scales.

In this webinar session we will highlight the benefits of several analytical techniques and applications, as chromatography, mass spectrometry and electron microscopy, in battery material and structural analysis.

Free education webinars
October 5 and 6, 2022 | 10:00 BST | 11:00 CEST
Part 1: Analysis of cathode materials
Part 2: Analysis of battery electrolytes

No time for live session? Register anyway and get access to the on-demand recording after the live broadcast.

Key learnings

- Learn about analytical solutions for lithium-ion battery material and structural analysis
- Discover how IC, GC-MS, ICP-OES, and SEM support battery value chain from raw materials to recycling
- Get deeper insights based-on selected applications and performance examples

Learn more at **thermofisher.com/battery-webinars**

thermo scientific

For Research Use Only. Not for use in diagnostic procedures. © 2022 Thermo Fisher Scientific Inc. All rights reserved. All trademarks are the property of Thermo Fisher Scientific and its subsidiaries unless otherwise specified. **fl-001396-em-en**

High-Efficiency and Reliable Smart Photovoltaic Windows Enabled by Multiresponsive Liquid Crystal Composite Films and Semi-Transparent Perovskite Solar Cells

Yu Xia, Xiao Liang, Yun Jiang, Shaofu Wang, Yuyang Qi, Yumin Liu, Li Yu,* Huai Yang,* and Xing-Zhong Zhao*

Smart photovoltaic windows (SPWs) are functional devices possessing the capabilities of electrical power output, energy saving, and privacy protection by managing sunlight under external stimuli and potentially applicable in the fields of energy-saving buildings, automobiles, and switchable optoelectronics. However, long response time, low power conversion efficiency (PCE), poor stability and cycling performance, and monostimuli responsive behavior restrict their practical applications. To address these issues, high-efficiency and reliable SPWs are demonstrated by coupling multiresponsive liquid crystal/polymer composite (LCPC) films and semi-transparent perovskite solar cells (ST-PSCs). In this design, fast and multiple stimuli-responsive LCPC films are utilized as an inside layer to control the transparency of SPWs. The ST-PSCs with competitive PCE and qualified transparency acting as an outside layer offer energy generation functionality. Benefiting from repeatable transparency transition modulated by external stimuli, a series of working modes are achieved in the SPWs providing distinguished and stable energy generation, energy saving, and privacy protection performances.

1. Introduction

The strained relationship between human beings and their surrounding environment caused by rapid development of science and technology has become an inescapable issue for both industrial and scientific fields.[1,2] To address this issue, it is urgent and essential to explore technologies for sustainable and efficient utilization of the energy.[3–5] Smart photovoltaic windows (SPWs) have been considered as a promising green technology due to their capability to integrate electrical power output, energy saving, and privacy protection into a single device by modulating the transmittance of sunlight under external stimuli including temperature, light, electric field, or moisture.[6–10] Utilizing SPWs as a key architectural component can efficiently reduce building service energy usage comprising ventilation, heating, and air conditioning which are major part of energy overconsumption.[11,12]

Generally, SPWs are categorized into two types according to their architectures and components. One type is smart photovoltaic cells containing stimuli-responsive absorption component.[13,14] The photovoltaic cells can reversibly switch between highly transparent state and opaque state triggered by external stimuli due to the phase transition of absorption component, such as $CsPbI_{3-x}Br_x$ and $CH_3NH_3PbI_3{\cdot}xCH_3NH_2$.[13,14] In this case, smart photovoltaic cells can achieve energy saving by the manipulation of the transmission of sunlight in addition to original function of converting solar energy directly into electrical power. The other type is the assembly of solar cells and chromogenic layer.[15–20] In this system, the solar cells only take the role of energy conversion while external chromogenic layer, as smart component, modulates the transparency

Y. Xia, Y. Jiang, Y. Y. Qi, Dr. Y. M. Liu, Prof. X.-Z. Zhao
Institute for Interdisciplinary Research (IIR)
Key Laboratory of Optoelectronic Chemical Materials and Devices
Ministry of Education
Jianghan University
Wuhan 430056, China
E-mail: ymliu@jhun.edu.cn

X. Liang, Prof. H. Yang
College of Engineering
Peking University
Beijing 100871, China
E-mail: yanghuai@pku.edu.cn

The ORCID identification number(s) for the author(s) of this article can be found under https://doi.org/10.1002/aenm.201900720.

DOI: 10.1002/aenm.201900720

S. F. Wang, Prof. X.-Z. Zhao
School of Physics and Technology
Key Laboratory of Artificial Micro/Nano Structures
Ministry of Education
Wuhan University
Wuhan 430072, China

Dr. L. Yu
Hubei Collaborative Innovation Center for Advanced Organic Chemical Materials
Key Laboratory for the Green Preparation and Application of Functional Materials
Ministry of Education
Hubei Key Laboratory of Polymer Materials
School of Materials Science and Engineering
Hubei University
Wuhan 430062, China
E-mail: li.yu@hubu.edu.cn

© 2019 WILEY-VCH Verlag GmbH & Co. KGaA, Weinheim

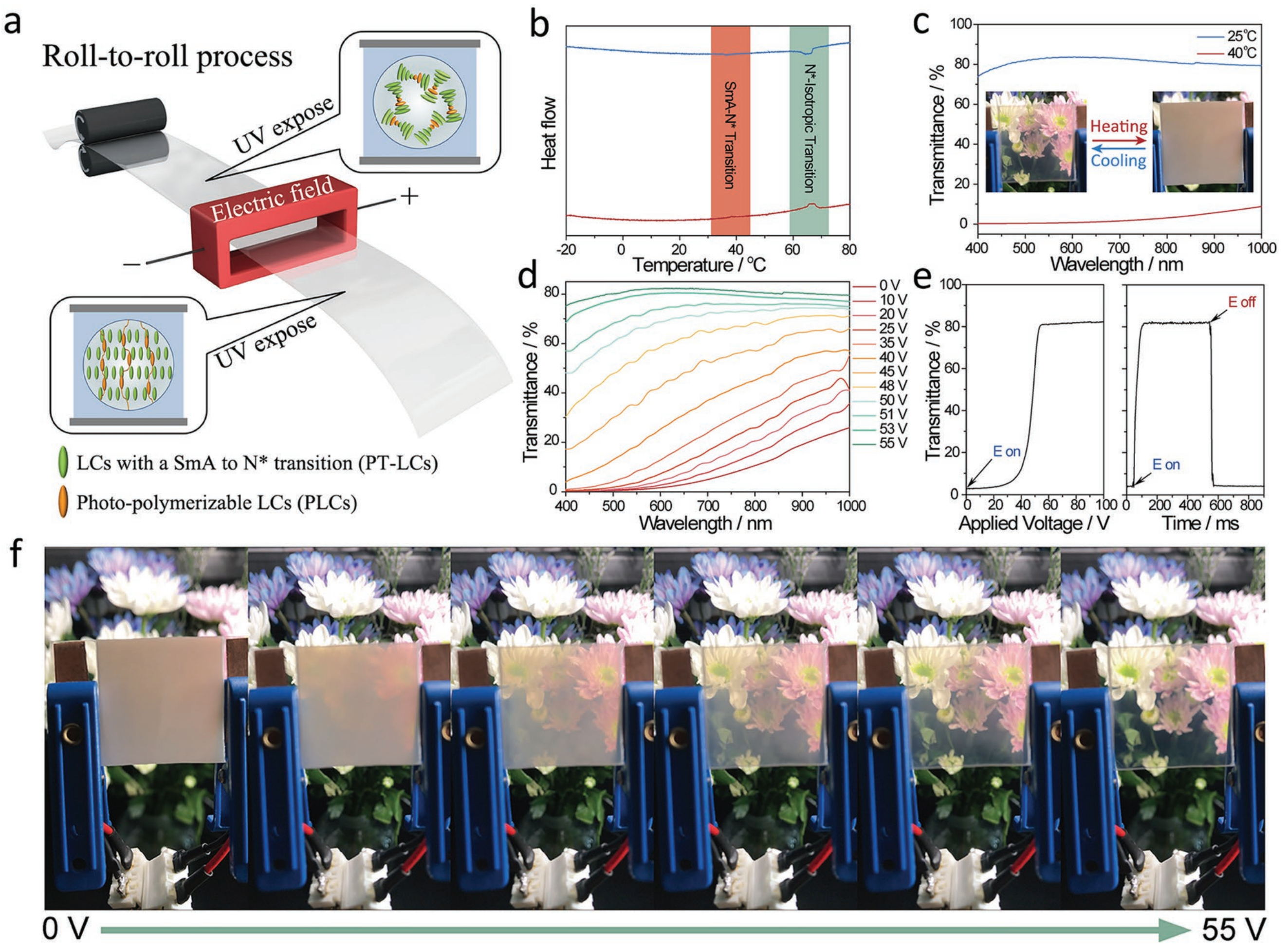

Figure 1. a) Schematic illustration of the roll-to-roll process for preparing multiresponsive LCPC films. b) Differential scanning calorimeter curve of the PT-LCs. c) The vis–NIR transmittance spectra of LCPC film at SmA (25 °C) and N* phase (40 °C). Insets show the photographs of LCPC film at these two phases. d) The vis–NIR transmittance spectra of N* phase LCPC film under various voltages. e) Electro-optical response of LCPC film. f) Photographs of N* phase LCPC film under various voltages in the range of 0 and 55 V.

according to the change of surrounding environment. Despite exciting progress in this field, all developed SPWs exist at least one of several limitations: long response time;[13] low power conversion efficiency (PCE) (<10%);[15–20] poor stability and cycling performance;[14] or monostimuli responsive behavior of smart component hard to meet the requirement for practical application.[15–20]

2. Results and Discussion

To overcome these limitations, we offer a facile avenue to design high-efficiency and reliable SPWs by coupling multiresponsive liquid crystal/polymer composites (LCPCs) and planar-structure semi-transparent perovskite solar cells (ST-PSCs). In our concept, LCPCs, a coexistent system containing polymer-dispersed LC (PDLC) and polymer stabilized LC (PSLC), are utilized as chromogenic layer due to their fast, reversible, and stable multiresponsive behaviors. As shown schematically in **Figure 1**a, there are three steps for utilizing roll-to-roll process to mass produce LCPC films (synthesis details are given in the Experimental Section and Figure S1, Supporting Information). First, homemade SmA-N* phase transition LCs (PT-LCs) (Figure 1b and Movies S1 and S2, Supporting Information), photopolymerizable LC monomer, non-LC polymerizable monomers, and photoinitiator were well mixed and injected into two plastic substrates. Then, the mixture was ultraviolet (UV) cured for a short time to initially form nonorientated PDLC and PSLC coexisted system resulting from the phase separation between LC and non-LC components. Finally, an electric field was used to homeotropically align LCs followed by further fully UV curing to obtain multiresponsive LCPCs (Figure S2, Supporting Information). The synthesized LCPC films represent high transparency of 79% at low temperature (–20–37.8 °C) due to similar refractive indices between non-LC part and LC part of SmA phase. Rising to high temperature (37.8–67 °C), LCPC films transform from highly transparent to strong light-scattering state (3% visible transparency) caused by the mismatch of refractive indices between non-LC part and LC part of N* phase (Figure 1b,c). Under external electric field, LC part can be modulated from N* phase to nematic phase of homeotropic alignment which possesses the refractive indices similar

 © 2019 WILEY-VCH Verlag GmbH & Co. KGaA, Weinheim

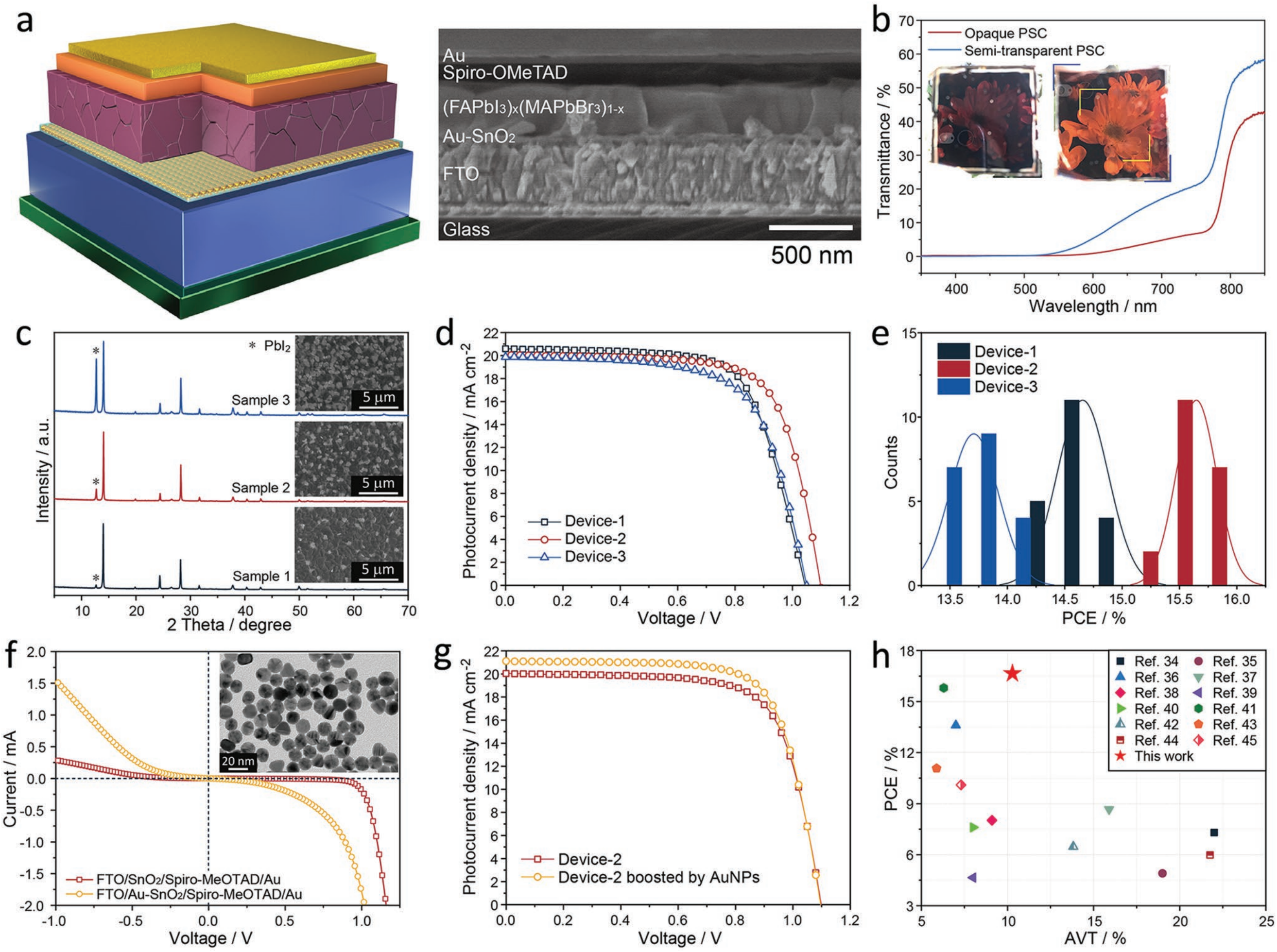

Figure 2. a) Schematic illustration of ST-PSC device configuration (left) and cross-sectional SEM image of corresponding complete device (right). b) UV–vis transmittance spectra of ST-PSC based on perovskite layer with different thickness. The insets show the photographs of the opaque (left) and semi-transparent device (right). c) X-ray diffraction patterns of perovskite samples with various amount of residual PbI_2. The insets show SEM images of corresponding perovskite samples. d) Photocurrent density–voltage (*J–V*) curves for the ST-PSCs with different residual PbI_2 amount on perovskite layer. The ST-PSC devices with low, moderate, and high residual PbI_2 amount in perovskite layer are labeled as Device-1, Device-2, and Device-3, respectively. e) Statistics of PCE for Device-1, Device-2, and Device-3. f) *I–V* characteristics of simple cells using SnO_2 and Au-SnO_2 nanocomposites as the ETL. The inset shows TEM image of AuNPs. g) *J–V* curves for Device-2 and Device-2 boosted by AuNPs. h) Comparative PCE-AVT charts for ST-PSCs. Our ST-PSC is labeled as red star.

to non-LC part.[21,22] As a result, LCPC films are able to change from opaque to highly transparent stimulated by external electric field at high temperature (37.8–67 °C). As presented in Figure 1d,f, the higher the applied voltage, the more transparent the films are. Under saturation voltage V_{sat} (55 V), highly transparent state of LCPCs (79%) can be rapidly recovered in ≈55 ms (Figure 1e). The switchable transparency behaviors stimulated by the temperature or electric field almost show no fatigue after 300 cycles indicating reversible and stable multiresponsive behaviors originating from the phase transition of LCs (Figure S3 and Movies S3 and S4, Supporting Information).

In addition to external chromogenic layer, LCPC films with reversible and stable multiresponsive behaviors, ST-SCs with high transparency and PCE are key to build up high-efficiency SPWs. In our work, metal halide perovskite based planar-structure PSCs (**Figure 2**a) are selected attributed to their high absorption coefficient, long charge diffusion length, and carrier life time.[23–27] However, there is an inevitable trade-off between PCE and transparency for ST-PSCs that relatively higher transparency realized by reducing the thickness of the perovskite layer would weaken the light harvesting capability of the perovskite layer resulting in negative effect on the performances of ST-PSCs. For example, the transparency of ST-PSCs is obviously improved when the thickness of perovskite layer decreases from 600 to 300 nm (Figure 2b and Figure S4, Supporting Information). On the contrary, the open-circuit voltage (V_{oc}) and short-circuit current density (J_{sc}) of the ST-PSCs drop with the decrease in thickness of perovskite layer (Figure S5, Supporting Information), Furthermore, the photocurrent attenuation of the ST-PSCs, especially in the region from 600 to 800 nm, is confirmed by the reduction of external quantum efficiency (EQE) and calculated integrated current densities (Figure S6, Supporting Information). For this balance, we modulate the amount of PbI_2 residue in the perovskite layer without

 © 2019 WILEY-VCH Verlag GmbH & Co. KGaA, Weinheim

changing its thickness in order to obtain good operational performance and comparatively higher transparency (Figure S7, Supporting Information). As presented in scanning electron microscope (SEM) images (Figure 2c, inset), the quantity of PbI_2 residue is controlled by the spin-coating speed of upper organic salt (formamidinium iodide (FAI)). Spin coating with higher speed leads less FAI left to react with PbI_2 so that more PbI_2 residue remains on the perovskite layer, which is proved by X-ray diffraction (XRD) patterns (Figure 2c). The influence of the amount of PbI_2 residue on photovoltaic performance of ST-PSCs is detailed shown in Figure 2d,e and Table S1 in the Supporting Information. Notably, ST-PSCs with moderate residual PbI_2 exhibit the highest PCE of 15.66% because moderate amount of residual PbI_2 can not only passivate the defects and reduce the recombination of the perovskite layer[28] but also act as a p-type semiconductor enhancing the carrier generation or hole transport.[29]

Moreover, we employ Au-SnO_2 nanocomposites as electron transport layer (ETL) to further improve PCE of ST-PSCs without sacrificing the transparency (Figure S8, Supporting Information). For this, an appropriate amount of as-synthesized Au nanoparticles (AuNPs) with a size around 15 nm (Figure 2f, inset) were added into commercial SnO_2 colloid precursor. The Au-SnO_2 colloid precursor shows a localized surface plasmon resonance (LSPR) peak centered at ≈520 nm, as shown in Figure S9 in the Supporting Information. Compared to ST-PSCs using SnO_2 as ETL, an improved J_{sc} from 20.10 to 21.13 mA cm^{-2} and a higher PCE of 16.67% are achieved by utilizing Au-SnO_2 as ETL (Figure 2g and Table S1, Supporting Information). As shown in Figure S10 in the Supporting Information, the EQE of the ST-PSC boosted by AuNPs exhibits a rise over the wavelength range of 400–800 nm, especially around the LSPR peak of AuNPs (≈520 nm), which confirms the role of LSPR effect of AuNPs for improving photovoltaic performance of the ST-PSCs. Furthermore, the electron transfer properties of the ETL based on SnO_2 and Au-SnO_2 nanocomposites are evaluated by measuring the I–V curve of simple cells composed of fluorine-doped tin oxide (FTO)/ETL/Spiro-OMeTAD/Au under dark, as shown in Figure 2f. In contrast to cell choosing SnO_2 as the ETL, the cell selecting Au-SnO_2 nanocomposites as the ETL display a higher current density (in the second quadrant) indicating that more electrons are injected from the ETL to FTO substrate.[30,31] Combining LSPR effect and enhanced electron transport, Device-2 boosted by AuNPs provides competitive PCE and average visible transmittance (AVT) for their application in SPWs (Figure 2h).[32–43]

In our design, SPWs are constructed by multiresponsive LCPC inside layer and planar-structure ST-PSC outside layer (Figure S11a, Supporting Information), labeled as ST-PSC/LCPC. Benefiting from multiresponsive behavior of LCPC layer (Figure 1), there are three distinct working modes for ST-PSC/LCPC (**Figure 3**a, and Movie S5, Supporting Information). At low temperature (–20–37.8 °C), ST-PSC/LCPC presents similar semi-transparent state as ST-PSCs due to high transparency of LCPCs at SmA phase (L-ST mode, Figure S11b, Supporting Information). Heating to high temperature (37.8–67 °C), ST-PSC/LCPC changes to opaque state because of strong light-scattering state of LCPCs caused by SmA to N* phase transition (H-LS mode, Figure S11c, Supporting Information). Stimulated by electric field, semi-transparent state of ST-PSC/LCPC can be recovered even at high temperature because of the phase transition of LCPCs from N* to nematic phase of homeotropic alignment (H-ST mode, Figure S11d, Supporting Information). The transitions between L-ST and H-LS modes stimulated by the temperature and that of between H-LS and H-ST modes stimulated by electrical field are stable and reversible. During the transition between different working modes, ST-PSC/LCPC exhibits reddish orange dominating by the color of ST-PSCs (Figure S12, Supporting Information). Among three working modes, ST-PSC/LCPC provides robust power output. As shown in photocurrent density–voltage (J–V) curves (Figure 3b), ST-PSC/LCPC possesses similar photovoltaic performance in L-ST and H-ST modes (**Table 1**). In H-LS mode, the photovoltaic performance of ST-PSC/LCPC is reinforced because strong light-scattering effect of inside layer LCPCs effectively increases the optical path length of the light and solar energy harvesting of the ST-PSCs. The J_{sc}, V_{oc}, fill factor (FF), and PCE reach as high as 22.69 mA cm^{-2}, 1.11 V, 71.88%, and 18.02%, respectively (Table 1). EQE curves of ST-PSC/LCPC under different modes are shown in Figure 3c. The calculated J_{sc} also confirms the contribution of the LCPCs to the device performances in H-LS mode, which is coincident with J–V characterizations. Moreover, there is no J–V hysteresis for ST-PSC/LCPC in three distinct working modes, which can be ascribed to enhanced charge transfer at the interfaces resulting from moderate residual PbI_2. To further confirm the reproducibility of ST-PSC/LCPC photovoltaic performance, over 60 individual devices are fabricated and tested under the same condition for each working mode. Average PCE for L-ST, H-LS, and H-ST modes is 16.66% ± 1.0%, 16.60% ± 0.9%, and 17.84% ± 0.9%, respectively (Figure 3d).

Besides achieving robust power output, our design addresses the issue of unstable and degraded photovoltaic performance during the switching between different working modes existed in SPWs based on ST-PSCs.[13,14] Even for unsealed ST-PSCs, PCE is stable after 50 cycle switches both between L-ST and H-LS modes triggered by the temperature (Figure 3e) and between H-LS and H-ST modes triggered by electrical field (Figure 3f). This impressively reliable photovoltaic performance of ST-PSC/LCPC originates from reversible and stable multiresponsive behaviors of inside LCPC layer. Furthermore, smart LCPCs in L-ST mode will not interfere with the illumination from the rear side of the ST-PSCs (Figure 3g, inset). When irradiated from the rear side, ST-PSC/LCPC in L-ST mode exhibits an efficiency of 6.52 % and 6.67 % under 1.0 and 0.3 sun illumination (Table S2, Supporting Information), respectively, as shown in Figure 3g.

The energy saving capability, an essential feature of SPWs, of ST-PSC/LCPC is also evaluated. As shown in **Figure 4**a, a xenon lamp with the intensity of 300 W is utilized as the light source to emulate sunlight and black plastic with good absorption is selected as a target object. The ST-PSC/LCPC is placed between the light source and target object. Using FTO glass as the reference, the heating effect of the light irradiation is obviously observed in Figure 4b. After continuous irradiation for 5 min, the temperature of target object becomes stable and rises from room temperature 22 to 53.2 °C due to high transparency of FTO glass (Figure S13, Supporting Information).

© 2019 WILEY-VCH Verlag GmbH & Co. KGaA, Weinheim

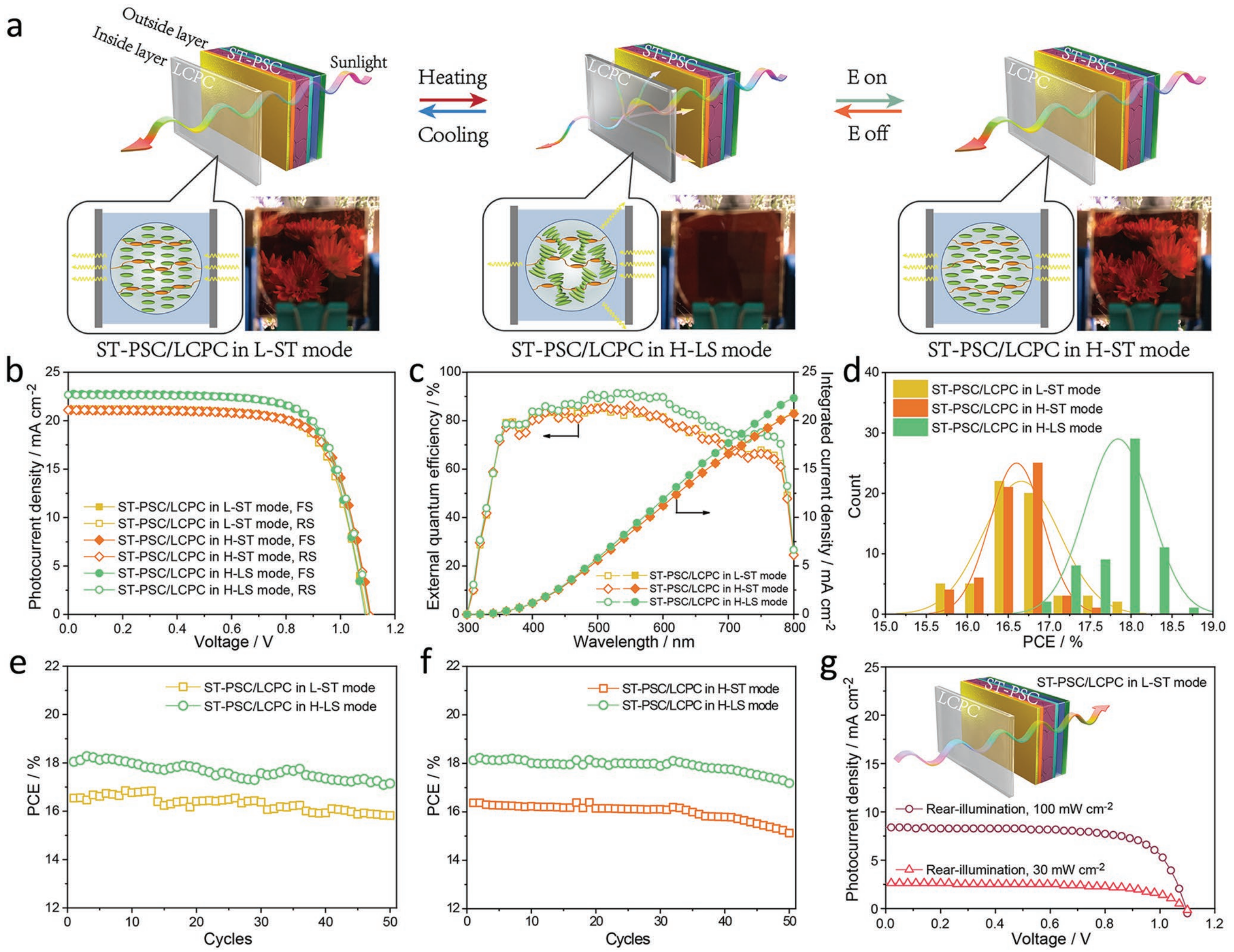

Figure 3. a) Schematic illustration of the configuration of designed SPWs, working principle of the SPWs at three different modes, L-ST, H-LS, and H-ST, and reversible switching behaviors between different modes. b) *J–V* curves, c) external quantum efficiency and integrated current density curves, and d) PCE distribution of ST-PSC/LCPC in three different working modes. The average PCE of ST-PSC/LCPC is obtained by Gaussian fitting. The PCE cycling curve of ST-PSC/LCPC during 50 switches e) between L-ST and H-LS modes stimulated by the temperature and f) between H-LS and H-ST modes stimulated by electrical field. g) *J–V* curves for ST-PSC/LCPC in L-ST mode from rear illumination under different incident light intensity.

For ST-PSC device, the temperature of target object goes up to 38.4 °C under the same illuminating condition (Figure 4c). Compared to FTO glass, the heating effect of light irradiation apparently weaken because a part of the light is absorbed by the ST-PSCs. For ST-PSC/LCPC, the temperature of target object slightly increases to 29.2 °C upon the same exposure (300 W xenon lamp for 5 min) indicating the heating effect of light irradiation is further depressed by the combination of strong

Table 1. Detailed photovoltaic parameters of ST-PSC/LCPC in three working modes under reverse and forward scan.

Working mode	Scan direction	J_{sc} a) [mA cm^{-2}]	V_{oc} b) [V]	FF c) [%]	PCE d) [%]
L-ST mode e)	RS f)	21.26	1.10	71.75	16.70
	FS g)	21.13	1.10	72.02	16.67
H-ST mode h)	RS	21.14	1.11	72.02	16.98
	FS	21.11	1.11	72.28	16.86
H-LS mode i)	RS	22.69	1.11	71.88	18.02
	FS	22.81	1.10	71.52	17.86

a) J_{sc}: short-circuit current density; b) V_{oc}: open-circuit voltage; c) FF: fill factor; d) PCE: power conversion efficiency; e) L-ST mode: low temperature semi-transparent state; f) RS: reverse scan; g) FS: forward scan; h) H-ST mode: high temperature semi-transparent state; i) H-LS mode: high temperature light-scattering state.

 © 2019 WILEY-VCH Verlag GmbH & Co. KGaA, Weinheim

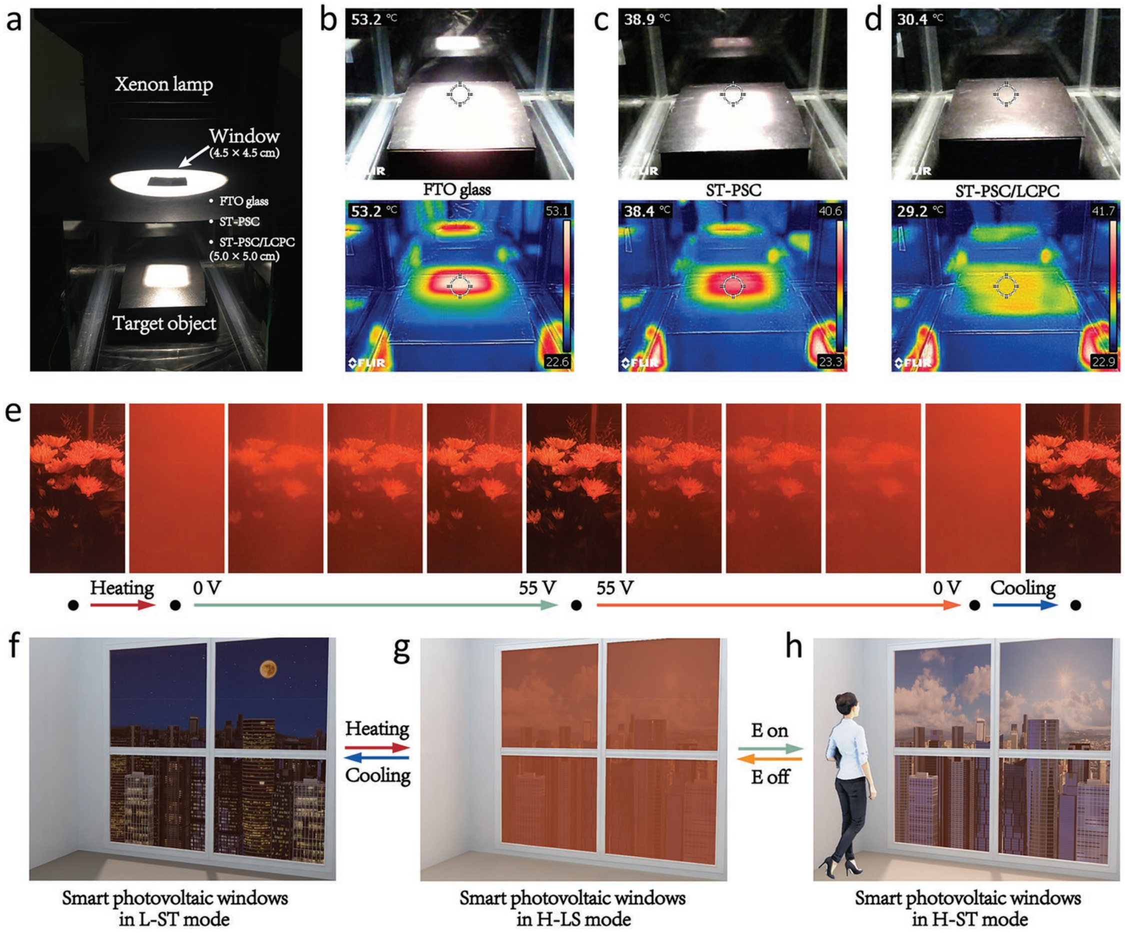

Figure 4. a) The setup for evaluating the energy saving property of ST-PSC/LCPC. Digital and thermal IR-camera images of target object corresponding to b) FTO glass, c) ST-PSC, and d) ST-PSC/LCPC for comparing energy saving property. e) Photographs of ST-PSC/LCPC with controllable transparency stimulated by the temperature and electrical field. f–h) Schematic illustration of ST-PSC/LCPC being applied in the architectures.

light-scattering effect of inside layer LCPCs in H-LS mode and absorption effect of outside layer ST-PSCs (Figure 4d). Additionally, low threshold transparency transition temperature (37.8 °C) and wide window temperature of ST-PSC/LCPC for H-LS mode (37.8–67 °C) make it potential for practical applications. When outside temperature is over 37.8 °C, which can be easily achieved under actual working conditions, ST-PSC/LCPC is activated into H-LS mode to block sunlight so that less energy for cooling load is required to balance the heating effect of light irradiation.

Multiple working modes in SPWs enabled by multistimuli responsive behavior of ST-PSC/LCPC have never been reported and represent a stepping-stone toward their real implementation (Figure 4e). Being integrated into energy-saving architectures, three different working modes achieved in ST-PSC/LCPC take indispensable and significant roles. For low temperature (–20–37.8 °C, L-ST mode), the ST-PSC/LCPC functions as normal windows with additional energy generation capability (Figure 4f). When the temperature increases to the range of 37.8–67 °C (H-LS mode), energy saving functionality and enhanced power output are realized by strong light-scattering state of the ST-PSC/LCPC (Figure 4g). Moreover, the transparency transformation from semi-transparent to opaque induced by the increase of the temperature can be utilized for privacy protection. Besides the temperature, the transparency of the ST-PSC/LCPC can be manipulated by electrical field according to occupant preference. Even on a hot day, we can watch the scenery outside the window after a long-time work anytime (Figure 4h). We anticipate these features of presented ST-PSC/LCPC can overcome the limitations for their practical applications in other fields, such as optoelectronics and automobiles.

3. Conclusions

In this work, we have demonstrated high-efficiency and reliable SPWs by combining multiresponsive LCPC films and ST-PSCs. Based on fast and stable multistimuli responsive behavior of LCPC films and competitive PCE and transparency of ST-PSCs, the designed SPWs with multiple working modes offer outstanding electrical power output, energy saving, and privacy protection performances. This concept overcomes the challenges for SPWs toward diverse application in energy-saving buildings, switchable optoelectronics, and automobiles.

© 2019 WILEY-VCH Verlag GmbH & Co. KGaA, Weinheim

4. Experimental Section

Materials: The SmA-N* phase transition LCs were synthesized according to the literature reported by Broer et al.[44] The N_e and N_o of homemade SmA-N* phase transition LCs are 1.51 and 1.72, respectively. RM82 (LC monomer, HCCH Company), hydroxypropyl methacrylate (HPMA, Tokyo Chemical Industry Co., Ltd), dodecyl methacrylate (LMA, Tokyo Chemical Industry Co., Ltd), polyethylene glycol diacrylate (PEGDA 600, Sartomer Co., Ltd), ethoxylate dimethacrylate (Bis-EMA15, Sigma-Aldrich), 2,2-dimethoxy-2-phenylacetophenone (Irgacure 651, Sigma-Aldrich), SnO_2 colloid precursor (tin (IV) oxide, Alfa Aesar). FAI, methylammonium bromide (MABr), and PbI_2, were purchased from Xi'an p-OLED Corp. *N,N*-dimethylformamide (DMF) and dimethylsulfoxide (DMSO) were obtained from Sigma-Aldrich. Hydrogen tetrachloroaurate(III) ($HAuCl_4{\cdot}3H_2O$) and trisodium citrate were purchased from Sinopharm Chemical Reagent Co., Ltd. All chemicals were used as received.

Preparation of Liquid Crystal/Polymer Composite (LCPC) Films: The LCPC films were prepared by a stepwise polymerization method proposed in our work.[45] First, homemade SmA-N* phase transition LCs, photopolymerizable LC (RM82), non-LC polymerizable monomers (HPMA, LMA, PEGDA 600, and Bis-EMA15), and photoinitiator (Irgacure 651) were homogeneously mixed and sandwiched between two pieces of transparent plastic. Then, the cell was illuminated by UV light for 90 s at room temperature (365 nm, 0.5 mw cm^{-2}). Finally, a voltage (square wave, 50 Hz, 150 V) was used to homeotropically orient LCs followed by UV curing for 5 min (365 nm, 0.5 mw cm^{-2}). The refractive index of polymer matrix (polymerized non-LC part) is 1.49.

Fabrication of Semi-Transparent Perovskite Solar Cells (ST-PSCs): FTO glass was cleaned by 2% Hellmanex III, deionized (DI) water, acetone, and ethanol sequentially. The SnO_2 colloid precursor was diluted by DI water to 2.67% and then spin coated on FTO substrates at 3000 rpm for 30 s and subsequently annealed in ambient air at 150 °C for 30 min. In order to prepare Au-SnO_2 colloid precursor, Au nanoparticles were first synthesized according to our previous literature.[46] Then, moderate amount of the as-synthesized AuNPs solution (0.45×10^{-3} M) was added into the diluted SnO_2 colloid precursor. The obtained Au-SnO_2 colloid precursor was deposited on FTO as the process mentioned above. After depositing the ETL, the substrates were treated with ultraviolet ozone for 15 min. Then the perovskite layer was deposited by a two-step spin-coating process. First, 1.3 M of PbI_2 in DMF/DMSO (9.5:0.5) was spin coated on the obtained ETL and then annealed at 70 °C for 1 min. After that, the mixture solution of FAI: MABr (60 mg:12 mg in 1 mL isopropanol) was spin coated onto the PbI_2, followed by thermal annealing at 150 °C for 15 min in glove box. The spin-coating speed of PbI_2 for all semi-transparent perovskite samples was fixed at 5000 rpm. The spin-coating rate of upper FAI layer was controlled at 3500, 4300, and 5100 rpm for sample 1, sample 2, and sample 3, respectively. For comparison, the opaque perovskite sample was obtained by the spin rate combination of 1500/1300 rpm. Then, the hole transporting material was spin coated on top of the obtained perovskite layer at 3000 rpm for 30 s, which consisted of 72.3 mg 2,2′,7,7′-tetrakis(*N,N*-dip-methoxyphenylamine)-9,9′-spiro-bifluorene (Spiro-OMeTAD), 35 µl bis(trifluoromethane) sulfonamide lithium salt (Li-TFSI) solution (170 mg Li-TFSI in 1 mL acetonitrile), and 28.8 µL 4-terbutylpyridine in 1 mL chlorobenzene. Finally, Au top-contact electrode with a thickness of ≈35 nm was thermal evaporated to fabricate completed devices.

Fabrication of Smart Photovoltaic Windows (SPWs): To fabricate SPWs, smart LCPC films were directly attached to the Au side of the ST-PSCs as shown in Figure S11a in the Supporting Information. The final ST-PSC/LCPC device for the performance test is shown in Figure S14 in the Supporting Information.

Characterizations: XRD measurements for all samples were performed on a PANalytical XRD system (X'Pert Powder) with Cu K_α radiation ($\lambda = 1.54056$ Å). The Vis–NIR transmittance spectra of the LCPC films were obtained by a UV/vis/NIR spectrophotometer (JASCO V-570) combined with a hot plate (Linkam LK-600PM). The optical textures of the LCs were characterized by polarizing optical microscopy (Carl Zeiss Axio Vision SE64) combined with a hot plate (Linkam LK-600PM). The Vis–NIR transmittance spectra of the LCPC films with various applied voltages were measured at 313 K by LC device parameters tester (LCT-5016C, Changchun Liancheng Instrument Co. Ltd., China) equipped with a hot plate (Linkam LK-600PM). The top-view and cross-section SEM images were taken on the field emission scanning electron microscopy (Hitachi SU8010). Transmission electron microscopy (TEM) and high-resolution TEM analyses were carried out using FEI Tecnai G20. UV–vis spectrophotometer (SHIMADZU, UV-2550) was used to measure the absorption and transmittance spectrum of all perovskite samples and devices. Photocurrent density–voltage (*J–V*) characteristics of the devices were taken under AM 1.5 simulated illuminations (100 mW cm^{-2}, Newport, 94023A). The light intensity was calibrated by means of a KG-5 Si diode and the 0.09 cm^2 nonreflective mask was used to determine the valid active area. EQE measurements of devices were taken on an EQE system (Newport, 66984) using monochromatic light ranging from 300 to 800 nm. The thermal images were recorded by a commercial infrared camera (FLIR, C2).

Supporting Information

Supporting Information is available from the Wiley Online Library or from the author.

Acknowledgements

Y.X. and X.L. contributed equally to this work. This project was supported by the National Key R&D Program of China (2018YFB0703704), the National Natural Science Foundation of China (51720105002, 61404060, and 51303007), the Hubei Provincial Natural Science Foundation of China (2018CFB508), and the Wuhan Youth Science and Technology Program (2015071704011602).

Conflict of Interest

The authors declare no conflict of interest.

Keywords

high-efficiency, liquid crystal composite films, multiresponsive, semi-transparent perovskite solar cells, smart photovoltaic windows

Received: March 3, 2019
Revised: May 31, 2019
Published online: July 18, 2019

[1] L. Gao, B. A. Bryan, *Nature* **2017**, *544*, 217.
[2] A. M. Omer, *Renewable Sustainable Energy Rev.* **2008**, *12*, 2265.
[3] Y. Zhai, Y. Ma, S. N. David, D. Zhao, R. Lou, G. Tan, R. Yang, X. Yin, *Science* **2017**, *355*, 1062.
[4] N. DeForest, A. Shehabi, J. O'Donnell, G. Garcia, J. Greenblatt, E. S. Lee, S. Selkowitz, D. J. Milliron, *Build. Environ.* **2015**, *89*, 107.
[5] C. Sun, R. X. Xia, H. Shi, H. F. Yao, X. Liu, J. H. Hou, F. Huang, H.-L. Yip, Y. Cao, *Joule* **2018**, *2*, 1657.
[6] C. Bechinger, S. Ferrer, A. Zaban, J. Sprague, B. A. Gregg, *Nature* **1996**, *383*, 608.
[7] A. L. Dyer, R. H. Bulloch, Y. H. Zhou, B. Kippelen, J. R. Reynolds, F. L. Zhang, *Adv. Mater.* **2014**, *26*, 4895.

© 2019 WILEY-VCH Verlag GmbH & Co. KGaA, Weinheim

[8] E. Amasawa, N. Sasagawa, M. Kimura, M. Taya, *Adv. Energy Mater.* **2014**, *4*, 1400379.
[9] F. Malara, A. Cannavale, S. Carallo, G. Gigli, *ACS Appl. Mater. Interfaces* **2014**, *6*, 9290.
[10] C. M. Lampert, *Sol. Energy Mater. Sol. Cells* **2003**, *76*, 489.
[11] Y. J. Ke, C. Z. Zhou, Y. Zhou, S. C. Wang, S. H. Chan, Y. Long, *Adv. Funct. Mater.* **2018**, *28*, 1800113.
[12] D. M. Kammen, D. A. Sunter, *Science* **2016**, *352*, 922.
[13] J. Lin, M. L. Lai, L. T. Dou, C. S. Kley, H. Chen, F. Peng, J. L. Sun, D. Lu, S. A. Hawks, C. L. Xie, F. Cui, A. P. Alivisatos, D. T. Limmer, P. D. Yang, *Nat. Mater.* **2018**, *17*, 261.
[14] L. M. Wheeler, D. T. Moore, R. Ihly, N. J. Stanton, E. M. Miller, R. C. Tenent, J. L. Blackburn, N. R. Neale, *Nat. Commun.* **2017**, *8*, 1722.
[15] N. C. Davy, M. Sezen-Edmonds, J. Gao, X. Lin, A. Liu, N. Yao, A. Kahn, Y.-L. Loo, *Nat. Energy* **2017**, *2*, 17104.
[16] A. Cannavale, G. E. Eperon, P. Cossari, A. Abate, H. J. Snaith, G. Gigli, *Energy Environ. Sci.* **2015**, *8*, 1578.
[17] F. Guo, S. Chen, Z. Chen, H. J. Luo, Y. F. Gao, T. Przybilla, E. Spiecker, A. Osvet, K. Forberich, C. J. Brabec, *Adv. Opt. Mater.* **2015**, *3*, 1524.
[18] A. Cannavale, M. Manca, L. D. Marco, R. Grisorio, S. Carallo, G. P. Suranna, G. Gigli, *ACS Appl. Mater. Interfaces* **2014**, *6*, 2415.
[19] H.-K. Kwon, K.-T. Lee, K. Hur, S. H. Moon, M. M. Quasim, T. D. Wilkinson, J.-Y. Han, H. Ko, I.-K. Han, B. Park, B. K. Min, B.-K. Ju, S. M. Morris, R. H. Friend, D.-H. Ko, *Adv. Energy Mater.* **2015**, *5*, 1401347.
[20] A. Cannavale, M. Manca, F. Malara, L. D. Marco, R. Cingolani, G. Gigli, *Energy Environ. Sci.* **2011**, *4*, 2567.
[21] D.-K. Yang, *J. Disp. Technol.* **2006**, *2*, 32.
[22] D. A. Higgins, *Adv. Mater.* **2000**, *12*, 251.
[23] M. V. Kovalenko, L. Protesescu, M. I. Bodnarchuk, *Science* **2017**, *358*, 745.
[24] M. Abdi-Jalebi, Z. Andaji-Garmaroudi, S. Cacovich, C. Stavrakas, B. Philippe, J. M. Richter, M. Alsari, E. P. Booker, E. M. Hutter, A. J. Pearson, S. Lilliu, T. J. Savenije, H. Rensmo, G. Divitini, C. Ducati, R. H. Friend, S. D. Stranks, *Nature* **2018**, *555*, 497.
[25] M. I. H. Ansari, A. Qurashi, M. K. Nazeeruddin, *J. Photochem. Photobiol., C* **2018**, *35*, 1.
[26] Q. Jiang, Z. M. Chu, P. Y. Wang, X. L. Yang, H. Liu, Y. Wang, Z. G. Yin, J. L. Wu, X. W. Zhang, J. B. You, *Adv. Mater.* **2017**, *29*, 1703852.
[27] T. Hwang, B. Lee, J. Kim, S. Lee, B. Gil, A. J. Yun, B. Park, *Adv. Mater.* **2018**, *30*, 1704208.
[28] Y. C. Kim, N. J. Jeon, J. H. Noh, W. S. Yang, J. Seo, J. S. Yun, A. Ho-Baillie, S. J. Huang, M. A. Green, J. Seidel, T. K. Ahn, S. I. Seok, *Adv. Energy Mater.* **2016**, *6*, 1502104.
[29] T. J. Jacobsson, J.-P. Correa-Baena, E. H. Anaraki, B. Philippe, S. D. Strankes, M. E. F. Bouduban, W. Tress, K. Schenk, J. Teuscher, J.-E. Moser, H. Rensmo, A. Hagfeldt, *J. Am. Chem. Soc.* **2016**, *138*, 10331.
[30] B. Peng, G. Jungmann, C. Jäger, D. Haarer, H.-W. Schmidt, M. Thelakkat, *Coord. Chem. Rev.* **2004**, *248*, 1479.
[31] S. Lee, J. H. Noh, H. S. Han, D. K. Yim, D. H. Kim, J.-K. Lee, J. Y. Kim, H. S. Jung, K. S. Hong, *J. Phys. Chem. C* **2009**, *113*, 6878.
[32] C. Roldán-Carmona, O. Malinkiewicz, R. Betancur, G. Longo, C. Momblona, F. Jaramillo, L. Camacho, H. J. Bolink, *Energy Environ. Sci.* **2014**, *7*, 2968.
[33] S. Aharon, M. Layani, B.-E. Cohen, E. Shukrun, S. Magdassi, L. Etgar, *Adv. Mater. Inter.* **2015**, *2*, 1500118.
[34] E. Della Gaspera, Y. Peng, Q. Hou, L. Spiccia, U. Bach, J. J. Jasieniak, Y.-B. Cheng, *Nano Energy* **2015**, *13*, 249.
[35] X.-L. Ou, M. Xu, J. Feng, H.-B. Sun, *Sol. Energy Mater. Sol. Cells* **2016**, *157*, 660.
[36] C. Hanmandlu, C.-Y. Chen, K. M. Boopathi, H.-W. Lin, C.-S. Lai, C.-W. Chu, *ACS Appl. Mater. Interfaces* **2017**, *9*, 32635.
[37] S. Rahmany, M. Layani, S. Magdassi, L. Etgar, *Sustainable Energy Fuels* **2017**, *1*, 2120.
[38] J.-H. Lu, Y.-L. Yu, S.-R. Chuang, C.-H. Yeh, C.-P. Chen, *J. Phys. Chem. C* **2016**, *120*, 4233.
[39] J. H. Heo, H. J. Han, M. Lee, M. Song, D. H. Kim, S. H. Im, *Energy Environ. Sci.* **2015**, *8*, 2922.
[40] K. Yang, F. Li, J. Zhang, C. P. Veeramalai, T. Guo, *Nanotechnology* **2016**, *27*, 095202.
[41] X. Dai, Y. Zhang, H. Shen, Q. Luo, X. Zhao, J. Li, H. Lin, *ACS Appl. Mater. Interfaces* **2016**, *8*, 4523.
[42] P. You, Z. Liu, Q. Tai, S. Liu, F. Yan, *Adv. Mater.* **2015**, *27*, 3632.
[43] L. Bu, Z. Liu, M. Zhang, W. Li, A. Zhu, F. Cai, Z. Zhao, Y. Zhou, *ACS Appl. Mater. Interfaces* **2015**, *7*, 17776.
[44] D. J. Broer, J. Boven, G. N. Mol, G. Challa, *Macromol. Chem. Phys.* **1989**, *190*, 2255.
[45] X. Liang, S. M. Guo, M. Chen, C. Y. Li, Q. Wang, C. Zou, C. H. Zhang, L. Y. Zhang, S. J. Guo, H. Yang, *Mater. Horiz.* **2017**, *4*, 878.
[46] Y. M. Liu, H. W. Zhai, F. Guo, N. Huang, W. W. Sun, C. H. Bu, T. Peng, J. K. Yuan, X. Z. Zhao, *Nanoscale* **2012**, *4*, 6863.

© 2019 WILEY-VCH Verlag GmbH & Co. KGaA, Weinheim

JOHN WILEY AND SONS LICENSE TERMS AND CONDITIONS

Sep 19, 2022

This Agreement between Harbin Institute of Technology -- Ruicong Zhang ("You") and John Wiley and Sons ("John Wiley and Sons") consists of your license details and the terms and conditions provided by John Wiley and Sons and Copyright Clearance Center.

License Number	5392430991357
License date	Sep 19, 2022
Licensed Content Publisher	John Wiley and Sons
Licensed Content Publication	Advanced Energy Materials
Licensed Content Title	High-Efficiency and Reliable Smart Photovoltaic Windows Enabled by Multiresponsive Liquid Crystal Composite Films and Semi-Transparent Perovskite Solar Cells
Licensed Content Author	Xing-Zhong Zhao, Huai Yang, Li Yu, et al
Licensed Content Date	Jul 18, 2019
Licensed Content Volume	9
Licensed Content Issue	33
Licensed Content Pages	8
Type of Use	Journal/Magazine
Requestor type	University/Academic
Is the reuse sponsored by or associated with a pharmaceutical or medical products company?	no
Format	Print and electronic
Portion	Figure/table
Number of figures/tables	2
Will you be translating?	No
Circulation	200 - 499
Title of new article	Advanced liquid crystal-based switchable optical devices for light protection applications: principles and strategies
Lead author	Ruicong Zhang, Zhibo Zhang, Jiecai Han, Lei Yang, Jiajun li, Zicheng Song Tianyu Wang, Jiaqi Zhu
Title of targeted journal	Light: Science & Applications
Publisher	Springer Nature
Expected publication date	Nov 2022
Portions	Figure 1a, Figure 3a 3g
Requestor Location	Harbin Institute of Technology<br>No. 92, Xidazhi Street, Nangang District<br><br>Harbin, 150080<br>China<br>Attn: Harbin Institute of Technology
Publisher Tax ID	EU826007151
Total	**0.00 USD**
Terms and Conditions	

TERMS AND CONDITIONS

This copyrighted material is owned by or exclusively licensed to John Wiley & Sons, Inc. or one of its group companies (each a"Wiley Company") or handled on behalf of a society with which a Wiley Company has exclusive publishing rights in relation to a particular work (collectively "WILEY"). By clicking "accept" in connection with completing this licensing transaction, you agree

that the following terms and conditions apply to this transaction (along with the billing and payment terms and conditions established by the Copyright Clearance Center Inc., ("CCC's Billing and Payment terms and conditions"), at the time that you opened your RightsLink account (these are available at any time at http://myaccount.copyright.com).

Terms and Conditions

- The materials you have requested permission to reproduce or reuse (the "Wiley Materials") are protected by copyright.

- You are hereby granted a personal, non-exclusive, non-sub licensable (on a stand-alone basis), non-transferable, worldwide, limited license to reproduce the Wiley Materials for the purpose specified in the licensing process. This license, **and any CONTENT (PDF or image file) purchased as part of your order,** is for a one-time use only and limited to any maximum distribution number specified in the license. The first instance of republication or reuse granted by this license must be completed within two years of the date of the grant of this license (although copies prepared before the end date may be distributed thereafter). The Wiley Materials shall not be used in any other manner or for any other purpose, beyond what is granted in the license. Permission is granted subject to an appropriate acknowledgement given to the author, title of the material/book/journal and the publisher. You shall also duplicate the copyright notice that appears in the Wiley publication in your use of the Wiley Material. Permission is also granted on the understanding that nowhere in the text is a previously published source acknowledged for all or part of this Wiley Material. Any third party content is expressly excluded from this permission.

- With respect to the Wiley Materials, all rights are reserved. Except as expressly granted by the terms of the license, no part of the Wiley Materials may be copied, modified, adapted (except for minor reformatting required by the new Publication), translated, reproduced, transferred or distributed, in any form or by any means, and no derivative works may be made based on the Wiley Materials without the prior permission of the respective copyright owner.**For STM Signatory Publishers clearing permission under the terms of the STM Permissions Guidelines only, the terms of the license are extended to include subsequent editions and for editions in other languages, provided such editions are for the work as a whole in situ and does not involve the separate exploitation of the permitted figures or extracts,** You may not alter, remove or suppress in any manner any copyright, trademark or other notices displayed by the Wiley Materials. You may not license, rent, sell, loan, lease, pledge, offer as security, transfer or assign the Wiley Materials on a stand-alone basis, or any of the rights granted to you hereunder to any other person.

- The Wiley Materials and all of the intellectual property rights therein shall at all times remain the exclusive property of John Wiley & Sons Inc, the Wiley Companies, or their respective licensors, and your interest therein is only that of having possession of and the right to reproduce the Wiley Materials pursuant to Section 2 herein during the continuance of this Agreement. You agree that you own no right, title or interest in or to the Wiley Materials or any of the intellectual property rights therein. You shall have no rights hereunder other than the license as provided for above in Section 2. No right, license or interest to any trademark, trade name, service mark or other branding ("Marks") of WILEY or its licensors is granted hereunder, and you agree that you shall not assert any such right, license or interest with respect thereto

- NEITHER WILEY NOR ITS LICENSORS MAKES ANY WARRANTY OR REPRESENTATION OF ANY KIND TO YOU OR ANY THIRD PARTY, EXPRESS, IMPLIED OR STATUTORY, WITH RESPECT TO THE MATERIALS OR THE ACCURACY OF ANY INFORMATION CONTAINED IN THE MATERIALS, INCLUDING, WITHOUT LIMITATION, ANY IMPLIED WARRANTY OF MERCHANTABILITY, ACCURACY, SATISFACTORY QUALITY, FITNESS FOR A PARTICULAR PURPOSE, USABILITY, INTEGRATION OR NON-INFRINGEMENT AND ALL SUCH WARRANTIES ARE HEREBY EXCLUDED BY WILEY AND ITS LICENSORS AND WAIVED BY YOU.

- WILEY shall have the right to terminate this Agreement immediately upon breach of this Agreement by you.

- You shall indemnify, defend and hold harmless WILEY, its Licensors and their respective directors, officers, agents and employees, from and against any actual or threatened claims, demands, causes of action or proceedings arising from any breach of this Agreement by you.

- IN NO EVENT SHALL WILEY OR ITS LICENSORS BE LIABLE TO YOU OR ANY OTHER PARTY OR ANY OTHER PERSON OR ENTITY FOR ANY SPECIAL, CONSEQUENTIAL, INCIDENTAL, INDIRECT, EXEMPLARY OR PUNITIVE DAMAGES, HOWEVER CAUSED, ARISING OUT OF OR IN CONNECTION WITH THE DOWNLOADING, PROVISIONING, VIEWING OR USE OF THE MATERIALS REGARDLESS OF THE FORM OF ACTION, WHETHER FOR BREACH OF CONTRACT, BREACH OF WARRANTY, TORT, NEGLIGENCE, INFRINGEMENT OR OTHERWISE (INCLUDING, WITHOUT LIMITATION, DAMAGES BASED ON LOSS OF PROFITS, DATA, FILES, USE, BUSINESS OPPORTUNITY OR CLAIMS OF THIRD PARTIES), AND WHETHER OR NOT THE PARTY HAS BEEN ADVISED OF THE POSSIBILITY OF SUCH DAMAGES. THIS LIMITATION SHALL APPLY NOTWITHSTANDING ANY FAILURE OF ESSENTIAL PURPOSE OF ANY LIMITED REMEDY PROVIDED HEREIN.

- Should any provision of this Agreement be held by a court of competent jurisdiction to be illegal, invalid, or unenforceable, that provision shall be deemed amended to achieve as nearly as possible the same economic effect as the original provision, and the legality, validity and enforceability of the remaining provisions of this Agreement shall not

be affected or impaired thereby.

- The failure of either party to enforce any term or condition of this Agreement shall not constitute a waiver of either party's right to enforce each and every term and condition of this Agreement. No breach under this agreement shall be deemed waived or excused by either party unless such waiver or consent is in writing signed by the party granting such waiver or consent. The waiver by or consent of a party to a breach of any provision of this Agreement shall not operate or be construed as a waiver of or consent to any other or subsequent breach by such other party.
- This Agreement may not be assigned (including by operation of law or otherwise) by you without WILEY's prior written consent.
- Any fee required for this permission shall be non-refundable after thirty (30) days from receipt by the CCC.
- These terms and conditions together with CCC's Billing and Payment terms and conditions (which are incorporated herein) form the entire agreement between you and WILEY concerning this licensing transaction and (in the absence of fraud) supersedes all prior agreements and representations of the parties, oral or written. This Agreement may not be amended except in writing signed by both parties. This Agreement shall be binding upon and inure to the benefit of the parties' successors, legal representatives, and authorized assigns.
- In the event of any conflict between your obligations established by these terms and conditions and those established by CCC's Billing and Payment terms and conditions, these terms and conditions shall prevail.
- WILEY expressly reserves all rights not specifically granted in the combination of (i) the license details provided by you and accepted in the course of this licensing transaction, (ii) these terms and conditions and (iii) CCC's Billing and Payment terms and conditions.
- This Agreement will be void if the Type of Use, Format, Circulation, or Requestor Type was misrepresented during the licensing process.
- This Agreement shall be governed by and construed in accordance with the laws of the State of New York, USA, without regards to such state's conflict of law rules. Any legal action, suit or proceeding arising out of or relating to these Terms and Conditions or the breach thereof shall be instituted in a court of competent jurisdiction in New York County in the State of New York in the United States of America and each party hereby consents and submits to the personal jurisdiction of such court, waives any objection to venue in such court and consents to service of process by registered or certified mail, return receipt requested, at the last known address of such party.

WILEY OPEN ACCESS TERMS AND CONDITIONS

Wiley Publishes Open Access Articles in fully Open Access Journals and in Subscription journals offering Online Open. Although most of the fully Open Access journals publish open access articles under the terms of the Creative Commons Attribution (CC BY) License only, the subscription journals and a few of the Open Access Journals offer a choice of Creative Commons Licenses. The license type is clearly identified on the article.

The Creative Commons Attribution License

The Creative Commons Attribution License (CC-BY) allows users to copy, distribute and transmit an article, adapt the article and make commercial use of the article. The CC-BY license permits commercial and non-

Creative Commons Attribution Non-Commercial License

The Creative Commons Attribution Non-Commercial (CC-BY-NC)License permits use, distribution and reproduction in any medium, provided the original work is properly cited and is not used for commercial purposes.(see below)

Creative Commons Attribution-Non-Commercial-NoDerivs License

The Creative Commons Attribution Non-Commercial-NoDerivs License (CC-BY-NC-ND) permits use, distribution and reproduction in any medium, provided the original work is properly cited, is not used for commercial purposes and no modifications or adaptations are made. (see below)

Use by commercial "for-profit" organizations

Use of Wiley Open Access articles for commercial, promotional, or marketing purposes requires further explicit permission from Wiley and will be subject to a fee.

Further details can be found on Wiley Online Library http://olabout.wiley.com/WileyCDA/Section/id-410895.html

Other Terms and Conditions:

v1.10 Last updated September 2015

Questions? customercare@copyright.com or +1-855-239-3415 (toll free in the US) or +1-978-646-2777.
