# Supplementary material for: Advanced liquid crystal-based switchable optical devices for light protection applications: principles and strategies
Source: Light Sci Appl. 2023 Jan 3;12:11. doi: 10.1038/s41377-022-01032-y (PMC9807646; doi:10.1038/s41377-022-01032-y)
Supplement: Supplementary file 2 — Fig 1 copyright promotion [file 41377_2022_1032_MOESM2_ESM.pdf]

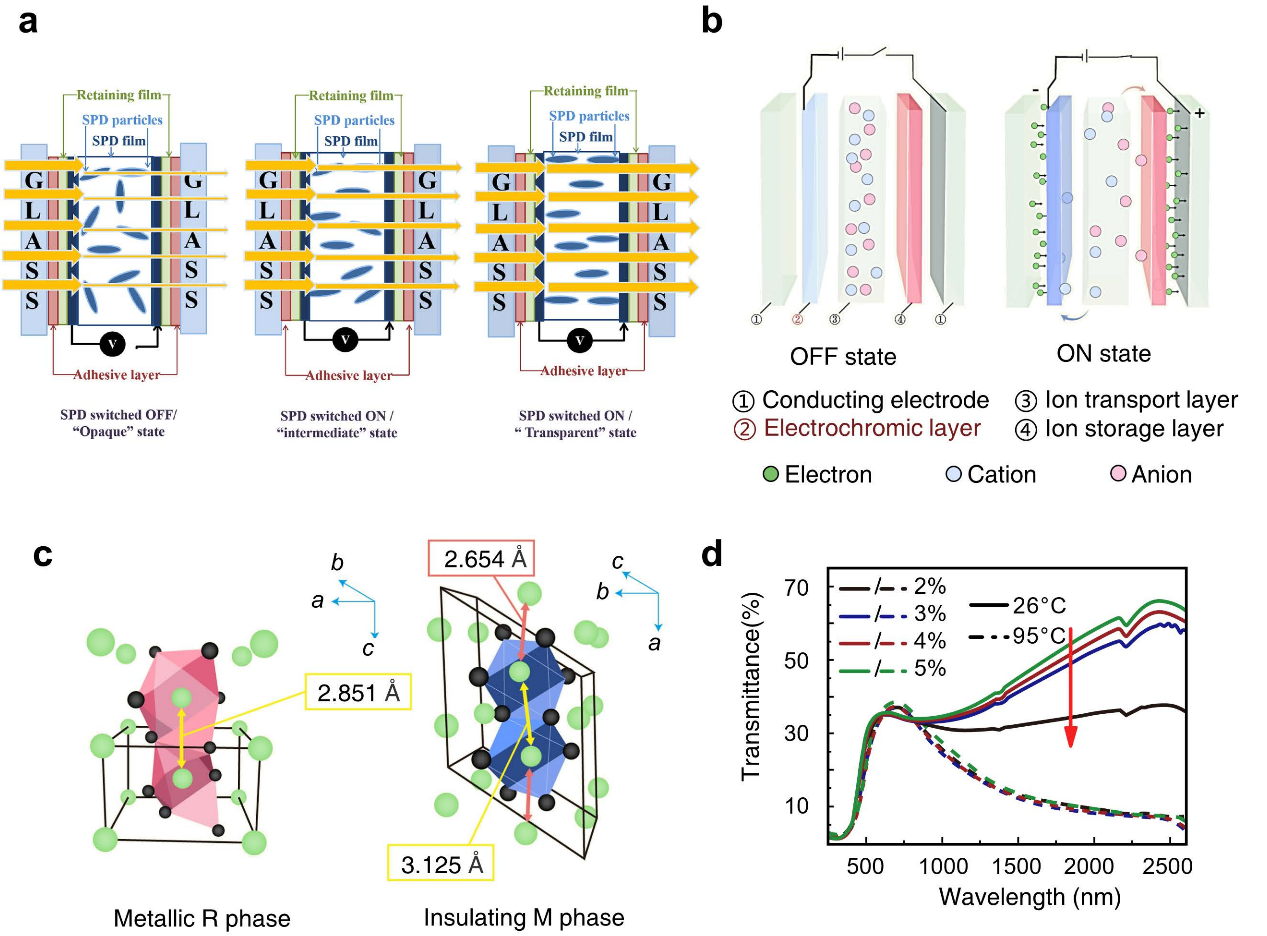
a
Retaining film
SPD particles
SPD film
G L A S S
V
Adhesive layer
SPD switched OFF/ "Opaque" state
SPD switched ON / "intermediate" state
SPD switched ON / " Transparent" state
b
OFF state
ON state
① Conducting electrode
② Electrochromic layer
③ Ion transport layer
④ Ion storage layer
Electron
Cation
Anion
c
2.851 Å
2.654 Å
3.125 Å
Metallic R phase
Insulating M phase
d
Transmittance(%)
Wavelength (nm)
2%
3%
4%
5%
26°C
95°C

ELSEVIER LICENSE TERMS AND CONDITIONS

Sep 19, 2022

This Agreement between Harbin Institute of Technology -- Ruicong Zhang ("You") and Elsevier ("Elsevier") consists of your license details and the terms and conditions provided by Elsevier and Copyright Clearance Center.

License Number	5392390103270
License date	Sep 19, 2022
Licensed Content Publisher	Elsevier
Licensed Content Publication	Renewable Energy
Licensed Content Title	Optimization of PV powered SPD switchable glazing to minimise probability of loss of power supply
Licensed Content Author	Aritra Ghosh,Brian Norton
Licensed Content Date	Feb 1, 2019
Licensed Content Volume	131
Licensed Content Issue	n/a
Licensed Content Pages	9
Start Page	993
End Page	1001
Type of Use	reuse in a journal/magazine
Requestor type	academic/educational institute
Portion	figures/tables/illustrations
Number of figures/tables/illustrations	1
Format	both print and electronic
Are you the author of this Elsevier article?	No
Will you be translating?	No
Title of new article	Advanced liquid crystal-based switchable optical devices for light protection applications: principles and strategies
Lead author	Ruicong Zhang, Zhibo Zhang, Jiecai Han, Lei Yang, Jiajun li, Zicheng Song Tianyu Wang, Jiaqi Zhu
Title of targeted journal	Light: Science & Applications
Publisher	Springer Nature
Expected publication date	Nov 2022
Portions	Fig 2
Requestor Location	Harbin Institute of Technology<br>No. 92, Xidazhi Street, Nangang District<br><br>Harbin, 150080<br>China<br>Attn: Harbin Institute of Technology
Publisher Tax ID	GB 494 6272 12
Total	**0.00 USD**
Terms and Conditions	

INTRODUCTION

1. The publisher for this copyrighted material is Elsevier. By clicking "accept" in connection with completing this licensing transaction, you agree that the following terms and conditions apply to this transaction (along with the Billing and Payment terms

and conditions established by Copyright Clearance Center, Inc. ("CCC"), at the time that you opened your Rightslink account and that are available at any time at http://myaccount.copyright.com).

GENERAL TERMS

2. Elsevier hereby grants you permission to reproduce the aforementioned material subject to the terms and conditions indicated.
3. Acknowledgement: If any part of the material to be used (for example, figures) has appeared in our publication with credit or acknowledgement to another source, permission must also be sought from that source. If such permission is not obtained then that material may not be included in your publication/copies. Suitable acknowledgement to the source must be made, either as a footnote or in a reference list at the end of your publication, as follows:
"Reprinted from Publication title, Vol /edition number, Author(s), Title of article / title of chapter, Pages No., Copyright (Year), with permission from Elsevier [OR APPLICABLE SOCIETY COPYRIGHT OWNER]." Also Lancet special credit - "Reprinted from The Lancet, Vol. number, Author(s), Title of article, Pages No., Copyright (Year), with permission from Elsevier."
4. Reproduction of this material is confined to the purpose and/or media for which permission is hereby given.
5. Altering/Modifying Material: Not Permitted. However figures and illustrations may be altered/adapted minimally to serve your work. Any other abbreviations, additions, deletions and/or any other alterations shall be made only with prior written authorization of Elsevier Ltd. (Please contact Elsevier's permissions helpdesk here). No modifications can be made to any Lancet figures/tables and they must be reproduced in full.
6. If the permission fee for the requested use of our material is waived in this instance, please be advised that your future requests for Elsevier materials may attract a fee.
7. Reservation of Rights: Publisher reserves all rights not specifically granted in the combination of (i) the license details provided by you and accepted in the course of this licensing transaction, (ii) these terms and conditions and (iii) CCC's Billing and Payment terms and conditions.
8. License Contingent Upon Payment: While you may exercise the rights licensed immediately upon issuance of the license at the end of the licensing process for the transaction, provided that you have disclosed complete and accurate details of your proposed use, no license is finally effective unless and until full payment is received from you (either by publisher or by CCC) as provided in CCC's Billing and Payment terms and conditions. If full payment is not received on a timely basis, then any license preliminarily granted shall be deemed automatically revoked and shall be void as if never granted. Further, in the event that you breach any of these terms and conditions or any of CCC's Billing and Payment terms and conditions, the license is automatically revoked and shall be void as if never granted. Use of materials as described in a revoked license, as well as any use of the materials beyond the scope of an unrevoked license, may constitute copyright infringement and publisher reserves the right to take any and all action to protect its copyright in the materials.
9. Warranties: Publisher makes no representations or warranties with respect to the licensed material.
10. Indemnity: You hereby indemnify and agree to hold harmless publisher and CCC, and their respective officers, directors, employees and agents, from and against any and all claims arising out of your use of the licensed material other than as specifically authorized pursuant to this license.
11. No Transfer of License: This license is personal to you and may not be sublicensed, assigned, or transferred by you to any other person without publisher's written permission.
12. No Amendment Except in Writing: This license may not be amended except in a writing signed by both parties (or, in the case of publisher, by CCC on publisher's behalf).
13. Objection to Contrary Terms: Publisher hereby objects to any terms contained in any purchase order, acknowledgment, check endorsement or other writing prepared by you, which terms are inconsistent with these terms and conditions or CCC's Billing and Payment terms and conditions. These terms and conditions, together with CCC's Billing and Payment terms and conditions (which are incorporated herein), comprise the entire agreement between you and publisher (and CCC) concerning this licensing transaction. In the event of any conflict between your obligations established by these terms and conditions and those established by CCC's Billing and Payment terms and conditions, these terms and conditions shall control.
14. Revocation: Elsevier or Copyright Clearance Center may deny the permissions described in this License at their sole discretion, for any reason or no reason, with a full refund payable to you. Notice of such denial will be made using the contact information provided by you. Failure to receive such notice will not alter or invalidate the denial. In no event will Elsevier or Copyright Clearance Center be responsible or liable for any costs, expenses or damage incurred by you as a result of a denial of your permission request, other than a refund of the amount(s) paid by you to Elsevier and/or Copyright Clearance Center for denied permissions.

LIMITED LICENSE

The following terms and conditions apply only to specific license types:
15. **Translation**: This permission is granted for non-exclusive world **English** rights only unless your license was granted for translation rights. If you licensed translation rights you may only translate this content into the languages you requested. A professional translator must perform all translations and reproduce the content word for word preserving the integrity of the article.
16. **Posting licensed content on any Website**: The following terms and conditions apply as follows: Licensing material from an Elsevier journal: All content posted to the web site must maintain the copyright information line on the bottom of each image; A hyper-text must be included to the Homepage of the journal from which you are licensing at http://www.sciencedirect.com/science/journal/xxxxx or the Elsevier homepage for books at http://www.elsevier.com; Central Storage: This license does not include permission for a scanned version of the material to be stored in a central repository such as that provided by Heron/XanEdu.
Licensing material from an Elsevier book: A hyper-text link must be included to the Elsevier homepage at http://www.elsevier.com . All content posted to the web site must maintain the copyright information line on the bottom of each image.

Posting licensed content on Electronic reserve: In addition to the above the following clauses are applicable: The web site must be password-protected and made available only to bona fide students registered on a relevant course. This permission is granted for 1 year only. You may obtain a new license for future website posting.
17. **For journal authors:** the following clauses are applicable in addition to the above:
Preprints:
A preprint is an author's own write-up of research results and analysis, it has not been peer-reviewed, nor has it had any other value added to it by a publisher (such as formatting, copyright, technical enhancement etc.).
Authors can share their preprints anywhere at any time. Preprints should not be added to or enhanced in any way in order to appear more like, or to substitute for, the final versions of articles however authors can update their preprints on arXiv or RePEc with their Accepted Author Manuscript (see below).
If accepted for publication, we encourage authors to link from the preprint to their formal publication via its DOI. Millions of researchers have access to the formal publications on ScienceDirect, and so links will help users to find, access, cite and use the best available version. Please note that Cell Press, The Lancet and some society-owned have different preprint policies. Information on these policies is available on the journal homepage.
Accepted Author Manuscripts: An accepted author manuscript is the manuscript of an article that has been accepted for publication and which typically includes author-incorporated changes suggested during submission, peer review and editor-author communications.
Authors can share their accepted author manuscript:

- immediately
  - via their non-commercial person homepage or blog
  - by updating a preprint in arXiv or RePEc with the accepted manuscript
  - via their research institute or institutional repository for internal institutional uses or as part of an invitation-only research collaboration work-group
  - directly by providing copies to their students or to research collaborators for their personal use
  - for private scholarly sharing as part of an invitation-only work group on commercial sites with which Elsevier has an agreement
- After the embargo period
  - via non-commercial hosting platforms such as their institutional repository
  - via commercial sites with which Elsevier has an agreement

In all cases accepted manuscripts should:

- link to the formal publication via its DOI
- bear a CC-BY-NC-ND license - this is easy to do
- if aggregated with other manuscripts, for example in a repository or other site, be shared in alignment with our hosting policy not be added to or enhanced in any way to appear more like, or to substitute for, the published journal article.

Published journal article (JPA): A published journal article (PJA) is the definitive final record of published research that appears or will appear in the journal and embodies all value-adding publishing activities including peer review co-ordination, copy-editing, formatting, (if relevant) pagination and online enrichment.
Policies for sharing publishing journal articles differ for subscription and gold open access articles:
Subscription Articles: If you are an author, please share a link to your article rather than the full-text. Millions of researchers have access to the formal publications on ScienceDirect, and so links will help your users to find, access, cite, and use the best available version.
Theses and dissertations which contain embedded PJAs as part of the formal submission can be posted publicly by the awarding institution with DOI links back to the formal publications on ScienceDirect.
If you are affiliated with a library that subscribes to ScienceDirect you have additional private sharing rights for others' research accessed under that agreement. This includes use for classroom teaching and internal training at the institution (including use in course packs and courseware programs), and inclusion of the article for grant funding purposes.
Gold Open Access Articles: May be shared according to the author-selected end-user license and should contain a CrossMark logo, the end user license, and a DOI link to the formal publication on ScienceDirect.
Please refer to Elsevier's posting policy for further information.
18. **For book authors** the following clauses are applicable in addition to the above: Authors are permitted to place a brief summary of their work online only. You are not allowed to download and post the published electronic version of your chapter, nor may you scan the printed edition to create an electronic version. **Posting to a repository:** Authors are permitted to post a summary of their chapter only in their institution's repository.
19. **Thesis/Dissertation**: If your license is for use in a thesis/dissertation your thesis may be submitted to your institution in either print or electronic form. Should your thesis be published commercially, please reapply for permission. These requirements include permission for the Library and Archives of Canada to supply single copies, on demand, of the complete thesis and include permission for Proquest/UMI to supply single copies, on demand, of the complete thesis. Should your thesis be published commercially, please reapply for permission. Theses and dissertations which contain embedded PJAs as part of the formal submission can be posted publicly by the awarding institution with DOI links back to the formal publications on ScienceDirect.

Elsevier Open Access Terms and Conditions

You can publish open access with Elsevier in hundreds of open access journals or in nearly 2000 established subscription journals that support open access publishing. Permitted third party re-use of these open access articles is defined by the author's choice of Creative Commons user license. See our open access license policy for more information.

Terms & Conditions applicable to all Open Access articles published with Elsevier:

Any reuse of the article must not represent the author as endorsing the adaptation of the article nor should the article be modified in such a way as to damage the author's honour or reputation. If any changes have been made, such changes must be clearly indicated.

The author(s) must be appropriately credited and we ask that you include the end user license and a DOI link to the formal publication on ScienceDirect.

If any part of the material to be used (for example, figures) has appeared in our publication with credit or acknowledgement to another source it is the responsibility of the user to ensure their reuse complies with the terms and conditions determined by the rights holder.

Additional Terms & Conditions applicable to each Creative Commons user license:

CC BY: The CC-BY license allows users to copy, to create extracts, abstracts and new works from the Article, to alter and revise the Article and to make commercial use of the Article (including reuse and/or resale of the Article by commercial entities), provided the user gives appropriate credit (with a link to the formal publication through the relevant DOI), provides a link to the license, indicates if changes were made and the licensor is not represented as endorsing the use made of the work. The full details of the license are available at http://creativecommons.org/licenses/by/4.0.

CC BY NC SA: The CC BY-NC-SA license allows users to copy, to create extracts, abstracts and new works from the Article, to alter and revise the Article, provided this is not done for commercial purposes, and that the user gives appropriate credit (with a link to the formal publication through the relevant DOI), provides a link to the license, indicates if changes were made and the licensor is not represented as endorsing the use made of the work. Further, any new works must be made available on the same conditions. The full details of the license are available at http://creativecommons.org/licenses/by-nc-sa/4.0.

CC BY NC ND: The CC BY-NC-ND license allows users to copy and distribute the Article, provided this is not done for commercial purposes and further does not permit distribution of the Article if it is changed or edited in any way, and provided the user gives appropriate credit (with a link to the formal publication through the relevant DOI), provides a link to the license, and that the licensor is not represented as endorsing the use made of the work. The full details of the license are available at http://creativecommons.org/licenses/by-nc-nd/4.0. Any commercial reuse of Open Access articles published with a CC BY NC SA or CC BY NC ND license requires permission from Elsevier and will be subject to a fee.

Commercial reuse includes:

- Associating advertising with the full text of the Article
- Charging fees for document delivery or access
- Article aggregation
- Systematic distribution via e-mail lists or share buttons

Posting or linking by commercial companies for use by customers of those companies.

20. **Other Conditions**:

v1.10

Questions? customercare@copyright.com or +1-855-239-3415 (toll free in the US) or +1-978-646-2777.

Renewable Energy 131 (2019) 993–1001

Contents lists available at ScienceDirect

Renewable Energy

journal homepage: www.elsevier.com/locate/renene

Optimization of PV powered SPD switchable glazing to minimise probability of loss of power supply

Aritra Ghosh [a, b, *], Brian Norton [a]

[a] Dublin Energy Lab, Dublin Institute of Technology, Dublin, Ireland
[b] Environmental and Sustainability Institute, University of Exeter, Penryn, Cornwall, UK

ARTICLE INFO

Article history:
Received 17 January 2018
Received in revised form
20 May 2018
Accepted 23 July 2018
Available online 26 July 2018

Keywords:
Suspended particle device (SPD)
Electrochromic (EC)
Loss of Power Supply Probability (LPSP)
Photovoltaic (PV)
Glazing
Model
Battery
Inverter

ABSTRACT

Suspended particle device (SPD) glazing is an electrically actuated switchable glazing. It requires alternate current (AC) power supply to switch from opaque to transparent state. To power this glazing using PV device requires inverter. Optimization of AC powered switchable SPD glazing using photovoltaic (PV) device has been evaluated using loss of power supply probability (LPSP). Electrically switchable direct current (DC) powered electrochromic glazing was also considered in this investigation as it doesn't need any inverter to couple with PV. It is concluded that behaviour of these glazings is the dominant factor in performance optimization outweighting than azimuthal orientation and inclination of PV.

© 2018 Elsevier Ltd. All rights reserved.

1. Introduction

Building consumes 40%–60% of total global energy due to heating load, cooling load and artificial lighting [1]. Significant heat loss and solar heat gain of a building can occur through windows. Smart switchable glazing has potential to reduce the incoming solar heat gain and control glare thereby permitting comfortable daylighting [2]. Electrically actuated smart switchable electrochromic (EC), liquid crystal (LC) and suspended particle device (SPD) glazings are attractive over non-electrically actuated thermochromic [3], thermotropic [4], gasochromic [5] and phase change material [6] glazings. Non-electrically actuated switchable glazings do not provide control of the glazing transmittance based on occupant choice. Electrically actuated electrochromic (EC) [7], liquid crystal (LC) [8] and suspended particle device (SPD) [9] glazing can be activated manually or can be switched contingent on internal conditions.

Alternating current (AC) powered electrically activated switchable LC glazing can be twisted nematic, ferroelectric, guest host, and polymer dispersed liquid crystal (PDLC) types [8,10]. PDLC types are best suitable for glazing as it does not require polarizer to operate [11]. LC glazing creates haze during its opaque state [12], which has limited the scope of applications of LC. However, haze free LC glazing is under investigation [13,14].

An EC glazing shown in Fig. 1 changes its state from "transparent" to "opaque" by a redox reaction in the presence of an applied direct current (DC) voltage typically from 0 to 5 V [15–17] reversible by inversion of electrical supply [18]. This colour change process requires less power at higher environment temperatures [19]. EC materials have potential to control visible [20–22] and near infra-red (NIR) solar radiation [23,24]. An EC glazing is opaque due to radiation absorption, rather than reflection. Thus an opaque EC glazing can be creating a heat source inside the building [25]. Five layer monolithic EC device [26], parallel double-layer-coated glass substrates joined by a polymer electrolyte EC [27], tungsten tri oxide (WO_3), prussian blue, polyvinyl butyral (PVB) electrolyte based laminated [18] and low cost polymer-foil-based laminated EC [7,28] are the major available EC glazing device.

SPD films are plastics containing suspended needle or rod shaped dihydrocinchonidine bisulfite polyiodide particles are suspended [29–34]. In the presence of AC power supply this particles

* Corresponding author. Dublin Energy Lab, Dublin Institute of Technology, Dublin, Ireland.
E-mail addresses: a.ghosh@exeter.ac.uk, aritraghosh_9@yahoo.co.in (A. Ghosh).

https://doi.org/10.1016/j.renene.2018.07.115
0960-1481/© 2018 Elsevier Ltd. All rights reserved.

Nomenclature

a_1	Ideality factor of 1st diode
a_2	Ideality factor of 2nd diode
C_{batt}	Battery capacity
DOD	Depth of discharge
E_b	Generated power from battery
E_L	Generated power from load
E_{pv}	Generated power from PV
I_v	Vertical plane global solar radiation (W/m^2)
I_{pv}	Photovoltaic current (A)
I_{01}	Diode saturation current of 1st diode (A)
I_{02}	Diode saturation current of 2nd diode (A)
I_{D1}	Diode current of 1st diode (A)
I_{D2}	Diode current of 2nd diode (A)
G	Variable input solar radiation (W/m^2)
G_n	Solar radiation at STC condition (W/m^2)
Isc	Short circuit current (A)
Im	Maximum current (A)
K	Stefan Boltzmann constant ($1.3806503 \times 10^{-23}$ J/K)
LPS	Loss of power supply
LPSP	Loss of power supply probability
N_{pv}	Number of PV
N_{batt}	Number of battery
N_{inv}	Number of inverter
SOC	State of charge
P_{invout}	Inverter output
$P_{inv,norm,out}$	Normalised inverter output
$P_{inv,rate}$	Inverter rated input
$P_{inv,norm,in}$	Inverter normalised input
P_{max}	Maximum power from photovoltaic
P_{pv}	Inverter input from PV
R_{ctg}	Resistance of charge transfer insertion reaction
R_{eg}	Resistive effect of electrical contact
R_s	Series Resistance (Ω)
R_p	Parallel or shunt resistance (Ω)
Voc	Open circuit voltage (V)
Vm	Maximum voltage (V)
V_T	Thermal voltage (V)
ν_0	Normalised self-consumption loss
ν_1	Linear efficiency coefficient
ν_2	Coefficient for losses proportional to input power squared
w_0	Warburg Impedance
X_j	Theoretical values
Y_j	Experimental values

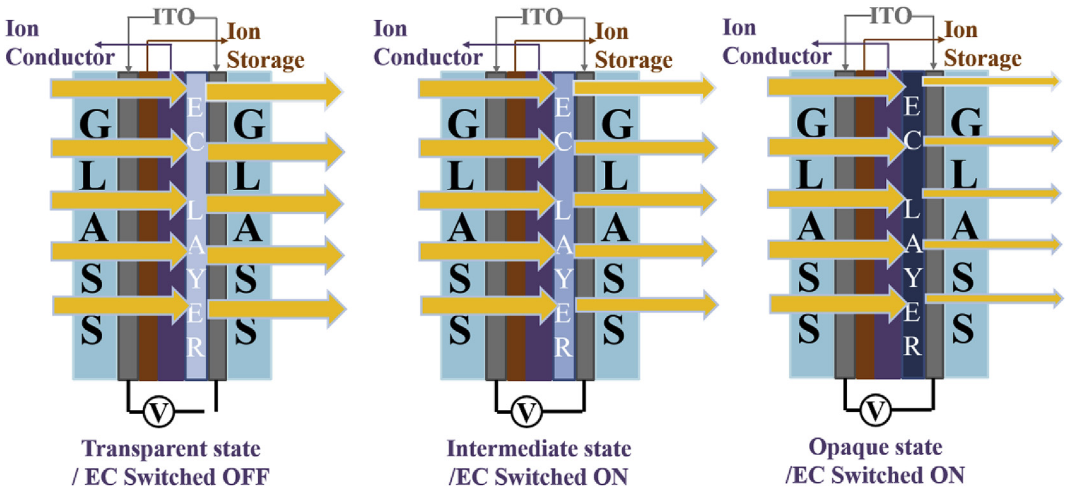

Fig. 1. Electrochromic (EC) glazing showing transparent/bleached, intermediate and opaque/coloured state.

are orientated perpendicular to the substrate and allow light passing through it [35] as shown in Fig. 2. When unpowered, SPD particles oriented randomly due to Brownian motion and absorbed or reflect lights. The SPD glazing sample was considered in this work changes transmission from 5% to 55% in the presence of 110 V, 0.07 W AC power supply [35]. Outdoor test cell characterisation of SPD glazing suggest that this glazing can be incorporated with double [36] or vacuum glazing [37,38] in a low heat loss window with variable solar heat gain [39,40]. SPD glazing with 30% transmission offers glare control throughout a day for clear sunny day with a useful daylight index [41].

Using integrated PV to power switchable windows gives a self-contained autonomous unit installed [35] [42–44] without connection to an electrical power supply nor the engagement of specialized electrical installer. Both these factors reduce the installation and operating costs. PV powered SPD glazing requires an inverter for transformation of DC PV power. Proper sizing of inverter is an essential criterion for PV powered SPD glazing [35].

Battery energy storage [45] is part of a PV-powered glazing system. The energy required for switching is the solar energy accumulated prior to switching rather than merely the instantaneously available solar energy. The PV area required is thus smaller than would be the soil no battery storage was in place.

In this work, optimization between PV, battery and AC powered SPD glazing and DC powered EC glazing were carried out using loss of power supply probability (LPSP) methods. To date no studies have been reported on PV powered switchable glazing optimization using LPSP or any other optimization methods.

2. Methodology

2.1. PV modelling

One-diode [46,47] and two-diode models [48,49] have been

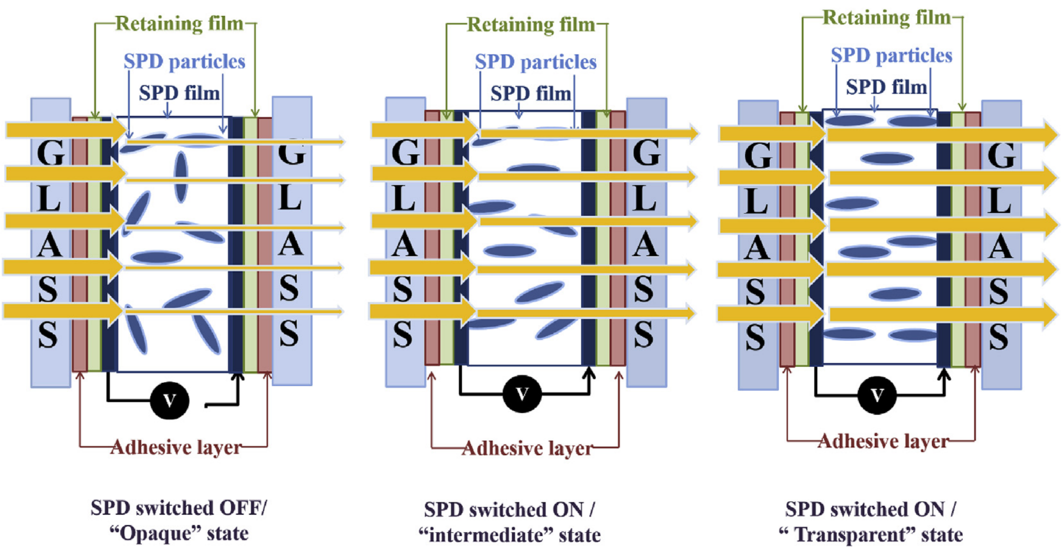

Fig. 2. Suspended particle device (SPD) glazing showing "transparent", intermediate and "opaque" state.

used to evaluate the maximum power output from a PV cell. A two-diode model in which the extra diode represents recombination carriers is used in this work giving the equivalent circuit shown in Fig. 3. The total current is given by;

$$I = I_{pv} - I_{d1} - I_{d2} - I_p \tag{1}$$

$$I = I_{pv} - I_{01}\left[\exp\left(\frac{V + IR_s}{a_1 V_{T1}}\right) - 1\right] - I_{02}\left[\exp\left(\frac{V + IR_s}{a_2 V_{T2}}\right)\right] - \left(\frac{V + IR_s}{R_p}\right) \tag{2}$$

Where thermal voltage is given by

$$V_T = \frac{N_s KT}{q}. \tag{3}$$

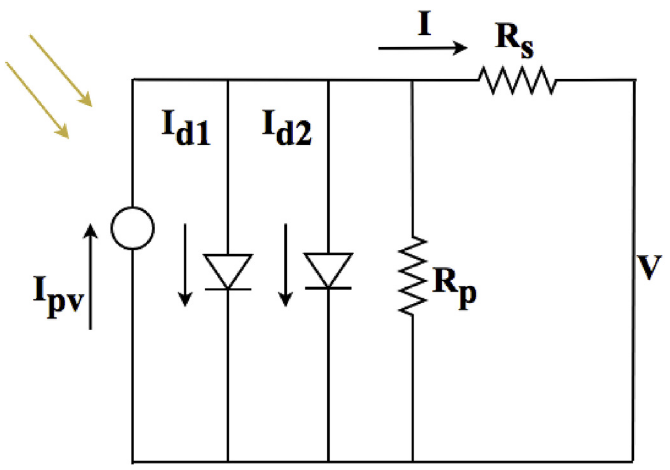

Fig. 3. Two-diode model of PV cell.

$$I_{pv} = (I_{scn} + K_i)\frac{G}{G_n} \tag{4}$$

$$I_0 = \frac{(I_{scn} + k_i)\Delta T}{\exp\left[\frac{V_{ocn} + k_v \Delta T}{a V_T}\right] - 1} \tag{5}$$

The power output from PV device is given by

$$P_{pv} = VI \tag{6}$$

Energy generated from PV device during time interval t to T (where t = day 1 and T = day 365) is given by

$$E_{pv} = \int_t^T P_{pv} dt. \tag{7}$$

2.2. Inverter output modelling

Inverter efficiency varies as a function of an inverter input power [50–52].

$$P_{invout} = P_{inv,norm,out} P_{inv,rate} \tag{8}$$

$$P_{inv,norm,out} = v_0 + v_1 P_{inv,norm,in} + v_2 P_{inv,norm,in}^2 \tag{9}$$

$$P_{inv,norm,in} = \frac{P_{pv}}{P_{inv,rate}} \tag{10}$$

The instantaneous inverter efficiency is given by

$$\eta_{inv} = \frac{P_{inv,norm,out}}{P_{inv,norm,in}} \tag{11}$$

Obtained energy after inverter during time interval t to T (where t = day 1 and T = day 365) is given by

$$E_{inv} = \int_t^T E_{pv}\eta_{inv}dt. \tag{12}$$

2.3. Battery model

Charging a battery using PV source system is neither a constant current source nor a constant voltage source [53,54]. Thus, a nonlinear battery storage model is used [55,56] where;

$$V_b = \varepsilon_0 + (a \times SOC) + (R_{\text{int}} \times I_{bat}) \tag{13}$$

Here V_b is the battery voltage for one element ε_0 is the battery equilibrium voltage, SOC is the state of charge of the battery, the parameter relates the voltage with the state of charge. For accurate calculation, knowledge of the SOC of a battery is necessary [57]. SOC_0 is the battery SOC of the starting point; t0 and t are the time of the starting point and the time of interest, respectively, C_{batt} is the battery capacity, I_{bat} is the battery current. Eq. (14) represents the calculation of battery SOC for ideal batteries.

$$SOC = SOC_0 + \int_{t_0}^{t} \frac{I_{bat}}{C_{batt}} d\tau \tag{14}$$

Energy stored in a battery during time interval t to T (where t = day 1 and T = day 365) is given by

$$E_b = \int_t^T SOC \cdot C_{batt} dt \tag{15}$$

2.4. Glazing model

Fig. 4 shows the complete circuit diagram of PV powered SPD and EC glazing. SPD [34] and EC [58] both glazing can be represented by using Randles circuit. This circuit contains the resistance for the charge transfer insertion (R_{ctg}), double-layer capacitor (C_{dlg}), resistance for electrical contacts (R_{eg}), and Warburg impedance (w_0) to consider diffusion of charges. In this circuit, an AC powered SPD glazing is considered as an AC load whereas DC powered EC is considered as DC load. Generated power from PV-inverter system supply power to the glazing. Excess power charges the battery. During no sunshine period, glazing is powered from battery.

$$P_{glazing} = \left(\frac{v_0 + v_1 P_{inv,norm,in} + v_2 P^2_{inv,norm,in}}{P_{pv}/P_{inv,rate}}\right) \times \left[V\left\{I_{pv} - I_{01}\left(\exp\left(\frac{V+IR_s}{a_1 V_{T1}}\right) - 1\right) - I_{02}\left(\exp\left(\frac{V+IR_s}{a_2 V_{T2}}\right) - 1\right) - \left(\frac{V+IR_s}{R_p}\right)\right\}\right] \tag{16}$$

Required energy demand for glazing for time interval t to T (where t = day 1 and T = 365) is given by

$$E_L = \int_t^T P_{glazing} dt \tag{17}$$

2.5. LPSP model

For a reliable hybrid energy systems the loss of power supply probability (LPSP) is defined by a number between 0 and 1 [59]. An LPSP of 1 indicates that the load will never be satisfied and an LPSP of 0 indicates that the load will be always satisfied. In this work, load is the glazing demand which is essential to remain the SPD glazing fully transparent state and EC glazing fully opaque state. For a specified elapsed period, T (one year in this study), LPSP is defined by

$$LPSP(t) = \frac{\sum_{t=1}^{T} LPS(t)}{\sum_{t=1}^{T} E_L(t)} \tag{18}$$

Where loss of power supply (LPS) can be found from equation (19). Generated energy from PV (E_{pv}) less than the load energy demand produces LPS. LPSP was calculated as shown in the flow diagram in Fig. 5.

$$LPS(t) = E_{load}(t) - \{E_{pv}(t) + E_b(t-1) - (SOC)C_{batt}N_{batt})\}\eta_{inv} \tag{19}$$

Excess power generated from PV after meeting the demand of SPD glazing, charges the battery. This battery will supply power in the night time to keep SPD fully transparent. The battery in the

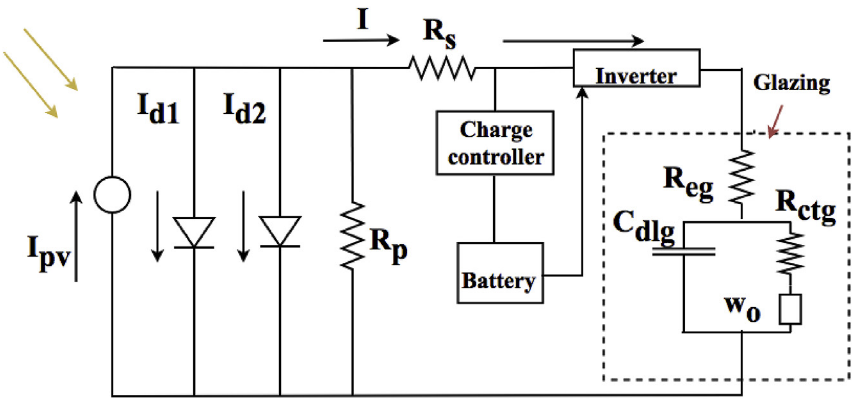

Fig. 4. Electrical circuit connection PV, charge controller, inverter, battery, glazing (glazing circuit represents both SPD and EC glazing).

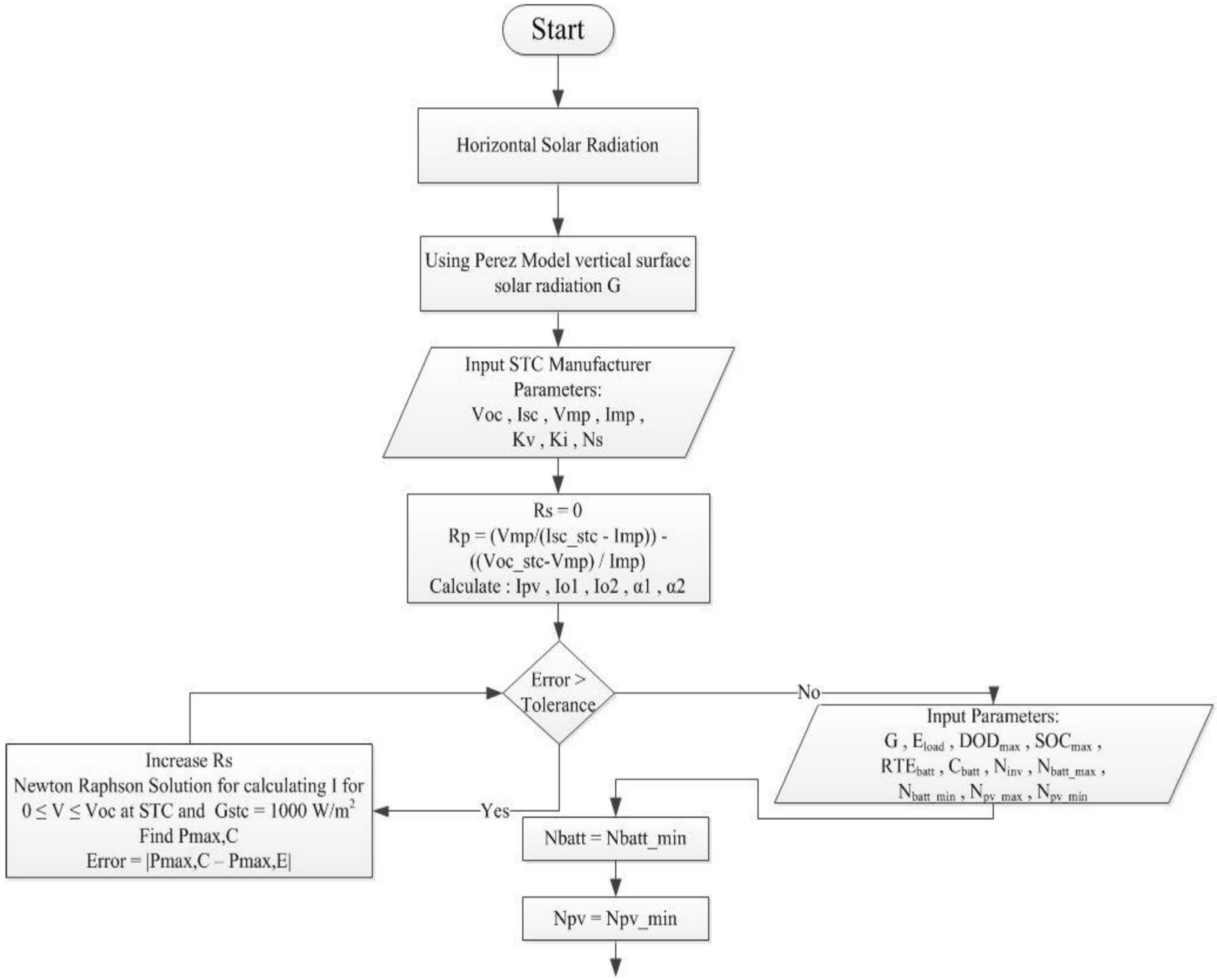

Fig. 5. Flow diagram for LPSP method.

system stores energy from PV during sun shine period and supply the energy to the SPD for night or overcast rainy condition. The PV module, inverter, battery and glazing specifications used are listed in Table 1, [35]. For this work, the glazing is assumed to have an active area of 1 m^2 with a 0.5 W power consumption [35] and was kept switched on for 24 h and 365 days. Lead acid battery was selected for this work due to its high energy efficiency, low self-discharge rate, and low up-front cost [45].

3. Simulation validation

The simulation model was compared with experimental measurements by Ref. [35]. Experimental work of PV powered SPD glazing was carried out in Dublin, Ireland (53.34°N, 6.25°W) from 1st of May to 1st July 2014. Fig. 6 (a) shows that the PV double diode PV model predicted power output closely match to experimental measurements. Fig. 6 (b) shows the simulated inverter outputs also closely agreed with experiment data. For an SPD glazing powered by PV evaluated experimentally by Ref. [35] as shown in Fig. 6c, available output from inverter was 8.9 kWh for a clear sunny day, without battery storage 8.42 kWh power was unused and inverter losses were 53%. Deviation of experimental and theoretical results were evaluated using root mean square percent deviation. Following expression was used to evaluate percentage deviation. Deviation for PV power generation and SPD power consumption (Figure c) was only 0.95%.

$$e = \sqrt{\frac{\sum_{t=1}^{n}\left(\frac{X_j - Y_j}{X_j}\right)^2}{n}} \tag{20}$$

4. Results & discussions

To obtain optimise low LPSP simulation work had been done varying battery capacity and PV power. Three different types of inverters were chosen. A dynamic type where output varies with solar radiation, a static 0.9 efficiency inverter for any solar radiation and a type where inverter efficiency was 1. Last type can be considered for EC glazing (as shown in Fig. 9) as EC glazing obviates need of any inverter.

Fig. 7 illustrates effect of different PV and battery combination on LPSP. Required LPSP varied from 0.6 to 0.74, due to the poor inverter performance. Higher PV rating matches closer to the inverter rating which creates less power loss and thus LPSP is low compared to lower rated PV.

Fig. 8 shows the PV and battery combinations with an inverter with a constant 90% efficiency. A constant efficiency inverter always provided higher output, which met the SPD glazing power requirement demand with less LPSP.

In Fig. 9 illustrates the LPSP for a 100% efficient inverter which

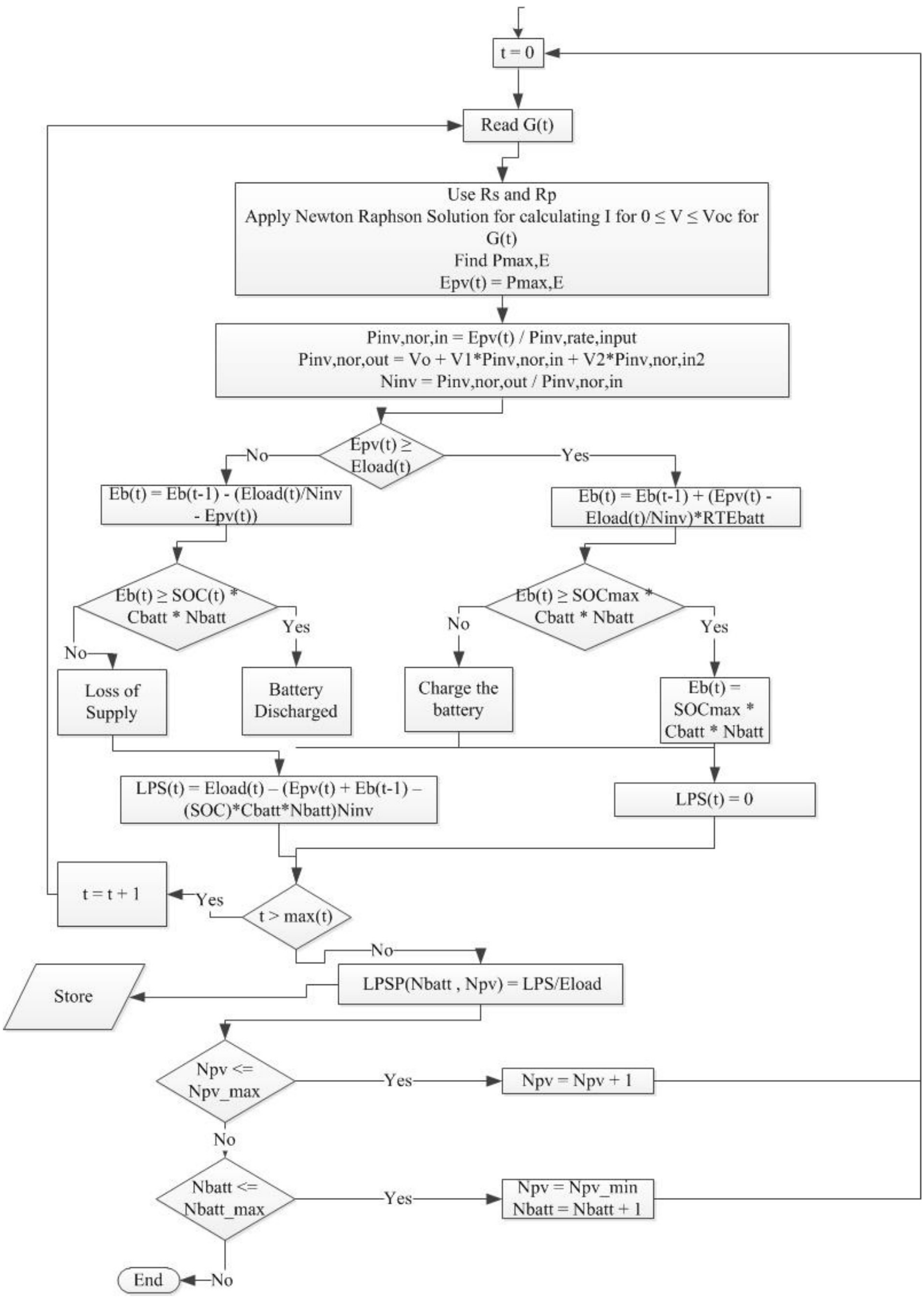

Fig. 5. (*continued*).

Table 1
Specifications of system components.

Polycrystalline PV module	Maximum Power (P_m)	40 W
	Open circuit voltage (V_{oc})	21.6 V
	Short circuit current (I_{sc})	2.51A
	Maximum voltage (V_m)	17.8 V
	Maximum current (I_m)	2.25A
	Efficiency (η)	16–16.5%
	Area (A)	0.52 m × 0.66 m (0.3432 m^2)
	Cost	550 euro/0.3434 m^2
Inverter	Max input voltage	12 V
	Max input current	16 A
	Rated output voltage	230 V
	Max output current	0.89A
	Cost	150 euro
Lead acid battery	Battery voltage	12 V
	Battery capacity	12 AH
	cost	35 euro
SPD glazing	Power requirement	0.07 W, 100 V (transparent) 0 V (opaque)
	Active area	0.0345 m^2

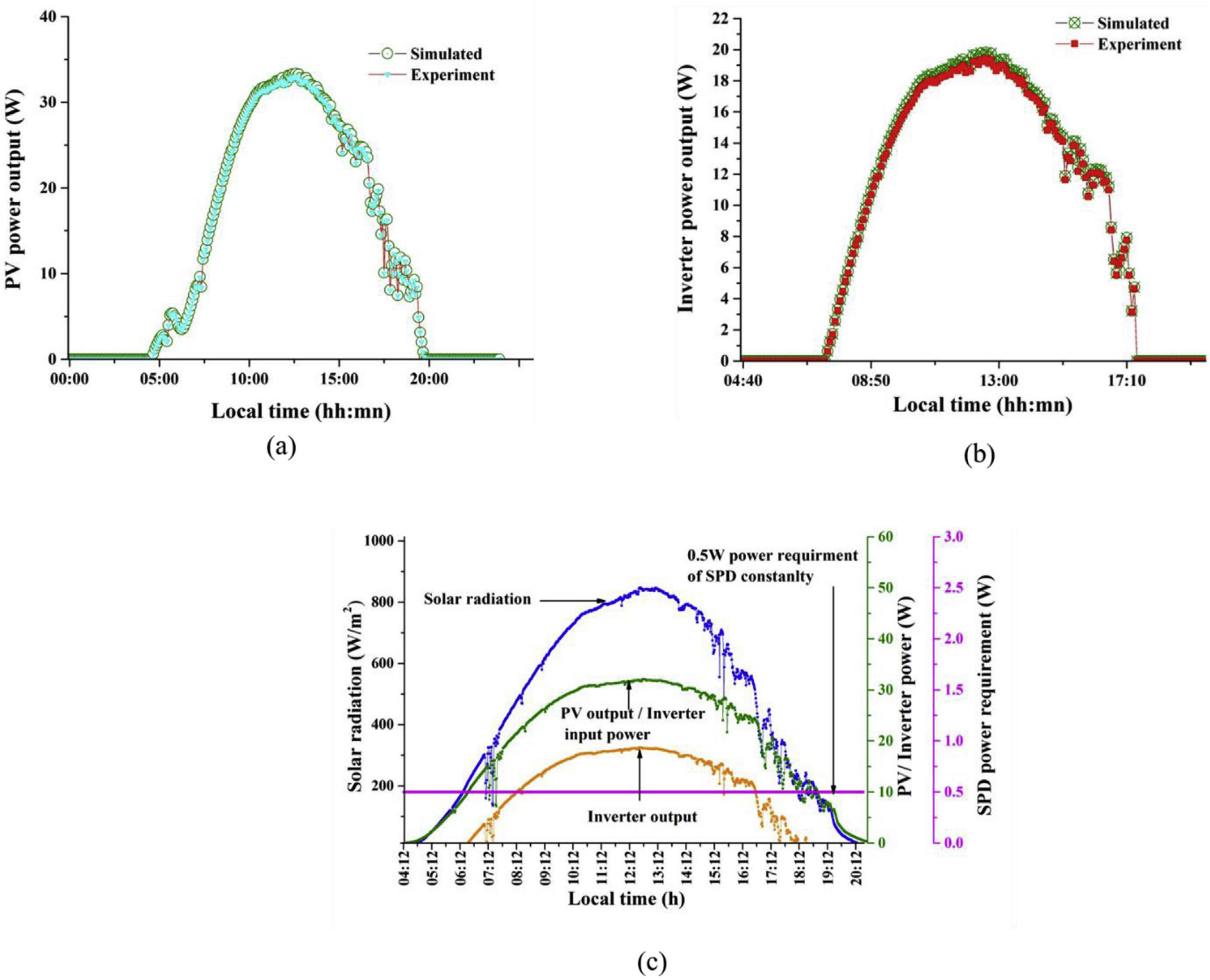

Fig. 6. (a) Comparison of PV output power from simulation and experiment, (b) Comparison of inverter output from simulation and experiment (c) diurnal performance of 40W_p PV powered 0.5 W SPD glazing.

provides all PV power output directly to the SPD glazing with no losses. As SPD works with only AC and a 100% inverter efficiency is not possible in reality, this case can be considered as equivalent to a DC powered EC glazing. For an EC glazing powered by 4 W PV with only 12AH battery had the highest LPSP at 0.05.

To obtain transparent state SPD glazing needs high voltage, low AC power. Whilst, EC glazing needs low voltage, low DC power to obtain opaque state. It is evident from this work that inverter requirement for PV-powered SPD glazing causes higher LPSP and enhances the number of battery storage. As SPD needs higher

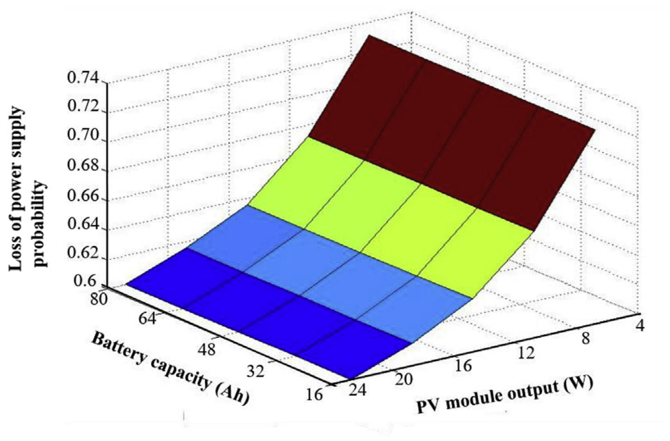

Fig. 7. LPSP for different PV module output and different battery capacity with variable inverter efficiency.

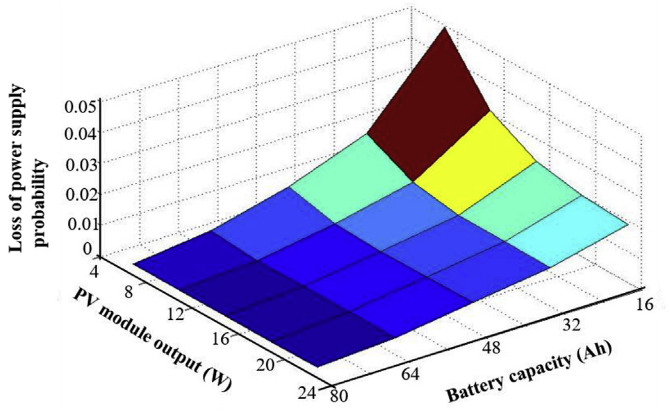

Fig. 8. LPSP for different PV outputs and different battery capacity with a constant inverter efficiency of 90%.

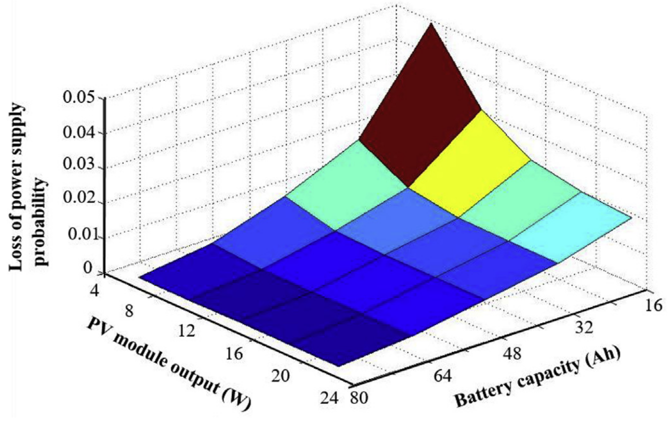

Fig. 9. LPSP for different PV module output and different battery capacity with constant inverter efficiency of 100% or a DC glazing.

voltage to modulate its transparency, side-by-side PV-SPD structure is promising while PV area should be large enough to generate this high voltage. Tandem structure PV -SPD is only possible if low driving voltage SPD is available. Tandem PV-EC structure is suitable due to its low voltage and no power inversion requirement.

5. Conclusions

Energy saving potential, control over switchable state and simpler installation make switchable glazing a potential choice for retrofit and new zero energy building. However, direct powering for these type of glazing from grid add to building's energy cost [35] [60]. Thus, PV powered electrically activated glazing is promising as they can transform an ordinary building to smart or zero energy building [2]. Using LPSP method optimization of PV powered SPD was conducted. Experimentally it was reported before that SPD glazing needs AC power to actuate which force to use inverter. Addition of inverter creates losses, which can be reduced using similar sizing area PV and inverter. In this work, PV powered SPD throughout the day and night and battery system was there to store excess energy from PV. Though EC needs power to obtain opaque state, no inverter requirement consume less PV power (thus less PV area required). Low power rated power electronics (low power rated inverter) are highly recommended for this PV-SPD integration. To enable low LPSP from PV powered SPD, investigations are required on low driving voltage SPD.

Switchable EC and SPD are primarily required to control the entering solar heat gain inside a room. SPD and EC both have potential to offer controllable intermediate states. EC becomes opaque in the presence of power supply where as SPD becomes opaque without power supply. Thus during the daytime, to control solar gain, EC can use PV power directly whereas power can be stored for SPD. Although, power consumption and changes of transparency for both glazing depend on occupants demand and behaviour, based on glazings operating performance it is clear that battery storage is essential for both glazing and SPD needs higher number of battery storage.

Acknowledgements

The work described in this paper was supported by the Graduate Research Education Programme of the Higher Education Authority, Ireland.

References

[1] F. Favoino, M. Overend, Q. Jin, The optimal thermo-optical properties and energy saving potential of adaptive glazing technologies, Appl. Energy 156 (2015) 1–15, https://doi.org/10.1016/j.apenergy.2015.05.065.
[2] A. Ghosh, B. Norton, Advances in switchable and highly insulating autonomous (self-powered) glazing systems for adaptive low energy buildings, Renew. Energy 126 (2018) 1003–1031, https://doi.org/10.1016/j.renene.2018.04.038.
[3] M.M. Seyfouri, R. Binions, Sol-gel approaches to thermochromic vanadium dioxide coating for smart glazing application, Sol. Energy Mater. Sol. Cells 159 (2017) 52–65, https://doi.org/10.1016/j.solmat.2016.08.035.
[4] A.C. Gladen, J.H. Davidson, S.C. Mantell, Selection of thermotropic materials for overheat protection of polymer absorbers, Sol. Energy 104 (2014) 42–51, https://doi.org/10.1016/j.solener.2013.10.026.
[5] W. Feng, L. Zou, G. Gao, G. Wu, J. Shen, W. Li, Gasochromic smart window: optical and thermal properties, energy simulation and feasibility analysis, Sol. Energy Mater. Sol. Cells 144 (2016) 316–323, https://doi.org/10.1016/j.solmat.2015.09.029.
[6] C. Liu, Y. Wu, D. Li, Y. Zhou, Z. Wang, X. Liu, Effect of PCM thickness and melting temperature on thermal performance of double glazing units, J. Build. Eng. 11 (2017) 87–95, https://doi.org/10.1016/j.jobe.2017.04.005.
[7] C.G. Granqvist, I. Bayrak Pehlivan, G.A. Niklasson, Electrochromics on a roll: web-coating and lamination for smart windows, Surf. Coating. Technol. (2017) 6–11, https://doi.org/10.1016/j.surfcoat.2017.08.006.
[8] A. Ghosh, B. Norton, T.K. Mallick, Solar Energy Materials and Solar Cells Daylight characteristics of a polymer dispersed liquid crystal switchable glazing, Sol. Energy Mater. Sol. Cells 174 (2018) 572–576, https://doi.org/10.1016/j.solmat.2017.09.047.
[9] A. Ghosh, B. Norton, A. Duffy, Behaviour of a SPD switchable glazing in an outdoor test cell with heat removal under varying weather conditions, Appl. Energy 180 (2016) 695–706, https://doi.org/10.1016/j.apenergy.2016.08.029.
[10] A. Ghosh, T.K. Mallick, Evaluation of colour properties due to switching behaviour of a PDLC glazing for adaptive building integration, Renew. Energy

120 (2018) 126–133, https://doi.org/10.1016/j.renene.2017.12.094.
[11] G. Macrelli, Optical characterization of commercial large area liquid crystal devices, Sol. Energy Mater. Sol. Cells 39 (1995) 123–131, https://doi.org/10.1016/0927-0248(95)00044-5.
[12] A. Ghosh, T.K. Mallick, Evaluation of optical properties and protection factors of a PDLC switchable glazing for low energy building integration, Sol. Energy Mater. Sol. Cells (2017), https://doi.org/10.1016/j.solmat.2017.10.026, 0–1.
[13] D. Jung, W. Choi, J.-Y. Park, K.B. Kim, N. Lee, Y. Seo, H.S. Kim, N.K. Kong, Inorganic gel and liquid crystal based smart window using silica sol-gel process, Sol. Energy Mater. Sol. Cells 159 (2017) 488–495, https://doi.org/10.1016/j.solmat.2016.10.001.
[14] S. Park, J.W. Hong, Polymer dispersed liquid crystal film for variable-transparency glazing, Thin Solid Films 517 (2009) 3183–3186, https://doi.org/10.1016/j.tsf.2008.11.115.
[15] C.M. Lampert, Electrochromic materials and devices for energy efficient windows, Sol. Energy Mater. 11 (1984) 1–27, https://doi.org/10.1016/0165-1633(84)90024-8.
[16] C.G. Granqvist, Electrochromic devices, J. Eur. Ceram. Soc. 25 (2005) 2907–2912, https://doi.org/10.1016/j.jeurceramsoc.2005.03.162.
[17] C.G. Granqvist, P.C. Lansåker, N.R. Mlyuka, G.A. Niklasson, E. Avendaño, Progress in chromogenics: new results for electrochromic and thermochromic materials and devices, Sol. Energy Mater. Sol. Cells 93 (2009) 2032–2039, https://doi.org/10.1016/j.solmat.2009.02.026.
[18] A. Kraft, M. Rottmann, Properties, performance and current status of the laminated electrochromic glass of Gesimat, Sol. Energy Mater. Sol. Cells 93 (2009) 2088–2092, https://doi.org/10.1016/j.solmat.2009.05.010.
[19] A. Ghosh, B. Norton, A. Duffy, Calculation of colouration voltage for a multi-functional glazing powered by photovoltaic, Conf. Pap. (2013) 3434–3436. http://arrow.dit.ie/engschcivcon/66.
[20] A. Piccolo, A. Pennisi, F. Simone, Daylighting performance of an electrochromic window in a small scale test-cell, Sol. Energy 83 (2009) 832–844, https://doi.org/10.1016/j.solener.2008.11.013.
[21] A. Piccolo, F. Simone, Effect of switchable glazing on discomfort glare from windows, Build. Environ. 44 (2009) 1171–1180, https://doi.org/10.1016/j.buildenv.2008.08.013.
[22] A. Piccolo, F. Simone, Performance Requirements for Electrochromic Smart Window, Elsevier, 2015, https://doi.org/10.1016/j.jobe.2015.07.002.
[23] A.P. Schuster, D. N.G.U.Y.E. N, O. Caporaletti, Solid state E L E C T R O C H R O M I C, Infrared Switchable Windows 13 (1986) 153–160.
[24] C. Lampert, Smart switchable glazing for solar energy and daylight control, Sol. Energy Mater. Sol. Cells 52 (1998) 207–221, https://doi.org/10.1016/S0927-0248(97)00279-1.
[25] Y. Fang, P.C. Eames, Thermal performance of an electrochromic vacuum glazing, Energy Convers. Manag. 47 (2006) 3602–3610, https://doi.org/10.1016/j.enconman.2006.03.016.
[26] N.L. Sbar, L. Podbelski, H.M. Yang, B. Pease, Electrochromic dynamic windows for office buildings, Int. J. Sustain. Built Environ. 1 (2012) 125–139, https://doi.org/10.1016/j.ijsbe.2012.09.001.
[27] M. Zinzi, Office worker preferences of electrochromic windows: a pilot study, Build. Environ. 41 (2006) 1262–1273, https://doi.org/10.1016/j.buildenv.2005.05.010.
[28] A. Azens, E. Avendaño, J. Backholm, L. Berggren, G. Gustavsson, R. Karmhag, G.A. Niklasson, A. Roos, G.G. Granqvist, Flexible foils with electrochromic coatings: science, technology and applications, Mater. Sci. Eng. B Solid-State Mater. Adv. Technol. 119 (2005) 214–223, https://doi.org/10.1016/j.mseb.2004.12.085.
[29] A. Ghosh, B. Norton, A. Duffy, Effect of sky conditions on light transmission through a suspended particle device switchable glazing, Sol. Energy Mater. Sol. Cells 160 (2017) 134–140, https://doi.org/10.1016/j.solmat.2016.09.049.
[30] A. Ghosh, B. Norton, Durability of switching behaviour after outdoor exposure for a suspended particle device switchable glazing, Sol. Energy Mater. Sol. Cells 163 (2017) 178–184, https://doi.org/10.1016/j.solmat.2017.01.036.
[31] A. Ghosh, B. Norton, Interior colour rendering of daylight transmitted through a suspended particle device switchable glazing, Sol. Energy Mater. Sol. Cells 163 (2017) 218–223, https://doi.org/10.1016/j.solmat.2017.01.041.
[32] D. Barrios, R. Vergaz, J.M. Sánchez-Pena, B. García-Cámara, C.G. Granqvist, G.A. Niklasson, Simulation of the thickness dependence of the optical properties of suspended particle devices, Sol. Energy Mater. Sol. Cells 143 (2015) 613–622, https://doi.org/10.1016/j.solmat.2015.05.044.
[33] D. Barrios, R. Vergaz, J.M. Sanchez-Pena, C.G. Granqvist, G.A. Niklasson, Toward a quantitative model for suspended particle devices: optical scattering and absorption coefficients, Sol. Energy Mater. Sol. Cells 111 (2013) 115–122, https://doi.org/10.1016/j.solmat.2012.12.012.
[34] R. Vergaz, J.M. Sánchez-Pena, D. Barrios, C. Vázquez, P. Contreras-Lallana, Modelling and electro-optical testing of suspended particle devices, Sol. Energy Mater. Sol. Cells 92 (2008) 1483–1487, https://doi.org/10.1016/j.solmat.2008.06.018.
[35] A. Ghosh, B. Norton, A. Duffy, First outdoor characterisation of a PV powered suspended particle device switchable glazing, Sol. Energy Mater. Sol. Cells 157 (2016) 1–9, https://doi.org/10.1016/j.solmat.2016.05.013.
[36] A. Ghosh, B. Norton, A. Duffy, Measured overall heat transfer coefficient of a suspended particle device switchable glazing, Appl. Energy 159 (2015) 362–369, https://doi.org/10.1016/j.apenergy.2015.09.019.
[37] A. Ghosh, B. Norton, A. Duffy, Effect of sky clearness index on transmission of evacuated (vacuum) glazing, Renew. Energy 105 (2017) 160–166, https://doi.org/10.1016/j.renene.2016.12.056.
[38] A. Ghosh, B. Norton, A. Duffy, Measured thermal & daylight performance of an evacuated glazing using an outdoor test cell, Appl. Energy 177 (2016) 196–203, https://doi.org/10.1016/j.apenergy.2016.05.118.
[39] A. Ghosh, B. Norton, A. Duffy, Measured thermal performance of a combined suspended particle switchable device evacuated glazing, Appl. Energy 169 (2016) 469–480, https://doi.org/10.1016/j.apenergy.2016.02.031.
[40] A. Ghosh, B. Norton, A. Duffy, Effect of atmospheric transmittance on performance of adaptive SPD-vacuum switchable glazing, Sol. Energy Mater. Sol. Cells 161 (2017) 424–431, https://doi.org/10.1016/j.solmat.2016.12.022.
[41] A. Ghosh, B. Norton, A. Duffy, Daylighting performance and glare calculation of a suspended particle device switchable glazing, Sol. Energy 132 (2016) 114–128, https://doi.org/10.1016/j.solener.2016.02.051.
[42] S.K. Deb, A novel electrophotographic system, Appl. Optic. 8 (1969) 192–195.
[43] A. Cannavale, G.E. Eperon, P. Cossari, A. Abate, H.J. Snaith, G. Gigli, Perovskite photovoltachromic cells for building integration, Energy Environ. Sci. 8 (2015) 1578–1584, https://doi.org/10.1039/C5EE00896D.
[44] L.M. Huang, C.W. Hu, H.C. Liu, C.Y. Hsu, C.H. Chen, K.C. Ho, Photovoltaic electrochromic device for solar cell module and self-powered smart glass applications, Sol. Energy Mater. Sol. Cells 99 (2012) 154–159, https://doi.org/10.1016/j.solmat.2011.03.036.
[45] X. Hu, C. Zou, C. Zhang, Y. Li, Technological developments in batteries: a survey of principal roles, types, and management needs, IEEE Power Energy Mag. 15 (2017) 20–31, https://doi.org/10.1109/MPE.2017.2708812.
[46] W. De Soto, S.A. Klein, W.A. Beckman, Improvement and validation of a model for photovoltaic array performance, Sol. Energy 80 (2006) 78–88, https://doi.org/10.1016/j.solener.2005.06.010.
[47] T.O. Saetre, O.M. Midtgård, G.H. Yordanov, A new analytical solar cell I-V curve model, Renew. Energy 36 (2011) 2171–2176, https://doi.org/10.1016/j.renene.2011.01.012.
[48] S.M. Hassan Hosseini, A.A. Keymanesh, Design and construction of photovoltaic simulator based on dual-diode model, Sol. Energy 137 (2016) 594–607, https://doi.org/10.1016/j.solener.2016.09.001.
[49] A. Zegaoui, P. Petit, M. Aillerie, J.P. Sawicki, A.W. Belarbi, M.D. Krachai, J.P. Charles, Photovoltaic cell/panel/array characterizations and modeling considering both reverse and direct modes, Energy Procedia 6 (2011) 695–703, https://doi.org/10.1016/j.egypro.2011.05.079.
[50] K. Peippo, P.D. Lund, Optimal sizing of solar array and inverter in grid-connected photovoltaic systems, Sol. Energy Mater. Sol. Cells 32 (1994) 95–114, https://doi.org/10.1016/0927-0248(94)90259-3.
[51] J.D. Mondol, Y.G. Yohanis, B. Norton, Optimal sizing of array and inverter for grid-connected photovoltaic systems, Sol. Energy 80 (2006) 1517–1539, https://doi.org/10.1016/j.solener.2006.01.006.
[52] K. Peippo, P.D. Lund, Optimal sizing of grid-connected PV-systems for different climates and array orientations: a simulation study, Sol. Energy Mater. Sol. Cells 35 (1994) 445–451, https://doi.org/10.1016/0927-0248(94)90172-4.
[53] X. Hu, S. Li, Y. Yang, Advanced machine learning approach for lithium-ion battery state estimation in electric vehicles, IEEE Trans. Transp. Electrif. 7782 (2015), https://doi.org/10.1109/TTE.2015.2512237, 1–1.
[54] X. Wu, X. Hu, S. Moura, X. Yin, V. Pickert, Stochastic control of smart home energy management with plug-in electric vehicle battery energy storage and photovoltaic array, J. Power Sources 333 (2016) 203–212, https://doi.org/10.1016/j.jpowsour.2016.09.157.
[55] A. Joyce, C. Rodrigues, R. Manso, Modelling a PV system, Renew. Energy 22 (2001) 275–280, https://doi.org/10.1016/S0960-1481(00)00031-8.
[56] W. Chen, H. Shen, B. Shu, H. Qin, T. Deng, Evaluation of performance of MPPT devices in PV systems with storage batteries, Renew. Energy 32 (2007) 1611–1622, https://doi.org/10.1016/j.renene.2006.06.009.
[57] S. Piller, M. Perrin, A. Jossen, Methods for state-of-charge determination and their applications, J. Power Sources 96 (2001) 113–120, https://doi.org/10.1016/S0378-7753(01)00560-2.
[58] J. Wang, J.M. Bell, I.L. Skryabin, Kinetics of Charge Injection in Sol — Gel Deposited WO, 1999, p. 56.
[59] B.S. Borowy, Z.M. Salameh, Methodology for optimally sizing the combination of a battery bank and PV array in a Wind/PV hybrid system, IEEE Trans. Energy Convers. 11 (1996) 367–373, https://doi.org/10.1109/60.507648.
[60] L. Winkless, One step closer to self-powered switchable glazing? Mater. Today 19 (2016) 370, https://doi.org/10.1016/j.mattod.2016.07.016.

ELSEVIER LICENSE
TERMS AND CONDITIONS

Sep 19, 2022

This Agreement between Harbin Institute of Technology -- Ruicong Zhang ("You") and Elsevier ("Elsevier") consists of your license details and the terms and conditions provided by Elsevier and Copyright Clearance Center.

License Number	5392390410084
License date	Sep 19, 2022
Licensed Content Publisher	Elsevier
Licensed Content Publication	Joule
Licensed Content Title	Thermochromic VO2 for Energy-Efficient Smart Windows
Licensed Content Author	Yuanyuan Cui,Yujie Ke,Chang Liu,Zhang Chen,Ning Wang,Liangmiao Zhang,Yang Zhou,Shancheng Wang,Yanfeng Gao,Yi Long
Licensed Content Date	Sep 19, 2018
Licensed Content Volume	2
Licensed Content Issue	9
Licensed Content Pages	40
Start Page	1707
End Page	1746
Type of Use	reuse in a journal/magazine
Requestor type	academic/educational institute
Portion	figures/tables/illustrations
Number of figures/tables/illustrations	2
Format	both print and electronic
Are you the author of this Elsevier article?	No
Will you be translating?	No
Title of new article	Advanced liquid crystal-based switchable optical devices for light protection applications: principles and strategies
Lead author	Ruicong Zhang, Zhibo Zhang, Jiecai Han, Lei Yang, Jiajun li, Zicheng Song Tianyu Wang, Jiaqi Zhu
Title of targeted journal	Light: Science & Applications
Publisher	Springer Nature
Expected publication date	Nov 2022
Portions	Figure 1B, Figure 2H
Requestor Location	Harbin Institute of Technology<br>No. 92, Xidazhi Street, Nangang District<br><br>Harbin, 150080<br>China<br>Attn: Harbin Institute of Technology
Publisher Tax ID	GB 494 6272 12
Total	**0.00 USD**
Terms and Conditions	

INTRODUCTION

1. The publisher for this copyrighted material is Elsevier. By clicking "accept" in connection with completing this licensing transaction, you agree that the following terms and conditions apply to this transaction (along with the Billing and Payment terms

and conditions established by Copyright Clearance Center, Inc. ("CCC"), at the time that you opened your Rightslink account and that are available at any time at http://myaccount.copyright.com).

GENERAL TERMS

2. Elsevier hereby grants you permission to reproduce the aforementioned material subject to the terms and conditions indicated.
3. Acknowledgement: If any part of the material to be used (for example, figures) has appeared in our publication with credit or acknowledgement to another source, permission must also be sought from that source. If such permission is not obtained then that material may not be included in your publication/copies. Suitable acknowledgement to the source must be made, either as a footnote or in a reference list at the end of your publication, as follows:
"Reprinted from Publication title, Vol /edition number, Author(s), Title of article / title of chapter, Pages No., Copyright (Year), with permission from Elsevier [OR APPLICABLE SOCIETY COPYRIGHT OWNER]." Also Lancet special credit - "Reprinted from The Lancet, Vol. number, Author(s), Title of article, Pages No., Copyright (Year), with permission from Elsevier."
4. Reproduction of this material is confined to the purpose and/or media for which permission is hereby given.
5. Altering/Modifying Material: Not Permitted. However figures and illustrations may be altered/adapted minimally to serve your work. Any other abbreviations, additions, deletions and/or any other alterations shall be made only with prior written authorization of Elsevier Ltd. (Please contact Elsevier's permissions helpdesk here). No modifications can be made to any Lancet figures/tables and they must be reproduced in full.
6. If the permission fee for the requested use of our material is waived in this instance, please be advised that your future requests for Elsevier materials may attract a fee.
7. Reservation of Rights: Publisher reserves all rights not specifically granted in the combination of (i) the license details provided by you and accepted in the course of this licensing transaction, (ii) these terms and conditions and (iii) CCC's Billing and Payment terms and conditions.
8. License Contingent Upon Payment: While you may exercise the rights licensed immediately upon issuance of the license at the end of the licensing process for the transaction, provided that you have disclosed complete and accurate details of your proposed use, no license is finally effective unless and until full payment is received from you (either by publisher or by CCC) as provided in CCC's Billing and Payment terms and conditions. If full payment is not received on a timely basis, then any license preliminarily granted shall be deemed automatically revoked and shall be void as if never granted. Further, in the event that you breach any of these terms and conditions or any of CCC's Billing and Payment terms and conditions, the license is automatically revoked and shall be void as if never granted. Use of materials as described in a revoked license, as well as any use of the materials beyond the scope of an unrevoked license, may constitute copyright infringement and publisher reserves the right to take any and all action to protect its copyright in the materials.
9. Warranties: Publisher makes no representations or warranties with respect to the licensed material.
10. Indemnity: You hereby indemnify and agree to hold harmless publisher and CCC, and their respective officers, directors, employees and agents, from and against any and all claims arising out of your use of the licensed material other than as specifically authorized pursuant to this license.
11. No Transfer of License: This license is personal to you and may not be sublicensed, assigned, or transferred by you to any other person without publisher's written permission.
12. No Amendment Except in Writing: This license may not be amended except in a writing signed by both parties (or, in the case of publisher, by CCC on publisher's behalf).
13. Objection to Contrary Terms: Publisher hereby objects to any terms contained in any purchase order, acknowledgment, check endorsement or other writing prepared by you, which terms are inconsistent with these terms and conditions or CCC's Billing and Payment terms and conditions. These terms and conditions, together with CCC's Billing and Payment terms and conditions (which are incorporated herein), comprise the entire agreement between you and publisher (and CCC) concerning this licensing transaction. In the event of any conflict between your obligations established by these terms and conditions and those established by CCC's Billing and Payment terms and conditions, these terms and conditions shall control.
14. Revocation: Elsevier or Copyright Clearance Center may deny the permissions described in this License at their sole discretion, for any reason or no reason, with a full refund payable to you. Notice of such denial will be made using the contact information provided by you. Failure to receive such notice will not alter or invalidate the denial. In no event will Elsevier or Copyright Clearance Center be responsible or liable for any costs, expenses or damage incurred by you as a result of a denial of your permission request, other than a refund of the amount(s) paid by you to Elsevier and/or Copyright Clearance Center for denied permissions.

LIMITED LICENSE

The following terms and conditions apply only to specific license types:
15. **Translation**: This permission is granted for non-exclusive world **English** rights only unless your license was granted for translation rights. If you licensed translation rights you may only translate this content into the languages you requested. A professional translator must perform all translations and reproduce the content word for word preserving the integrity of the article.
16. **Posting licensed content on any Website**: The following terms and conditions apply as follows: Licensing material from an Elsevier journal: All content posted to the web site must maintain the copyright information line on the bottom of each image; A hyper-text must be included to the Homepage of the journal from which you are licensing at http://www.sciencedirect.com/science/journal/xxxxx or the Elsevier homepage for books at http://www.elsevier.com; Central Storage: This license does not include permission for a scanned version of the material to be stored in a central repository such as that provided by Heron/XanEdu.
Licensing material from an Elsevier book: A hyper-text link must be included to the Elsevier homepage at http://www.elsevier.com . All content posted to the web site must maintain the copyright information line on the bottom of each image.

Posting licensed content on Electronic reserve: In addition to the above the following clauses are applicable: The web site must be password-protected and made available only to bona fide students registered on a relevant course. This permission is granted for 1 year only. You may obtain a new license for future website posting.
17. **For journal authors:** the following clauses are applicable in addition to the above:
Preprints:
A preprint is an author's own write-up of research results and analysis, it has not been peer-reviewed, nor has it had any other value added to it by a publisher (such as formatting, copyright, technical enhancement etc.).
Authors can share their preprints anywhere at any time. Preprints should not be added to or enhanced in any way in order to appear more like, or to substitute for, the final versions of articles however authors can update their preprints on arXiv or RePEc with their Accepted Author Manuscript (see below).
If accepted for publication, we encourage authors to link from the preprint to their formal publication via its DOI. Millions of researchers have access to the formal publications on ScienceDirect, and so links will help users to find, access, cite and use the best available version. Please note that Cell Press, The Lancet and some society-owned have different preprint policies. Information on these policies is available on the journal homepage.
Accepted Author Manuscripts: An accepted author manuscript is the manuscript of an article that has been accepted for publication and which typically includes author-incorporated changes suggested during submission, peer review and editor-author communications.
Authors can share their accepted author manuscript:

- immediately
  - via their non-commercial person homepage or blog
  - by updating a preprint in arXiv or RePEc with the accepted manuscript
  - via their research institute or institutional repository for internal institutional uses or as part of an invitation-only research collaboration work-group
  - directly by providing copies to their students or to research collaborators for their personal use
  - for private scholarly sharing as part of an invitation-only work group on commercial sites with which Elsevier has an agreement
- After the embargo period
  - via non-commercial hosting platforms such as their institutional repository
  - via commercial sites with which Elsevier has an agreement

In all cases accepted manuscripts should:

- link to the formal publication via its DOI
- bear a CC-BY-NC-ND license - this is easy to do
- if aggregated with other manuscripts, for example in a repository or other site, be shared in alignment with our hosting policy not be added to or enhanced in any way to appear more like, or to substitute for, the published journal article.

Published journal article (JPA): A published journal article (PJA) is the definitive final record of published research that appears or will appear in the journal and embodies all value-adding publishing activities including peer review co-ordination, copy-editing, formatting, (if relevant) pagination and online enrichment.
Policies for sharing publishing journal articles differ for subscription and gold open access articles:
Subscription Articles: If you are an author, please share a link to your article rather than the full-text. Millions of researchers have access to the formal publications on ScienceDirect, and so links will help your users to find, access, cite, and use the best available version.
Theses and dissertations which contain embedded PJAs as part of the formal submission can be posted publicly by the awarding institution with DOI links back to the formal publications on ScienceDirect.
If you are affiliated with a library that subscribes to ScienceDirect you have additional private sharing rights for others' research accessed under that agreement. This includes use for classroom teaching and internal training at the institution (including use in course packs and courseware programs), and inclusion of the article for grant funding purposes.
Gold Open Access Articles: May be shared according to the author-selected end-user license and should contain a CrossMark logo, the end user license, and a DOI link to the formal publication on ScienceDirect.
Please refer to Elsevier's posting policy for further information.
18. **For book authors** the following clauses are applicable in addition to the above: Authors are permitted to place a brief summary of their work online only. You are not allowed to download and post the published electronic version of your chapter, nor may you scan the printed edition to create an electronic version. **Posting to a repository:** Authors are permitted to post a summary of their chapter only in their institution's repository.
19. **Thesis/Dissertation**: If your license is for use in a thesis/dissertation your thesis may be submitted to your institution in either print or electronic form. Should your thesis be published commercially, please reapply for permission. These requirements include permission for the Library and Archives of Canada to supply single copies, on demand, of the complete thesis and include permission for Proquest/UMI to supply single copies, on demand, of the complete thesis. Should your thesis be published commercially, please reapply for permission. Theses and dissertations which contain embedded PJAs as part of the formal submission can be posted publicly by the awarding institution with DOI links back to the formal publications on ScienceDirect.

Elsevier Open Access Terms and Conditions

You can publish open access with Elsevier in hundreds of open access journals or in nearly 2000 established subscription journals that support open access publishing. Permitted third party re-use of these open access articles is defined by the author's choice of Creative Commons user license. See our open access license policy for more information.

Terms & Conditions applicable to all Open Access articles published with Elsevier:

Any reuse of the article must not represent the author as endorsing the adaptation of the article nor should the article be modified in such a way as to damage the author's honour or reputation. If any changes have been made, such changes must be clearly indicated.

The author(s) must be appropriately credited and we ask that you include the end user license and a DOI link to the formal publication on ScienceDirect.

If any part of the material to be used (for example, figures) has appeared in our publication with credit or acknowledgement to another source it is the responsibility of the user to ensure their reuse complies with the terms and conditions determined by the rights holder.

Additional Terms & Conditions applicable to each Creative Commons user license:

CC BY: The CC-BY license allows users to copy, to create extracts, abstracts and new works from the Article, to alter and revise the Article and to make commercial use of the Article (including reuse and/or resale of the Article by commercial entities), provided the user gives appropriate credit (with a link to the formal publication through the relevant DOI), provides a link to the license, indicates if changes were made and the licensor is not represented as endorsing the use made of the work. The full details of the license are available at http://creativecommons.org/licenses/by/4.0.

CC BY NC SA: The CC BY-NC-SA license allows users to copy, to create extracts, abstracts and new works from the Article, to alter and revise the Article, provided this is not done for commercial purposes, and that the user gives appropriate credit (with a link to the formal publication through the relevant DOI), provides a link to the license, indicates if changes were made and the licensor is not represented as endorsing the use made of the work. Further, any new works must be made available on the same conditions. The full details of the license are available at http://creativecommons.org/licenses/by-nc-sa/4.0.

CC BY NC ND: The CC BY-NC-ND license allows users to copy and distribute the Article, provided this is not done for commercial purposes and further does not permit distribution of the Article if it is changed or edited in any way, and provided the user gives appropriate credit (with a link to the formal publication through the relevant DOI), provides a link to the license, and that the licensor is not represented as endorsing the use made of the work. The full details of the license are available at http://creativecommons.org/licenses/by-nc-nd/4.0. Any commercial reuse of Open Access articles published with a CC BY NC SA or CC BY NC ND license requires permission from Elsevier and will be subject to a fee.

Commercial reuse includes:

- Associating advertising with the full text of the Article
- Charging fees for document delivery or access
- Article aggregation
- Systematic distribution via e-mail lists or share buttons

Posting or linking by commercial companies for use by customers of those companies.

20. **Other Conditions**:

v1.10

Questions? customercare@copyright.com or +1-855-239-3415 (toll free in the US) or +1-978-646-2777.

Joule

CellPress REVIEWS

Review

Thermochromic VO_2 for Energy-Efficient Smart Windows

Yuanyuan Cui,[1,5] Yujie Ke,[2,5] Chang Liu,[2] Zhang Chen,[1] Ning Wang,[2,3] Liangmiao Zhang,[1] Yang Zhou,[2] Shancheng Wang,[2] Yanfeng Gao,[1,*] and Yi Long[2,4,*]

ABSTRACT

Rapid development of the thermochromic glazing technique promises next-generation architectural windows with energy-saving characteristics by intelligently regulating indoor solar irradiation via modulating windows' optical properties in response to the surrounding temperature. Vanadium dioxide (VO_2) is a promising material for energy-saving smart windows due to its reversible metal-to-insulator transition near room temperature and accompanying large changes in its optical properties. This review provides a comprehensive overview of the application of VO_2 to smart windows with particular emphasis on recent progress from the electronic, atomic, nano, and micron perspectives. The effects of intrinsic atomic defects, elemental doping, and lattice strain on VO_2 nanocrystals are examined. Nano- and microscale morphology engineering approaches that aim to enhance the thermochromic performance and impart practical multi-functionalities are summarized. Finally, the challenges and future directions of VO_2-based smart windows are elaborated to bridge the gap between the lab research and large-scale practical applications.

Introduction

VO_2 and Its Metal-to-Insulator Phase Transition

In recent years, both monoclinic (M) and rutile (R) phase vanadium dioxide (VO_2) have aroused great attention as a promising candidate for smart windows owing to the reversible metal-to-insulator transition (MIT) at a critical temperature of 68°C (341 K).[1,2] This thermally induced phase transition is reversible, accompanied by a dramatic change in the optical properties in the near-infrared region from a low-temperature transparent state to a more blocking state at high temperatures, which imbues the VO_2-based window with the ability to regulate solar heat flux by responding to temperature automatically. Compared with other thermochromic materials, VO_2 possesses a relatively "silent" condition with a negligible optical property change in the visible spectrum range.[3] In fact, VO_2(M/R) is also a widely studied material in physical chemistry and condensed-matter physics because of its specific phase-transition features.[4–6]

The low-temperature insulating phase has a monoclinic structure (M, space group $P2_1/c$, a_M = 5.75 Å, b_M = 4.52 Å, c_M = 5.38 Å, β = 122.6°).[7,8] When the temperature is above 68°C, this low-temperature insulating phase transforms to the high-temperature metallic phase, which displays a tetragonal structure (R, space group $P4_2/nm$, a_R = b_R = 4.55 Å, c_R = 2.86 Å)[9] (Figure 1B). In VO_2(R), the vanadium atoms occupy the lattice point of the body-centered cubic structure and are located at the centers of the tilted VO_6 octahedra.[10] Four of the six oxygen atoms in the VO_6 octahedra are located closer to the vanadium atom, and the V-O bond distances are 1.92 and 1.93 Å, respectively. Chains of edge-sharing VO_6 octahedra

Context & Scale

Vanadium dioxide (VO_2) is a promising material for energy-saving smart windows due to its reversible metal-to-insulator transition near room temperature. This thermally induced phase transition is reversible, and it is accompanied by a dramatic change in the optical properties in the near-infrared region from a low-temperature transparent state to a more blocking state at high temperatures, imbuing the VO_2-based window with the ability to regulate solar heat flux by responding to temperature automatically. In this review, the progress in VO_2-based smart windows is overviewed, from the band structure designing at the electronic and atomic scales to morphology engineering from nano- to microscales.

We discuss the effects of intrinsic atomic defects, elemental doping, and lattice strain on the electronic and atomic structures of VO_2. Subsequently, nano- and microscale engineering methods to enhance the performance of smart windows are presented, including incorporating particles, tuning the porosity, designing nanocomposites, developing biomimetic patterns, creating grids, and applying multifunctional antireflection coatings. The energy efficiency is

CellPress REVIEWS

have a corner-sharing arrangement with other VO_6 octahedra and are arranged linearly along the crystallographic *c* axis, with a V-V distance of ~2.85 Å.[11] During the phase transition from VO_2(R) to VO_2(M), the vanadium atoms move along the V-V direction, resulting in the pairing and tilting of VO_6 octahedra in this direction.[12] Two distinct sets of long and short V-V distances (3.12 Å and 2.65 Å) exist as a result of the new positions of vanadium atoms in VO_2(M). The number of atoms in one VO_2(M) unit cell is 12, which is doubled as compared with the 6 atoms in one VO_2(R) unit cell.

To fully describe the phase-transition process of VO_2(M/R), a molecular picture based on relatively simple crystal-field theory is typically considered, as first proposed by Goodenough in 1971.[14] In brief and as shown in Figure 1A, in the VO_2(R), a wide π bond and a narrow π^* anti-bond are formed between the V^{4+} and O^{2-} orbitals, and a $d_{//}$ nonbond is formed between adjacent V^{4+} orbitals along the crystallographic *c* axis. Although the energy band of VO_2(R) is approximately 2.5 eV, the unfilled π^* and $d_{//}$bands partially overlap, and the Fermi level falls at the point where the π^* and $d_{//}$ bands overlap, giving it metallic characteristics. When the temperature decreases, the tilting of the VO_6 octahedra enhances the π overlap between the V^{4+} and O^{2-} orbitals, thereby elevating the anti-bonding π^* level, whereas the $d_{//}$ bonds interact strongly in V-V pairs and then split into $d_{//}$-bonding and anti-bonding components. A band gap of ~0.7 eV is formed between the π^* and $d_{//}$ bonds, leading to the formation of an insulating phase. Although this model exhibits several discrepancies from the experimental data, it can nevertheless qualitatively explain the nature of the phase transition in VO_2.

A fierce debate over the driving force of the VO_2 phase transition has persisted for several decades, with respect to whether the electron-electron correlation is strong enough to localize the electrons by forming a Mott-Hubbard insulator (Mott model) or whether structural distortions alone can induce the insulating phase (Peierls model).[15–19] Wentzcovitch et al. revealed a distorted monoclinic ground state to be in good agreement with the experimental findings and a nearly open gap with respect to charge excitations; thus, they believed that VO_2 may be more band-like than correlated.[19] This result was supported by Cavalleri et al., who employed ultrafast spectroscopy to construct a time-domain hierarchy between the structural and electronic effects, and found that the initiation of the metallic phase formation was prompted by hole photo-doping into the valence band of the insulator phase; therefore, the phase transition could be retarded with respect to hole injection and exhibits a bottleneck timescale.[20–22] Baum et al. used four-dimensional (4D) ultrafast electron microscopy to study the phase transition of VO_2 initiated by near-infrared excitation.[23] Their results suggest that the transition from the insulating phase to the metallic phase initially involves the expansion of the primary V-V bond with local displacements (on femtosecond and picosecond timescales), followed by long-range shear rearrangements (on a nanoseconds timescale and at the speed of sound), revealing a structural pathway and non-concerted transformation mechanism.

However, a number of phenomena, such as anomalously low conductivity and other unusual properties of the metallic phase, have suggested that the MIT phase transition involves strong electron-electron correlations (Mott model).[24] The fact that an intermediate monoclinic phase is insulating despite the presence of undimerized V-V chains; and the dependence on excitation power observed experimentally, indicate the sensitivity to the density of the excited carriers. The alternative Mott picture ascribes the presence of a band gap in VO_2(M) to strong electron-electron

also discussed from both simulations and experimental aspects. Lastly, challenges and future directions are discussed. We hope that this work may inspire more innovative progress and accelerate the development of this technique from the lab to industry.

[1]School of Materials Science and Engineering, Shanghai University, Shanghai 200444, China

[2]School of Materials Science and Engineering, Nanyang Technological University, 50 Nanyang Avenue, Singapore 639798, Singapore

[3]Shenzhen Institutes of Advanced Technology, Chinese Academy of Sciences, Shenzhen 518055, China

[4]Singapore-HUJ Alliance for Research and Enterprise (SHARE), Nanomaterials for Energy and Energy-Water Nexus (NEW), Campus for Research Excellence and Technological Enterprise (CREATE), Singapore 138602, Singapore

[5]These authors contributed equally

*Correspondence: yfgao@shu.edu.cn (Y.G.), longyi@ntu.edu.sg (Y.L.)

https://doi.org/10.1016/j.joule.2018.06.018

CellPress
REVIEWS

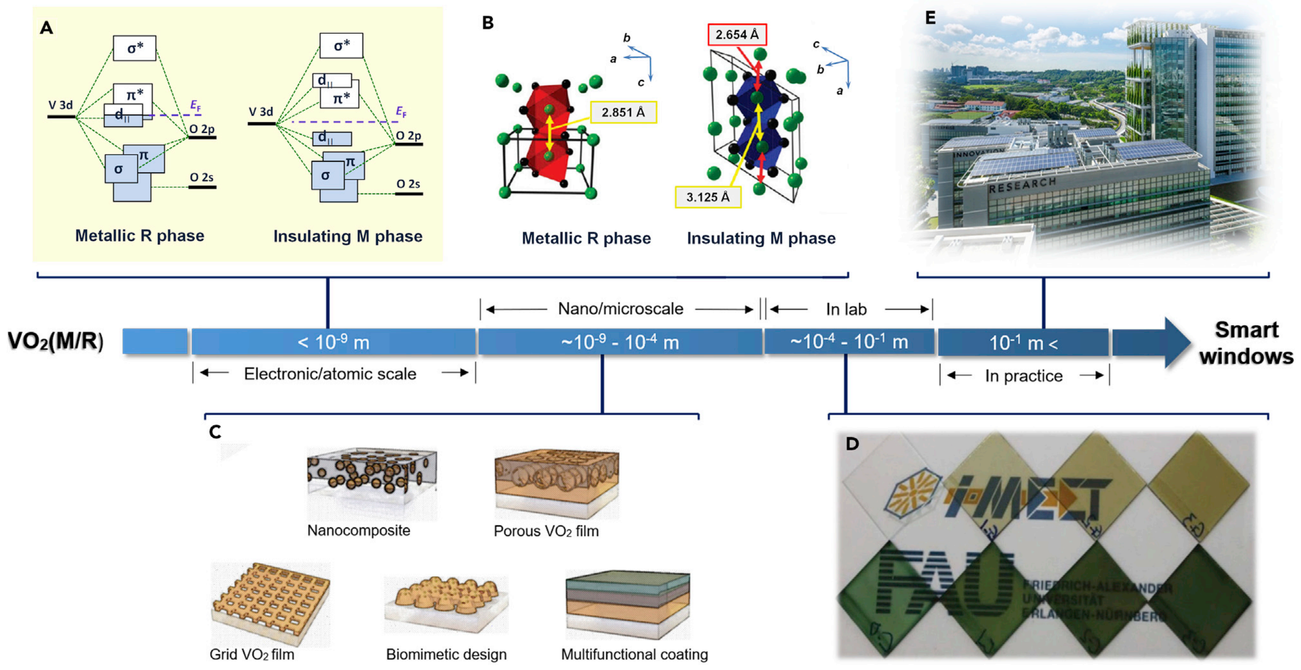

Figure 1. Overview of the Development of VO_2 Smart Windows from the Electronic/Atomic Scale to the Nano/Microscale, Then to In-Lab Tests, and Finally Their Practical Application in Architecture

(A) Band structures of the metallic R and insulating M phases of VO_2 depicted by molecular orbital diagrams.

(B) Schematic of the atomic structures of the high-temperature metallic tetragonal phase R and the low-temperature insulating monoclinic phase M. The V-V distances in each crystal structure are highlighted.

(C) Nano/microengineering toward performance enhancement.

(D) Photographs of VO_2-based thermochromic samples in the lab.

(E) Photographs of architectural windows in practice.

Figures reproduced with permission from: (A) and (B), Whittaker et al.,[11] American Chemical Society; (D), Guo et al.,[13] Wiley.

correlations, and the results of several experiments have indicated that if the thermally, optically or gate-voltage-induced excitation of carriers reaches a threshold, the metallic phase could develop even without a phase transformation.[11]

Yuan et al. recently reported measurements and calculations of the VO_2 phase transition and suggested that it might be both electronically and structurally driven,[25] in accordance with the stepwise non-concerted mechanism of photo-excitation-induced phase transition in VO_2 (M/R) proposed by Baum et al.[23] In brief, at least three distinct time scales emerge in the phase transition. The first step is the rapid dilation of the V-V bond (~307 fs); this step is followed by a slower, transverse motion of the VO_6 octahedra that locally rearrange to adopt a more rutile-like geometry (~9.2 ps) and a subsequent and far slower motion (~100 ps) that is ascribed to shear movements propagating at the speed of sound. When the phase transition is approached after the three aforementioned steps, a spontaneous Peierls distortion would start initially for the cation chains. Finally, the extent of the distortion increases before the Peierls distortion spreads to the orthogonal chains.

Optical Performance

Among the issues concerning VO_2-based thermochromic windows, optical performance is the most important because it directly determines the energy-conservation efficiency of the windows. The optical performance of a VO_2-based thermochromic window can be mainly characterized in terms of its luminous transmittance

(T_{lum}, 380–780 nm), solar-energy modulation ability (defined as ΔT_{sol}, the difference in solar-energy transmittance T_{sol} before and after the phase transition, which is 240–2,500 nm). These quantities are calculated as follows:

$$T_i = \int \phi_i(\lambda)T(\lambda)d\lambda \Big/ \int \phi_i(\lambda)d\lambda, \quad \text{(Equation 1)}$$

$$\Delta T_{sol} = T_{sol}(T<T_c) - T_{sol}(T>T_c), \quad \text{(Equation 2)}$$

where $T(\lambda)$ represents the transmittance at wavelength λ, i denotes lum or sol, $\phi_{lum}(\lambda)$ is the standard luminous efficiency function for vision (380–780 nm), and $\phi_{sol}(\lambda)$ is the solar irradiance spectrum at air mass 1.5 (corresponding to the sun standing 37° above the horizon).[26] For simplicity, all T_{lum} values presented in this review represent the average of the T_{lum} values at low and high temperatures.

Both high T_{lum} and ΔT_{sol} are important as the former can save lighting while the latter determines the energy-conservation efficiency of VO_2-based thermochromic windows. However, they have a trade-off relationship, meaning that it is difficult to improve both to an acceptable value; therefore most studies of thermochromic devices have focused on such attempts. For a continuous VO_2 thin film, the T_{lum} and ΔT_{sol} are limited and are unsatisfactory for practical use. Reported solutions have involved three different approaches: optimization of the optical thin films, design of the film microstructure, and formation of nanocomposites.

Another important parameter to characterize the energy-conservation efficiency of VO_2-based smart windows is the thermal emissivity (ε_T), which is referred as the ratio of energy radiated by the windows to energy radiated by a black body at a defined temperature.[27] In thermal equilibrium conditions, the absorptivity of a subject is equal to its emissivity. Therefore, a black body absorbs all electromagnetic radiation that falls on it and shows ε_T = 1, whereas a perfect reflector reflects all electromagnetic radiation and presents ε_T = 0. The value of ε_T lies between 0 and 1. Generally, ε_T is calculated by weighting the film reflectance with the black-body emission spectrum from 4.5 to 25 mm as follows:

$$\varepsilon_T = \sum_{4.5}^{2.5} G_T(\lambda)E(\lambda)\Delta\lambda, \quad \text{(Equation 3)}$$

where $G_T(\lambda)$ is the normalized relative spectral distribution of black-body radiation at temperature T (T is chosen to be 20°C according to CNS GB/T 1895.2–2002). $E(\lambda)$ refers to the emittance, i.e., the fraction of the black-body radiation.

There are several recently published review papers. Gao et al. specifically discussed the VO_2 in thermochromic glass application via solution processes.[2] Li et al. concluded the hydrothermal method and the transformation of VO_2(M) from its polymorphs.[28] Wu and Xie et al. emphasized the engineering microstructures of VO_2 with control of its electrical properties.[29] Yu et al. outlined the deposition methods and the progress in performance enhancement.[30] Wang et al. summarized the chemical vapor deposition (CVD) deposition of VO_2 in energy conservation and storage.[31] Granqvist's group focused on the state of the art for VO_2-based thin films and nanocomposites.[32] Few of them focus on the theoretical simulations and experimental works regarding the structure/property relationship from atomic, nano, and micron perspectives, which may provide a comprehensive and insightful guidance of VO_2-based smart windows.

In this review, the progress in VO_2-based smart windows is overviewed, from the band structure designing at the electronic and atomic scales to morphology

engineering from nano- to microscales (Figure 1). The second section discusses the effects of intrinsic atomic defects, elemental doping, and lattice strain on the electronic and atomic structures of VO_2. Subsequently, nano- and microscale engineering methods to enhance the performance of smart windows are presented, including incorporating particles, tuning the porosity, designing nanocomposites, developing biomimetic patterns, creating grids, and applying multifunctional antireflection coatings (Figure 1C). The energy efficiency is also discussed from both simulations and experimental aspects. Lastly, challenges and future directions are shared. We hope that this work may inspire more innovative progress and accelerate the development of this technique from the lab to industry (Figures 1D and 1E).

Electronic and Atomic Structure of VO_2

In this section the intrinsic point defects, dopant, and strain influence on VO_2 will be overviewed with special focus on reducing the phase-transition temperature (τ_c) of VO_2 from electronic and atomic perspectives.

Intrinsic Point Defects

Intrinsic point defects, such as cation nonstoichiometry or oxygen vacancies, always exist in metallic oxides and are desirable or even crucial in some cases to affect certain properties.[33] Recently, the introduction of oxygen vacancies into VO_2 presents great potential in reducing its τ_c and even alters its optical and electrical properties.[14,34]

The underlying mechanism can be interpreted as follows: The reaction to form one oxygen vacancy can be expressed as $O_o^{\times} \rightarrow V_o + 2e + 0.5O_2$,[35] where the two resulting electrons are trapped by V^{4+} sites and then lead to lower valence states of V. This further induces multiple donor levels below the π^* band, resulting in narrowed band gap for VO_2(M) (Figures 2A–2C),[35,36] which is closely correlated with the τ_c and the optical properties of VO_2.

To probe the effect of oxygen vacancies in VO_2, Chen et al. conducted first-principles calculations on the VO_{2-x} crystalline models.[37,40] Their simulations revealed that the oxygen vacancies give rise to an enhanced concentration of electrons, and the calculated τ_c of $VO_{1.984}$ and $VO_{1.969}$ are reduced to 226 and 142 K, respectively (Figure 2D), which are much lower than that of pure VO_2 (340 K).[37,40] In addition, the calculated absorption coefficients ($a(\omega)$) of VO_2 and $VO_{1.984}$ (Figure 2E) showed that after the introduction of oxygen vacancies, a new peak appeared in the low-energy region at approximately 0.4 eV in VO_{2-x}(M), which could improve the sunlight utilization in the infrared region.[40] The calculated reflectivity spectra of $VO_{1.984}$ (Figure 2F) illustrates that the reflectivity ($R(\omega)$) of $VO_{1.984}$(M) and $VO_{1.984}$(R) are higher than those of pure VO_2, suggesting that the introduction of oxygen vacancies into VO_2 can improve the reflectivity in the infrared region.[37]

Experimentally, oxygen vacancies are frequently introduced by adjusting the oxygen flow ratio during the process of preparation. For instance, Zhang et al. fabricated a series of VO_2 nanobeams and found that oxygen vacancies can stabilize the VO_2(R) phase at 103 K, suppressing of the phase transition by 238 K (Figure 2G).[38] Zhang et al. also reported that the presence of oxygen vacancies induced by the oxygen pressure decreases the τ_c.[41] Jiang plotted the transmittance spectrum (Figure 2H) and absorptivity spectrum (Figure 2I) of the VO_2 thin films at different oxygen flow ratios and found that the low-temperature VO_2(M) phase showed a gradual decrease in the near-infrared (NIR) transmittance but increased absorptivity with decreasing

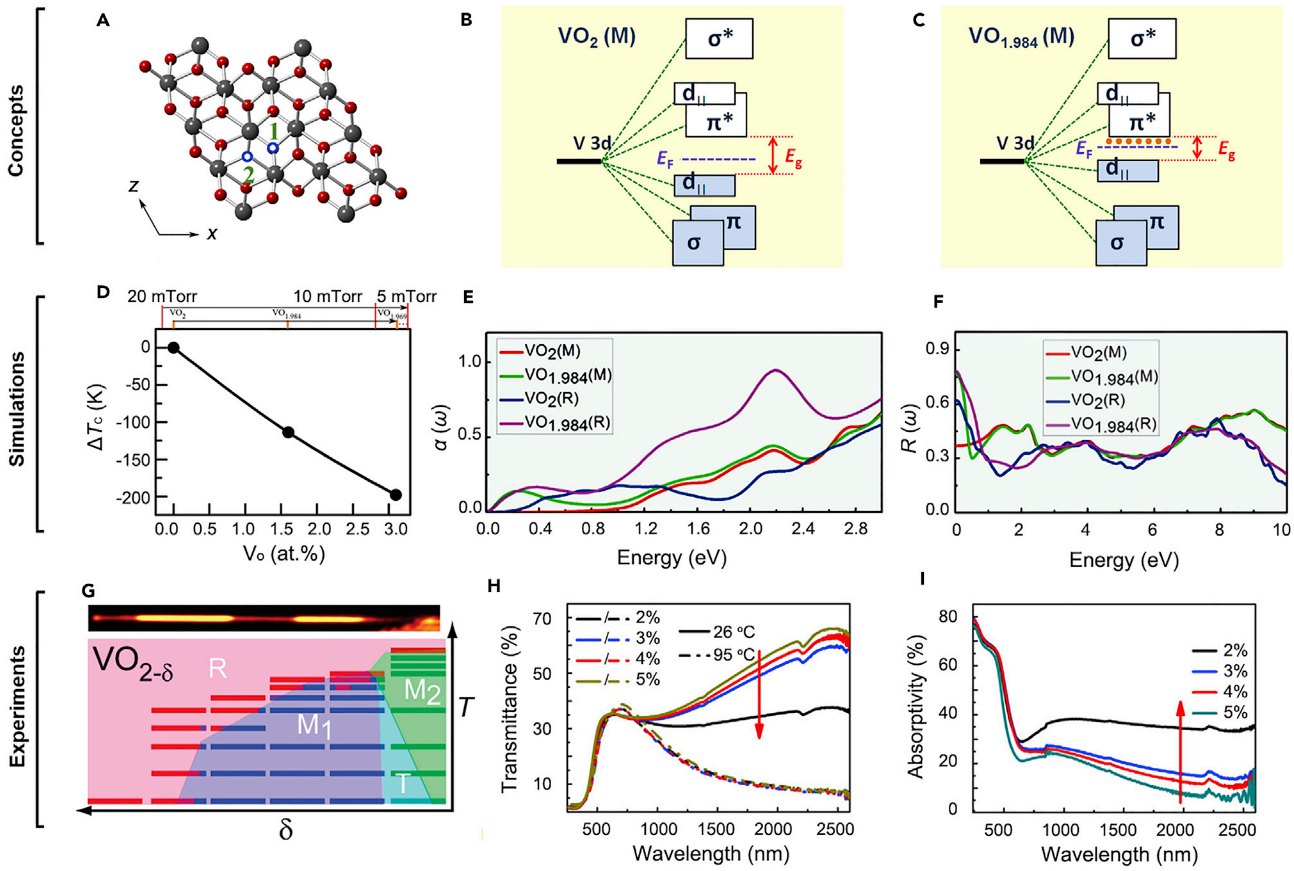

Figure 2. The Effect of Oxygen Vacancies on the Thermochromic Performance of VO_2

(A) Side view of the VO_2(M) crystal structure with oxygen vacancies. The small red spheres represent oxygen atoms and the large gray spheres represent vanadium atoms, O1 and O2 represent two types of oxygen vacancies in VO_2(M), and O1 site is energetically favorable over the O2 site.

(B and C) Outline of the band scheme of (B) VO_2(M) and (C) $VO_{1.984}$(M).

(D) Reduction of the transition temperature with an increase in the oxygen vacancy concentration.

(E) The absorption coefficients ($\alpha(\omega)$) of VO_2 and $VO_{1.984}$ given in 10^5 cm^{-1}.

(F) The reflectivity ($R(\omega)$) spectra of VO_2 and $VO_{1.984}$.

(G) Structural phase diagram showing the impact of annealing on the transition temperature.

(H and I) Transmittance (H) and absorptivity (I) spectra of VO_2 films fabricated with different oxygen flow ratios.

Figures reproduced with permission from: (A), Cui et al.,[36] American Institute of Physics; (D) to (F), Chen et al.,[37] Royal Society of Chemistry; (G), Zhang et al.,[38] American Chemical Society; (H) to (I), Jiang et al.,[39] Elsevier.

oxygen flow ratio.[39] Oxygen vacancies can also be introduced by loading an electric field on VO_2. For instance, Jeong et al. suppressed the phase transition below 5 K in VO_2 through the formation of electric field-induced oxygen vacancies.[42] Later, Chen et al. examined the formation, diffusion, and recovery of oxygen vacancies in an electrolyte-gated VO_2 crystal lattice by temperature-dependent *in situ* resistance measurements and first-principles calculations.[43] Their results showed that oxygen vacancies result in the deformation of crystal structures and induce polarization charges, therefore modulating the d-orbital occupancy in VO_2.[43]

Elemental Doping

Elemental doping is a process that intentionally introduces other element(s) into a pure material to modify its electrical or optical properties. For VO_2, elemental doping is one of the conventional strategies to tailor its τ_c and optical properties. Presently, more than 60 elements have been investigated as dopants for the VO_2

Table 1. Elemental Doping Effects on the Thermochromic Performance of VO_2 Thin Films

Group	Dopant	Doping Level (%)	T_{lum} (%)	ΔT_{sol} (%)	$d\tau_c/dx$ (°C/at.%)	Ref.
IA	H	3	–	–	−38	simul. Cui et al.,[92] 2015
	Li	3	–	–	−43	simul. Cui et al.,[96] 2016
	NA	3	–	–	−49	simul. Cui et al.,[96] 2006
	K	3	–	–	−94	simul. Cui et al.,[96] 2016
IIA	Be	3	–	–	−58	simul. Zhang et al.,[68] 2013
	Mg	5	82.1	4.8	−3	expt. Wang et al.,[86] 2015
	Ca	1.3	–	7.6	–	expt. Dietrich et al.,[91] 2015
	Sr	9.6	54.3	5.0	–	expt. Dietrich et al.,[91] 2015
	Sr	6.8	50.3	6.5	–	expt. Dietrich et al.,[91] 2015
	Ba	8.3	–	7.5	–	expt. Dietrich et al.,[91] 2015
IIIA	B	–	–	–	−83	simul. Zhang et al.,[75] 2014
VIIA	F	2.93	48.7	10.7	−11.3	expt. Dai et al.,[71] 2013
Transition metal	W	2	45.1	6.9	−20	expt. Hu et al.,[97] 2016
	Mo	2	–	–	−11	expt. Mai et al.,[55] 2006
	Nb	2–3	–	–	−7.8	expt. Piccirillo et al.,[56] 2007
	Zr	9.8	60.4	14.1	−0.4	expt. Shen et al.,[98] 2014
	Ti	1.1	53	17.2	0	expt. Chen et al.,[72] 2013
Rare earth	Eu	4	54	6.7	−6.5	expt. Cao et al.,[83] 2014
	Tb	4	65.9	4.6	−1.5	expt. Wang et al.,[99] 2016
	La	4	50.1	10.3	−1.1	expt. Wang et al.,[100] 2016
Co-doping	Mg + W	4% Mg + 2% W	81.3	4.3	−5.5	expt. Wang et al.,[86] 2015
	F + W	2.1% F + 1.8% W	–	–	−17.4	expt. Burkhardt et al.,[53] 2002
	Zr + W	8.5% Zr + 0.6% W	56.4	12.3	−1.3	expt. Shen et al.,[98] 2014
	Mo + W	1.02% Mo + 0.36% W	–	–	−23	expt. Xu et al.,[61] 2012

"–" means data not available.

system either experimentally or theorectically.[44–95] The effects of some doping elements are summarized in Table 1.

The electronic phase transition in VO_2 is proposed to be accompanied by a (nearly) simultaneous structural phase transition between the VO_2(R) and VO_2(M) phase.[25,101] If the energy barrier across the phase transition could be lowered by doping, τ_c would decrease. Accordingly, the selection of the doping element is commonly based on two aspects: increasing the carrier concentration to accelerate the electronic phase transition, or introducing distortion into the atomic structure to assist the structural phase transition.

Firstly, the doping element should increase the carrier concentration of VO_2. When the doping element serves as the donor or acceptor in VO_2, it injects its electrons or holes into the electronic structure of VO_2, which increases the carrier concentration in the system. Because the electronic phase transition of VO_2 is a debatably typical Mott phase transition,[102] the increased carrier concentration lowers the energy barrier across the phase transition, thus decreasing the τ_c. There are two doping strategies to increase the carrier concentration. One is to insert smaller-sized doping atoms, such as H,[92] Li,[96] Na,[96] and B,[75] into the interstitial sites of VO_2 (Figure 3A). For instance, hydrogen is the lightest atom, which possesses one electron in its outermost orbital. When doped

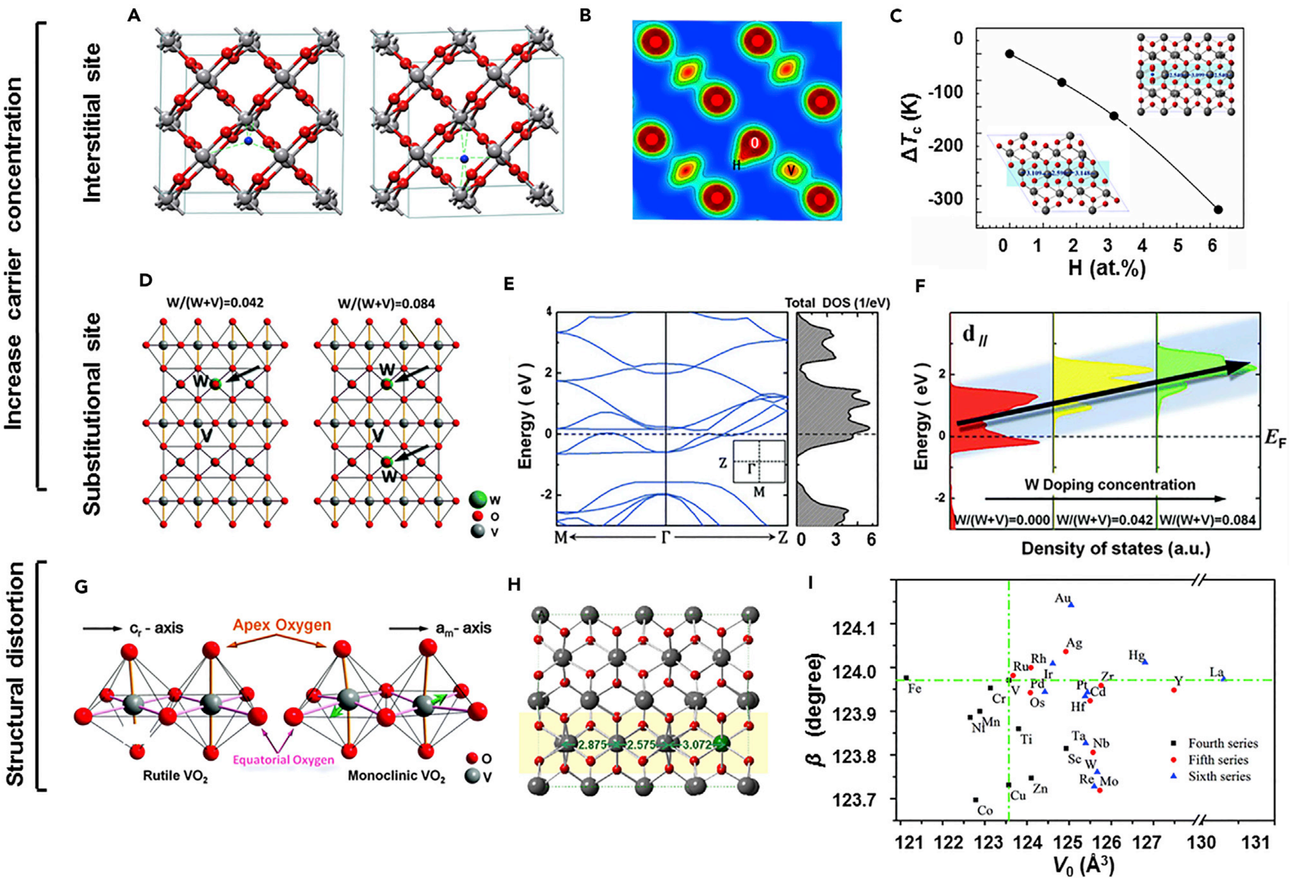

Figure 3. The Effect of Elemental Doping on the Thermochromic Performance of VO_2

(A) The location of H atom at the tetrahedral (left) and octahedral (right) interstitial site in VO_2, where H, V, and O atoms are indicated by blue, gray, and red spheres, respectively.

(B) The electron density of the (0 0 1) plane in H-doped VO_2(R).

(C) The dependence of the transition temperature reduction on the H-doping concentration.

(D) The location at which a W atom substitutes the V atom in VO_2, where the W, V, and O atoms are indicated by green, gray, and red spheres, respectively.

(E) Band structure and total density of states (DOS) of pure VO_2(R).

(F) Partial DOS of the $d_{\|}$ orbital in VO_2 with different W concentrations.

(G) Atomic structures for VO_2(R) and VO_2(M) during phase transition; the green arrows indicate the directions in which the V atoms.

(H) Sb dopant causes the V-V distances to alternatively vary in VO_2(R).

(I) Distribution of transition metal doped VO_2(M) with respect to the volume and β angle.

Figures reproduced with permission from: (A) to (C), Cui et al.,[92] Royal Society of Chemistry; (D) to (G), He et al.,[89] Royal Society of Chemistry; (H), Cui et al.,[105] Elsevier; (I), Sun et al.,[78] Royal Society of Chemistry.

into VO_2, the H atoms are located at the interstitial sites and inject their electrons into the VO_2 system (Figure 3B).[57,77,92] Gao et al. reported that H efficiently reduces the τ_c by 38 K/at% H (Figure 3C).[92] The other strategy to increase the carrier concentration is to substitute the V sites with high-valance elements (Figure 3D) such as W,[97,103] Nb,[56,80] and Mo.[55,104] The doped W atoms have been shown to inject some of their electrons into the V 3d valence bands and reduce the $\hat{o}_c$ by 20–26 K/at%[103]; He et al. gained the results that the optical band gap can be narrowed from 0.65 to 0.54 eV by increasing the W-doping concentration (Figures 3E and 3F).[89]

Secondly, the doping element should introduce structural distortion into VO_2, especially along the V-V chains. VO_2(M) and VO_2(R) belong to the space groups *P*21/*c* and

P42/mnm, respectively, and their apparent difference lies in the lengths of the V-V bonds, which are constant in VO_2(R) but alternatively varying in VO_2(M) (Figure 3G).[7,8] If a dopant causes the V-V distances in VO_2(R) to alternatively change (Figure 3H) thereby resembling that of VO_2(M), the dopant will lower the τ_c. For instance, Zhang et al. reported that Be-doped VO_2(R) displays structural distortion around the Be atom, where the V-V chains present dimerization similar to those in VO_2(M).[68] Through first-principles calculations, they predicted that the reduction of τ_c in the Be-doped VO_2 is as large as 58 K/at%.[68] In addition to the V-V distance, the lattice parameters can also be modified by dopants, which will influence the τ_c. Sun et al. proposed that if a dopant can introduce changes in the lattice parameters of VO_2(M) to resemble those of VO_2(R), the dopant will decrease the τ_c.[78] They conducted density functional theory calculations and found that the τ_c decreases with the expansion of the lattice and decrease in the β angle of VO_2(M) with transition metal doping (Figure 3I).[78]

Variation in the carrier concentration and structural distortion frequently occurs together, as the doping element either occupies the interstitial sites or substitutes the lattice site of V or O atoms, leading to variations in both the electronic and atomic structures of VO_2.

It was recently reported that elemental doping can also modify the emissivity, which is another important parameter to characterize the energy-conservation efficiency of VO_2.[106] For instance, Liu et al. synthesized pure VO_2 thin film on fused quartz substrate by using the sol-gel process, finding that the emissivity of VO_2 film (thickness of 900 nm) can be changed by 0.6 in the 7.5- to 14-μm region across the phase transition. They then prepared W-doped VO_2 films through the same process followed by the post-annealing.[106] Their results indicated that the emissivity of W-doped VO_2 thin films was decreased gradually with increasing doping amount of W, and the emissivity of VO_2 thin film (doping level of 4 at% W) dropped to 0.4 when the temperature was high then 30°C.[106]

Impacts of Strain on τ_c

In 1969, Ladd et al. first reported that the hydrostatic pressure only has a slight impact on the τ_c of VO_2, with a rate of $d\tau_c/dP$ = 0.6 K/GPa, whereas uniaxial stress along the *c* axis displays a noticeable effect, with a rate of $d\tau_c/dP = -12$ K/GPa.[107] Since then, there has been great interest in scaling the τ_c of VO_2 by loading strain or pressure on it.[38,108–111]

The τ_c is known to be closely correlated with the length of the *c* axis (adjacent V-V distance) of VO_2 (Figure 4A). Loading compressive strain along the *c* axis leads to further overlap of the d orbitals and the increasing width of the d band, thus stabilizing the VO_2(R) phase and reducing the τ_c. Two strategies are commonly used to apply strain to VO_2: bending suspended VO_2 beams along the length direction (Figure 4B)[24,112] and depositing VO_2 thin films on certain substrates to introduce an interaction between the film and substrate (Figure 4C).[113–118]

Experimentally, Cao et al. fabricated single-crystalline VO_2 beams followed by three-point bending along the length direction of the beams.[112] They constructed a phase-strain diagram and found that the τ_c decreased upon loading compressive strain on the VO_2 beams and increased upon loading tensile strain (Figure 4D).[112] Wei et al. patterned a series of vanadium metal contacts onto a VO_2 nanobeam followed by removing the underlying SiO_2 to suspend the nanobeam sections between the contacts and subsequently cycled it between room temperature and 120°C.[24] They also sketched a phase-pressure diagram of VO_2 (Figure 4E), illustrating

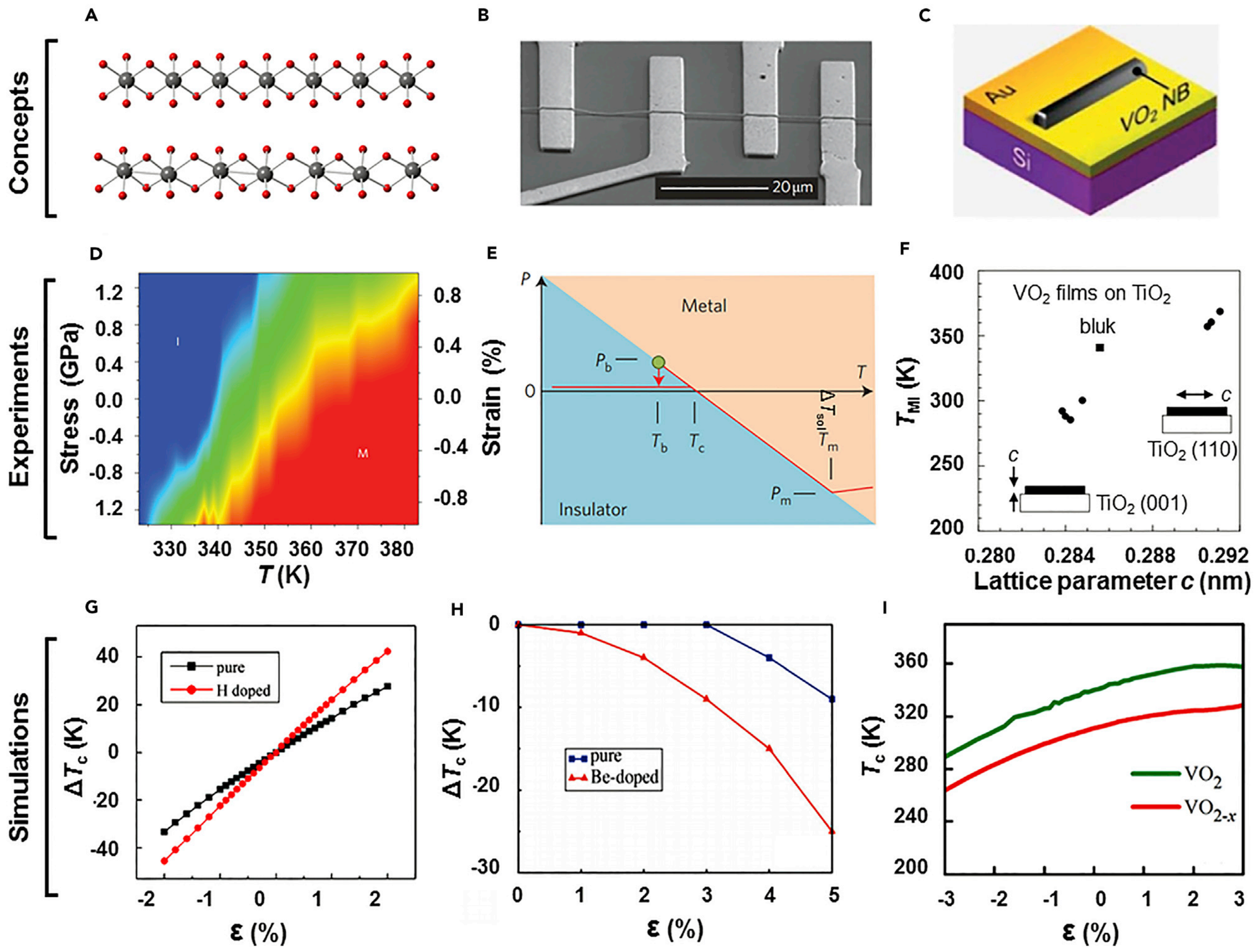

Figure 4. The Effect of Strain on the Phase Transition Temperature of VO_2

(A) V-V chains for pure VO_2(R) (upper) and VO_2(M) (lower); the small red spheres are oxygen atoms and the large gray spheres vanadium atoms.
(B) Scanning electron microscopy (EM) image of the suspended VO_2 nanobeam device.
(C) Schematic illustration of individual VO_2 nanobeams on a Au-coated Si substrate.
(D) Phase diagram of VO_2(M) fraction with respect to temperature, uniaxial stress, and uniaxial strain.
(E) Phase diagram of metallic and insulating VO_2 phases with respect to temperature and strain.
(F) τ_c versus the lattice parameter *c* for VO_2 films deposited on TiO_2(110) and TiO_2(001) substrates.
(G) Relationship between the strain and the reduction of τ_c in pure and H-doped VO_2.
(H) Relationship between the strain and the reduction of τ_c in pure and the Be-doped VO_2.
(I) Dependence of τ_c on the strain loaded on VO_{2-x}.
Figures reproduced with permission from: (B), Wei et al.,[24] Nature Publishing Group; (C), Chang et al.,[118] Royal Society of Chemistry; (D), Cao et al.,[112] Nature Publishing Group; (E), Wei et al.,[24] Nature Publishing Group; (F), Muraoka et al.,[116] Elsevier; (G), Cui et al.,[92] Royal Society of Chemistry; (H), Zhang et al.,[68] Royal Society of Chemistry; (I), Chen et al.,[40] Royal Society of Chemistry.

reduced τ_c under compressive strain and enhanced τ_c under tensile strain.[24] Muraoka et al. prepared VO_2 thin films by pulsed laser deposition on TiO_2 (001) and (110) substrates and found that the τ_c decreased to 300 K for the VO_2 film grown on TiO_2 (001), because the *c* axis was shortened due to the epitaxial stress, whereas the τ_c increased to 369 K for the VO_2 film grown on TiO_2 (110), because the *c* axis was elongated, as illustrated in Figure 4F.[115,116]

Recent computational simulations confirmed the combined impacts of strain and doping (or O-vacancies).[40,68,92] Cui et al. conducted first-principles calculations

Box 1. Summary of the Preparation Methods of VO_2 Particles

Category	Preparation Method	Advantage	Disadvantage	Particle Size	Particle Shape	Ref.
Solid phase reaction	thermal reduction	massive production and low cost	impurity of the product, rigid experimental conditions, and toxicity of V_2O_5	micro	rhombohedral	Qi et al.,[124] 2008
	thermolysis	massive production and low cost	poor stability of the product, aggregation of particle	micro	irregular	Zheng et al.,[122] 2000
Gas phase reaction	pulsed laser deposition	precise control of size and shape	rigid experimental conditions	micro/nano	rod	Rama and Ramachandra Rao,[120] 2000
	chemical vapor deposition	high crystallinity	low yield, dependence on the substrates	nano	wire	Kim et al.,[119] 2009
Liquid phase reaction	hydrothermal method	high crystallinity and low cost	difficulty in dispersing particles in matrix materials	nano	snowflake	Cao et al.,[125] 2008
	seeded growth strategy	controllability of crystal growth	–	nano	star	Whittaker et al.,[128] 2011
	precursor transformation	time-saving	low yield	–	–	Wu et al.,[130] 2011
	direct combustion	time-saving	low yield	micron/nano	irregular	Wu et al.,[131] 2010

"–" means data not available.

and identified that a 2% compressive strain on H-doped VO_2 (with a doping level of 1 at%) would decrease the τ_c by 51 K (Figure 4G).[92] Zhang et al. presented that the τ_c of Be-doped VO_2 could be further reduced by uniaxial strain, which is in close correlation with the dimerization of V-V chains in rutile VO_2 (Figure 4H).[68] Chen et al. found that a 2% compressive strain on pure VO_2 corresponds to a reduction in τ_c of 31.16 K, whereas the reduction increased to 56.74 K when the same compressive strain was loaded on the O-deficient VO_{2-x} (Figure 4I).[40]

Nano- and Microstructure of VO_2

Here the nano- and microscale morphology engineering approaches on VO_2 are elaborated with the aim of enhancing its thermochromic properties, namely achieving large T_{lum} and ΔT_{sol} simultaneously.

VO_2 Crystals

Monodisperse, nanosize, high-crystallinity VO_2(M/R) particles are more favorable because these particles can be dispersed in the aqueous solvent and be cast into films with good visible transmittance and regulation ability of infrared light. A number of methods, summarized in Box 1, have been developed to obtain VO_2(M/R) particles. Gas phase reactions, such as CVD,[119] pulsed laser deposition (PLD),[120,121] and solid phase reaction, such as thermolysis[122,123] and thermal reduction,[124] have long been regarded as the exclusive strategies to obtain VO_2 particles. For the gas or solid phase preparation methods of VO_2 particles, the reader is referred to the well-written review paper.[121] The experimental conditions of gas or solid phase reactions frequently require precisely scaled inert gas atmospheres, rigidly controlled temperatures, post-treatment, and long synthesis time. Over the past decade, a number of liquid phase reaction approaches have been reported, such as the hydrothermal method,[125–127] seeded growth method,[128] precursors transformation method,[129,130] and direct combustion method.[131] According to the transformation path, the methods to obtain VO_2(M/R) particles

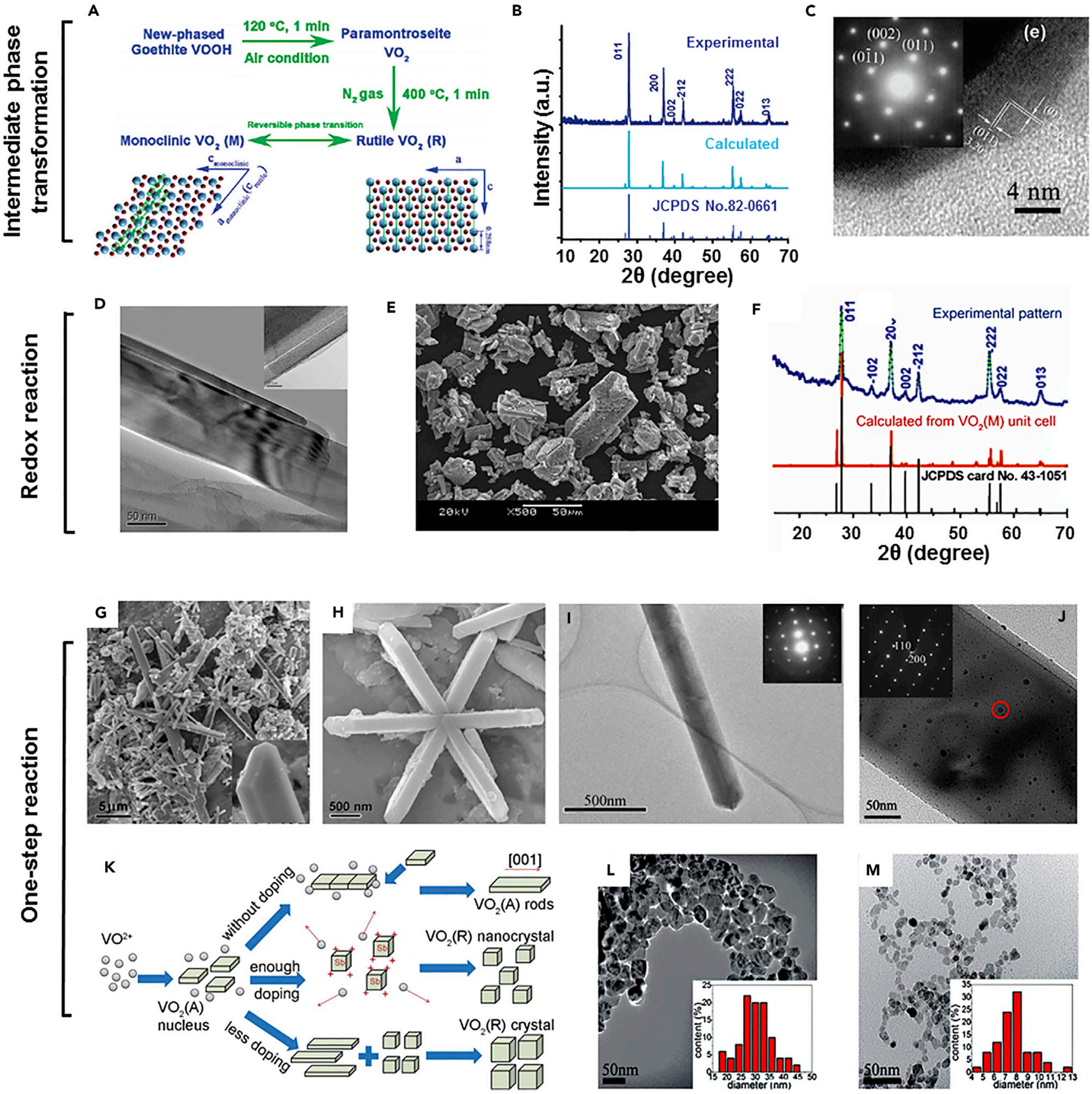

Figure 5. Different Transformation Paths to Obtain VO_2(M/R) Particles

(A) Illustration of the crystallographic transformation from the goethite VOOH to paramontroseite VO_2 to VO_2(M/R).

(B) Experimental, calculated, and standard (JCPDS card no. 82-0661) XRD patterns of VO_2(M) crystals.

(C) High-resolution transmission electron microscopy (HRTEM) image captured on the edge of a VO_2(M) nanoparticle and the corresponding and selected area electron diffraction (SAED) pattern (inset).

(D) HRTEM image of VO_2(M) nanosheets fabricated from V_2O_5 powder and absolute EtOH in a high-purity N_2 atmosphere.

(E) Scanning EM image of the VO_2(M) particles obtained by thermal reduction of V_2O_5 in ammonia gas.

(F) XRD pattern of the VO_2 particles obtained by direct confined-space combustion.

(G) Field-emission scanning EM images of the VO_2 powders.

(H) Snowflake-shaped single-crystal W-doped VO_2 nanoparticles synthesized through a one-step hydrothermal reaction.

(I) Transmission electron microscopy (TEM) image of a VO_2 nanorod and the corresponding SAED pattern (inset).

(J) TEM image and the SAED pattern (inset) of the VO_2 powders produced through the hydrothermal method at 260°C for 8 hr. The nanobump is indicated by the red circle.

Figure 5. *Continued*

(K–M) Schematic illustration of the evolution of Sb-doped VO_2 nanoparticles (K). TEM images of the VO_2 nanocrystals produced through the hydrothermal method at 260°C for 12 hr with 3% (L) and 40% (M) Sb^{3+} addition, respectively. Insets of (L) and (M) show the corresponding calculated size distributions.

Figures reproduced with permission from: (A) to (C), Wu et al.,[130] American Chemical Society; (D), Liu et al.,[139] Elsevier; (E), Qi et al.,[124] Elsevier; (F), Wu et al.,[131] Wiley; (G) and (H), Cao et al.,[125] American Chemical Society; (I) Ji et al.,[126] Elsevier; (J), Dai et al.,[127] Elsevier; (K), Gao et al.,[65] Royal Society of Chemistry.

are divided into intermediate phase transformations, redox reactions, and one-step reactions.

Intermediate phase transformation is the main strategy to obtain VO_2(M/R) particles before the discovery of one-step reaction. Most studies on intermediate phase transformation focus on the fabrication of VO_2(B) nanoparticles followed by thermal treatment to transform VO_2(B) to VO_2(R) at elevated temperature.[132–138] For instance, Kam et al. first synthesized VO_2(B) nanorods through the hydrothermal method and then obtained VO_2(M) nanorods by thermal treatment of the metastable VO_2(B) at 700°C in N_2 atmosphere.[133] In addition to the transformation from VO_2(B), Zhang et al. reported the synthesis of belt-like VO_2(M) particles through the transformation of VO_2(A) to VO_2(M).[137] Liu et al. discovered a new metastable phase, VO_2(D), which could guide the formation of VO_2(R/M) through a structural transition from VO_2(D) to VO_2(R).[138] Wu et al. reported a novel transformation pathway from the goethite VOOH to VO_2(P) to VO_2(R), with each step taking less than a minute, realizing an alternative ultrafast transformation to VO_2(M) (Figures 5A–5C).[130] The intermediate phase transformation method has two weaknesses. One is that the VO_2(M/R) particles obtained from intermediate phase transformation usually present the belt-like or rod-like morphologies, which are unfavorable for dispersion in the aqueous solvent. The other weakness is that the transformation typically requires a high temperature (>400°C) and a long transformation time to obtain the fully transformed VO_2(M/R) phase.

Redox reaction is another strategy to prepare VO_2(M/R) particles, and V_2O_5 is usually the raw material. V_2O_5 can be reduced to VO_2 by supplementary reducing agents (e.g., $H_2C_2CO_4$, amine, alcohols, and N_2H_4) in hydrothermal/solvothermal reactions[139–142] or in a reductive atmosphere during high-temperature annealing.[124] For instance, Liu et al. reported that the VO_2(M) nanosheets (Figure 5D) were obtained by the solvothermal reaction and subsequent heat treatment of the mixture of commercial V_2O_5 powder and absolute EtOH in a high-purity N_2 atmosphere.[139] Qi et al. employed the thermal reduction of V_2O_5 in ammonia gas to synthesize high-purity VO_2(M) particles (Figure 5E).[124] Chen et al. demonstrated a method to fabricate VO_2(R) particles through an electrochemical process using V_2O_5 as the cathode; after precisely controlling the electrical discharging currents, V^{5+} is reduced to V^{4+}, and VO_2(R) particles are obtained.[131,143] Wu et al. obtained VO_2(M) particles by directly combusting an ethanol solution containing $VO(acac)_2$ (ac = acetylacetonate) in a confined space,[131] which provided not only sufficient energy but also necessary reductive atmosphere to maintain the +4 valence state of vanadium, contributing to the formation of thermodynamically stable VO_2(R) (Figure 5F). The redox reaction method has two weaknesses: the impurity of the product and the toxicity of V_2O_5.

One-step reaction is regarded as an effective strategy to fabricate VO_2(M/R) particles with high crystallinity and at low cost. In 2008, Gao's group reported for the first time the synthesis of snowflake-shaped single-crystal W-doped VO_2 nanoparticles through a one-step hydrothermal reaction (Figures 5G and 5H).[125]

Later, Ji et al. found that H_2SO_4 could serve as a morphology control agent in the same reaction system, so that VO_2 nanorods could be obtained directly from the one-step hydrothermal reaction (Figure 5I), and these nanorods presented excellent thermochromic properties with decreased τ_c and narrowed hysteresis.[126] In 2011, Gao et al. extended their research and found that the τ_c was closely correlated with the sizes of VO_2(M) nanobumps with a critical size of 13.0 nm (Figure 5J).[127] Gao et al.[127] developed a new approach to modulate the τ_c in VO_2 systems by controlling the nanoparticle size. Although the one-step hydrothermal method proposed by Gao et al. was powerful and could be employed to prepare pure and thermodynamically stable VO_2(M) nanoparticles in large quantities, the prepared VO_2(M) particles displayed snowflake-like aggregation with oriented growth, hindering their dispersion in matrix materials. In 2012, Gao et al.[65] proposed a doping strategy to simultaneously control the morphologies and sizes of VO_2 nanoparticles. Using a one-step hydrothermal method, Sb-doped VO_2(M) nanoparticles with controllable sizes were prepared (Figures 5K–5M). These nanoparticles exhibited obvious phase-transition characteristics when dispersed in aqueous solvent, and the foils fabricated by casting the VO_2(M) nanoparticles illustrated outstanding optical properties.[65] Very recently, Ji et al. prepared uniform VO_2 nanoparticles by the one-step hydrothermal method, finding the tunability of emissivity of VO_2 nanoparticles in both mid- and far-IR thermal atmospheric windows for the first time.[144]

Nanocomposites Based on VO_2 Crystals

Simulation. In the continuous VO_2 thin films, the thickness needs to be increased to overcome the insufficient ΔT_{sol}, but at the cost of depressing the T_{lum}.[145] A promising way to tackle the trade-off between ΔT_{sol} and T_{lum} is by fabricating nanothermochromic composite, which can be achieved by embedding VO_2-based nanoparticles in a dielectric matrix. The reported simulated and experimental results concerning VO_2-based nanocomposites are summarized in Table 2.

This concept was first developed by Li et al., who demonstrated via calculations based on effective medium theory (EMT) that a system composed of well-dispersed VO_2 nanoparticles (reflective index of ε_p) in a dielectric host has advantages over continuous thin films.[146,147] Dilute composites composed of three types of VO_2 nanoparticles (spheres, ellipsoids, and core-shell) embedded in a dielectric matrix were considered (Figures 6A–6C), and the aspect ratio m is defined as a/c to describe the spheroidal geometry in the calculation. The selected refractive index of the matrix (ε_m) was similar to that of glass or a polymer. The composite thickness was set to 5 μm with a filling factor f of 0.01, which means that the effective thickness (including the VO_2 nanoparticles) is 0.05 μm, identical to one of the conditions calculated for the continuous films. They demonstrated than an increase in the aspect ratio leads to the higher $T(\lambda)$. Moreover, the highest $T(\lambda)$ for randomly oriented particles occurs when $m = 1$, i.e., when the particles have a spherical shape. Compared with continuous thin films (T_{lum} = 38% at the same effective thickness), the T_{lum} for spheres ($m = 1$) increased to ~72% and ~62% for the insulating and metallic states, respectively. Meanwhile, for spheroids, the ΔT_{sol} between the two phases is ~20%, while it is only ~7% for continuous films. For the core-shell structure (Figure 6C), ε_c and ε_p denote the dielectric functions of the core (with diameter x) and the VO_2 shell (with thickness t), respectively.[147] Calculations were done by varying the refractive indexes of the core (n_c) and changing the ratio of x/t. $T(\lambda)$ was found to decrease with an increase in x/t. The adverse effect of a high x/t ratio on T_{lum} and T_{sol} becomes more pronounced with increasing n_c. The largest ΔT_{sol} was 20.9%, observed for hollow nanospheres ($n_c = 1$) with $x/t = 10$, which is superior to that of solid nanospheres

Table 2. Summary of Experimental and Simulation Results of VO_2-Based Thermochromic Nanocomposites

Category	Matrix		Embedded Nanocrystals		T_{lum} (%)	ΔT_{sol} (%)	τ_c	Ref.
			Types	Dopant/Structures				
Simulation	non-responsive	–	spheroidal VO_2 particles		67.0	20.0	–	Li et al.,[146] 2010
		–	VO_2-based core-shell structures		59.0	20.9	–	Li et al.,[147] 2011
Experiment	non-responsive	PU	VO_2		45.6	22.3	—	Chen et al.,[148] 2104
			VO_2 and Sb-doped SnO_2		51.4	11.7	–	Gao et al.,[149] 2012
			doped VO_2	F	48.7	10.7	↓	Dai et al.,[71] 2013
				Mg	54.2	10.6	↓	Zhou et al.,[66] 2013
				Zr	60.4	14.1	↓	Shen et al.,[98] 2014
				W + Zr	56.4	12.3	↓	Shen et al.,[98] 2014
				W	56.0	12.7	↓	Chen et al.,[148] 2014
				Ti	53.0	17.2	—	Chen et al.,[72] 2013
			core-shell structure	VO_2-@-SiO_2	27.8	13.6	—	Gao et al.,[150] 2012; Zhou et al.,[151] 2013
				$V_xW_{1-x}O_2$-@-SiO_2	50.6	14.7	↓	Zhu et al.,[152] 2015
		Si-Al gel	VO_2		59.1	12.0	—	Liu et al.,[153] 2014
		PDMS	VO_2		85	–	—	Moot et al.,[154] 2016
		TiO_2	VO_2		61.2	14.6	—	Chen et al.,[155] 2014
	responsive	PNIPAm	VO_2		62.6	34.7	↓	Zhou et al.,[156] 2014; Zhou et al.,[157] 2015
		HPC	doped VO_2	W	56.0	36.0	↓	Yang et al.,[158] 2017
		IL-Ni-Cl	VO_2		55.2	26.5	–	Zhu et al.,[159] 2016
		CLETS	VO_2		59.2	20.8	–	Zhu et al.,[160] 2016
		NLETS	VO_2		71.0	18.2	–	Zhu et al.,[161] 2017

"—" means unchanged; "↓" means decrease; "–" means data not available. PNIPAm, poly(N-isopropylacrylamide); HPC, hydroxypropyl cellulose; CLETS, Co-based ligand exchange thermochromic system; IL-Ni-Cl, ionic liquid-nickel-chlorine complex; NLETS, Ni-based ligand exchange thermochromic system.

(~16.7%). However, T_{lum} shows a concomitant decrease from 73.5% to 59%. Moreover, they demonstrated that inverted core-shell structures constructed by an outermost shell with a refractive index ranging from 0 to 2.5 surrounding a VO_2 core without remarkable enhancement of thermochromic performance.

EMT does not consider light scattering by the nanoparticles, as the radii of the embedded nanoparticles are assumed to be substantially smaller than the wavelength, which is small enough to neglect light scattering. Hence, Laaksonen et al. further applied the four-flux method, a simplification of the multiple-scattering approach, together with EMT to determine the onset of light scattering.[162] In this approach, different fluxes in the forward and backward directions were collected to acquire the transmittance (direct and diffuse) and reflectance (specular and diffuse). The nanothermochromic properties of the VO_2-based nanocomposites were simulated for different-sized nanoparticles with radii of 5, 20, 50, and 100 nm.[162] The inference-free EMT showed good agreement with the four-flux theory when the radius of the embedded particles was less than 20 nm. As the nanoparticle size increased the scattering was enhanced, and the difference between the four-flux theory and EMT became obvious. Such significant light-scattering results in remarkable absorption and insufficient transmittance, which are not desired for applications of VO_2-based nanocomposites. Therefore, they concluded that VO_2 particles with sizes below or approximately 20 nm and fine crystallinity are crucial for applying VO_2-based nanocomposite coatings in practice.

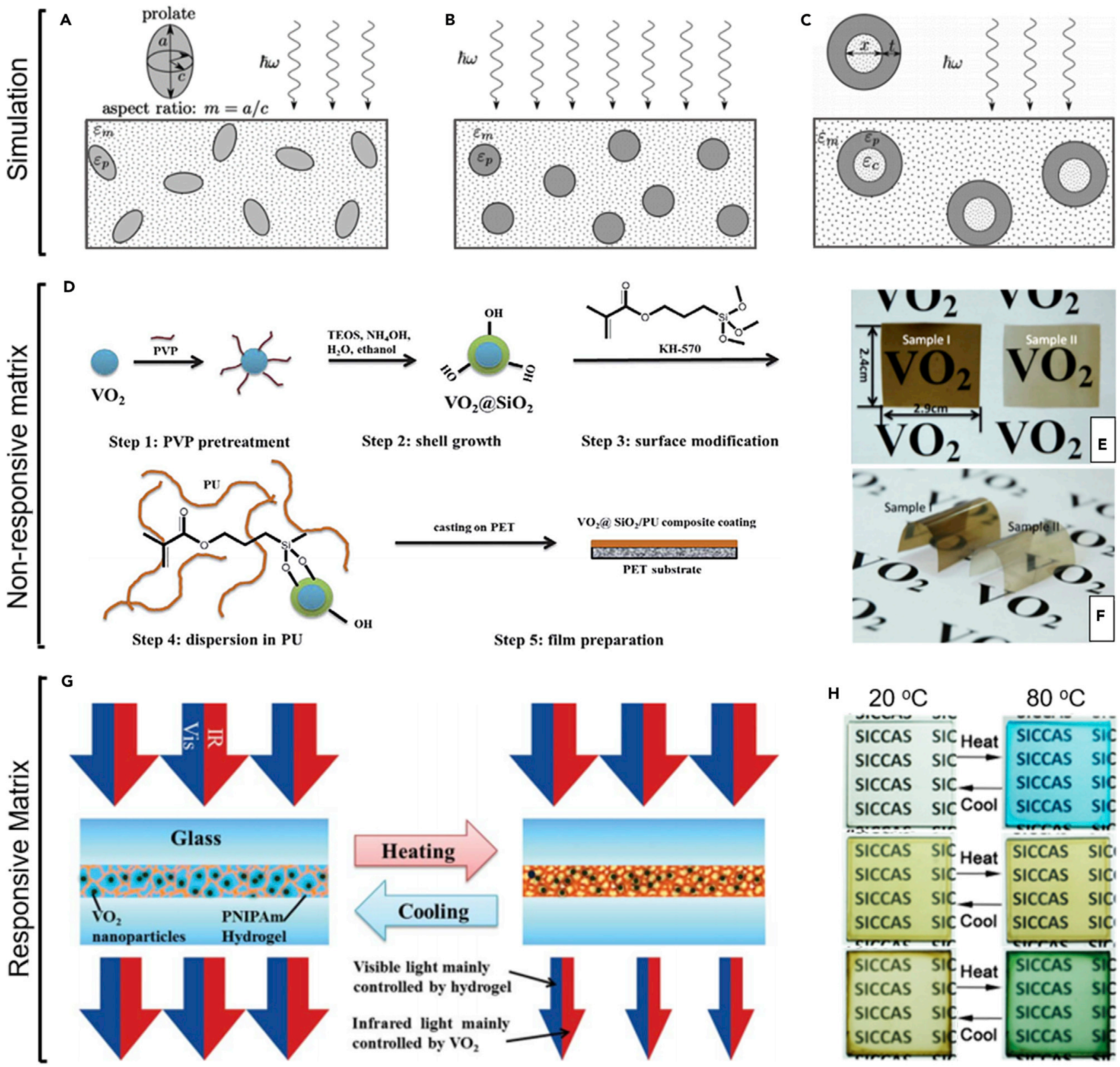

Figure 6. Nanocomposites Based on VO_2 Crystals to Enhance the Thermochromic Properties

(A–C) Structural models of composites embedded with nanocrystals having three different nanostructures: (A) ellipsoids, (B) spheres, and (C) core-shell nanoparticles. The dielectric functions of the matrix, nanoparticle, and core in the core-shell structure are denoted ε_m, ε_p, and ε_c, respectively. The aspect ratio is defined as m = a/c for ellipsoids and spheres (m = 1). The *x* and *t* indicate the core diameter and the shell thickness, respectively. The incident light is characterized as $\hbar\omega$.

(D) Scheme of the preparation procedure for the VO_2@SiO_2/PU composite, starting from VO_2 nanoparticles.

(E and F) Photographs of flexible thermochromic films based on VO_2@SiO_2/PU composites with high (Sample I) and low (Sample II) VO_2 contents.

(G) Illustration of the solar modulation behavior of the VO_2/PNIPAm-based composites. Both the visible and infrared light transmittance decreased when the films were heated.

(H) Photographs of the films based on the pure IL-Ni-Cl complex (top row), VO_2 nanoparticles (middle row), and VO_2/IL-Ni-Cl composite (bottom row) at 20°C (left column) and 80°C (right column), respectively. The IL-Ni-Cl complex and VO_2/IL-Ni-Cl composite have distinct temperature-responsive color changes.

Figures reproduced with permission from: (A) and (B), Li et al.,[146] American Institute of Physics; (C), Li et al.,[147] American Institute of Physics; (D) to (F), Gao et al.,[150] Royal Society of Chemistry; (G), Zhou et al.,[157] Royal Society of Chemistry; (H), Zhu et al.,[159] American Chemical Society.

Experiment

Non-responsive Matrix. Over the past decade, many methods for preparing VO_2 nanoparticles with fine crystallinity have been explored and implemented, followed by dispersion in various host materials, for practical applications of nanothermochromics. These host materials are classified as non-responsive or responsive matrixes, depending on whether they display distinct optical modulation during heating.

Polyurethane (PU) has been widely studied as a host material for VO_2-based thermochromic composites. The incorporation of pure VO_2 nanocrystals with PU was achieved by Chen et al., with excellent thermochromic properties observed.[148] Finely crystalline VO_2 nanoparticles with average diameters of 26 nm were prepared via a hydrothermal method based on the burst-nucleation process induced by precursor decomposition at a critical temperature. Through the assistance of polyvinylpyrrolidone (PVP), VO_2 nanoparticles were well dispersed in a PU matrix to form a visible homogeneous film. The composite displayed a much lower reflectance (below 8%) than the pure VO_2 coating on bare glass. The low reflectance, combined with the small size and high crystallinity of the VO_2 nanoparticles, revealed a remarkable ΔT_{sol} of 22% and an acceptable T_{lum} of 45.6% at low temperature and 40.0% at high temperature, which is very close to the highest simulation results (ΔT_{sol} = 23.7%). Shen et al. used a solid-state reaction to form aggregations of spherical VO_2 nanoparticles with a τ_c in the range of 43.5°C–59.3°C.[163] The reduction of τ_c may be due to the presence of an amorphous VO_2 phase surrounding the crystallized phases. However, their best-performing composite only showed a ΔT_{sol} of 9%. Gao et al. introduced the VO_2-PU composite to Sb-doped SnO_2 (ATO), a typical material that can largely block transmittance in the IR range while maintaining high transparency.[149] By optimizing the experiment, the ATO-VO_2-PU composite achieved ΔT_{sol} and T_{lum} comparable with that of the VO_2-PU foil without ATO. They further tested their applied properties in two model houses and demonstrated that the addition of ATO filler made the composite more effective at shielding IR transmittance than the VO_2-PU composite.

Embedding doped VO_2 nanoparticles into the PU matrix also achieved superior nanothermochromic properties. The primary reason for doping other elements into VO_2 nanoparticles is to reduce the τ_c from ~70°C to around room temperature. Upon substituting V atoms with F,[71] Mg,[66] W, or Zr atoms,[98] both VO_2(M) and VO_2(R) lattice structures are distorted due to structural defects induced by the dopants. Upon increasing the doping level, the structural difference between the high-temperature and low-temperature states decreases, which in turn leads to the reduction of the metal-semiconductor transition latent energy, therefore resulting in a decrease in the τ_c. An increase in dopant concentration usually deteriorates the solar modulation performance due to the appearance of lattice defects and morphological changes in the nanoparticles. In addition to Mg and W + Zr dopants, T_{lum} can be fairly maintained by F doping or slightly increased by introducing Zr into the VO_2 lattice. Among the dopants, F and W were able to lower the τ_c to a comfortable temperature of 35°C and 29°C, respectively, but large reduction in τ_c requires a high doping level at the cost of weakening the thermochromic performance. Although Ti doping fails to reduce τ_c, 1.1 at% Ti achieves the simultaneous improvement of T_{lum} (from 46% undoped to 53%) and ΔT_{sol} (from 13% undoped to 17%).[72] However, further increasing the Ti doping level does not further benefit the thermochromic properties. Dopant of Ti not only affects the thermochromic properties of VO_2 nanoparticles but also modifies the color of VO_2-based nanocomposite foils, as it widens the band gap of VO_2, inducing a blue shift in the absorption spectrum that lightens the original brownish-yellow color to a faded yellow color.

CellPress REVIEWS

The motivation for employing core-shell structures is to protect VO_2 nanoparticles from oxidation and maintain the outstanding nanothermochromic properties. The SiO_2 shell can act as a protective layer to restrain oxygen diffusion and prevent thermodynamically unstable VO_2 from being oxidized. Gao et al. successfully prepared VO_2-SiO_2 core-shell nanocrystals and integrated them in a PU matrix to produce flexible thermochromic foils.[150] The fabrication process is described in Figure 6D. VO_2 nanoparticles were sequentially treated by PVP and tetraethyl orthosilicate (TEOS) to obtain silica shells. To promote the dispersion of the core-shell structures in PU, they were further treated with a trace amount of silane coupler. The composite was cast on a polyethylene terephthalate (PET) substrate. As shown in Figures 6E and 6F, the films with high (Sample I) and low (Sample II) VO_2 content display excellent flexibility. Jin's group further explored core-shell structures by producing VO_2@SiO_2 nanorod structures or introducing W-doped VO_2-SiO_2 core-shell nanoparticles.[151,152] Both methods achieved decent performance, including high solar modulation and luminous transmittance, as well as improved stability. They suggested that the thermochromic performance benefited from surface plasmon resonance (SPR) and calculated the tunable SPR position by varying the filling factor and aspect ratio of the VO_2@SiO_2 nanorod structure. When 3 at% W is doped into VO_2 nanoparticles with SiO_2 shells to ensure excellent weatherability, the resulting $V_xW_{1-x}O_2$@SiO_2 nanocomposites, with their near-room-temperature transition, can achieve a T_{lum} of 49% and ΔT_{sol} of 15%. They noted that based on four-flux theory, a small particle size (~20 nm), good crystallinity, and good dispersion of nanocrystals in the composite are essential to achieve outstanding nanothermochromic properties. Ji et al. synthesized the VO_2@ZnS core-shell nanoparticles through the homogeneous precipitation method, finding that the VO_2 nanoparticle in the center exhibited tunable emissivity in the mid-wavelength and long-wavelength thermal atmospheric windows, whereas the ZnS shell, as an infrared transparent material, not only modified the color of VO_2 nanoparticle but also enhanced the oxidation resistance.[164]

Alternatively, Long's group adopted a transparent Si-Al gel as the host material. They utilized mechanical attrition (bead-milling) to obtain VO_2 nanoparticles without using a heating source.[153] The nanoparticles were well dispersed in the Si-Al gel and then coated on glass. An optimized thermochromic performance was achieved with a 3-μm-thick film containing 10 wt% VO_2. Following this work, the Si-Al gel/VO_2 composite was further developed to various micropatterned structures via a facile screen-printing method.[165] By optimizing the size of the mesh opening, the VO_2 load, and the film thickness, they demonstrated that the micropatterned structures simultaneously improved the ΔT_{sol} (8.8%) and T_{lum} (67%) over those of continuous films (ΔT_{sol} of 6.9% and T_{lum} of 60%) and the best preformed film had ΔT_{sol} of 14.9% combined with T_{lum} of 43.3%. An elastomeric matrix of polydimethylsiloxane (PDMS) was employed by Moot et al. to incorporate 10- to 200-nm VO_2 nanoparticles into stretchable composite films,[154] In addition, the film thickness could be easily reduced by stretching the films, which induces the formation of voids at the high-stress area and thus facilitates a significant enhancement in the T_{lum} from 45.6% to 55.4% as well as a slight increase in the IR modulation from 7.6% to 8.1% (due to the blue shift of the plasmon resonance spectral position).

Inorganic host materials, such as TiO_2, were explored by Gao's group.[155] They successfully fabricated inorganic-inorganic composite coatings by the two-step annealing of VO_2 nanoparticles in TiO_2 sol. In addition to acceptable thermochromic effects, these coatings showed additional advantages of self-cleaning, low contact angle, and photocatalytic decomposition of organic contaminants. These functions

introduced by the TiO_2 matrix improved the weatherability of VO_2-based smart windows.

Responsive Matrix. Although numerous advancements in VO_2-based non-responsive composites have improved the thermochromic performance, the best T_{sol} is still below 30%, which is limited by the spectral range that VO_2 can modulate. Solar energy is dense in the visible range, but VO_2 cannot regulate in this region due to the limited difference of the optical constants in spectrum range of 400–800 nm between its rutile and monoclinic phases. Incorporating a thermoresponsive host material with VO_2 has been proved to be a promising way to overcome this limitation.

Recently, Zhou et al. applied a thermoresponsive matrix to a VO_2-based composite for solar-energy modulation.[156,157] Pure poly-N-isopropylacrylamide (PNIPAm) is transparent below the lower critical solution temperature (LCST) and becomes translucent when the temperature increases, accompanied by a change in the NIR transmittance. In this research, phase separation of the PNIPAm hydrogel controlled the luminous modulation, while the phase transition of VO_2 nanoparticles contributed to the NIR modulation, providing an extremely high ΔT_{sol} of 35% and a high average T_{lum} of 60%. Figure 6G illustrates the formation of the laminated VO_2/hydrogel hybrid thin film and the mechanism to regulate the solar energy at different temperature. Compared with pure hydrogel of the same thickness, the VO_2/PNIPAm hybrid thin film had a slightly lower T_{lum} at both 20°C and 90°C due to the introduction of the VO_2 nanoparticles, but the IR modulation ability was dramatically increased, which led to the high ΔT_{sol}. The large contrast in the hybrid structure occurred in both the visible and NIR range, leading to a high solar modulation that is difficult to be reached by the non-responsive matrix. Later, Yang et al. developed a hydrogel based on hydroxypropyl cellulose (HPC).[158] Similar to PNIPAm, HPC becomes translucent during heating due to its LCST behavior. In addition to excellent thermochromic performance (ΔT_{sol} of 36.0% and T_{lum} of 56%), the prepared composite displayed a suitable τ_c of approximately 50°C by adjusting the τ_c of W-doped VO_2 nanoparticles and HPC. They suggested that the small liquid pores and aggregated polymeric blocks at elevated temperature could scatter the incident visible light and become translucent or opaque above the LCST.

In a different work, Jin's group combined VO_2 nanoparticles with a thermochromic ionic liquid, which typically increases the absorbance during heating over the range of 650–750 nm rather than over the whole visible range as the hydrogel does.[159] In the experiment, an ionic liquid-nickel-chlorine (IL-Ni-Cl) complex was observed to undergo a gradual color change from colorless to blue during heating. The VO_2/IL-Ni-Cl composite demonstrated outstanding optical regulation properties (ΔT_{sol} of 26.5%) and maintained good transparency (T_{lum} of 50%). More interestingly, the films exhibited a distinct color change from brown at 20°C to green at 80°C, which was believed to be a synergistic effect of the color variations in pure VO_2 and pure IL-Ni-Cl films (Figure 6H). This method provides an alternative way to simultaneously improve the unfavorable brownish-yellow color of VO_2 and achieve good solar-energy modulation. They then applied similar methods to cobalt(II)- and nickel(II)-based ligand exchange thermochromic systems (Co-based ligand exchange thermochromic system [CLETS] and Ni-based ligand exchange thermochromic system [NLETS][160,161]), from which similar results were obtained.

Porous VO_2 Films

The introduction of air-filled nanopores, which are considered to be a secondary component in VO_2 films, has been proved to be an effective method to improve

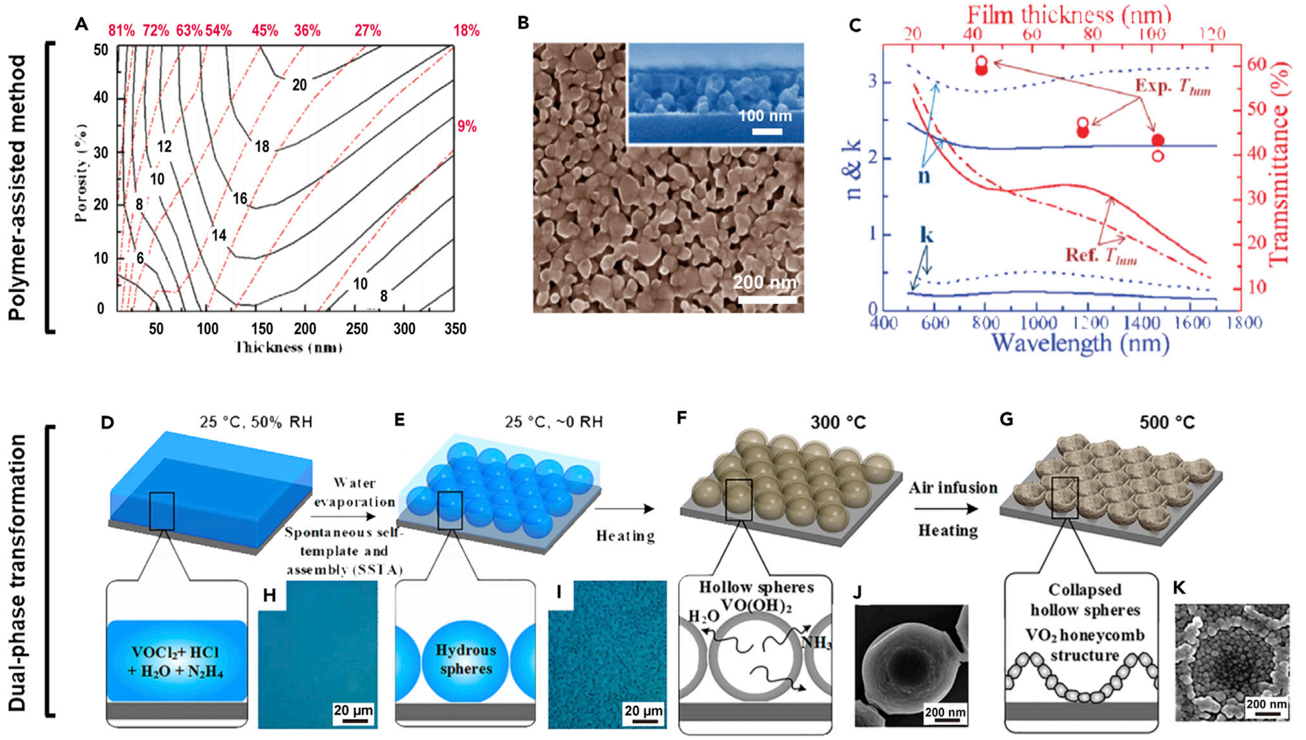

Figure 7. Porous VO_2 Films to Enhance the Thermochromic Properties

(A) Summary of the calculated thermochromic performance as a function of porosity and film thickness. $T_{lum,low}$ (visible transmittance of the porous VO_2 film at low temperature) and ΔT_{sol} are denoted by the red dotted lines and black solid lines, respectively.

(B) The porous morphology of a 147 nm-think VO_2 film, as an example, from the top and side view (inset).

(C) Optical properties of porous VO_2 films comparing with reference (non-porous VO_2 film). The optical constants (n and k) in experiment and reference are indicated as the solid and dotted blue lines. Experimental T_{lum} is recorded at 20°C (solid sphere) and 90°C (open circle). The reference of T_{lum} at 20°C (solid red line) and 90°C (dashed red line) are presented.

(D–K) Schematic of the self-templating and assembly during the dual-phase transformation process, which includes four steps: (D) deposition of a homogeneous solution-based precursor on a substrate, (E) assembly of self-templated hydrous sphere arrays, (F) formation of hollow $VO(OH)_3$ spheres, and (G) formation of the honeycomb-nanostructured VO_2 film via the collapse of hollow spheres. (H) and (I) are the corresponding photographs of steps (D) and (E), respectively. (J) and (K) are the scanning EM images presented as demonstrations of steps (F) and (G), respectively.

Figures reproduced with permission from: (A) to (C), Kang et al.,[170] American Chemical Society; (D) to (K), Liu et al.,[171] American Chemical Society.

the thermochromic performance, especially the T_{lum}. Gao et al. calculated the spectral transmittance of a nanoporous VO_2 film on a fused-silica-glass substrate with variable porosity and thickness by employing the optical-admittance recursive method, which is based on the optical-admittance function.[166–169] Based on the calculation, they plotted a useful diagram that correlates the thermochromic performance, including ΔT_{sol} and visible transmittance of porous VO_2 films at low temperature ($T_{lum,low}$), with the thickness and porosity of the film (Figure 7A). An optimizing thermochromic performance is demonstrated up to a ΔT_{sol} of 20% with a $T_{lum,low}$ of 45%. They suggested that ΔT_{sol} could be enhanced without a decrease in $T_{lum,low}$ because of the depression of the porosity-derived reflection. The progress of simulation and experimental research of porous VO_2 films are summarized in Table 3.

Incorporating removable additives into the vanadium precursor has been demonstrated to be a facile way to fabricate porous VO_2 films. Gao et al. pioneered the porous films and successfully produced nanoporous thermochromic VO_2 films by a polymer-assisted deposition method.[170] The pores in the final films are formed from PVP degradation and shrinkage of the gel films during high-temperature annealing (Figure 7B). These air-filled pores had feature sizes much smaller than the

Table 3. Summary of Porous VO_2 Films

Category	Preparation Method		Structure	Pore Size	ΔT_{sol}(%)	T_{lum} (%)	Ref.
Simulation	–		random porosity	nano	20	45	Kang et al.,[170] 2011
Experiment	incorporating removable additive	PVP	–	nano	14.1	43.3	Kang et al.,[170] 2011
		CTAV	hierarchical porous structure	nano/micro	–	46.5	Ding et al.,[172] 2013
		CTAB	–	nano	–	–	Xu et al.,[61] 2012
		PEG	–	nano	–	–	Xu et al.,[173] 2013
		SDS	–	nano	–	–	Xu et al.,[174] 2013
	process control	freeze-drying	–	nano	14.7	50.0	Cao et al.,[175] 2014
		sintering in CO_2 atmosphere	–	nano	2.2	35.9	Wang et al.,[176] 2013
	self-assembly	dual-phase transformation	quasi-honeycomb	nano	5.5	–	Liu et al.,[171] 2017
		crystallographic orientation control	interconnected nanonet	nano	–	–	Zhang et al.,[177] 2015

"–" means data not available.

visible wavelength lower the low optical constants (Figure 7C), resulting in a high luminous transmittance, T_{lum} = 43.3%, and solar modulation, ΔT_{sol} = 14.1%. A micro-/nanosized (20–50 μm/100–500 μm) hierarchical porous structure was developed by Huang et al. through the self-assembly of cetyltrimethylammonium vanadate (CTAV), which exhibited largely enhanced visible-light transmittance.[172] Later, surfactants such as cetyltrimethyl ammonium bromide (CTAB),[61] polyethylene glycol (PEG),[173] and SDS[174] were reported to successfully serve as nanostructure-directing agents for VO_2 films.

Instead of incorporating removable additives into the precursor, Long's group achieved porous VO_2 films by controlling the drying procedure via a lyophilization method (freeze-drying).[175] In their experiment the sols were frozen at low temperature and pressure, after which the solvent was removed via sublimation. This procedure can easily circumvent the collapsed pores induced by evaporation under atmospheric pressure. This group further explored the effect of gas effect during annealing.[176] When a CO_2 atmosphere was applied during sintering nanoporous VO_2 films were formed, and the crystallization temperature of VO_2 was reduced to 550°C from 750°C when sintering in vacuum.

A spontaneous self-templating and assembly process during dual-phase transformation was successfully applied by Liu et al. to prepare porous VO_2 structures.[171] In the experiment, an aqueous vanadium precursor including vanadyl dichloride (VCl_2), hydrazine (N_2H_4), hydrochloric acid (HCl), and PVP was spin-coated on a quartz substrate and then dried under nitrogen (Figure 7D). The formation of hydrous colloids was promoted by N_2H_4 but impeded by HCl. During the evaporation process, the promotional effect of N_2H_4 was enhanced, but the quenching effect of HCl weakened, forming hydrous colloidal spheres from the homogeneous precursor. These spheres self-assembled into a close-packed structure at the colloid-substrate interface when saturation was reached in the hydrous colloids (Figure 7E). Thereafter, the hollow sphere structures developed at 300°C and then collapsed and crystallized to VO_2 during annealing at 500°C (Figures 7F and 7G). These processes have been demonstrated experimentally (Figures 7H–7K). By optimizing the experimental conditions, a high transmission (95.4% at 700 nm) was achieved, accompanied by ΔT_{sol} = 5.5%, for a honeycomb VO_2 film with a thickness of 65 nm. In contrast to the self-assembly relying on dual-phase transformation, Zhang et al. prepared

Table 4. Summary of Grid-Structured VO_2 Films

Category	Preparation Method	Structure	ΔT_{sol} (%)	T_{lum} (%)	Ref.
Simulation	–	grid	14.0	76.5	Liu et al.,[178] 2015
		nanoparticle array	–	–	Ke et al.,[179] 2017
Experiment	electrodeposition	microgrid	13.9	38.4	Liu et al.,[180] 2017
	mesh printing	micropatterned	14.9	43.3	Lu et al.,[165] 2016
	nanosphere lithography	nanonet	7.9	–	Zhou et al.,[181] 2013
		nanonet	–	–	Ke et al.,[179] 2017
	modified nanosphere lithography	nanoparticle array	13.2	46.0	Ke et al.,[179] 2017
		nanodome array	–	–	Ke et al.,[179] 2017

" – " means data not available.

self-assembled VO_2 nanonets on a (001) single-crystal sapphire substrate by synchronously controlling the growth direction and crystallographic orientation.[177] The key is the lattice-matching between the low-surface-energy plane of VO_2 and the 3-fold symmetric (001) plane of sapphire, which induced the growth of (020) VO_2 on the (001) sapphire substrate followed by the growth of (001) VO_2 to suspended nanorods in three equivalent directions. The films were produced on the wafer scale and displayed dramatic fatigue endurance.

Grid VO_2 Films

Films based on grid-structured VO_2 have attracted increasing interest due to the great potential of structure-induced thermochromic enhancements, including but not limited to increased transmittance, antireflection, and localized surface plasmon resonance (LSPR). Previous studies, including simulation and experimental works, are summarized and classified in Table 4.

The group of Long demonstrated that nanogrid structure is able to significantly enhance the T_{lum} without deteriorating ΔT_{sol} and was demonstrated by 3D finite difference time domain (FDTD) method to numerically optimize a number of parameters, including structural models, cavity size, periodicity, film thickness, and fill factor.[178] In the simulation, three structural models are calculated: square holes in square lattices, circular holes in square lattices, and circular holes in hexagonal arrangement (Figure 8A). The thermochromic performance of square holes in square lattices is summarized in Figure 8B. Moreover, a competitive thermochromic performance (T_{lum} = 76.5% and ΔT_{sol} = 14.0%) was demonstrated on hexagonal-packed circular cells of VO_2 with a radius of 80 nm, periodicity of 160 nm, and thickness of 300 nm.

Grid VO_2 films with periodicity in microscale are processable and the same group has applied the electrodeposition method to successfully assemble the VO_2 nanoparticles to electrodes by tuning the ionic strength (Figure 8C).[180] Flexible thermochromic film was produced by depositing the VO_2 nanoparticles on to grid Cu/PET substrates, in which the nanoparticles assembled alone the grid Cu electrodes (Figure 8D). An optimized ΔT_{sol} of 13.9% and T_{lum} of 38.4% were demonstrated. Another facile method, the mesh printing method, was reported by Lu et al. to prepare micropatterned VO_2/Si-Al gel composites by mounting a mesh above a glass substrate with controllable distance (Figure 8E).[165] Films via the method display competitive

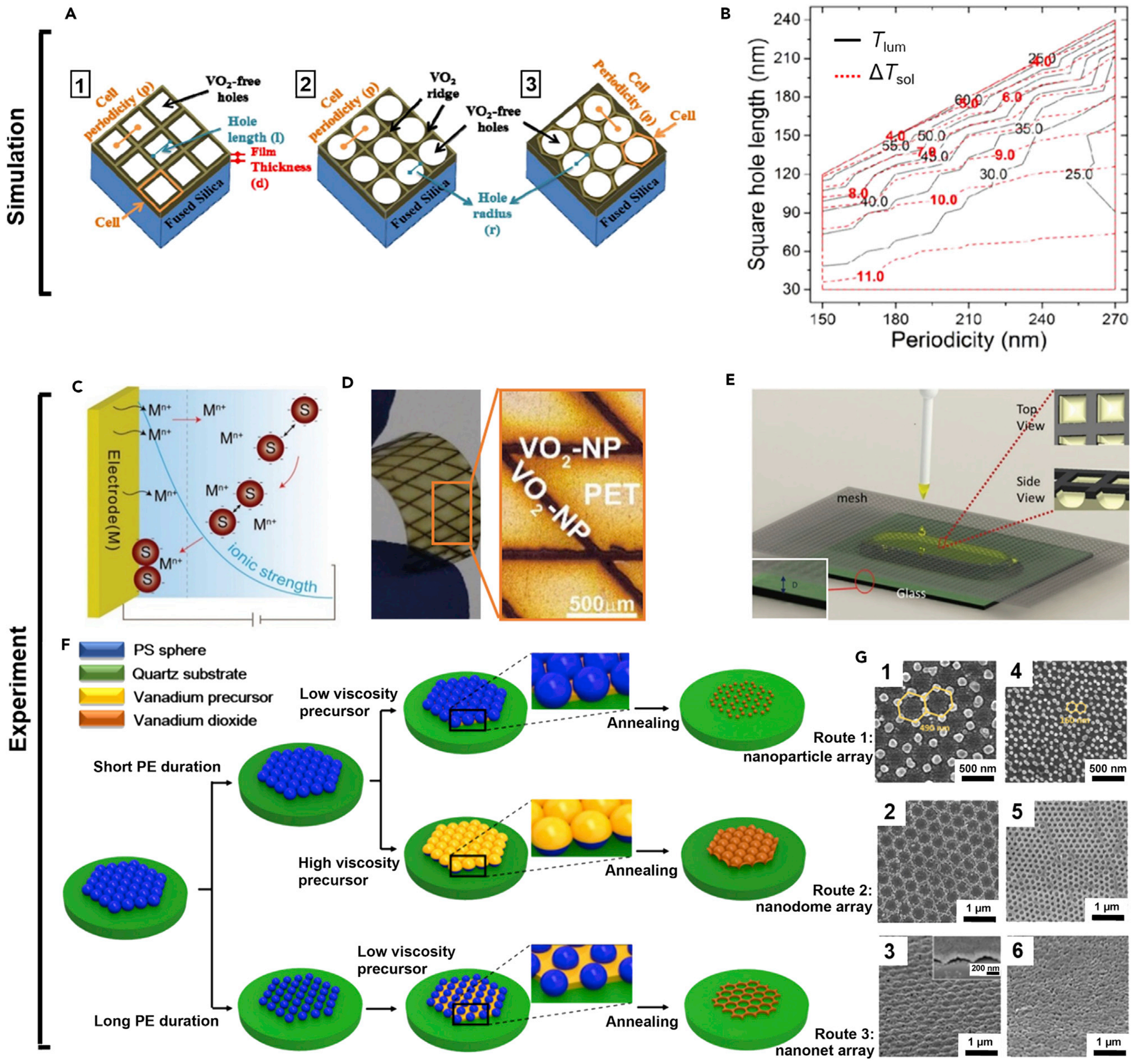

Figure 8. Films Based on Grid-Structured VO_2 to Enhance the Thermochromic Performance

(A) Simulation models of the grid films with square holes (a1) and circular holes (a2) in square lattices as well as the circular holes in hexagonal arrangement (a3).

(B) Summary of the calculated T_{lum} and ΔT_{sol} of the model with square holes in square lattice under various periodicity and square hole length.

(C) Schematic of the electrodeposition process based on ionic strength. The metal ions (M^{n+}) can reduce the interparticle repulsion among substances (S) and facilitate the S deposition.

(D) Photograph of the sample which contains VO_2 nanoparticles deposited on flexible grid Cu/PET film (left) and the optical microscopy image of the grid structure (right).

(E) Illustration of the mesh printing method, in which the thickness of sample is controlled by the distance (D) between the glass substrate and the mesh mounted above.

(F) Process of the template-assisted method for patterned VO_2 nanocrystals. Three types of the 2D periodic VO_2 nanocrystals, namely, nanoparticle, nanonet, and nanodome arrays, are derived from the nanosphere MCC by controlling the PE duration and the precursor viscosity through routes 1–3 in (A).

(G) Scanning EM images of periodic nanoparticle, nanonet, and nanodome VO_2 arrays with periodicities of 490 nm (1–3) and 160 nm (4–6). Photos 3 and 6 are the tilted-view scanning EM images. The periodicity can also be controlled to be the same as the nanosphere diameter of the MCC templates.

Figures reproduced with permission from: (A) and (B), Liu et al.,[178] The Optical Society; (C) and (D), Liu et al.,[180] Royal Society of Chemistry; (E), Lu et al.,[165] Royal Society of Chemistry; (F) and (G), Ke et al.,[179] American Chemical Society.

performance with a ΔT_{sol} of up to 14.9% under a T_{lum} of 43.3%. These two fabrication methods are facile, efficient, and controllable.

Preparation of grid films with periodicity in nanoscale is challenging. The colloidal nanolithography method was developed as a facile and productive method to prepare patterned nanostructures.[182] Xie's group applied monolayer colloidal crystal (MCC) templates made of polystyrene (PS) nanospheres to prepare periodic porous VO_2 films.[181] In their study, MCC templates consisted of closed-packed polystyrene nanospheres were sequentially immersed vanadium precursor, then removed during annealing to leave the VO_2 nanonet structures. Ke et al. modified the nanosphere lithography method and produced diverse patterned VO_2 films with tunable periodicity and nanostructures, including nanoparticle, nanonet, and nanodome arrays.[179] The fabrication process is flexible by controlling the plasma etching (PE) duration and precursor viscosity, illustrating as the synthetic routes in Figure 8F. When a short PE duration is applied, nanoparticle and nanodome arrays can be produced using low-viscosity (Route 1) and high-viscosity (Route 2) precursors, respectively. Nanonet arrays can be fabricated by prolonging the PE duration and using low-viscosity precursors (Route 3). The produced 2D patterned VO_2 arrays are highly uniform (Figure 8G). The patterned VO_2 films were further explored in thermochromic smart window and demonstrated an optimizing performance of ΔT_{sol} = 13.2% and T_{lum} = 46%. For the first time, hexagonally patterned VO_2 nanoparticle arrays with average diameters down to 60 nm and a periodicity of 160 nm were fabricated on the centimeter scale. Interestingly, such a structure gives rise to tunable peak positions and intensities of the LSPR at different temperatures. The LSPR was also found to red shift with an increase in the particle size and the reflective index of the media, and these results fit well with the trend calculated using the 3D FDTD method.

Biomimetic VO_2 Patterning

The usual properties of biomimetic structures have attracted great research interest, especially in light manipulation and the design of high-performance optics.[183] Recently, the integration of a bioinspired artificial surface with VO_2-based thermochromic smart windows was shown to have great potential through the simultaneous enhancement of ΔT_{sol} and T_{lum} as well as the efficient modification of the unfavorable brownish-yellow color of VO_2.

Moth-eye nanostructures can efficiently eliminate reflection because the sub-wavelength nipple array generates a continuous refractive index gradient between the air and the medium, effectively reducing the refractive index gap at the air-medium interface (Figures 9A and 9B).[184,185] Taylor et al. introduced the moth-eye structures to thermochromic VO_2-based intelligent glazing.[186] In their simulation, the VO_2-coated nipple arrays were designed to be hexagonal-close-packed (HCP) on a glass substrate (Figure 9C). The thermochromic performances were calculated by numerically optimizing these dimensions using the 3D FDTD method and are plotted in Figure 9D. As shown in Figure 9D, structures with heights lower than 500 nm are preferred, as these structures are reflective, especially in visible and dense solar-energy regions. A decent thermochromic performance of T_{lum} = 59.9% combined with ΔT_{sol} = 19.4% was revealed at point C in Figure 9D. VO_2 films with moth-eye structures were successfully prepared by Qian et al. by coating VO_2 onto fused silica substrates with moth-eye structures that were pre-fabricated via nanosphere lithography (Figures 9E and 9F).[187] The periodicity of the prepared films was precisely controlled to range from 210 to 1,000 nm. They found that the T_{lum} increases with decreasing periodicity, and moreover the sample with a periodicity of 210 nm simultaneously enhanced T_{lum} and ΔT_{sol} over those of the planar sample. Future research

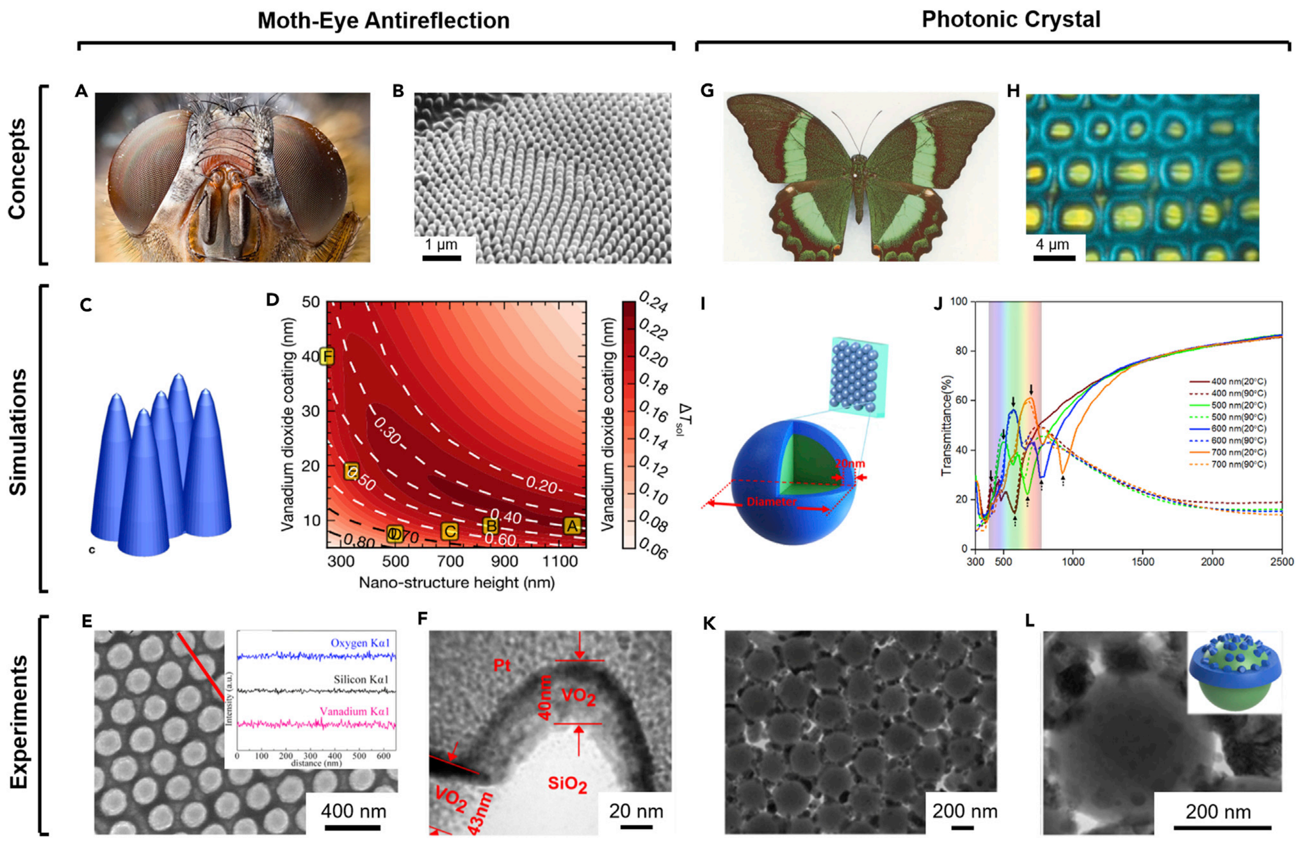

Figure 9. Integration of a Bioinspired Artificial Surface to Enhance the Thermochromic Performance
(A) Photograph of the compound eyes of Calliphora.
(B) The antireflection structures on the surface of its ommatidium.
(C) Three-dimensional illustration of the VO_2-coated nipple arrays used in the simulation and (D) the calculated ΔT_{sol} map based on the FDTD parameter search.
(E and F) Produced moth-eye nanostructured VO_2 films (E) containing the individual nipple structure of VO_2-coated silica (F).
(G and I) Photograph of a butterfly (G) and the two distinct structure-induced colors simultaneously appearing on its wings (H).
(I and J) Three-dimensional illustration of the 2D SiO_2-VO_2 core-shell photonic crystals used in the simulation (I) and the calculated transmittance spectra with diameters ranging from 400 to 700 nm at 20°C and 90°C (J).
(K and L) TEM images of the produced 2D HCP SiO_2-VO_2 core-shell photonic crystals under low and high magnification. Inset of (L) is the illustration of an individual photonic crystal.
Figures reproduced with permission from: (B), Vukusic and Sambles,[185] Nature Publishing Group; (C) and (D), Taylor et al.,[186] The Optical Society; (G) to (H), Vukusic and Sambles,[185] Nature Publishing Group; (I) to (L), Ke et al.,[188] American Chemical Society.

can be directed at reducing the periodicity, which may be a challenge for the fabrication of sub-100-nm patterns.

The opalescent or iridescent colors commonly observed in butterflies,[187] flora,[185] and so forth are produced by "photonic crystals," a concept first proposed in the 1980s (Figures 9G and 9H).[189] Photonic crystals are composed of periodically structured materials, generating a photonic band gap (PBG) and distinct structural colors by the coherent diffraction of visible light.[190] Ke et al. applied photonic crystals to VO_2-based smart windows to successfully modulate the unfavorable brownish-yellow color commonly observed for VO_2 films.[188] They prepared 2D HCP SiO_2-VO_2 core-shell structures on glass substrates with a fixed shell thickness of 20 nm (Figure 9I). As demonstrated by FDTD simulation, thermochromic films relaying on such photonic structures display statically diameter-dependent modulation in the visible range, while maintaining good thermochromic performance (up to

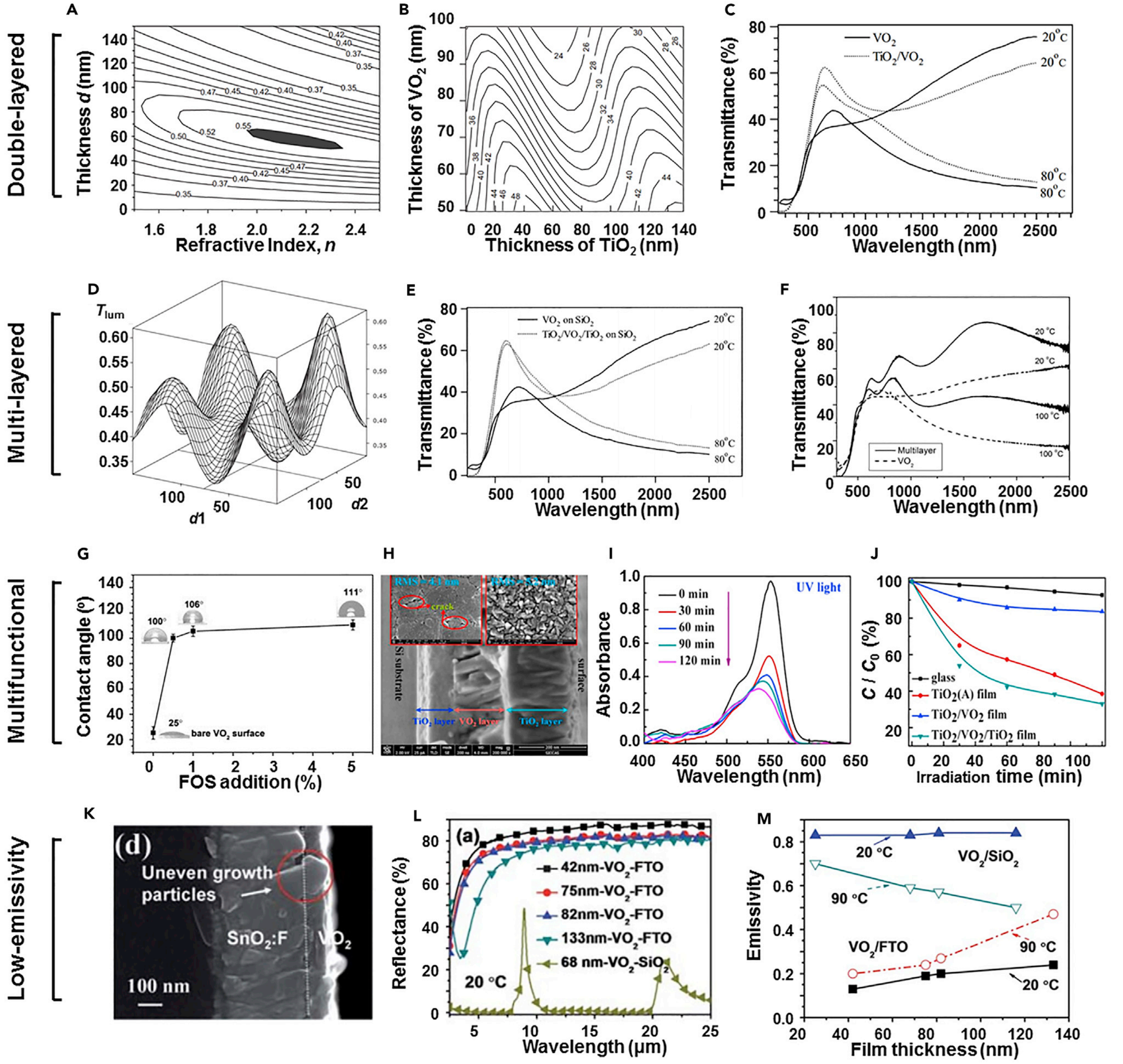

Figure 10. Multifunctional Antireflection Coating to Enhance the Thermochromic Performace of VO_2

(A) The effects of the refractive index *n* and thickness *d* of the ARC on the T_{lum} of planar 50 nm VO_2 films. The optimized *n* and *d* are presented as the dark area on the contour map.

(B) The thickness effect on the T_{lum} of TiO_2 and VO_2 on fused silica glass substrates.

(C) Respective transmittance spectra of 50 nm VO_2 films with or without an ARC (40 nm of TiO_2) at 20°C and 80°C.

(D) Calculated T_{lum} of the $TiO_2/VO_2/TiO_2$ layered structure, *d*1 and *d*2 are the thicknesses of the top and bottom TiO_2 layers, respectively.

(E) Transmittance spectra of a TiO_2 (20 nm)/VO_2 (50 nm)/TiO_2 (25 nm) sandwich structure at 20°C and 80°C.

(F) Transmittance spectra of a $TiO_2/VO_2/TiO_2/VO_2/TiO_2$ multilayer film at 20°C and 100°C. The results of the VO_2 films in (E) and (F) represent control samples.

(G) The effect of fluorooctyl triethoxysilane (FOS) addition on the contact angle on the VO_2 surface.

(H) Cross-sectional field-emission scanning EM (FESEM) image of the multilayer film (the insets are the surface morphology of VO_2(M) [left] and TiO_2(A) layers [right], respectively).

(I) Time-resolved absorption spectra of a rhodamine B (RhB) solution, which indicates the gradual degradation of RhB on the multilayer film.

(J) Photodegradation of RhB solution over the films (TiO_2(A), TiO_2(R)/VO_2(M) and TiO_2(R)/VO_2(M)/TiO_2(A)) under UV light (C_0 and *C* represent the initial and real-time concentration of RhB during the irradiation test).

Figure 10. *Continued*

(K) The cross-sectional FESEM image of a VO_2 film on an FTO (F-doped SnO_2) substrate.

(L) Thickness-dependent reflectance spectra of the VO_2 film on an FTO substrates at 20°C. The sample with a SiO_2 substrate is a reference.

(M) Thickness-dependent emissivity of the VO_2 film on an FTO substrate.

Figures reproduced with permission from: (A), Xu et al.,[196] Elsevier; (B) and (C), Jin et al.,[197] Japan Society of Applied Physics; (D) to (F), Jin et al.,[200] Elsevier; (G), Liu et al.,[192] Elsevier; (H) to (J), Zheng et al.,[201] Elsevier; (K) to (M), Zhang et al.,[199] Royal Society of Chemistry.

T_{lum} = 49.6% and ΔT_{sol} = 11.0%) (Figure 9J). The PBG-induced transmittance peaks and troughs were further verified experimentally with a variation in diameter, and structure-induced colors were observed to change from yellow-brown to red, blue, or green. However, the optimized ΔT_{sol} is limited to 3.1% in the experiment, much lower than the simulated result (11.0%). This difference is mainly attributed to the sol-gel method employed in the experiment, which resulted in half-coated spheres (Figures 9K and 9L) rather than perfect core-shell structures. The coating uniformity could be improved by using vapor deposition methods with different core materials and assembly.

Multifunctional Antireflection Coating

Challenges remain in the development of VO_2-based smart windows. A major issue is the low T_{lum} attributed to the strong reflection and absorption in the visible-light region (λ = 380–760 nm).[2] Antireflection coatings (ARCs) have been proved to be one of the effective strategies for enhancing the low T_{lum} in the visible region without degrading the thermochromic properties of the VO_2 films.[30,32] In addition, integrations with other cutting-edge glazing techniques can introduce some practical functions that are unable to be achieved by pure VO_2 films, such as antioxidation,[191] hydrophobicity,[192] and photocatalysis.[193]

The selection of ARC relies on the light interference between thin-film interfaces, which is determined by the optical constants and thicknesses of the ARC.[194] The refractive index (RI) of VO_2 is around 2.8 in the visible-light region (λ = 380–760 nm).[195] For a VO_2 film with a thickness of 50 nm, the highest enhancement in T_{lum} occurs when the thickness of the ARC is approximately 55 nm and the RI is approximately 2.2, as shown in Figure 10A.[196] According to this proposed refractive index criterion,[196] a variety of materials are potential candidates for the ARCs, such as TiO_2,[197] ZrO_2,[196] CeO_2,[198] and SnO_2.[199] Table 5 summarizes the T_{lum} and ΔT_{sol} of several VO_2-based multilayers before and after the application of an ARC.

For the VO_2-based double-layered films, TiO_2 is commonly selected as the ARC due to its RI of 2.2.[196] For instance, in the TiO_2(40 nm)/VO_2(50 nm) double-layered structure designed by Jin et al., the thicknesses of the TiO_2 and VO_2 layers were optimized by calculation to improve the T_{lum} of the TiO_2/VO_2 double-layer structure (from 30% to 49%) (Figures 10B and 10C).[197] Xu et al. fabricated a ZrO_2(56 nm)/VO_2(50 nm) double-layer structure by sputter deposition, in which the ZrO_2 layer acted as the ARC, showing that the T_{lum} was improved (from 32.3% to 50.5%).[196] Koo et al. prepared a CeO_2(60 nm)/VO_2(39 nm) double-layered structure, where the CeO_2 layer acted as the ARC due to its high RI (2.3) and high transparency to visible as well as NIR light.[198] The sample exhibited an obviously enhanced T_{lum} (from 40.0% to 67.5%), and the CeO_2 layer also acted as an antioxidation layer for the VO_2 layer.[198]

For the VO_2-based multilayered films, Jin et al. reported a TiO_2/VO_2/TiO_2 triple-layered structure with an elevated T_{lum} (from 30.9% to 57.6%) (Figures 10D and 10E).[200] However, this VO_2-based multilayered film exhibited a low ΔT_{sol} of 2.9%. This intrinsically low ΔT_{sol} (<16%) is due to the fact that VO_2 has a higher RI from

Table 5. The T_{lum} and ΔT_{sol} before and after the Application of Antireflection Coatings in Different Multilayered Structures

Layered Structure	ARC	T_{lum} (%) (without ARC)	T_{lum} (%) (with ARC)	ΔT_{sol} (%) (without ARC)	ΔT_{sol} (%) (with ARC)	Ref.
TiO_2/VO_2	TiO_2	32	49	4.4	7.0	Jin et al.,[197] 2002
ZrO_2/VO_2	ZrO_2	32.3	50.5	–	–	Xu et al.,[196] 2004
CeO_2/VO_2	CeO_2	40	67.5	5.2	5.4	Koo et al.,[198] 2014
TEOS/VO_2	TEOS	47.3	52.7	13.6	16.4	Liu et al.,[202] 2018
Si-Al/VO_2/ITO	Si-Al	51.0	62.3	3.4	4.0	Liu et al.,[192] 2013
$TiO_2/VO_2/TiO_2$	TiO_2	30.9	57.6	3.9	2.9	Jin et al.,[200] 2003
$TiO_2/VO_2/SiO_2$	TiO_2	40.3	61.5	7.4	6.9	Chen et al.,[203] 2011
TiO_2/VO_2/FTO	TiO_2	34	44	4.4	8.8	Zhang et al.,[199] 2011
SiO_2/Pt/VO_2	SiO_2	25.1	37.9	–	–	Kang et al.,[204] 2010

"–" means data not available. ARC, antireflection coatings.

500 to 2,200 nm wavelength below its τ_c, which causes excessive reflection at a lower temperature. Liu et al. designed an RI-tunable ARC coating to improve the antireflection effect at a lower temperature, thereby maximizing ΔT_{sol} for various VO_2 nanosubstrates, such as the continuous thin films, nanocomposites, and periodic micropatterning films.[202] The best-performing coatings could maximize ΔT_{sol} (from 15.7% to 18.9%) and increase T_{lum} (from 39% to 44%) simultaneously.[202] Chen et al. conducted a simulation to optimize the thickness of each layer in the $VO_2/TiO_2/SiO_2$ multilayers and then developed an all-solution method to fabricate the double-layered film consisting of a TiO_2 antireflection layer on a planar VO_2 film.[203] Their results showed that T_{lum} was enhanced from 40.3% to 61.5% and could be further enhanced to 84.8%; moreover, the value of ΔT_{sol} could be improved to 15.1%, which is much higher than the value of the single VO_2 films of 10%.[203] Other designs have also been reported. For instance, Mlyuka et al. prepared a $TiO_2/VO_2/TiO_2/VO_2/TiO_2$ five-layered structure with T_{lum} increased by 4% (from 41% to 45%) (Figure 10F).[205] However, the optical performances of structures with more than five layers have not been extensively investigated, probably due to the difficulties in both optical design and process control.

Some unique properties, such as antioxidation,[206,207] hydrophobicity,[187,192] and self-cleaning,[201] have been recently introduced by designing VO_2-based multilayered structures. For instance, Liu et al. prepared a Si-Al-based ARC with greatly enhanced T_{lum} (from 51.0% to 62.3%), hydrophobicity (contact angle of 111°) (Figure 10G), and antioxidation protections.[192] Zheng et al. reported a large-scale (400 × 400 mm^2) TiO_2(A)/VO_2(M)/TiO_2(R) multilayered film that presented at least three functions, antifogging/self-cleaning, thermochromic, and antireflective properties attributed to the top TiO_2(A), the middle VO_2(M), and the bottom TiO_2(R) layers, respectively (Figures 10H–10J).[201]

Thermal emissivity is another important property of VO_2-based smart windows. A high value of thermal emissivity implies that there is an intensive energy exchange between the window surface and its surroundings through thermal radiation and absorption, which weakens the thermal insulating ability of the window. To achieve smart functionality, a VO_2-based window should have suitable transmittance to control heat gain as well as low emissivity to modulate the heat loss. In cold weather, the heat flux is from indoors to outdoors, so a smart window should have high transmittance of heat from sunshine as well as low emissivity to prevent heat loss from indoors to outdoors. In hot weather, the direction of heat flux is opposite, from outdoors to indoors; therefore a smart window should transmit

Table 6. The Emissivity (ε_T) of VO_2-Based Multilayered Structures

Layered Structure	ε_T of VO_2(R)	ε_T of VO_2(M)	Thickness of VO_2 (nm)	Ref.
VO_2 single layer	0.59	0.83	68	Zhang et al.,[208] 2010
TiO_2/VO_2/FTO	0.24	0.13	55	Zhang et al.,[199] 2011
VO_2/FTO	0.27	0.19	65	Zhang et al.,[199] 2011
VO_2(W and Zn doped)/FTO	0.33	0.20	–	Du et al.,[210] 2013
VO_2/SiO_2/Au	0.71	0.22	30	Hendaoui et al.,[211] 2013
AZO/VO_2	0.31	0.32	40	Khang et al.,[206] 2011
Pt/VO_2	0.53	0.56	40	Khang et al.,[204] 2010

"–" means data not available.

less possible IR light as well as have low emissivity to prevent heat flow from outdoors to indoors.

The thermal emissivity is 0.59 and 0.83 for typical VO_2(R) and VO_2(M) films (thickness of 68 nm), respectively.[208] This high emissivity indicates the strong ability of the VO_2-based windows to exchange energy with its surroundings through thermal radiation processes. Recently, a number of papers have concerned the combination of the thermochromic properties of VO_2 and the low emissivity, in which transparent conductive oxides such as F-doped SnO_2 (FTO)[199] and Al-doped ZnO (AZO)[206] or noble metals (Ag[209] and Pt[204]) with low emissivity have been incorporated into VO_2-based multilayered films. Table 6 summarizes the emissivity of the VO_2 based multilayered structures. For instance, Zhang et al. deposited VO_2 thin films on FTO glasses substrates (Figure 10K), and then incorporated a TiO_2 ARC on the VO_2 thin films to form a TiO_2/VO_2/FTO three-layered structure, which boosted the T_{lum} (from 34% to 44%) and elevated the reflectance in the IR region while retaining the low-emissivity performance of the original VO_2/FTO double-layered structure (from 0.13 to 0.24) (Figures 10L–10M).[199] Kang et al. prepared $SiO_2/Pt/VO_2$ multilayered films, in which the Pt layer depressed the emissivity (from 0.85 to 0.56 for VO_2(M), and from 0.84 to 0.53 for VO_2(R)), and the SiO_2 layer acted as an ARC to enhance the T_{lum} (from 25.1% to 37.9%).[204]

Energy Efficiency

Practical energy conservation is the ultimate purpose of developing VO_2-based thermochromic layers, and researchers have been investigating this topic from both simulations and experimental aspects.

In the simulational studies, Saeli et al. firstly used energy-modeling studies to investigate the behavior of a series of VO_2 films and their associated energy consumptions (Figures 11A and 11B).[212,213] They compared three different VO_2 films: one is prepared via atmospheric pressure CVD, one is the commercial product (sputtered silver-coated glass or blue body-tinted glass), and the other is thermochromic films with "ideal" optical properties based on experimentally obtainable options. Their results indicated that the ideal coatings have a clear advantage in reducing energy consumption compared with the commercial products (Figure 11C), but the best performance of a real VO_2 film was observed for the sample with the lowest phase-transition temperature, which meant that the film was always in the metallic state. These results suggested that the heat reflecting and absorbing properties of the VO_2 layers contributed more strongly to their energy-conservation performance than their thermochromic nature. Increased

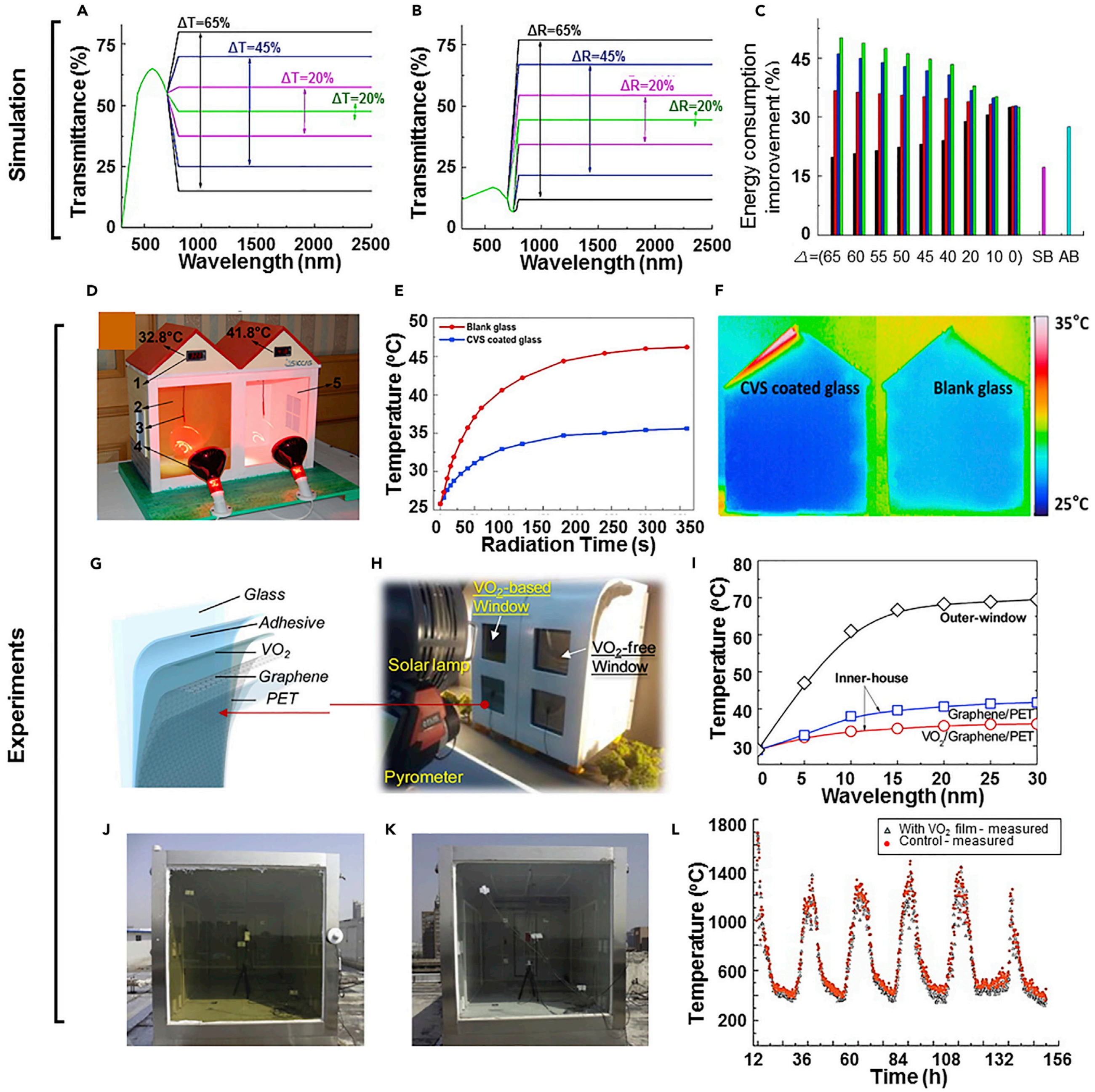

Figure 11. Practical Energy Efficiency Estimated from Simulations and Experimental Aspects

(A–C) Transmittance (A) and reflectance (A) spectra for ideal thermochromic coatings, demonstrating cold-hot decreases of 65%, 45%, 20%, or 0%, and energy consumption improvement (C) for ideal thermochromic films with various changes in transmittance and reflectance. Phase-transition temperature is 35°C (black), 30°C (red), 25°C (blue), 20°C (green); SB, sputtered silver-coated glass; AB, blue body-tinted glass.

(D) Photograph of a model house (1, temperature monitor; 2, VO_2 glass; 3, temperature probe; 4, infrared lamp; 5, blank float glass).

(E) Temperature curve inside the model house with $Cr_2O_3/VO_2/SiO_2$-coated glass (blue line) and blank glass (red line).

(F) Infrared thermal images of model house with $Cr_2O_3/VO_2/SiO_2$-coated glass and blank glass.

(G) Graphene-supported VO_2 film.

(H) Photograph of model house equipped with VO_2-based (VO_2/graphene/PET film) and VO_2-free window (graphene/PET film).

(I) Temperature change of model house upon solar irradiation as a function of exposure time.

(J and K) Photographs of Room A with VO_2 foils (J) and Room B with ordinary glazing (K).

(L) Variations in the simulated cooling load (August 1 to August 7, 2013).

Figures reproduced with permission from: (A) to (C), Saeli et al.,[213] Elsevier; (D), Gao et al.,[2] Elsevier; (E) and (F), Chang et al.,[214] Elsevier; (G) to (I), Kim et al.,[215]American Chemical Society; (J) to (L), Ye et al.,[216] Elsevier.

absorption might present an advantage for the moth-eye class of smart window, because the window's temperature will be strongly influenced by the light intensity and not merely the temperature. This behavior could endow the window with additional photochromic properties.

In the experimental studies, building the model houses is the common strategy to estimate the energy efficiency of VO_2-based smart windows. The VO_2-based layers are coated onto the flat glass and then fixed as windows or roofs for a model house. Blank glasses of the same size are used in another model house as a control experiment. Two IR lamps are employed as the irradiation source and placed at a certain distance from the model houses. Two thermocouples are placed at the same position in the model houses to monitor temperature changes. For instance, Gao et al. prepared a single-layer VO_2 structure (30×40 cm^2) on glass using the polymer-assisted deposition method, then built a model house to evaluate the optical properties of VO_2-based smart windows.[2] Their results indicated that under similar infrared irradiation, the temperature difference between the two inner rooms was approximately 9°C (Figure 11D), suggesting that a significant amount of irradiation was blocked. Chang et al. recently developed a sandwich structure of $Cr_2O_3/VO_2/SiO_2$ multilayers.[214] For quantitative characterization of their practical energy conservation, two pieces of flat glass (75×75 mm^2) coated with $Cr_2O_3/VO_2/SiO_2$ multilayer were roofed for a model house.[214] The results showed that the temperature in the model house with blank glass was increased by 84.8%, whereas the temperature of the house with $Cr_2O_3/VO_2/SiO_2$-coated glass was only increased by 29.8% (Figure 11E). The IR thermal imaging demonstrated that after 6 min of irradiation, the glass coated by $Cr_2O_3/VO_2/SiO_2$ structure showed a pink hue indicating high thermal emissivity. On the contrary, relatively low thermal emissivity has been shown by the laurel green hue of the blank glass (Figure 11F). Kim et al. prepared a graphene-supported VO_2 flexible film and then fabricated a model house with VO_2/graphene/PET windows to measure the inner-house temperature changes (Figures 11G and 11H).[215] The results showed that the temperature difference between two inner rooms (graphene/PET and VO_2/graphene/PET) was about 5.8°C (Figure 11I), indicating that a significant amount of irradiation was blocked by the graphene/VO_2 window.

Ye, Yang et al. evaluated the energy-saving efficiency of VO_2 layers in both theoretical simulations and experiments.[217,218] The VO_2 layers used in the experiments were VO_2 nanocomposite foils provided by Gao et. al. The researchers constructed a $2.9 \times 1.8 \times 1.8$-m^3 low-mass room with a window size of 1.65×1.65 m^2 (Figures 11J and 11K). The results measured by Yang et al. indicated that the room equipped with the VO_2 foils experienced a 10.2%–19.9% saving in cumulative cooling load compared with the room equipped with ordinary glazing. The extension of this observed performance to a conventional residential room in the hot-summer and warm-winter zone was simulated using BuildingEnergy (simulation software developed by Ye et al.). The simulated results indicated that the use of the VO_2 glazing could yield a saving of ~9.4% in electricity consumption (Figure 11L).

Ye et Al. have conducted a series of comprehensive investigations on the energy-saving performance of VO_2-based smart windows.[219–221] They found that not all VO_2 glazings are "smart" due to the absorption of the coatings, and concluded that the VO_2 glazing tends to exhibit smart regulation capacities if it experiences a high decrease in solar transmittance and a low increase in solar absorptivity after the VO_2 changes into its metallic state. In their other work of evaluating the energy-saving performance of materials and components in passive buildings, the

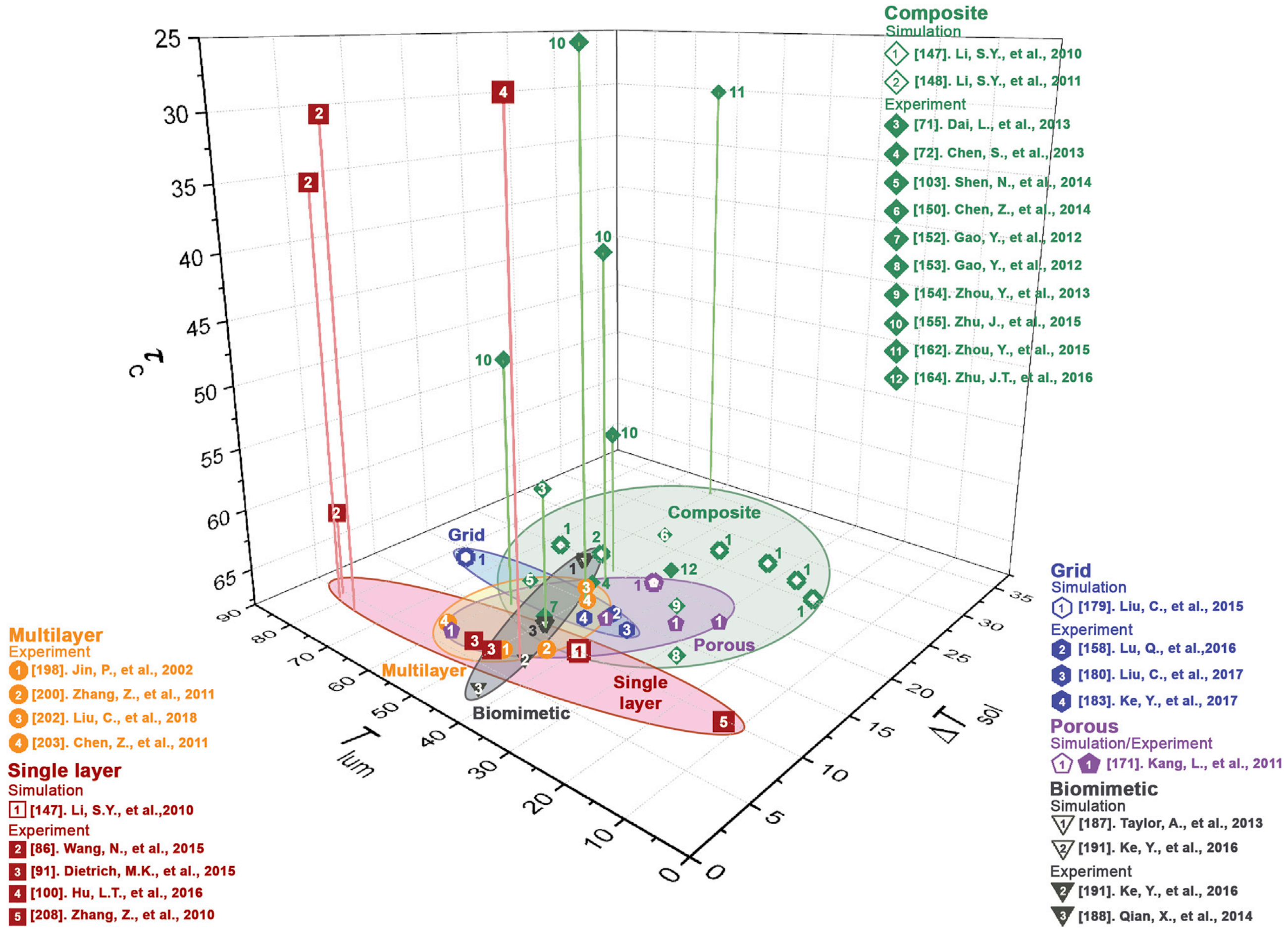

Figure 12. Summary of Thermochromic Performance (τ_c, ΔT_{sol}, and T_{lum}) in Some of the Best Reported and Selected Works

results showed that a representative VO_2 glazing is "energy-saving" in summer but is "energy-wasting" in winter due to its low solar transmittance. These studies reveal the importance of the transmittance and absorption of VO_2 film/coatings in practical energy-saving efficiency and provide a direction for the development of VO_2-based smart windows.

Conclusions and Perspectives

Smart windows represent an important technology for increasing indoor comfort and reducing electrical consumption in the automotive and building sectors. We summarize the progress in VO_2-based thermochromic smart windows in Figure 12 by classifying different categories and distinguishing the simulation and experiment results in terms of τ_c, ΔT_{sol}, and T_{lum}. Thermochromic windows are highly promising for constant T_{lum}, and smart regulation of indoor solar transmission automatically based on their achieved transmittance-modulation ranges together with the relatively simple structure, facile fabrication, and low cost. Although the ΔT_{sol} of VO_2 is lower than that of a gasochromic and electrochromic window, it can be further improved by integrating with responsive matrix and morphology engineering. Hence, VO_2 smart windows are an important category in energy-saving smart window applications. Indeed, flexible VO_2 foils fabricated by dispersing VO_2 nanoparticles into a matrix and coating of this VO_2-incorporated matrix onto a polymer substrate have been produced on the industrial scale.

The application of VO_2 thermochromic smart windows is promising and just beginning. Some problems still need to be addressed to further widen their applications.

A Balance between Transition Temperature, Visible Transmittance, and Solar Modulation Ability

Based on recent studies, the simultaneous reduction of τ_c, enhancement of ΔT_{sol} and T_{lum} is rather difficult, as shown in Figure 12. Low VO_2 loading is generally favorable for increasing the T_{lum} but jeopardizes ΔT_{sol}. Doping is undoubtedly an effective strategy to decrease the τ_c, but the other two parameters are typically degraded as well. Currently doping is limited in continuous films and nanocomposites (Figure 12); more investigation can be extended to other categories such as biomimetics, gridding, controlled porosity, and multilayered structure engineering. For the practical applications, more dopants and co- or multidoping need to be examined at the atomic level via the combination of simulations and experiments together with more structure designs at the nano- or microlevel.

It is worth mentioning that IR blocking in warm weather is an important aspect for energy-saving applications of thermochromic materials. For comparison, commercial low-emission glass with the application of three Ag layers can reach 90% blockage while VO_2 in state-of-art designs can reach less than 70%. ΔT_{sol} is another important index of energy saving that can scarcely reach up to 30%. To facilitate the commercialization of VO_2 films, ideally the IR blockage and ΔT_{sol} should be largely increased to 90% and 50%, respectively. However, this is a veritable challenge.

Color

Due to its strong absorption, VO_2 typically demonstrates a brownish-yellow color. The color has limited effects on the visible transmittance, and color preference is closely related to cultural background. Generally a colorless coating or coloration with light gray/blue is most favorable (at least in China). In some areas of Southeastern Asia or Arabia, a golden color is more favorable. In this case, the brownish-yellow color of VO_2 would be acceptable. However, certain modifications are still required to tune the color. Although some simulations have suggested that doping may be an effective strategy to alter the color by shifting the adsorption edges, experimental results have shown that only limited changes can be achieved. Complexion with dyes or responsive matrix, either organic or inorganic, can modulate the original color of VO_2 at the expense of the ΔT_{sol} and T_{lum} of VO_2 films or transparency, which may not be a good solution in some applications. Structural coloration has been proved effective for changing its color, but some degradation of the thermochromic performance and the complicated fabrication approach need to be investigated further. To address this concern, more creative ideas are needed.

Emissivity

Solar heating results from the NIR region of the solar spectrum, whereas black-body emissivity of room temperature objects occurs in the mid-IR regions. The former contributes to heat gains from the sun, while the latter is an important way to transmit heat from the hot side to the cold side. To achieve smart energy-saving functionality, a coating should have switchable transmittance to control heat gain from the sun as well as low emissivity to restrict the heat exchange between indoors and outdoors. VO_2 films, especially those porous films synthesized by solution-based process, usually demonstrate emissivity as large as 0.83, which is much larger than that of double-layered silver-based low-emission glass (0.3–0.4). This implies that glass coated with only VO_2 layers performs poorly in the management of environmental heat. Using a

Cell Press REVIEWS

double-layered film composed of VO_2 and a layer of noble metals or transparent conductive oxides can decrease the emissivity, accompanied unfavorably with a large decrease in the ΔT_{sol}. To resolve this problem, some new structures and/or material systems together with more fundamental studies should be developed and integrated with VO_2.

Stability

As a transition metal, vanadium is multivalance element, which exhibits +3, +4, +5, and mixed valences. VO_2(M) can be oxidized to vanadium oxides or their hydroxides, especially in the presence of moisture. This instability results in serious durability problems in practical applications. One solution involves the formation of VO_2 in an inert oxide shell, which is used to separate VO_2 from oxygen. This method has been certified to be useful to some degree, but the process to prepare a core-shell structure is not easy to control. Furthermore, this treatment step leads to the aggregation of well-dispersed nanoparticles, making them difficult to use. Increasing the crystallinity of VO_2 is also effective, but this method cannot completely address the problem.

Toxicity

The fate of nanoparticles in a variety of environmental and manufacturing settings has attracted immense attention and is a major obstacle for practical applications, especially in applications that have close contact with people and pets. Although some VO_2 products are commercially available (such as VO_2 foils in China), thorough research into the toxicological impact and possible hazards of VO_2 materials, especially in the form of nanoparticles, to human health and the environment is still in its infancy and is an urgent task in achieving large-scale applications of VO_2. Addressing this issue seems more meaningful when considering that some vanadium oxides known today, for example V(V) and V(III), are toxic, although the toxicity of these oxides is closely related to their quantity. Therefore, collaboration studies should be done on the mechanisms at the cellular level, entry routes into the body, and possible impacts on public health. In addition, life cycle assessment studies should be applied to analyze the toxicity of VO_2 nanoparticles.

ACKNOWLEDGMENTS

This work is supported by the Singapore Minster of Education (MOE) Academic Research Fund Tier One, RG124/16 and RG200/17, the National Research Foundation, Prime Minister's Office, Singapore under its Campus for Research Excellence and Technological Enterprise (CREATE) program, the National Natural Science Foundation of China (51325203, 51402182, 51702209, 51702208), the Ministry of Science and Technology of China (2016YFB0303901-05), and the Shanghai Municipal Science and Technology Commission (18JC1412800). To Ms. Chai. You called on environmental protection. We responded with science.

AUTHOR CONTRIBUTIONS

Y.C. wrote the sections Intrinsic Point Defects, Elemental Doping, Impacts of Strain on τ_c, Multifunctional Antireflection Coating, and Energy Efficiency. Y.K. wrote the Introduction, all but the last section of Nano- and Microstructure of VO_2, and Conclusions and Perspectives. Y.L. and Y.G. designed the framework of the manuscript and drafted the Introduction and the section Conclusions and Perspectives. C.L., Z.C., N.W., L.Z., Y.Z., and S.W. collected the data and participated in drawing the figures and examining the technical details. All authors read and approved the final manuscript.

REFERENCES

1. Morin, F.J. (1959). Oxides which show a metal-to-insulator transition at the Neel temperature. Phys. Rev. Lett. *3*, 34–36.
2. Gao, Y., Luo, H., Zhang, Z., Kang, L., Chen, Z., Du, J., Kanehira, M., and Cao, C. (2012). Nanoceramic VO_2 thermochromic smart glass: A review on progress in solution processing. Nano Energy *1*, 221–246.
3. Ke, Y., Zhou, C., Zhou, Y., Wang, S., Chan, S.H., and Long, Y. (2018). Emerging thermal-responsive materials and integrated techniques targeting the energy-efficient smart window application. Adv. Funct. Mater *28*, 1800113.
4. Cheng, Q., Paradis, S., Bui, T., and Almasri, M. (2010). Design of dual-band uncooled infrared microbolometer. IEEE Sens. J. *11*, 167–175.
5. Zhu, H., Xiao, C., Cheng, H., Grote, F., Zhang, X., Yao, T., Li, Z., Wang, C., Wei, S., and Lei, Y. (2014). Magnetocaloric effects in a freestanding and flexible graphene-based superlattice synthesized with a spatially confined reaction. Nat. Commun *5*, 3960.
6. Yoon, H., Choi, M., Lim, T.W., Kwon, H., Ihm, K., Kim, J.K., Choi, S.Y., and Son, J. (2016). Reversible phase modulation and hydrogen storage in multivalent VO_2 epitaxial thin films. Nat. Mater *15*, 1113–1119.
7. Andersson, G., Paju, J., Lang, W., and Berndt, W. (1954). Studies on vanadium oxides. I. Phase analysis. Acta Chem. Scand *8*, 1599–1606.
8. Andersson, G., Parck, C., Ulfvarson, U., Stenhagen, E., and Thorell, B. (1956). Studies on vanadium oxides. II. The crystal structure of vanadium dioxide. Acta Chem. Scand *10*, 623–628.
9. Longo, J.M., and Kierkegaard, P. (1970). Studies on vanadium oxides. Acta Chem. Scand *24*, 420–426.
10. Haverkort, M.W., Hu, Z., Tanaka, A., Reichelt, W., Streltsov, S.V., Korotin, M.A., Anisimov, V.I., Hsieh, H.H., Lin, H.J., Chen, C.T., et al. (2005). Orbital-assisted metal-insulator transition in VO_2. Phys. Rev. Lett. *95*, 196404.
11. Whittaker, L., Patridge, C.J., and Banerjee, S. (2011). Microscopic and nanoscale perspective of the metal-insulator phase transitions of VO_2: Some new twists to an old tale. J. Phys. Chem. Lett. *2*, 745–758.
12. Belozerov, A.S., Korotin, M.A., Anisimov, V.I., and Poteryaev, A.I. (2012). Monoclinic M1 phase of VO_2: Mott-Hubbard versus band insulator. Phys. Rev. B *85*, 276–279.
13. Guo, F., Chen, S., Chen, Z., Luo, H., Gao, Y., Przybilla, T., Spiecker, E., Osvet, A., Forberich, K., and Brabec, C.J. (2015). Printed smart photovoltaic window integrated with an energy-saving thermochromic layer. Adv. Opt. Mater *3*, 1524–1529.
14. Goodenough, J.B. (1971). The two components of the crystallographic transition in VO_2. J. Solid State Chem *3*, 490–500.
15. Eyert, V. (2011). VO_2: A novel view from band theory. Phys. Rev. Lett *107*, 016401.
16. Petrov, G.I., Yakovlev, V.V., and Squier, J. (2002). Raman microscopy analysis of phase transformation mechanisms in vanadium dioxide. Appl. Phys. Lett. *81*, 1023–1025.
17. Ishida, H., Bihlmayer, G., and Liebsch, A. (2005). Coulomb correlations and orbital polarization in the metal-insulator transition of VO_2. Phys. Rev. B *71*, 085109.
18. Rice, T.M., Launois, H., and Pouget, J.P. (1994). Comment on "VO_2: Peierls or Mott-Hubbard? A view from band theory". Phys. Rev. Lett. *73*, 3042.
19. Wentzcovitch, R.M., Schulz, W.W., and Allen, P.B. (1994). VO_2: Peierls or Mott-Hubbard? A view from band theory. Phys. Rev. Lett. *72*, 3389.
20. Dekorsy, T., Chong, H.H.W., Kieffer, J.C., Schoenlein, R.W., and Cavalleri, A. (2004). Evidence for a structurally-driven insulator-to-metal transition in VO_2: A view from the ultrafast timescale. Phys. Rev. B *70*, 161102.
21. Cavalleri, A., Tóth, C., Siders, C.W., Squier, J.A., Ráksi, F., Forget, P., and Kieffer, J.C. (2001). Femtosecond structural dynamics in VO_2 during an ultrafast solid-solid phase transition. Phys. Rev. Lett. *87*, 237401.
22. Rini, M., Chong, H.H.W., Fourmaux, S., Glover, T.E., Heimann, P.A., Kieffer, J.C., Schoenlein, R.W., and Cavalleri, A. (2005). Band-selective measurements of electron dynamics in VO_2 using femtosecond near-edge X-ray absorption. Phys. Rev. Lett *95*, 067405.
23. Baum, P., Yang, D.S., and Zewail, A.H. (2007). 4D visualization of transitional structures in phase transformations by electron diffraction. Science *318*, 788.
24. Wei, J., Wang, Z.H., Chen, W., and Cobden, D.H. (2009). New aspects of the metal-insulator transition in single-domain vanadium dioxide nanobeams. Nat. Nanotechnol *4*, 420–424.
25. Yuan, X., Zhang, W., and Zhang, P. (2013). Hole-lattice coupling and photoinduced insulator-metal transition in VO_2. Phys. Rev. B *88*, 035119.
26. ASTM. (2012) Standard tables of reference solar spectral irradiances: Direct normal and hemispherical on a 37° tilted surface. Active standard ASTM G173. https://www.astm.org/Standards/G173.htm.
27. Lang, F., Wang, H., Zhang, S., Liu, J., and Yan, H. (2018). Review on variable emissivity materials and devices based on smart chromism. Int. J. Thermophys *39*, 6.
28. Li, M., Magdassi, S., Gao, Y., and Long, Y. (2017). Hydrothermal synthesis of VO_2 polymorphs: Advantages, challenges and prospects for the application of energy efficient smart windows. Small *13*, 1701147.
29. Wu, C., Feng, F., and Xie, Y. (2013). Design of vanadium oxide structures with controllable electrical properties for energy applications. Chem. Soc. Rev. *42*, 5157–5183.
30. Wang, S., Liu, M., Kong, L., Long, Y., Jiang, X., and Yu, A. (2016). Recent progress in VO_2 smart coatings: Strategies to improve the thermochromic properties. Prog. Mater. Sci. *81*, 1–54.
31. Wang, S., Owusu, K.A., Mai, L., Ke, Y., Zhou, Y., Hu, P., Magdassi, S., and Long, Y. (2018). Vanadium dioxide for energy conservation and energy storage applications: Synthesis and performance improvement. Appl. Energy *211*, 200–217.
32. Granqvist, C.G., and Niklasson, G.A. (2017). Thermochromic oxide-based thin films and nanoparticle composites for energy-efficient glazings. Buildings *7*, 3.
33. Aschauer, U., Pfenninger, R., Selbach, S.M., Grande, T., and Spaldin, N.A. (2013). Strain-controlled oxygen vacancy formation and ordering in $CaMnO_3$. Phys. Rev. B *88*, 054111.
34. Marucco, J.F., Poumellec, B., and Lagnel, F. (1986). Stoichiometry of vanadium dioxide. J Mater. Sci. Lett. *5*, 99–100.
35. Chen, C., and Fan, Z. (2009). Changes in VO_2 band structure induced by charge localization and surface segregation. Appl. Phys. Lett. *95*, 262106.
36. Cui, Y., Liu, B., Chen, L., Luo, H., and Gao, Y. (2016). Formation energies of intrinsic point defects in monoclinic VO_2 studied by first-principles calculations. AIP Adv *6*, 105301.
37. Chen, L., Wang, X., Wan, D., Cui, Y., Liu, B., Shi, S., Luo, H., and Gao, Y. (2016). Tuning the phase transition temperature, electrical and optical properties of VO_2 by oxygen nonstoichiometry: Insights from first-principles calculations. RSC Adv *6*, 73070–73082.
38. Zhang, S., Kim, I.S., and Lauhon, L.J. (2011). Stoichiometry engineering of monoclinic to rutile phase transition in suspended single crystalline vanadium dioxide nanobeams. Nano Lett. *11*, 1443–1447.
39. Jiang, M., Cao, X., Bao, S., Zhou, H., and Jin, P. (2014). Regulation of the phase transition temperature of VO_2 thin films deposited by reactive magnetron sputtering without doping. Thin Solid Films *562*, 314–318.
40. Chen, L., Cui, Y., Shi, S., Liu, B., Luo, H., and Gao, Y. (2016). First-principles study of the effect of oxygen vacancy and strain on the phase transition temperature of VO_2. RSC Adv *6*, 86872–86879.
41. Zhang, P., Jiang, K., Deng, Q., You, Q., Zhang, J., Wu, J., Hu, Z., and Chu, J. (2015). Manipulations from oxygen partial pressure on the higher energy electronic transition and dielectric function of VO_2 films during a metal-insulator transition process. J. Mater. Chem. C *3*, 5033–5040.
42. Jeong, J., Aetukuri, N., Graf, T., Schladt, T.D., Samant, M.G., and Parkin, S.S.P. (2013). Suppression of metal-insulator transition in VO_2 by electric field-induced oxygen vacancy formation. Science *339*, 1402–1405.
43. Chen, S., Wang, X.J., Fan, L., Liao, G., Chen, Y., Chu, W., Song, L., Jiang, J., and Zou, C. (2016). The dynamic phase transition modulation of ion-liquid gating VO_2 thin film: Formation, diffusion, and recovery of

oxygen vacancies. Adv. Funct. Mater *26*, 3532–3541.

44. Marezio, M., McWhan, D.B., Remeika, J.P., and Dernier, P.D. (1972). Structural aspects of the metal-insulator transitions in Cr-doped VO_2. Phys. Rev. B *5*, 2541–2551.

45. Goodenough, J.B., and Hong, H.Y.P. (1973). Structures and a two-band model for the system $V_{1-x}Cr_xO_2$. Phys. Rev. B *8*, 1323–1331.

46. Pouget, J.P., Launois, H., Rice, T.M., Dernier, P., Gossard, A., Villeneuve, G., and Hagenmuller, P. (1974). Dimerization of a linear Heisenberg chain in the insulating phases of $V_{1-x}Cr_xO_2$. Phys. Rev. B *10*, 1801–1815.

47. Bruckner, W., Gerlach, U., Bruckner, H.P., Moldenhauer, W., and Oppermann, H. (1976). Influence of nonstoichiometry on the phase transitions in Ga-, Al-, and Fe-doped VO_2. Phys. Status Solidi. A *42*, 295–303.

48. Bruckner, W., Bruckner, H.P., Gerlach, U., Thuss, B., and Forsterling, G. (1976). The phase transition M1→T in $V_{1-x}Ga_xO_2$. Phys. Status Solidi. A *38*, K13–K16.

49. Tang, C., Georgopoulos, P., Fine, M.E., Cohen, J.B., Nygren, M., Knapp, G.S., and Aldred, A. (1985). Local atomic and electronic arrangements in $W_xV_{1-x}O_2$. Phys. Rev. B *31*, 1000–1011.

50. Khan, K.A., Niklasson, G.A., and Granqvist, C.G. (1988). Optical properties at the metal-insulator transition in thermochromic $VO_{2-x}F_x$ thin films. J. Appl. Phys. *64*, 3327.

51. Lee, M.H., Kim, M.G., and Song, H.K. (1996). Thermochromism of rapid thermal annealed VO_2 and Sn-doped VO_2 thin films. Thin Solid Films *290-291*, 30–33.

52. Burkhardt, W., Christmann, T., Meyer, B.K., Niessner, W., Schalch, D., and Scharmann, A. (1999). W- and F-doped VO_2 films studied by photoelectron spectrometry. Thin Solid Films *345*, 229–235.

53. Burkhardt, W., Christmann, T., Franke, S., Kriegseis, W., Meister, D., Meyer, B.K., Niessner, W., Schalch, D., and Scharmann, A. (2002). Tungsten and fluorine co-doping of VO_2 films. Thin Solid Films *402*, 226–231.

54. Pan, M., Zhong, H., Wang, S., Li, Z., Chen, X., and Lu, W. (2004). First-principle study on the chromium doping effect on the crystal structure of metallic VO_2. Chem. Phys. Lett. *398*, 304–307.

55. Mai, L.Q., Hu, B., Hu, T., Chen, W., and Gu, E.D. (2006). Electrical property of Mo-doped VO_2 nanowire array film by melting-quenching. J. Phys. Chem. B *110*, 19083–19086.

56. Piccirillo, C., Binions, R., and Parkin, I.P. (2007). Nb-doped VO_2 thin films prepared by aerosol-assisted chemical vapour deposition. Eur. J. Inorg. Chem. *2007*, 4050–4055.

57. Andreev, V.N., Kapralova, V.M., and Klimov, V.A. (2007). Effect of hydrogenation on the metal-semiconductor phase transition in vanadium dioxide thin films. Phys. Solid State *49*, 2318–2322.

58. Mlyuka, N.R., Niklasson, G.A., and Granqvist, C.G. (2009). Mg doping of thermochromic VO_2 films enhances the optical transmittance and decreases the metal-insulator transition temperature. Appl. Phys. Lett. *95*, 171909.

59. Wu, C., Feng, F., Feng, J., Dai, J., Peng, L., Zhao, J., Yang, J., Si, C., Wu, Z., and Xie, Y. (2011). Hydrogen-incorporation stabilization of metallic VO_2(R) phase to room temperature, displaying promising low-temperature thermoelectric effect. J. Am. Chem. Soc. *133*, 13798–13801.

60. Du, J., Gao, Y., Luo, H., Kang, L., Zhang, Z., Chen, Z., and Cao, C. (2011). Significant changes in phase-transition hysteresis for Ti-doped VO_2 films prepared by polymer-assisted deposition. Sol. Energy Mater. Sol. Cells *95*, 469–475.

61. Xu, Y., Huang, W., Shi, Q., Zhang, Y., Song, L., and Zhang, Y. (2012). Synthesis and properties of Mo and W ions co-doped porous nano-structured VO_2 films by sol-gel process. J. Solgel Sci. Technol. *64*, 493–499.

62. Wei, J., Ji, H., Guo, W., Nevidomskyy, A.H., and Natelson, D. (2012). Hydrogen stabilization of metallic vanadium dioxide in single-crystal nanobeams. Nat. Nanotechnol *7*, 357–362.

63. Tan, X., Yao, T., Long, R., Sun, Z., Feng, Y., Cheng, H., Yuan, X., Zhang, W., Liu, Q., Wu, C., et al. (2012). Unraveling metal-insulator transition mechanism of VO_2 triggered by tungsten doping. Sci. Rep *2*, 466.

64. Hu, S., Li, S.Y., Ahuja, R., Granqvist, C.G., Hermansson, K., Niklasson, G.A., and Scheicher, R.H. (2012). Optical properties of Mg-doped VO_2: Absorption measurements and hybrid functional calculations. Appl. Phys. Lett. *101*, 201902.

65. Gao, Y., Cao, C., Dai, L., Luo, H., Kanehira, M., Ding, Y., and Wang, Z.L. (2012). Phase and shape controlled VO_2 nanostructures by antimony doping. Energy Environ. Sci. *5*, 8708–8715.

66. Zhou, J., Gao, Y., Liu, X., Chen, Z., Dai, L., Cao, C., Luo, H., Kanahira, M., Sun, C., and Yan, L. (2013). Mg-doped VO_2 nanoparticles: Hydrothermal synthesis, enhanced visible transmittance and decreased metal-insulator transition temperature. Phys. Chem. Chem. Phys. *15*, 7505–7511.

67. Zhang, Y., Zhang, J., Zhang, X., Huang, C., Zhong, Y., and Deng, Y. (2013). The additives W, Mo, Sn and Fe for promoting the formation of VO_2(M) and its optical switching properties. Mater. Lett. *92*, 61–64.

68. Zhang, J., He, H., Xie, Y., and Pan, B. (2013). Giant reduction of the phase transition temperature for beryllium doped VO_2. Phys. Chem. Chem. Phys. *15*, 4687–4690.

69. Song, L., Zhang, Y., Huang, W., Shi, Q., Li, D., Zhang, Y., and Xu, Y. (2013). Preparation and thermochromic properties of Ce-doped VO_2 films. Mater. Res. Bull. *48*, 2268–2271.

70. Li, S.Y., Mlyuka, N.R., Primetzhofer, D., Hallen, A., Possnert, G., Niklasson, G.A., and Granqvist, C.G. (2013). Bandgap widening in thermochromic Mg-doped VO_2 thin films: Quantitative data based on optical absorption. Appl. Phys. Lett. *103*, 161907.

71. Dai, L., Chen, S., Liu, J., Gao, Y., Zhou, J., Chen, Z., Cao, C., Luo, H., and Kanehira, M. (2013). F-doped VO_2 nanoparticles for thermochromic energy-saving foils with modified color and enhanced solar-heat shielding ability. Phys. Chem. Chem. Phys. *15*, 11723–11729.

72. Chen, S., Dai, L., Liu, J., Gao, Y., Liu, X., Chen, Z., Zhou, J., Cao, C., Han, P., Luo, H., et al. (2013). The visible transmittance and solar modulation ability of VO_2 flexible foils simultaneously improved by Ti doping: An optimization and first principle study. Phys. Chem. Chem. Phys. *15*, 17537–17543.

73. Zhao, Y., Karaoglan, B.G., Pan, X., Holtz, M., Bernussi, A.A., and Fan, Z. (2014). Hydrogen-doping stabilized metallic VO_2(R) thin films and their application to suppress Fabry-Perot resonances in the terahertz regime. Appl. Phys. Lett. *104*, 241901.

74. Zhang, W., Wang, K., Fan, L., Liu, L., Guo, P., Zou, C., Wang, J., Qian, H., Ibrahim, K., Yan, W., et al. (2014). Hole carriers doping effect on the metal-insulator transition of N-incorporated vanadium dioxide thin films. J. Phys. Chem. C *118*, 12837–12844.

75. Zhang, J.J., He, H.Y., Xie, Y., and Pan, B.C. (2014). Boron-tuning transition temperature of vanadium dioxide from rutile to monoclinic phase. J. Chem. Phys. *141*, 194707.

76. Wu, Y., Fan, L., Chen, S., Chen, S., Chen, F., Zou, C., and Wu, Z. (2014). A novel route to realize controllable phases in an aluminum (Al^{3+})-doped VO_2 system and the metal-insulator transition modulation. Mater. Lett. *127*, 44–47.

77. Warnick, K.H., Wang, B., and Pantelides, S.T. (2014). Hydrogen dynamics and metallic phase stabilization in VO_2. Appl. Phys. Lett. *104*, 101913.

78. Sun, C., Yan, L., Yue, B., Liu, H., and Gao, Y. (2014). The modulation of metal-insulator transition temperature of vanadium dioxide: A density functional theory study. J. Mater. Chem. C *2*, 9283–9293.

79. Ren, Q., Wan, J., and Gao, Y. (2014). Theoretical study of electronic properties of X-doped (X = F, Cl, Br, I) VO_2 nanoparticles for thermochromic energy-saving foils. J. Phys. Chem. A *118*, 11114–11118.

80. Miyazaki, K., Shibuya, K., Suzuki, M., Wado, H., and Sawa, A. (2014). Correlation between thermal hysteresis width and broadening of metal-insulator transition in Cr- and Nb-doped VO_2 films. Jpn. J. Appl. Phys. *53*, 71102.

81. Li, S.Y., Niklasson, G.A., and Granqvist, C.G. (2014). Thermochromic undoped and Mg-doped VO_2 thin films and nanoparticles: Optical properties and performance limits for energy efficient windows. J. Appl. Phys *115*, 053513.

82. Hur, M.G., Masaki, T., and Yoon, D.H. (2014). Thermochromic properties of Sn, W Co-doped VO_2 nanostructured thin film deposited by pulsed laser deposition. J. Nanosci. Nanotechnol *14*, 8941–8945.

83. Cao, X., Wang, N., Magdassi, S., Mandler, D., and Long, Y. (2014). Europium doped vanadium dioxide material: Reduced phase

transition temperature, enhanced luminous transmittance and solar modulation. Sci. Adv. Mater *6*, 558–561.

84. Zhang, H., Wu, Z., Niu, R., Wu, X.F., He, Q., and Jiang, Y.D. (2015). Metal-insulator transition properties of sputtered silicon-doped and un-doped vanadium dioxide films at terahertz range. Appl. Surf. Sci. *331*, 92–97.

85. Wu, Y., Fan, L., Liu, Q., Chen, S., Huang, W., Chen, F., Liao, G., Zou, C., and Wu, Z. (2015). Decoupling the lattice distortion and charge doping effects on the phase transition behavior of VO_2 by Titanium (Ti^{4+}) doping. Sci Rep *5*, 9328.

86. Wang, N., Liu, S., Zeng, X.T., Magdassi, S., and Long, Y. (2015). Mg/W-codoped vanadium dioxide thin films with enhanced visible transmittance and low phase transition temperature. J. Mater. Chem. C *3*, 6771–6777.

87. Miller, M.J., and Wang, J. (2015). Influence of Na diffusion on thermochromism of vanadium oxide films and suppression through mixed-alkali effect. Mater. Sci. Eng. B *200*, 50–58.

88. Li, W., Ji, S., Qian, K., and Jin, P. (2015). Preparation and characterization of VO_2(M)-SnO_2 thermochromic films for application as energy-saving smart coatings. J. Colloid Interface Sci. *456*, 166–173.

89. He, X., Zeng, Y., Xu, X., Gu, C., Chen, F., Wu, B., Wang, C., Xing, H., Chen, X., and Chu, J. (2015). Orbital change manipulation metal-insulator transition temperature in W-doped VO_2. Phys. Chem. Chem. Phys. *17*, 11638–11646.

90. Gu, D., Sun, Z., Zhou, X., Guo, R., Wang, T., and Jiang, Y.D. (2015). Effect of yttrium-doping on the microstructures and semiconductor-metal phase transition characteristics of polycrystalline VO_2 thin films. Appl. Surf. Sci. *359*, 819–825.

91. Dietrich, M.K., Kramm, B.G., Becker, M., Meyer, B.K., Polity, A., and Klar, P.J. (2015). Influence of doping with alkaline earth metals on the optical properties of thermochromic VO_2. J. Appl. Phys. *117*, 185301.

92. Cui, Y., Shi, S., Chen, L., Luo, H., and Gao, Y. (2015). Hydrogen-doping induced reduction in the phase transition temperature of VO_2: A first-principles study. Phys. Chem. Chem. Phys. *17*, 20998–21004.

93. Wu, X., Wu, Z., Ji, C., Zhang, H., Su, Y., Huang, Z., Gou, J., Wei, X., Wang, J., and Jiang, Y. (2016). THz transmittance and electrical properties tuning across IMT in vanadium dioxide films by Al doping. ACS Appl. Mater. Interfaces *8*, 11842–11850.

94. Wu, X.F., Wu, Z.M., Liu, Z.J., Ji, C.H., Huang, Z.H., Su, Y.J., Gou, J., Wang, J., and Jiang, Y.D. (2016). Rebound effect of IMT properties by different doping form in Si-doped vanadium dioxide films. Appl. Phys. Lett. *109*, 111903.

95. Wilkinson, M., Kafizas, A., Bawaked, S.M., Obaid, A.Y., Al-Thabaiti, S.A., Basahel, S.N., Carmalt, C.J., and Parkin, I.P. (2013). Combinatorial atmospheric pressure chemical vapor deposition of graded TiO_2-VO_2 mixed-phase composites and their dual functional property as self-cleaning and photochromic window coatings. ACS Comb. Sci. *15*, 309–319.

96. Cui, Y., Wang, Y., Liu, B., Luo, H., and Gao, Y. (2016). First-principles study on the phase transition temperature of X-doped (X = Li, Na or K) VO_2. RSC Adv *6*, 64394–64399.

97. Hu, L.T., Tao, H.Z., Chen, G.H., Pan, R.K., Wan, M.N., Xiong, D.H., and Zhao, X.J. (2016). Porous W-doped VO_2 films with simultaneously enhanced visible transparency and thermochromic properties. J. Solgel Sci. Technol *77*, 85–93.

98. Shen, N., Chen, S., Chen, Z., Liu, X., Cao, C., Dong, B., Luo, H., Liu, J., and Gao, Y. (2014). The synthesis and performance of Zr-doped and W-Zr-codoped VO_2 nanoparticles and derived flexible foils. J. Mater. Chem. A *2*, 15087–15093.

99. Wang, N., Duchamp, M., Dunin-Borkowsk, R.E., Liu, S., Zeng, X., Cao, X., and Long, Y. (2016). Terbium-doped VO_2 thin films: Reduced phase transition temperature and largely enhanced luminous transmittance. Langmuir *32*, 759–764.

100. Wang, N., Shun, N.T.C., Duchamp, M., Dunin-Borkowski, R.E., Li, Z., and Long, Y. (2016). Effect of lanthanum doping on modulating the thermochromic properties of VO_2 thin films. RSC Adv *6*, 48455–48461.

101. Yuan, X., Zhang, Y., Abtew, T.A., Zhang, P., and Zhang, W. (2012). VO_2: Orbital competition, magnetism, and phase stability. Phys. Rev. B *86*, 235103.

102. Cyrot, M. (1972). Theory of Mott transition: Applications to transition metal oxides. J. Phys. (France) *33*, 125–134.

103. Zhang, J., He, H., Xie, Y., and Pan, B. (2013). Theoretical study on the tungsten-induced reduction of transition temperature and the degradation of optical properties for VO_2. J. Chem. Phys. *138*, 114705.

104. Li, D., Li, M., Pan, J., Luo, Y., Wu, H., Zhang, Y., and Li, G. (2014). Hydrothermal synthesis of Mo-doped VO_2/TiO_2 composite nanocrystals with enhanced thermochromic performance. ACS Appl. Mater. Interfaces *6*, 6555–6561.

105. Cui, Y., Cao, C., Chen, Z., Luo, H., and Gao, Y. (2017). Atomic and electronic structures of thermochromic VO_2 with Sb-doping. Comp. Mater. Sci. *130*, 103–108.

106. Liu, D., Cheng, H., Xing, X., Zhang, C., and Zheng, W. (2016). Thermochromic properties of W-doped VO_2 thin films deposited by aqueous sol-gel method for adaptive infrared stealth application. Infrared Phys. Technol *77*, 339–343.

107. Ladd, L.A., and Paul, W. (1969). Optical and transport properties of high quality crystals of V_2O_4 near the metallic transition temperature. Solid State Commun *7*, 425–428.

108. Arcangeletti, E., Baldassarre, L., Di Castro, D., Lupi, S., Malavasi, L., Marini, C., Perucchi, A., and Postorino, P. (2007). Evidence of a pressure-induced metallization process in monoclinic VO_2. Phys. Rev. Lett. *98*, 196406.

109. Marini, C., Arcangeletti, E., Di Castro, D., Baldassarre, L., Perucchi, A., Lupi, S., Malavasi, L., Boeri, L., Pomjakushina, E., Conder, K., et al. (2008). Optical properties of $V_{1-x}Cr_xO_2$ compounds under high pressure. Phys. Rev. B *77*, 235111.

110. Marini, C., Bendele, M., Joseph, B., Kantor, I., Mitrano, M., Mathon, O., Baldini, M., Malavasi, L., Pascarelli, S., and Postorino, P. (2014). Probing the electronic and local structural changes across the pressure-induced insulator-to-metal transition in VO_2. EPL *108*, 36003.

111. Okuyama, D., Nakano, M., Takeshita, S., Ohsumi, H., Tardif, S., Shibuya, K., Hatano, T., Yumoto, H., Koyama, T., Ohashi, H., et al. (2014). Gate-tunable gigantic lattice deformation in VO_2. Appl. Phys. Lett *104*, 023507.

112. Cao, J., Ertekin, E., Srinivasan, V., Fan, W., Huang, S., Zheng, H., Yim, J.W.L., Khanal, D.R., Ogletree, D.F., Grossmanan, J.C., et al. (2009). Strain engineering and one-dimensional organization of metal-insulator domains in single-crystal vanadium dioxide beams. Nat. Nanotechnol *4*, 732–737.

113. Case, F.C. (1988). The influence of substrate temperature on the optical properties of ion-assisted reactively evaporated vanadium oxide thin films. J. Vac. Sci. Technol. A *6*, 2010–2014.

114. Jin, P., Yoshimura, K., and Tanemura, S. (1997). Dependence of microstructure and thermochromism on substrate temperature for sputter-deposited VO_2 epitaxial films. J. Vac. Sci. Technol. A *15*, 1113–1117.

115. Muraoka, Y., and Hiroi, Z. (2002). Metal-insulator transition of VO_2 thin films grown on TiO_2 (001) and (110) substrates. Appl. Phys. Lett. *80*, 583–585.

116. Muraoka, Y., Ueda, Y., and Hiroi, Z. (2002). Large modification of the metal-insulator transition temperature in strained VO_2 films grown on TiO_2 substrates. J. Phys. Chem. Solids *63*, 965–967.

117. Wu, J., Gu, Q., Guiton, B.S., de Leon, N.P., Lian, O., and Park, H. (2006). Strain-induced self organization of metal-insulator domains in single-crystalline VO_2 nanobeams. Nano Lett. *6*, 2313–2317.

118. Chang, S., Park, J.B., Lee, G., Kim, H.J., Lee, J., Bae, T., Han, Y., Park, T.J., Huh, Y.S., and Hong, W. (2014). In situ probing of doping- and stress-mediated phase transitions in a single-crystalline VO_2 nanobeam by spatially resolved Raman spectroscopy. Nanoscale *6*, 8068–8074.

119. Kim, M.H., Lee, B., Lee, S., Larson, C., Baik, J.M., Yavuz, C.T., Seifert, S., Vajda, S., Winans, R.E., Moskovits, et al.. (2009). Growth of metal oxide nanowires from supercooled liquid nanodroplets. Nano Lett. *9*, 4138–4146.

120. Rama, N., and Ramachandra Rao, M.S. (2010). Synthesis and study of electrical and magnetic properties of vanadium oxide micro and nanosized rods grown using pulsed laser deposition technique. Solid State Commun *150*, 1041–1044.

121. Nag, J., and Haglund, R.F., Jr. (2008). Synthesis of vanadium dioxide thin films and nanoparticles. J. Phys.Condens. Matter *20*, 264016.

122. Zheng, C.M., Zhang, J.L., Luo, G.B., Ye, J.Q., and Wu, M.M. (2000). Preparation of vanadium dioxide powders by thermolysis of a precursor at low temperature. J. Mater. Sci. *35*, 3425–3429.

123. Peng, Z., Jiang, W., and Liu, H. (2007). Synthesis and electrical properties of tungsten-doped vanadium dioxide nanopowders by thermolysis. J. Phys. Chem. C *111*, 1119–1122.

124. Qi, J., Ning, G., and Lin, Y. (2008). Synthesis, characterization, and thermodynamic parameters of vanadium dioxide. Mater. Res. Bull. *43*, 2300–2307.

125. Cao, C., Gao, Y., and Luo, H. (2008). Pure single-crystal rutile vanadium dioxide powders: Synthesis, mechanism and phase-transformation property. J. Phys. Chem. C *112*, 18810–18814.

126. Ji, S., Zhao, Y., Zhang, F., and Jin, P. (2010). Direct formation of single crystal VO_2(R) nanorods by one-step hydrothermal treatment. J. Cryst. Growth *312*, 282–286.

127. Dai, L., Cao, C., Gao, Y., and Luo, H. (2011). Synthesis and phase transition behavior of undoped VO_2 with a strong nano-size effect. Sol. Energy Mater. Sol. Cells *95*, 712–715.

128. Whittaker, L., Velazquez, J.M., and Banerjee, S. (2011). A VO-seeded approach for the growth of star-shaped VO_2 and V_2O_5 nanocrystals: Facile synthesis, structural characterization, and elucidation of electronic structure. CrystEngComm *13*, 5328–5336.

129. Zhou, Y., Ji, S., Li, Y., Gao, Y., Luo, H., and Jin, P. (2014). Microemulsion-based synthesis of $V_{1-x}W_xO_2$@SiO_2 core-shell structures for smart window applications. J. Mater. Chem. C *2*, 3812–3819.

130. Wu, C., Feng, F., Feng, J., Dai, J., Yang, J., and Xie, Y. (2011). Ultrafast solid-state transformation pathway from new-phased goethite VOOH to paramontroseite VO_2 to rutile VO_2(R). J. Phys. Chem. C *115*, 791–799.

131. Wu, C., Dai, J., Zhang, X., Yang, J., Qi, F., Gao, C., and Xie, Y. (2010). Direct confined-space combustion forming monoclinic vanadium dioxides. Angew. Chem. Int. Ed *49*, 134–137.

132. Xu, C.L., Ma, X., Liu, X., Qiu, W.Y., and Su, Z.X. (2004). A novel reduction-hydrolysis method of preparing VO_2 nanopowders. Mater. Res. Bull. *39*, 881–886.

133. Kam, K.C., and Cheetham, A.K. (2006). Thermochromic VO_2 nanorods and other vanadium oxides nanostructures. Mater. Res. Bull. *41*, 1015–1021.

134. Zhang, K., Liu, X., Su, Z., and Li, H. (2007). VO_2(R) nanobelts resulting from the irreversible transformation of VO_2(B) nanobelts. Mater. Lett. *61*, 2644–2647.

135. Yamamoto, S., Kasai, N., and Shimakawa, Y. (2009). Preparation of monodisperse and spherical rutile VO_2 fine particles. Chem. Mater *21*, 198–200.

136. Whittaker, L., Zhang, H., and Banerjee, S. (2009). VO_2 nanosheets exhibiting a well-defined metal-insulator phase transition. J. Mater. Chem. *19*, 2968–2974.

137. Zhang, Y., Fan, M., Niu, F., Wu, W., Huang, C., Liu, X., Li, H., and Liu, X. (2012). Belt-like VO_2(M) with a rectangular cross section: A new route to prepare, the phase transition and the optical switching properties. Curr. Appl. Phys. *12*, 875–879.

138. Liu, L., Cao, F., Yao, T., Xu, Y., Zhou, M., Qu, B., Pan, B., Wu, C., Wei, S., and Xie, Y. (2012). New-phase VO_2 micro/nanostructures: Investigation of phase transformation and magnetic property. New J. Chem. *36*, 619–625.

139. Liu, X., Huang, C., Yi, S., Xie, G., Li, H., and Luo, Y. (2007). A new solvothermal method of preparing VO_2 nanosheets and petaloid clusters. Solid State Commun *144*, 259–263.

140. Zhang, S., Fu, J., Su, Q., Fu, C., and Li, X. (2015). Preparation of VO_2 superfine powders by a redox method and in situ characterization on the reversible phase transition. Rare Metal Mat. Eng *44*, 738–742.

141. Ye, J., Zhou, L., Liu, F., Qi, J., Gong, W., Lin, Y., and Ning, G. (2010). Preparation, characterization and properties of thermochromic tungsten-doped vanadium dioxide by thermal reduction and annealing. J. Alloys Compd *504*, 503–507.

142. Gui, Z., Fan, R., Mo, W.Q., Chen, X.H., Yang, L., Zhang, S.Y., Hu, Y., Wang, Z.Z., and Fan, W.C. (2002). Precursor morphology controlled formation of rutile VO_2 nanorods and their self-assembled structure. Chem. Mater *14*, 5053–5056.

143. Chen, J., Liu, X., Dai, L., Chen, L., Gao, Y., and Chen, N. (2012). Deoxidization of V_2O_5 powder into VO_2 assisted by an electrochemical lithium intercalation technique. Int. J. Appl. Ceram. Technol *9*, 942–946.

144. Ji, H., Liu, D., Cheng, H., Zhang, C., and Yang, L. (2018). Vanadium dioxide nanopowders with tunable emissivity for adaptive infrared camouflage in both thermal atmospheric windows. Sol. Energy Mater. Sol. Cells *175*, 96–101.

145. Granqvist, C.G. (2007). Transparent conductors as solar energy materials: A panoramic review. Sol. Energy Mater. Sol. Cells *91*, 1529–1598.

146. Li, S.Y., Niklasson, G.A., and Granqvist, C.G. (2010). Nanothermochromics: Calculations for VO_2 nanoparticles in dielectric hosts show much improved luminous transmittance and solar energy transmittance modulation. J. Appl. Phys. *108*, 63525.

147. Li, S.Y., Niklasson, G.A., and Granqvist, C.G. (2011). Nanothermochromics with VO_2-based core-shell structures: Calculated luminous and solar optical properties. J. Appl. Phys. *109*, 113515.

148. Chen, Z., Gao, Y., Kang, L., Cao, C., Chen, S., and Luo, H. (2014). Fine crystalline VO_2 nanoparticles: Synthesis, abnormal phase transition temperatures and excellent optical properties of a derived VO_2 nanocomposite foil. J. Mater. Chem. A *2*, 2718–2727.

149. Gao, Y., Wang, S., Kang, L., Chen, Z., Du, J., Liu, X., Luo, H., and Kanehira, M. (2012). VO_2-Sb:SnO_2 composite thermochromic smart glass foil. Energy Environ. Sci. *5*, 8234–8237.

150. Gao, Y., Wang, S., Luo, H., Dai, L., Cao, C., Liu, Y., Chen, Z., and Kanehira, M. (2012). Enhanced chemical stability of VO_2 nanoparticles by the formation of SiO_2/VO_2 core/shell structures and the application to transparent and flexible VO_2-based composite foils with excellent thermochromic properties for solar heat control. Energy Environ. Sci. *5*, 6104–6110.

151. Zhou, Y., Huang, A., Li, Y., Ji, S., Gao, Y., and Jin, P. (2013). Surface plasmon resonance induced excellent solar control for VO_2@SiO_2 nanorods-based thermochromic foils. Nanoscale *5*, 9208–9213.

152. Zhu, J., Zhou, Y., Wang, B., Zheng, J., Ji, S., Yao, H., Luo, H., and Jin, P. (2015). Vanadium dioxide nanoparticle-based thermochromic smart coating: High luminous transmittance, excellent solar regulation efficiency, and near room temperature phase transition. ACS Appl. Mater. Interfaces *7*, 27796–27803.

153. Liu, C., Cao, X., Kamyshny, A., Law, J.Y., Magdassi, S., and Long, Y. (2014). VO_2/Si-Al gel nanocomposite thermochromic smart foils: Largely enhanced luminous transmittance and solar modulation. J. Colloid Interface Sci. *427*, 49–53.

154. Moot, T., Palin, C., Mitran, S., Cahoon, J.F., and Lopez, R. (2016). Designing plasmon-enhanced thermochromic films using a vanadium dioxide nanoparticle elastomeric composite. Adv. Opt. Mater *4*, 578–583.

155. Chen, Z., Cao, C., Chen, S., Luo, H., and Gao, Y. (2014). Crystallised mesoporous TiO_2(A)-VO_2(M/R) nanocomposite films with self-cleaning and excellent thermochromic properties. J. Mater. Chem. A *2*, 11874–11884.

156. Zhou, Y., Cai, Y.F., Hu, X., and Long, Y. (2014). Temperature-responsive hydrogel with ultra-large solar modulation and high luminous transmission for "smart window" applications. J. Mater. Chem. A *2*, 13550–13555.

157. Zhou, Y., Cai, Y.F., Hu, X., and Long, Y. (2015). VO_2/hydrogel hybrid nanothermochromic material with ultra-high solar modulation and luminous transmission. J. Mater. Chem. A *3*, 1121–1126.

158. Yang, Y.S., Zhou, Y., Chiang, F., and Long, Y. (2017). Tungsten doped VO_2/microgels hybrid thermochromic material and its smart window application. RSC Adv *7*, 7758–7762.

159. Zhu, J.T., Huang, A.B., Ma, H.B., Ma, Y.N., Tong, K., Ji, S.D., Bao, S.H., Cao, X., and Jin, P. (2016). Composite film of vanadium dioxide nanoparticles and ionic liquid-nickel-chlorine complexes with excellent visible thermochromic performance. ACS Appl. Mater. Interfaces *8*, 29742–29748.

160. Zhu, J.T., Huang, A.B., Ma, H.B., Bao, S.H., Ji, S.D., and Jin, P. (2016). Solar-thermochromism of a hybrid film of VO_2 nanoparticles and Co^{II}-Br-TMP complexes. RSC Adv *6*, 67396–67399.

161. Zhu, J.T., Huang, A.B., Ma, H.B., Chen, Y.X., Zhang, S.P., Ji, S.D., Bao, S.H., and Jin, P. (2017). Hybrid films of VO_2 nanoparticles and a nickel(II)-based ligand exchange thermochromic system: Excellent optical performance with a temperature responsive colour change. New J. Chem. *41*, 830–835.

162. Laaksonen, K., Li, S.Y., Puisto, S.R., Rostedt, N.K.J., Ala-Nissila, T., Granqvist, C.G., Nieminen, R.M., and Niklasson, G.A. (2014). Nanoparticles of TiO_2 and VO_2 in dielectric media: Conditions for low optical scattering, and comparison between effective medium and four-flux theories. Sol. Energy Mater. Sol. Cells *130*, 132–137.

163. Shen, N., Dong, B., Cao, C., Chen, Z., Luo, H., and Gao, Y. (2015). Solid-state-reaction synthesis of VO_2 nanoparticles with low phase transition temperature, enhanced chemical stability and excellent thermochromic properties. RSC Adv *5*, 108015–108022.

164. Ji, H., Liu, D., Zhang, C., and Cheng, H. (2018). VO_2/ZnS core-shell nanoparticle for the adaptive infrared camouflage application with modified color and enhanced oxidation resistance. Sol. Energy Mater. Sol. Cells *176*, 1–8.

165. Lu, Q., Liu, C., Wang, N., Magdassi, S., Mandler, D., and Long, Y. (2016). Periodic micro-patterned VO_2 thermochromic films by mesh printing. J. Mater. Chem. C *4*, 8385–8391.

166. Hild, E., and Grofcsik, A. (1978). Calculation of IR reflection spectra of inhomogeneously doped semiconductors. Infrared Phys. *18*, 23–33.

167. Hild, E., and Evans, M.W. (1986). Calculation of the infrared reflection spectra of inhomogeneously doped silicon semiconductor layers at an arbitrary angle of incidence. J. Appl. Phys. *59*, 1822–1828.

168. Deak, A., Hild, E., Kovacs, A.L., and Horvolgyi, Z. (2007). Contact angle determination of nanoparticles: Film balance and scanning angle reflectometry studies. Phys. Chem. Chem. Phys. *9*, 6359–6370.

169. Hild, E., and Deák, A. (2007). Use of the optical admittance function and its WKB approximation to simulate and evaluate transmittance spectra of graded-index colloidal films. J. Opt. A-Pure Appl. Opt *9*, 920.

170. Kang, L., Gao, Y., Luo, H., Chen, Z., Du, J., and Zhang, Z. (2011). Nanoporous thermochromic VO_2 films with low optical constants, enhanced luminous transmittance and thermochromic properties. ACS Appl. Mater. Interfaces *3*, 135–138.

171. Liu, M., Su, B., Kaneti, Y., Chen, Z., Tang, Y., Yuan, Y., Gao, Y., Jiang, L., Jiang, X., and Yu, A. (2017). Dual-phase transformation: Spontaneous self-template surface-patterning strategy for ultra-transparent VO_2 solar modulating coatings. ACS Nano *11*, 407–415.

172. Ding, S., Liu, Z., Li, D., Zhao, W., Wang, Y., Wan, D., and Huang, F. (2013). Tunable assembly of vanadium dioxide nanoparticles to create porous film for energy-saving applications. ACS Appl. Mater. Interfaces *5*, 1630–1635.

173. Xu, Y.J., Huang, W.X., Shi, Q.W., Zhang, Y.B., Wu, J., and Song, L.W. (2013). Shape-dependent thermochromic phenomenon in porous nanostructured VO_2 films. Mater. Res. Bull. *48*, 4146–4149.

174. Xu, Y.J., Huang, W.X., Shi, Q.W., Zhang, Y.B., Wu, J., and Song, L.W. (2013). Porous nano-structured VO_2 films with different surfactants: Synthesis mechanisms, characterization, and applications. J. Mater. Sci.-Mater. Electron *24*, 3823–3829.

175. Cao, X., Wang, N., Law, J.Y., Loo, S., Magdassi, S., and Long, Y. (2014). Nanoporous thermochromic VO_2(M) thin films: Controlled porosity, largely enhanced luminous transmittance and solar modulating ability. Langmuir *30*, 1710–1715.

176. Wang, N., Huang, Y.Z., Magdassi, S., Mandler, D., Liu, H., and Long, Y. (2013). Formation of VO_2 zero-dimensional/nanoporous layers with large supercooling effects and enhanced thermochromic properties. RSC Adv *3*, 7124–7128.

177. Zhang, J., Jin, H., Chen, Z., Cao, M., Chen, P., Dou, Y., Zhao, Y., and Li, J. (2015). Self-assembling VO_2 nanonet with high switching performance at wafer-scale. Chem. Mater *27*, 7419–7424.

178. Liu, C., Balin, I., Magdassi, S., Abdulhalim, I., and Long, Y. (2015). Vanadium dioxide nanogrid films for high transparency smart architectural window applications. Opt. Express *23*, A124–A132.

179. Ke, Y., Wen, X., Zhao, D., Che, R., Xiong, Q., and Long, Y. (2017). Controllable fabrication of two-dimensional patterned VO_2 nanoparticle, nanodome, and nanonet arrays with tunable temperature-dependent localized surface plasmon resonance. ACS Nano *11*, 7542–7551.

180. Liu, C., Long, Y., Magdassi, S., and Mandler, D. (2017). Ionic strength induced electrodeposition: A universal approach for nanomaterial deposition at selective areas. Nanoscale *9*, 485–490.

181. Zhou, M., Bao, J., Tao, M., Zhu, R., Lin, Y., Zhang, X., and Xie, Y. (2013). Periodic porous thermochromic VO_2(M) films with enhanced visible transmittance. Chem. Commun *49*, 6021–6023.

182. Ye, X., and Qi, L. (2011). Two-dimensionally patterned nanostructures based on monolayer colloidal crystals: Controllable fabrication, assembly, and applications. Nano Today *6*, 608–631.

183. Yu, K., Fan, T., Lou, S., and Zhang, D. (2013). Biomimetic optical materials: Integration of nature's design for manipulation of light. Prog. Mater. Sci. *58*, 825–873.

184. Li, Y.F., Zhang, J.H., Zhu, S.J., Dong, H.P., Jia, F., Wang, Z.H., Sun, Z.Q., Zhang, L., Li, Y., Li, H.B., et al. (2009). Biomimetic surfaces for high-performance optics. Adv. Mater *21*, 4731–4734.

185. Vukusic, P., and Sambles, J.R. (2003). Photonic structures in biology. Nature *424*, 852–855.

186. Taylor, A., Parkin, I., Noor, N., Tummeltshammer, C., Brown, M.S., and Papakonstantinou, I. (2013). A bioinspired solution for spectrally selective thermochromic VO_2 coated intelligent glazing. Opt. Express *21*, A750–A764.

187. Qian, X., Wang, N., Li, Y., Zhang, J., Xu, Z., and Long, Y. (2014). Bioinspired multifunctional vanadium dioxide: Improved thermochromism and hydrophobicity. Langmuir *30*, 10766–10771.

188. Ke, Y., Balin, I., Wang, N., Lu, Q., Tok, A.T.Y., White, T.J., Magdassi, S., Abdulhalim, I., and Long, Y. (2016). Two-dimensional SiO_2/VO_2 photonic crystals with statically visible and dynamically infrared modulated for smart window deployment. ACS Appl. Mater. Interfaces *8*, 33112–33120.

189. Yablonovitch, E. (1987). Inhibited spontaneous emission in solid-state physics and electronics. Phys. Rev. Lett. *58*, 2059–2062.

190. Aguirre, C.I., Reguera, E., and Stein, A. (2010). Tunable colors in opals and inverse opal photonic crystals. Adv. Funct. Mater *20*, 2565–2578.

191. Saitzek, S., Guinneton, F., Sauques, L., Aguir, K., and Gavarri, J. (2007). Thermochromic CeO_2-VO_2 bilayers: Role of ceria coating in optical switching properties. Opt. Mater *30*, 407–415.

192. Liu, C., Wang, N., and Long, Y. (2013). Multifunctional overcoats on vanadium dioxide thermochromic thin films with enhanced luminous transmission and solar modulation, hydrophobicity and anti-oxidation. Appl. Surf. Sci. *283*, 222–226.

193. Evans, P., Pemble, M.E., Sheel, D.W., and Yates, H.M. (2007). Multi-functional self-cleaning thermochromic films by atmospheric pressure chemical vapour deposition. J. Photochem. Photobiol. A Chem. *189*, 387–397.

194. Raut, H.K., Ganesh, V.A., Nair, A.S., and Ramakrishna, S. (2011). Anti-reflective coatings: A critical, in-depth review. Energy Environ. Sci. *4*, 3779–3804.

195. Tazawa, M., Jin, P., and Tanemura, S. (1998). Optical constants of V1-xWxO_2 films. Appl. Optics *37*, 1858–1861.

196. Xu, G., Jin, P., Tazawa, M., and Yoshimura, K. (2004). Optimization of antireflection coating for VO_2-based energy efficient window. Sol. Energy Mater. Sol. Cells *83*, 29–37.

197. Jin, P., Xu, G., Tazawa, M., and Yoshimura, K. (2002). A VO_2-based multifunctional window with highly improved luminous transmittance. Jpn. J. Appl. Phys. *41*, L278–L280.

198. Koo, H., Shin, D., Bae, S., Ko, K., Chang, S., and Park, C. (2014). The effect of CeO_2 antireflection layer on the optical properties of thermochromic VO_2 film for smart window system. J. Mater. Eng. Perform *23*, 402–407.

199. Zhang, Z., Gao, Y., Luo, H., Kang, L., Chen, Z., Du, J., Kanehira, M., Zhang, Y., and Wang, Z.L. (2011). Solution-based fabrication of vanadium dioxide on F:SnO_2 substrates with largely enhanced thermochromism and low-emissivity for energy-saving applications. Energy Environ. Sci. *4*, 4290–4297.

200. Jin, P., Xu, G., Tazawa, M., and Yoshimura, K. (2003). Design, formation and characterization of a novel multifunctional window with VO_2 and TiO_2 coatings. Appl. Phys. A Mater. Sci. Process *77*, 455–459.

201. Zheng, J., Bao, S., and Jin, P. (2015). TiO_2(R)/VO_2(M)/TiO_2(A) multilayer film as smart window: Combination of energy-saving, antifogging and self-cleaning functions. Nano Energy *11*, 136–145.

202. Liu, C., Wang, S., Zhou, Y., Yang, H., Lu, Q., Mandler, D., Magdassi, S., Tay, C.Y., and Long, Y. (2018). Index-tunable anti-reflection coatings: Maximizing solar modulation ability for vanadium dioxide-based smart thermochromic glazing. J. Alloys Compd *731*, 1197–1207.

203. Chen, Z., Gao, Y., Kang, L., Du, J., Zhang, Z., Luo, H., Miao, H., and Tan, G. (2011). VO_2-based double-layered films for smart windows: Optical design, all-solution preparation and improved properties. Sol. Energy Mater. Sol. Cells *95*, 2677–2684.

204. Kang, L., Gao, Y., Chen, Z., Du, J., Zhang, Z., and Luo, H. (2010). Pt/VO_2 double-layered films combining thermochromic properties with low emissivity. Sol. Energy Mater. Sol. Cells *94*, 2078–2084.

205. Mlyuka, N.R., Niklasson, G.A., and Granqvist, C.G. (2009). Thermochromic multilayer films of VO_2 and TiO_2 with enhanced transmittance. Sol. Energy Mater. Sol. Cells *93*, 1685–1687.

206. Kang, L., Gao, Y., Luo, H., Wang, J., Zhu, B., Zhang, Z., Du, J., Kanehira, M., and Zhang, Y. (2011). Thermochromic properties and low emissivity of ZnO:Al/VO_2 double-layered films with a lowered phase transition temperature. Sol. Energy Mater. Sol. Cells *95*, 3189–3194.

207. Zhang, Z., Gao, Y., Kang, L., Du, J., and Luo, H. (2010). Effects of a TiO_2 buffer layer on solution-deposited VO_2 films: Enhanced oxidization durability. J. Phys. Chem. C *114*, 22214–22220.

208. Zhang, Z., Gao, Y., Chen, Z., Du, J., Cao, C., Kang, L., and Luo, H. (2010). Thermochromic VO_2 thin films: Solution-based processing, improved optical properties, and lowered phase transformation temperature. Langmuir *26*, 10738–10744.

209. Voti, R.L., Larciprete, M.C., Leahu, G., Sibilia, C., and Bertolotti, M. (2012). Optical response of multilayer thermochromic VO_2-based structures. J. Nanophotonics *6*, 61601.

210. Du, J., Gao, Y., Chen, Z., Kang, L., Zhang, Z., and Luo, H. (2013). Enhancing thermochromic performance of VO_2 films *via* increased microroughness by phase separation. Sol. Energy Mater. Sol. Cells *110*, 1–7.

211. Hendaoui, A., Emond, N., Chaker, M., and Haddad, E. (2013). Highly tunable-emittance radiator based on semiconductor-metal transition of VO_2 thin films. Appl. Phys. Lett *102*, 061107.

212. Saeli, M., Piccirillo, C., Parkin, I.P., Ridley, I., and Binions, R. (2010). Nano-composite thermochromic thin films and their application in energy-efficient glazing. Sol. Energy Mater. Sol. Cells *94*, 141–151.

213. Saeli, M., Piccirillo, C., Parkin, I.P., Binions, R., and Ridley, I. (2010). Energy modelling studies of thermochromic glazing. Energy Build *42*, 1666–1673.

214. Chang, T., Cao, X., Dedon, L.R., Long, S., Huang, A., Shao, Z., Li, N., Luo, H., and Jin, P. (2018). Optical design and stability study for ultrahigh-performance and long-lived vanadium dioxide-based thermochromic coatings. Nano Energy *44*, 256–264.

215. Kim, H., Kim, Y., Kim, K.S., Jeong, H.Y., Jang, A., Han, S.H., Yoon, D.H., Suh, K.S., Shin, H.S., Kim, T., et al. (2013). Flexible thermochromic window based on hybridized VO_2/graphene. ACS Nano *7*, 5769–5776.

216. Ye, H., Long, L., Zhang, H., Xu, B., Gao, Y., Kang, L., and Chen, Z. (2013). The demonstration and simulation of the application performance of the vanadium dioxide single glazing. Sol. Energy Mater. Sol. Cells *117*, 168–173.

217. Yang, J., Xu, Z., Ye, H., Xu, X., Wu, X., and Wang, J. (2015). Performance analyses of building energy on phase transition processes of VO_2 windows with an improved model. Appl. Energy *159*, 502–508.

218. Ye, H., Meng, X., and Xu, B. (2012). Theoretical discussions of perfect window, ideal near infrared solar spectrum regulating window and current thermochromic window. Energy Build. *49*, 164–172.

219. Long, L., and Ye, H. (2014). How to be smart and energy efficient: a general discussion on thermochromic windows. Sci. Rep. *4*, 6427.

220. Long, L., and Ye, H. (2014). Discussion of the performance improvement of thermochromic smart glazing applied in passive buildings. Sol. Energy *107*, 236–244.

221. Ye, H., and Long, L. (2014). Smart or not? A theoretical discussion on the smart regulation capacity of vanadium dioxide glazing. Sol. Energy Mater. Sol. Cells *120*, 669–674.
