# Supplementary material for: Advanced liquid crystal-based switchable optical devices for light protection applications: principles and strategies
Source: Light Sci Appl. 2023 Jan 3;12:11. doi: 10.1038/s41377-022-01032-y (PMC9807646; doi:10.1038/s41377-022-01032-y)
Supplement: Supplementary file 3 — Fig 3 copyright promotion [file 41377_2022_1032_MOESM3_ESM.pdf]

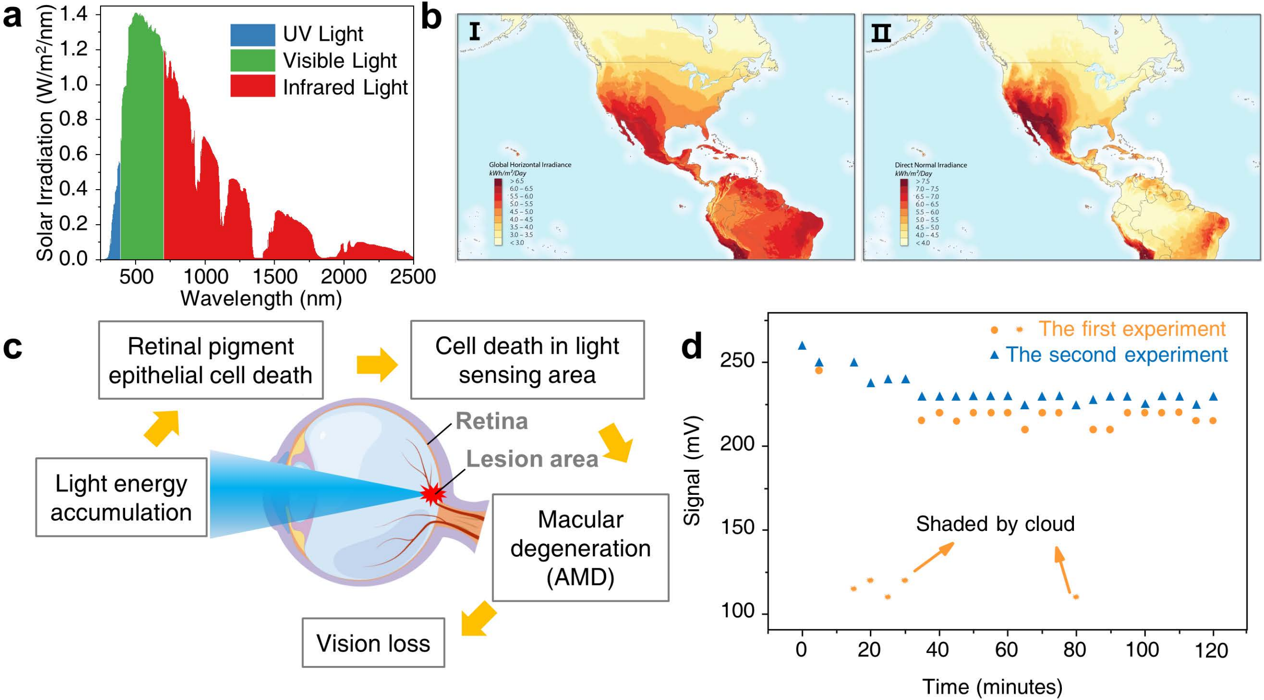

a
Solar Irradiation (W/m²/nm)
1.4
1.2
1.0
0.8
0.6
0.4
0.2
0.0
UV Light
Visible Light
Infrared Light
500
1000
1500
2000
2500
Wavelength (nm)
b
I
Global Horizontal Irradiance
kWh/m²/Day
> 6.5
6.0 – 6.5
5.5 – 6.0
5.0 – 5.5
4.5 – 5.0
4.0 – 4.5
3.5 – 4.0
3.0 – 3.5
< 3.0
II
Direct Normal Irradiance
kWh/m²/Day
> 7.5
7.0 – 7.5
6.5 – 7.0
6.0 – 6.5
5.5 – 6.0
5.0 – 5.5
4.5 – 5.0
4.0 – 4.5
< 4.0
c
Retinal pigment epithelial cell death
Cell death in light sensing area
Retina
Lesion area
Light energy accumulation
Macular degeneration (AMD)
Vision loss
d
The first experiment
The second experiment
Signal (mV)
250
200
150
100
Shaded by cloud
0
20
40
60
80
100
120
Time (minutes)

JOHN WILEY AND SONS LICENSE TERMS AND CONDITIONS

Sep 19, 2022

This Agreement between Harbin Institute of Technology -- Ruicong Zhang ("You") and John Wiley and Sons ("John Wiley and Sons") consists of your license details and the terms and conditions provided by John Wiley and Sons and Copyright Clearance Center.

License Number	5392390962612
License date	Sep 19, 2022
Licensed Content Publisher	John Wiley and Sons
Licensed Content Publication	Advanced Energy Materials
Licensed Content Title	Infrared Regulating Smart Window Based on Organic Materials
Licensed Content Author	Hitesh Khandelwal, Albertus P. H. J. Schenning, Michael G. Debije
Licensed Content Date	Mar 2, 2017
Licensed Content Volume	7
Licensed Content Issue	14
Licensed Content Pages	18
Type of Use	Journal/Magazine
Requestor type	University/Academic
Is the reuse sponsored by or associated with a pharmaceutical or medical products company?	no
Format	Print and electronic
Portion	Figure/table
Number of figures/tables	1
Will you be translating?	No
Circulation	100 - 199
Title of new article	Advanced liquid crystal-based switchable optical devices for light protection applications: principles and strategies
Lead author	Ruicong Zhang, Zhibo Zhang, Jiecai Han, Lei Yang, Jiajun li, Zicheng Song Tianyu Wang, Jiaqi Zhu
Title of targeted journal	Light: Science & Applications
Publisher	Springer Nature
Expected publication date	Nov 2022
Portions	Figure 1 b
Requestor Location	Harbin Institute of Technology<br>No. 92, Xidazhi Street, Nangang District<br><br>Harbin, 150080<br>China<br>Attn: Harbin Institute of Technology
Publisher Tax ID	EU826007151
Total	**0.00 USD**
Terms and Conditions	

TERMS AND CONDITIONS

This copyrighted material is owned by or exclusively licensed to John Wiley & Sons, Inc. or one of its group companies (each a"Wiley Company") or handled on behalf of a society with which a Wiley Company has exclusive publishing rights in relation to a particular work (collectively "WILEY"). By clicking "accept" in connection with completing this licensing transaction, you agree that the following terms and conditions apply to this transaction (along with the billing and payment terms and conditions

established by the Copyright Clearance Center Inc., ("CCC's Billing and Payment terms and conditions"), at the time that you opened your RightsLink account (these are available at any time at http://myaccount.copyright.com).

Terms and Conditions

- The materials you have requested permission to reproduce or reuse (the "Wiley Materials") are protected by copyright.

- You are hereby granted a personal, non-exclusive, non-sub licensable (on a stand-alone basis), non-transferable, worldwide, limited license to reproduce the Wiley Materials for the purpose specified in the licensing process. This license, **and any CONTENT (PDF or image file) purchased as part of your order,** is for a one-time use only and limited to any maximum distribution number specified in the license. The first instance of republication or reuse granted by this license must be completed within two years of the date of the grant of this license (although copies prepared before the end date may be distributed thereafter). The Wiley Materials shall not be used in any other manner or for any other purpose, beyond what is granted in the license. Permission is granted subject to an appropriate acknowledgement given to the author, title of the material/book/journal and the publisher. You shall also duplicate the copyright notice that appears in the Wiley publication in your use of the Wiley Material. Permission is also granted on the understanding that nowhere in the text is a previously published source acknowledged for all or part of this Wiley Material. Any third party content is expressly excluded from this permission.

- With respect to the Wiley Materials, all rights are reserved. Except as expressly granted by the terms of the license, no part of the Wiley Materials may be copied, modified, adapted (except for minor reformatting required by the new Publication), translated, reproduced, transferred or distributed, in any form or by any means, and no derivative works may be made based on the Wiley Materials without the prior permission of the respective copyright owner.**For STM Signatory Publishers clearing permission under the terms of the STM Permissions Guidelines only, the terms of the license are extended to include subsequent editions and for editions in other languages, provided such editions are for the work as a whole in situ and does not involve the separate exploitation of the permitted figures or extracts,** You may not alter, remove or suppress in any manner any copyright, trademark or other notices displayed by the Wiley Materials. You may not license, rent, sell, loan, lease, pledge, offer as security, transfer or assign the Wiley Materials on a stand-alone basis, or any of the rights granted to you hereunder to any other person.

- The Wiley Materials and all of the intellectual property rights therein shall at all times remain the exclusive property of John Wiley & Sons Inc, the Wiley Companies, or their respective licensors, and your interest therein is only that of having possession of and the right to reproduce the Wiley Materials pursuant to Section 2 herein during the continuance of this Agreement. You agree that you own no right, title or interest in or to the Wiley Materials or any of the intellectual property rights therein. You shall have no rights hereunder other than the license as provided for above in Section 2. No right, license or interest to any trademark, trade name, service mark or other branding ("Marks") of WILEY or its licensors is granted hereunder, and you agree that you shall not assert any such right, license or interest with respect thereto

- NEITHER WILEY NOR ITS LICENSORS MAKES ANY WARRANTY OR REPRESENTATION OF ANY KIND TO YOU OR ANY THIRD PARTY, EXPRESS, IMPLIED OR STATUTORY, WITH RESPECT TO THE MATERIALS OR THE ACCURACY OF ANY INFORMATION CONTAINED IN THE MATERIALS, INCLUDING, WITHOUT LIMITATION, ANY IMPLIED WARRANTY OF MERCHANTABILITY, ACCURACY, SATISFACTORY QUALITY, FITNESS FOR A PARTICULAR PURPOSE, USABILITY, INTEGRATION OR NON-INFRINGEMENT AND ALL SUCH WARRANTIES ARE HEREBY EXCLUDED BY WILEY AND ITS LICENSORS AND WAIVED BY YOU.

- WILEY shall have the right to terminate this Agreement immediately upon breach of this Agreement by you.

- You shall indemnify, defend and hold harmless WILEY, its Licensors and their respective directors, officers, agents and employees, from and against any actual or threatened claims, demands, causes of action or proceedings arising from any breach of this Agreement by you.

- IN NO EVENT SHALL WILEY OR ITS LICENSORS BE LIABLE TO YOU OR ANY OTHER PARTY OR ANY OTHER PERSON OR ENTITY FOR ANY SPECIAL, CONSEQUENTIAL, INCIDENTAL, INDIRECT, EXEMPLARY OR PUNITIVE DAMAGES, HOWEVER CAUSED, ARISING OUT OF OR IN CONNECTION WITH THE DOWNLOADING, PROVISIONING, VIEWING OR USE OF THE MATERIALS REGARDLESS OF THE FORM OF ACTION, WHETHER FOR BREACH OF CONTRACT, BREACH OF WARRANTY, TORT, NEGLIGENCE, INFRINGEMENT OR OTHERWISE (INCLUDING, WITHOUT LIMITATION, DAMAGES BASED ON LOSS OF PROFITS, DATA, FILES, USE, BUSINESS OPPORTUNITY OR CLAIMS OF THIRD PARTIES), AND WHETHER OR NOT THE PARTY HAS BEEN ADVISED OF THE POSSIBILITY OF SUCH DAMAGES. THIS LIMITATION SHALL APPLY NOTWITHSTANDING ANY FAILURE OF ESSENTIAL PURPOSE OF ANY LIMITED REMEDY PROVIDED HEREIN.

- Should any provision of this Agreement be held by a court of competent jurisdiction to be illegal, invalid, or unenforceable, that provision shall be deemed amended to achieve as nearly as possible the same economic effect as the original provision, and the legality, validity and enforceability of the remaining provisions of this Agreement shall not

be affected or impaired thereby.

- The failure of either party to enforce any term or condition of this Agreement shall not constitute a waiver of either party's right to enforce each and every term and condition of this Agreement. No breach under this agreement shall be deemed waived or excused by either party unless such waiver or consent is in writing signed by the party granting such waiver or consent. The waiver by or consent of a party to a breach of any provision of this Agreement shall not operate or be construed as a waiver of or consent to any other or subsequent breach by such other party.
- This Agreement may not be assigned (including by operation of law or otherwise) by you without WILEY's prior written consent.
- Any fee required for this permission shall be non-refundable after thirty (30) days from receipt by the CCC.
- These terms and conditions together with CCC's Billing and Payment terms and conditions (which are incorporated herein) form the entire agreement between you and WILEY concerning this licensing transaction and (in the absence of fraud) supersedes all prior agreements and representations of the parties, oral or written. This Agreement may not be amended except in writing signed by both parties. This Agreement shall be binding upon and inure to the benefit of the parties' successors, legal representatives, and authorized assigns.
- In the event of any conflict between your obligations established by these terms and conditions and those established by CCC's Billing and Payment terms and conditions, these terms and conditions shall prevail.
- WILEY expressly reserves all rights not specifically granted in the combination of (i) the license details provided by you and accepted in the course of this licensing transaction, (ii) these terms and conditions and (iii) CCC's Billing and Payment terms and conditions.
- This Agreement will be void if the Type of Use, Format, Circulation, or Requestor Type was misrepresented during the licensing process.
- This Agreement shall be governed by and construed in accordance with the laws of the State of New York, USA, without regards to such state's conflict of law rules. Any legal action, suit or proceeding arising out of or relating to these Terms and Conditions or the breach thereof shall be instituted in a court of competent jurisdiction in New York County in the State of New York in the United States of America and each party hereby consents and submits to the personal jurisdiction of such court, waives any objection to venue in such court and consents to service of process by registered or certified mail, return receipt requested, at the last known address of such party.

WILEY OPEN ACCESS TERMS AND CONDITIONS
Wiley Publishes Open Access Articles in fully Open Access Journals and in Subscription journals offering Online Open. Although most of the fully Open Access journals publish open access articles under the terms of the Creative Commons Attribution (CC BY) License only, the subscription journals and a few of the Open Access Journals offer a choice of Creative Commons Licenses. The license type is clearly identified on the article.
The Creative Commons Attribution License
The Creative Commons Attribution License (CC-BY) allows users to copy, distribute and transmit an article, adapt the article and make commercial use of the article. The CC-BY license permits commercial and non-
Creative Commons Attribution Non-Commercial License
The Creative Commons Attribution Non-Commercial (CC-BY-NC)License permits use, distribution and reproduction in any medium, provided the original work is properly cited and is not used for commercial purposes.(see below)

Creative Commons Attribution-Non-Commercial-NoDerivs License
The Creative Commons Attribution Non-Commercial-NoDerivs License (CC-BY-NC-ND) permits use, distribution and reproduction in any medium, provided the original work is properly cited, is not used for commercial purposes and no modifications or adaptations are made. (see below)
Use by commercial "for-profit" organizations
Use of Wiley Open Access articles for commercial, promotional, or marketing purposes requires further explicit permission from Wiley and will be subject to a fee.
Further details can be found on Wiley Online Library http://olabout.wiley.com/WileyCDA/Section/id-410895.html

Other Terms and Conditions:

v1.10 Last updated September 2015

Questions? customercare@copyright.com or +1-855-239-3415 (toll free in the US) or +1-978-646-2777.

Thermo Fisher
SCIENTIFIC

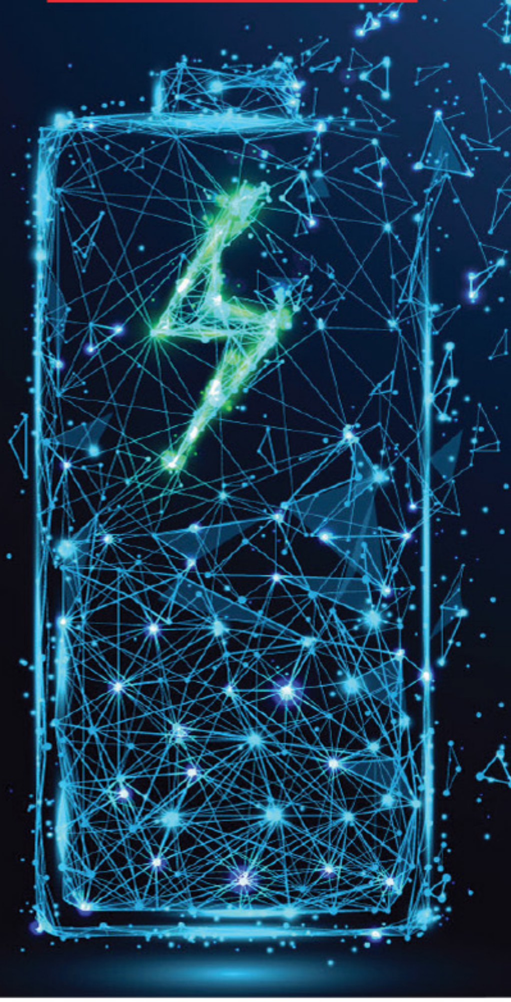

Lithium-ion battery

PowerUp webinars

Analytical solutions for lithium-ion battery material analysis and testing

PowerUp your battery material analysis - Join our educational webinars

Heading toward zero emission goals, lithium-ion battery are expected to generate an unprecedented demand for battery raw material in the upcoming decade. At the same time battery research, production, and quality control will need to keep up with the accelerated demand for electric storage capacities. Battery manufacturers must deliver consistently high quality throughout the entire battery value chain. Analysis and testing of batteries raw material and components requires therefore a variety of analytical methods that provide insights of quality and properties at various scales.

In this webinar session we will highlight the benefits of several analytical techniques and applications, as chromatography, mass spectrometry and electron microscopy, in battery material and structural analysis.

Free education webinars
October 5 and 6, 2022 | 10:00 BST | 11:00 CEST
Part 1: Analysis of cathode materials
Part 2: Analysis of battery electrolytes

No time for live session? Register anyway and get access to the on-demand recording after the live broadcast.

Key learnings

- Learn about analytical solutions for lithium-ion battery material and structural analysis
- Discover how IC, GC-MS, ICP-OES, and SEM support battery value chain from raw materials to recycling
- Get deeper insights based-on selected applications and performance examples

Learn more at **thermofisher.com/battery-webinars**

thermo scientific

For Research Use Only. Not for use in diagnostic procedures. © 2022 Thermo Fisher Scientific Inc. All rights reserved. All trademarks are the property of Thermo Fisher Scientific and its subsidiaries unless otherwise specified. **FL-001396-em-en**

Infrared Regulating Smart Window Based on Organic Materials

Hitesh Khandelwal, Albertus P. H. J. Schenning, and Michael G. Debije**

Windows are vital elements in the built environment that have a large impact on the energy consumption in indoor spaces, affecting heating and cooling and artificial lighting requirements. Moreover, they play an important role in sustaining human health and well-being. In this review, we discuss the next generation of smart windows based on organic materials which can change their properties by reflecting or transmitting excess solar energy (infrared radiation) in such a way that comfortable indoor temperatures can be maintained throughout the year. Moreover, we place emphasis on windows that maintain transparency in the visible region so that additional energy is not required to retain natural illumination. We discuss a number of ways to fabricate windows which remain as permanent infrared control elements throughout the year as well as windows which can alter transmission properties in presence of external stimuli like electric fields, temperature and incident light intensity. We also show the potential impact of these windows on energy saving in different climate conditions.

1. Introduction

More than 50% of the total energy used in the building envelope in the Western world is spent on cooling, heating and lighting the interior places (**Figure 1**a).[1,2] A significant fraction of this energy use is related to our inability to control the ingress and egress of infrared light from the sun through windows. Near infrared light (IR), here defined as light with wavelengths between 700 nm and 2500 nm, accounts for around 50% of the total energy emitted by the sun reaching Earth (Figure 1b),[3,4] and this light produces interior heating but is invisible to the unaided eye.

The absorption of sunlight by building materials and passage of IR through transparent surfaces such as windows is responsible for much of the interior overheating of office rooms, automobile interiors, greenhouses, and other similar spaces. The use of artificial cooling and heating systems will only increase with the continued influence of global climate change, with energy used for cooling systems surpassing energy used for heating around the year 2070, and a 40 fold increase in air cooling energy use is expected by 2100.[5] By controlling the influx of radiant heat transfer, calculations show that more than 50% of the energy used in lighting, heating and cooling could be saved by deploying better control systems over only 18% of available window stock.[6]

In areas with human inhabitants employing windows, more aspects must be considered than simply reducing the use of energy in the room: any switchable window used in, for example, a commercial office space has several other requirements that must be met before it may be installed. Among these requirement are reasonably fast switching speeds[7] (although for IR control, relatively longer times compared to visible light switching should be acceptable), good optical transparency with minimum haze, an acceptable device lifetime,[8] and functionality over a range of exterior temperatures. Controlling the excess of solar energy without compromising the visible transparency of the window is an important consideration for human health: maintaining inside/outside contact and daylighting are vital in retaining well-being and productivity, as well as providing economic and aesthetic gain by reducing the need for artificial lighting systems.[9,10] These are challenging goals for a window to realize.

A number of materials have been developed over the past few decades to maintain indoor temperatures. Many of these focus on the opaque structural building elements like walls and roofing.[10–13] Other solutions target the transparent window, employing external mechanical shutters and blinds,[14] phase change materials (PCMs),[15] thermochromic materials,[16] aerogels,[17] trapped gas in fluid membranes,[18] and even phononic materials,[19] among other options. Indeed, controlling heat passage through the window in response to changing climate conditions is a great challenge; ideally, one would accomplish

H. Khandelwal, Prof. A. P. H. J. Schenning, Dr. M. G. Debije
Functional Organic Materials and Devices
Department of Chemical Engineering and Chemistry
Eindhoven University of Technology
Den Dolech 2, 5600 MB Eindhoven, The Netherlands
E-mail: A.P.H.J.Schenning@tue.nl; m.g.debije@tue.nl
H. Khandelwal
Dutch Polymer Institute (DPI)
P.O. Box 902, 5600 AX Eindhoven, The Netherlands
Prof. A. P. H. J. Schenning
Institute for Complex Molecular Systems (ICMS)
Eindhoven University of Technology
5600 MB Eindhoven, The Netherlands
Prof. A. P. H. J. Schenning
Laboratory of Device Integrated Responsive Materials (DIRM)
Guangzhou, China

This is an open access article under the terms of the Creative Commons Attribution-NonCommercial License, which permits use, distribution and reproduction in any medium, provided the original work is properly cited and is not used for commercial purposes.

DOI: 10.1002/aenm.201602209

this without compromising the influx of visible light and the integrity of the view beyond the window.[20]

The focus of this review is on infrared regulating windows based on organic materials which can adjust the transmittance of IR radiations depending on environmental conditions (**Figure 2**). There are advantages to employing organic rather than inorganic materials in IR window control systems: for instance, since they are non-metallic, they do not corrode or interfere with electromagnetic waves (signals from/to radios, cell phones, GPS, or garage door openers, for example)[21–23] and often are much easier to process at lower temperatures than inorganic materials.

Cholesteric (or 'Chiral nematic') liquid crystalline (Ch-LC) materials have attracted much attention for development of infrared regulating windows. They are formed when nematic liquid crystals are doped with chiral molecules. The chiral dopants generate a LC organization wherein successive layers of nematic LC are displaced by a small rotation in molecular director with respect to their neighboring layers. The 'twist' generated may be either right- or left-handed, depending on the nature of the chiral dopant molecule. The central reflection band of Ch-LC is determined by the pitch (P), average refractive index (n_{avg}) of the material and incident angle of light (Equation (1)). Pitch (P) of the Ch-LC depends on the concentration (C) and helical twisting power (HTP) of the chiral dopants (Equation (2)). Ch-LC selective mirrors demonstrate a distinct advantage over, say, an inorganic Bragg reflector in that the LC self-organizes into a helical structure and can be easily processed from solution. Moreover, Ch-LCs can be made responsive to external stimuli, including temperature, electric/magnetic fields, light, pH, humidity and gasses that makes them interesting for a variety of applications.[24,25] It is important to note that since a cholesteric-based reflector has a degree of angular dependence with respect to the incident light (Equation (1)), a blue shift in reflection band will be observed on deviating from the normal incident angle.[26,27] The bandwidth of the light reflected by the Ch-LC is determined by the difference between the extraordinary (n_e) and ordinary (n_o) refractive indices and the pitch of the host LC (Equation (3)).

$$\lambda_o = P \times n_{avg} \times \cos\theta \tag{1}$$

$$\mathrm{P} = \frac{1}{\mathrm{C} \times HTP} \tag{2}$$

$$\Delta\lambda = (n_e - n_0) \times P \tag{3}$$

The maximum reflection by the cholesteric reflector layer is limited to 50% of the incident sunlight, matching the polarization of the helix: that is, a right-handed cholesteric will reflect only right circularly-polarized light. Both left-circularly polarized light and light outside of the cholesteric reflection bandwidth are unaffected by the liquid crystal matrix and are transmitted normally (**Figure 3**).[25]

LC molecules can be oriented in number of ways between two glass plates (**Figure 4**). The arrangements of the LC molecules determine their collective optical properties. For example, when the molecules are arranged in a helical fashion and parallel to the substrate, known as planar alignment, the layer reflects light of specific wavelengths depending on the pitch and

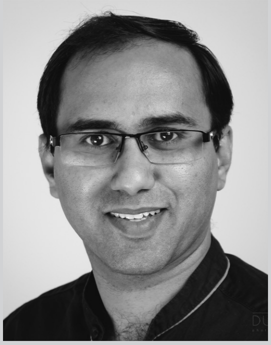

Hitesh Khandelwal received Integrated Bachelor and Master degree from Indian Institute of Science Education and Research, Thiruvananthapuram (IISER-TVM), India (2008–2013). In April 2013, he defended his Master thesis from the group of Dr. Mahesh Hariharan. In May 2013, he joined the group of Functional Organic Materials and Devices, Eindhoven University of Technology for Ph.D. He is currently working on infrared reflecting smart windows based on liquid crystal polymer under the supervision of Dr. Michal Debije, Prof. Dick J Broer and Prof. Albert Schenning. He received INSPIRE fellowship from year 2008–2013.

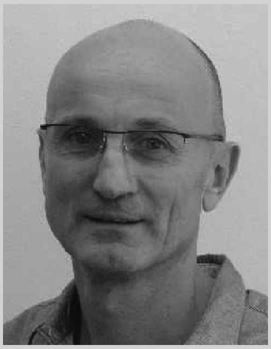

Albert Schenning received his Ph.D. degree at the University of Nijmegen in 1996 under the direction of Dr. M. C. Feiters and Prof. Dr. R. J. M. Nolte. Thereafter he was a post-doctoral fellow in the group of Prof. Dr. E. W. Meijer at Eindhoven University of Technology, and in 1997 he joined the group of Prof. Dr. F. Diederich at the ETH in Zurich. From 1998 until 2002, he was a Royal Netherlands Academy of Science (KNAW) fellow at Eindhoven University of Technology. He is currently full professor at the Eindhoven University of Technology. His research interests center on sitmuli-reponsive functional organic materials and devices.

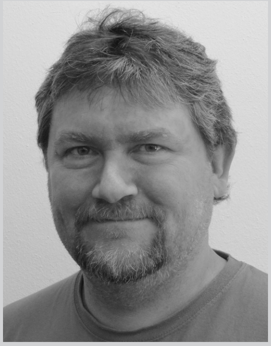

Michael Debije received an M.Sc. degree in High-Energy Physics from Iowa State University, Ames, Iowa in 1994 and a Ph.D. in Biophysics from the University of Rochester, NY, USA in 2000. After completing a postdoc at the Interfaculty Reactor Institute at the Delft University of Technology in the group of John Warman in 2003, he joined the staff of the Functional Organic Materials and Devices group under Prof. Dick Broer at the Eindhoven University of Technology, and is responsible for the Energy cluster within the program.

 © 2017 The Authors. Published by WILEY-VCH Verlag GmbH & Co. KGaA, Weinheim

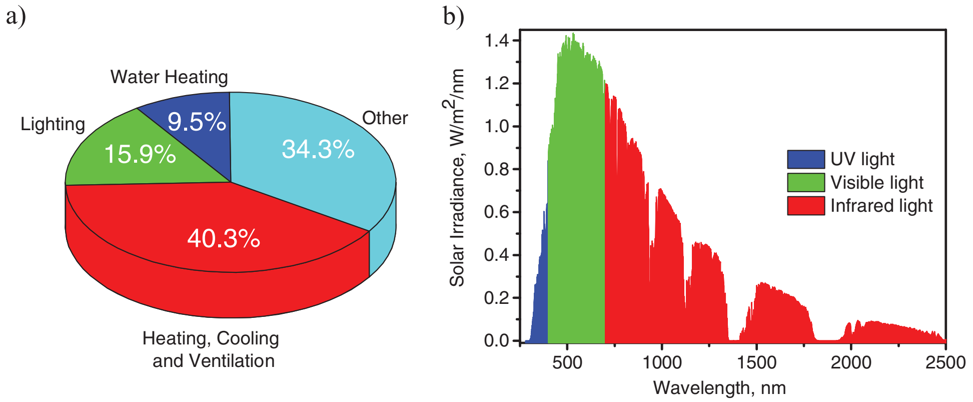

Figure 1. a) U.S. Buildings Energy End-Use in 2008.[2] b) Solar spectrum on Earth (Data taken from National Renewable Energy Laboratory).

transparent for the rest of the wavelengths as described above. In a focal conic alignment, which consists of aligned molecules where the helical structure is preserved but tilted with respect to the substrate, results in more scattering of the incident light as the refractive index changes continuously from the top to the bottom of cell. In the homeotropic alignment, where molecules extend perpendicular to the substrate, the layer is transparent to all the wavelengths of light.

Apart from LCs, we also briefly discuss other organic materials originally intended for control of visible light that could be adapted to IR control elements. Furthermore, we also discuss the influence of these IR managing windows on temperature control and energy savings in the built environment. Inorganic-based window solutions, including metallic based reflective layers,[28–31] photochromic,[3,32,33] electrochromic,[3,8,34–39] and thermochromic[3,40–43] systems, plasmonic nanoparticles,[36,44–46] aerogel glazing,[47] privacy windows,[48] thin film photovoltaics,[49] and even microfluidic[50] based windows have not been discussed in this review, as they have already received considerable attention and discussion. Additionally, organic based window devices and materials primarily intended to absorb and control visible light passage, including electro-,[51–53] photo-, and thermochromic[3] windows, are also beyond the scope of this review of infrared control materials: they have already been detailed in a number of excellent review articles.

This review is separated into three parts. The first two will describe efforts in the areas of static systems and dynamic (adjustable) IR regulating elements. The final section will speculate as to some possible future research areas that are ripe for exploitation.

2. Static IR Regulating Window

We define a static IR regulating window as a window whose properties do not change with external stimuli. In other words, the infrared control is a permanent feature of the window, regardless of exterior conditions.

2.1. Absorption Based Technologies

The simplest IR control solution is to use a dye which is transparent in the visible region and absorbs only infrared radiation.[54] The shortcoming of absorbing based systems is the majority of absorbed energy is eventually re-released as heat, with approximately half the heat being radiated into the room space. A more advanced absorption-based concept is the luminescent solar concentrator (or LSC),[55] illustrated in **Figure 5**. The LSC uses dyes embedded in the polymer or glass plate which functions as the window. The dyes absorb the near IR sunlight and subsequently fluoresce at a longer wavelength. A fraction of this re-emitted light is trapped in the higher refractive index polymer or glass panel which acts as a lightguide.

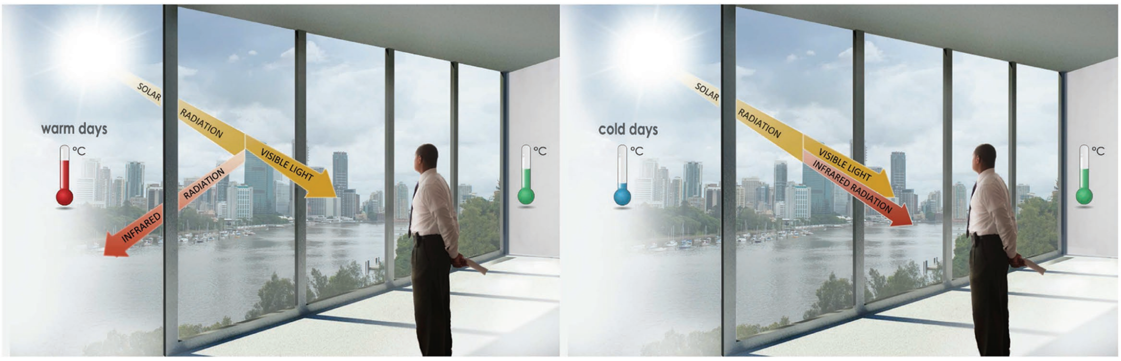

Figure 2. Schematic diagram of an ideal smart window reflecting infrared radiations in warm days (left) and allowing it to enter in cold days (right), while remaining transparent in visible region in both climate conditions.

 © 2017 The Authors. Published by WILEY-VCH Verlag GmbH & Co. KGaA, Weinheim

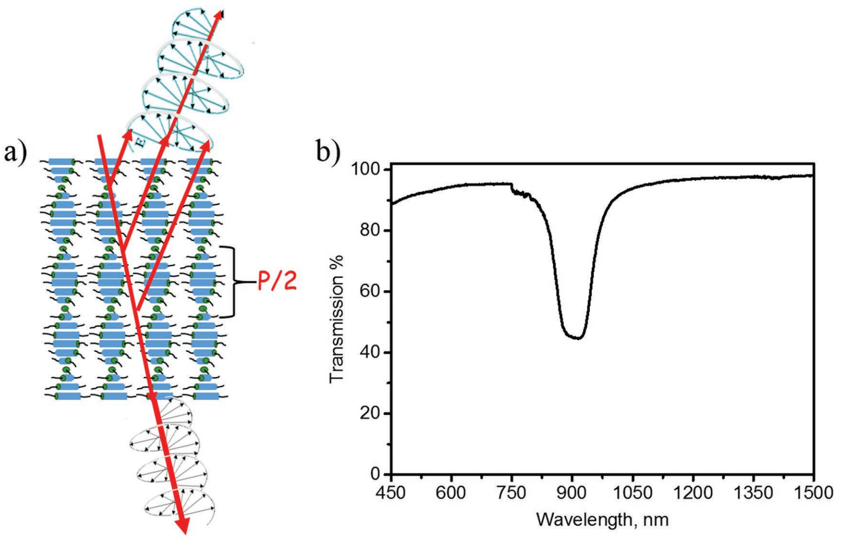

Figure 3. a) Schematic diagram showing the reflection of light by Ch-LCs: reflecting circular polarized light of same handedness. b) Typical transmission spectrum of Ch-LC.

The trapped emission light is transported by total internal reflection and only exits at the edges of the window, where it may be converted to electricity via the use of attached photovoltaic cells.[56] A significant fraction of absorbed light energy in LSCs is still lost through the top and bottom surfaces,[57] and thus still would contribute towards interior heating. In addition, the absorption ranges of the dyes in such devices are still quite limited, often with significant absorption in the visible wavelength region and thus only process a small fraction of the total incident light.[58]

2.2. Reflection Based Technologies

There is an enormous literature of Ch-LCs being employed to reflect visible light for a wide variety of (display) applications.[59] What has not been as widely exploited are cholesterics as IR control elements in transparent windows in buildings and automobiles. One of the key challenges to employ Ch-LCs as IR reflectors are their limited bandwidths when directly processed from solution. For regular cholesterics, bandwidth is restricted to around 100 nm in the IR due to the limited Δn of the LC itself (Equation (3)), which would have limited impact on controlling interior temperatures.

The range of IR wavelengths reflected may be increased by creating a broadband cholesteric reflector. There are a variety of ways in which this may be achieved. The simplest is to simply layer narrow band cholesterics of different pitches on top of one another.[60] The drawback of this is that the layers need to be laminated together in an extra processing step, and the number of layers necessary for effective IR control grow rapidly: to cover the spectrum from 750–1100 nm requires a minimum of three cholesterics layers. However, as mentioned earlier, even this will allow a maximum of 50% reflection (one handedness of the incident light), so a full six layers will be minimally required for effective IR control. The lamination of additional layers has an added potential disadvantage of introducing additional haze into the system, something that an IR reflector to be employed in a window should seek to avoid as much as possible.

A second option to obtain a broadband reflector is to create a pitch gradient in a single layer; in other words, for the Ch-LC to display a number of different pitches within the same film. A number of methods have been developed to fabricate pitch gradient broadband reflectors in the visible region,[59] some of these methods have produced reflection bands in the infrared region appropriate for window applications. The most common method for creating broadband Ch-LC reflectors was developed by Broer et al.,[61] and employed a liquid crystal mixture consisting of a nematic monoacrylate and chiral diacrylate which acts as the dopant. Exposing the acrylate mixture to a low-intensity UV light induces polymerization in a non-uniform manner.[62] The differing reaction rates of the monoacrylate and diacrylate to the UV light results in diffusion of more reactive (diacrylate) materials towards the illuminated side, generating a variation of the chiral dopant concentration between the top and bottom of the film (**Figure 6**a), resulting in a pitch gradient. In this way, a broadband Ch-LC reflecting from 750 to 1050 nm was fabricated.[62] By combining two broadband reflectors, either stacking a right- and left-handed film on top of one

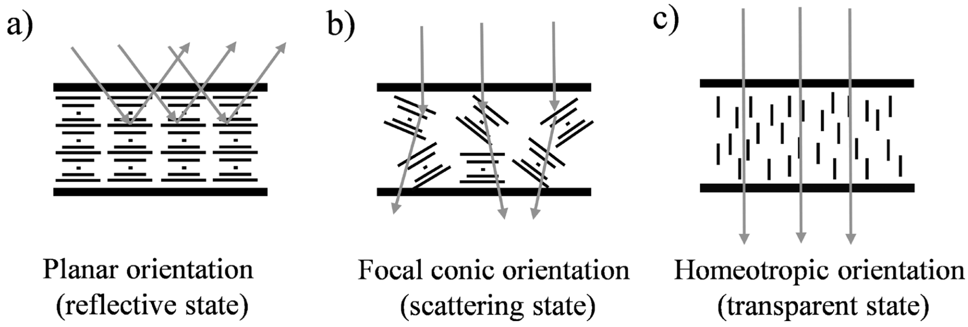

Figure 4. Different orientation of Ch-LC molecules in the cell and their optical behaviors. a) Planar orientation: reflecting a certain wavelength of light depending on the pitch, b) focal conic orientation: scattering the incident light, c) homeotropic orientation: transparent for all the wavelengths of light.

 © 2017 The Authors. Published by WILEY-VCH Verlag GmbH & Co. KGaA, Weinheim

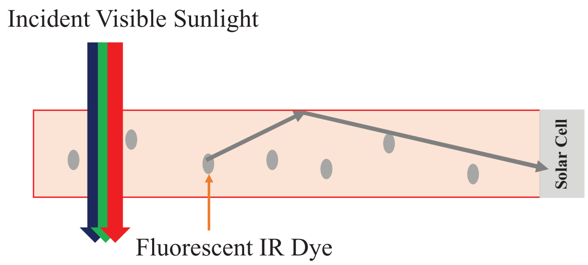

Figure 5. Schematic of the visible transparent luminescent solar concentrator. Infrared radiations from the sun is absorbed by the fluorescent IR dye and re-emitted at the longer wavelength which undergoes total internal reflection and reaches to the edge where an attached solar cell converts it to electricity.

another or by stacking two identical like-handed films separated by half-wave plate which converts right-circular to left-circular polarized light (and vice versa), a full IR reflector for sunlight (reflecting both the polarization of light) with a bandwidth of 300 nm has been produced (Figure 6b).[62] The amount of the infrared light that is reflected by this broadband Ch-LC is close to 60% of the total infrared energy while remaining transparent in the visible region (inset Figure 6b).

Using the transmission spectra of the fully-reflecting broadband cholesterics, the effects of using such films on windows in various locations across the globe were simulated, and it was determined that such reflectors could result in differences between exterior and interior temperatures of up to 6 °C compared to standard double glazing window (Figure 6c) in a Chicago environment, as an example. However, since the reflectors are permanent (static), in temperate climates the net effect of using a permanent reflector was actually negative in wintertime, when entry of IR light might be considered a benefit.[62] At steeper incident angles of light, there is a pronounced blue shift in the reflection band, approaching some 140 nm or more at incident angles around 60 degrees. Thus, care must be taken that the short band edge of the broad reflector does not shift into visible wavelength reflection range, else a pink reflection color will be evident at greater incidence angles.

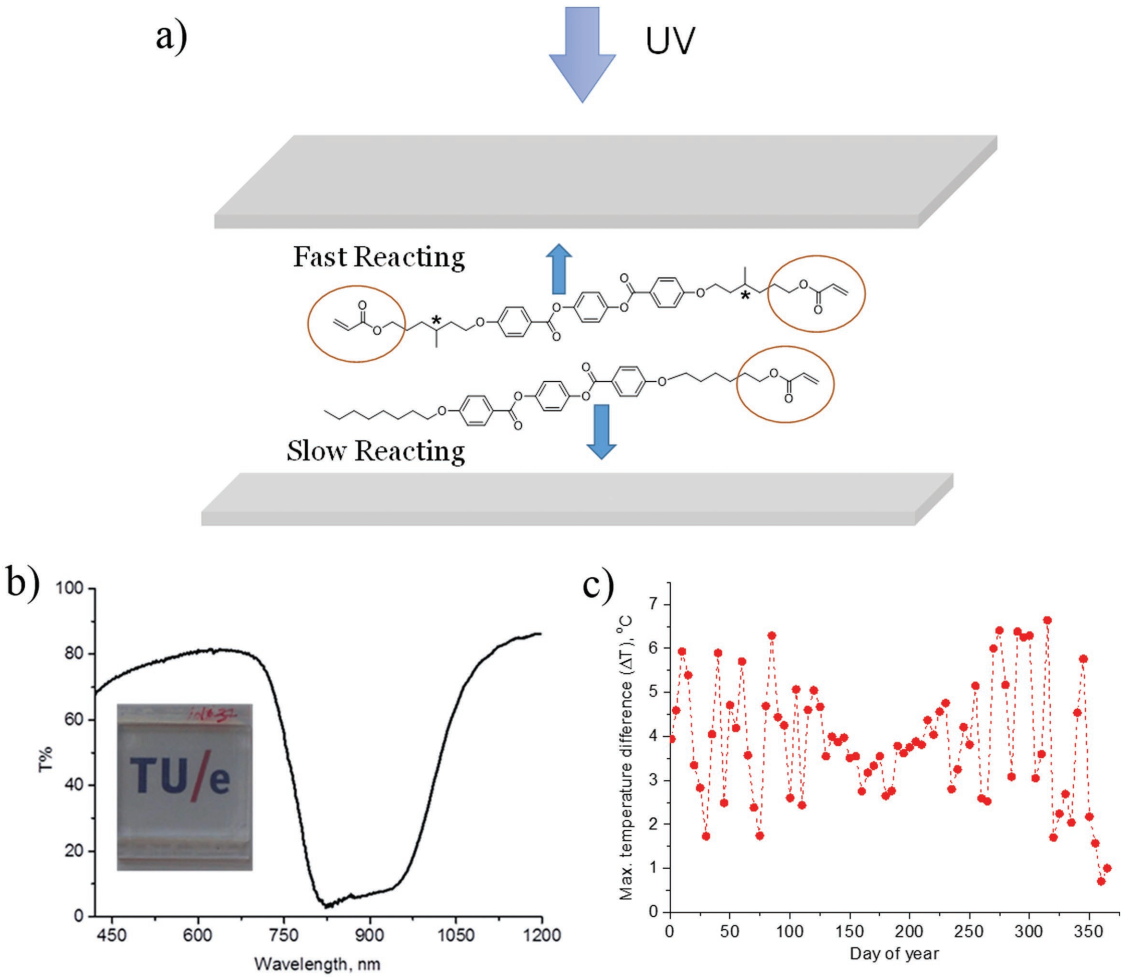

Figure 6. a) Schematic diagram showing the diffusion direction of different monomers in the film during polymerization in the cell. b) Cell transmission spectrum of right- and left- handed films superimposed on each other (inset: photograph of the sample demonstrating the transparency in the visible region). c) Predicted decrease in interior maximum temperature as a function of the day of the year for superimposed right-and left-handed cholesterics compared to standard double glazing window for an office room in Chicago, USA. Reproduced with permission.[62] Copyright 2014, The Royal Society of Chemistry.

Another method to fabricate the broadband reflector is developed by Zhang et al. They fabricated an ultra-broadband reflector using the properties of so-called layered smectic A (SmA)-like short-range ordering (SSO) structures. SSO structures are formed just above the SmA to cholesteric transition temperature where the layers start to twist and consist of very large pitches. The LC mixture was photo-polymerized just above the SmA-Ch transition temperature to induce diffusion of the nematic (diacrylate) molecules toward the illuminated side (**Figure 7**a), resulting in a gradient from cholesteric to SmA-like short-range ordering structures (Figure 7b), forming the ultra-broad reflector which reflects light from 780 to 14000 nm (Figure 7c).[63] Using two layers of opposite handedness (reflecting 100% of total infrared energy), such an ultra-broadband reflector could have a significant impact on energy savings in the built environment.

Chen et al. made a broadband infrared reflector reflecting light from 1000 nm to 2400 nm by polymerizing LC crosslinker in the presence of a chiral photoisomer, which was an azobenzene derivative displaying different HTPs in the cis and trans forms (**Figure 8**a).[64] Upon illuminating the monomer mixture with UV light, the trans- azobenzene isomerizes into cis. Due to absorption of UV by the azobenzene, a UV light gradient was formed through the thickness of the film. This causes the crosslinker to polymerize faster at the illuminated side compared to the bottom of the cell, resulting in diffusion of the crosslinker from the bottom to the top of the cell.[65] Upon subsequent exposure of the film to visible light, the cis-azobenzene isomerizes back to the trans- form, but only in the lightly crosslinked region at the bottom of the cell, whereas the higher crosslinked areas at the top of the cell remain in the cis-form as there is less network flexibility to allow the embedded azobenzene to isomerize (Figure 8b). Due to the presence of both cis (low HTP) and trans (high HTP) state azobenzenes throughout the thickness of the film, there is a gradient of the pitch from top to bottom, and hence there is formation of a broadband ($\Delta\lambda$ = 1400 nm) reflector (Figure 8c). However, due to absorption of the azobenzene at shorter wavelengths, such reflectors are colored and thus, are not completely transparent in the visible region.

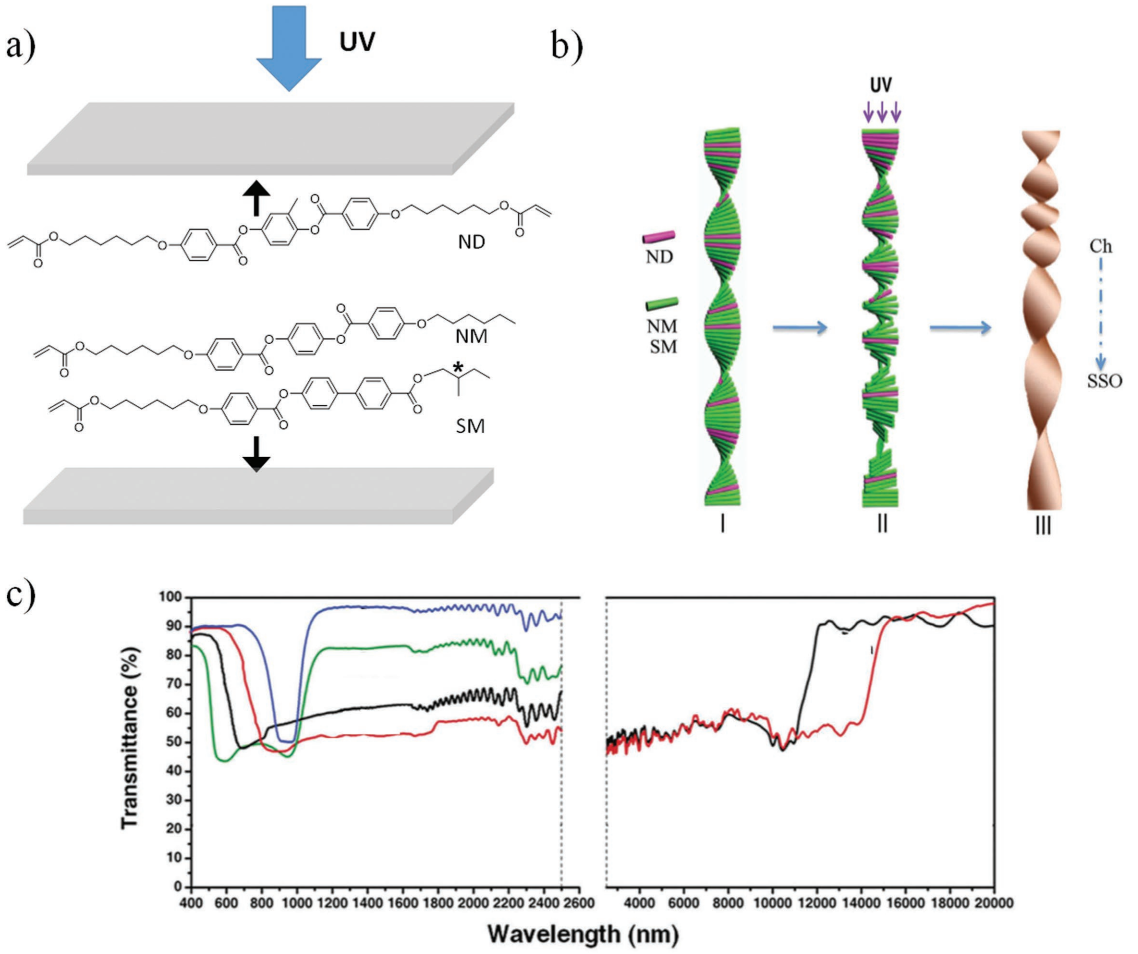

Figure 7. a) Schematic diagram of diffusion of the monomers. b) Schematic presentation of the procedure of film preparation: (I) formation of a homogenous Ch-LC thin film from a mixture of nematic monoacrylate (NM), nematic diacrylate (ND), smectic monoacrylate (SM), dye and photoinitiator; (II) UV radiation creates a ND concentration gradient inducing the intensity gradient of the SSO, and (III) a film with a pitch gradient is prepared after polymerization. c) Transmission spectra of different Ch-LC polymer films showing that the bandwidth of the reflection band can be tuned by varying the composition and polymerization conditions. Adapted with permission.[63] Copyright 2016, Taylor & Francis Ltd.

 © 2017 The Authors. Published by WILEY-VCH Verlag GmbH & Co. KGaA, Weinheim

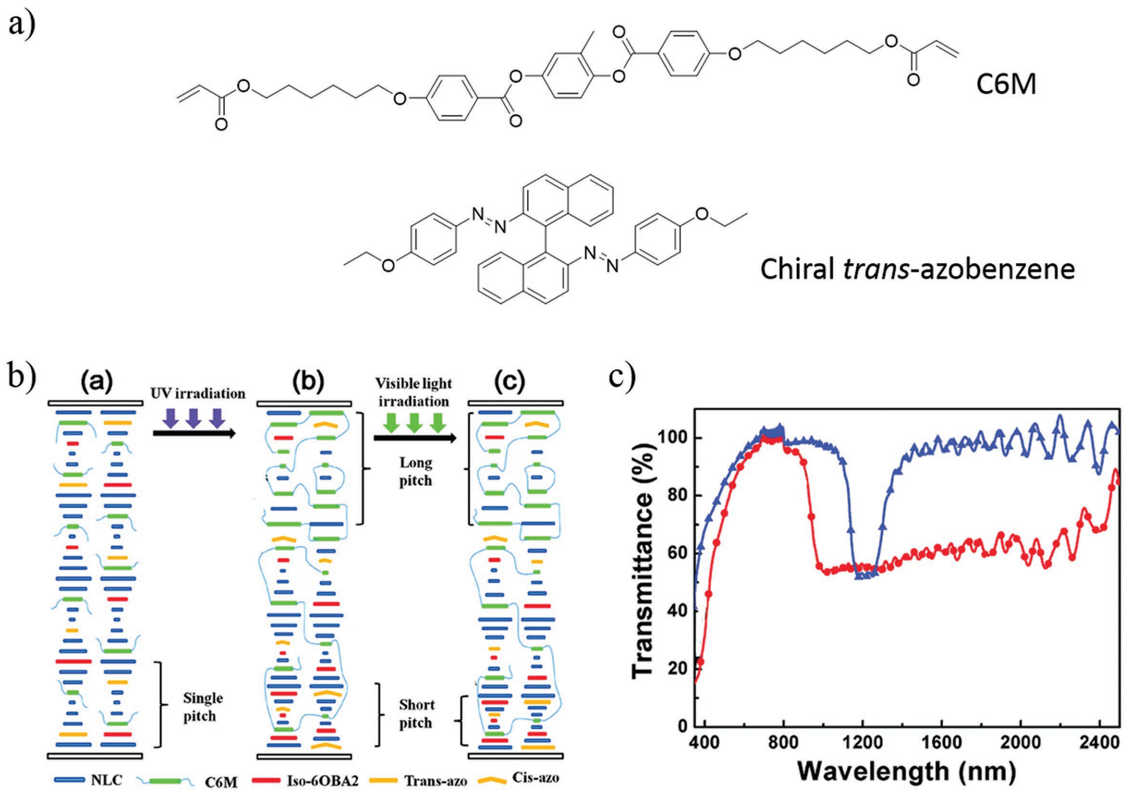

Figure 8. a) Molecular structure of the crosslinker C6M and chiral trans-azobenzene. b) Schematic diagram showing the principle of fabrication of broadband reflector using chiral azobenzene in polymer-stabilized Ch-LC. c) Transmission spectrum Ch-LC film before (blue) and after illuminating the UV-light (red). Adapted with permission.[64] Copyright 2013, The Royal Society of Chemistry.

Gao et al. fabricated IR reflecting films by blending cholesteric side chain liquid crystal polymers (ChSCLCPs) with the glass laminating transparent material ethylene-vinyl acetate (EVA) copolymer.[66] ChSCLCPs were used for window application due to their thermal stability and easy processability. ChSCLCPs of different pitches with reflection bands centered at 1000 nm (PI), 1400 nm (PII) and 1800 nm (PIII) were blended with EVA and stacked together (**Figure 9**). The film was then heated and compressed to thermally diffuse the different pitches into each other so that a continual infrared-broadband reflector could be achieved. An energy conservation efficiency of 40.4% was determined by calculating the change in the absorbed energy of the model house with and without the IR reflector. A temperature difference of 3 to 4 °C was observed using these materials compared to window consisting of only two layers of a polyethylene terephthalate film laminated with EVA. Peeling strength of the EVA/ChSCLCPs film was measured to be the same as pure EVA laminated film, which makes the mix suitable for practical application. Unfortunately, the scattering of visible light was relatively high, impairing vision of objects through the window.

Apart from LC materials, distributed Bragg reflectors using purely organic materials can also be made,[67] but in general the difference in refractive indexes of the organic materials are too low to make effective reflectors without a great number of layers. Hybrid organic/inorganic distributed Bragg reflectors are also an option, for example using three alternating pairs of sputtered CF_x/TiO_x,[68] or $CF_x/CF_x(Au)$ resulting in reflection bands 400–500 nm broad.[69]

Organometallics display reflective properties in the IR, including copper phthlocyanines[70] and chlorophyll,[71] although the cause of reflection of IR by the latter has not been confirmed; it may well be a supramolecular or other structure in the leaf that is actually responsible for the reflection.[72] More exotic structures are found in nature, such as fractal superlattices that can reflect a broadband of IR light, although they often reflect components of visible light as well.[73]

3. Dynamic IR Regulating Window

In areas of the world under constant heat stress, such as the Middle East, continual rejection of IR light could be quite desirable. However, in more temperate zones, such as the Midwestern United States, it may be more appropriate to employ materials capable of reflecting unwanted IR light in periods of high environmental temperatures in summer months, but allowing passage of IR light in periods when warming from external sunlight would be desirable, such as spring, autumn, and winter (Figure 2). This section describes several responsive systems that regulate their properties to reflect/transmit the IR light to balance the indoor temperature conditions throughout the year. The trigger for this switch could be a variety of stimuli: electrical field, temperature, or perhaps even the intensity of incident light. However, electric fields, used to regulate window properties manually, and temperature, used to regulate window properties autonomously

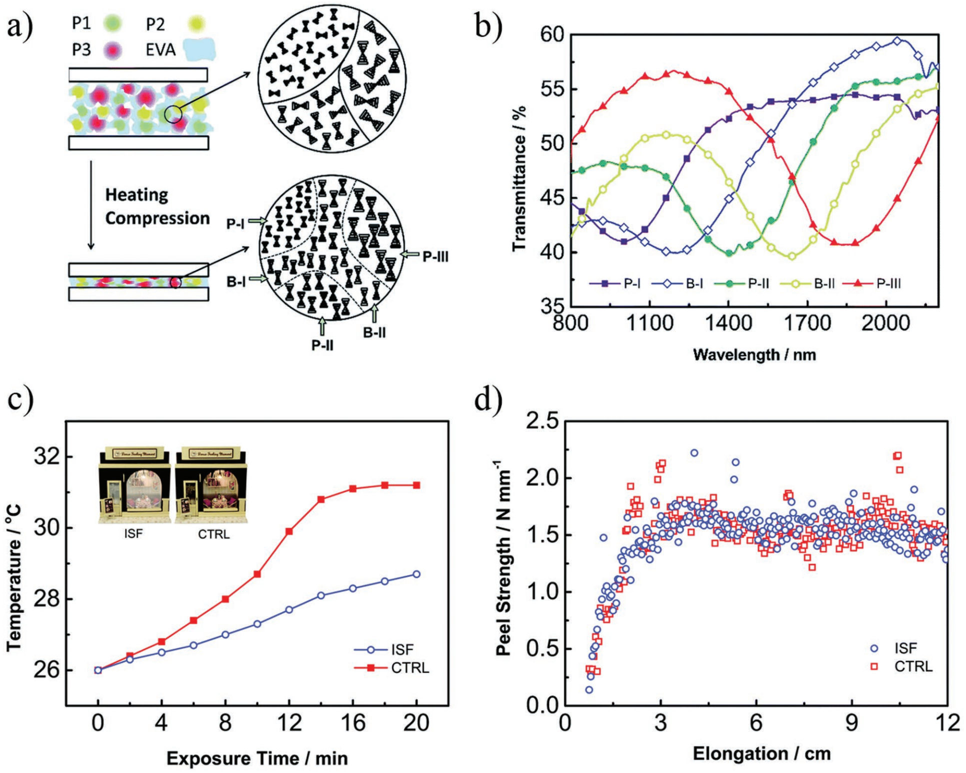

Figure 9. a) Schematic showing the fabrication of IR shielding film with different pitch length shown as PI, PII and PIII. b) Transmission spectra of the film in different regions. c) Change in temperature of the model house under exposure of sunlight. Here, ISF: the house with the IR shielding film attached to its window, CTRL: the house with two layers of Polyethylene terephthalate film laminated with EVA attached to its window as a control experiment (inset picture is the photo of the model houses). d) Peeling strength results of the samples (ISF) and control film (CTRL). Reproduced with permission.[66] Copyright 2016, The Royal Society of Chemistry.

with environment changes, are the most common triggers for the window application. We will discuss these different aspects under separate headings, with the last approach (intensity of light as trigger) delayed until part 3, future options.

3.1. Electrically Responsive Window

Before we discuss infrared regulators based on LC materials, it is important to understand the anisotropy of the LC molecules. In simple terms, a positive dielectric ($+\Delta\varepsilon$) anisotropic rod-like LC molecule exhibits dipole moment along the molecular axis, whereas negative dielectric ($-\Delta\varepsilon$) anisotropic molecules display dipole moments perpendicular to the main molecular axis. Therefore, on application of an electric field between two glass plates of a planar aligned LC cell, LC molecules with $+\Delta\varepsilon$ undergo homeotropic orientation, in contrast to $-\Delta\varepsilon$ LCs, which remain undisturbed in their initial planar state under the same electric field. In this review, the anisotropy of the molecule should be considered as positive, if not mentioned specifically.

3.1.1. Scattering and Absorption Based Technologies

A number of switchable privacy windows based on polymer dispersed liquid crystals (PDLC) and polymer stabilized liquid crystals (PSLC) have been designed to control visible light. In PDLC based windows, micrometer sized LC droplets are dispersed in a polymer matrix, whereas PSLC windows employ composites of non-polymerizable LC mesogens and a polymer network. (**Figure 10**). Two modes are generally available in these privacy based windows: a normal mode, where the window is in a scattering/privacy mode in the 'off' state, and a reverse mode, where the window is transparent in the 'off' state.[74] Generally, the reverse mode is preferred, since in case of 'power failure', the window remains in a transparent state rather than in a scattering state. These privacy windows which are focused on visible light control have been reviewed, and beyond the scope of this work.[75–80] PDLC based windows are already commercially available[78] but mostly used for indoor purpose. Privacy based windows have also been developed using smectic/cholesteric liquid crystal phases where the window can be switched reversibly between transparent (planar and homeotropic) and scattering (focal conic) states.[81–83]

A recent nanoparticle/polymer construct using indium tin oxide (ITO) nanoparticles and polythiophenes showed electrochromic behavior and demonstrated promising characteristics in being able to reversibly control IR ingress above 800 nm by applying just 1.25 V (**Figure 11**).[84] However, there is evidence of considerable absorption in the visible light range. A similar situation is seen in Co(II)-based metallo-supramolecular polymer systems.[85] Other options, such as organic

© 2017 The Authors. Published by WILEY-VCH Verlag GmbH & Co. KGaA, Weinheim

REVIEW

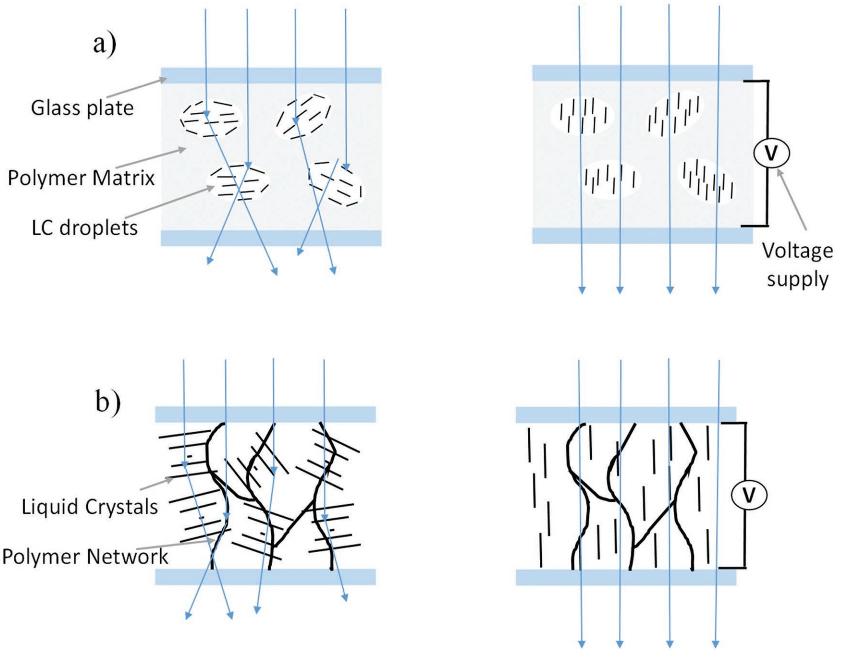

Figure 10. Schematic diagram of normal mode a) PDLC and b) PSLC based window showing scattering of light in 'off' state (left) and transparent in presence of electric field (right).

planar (reflective state) to homeotropic (transparent state) orientations (Figure 4). A number of Ch-LC based narrowband reflectors, both in visible and infrared regions, have also been developed which can tune the position of reflection notch within a limited wavelength region in presence of an electric field.[90,91] Such narrow bandwidth reflectors can influence only a minor fraction of infrared light, and would have only limited impact on interior temperatures.

Binet et al. have fabricated broadband infrared reflectors by inter-diffusing two layers of different pitch lengths, consisting of polymer stabilized siloxane and non-reactive LC mesogens.[92] Interestingly, the reflection band obtained is not simply the sum of the reflection band of the individual layers and can be controlled by UV curing conditions (**Figure 12**a). These siloxane-based layers can be further switched between planar and homeotropic states by application of an electric field, and therefore can be used to make switchable IR reflectors (Figure 12b). The bandwidth of the broadband, centered around 1810 nm, is 310 nm. However, this can further be tuned to an onset wavelength 700 nm to have greater impact on the energy savings.

electrochromics, appear confined to visible wavelengths.[86–88] An electrically responsive system which functions by redox reaction of the organic based ionic liquid crystals to control visible light passage has also been studied,[89] but to our knowledge no subsequent efforts in the IR region have been reported.

3.1.2. Reflection Based Technologies

Using uniform pitch Ch-LCs, a number of narrowband reflectors with bandwidths of 100–200 nm have been fabricated. Upon exposure to an electric field, they could be switched between

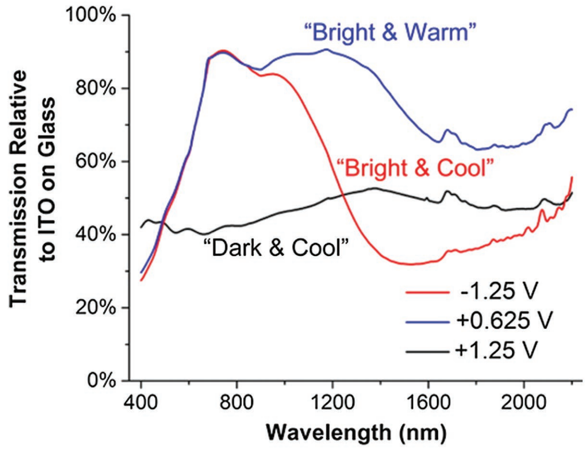

Figure 11. Transmission spectrum of the poly (3,3-dimethyl-2,2-bithiophenyl)-ITO film showing that different fraction of IR and visible light can be control by applying different voltage. Reproduced with permission.[84] Copyright 2016, American Chemical Society.

Recently, we have fabricated an electrically switchable IR reflector using a low molar mass non-reactive LC mesogens in a polymer stabilized network.[93,94] By using a mixture of reactive chiral mesogens and non-reactive materials in the presence of a UV-absorbing dye, the reactive fraction in the LC mix begins to polymerize at the side being illuminated. This causes a diffusion of chiral reactive mesogens to the polymerized region, which in turn leads to formation of the pitch gradient throughout the thickness of the film. This results in a cell containing a skeletal network of polymerized materials to act as a 'memory' state for the unpolymerized materials, which at rest will assume the order instilled by the polymerized network. Upon exposure to an electric field, the non-polymerized molecules can reorient to the homeotropic state (**Figure 13**a). Since there is not enough polymerized network to form a reflection state by itself, the reflective state is lost and the system becomes purely transmissive. Upon removal of the field, the LCs relax and are directed by the polymerized skeleton to resume their original orientation and reflective state.[93,95] Using this technique, we have fabricated a broadband infrared reflector which reflects light from 700 to 1400 nm that remains transparent in the visible region.[93] On application of 8.6 V μm^{-1}, the LC molecule arranges themselves homeotropically, which results in transparency in the visible as well as infrared region (Figure 13b).

To better understand the relation between global climate conditions and potential energy savings using these particular switchable IR control windows, simulations were carried out assuming three different climates: (1) Abu Dhabi, United Arab Emirates (a warm climate) (2) Amsterdam, the Netherlands (a cold climate) and (3) Madrid, Spain (a moderate climate). For

 © 2017 The Authors. Published by WILEY-VCH Verlag GmbH & Co. KGaA, Weinheim

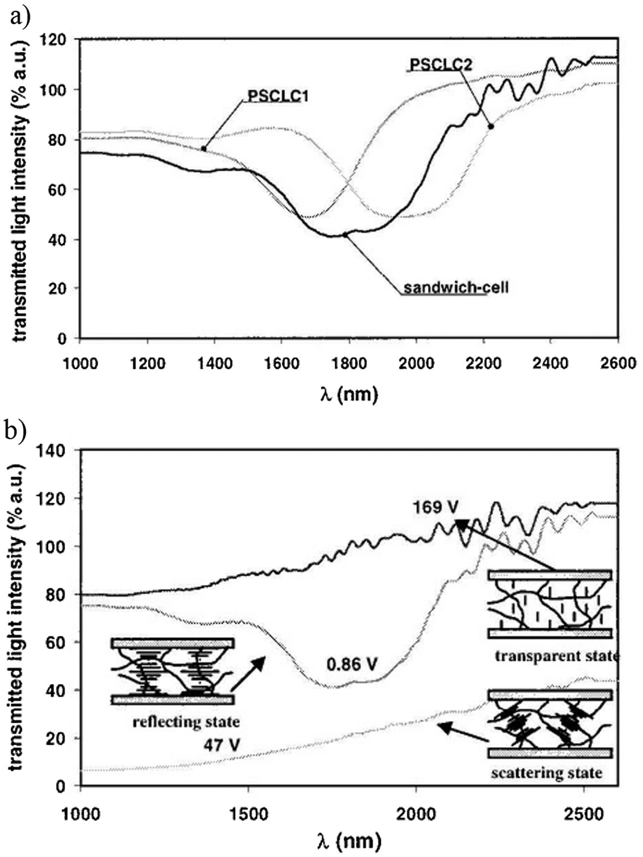

Figure 12. a) Transmission spectra of films PSCLC1, PSCLC2 and UV-cured sandwich cell of PSCLC1 and PSCLC2. b) Transmission spectra of sandwich cell upon application of different voltage showed that polymer stabilized Ch-LC can be switched from reflection (0.86 V) to scattering (47 V) to transparent state (169 V). Adapted with permission.[92] Copyright 2001, AIP Publishing.

each climate condition, we calculated the energy consumption of a standard office building equipped with double glazing windows, and compared its performance to a building using either a broadband static or dynamic IR reflector; the latter window was to switch to the transparent state whenever the external temperature dropped below 22 °C. As shown in Figure 13c, it was found that static IR reflectors performed best in warm climates like Abu Dhabi, as IR needs to be reflected almost constantly throughout the year. Double glazing was found to be optimum in cold regions like Amsterdam where IR radiations were desirable throughout the year. The performance of the switchable IR reflector was most favorable in the moderate climate of Madrid. It was predicted that the switchable IR reflectors could save more than 12% on the building's energy consumption, whereas static IR reflectors could save only 3% on energy compared to standard double glazing window. It is important to note that the energy required to switch the window from reflective to transparent states was not included in this initial simulation study; the power/voltages needed to switch the window were relatively high. In addition, switchable IR reflectors were revealed not to be user friendly in moderate climate condition, as it is difficult to decide whether to keep window in the reflection or transmission state.

To reduce the switching voltage/power and fabricate a more user-friendly window, we employed a negative dielectric anisotropic ($-\Delta\varepsilon$) LC and the technique of designing an ion-accepting linker molecule suggested by the work of White et al.[96] The (poly)ethylene glycol (PEG) based linker molecule (**Figure 14**a) enhanced the collection of positive charges and increased the distortion of the partially polymerized LC network under an electric field, resulting in compression of pitches at negative electrode whereas at positive electrode, it expanded (Figure 14b).[97] In a cell of sufficient thickness so that the number of pitches is relatively high, there are pitches in the middle of the cell which remain undisturbed by an applied field. So, the combination of expanded, compressed and unchanged pitches results in formation of a 1100 nm broad reflection band spanning from 700 to 1800 nm with minimum impact on visible light transmission, while simultaneously reducing the voltage required to switch the system to around 1.2 V μm^{-1} (Figure 14c,d).[97] By applying 0 to 1.2 V μm^{-1}, a bandwidth from 120 nm to 1100 nm can be tuned, corresponding to rejection of 8 to 45% of the total incident infrared energy. Using this system, one could select the fraction of infrared to be reflected depending on the external climate conditions so that a comfortable indoor temperature can be maintained throughout the year. Calculation shows that more than 12% (including the energy to switch the window) of the total energy used in the built environment can be saved in the Madrid climate using the fabricated bandwidth tunable reflector compared to a standard double glazing window.[93,97]

Hu et al. demonstrated a broadband reflector in the infrared region by using a charged chiral ionic liquid in a Ch-LC mixture (**Figure 15**a).[98,99] Due to diffusion of the charged chiral dopant in a negative dielectric anisotropic ($-\Delta\varepsilon$) Ch-LC mixture in the presence of DC electric field, a pitch gradient is created throughout the thickness of the cell, leading to formation of the broadband (Figure 15b). At the same time, DC electric fields also introduce disorder in alignment of the LC molecules, resulting in light scattering. To reduce scattering, an AC electric field was applied and turned off quickly just after application of a DC field to obtain the planar orientation of the Ch-LC mixture. On further applying the reverse DC bias with a suitable field, broadband was switched to narrowband as a result of uniform distribution of the chiral dopant throughout the thickness of the film (Figure 15c). Similar mechanisms using chiral ionic polymer networks in $-\Delta\varepsilon$ Ch-LC has also been used to generate tunable broad-to-narrow bandwidth reflectors upon application of electric fields.[100] These systems can be further optimized for real applications by tuning the broadband to the near infrared region and improving the transparency in the visible region.

Xiang et al. have designed narrowband tunable reflectors which can modify their reflection positions over a wide range by utilizing the properties of the heliconical Ch-LC state.[101] By systematically increasing the electric field, a blue shift in the reflection band with changes in the positon of reflection notch from 1100 to 300 nm was observed. The limitation of these systems is that they reflect only a fraction of infrared energy because of their inherent narrowband nature, which results in limited impact on indoor temperature. However, this system can be improved by stacking multiple switchable cholesteric windows for full spectral coverage, but this would be a considerable design and manufacturing challenge.

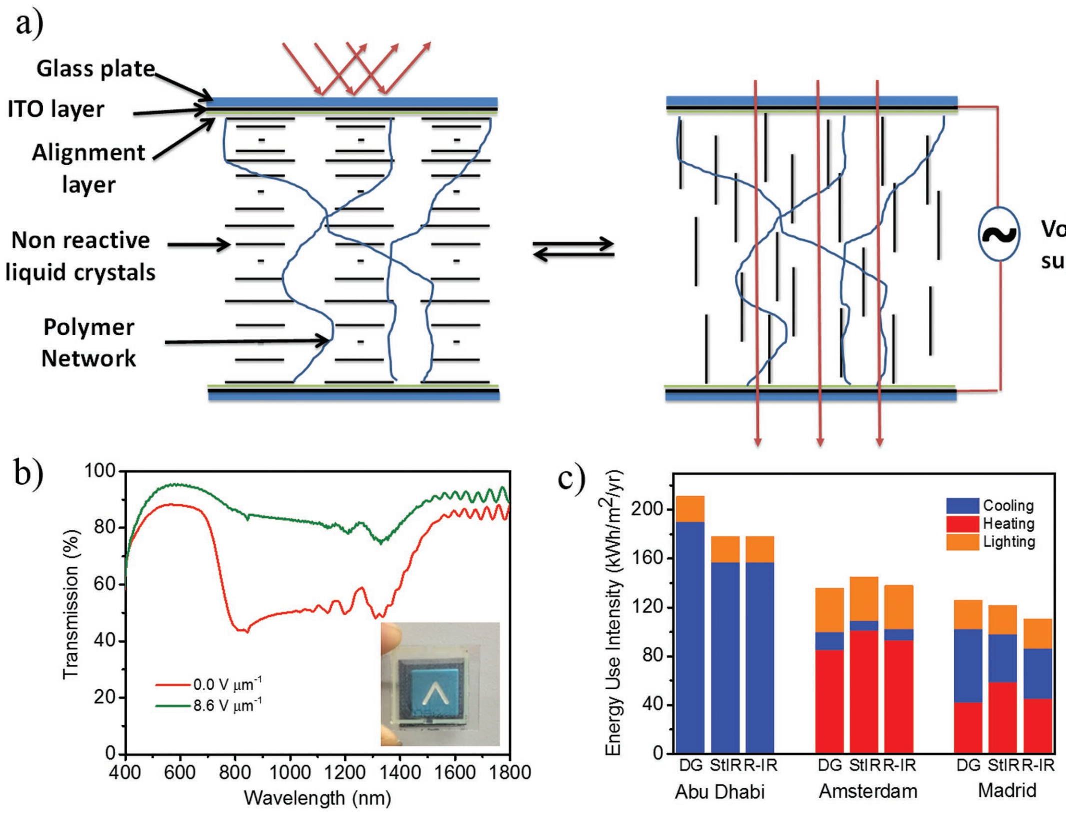

Figure 13. a) Schematic diagram showing the planar (left) and homeotropic (right) states on applying an electric field. b) Transmission spectrum of cholesteric gel in reflective and transmissive states at 0 V µm^{-1} and 8.6 V µm^{-1} (inset: photograph of the sample demonstrating transparency in visible region). c) Comparison of energy use intensity for a normal double glazed window (DG), static IR reflector (StIR) and switchable (responsive) infrared reflector (R-IR) for three different climates. Adapted with permission.[93] Copyright 2015, Nature Publishing Group.

3.2. Temperature Responsive Windows

3.2.1. Scattering and Absorption Based Technologies

Hydrogels based on poly(N-isopropylacrylmide) have been used to control visible light at elevated temperatures.[102] The hydrogel film of a specific thickness is transparent at room temperature but as the temperature rises above the lower critical solution temperature (LCST), the film starts scattering, resulting in less light entering the interior spaces (**Figure 16**). Similar behavior has also been demonstrated using hydroxypropylcellulose based hydrogel.[103] Photo-thermotropic hydrogels can alter their transparency and reflective properties when illuminated by sunlight.[104] In these systems, a material such as graphene is dispersed in a hydrogel network. The graphene enhances uptake of energy which is stored in the water while maintaining transparency. Upon an increase in the water temperature to a specified level, the network breaks apart, resulting in a semi-reflective scattering state and loss of transparency.

Phase change materials (PCMs) have most often been applied to opaque elements but there are examples of application in window systems.[105] The PCM takes advantage of the latent heat storage potential in the phase transition between the liquid and solid states. This transition can be used for heat storage and by increasing the thermal inertia of the window, which both aid in maintaining interior temperatures. The transmission of light through the window for both the solid and liquid states have, however, often proven to be only modest using commercially available materials[106] and often resulted in scattering, and thus completely disrupt the exterior view.[107] While the commercial materials tend to be inorganic based, there are organic PCMs that could conceivably fill this role,[108] but it remains to be seen if they could be considered for use in window applications.

The phase transition from SmA to cholesteric at elevated temperature has been used to fabricate a scattering-based visible light controlling window. Homeotropic orientation of SmA LC at lower temperatures results in the transparent state of the window whereas at elevated temperature, LC changes to cholesteric phase, leading to change in the orientation of the molecules to a focal conic state, resulting in scattering of light.[109] A particularly interesting system employed a printed organic-based photovoltaic with embedded inorganic VO_2 nanoparticles that would change transmission upon exposure to increased temperature.[110] While the device interfered with visible light, the authors were clear in their desire to target the NIR spectrum for purposes of interior temperature control while simultaneously generating electricity from visible light. If this could be accomplished by using embedded organic materials absorbing outside the visible range, this could be a very attractive device.

An exotic example of a dynamic heat control element using organics are holographic polymer dispersed liquid crystals.[111]

REVIEW

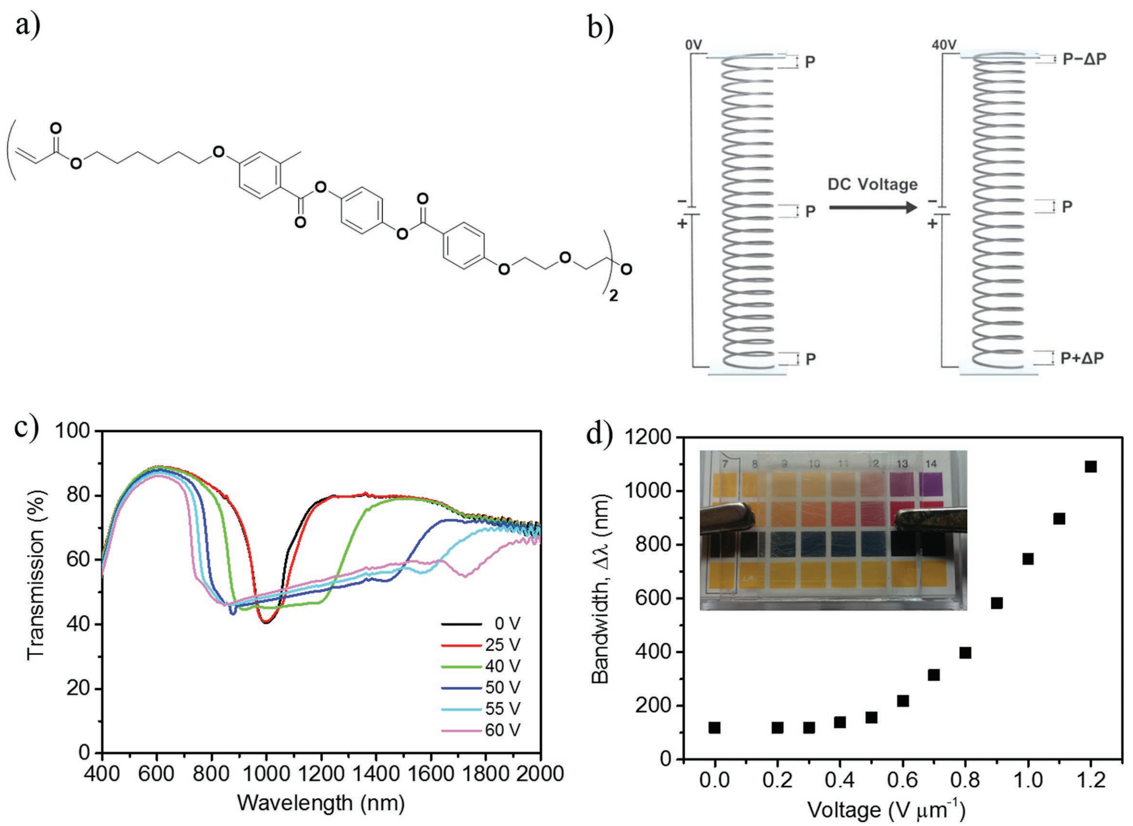

Figure 14. a) Molecular structure of the twin molecule used to fabricate the smart tunable window. b) Schematic diagram showing the working principle. c) Transmission spectra of Ch-LC mixture in 50 μm cell on application of 0–60 V. d) Bandwidth of the Ch-LC with respect to the applied voltage per micron thickness of the cell (inset: photograph of the IR reflector showing the transparency for the visible light at 0 V). Reproduced with permission.[97] Copyright 2016,The Royal Society of Chemistry.

These films are quite angularly dependent, and while can be efficient on excluding IR light at specific incident angles, they are generally transparent at other incident angles, which makes control of diffuse light difficult. The report demonstrated a change of 15% in transmission through a sample cell as the temperature approached 30 °C.

3.2.2. Reflection Based Technologies

Yang et al. developed a method which would be quite effective if used as a temperature responsive window.[112] The device used a chiral dopant which increases its helical twisting power in a polymer stabilized Ch-LC upon increasing the temperature (**Figure 17**a). At higher temperatures, the LC molecules will not be able to twist as much in areas of high polymer network density due to the anchoring effect from the polymer network, whereas in the low polymer network density region, the LC molecule will be able to twist to accommodate the increased HTP of the chiral dopant.[113–115] Therefore, via the combination of lower (larger wavelength reflection band) and higher (smaller wavelength reflection band) twisting in a single sample, a broadband infrared reflector was formed at higher temperature (Figure 17b). At lower temperatures (around 5 °C), the device reflected light from 2050–2400 nm. As the temperature increased to 40 °C and 50 °C, this polymer stabilized system reflected light from 950–2400 nm and 800–2400 nm, respectively (Figure 17c). This configuration allows the maximum amount of infrared energy to enter in winter while reflecting a large amount of solar infrared energy in summer, so that it would save on both heating and cooling energy demands in the built environment.

Natarajan et al. have demonstrated a remarkable blue shift of a narrow band reflector from 2300 to 500 nm on increasing the ambient temperature, as shown in **Figure 18**.[91] This thermal tuning was attributed to the pre-transitional effect of smectic-to-cholesteric phase transition. As the infrared energy from the sun increases continuously from far to near infrared region, with this method a lesser-to-greater amount of infrared energy can be reflected on continual increase in temperature. However, the tuning temperature is not ideal for window applications (from 30–60 °C), but could be further optimized. The total amount of energy reflected is limited as only a small bandwidth of IR light is reflected, but by optimizing the LC mixture it could be useful where the change in the temperature is not extreme.

4. Outlook and Future Challenges

So far, we have discussed different kinds of infrared regulating devices using primarily organic materials. Many of these

 © 2017 The Authors. Published by WILEY-VCH Verlag GmbH & Co. KGaA, Weinheim

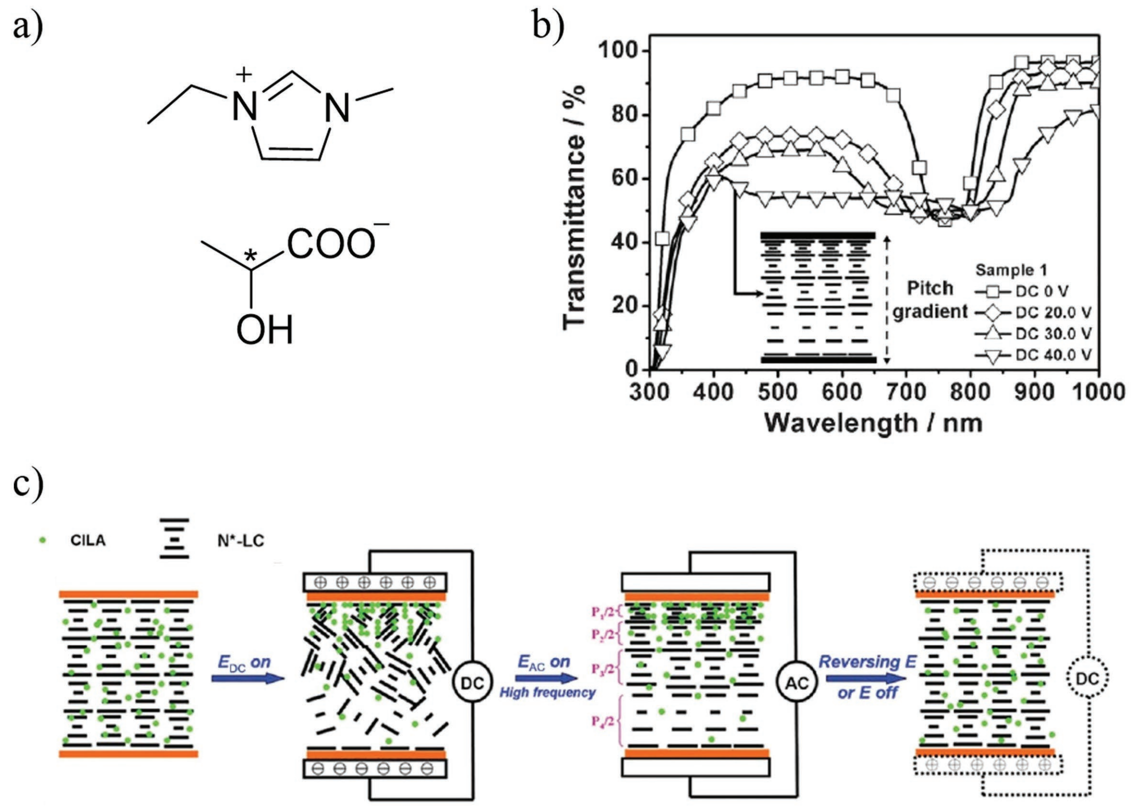

Figure 15. a) Molecular structure of the ionic chiral dopant used. b) Transmission spectra of the cell upon application of different DC voltages. c) Schematic drawing showing the mechanism of fabrication of broadband from narrowband reflector and vice versa. Adapted with permission.[99] Copyright 2010, John Wiley & Sons.

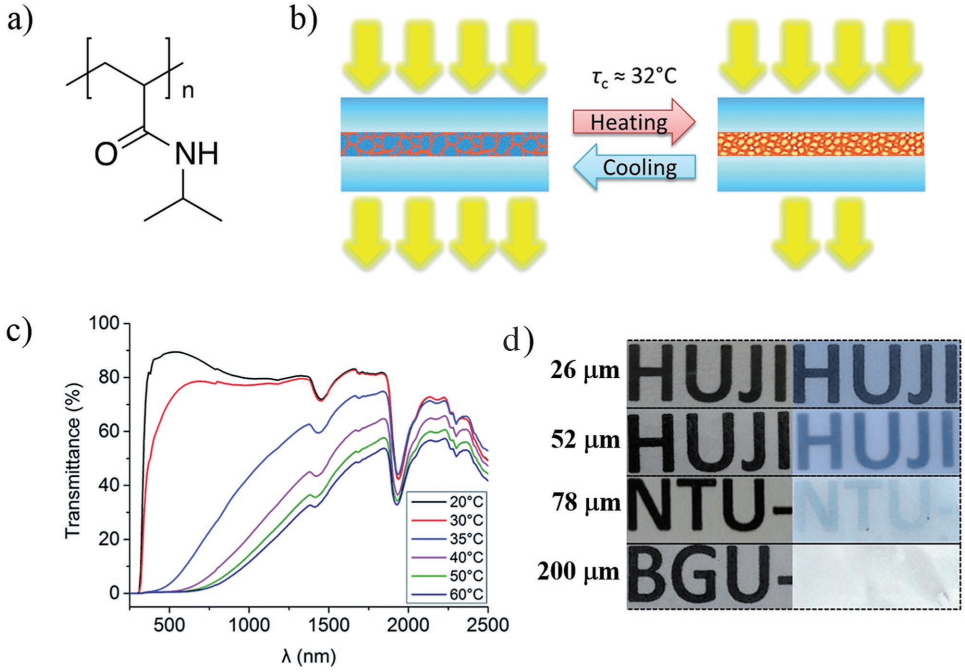

Figure 16. a) Molecular structure of the poly(N-isopropylacrylmide) hydrogel. b) Schematic diagram showing the change in solar transmittance below and above LCST (τ_c). c) Temperature dependence transmission spectra of the sample of 200 μm thickness. d) Hydrogel of different thickness at room temperature (left) and 35 °C (right). Reproduced with permission.[102] Copyright 2014, The Royal Society of Chemistry.

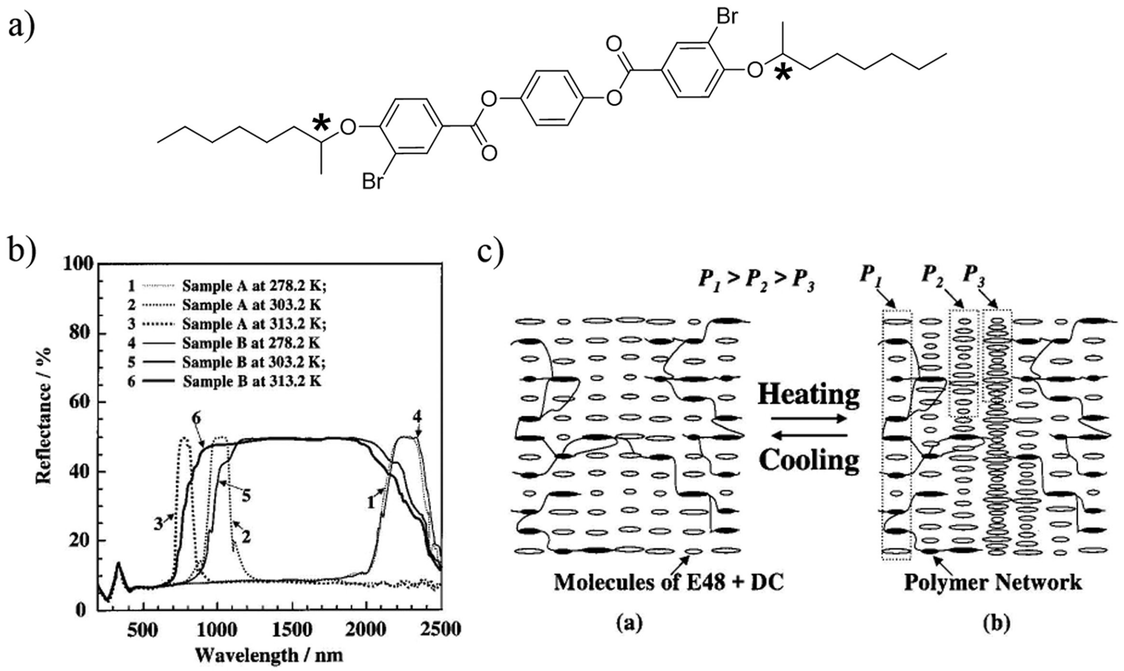

Figure 17. a) Molecular structure of the chiral dopant used. b) Temperature dependent change in transmission of cell containing polymer stabilized and non-polymer stabilized Ch-LC (Sample A and B are non-polymerized and polymerized LC mixture, respectively). c) Mechanism of formation of broadband at elevated temperature. Reproduced with permission.[112] Copyright 2003, AIP Publishing.

systems were designed for visible region and targeted for some other applications. However, the potential use of the materials as infrared control elements for buildings or automobiles were not considered in the original work. As more attention is directed towards solving the increasing energy problem, more materials can be adapted for this application. In this section we discuss some potential future options.

An interesting trigger to switch the properties of the window could be intensity of light. In the past decade, a number of light responsive chiral dopants which can change their HTP either upon isomerization or helical inversion have been used to tune the position of a cholesteric reflection notch over a wide range.[116–118] A light responsive system which can change its reflection bandwidth based on the intensities of light would be very attractive. First steps for such a responsive system in the visible and infrared regions have been taken by White et al. using a chiral azobenzene photoisomer doped in a Ch-LC cell.[119] The reflection bandwidth was increased to 1700 nm with a specific sample thickness and intensity of light. This system would be even more interesting if the effective reflection bandwidth is stable for longer duration of time and could be accomplished using a light responsive dye which does not absorb visible light. Other potential approach is to design systems responsive to multiple triggers simultaneously. As an

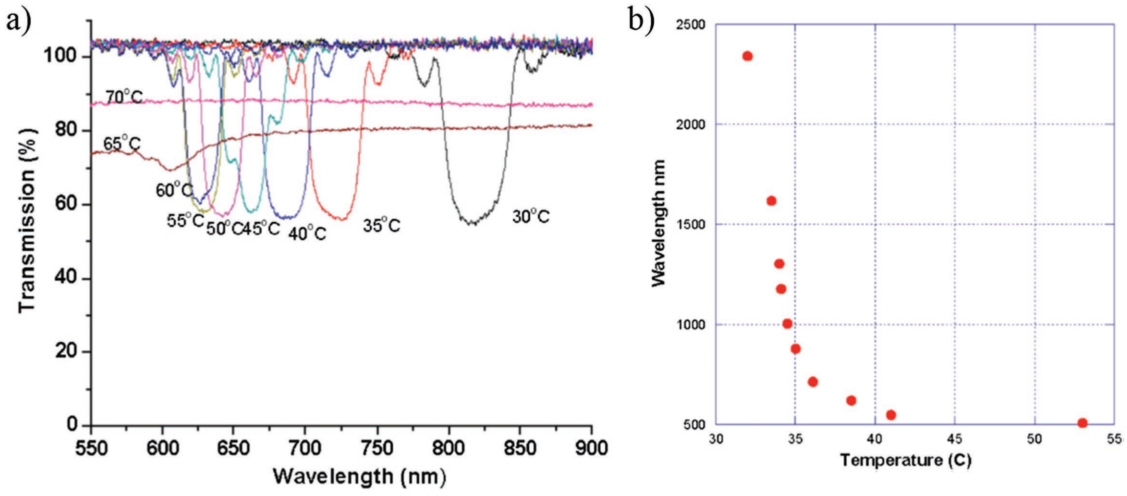

Figure 18. a) Temperature dependent transmission spectra of Ch-LC containing S811 (chiral dopant, 28 wt%) in nematic LC host. b) Change in the position of reflection notch of the cell containing 26 wt% S811 in nematic LC host. Reproduced with permission.[91] Copyright 2008, AIP Publishing.

 © 2017 The Authors. Published by WILEY-VCH Verlag GmbH & Co. KGaA, Weinheim

example of a multi-trigger system, an IR reflective matrix that may be manually tuned by exposure to an electric potential, or automatically in response to temperature increases has been produced which relies on a polymer stabilization network very finely tuned as to its crosslink density.[120] Such a system architecture would be interesting if this can be produced without scattering light in the visible region.

Responsive liquid crystal blue phases reflecting in the infrared region could also be employed for window applications. Most research carried out to date using blue phases has concentrated on controlling visible light.[121] A red shift in reflection band has been demonstrated in the presence of an electric field.[122] Recently, broadening of the reflecting bandwidth upon application of electric fields has been shown in polymer stabilized blue phase LCs in the visible region.[123] The broadening was attributed to the inhomogeneously distributed polymer network throughout the thickness of the cell, which causes non-uniform displacement of the polymer in presence of a DC electric field. Temperature-dependent blue shifts in the reflection band due to lattice distortion have also been reported.[124–126] Red shifts in the reflection band upon illuminating azobenzene doped blue phases with light have also been demonstrated in the visible region.[127,128] Furthermore, there are also number of other organic based 2D and 3D photonic materials which reflect narrow bandwidths of light, mostly in the visible region.[129,130] These materials could be adjusted to interact in the infrared region. If multilayers of these photonic materials reflecting in different wavelengths could be produced, this would allow for broad reflection bands, and could find application as a smart window.

As discussed in the previous sections, there are several advantages of using Ch-LCs for the window application but one of the main limitations of existing Ch-LC based technologies is that they reflect only one polarization of light, which leads to maximum 50% reflection of unpolarized light. Reflection can be improved to 100% by using two films of opposite handedness or inserting a halfwave plate between same-handed films.[131] Few methods have also been developed to enhance the reflection past 50% in a single film. One example is using thermal helicity inversion of the chiral dopant;[132,133] in this system, a chiral dopant is used that changes its handedness upon heating. A second example is using a wash-out procedure where non-polymerizable LC is removed from a lightly-crosslinked LC cholesteric polymer network, and the resulting empty space is refilled with cholesteric forming LCs with opposite handedness.[134–136] However, a scalable method which can reflect a broad wavelength region and both polarization of infrared light is yet to be realized.

Reflection properties of Ch-LCs, similar to all other standard Bragg reflectors, are incident angle dependent. To reduce the angular dependency of Bragg reflection based smart windows, polymer stabilized Ch-LC particles could be a good alternative.[137] Other advantages of using the polymer stabilized Ch-LC particles are that they do not require an alignment layer on the substrate used to fabricate the device. Moreover, they can be scaled up in large quantities for effective commercial application, unlike the polymer stabilized cholesteric films.[138] These particles could be dispersed in a polymer matrix and applied as a coating for existing windows. Other polymer based systems, such as using dispersed cholesteric flakes reflecting IR light, and siloxane based responsive broadband reflectors which can directly be coated on the existing windows should also be researched.[139] Other responsive polymer-based photonic materials could also be developed in the infrared region for coating purposes.[140]

To reduce the power consumption of the various electrically switchable cholesteric-based LC windows, some form of bistable system could be envisioned.[141] Bistable systems consume energy only to switch from one state to another and no energy is required to remain in any one state. Earlier reports have provided some examples, such as the switchable scattering-to-clear state system using the cholesteric/SmA phase.[82,141–145] Of course, these mixtures often rely on polymer stabilization, with its concomitant challenges in reducing scattering in the visible spectrum.

Another future application could combine the function of the cholesteric-based switchable broadband reflective window with an energy generating window.[146,147] Most of the reported organic and inorganic materials based energy generating window absorb in the visible region.[148–151] These energy generating windows relied on luminescent dye absorbing incident light and re-emitting it at a longer wavelength, based on the principle of LCS. In this modified device, rather than deploying fluorescent dyes in the nematic LC host, the fluorophores are instead introduced into a polymer stabilized broadband IR reflecting Ch-LCs.[93] It was demonstrated that the visible-light control aspects of the dye are preserved (and thus the potential to generate an electrical current), as is the ability to control IR light. To make this device commercially viable the level of visible light control need to be improved.[152,153] It is also important to show significant potential for electricity generation (at least enough to switch the window) by using IR dyes which are completely transparent in the visible region.[56]

As mentioned in the introduction, one of the key features required for deployment of organic IR control elements will be their stability. While to our knowledge no in-depth study has yet probed this question, other liquid crystal based devices have solved these problems to a large extent: for example, large area LCD screens designed for outdoor use are in the marketplace. This gives some confidence that stability challenges to be faced in the emerging smart IR window industry could be similarly addressed. Another important factor for commercial acceptance of the windows is their eventual cost. Since the windows will offer a type of 'pay back' via the reduction of building energy use (and financial outlay for) electricity for lighting, heating, and cooling, this could offset a somewhat higher price.

We also envisage these responsive windows to be employed as a single glazing layer in a standard double or triple glazing window unit. This would allow retrofitting of existing windows while maintaining similar insulation and sound control qualities.

Currently, a number of industries are working for the development of both privacy and infrared reflection based smart windows for energy savings. BASF has developed a static IR reflecting coating which is transparent to visible light and reflecting near infrared light. SABIC Specialty Film and Sheet, FUJIFIM and 3M have also developed a film/sheet which can block infrared radiations. Merck chemical company has

 © 2017 The Authors. Published by WILEY-VCH Verlag GmbH & Co. KGaA, Weinheim

developed dye doped nematic LC based smart windows that can tune their absorption or transparency on application of an electric field. These examples demonstrate the intense commercial interest in these visibly transparent heat control systems.

5. Conclusions

Windows have a significant influence on energy consumption in the built environment and human health of the occupants of the buildings. Several methods, starting from blinds and shutters to advanced liquid crystals-based technologies have been developed to control indoor temperatures. Recent requirements for windows demand that they should simultaneously regulate indoor temperatures by controlling passage of excess of solar energy while also maintaining high transparency in the visible region to reduce dependence on artificial lighting. In this direction, a static infrared reflector which can completely block IR radiation from the sun without interfering with the visible transparency has been developed. Static systems are very useful in hot climates like Abu Dhabi, where continuous rejection of heat is required, but have limited usefulness in the moderate climate conditions. Manually controlled, electrically responsive and autonomous temperature responsive technologies which can change reflection properties depending on the external environmental conditions have also been developed. Various experimental and simulation studies have shown that significant amount of energy can be saved by responsive IR reflectors compared to the standard double glazing windows. Developing new technologies like bistable or energy generating windows could lead to significant improvement in energy savings and healthier, happier room occupants. It is also important to mention that most of the IR regulating based window technologies are still in the research and development phase and limited to the sample size. One of the biggest challenge while scaling-up is to achieve minimum haze in the visible region with good thermal and UV stability. The advantages of using smart windows is not only limited to the built environment: the interior temperature of automobiles could be better maintained by using such windows, leading to additional savings in fuel energy that is spent on running air-conditioners. Finally, such smart windows could also be very helpful in improving the growing conditions within greenhouses where control of temperature is seasonal and vital for the health of the plant.

Acknowledgements

This research forms part of the research program of the Dutch Polymer Institute (DPI), project 764 and PolyArch project. We would like to thank Alkistis Stergiani Karstiou for the artwork of Figure 2 and Jan Hensen and Roel Loonen of the Eindhoven University of Technology and Tillman Klein and Eric van Ham of the Eindhoven University of Technology for their fruitful discussions. The authors would also like to acknowledge the many discussions with and contributions of all our former and current colleagues and a special word of thanks to Prof. Dick Broer and Prof. Cees Bastiaansen.

Received: October 5, 2016
Revised: November 14, 2016
Published online: March 2, 2017

[1] A. M. Omer, *Renew. Sustain. Energy Rev.* **2008**, *12*, 2265.
[2] U.S. Department of Energy, http://Buildingsdatabook.Eren.Doe.Gov/. **2010**, 1, accessed: October, 2016.
[3] Y. Wang, E. L. Runnerstom, D. J. Milliron, *Annu. Rev. Chem. Biomol. Eng.* **2016**, *7*, 80615.
[4] "Calculated from the data given at the wedsite of National Research Energy Laboratory," can be found under http://www.nrel.gov, accessed: October, 2016.
[5] M. Isaac, D. P. van Vuuren, *Energy Policy* **2009**, *37*, 507.
[6] N. DeForest, A. Shehabi, J. O'Donnell, G. Garcia, J. Greenblatt, E. S. Lee, S. Selkowitz, D. J. Milliron, *Build. Environ.* **2015**, *89*, 107.
[7] E. S. Lee, D. L. DiBartolomeo, *Sol. Energy Mater. Sol. Cells* **2002**, *71*, 465.
[8] R. Baetens, B. P. Jelle, A. Gustavsen, *Sol. Energy Mater. Sol. Cells* **2010**, *94*, 87.
[9] J. Marchwinski, *Energy Procedia* **2014**, *57*, 1677.
[10] R. C. G. M. Loonen, M. Trčka, D. Cóstola, J. L. M. Hensen, *Renew. Sustain. Energy Rev.* **2013**, *25*, 483.
[11] S. B. Sadineni, S. Madala, R. F. Boehm, *Renew. Sustain. Energy Rev.* **2011**, *15*, 3617.
[12] J. Pockett, M. Belusko, in *48th AuSES Annu. Conf.*, **2010**, p. 1.
[13] M. Baneshi, S. Maruyama, H. Nakai, A. Komiya, *J. Quant. Spectrosc. Radiat. Transf.* **2009**, *110*, 192.
[14] F. Horowitz, M. B. Pereira, G. B. De Azambuja, **2011**, *50*, C250.
[15] A. Sharma, V. V. Tyagi, C. R. Chen, D. Buddhi, *Renew. Sustain. Energy Rev.* **2009**, *13*, 318.
[16] Y. Ma, J. Xu, B. ZHu, K. Wu, *J. Coatings Technol.* **2003**, *75*, 16.
[17] S. Kim, J. Cha, S. Kim, K. W. Park, D. R. Lee, J. H. Jo, *J. Therm. Anal. Calorim.* **2014**, *116*, 219.
[18] A. Fazel, A. Izadi, M. Azizi, *Sol. Energy* **2016**, *133*, 274.
[19] M. Maldovan, *Nature* **2013**, *503*, 209.
[20] G. Smith, A. Gentle, M. Arnold, M. Cortie, *Nanophotonics* **2016**, *5*, 55.
[21] B. Newill, M. Wagner, T. Pendell, B. Roushia, B. Holbrook, P. J. Weber, J. P. Moening, T. Hebrink, R. J. Strharsky, *Conf. Rec. IEEE Photovolt. Spec. Conf.* **2013**, 459.
[22] M. Gustafsonn, A. Karlsson, A. P. Pontes Rebelo, B. Widenberg, *IEEE Transactions on Antennas and Propegation* **2006**, *54*, 1897.
[23] G. I. Kiani, L. G. Olsson, A. Karlsson, K. P. Esselle, *IET Microwaves, Antennas Propag.* **2010**, *4*, 955.
[24] D. J. Broer, C. M. W. Bastiaansen, M. G. Debije, A. P. H. J. Schenning, *Angew. Chemie Int. Ed.* **2012**, *51*, 7102.
[25] D. Mulder, A. P. H. J. Schenning, C. Bastiaansen, *J. Mater. Chem. C* **2014**, *2*, 6695.
[26] V. Belyakov, V. Dmitrienko, V. Orlov, *Sov. Phys. Uspekhi* **1979**, *261*, 63.
[27] R. Dreher, G. Meier, *Phys. Rev. A* **1973**, *8*, 1616.
[28] C. A. Estrada-Gasca, G. Alvarez-Garcia, P. K. Nair, *J. Phys. D. Appl. Phys.* **1993**, *26*, 1304.
[29] C. M. Lampert, *Sol. Energy Mater.* **1981**, *6*, 1.
[30] C. G. Granqvist, *Sol. Energy Mater. Sol. Cells* **2007**, *91*, 1529.
[31] T. Tani, S. Hakuta, N. Kiyoto, M. Naya, *Opt. Express* **2014**, *22*, 9262.
[32] V. Shibaev, A. Bobrovsky, N. Boiko, *J. Photochem. Photobiol. A Chem.* **2003**, *155*, 3.
[33] T. Liu, B. Liu, J. Wang, L. Yang, X. Ma, H. Li, Y. Zhang, S. Yin, T. Sato, T. Sekino, Y. Wang, *Sci. Rep.* **2016**, *6*, 27373.
[34] A. M. Nilsson, A. Roos, *Thin Solid Films* **2009**, *517*, 3173.
[35] C. G. Granqvist, *Thin Solid Films* **2014**, *564*, 1.
[36] E. L. Runnerstrom, A. Llordés, S. D. Lounis, D. J. Milliron, A. Llordes, S. D. Lounis, D. J. Milliron, *Chem. Commun.* **2014**, *50*, 10555.
[37] G. Cai, J. Wang, P. S. Lee, *Acc. Chem. Res.* **2016**, *49*, 1469.
[38] S. K. Deb, *Proceedings of World Renewable Energy Congress VI* **2000**, 1.
[39] R.-T. Wen, C. G. Granqvist, G. A. Niklasson, *Nat. Mater.* **2015**, *14*, 996.

 © 2017 The Authors. Published by WILEY-VCH Verlag GmbH & Co. KGaA, Weinheim

[40] M. Kamalisarvestani, R. Saidur, S. Mekhilef, F. S. Javadi, *Renew. Sustain. Energy Rev.* **2013**, *26*, 353.
[41] S. Hoffmann, E. S. Lee, C. Clavero, *Sol. Energy Mater. Sol. Cells* **2014**, *123*, 65.
[42] M. J. Powell, R. Quesada-Cabrera, A. Taylor, D. Teixeira, I. Papakonstantinou, R. G. Palgrave, G. Sankar, I. P. Parkin, *Chem. Mater.* **2016**, *28*, 1369.
[43] V. Costanzo, G. Evola, L. Marletta, *Sol. Energy Mater. Sol. Cells* **2016**, *149*, 110.
[44] S. Schelm, G. B. Smith, P. D. Garrett, W. K. Fisher, *J. Appl. Phys.* **2005**, *97*, 12314.
[45] K. Yoshimura, C. Langhammer, B. Dam, *MRS Bull.* **2013**, *38*, 495.
[46] S. Eda, K. Moriyasu, M. Fujishima, S. Nomura, H. Tada, *RSC Adv.* **2013**, *3*, 10414.
[47] J. M. Schultz, K. I. Jensen, *Vacuum* **2008**, *82*, 723.
[48] S. Shian, D. R. Clarke, *Opt. Lett.* **2016**, *41*, 1289.
[49] M. A. Green, *J. Mater. Sci. Mater. Electron.* **2007**, *18*, 15.
[50] B. D. Hatton, I. Wheeldon, M. J. Hancock, M. Kolle, J. Aizenberg, D. E. Ingber, *Sol. Energy Mater. Sol. Cells* **2013**, *117*, 429.
[51] V. Jain, H. Yochum, H. Wang, R. Montazami, M. A. Vidales Hurtado, A. Mendoza-Galvan, H. W. Gibson, J. R. Heflin, *Macromol. Chem. Phys.* **2008**, *209*, 150.
[52] C. M. Lampert, *Sol. Energy Mater.* **1984**, *11*, 1.
[53] B. P. Jelle, G. Hagen, *J. Appl. Electrochem.* **1998**, *28*, 1061.
[54] W. Zhao, E. M. Carreira, *Chem. Eur. J.* **2006**, *12*, 7254.
[55] M. G. Debije, P. P. C. Verbunt, *Adv. Energy Mater.* **2012**, *2*, 12.
[56] Y. Zhao, G. a. Meek, B. G. Levine, R. R. Lunt, *Adv. Opt. Mater.* **2014**, *2*, 606.
[57] M. G. Debije, P. P. C. Verbunt, B. C. Rowan, B. S. Richards, T. L. Hoeks, *Appl. Opt.* **2008**, *47*, 6763.
[58] M. G. Debije, P. P. C. Verbunt, P. J. Nadkarni, S. Velate, K. Bhaumik, S. Nedumbamana, B. C. Rowan, B. S. Richards, T. L. Hoeks, *Appl. Opt.* **2011**, *50*, 163.
[59] M. Mitov, *Adv. Mater.* **2012**, *24*, 6260.
[60] H. Choi, J. Kim, S. Nishimura, T. Toyooka, F. Araoka, K. Ishikawa, J. W. Wu, H. Takezoe, *Adv. Mater.* **2010**, *22*, 2680.
[61] D. J. Broer, J. Lub, G. N. Mol, *Nature* **1995**, *378*, 467.
[62] H. Khandelwal, R. C. G. M. Loonen, J. L. M. Hensen, A. P. H. J. Schenning, M. G. Debije, *J. Mater. Chem. A* **2014**, *2*, 14622.
[63] L. Zhang, M. Wang, L. Wang, D. Yang, H. Yu, H. Yang, *Liq. Cryst.* **2016**, *43*, 750.
[64] X. Chen, L. Wang, Y. Chen, C. Li, G. Hou, X. Liu, X. Zhang, W. He, H. Yang, *Chem. Commun.* **2013**, *50*, 691.
[65] D. J. Broer, G. N. Mol, J. a. M. M. Van Haaren, J. Lub, *Adv. Mater.* **1999**, *11*, 573.
[66] Y. Gao, W. Yao, J. Sun, H. Zhang, Z. Wang, L. Wang, D. Yang, L. Zhang, H. Yang, *J. Mater. Chem. A* **2015**, *3*, 10738.
[67] D. Yokoyama, K. Nakayama, T. Otani, J. Kido, *Adv. Mater.* **2012**, *24*, 6368.
[68] M. De Vittorio, M. Lomascolo, A. Passaseo, R. Cingolani, A. Convertino, A. Valentini, *Superlattices Microstruct.* **1999**, *25*, 313.
[69] A. Convertino, A. Valentini, R. Cingolani, *Appl. Phys. Lett.* **1999**, *75*, 322.
[70] F. Bäbler, *IR Reflective Pigment Compositions* **2006**, US6989056B2.
[71] T. R. Sliwinski, R. A. Pipoly, R. P. Blonski, *Infrared Reflective Color Pigment* **2001**, US6174360B1.
[72] L. Xie, Y. Ying, T. Ying, *J. Agric. Food Chem.* **2007**, *55*, 4645.
[73] J. A. Bossard, L. Lin, D. H. Werner, *J. R. Soc. Interface* **2016**, *13*, 20150975.
[74] D. Coates, *Displays* **1993**, *14*, 94.
[75] D. Coates, *J. Mater. Chem.* **1995**, *5*, 2063.
[76] G. Manfre, *Mol. Cvsr. Liq. Cyst.* **2001**, *360*, 41.
[77] I. Dierking, *Polym. Chem.* **2010**, *1*, 1153.
[78] D. K. Yang, Q. Li, *Polymer Stabilized Cholesteric Liquid Crystal for Switchable Windows*, John Wiley & Sons, Inc., **2012**.
[79] Q. Li, *Liquid Crystals Beyond Displays: Chemistry, Physics, and Applications*, John Wiley & Sons, **2012**.
[80] P. S. Drzaic, *Liq. Cryst. Today* **1995**, *5*, 2.
[81] K.-H. Kim, H.-J. Jin, K.-H. Park, J.-H. Lee, J. C. Kim, T.-H. Yoon, *Opt. Express* **2010**, *18*, 16745.
[82] D. J. Gardiner, S. M. Morris, H. J. Coles, *Sol. Energy Mater. Sol. Cells* **2009**, *93*, 301.
[83] I.-H. Lee, Y.-C. Chao, C.-C. Hsu, L.-C. Chang, T.-L. Chiu, J.-Y. Lee, F.-J. Kao, C.-K. Lee, J.-H. Lee, *Proc. SPIE* **2010**, *7618*, 761816.
[84] C. J. Barile, D. J. Slotcavage, M. D. McGehee, *Chem. Mater.* **2016**, *28*, 1439.
[85] C. Y. Hsu, J. Zhang, T. Sato, S. Moriyama, M. Higuchi, *ACS Appl. Mater. Interfaces* **2015**, *7*, 18266.
[86] T. Abidin, Q. Zhang, K. L. Wang, D. J. Liaw, *Polym. (United Kingdom)* **2014**, *55*, 5293.
[87] T. Soganci, M. Ak, E. Giziroglu, H. C. Söyleyici, *RSC Adv.* **2016**, *6*, 1744.
[88] Y. Alesanco, A. Viñuales, J. Palenzuela, I. Odriozola, G. Cabañero, J. Rodriguez, R. Tena-Zaera, *ACS Appl. Mater. Interfaces* **2016**, *8*, 14795.
[89] R. Brooke, M. Fabretto, M. Krasowska, P. Talemi, S. Pering, P. J. Murphy, D. Evans, *J. Mater. Chem. C* **2016**, *4*, 1550.
[90] S. S. Choi, S. M. Morris, W. T. S. Huck, H. J. Coles, *Adv. Mater.* **2009**, *21*, 3915.
[91] L. V. Natarajan, J. M. Wofford, V. P. Tondiglia, R. L. Sutherland, H. Koerner, R. A. Vaia, T. J. Bunning, *J. Appl. Phys.* **2008**, *103*, 93107.
[92] C. Binet, M. Mitov, M. Mauzac, *J. Appl. Phys.* **2001**, *90*, 1730.
[93] H. Khandelwal, R. C. G. M. Loonen, J. L. M. Hensen, M. G. Debije, A. P. H. J. Schenning, *Sci. Rep.* **2015**, *5*, 11773.
[94] R. A. M. Hikmet, H. Kemperman, *Nature* **1998**, *392*, 476.
[95] P. Lemarchand, J. Doran, B. Norton, *Energy Procedia* **2014**, *57*, 1878.
[96] V. P. Tondiglia, L. V. Natarajan, C. A. Bailey, M. E. McConney, K. M. Lee, T. J. Bunning, R. Zola, H. Nemati, D.-K. Yang, T. J. White, *Opt. Mater. Express* **2014**, *4*, 1465.
[97] H. Khandelwal, M. Debije, T. White, A. P. H. J. Schenning, *J. Mater. Chem. A* **2016**, *4*, 6064.
[98] W. Hu, L. Zhang, H. Cao, L. Song, H. Zhao, Z. Yang, Z. Cheng, H. Yang, L. Guo, *Phys. Chem. Chem. Phys.* **2010**, *12*, 2632.
[99] W. Hu, H. Zhao, L. Song, Z. Yang, H. Cao, Z. Cheng, Q. Liu, H. Yang, *Adv. Mater.* **2010**, *22*, 468.
[100] H. Lu, J. Hu, Y. Chu, W. Xu, L. Qiu, X. Wang, G. Zhang, J. Hu, J. Yang, *J. Mater. Chem. C* **2015**, *3*, 5406.
[101] J. Xiang, Y. Li, Q. Li, D. A. Paterson, J. M. D. Storey, C. T. Imrie, O. D. Lavrentovich, *Adv. Mater.* **2015**, *27*, 3014.
[102] Y. Zhou, Y. Cai, X. Hu, Y. Long, *J. Mater. Chem. A* **2014**, *2*, 13550.
[103] Y.-S. Yang, Y. Zhou, F. B. Yin Chiang, Y. Long, *RSC Adv.* **2016**, *6*, 61449.
[104] D. Kim, E. Lee, H. S. Lee, J. Yoon, *Sci. Rep.* **2015**, *5*, 7646.
[105] K. A. R. Ismail, J. Henriquez, *Appl. Therm. Eng.* **2001**, *21*, 1909.
[106] S. Grynning, F. Goia, B. Time, *Energy Procedia* **2015**, *78*, 85.
[107] A. Seeboth, R. Ruhmann, O. Mühling, *Materials (Basel).* **2010**, *3*, 5143.
[108] R. Baetens, B. P. Jelle, A. Gustavsen, *Energy Build.* **2010**, *42*, 1361.
[109] G. Pan, H. Cao, R. Guo, W. Li, J. Guo, Z. Yang, W. Huang, W. He, X. Liang, D. Zhang, H. Yang, *Opt. Mater. (Amst).* **2009**, *31*, 1163.
[110] F. Guo, S. Chen, Z. Chen, H. Luo, Y. Gao, T. Przybilla, E. Spiecker, A. Osvet, K. Forberich, C. J. Brabec, *Adv. Opt. Mater.* **2015**, *3*, 1524.
[111] H. Kakiuchida, M. Tazawa, K. Yoshimura, A. Ogiwara, *Sol. Energy Mater. Sol. Cells* **2010**, *94*, 1747.
[112] H. Yang, K. Mishima, K. Matsuyama, K.-I. Hayashi, H. Kikuchi, T. Kajiyama, *Appl. Phys. Lett.* **2003**, *82*, 2407.
[113] W. Huang, X. Zhang, J. Guo, L. Zhang, Z. Bian, D. Zhao, W. He, H. Cao, H. Yang, *Liq. Cryst.* **2009**, *36*, 497.

 © 2017 The Authors. Published by WILEY-VCH Verlag GmbH & Co. KGaA, Weinheim

[114] X. Yuan, L. Zhang, H. Yang, *Liq. Cryst.* **2010**, *37*, 445.
[115] R. Guo, K. Li, H. Cao, X. Wu, G. Wang, Z. Cheng, F. Wang, H. Zhang, H. Yang, *Polymer* **2010**, *51*, 5990.
[116] T. J. White, S. A. Cazzell, A. S. Freer, D.-K. Yang, L. Sukhomlinova, L. Su, T. Kosa, B. Taheri, T. J. Bunning, *Adv. Mater.* **2011**, *23*, 1389.
[117] T. J. White, R. L. Bricker, L. V. Natarajan, N. V. Tabiryan, L. Green, Q. Li, T. J. Bunning, *Adv. Funct. Mater.* **2009**, *19*, 3484.
[118] M. Mathews, R. S. Zola, S. Hurley, D. K. Yang, T. J. White, T. J. Bunning, Q. Li, *J. Am. Chem. Soc.* **2010**, *132*, 18361.
[119] T. J. White, A. S. Freer, N. V. Tabiryan, T. J. Bunning, *J. Appl. Phys.* **2010**, *107*, 73110.
[120] H. Khandelwal, G. H. Timmermans, M. G. Debije, A. P. H. J. Schenning, *Chem. Commun.* **2016**, *52*, 10109.
[121] T.-H. Lin, W. C. Chen, Q. Li, *Anisotropic Nanomaterials- Preparation, Properties, and Applications (Chapter 9)*, Springer, **2015**.
[122] S.-Y. Lu, L.-C. Chien, *Opt. Lett.* **2010**, *35*, 562.
[123] C. W. Chen, C. C. Li, H. C. Jau, L. C. Yu, C. L. Hong, D. Y. Guo, C. T. Wang, T. H. Lin, *ACS Photonics* **2015**, *2*, 1524.
[124] S. T. Hur, B. R. Lee, M. J. Gim, K. W. Park, M. H. Song, S. W. Choi, *Adv. Mater.* **2013**, *25*, 3002.
[125] C.-T. Wang, T.-H. Lin, *Opt. Mater. Express* **2011**, *1*, 1457.
[126] H.-Y. Liu, C.-T. Wang, C.-Y. Hsu, T.-H. Lin, *Appl. Opt.* **2011**, *50*, 1606.
[127] T. H. Lin, Y. Li, C. T. Wang, H. C. Jau, C. W. Chen, C. C. Li, H. K. Bisoyi, T. J. Bunning, Q. Li, *Adv. Mater.* **2013**, *25*, 5050.
[128] H. K. Bisoyi, Q. Li, *Acc. Chem. Res.* **2014**, *47*, 3184.
[129] J. Ge, Y. Yin, *Angew. Chemie - Int. Ed.* **2011**, *50*, 1492.
[130] L. Nucara, F. Greco, V. Mattoli, *J. Mater. Chem. C* **2015**, *3*, 8449.
[131] D. M. Makow, *Appl. Opt.* **1980**, *19*, 1274.
[132] M. Mitov, N. Dessaud, *Nat. Mater.* **2006**, *5*, 361.
[133] M. Mitov, N. Dessaud, *Liq. Cryst.* **2007**, *34*, 183.
[134] J. Guo, F. Liu, F. Chen, J. Wei, H. Yang, *Liq. Cryst.* **2010**, *37*, 171.
[135] J. Guo, H. Yang, R. Li, N. Ji, X. Dong, H. Wu, J. Wei, *J. Phys. Chem. C* **2009**, *113*, 16538.
[136] M. E. McConney, V. P. Tondiglia, J. M. Hurtubise, T. J. White, T. J. Bunning, *Chem. Commun.* **2011**, *47*, 505.
[137] E. Beltran-Gracia, O. L. Parri, *J. Mater. Chem. C* **2015**, *3*, 11335.
[138] H. P. C. van Kuringen, D. J. Mulder, E. Beltran, D. J. Broer, A. P. H. J. Schenning, *Polym. Chem.* **2016**, *7*, 4712.
[139] E. M. Korenic, S. D. Jacobs, S. M. Fare, L. Li, *Mol. Cryst. Liq. Cryst. Sci. Technol. Sect. A. Mol. Cryst. Liq. Cryst.* **1998**, *317*, 197.
[140] J. E. Stumpel, D. J. Broer, A. P. H. J. Schenning, *Chem. Commun.* **2014**, *50*, 15839.
[141] D.-K. Yang, X.-Y. Huang, Y.-M. Zhu, *Annu. Rev. Mater. Sci.* **1997**, *27*, 117.
[142] H. H. Liang, C. C. Wu, P. H. Wang, J. Y. Lee, *Opt. Mater. (Amst).* **2011**, *33*, 1195.
[143] K. M. Lee, V. P. Tondiglia, T. J. White, *MRS Commun.* **2015**, *5*, 223.
[144] R. Bao, C.-M. Liu, D.-K. Yang, *Appl. Phys. Express* **2009**, *2*, 112401.
[145] J. Guo, H. Xing, O. Jin, Y. Shi, J. Wei, *Mol. Cryst. Liq. Cryst.* **2013**, *582*, 21.
[146] M. G. Debije, *Adv. Funct. Mater.* **2010**, *20*, 1498.
[147] C. van Oosten, M. Zitto, *SID Int. Symp. Dig. Tech. Pap.* **2016**, *47*, 376.
[148] H.-K. Kwon, K.-T. Lee, K. Hur, S. H. Moon, M. M. Quasim, T. D. Wilkinson, J.-Y. Han, H. Ko, I.-K. Han, B. Park, B. K. Min, B.-K. Ju, S. M. Morris, R. H. Friend, D.-H. Ko, *Adv. Energy Mater.* **2015**, *5*, 1401347.
[149] F. Malara, A. Cannavale, S. Carallo, G. Gigli, *ACS Appl. Mater. Interfaces* **2014**, *6*, 9290.
[150] C. C. Wu, J. C. Liou, C. C. Diao, *Chem. Commun.* **2015**, *51*, 2.
[151] M.-H. Yeh, L. Lin, P.-K. Yang, Z. L. Wang, *ACS Nano* **2015**, *9*, 4757.
[152] A. M. Kendhale, A. P. H. J. Schenning, M. G. Debije, *J. Mater. Chem. A* **2013**, *1*, 229.
[153] J. Ter Schiphorst, A. M. Kendhale, M. G. Debije, C. Menelaou, L. M. Herz, A. P. H. J. Schenning, *Chem. Mater.* **2014**, *26*, 3876.

 © 2017 The Authors. Published by WILEY-VCH Verlag GmbH & Co. KGaA, Weinheim

ELSEVIER

Renewable and Sustainable Energy Reviews

Volume 89, June 2018, Pages 51-60

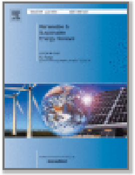

The National Solar Radiation Data Base (NSRDB)

Manajit Sengupta [a], Yu Xie [a], Anthony Lopez [b], Aron Habte [a], Galen Maclaurin [b], James Shelby [c]

Show more

Outline | + Add to Mendeley Share Cite

https://doi.org/10.1016/j.rser.2018.03.003 Get rights and content

Under a Creative Commons license

Open access

Renewable and Sustainable Energy Reviews 89 (2018) 51–60

Contents lists available at ScienceDirect

Renewable and Sustainable Energy Reviews

journal homepage: www.elsevier.com/locate/rser

The National Solar Radiation Data Base (NSRDB)

Manajit Sengupta[a], Yu Xie[a,*], Anthony Lopez[b], Aron Habte[a], Galen Maclaurin[b], James Shelby[c]

[a] Power Systems Engineering Center, National Renewable Energy Laboratory, Golden, CO 80401, United States
[b] Strategic Energy Analysis Center, National Renewable Energy Laboratory, Golden, CO 80401, United States
[c] Computational Science Center, National Renewable Energy Laboratory, Golden, CO 80401, United States

ARTICLE INFO

Keywords:
Solar Radiation
Satellite

ABSTRACT

The National Solar Radiation Data Base (NSRDB), consisting of solar radiation and meteorological data over the United States and regions of the surrounding countries, is a publicly open dataset that has been created and disseminated during the last 23 years. This paper briefly reviews the complete package of surface observations, models, and satellite data used for the latest version of the NSRDB as well as improvements in the measurement and modeling technologies deployed in the NSRDB over the years. The current NSRDB provides solar irradiance at a 4-km horizontal resolution for each 30-min interval from 1998 to 2016 computed by the National Renewable Energy Laboratory's (NREL's) Physical Solar Model (PSM) and products from the National Oceanic and Atmospheric Administration's (NOAA's) Geostationary Operational Environmental Satellite (GOES), the National Ice Center's (NIC's) Interactive Multisensor Snow and Ice Mapping System (IMS), and the National Aeronautics and Space Administration's (NASA's) Moderate Resolution Imaging Spectroradiometer (MODIS) and Modern Era Retrospective analysis for Research and Applications, version 2 (MERRA-2). The NSRDB irradiance data have been validated and shown to agree with surface observations with mean percentage biases within 5% and 10% for global horizontal irradiance (GHI) and direct normal irradiance (DNI), respectively. The data can be freely accessed via https://nsrdb.nrel.gov or through an application programming interface (API). During the last 23 years, the NSRDB has been widely used by an ever-growing group of researchers and industry both directly and through tools such as NREL's System Advisor Model.

1. Introduction

Understanding long-term spatial and temporal variability of the solar resource is fundamental for energy policy decisions, the optimal design of solar energy conversion systems, transmission interconnection planning, power systems integration, market operations, and reducing uncertainty in investments [1–4]. Historical solar resource data for those purposes can be provided by ground-based in situ measurements or satellite remote sensing [3].

Pyranometers and pyrheliometers, which use either thermoelectric or photoelectric detectors, are the most common ground-based radiometers to measure global horizontal irradiance (GHI) and direct normal

Abbreviations: %RMSE, Percentage RMSE; ACS, American Cancer Society; AERONET, Aerosol Robotic Network; AOD, Aerosol Optical Depth; API, Application Programming Interface; ARM, Atmospheric Radiation Measurement; ASHRAE, American Society of Heating Refrigerating and Air-Conditioning Engineers; AVHRR, Advanced Very High Resolution Radiometer; BND, Bondville, Illinois; CPR, Clean Power Research; DHI, Diffuse Horizontal Irradiance; DISC, Direct Insolation Simulation Code; DISORT, Discrete Ordinates Radiative Transfer; DNI, Direct Normal Irradiance; DOE, Department of Energy; DRA, Desert Rock, Nevada; FARMS, Fast All-sky Radiation Model for Satellite applications; FPK, Fort Peck, Montana; GCM, Goodwin Creek, Mississippi; GEOS-4, Goddard Earth Observing System Version 4; GHI, Global Horizontal Irradiance; GIS, Geographic Information System; GOES, Geostationary Operational Environmental Satellite; GUM, Guide to the Expression of Uncertainty in Measurement; HDF5, Hierarchical Data Format; HPC, High-Performance Computing; IMS, Interactive Multisensor Snow and Ice Mapping System; ISCCP, International Satellite Cloud Climatology Project; ISD, Integrated Surface Database; MAE, Mean Absolute Error; MBE, Mean Bias Error; MERRA-2, Modern Era Retrospective analysis for Research and Applications, version 2; METSTAT, Meteorological Statistical; MODIS, Moderate Resolution Imaging Spectroradiometer; MPE, Mean Percentage Error; NASA, National Aeronautics and Space Administration; NCEI, National Center for Environmental Information; NIC, National Ice Center; NOAA, National Oceanic and Atmospheric Administration; NREL, National Renewable Energy Laboratory; NSRDB, National Solar Radiation Data Base; NWS, National Weather Service; PATMOS-x, Pathfinder Atmospheres-Extended; PSM, Physical Solar Model; PSU, Pennsylvania State University, Pennsylvania; PV, Photovoltaic; RMSE, Root Mean Square Error; RRTM, Rapid Radiative Transfer Model; SAM, System Advisor Model; SGP, Southern Great Plains; SOLRAD, Solar Radiation Network; SRB, Surface Radiation Budget; SUNY, State University of New York at Albany; SURFRAD, Surface Radiation Budget Network; SXF, Sioux Falls, South Dakota; TBL, Boulder, Colorado; TMY, Typical Meteorological Year; TOA, Top of the Atmosphere

* Corresponding author.
E-mail address: Yu.Xie@nrel.gov (Y. Xie).

https://doi.org/10.1016/j.rser.2018.03.003
Received 21 September 2017; Received in revised form 9 January 2018; Accepted 8 March 2018
Available online 19 March 2018
1364-0321/ © 2019 The Authors. Published by Elsevier Ltd. This is an open access article under the CC BY license (http://creativecommons.org/licenses/BY/4.0/).

irradiance (DNI), respectively [5,6]. The accuracy of measurements by these instruments is highly dependent on instrument design, hardware installation schemes, data acquisition methods, and calibration method and frequency [7–9]. Measurements by accurately calibrated and well-maintained pyrheliometers and pyranometers can provide reliable long-term solar radiation data at specific locations [10–13] that are frequently used for cloud and radiation studies and validation of satellite-derived solar radiation [14–17].

The high cost of operating quality ground stations has resulted in existing surface radiation networks being sparsely distributed and insufficient to meet the needs of the rapidly growing solar energy industry. The other reliable and practical option is to use information from geostationary weather satellites that provides continuous solar radiation estimates covering a wide spectrum of temporal and spatial scales. To retrieve solar radiation from satellite data, solar irradiance models are essential to compute surface GHI and DNI from observations of radiances at the top of the atmosphere (TOA). During the last few decades, numerous solar irradiance models have been developed using empirical, semi-empirical or physical models [18–21]. Empirical models develop regression functions relating long-term GHI measurements at selected local stations to the simultaneous data recorded by satellites' visible channels [22–25] which are then used to simulate GHI from global satellite observations. The GHI is combined with empirical relationships developed using modeled or observed solar radiation to retrieve DNI [26,27]. A well-known solar radiation dataset developed by an empirical model is HelioClim based on the observations of Meteosat geostationary satellites covering Europe, Africa, the Mediterranean Basin, the Atlantic Ocean, and part of the Indian Ocean [22,28,29]. Compared to empirical models, semi-empirical models use a hybrid approach to derive solar radiation in which clear-sky background irradiance is solved from simple radiative transfer schemes [25,30,31]. A cloud index representing the proportion of radiation reflecting back to the satellite is converted to a clearness index that represents the proportion of incident radiation reaching the surface. The clearness index scales the clear sky radiation to estimate the GHI and then partitioned to estimate the DNI, which is similar to the empirical models. This semi-empirical approach has been widely implemented in global solar radiation datasets, including SolarGIS [30,31] and SolarAnyWhere [25].

Physical models are conventionally categorized by single-step and two-step models according to the procedures to determine solar radiation [3]. Single-step models directly solve for GHI using satellite observations and radiative transfer theory [32–34]. Two-step models intend to understand the complete physics affecting the transmission of solar radiation from the TOA to land surface. They retrieve aerosol, cloud and other atmospheric properties from various satellite channels or modeling efforts and use the information to precisely simulate GHI by solving the radiative transfer equation [18–21]. A typical product of a two-step model is the National Aeronautics and Space Administration's (NASA's) global Surface Radiation Budget (SRB) in which International Satellite Cloud Climatology Project (ISCCP) pixel-level data and Goddard Earth Observing System Version 4 (GEOS-4) reanalysis products are used to infer atmospheric properties at a 250-km resolution every 3 h. The solar radiation is then derived using the atmospheric properties and a model developed by Pinker and Laszlo [35].

Compared to empirical, semi-empirical and single-step physical models, most two-step physical models require significant computational capability because extensive information from satellite observations and other ancillary inputs need to be processed to estimate solar radiation. The multiple processes in the production chain require sufficient quality inputs to make full use of the advanced models to reduce uncertainties of GHI and DNI. During the years, the rapid development of satellite technologies and modeling capabilities (e.g. the availability of Moderate Resolution Imaging Spectroradiometer (MODIS) spectral channels and multi-channel geostationary satellites) have effectively increased the reliability and accuracy of the two-step physical models [3,36–39]. More recently the expansion of spectral channels with better temporal and spatial resolutions on the third-generation Geostationary Operational Environmental Satellite-16 (GOES-16; previously GOES-R) is expected to lead to remarkable improvements in aerosol and cloud products [40] which the two-step physical models are capable of exploiting. The improvements in reanalysis data, such as NASA's Modern Era Retrospective analysis for Research and Applications, version 2 (MERRA-2), bring observations and numerical models together in a unified standardized framework, resulting in high-quality ancillary information that significantly enhances the quality of the two-step physical models [41]. In contrast, empirical, semi-empirical and single-step physical models are not expected to reap equivalent benefits from advances in satellite technology and reanalysis datasets because of inherent limitations in the underlying methods.

The National Renewable Energy Laboratory (NREL) has an extensive history of developing solar resource data over the United States using various sources of observations and modeling tools. This paper reviews the evolution of NREL's National Solar Radiation Data Base (NSRDB) and the recent efforts on developing the Physical Solar Model (PSM) and satellite-based solar radiation to enhance the resolution and accuracy of the NSRDB. The remainder of this paper is structured as follows. Section 2 provides a historical review of the NREL's NSRDB. Section 3 introduces the technical details of the PSM and data validation using surface-based solar radiation measurements. Section 4 describes the users and applications of the NSRDB, and the last section concludes and explores future work to further improve the NSRDB.

2. A historical review of the NSRDB

The NSRDB is one of the most accessed public datasets providing a serially complete collection of solar energy and meteorological data, including the three most common measurements of solar radiation: GHI, DNI, and diffuse horizontal irradiance (DHI), which have been collected over the United States and a growing list of international locations with high temporal (30 min) and spatial (4 km) resolutions to accurately represent the global and regional solar radiation climates. It supports the U.S. Department of Energy's (DOE's) SunShot goals of reducing barriers to high-penetration levels of solar energy technologies by providing easy access to high-quality, foundational data that are essential for innovative product development and downstream modeling. There have been substantial improvements in data collection and modeling technologies throughout the NSRDB's more than 20 years of existence. Therefore, NREL implemented major updates to the original database three times in 2007, 2012, and 2017. The NSRDB versions are briefly reviewed below.

The first version of the NSRDB, covering 1961–1991, originated in 1994 to replace the SOLMET/ERSATZ dataset developed by the National Oceanic and Atmospheric Administration (NOAA) and DOE [47]. This version contains hourly solar irradiance data for locations over 239 ground stations across the United States with a combination of measurements (approximately 7% of the total data) and simulations using NREL's Meteorological-Statistical (METSTAT) model [42]. Cloud observations from NOAA's National Center for Environmental Information (NCEI) Integrated Surface Database (ISD) were used as inputs to the METSTAT, and measured solar irradiances were directly obtained from the National Weather Service (NWS) solar radiation network (SOLRAD).

The first version of the NSRDB was updated in 2007 to cover 1991–2005. The major updates include 10×10 km solar irradiances from hourly GOES data and the use of an empirical model developed by the State University of New York at Albany (SUNY) [43]. The satellite-based products covered the contiguous 48 states of the United States from 1998 to 2005 while the solar irradiance data in Alaska were computed by the METSTAT model. This version of the NSRDB also provides measured (less than 1%) and modeled solar irradiances as well as other meteorological data from 1454 ground stations during 1991–2005 [43].

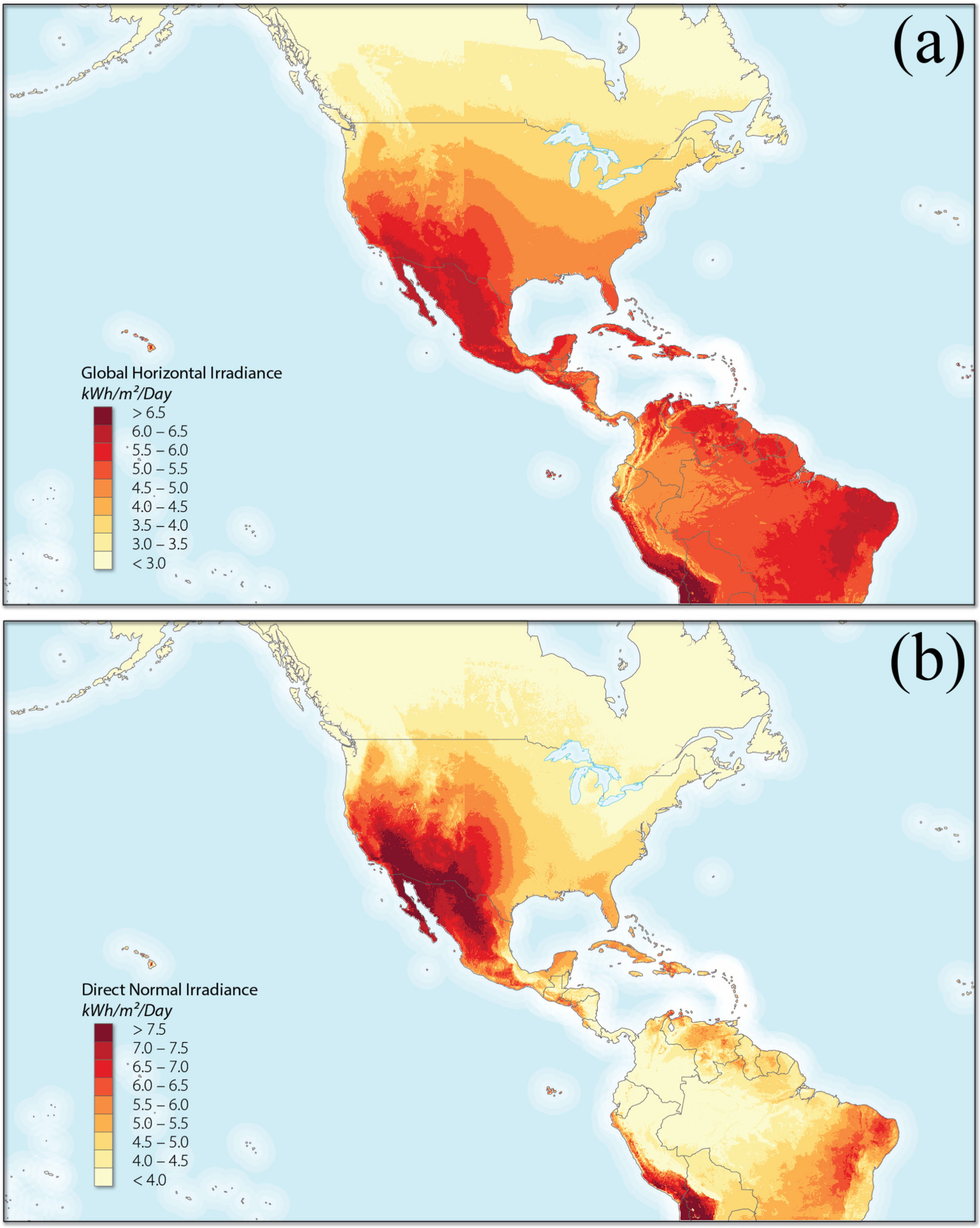

Fig. 1. Daily average of (a) GHI and (b) DNI during 1998–2016.

In 2012, NREL, in collaboration with Clean Power Research (CPR), updated the NSRDB to cover the years from 1991 to 2010. This version of the NSRDB was developed using an improved SUNY model in an hourly interval with a spatial resolution of 10 × 10 km. The data package also includes measurements from 1454 ground stations (1991–1997), including meteorological data from the NCEI ISD stations. The gridded NSRDB (1998–2010) was released through NREL's Solar Prospector web portal which was decommissioned at the end of September 2016.

The latest version of the NSRDB was released in 2017 containing

gridded data from 1998 to 2016 in half-hourly temporal and 4 × 4 km spatial resolutions. This dataset was developed using the GOES data that cover the entire Western Hemisphere from 60° North to 20° South latitude including the contiguous United States and Central America. The average values of the daily GHIs and DNIs from 1998 to 2016 are illustrated in Fig. 1. This version used the two-step physical model, PSM, which opened the door to the use of next-generation satellite datasets for solar resource assessment and forecasting. Details about the development of this latest NSRDB are introduced below.

3. Development, validation, and delivery of the latest NSRDB (1998–2016)

With the advancement of satellite technology, information available from accurately retrieved atmospheric properties is continuously growing. This coupled with the fast advancement in computing technology has resulted in significant improvements in solar radiation simulations. One such approach is the use of two-step physical models where cloud and aerosol properties derived in the first step are fed into a radiative transfer model in the subsequent step. This approach provides the opportunity to directly calculate DNI with better accuracy from improved retrievals of water vapor, aerosol, and cloud properties [33,44,45]. NREL employed this technology to produce the latest NSRDB, which contains long-term high-resolution solar radiation from geostationary satellites.

3.1. The PSM for developing the latest NSRDB

Fig. 2 displays a flowchart of the PSM, a two-step physical model to compute solar radiation from satellite data, which was developed through collaboration among NREL, the University of Wisconsin, and NOAA. As shown in the figure, aerosol, water vapor and other meteorological properties are combined with satellite-derived cloud properties and used in the Fast All-sky Radiation Model for Solar applications (FARMS) [17] to compute GHI. For clear scenes, FARMS is also used to compute DNI, whereas the Direct Insolation Simulation Code (DISC) [27] decomposition model is used for cloudy scenes.

3.1.1. Cloud properties

NOAA developed an Advanced Very High Resolution Radiometer (AVHRR) Pathfinder Atmospheres-Extended (PATMOS-x) system to efficiently retrieve cloud physical and optical properties from the synergetic use of satellite measurements in visible, near-infrared, and infrared channels [46]. The system has been implemented with data from GOES for continuous weather monitoring and forecasting. Cloud products—including cloud height, thermodynamic phase, optical thickness and effective particle size—are retrieved from PATMOS-x and GOES-West and GOES-East satellites at 4 × 4 km over the continental United States for each 30 min during day time. These products are employed by the PSM to produce cloudy-sky solar radiation from 1998 to 2016.

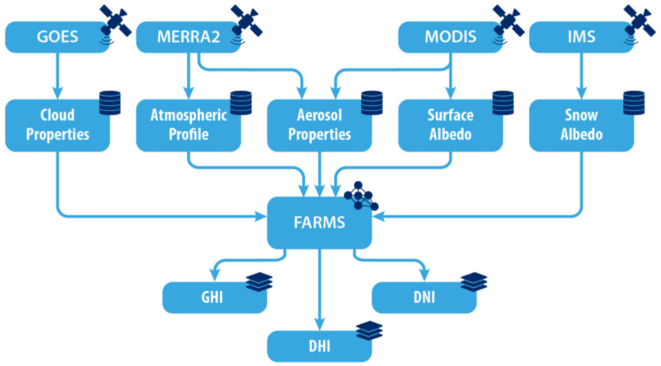

Fig. 2. A flowchart of the PSM.

3.1.2. Aerosol properties

The aerosol optical depth (AOD) used by the PSM is based on monthly MODIS data in combination with the MERRA-2 aerosol dataset. The monthly data are first scaled to a 0.5 × 0.5° resolution using an elevation weighting scheme and evaluated by surface-based Aerosol Robotic Network (AERONET) data. According to the evaluation, North America is divided into two regions. Over the southern and western United States, and northern Mexico, dominated by arid areas, the AOD is given by an optimal linear combination of MERRA-2 and MODIS data when the latter are available. Only MERRA-2 data are employed when MODIS observations are missing due to high surface albedo, cloudiness, large solar zenith angles, etc. Over the eastern and northern United States, Canada, southern Mexico and central America that are dominated by vegetation or urban areas, only MERRA-2 data are used because they are found to have similar accuracy as MODIS which suffers from a large fraction of missing data, especially in the high-latitude areas during winter. The monthly AOD data are then interpolated at 4 × 4 km on the basis of a daily average to match the NSRDB grids and improve the accuracy of surface solar radiation [47].

3.1.3. Other atmospheric and land surface data

The MODIS instruments onboard the Terra and Aqua satellites provide high-quality measurements of surface albedo at 30 arc-seconds for each 8-day interval [48]. Maclaurin et al. [49] matched the white-sky albedos from the MODIS MCD43GF product to the NSRDB grids. A point-in-polygon approach was employed to assemble MODIS pixels and compute the effective values of surface albedo within the NSRDB grids. This new product was integrated with National Ice Center's (NIC's) Interactive Multisensor Snow and Ice Mapping System (IMS) to coordinate the influence of snow and ice on surface albedo.

The other atmospheric and land properties used or provided by the NSRDB—e.g. the atmospheric profile, wind direction and speed, snow depth, surface temperature and pressure, etc. —are based on data from NASA's MERRA-2.

3.1.4. The all-sky radiative transfer model

The PSM applies satellite-derived atmospheric and land surface properties to radiative transfer models to numerically solve for solar radiation through the Earth's atmosphere. When clouds are absent, the extinction of solar radiation is associated with the light scattering by aerosols and air molecules and absorption by the trace gases in the atmosphere such as water vapor, carbon dioxide, ozone, oxygen, and methane. Although high-spectral-resolution radiative transfer models—e.g. the Line-By-Line radiative transfer model [50]—provide rigorous expression of radiation in narrow bands of wavelength, they are less efficient when solving for broadband solar radiation. Thus, many clear-sky radiative transfer models parameterize the extinction of broadband solar radiation from surface-based meteorological data or simulations in numerous spectral bands [51–57].

Badescu et al. [54] evaluated 54 clear-sky radiative transfer models using surface observations of GHI and DHI from Kipp and Zonen radiometers in Cluj-Napoca and Bucharest-Afumati. Although the best model for all scenarios was not found, REST2 [51] was ranked among the first-tier, along with three other models, in terms of the accuracy for both GHI and DHI. Because of the concise equations and parameters and the consequent efficiency in computing, REST2 is used in the development of the NSRDB [51].

The radiative transfer problem under a cloudy sky is much more complicated because of the combination of absorption and multiple scattering within the cloud. Thus, solving the radiative transfer equation for clouds is the only rigorous approach to compute cloudy-sky radiation [58,59]. Despite numerous approximations—e.g. the two-stream approach and delta-M truncation scheme [60]—conventional radiative transfer models are still time consuming in numerically solving the radiative transfer equation.

To meet the needs of developing the NSRDB and other solar energy

applications, Xie et al. [17] proposed FARMS to efficiently simulate all-sky solar radiation at land surfaces. In contrast to solving the radiative transfer equation, FARMS uses pre-computed cloud transmittances and reflectances of irradiances by the Rapid Radiative Transfer Model (RRTM) [61,62] with a 16-stream Discrete Ordinates Radiative Transfer (DISORT) model [63]. To further reduce the computing burden, the cloud transmittances and reflectances were parameterized as functions of solar zenith angle, as well as cloud thermodynamic phase, optical thickness, and particle size. The parameterization is coupled with surface albedo and REST2 accounting for clear-sky transmittances and reflectances to compute all-sky downwelling solar irradiances. The evaluation using 16-stream RRTM and DOE's Atmospheric Radiation Measurement (ARM) Southern Great Plains (SGP) site indicates that FARMS is as accurate as the two-stream approach; however, FARMS is approximately 1000 times faster than the two-stream approach, which has substantially accelerated the computation of the NSRDB. More detailed algorithm and performance evaluation can be found in [17]. It is also worth noting that the algorithm to compute clear-sky radiation is consistent in both clear-sky and cloudy-sky conditions because FARMS is coupled with REST2.

3.2. Data processing

Despite the progressive efficiency provided by the FARMS, developing the NSRDB is still a computationally intense process because of its advanced temporal and spatial resolutions and a large volume of input data including approximately 50 terabytes of GOES and 1.5 terabytes of the other data. Therefore, the NSRDB data are produced by the NREL's flagship high-performance computing (HPC) system that is capable of 2.26 PetaFLOPS with a total of 58,752 Intel Xeon processor cores, including 6912 E5–2670 SandyBridge, 24192 E5–2695v2 IvyBridge, and 27648 E5–2670v3 Haswell processor cores. To ensure timely production, allowing for rapid computation and quality check, we use Hierarchical Data Format (HDF5) for storage and a highly parallel and vectorized framework based on Python with the fundamental packages of NumPy and MPI4Py.

In the PSM shown in Fig. 2, the development of a serially complete NSRDB with consistent spatial and temporal mapping requires four additional steps prior to the computation by FARMS: regridding, temporal interpolation, time shifting, and gap filling.

The regridding step organizes all input data and reprocesses them into the NSRDB grids with a resolution of 4 × 4 km. The cloud properties are regridded using a nearest-neighbor approach because the GOES data have grids that are very similar to the NSRDB. Different regridding procedures based on physics laws are employed in the MERRA-2 and AOD data when their spatial resolutions are significantly lower than the NSRDB. For example, data with land-surface pressure and temperature with a 0.5° spatial resolution are regridded using an elevation scaling by considering the hydrostatic equation and a temperature lapse rate of 6 °C/km, respectively. The 0.5° resolution AOD data are first reduced to sea-level using an exponential scale height of 2950 m and then regridded to the NSRDB resolution by applying the same scale height to the pixel elevation. However, the specific humidity, wind speed, and direction are converted to the NSRDB resolution using a nearest-neighbor approach.

The temporal-interpolation step assigns the regridded data to the NSRDB intervals every 30 min. Specifically, the wind directions are determined from the nearest values of the hourly MERRA-2 data, and other properties are interpolated to the NSRDB resolution using a simple linear relationship. With a mean-conserving algorithm, the monthly-mean AOD data are interpolated to daily intervals.

Data from the GOES-West are given at each integral hour and 30 min past, whereas those for the GOES-East are available at 15 and 45 min past the integral hours. To develop the NSRDB with a consistent time stamp matching the GOES-West, the time-shifting step projects the 15-min delayed cloud properties from the GOES-East data to the NSRDB time stamps.

The gap-filling step supplements the NSRDB because data gaps in cloud properties routinely exist in long-term satellite-based observations. Times with missing cloud properties, caused by various reasons, are represented by the clear-sky GHI and the ratio of GHI to clear sky GHI available in the nearest previous time point.

3.3. Validation

A comprehensive evaluation of the NSRDB is essential when discussing “bankable data” for all phases of solar energy conversion projects, from the conceptual phase to routine solar power plant operation. Solar radiation data with known uncertainties help reduce the expense associated with mitigating performance and financing risk for solar energy conversion systems.

The performance of the latest NSRDB was recently investigated by Habte et al. [64] using surface observations from NREL's SRRL; ARM SGP; and Surface Radiation Budget Network (SURFRAD) sites at Bondville, Illinois (BND); Desert Rock, Nevada (DRA); Fort Peck, Montana (FPK); Goodwin Creek, Mississippi (GCM); Pennsylvania State University, Pennsylvania (PSU); Sioux Falls, South Dakota (SXF); and Boulder, Colorado (TBL). Various statistics, such as mean bias error (MBE), mean absolute error (MAE), mean percentage error (MPE), root mean square error (RMSE), and percentage RMSE (%RMSE) were calculated for various locations and time scales. Fig. 3 demonstrates the spatial distribution of the surface sites that represent diverse geographical and climatic features at locations throughout the continental United States.

Fig. 4 shows the MPEs of the GHI and DNI that are defined as:

$$MPE = \frac{\sum_{i=1}^{n} (x_N - x_S)}{\sum_{i=1}^{n} x_S} \times 100\% \tag{1}$$

where x_N denotes hourly-, daily-, monthly- or annually-averaged NSRDB data during 1998–2016; x_S represents the corresponding surface observations in the same timescales; and n is the number of data points for each averaging timescale. The biases of the NSRDB do not significantly vary with the averaging timescales, which indicates that its performance is consistent when used for different phases of solar energy deployment projects. The GHIs from the NSRDB are generally over- and underestimated, respectively, in the eastern and western United States; whereas the overall percentage biases are within 5%. The MPEs of the DNIs are within 10% on average, whereas those over the eastern United States are significantly higher than the western area, because of the presence of more clouds. However, challenges exist in accurately quantifying irradiances in the western areas that are susceptible to high occurrences of clouds, snow, and bright surfaces, such as FPK and TBL. The uncertainty and bias of DNI is generally greater than GHI because DNI is directly related to extinction in the atmosphere including clouds and aerosols whereas GHI includes radiation that is scattered to the surface [57]. Thus, advanced technology in satellite remote sensing of clouds and improvements in radiative transfer models are needed to improve the accuracy in the DNI simulation. Sub-pixel variability and the parallax effect in clouds contribute to increased uncertainty and biases for both GHI and DNI, which will be investigated in future updates of the NSRDB.

It was also reported by Habte et al. [64] that the %RMSEs of the hourly-averaged GHI and DNI can reach up to 20% and 40%, respectively, when compared to the surface-based measurements. An interannual variability of the GHI and DNI was found to be less than 5% for both the NSRDB and surface observations.

It is known that uncertainties in ground-based measurements might affect the evaluation of biases from satellite data. For a more comprehensive analysis, we evaluated the NSRDB using a quantification method based on the Guide to the Expression of Uncertainty in Measurement (GUM) [65], which implements the effect of statistical

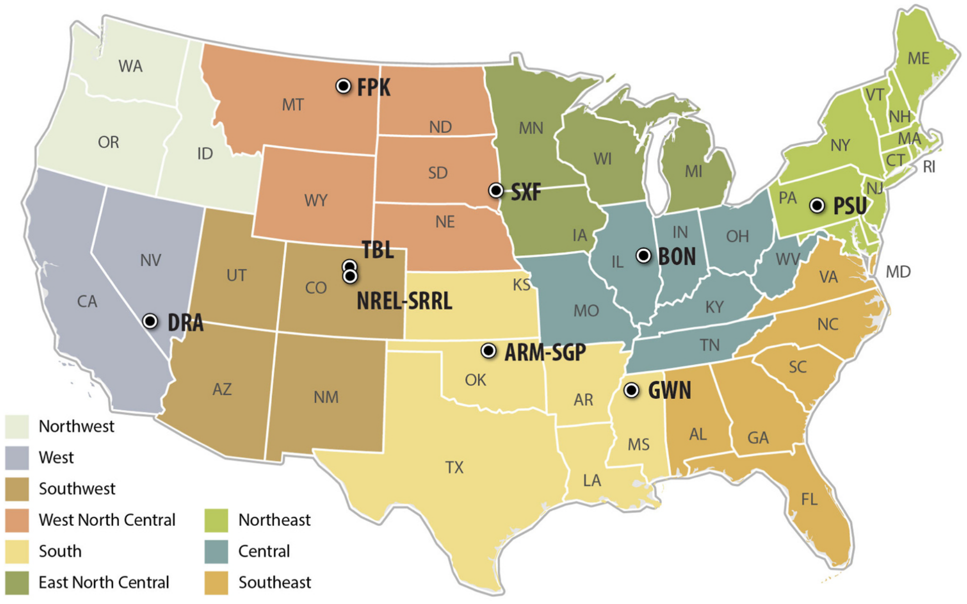

Fig. 3. The spatial distribution of NREL SRRL, ARM SGP, and SURFRAD sites.

distributions when comparing the model simulations and surface observations, accounts for the uncertainties in surface observations, and represents the model biases with a confidence interval. Eq. (2) denotes the percentage bias for a 95% confidence interval:

$$U_{95} = \pm\sqrt{U_S^2 + MPE^2 + \%RMSE^2} \quad (2)$$

where U_S is the percentage bias of the surface measurements and % RMSE is defined by

$$\%RMSE = \sqrt{\frac{\sum_{i=1}^{n}(x_N - x_S)^2}{\sum_{i=1}^{n} x_S^2}} \times 100\% \quad (3)$$

The magnitudes of U_{95} for the GHI at the surface sites are illustrated in Fig. 5. A U_S of 5% is assumed for all the sites because most well-maintained surface-based pyranometers report uncertainties ranging from 3% to 5% [8,66]. The stability of U_S and its impact on the U_{95} might require further studies, as discussed by [64]. Fig. 5 shows that the

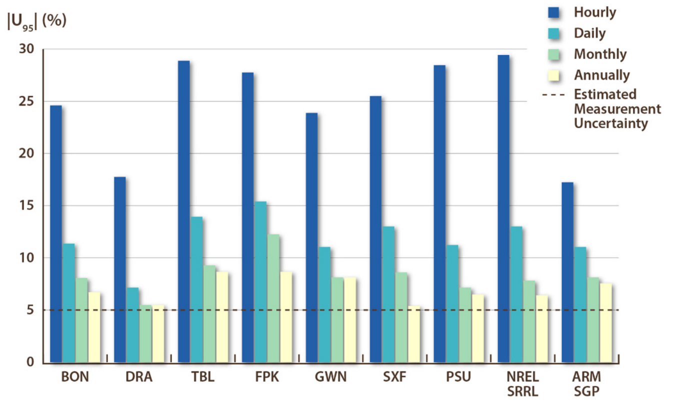

Fig. 5. The magnitudes of U_{95} for the hourly-, daily-, monthly-, and annually-averaged GHIs. The percentage bias of the surface measurements is assumed to be 5% for all the sites.

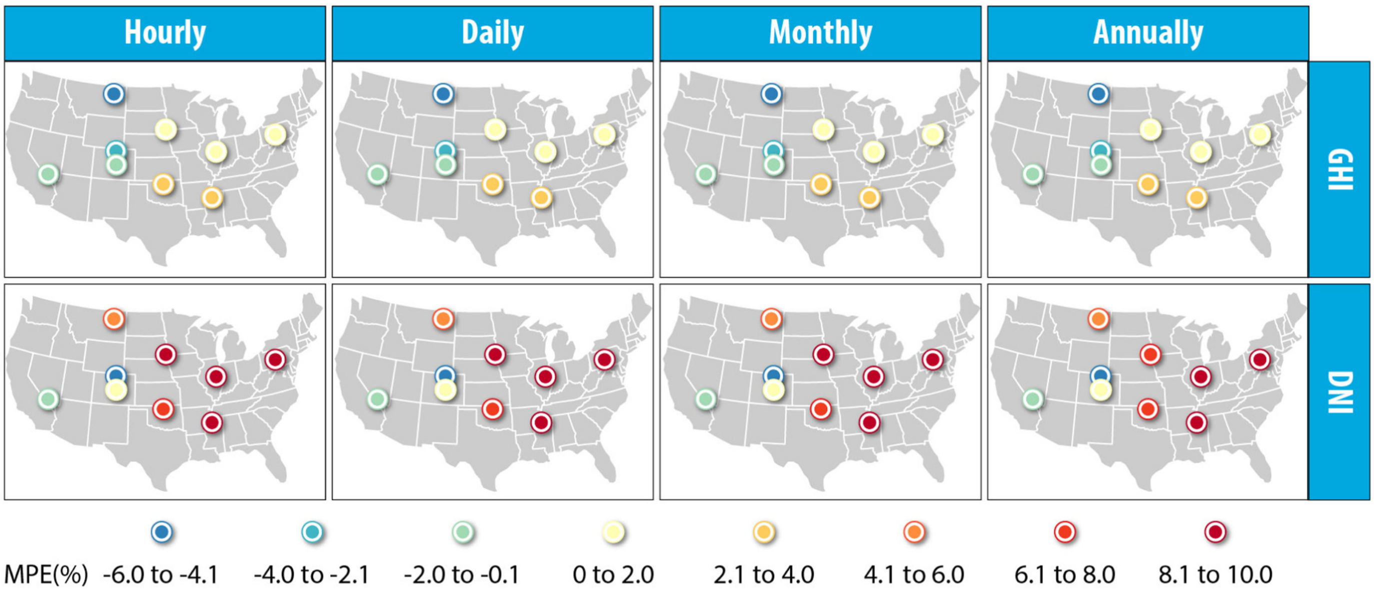

Fig. 4. The MPEs of the hourly-, daily-, monthly-, and annually-averaged GHI and DNI during 1998–2016.

magnitude of U_{95} dramatically decreases when the averaging timescale varies from hourly to annually. Therefore, %RMSE plays a dominant role in understanding the uncertainty of hourly-averaged GHI whereas the bias from surface observations becomes important in the monthly- and annually-averaged solar radiation. More details in the validation of the NSRDB can be found in [64].

3.4. Dissemination

Geographic Information System (GIS) layers are the core mechanism for developers, transmission planners, or conservationists to display geographic datasets in multiple geospatial processing programs. The GIS layers present in the NSRDB data include annual and multi-year mean GHI and DNI and multi-year mean capacity factors modeled for single-axis tracking photovoltaic (PV) panels and those with a fixed tilt angle of 20°. All the GIS layers are downloadable in several formats including the commonly used shapefile.

The NSRDB Viewer (https://nsrdb.nrel.gov/nsrdb-viewer), demonstrated in Fig. 6, is the major data delivery tool. Built on the NREL's OpenCarto, a web-based GIS framework, the NSRDB Viewer provides an intuitive, map-based interface for accessing raw time-series data or summarized GIS layers. Detailed instructions for downloading data from the NSRDB Viewer can be found on the website.

The NSRDB also provides an Application Programming Interface (API) (http://developer.nrel.gov/docs/solar/nsrdb) giving researchers, analysts, and developers an alternative way to efficiently download data using modern scripting languages, e.g., Python, MATLAB, or R. In addition, the API enables web developers to build their own applications without storing the approximately 50 terabytes of the NSRDB data.

4. NSRDB users and applications

Because of the improved accuracy and availability, the latest NSRDB has become a heavily and increasingly used dataset since its deployment. According to the web-based counter, the monthly data visit from the NSRDB viewer has doubled to more than 10,000 in 12 months, whereas more than 40% of the visit was from unique users. The NSRDB users include universities, local and federal governments, research institutes, public utilities, and numerous energy and high-technology companies across the world. Major use of the NSRDB can be categorized by energy-related and other applications.

The energy-related applications of the NSRDB include, but are not limited to, site and building design, facility integration, transmission and distribution planning, and strategic analysis. The serially completed, spatially continuous solar radiation from the NSRDB naturally meets the demands in developing solar radiation time-series [67] and Typical Meteorological Year (TMY) data for building analysis and the forecast and comparison of solar system performance [68–71]. In addition, capacity expansion and integrated assessment models rely on the spatially continuous NSRDB data to quantify the supply and quality of solar power and assess costs and feasibilities at a national scale. Production cost models used in grid integration studies to evaluate and optimize power plant dispatch require the high temporal-resolution NSRDB data from thousands of locations. Models that are intrinsically coupled with capacity expansion and production cost models—known as Geodesign models—are used to characterize and quantify solar supply. Those models use continuous or climatological data based on the NSRDB to evaluate land-use impacts, barriers, and scenarios of development futures. Solar energy facility developers use PVSyst or System Advisor Model (SAM) with long-term NSRDB data to estimate power output and assess specific cost and feasibility. A brief summary

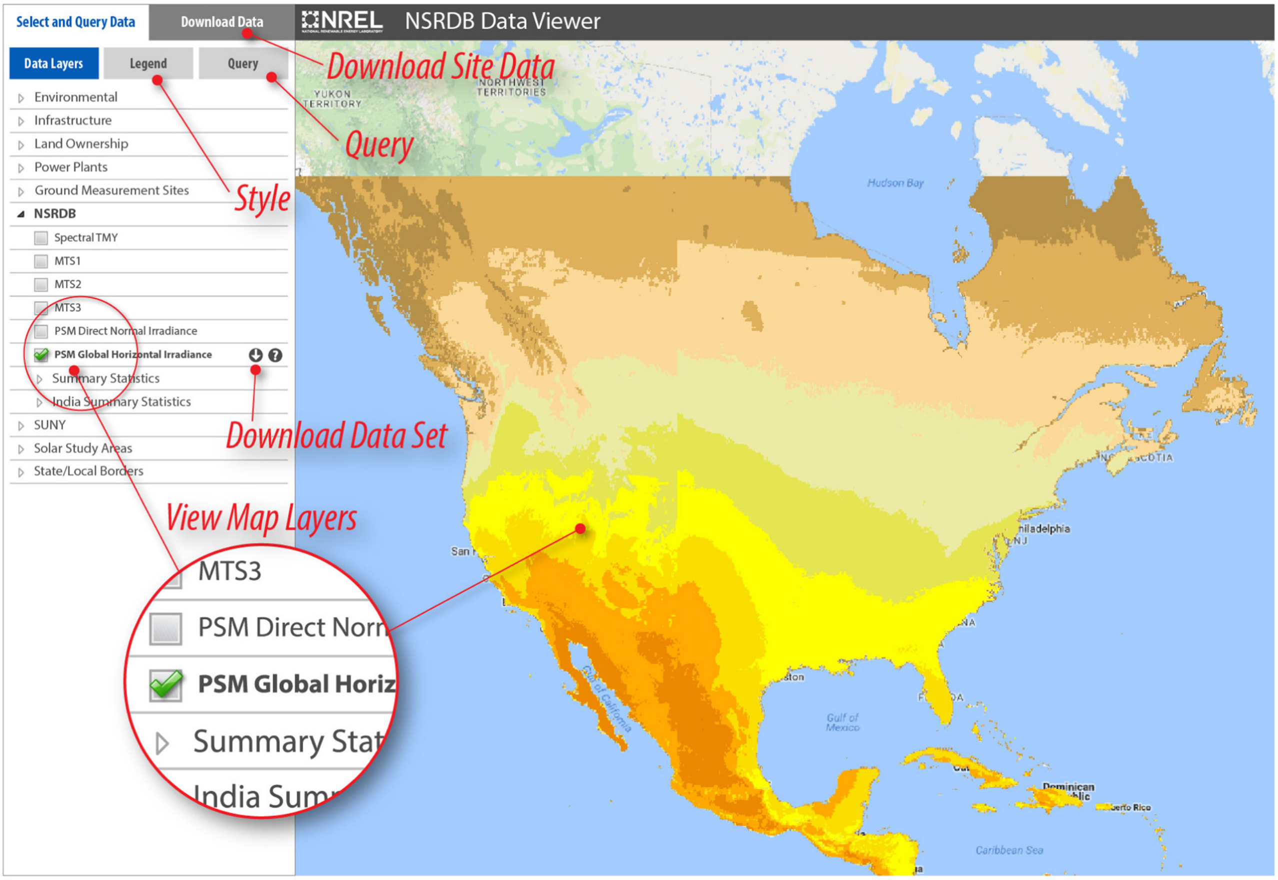

Fig. 6. A screenshot of the NSRDB Viewer.

Table 1
Models developed using the NSRDB.

Model	Description	NSRDB data	References
EnergyPlus	A whole building energy simulation program to model energy consumption and water use in buildings	TMY or historical solar radiation	[73]
HAIKU	A simulation model of regional electricity markets and interregional electricity trade in the continental United States	TMY or historical solar radiation	[74]
IPM	A multi-regional, dynamic, deterministic linear programming model of the U.S. electric power sector	Historical solar radiation	[75]
MARKAL	A generic model to represent the evolution during a period of 40–50 years of a specific energy system at a national, regional, state/province, or community level	TMY or historical solar radiation	[76]
NEMS	An integrated model of the U.S. energy system linked to a macroeconomic model	TMY or historical solar radiation	[77]
PLEXOS	A commercial production cost model	Historical solar radiation	[78,79]
PVSyst	A performance model for architecture, engineering, and education	TMY or historical solar radiation	[80]
PVWatts	For estimating the energy production of a grid-connected PV system	TMY or historical solar radiation	[81]
ReEDS	An optimization model of U.S. electricity sector with special focus on issues relating to renewable energy technologies	TMY or historical solar radiation	[82]
RPM	A capacity expansion model with high spatial and temporal resolution that can be used for mid- and long-term scenario planning of regional power systems	Historical solar radiation	[83]
SAM	A performance and financial model designed to facilitate decision making for people involved in the renewable energy industry	TMY or historical solar radiation	[84]
SIND Toolkit	For regional solar generation integration studies by providing modeled, coherent sub-hourly solar power data, information, and tools	1-min solar radiation from a statistical up sampling of the 30-min NSRDB.	[85]
SolarDS	A distributed solar capacity expansion model to evaluate scenarios in the U.S.	Historical solar radiation	[86]

and description of the abovementioned models is provided in Table 1. The NSRDB has also been used in bioenergy to evaluate algal biomass productivity potential in a variety of climatic zones [72].

In addition to the energy-related applications, the NSRDB has been employed in many other research areas. For example, the American Society of Heating Refrigerating and Air-Conditioning Engineers (ASHRAE) uses the NSRDB for climate research. The NSRDB has also been used by the American Cancer Society (ACS) to conduct cancer research because solar exposure is the primary vitamin D source that is associated with survival in multiple cancers. The residence-based ultraviolet radiation data from the NSRDB is used to examine its relationship to cancer outcomes and help understand the geographic disparities in cancer prognosis.

5. Conclusions and future plans

The NSRDB is a widely used public solar resource dataset that has been developed and updated during more than 20 years to reflect advances in solar radiation measurement and modeling. The most recent version of the NSRDB uses 30-min satellite products at a 4 × 4 km resolution that cover the period 1998–2016. The NREL-developed PSM was the underlying model for developing this recent update, which used this two-step physical model and took advantage of the progressive computing capabilities and high-quality meteorological datasets from NOAA's GOES; NIC's IMS; and NASA's MODIS and MERRA-2 products. The percentage biases in the latest NSRDB are approximately 5% for GHI and approximately 10% for DNI when compared to the long-term solar radiation observed by the ARM, NREL, and SURFARD stations across the United States.

Future updates of the NSRDB are expected annually. Advanced information in the planned dataset will involve new satellite retrievals and improved AOD data. However, future advancements in the PSM—e.g. identifying low clouds and fog in coastal areas, improving the discrimination of clouds from snow, providing specular reflection on bright surface, and reducing uncertainties of parallax especially under high-resolution conditions—are desired to further increase the accuracy of the NSRDB. Further, the Lambert-Bouguer Law [59] is almost non-exclusively utilized by physics-based radiative transfer models, including FARMS, that assume DNI is constituted of an infinite narrow beam. This assumption is interpreted differently in surface-based observations by pyrheliometers where direct solar radiation is defined as the "radiation received from a small solid angle centered on the sun's disc" [87]. To reduce this disagreement in principle, we employed an empirical model, DISC [27], to decompose DNI from the GHI in cloudy situations. Further efforts are underway in developing a new DNI model to bridge the gap between model simulation and surface observation. Additionally, the launch of GOES-16 is also expected to provide improved cloud products; however, this requires better capabilities to process larger volumes of data. Finally, while the PSM has been applied to the GOES satellites the methods and models are equally applicable to any other geostationary satellites. Therefore, future work will involve developing global capabilities in collaboration with various national and international partners.

Acknowledgements

This work was supported by the U.S. Department of Energy under Contract No. DE-AC36-08GO28308 with the National Renewable Energy Laboratory. The U.S. Government retains and the publisher, by accepting the article for publication, acknowledges that the U.S. Government retains a nonexclusive, paid-up, irrevocable, worldwide license to publish or reproduce the published form of this work, or allow others to do so, for U.S. Government purposes.

We acknowledge the DOE Solar Energy Technologies Office for supporting this research. Specifically, we thank Dr. Tassos Golnas, technology manager for the Systems Integration team of the DOE, Office of Energy Efficiency and Renewable Energy, Solar Energy Technologies Office, for his support and encouragement. We also thank Billy Roberts for producing many high-quality figures that appear throughout the text, Dr. Michael Foster from the University of Wisconsin, Dr. Andy Heidinger from NOAA for providing the cloud property retrievals, Dr. Christian Gueymard from Solar Consulting Services for providing the aerosol data and the REST2 model, and NASA's MERRA-2 team for making their product available. The DOE ARM program and NOAA's Global Monitoring Division deserve special thanks for making high-quality solar radiation data available because they were crucial to our validation efforts. We also thank NIC for making the IMS data available and Professor Crystal Schaaf and her team from Boston University for developing the MODIS products of surface albedo. Finally, we appreciate the tireless efforts of NREL's HPC team, who provide us with the computing resources on the Peregrine supercomputer.

References

[1] He G, Kammen D. Where, when and how much solar is available? A provincial-scale solar resource assessment for China. Renew Energy 2016;85:74–82.
[2] Master G. Renewable and efficient electric power systems. Hoboken, NJ: John Wiley & Sons; 2004.
[3] Kleissl J. Solar energy forecasting and resource assessment. Academic Press; 2013.
[4] Gurtuna O, Prevot A. An overview of solar resource assessment using meteorological satellite data. Recent Advances in Space Technologies (RAST), 2011 5th International Conference on. 10.1109/RAST.2011.5966825:209 − 12; 2011.
[5] Fuquay D, Buettner K. Laboratory investigation of some characteristics of the Eppley pyrheliometer. Eos Trans Am Geophys Union 1957:38. https://doi.org/10.1029/TR038i001p00038.
[6] Kerr J, Thurtell G, Tanner C. An integrating pyranometer for climatological observer stations and mesoscale networks. J Appl Meteorol Climatol 1967;6:688–94.
[7] Reda I, Stoffel T, Myers D. A method to calibrate a solar pyranometer for measuring reference diffuse irradiance. Sol Energy 2003;74:103–12.
[8] Reda I. Method to calculate uncertainties in measuring shortwave solar irradiance using thermopile and semiconductor solar radiometers. NREL/TP-3B10-52194; 2011.
[9] Stoffel T, Reda I, Myers D, Renne D, Wilcox S, Treadwell J. Current issues in terrestrial solar radiation instrumentation for energy, climate, and space applications. Metrologia 2000;37:399–402.
[10] Stokes GM, Schwartz SE. The atmospheric radiation measurement (ARM) Program: programmatic background and design of the cloud and radiation test bed. Bull Am Meteor Soc 1994;75:1201–21.
[11] Ohmura A, Dutton EG, Forgan B, Frohlich C, Gilgen H, Hegner H, et al. Baseline Surface Radiation Network (BSRN/WCRP): new precision radiometry for climate research. Bull Am Meteorol Soc 1998;79:2115–36.
[12] Augustine J, DeLuisi J, Long C. SURFRAD—A national surface radiation budget network for atmospheric research. Bull Am Meteorol Soc 2000;81:2341–57.
[13] Hicks B, DeLuisi J, Matt D. The NOAA integrated surface irradiance study (ISIS)—A new surface radiation monitoring network. Bull Am Meteorol Soc 1996;77:2857–64.
[14] Xie Y, Liu YG. A new approach for simultaneously retrieving cloud albedo and cloud fraction from surface-based shortwave radiation measurements. Environ Res Lett 2013:8. https://doi.org/10.1088/748-9326/8/4/044023.
[15] Xie Y, Liu YG, Long CN, Min QL. Retrievals of cloud fraction and cloud albedo from surface-based shortwave radiation measurements: a comparison of 16 year measurements. J Geophys Res Atmos 2014;119:8925–40.
[16] Long C, Ackerman TP, Gaustad KL, Cole JNS. Estimation of fractional sky cover from broadband shortwave radiometer measurements. J Geophys Res 2006:111. https://doi.org/10.1029/2005JD006475.
[17] Xie Y, Sengupta M, Dudhia J. A fast all-sky radiation model for solar applications (FARMS): algorithm and performance evaluation. Sol Energy 2016;135:435–45.
[18] Pinker R, Frouin R, Li Z. A review of satellite methods to derived surface shortwave irradiance. Remote Sens Environ 1995;51:108–24.
[19] Schmetz J. Towards a surface radiation climatology: retrieval of downward irradiances from satellites. Atmos Res 1989;23:287–321.
[20] Myers D. Solar radiation: practical modeling for renewable energy applications. New York: CRC Press; 2013.
[21] Hammer A, Heinemann D, Hoyer C, Kuhlemann R, Lorenz E, Müller R, et al. Solar energy assessment using remote sensing technologies. Remote Sens Environ 2003;86:423–32.
[22] Cano D, Monget J, Albuisson M, Guillard H, Regas N, Wald L. A method for the determination of the global solar radiation from meteorological satellite data. Sol Energy 1986;37:31–9.
[23] Justus C, Paris M, Tarpley J. Satellite-measured insolation in the United States, Mexico, and South America. Remote Sens Environ 1986;20:57–83.
[24] Tarpley J. Estimating incident solar radiation at the surface from geostationary satellite data. J Appl Meteorol 1979;18:1172–81.
[25] Perez R, Ineichen P, Moore K, Kmiecik M, Chain C, George R, et al. A new operational model for satellite-derived irradiances: description and validation. Sol Energy 2002;73:307–17.
[26] Ineichen P, Perez R, Seal R, Maxwell E, Zalenka A. Dynamic global-to-direct irradiance conversion models. Ashrae Trans 1992;98:354–69.
[27] Maxwell E. A quasi-physical model for converting hourly global horizontal to direct normal insolation. Golden, CO: Solar Energy Research Inst; 1987.
[28] Rigollier C, Lefèvre M, Wald L. The method Heliosat-2 for deriving shortwave solar radiation from satellite images. Sol Energy 2004;77:159–69.
[29] Diabaté L, Demarcq H, Michaud-Regas N, Wald L. Estimating incident solar radiation at the surface from images of the Earth transmitted by geostationary satellites: the Heliosat Project. Int J Sol Energy 1987;5:261–78.
[30] Cebecauer T, Šúri M, Perez R. High performance MSG satellite model for operational solar energy applications. ASES National Solar Conference. Phoenix, USA; 2010.
[31] Šúri M, Cebecauer T, Skoczek A. SolarGIS: solar data and online applications for PV planning and performance assessment. 26th European photovoltaics solar energy conference. Hamburg, Germany; 2011.
[32] Diak G, Gautier C. Improvements to a simple physical model for estimating insolation from GOES data. J Clim Appl Meteorol 1983;22:505–8.
[33] Gautier C, Diak G, Masse S. A simple physical model to estimate incident solar radiation at the surface from GOES satellite data. J Appl Meteorol 1980;19:1005–12.
[34] Gautier C. Mesoscale insolation variability derived from satellite data. J Appl Meteorol 1982;21:52–8.
[35] Pinker RT, Laszlo I. Modeling surface solar irradiance for satellite applications on a global scale. J Appl Meteorol 1992;31:194–211.
[36] Xie Y. Study of ice cloud properties from synergetic use of satellite observations and modeling capabilities. College Station, TX: Texas A&M University; 2010.
[37] Xie Y, Yang P, Kattawar GW, Minnis P, Hu YX, Wu D. Determination of ice cloud models using MODIS and MISR data. Int Remote Sens 2012;33:4219–53.
[38] Xie Y, Yang P, Liou KN, Minnis P, Duda DP. Parameterization of contrail radiative properties for climate studies. Geophys Res Lett 2012:39. https://doi.org/10.1029/2012GL054043.
[39] Minnis P, Sun-Mack S, Young D, Heck PW, Garber DP, Chen Y, et al. CERES edition-2 cloud property retrievals using TRMM VIRS and TERRA and AQUA MODIS data, part I: algorithms. IEEE Trans Geosci Remote Sens 2011;49:4374–400.
[40] Schmit T, Gunshor M, Menzel W, Gurka J, Li J, Bachmeier A. Introducing the next-generation advanced baseline imager on GOES-R. Bull Am Meteorol Soc 2005;86:1079–96.
[41] Gelaro R, McCarty W, Suárez M, Todling R, Molod A, Takacs L, et al. The modern-era retrospective analysis for research and applications, version 2 (merra-2). J Clim 2017;30:5419–54.
[42] Maxwell E. METSTAT—The solar radiation model used in the production of the National Solar Radiation Data Base (NSRDB). Sol Energy 1998;62:263–79.
[43] Wilbert S. National solar radiation database 1991–2005 update: user's manual. Golden, CO: National Renewable Energy Laboratory; 2007.
[44] Möser W, Raschke E. Mapping of global radiation and cloudiness from METEOSAT image data. Meteorol Rundsch 1983;36:33–41.
[45] Dedieu G, Deschamps P, Kerr Y. Satellite estimation of solar irradiance at the surface of the earth and of surface albedo using a physical model applied to meteosat data. J Clim Appl Meteorol 1987;26:79–87.
[46] Heidinger A, Foster M, Walther A, Zhao X. The pathfinder atmospheres-extended AVHRR climate dataset. Bull Am Meteorol Soc 2014;95:909–22.
[47] Sengupta M, Gotseff P. Evaluation of clear sky models for satellite-based irradiance estimates. NREL/TP-5D00-60735; 2013.
[48] Sun Q. Assessing change in the Earth's land surface albedo with moderate resolution satellite imagery. Boston, MA: Boston University; 2014.
[49] Gaclaurin G, Sengupta M, Xie Y, Gilroy N. Development of MODIS-derived surface albedo: enhanced model input to the NSRDB. Golden, CO: National Renewalbe Energy Laboratory; 2016.
[50] Clough S, Iacono M, Moncet J. Line-by-line calculations of atmospheric fluxes and cooling rates: application to water vapor. J Geophys Res 1992;97:15761–85.
[51] Gueymard C. REST2: high-performance solar radiation model for cloudless-sky irradiance, illuminance, and photosynthetically active radiation - Validation with a benchmark dataset. Sol Energy 2008;82:272–85.
[52] Gueymard C. A two-band model for the calculation of clear sky solar irradiance, illuminance, and photosynthetically active radiation at the earth's surface. Sol Energy 1989;43:253–65.
[53] Bird R, Hulstrom R. A simplified clear sky model for direct and diffuse insolation on horizontal surfaces. Golden, CO: Solar Energy Research Institute; 1981.
[54] Badescu V, Gueymard C, Cheval S, Oprea C, Baciu M, Dumitrescu A, et al. Computing global and diffuse solar hourly irradiation on clear sky. Review and testing of 54 models. Renew Sustain Energy Rev 2012;16:1636–56.
[55] Kambezidis H. Solar radiation modelling: the latest version and capabilities of MRM. J Fundam Renew Energy Appl 2017;7. https://doi.org/10.4172/2090-4541.1000e114.
[56] Kambezidis H, Psiloglou B, Karagiannis D, Dumka U, Kaskaoutis D. Recent improvements of the meteorological radiation model for solar irradiance estimates under all-sky conditions. Renew Energy 2017;93:142–58.
[57] Kambezidis H, Psiloglou B, Karagiannis D, Dumka U, Kaskaoutis D. Meteorological radiation model (MRMv6.1): improvements in diffuse radiation estimates and a new approach for implementation of cloud products. Renew Sustain Energy Rev 2017;74:616–37.
[58] Chandrasekhar S. Radiative transfer. Oxford: Oxford Univ. Press; 1950.
[59] Liou KN. An introduction to atmospheric radiation. 2nd ed Amsterdam; Boston: Academic Press; 2002.
[60] Wiscombe WJ. The delta-M method: rapid yet accurate radiative flux calculations for strongly asymmetric phase functions. J Atmos Sci 1977;34:1408–22.
[61] Mlawer EJ, Taubman SJ, Brown PD, Iacono MJ, Clough SARRTM. A validated correlated-k model for the longwave. J Geophys Res 1997;102:16663–82.
[62] Oreopoulos L, Barker HW. Accounting for subgrid-scale cloud variability in a multi-layer 1-D solar radiative transfer algorithm. Q J R Meteorol Soc 1999;125:301–30.
[63] Stamnes K, Tsay SC, Wiscombe W, Jayaweera K. Numerically stable algorithm for discrete-ordinate-method radiative transfer in multiple scattering and emitting layered media. Appl Opt 1988;27:2502–9.
[64] Habte A, Sengupta M, Lopez A. Evaluation of the National Solar Radiation Database (NSRDB): 1998–2015 Golden, CO: National Renewable Energy Laboratory; 2017.
[65] BIPM, IFCC, ISO, UIUPAP, OIML. Evaluation of measurement data — Guide to the expression of uncertainty in measurement. Joint Committee for Guides in Metrology (JCGM); 2008.
[66] Habte A, Sengupta M, Andreas A, Wilcox S, Stoffel T. Intercomparison of 51 radiometers for determining global horizontal irradiance and direct normal irradiance measurements. Sol Energy 2016;133:372–93.
[67] Fortuna L, Nunnan G, Nunnan S. Nonlinear modeling of solar radiation and wind speed time series. Switzerland: Springer; 2016.
[68] Wilcox S, Marion W. Users manual for TMY3 data sets. NREL Technical Report, NREL/TP-581-4315; 2008.
[69] Denholm P, Margolis R. Land-use requirements and the per-capita solar footprint for photovoltaic generation in the United States. Energy Policy 2008;36:3531–43.

[70] Renne D, George R, Wilcox S, Stoffel T, Myers D, Heimiller D. Solar resource assessment. Golden, CO: National Renewable Energy Laboratory; 2008.
[71] Williams M, Kerrigan S. How typical is solar energy? A 6 year evaluation of typical meteorological data (TMY3). Proceedings of World Renewable Energy Forum; 2012.
[72] McGowen J, Knoshaug E, Laurens L, Dempster T, Pienkos P, Wolfrum E, et al. The algae testbed public-private partnership (ATP3) framework: establishment of a national network of testbed sites to support sustainable algae production. Algal Res 2017;25:168–77.
[73] Crawley D, Pedersen C, Lawrie L, Winkelmann F. EnergyPlus: energy simulation program. ASHRAE J 2000;42:49.
[74] Paul A, Burtraw D. The RFF haiku electricity market model. Washington, DC: Resources for the Future; 2002.
[75] EPA US. Documentation for EPA base case v.4.10 using the integrated planning model; 2010.
[76] Fishbone L, Abilock H. Markal, a linear-programming model for energy systems analysis: technical description of the bnl version. Int J Energy Res 1981;5:353–75.
[77] Gabriel S, Kydes A, Whitman P. The national energy modeling system: a large-scale energy-economic equilibrium model. Oper Res 2001;49:14–25.
[78] Ellison J, Bhatnagar D, Karlson B. Maui energy storage study. Albuquerque, NM: Sandia National Laboratory; 2012.
[79] Rastler D. MISO energy storage study phase 1 report. Pala Alto, CA: Electric Power Research Institute; 2011.
[80] Mermoud A. Pvsyst: software for the study and simulation of photovoltaic systems. Geneva, Switzerland: University of Geneva; 2012.
[81] Dobos A. PVWatts version 5 manual. Gloden, CO: National Renewable Energy Laboratory; 2014.
[82] Short W, Sullivan P, Mai T, Mowers M, Urarte C, Blair N, et al. Regional energy deployment system (ReEDS). Golden, CO: National Renewable EnergyLaboratory; 2011.
[83] Gable G. Large package software: a neglected technology. J Glob Inf Manag 1998;6:3–4.
[84] Blair N, Dobos A, Freeman J, Neises T, Wagner M, Ferguson T, et al. System advisor model, SAM 2014.1. 14: general description. Golden, CO: National Renewable Energy Laboratory; 2014.
[85] Hummon M, Ibanez E, Brinkman G, Lew D. Sub-hour solar data for power system modeling from static spatial variability analysis. 2nd international workshop on integration of solar power in power systems. Lisbon, Portugal; 2012.
[86] Denholm P, Drury E, Margolis R. Solar deployment system (SolarDS) model: documentation and sample results. Golden, CO: National Renewable Energy Laboratory; 2009.
[87] ISO. ISO 9060: 1990: Specification and classification of instruments for measuring hemispherical solar and direct solar radiation. Geneva, Switzerland; 1990.
