# Supplementary material for: Advanced liquid crystal-based switchable optical devices for light protection applications: principles and strategies
Source: Light Sci Appl. 2023 Jan 3;12:11. doi: 10.1038/s41377-022-01032-y (PMC9807646; doi:10.1038/s41377-022-01032-y)
Supplement: Supplementary file 4 — Fig 4 copyright promotion [file 41377_2022_1032_MOESM4_ESM.pdf]

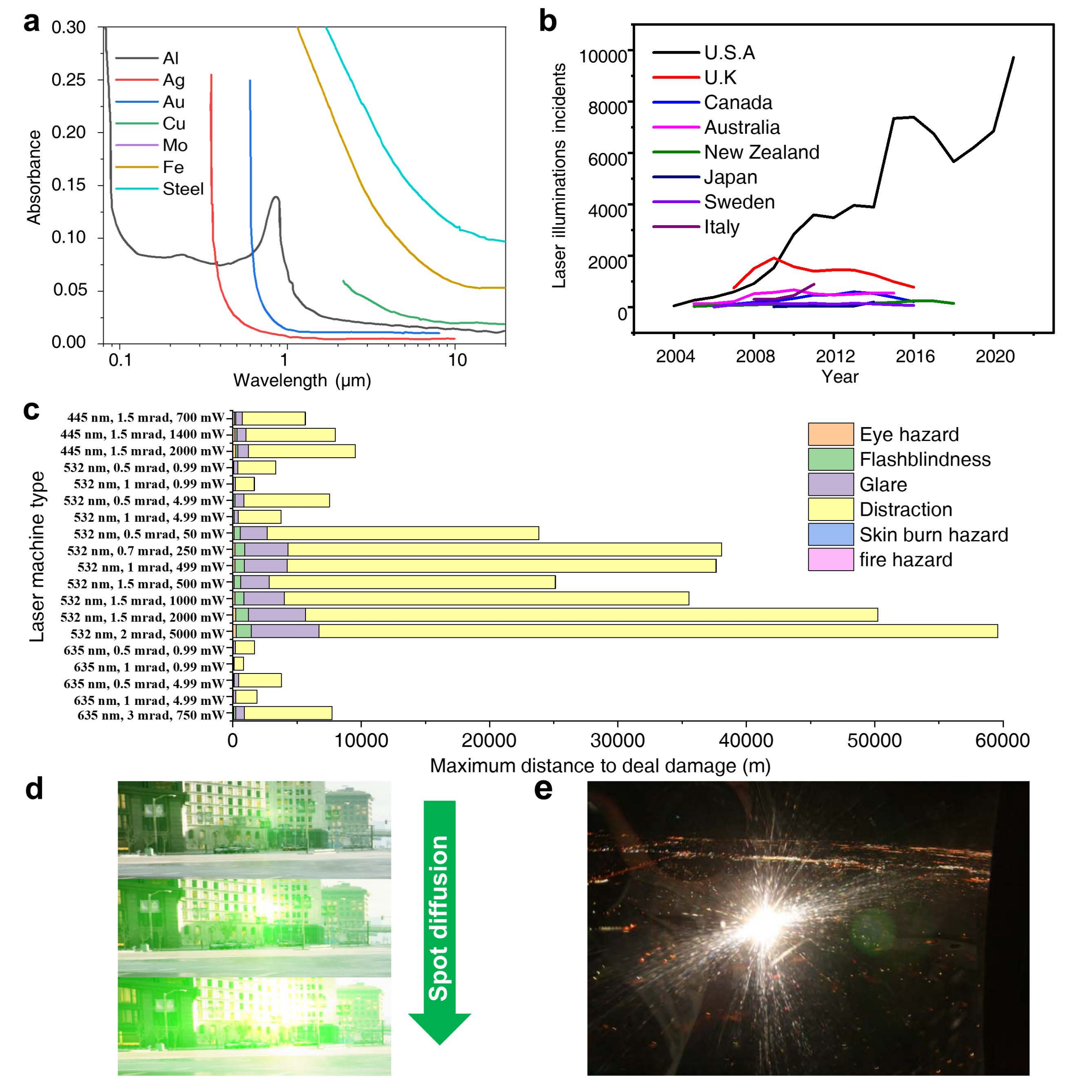

a
b
c
d
e
Absorbance
Wavelength (μm)
Al
Ag
Au
Cu
Mo
Fe
Steel
Laser illuminations incidents
Year
U.S.A
U.K
Canada
Australia
New Zealand
Japan
Sweden
Italy
Laser machine type
445 nm, 1.5 mrad, 700 mW
445 nm, 1.5 mrad, 1400 mW
445 nm, 1.5 mrad, 2000 mW
532 nm, 0.5 mrad, 0.99 mW
532 nm, 1 mrad, 0.99 mW
532 nm, 0.5 mrad, 4.99 mW
532 nm, 1 mrad, 4.99 mW
532 nm, 0.5 mrad, 50 mW
532 nm, 0.7 mrad, 250 mW
532 nm, 1 mrad, 499 mW
532 nm, 1.5 mrad, 500 mW
532 nm, 1.5 mrad, 1000 mW
532 nm, 1.5 mrad, 2000 mW
532 nm, 2 mrad, 5000 mW
635 nm, 0.5 mrad, 0.99 mW
635 nm, 1 mrad, 0.99 mW
635 nm, 0.5 mrad, 4.99 mW
635 nm, 1 mrad, 4.99 mW
635 nm, 3 mrad, 750 mW
Maximum distance to deal damage (m)
Eye hazard
Flashblindness
Glare
Distraction
Skin burn hazard
fire hazard
Spot diffusion

Kampweg 5
P.O. Box 23
3769 ZG Soesterberg
The Netherlands

www.tno.nl

T +31 34 635 62 11
F +31 34 635 39 77
info-DenV@tno.nl

TNO report

TNO-DV 2009 C264

The outdoor use of lasers and other high intensity light sources in relation to air traffic safety

Date	June 2009
Author(s)	Dr A. Toet<br>N. van der Leden, BSc<br>J.W.A.M. Alferdinck, BSc
Assignor	Inspectie van Verkeer en Waterstaat
Project number	032.13663
Classification report	Unclassified
Title	Unclassified
Abstract	-
Report text	-
Appendices	-
Number of pages	54 (incl. appendices)
Number of appendices	-

All rights reserved. No part of this report may be reproduced and/or published in any form by print, photoprint, microfilm or any other means without the previous written permission from TNO.

All information which is classified according to Dutch regulations shall be treated by the recipient in the same way as classified information of corresponding value in his own country. No part of this information will be disclosed to any third party.

In case this report was drafted on instructions, the rights and obligations of contracting parties are subject to either the Standard Conditions for Research Instructions given to TNO, or the relevant agreement concluded between the contracting parties. Submitting the report for inspection to parties who have a direct interest is permitted.

© 2009 TNO

Samenvatting

Lichtbronnnen met een hoge intensiteit zoals skybeamers worden steeds vaker gebruikt in de open lucht bij evenementen of als aandachttrekkers bij commerciële vestigingen. Andere lichtbronnen met een hoge intensiteit zoals spotlights worden vaak ingezet bij bouwwerkzaamheden en sportevenementen. Als gevolg daarvan ontvangt de Inspectie Verkeer en Waterstaat (IVW) regelmatig het verzoek om toestemming te verlenen voor het gebruik van lichtbronnen met een hoge intensiteit in de open lucht. Omdat niet bekend is hoe deze lichtbronnen de luchtvaartveiligheid kunnen beïnvloeden, is het IVW momenteel niet in staat de risico's die het gebruik van deze lichtbronnen opleveren voor de luchtvaartveiligheid goed in te schatten en hun gebruik op een verantwoorde manier te reguleren. IVW heeft daarom TNO gevraagd om te onderzoeken welke effecten lichtbronnen met een hoge intensiteit (behalve lasers) kunnen hebben op de luchtvaartveiligheid , en welke veiligheidsmaatregelen eventueel genomen zouden moeten worden om deze lichtbronnen veilig in het open luchtruim te kunnen gebruiken. Verder verzocht IVW TNO om na te gaan welke veiligheidsmaatregelen er reeds zijn getroffen bij enkele lasersystemen die in Nederland worden gebruikt voor atmosferisch onderzoek, en om vast te stellen of die maatregelen afdoende zijn om de luchtvaartveiligheid te garanderen, en om eventueel aanvullend maatregelen voor te stellen.

We hebben de effecten onderzocht van verschillende hoge intensiteits lichtbronnen met een breed-spectrum, en van een 3,5 mW laser pointer die groen licht produceert. Verder onderzochten we de veiligheidsmaatregelen die worden toegepast bij twee lasersystemen die in Nederland worden gebruikt voor atmosferisch onderzoek.

Kennis over de visuele effecten van hoge intensiteits lichtbronnen is essentieel om te kunnen bepalen welke risico's hun gebruik in de open lucht oplevert voor de luchtvaart, en welke maatregelen moeten worden genomen om die risico's te beperken. Evenzo dient de effectiviteit van veiligheidsmaatregelen voor lasers bekend te zijn om vast te kunnen stellen welke risico's deze voor de luchtvaart vormen.

Omdat de visuele en biologische effecten van hoge intensiteits lichtbronnen gelijk zijn aan die van lasers, kan de bestaande laser veiligheids zonering rond luchthavens ook worden gebruikt om het gebruik van hoge intensiteits lichtbronnen te reguleren.

Op de grond hebben we de luminantie van verschillende representatieve lichtbronnen met een hoge intensiteit gemeten als functie van de afstand tot de bron, alsmede hun bundeldiameter. Uit deze metingen berekenden we de afstanden waarop hun illuminantie de kritieke waarden overschreed die is voorgeschreven voor de laser veiligheidszones rond luchthavens. We hebben ook de operators van twee wetenschappelijke lasersystemen geïnterviewd over hun veiligheidsprotocollen.

We observeerden de lichtbronnen ook vanuit een helikopter om de berekende hinder afstanden te verifiëren. We vonden dat bij normaal gebruik en zowel stilstaand als bewegend hoge intensiteits lichtbronnen in de vrije atmosfeer buiten de laser-vrije zone geen gevaar vormen voor de luchtvaartveiligheid. Binnen de laser-vrije zone dient het gebruik van hoge intensiteits lichtbronnen te worden vermeden omdat ze afleiding kunnen veroorzaken. Het doelbewust aanstralen van een luchtvaartuig met elke hoge intensiteits lichtbron kan in principe verblinding en afleiding veroorzaken. Verder kan

er natuurlijk afleiding voorkomen wanneer een hoge intensiteits lichtbron de enige heldere lichtbron in de omgeving is, zelfs als deze niet direct op het luchtvaartuig is gericht.

De door ons gebruikte laser pointer veroorzaakte zeer hinderlijke visuele effecten wanneer zijn bundel op de canopy van de helikopter was gericht. Het licht van de laser pointer was onacceptabel helder en veroorzaakte aanzienlijke verblinding op een afstand van 500 m. Op deze afstand ervoeren de waarnemers ook kortstondige nabeelden. De laser pointer was alleen zichtbaar wanneer hij direct op het oog was gericht.

De veiligheidsmaatregelen die worden toegepast bij de twee lasersystemen die momenteel in Nederland worden gebruikt voor atmosferisch onderzoek (waarnemers en radarsystemen) beperken de risico’s die hun gebruik in de open lucht oplevert voor de luchtvaart tot een minimum.

Wij stellen vast dat er geen reden is om het gebruik van hoge intensiteits lichtbronnen buiten de laser vrije zone te beperken. Binnen de laser vrije zone dient het gebruik van hoge intensiteits lichtbronnen te worden vermeden omdat ze afleiding kunnen veroorzaken. Het doelbewust volgen van een vliegtuig met een hoge intensiteits lichtbron, en in het bijzonder met een laser, kan het gezichtvermogen van de piloot ernstig hinderen. De veiligheidmaatregelen die zijn genomen om een veilig gebruik van wetenschappelijke lasers in de open lucht te garanderen zijn voldoende.

Summary

High intensity light sources like searchlights (in the Netherlands sometimes referred to as skybeamers) are increasingly deployed in the open air at public events, funfairs, or to attract attention to commercial venues. Other high intensity light sources like (handheld) spotlights are frequently used near construction sites or sport events.
As a result, the Inspectie Verkeer en Waterstaat (the Dutch Civil Aviation Authority, CAA NL) regularly receives applications for permission to use high intensity light sources in the open airspace. Since it is currently not known how high intensity light sources may affect air traffic safety, the CAA NL is not able to assess the risks involved in the deployment of high intensity lights in the navigable airspace or to appropriately regulate their use. The CAA NL therefore commissioned TNO to investigate the effects of high intensity light sources (other than lasers) on air traffic, and (if necessary) to propose regulations and/or safety measures. In addition, the CAA NL asked TNO to investigate the safety measures that have been implemented or that may be required to guarantee the safe use of high power scientific laser installations in the Netherlands.

We investigated the effects of several broadband high intensity light sources and a common 3.5 mW green laser pointer on pilot vision at night. We also investigated the safety measures that are deployed for two laser systems for atmospheric research in the Netherlands.

Accurate knowledge of the visual effects induced by high intensity lights is required to assess the risks for air traffic related to their deployment in the navigable airspace and to appropriately regulate their use. Similarly, the effectiveness of safety measures for scientific laser systems should be known in order to assess their risks for air traffic.

Since the visual and biological effects of high intensity lights are similar to those induced by lasers, existing laser safety zoning around airports can also be applied to regulate the use of other high intensity light sources.

On the ground we measured the luminance of several representative high intensity lights as a function of distance, and their beam width. From these measurements we calculated the distances at which their illuminance exceeded the critical levels associated with laser safety zones around airports. We also interviewed the operators of two operational scientific laser systems on their safety protocols.

We also performed helicopter flight tests to validate the computed visual interference distances. We found that in normal use, broadband high intensity light sources pointing in navigable airspace, whether stationary or moving, cause no concern for aviation safety outside the Laser Free Zone. Intentional tracking of an aircraft with a high intensity light source may cause serious glare and distraction. Distraction may also occur when the high intensity light is the only bright light source in its environment, even when the light is not directed at the plane.

The visual impact of the laser pointer was serious when its beam hit the canopy of the helicopter. The light was unacceptably bright and caused serious glare at a distance of about 500 m. At this distance it also elicited brief afterimages. The laser pointer could not be seen when its beam did not directly hit the eye.

Both scientific systems (at ESA-ESTEC, Noordwijk and at RIVM, Bilthoven) that are currently operational for atmospheric research have sufficient safety precautions (radar and human observers) and cause no appreciable risk for aviation safety.

We conclude that there is no need to restrict broadband high intensity light sources pointing in navigable airspace anywhere outside the Laser Free Zone. The use of high intensity light sources inside the Laser Free Zone should be avoided since they can cause distraction. Intentionally tracking aircraft with a high intensity light, and especially with lasers, can seriously impair pilot vision. The safety protocols of the scientific laser systems that are currently used in the Netherlands (radar and human observers) are sufficient to guarantee aviation safety for commercial aircraft.

Contents

Samenvatting 3

Summary 5

1 Introduction 9
1.1 High intensity lights and their outdoor use 9
1.2 Types of high intensity lights 10
1.3 Outdoor applications of high intensity lights 11

2 High intensity lights and pilot vision 13
2.1 Lasers 13
2.2 Broadband high intensity lights 15

3 Experimental validation studies and observations 19
3.1 The high intensity lights 19
3.2 Ground measurements 23
3.3 Flight measurements and oberservations 31
3.4 The Luxor light beam 41

4 Safety measures 45
4.1 Measures by proponents 45
4.2 Measures by aviation authorities 45

5 Two scientific laser systems and their safety protocols 47
5.1 The Esa ESTEC LID AR system 47
5.2 The RIVM LIDAR system 48

6 Conclusions 51

7 References 52

8 Signature 54

1 Introduction

The Inspectie Verkeer en Waterstaat (the Dutch Civil Aviation Authority, CAA NL) commissioned TNO to investigate the effects of high intensity light sources (other than lasers) on air traffic, and (if necessary) to propose regulations and/or safety measures. In addition, the CAA NL asked TNO to investigate the safety measures that have been implemented or that may be required to guarantee the safe use of high power scientific laser installations in the Netherlands.

High intensity light sources like searchlights (in the Netherlands sometimes referred to as skybeamers) are increasingly deployed in the open air at public events, funfairs, or to attract attention to commercial venues. Other high intensity light sources like (handheld) spotlights are frequently used near construction sites or sport events. As a result, the CAA NL regularly receives applications for permission to use high intensity light sources in the open airspace. The ICAO (International Civil Aviation Organization), the SAE (Society of Automotive Engineers) and the ANSI (American National Standards Institute) have published guidelines and control measures for the safe use of lasers in the navigable airspace (American National Standards Institute, 2005; ICAO-LFSSG, 2003; SAE, 1999; SAE, 2003; SAE, 2004; SAE, 2005). However, there are no similar publications on other high intensity light sources since their effects on air traffic are simply not known. The CAA NL is therefore currently not able to accurately estimate the risks for air traffic related to the deployment of high intensity lights in the navigable airspace or to appropriately regulate their use.

High power lasers are increasingly deployed in the navigable airspace in the Netherlands for atmospheric research purposes. These lasers produce powerful beams that usually point straight upwards. Since air traffic is extremely dense in the Netherlands, and since at least one of these laser systems is near Schiphol Airport (a main international airport in the Netherlands) and directly underneath one of its main approach routes, airplanes will frequently encounter these laser beams. It is therefore important to know which effects these laser beams may have on pilots, and to know which safety measures (if any) should be taken to avoid that these beams cause any adverse effects on pilots.

In this study we first performed a literature study to find out what is currently known about the effects of high intensity lights (other than lasers) on pilot vision. Based on the outcome of this study we derived threshold intensity measures that can be used to implement safety zones around airports. Then we performed some experiments to validate these derived measures. Our validation studies involved actual nighttime low-level helicopter flights through and around high intensity (search light) beams. Finally, we interviewed the operators of two operational scientific laser systems in the Netherlands and assessed the effectiveness of safety protocols of these systems.

1.1 High intensity lights and their outdoor use

Following the SAE (SAE, 2008) we will refer to light sources exceeding 250,000 candela as high intensity lights. In this study we will only consider high intensity lights that are used in the open airspace.

Lights not directed or reflected into the navigable airspace are not likely to interfere with aircraft operations. Laser systems are beyond the scope of this study.

In the next sections we will first review the different types of high intensity light sources that are frequently used in outdoor operations. Then we will give a brief overview of the most common applications of these lights.

1.2 Types of high intensity lights

In this section we will discuss the main technologies that are currently applied in high intensity lighting.

1.2.1 Carbon arc lights

This type of lamp produces light by an electric arc (spark) that crosses the gap between two closely spaced carbon electrodes when a direct current is applied. Although carbon arc searchlights are based on an older technology, they are still in use today.

1.2.2 Enclosed arc lights

This type of lamp consists of electrodes (typically made of tungsten) that are enclosed in a bulb made from optically clear quartz material. These lamps have the highest luminance and radiance of any continuously operating light source and are the closest approximation to a true "point" source.

1.2.3 High-intensity discharge lights

In high-intensity discharge (HlD) lighting the electric arc that is established between two electrodes in a gas-filled tube causes a metallic vapor to produce radiant energy. The electrodes are close together and the gases in the tube may be highly pressurized. This allows the arc to generate extremely high temperatures, causing metallic elements within the gas atmosphere to vaporize and release large amounts of visible radiant energy. The type of lamp is often named by the gas or vapor contained in the bulb; including neon, argon, xenon, krypton, sodium, metal halide, and mercury.

Mercury vapor or halide lighting is the most common HID technology. The mercury arc produces a bluish light that renders colors poorly but improves the efficiency over incandescent lamps. Many mercury vapor lamps have halide additives that improve the color rendering index (CRl).

Xenon lamps are high-pressure, compact arc lamps that reach 80% of final output within 10 minutes of starting. The arc color is close to daylight (6000 K). The spectrum is continuous in the visible wavelength range and extends both into the UV and near infrared (800 to 1000 nm). Xenon lamps are made with rated wattages from 75 to 30000 watts and are available for operation in either a vertical or horizontal position.

HMI® is the registered trademark of Osram Lighting. The H stands for mercury (Hg), M indicates presence of Metals, and the I refers to the addition of halogen components (iodide, bromide). Originally designed for television lighting, they are now used for location film lighting and as a source for many common entertainment lights.
The modern HMI lamp produces a spectrum similar to daylight with a color temperature of 5600 K.

1.3 Outdoor applications of high intensity lights

High intensity lights in the open air may interfere with normal aircraft operations when their beams hit the eyes of the aircrew. Some typical examples of outdoor applications of high intensity lights are the following:

Searchlights – These lights are frequently used for advertisement or entertainment purposes. They may project one or multiple beams of light. The beams may either be stationary or moving in a regular or random pattern. These lights are very powerful, and are generally operated by professionals in the lighting industry. Searchlights are collimated light sources, producing a column of light whose rays are approximately parallel, with a beam divergence of typically about 3 degrees.

Handheld Spotlights – These lights are readily available to the general public. Commercially available units have an advertised output rating of up to 10,000,000 candle power.

Portable Lighting Systems - These systems are used for a number of activities including night construction, temporary parking lots, entertainment venues, etc. These systems are being utilized with increased frequency and include high powered, large aperture light arrays.

Stadium Lights - These lights are used to illuminate outdoor events. They are normally large arrays, fixed in location and direction.

Architectural Lighting - These lights generally point upwards towards buildings. They are used to wash a building in light and are therefore defocused, which limits their impact on aircraft operations.

Beacons and Lighthouses - These lights are generally used to support aviation and maritime navigation. They usually mark fixed hazards and to help identify airports and landmarks.

2 High intensity lights and pilot vision

The effects of lasers on human vision in general (Gloor et al., 1995; Gloor et al., 1996; Krebs et al., 1994a; Krebs et al., 1994b; Payne et al., 1999; Reddix et al., 1990; Sheehy, 1989; Wütrich et al., 1997; Zheltov et al., 1989) and pilot vision in particular (Barsalou & Reddix, 2000; Elias, 2005; Nakagarawa et al., 2006; Nakagarawa et al., 2007; Nakagarawa et al., 2008c; Nakagarawa et al., 2008b; Nakagarawa et al., 2008a) have been extensively investigated. This enabled the development of guidelines and control measures for the safe use of lasers in the navigable airspace (American National Standards Institute, 2005; ICAO-LFSSG, 2003; SAE, 1999; SAE, 2003; SAE, 2004; SAE, 2005). However, currently no such guidelines exist for other high intensity light sources since their effects on air traffic are simply not known. In the next sections we will briefly review the existing safety guidelines and regulations for outdoor laser operations, and we will then indicate how these can be adapted for other high intensity light sources.

2.1 Lasers

Existing guidelines and regulations for the safe use of lasers in navigable airspace consider the potential visual and biological adverse effects of a proposed laser operation on flight crews in relation to the distance of the airplane from the airport. In the next two sections we will briefly review both the potential visual and biological effects of laser illumination, and their associated laser safety zones around airports.

2.1.1 Visual and biological effects of laser illumination

Vision is the most critical physiological capability in aviation since most of the flight information is visually presented to the pilot. Laser illumination may impair a pilot's vision, and thus compromise his ability to fly an aircraft.

The seriousness of a laser induced physiological effect depends on the intensity of its beam. Permanent eye injuries may occur when laser irradiances exceed the maximum permissible exposure (MPE) level. Below the MPE, laser irradiance may cause the following temporary visual and psychological effects that last from several seconds to several minutes (American National Standards Institute, 2005):

Distraction: an attentional effect resulting from the natural inclination to look toward (pay attention to) a bright light, particularly when it is moving or flickering.

Flashblindness: a temporary visual interference effect that persists after the source of illumination has ceased.

Afterimage: a transient image left in the visual field after an exposure to a bright light source.

Glare: contrast degradation of details in the central visual field due to a bright light source located near the line of sight.

2.1.2 *Laser safety zoning around airports*

Laser operations should be able to not cause permanent eye damage anywhere in the navigable airspace. Laser illumination levels that can not cause permanent eye injury may however induce temporary visual effects like distraction, disorientation, or discomfort, thus creating hazardous situations for pilots performing critical flight operations (e.g. when the aircraft is conducting an approach to or departure from an airport). The seriousness of these effects increases with decreasing distance to the airport. The ICAO (ICAO-LFSSG, 2003) and the FAA (FAA, 2008) identify four zones of protected airspace around airports, and assign specific exposure limits to each of these zones (see Figure 1 and Figure 2). Within these zones, laser emissions exceeding the corresponding irradiance limits are prohibited in order to prevent vision impairments that may interfere with normal flight operations. The four zones are respectively:

Laser Free Zone: The Laser Free Zone serves to minimize any visual interference (startle or distraction) to pilots performing safety critical tasks in the airport environment. The maximum allowable irradiance in the Laser Free Zone is 50 nW/cm^2.

Critical Flight Zone: The critical flight zone serves to minimize transient visual effects due to glare that can interfere with performance of critical flight tasks near the airport. The maximum allowable irradiance in the critical flight zone is 5 $\mu W/cm^2$.

Sensitive Flight Zone: The sensitive flight zone serves to minimize the potential for temporary visual impairment (afterimages or flashblindness) that may persist after the actual laser beam exposure. The maximum irradiance allowed in the sensitive flight zone is 100 $\mu W/cm^2$.

Normal Flight Zone: All navigable airspace not defined by the laser free, critical, or sensitive flight zones. The exposure limit for the normal flight zone is the applicable MPE (e.g. 2.6 mW/cm^2 for visible continuous wave lasers; see e.g. American National Standards Institute, 2007; CIE, 2006). This restriction serves to minimize the potential for lasting eye damage.

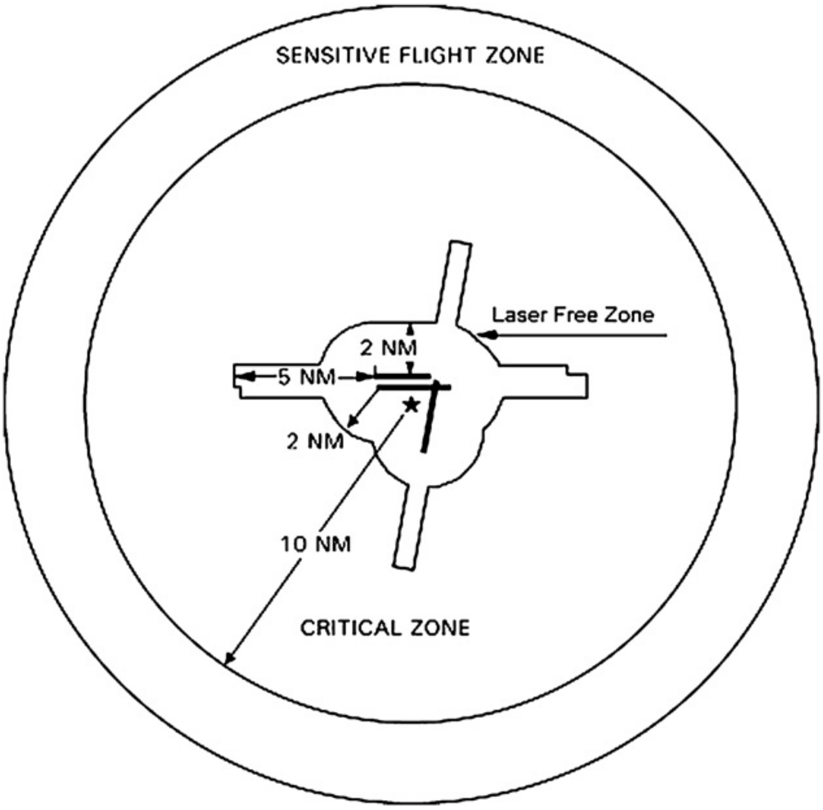

Figure 1 Protected airspace zones around a multiple runway airport. (NM = nautical mile = 1.85 km; from FAA, 2008; ICAO-LFSSG, 2003).

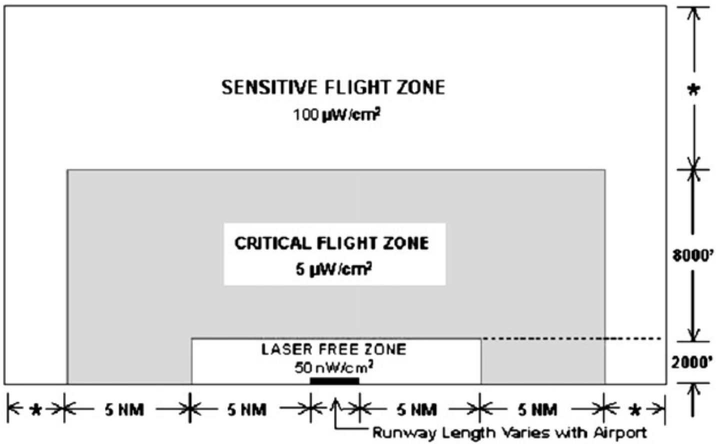

Figure 2 Protected airspace zones and corresponding flight-safe exposure limits around a single runway airport (sources: FAA, 2008; ICAO-LFSSG, 2003).

2.2 Broadband high intensity lights

In the next two sections we will briefly review both the visual and biological effects of high intensity illumination, and assess their implications for safety zoning around airports.

2.2.1 Visual and biological effects of broadband high intensity illumination

The visual and biological effects of high intensity lights are similar to those induced by lasers (see Section 2.1.1).

When viewing directly into the beam of a high intensity light at close range the exposure limits of the eye may be exceeded and eye damage may result. Fortunately, the eye's natural aversion response limits the potential for permanent retinal injury (i.e., blindness). The SAE experimentally determined retinal thermal hazard distances of 100 m and 400 m for high intensity searchlights with an effective source temperature of 5900 K and a diameter of respectively 15 and 60 inches (SAE, 2008). Eye injury or damage is therefore not expected beyond 400 meters from any high intensity light source.

At larger intrabeam viewing distances high intensity lights may cause temporary visual impairments such as glare, flashblindness, and afterimages. Glare only occurs in the presence of the light source. Flashblindness and afterimages may persist for several minutes after viewing the lightsource.

Preservation of optimal night vision is crucial for pilots operating an aircraft at night. When the eyes are adapted to low light levels, exposure to a bright light can result in temporary visual impairment due to glare, flashblindness, and afterimages. There are reports that vision problems resulting from exposure to bright lights at night may have contributed to aviation accidents and incidents (Nakagarawa et al., 2007).
The majority of accidents occurred during the approach and landing phase of flight. lncidents occurred most frequently while taxiing and during approach and landing.

Rotating incandescent beacons (high intensity lights) located at airports or at lighthouses are not known to interfere with visual function of pilots at night.

On April 25, 2007, the FAA conducted actual flight observations of high intensity lights over a test site provided by a SkyView, a major searchlight manufacturer/operator near San Antonio, TX, during twilight and nighttime, visual flight rules (VFR) conditions. During the test an FAA flight check aircraft (Hawker HS125) flew directly toward and over the test site at an altitude of approximately 2000 feet above ground level.
The observers onboard the aircraft included two pilots and two members of the SAE G10T HIL working group. Various types of commercial searchlights pointed at different elevation angles (both rotating and stationary) in the general direction of the approaching aircraft. Observations were also made while various searchlights and a handheld spotlight intentionally tracked the aircraft. A summary of the observations of an FAA aviation vision expert and the aircrew are as follows (SAE, 2008):

- Stationary lights were more difficult to see than rotating lights.
- Lights with smaller reflectors were more difficult to see than those with larger reflectors.
- Lower power lights were more difficult to see than higher power lights.
- Rotating lights had only a short dwell time on the aircraft.
- Intentional tracking of the aircraft was easily accomplished and was disturbing to the pilot.

Note that these observations only reflect a few reported sightings in a single type of aircraft. Also, due to the nature of the aircraft and its flight path restrictions it was only possible to perform only a few observations.

2.2.2 *Proposed safety zoning for broadband high intensity lights around airports*

In contrast to the effects of lasers, the effects of high intensity lights on pilot vision in low ambient light conditions have not been sufficiently investigated. As a result there are currently no well-defined threshold illuminance levels corresponding to the different visual interference effects that may occur when a pilot is confronted with a high intensity light while performing his operational duties. The SAE therefore proposed to adopt the existing laser safety zoning around airports also for other high intensity light sources and to convert the corresponding maximum admissible irradiance exposure levels to values that apply to broadband high intensity lights (SAE, 2008).

The ANSI Z136.6 standard defines laser irradiance (W/cm^2) threshold exposure levels for visual interference at dusk or at night (American National Standards Institute, 2005, Table 5). When applied to broadband high intensity lights, the appropriate corresponding measure is illuminance (lm/cm^2). The laser irradiance threshold levels corresponding to the different visual interference effects can be converted into illuminance threshold levels through multiplication by the luminous efficacy at the peak photopic response, which is 683 lm/W at 555 nm (Arecchi et al., 2007; see Figure 3 and Table 1, adopted from SAE, 2008). Note that this conversion is based on the assumption that photopic vision applies. The SAE argues that this assumption is reasonable because the aviator's eyes are generally not entirely dark adapted at night due to ambient lighting, such as illuminated cockpit gauges and ground lights (SAE, 2008). Further studies may be required to assess the validity of this assumption.

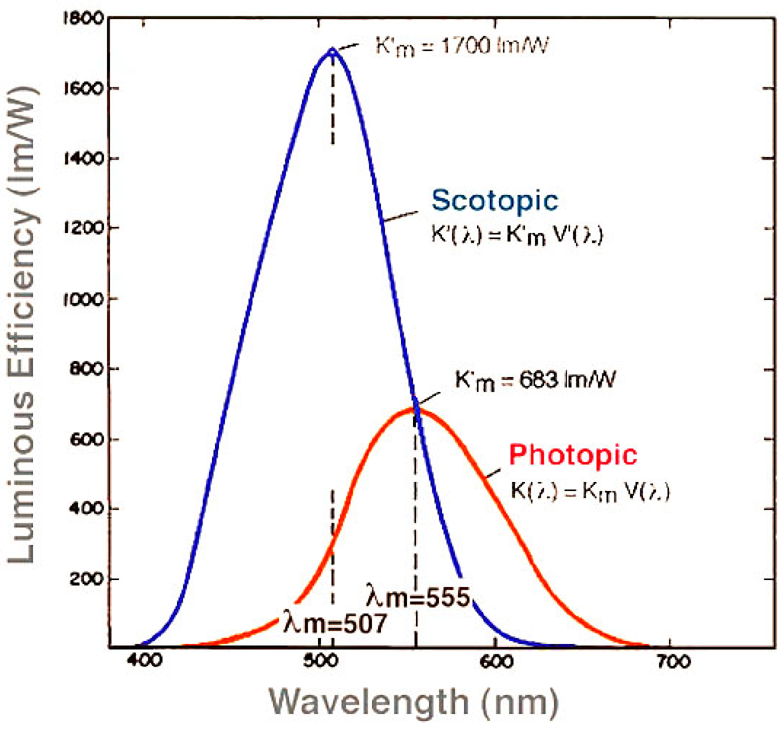

Figure 3 The scotopic and photopic curves of spectral luminous efficacy.

Table 1 Broadband illuminance threshold exposure levels corresponding to safety zones around airports, derived from laser irradiance limits as defined by ANSI Z136.6 (American National Standards Institute, 2005, Table 5) .

Flight Zone	Irradiance Limit (W/cm^2)	Illuminance Limit (lm/cm^2)
Normal	2.5×10^{-3}	1.7
Sensitive	100×10^{-6}	6.8×10^{-2}
Critical	5×10^{-6}	3.4×10^{-3}
Laser Free	50×10^{-9}	3.4×10^{-5}

3 Experimental validation studies and observations

The experiments described in this section were performed to validate the illuminance threshold exposure levels corresponding to safety zones around airports as proposed by the SAE (see Table 1).

During two consecutive nights (the nights of February 10 and 11, 2009) we performed photometric and observation experiments at the site of a major searchlight manufacturer and operator (SkyView, see www.sky-view.com) in San Antonio, Texas. On the first night we used several typical high intensity searchlights and we measured the illuminance they yielded on a surface oriented orthogonal to the beam and along the axis of maximum intensity, as a function of distance along the ground. On the second night we observed these lights from a helicopter while flying over the test site.

After performing the experiments in San Antonio, we also observed the high intensity light beam of the Luxor hotel while circling around the beam in a helicopter.

3.1 The high intensity lights

For our experiments we selected a set of 6 lights that are representative for the main technologies currently applied in high intensity lighting. For comparison we also tested a green laser pointer and a handheld flashlight. The lights we tested were the following:

Arclight: AD Light HID, with a 850 W lamp and a 16” reflector.

Dominator: Osram HMI, 12,000 W with a 60” reflector.

Skytracker: 4 W Xenon lamp with 4 separate armatures and 16” reflectors; only 1 lamp was measured.

Prolight: 1,200 W HMI with a 16” reflector, 120V.

2k Xenon: 2,000 W Xenon with a 16” reflector, 19.8 A.

Carbon Arclight: Carbon plasma light with a real flame as light source and a 60” reflector.

Green laser pointer: A 3.5 mW 532 nm green Class IIIA laser pointer (RadioShack; see www.RadioShack.com).

Flashlight: A high power handheld flashlight with an 8.5” reflector.

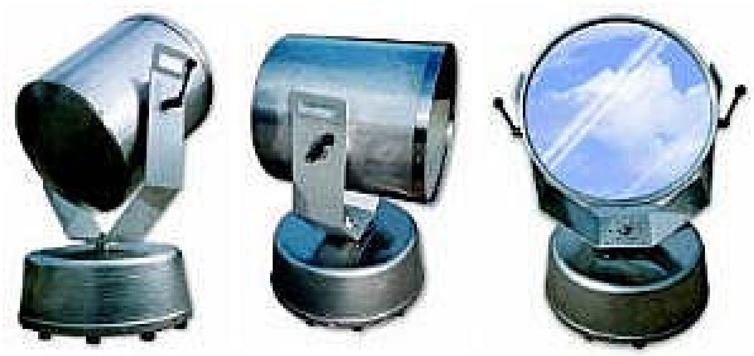

Figure 4 The Arclight: AD Light HID with an 850 Watt lamp.

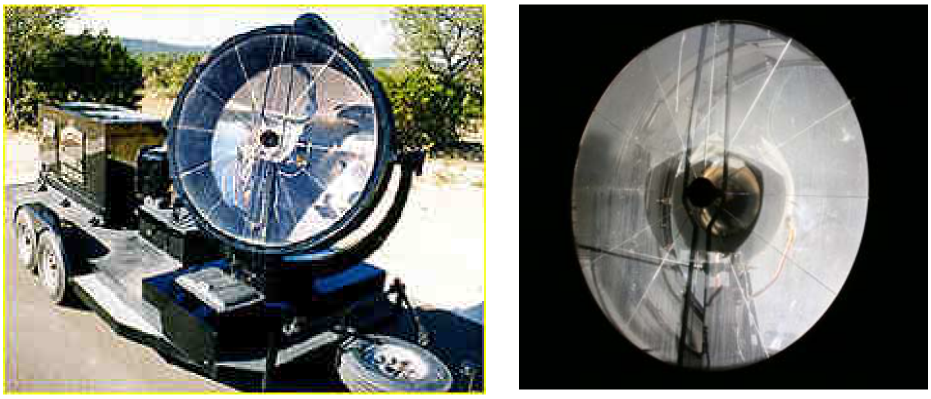

Figure 5 The Dominator with an 18k HMI lamp.

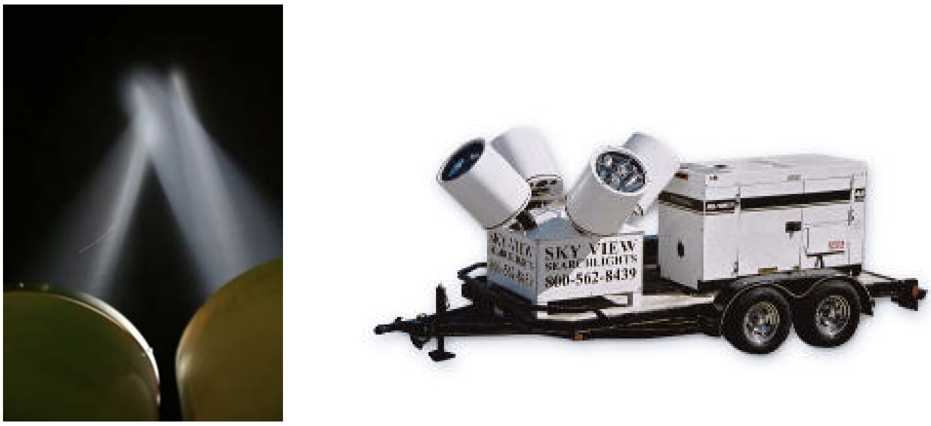

Figure 6 The Skytracker Xenon HID with a 4k Xenon lamp.

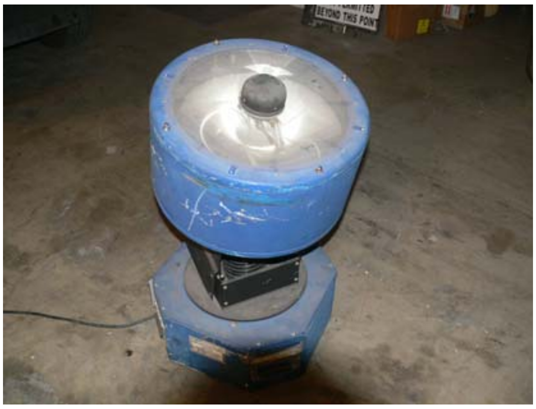

Figure 7 The Prolight HMI with a 1200 Watt lamp.

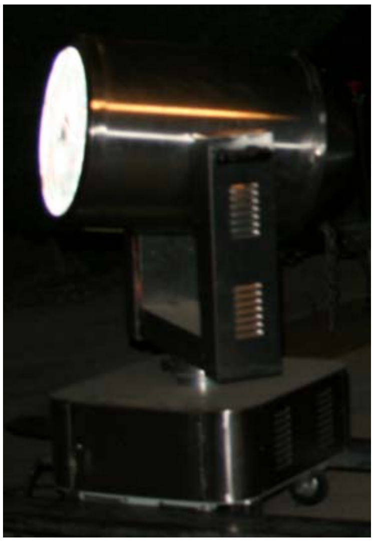

Figure 8 The 2k Xenon light.

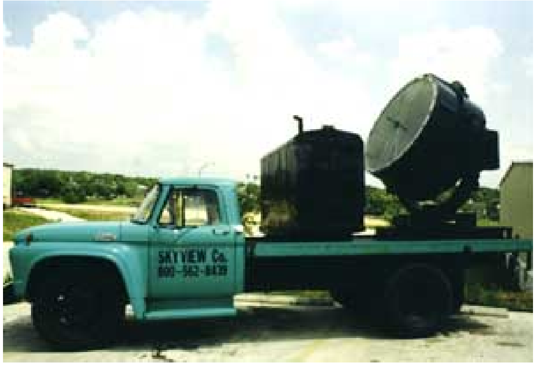

Figure 9 The Carbon Arclight.

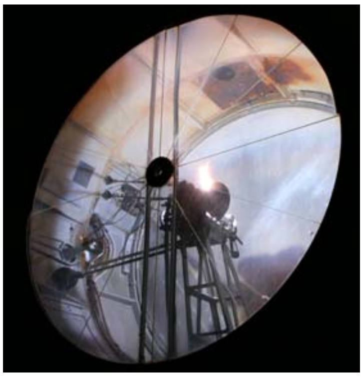

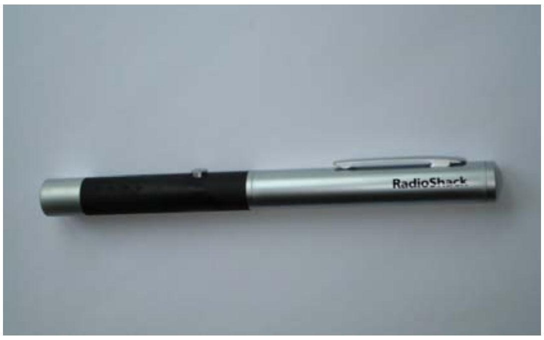

Figure 10 The green laser pointer (Radio Shack, 532 nm).

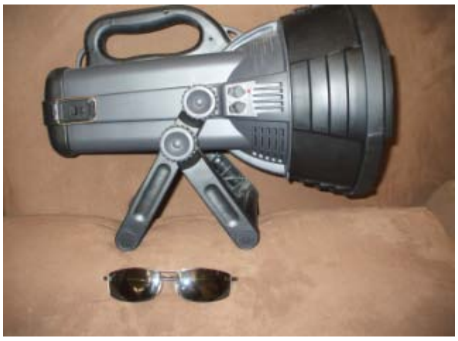

Figure 11 The handheld Flash light with an 8.5’’ reflector.

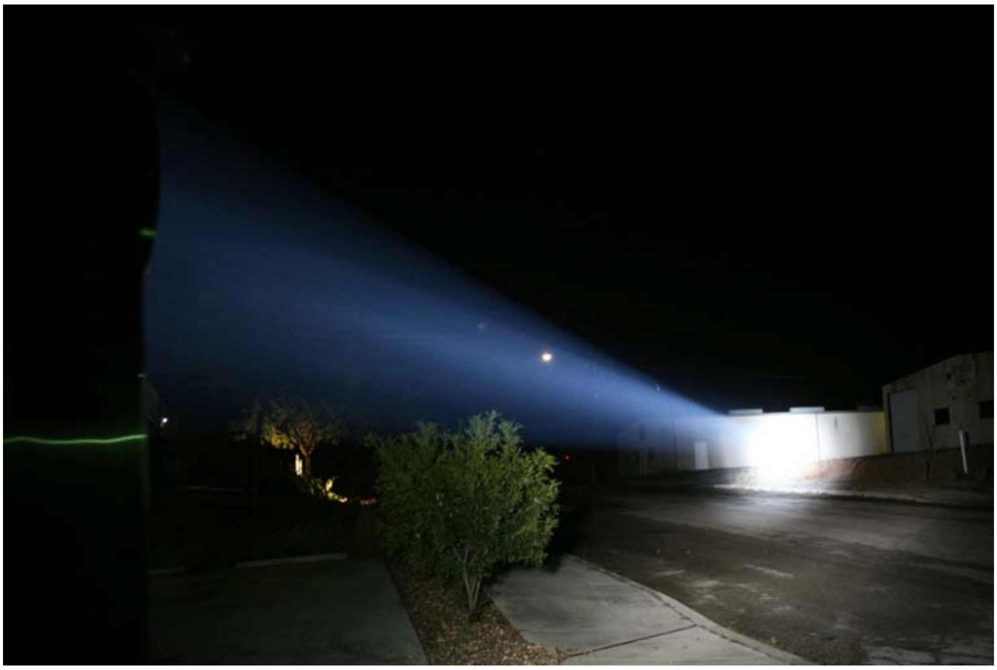

Figure 12 Searchlight projected onto a wall at a distance of 100 meters.

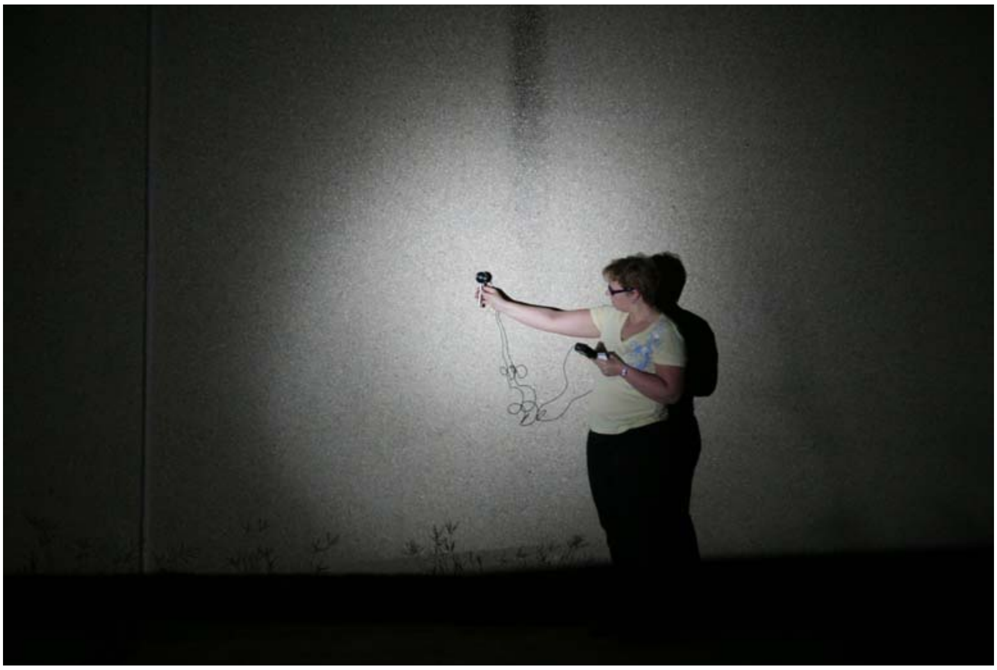

Figure 13 Illuminance measurement of a searchlight.

3.2 Ground measurements

During the night of February 10, 2009 we measured the illuminance, the luminance and the beam width of the 6 high intensity lights listed in Section 3.1. The sky was partly overcast during the measurements. The temperature was around 18 degrees Celsius. There was some moonlight, but that did not affect the measurements. The searchlights were placed such that their beams projected along the horizontal and onto a white wall at a distance of 100 meters (see Figure 12).

Illuminance measurements were performed with a calibrated illuminance meter from Lichtmesstechnik (LMT), Berlin, Germany. The wall illuminance caused by the searchlights was measured at four different distances from the lightsource: at 25, 50, 75 and 100 meters. lluminances at larger distances from the lightsource can be extrapolated using the inverse square law:

$$E = \frac{I}{d^2} \qquad [1]$$

where:

E = illuminance (lx)
I = luminous intensity (cd)
d = distance from lightsource (m)

The luminance distribution of the projection of the searchlights onto the white wall was registered by a Canon EOS 400D photo camera, from a distance of 20 m perpendicular to the wall. The photographs taken with the Canon camera were then transformed into luminance images by a Matlab computer program. The resulting luminance images provide a reliable representation of luminance distribution of the scene. The Canon photo camera pictures were calibrated for luminance and color using a calibrated Photo Research Spectroradiometer 650.

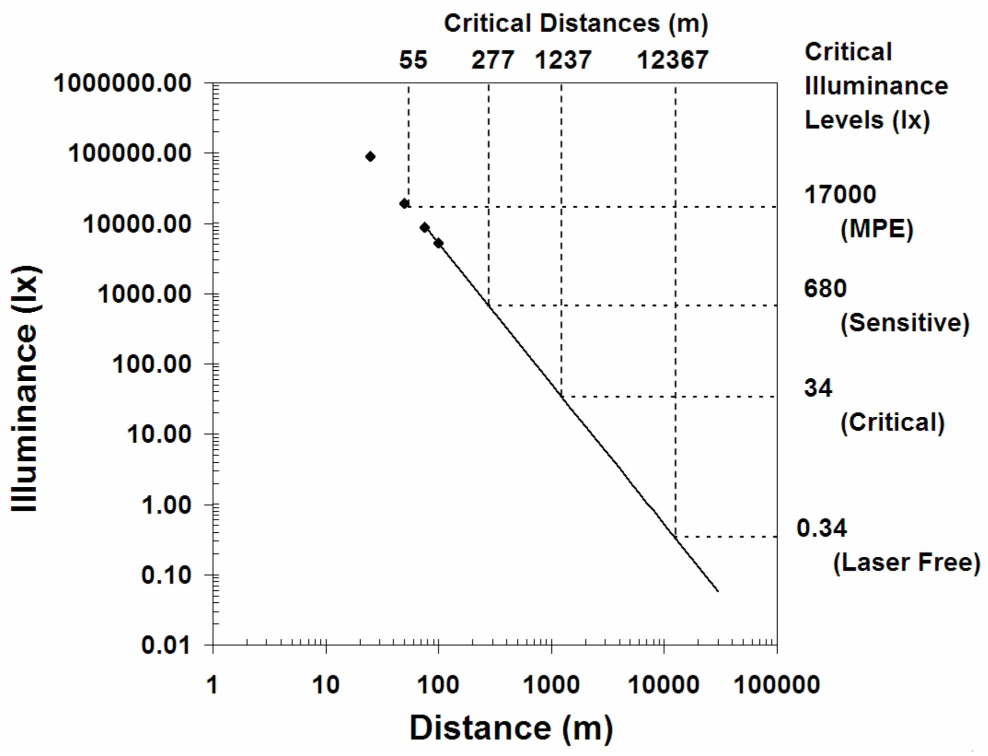

Figure 14 The illuminance of the Arclight at different distances.

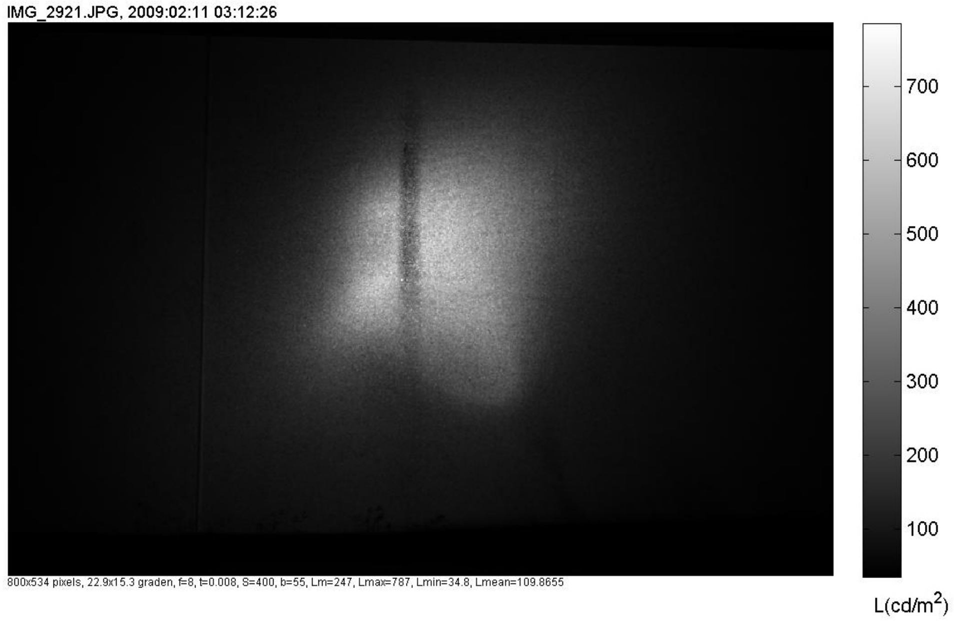

Figure 15 The luminance at the wall when light from the handheld Arclight is projected from a distance of 100 meter.

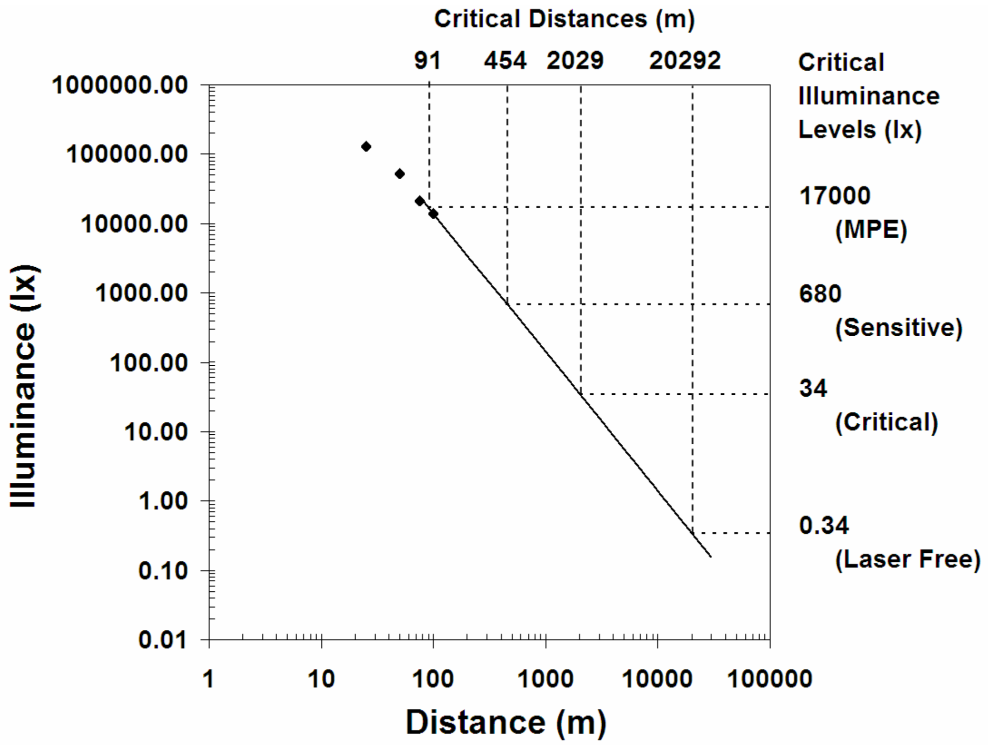

Figure 16 The illuminance of the Dominator as a function of distance.

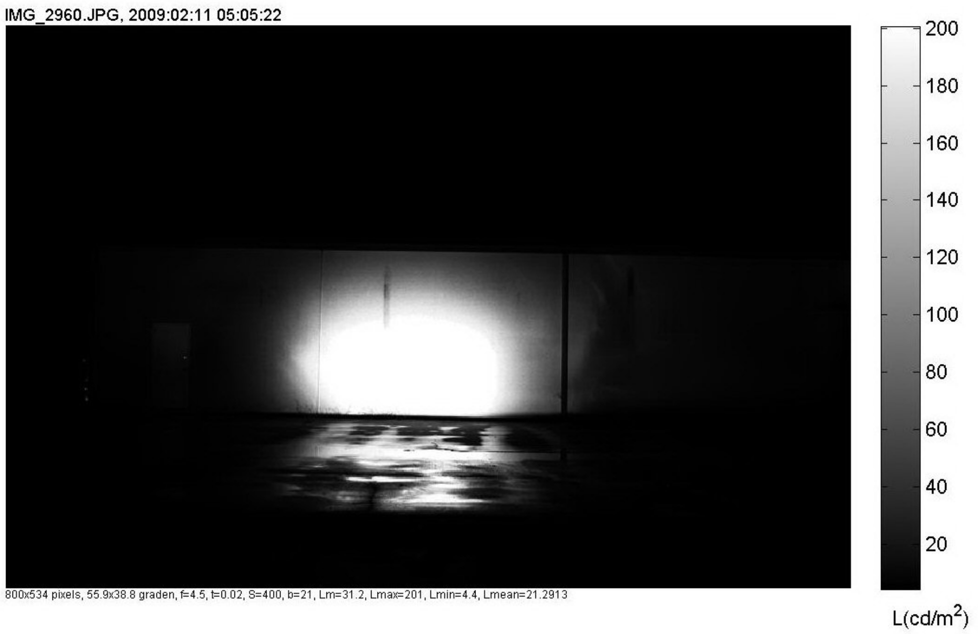

Figure 17 The luminance distribution of the Dominator projected onto a wall at a distance of 100 meter.

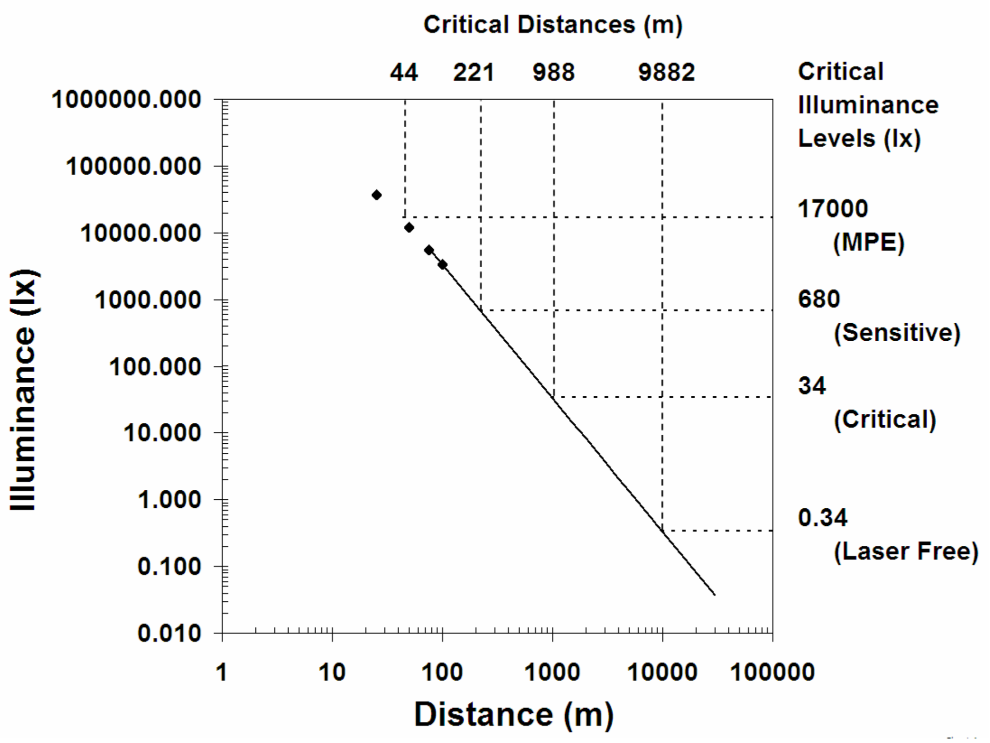

Figure 18 The illuminance of the Skytracker as a function of distance.

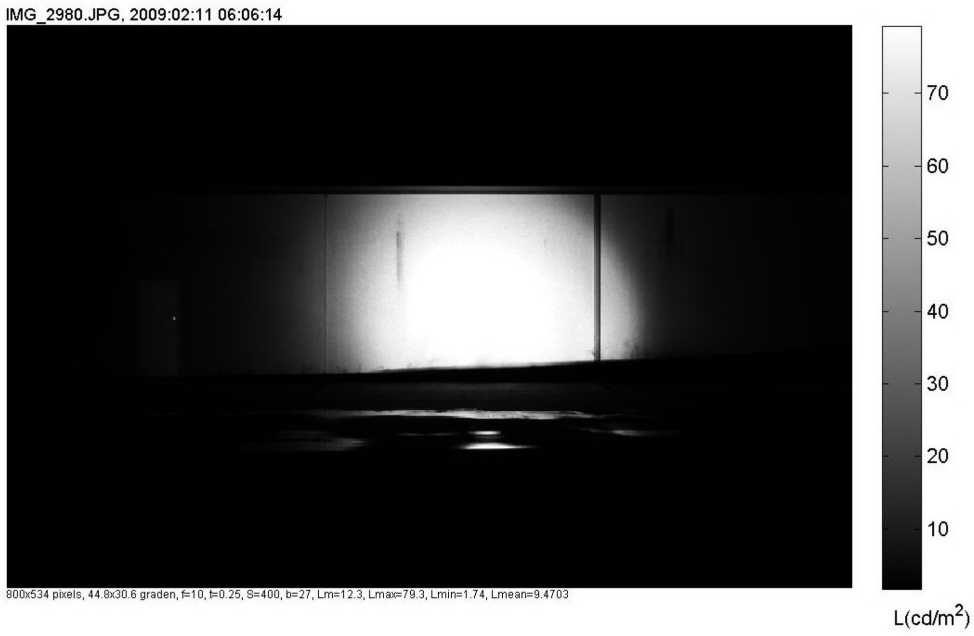

Figure 19 The luminance of the Skytracker projected onto a wall at a distance of 100 meter.

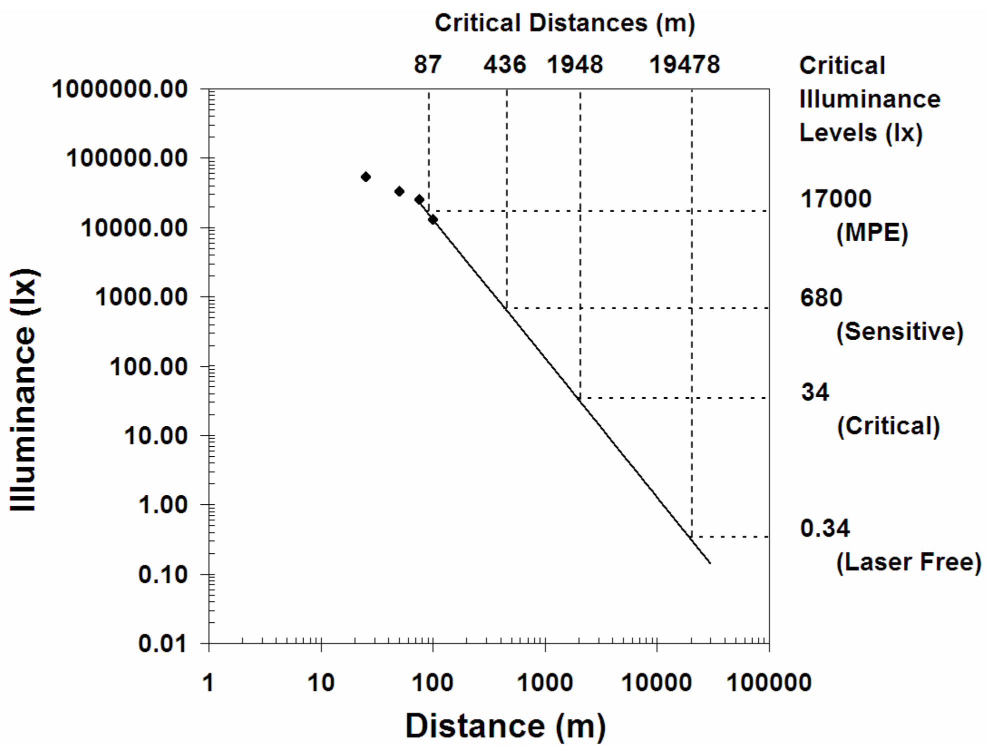

Figure 20 The illuminance of the Carbon Arclight at different distances.

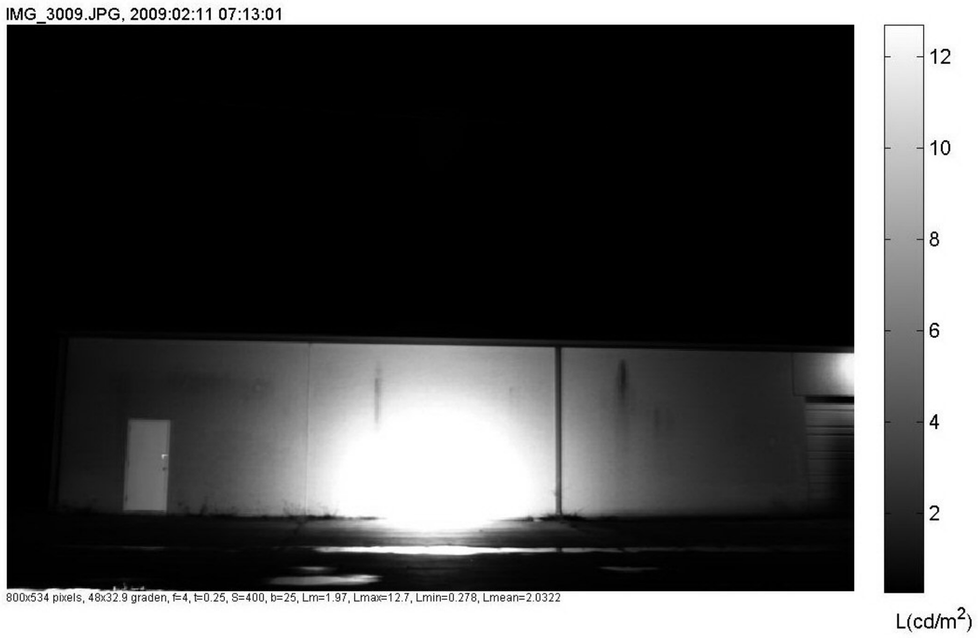

Figure 21 The luminance of the Carbon Arclight projected onto a wall at a distance of 100 meter.

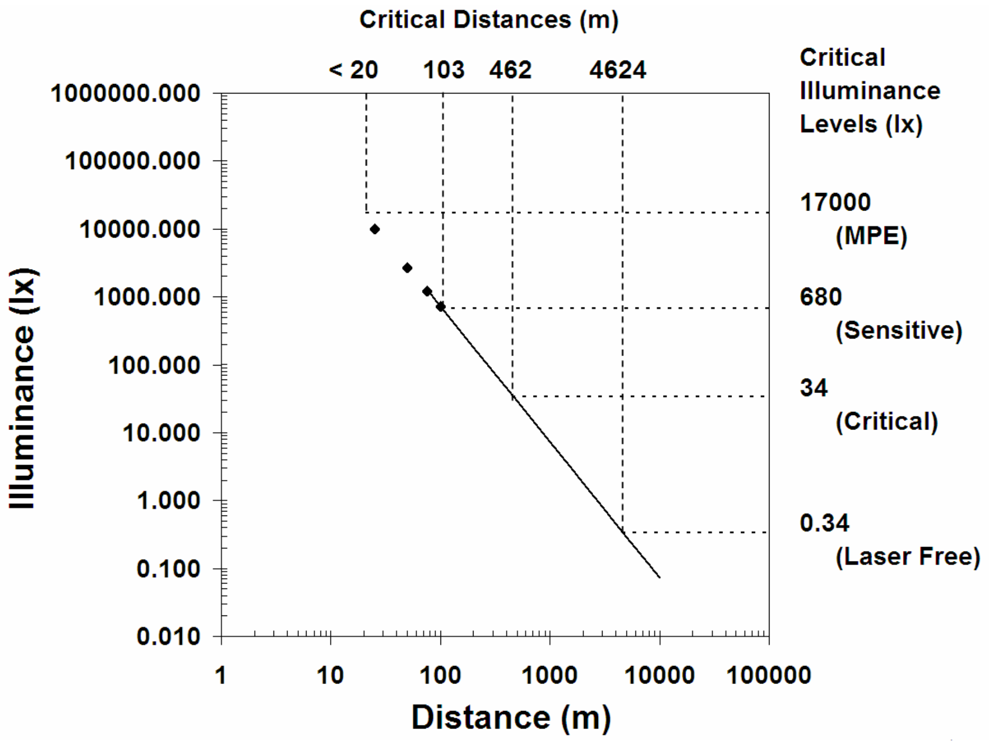

Figure 22 The illuminance of the Prolight at different distances.

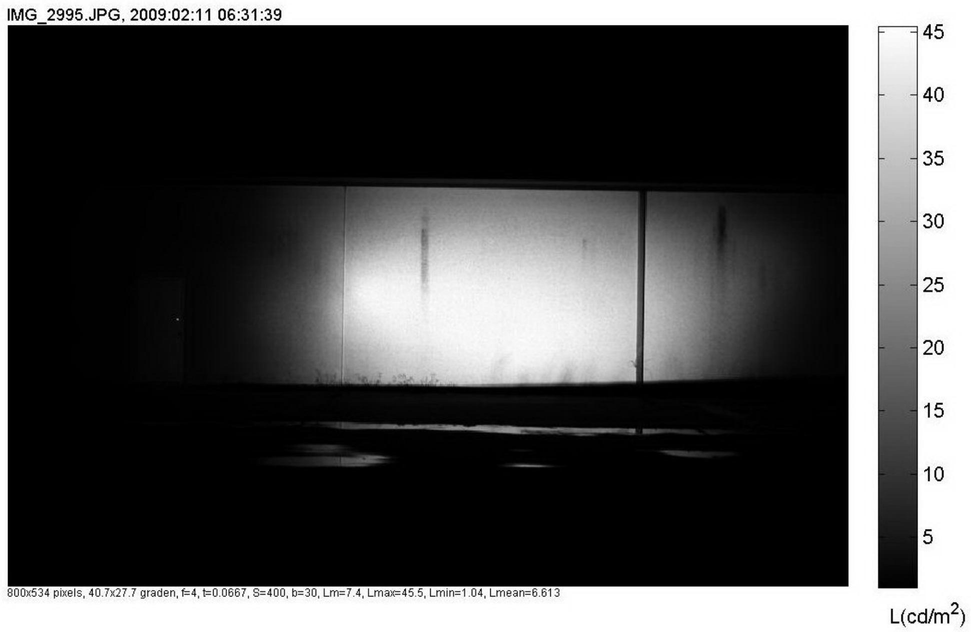

Figure 23 The luminance of the Prolight projected onto a wall at a distance of 100 meter.

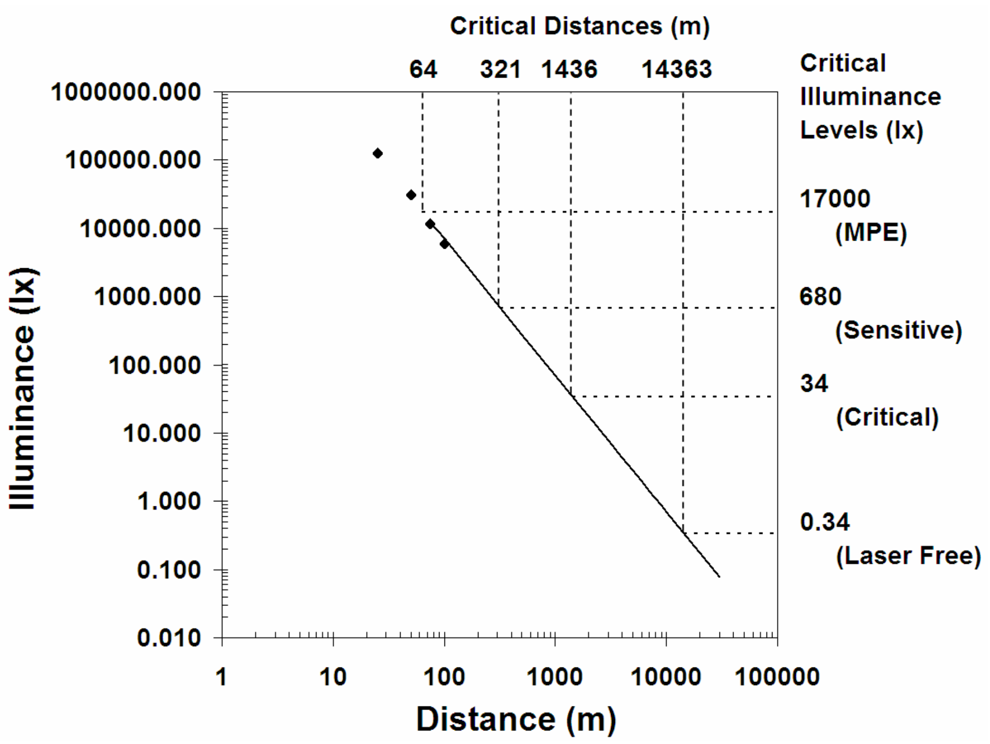

Figure 24 The illuminance of the 2k Xenon Light at different distances.

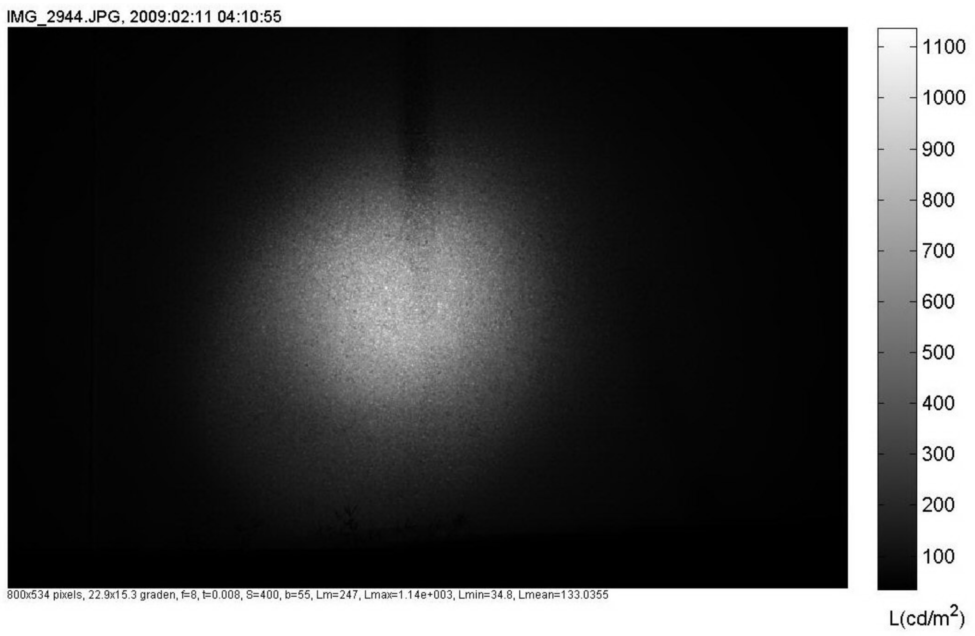

Figure 25 The luminance of the 2k Xenon Light projected onto a wall at a distance of 100 meter.

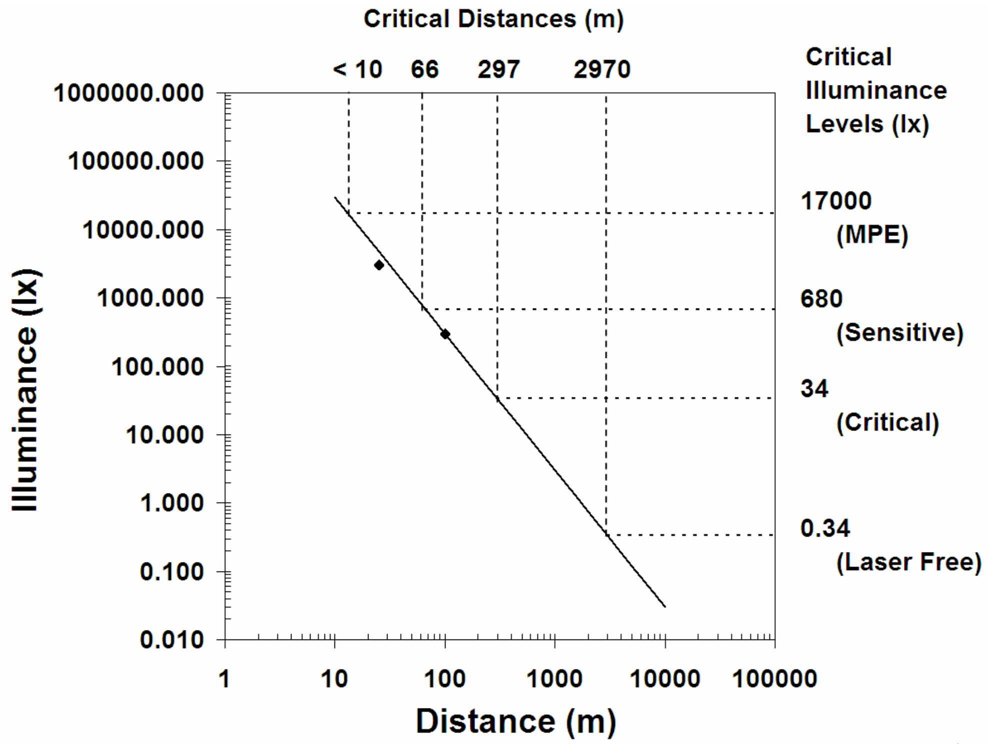

Figure 26 The illuminance of the green laser pointer at different distances.

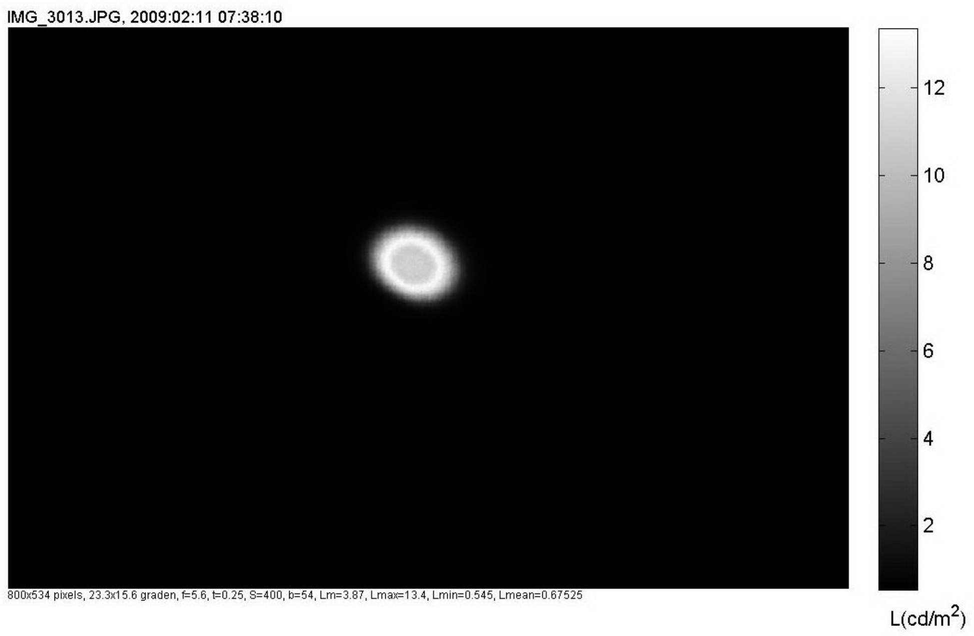

Figure 27 The luminance of the green laser pointer projected onto a wall at a distance of 100 meter.

The luminous intensity and the luminance of the searchlights can be calculated from the measured illuminance. The luminous intensity can be calculated by formula (1).
The luminance can be calculated by:

$$L = \frac{I}{A}; \qquad A = 0{,}25 \cdot \pi \cdot d^2 \qquad (2)$$

where:

L = luminance (cd/m²)
I = luminous intensity (cd)
A = surface (m²)
π = 3.14
d = beam diameter (m)

Table 2 The diameter, illuminance, luminous intensity, luminance and beam width of the searchlights and the laser pointer. The corresponding values for the sun are provided as a reference.

Searchlight	Diameter Searchlight (m)	Illuminance @ 100 meter (lx)	Luminous Intensity (cd)	Luminance (cd/m²)	Beam width (deg)
Arclight	1.524	5200	5.20×10^{7}	2.87×10^{7}	0.93
Dominator	1.524	14000	1.40×10^{8}	7.72×10^{7}	2.93
Prolight	0.406	727	7.27×10^{6}	5.60×10^{7}	1.68
Xenon	0.406	7013	7.01×10^{7}	5.41×10^{8}	1.72
Skytracker	0.406	3320	3.32×10^{7}	2.56×10^{8}	2.67
Carbon arc	1.524	12900	1.29×10^{8}	4.11×10^{9}	2.85
Laser pointer	0.003	300	3.00×10^{6}	2.39×10^{11}	0.05
		**Illuminance (lx)**			
Sun		150000	3.33×10^{27}	2.19×10^{9}	

3.3 Flight measurements and oberservations

On the night of February 11, 2009, we observed the beams of the high intensity lights listed in Section 3.1 (i.e. the 6 searchlights, the handheld flashlight and the green laser pointer) while flying towards them in a Robertson R44 Astro helicopter (see Figure 28).

During these trials the sky was clear (only some occasional clouds at 25.000 ft) and visibility was optimal. The temperature was around 19º C, with a dewpoint at -2º C. Air pressure was 1017.9 mbar. The helicopter pilot was highly experienced. During the whole experiment the helicopter maintained a cruising altitude between 600 and 700 feet above ground level (in meters between 180 and 210 m). On each trial the helicopter repeatedly approached or circled around the test site (the location of SkyView company). The maximal approach distance and the radius of the circular flight paths varied from about 500 m to about 200 m. Communication with the ground site was established by means of a cell phone with a bluetooth connection to a headphone that was plugged into the helicopter's communication system.
The experiments were performed between 20.00 and 21.00 hrs local time.

Before take-off the pilot was instructed that safety had the main priority at all times, and that he should avoid the searchlights whenever he thought that there was even a remote chance that the situation might become dangerous in any respect. It turned out that this

never occurred during the experiments. In fact, the pilot never experienced any adverse effects of the light beams, and reported that he had no problems reading his instruments or viewing outside.

The 6 searchlights were placed in a fixed position such that their beams projected into the sky under an angle of 45 degrees with the horizontal (ground plane). Each light was tested individually during several runs. On each test run the helicopter approached the beam of the high intensity light source in a straight line (i.e. the linear approach path and the beam were both in the same vertical plane) before actually crossing it.
This procedure was repeated several times for each light source. During several test runs the green laser pointer was also aimed at the canopy of the helicopter and attempts were made to track the helicopter with the laser beam. Finally, at the end of the experiment, the helicopter was repeatedly tracked by the Dominator. The order in which the high intensity lights were observed was as follows:
1. Dominator
2. Skytracker
3. Arclight
4. Prolight
5. 2K Xenon
6. Carbon arc
7. Flashlight
8. Green Laser pointer

Movies showing the visual effects of each of these high intensity light sources are included on the CD-ROM accompanying this report.

We used a Garmin GPSmap 76 CSX GPS tracker to calculate the distance from the helicopter to the test site. A GISTEQ PhotoTrackr was used to plot the helicopter flight path onto Google maps.

During the flight we made both objective (luminance photographs and incidental illuminance measurements) and subjective (visual impressions) measurements. We also repeatedly asked the pilot for his opinion about the brightness of the searchlights and their impact on the visibility of both the outside world and his instruments. The pilot experienced no problems at any time and could easily perceive his instruments and the outside world, even while he was continuously tracked by the Dominator, which lighted up the whole cockpit and shone straight into his eyes (see Figure).
The experimenters verified that the pilot showed no signs of irritation, and did not frown or look away or closed his eyelids during the impacts. The pilot confirmed that in practice pilots frequently use searchlights with known fixed positions as navigation beacons.

Table 3 Computed visual interference threshold distances below which the high intensity lights may induce the corresponding visual effects (from Figure 14-Figure 22). Cells that are shaded in gray represent visual effects that were expected to occur during the present trials.

Exposure Label	Visual Effects	Dominator	Sky tracker	Arclight	Prolight	2k Xenon Light	Carbon Arclight	Laser pointer
**MPE**	eye damage	91 m	44 m	55 m	< 20 m	64 m	87 m	< 10 m
**Sensitive**	afterimages	454 m	221 m	277 m	103 m	321 m	436 m	66 m
**Critical**	glare	2 km	1 km	1.2 km	0.5 km	1.4 km	2 km	0.3 km
**Laser Free**	distraction	20 km	10 km	12 km	5 km	14 km	20 km	3 km

For each of the lights tested we computed the visual interference threshold distances. Below each of these distances the lights may induce the visual effects corresponding to each of the protected airspace zones (from Figure 14-Figure 22). The results are listed in Table 3. The shaded cells correspond to visual effects that were expected to occur during our present trials.
The helicopter flight tests reported here were performed to validate the computed visual interference threshold distances listed in Table 3. Figure 31 and Figure show respectively the visual impact and the corresponding luminance distribution of the six searchlights, the flashlight and the green laser pointer, as seen while approaching their beams in a helicopter flying at an altitude between 600 and 700 feet above ground level.

Except for the handheld flashlight, all high intensity lights tested induced distraction effects below 500 m when their beam pointed directly at the helicopter canopy.
No distraction effects were observed when the beam was not directly hitting the canopy. This is probably because there were many other comparable light present in the background (street lighting, industrial lighting, and car headlights). Car headlights were experienced as even more distracting since these moved around (moving light sources are strong distractors). The lights tested here may of course cause distraction in sparsely lit (e.g. rural) environments; i.e. in conditions where the high intensity light is the only bright light in the background.

Except for the handheld flashlight, all high intensity lights tested also induced some glare below 500 m when their beam pointed directly at the helicopter canopy.
This amount of glare was minimal though and had no effect on the ability of the pilot and the experimenters to view the instruments in the cockpit and the outside world immediately surrounding the light source. However, the green laser pointer induced a considerable amount of glare when it hit the eyes of the observers. When hit by the laser pointer it was impossible for the observers to perceive details in the outside world. Both the pilot and the experimenters found the impact of the laser pointer unacceptable. All observers felt the need to close their eyes to avoid looking into the beam. None of the other tested high intensity light sources produced any glare effects remotely comparable to that of the green laser pointer.

Except for the laser pointer, none of the other lights tested in this study induced any temporary visual impairments like flashblindness or afterimages. Based on the calculations listed in Table 3 we had expected to observe aftereffects from most of the lights except the Prolight and the laser pointer. However, it turned out that none of the lights was strong enough to induce aftereffects within our flight range between 200 and 500 m. Thus, the threshold values for the high intensity lights used in the computation of the sensitive zones listed in Table 3 appear to be too conservative. This may be a result of the fact that the observers were only partly dark adapted during this experiment (because of the city and car lights surrounding the test site), resulting in mesopic vision with increased visual interference thresholds. The thresholds used by the ICAO (ICAO-LFSSG, 2003) and the FAA (FAA, 2008) in their definition of the interference zones are lower since they are based on fully dark adapted eye (scotopic vision).

According to our calculations the 3.5 mW green laser pointer should be:

- a potential eye hazard at distances below 10 m (about 30 feet).
- a temporary flashblindness hazard to about 70 m (about 200 feet).
- a glare hazard to about 300 m (about 1000 ft), and
- a distraction hazard to over two miles

Figure illustrates the hazard distances for a 5 mW green laser pointer (source: www.laser pointersafety.com). The inset photographs with yellow borders were taken in an FAA flight simulator. They illustrate what a pilot may see on landing approach, during a 5 mW laser illumination. The inset photograph with the red border was taken during the experiments reported here, and shows the helicopter canopy while being hit by the beam of our 3.5 mW green laser pointer at a distance of about 1,600 ft.

The fact that the laser pointer had a much larger visual impact than predicted by our calculations (it did cause brief afterimages even at 500 m) may be due to its small beamwidth (it appeared as a much smaller and more intense spot to the observers: see also Table 4) and its monochromatic color (green) which was significantly different from its local background. In daylight (photopic vision) the eye is most sensitive to greenish-yellow light at 555 nm. When the eye adapts to darkness (scotopic vision) the eye's peak sensitivity shifts to bluish-green light at 507 nm (see Figure 3). During our experiment the pilot may have been partly dark adapted, resulting in mesopic vision (a combination of photopic vision and scotopic vision in low but not quite dark lighting situations). This may have resulted in a brighter appearance of the green light produced by the laser pointer (532 nm). In addition to its high intensity, the laser pointer caused an additional startle effect because it could not be seen before it actually hit the eye. The other bright light sources that were used in this study could be seen at all times, mostly as point sources, that gradually increase in size and intensity when their beams approached the eye of the observers. As a result, a beam crossing of the broad spectrum high intensity light was mostly anticipated well in advance. In contrast, an impact of a laser beam was generally an unexpected event, thus causing additional startle.

While approaching a searchlight it mostly appeared similar or even less in brightness to other city lights or car lights, and was therefore hard to distinguish from these. As a result, the high intensity searchlights tested in this study will not induce any adverse visual effects when they are not pointed directly at an airplane. During the experiments we noticed that the lights of cars driving uphill and in the direction of the helicopter appeared quite similar in intensity to the searchlights when these were directed at the helicopter.

In practice it proved difficult to fly directly into the beam of a high intensity light because the width of the beams is very small. The likelihood that a directed high intensity light beam will hit an aircraft is therefore very small. Moreover, when a hit actually occurs it will last only for a very brief moment (due to the high speed of the aircraft and the small diameter of the beam). Thus, a pilot whose canopy is hit by a beam the light will only notice a brief flash of bright light. We found that the night vision of the pilot was not affected in any way when flying through the beams of the high intensity lights tested in this study. Only the laser pointer considerable impaired his momentary visual abilities and even caused short lasting afterimages.

Table 4 Typical size and angular extent of the high intensity lights used in this study, seen at a distance of 500 m.

Lights	Diameter lamp (m)	Beamwidth (arcmin)
Small	0.4	2.7
Large	1.5	10.3
Laser	0.003	0.02

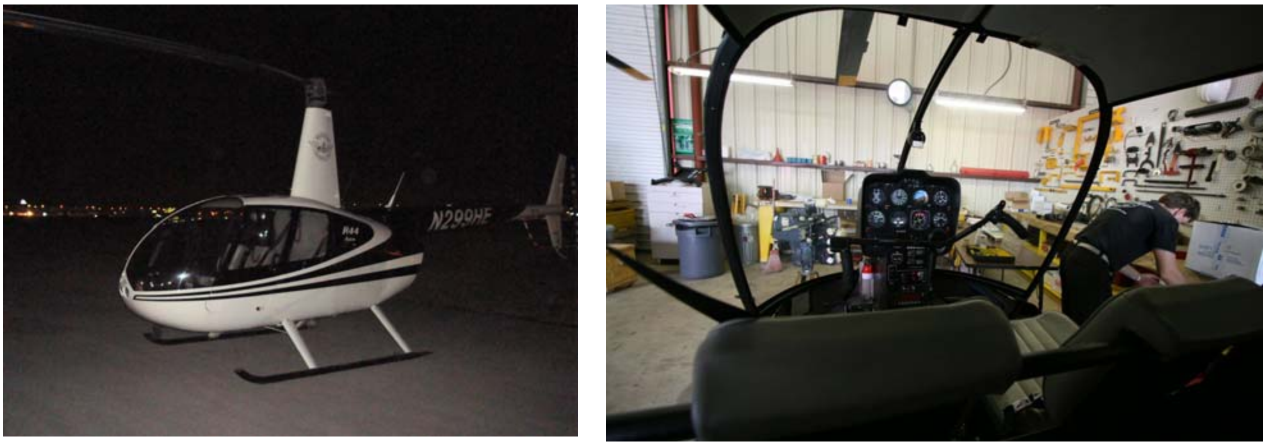

Figure 28 The Robertson R44 Astro helicopter from the outside (left) and inside (right).

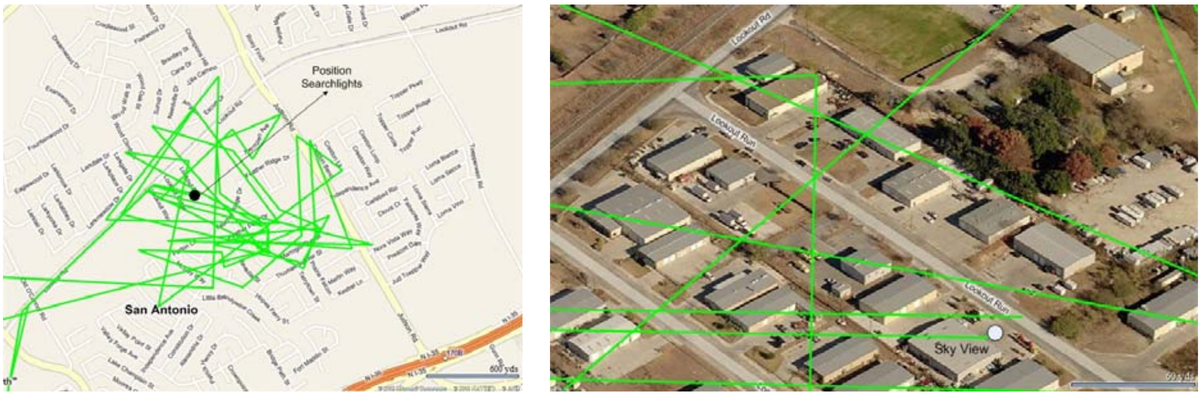

Figure 29 Flight path of the helicopter projected onto Google maps.

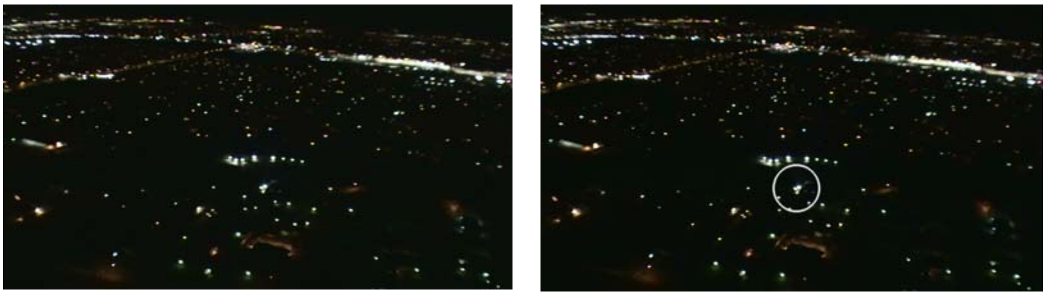

Figure 30 Searchlight surrounded by city lights, in the second image the searchlight is circled.

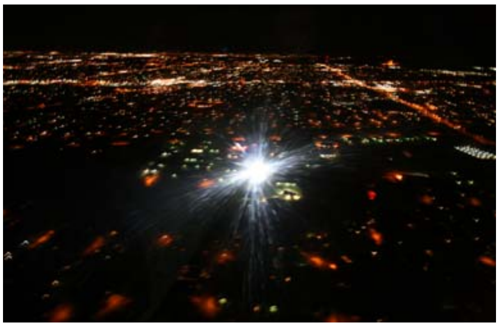

Dominator

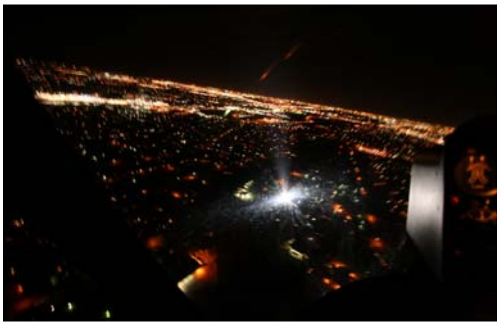

Skytracker

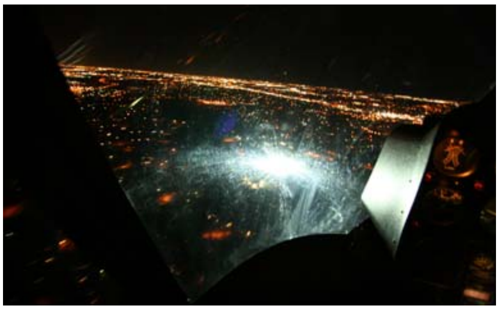

Arclight

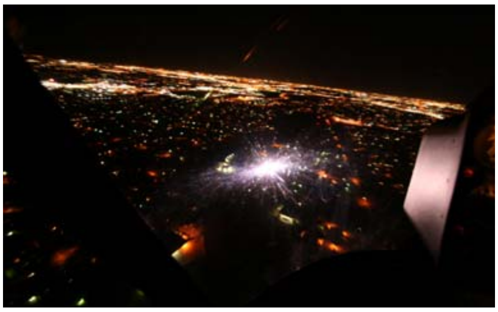

Prolight

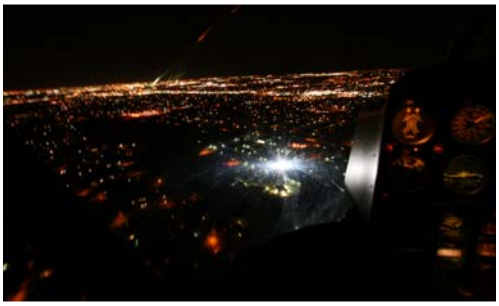

2k Xenon

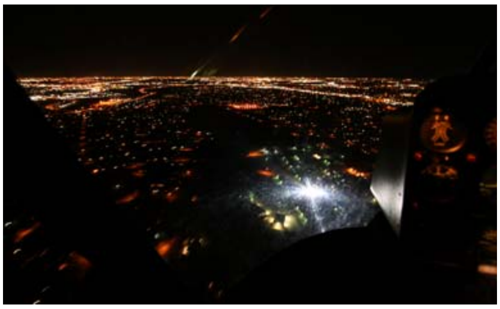

Carbon arc

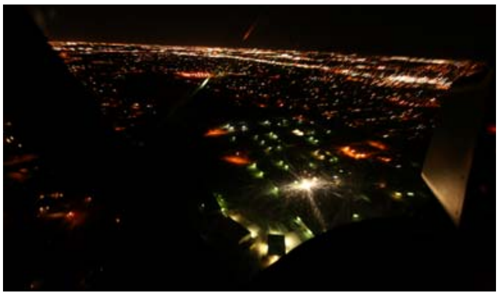

Flashlight

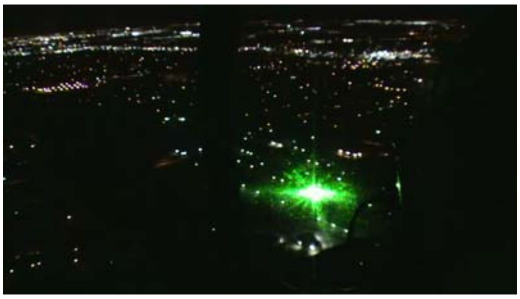

Green laser pointer

Figure 31 The six searchlights, the flashlight and the green laser pointer as seen while approaching their beams in a helicopter flying at an altitude between 600 and 700 feet above ground level.

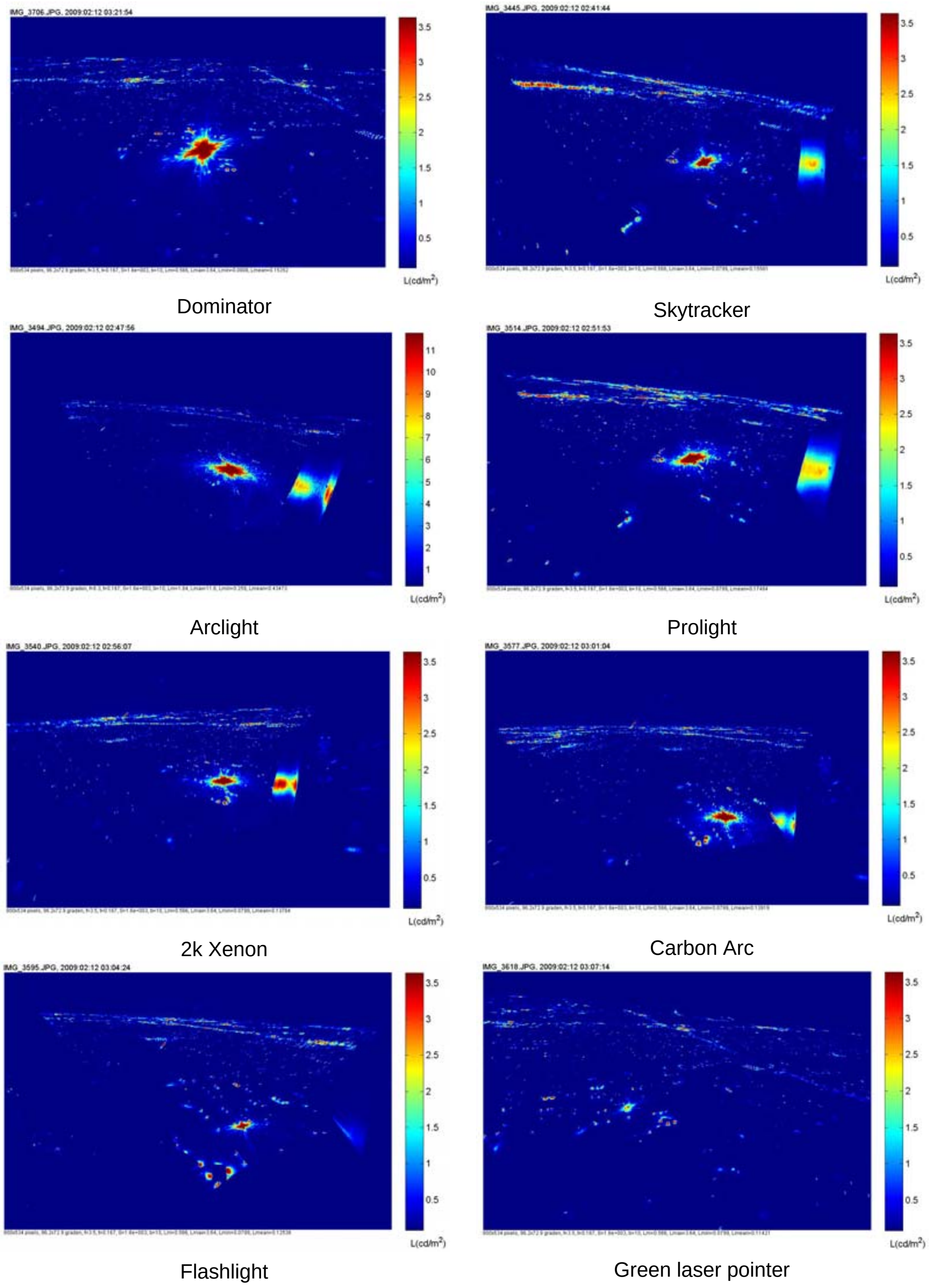

Figure 32 The luminance distribution of the six searchlights, the flashlight and the green laser pointer (as seen in Figure) registered while approaching their beams in a helicopter flying at an altitude between 600 and 700 feet above ground level.

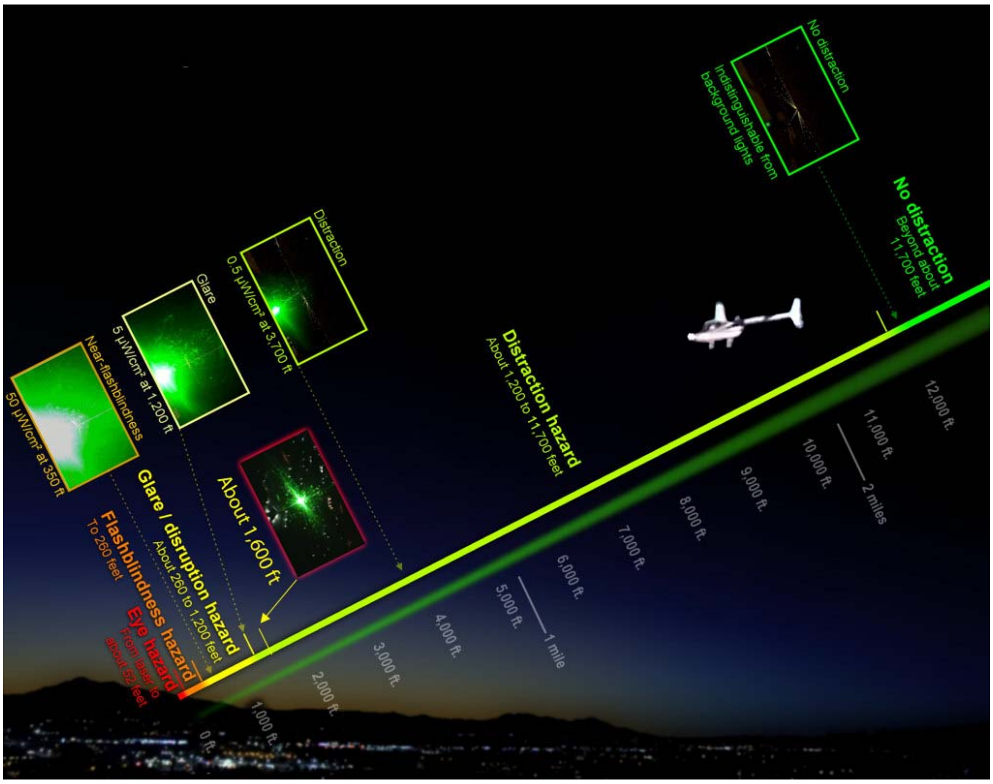

Figure 33 Safety distances for a 5 mW green (532 nm) laser pointer (adapted from : www.laser pointersafety.com). The insets with yellow borders are photographs taken in an FAA simulator. The inset with the red border is a photograph taken during our experiments at a distance of about 1,600 ft from the 3.5 mW green laser pointer (see also Figure).

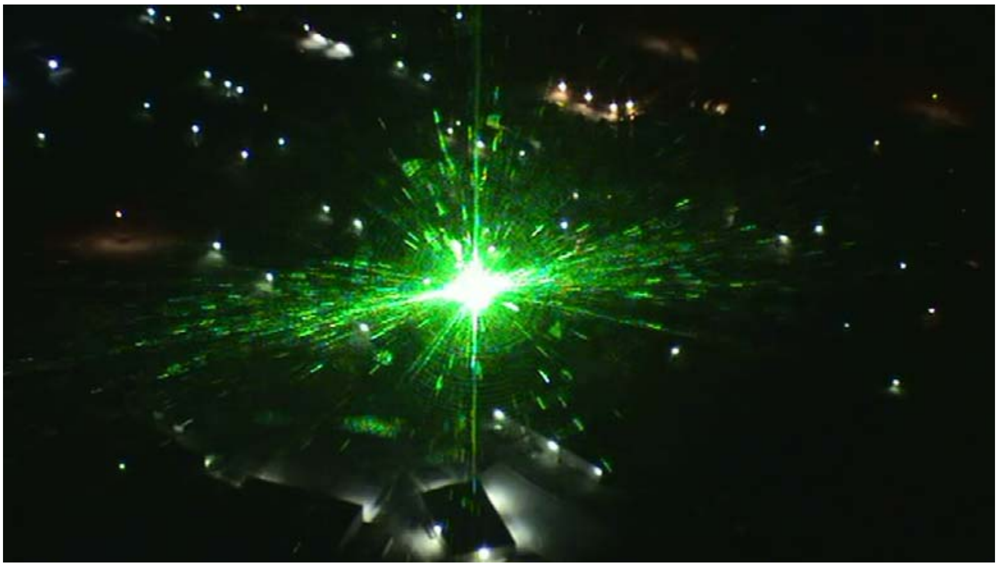

Figure 34 Helicopter canopy being hit by the beam of 3.5 mW green laser pointer at a distance to the source of about 1,600 ft (this image corresponds to the inset with the red border in Figure 33).

After finishing the measurements for the eight light sources in a steady position the helicopter was tracked with the Dominator (see Figure). The helicopter flew anticlockwise circles around the test site so that the light beam of the Dominator hit the

canopy of the helicopter on the left side (see Figure). This was done both as a precaution and to enable light measurements. If the beam of the Dominator had been in front of the helicopter the pilot might have experienced problems reading his instruments and looking outside. Since the light beam was on the left side of the helicopter at all times we could perform light measurements without endangering the flight in any way. The pilot voluntarily looked into the beam several times and for longer periods (in the order of a minute) and reported that he experienced no problems. The mean illuminance of the outside world in the field of view of the pilot was 0.6 lux. The illuminance in the direction of the Dominator was 470 lx at a distance between about 550 and 600 meter. This value agrees with the value we computed by extrapolating the ground measurements (see Figure 16).

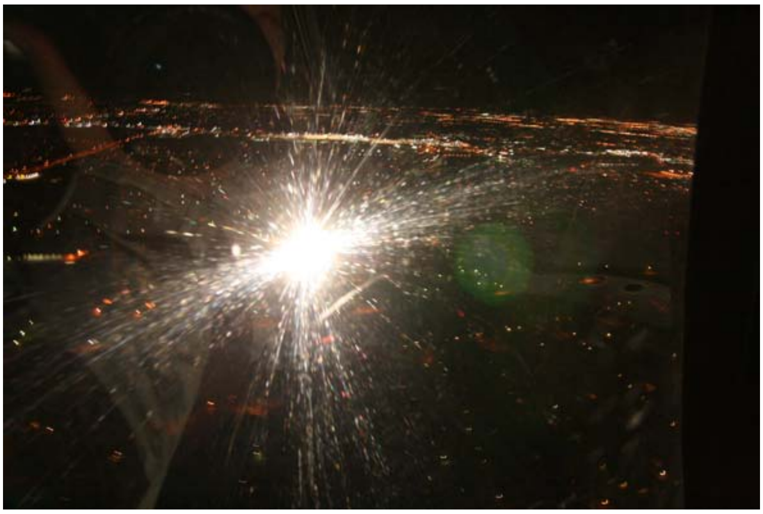

Figure 35 The impact of the tracking Dominator.

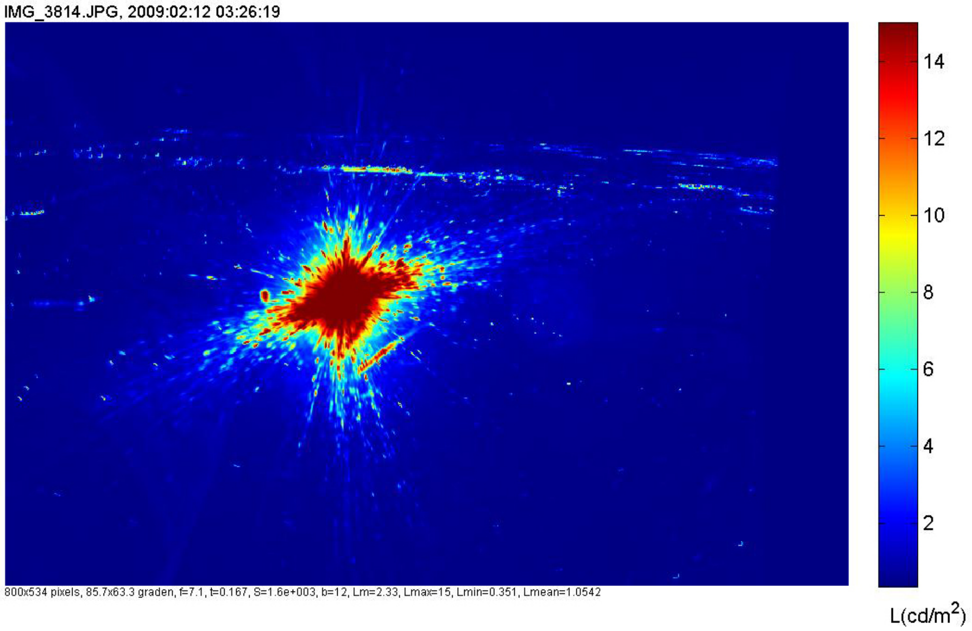

Figure 36 The luminance distribution resulting from the impact of the tracking Dominator.

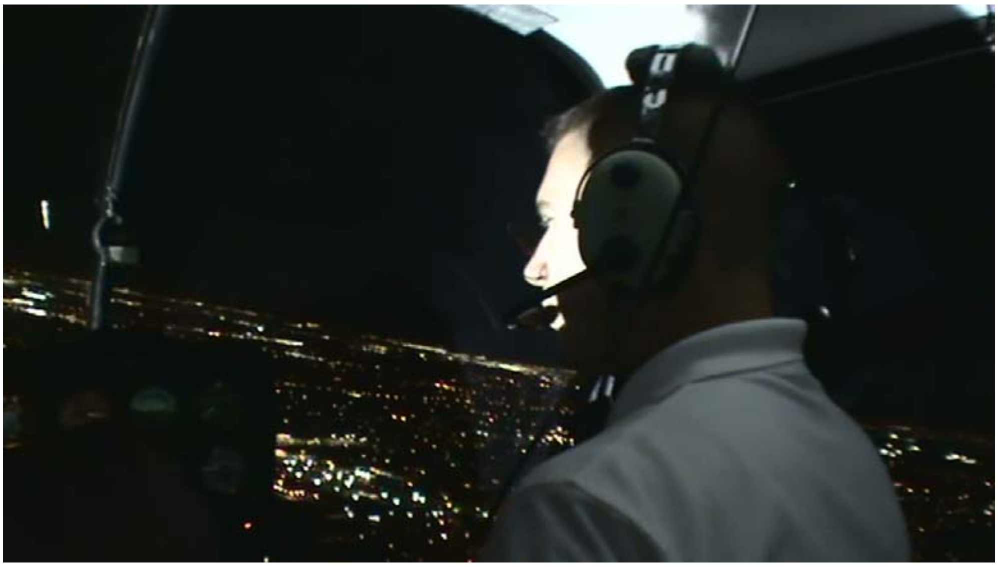

Figure 37 The helicopter pilot looking outside while the helicopter cockpit is illuminated by the beam of the Dominator.

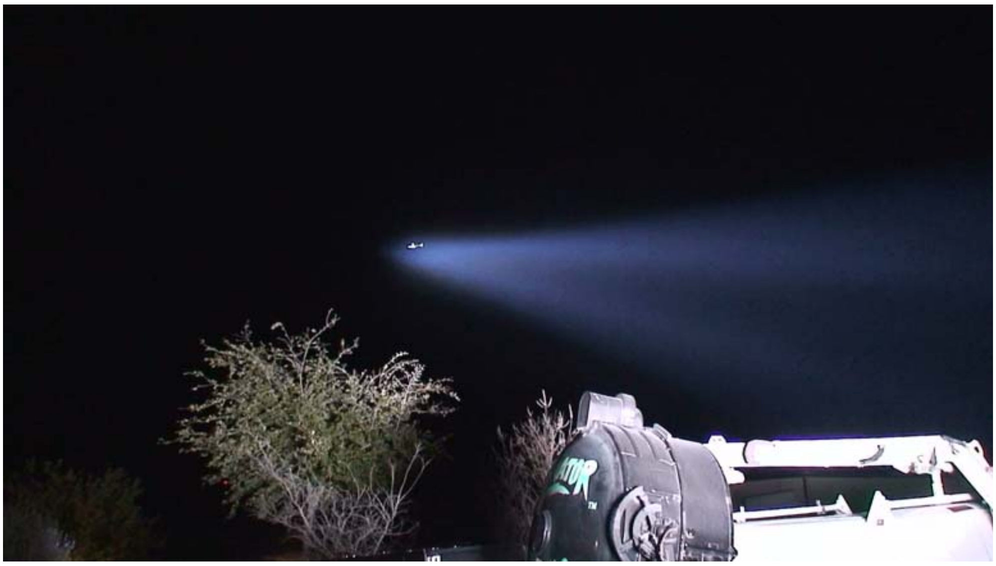

Figure 38 The helicopter being tracked by the beam of a searchlight.

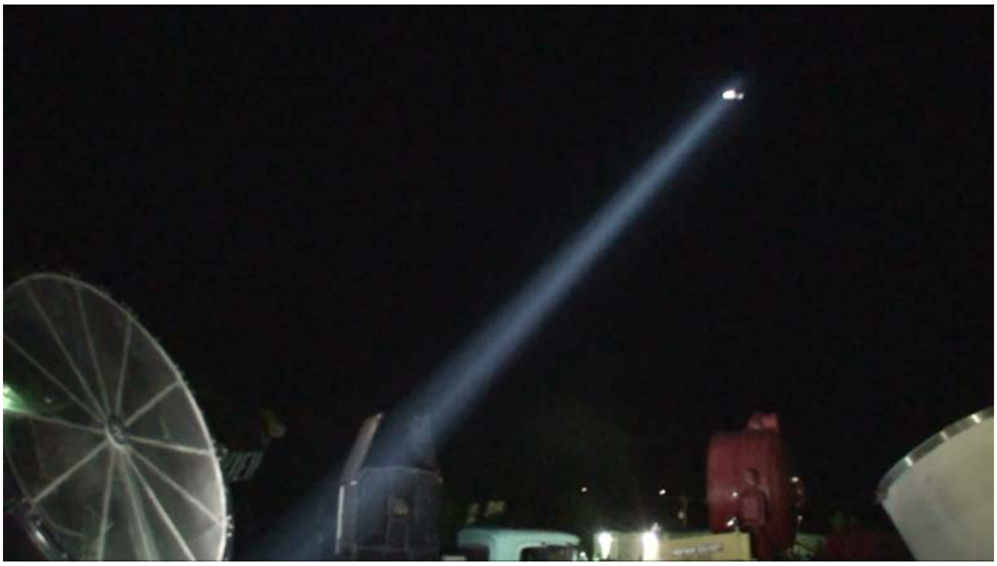

Figure 39 The helicopter being hit by the beam of a searchlight.

3.4 The Luxor light beam

On February 12, 2009, around 9.00 PM local time, we observed the high intensity light beam on top of the Luxor hotel in Las Vegas, while circling around it in a helicopter. Although the helicopter company had obtained permission (both from the FAA and from the local aviation authorities) to actually fly through the beam itself, it turned out that the pilot refused to cross the beam during the experiment. We could therefore only observe the beam sideways. The pilot's refusal to cross the beam was probably due to the unusual nature of our request, and had nothing to do with the characteristics of the beam itself. The pilot mentioned that the beam is actually appreciated by most pilots as a useful navigation beacon. Since this beam is pointing straight up into the sky the pilot would not have looked straight into the beam at any time if he had actually flown through it (the beam would have been shielded by the lower part of the helicopter). However, light measurements would have been possible by sticking a sensor out of the windows.

At 42.3 billion candela, the Luxor Sky Beam is claimed to be the most powerful beam of light in the world. Computer-designed curved mirrors collect the light from 39 Xenon lamps, focusing them into one intense, narrow beam. On a clear night, the Sky Beam is visible up to 250 miles away to an airplane at cruising altitude, and is clearly visible from outer space (source www.luxor.com). To save energy, most nights only half of the lamps are activated. Figure shows the calculated illuminance of the Luxor beam at different distances when it operates at full power (i.e. when all its lights are on). The calculation is based on the 42.3 billion Candela specification provided by the Luxor hotel itself (www.luxor.com). If only half of the lamps are on the given values should be divided by two.

The beam of the Luxor hotel was easier to see from the ground (Figure) than from the sky (Figure). From the ground one sees the light that is reflected downwards by the dust particles in the atmosphere (Figure). From a distance in the air the beam is seen with low contrast against the background which is also covered with high intensity city lights. This finding is illustrated in Figure which shows a sideways view of the Luxor

hotel in Las Vegas by night and the corresponding luminance distribution around the hotel and the beam. Figure shows that the beam seen from the side has only a very low contrast with it surroundings, whereas other signs in the scene have much higher contrast. Closer to the hotel and looking down onto the top of the hotel one can only see the light source itself (Figure). Although the Luxor beam may be the brightest searchlight around, we also noticed many other light sources while flying over Las Vegas at night that appeared equally bright.

When circling the Luxor beam with the helicopter we observed that the beam did not cause any visual interference effects, even close to the beam. This agrees with our findings in the previous section for the other high intensity lights, which only caused slight interference effects when they were directly pointed into the cockpit.

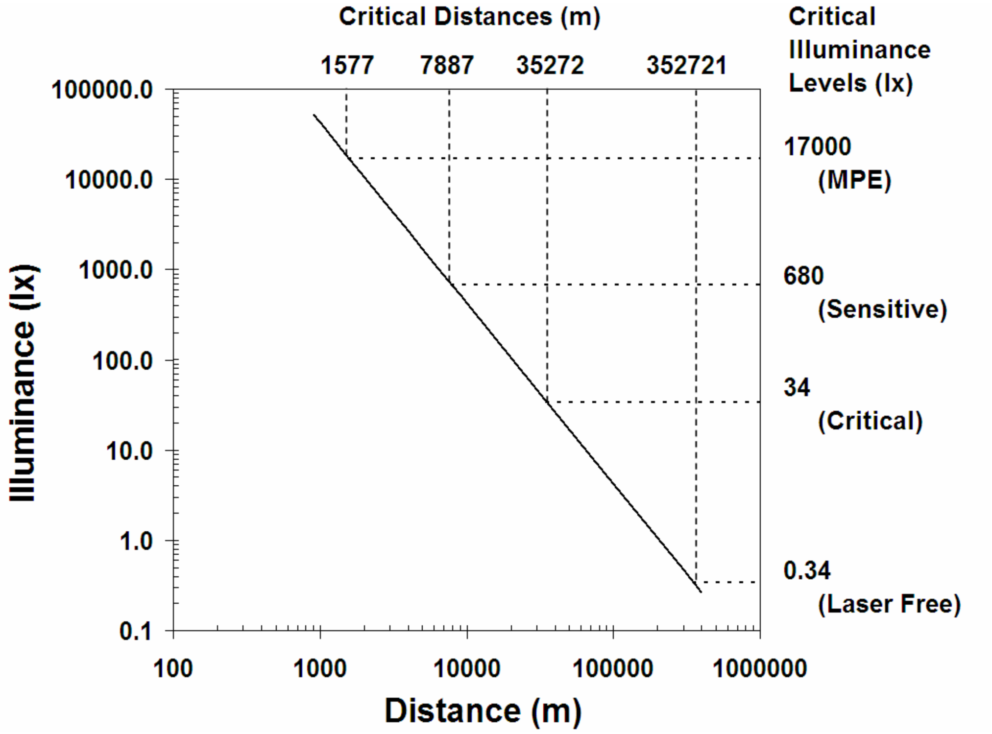

Figure 40 The illuminance of the Luxor beam as a function of distance when it all its lights are activated.

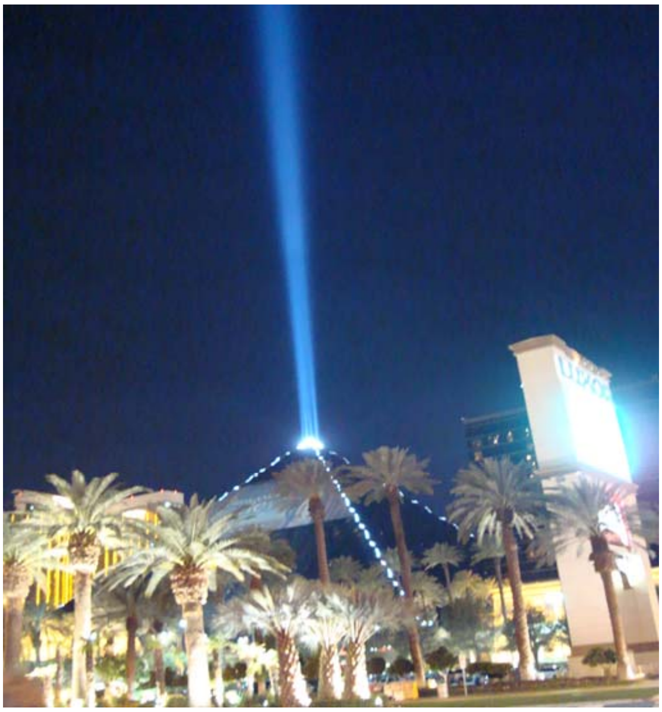

Figure 41 The Luxor hotel in Las Vegas by night. The beam of the high intensity light on top points straight up into the sky.

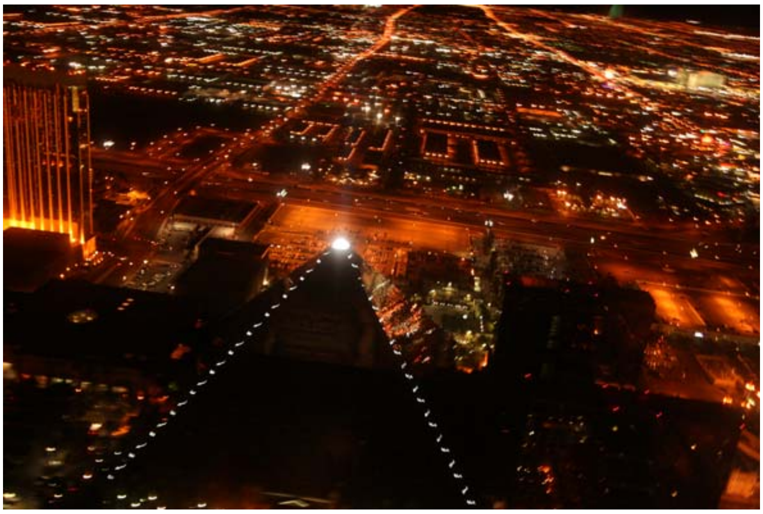

Figure 42 The high intensity light on top of the Luxor hotel seen from the helicopter.

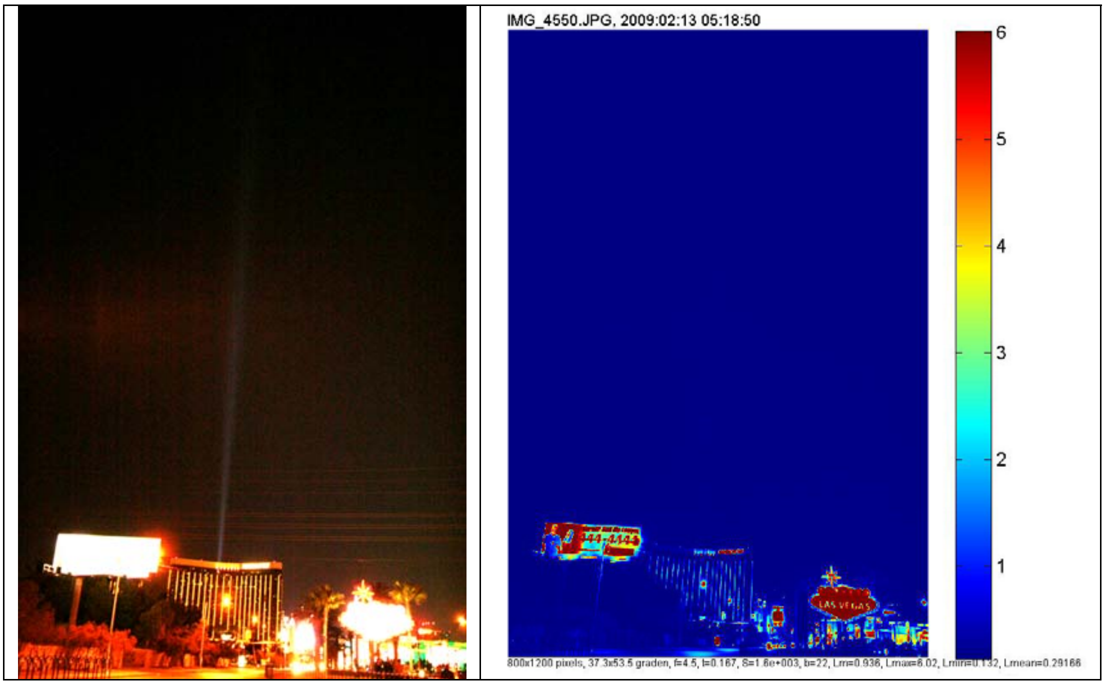

Figure 43 The luminance distribution around the Luxor hotel in Las Vegas by night.

4 Safety measures

In our flight experiments we observed no glare or flashblindness effects of high intensity light sources in the field of view. Glare only occurred when the light source intentionally tracked the helicopter for a longer period of time (minutes). Hence there appears to be no need for safety measures outside the Laser Free Zone. Inside the Laser Free Zone high intensity light sources may cause distraction. The next two sections list some safety measures that can be taken by respectively the proponents of outdoor high intensity lights and by the aviation authorities, in order to guarantee the safety of aircraft operations when high intensity lights need to be used near or inside the Laser Free Zone.

4.1 Measures by proponents

Proponents of high intensity lights in the open air should take appropriate control measures to ensure that their systems do not exceed the flight zone exposure levels at any time. One or more of the following control measures may be used to mitigate the risk of interference with aircraft operations (SAE, 2008).

Shielding: Physical barriers, baffles, or shields may be used to prevent illumination in an unwanted direction. These barriers may also include adjacent fixed structures, such as buildings, or natural terrain.

Pointing Direction. Azimuth and elevation (i.e., pan and tilt) control to prevent illumination in an unwanted direction.

Motion Limitation. The motion of a beam may be limited such that it either follows a predictable pattern or remains stationary.

Safety Observer. An observer may be tasked with watching the airspace through which a high intensity light beam is projected to ensure that the beam does not illuminate any aircraft.

Rotation Speed. The rotation speed of a high intensity light should not appear similar to that of a civilian or military airport beacon.

Onsite Communication with ATC (air traffic control). The onsite contact will have direct communication available to reposition or turn off the light at the request of the appropriate ATC facility. This measure may be used when the high intensity light is operating in an area where it would be otherwise objectionable.

4.2 Measures by aviation authorities

The local aviation authorities can inform air traffic about activities involving high intensity lights in the navigable airspace by issuing a NOTAM (Notice to Airmen).

In addition, the local aviation authorities can also impose safety zones around airports to mitigate the risk of interference of high intensity lights with aircraft operations

.

5 Two scientific laser systems and their safety protocols

LIDAR (Light Detection and Ranging) is an optical remote sensing technology that measures properties of scattered light to find range and/or other information of a distant target. The prevalent method to determine distance to an object or surface is to use laser pulses. Like the similar radar technology, which uses radio waves instead of light, the range to an object is determined by measuring the time delay between transmission of a pulse and detection of the reflected signal. LIDAR technology plays a key role in atmospheric profiling. Raman LIDAR exploits inelastic scattering to single out the gas of interest from all other atmospheric constituents. A small portion of the energy of the transmitted light is deposited in the gas during the scattering process, which shifts the scattered light to a longer wavelength by an amount that is unique to the species of interest. The higher the concentration of the gas, the stronger the magnitude of the backscattered signal. Raman LIDAR offers the possibility to routinely perform water vapor, aerosols and clouds measurements simultaneously and in exactly the same atmospheric volume with a single instrument. This enables the study of interrelationships between aerosols, clouds and water vapor without ambiguities of differences in space and time.

In the Netherlands there are currently two operational LIDAR systems for atmospheric research. One is operated by ESA-ESTEC (European Space Agency – European Space Research and Technology Centre) in Noordwijk, and the other one by RIVM (Dutch National Institute of Public Health and Environment Protection) in Bilthoven. We visited both systems and interviewed their operators on the safety procedures that have been implemented to guarantee air traffic safety when these systems are operational.

5.1 The Esa ESTEC LIDAR system

The laser source of the ESA-ESTEC LIDAR system (see Figure) is a pulsed Nd:YAG laser (CFR37) operating at 1064 nm (95 mJ, 11.9 ns), 532 nm (47 mJ, 9.8 ns), and 355 nm (72 mJ, 8.3 ns). The maximum pulse frequency is 10 Hz. The beam divergence at 86.5 % is 0.8 mrad for both the second and third hamonics before the beam expanders. The LIDAR is positioned in a Mercedes 207 Mini Van with a roof hatch for vertical operation (see Figure c). Although the system is mobile, the LIDAR will operate only when the van is completely stopped on a predetermined location.

ESA-ESTEC performed an extensive risk evaluation (Nillesen, 2008). The laser safety calculations in this hazard and risk assessment are based on the laser safety standard IEC EN 60825 (IEC, 2001). The nominal ocular hazard distances (the threshold distances below which eye damage may occur on extended viewing) calculated for worst case conditions (no atmospheric attenuation etc.) are 381 m, 69 km, and 34.8 km for respectively the 355, 532 and 1064 nm bands. Since the laser beam is directed vertical, all types of airplanes could get in contact with this laser radiation. It is unlikely, but not impossible, that the laser light causes temporarily or permanent eye injuries to airspace users. A potential risk exists, getting exposed to laser light, for airspace users (i.e. ‘delta kites’, sailplanes, helicopters, ‘Cessna types’ and jet types) who are making a sharp curve and ‘delta kite’ flyers who are looking down to earth and simultaneously crossing the vertical laser beam. Large airplanes can be excluded from

this potential risk since this type of airplane has a maximum tilt, never exceeding 20°, so the laser beam can never enter the aircraft, unless if pointed at an angle different from the vertical.

The LIDAR operator will be instructed with the laser safety issues. During the measurements the operator will be present at all times and will be able to activate the remote interlock/laser safety shutter or emergency button. When the system is activated laser safety observers will watch its surrounding airspace, and can instruct the LIDAR operator to interrupt laser transmissions when aircraft appear overhead. Since the testing locations are preselected advance notice can also be given to the appropriate aviation authorities.

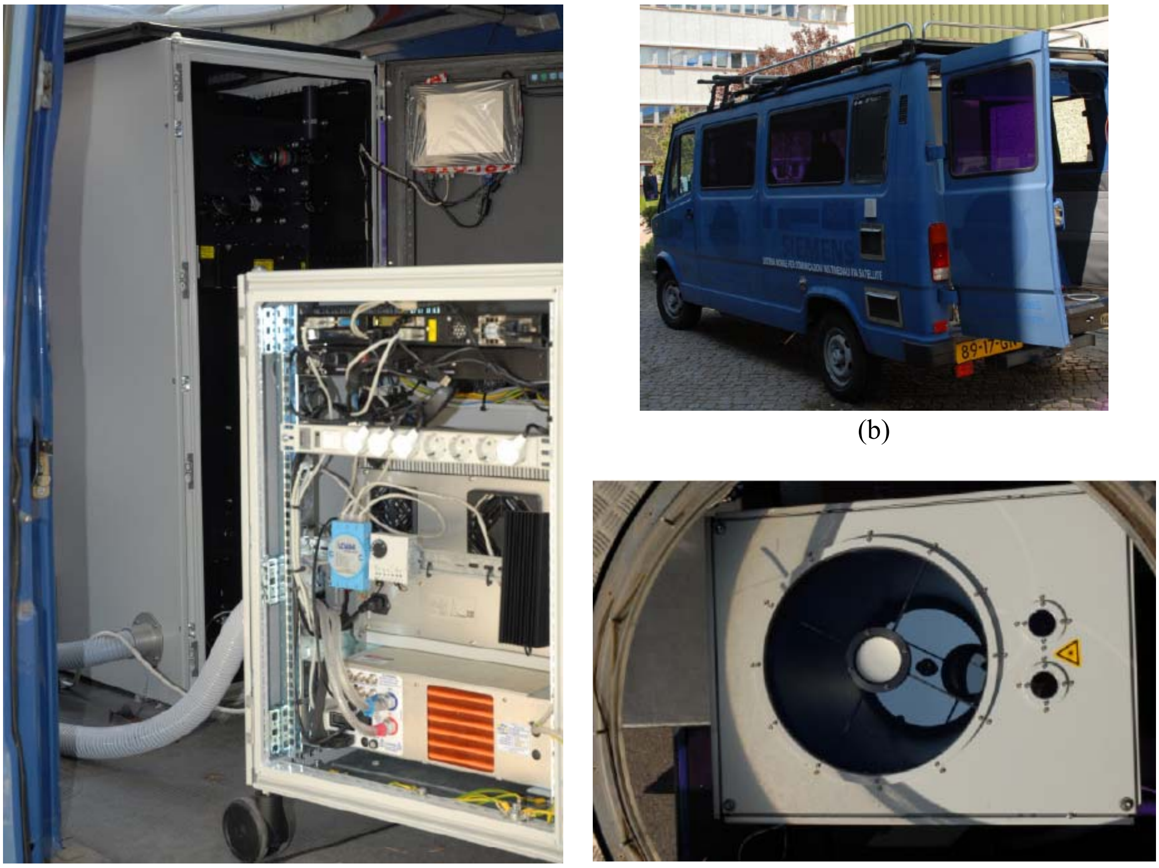

(a) (b) (c)

Figure 44 The ESA-ESTEC LIDAR system (source: ESA-ESTEC). (a) The 4 channel Raman LIDAR. (b) Laser aperture and collector optics. (c) Mercedes 207 Minivan.

5.2 The RIVM LIDAR system

The RIVM (National Institute for Public Health and the Environment) in Bilthoven has built CAELI (Cesar Water Vapour, Aerosol and Cloud Lidar) - an advanced high-power Raman LIDAR system to routinely monitor water vapour, aerosols and clouds (Wilson et al., 2008). The system contains a Nd:YAG laser with second and third harmonic generators.

This laser emits at 1064 nm (fundamental wavelength), 532 nm (second harmonic) and 355 nm (third harmonic), at 30 Hz, and emits about 12 W at
355 nm, with a pulse duration of 8- 10 ns at a 10 Hz repetition rate.

The system is currently tested in Bilthoven at the RIVM site (coordinates: 52.12 N, 5.2 E) and will ultimately be deployed at the Cabauw Experimental Site for Atmospheric Research (CESAR - site coordinates: (51.97° N, 4.92° E; site link: http://www.cesar-observatory.nl) where a whole suite of advanced instruments for atmospheric measurements is operated with emphasis on atmospheric profiling.

When the system is activated laser safety observers watch its surrounding airspace, and can instantaneously interrupt laser transmissions when aircraft appear overhead. However, since even highly trained and diligent observers may miss small aircraft or aircraft flying at high altitudes, the system will be extended with a modified X-band radar system specially designed to detect approaching aircraft (Duck et al., 2005). Risk analysis indicates that the probability of accidentally illuminating an aircraft with the LIDAR laser beam during joint LIDAR–radar operations is very low.

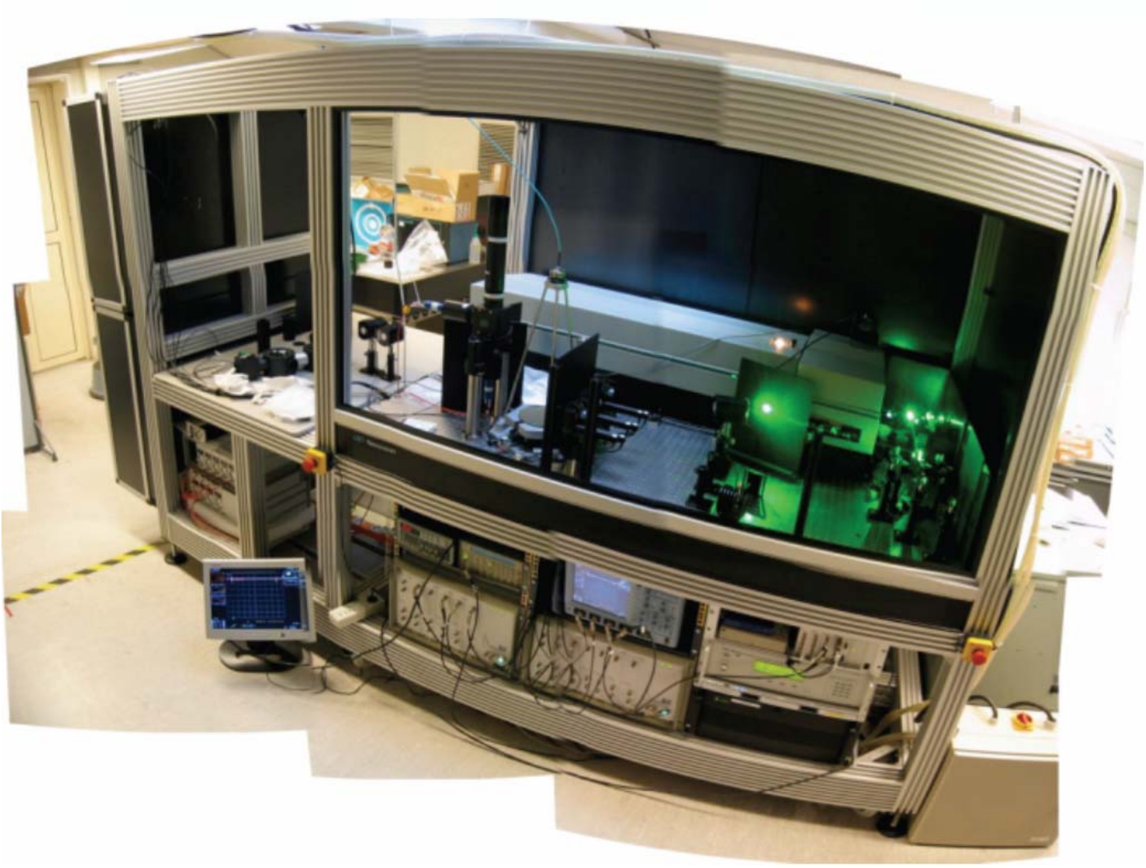

Figure 45 Complete system layout (source: RIVM). The frame containing the LIDAR system is in the middle of a room in a 20 ft container, so that it can be accessed from all sides for maintenance. Service elements such as large power supplies are in a separate compartment.

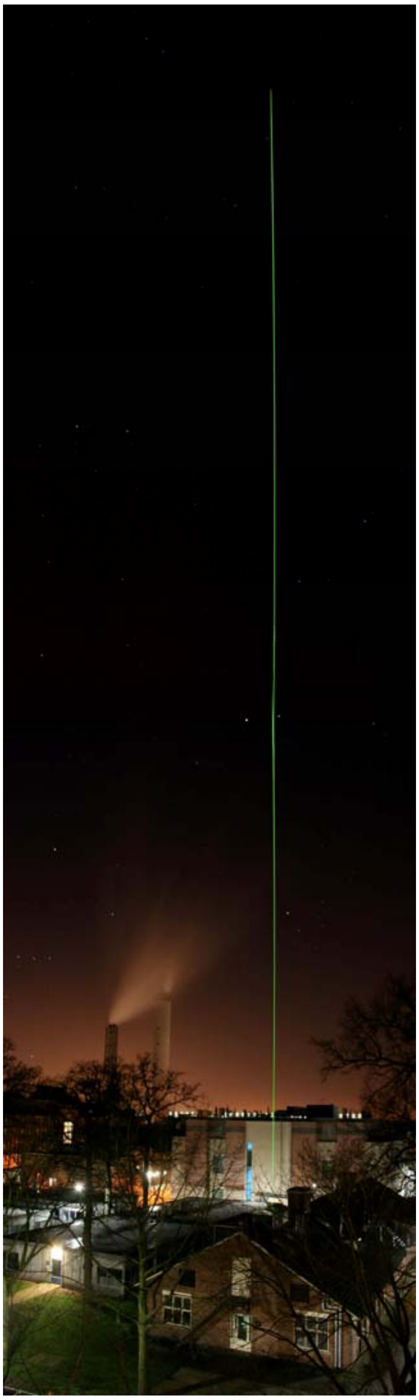

Figure 46 The RIVM LIDAR in operation at night.

6 Conclusions

We investigated the effects of several high intensity light sources (other than lasers) on pilot vision. In addition, we also tested the effects of a common 3.5 mW green laser pointer.

The broadband high intensity searchlights used in this study cannot cause physical damage at distances above 100 m. Below 100 m these lights cannot cause physical damage without prolonged exposure times. This conclusion agrees with earlier findings of the FAA (SAE, 2008).

In normal use, broadband high intensity light sources pointing in navigable airspace, whether stationary or moving, cause no concern for aviation safety outside the Laser Free Zone. Outside the Laser Free Zone pilots may experience glare and distraction when a high intensity light beam is intentionally tracking the canopy of the aircraft. Distraction may also occur when the high intensity light is the only bright light source in its environment.

The visual impact of a green laser pointer was more serious than expected. The light was unacceptably bright and caused serious glare at a distance of about 500 m. At this distance it also elicited brief afterimages. The laser pointer could not be seen when its beam did not hit the eye.

Both LIDAR systems (at ESA-ESTEC, Noordwijk and at RIVM, Bilthoven) that are currently operational for atmospheric research have sufficient safety precautions and cause no appreciable risk for aviation safety.

We conclude that there is no need to restrict broadband high intensity light sources pointing in navigable airspace anywhere outside the Laser Free Zone. Intentionally tracking aircraft with a high intensity light, and especially with lasers, shall be prohibited. The safety protocols of the scientific laser systems that are currently used in the Netherlands are sufficient to guarantee aviation safety.

7 References

American National Standards Institute (2005). *American national standard for the safe use of lasers* (Standard Z136.6). New York, USA: The Laser Institute of America.

American National Standards Institute (2007). *American national standard for the safe use of lasers* (Standard Z136.1). New York, USA: The Laser Institute of America.

Arecchi, A.V., Messadi, T. & Koshel, R.J. (2007). *Field Guide to Illumination.* Bellingham, WA: SPIE Press.

Barsalou, N. & Reddix, M.D. (2000). Bounding the tactical significance of lasers on TACAIR and CSAR missions. *Countering the directed energy threat: are closed cockpits the ultimate answer?*, RTO-MP-30 (pp. 9-1-9-8). Neuilly-sur-Seine, France: North Atlantic Treaty Organization - Research Technology Organization.

CIE. (2006). *Photobiological safety of lamps and lamp systems* (Report CIE S 009/E:2002). Vienna, Austria: International Commission on Illumination CIE.

Duck, T.J., Firanski, B., Lind, F.D. & Sipler, D. (2005). Aircraft-protection radar for use with atmospheric lidars. *Applied Optics, 44(23),* 4937-4945.

Elias, B. (2005). *Lasers Aimed at Aircraft Cockpits: Background and Possible Options to Address the Threat to Aviation Safety and Security* (Report RS22033). Washington, DC: Congressional Research Service - The Library of Congress.

FAA. (2008). *Procedures for handling airspace matters* (Report Order JO 7400.2G, available at http://www.faa.gov/airports_airtraffic/air_traffic/publications/atpubs/AIR/). Washington D.C.: U.S. Federal Aviation Administration.

Gloor, S., Wütrich, S., Lüthy, W. & Weber, H.P. (1995). *Recovery time of the human eye after exposure to foveal glare of near-IR and near-UV monochromatic light* (Report 1994/95 Ly-33). Bern, Swiss: Institute of Applied Physics, University of Bern.

Gloor, S., Wütrich, S., Lüthy, W. & Weber, H.P. (1996). Recovery time of the human eye after exposure to extrafoveal monochromatic glare. *International Journal of Lighting Research and Technology, 28(3),* 117-121.

ICAO-LFSSG (2003). *Manual on laser emitters and flight safety* (Doc 9815 AN/447). Montreal, CA: International Civil Aviation Organization.

IEC (2001). *Safety of laser products, Part 1: Equipment classification, requirements and user's guide* (IEC 60825-1:2001). Geneva, Switzerland: International Electronic Commission.

Krebs, T., Gloor, S., Wütrich, S., Lüthy, W. & Weber, H.P. (1994a). Recovery time of the human eye after brief exposures to monochromatic glaring light. *International Journal of Lighting Research and Technology, 26(4),* 195-197.

Krebs, T., Gloor, S., Wütrich, S., Lüthy, W. & Weber, H.P. (1994b). Recovery time of the human eye after exposure to a glare source at various intensities. *International Journal of Lighting Research and Technology, 26(4),* 199-201.

Nakagarawa, V.B., Montgomery, R.W., Dillard, A.E., McLin, L. & Connor, C.W. (2008a). *The effects of laser illumination on operational and visual performance of pilots during final approach* (Report DOT/FAA/AM-04/9). Washington, DC, SA: Office of Aerospace Medicine.

Nakagarawa, V.B., Montgomery, R.W. & Wood, K.J. (2007). Aircraft accidents and incidents associated with visual effects from bright light exposures during low-light flight operations. *Optometry - Journal of the American Optometric Association, 78(8),* 415-420.

Nakagarawa, V.B., Montgomory, R.W. & Wood, K.J. (2008b). *Laser illumination of aircraft by geographic location for a 3-year period (2004-2006)* (Report DOT/FAA/AM-08/14). Washington, DC: Office of Aerospace Medicine.

Nakagarawa, V.B., Wood, K.J. & Montgomory, R.W. (2006). *A review of recent laser illumination events in the aviation environment* (Report DOT/FAA/AM-06/23). Washington, DC: Office of Aerospace Medicine.

Nakagarawa, V.B., Wood, K.J. & Montgomory, R.W. (2008c). Laser exposure incidents: Pilot ocular health and aviation safety issues. *Optometry - Journal of the American Optometric Association, 79(9),* 518-524.

Nillesen, C. (2008). *LIDAR Hazard and risk assessment* (Report LAPROCON Report Number LPC 08011CN071). Heeze, The Netherlands: Ingenieurs & Adviesbureau LAPROCON.

Payne, D.J., et al. (1999). Comparative study of laser damage threshold energies in the artificial retina. *Journal of Biomedical Optics, 4(3),* 337-344.

Reddix, M.D., DeVietti, T.L., Knepton, J.C. & D'Andrea, J.A. (1990). *The effect of three levels of laser glare on the speed and accuracy of target location performance when viewing abriefly presented visual array* (Report NAMRL-1359). Pensacola, FL: Naval Aerospace Medical Research Laboratory.

SAE (1999). *Human factors considerations for outdoor laser operations in navigable airspace* (Aerospace Standard SAE AS4970). Warrendale, PA, USA: Society of Automotive Engineers.

SAE. (2003). *Observers for laser safety in the navigable airspace* (Report ARP5535). Warrendale, PA: SAE International.

SAE. (2004). *Control measures for laser safety in the navigable airspace* (Report ARP5572). Warrendale, PA: SAE International.

SAE. (2005). *Safety considerations for lasers projected in the navigable airspace* (Report ARP5293). Warrendale, PA: SAE International.

SAE. (2008). *Safety considerations for high-intensity lights (HIL) directed into the navigable airspace* (Report ARP5560). Warrendale, PA: SAE International.

Sheehy, J.B. (1989). *Dazzling glare: protection criteria versus visual performance* (Report AD-A219676 ; NADC-89076-60). Warminster, PA: Naval Air Development Center.

Wilson, K.M., Apituley, A. & Potma, C. (2008). CAELI - a multi-wavelength Raman lidar for the diurnal observation of clouds, aerosol, and water vapour profiles and boundary layer structures. *Proceedings of the 24th International Laser Radar Conference*, (pp. 91-94). Boulder, Colorado.

Wütrich, S., Schmid, M., Lüthy, W. & Weber, H.P. (1997). Recovery time of the human eye after exposure to laser light. In S.K. Park & R.D. Juday (Eds.), *Visual Information Processing VI*, SPIE-3074 (pp. 2-12). Bellingham, WA: The International Society for Optical Engineering.

Zheltov, G., Glazkov, V., Podol'tzef, A., Linnik, L. & Privalov, A. (1989). Retinal damage from intense visible light. *Helath Physics, 56(5),* 625-630.

8 Signature

Soesterberg, June 2009

TNO Defence, Security and Safety

P.A.J. Punte, MSc
Head of department

Dr A. Toet
Author

Distribution list

The following agencies/people will receive a complete copy of the report.

5 ex.	Inspectie van Verkeer en Waterstaat<br>ing. H. van den Berg
2 ex.	TNO Defensie en Veiligheid, vestiging Soesterberg<br>Archief
3 ex.	TNO Defensie en Veiligheid, vestiging Soesterberg<br>Dr A. Toet<br>N. van der Leden, BSc<br>J.W.A.M. Alferdinck, BSc

BACK TO SEARCH RESULTS

TNO

PUBLICATION

THE OUTDOOR USE OF LASERS AND OTHER HIGH INTENSITY LIGHT... (2009)

OPEN ACCESS

Main

Save publication

Title	The outdoor use of lasers and other high intensity light sources in relation to air traffic safety
Author	Toet, A.; Leden, N. van der; Alferdinck, J.W.A.M.
Originator	TNO Defensie en Veiligheid
Date issued	2009-01-01
Access	Open Access
Language	English
Type	Report
Publisher	TNO

Abstract

Lichtbronnnen met een hoge intensiteit zoals skybeamers worden steeds vaker gebruikt in de open lucht bij evenementen of als aandachttrekkers bij commerciële vestigingen. Andere lichtbronnen met een hoge intensiteit zoals spotlights worden vaak ingezet bij bouwwerkzaamheden en sportevenementen. Als gevolg daarvan ontvangt de Inspectie Verkeer en Waterstaat (IVW) regelmatig het verzoek om toestemming te verlenen voor het gebruik van lichtbronnen met een hoge intensiteit in de open lucht. Omdat niet bekend is hoe deze lichtbronnen de luchtvaartveiligheid kunnen beïnvloeden, is het IVW momenteel niet in staat de risico's die het gebruik van deze lichtbronnen opleveren voor de luchtvaartveiligheid goed in te schatten en hun gebruik op een verantwoorde manier te reguleren. IVW heeft daarom TNO gevraagd om te onderzoeken welke effecten lichtbronnen met een hoge intensiteit (behalve lasers) kunnen hebben op de luchtvaartveiligheid , en welke veiligheidsmaatregelen eventueel genomen zouden moeten worden om deze lichtbronnen veilig in het open luchtruim te kunnen gebruiken. Verder verzocht IVW TNO om na te gaan welke veiligheidsmaatregelen er reeds zijn getroffen bij enkele lasersystemen die in Nederland worden gebruikt voor atmosferisch onderzoek, en om vast te stellen of die maatregelen afdoende zijn om de luchtvaartveiligheid te garanderen, en om eventueel aanvullend maatregelen voor te stellen. We hebben de effecten onderzocht van verschillende hoge intensiteits lichtbronnen met een breed-spectrum, en van een 3,5 mW laser pointer die groen licht produceert. Verder onderzochten we de veiligheidsmaatregelen die worden toegepast bij twee lasersystemen die in Nederland worden gebruikt voor atmosferisch onderzoek. Kennis over de visuele effecten van hoge intensiteits
