# Supplementary material for: Advanced liquid crystal-based switchable optical devices for light protection applications: principles and strategies
Source: Light Sci Appl. 2023 Jan 3;12:11. doi: 10.1038/s41377-022-01032-y (PMC9807646; doi:10.1038/s41377-022-01032-y)
Supplement: Supplementary file 5 — Fig 5 copyright promotion [file 41377_2022_1032_MOESM5_ESM.pdf]

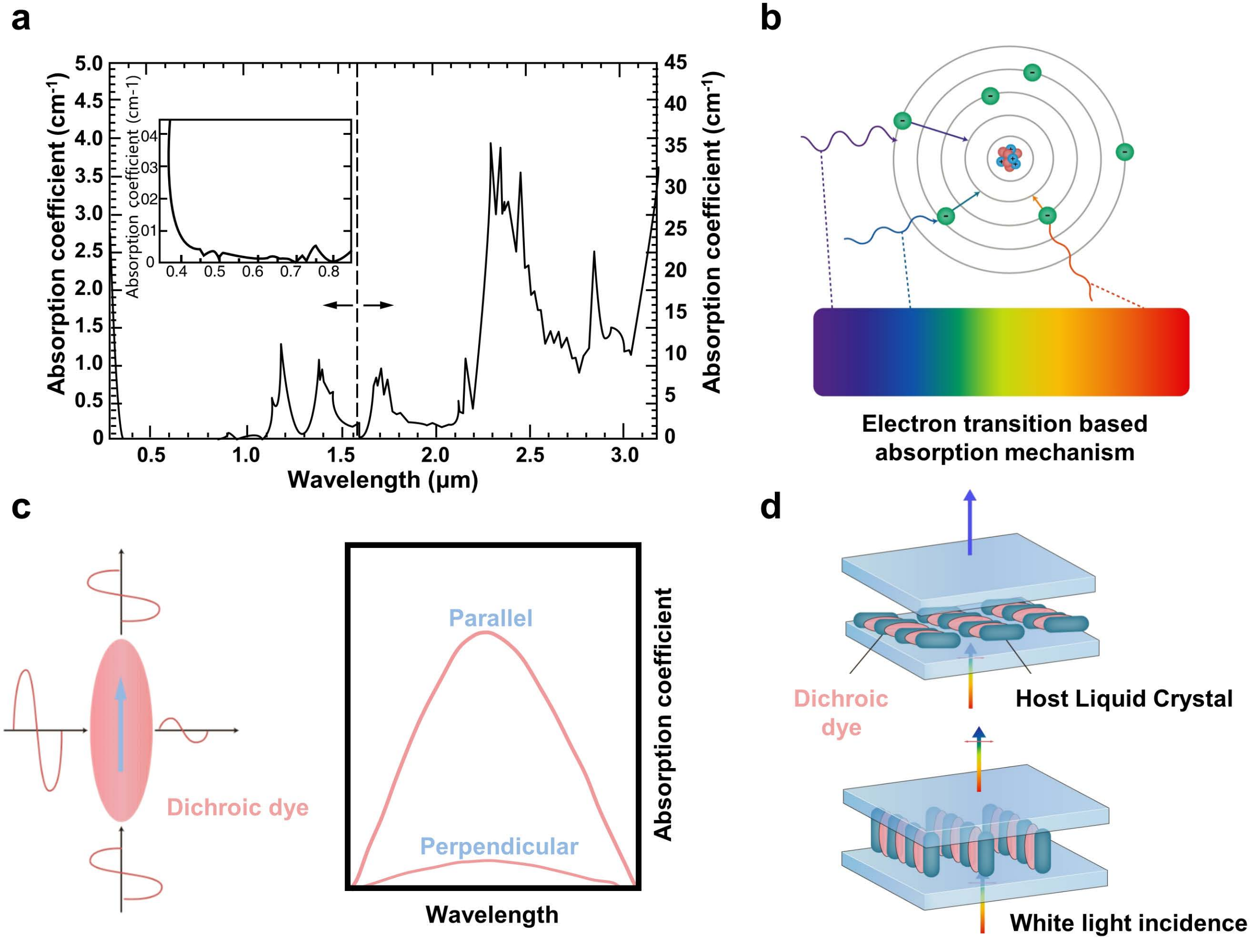

a
Absorption coefficient (cm-1)
Absorption coefficient (cm-1)
Wavelength (μm)
b
Electron transition based absorption mechanism
c
Dichroic dye
Parallel
Perpendicular
Absorption coefficient
Wavelength
d
Dichroic dye
Host Liquid Crystal
White light incidence

AIP PUBLISHING LICENSE TERMS AND CONDITIONS

Sep 20, 2022

This Agreement between Harbin Institute of Technology -- Ruicong Zhang ("You") and AIP Publishing ("AIP Publishing") consists of your license details and the terms and conditions provided by AIP Publishing and Copyright Clearance Center.

License Number	5392971446709
License date	Sep 20, 2022
Licensed Content Publisher	AIP Publishing
Licensed Content Publication	Journal of Applied Physics
Licensed Content Title	Absorption measurements of liquid crystals in the ultraviolet, visible, and infrared
Licensed Content Author	Shin-Tson Wu
Licensed Content Date	Oct 15, 1998
Licensed Content Volume	84
Licensed Content Issue	8
Type of Use	Journal/Magazine
Requestor type	Author/Researcher/Scientist
Format	Print and electronic
Portion	Figure/Table
Number of figures/tables	1
Will you be translating?	No
Title of new article	Advanced liquid crystal-based switchable optical devices for light protection applications: principles and strategies
Lead author	Ruicong Zhang, Zhibo Zhang, Jiecai Han, Lei Yang, Jiajun li, Zicheng Song Tianyu Wang, Jiaqi Zhu
Title of targeted journal	Light: Science & Applications
Publisher	Springer Nature
Expected publication date	Nov 2022
Portions	Figure 4
Requestor Location	Harbin Institute of Technology<br>No. 92, Xidazhi Street, Nangang District<br><br>Harbin, 150080<br>China<br>Attn: Harbin Institute of Technology
Total	**0.00 USD**

Terms and Conditions

AIP Publishing -- Terms and Conditions: Permissions Uses

AIP Publishing hereby grants to you the non-exclusive right and license to use and/or distribute the Material according to the use specified in your order, on a one-time basis, for the specified term, with a maximum distribution equal to the number that you have ordered. Any links or other content accompanying the Material are not the subject of this license.

1. You agree to include the following copyright and permission notice with the reproduction of the Material:"Reprinted from [FULL CITATION], with the permission of AIP Publishing." For an article, the credit line and permission notice must be printed on the first page of the article or book chapter. For photographs, covers, or tables, the notice may appear with the Material, in a footnote, or in the reference list.
2. If you have licensed reuse of a figure, photograph, cover, or table, it is your responsibility to ensure that the material is original to AIP Publishing and does not contain the copyright of another entity, and that the copyright notice of the figure, photograph, cover, or table does not indicate that it was reprinted by AIP Publishing, with permission, from another source. Under no circumstances does AIP Publishing purport or intend to grant permission to reuse material to which it

does not hold appropriate rights.
You may not alter or modify the Material in any manner. You may translate the Material into another language only if you have licensed translation rights. You may not use the Material for promotional purposes.

3. The foregoing license shall not take effect unless and until AIP Publishing or its agent, Copyright Clearance Center, receives the Payment in accordance with Copyright Clearance Center Billing and Payment Terms and Conditions, which are incorporated herein by reference.
4. AIP Publishing or Copyright Clearance Center may, within two business days of granting this license, revoke the license for any reason whatsoever, with a full refund payable to you. Should you violate the terms of this license at any time, AIP Publishing, or Copyright Clearance Center may revoke the license with no refund to you. Notice of such revocation will be made using the contact information provided by you. Failure to receive such notice will not nullify the revocation.
5. AIP Publishing makes no representations or warranties with respect to the Material. You agree to indemnify and hold harmless AIP Publishing, and their officers, directors, employees or agents from and against any and all claims arising out of your use of the Material other than as specifically authorized herein.
6. The permission granted herein is personal to you and is not transferable or assignable without the prior written permission of AIP Publishing. This license may not be amended except in a writing signed by the party to be charged.
7. If purchase orders, acknowledgments or check endorsements are issued on any forms containing terms and conditions which are inconsistent with these provisions, such inconsistent terms and conditions shall be of no force and effect. This document, including the CCC Billing and Payment Terms and Conditions, shall be the entire agreement between the parties relating to the subject matter hereof.

This Agreement shall be governed by and construed in accordance with the laws of the State of New York. Both parties hereby submit to the jurisdiction of the courts of New York County for purposes of resolving any disputes that may arise hereunder.

V1.2

Questions? customercare@copyright.com or +1-855-239-3415 (toll free in the US) or +1-978-646-2777.

AIP | Journal of Applied Physics

Absorption measurements of liquid crystals in the ultraviolet, visible, and infrared

Shin-Tson Wu

Citation: J. Appl. Phys. **84**, 4462 (1998); doi: 10.1063/1.368671
View online: http://dx.doi.org/10.1063/1.368671
View Table of Contents: http://jap.aip.org/resource/1/JAPIAU/v84/i8
Published by the American Institute of Physics.

Related Articles

Kinetic and geometric isotope effects originating from different adsorption potential energy surfaces: Cyclohexane on Rh(111)
J. Chem. Phys. 136, 214705 (2012)

Photothermal cantilever deflection spectroscopy of a photosensitive polymer
Appl. Phys. Lett. 100, 204103 (2012)

Infrared spectroscopy and modeling of co-crystalline CO2·C2H2 aerosol particles. I. The formation and decomposition of co-crystalline CO2·C2H2 aerosol particles
J. Chem. Phys. 136, 094509 (2012)

Infrared spectroscopy and modeling of co-crystalline CO2·C2H2 aerosol particles. II. The structure and shape of co-crystalline CO2·C2H2 aerosol particles
J. Chem. Phys. 136, 094510 (2012)

X-ray Raman scattering provides evidence for interfacial acetonitrile-water dipole interactions in aqueous solutions
J. Chem. Phys. 135, 164509 (2011)

Additional information on J. Appl. Phys.

Journal Homepage: http://jap.aip.org/
Journal Information: http://jap.aip.org/about/about_the_journal
Top downloads: http://jap.aip.org/features/most_downloaded
Information for Authors: http://jap.aip.org/authors

ADVERTISEMENT

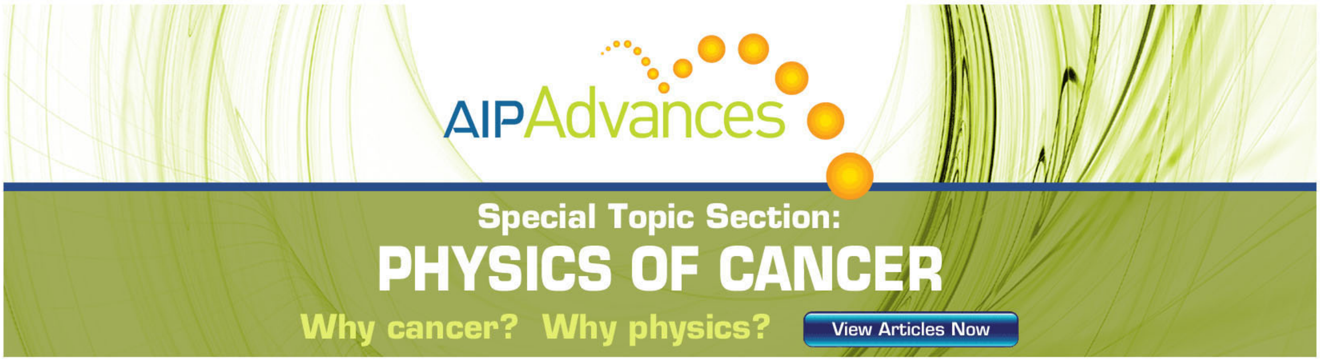

Downloaded 19 Jun 2012 to 139.184.30.132. Redistribution subject to AIP license or copyright; see http://jap.aip.org/about/rights_and_permissions

Absorption measurements of liquid crystals in the ultraviolet, visible, and infrared

Shin-Tson Wu[a)]
HRL Laboratories, 3011 Malibu Canyon Road, Malibu, California 90265

(Received 4 December 1997; accepted for publication 14 July 1998)

Complete absorption spectra of three liquid crystals with different conjugation length were measured in the 0.185–20 μm spectral range. In the ultraviolet region, the absorption is dependent on whether σ or π electrons are involved. In the mid and long infrared regions, several localized molecular vibration bands exist. The saddle absorption minimum occurs at around $\lambda \sim 0.8$ μm that is far from the electronic resonance and is right before the overtone molecular vibration begins. © *1998 American Institute of Physics.* [S0021-8979(98)03320-9]

I. INTRODUCTION

In many electro-optic applications using liquid crystal (LC) devices, light absorption could be an important issue depending on the wavelength region of interest. For instance, to steer a high power laser beam[1] or to project images to a large screen employing a high power lamp,[2] the absorbed light is converted to thermal energy and consequently heats up the LC cell.[3] Since the physical properties of a thermotropic LC (e.g., birefringence and viscosity) are sensitive to the temperature, the performance of the LC device will be affected through the absorption of light.

The major absorption of a LC compound occurs in two spectral regions: ultraviolet (UV) and infrared (IR). The $\sigma \rightarrow \sigma^*$ electronic transitions take place in the vacuum UV (100–180 nm) region whereas the $\pi \rightarrow \pi^*$ electronic transitions occur in the UV (180–400 nm) region.[4,5] The longer the molecular conjugation, the longer the absorption wavelength. The UV absorption affects the photostability and lifetime of a LC device. A high energy UV photon may break the chemical bond of a long chain LC molecule and cause deterioration in molecular alignment which, in turn, degrades the device performance.[6] In the visible region, the absorption is usually quite small. The major optical loss originates from light scattering due to the LC director fluctuations, rather than absorption.[7,8] In the near IR region, some overtone molecular vibration bands begin to appear. In the mid (3–5 μm) and long IR (7–14 μm) regions, there are many fundamental molecular vibration bands.[9,10] As a result, the baseline absorption can be quite large depending on the detailed molecular composition.

In this letter, we report complete absorption spectra of three LC compounds in the UV, visible, and IR regions. In Sec. II, we describe the molecular structures and specific features of the samples chosen for this study. The UV (185–400 nm) measurement method and experimental results are described in Sec. III. In Sec. IV, we present the measurement method and experimental data in the 0.4–3.2 μm region. The absorption in the visible and near IR regions are emphasized. Finally, in Sec. V, we present experimental results in the 2.5–20 μm region.

[a)]Electronic mail: swu@hrl.com

II. LC STRUCTURES

The objective of this study is to correlate absorption coefficient with molecular structures and then identify the low loss region for device applications. We have selected three LC compounds: CCH-301, PCH-32 and 5CB with their molecular structures shown below:

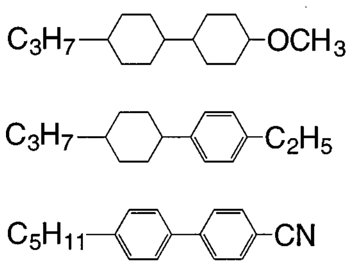

(CCH-301)

(PCH-32)

(5CB)

The phase transition temperatures (in °C) of CCH-301, PCH-32 and 5CB are listed in the following order: $\{K9N15I\}$, $\{K-1.5N(-70)I\}$, and $\{K24N35.3I\}$; where K, N and I represent crystalline, nematic and isotropic phases, respectively. The compound CCH-301 exhibits an enantiotropic phase, but its nematic-to-isotropic clearing point (15 °C) is below room temperature (~22 °C). This feature is particularly desirable for the absorption studies at room temperature where no heating equipment is required. In the nematic phase, light scattering surpasses absorption in the visible region and dominates the optical loss.[8] At an isotropic state, the long range ordering of LC molecules disappears so that the light scattering is greatly reduced. Therefore, the measured optical loss is mainly contributed from absorption. The PCH-32 has a monotropic phase transition, i.e., its nematic phase occurs during cooling process. This compound also possesses an isotropic phase at room temperature. Since the

 © 1998 American Institute of Physics

Downloaded 19 Jun 2012 to 139.184.30.132. Redistribution subject to AIP license or copyright; see http://jap.aip.org/about/rights_and_permissions

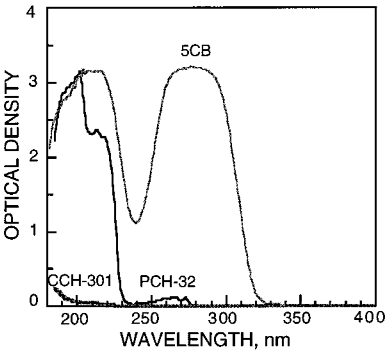

FIG. 1. Optical density of CCH-301, PCH-32 and 5CB. Cell gap $d \sim 0.3\ \mu m$. Both CCH-301 and PCH-32 were measured at $T \sim 22\ °C$, and 5CB at $T \sim 50\ °C$. To convert OD to absorption coefficient (α), use OD $= \alpha d/2.3$.

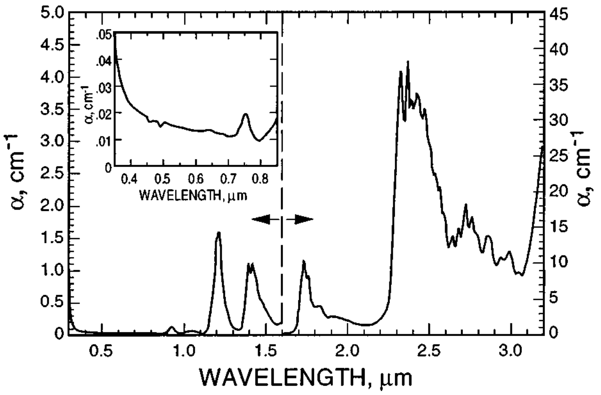

FIG. 2. Measured absorption coefficient of CCH-301 at $T \sim 22\ °C$.

clearing point of 5CB is 35.3 °C, all the measurements related to 5CB as reported here were conducted at 50 °C in order to avoid light scattering.

III. UV MEASUREMENTS

For the absorption measurements ranging from 185 to 3200 nm, we used the computer-controlled dual-beam Perkin-Elmer Lambda 9 spectrophotometer. For UV measurements, we limit the spectral range from 185 to 400 nm. Two experimental methods are frequently employed for UV measurements: (1) to use a very thin sample; and (2) to use the guest-host method.[5] In this work, we took the thin cell approach. A thin cell using highly transparent BaF_2 substrates was prepared. The cell gap was controlled to be $d \sim 0.3\ \mu m$. The sample cell was then measured against an identical BaF_2 substrate for correcting the surface reflections. Results of the selected three LC compounds are shown in Fig. 1. To convert the measured optical density (OD) to absorption coefficient (α), one can use the following relationship: OD$= \alpha d/2.3$.

From Fig. 1, CCH-301 has the weakest absorption in the 185–400 nm region. This is because the cyclohexane rings are saturated. The $\sigma \rightarrow \sigma^*$ electronic transitions occur at vacuum UV, but its tail extends to the 200 nm region, as observed from Fig. 1. On the other hand, the phenyl ring in PCH-32 and 5CB consists of two unsaturated $\pi \rightarrow \pi^*$ electronic transitions bands, designated as λ_1 and λ_2 (assuming $\lambda_1 < \lambda_2$). The λ_1 band consists of two closely overlapped bands. Their intensity is very strong. By contrast, the intensity and wavelength of the λ_2 band depends on the molecular conjugation length. The longer the conjugation, the stronger the transition intensity and the longer the transition wavelength. For PCH-32, the λ_2 band occurs at ~265 nm and has a relatively weak intensity. For 5CB, the λ_2 band shifts to 280 nm and its intensity is very strong. From the three-band model,[11] the transition intensity and wavelength of the $\sigma \rightarrow \sigma^*$ and two $\pi \rightarrow \pi^*$ bands determine the refractive index and birefringence dispersion of a LC material. From the data shown in Fig. 1, one can predict that 5CB would exhibit a much higher birefringence than PCH-32 when compared at the same reduced temperature and same wavelength.

For some electro-optic modulators in the UV region, low absorption LC materials need to be developed. From Fig. 1, the CCH series of LC compounds do possess a very small absorption at $\lambda > 250$ nm. Through a proper modification of LC structures, potential applications of LCs in the UV region should be possible.

IV. VISIBLE TO MID-IR MEASUREMENTS

In this wide spectral region (0.4–3.2 μm), the LC absorption could vary by nearly three orders in magnitude. In order to enhance the signal-to-noise ratio and improve the measurement accuracy, we used quartz cells with $d = 1$, 2, 10 and 20 mm. The quartz substrate we employed has a strong and relatively broad absorption band centered at ~2.73 μm. To correct the substrate absorption and surface reflections, we used the dual-beam method to obtain the normalized optical density. In each measurement, the sample cell was measured with reference to the 1 mm cell. In addition, we divide the measurements into two spectral regimes: 0.4–1.6 μm and 1.6–3.2 μm to accommodate the different absorption characteristics.

In the 0.4–1.6 μm regime, the absorption of LC is relatively weak. Thus, we chose to use a 10 mm sample cell. The measured optical density is equivalent to that of a 9 mm sample. These results were validated by measuring the optical density of the 10 and 1 mm cells separately. In the 0.35–0.85 μm region, the absorption is too small to be resolved by the 10 mm cell so that we used the 20 mm cell in order to improve the experimental accuracy. On the other hand, the LC absorption in the 1.6–3.2 μm regime is so strong that we need to reduce the cell thickness to 2 mm in order to maintain a reasonable transmittance. The experimental results of CCH-301, PCH-32 and 5CB are shown in Figs. 2, 3, and 4, respectively.

By examining the experimental results in these figures, we find a general tendency for all three LCs studied. The LC absorption decreases with wavelength rapidly in the visible region, reaching a minimum at around 0.7–0.8 μm and then gradually climb up at beyond 0.9 μm. The saddle absorption valley which appears in the near IR region is an interesting phenomenon. From the short wavelength side, the electronic resonance absorption declines rapidly as the wavelength gets farther away from UV. From the long wavelength side, the

Downloaded 19 Jun 2012 to 139.184.30.132. Redistribution subject to AIP license or copyright; see http://jap.aip.org/about/rights_and_permissions

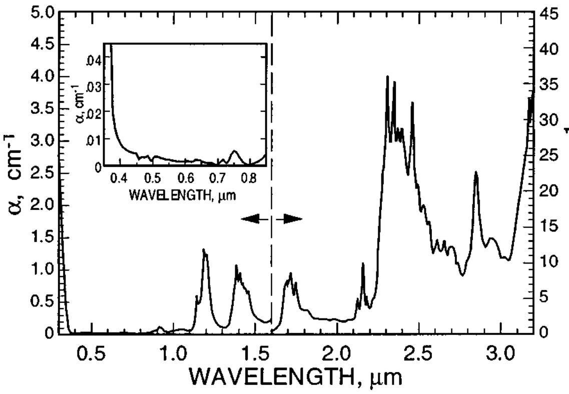

FIG. 3. Measured absorption coefficient of PCH-32 at $T\sim 22$ °C.

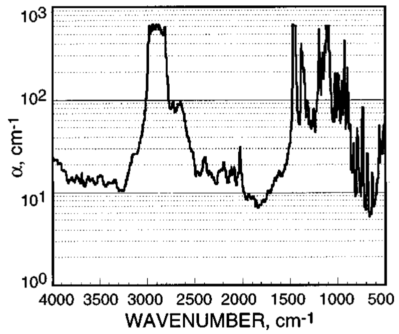

FIG. 5. Measured IR absorption coefficient of CCH-301 at $T\sim 22$ °C.

fundamental molecular vibration absorption bands which occur in the mid and long IR regions extend to near IR through their higher harmonics. As a result, a saddle absorption minimum occurs at a wavelength which is right before the overtone molecular vibration starts.

In the 0.9–2 μm region, some higher harmonics of the fundamental molecular vibration bands exist. This is manifested by two weak absorption bands located in the 0.9–1.1 μm region and three modest absorption bands centered at 1.2, 1.4 and 1.7 μm. For example, the 1.7 μm band is likely to originate from the second harmonic frequency of the hydrocarbon vibration centered at $\sim$3.4 μm.

From Figs. 2, 3, and 4, the two popular wavelengths ($\lambda = 1.3$ and 1.55 μm) for fiberoptic communications just happen to locate in two separate absorption valleys. Their absorption coefficients are found to be 0.08 and 0.13 cm^{-1}, respectively; not too sensitive to the LC structures investigated.

From 2.1 to 3.2 μm, several closely separated absorption bands appear. They overlap tightly so that the baseline absorption throughout this region is relatively large. The absorption band located at $\sim$2.4 μm consists of several spikes and its absorption is quite high ($\alpha \sim 30$ cm^{-1}). In the vicinity of 3.2 μm, the absorption is surging rapidly owing to the strong hydrocarbon vibrations.

In the visible region, the absorption is mainly influenced by the UV absorption tail. Therefore, both electronic transition wavelength and intensity make important contributions to the absorption in this region. A LC with a longer transition wavelength and higher intensity would undoubtedly lead to a larger absorption coefficient in the visible. This structural effect is clearly demonstrated in 5CB and PCH-32, as shown in the inlets of Figs. 3 and 4. For 5CB, its absorption decreases from 0.04 cm^{-1} at $\lambda = 0.4$ μm to below 0.005 cm^{-1} at $\lambda = 0.8$ μm. By contrast, the absorption of PCH-32 is kept below 0.005 cm^{-1} in most of the visible region. This is because 5CB has a longer λ_2 band and stronger transition intensity than that of PCH-32. The compound CCH-301 represents an exception. From Fig. 1, CCH-301 has the weakest UV absorption among the three LCs studied so that its absorption in the visible should be the lowest. However, from Fig. 2, CCH-301 possesses a surprisingly high absorption coefficient ($\alpha \sim 0.01$–0.02 cm^{-1}) in the visible. The detailed mechanism responsible for the observed anomaly is not yet completely understood.

V. MID TO LONG IR MEASUREMENTS

In this spectral region (>2.5 μm), there exist several vibration bands. We used a computer-controlled Nicolet 740 Fourier transform IR spectrophotometer for all these mid to long IR measurements. The substrates we used are 3 mm thick potassium chloride (KCl) windows. Its cutoff wavelength is $\sim$20μm, or 500 cm^{-1} in wave number. For the interest of obtaining absorption coefficient in some low absorption regions, we used a relatively thick ($\sim$150 μm) LC layer. The cell gap was controlled by a mylar spacer. To conveniently present data in such a wide spectral range, we use wave number, instead of wavelength.

Results of these measurements are shown in Figs. 5, 6,

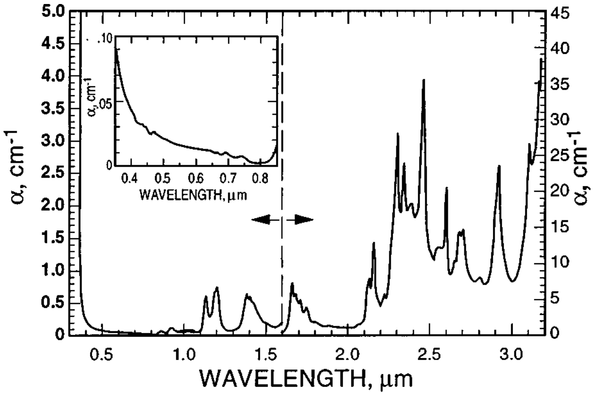

FIG. 4. Measured absorption coefficient of 5CB at $T\sim 50$ °C.

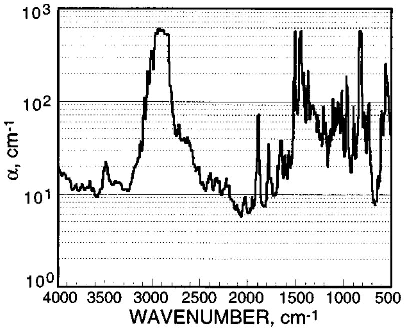

FIG. 6. Measured IR absorption coefficient of PCH-32 at $T\sim 22$ °C.

Downloaded 19 Jun 2012 to 139.184.30.132. Redistribution subject to AIP license or copyright; see http://jap.aip.org/about/rights_and_permissions

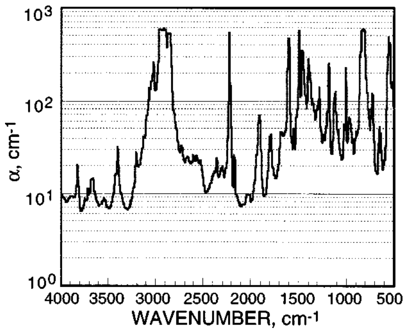

FIG. 7. Measured IR absorption coefficient of 5CB at $T\sim 50$ °C.

and 7 for CCH-301, PCH-32 and 5CB, respectively. The purpose of our study is to search for transparent regions where potential IR application can be realized. The assignments of some common absorption bands can be found in Refs. 9 and 12. The Nicolet and Lambda 9 spectrophotometers have an overlapped spectral region from 2.5 to 3.2 μm. In this common region, the measured absorption coefficients agree within 10% even though the instruments, substrates and LC sample thickness were all different.

From Figs. 5, 6, and 7, there are several overlapped and isolated absorption spikes. The well-known CH, CH_2 and CH_3 vibration bands overlap together to form a strong and broad absorption band ranging from 2700 to 3100 cm^{-1}. Its peak absorption coefficient exceeds 600 cm^{-1}. Owing to the thick LC layer used, the absorption coefficients of some absorption bands are saturated, as shown in Figs. 5, 6, and 7. To resolve the absorption coefficient of these strong vibration bands, a LC layer of about 5–10 μm has to be used. The absorption coefficient of the peak CH vibration band in 5CB has been measured previously and result is $\alpha\sim 0.3\ \mu\mathrm{m}^{-1}$.[10] As compared to the electronic absorption band of 5CB ($\alpha \sim 23\ \mu\mathrm{m}^{-1}$ at $\lambda_2 = 280$ nm), the transition intensity of the hydrocarbon vibration band is still ~8 times weaker.

Between 2500 and 1500 cm^{-1}, both CCH-301 and PCH-32 are relatively clear except for few weak absorption bands. The absorption coefficient in the vicinity of 2000 cm^{-1} is around 8 cm^{-1}. From Fig. 7, the strong CN stretching vibration of 5CB peaks at ~2225 cm^{-1} with about ± 15 cm^{-1} bandwidth. This narrow bandwidth implies that the CN stretching is localized; i.e., its influence to IR absorption does not extend too far.

From 1500 to 500 cm^{-1}, there are many spikes overlapping closely. Thus, the baseline absorption coefficient is raised to ~20 cm^{-1}. In some regions where the absorption bands are farther apart, the transparent windows become wider.

In some IR LC spatial light modulators developed for seeker simulation[13] and optical phased array for laser beam steering,[1] the LC thickness is usually kept below 10 μm in order to obtain fast response time. If the IR absorption coefficient is 20 cm^{-1}, the absorption loss is only 2%. However, if the application requires a centimeter long LC device, such as a wave guide, then the absorption loss would not be acceptable.

VI. CONCLUSION

The UV, visible, and IR absorption of three liquid crystal structures are evaluated. Both electronic transitions and molecular vibration absorption are all dependent on the detailed molecular structures. The UV absorption depends heavily on whether σ or π electrons are involved. On the other hand, the molecular vibrations depend on the specific molecular constituents. In the near IR region, the absorption resides on the tail of the electronic transitions and on the beginning of higher harmonic molecular vibrations. As a result, a saddle minimum occurs at around 0.7–0.8 μm region.

ACKNOWLEDGMENTS

The author is indebted to S. Anderson for useful discussion and L. Momoda, H. Olsen and W. H. Smith, Jr. for technical assistance.

[1] T. A. Dorschner, L. Friedman, M. Holz, D. P. Resler, R. C. Sharp, and I. W. Smith, Proceedings of the IEEE International Symposium on Phased-Array Systems and Technology, 15–18 October 1996, Boston, MA, p. 119.
[2] M. A. Karim, *Electro-optical Displays* (M. Dekker, New York, 1992).
[3] I. C. Khoo and R. Normandin, IEEE J. Quantum Electron. **QE21**, 329 (1985).
[4] H. H. Jaffe and M. Orchin, *Theory and Applications of Ultraviolet Spectroscopy* (Wiley, New York, 1962).
[5] S. T. Wu, J. Appl. Phys. **68**, 78 (1990).
[6] A. M. Lackner, J. D. Margerum, and C. I. VanAst, Mol. Cryst. Liq. Cryst. **141**, 289 (1986).
[7] P. G. de Gennes, *The Physics of Liquid Crystals* (Clarendon, Oxford, 1974).
[8] S. T. Wu and K. C. Lim, Appl. Opt. **26**, 1722 (1987).
[9] L. J. Bellamy, *The Infrared Spectra of Complex Molecules* (Wiley, New York, 1958).
[10] I. C. Khoo and S. T. Wu, *Optics and Nonlinear Optics of Liquid Crystals* (World Scientific, Singapore, 1993).
[11] S. T. Wu, J. Appl. Phys. **69**, 2080 (1991).
[12] P. K. Rajalakshmi, N. C. Shivaprakash, and J. S. Prasad, Mol. Cryst. Liq. Cryst. **60**, 311 (1980).
[13] R. Forber, A. Au, U. Efron, K. Sayyah, and S. T. Wu, Proc. SPIE **1665**, 259 (1992).

Downloaded 19 Jun 2012 to 139.184.30.132. Redistribution subject to AIP license or copyright; see http://jap.aip.org/about/rights_and_permissions
