# Supplementary material for: Advanced liquid crystal-based switchable optical devices for light protection applications: principles and strategies
Source: Light Sci Appl. 2023 Jan 3;12:11. doi: 10.1038/s41377-022-01032-y (PMC9807646; doi:10.1038/s41377-022-01032-y)
Supplement: Supplementary file 6 — Fig 6 copyright promotion [file 41377_2022_1032_MOESM6_ESM.pdf]

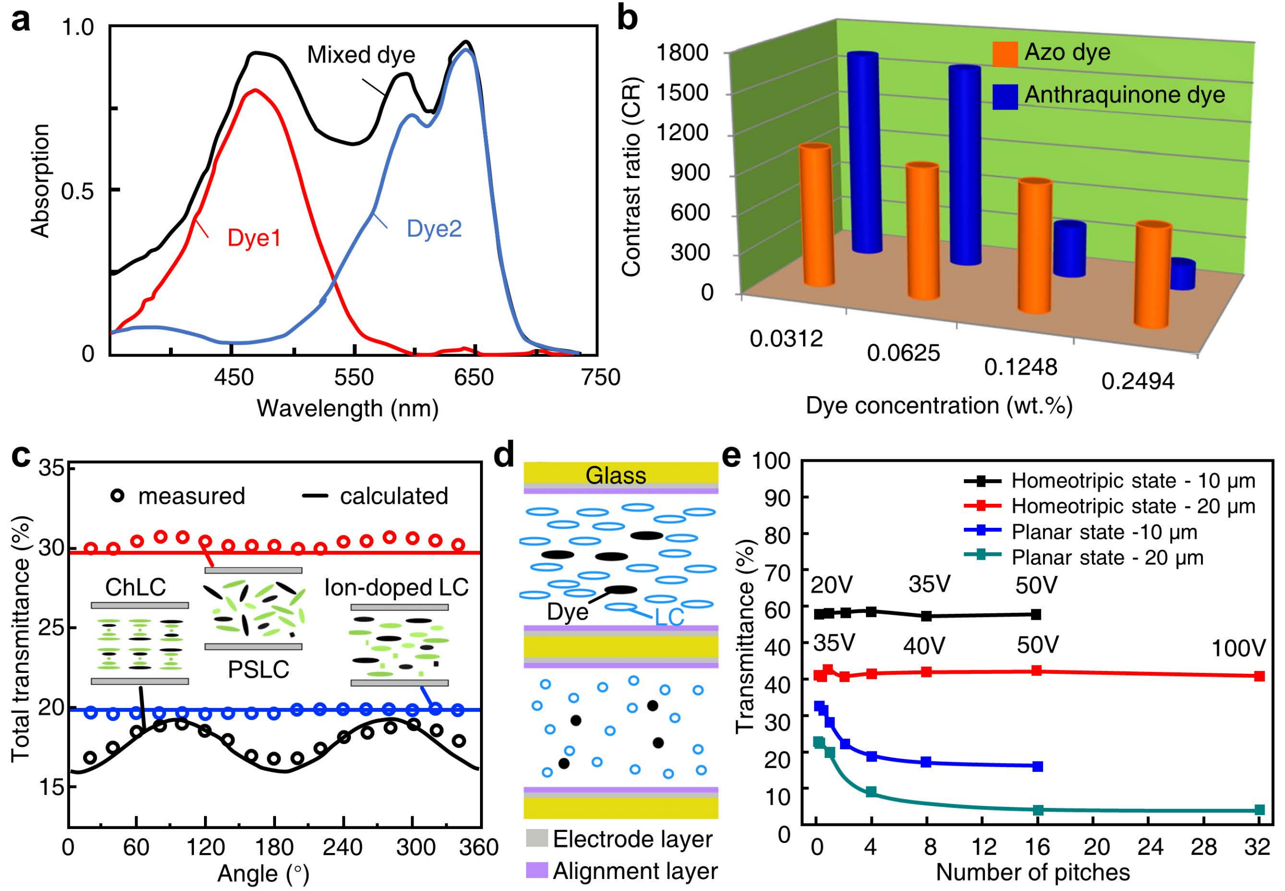
a
Mixed dye
Dye1
Dye2
Absorption
1.0
0.5
0
450
550
650
750
Wavelength (nm)
b
Azo dye
Anthraquinone dye
Contrast ratio (CR)
1800
1500
1200
900
600
300
0
0.0312
0.0625
0.1248
0.2494
Dye concentration (wt.%)
c
measured
calculated
ChLC
PSLC
Ion-doped LC
Total transmittance (%)
35
30
25
20
15
0
60
120
180
240
300
360
Angle (°)
d
Glass
Dye
LC
Electrode layer
Alignment layer
e
Homeotripic state - 10 μm
Homeotripic state - 20 μm
Planar state -10 μm
Planar state - 20 μm
20V
35V
50V
35V
40V
50V
100V
Transmittance (%)
100
90
80
70
60
50
40
30
20
10
0
0
4
8
12
16
20
24
28
32
Number of pitches

ELSEVIER LICENSE
TERMS AND CONDITIONS

Sep 20, 2022

This Agreement between Harbin Institute of Technology -- Ruicong Zhang ("You") and Elsevier ("Elsevier") consists of your license details and the terms and conditions provided by Elsevier and Copyright Clearance Center.

License Number	5392980397793
License date	Sep 20, 2022
Licensed Content Publisher	Elsevier
Licensed Content Publication	Journal of Molecular Liquids
Licensed Content Title	Studies on inter-dependency of electrooptic characteristics of orange azo and blue anthraquinone dichroic dye doped polymer dispersed liquid crystals
Licensed Content Author	Pankaj Kumar,Vandna Sharma,K.K. Raina
Licensed Content Date	Feb 1, 2018
Licensed Content Volume	251
Licensed Content Issue	n/a
Licensed Content Pages	10
Start Page	407
End Page	416
Type of Use	reuse in a journal/magazine
Requestor type	academic/educational institute
Portion	figures/tables/illustrations
Number of figures/tables/illustrations	1
Format	both print and electronic
Are you the author of this Elsevier article?	No
Will you be translating?	No
Title of new article	Advanced liquid crystal-based switchable optical devices for light protection applications: principles and strategies
Lead author	Ruicong Zhang, Zhibo Zhang, Jiecai Han, Lei Yang, Jiajun li, Zicheng Song Tianyu Wang, Jiaqi Zhu
Title of targeted journal	Light: Science & Applications
Publisher	Springer Nature
Expected publication date	Nov 2022
Portions	Figure 8
Requestor Location	Harbin Institute of Technology<br>No. 92, Xidazhi Street, Nangang District<br><br>Harbin, 150080<br>China<br>Attn: Harbin Institute of Technology
Publisher Tax ID	GB 494 6272 12
Total	**0.00 USD**

Terms and Conditions

INTRODUCTION

1. The publisher for this copyrighted material is Elsevier. By clicking "accept" in connection with completing this licensing transaction, you agree that the following terms and conditions apply to this transaction (along with the Billing and Payment terms

and conditions established by Copyright Clearance Center, Inc. ("CCC"), at the time that you opened your Rightslink account and that are available at any time at http://myaccount.copyright.com).

GENERAL TERMS

2. Elsevier hereby grants you permission to reproduce the aforementioned material subject to the terms and conditions indicated.
3. Acknowledgement: If any part of the material to be used (for example, figures) has appeared in our publication with credit or acknowledgement to another source, permission must also be sought from that source. If such permission is not obtained then that material may not be included in your publication/copies. Suitable acknowledgement to the source must be made, either as a footnote or in a reference list at the end of your publication, as follows:
"Reprinted from Publication title, Vol /edition number, Author(s), Title of article / title of chapter, Pages No., Copyright (Year), with permission from Elsevier [OR APPLICABLE SOCIETY COPYRIGHT OWNER]." Also Lancet special credit - "Reprinted from The Lancet, Vol. number, Author(s), Title of article, Pages No., Copyright (Year), with permission from Elsevier."
4. Reproduction of this material is confined to the purpose and/or media for which permission is hereby given.
5. Altering/Modifying Material: Not Permitted. However figures and illustrations may be altered/adapted minimally to serve your work. Any other abbreviations, additions, deletions and/or any other alterations shall be made only with prior written authorization of Elsevier Ltd. (Please contact Elsevier's permissions helpdesk here). No modifications can be made to any Lancet figures/tables and they must be reproduced in full.
6. If the permission fee for the requested use of our material is waived in this instance, please be advised that your future requests for Elsevier materials may attract a fee.
7. Reservation of Rights: Publisher reserves all rights not specifically granted in the combination of (i) the license details provided by you and accepted in the course of this licensing transaction, (ii) these terms and conditions and (iii) CCC's Billing and Payment terms and conditions.
8. License Contingent Upon Payment: While you may exercise the rights licensed immediately upon issuance of the license at the end of the licensing process for the transaction, provided that you have disclosed complete and accurate details of your proposed use, no license is finally effective unless and until full payment is received from you (either by publisher or by CCC) as provided in CCC's Billing and Payment terms and conditions. If full payment is not received on a timely basis, then any license preliminarily granted shall be deemed automatically revoked and shall be void as if never granted. Further, in the event that you breach any of these terms and conditions or any of CCC's Billing and Payment terms and conditions, the license is automatically revoked and shall be void as if never granted. Use of materials as described in a revoked license, as well as any use of the materials beyond the scope of an unrevoked license, may constitute copyright infringement and publisher reserves the right to take any and all action to protect its copyright in the materials.
9. Warranties: Publisher makes no representations or warranties with respect to the licensed material.
10. Indemnity: You hereby indemnify and agree to hold harmless publisher and CCC, and their respective officers, directors, employees and agents, from and against any and all claims arising out of your use of the licensed material other than as specifically authorized pursuant to this license.
11. No Transfer of License: This license is personal to you and may not be sublicensed, assigned, or transferred by you to any other person without publisher's written permission.
12. No Amendment Except in Writing: This license may not be amended except in a writing signed by both parties (or, in the case of publisher, by CCC on publisher's behalf).
13. Objection to Contrary Terms: Publisher hereby objects to any terms contained in any purchase order, acknowledgment, check endorsement or other writing prepared by you, which terms are inconsistent with these terms and conditions or CCC's Billing and Payment terms and conditions. These terms and conditions, together with CCC's Billing and Payment terms and conditions (which are incorporated herein), comprise the entire agreement between you and publisher (and CCC) concerning this licensing transaction. In the event of any conflict between your obligations established by these terms and conditions and those established by CCC's Billing and Payment terms and conditions, these terms and conditions shall control.
14. Revocation: Elsevier or Copyright Clearance Center may deny the permissions described in this License at their sole discretion, for any reason or no reason, with a full refund payable to you. Notice of such denial will be made using the contact information provided by you. Failure to receive such notice will not alter or invalidate the denial. In no event will Elsevier or Copyright Clearance Center be responsible or liable for any costs, expenses or damage incurred by you as a result of a denial of your permission request, other than a refund of the amount(s) paid by you to Elsevier and/or Copyright Clearance Center for denied permissions.

LIMITED LICENSE

The following terms and conditions apply only to specific license types:
15. **Translation**: This permission is granted for non-exclusive world **English** rights only unless your license was granted for translation rights. If you licensed translation rights you may only translate this content into the languages you requested. A professional translator must perform all translations and reproduce the content word for word preserving the integrity of the article.
16. **Posting licensed content on any Website**: The following terms and conditions apply as follows: Licensing material from an Elsevier journal: All content posted to the web site must maintain the copyright information line on the bottom of each image; A hyper-text must be included to the Homepage of the journal from which you are licensing at http://www.sciencedirect.com/science/journal/xxxxx or the Elsevier homepage for books at http://www.elsevier.com; Central Storage: This license does not include permission for a scanned version of the material to be stored in a central repository such as that provided by Heron/XanEdu.
Licensing material from an Elsevier book: A hyper-text link must be included to the Elsevier homepage at http://www.elsevier.com . All content posted to the web site must maintain the copyright information line on the bottom of each image.

Posting licensed content on Electronic reserve: In addition to the above the following clauses are applicable: The web site must be password-protected and made available only to bona fide students registered on a relevant course. This permission is granted for 1 year only. You may obtain a new license for future website posting.
17. **For journal authors:** the following clauses are applicable in addition to the above:
Preprints:
A preprint is an author's own write-up of research results and analysis, it has not been peer-reviewed, nor has it had any other value added to it by a publisher (such as formatting, copyright, technical enhancement etc.).
Authors can share their preprints anywhere at any time. Preprints should not be added to or enhanced in any way in order to appear more like, or to substitute for, the final versions of articles however authors can update their preprints on arXiv or RePEc with their Accepted Author Manuscript (see below).
If accepted for publication, we encourage authors to link from the preprint to their formal publication via its DOI. Millions of researchers have access to the formal publications on ScienceDirect, and so links will help users to find, access, cite and use the best available version. Please note that Cell Press, The Lancet and some society-owned have different preprint policies. Information on these policies is available on the journal homepage.
Accepted Author Manuscripts: An accepted author manuscript is the manuscript of an article that has been accepted for publication and which typically includes author-incorporated changes suggested during submission, peer review and editor-author communications.
Authors can share their accepted author manuscript:

- immediately
  - via their non-commercial person homepage or blog
  - by updating a preprint in arXiv or RePEc with the accepted manuscript
  - via their research institute or institutional repository for internal institutional uses or as part of an invitation-only research collaboration work-group
  - directly by providing copies to their students or to research collaborators for their personal use
  - for private scholarly sharing as part of an invitation-only work group on commercial sites with which Elsevier has an agreement
- After the embargo period
  - via non-commercial hosting platforms such as their institutional repository
  - via commercial sites with which Elsevier has an agreement

In all cases accepted manuscripts should:

- link to the formal publication via its DOI
- bear a CC-BY-NC-ND license - this is easy to do
- if aggregated with other manuscripts, for example in a repository or other site, be shared in alignment with our hosting policy not be added to or enhanced in any way to appear more like, or to substitute for, the published journal article.

Published journal article (JPA): A published journal article (PJA) is the definitive final record of published research that appears or will appear in the journal and embodies all value-adding publishing activities including peer review co-ordination, copy-editing, formatting, (if relevant) pagination and online enrichment.
Policies for sharing publishing journal articles differ for subscription and gold open access articles:
Subscription Articles: If you are an author, please share a link to your article rather than the full-text. Millions of researchers have access to the formal publications on ScienceDirect, and so links will help your users to find, access, cite, and use the best available version.
Theses and dissertations which contain embedded PJAs as part of the formal submission can be posted publicly by the awarding institution with DOI links back to the formal publications on ScienceDirect.
If you are affiliated with a library that subscribes to ScienceDirect you have additional private sharing rights for others' research accessed under that agreement. This includes use for classroom teaching and internal training at the institution (including use in course packs and courseware programs), and inclusion of the article for grant funding purposes.
Gold Open Access Articles: May be shared according to the author-selected end-user license and should contain a CrossMark logo, the end user license, and a DOI link to the formal publication on ScienceDirect.
Please refer to Elsevier's posting policy for further information.
18. **For book authors** the following clauses are applicable in addition to the above: Authors are permitted to place a brief summary of their work online only. You are not allowed to download and post the published electronic version of your chapter, nor may you scan the printed edition to create an electronic version. **Posting to a repository:** Authors are permitted to post a summary of their chapter only in their institution's repository.
19. **Thesis/Dissertation**: If your license is for use in a thesis/dissertation your thesis may be submitted to your institution in either print or electronic form. Should your thesis be published commercially, please reapply for permission. These requirements include permission for the Library and Archives of Canada to supply single copies, on demand, of the complete thesis and include permission for Proquest/UMI to supply single copies, on demand, of the complete thesis. Should your thesis be published commercially, please reapply for permission. Theses and dissertations which contain embedded PJAs as part of the formal submission can be posted publicly by the awarding institution with DOI links back to the formal publications on ScienceDirect.

Elsevier Open Access Terms and Conditions

You can publish open access with Elsevier in hundreds of open access journals or in nearly 2000 established subscription journals that support open access publishing. Permitted third party re-use of these open access articles is defined by the author's choice of Creative Commons user license. See our open access license policy for more information.

Terms & Conditions applicable to all Open Access articles published with Elsevier:

Any reuse of the article must not represent the author as endorsing the adaptation of the article nor should the article be modified in such a way as to damage the author's honour or reputation. If any changes have been made, such changes must be clearly indicated.

The author(s) must be appropriately credited and we ask that you include the end user license and a DOI link to the formal publication on ScienceDirect.

If any part of the material to be used (for example, figures) has appeared in our publication with credit or acknowledgement to another source it is the responsibility of the user to ensure their reuse complies with the terms and conditions determined by the rights holder.

Additional Terms & Conditions applicable to each Creative Commons user license:

CC BY: The CC-BY license allows users to copy, to create extracts, abstracts and new works from the Article, to alter and revise the Article and to make commercial use of the Article (including reuse and/or resale of the Article by commercial entities), provided the user gives appropriate credit (with a link to the formal publication through the relevant DOI), provides a link to the license, indicates if changes were made and the licensor is not represented as endorsing the use made of the work. The full details of the license are available at http://creativecommons.org/licenses/by/4.0.

CC BY NC SA: The CC BY-NC-SA license allows users to copy, to create extracts, abstracts and new works from the Article, to alter and revise the Article, provided this is not done for commercial purposes, and that the user gives appropriate credit (with a link to the formal publication through the relevant DOI), provides a link to the license, indicates if changes were made and the licensor is not represented as endorsing the use made of the work. Further, any new works must be made available on the same conditions. The full details of the license are available at http://creativecommons.org/licenses/by-nc-sa/4.0.

CC BY NC ND: The CC BY-NC-ND license allows users to copy and distribute the Article, provided this is not done for commercial purposes and further does not permit distribution of the Article if it is changed or edited in any way, and provided the user gives appropriate credit (with a link to the formal publication through the relevant DOI), provides a link to the license, and that the licensor is not represented as endorsing the use made of the work. The full details of the license are available at http://creativecommons.org/licenses/by-nc-nd/4.0. Any commercial reuse of Open Access articles published with a CC BY NC SA or CC BY NC ND license requires permission from Elsevier and will be subject to a fee.

Commercial reuse includes:

- Associating advertising with the full text of the Article
- Charging fees for document delivery or access
- Article aggregation
- Systematic distribution via e-mail lists or share buttons

Posting or linking by commercial companies for use by customers of those companies.

20. **Other Conditions**:

v1.10

Questions? customercare@copyright.com or +1-855-239-3415 (toll free in the US) or +1-978-646-2777.

Journal of Molecular Liquids 251 (2018) 407–416

Contents lists available at ScienceDirect

Journal of Molecular Liquids

journal homepage: www.elsevier.com/locate/molliq

Studies on inter-dependency of electrooptic characteristics of orange azo and blue anthraquinone dichroic dye doped polymer dispersed liquid crystals

Pankaj Kumar [a,b,*], Vandna Sharma [a,b], K.K. Raina [c]

[a] Chitkara University Research and Innovation Network, Chitkara University, Jhansla, Rajpura, Patiala, 140401, Punjab, India
[b] Department of Applied Sciences, Chitkara University, Jhansla, Rajpura, Patiala, 140401, Punjab, India
[c] DIT University, Mussoorie-Diversion Road, Dehradun, Uttarakhand -248009, India

ARTICLE INFO

Article history:
Received 26 October 2017
Received in revised form 14 December 2017
Accepted 19 December 2017
Available online 20 December 2017

Keywords:
Nematic liquid crystal
Polymer dispersed liquid crystal
Azo and anthraquinone dyes
Absorption
Contrast ratio

ABSTRACT

The optical properties of dyes dissolved in polymer dispersed liquid crystals (PDLCs) typically required fulfilling their absorption properties, degree of alignment and stability for practical applications. Reported herein is the absorption and electro optic behaviour of dye doped PDLCs prepared by equal weight ratio of nematic liquid crystal (LC) and photo-curable polymer (NOA 65) with different weight concentrations (0.0312, 0.0625, 0.1248, and 0.2494%) of azo and anthraquinone dyes. Considering Beer's law, OFF state absorption coefficients were measured followed by dyes' extinction coefficients. Higher absorption coefficient of azo dye doped PDLCs with dye concentrations (0.1248 and 0.2494%) resulted in superior contrast ratio (CR) as compared with anthraquinone dye doped PDLCs. However, at lower concentrations (0.0312 and 0.0625%) of anthraquinone dye, ON state transmittance of dye doped PDLCs is more dominating which resulted in higher CR at these concentrations. The experimental results were found be consistent with the theoretical results. In addition, inter-dependency among transmission, absorption coefficient, CR, applied voltage and response time characteristics of azo and anthraquinone dye doped PDLCs have been discussed and analyzed.

© 2017 Elsevier B.V. All rights reserved.

1. Introduction

Polymer dispersed liquid crystals (PDLCs) with unique characteristics can be obtained by modifying the microstructures of polymer/liquid crystal (LC) composites [1–10]. PDLCs are fundamentally prepared by dispersing LC droplets in a polymer matrix. Various tools/techniques, methods and doping materials like nano-particles, surfactants as well as different dyes in PDLCs [11–14] have been used for their intensifying advancement. Studies of azo dyes and anthraquinone dyes dominate particularly in the context of display applications. The earliest explanation on PDLCs window by incorporating a fluorescent dye in LC host was reported in 1968 [15].These kinds of windows were potentially enables light absorbed in their absorbing state and used for solar energy conversion [16]. Also, the chiral nematic LCs with the dye molecules potentially used for rigid and flexible transparent displays [17–22], light shutters [23], privacy and smart windows [24], switching gratings [25], sensors [26], microlenses [27], lasers [28], smart food packaging [29], electrically switchable high-fold-helix spiral phase plates [30] and biomedical devices [31–33]. Former studies also notified that anthraquinone dyes are more attractive due to their superior stability and light-fastness properties [34,35]. However, the molecular structure of the anthraquinone chromophore does not lend itself as readily to creating rod-like structures as the azo chromophore does. Thus, azo dyes resulted in the high degree of alignment and a wide range of colours displays [36]. Characteristically, in PDLCs dye dissolved mainly in the LC within droplets exhibits dichroic properties and enhances their characteristics [36–40]. In their OFF state, the symmetry axes of the bipolar droplets are assumed randomly oriented with the haphazard orientation of the dye molecules. Moreover, in the ON state, the droplet director and dye molecules in the droplet are aligned normal to the surface of the film and resulted in enhanced transmission [12,13]. Consequently, in this paper considering the Beer's law, we have established absorbance based theoretical inference of PDLCs doped with two different dyes namely orange azo and blue anthraquinone. The morphological and electrooptic results of both dye doped systems are compared and analyzed for display application.

2. Theory of absorbance in dye doped PDLCs

Absorption of light due to the azo/anthraquinone dye molecules in PDLC effects the transmission and scattering properties, as dye molecules within LC droplets can be switched. When the polarization of the

* Corresponding author at: Chitkara University Research and Innovation Network, Chitkara University, Jhansla, Rajpura, Patiala, 140401, Punjab, India.
E-mail address: pankaj.kumar@chitkara.edu.in (P. Kumar).

https://doi.org/10.1016/j.molliq.2017.12.095
0167-7322/© 2017 Elsevier B.V. All rights reserved.

incident light is parallel to the long axis of the dye molecules, the light is strongly absorbed whereas light is weakly absorbed by the dye molecules when the polarized light is perpendicular to the long axis of the dye molecules. Now, theoretically for the OFF-state transmittance of azo/anthraquinone dye doped PDLCs and conventional PDLC, the parameter extinction coefficient (γ), according to Beer's law [41] is given by:

$$\gamma = \alpha + \beta \tag{1}$$

where α and β represent the scattering and absorption coefficient of the film, respectively.

The OFF-state transmittance (T) can be written as:

$$T = \frac{I_t}{I_o} = e^{-(\alpha+\beta)d} \tag{2}$$

where I_t *and* I_o are the transmitted and incident light intensities respectively, and d is the sample thickness. α is a function of the LC droplet size and curing temperature. Since visible light is not absorbed notably by polymer and LC molecules, therefore absorption of a PDLC film results from dye molecules only. Thus, the β is given by

$$\beta = \varepsilon x l \tag{3}$$

where ε *and* x are the dye extinction coefficient and dye concentration, respectively. The symbol$l$stands for the ratio of the distance travelled by light through the film thickness (d). For normal incidence, $l > 1$ because the scattering and where the scattering effect is small $l \approx 1$ (for thin film). Then, again using Beer's law, the OFF-state transmittance of the PDLC film will be

$$T = \frac{I_t}{I_o} = e^{-(\gamma d)} \tag{4}$$

Thus, in line with theoretical consideration, the dye extinction coefficient can also be analyzed by experimental value of OFF-state transmittance and vice versa using Eq. (4). As, in conventional PDLC droplets axes are randomly and uniformly directed along the three dimensional xyz-space. For instance in our case, let the extinction coefficient of the conventional PDLC be γ_1 and is independent of the polarization direction of the incident light. Now, we let here the extinction coefficients of dye doped PDLC are $\gamma_{1\|}$and $\gamma_{1\perp}$. Here, $\gamma_{1\|}$ *and* $\gamma_{1\perp}$ are the extinction coefficients parallel to the long and minor axes respectively of a dye molecule of bipolar LC droplets.

Generally, for scattering effect in PDLC, the LC director varies randomly from droplet to droplet and the ordinary (n_o) as well as extra-ordinary (n_e) refractive indices of LC do not match with polymer refractive index (n_p). For a LC droplet of bipolar structure, most of the LC directors have small angles with respect to the droplet axis. Thus in case $n_e > n_o \approx n_p$, the average LC droplet refractive index experienced by the incident light with polarization direction parallel to the droplet axis is larger than that experienced by the incident light with polarization direction perpendicular to the droplet axis. Obviously, the scattering coefficient $\alpha_{1\|}$ is larger than$\alpha_{1\perp}$. Similarly, for dye doped PDLC with positive dichroic dye molecules and bipolar structure of LC droplets, the absorption coefficients $\beta_{1\|}$ are larger than $\beta_{1\perp}$. There are different cases be arisen for our experimental results intended for dye doped PDLCs as per dye concentrations.

Case I. Consider a state in which the LC droplet axes are distributed in a three or one dimensional space, a simple relationship between the extinction coefficients γ_1, $\gamma_{1\|}$ and $\gamma_{1\perp}$ can be expressed as [41].

$$\gamma_1 \approx \frac{\gamma_{1\|} + 2\gamma_{1\perp}}{3} \approx 0.333\gamma_{1\|} + 0.667\gamma_{1\perp} \text{ and } \gamma_{1\|} > \gamma_1 > \gamma_{1\perp} \tag{5}$$

Case II. Again, there will be two sub cases (A_1 and B_1) for a similar concentration of anthraquinone and azo dye.

A_1. Consider a state in which the nematic LC droplet are doped with 0.0312% anthraquinone dye, the absorption coefficient $\beta_{21\|}$ is larger than $\beta_{21\perp}$ due to the more absorption parallel to the major axis of dopant, then

$$\gamma_{21} \approx \frac{\gamma_{21\|} + 2\gamma_{21\perp}}{3} \text{ and } \gamma_{21\|} > \gamma_{21} > \gamma_{21\perp} \tag{6}$$

Here, γ_{21} is the extinction coefficient of 0.0312% anthraquinone dye doped PDLC. $\gamma_{21\|}$ and $\gamma_{21\perp}$ are the extinction coefficients along the major and minor axis of anthraquinone dye molecule.

From Eqs. (5) and (6).

$$\gamma_{21\|} > \gamma_{1\|},\ \gamma_{21\perp} > \gamma_{1\perp} \text{ and } \gamma_{21} > \gamma_1 \tag{7}$$

$$\gamma_{21\|} > \gamma_{1\|} > \gamma_1 \textit{ and } \gamma_{21\|} > \gamma_{21\perp} > \gamma_{1\perp} \tag{8}$$

From the above inequality, we can find parallel and perpendicular component of γ_{21} extinction coefficient as following:

$\gamma_{21\|} > \gamma_{1\|} > \gamma_1$, now using Eq. (5),

$$\gamma_{1\|} \approx \frac{\gamma_{21\|} + 2\gamma_1}{3} \text{ or } \gamma_{21\|} \approx 2.33\gamma_{1\|} - 1.33\gamma_{1\perp} \tag{9}$$

From inequality (8), $\gamma_{21\|} > \gamma_{21\perp} > \gamma_{1\perp}$, then using Eqs. (5) and (9) we have.

$$\gamma_{2\perp} \approx \frac{\gamma_{21\|} + 2\gamma_{1\perp}}{3} \text{ or } \gamma_{21\perp} \approx 0.777\gamma_{1\|} - 0.223\gamma_{1\perp} \tag{10}$$

On solving Eqs. (6), (9) and (10)

$$\gamma_{21} \approx 1.296\gamma_{1\|} - 0.295\gamma_{1\perp} \tag{11}$$

B_1. Consider a state in which the nematic LC droplet are doped with 0.0312% azo dye, the absorption coefficients $\beta_{31\|}$ is larger than $\beta_{31\perp}$, then.

$$\gamma_{31} \approx \frac{\gamma_{31\|} + 2\gamma_{31\perp}}{3} \text{ and } \gamma_{31\|} > \gamma_{31} > \gamma_{31\perp} \tag{12}$$

Here, γ_{31} is the extinction coefficient of 0.0312% azo dye doped PDLC. $\gamma_{31\|}$ and $\gamma_{31\perp}$ are the extinction coefficients along the major and minor axis of azo dye molecule.

From Eqs. (5), (6) and (12).

$$\gamma_{31\|} > \gamma_{21\|} > \gamma_{1\|},\ \gamma_{31\perp} > \gamma_{21\perp} > \gamma_{1\perp} \text{ and } \gamma_{31} > \gamma_{21} > \gamma_1 \tag{13}$$

$$\gamma_{31\|} > \gamma_{21\|} > \gamma_{1\|} > \gamma_{21} > \gamma_1 \textit{ and } \gamma_{31\|} > \gamma_{31\perp} > \gamma_{21\perp} > \gamma_{1\perp} \tag{14}$$

From the above inequality, we can find parallel and perpendicular component of γ_{31} extinction coefficient as following:

$\gamma_{31\|} > \gamma_{21\|} > \gamma_{21}$, Again using Eq. (5) we have

$$\gamma_{21\|} \approx \frac{\gamma_{31\|} + 2\gamma_{21}}{3}, \text{ or } \gamma_{31\|} \approx 5.296\gamma_{1\|} - 4.296\gamma_{1\perp} \tag{15}$$

$\gamma_{31\|} > \gamma_{31\perp} > \gamma_{21\perp}$, now using Eqs. (5) and (15),

$$\gamma_{31\perp} \approx \frac{\gamma_{31\|} + 2\gamma_{21\perp}}{3.} \text{ or } \gamma_{21\perp} \approx 2.283\gamma_{1\|} - 1.283\gamma_{1\perp} \tag{16}$$

On solving Eqs. (12), (15) and (16)

$$\gamma_{31} \approx 3.287\gamma_{1\|} - 2.287\gamma_{1\perp} \quad (17)$$

Case III. Let us consider this case for a higher concentration of dyes and in similar way there will be two sub cases (A_2 and B_2).

A_2. Consider a state in which the nematic LC droplet are doped with 0.0625% anthraquinone dye, the absorption coefficients $\beta_{22\|}$ is larger than $\beta_{22\perp}$, then

$$\gamma_{22} \approx \frac{\gamma_{22\|} + 2\gamma_{22\perp}}{3} \text{ and } \gamma_{22\|} > \gamma_{22} > \gamma_{22\perp} \quad (18)$$

Here, γ_{22} is the extinction coefficient of 0.0625% anthraquinone dye doped PDLC. $\gamma_{22\|}$ and $\gamma_{22\perp}$ are the extinction coefficients along the major and minor axis of anthraquinone dye molecule.

From Eqs. (5), (6), (12) and (18)

$$\gamma_{22\|} > \gamma_{31\|} > \gamma_{21\|} > \gamma_{1\|}, \gamma_{22\perp} > \gamma_{31\perp} > \gamma_{21\perp} > \gamma_{1\perp} \text{ and } \gamma_{22} > \gamma_{31} > \gamma_{21} > \gamma_{1} \quad (19)$$

$$\gamma_{22\|} > \gamma_{31\|} > \gamma_{21\|} > \gamma_{1\|} > \gamma_{31} > \gamma_{21} > \gamma_{1} \textit{ and } \gamma_{22\|} > \gamma_{22\perp} > \gamma_{31\perp} > \gamma_{21\perp} > \gamma_{1\perp} \quad (20)$$

From the above inequality, we can find parallel and perpendicular component of γ_{21} extinction coefficient as following:

$\gamma_{22\|} > \gamma_{31\|} > \gamma_{31}$, now using Eq. (5),

$$\gamma_{31\|} \approx \frac{\gamma_{22\|} + 2\gamma_{31}}{3} \text{ or } \gamma_{22\|} \approx 9.314\gamma_{1\|} - 8.314\gamma_{1\perp} \quad (21)$$

As per inequality (18) $\gamma_{22\|} > \gamma_{22\perp} > \gamma_{31\perp}$, Eqs. (5) and (21) give

$$\gamma_{22\perp} \approx \frac{\gamma_{22\|} + 2\gamma_{31\perp}}{3} \text{ or } \gamma_{22\perp} \approx 4.627\gamma_{1\|} - 3.627\gamma_{1\perp} \quad (22)$$

On solving Eqs. (18), (21) and (22)

$$\gamma_{22} \approx 6.189\gamma_{1\|} - 5.189\gamma_{1\perp} \quad (23)$$

B_2. Consider a state in which the nematic LC droplet are doped with 0.0625% azo dye, the absorption coefficients$\beta_{32\|}$ is larger than $\beta_{32\perp}$ due to the more absorption parallel to the major axis of dopant, then

$$\gamma_{32} \approx \frac{\gamma_{32\|} + 2\gamma_{32\perp}}{3} \text{ and } \gamma_{32\|} > \gamma_{32} > \gamma_{32\perp} \quad (24)$$

Here, γ_{32} is the extinction coefficient of 0.0625% azo dye doped PDLC. $\gamma_{32\|}$ and $\gamma_{32\perp}$ are the extinction coefficients along the major and minor axis of azo dye molecule.

From Eqs. (5), (6), (12), (18) and (24)

$$\gamma_{32\|} > \gamma_{22\|} > \gamma_{31\|} > \gamma_{21\|} > \gamma_{1\|}, \gamma_{32\perp} > \gamma_{22\perp} > \gamma_{31\perp} > \gamma_{21\perp} > \gamma_{1\perp} \text{and } \gamma_{32} > \gamma_{22} > \gamma_{31} > \gamma_{21} > \gamma_{1} \quad (25)$$

$$\gamma_{32\|} > \gamma_{22\|} > \gamma_{31\|} > \gamma_{21\|} > \gamma_{1\|} > \gamma_{22} > \gamma_{31} > \gamma_{21} > \gamma_{1} \text{and } \gamma_{32\|} > \gamma_{32\perp} > \gamma_{22\perp} > \gamma_{31\perp} > \gamma_{21\perp} > \gamma_{1\perp} \quad (26)$$

From the above inequality, we can find parallel and perpendicular component of γ_{32} extinction coefficient as following:

$\gamma_{32\|} > \gamma_{22\|} > \gamma_{22}$, using Eq. (5)

$$\gamma_{22\|} \approx \frac{\gamma_{32\|} + 2\gamma_{22}}{3} \text{ or } \gamma_{32\|} \approx 15.564\gamma_{1\|} - 14.564\gamma_{1\perp} \quad (27)$$

$\gamma_{32\|} > \gamma_{32\perp} > \gamma_{22\perp}$, using Eqs. (5) and (27), $\gamma_{32\perp} \approx \frac{\gamma_{32\|} + 2\gamma_{22\perp}}{3}$

$$\gamma_{32\perp} \approx 8.273\gamma_{1\|} - 7.273\gamma_{1\perp} \quad (28)$$

On solving Eqs. (24), (27) and (28)

$$\gamma_{32} \approx 10.70\gamma_{1\|} - 10.37\gamma_{1\perp} \quad (29)$$

On generalizing, for other cases, extinction coefficients γ_{23} *and* γ_{33} are at 0.1248% as well as γ_{24} *and* γ_{34} are at 0.2494% for azo and anthraquinone dye doped PDLCs respectively, can be calculated in the same manner and given by

$$\gamma_{23} \approx 17.73\gamma_{1\|} - 15.98\gamma_{1\perp} \quad (30)$$

$$\gamma_{33} \approx 28.65\gamma_{1\|} - 26.05\gamma_{1\perp} \quad (31)$$

$$\gamma_{24} \approx 45.65\gamma_{1\|} - 41.71\gamma_{1\perp} \quad (32)$$

$$\gamma_{34} \approx 72.089\gamma_{1\|} - 66.07\gamma_{1\perp} \quad (33)$$

In practice, when the incident light is natural light, the OFF-state transmittances of the PDLC films using Eq. (4), can be expressed as

$$T = \begin{cases} T_1 \approx \exp(-\gamma_1 d), & \textit{for a conventional PDLC} \\ T_2 \approx \exp(-\gamma_{21} d), & \textit{for } 0.0312\%\text{anthraquinone } \textit{dye dopedPDLC} \\ T_3 \approx \exp(-\gamma_{31} d), & \textit{for } 0.0312\%\textit{azo dye dopedPDLC} \\ T_4 \approx \exp(-\gamma_{22} d), & \textit{for } 0.0625\%\text{anthraquinone } \textit{dye dopedPDLC} \\ T_5 \approx \exp(-\gamma_{32} d), & \textit{for } 0.0625\%\textit{azo dye dopedPDLC} \\ T_6 \approx \exp(-\gamma_{23} d), & \textit{for } 0.1248\%\text{anthraquinone } \textit{dye dopedPDLC} \\ T_7 \approx \exp(-\gamma_{33} d), & \textit{for } 0.1248\%\textit{azo dye dopedPDLC} \\ T_8 \approx \exp(-\gamma_{24} d), & \textit{for } 0.2494\%\text{anthraquinone } \textit{dye dopedPDLC} \\ T_9 \approx \exp(-\gamma_{34} d), & \textit{for } 0.2494\%\textit{azo dye dopedPDLC} \end{cases} \quad (34)$$

Using Eqs. (5), (11), (17), (23), (29), (30), (31), (32), (33) and (34):

$$T = \begin{cases} T_1 \approx \exp\left[-\left(0.333\gamma_{1\|} + 0.667\gamma_{1\perp}\right)d\right], & \textit{for a conventional PDLC} \\ T_2 \approx \exp\left[-\left(1.296\gamma_{1\|} - 0.295\gamma_{1\perp}\right)d\right], & \textit{for } 0.0312\%\text{anthraquinone } \textit{dye dopedPDLC} \\ T_3 \approx \exp\left[-\left(3.287\gamma_{1\|} - 2.287\gamma_{1\perp}\right)d\right], & \textit{for } 0.0312\%\textit{azo dye dopedPDLC} \\ T_4 \approx \exp\left[-\left(6.189\gamma_{1\|} - 5.189\gamma_{1\perp}\right)d\right], & \textit{for } 0.0625\%\text{anthraquinone } \textit{dye dopedPDLC} \\ T_5 \approx \exp\left[-\left(10.70\gamma_{1\|} - 10.37\gamma_{1\perp}\right)d\right], & \textit{for } 0.0625\%\textit{azo dye dopedPDLC} \\ T_6 \approx \exp\left[-\left(17.73\gamma_{1\|} - 15.98\gamma_{1\perp}\right)d\right], & \textit{for } 0.1248\%\text{anthraquinone } \textit{dye dopedPDLC} \\ T_7 \approx \exp\left[-\left(28.65\gamma_{1\|} - 26.05\gamma_{1\perp}\right)d\right], & \textit{for } 0.1248\%\textit{azo dye dopedPDLC} \\ T_8 \approx \exp\left[-\left(45.65\gamma_{1\|} - 41.71\gamma_{1\perp}\right)d\right], & \textit{for } 0.2494\%\text{anthraquinone } \textit{dye dopedPDLC} \\ T_9 \approx \exp\left[-\left(72.089\gamma_{1\|} - 66.07\gamma_{1\perp}\right)d\right] & \textit{for } 0.2494\%\textit{azo dye dopedPDLC} \end{cases} \quad (35)$$

The fact that the fabricating conditions are all the same except the doping of dye molecules which are accounts for why the ON-state transmittances of these PDLC films are expected to have the same value. However, when the incident light is linearly polarized with its polarization direction parallel to the absorbance axis of dye molecule, as well as in case that the scattering coefficient $\gamma_{1\perp} \rightarrow 0$, the OFF-state transmittances

of the PDLC films can be expressed as

$$T=\begin{cases} T_1 \approx \exp\left[-\left(0.333\gamma_{1\|}\right)d\right], & \text{for a conventional PDLC} \\ T_2 \approx \exp\left[-\left(1.296\gamma_{1\|}\right)d\right], & \text{for } 0.0312\%\text{anthraquinone dye dopedPDLC} \\ T_3 \approx \exp\left[-\left(3.287\gamma_{1\|}\right)d\right], & \text{for } 0.0312\%\text{azo dye dopedPDLC} \\ T_4 \approx \exp\left[-\left(6.189\gamma_{1\|}\right)d\right], & \text{for } 0.0625\%\text{anthraquinone dye dopedPDLC} \\ T_5 \approx \exp\left[-\left(10.70\gamma_{1\|}\right)d\right], & \text{for } 0.0625\%\text{azo dye dopedPDLC} \\ T_6 \approx \exp\left[-\left(17.73\gamma_{1\|}\right)d\right], & \text{for } 0.1248\%\text{anthraquinone dye dopedPDLC} \\ T_7 \approx \exp\left[-\left(28.65\gamma_{1\|}\right)d\right], & \text{for } 0.1248\%\text{azo dye dopedPDLC} \\ T_8 \approx \exp\left[-\left(45.65\gamma_{1\|}\right)d\right], & \text{for } 0.2494\%\text{anthraquinone dye dopedPDLC} \\ T_9 \approx \exp\left[-\left(72.089\gamma_{1\|}\right)d\right], & \text{for } 0.2494\%\text{azo dye dopedPDLC} \end{cases} \tag{36}$$

Thus according to our theoretical consideration based on Beer's law, Eq. (36) gives the minimum OFF state transmission (T_2 to T_9) for azo and anthraquinone dye doped PDLC with 0.0312, 0.0625, 0.1248 and 0.2494% of dye concentrations. Eq. (36) explicitly shows that the azo dye doped PDLCs have the lower OFF-state transmittance as compared with anthraquinone dye doped PDLCs at the same concentrations, thereby azo dye doped PDLCs must lead to the higher contrast ratio (CR). As a result the theoretical calculation replicated the experimental consequence of higher β value as shown in Fig. 5 for azo and anthraquinone dye doped PDLCs. Eq. (36) is the extreme case under the assumption $\gamma_{1\perp} \to 0$. However, in an actual case, $\gamma_{1\perp}$ is not small enough to be negligible. In addition, for a conventional PDLC film, LC molecules are inevitably aligned with some degree of order because the material should be spread from one direction into the sample cell. Therefore, the extinction coefficient γ_1 is anisotropic and must be expressed as $\gamma_{1\|}$ and $\gamma_{1\perp}$.

3. Experimental specifications

3.1. Materials

3.1.1. Nematic LC and polymer

A room temperature nematic E7 LC mixture (Merck, UK) [42], of components 5CB, 7CB, 8OCB and 5CT, of refractive indices ($n_o = 1.525$, $n_e = 1.736$) with nematic phase up to 60°C and an ultraviolet (UV) curable liquid photopolymer NOA 65 (NORLAND, NJ) [43] of refractive index ($n_o = 1.524$) were used as underpinning for dye doped PDLCs. Fig. 1 (a-d) shows the chemical structure of components (5CB, 7CB, 8OCB and 5CT) of commercially available nematic E7 LC mixture.

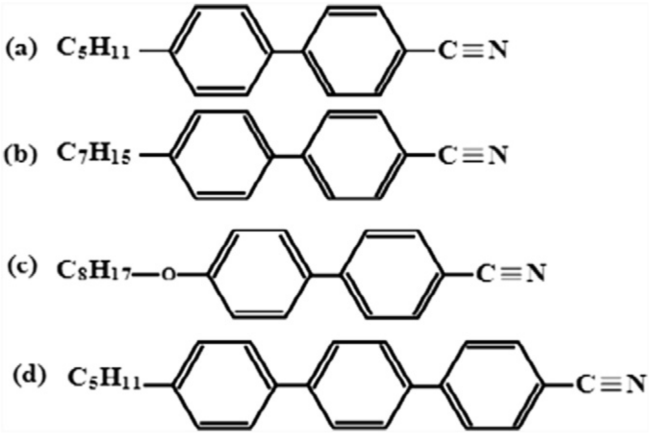

Fig. 1. (a-d) Chemical structure of components (5CB, 7CB, 8OCB and 5CT) of nematic LC E7 mixture (from Merck).

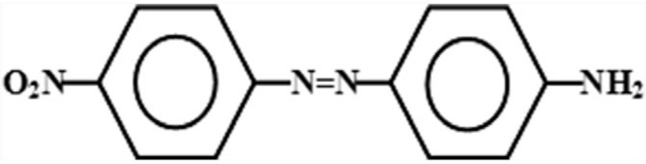

Fig. 2. Chemical structure of azo dye.

3.1.2. Azo and anthraquinone dichroic dyes

Azo dye containing the azo (–N=N–) linkage group as shown in Fig. 2 is the prevalent class of commercial dyes and produce coloured compounds [34]. Azo dyes have optical and alignment properties as well as high solubility in LC hosts [35,44,45], which made easy to devices to exhibit intense colours and high-contrast ratios. The chemical structure of azo dye used in experiment is shown in Fig. 2.

Anthraquinone dyes are another important class of commercial dyes, and they are based upon the fused ring chromophore as shown in Fig. 3. However, typically the colours of anthraquinone dyes are weaker than those of azo dyes [34].

3.2. Compositions and preparation of dye doped PDLCs

Four different compositions of dyes were taken for preparing dye doped PDLCs. These doped PDLCs were prepared by polymerization induced phase separation (PIPS) process taking the nematic LC E7 mixture and liquid photopolymer NOA65 in equal (wt./wt.) ratio. Hereafter different concentrations (wt./wt. ratio) of orange azo and blue anthraquinone dichroic dyes, particularly 0.0312, 0.0625, 0.1248 and 0.2494% of the LC-monomer mixture, were dissolved with nematic LC mixture. Subsequently, these compositions were mixed into the polymer material and ensured homogenous mixing by shaken and heated simultaneously to an isotropic temperature.

Several dye doped PDLC cells of 10 μm thicknesses were prepared using indium tin oxide (ITO) coated glass substrates. The cell gap was controlled by a polymeric mylar spacer. Subsequently, cells were filled with various mixtures for dye doped PDLC materials through capillary action by heating the material to its isotropic temperature. It is worth to be perceived that, the proportion of LC, polymer and dye filled into the different cells will be [49.98% + 49.98% + 0.0312%], [49.97% + 49.97% + 0.0625%], [49.94% + 49.94% + 0.1248%] and [49.88% + 49.88% + 0.2494%] for compositions 1, 2, 3 and 4 respectively, as shown in Fig. 4. These cells were sealed and exposed to UV light (intensity ~2 mW/cm^2) for an hour at room temperature to make dye doped PDLC films.

3.3. Apparatus employed

The morphological study of dye doped PDLC cells were carried out using a polarizing optical microscope (POM) (Olympus model BX51P), fitted with charge-coupled device (CCD) camera interfaced to a computer system for recording and data acquisition. The samples' temperature was controlled to an accuracy of 0.1 °C using a programmable temperature controller and hot stage (LINKAM Model TP94 and THMS600). The EO responses were studied under an applied electric field using a function generator (Model- Philips FG −8002) and a photo-multiplier tube (Model

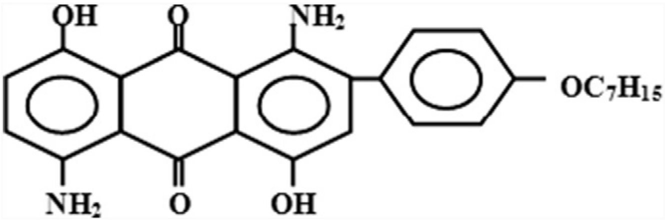

Fig. 3. Chemical structure of anthraquinone dye.

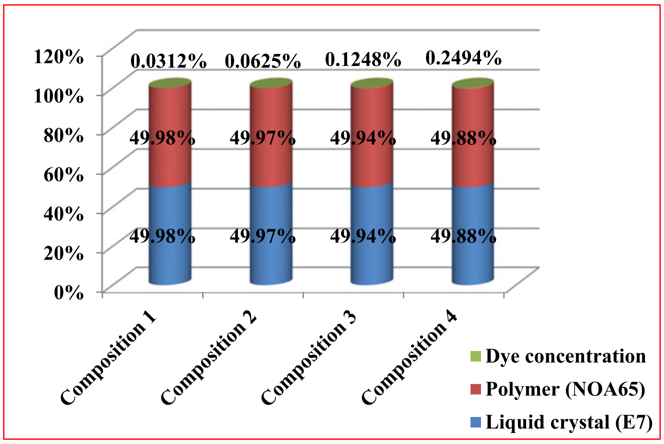

Fig. 4. Absolute concentrations of samples filled in to different dye doped PDLC cells.

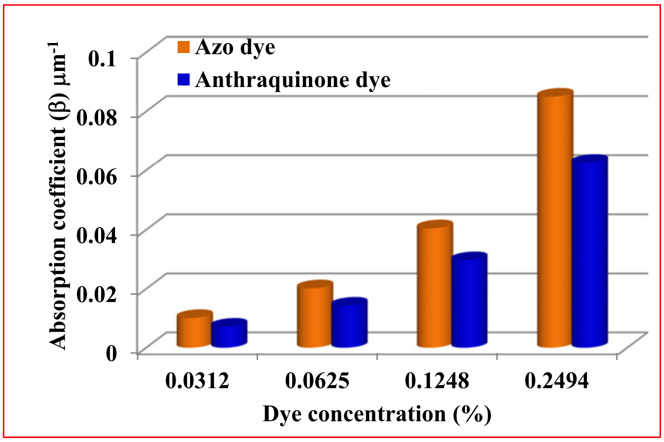

Fig. 5. Absorption coefficient of azo and anthraquinone dyes at concentrations of 0.0312, 0.0625, 0.1248 and 0.2494%.

RCA 931-A). The data was acquired in the computer interfaced with digital storage Oscilloscope (Model-Tektronix Model TDS 2024).

4. Results and discussion

4.1. Absorption coefficient vs. dye concentrations

The OFF state β of the dye doped PDLCs were calculated using experimentally observed OFF state transmittance by Eqs. (3) and (4). Here, the dopants' concentrations were taken 0.0312, 0.0625, 0.1248 and 0.2494% and the ratio of the distance travelled by light through the film thickness (d) was considered unity. Fig. 5 shows the calculated β values of PDLCs doped with azo/anthraquinone dichroic dyes for these concentrations. As shown in Fig. 5, the β was found higher for azo dye doped PDLCs as compared with anthraquinone dye doped PDLCs with respect to their corresponding dye concentrations. The higher absorbance values (~26–30%) may possibly be due to higher solubility, better optical and alignment properties of azo dyes in nematic LC host [35,45].

4.2. Absorption coefficient vs. applied voltage

The effect of applied voltage on the β of azo and anthraquinone dye doped PDLCs for 0.0312, 0.0625, 0.1248 and 0.2494% dye concentrations with white light illuminated has been measured and shown in Fig. 6 (a, b). With the application of voltage, the random motion of major axis (optical axis) LC molecules and longer axis (absorption axis) of associated dye molecules tend to be in the direction of external applied voltage. For higher fields, the decrease in the absorption itself affirms higher-order alignment of azo/anthraquinone dye-LC systems in the direction of electric field [46]. Thus at higher voltages most of the LC droplets are aligned and marginal change was observed in the absorption values. The experimental results again reveal the higher β in case of orange azo dichroic dye doped PDLC than of anthraquinone dye doped PDLC in OFF state and follow the same manner from OFF to ON state up to saturation values.

4.3. Transmission vs absorption characteristics

Fig. 7 shows the transmission behaviour with respect to β of azo/anthraquinone dichroic dye doped PDLCs for ON to OFF state at different concentrations of dyes. As observed in figure, with the increase of dye concentrations, the transmission curve trends to an exponential behaviour because their β increases, simultaneously. Moreover, the curves move towards their respective maximum transmission, as all of their β approaches to zero in ON state. For each concentration of both azo and anthraquinone dyes, the transmission curves show the predisposition to each other for all gray to gray scale.

4.4. Contrast ratio vs absorption coefficient

Contrast ratio (CR), an important consideration of the EO performance for dye doped PDLCs, was determine with their maximum (ON state) and minimum (OFF state) transmittance values. In the present theoretical consideration, the CR can be measured by the relation

$$CR = \exp[(\gamma_{OFF} - \gamma_{ON})d] \quad (37)$$

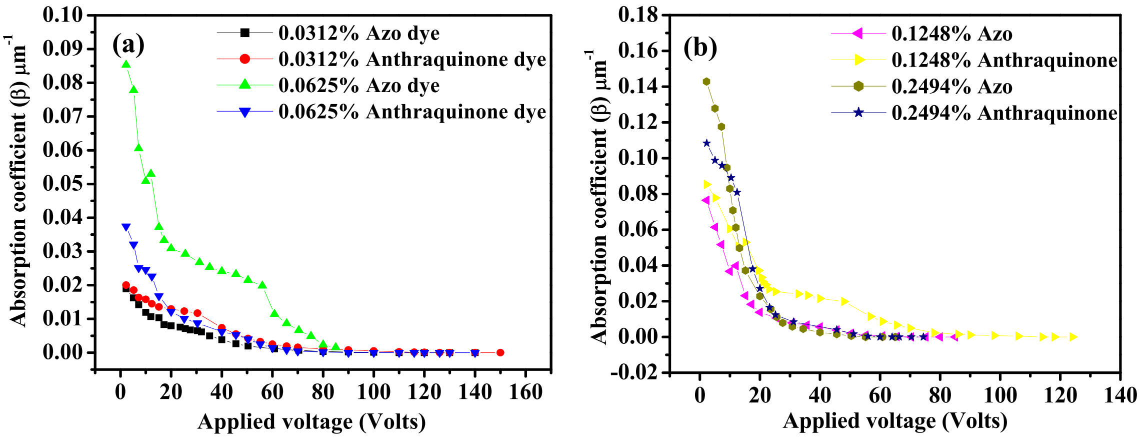

Fig. 6. (a-b) Absorption coefficient of azo and anthraquinone dye doped PDLCs at 0.0312, 0.0625, 0.1248 and 0.2494% dye concentrations.

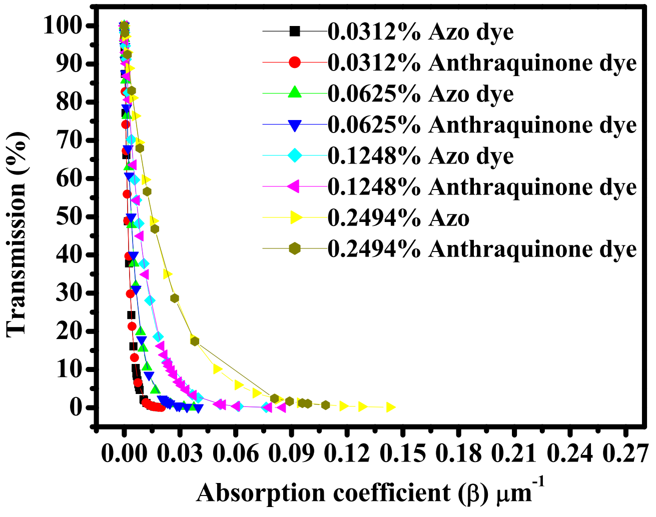

Fig. 7. ON to OFF state transmission behaviors with respect to absorption coefficient of azo/anthraquinone dichroic dye doped PDLCs at 0.0312, 0.0625, 0.1248 and 0.2494% concentrations of dyes.

Here, γ_{OFF} and γ_{ON} are the extinction coefficient of PDLC samples in OFF and ON states, respectively, depend on the β and here d is the cell thickness. Fig. 8 (a, b) shows the experimental results of CR as a function of β for both azo/anthraquinone dichroic dye doped PDLCs. Again, sort of exponential behaviour of the same was obtained for OFF to ON state. It can be seen from the Fig. 8 (a) that the anthraquinone dye doped PDLC has higher CR than azo dye doped PDLC at each value of the β with 0.0312, 0.0625% of dye concentrations. This higher CR of anthraquinone dye doped PDLC is arisen due to the much higher transmittance in the ON state compared with azo dye doped PDLCs. However, with the increase of dye concentration (≥0.1248%) azo dye doped PDLCs showed higher CR as that of the anthraquinone dye doped PDLCs showing much lower ON state transmittance at these concentrations. Thus, adding dye to the LC to decrease OFF state transmittance also reduces ON state transmittance in both cases. This indicates that a control amount of dye is always a crucial measure for an adequate PDLC performance.

Fig. 8(c) shows the experimentally obtained values of CR as a function of dye concentration. Overall, PDLC films with the low dye concentrations exhibit the higher CR values in both dyes cases. Actually, adding a controlled amount of absorbing dyes to the LC prior to PDLC film formation significantly increases the CR, as light absorbed by the dye is not merely redirected; rather, it never emerges from the film [47]. Moreover, as per Eq. (36), the dye doped PDLCs have higher extinction coefficient compared with extinction coefficient [$\exp(-\frac{\gamma_{\parallel}d}{3})$] of PDLC, which resulted into the degraded OFF-state transmittance and advanced CR. Comparatively, Eq. (36) also comprises that azo dye doped PDLCs lead to higher CR at higher dye concentrations (when maximum transmissions are not dominating for both dye doped PDLCs). Higher CR at higher concentrations for azo dye may also have been arisen due better optical and alignment properties of dye at these concentrations [35,44,45].

4.5. Droplet morphology

LC droplets' structures and orientations have been studied with POM from the top of the cell (i.e., viewing direction is perpendicular to the

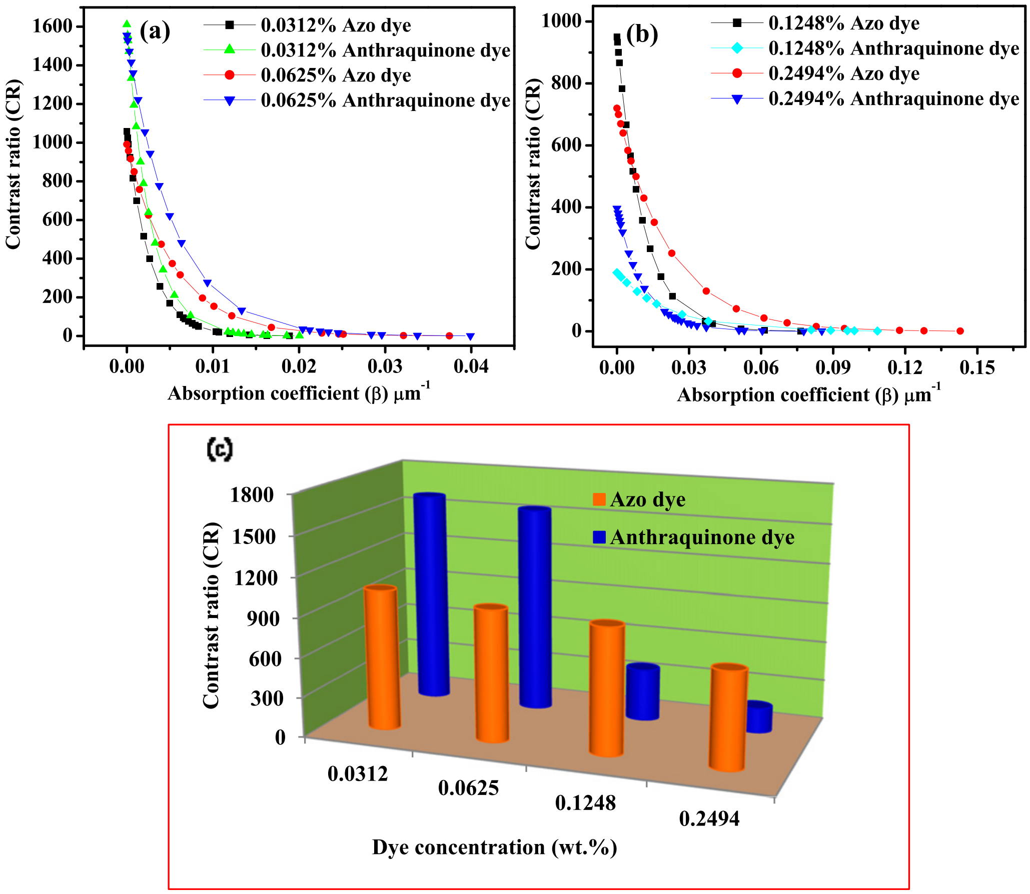

Fig. 8. (a, b) ON to OFF state CR with respect to absorption coefficient and (c) contrast ratio versus dye concentration of azo/anthraquinone dichroic dye doped PDLCs at 0.0312, 0.0625, 0.1248 and 0.2494%.

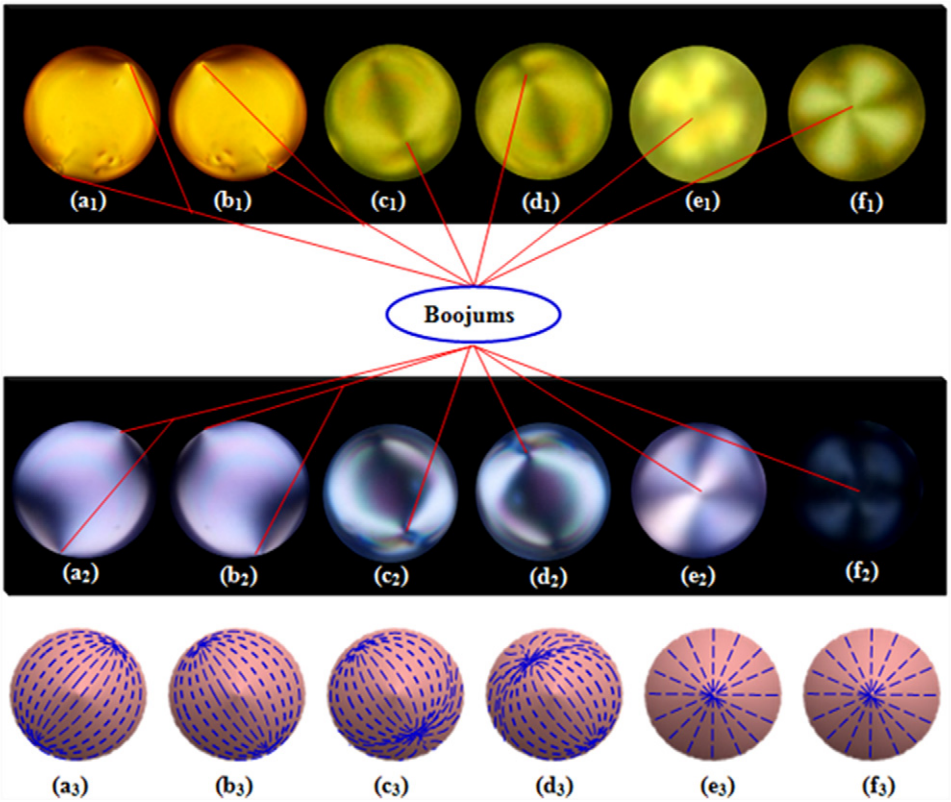

Fig. 9. (a_1-f_1) and (a_2-f_2) POM images of LC droplets with their different shapes in azo and anthraquinone dye doped PDLCs, respectively: (a_1-a_2) and (b_1-b_2) face and rear view of LC droplets along the equator, (c_1-c_2) and (d_1-d_2) face and rear single boojum in LC droplets, (e_1-e_2) and (f_1-f_2) OFF and ON state top view of LC droplets (director and viewing angle are parallel), (a_3 to f_3) corresponding molecules arrangement and boojum position for each droplet. All images were captured under cross polarizer at 50× magnification.

substrates) for both dyes doped PDLCs as shown in Fig. 9. POM study confirmed that the LC droplets formed in the polymer matrix were randomly oriented and predominantly bipolar in shape, however several other shapes like radial, axial and toroidal were also observed as viewing angles with respect to the director of the each LC droplet tare varied. It indicates that randomly oriented LC droplets are initially bipolar in structure and different shapes of droplets are observed due to random orientation cum different side view to the observer. The detailed droplet morphologies of both systems can found in our pervious publications [12,13,48]. On considering the face and rear side of the LC droplets along the equator, with viewing angle of 90° to the director of LC molecules inside the droplet, the perfect bipolar droplets can be seen with two boojums stared at the boundaries as shown in Figs. 9 (a_1-a_2) and (b_1-b_2), respectively. Further, Figs. 9(c_1-c_2) and (d_1-d_2) show the face and rear single boojum in each LC droplet. These structures have been observed when some angle was found in between the viewer and director of the droplet. Figs. 9 (e_1-e_2) and (f_1-f_2) show the radial type structures and could be observed when angle between the viewer and director of the LC droplet is 0° (i.e. when director and viewing angle are parallel). In this condition all molecules will become vertical to the substrate and form radial type structure. The hypothetical model in Fig. 9 (a_3 to f_3) shows the corresponding molecules arrangement and boojum position for each droplet.

4.6. Voltage transmission characteristics

The effect of different concentrations (0.0312, 0.0625, 0.1248 and 0.2494%) of azo and anthraquinone dyes on voltage-transmission characteristics PDLCs has been studied. As shown in Fig. 10, by taking the maximum transmission 100%, on comparison, the maximum

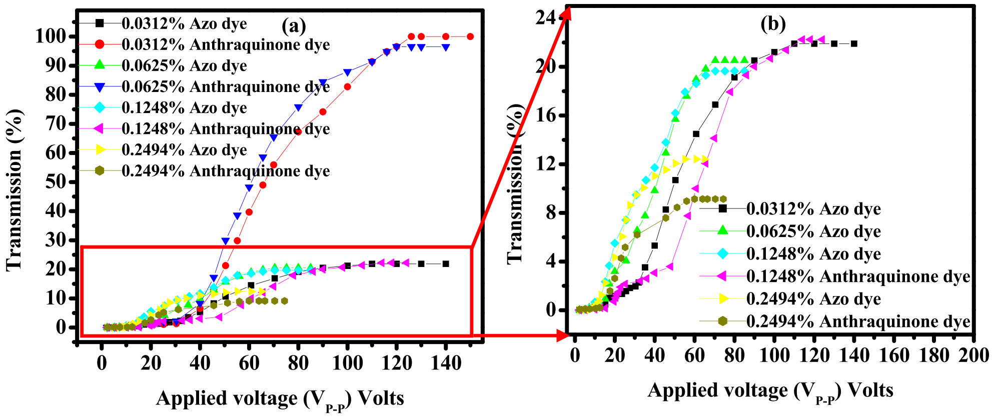

Fig. 10. Dye concentration dependent voltage-transmission characteristic of azo and anthraquinone dye doped PDLCs by taking maximum transmission 100%.

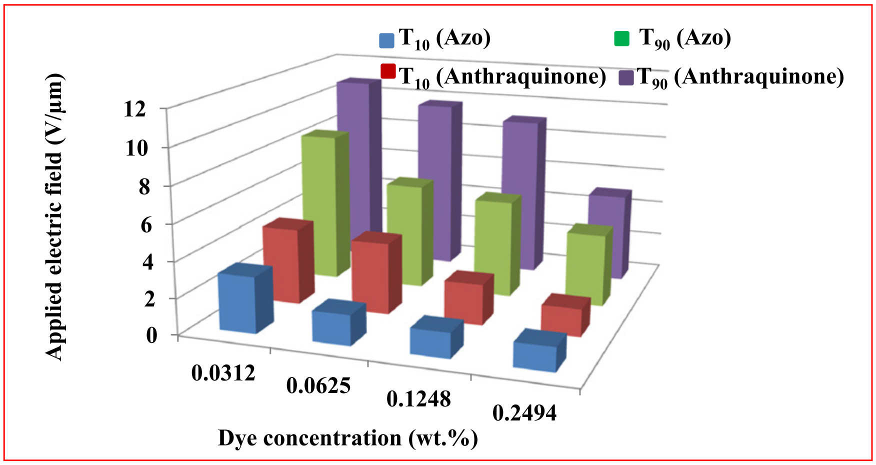

Fig. 11. Threshold (T_{10}) and operating (T_{90}) fields vs dye concentrations of azo and anthraquinone dye doped PDLCs.

transmission was achieved at the low dye concentration (0.0312%). With the increase of dye concentrations transmission were found reduced in both azo and anthraquinone dye doped PDLCs. The higher transmission at low content of dye might be due to the fact that the ordinary refractive index n_o of the LC matches more closely with that of the polymer at low dye concentration [12,13]. In fact, these dye molecules reduce the scattering with the alignment of the LC molecules along the director and enhance ON state transmission. Further, it is also summarized that the transmission was found higher with minutely elevated operating voltage in anthraquinone dye doped PDLCs, which may arise due to lower absorbance (as reported in previous section) in case of the anthraquinone dye doped PDLCs rather than the azo dye doped PDLCs. Moreover, on comparing the results obtained from azo and anthraquinone dye doped PDLCs for different dye concentrations, it is precised that the lesser operating voltages are required in case of azo dye doped PDLCs.

Further, as shown in Fig. 10 (a), for all azo and anthraquinone dye doped PDLCs, the transmission increases with applied voltage up to complete orientation of director at that saturation value. In comparison, the transmission was found >4.5 times higher at 0.0312 and 0.0625% concentrations of anthraquinone dye then the transmission observed at same concentrations of azo dye. However, at higher (≥0.1248) concentrations, the maximum transmissions were found much lower for anthraquinone dye with compared to azo dye doped samples.

Fig. 11 shows the threshold (T_{10}) and operating (T_{90}) fields versus dye concentrations of azo and anthraquinone dye doped PDLCs. The threshold electric fields were observed 3.13 and 4.27, 1.68 and 4.0, 1.39 and 2.27, 1.32 and 1.53 V/µm, respectively for azo and anthraquinone dye doped PDLCs at 0.0312, 0.0625, 0.1248 and 0.2494% dye concentration. In the same system, the operating fields were observed 8.43 and 11.10, 5.92 and 9.80, 5.45 and 9.04, 4.06 and 4.97 V/µm, at 0.0312, 0.0625, 0.1248 and 0.2494% concentration of azo and anthraquinone dyes. Thus the azo dye doped PDLCs show lower threshold and operating fields for all concentrations as compared with anthraquinone dye doped PDLCs.

5. Response time characteristics

The response time [rise time (τ_r) + decay time (τ_d)] characteristic empowers performance of PDLC for practical applications. The rise time is computed as [49]:

$$\frac{1}{\tau_r} = \frac{1}{\acute{\eta}}\left[\Delta\varepsilon E^2 + \frac{K\left(l^2-1\right)}{R^2}\right] \tag{38}$$

where $\acute{\eta}$ is the rotational viscosity coefficient, E is the applied electric filed, $\Delta\varepsilon$ is the dielectric anisotropy and l is the aspect ratio of LC droplets. Here, τ_r is given by

$$\tau_r \cong \frac{\gamma_1}{\Delta\varepsilon E^2} \text{ or, } \tau_r \propto \frac{1}{E^2} \tag{39}$$

The decay time (τ_d), which depends on the viscosity of LCs, the elasticity of the LCs, shape and size of the LC droplets, is computed as

$$\tau_d \cong \frac{\gamma R^2}{K\left(l^2-1\right)} \tag{40}$$

Fig. 12 shows the magnitude of rise time, decay time and total response time of azo/ anthraquinone dye doped PDLCs as a function of different dye concentrations (0.0312, 0.0625, 0.1248 and 0.2494%) at their respective operating electric field.

Experimental results showed that the rise time does not show any considerable disparity in both cases as a function of dye concentration. Although, a small increase in the τ_r was found with increasing the azo dye concentration, at the same time, the dye decay time initially decreases when anthraquinone dye concentration is increased up to 0.1248%. On further increase of the anthraquinone dye concentration the decay time also increased simultaneously. Thus, total response time for 0.1248% anthraquinone dye doped PDLC is faster among all

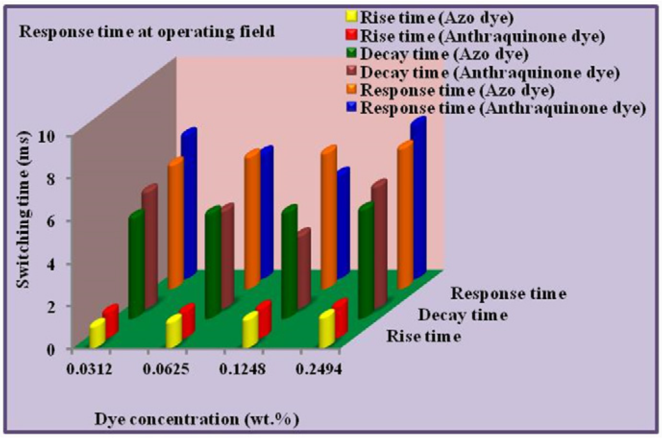

Fig. 12. Response time characteristic of azo and anthraquinone dye doped PDLCs as a function of dye concentrations (0.0312, 0.0625, 0.1248 and 0.2494%).

anthraquinone/azo dye doped PDLCs. At higher dye concentrations, an increase in response time might be due to inter atomic and elastic forces between dye and LC molecules. Further, increased viscosity may also slow the response time up to some extent [50,51].

6. Conclusion

Azo and anthraquinone dye doped PDLCs with varying concentrations 0.0312, 0.0625, 0.1248, and 0.2494% has been prepared as well as analyzed on the basis of the absorption considering established Beer's Law. β as a function of dye concentration and applied voltage was measured. The azo dye doped PDLCs exhibited more dye extinction coefficient at >0.0625% of dye concentration, which resulted in higher absorbance and higher CR. For each concentration of both azo and anthraquinone dye, the transmission versus β curves show the predisposition to each other for all gray to gray scale. Higher CR of anthraquinone dye doped PDLC at lower dye concentrations is arisen due to higher ON state transmittance as compared with azo dye doped PDLCs. POM study confirmed that randomly oriented LC droplets are initially bipolar in structure and different shapes of droplets are observed due to random orientation cum different side view to the observer. The ideal bipolar LC droplets was observed with two boojums stared at the boundaries along the equator when observer is at right angle with the droplet director. Azo dye doped PDLCs show lower threshold and operating fields with consistent response time for all concentrations as compared with anthraquinone dye doped PDLCs.

Acknowledgement

One of the authorsVandna Sharma [IF150442] gratefully acknowledges Department of Science and Technology, India for financial support under INSPIRE fellowship.

References

[1] L. Yu, Z.X. Cheng, Z.J. Dong, Y.H. Zhang, H.F. Yu, Photomechanical response of polymer-dispersed liquid crystals/graphene oxide nanocomposites, J. Mater. Chem. C 2 (2014) 8501–8506.
[2] Z.X. Cheng, T.J. Wang, X. Li, Y.H. Zhang, H.F. Yu, NIR-VIS-UV light-responsive actuator films of polymer-dispersed liquid crystal/graphene oxide nanocomposites, ACS Appl. Mater. Interfaces 7 (2015) 27494–27501.
[3] S.–M. Guo, X. Liang, C.–H. Zhang, M. Chen, C. Shen, L.–Y. Zhang, X. Yuan, B.–F. He, H. Yang, Preparation of a thermally light-transmittance-controllable film from a coexistent system of polymer-dispersed and polymer- stabilized liquid crystals, ACS Appl. Mater. Interfaces 9 (2017) 2942–2947.
[4] N. Kumano, T. Seki, M. Ishii, H. Nakamura, T. Umemura, Y. Takeoka, Multicolor polymer-dispersed liquid crystals, Adv. Mater. 23 (2011) 884–888.
[5] J. Seo, M. Song, J. Jeong, S. Nam, I. Heo, S.Y. Park, I.K. Kang, J.H. Lee, H. Kim, Y. Kim, Broadband pH-sensing organic transistors with polymeric sensing layers featuring liquid crystal microdomains encapsulated by Di-block copolymer chains, ACS Appl. Mater. Interfaces 8 (2016) 23862–23867.
[6] D.J. Broer, J. Lub, G.N. Mol, Wide-band reflective polarizars from cholesteric polymer networks with a pitch gradien, Nature 378 (1995) 467–469.
[7] M. Kim, K.J. Park, S. Seok, J.M. Ok, H.T. Jung, J. Choe, D.H. Kim, Fabrication of microcapsules for dye-doped polymer- dispersed liquid crystal-based smart windows, ACS Appl. Mater. Interfaces 7 (2015) 17904–17909.
[8] L. Yu, H.F. Yu, Light-powered tumbler movement of graphene oxide/polymer nanocomposites, ACS Appl. Mater. Interfaces 7 (2015) 3834–3839.
[9] H. Kikuchi, M. Yokota, Y. Hisakado, H. Yang, T. Kajiyama, Polymer-stabilized liquid crystal blue phases, Nat. Mater. 1 (2002) 64–68.
[10] Z.G. Zheng, H.F. Wang, G. Zhu, X.W. Lin, J.N. Li, W. Hu, H.Q. Cui, D. Shen, Y.Q. Lu, Low-temperature-applicable polymer-stabilized blue-phase liquid crystal and its kerr effect, J. Soc. Inf. Disp. 20 (2012) 326–332.
[11] P. Kumar, K.K. Raina, Morphological and electro-optical responses of dichroic polymer dispersed liquid crystal films, Curr. Appl. Phys. 7 (2007) 636–642.
[12] P. Kumar, V. Sharma, C. Jaggi, P. Malik, K.K. Raina, Orientational control of liquid crystal molecules via carbon nanotubes and dichroic dye in polymer dispersed liquid crystal, Liq. Cryst. 44 (2017) 843–853.
[13] P. Kumar, V. Sharma, C. Jaggi, K.K. Raina, Dye-dependent studies on droplet pattern and electro-optic behaviour of polymer dispersed liquid crystal displays, Liq. Cryst. 44 (2017) 757–767.
[14] V. Sharma, P. Kumar, Studies of absorption coefficient cum electro-optic performance of polymer dispersed liquid crystal doped with CNT and dichroic dye, Phys. B Condens. Matter 524 (2017) 118–122.
[15] G.H. Heilmeier, L.A. Zanoni, Guest-host interactions in nematic liquid crystals. A new electro-optic effect, Appl. Phys. Lett. 13 (1968) 91–92.
[16] M.G. Debije, Solar energy collectors with tunable transmission, Adv. Funct. Mater. 20 (2010) 1498–1502.
[17] H. Xie, L. Wang, H. Wang, C. Zou, M. Wang, B. Wang, Z. Chen, L. Zhang, X. Zhang, Z. Yang, H. Yang, Electrically tunable properties of wideband-absorptive and reflection selective films based on multi-dichroic dye-doped cholesteric liquid crystal, Liq. Cryst. 42 (2015) 1698–1705.
[18] C.W. Su, M.Y. Chen, Polymer-dispersed liquid crystal applied in active-matrix transparent display, J. Disp. Technol. 10 (2014) 683–687.
[19] C.W. Su, C.C. Liao, M.Y. Chen, Color transparent display using polymer-dispersed liquid crystal, J. Disp. Technol. 12 (2016) 31–34.
[20] P. Mach, S.J. Rodriguez, R. Nortrup, P. Wiltzius, J.A. Rogers, Monolithically integrated, flexible display of polymer-dispersed liquid crystal driven by rubber-stamped organic thin-film transistors, Appl. Phys. Lett. 78 (2001) 3592–3594.
[21] C.D. Sheraw, L. Zhou, J.R. Huang, D.J. Gundlach, T.N. Jackson, Organic thin-film transistor-driven polymer-dispersed liquid crystal displays on flexible polymeric substrates, Appl. Phys. Lett. 80 (2002) 1088–1090.
[22] J. Lee, D.H. Kim, J.Y. Kim, B. Yoo, J.W. Chung, J.I. Park, B.L. Lee, J.Y. Jung, J.S. Park, B. Koo, S. Im, J.W. Kim, B. Song, M.H. Jung, J.E. Jang, Y.W. Jin, S.Y. Lee, Reliable and uniform thin-film transistor arrays based on inkjet-printed polymer semiconductors for full color reflective displays, Adv. Mater. 25 (2013) 5886–5892.
[23] Y.J. Liu, X. Ding, S.C.S. Lin, J. Shi, I.-K. Chiang, T.J. Huang, Surface acoustic wave driven light shutters using polymer-dispersed liquid crystals, Adv. Mater. 23 (2011) 1656–1659.
[24] J.W. Lee, J.K. Kim, F. Ahmad, M. Jamil, Y.J. Jeon, Properties of thiol–vinyl PDLC films without additional photoinitiator, Liq. Cryst. 41 (2014) 1109–1115.
[25] Y.-C. Su, C.-C. Chu, W.-T. Chang, V.K.S. Hsiao, Characterization of optically switchable holographic polymer-dispersed liquid crystal transmission gratings, Opt. Mater. 34 (2011) 251–255.
[26] Y.-T. Lai, J.-C. Kuo, Y.-J. Yang, A novel gas sensor using polymer-dispersed liquid crystal doped with carbon nanotubes, Sensors Actuators A Phys. 215 (2014) 83–88.
[27] G.-R. Xiong, G.-Z. Han, C. Sun, H. Xu, H.–M. Wei, Z.-Z. Gu, Phototunable microlens array based on polymer dispersed liquid crystals, Adv. Funct. Mater. 19 (2009) 1082–1086.
[28] L. Li, L. Wang, L. Deng, Low threshold random lasing in DDPDLCs, DDPDLC and ZnO nanoparticles and dye solution @ ZnO nanoparticle capillaries, Laser Phys. 11 (2014), 025201.
[29] E. Perju, L. Marin, V.C. Grigoras, M. Bruma, Thermotropic and optical behaviour of new PDLC systems based on a polysulfone matrix and a cyanoazomethine liquid crystal, Liq. Cryst. 38 (2011) 893–905.
[30] M.S. Li, A.Y.-G. Fuh, Y.H. Huang, C.H. Yan, S.T. Wu, Electrically switchable high-fold-helix spiral phase plate based on polymer dispersed liquid crystals, Appl. Phys. Express 6 (2013) 112201.
[31] T.-C. Hsu, C.-H. Lu, Y.-T. Huang, W.-P. Shih, W.-S. Chen, Concentric polymer-dispersed liquid crystal rings for light intensity modulation, Sensors Actuators A Phys. 169 (2011) 341–346.
[32] Y.-H. Lin, T.-Y. Chu, Y.-S. Tsou, K.-H. Chang, Y.-P. Chiu, An electrically switchable surface free energy on a liquid crystal and polymer composite film, Appl. Phys. Lett. 101 (2012) 233502.
[33] E. Perju, E. Paslaru, L. Marin, Polymer-dispersed liquid crystal composites for bio-applications: thermotropic, surface and optical properties, Liq. Cryst. 42 (2015) 370–382.
[34] R.M. Christie, Colour chemistry: Colour- a brief historical perspective, Royal society of chemistry publishing, Cambridge, 2001 1–11 Chapter 1.
[35] J. Griffiths, K.-C. Feng, The influence of intramolecular hydrogen bonding on the order parameter and photostability properties of dichroic azo dyes in a nematic liquid crystal host, J. Mater. Chem. 9 (1999) 2333–2338.
[36] H.K. Lee, A. Kanazawa, T. Shiono, T. Ikeda, All optically controllable polymer liquid crystal composite films containing the azobenzene liquid crystal, Chem. Mater. 10 (1998) 1402–1407.
[37] O. Zaplo, J. Stumpe, Photochemically induced modification of polymer dispersed liquid crystals with photochromic stilbene, Mol. Cryst. Liq. Cryst. 213 (1992) 153–161.
[38] T. Kajiyama, H. Kikuchi, K. Nakamura, Photoresponsive electro-optical effect of liquid crystalline polymer/low molecular weight liquid crystal composite system, Proc. SPIE (1993) (1911) 111–121.
[39] P. Kumar, Neeraj, S.W. Kang, S.H. Lee, K.K. Raina, Analysis of dichroic dye-doped polymer-dispersed liquid crystal materials for display devices, Thin Solid Films 520 (2011) 457–463.
[40] P. Malik, K.K. Raina, Dichroic dye-dependent studies in guest–host polymer-dispersed liquid crystal films, Phys. B Condens. Matter 405 (2010) 161–166.
[41] J.J. Wu, C.–M. Wang, W.–Y. Li, S.–H. Chen, Electrooptical properties of aligned dye-doped polymer dispersed liquid crystal films, Jpn. J. Appl. Phys. 37 (1998) 6434–6439.
[42] Technical data sheet. , Merck KGaA, Darmstadt (Germany), 2001.
[43] Norland Optical Adhesive, Technical data sheet, Norland Products, Cranbury (NJ), 2015.
[44] G.H. Heilmeier, L.A. Zanoni, Guest-host interactions in nematic liquid crystals. A new electro-optic effect, Appl. Phys. Lett. 13 (1968) 91–92.
[45] L.O. Palsson, M. Szablewski, A. Roberts, A. Masutani, G.D. Love, G.H. Cross, D. Bloor, A.J. Kay, A.D. Woolhouse, A. Masutani, A. Yasuda, Orientation and solvatochromism of dyes in liquid crystals, Mol. Cryst. Liq. Cryst. 402 (2003) 279–289.
[46] H. Iwanaga, K. Naito, Highly soluble anthraquinone dyes with CF3-groups for guest-host liquid crystal displays, Jpn. J. Appl. Phys. Part 2 (37) (1998) L356–L358.
[47] G.P. Montgomery Jr., N.A. Vaz, Contrast ratios of polymer-dispersed liquid crystal films, Appl. Opt. 26 (1987) 738–743.

[48] V. Sharma, P. Kumar, A. Sharma, Chinky, K.K. Raina, Droplet configuration control with orange azo dichroic dye in polymer dispersed liquid crystal for advanced electro-optic characteristics, J. Mol. Liq. 233 (2017) 122–130.
[49] K. Kondo, M. Arakawa, A. Fukuda, E. Kuze, Light propagation in Williams domains as analyzed numerically by geometrical optics, Jpn. J. Appl. Phys. 22 (1983) 394–399.
[50] R.R. Deshmukh, A.K. Jain, The complete morphological, electro-optical and dielectric study of dichroic dye-doped polymer-dispersed liquid crystal, Liq. Cryst. 41 (2014) 960–975.
[51] S.P. Yadav, K.K. Pandey, A.K. Misra, R. Manohar, Electro-optical behavior of dye doped nematic liquid crystal, Acta Phys. Pol. A 119 (2011) 824–828.

ELSEVIER LICENSE TERMS AND CONDITIONS

Sep 19, 2022

This Agreement between Harbin Institute of Technology -- Ruicong Zhang ("You") and Elsevier ("Elsevier") consists of your license details and the terms and conditions provided by Elsevier and Copyright Clearance Center.

License Number	5392410844221
License date	Sep 19, 2022
Licensed Content Publisher	Elsevier
Licensed Content Publication	Dyes and Pigments
Licensed Content Title	Ion-doped liquid-crystal cell with low opaque-state specular transmittance based on electro-hydrodynamic effect
Licensed Content Author	Jae-Won Huh,Jin-Hun Kim,Seung-Won Oh,Seong-Min Ji,Tae-Hoon Yoon
Licensed Content Date	Mar 1, 2018
Licensed Content Volume	150
Licensed Content Issue	n/a
Licensed Content Pages	5
Start Page	16
End Page	20
Type of Use	reuse in a journal/magazine
Requestor type	academic/educational institute
Portion	figures/tables/illustrations
Number of figures/tables/illustrations	3
Format	both print and electronic
Are you the author of this Elsevier article?	No
Will you be translating?	No
Title of new article	Advanced liquid crystal-based switchable optical devices for light protection applications: principles and strategies
Lead author	Ruicong Zhang, Zhibo Zhang, Jiecai Han, Lei Yang, Jiajun li, Zicheng Song Tianyu Wang, Jiaqi Zhu
Title of targeted journal	Light: Science & Applications
Publisher	Springer Nature
Expected publication date	Nov 2022
Portions	Figure 5,Figure 6,Figure 7
Requestor Location	Harbin Institute of Technology<br>No. 92, Xidazhi Street, Nangang District<br><br>Harbin, 150080<br>China<br>Attn: Harbin Institute of Technology
Publisher Tax ID	GB 494 6272 12
Total	**0.00 USD**
Terms and Conditions	

INTRODUCTION

1. The publisher for this copyrighted material is Elsevier. By clicking "accept" in connection with completing this licensing transaction, you agree that the following terms and conditions apply to this transaction (along with the Billing and Payment terms

and conditions established by Copyright Clearance Center, Inc. ("CCC"), at the time that you opened your Rightslink account and that are available at any time at http://myaccount.copyright.com).

GENERAL TERMS

2. Elsevier hereby grants you permission to reproduce the aforementioned material subject to the terms and conditions indicated.
3. Acknowledgement: If any part of the material to be used (for example, figures) has appeared in our publication with credit or acknowledgement to another source, permission must also be sought from that source. If such permission is not obtained then that material may not be included in your publication/copies. Suitable acknowledgement to the source must be made, either as a footnote or in a reference list at the end of your publication, as follows:
"Reprinted from Publication title, Vol /edition number, Author(s), Title of article / title of chapter, Pages No., Copyright (Year), with permission from Elsevier [OR APPLICABLE SOCIETY COPYRIGHT OWNER]." Also Lancet special credit - "Reprinted from The Lancet, Vol. number, Author(s), Title of article, Pages No., Copyright (Year), with permission from Elsevier."
4. Reproduction of this material is confined to the purpose and/or media for which permission is hereby given.
5. Altering/Modifying Material: Not Permitted. However figures and illustrations may be altered/adapted minimally to serve your work. Any other abbreviations, additions, deletions and/or any other alterations shall be made only with prior written authorization of Elsevier Ltd. (Please contact Elsevier's permissions helpdesk here). No modifications can be made to any Lancet figures/tables and they must be reproduced in full.
6. If the permission fee for the requested use of our material is waived in this instance, please be advised that your future requests for Elsevier materials may attract a fee.
7. Reservation of Rights: Publisher reserves all rights not specifically granted in the combination of (i) the license details provided by you and accepted in the course of this licensing transaction, (ii) these terms and conditions and (iii) CCC's Billing and Payment terms and conditions.
8. License Contingent Upon Payment: While you may exercise the rights licensed immediately upon issuance of the license at the end of the licensing process for the transaction, provided that you have disclosed complete and accurate details of your proposed use, no license is finally effective unless and until full payment is received from you (either by publisher or by CCC) as provided in CCC's Billing and Payment terms and conditions. If full payment is not received on a timely basis, then any license preliminarily granted shall be deemed automatically revoked and shall be void as if never granted. Further, in the event that you breach any of these terms and conditions or any of CCC's Billing and Payment terms and conditions, the license is automatically revoked and shall be void as if never granted. Use of materials as described in a revoked license, as well as any use of the materials beyond the scope of an unrevoked license, may constitute copyright infringement and publisher reserves the right to take any and all action to protect its copyright in the materials.
9. Warranties: Publisher makes no representations or warranties with respect to the licensed material.
10. Indemnity: You hereby indemnify and agree to hold harmless publisher and CCC, and their respective officers, directors, employees and agents, from and against any and all claims arising out of your use of the licensed material other than as specifically authorized pursuant to this license.
11. No Transfer of License: This license is personal to you and may not be sublicensed, assigned, or transferred by you to any other person without publisher's written permission.
12. No Amendment Except in Writing: This license may not be amended except in a writing signed by both parties (or, in the case of publisher, by CCC on publisher's behalf).
13. Objection to Contrary Terms: Publisher hereby objects to any terms contained in any purchase order, acknowledgment, check endorsement or other writing prepared by you, which terms are inconsistent with these terms and conditions or CCC's Billing and Payment terms and conditions. These terms and conditions, together with CCC's Billing and Payment terms and conditions (which are incorporated herein), comprise the entire agreement between you and publisher (and CCC) concerning this licensing transaction. In the event of any conflict between your obligations established by these terms and conditions and those established by CCC's Billing and Payment terms and conditions, these terms and conditions shall control.
14. Revocation: Elsevier or Copyright Clearance Center may deny the permissions described in this License at their sole discretion, for any reason or no reason, with a full refund payable to you. Notice of such denial will be made using the contact information provided by you. Failure to receive such notice will not alter or invalidate the denial. In no event will Elsevier or Copyright Clearance Center be responsible or liable for any costs, expenses or damage incurred by you as a result of a denial of your permission request, other than a refund of the amount(s) paid by you to Elsevier and/or Copyright Clearance Center for denied permissions.

LIMITED LICENSE

The following terms and conditions apply only to specific license types:
15. **Translation**: This permission is granted for non-exclusive world **English** rights only unless your license was granted for translation rights. If you licensed translation rights you may only translate this content into the languages you requested. A professional translator must perform all translations and reproduce the content word for word preserving the integrity of the article.
16. **Posting licensed content on any Website**: The following terms and conditions apply as follows: Licensing material from an Elsevier journal: All content posted to the web site must maintain the copyright information line on the bottom of each image; A hyper-text must be included to the Homepage of the journal from which you are licensing at http://www.sciencedirect.com/science/journal/xxxxx or the Elsevier homepage for books at http://www.elsevier.com; Central Storage: This license does not include permission for a scanned version of the material to be stored in a central repository such as that provided by Heron/XanEdu.
Licensing material from an Elsevier book: A hyper-text link must be included to the Elsevier homepage at http://www.elsevier.com . All content posted to the web site must maintain the copyright information line on the bottom of each image.

Posting licensed content on Electronic reserve: In addition to the above the following clauses are applicable: The web site must be password-protected and made available only to bona fide students registered on a relevant course. This permission is granted for 1 year only. You may obtain a new license for future website posting.
17. **For journal authors:** the following clauses are applicable in addition to the above:
Preprints:
A preprint is an author's own write-up of research results and analysis, it has not been peer-reviewed, nor has it had any other value added to it by a publisher (such as formatting, copyright, technical enhancement etc.).
Authors can share their preprints anywhere at any time. Preprints should not be added to or enhanced in any way in order to appear more like, or to substitute for, the final versions of articles however authors can update their preprints on arXiv or RePEc with their Accepted Author Manuscript (see below).
If accepted for publication, we encourage authors to link from the preprint to their formal publication via its DOI. Millions of researchers have access to the formal publications on ScienceDirect, and so links will help users to find, access, cite and use the best available version. Please note that Cell Press, The Lancet and some society-owned have different preprint policies. Information on these policies is available on the journal homepage.
Accepted Author Manuscripts: An accepted author manuscript is the manuscript of an article that has been accepted for publication and which typically includes author-incorporated changes suggested during submission, peer review and editor-author communications.
Authors can share their accepted author manuscript:

- immediately
    - via their non-commercial person homepage or blog
    - by updating a preprint in arXiv or RePEc with the accepted manuscript
    - via their research institute or institutional repository for internal institutional uses or as part of an invitation-only research collaboration work-group
    - directly by providing copies to their students or to research collaborators for their personal use
    - for private scholarly sharing as part of an invitation-only work group on commercial sites with which Elsevier has an agreement
- After the embargo period
    - via non-commercial hosting platforms such as their institutional repository
    - via commercial sites with which Elsevier has an agreement

In all cases accepted manuscripts should:

- link to the formal publication via its DOI
- bear a CC-BY-NC-ND license - this is easy to do
- if aggregated with other manuscripts, for example in a repository or other site, be shared in alignment with our hosting policy not be added to or enhanced in any way to appear more like, or to substitute for, the published journal article.

Published journal article (JPA): A published journal article (PJA) is the definitive final record of published research that appears or will appear in the journal and embodies all value-adding publishing activities including peer review co-ordination, copy-editing, formatting, (if relevant) pagination and online enrichment.
Policies for sharing publishing journal articles differ for subscription and gold open access articles:
Subscription Articles: If you are an author, please share a link to your article rather than the full-text. Millions of researchers have access to the formal publications on ScienceDirect, and so links will help your users to find, access, cite, and use the best available version.
Theses and dissertations which contain embedded PJAs as part of the formal submission can be posted publicly by the awarding institution with DOI links back to the formal publications on ScienceDirect.
If you are affiliated with a library that subscribes to ScienceDirect you have additional private sharing rights for others' research accessed under that agreement. This includes use for classroom teaching and internal training at the institution (including use in course packs and courseware programs), and inclusion of the article for grant funding purposes.
Gold Open Access Articles: May be shared according to the author-selected end-user license and should contain a CrossMark logo, the end user license, and a DOI link to the formal publication on ScienceDirect.
Please refer to Elsevier's posting policy for further information.
18. **For book authors** the following clauses are applicable in addition to the above: Authors are permitted to place a brief summary of their work online only. You are not allowed to download and post the published electronic version of your chapter, nor may you scan the printed edition to create an electronic version. **Posting to a repository:** Authors are permitted to post a summary of their chapter only in their institution's repository.
19. **Thesis/Dissertation**: If your license is for use in a thesis/dissertation your thesis may be submitted to your institution in either print or electronic form. Should your thesis be published commercially, please reapply for permission. These requirements include permission for the Library and Archives of Canada to supply single copies, on demand, of the complete thesis and include permission for Proquest/UMI to supply single copies, on demand, of the complete thesis. Should your thesis be published commercially, please reapply for permission. Theses and dissertations which contain embedded PJAs as part of the formal submission can be posted publicly by the awarding institution with DOI links back to the formal publications on ScienceDirect.

Elsevier Open Access Terms and Conditions

You can publish open access with Elsevier in hundreds of open access journals or in nearly 2000 established subscription journals that support open access publishing. Permitted third party re-use of these open access articles is defined by the author's choice of Creative Commons user license. See our open access license policy for more information.

Terms & Conditions applicable to all Open Access articles published with Elsevier:

Any reuse of the article must not represent the author as endorsing the adaptation of the article nor should the article be modified in such a way as to damage the author's honour or reputation. If any changes have been made, such changes must be clearly indicated.

The author(s) must be appropriately credited and we ask that you include the end user license and a DOI link to the formal publication on ScienceDirect.

If any part of the material to be used (for example, figures) has appeared in our publication with credit or acknowledgement to another source it is the responsibility of the user to ensure their reuse complies with the terms and conditions determined by the rights holder.

Additional Terms & Conditions applicable to each Creative Commons user license:

CC BY: The CC-BY license allows users to copy, to create extracts, abstracts and new works from the Article, to alter and revise the Article and to make commercial use of the Article (including reuse and/or resale of the Article by commercial entities), provided the user gives appropriate credit (with a link to the formal publication through the relevant DOI), provides a link to the license, indicates if changes were made and the licensor is not represented as endorsing the use made of the work. The full details of the license are available at http://creativecommons.org/licenses/by/4.0.

CC BY NC SA: The CC BY-NC-SA license allows users to copy, to create extracts, abstracts and new works from the Article, to alter and revise the Article, provided this is not done for commercial purposes, and that the user gives appropriate credit (with a link to the formal publication through the relevant DOI), provides a link to the license, indicates if changes were made and the licensor is not represented as endorsing the use made of the work. Further, any new works must be made available on the same conditions. The full details of the license are available at http://creativecommons.org/licenses/by-nc-sa/4.0.

CC BY NC ND: The CC BY-NC-ND license allows users to copy and distribute the Article, provided this is not done for commercial purposes and further does not permit distribution of the Article if it is changed or edited in any way, and provided the user gives appropriate credit (with a link to the formal publication through the relevant DOI), provides a link to the license, and that the licensor is not represented as endorsing the use made of the work. The full details of the license are available at http://creativecommons.org/licenses/by-nc-nd/4.0. Any commercial reuse of Open Access articles published with a CC BY NC SA or CC BY NC ND license requires permission from Elsevier and will be subject to a fee.

Commercial reuse includes:

- Associating advertising with the full text of the Article
- Charging fees for document delivery or access
- Article aggregation
- Systematic distribution via e-mail lists or share buttons

Posting or linking by commercial companies for use by customers of those companies.

20. **Other Conditions**:

v1.10

Questions? customercare@copyright.com or +1-855-239-3415 (toll free in the US) or +1-978-646-2777.

Dyes and Pigments 150 (2018) 16–20

Contents lists available at ScienceDirect

Dyes and Pigments

journal homepage: www.elsevier.com/locate/dyepig

Ion-doped liquid-crystal cell with low opaque-state specular transmittance based on electro-hydrodynamic effect

Jae-Won Huh, Jin-Hun Kim, Seung-Won Oh, Seong-Min Ji, Tae-Hoon Yoon*

Department of Electronics Engineering, Pusan National University, Busan 46241, South Korea

ARTICLE INFO

Keywords:
Liquid crystals
Light shutter
Dichroic dye
Electro-hydrodynamic effect

ABSTRACT

We demonstrate an ion-doped liquid-crystal (LC) cell that can provide a very low specular transmittance in the opaque state using the electro-hydrodynamic effect. In the opaque state, the LC and dye molecules are oriented randomly in planes parallel to the substrates because of the electrohydrodynamic effect. We found that the total transmittance of an ion-doped LC cell is almost the same as that of a light shutter based on light absorption and its haze value is the same as that of a light shutter based on light scattering. Moreover, an ion-doped LC cell can be fabricated without an ultraviolet curable process. Owing to its excellent opaque state properties, an ion-doped LC cell can be used in see-through display and smart-window applications.

1. Introduction

Liquid-crystal (LC) light shutters can be used for various applications, such as light modulators, smart windows, and see-through displays [1–21]. In particular, light shutters, which can be used to control the haze and transmittance simultaneously, have been actively studied for smart-window and see-through display applications [6–16,19]. By simply adding dichroic dye molecules in an LC light shutter based on the light scattering, the light scattering and absorption can be simultaneously controlled.

However, there is a trade-off between the light scattering and absorption. Light scattering relies on the random orientation of LC molecules, whereas light absorption can be maximized by orienting the dye molecules in planes parallel to the substrates. To overcome this trade-off, double-layered devices can be employed [11,16]. One layer is used for light scattering and the other for light absorption. However, these light shutters may suffer from disadvantages, including a low transmittance in the transparent state, a high thickness, and high fabrication costs.

In this study, we demonstrate a single-layered LC light shutter in which LC molecules with negative dielectric anisotropy are oriented randomly in planes parallel to the substrates using the electro-hydrodynamic effect when an electric field is applied between the two substrates. We confirmed that the proposed light shutter exhibits not only almost the same total transmittance as a light shutter based on light absorption, but also the same haze value as a light shutter based on light scattering. Owing to its excellent opaque state and simple structure, we consider the proposed LC light shutter a promising candidate for smart-window and see-through display applications.

2. Principle of operation

2.1. A light shutter using the electro-hydrodynamic effect

Simply by adding ion dopants in LCs, we can realize a light shutter that is based on light scattering using the electro-hydrodynamic effect [22–24]. For switching the light shutter, the frequency of the applied voltage wave must be carefully chosen because the haze value and operating voltage strongly depend on it.

To confirm the frequency dependence, an LC cell fabricated using ion-doped negative LCs was observed using a polarized optical microscope (POM), as shown in Fig. 1(a). We also measured the total transmittance, specular transmittance, and haze using a haze meter (HM-65W, Murakami Color Research Laboratory) while changing the frequency of the applied voltage wave (direct current (DC), 10 Hz, 100 Hz, 1 kHz, and 10 kHz), as shown in Fig. 1(b).

The specular [diffuse] transmittance T_s [T_d] refers to the ratio of the power of the beam that emerges from a cell, which is parallel (within a range of angles of 2.5°) [not parallel] to a beam entering the cell, to the power carried by the beam entering the LC cell. The total transmittance T_t is the sum of the specular transmittance T_s and the diffuse transmittance T_d: $T_t = T_s + T_d$. The haze H is the ratio of the diffuse transmittance to the total transmittance: $H = T_d/T_t$.

In the initial transparent state, the POM images exhibit the dark state because of vertically aligned LC molecules. When a voltage wave with a frequency between 0 and 1 kHz is applied, the negative LCs are

* Corresponding author.
E-mail address: thyoon@pusan.ac.kr (T.-H. Yoon).

https://doi.org/10.1016/j.dyepig.2017.11.001
Received 7 September 2017; Received in revised form 1 November 2017; Accepted 2 November 2017
Available online 05 November 2017
0143-7208/ © 2017 Elsevier Ltd. All rights reserved.

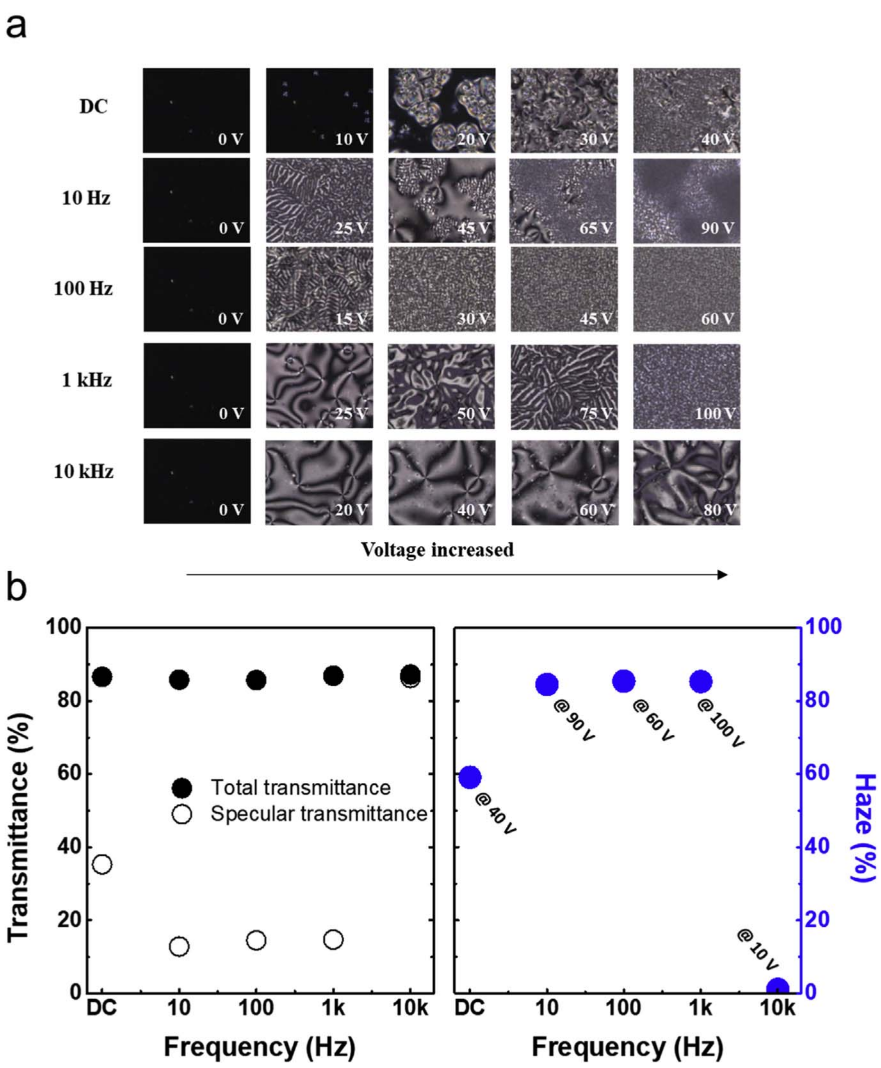

Fig. 1. (a) POM images and (b) the measured total transmittance, specular transmittance, haze of an ion-doped LC cell when DC, 10-Hz, 100-Hz, 1-kHz, and 10-kHz voltage waves were applied.

aligned parallel to the substrates. At the same time, the rotation of ions generates turbulence in the cell and POM image shows patterns called the 'Williams domain' [25]. As the applied voltage is increased, the turbulence becomes stronger, and the patterns become small domains which can scatter the incident light strongly. When the frequency of the applied voltage wave was 1 kHz, the cell showed a high haze of 85.3%. The POM image showed dependence on the frequency and amplitude of the applied voltage because the rotation of ions was affected by them [22–24]. When a 10-kHz voltage wave is applied to the cell, negative LCs is aligned parallel to the substrates and distributed randomly. However, the cell showed a low haze of 1.1% because the domains were not small enough for scattering when there was no turbulence. We set the operating frequency as 100 Hz, at which the operating voltage is lowest.

2.2. Ion-doped LC cell based on light scattering and absorption

The operating principle of the proposed light shutter is illustrated in Fig. 2. For light absorption by the LC cell, we use dichroic dye molecules because they are easily aligned along the LC alignment direction. When the polarization direction of the incident light is parallel to the absorption axis of the dye molecules, the incident light is strongly absorbed. Conversely, the incident light is weakly absorbed when the direction of polarization is perpendicular to the absorption axis [1,10]. For light scattering via the electro-hydrodynamic effect, we doped the LC mixture with an ionic material (tetra-n-butylammonium bromide, TBAB).

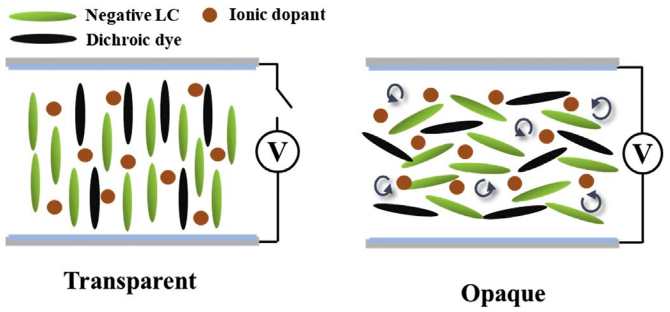

Fig. 2. Structure and operation of an ion-doped LC cell in the transparent and opaque states.

In the initial transparent state, most of the incident light passes through the LC cell because the LC and dye molecules are aligned perpendicular to the substrates. When a 100-Hz voltage wave is applied to the LC cell, the negative LC and dye molecules try to orient perpendicular to the vertical electric field. At the same time, rotation of ions caused by the electro-hydrodynamic effect brings about turbulence, which distributes LCs and dye molecules randomly in planes parallel to the substrates. In this state, the incident light is strongly absorbed because most of the dye molecules are oriented parallel to the substrates and scattered strongly by randomly distributed negative LCs with small domains. Using this scheme, an ion-doped LC cell can

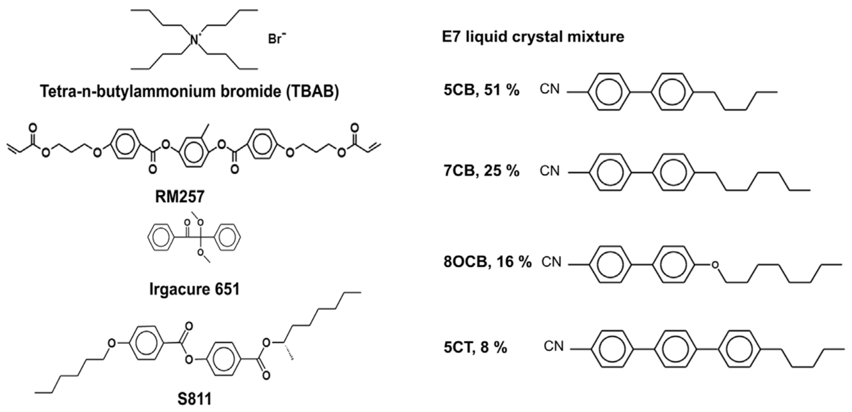

Fig. 3. Structure of the ionic material, monomer, photo-initiator, and positive LC mixture used for the fabrication of an ion-doped LC cell.

provide an excellent opaque state without a trade-off between light absorption and scattering.

3. Cell fabrication

To investigate the electro-optic characteristics, we fabricated an ion-doped LC cell. We mixed negative LCs (BHR28300-400, Bayi, Δn: 0.230 and $\Delta\varepsilon$: -9.3) with 0.1 wt% of TBAB (Sigma–Aldrich, the structure is shown in Fig. 3) for the electro-hydrodynamic effect and 1.5 wt% of dichroic dye for light absorption. We used a dichroic dye mixture consisting of X12 (BASF) and S-428 (Mitsui) for a ratio of 7:3. We used the homeotropic alignment layer (SE-5662, Nissan Chemical) and a cell gap of 10 µm for the LC cell.

To compare the transmission characteristics of an ion-doped LC cell with other LC light shutters, we fabricated polymer-stabilized LC (PSLC) and cholesteric LC (ChLC) cells [17,20]. For the same characteristics in the transparent state, we used the same cell gap, dichroic dye mixture, and concentration.

For the PSLC cell, we mixed negative LCs (RTA93000-100, HCCH, Δn: 0.200 and $\Delta\varepsilon$: -5.5) with 1.6 wt% of ultraviolet (UV) curable monomer (RM257, Merck, the structure is shown in Fig. 3), 0.4 wt% of photo-initiator, (Irgacure 651, BASF, the structure is shown in Fig. 3) and 1.5 wt% of the dichroic dye mixture. We used the same homeotropic alignment layer for the initial transparent state. Then, the LC cell was exposed to UV light of 30 mW/cm^2 for 10 min to form the polymer structure.

For the ChLC cell, we mixed positive LCs (E7, Merck, Δn: 0.237 and $\Delta\varepsilon$: 14.1, the structure is shown in Fig. 3) with 10 wt% of a chiral dopant (S811, Merck, reflection wavelength = 1600 nm, number of pitches: 10) and 1.5 wt% of the dichroic dye mixture. We used a homogeneous alignment layer (PIA-5310, Nissan Chemical) and rubbed in the anti-parallel direction.

4. Experimental results and discussion

To evaluate the electro–optic characteristics of the fabricated LC cells, we measured the total transmittance, specular transmittance, and haze using a haze meter (HM-65W, Murakami Color Research Laboratory) while changing the frequency of the applied voltage wave because the electro-hydrodynamic effect strongly depends on the frequency of the applied voltage wave.

Fig. 4 shows the measured total transmittance, specular transmittance, and haze of an ion-doped LC cell. In the initial transparent state, the total transmittance, specular transmittance, and haze values of the ion-doped LC cell were 61.2%, 60.1%, and 0.7%, respectively. When a 100-Hz voltage wave was applied to the cell, the total transmittance and specular transmittance decreased, and haze increased as the amplitude of the applied voltage wave was increased because the negative LC and dye molecules were randomly oriented parallel to the substrates

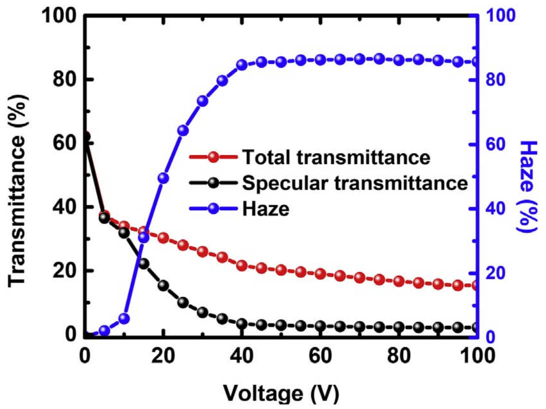

Fig. 4. Measured total transmittance, specular transmittance, and haze of an ion-doped LC cell.

by the electro-hydrodynamic effect.

We verified the trade-off between the scattering and absorption in the opaque state by measuring the transmission characteristics of an ion-doped LC cell and other light shutters. The measured specular transmittance can be influenced not only by light absorption but also light scattering, whereas the measured total transmittance can be influenced only by light absorption.

As shown in Fig. 5, the fabricated LC cells exhibited almost the same specular transmittance in the transparent state because we used the same dye mixture and concentration. In the opaque state, the ion-doped LC cell exhibited the lowest specular transmittance among the fabricated LC cells. A ChLC cell in the planar state exhibited a relatively high

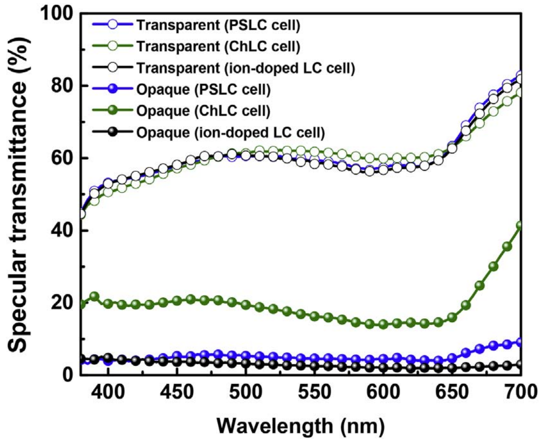

Fig. 5. Measured transmission spectra in the transparent and opaque states of ChLC, PSLC, and ion-doped LC cells.

Table 1
Total transmittance, specular transmittance, and haze values of ChLC, PSLC, and ion-doped LC cells.

	Transparent state			Opaque state		
	ChLC cell (homeotropic 45 V)	PSLC cell	Ion-doped LC cell	ChLC cell (planar)	PSLC cell (50 V)	Ion-doped LC cell (40 V)
Total transmittance (%)	60.9	62.9	61.2	18.9	31.4	20.8
Specular transmittance (%)	60.5	60.8	60.1	18.7	5.3	3.0
Haze (%)	0.7	4.3	0.7	1.1	82.7	85.6

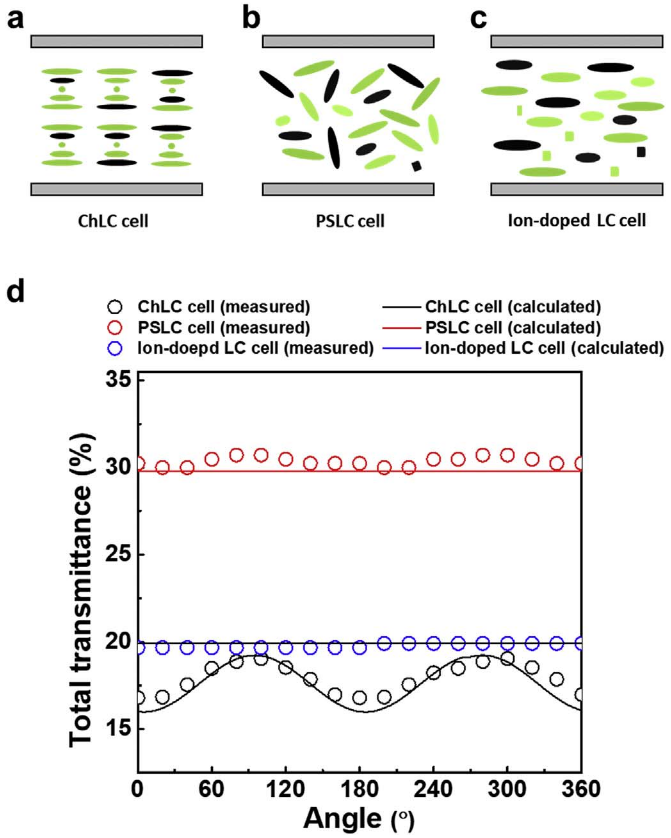

Fig. 6. LC and dye distributions in (a) ChLC, (b) PSLC, and (c) ion-doped LC cells assumed for the numerical calculation and (d) the angular dependence of the total transmittance measured or calculated using a polarized light source. The angle 0° is defined as the rubbing direction in a ChLC cell.

specular transmittance because it does not rely on light scattering.

However, the total transmittance of the fabricated cells in the opaque state shows a tendency different from the specular transmittance, as shown in Table 1. Among the fabricated LC cells, the ChLC cell exhibited the lowest total transmittance, whereas the PSLC cell exhibited the highest total transmittance. The total transmittance of an ion-doped LC cell is similar to that of a ChLC cell, whereas the haze value is similar to that of a PSLC cell.

We expect that an ion-doped LC cell strongly absorbs the incident light because of the negative LCs and dye molecules that are aligned parallel to the two substrates and strongly scatters the incident light via the electro-hydrodynamic effect. The operating voltage (40 V) of the ion-doped LC cell was lower than those of the PSLC (50 V) and ChLC (45 V) cells.

To investigate the distributions of the LC and dichroic dye molecules in the opaque state, we measured the total transmittance using a haze meter while rotating the polarization angle of the light source. For comparison, we calculated the distributions of the LC and dye by using the commercial software Techwiz LCD 1D (Sanayi System Company, Ltd.). For numerical calculations, we assumed that all the LC and dye molecules in the ChLC cell were aligned in planes parallel to the substrates, as shown in Fig. 6(a). We assumed that the LC and dye molecules in the PSLC cell were oriented randomly, as shown in Fig. 6(b). We assumed that the LC and dye molecules in the ion-doped LC cell were distributed randomly but that all of them had a tilt angle of 0°, as shown in Fig. 6(c). As shown in Fig. 6(d), the calculated results match the experimental results very well. The angular dependence of the total transmittance in the ChLC cell is caused by the wave-guiding effect [10].

The ChLC cell exhibited the lowest transmittance regardless of the polarization angle because all the LC and dye molecules were aligned parallel to the substrates, and it had a twisted structure in the planar state. The PSLC cell exhibited the highest transmittance among all the fabricated LC cells because most of the LC and dye molecules were not aligned parallel to the substrates. The ion-doped LC cell exhibited a slightly higher transmittance than the ChLC cell because all the LC and dye molecules were not oriented parallel to the substrates.

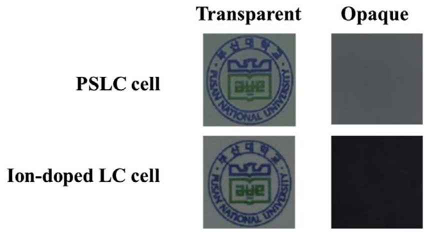

Fig. 7. Images of PSLC and ion-doped LC cells placed on a printed paper.

Fig. 7 shows the images of PSLC and ion-doped LC cells placed on a printed paper. We can identify the printed images clearly in the transparent state of both types of LC cells. In the opaque state, both types of cells could hide objects behind them. However, the ion-doped LC cell exhibited a far darker state than the PSLC cell because of the lower total transmittance.

5. Conclusion

We demonstrated an LC cell that can provide a very low transmittance in the opaque state. To realize light absorption and the scattering effect simultaneously, we doped the LC with TBAB and dichroic dye. In the opaque state, an ion-doped LC cell can have a very low total transmittance because most of the dye molecules are oriented nearly parallel to the substrates; thus, we can achieve an excellent opaque state. Moreover, the cell can be fabricated without a UV curing process. We consider the ion-doped LC cell an excellent new candidate for see-through display and smart-window applications.

Acknowledgement

This work was supported by the National Research Foundation of Korea (NRF) grant funded by the Korean government (MSIP) (No. 2017R1A2A1A0500106).

References

[1] Heilmeier GH, Zanoni LA. Guest-host interactions in nematic liquid crystals. a new electrooptic effect. Appl Phys Lett 1968;13(3):91–2.
[2] Doane JW, Vaz NA, Wu BG, Žumer S. Field controlled light scattering from nematic microdroplets. Appl Phys Lett 1986;48(4):269–71.
[3] Drzaic PS. Polymer dispersed nematic liquid crystal for large area displays and light valves. J Appl Phys 1986;60(6):2142–8.
[4] Hikmet RAM. Electrically induced light scattering from anisotropic gels. J Appl Phys 1990;68(9):4406–12.
[5] Yang D-K, West JL, Chien L-C, Doane JW. Control of reflectivity and bistability in displays using cholesteric liquid crystals. J Appl Phys 1994;76(2):1331–3.
[6] Lin Y-H, Yang J-M, Lin Y-R, Jeng S-C, Liao C-C. A polarizer-free flexible and reflective electro-optical switch using dye-doped liquid crystal gels. Opt Express 2008;16(3):1777–85.
[7] Fuh Y-G, Chen C-C, Liu C-K, Cheng K-T. Polarizer-free, electrically switchable and optically rewritable displays based on dye-doped polymer-dispersed liquid crystals. Opt Express 2009;17(9):7088–94.
[8] Lee GH, Hwang KY, Jang JE, Jin YW, Lee SY, Jung JE. Characteristics of color optical shutter with dye-doped polymer network liquid crystal. Opt Lett 2011;36(5):754–6.
[9] Wang C-T, Lin T-H. Bistable reflective polarizer-free optical switch based on dye-doped cholesteric liquid crystal. Opt Mater. Express 2011;1(8):1457–62.
[10] Yu B-H, Huh J-W, Kim K-H, Yoon T-H. Light shutter using dichroic-dye-doped long-pitch cholesteric liquid crystals. Opt Express 2013;21(24):29332–7.
[11] Huh J-W, Yu B-H, Heo J, Yoon T-H. Double-layered light shutter using long-pitch cholesteric liquid crystal cells. Appl Opt 2015;54(12):3792–5.
[12] Heo J, Huh J-W, Yoon T-H. Fast-switching initially-transparent liquid crystal light shutter with crossed patterned electrodes. AIP Adv 2015;5(4):047118.
[13] Yu B-H, Huh J-W, Heo J, Yoon T-H. Simultaneous control of haze and transmittance using a dye-doped cholesteric liquid crystal cell. Liq Cryst 2015;42(10):1460–4.
[14] Kim M, Park KJ, Seok S, Ok JM, Jung H-T, Choe J, et al. Fabrication of microcapsules for dye-doped polymer-dispersed liquid crystal-based smart windows. ACS Appl Mater. Interfaces 2015;7(32):17904–9.
[15] Huh J-W, Ji S-M, Heo J, Yu B-H, Yoon T-H. Bistable light shutter using dye-doped cholesteric liquid crystals with crossed patterned electrodes. J Disp Technol 2016;12(8):779–83.
[16] Oh S-W, Baek J-M, Heo J, Yoon T-H. Dye-doped cholesteric liquid crystal light shutter with a polymer-dispersed liquid crystal film. Dyes Pigments 2016;134:36–40.
[17] Moheghi A, Nemati H, Li Y, Li Q, Yang D-K. Bistable salt doped cholesteric liquid crystals light shutter. Opt Mater 2016;52:219–23.
[18] Cheng K-T, Lee P-Y, Qasim MM, Liu C-K, Cheng W-F, Wilkinson TD. Electrically switchable and permanently stable light scattering modes by dynamic fingerprint chiral texture. ACS Appl Mater. Interfaces 2016;8(16):10483–93.
[19] Huh J-W, Yu B-H, Heo J, Ji S-M, Yoon T-H. Technologies for display application of liquid crystal light shutters. Mol Cryst Liq Cryst 2017;644(1):120–9.
[20] Choi T-H, Huh J-W, Woo J-H, Kim J-H, Jo Y-S, Yoon T-H. Switching between transparent and translucent states of a two-dimensional liquid crystal phase grating device with crossed interdigitated electrodes. Opt Express 2017;25(10):11275–82.
[21] Choi T-H, Woo J-H, Baek J-M, Choi Y, Yoon T-H. Fast control of haze value using electrically switchable diffraction in a fringe-field switching liquid crystal device. IEEE Tans Electron Dev 2017;64(8):3213–8.
[22] Heilmeier GH, Zanoniand LA, Barton LA. Dynamic scattering: a new electrooptic effect in certain classes of nematic liquid crystals. IEEE Proc 1968;56(7):1162–71.
[23] Heilmeier GH, Zanoni LA, Barton LA. Further studies of the dynamic scattering mode in nematic liquid crystals. IEEE T. Electron Dev 1970;17(1):22–6.
[24] Wang H, Wang L, Xie H, Li C, Guo S, Wang M, et al. Electrically controllable microstructures and dynamic light scattering properties of liquid crystals with negative dielectric anisotropy. RSC Adv 2015;5:33489–95.
[25] Williams R. Domains in liquid crystals. J Chem Phys 1963;39:384–8.

主　题:	RE: Reprint Permissions	
发件人:	"Optica Publishing Group Copyright" <copyright@osa.org>	2022-10-25 22:35:18
收件人:	"张锐聪" <ruicong_zhang@stu.hit.edu.cn>, "Optica Publishing Group Copyright" <copyright@osa.org>	

Dear Dr. Zhang,

Thank you for contacting Optica Publishing Group.

For the use of material from Chun-Wei Chen, Alyssa N. Brigeman, Tsung-Jui Ho, and Iam Choon Khoo, "Normally transparent smart window based on electrically induced instability in dielectrically negative cholesteric liquid crystal," Opt. Mater. Express 8, 691-697 (2018):

> There are only 5 figures in this article. Please let us know if you would like to reproduce one of these figures.

For the use of figure 2 from Ghadah H. Sheetah, Qingkun Liu, Bohdan Senyuk, Blaise Fleury, and Ivan. I. Smalyukh, "Electric switching of visible and infrared transmission using liquid crystals co-doped with plasmonic gold nanorods and dichroic dyes," Opt. Express 26, 22264-22272 (2018), figure 1 from Bing-Xiang Li, Rui-Lin Xiao, Sathyanarayana Paladugu, Sergij V. Shiyanovskii, and Oleg D. Lavrentovich, "Dye-doped dual-frequency nematic cells as fast-switching polarization-independent shutters," Opt. Express 27, 3861-3866 (2019) and figure 5 from P. Lakshmi Madhuri, Saranya Bhupathi, S. Shuddhodana, Zaher M. A. Judeh, Sheng-Hsiung Yang, Yi Long, and Ibrahim Abdulhalim, "Hybrid vanadium dioxide-liquid crystal tunable non-reciprocal scattering metamaterial smart window for visible and infrared radiation control," Opt. Mater. Express 11, 3023-3037 (2021):

> Optica Publishing Group considers your requested use of its copyrighted material to be Fair Use under United States Copyright Law. We request that a complete citation of the original material be included in any publication.

> As this article is published under the terms of the Optica Publishing Group Open Access Publishing Agreement, when adapting or otherwise creating a derivative version of the article, users must maintain attribution to the author(s) and the published article's title, journal citation, and DOI. Users should also indicate if changes were made and avoid any implication that the author or Optica Publishing Group endorses the use.

While your publisher should be able to provide additional guidance, we prefer the below citation formats:

> For citations in figure captions:

> [Reprinted/Adapted] with permission from [ref #] © The Optical Society. (Please include the full citation in your reference list)

> For images without captions:

> Journal Vol. #, first page (year published) An example: Opt. Express 27, 1164 (2019)

Please let me know if you have any questions.

Kind Regards,

Hannah Greenwood

Hannah Greenwood

October 25, 2022

Authorized Agent, Optica Publishing Group

OPTICA PUBLISHING GROUP | Formerly OSA

From: 张锐聪 <ruicong_zhang@stu.hit.edu.cn>
Sent: Wednesday, October 19, 2022 8:18 AM
To: Optica Publishing Group Copyright <copyright@osa.org>
Subject: Reprint Permissions

ear Editor,

Hello! We would like to apply for permission to reproduce images from papers published by your publisher, as detailed below.

Title : Electric switching of visible and infrared transmission using liquid crystals co-doped with plasmonic gold nanorods and dichroic dyes
Author : Ghadah H. Sheetah, Qingkun Liu, Bohdan Senyuk, Blaise Fleury, and Ivan. I. Smalyukh
Volume, issue, and page numbers: 26, 22264-22272 (2018)
Journal : Optics Express
Expected content to be reproduced : Fig.2(a), Fig.2(e) and Fig.2(f)

Special request: We hope to allow us to rearrange these pictures, as shown below.
Original picture:

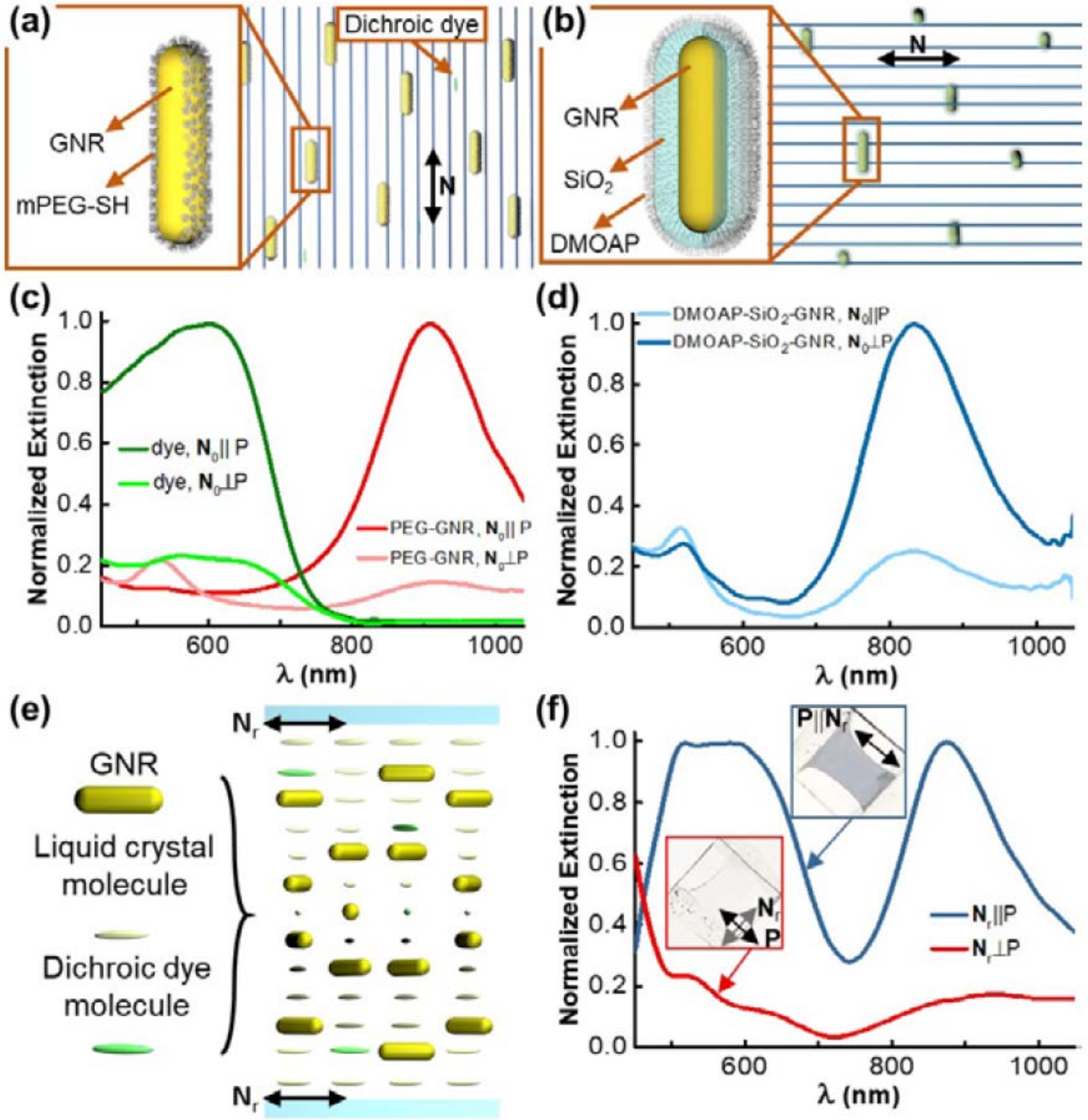

Fig. 2. Unidirectionally aligned and twisted structures of LC doped with GNRs and dichroic dye molecules. (a) Schematic of GNRs capped with mPEG-SH following **N** (blue lines) in a uniformly aligned LC; the inset shows details of surface functionalization of GNRs. (b) Schematic diagram of DMOAP-SiO_2-GNRs that exhibit self-alignment perpendicular to **N**; the inset shows details of silica shells around GNRs and DMOAP surface functionalization. (c,d)

Our contributions:

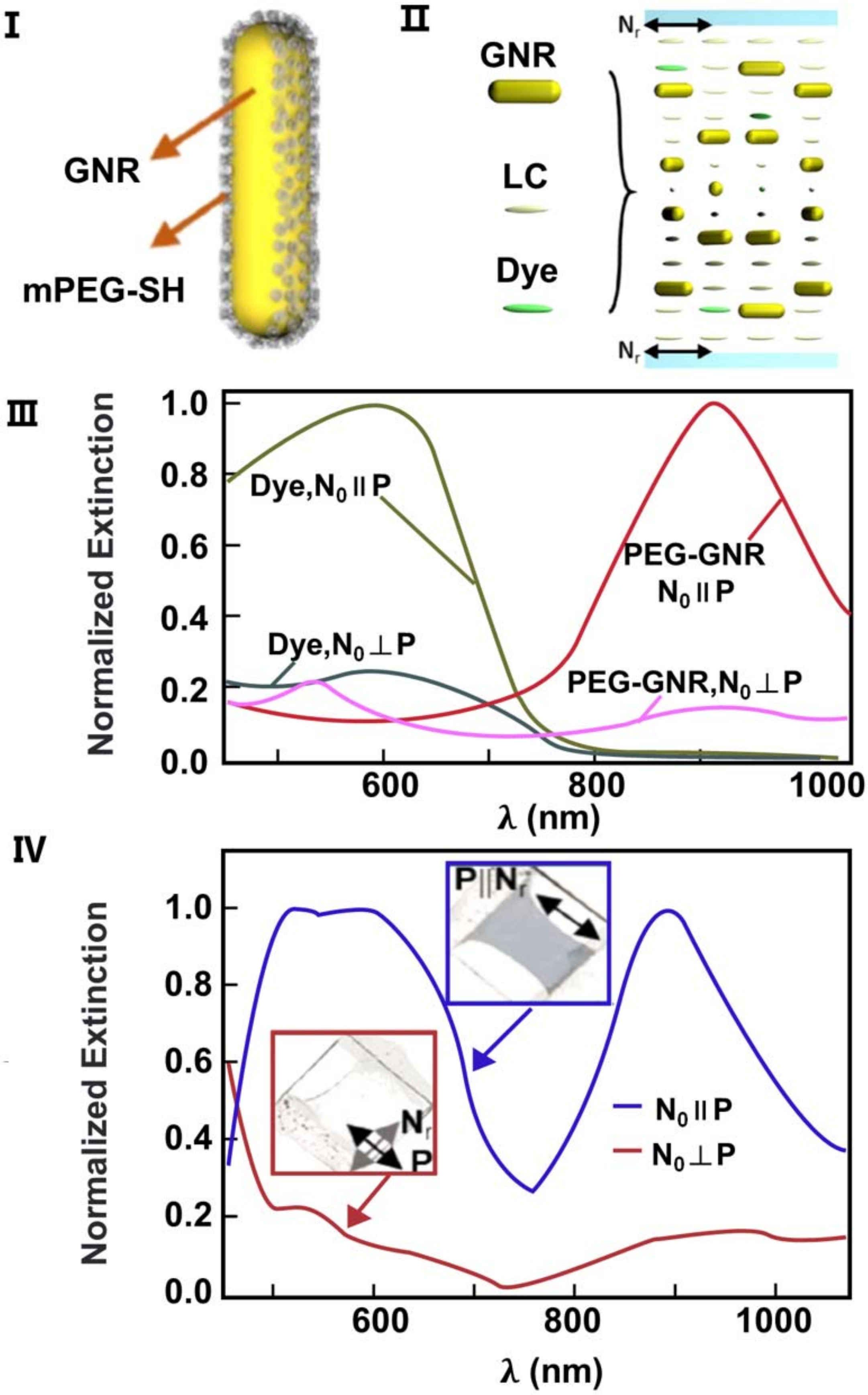

Information on the papers we plan to publish is as follows.
Title : Advanced liquid crystal-based switchable optical devices for light protection applications: principles and strategies
Publisher : Springer Nature
Journal : Light: Science & Applications
Estimated publication time : In November 2022

Main author of the article : Ruicong Zhang, Jiajun li, Zhibo Zhang, Jiecai Han, Lei Yang, Tianyu Wang, Jiaqi Zhu

We admire your society's contribution to the Institute of Optics and hope to obtain permission to reprint images.

With thanks!

Ruicong Zhang

Harbin Institute of Technology

主　题:	Reprint Permissions	
发件人:	"张锐聪" <ruicong_zhang@stu.hit.edu.cn>	2022-10-20 14:51:17
收件人:	copyright@osa.org	

Dear Editor,

Hello! We would like to apply for permission to reproduce images from papers published by your publisher, as detailed below.

Title : Dye-doped dual-frequency nematic cells as fast-switching polarization-independent shutters
Author : Bing-Xiang Li, Rui-Lin Xiao, Sathyanarayana Paladugu, Sergij V. Shiyanovskii, and Oleg D.Lavrentovich
Volume, issue, and page numbers: 27, 3861-3866 (2019)
Journal : Optics Express
Expected content to be reproduced : Fig.1
Special request: We hope to allow us to rearrange these pictures, as shown below.
Original picture:

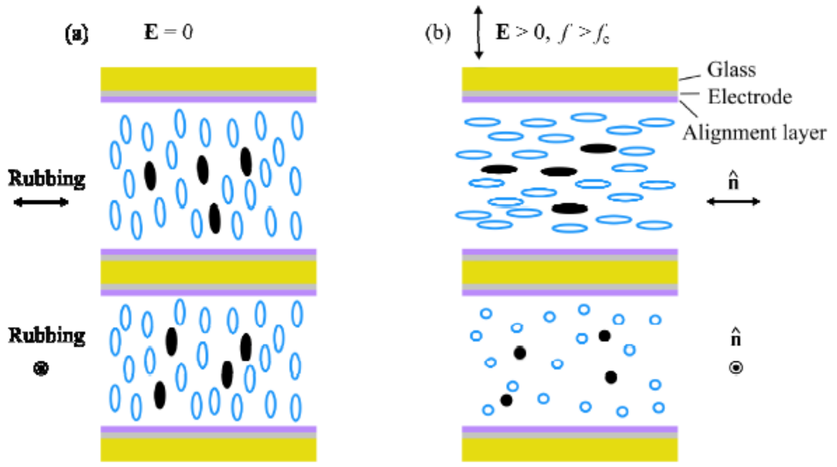

Fig. 1. Electro-optic shutter comprised of a pair of cells for polarization-independent light absorption. (a) Field-free state; the nearly homeotropic alignment of the dual frequency nematic doped with a dichroic dye makes the cells transparent to normally impinging light. The rubbing direction of two cells is perpendicular to each other. (b) Light-absorbing state is formed when a high-frequency electric field ($f > f_c$) is applied and realigns the director and dye molecules parallel to the bounding plates. The state is light-absorbing for all polarizations of light. The open and closed ellipsoids represent the nematic and dye molecules, respectively.

Our contributions:

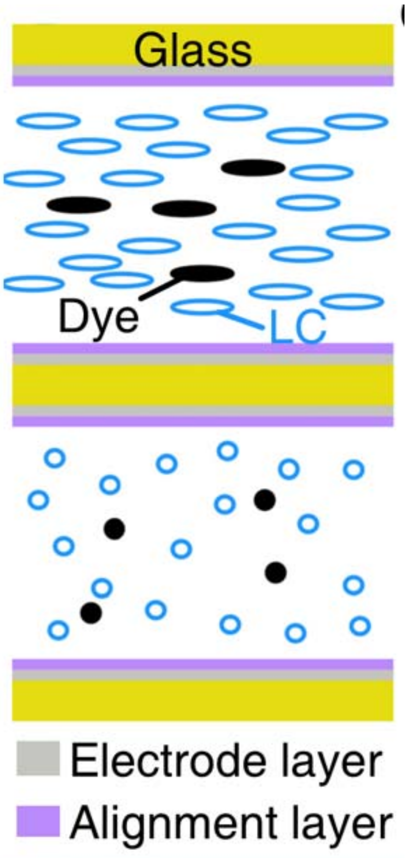

Information on the papers we plan to publish is as follows.
Title : Advanced liquid crystal-based switchable optical devices for light protection applications: principles and strategies
Publisher : Springer Nature
Journal : Light: Science & Applications
Estimated publication time : In November 2022

Main author of the article : Ruicong Zhang, Jiajun li, Zhibo Zhang, Jiecai Han, Lei Yang, Tianyu Wang, Jiaqi Zhu

We admire your society's contribution to the Institute of Optics and hope to obtain permission to reprint images.

With thanks!

Ruicong Zhang

Harbin Institute of Technology

Research Article | Vol. 27, No. 4 | 18 Feb 2019 | OPTICS EXPRESS 3861
Optics EXPRESS

Dye-doped dual-frequency nematic cells as fast-switching polarization-independent shutters

BING-XIANG LI,[1,2] RUI-LIN XIAO,[1,2] SATHYANARAYANA PALADUGU,[1] SERGIJ V. SHIYANOVSKII,[1] AND OLEG D. LAVRENTOVICH[1,2,3,*]

[1]*Advanced Materials and Liquid Crystal Institute, Kent State University, Kent, Ohio 44242, USA*
[2]*Chemical Physics Interdisciplinary Program, Kent State University, Kent, Ohio 44242, USA*
[3]*Department of Physics, Kent State University, Kent, OH 44242, USA*
**olavrent@kent.edu*

Abstract: We present polarization-independent optical shutters with a sub-millisecond switching time. The approach utilizes dual-frequency nematics doped with a dichroic dye. Two nematic cells with orthogonal alignment are driven simultaneously by a low-frequency or high-frequency electric field to switch the shutter either into a transparent or a light-absorbing state. The switching speed is accelerated via special short pulses of high amplitude voltage. The approach can be used in a variety of electro-optical devices.

© 2019 Optical Society of America under the terms of the OSA Open Access Publishing Agreement

1. Introduction

Anisotropic dielectric and optical properties of nematic liquid crystals (NLCs) enable a large number of electro-optical applications [1]. The dielectric anisotropy ($\Delta\varepsilon$) of an NLC determines the effect of the applied electric field $\mathbf{E}$ on the orientation of the NLC. The latter is characterized by a director $\hat{\mathbf{n}}$, which is also an optical axis of the NLC. The director aligns parallel or perpendicular to the applied electric field, depending whether $\Delta\varepsilon$ is positive or negative, respectively. The main issue of the nematic electro-optic effects is a slow relaxation time $\tau_{off} = \gamma d^2 / \pi^2 K$ when the field is switched off, typically on the order of milliseconds; here γ is the rotational viscosity, K is the elastic constant, and d is the cell thickness [1]. One of the effective approaches to accelerate the process is to use the so-called dual-frequency nematic liquid crystal (DFLC) in which $\Delta\varepsilon > 0$ at field frequencies below some cross-over frequency f_c and $\Delta\varepsilon < 0$ above f_c [2–6]. To achieve a sub-millisecond switching time, Golovin et al [2] and Yin et al [3] proposed to use a DFLC in a high-pretilt cell. In absence of the electric field, the director makes an angle θ about 45 degrees with the normal to the substrates. When the electric field is applied, this large tilt ensures a substantial realigning torque proportional to $\sin\theta\cos\theta$ and thus a faster response time as compared to cells with either planar, $\theta = \pi/2$, or homeotropic, $\theta = 0$, alignment.

In this work, we advance the previous dual-frequency approach to construct polarization-independent sub-millisecond shutters. Shuttering is caused by the guest-host effect [7] in the DFLC cells doped with dichroic dye molecules oriented along $\hat{\mathbf{n}}$. To achieve a polarization-independent switching, we use a sandwich-type structure comprised of two identical almost homeotropic cells, in which the small in-plane director projections are orthogonal to each other, Fig. 1. In the field-off state, absorption by the dichroic dye is weak, since $\hat{\mathbf{n}}$ in both cells is nearly perpendicular to any light polarization. To create a dark state, a high-frequency voltage is applied so that $\Delta\varepsilon < 0$ and the NLC and the dye molecules realign mostly parallel to the bounding plates along the mutually orthogonal directions in the two cells. In this state, the two-cells system absorbs light strongly for all polarizations of light. To accelerate the switching from the transparent state to the light-absorbing state, a special short pulse of high

#347540 https://doi.org/10.1364/OE.27.003861
Journal © 2019 Received 9 Oct 2018; revised 7 Nov 2018; accepted 4 Dec 2018; published 4 Feb 2019

amplitude and high frequency is applied prior to the high-frequency holding voltage. For a quick reverse switching, another special short pulse is used, this time of a direct current (DC) type. We achieve contrast ratio 10:1, with the transmittance changing from 48% ± 1% in the transparent state to 5% ± 1% in the dark state with the switching time in both directions being less than 0.3 ms.

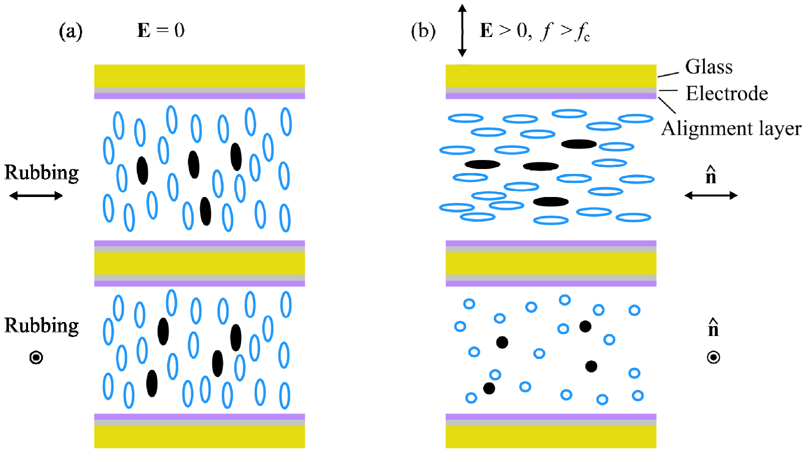

Fig. 1. Electro-optic shutter comprised of a pair of cells for polarization-independent light absorption. (a) Field-free state; the nearly homeotropic alignment of the dual frequency nematic doped with a dichroic dye makes the cells transparent to normally impinging light. The rubbing direction of two cells is perpendicular to each other. (b) Light-absorbing state is formed when a high-frequency electric field ($f > f_c$) is applied and realigns the director and dye molecules parallel to the bounding plates. The state is light-absorbing for all polarizations of light. The open and closed ellipsoids represent the nematic and dye molecules, respectively.

2. Experimental materials

We used homeotropic (alignment layer of polyimide SE1211, Merck) and planar (alignment layer of polyimide PI2555, HD MicroSystems) cells comprised of two glass plates with transparent indium tin oxide (ITO) electrodes of an active area $5\times5\ \text{mm}^2$. The thickness d of the cells was fixed by glass spheres of diameter 2-6 µm. The temperature was controlled using a Linkam LTS350 hot stage.

The switching speed of the shutter is determined by the properties of DFLCs such as rotational viscosity γ and $\Delta\varepsilon$. We explored three DFLCs, namely, DP002-016 and DP002-026 (Jiangsu Hecheng Display Technology), and MLC-2048 (EM Industries). MLC-2048 was ruled out since its viscosity, $\gamma = 200\ \text{mPa}\cdot\text{s}$, is four times larger than that of DP002-016 ($\gamma = 48\ \text{mPa}\cdot\text{s}$) and DP002-026 ($\gamma = 51\ \text{mPa}\cdot\text{s}$) at $25°\text{C}$. DP002-026 shows a crossover frequency that is significantly lower than that of DP002-016, Fig. 2(a), which implies that the cells can be driven by a lower-frequency voltages. We thus selected DP002-026 as the host material for shutters. Its dielectric permittivities, $\varepsilon_{\parallel}$ and $\varepsilon_{\perp}$, measured by using an LCR meter 4284A (Hewlett-Packard), in homeotropic and planar cells, respectively, are shown in Fig. 2(b).

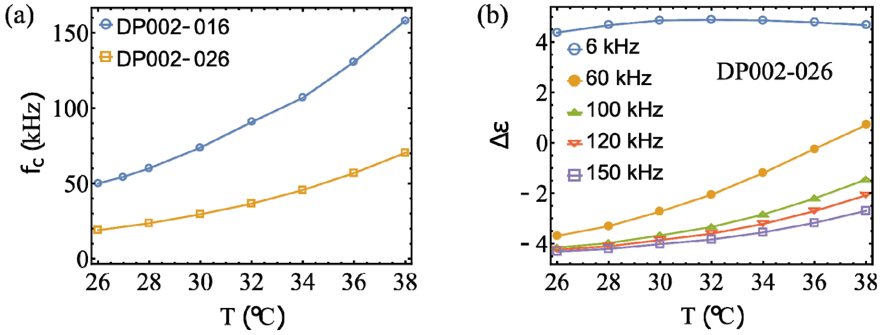

Fig. 2. Temperature dependence of (a) the crossover frequency of two studied materials, (b) dielectric anisotropy of DP002-026 at various frequencies.

The contrast ratio of the shutter is determined by the order parameter S of the dye. We characterized three dichroic dyes, Sudan III (Sigma-Aldrich), AB4 (Nematel GmbH), and G-472 (Mitsui Fine Chemicals) added to DP002-026 in planar cells of 4 μm thickness at the concentration $c = 2$ wt%. Each cell was probed with a linearly polarized He-Ne laser (543 nm for Sudan III, 632 nm for AB4 and G-472) at normal incidence. The transmittances of the dye- doped DFLCs are $T_{\parallel} = T_{cell} e^{-cd\alpha_{iso}(1+2S)}$ and $T_{\perp} = T_{cell} e^{-cd\alpha_{iso}(1-S)}$ for the light polarization parallel and perpendicular to the director, respectively; here α_{iso} is the absorption in the isotropic phase, and $T_{cell} \approx 0.9$ is the transmittance of a single cell filled with a dye-free DFLC. The order parameter is calculated from the transmission measurements, $S = \left[1 - 3\ln(T_{\perp}/T_{cell})/\ln(T_{\perp}/T_{\parallel})\right]^{-1}$, Table 1.

Table 1. Order parameter of three dichroic dyes in DP002-026.

Material	$T_{\parallel}$	$T_{\perp}$	S	Dye color
Sudan III	0.006	0.354	0.58	Red
AB4	0.045	0.600	0.68	Black
G-472	0.021	0.651	0.78	Black

The electro-optic cells comprising the proposed shutter shown in Fig. 1 are prepared with the homeotropic alignment layer SE1211 rubbed unidirectionally in order to provide a small directional tilt of $\hat{\mathbf{n}}$ when the electric field is absent. Each cell is assembled from pairs of plates rubbed in an antiparallel fashion. The tilt angle θ is 3.5 degrees, as determined by the crystal rotation method [8]. To achieve polarization independence for the shutter, two rubbed homeotropic cells of the same thickness are stacked such that the rubbing directions of cells were perpendicular to each other. In the field-free transparent state, the shutter transmittance is $T_{transparent} = T_{\perp}^2$, or $T_{transparent} = T_{cell}^2 \left(T_{\parallel}/T_{\perp}\right)^{2(1-S)/3S}$. The transmittance can be improved to $T_{transparent} = T_{cell} \left(T_{\parallel}/T_{\perp}\right)^{2(1-S)/3S}$ by using a matching fluid between the two cells. The shutter switches to an absorbing state with a low transmittance $T_{absorbing} = T_{\parallel} T_{\perp}$ when one applies a high-frequency voltage. In order to achieve $T_{transparent} = 0.55$ and the contrast ratio $T_{\perp}/T_{\parallel} = 10:1$, the order parameter should be $S \approx 0.76$ or higher, Fig. 3. Because of this, G-472 was chosen as a dye dopant, see Table 1.

Optics EXPRESS

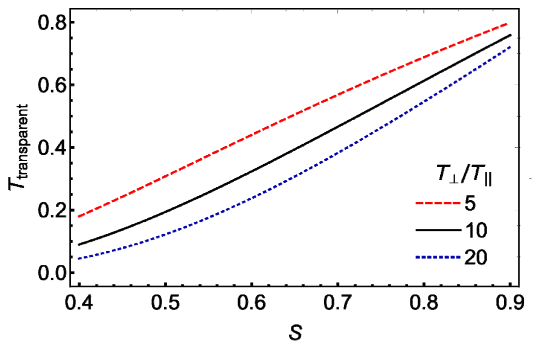

Fig. 3. Dependence of the required transmission $T_{transparent}$ at the transparent state on the minimum order parameter S and contrast ratio $T_{\perp} / T_{||}$.

3. Electro-optic performance of the shutter

To test the electro-optic performance, the cells were probed with a normally incident He-Ne laser beam (632 nm). The alternating current (AC) voltage was generated by a waveform generator (Stanford Research Systems, Model DS345) and an amplifier (Krohn-hite Corporation, Model 7602). We first characterize the performance of a single cell Fig. 4, and then the double-cell shutter, Fig. 5, filled with a mixture of DP002-026 and 2wt% of G-472.

In order to speed up the electro-optic switching, we designed a specific voltage waveform, Figs. 4 and 5. The response time for the field-induced reorientation of the nematic director is proportional to $1/E^2$ [1]. Thus, we introduced two short special pulses (SSPs) with a high voltage amplitude, Figs. 4(a), 4(b) and 5(a). The first SSP is applied to speed-up the formation of the dark planar state. This SSP is comprised of a DC pulse of duration 0.1 ms, followed by the AC pulse of frequency 60 kHz, Figs. 4(a) and 5(a). The dark state is held steady by a voltage of 7 V at 60 kHz. To switch from the dark to the transparent homeotropic state, the second SSP of a DC type was applied for 1.0 ms, after which the voltage was switched off, Figs. 4(b) and 5(a). Figures 4(c) and 4(d) show that the transmission changes are very fast, within at least ~0.2 ms, if one takes into account 0.1 ms delay caused by 0.1 ms DC pulse in the first SSP.

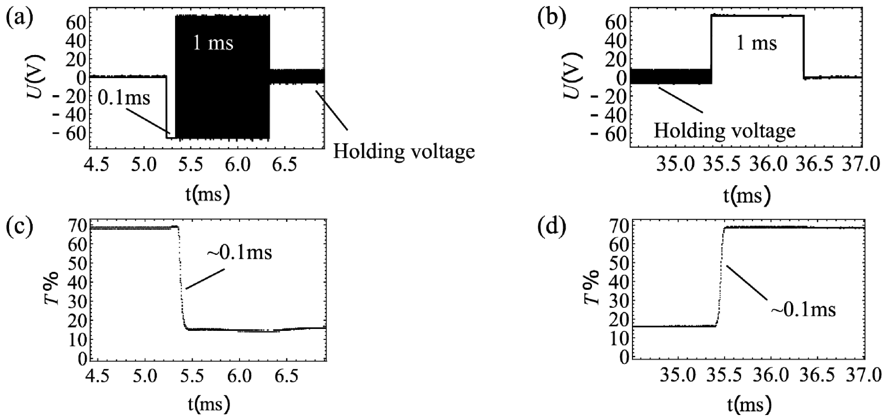

Fig. 4. Voltage waveform for transition (a) from the transparent to the dark state and (b) from the dark state to the transparent state and (c, d) the corresponding fast transmission changes measured for a single cell, $d = 5$ μm , filled with DP002-026 and G-472 mixture. The polarization of incident light is along the rubbing direction of the cell. The holding voltage at 60 kHz is 7 V.

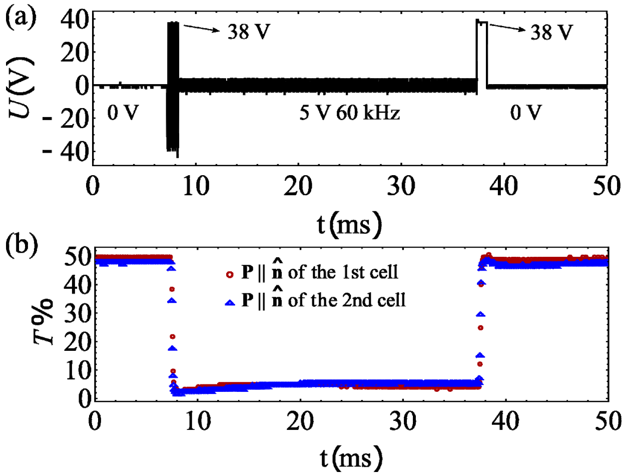

Fig. 5. (a) Voltage waveform and (b) the corresponding switching of transparency by the two-cell shutter filled with the mixture of DP002-026 and 2 wt% of G-472. The holding voltage for the dark state is 5 V at 60 kHz.

Figure 5 shows the overall performance of the shutter constructed from two cells, as in Fig. 1, each of a thickness 4.5 μm. The cells are assembled with the rubbing directions being perpendicular to each other. The gap between the cells is filled with the immersion oil (Olympus) of a refractive index 1.52. The waveform is similar to the one in Fig. 4, but with the two SSPs and the holding voltages being of a smaller amplitude, because of the smaller thickness of the cells used. The transmittance is switched from 48% ± 1% in the transparent state to 5% ± 1% in the light-absorbing state for any polarization of incident light. The switching times are less than 0.3 ms. Depositing antireflective coatings at the external surfaces of the cells would increase the transmittance by approximately 10%, preserving the same contrast.

The contrast ratio of the device, $T_{\perp}/T_{\|}=\exp(3cSd\alpha_{iso})$ can be enhanced by increasing the dye concentration c. Of course, in some cases an increase of c might lead to a decrease of S, but, as shown, for example, in Ref [9], the product cS generally increases with c. The increase of c also implies a decrease of $T_{transparent}$. If the latter is not a critical issue, and can be reduced, say, by a factor of 3, then the contrast ratio would reach 1000:1. The contrast ratio can also be enhanced significantly by increasing the thickness of the cells. In this work, we intentionally selected c = 2wt% of G-472 in the DP002-026 in order to achieve the transmittance $T_{\text{transparent}}$~50% of the shutter.

Another important parameter of fast switching shutters is their power consumption. To explore the issue, we consider the NLC cell as a series of a resistor R ($10\,\Omega/\square$) and a dielectric capacitor C. We determined the impedance in a cell of thickness ~4 μm and the active area $\mathrm{A}=2.5\times2.5\,\mathrm{cm}^2$ filled with DP002-026 and G-472. The results show that the magnitude and the phase of the impedance of the mixture at 60 kHz are $Z\approx450\,\Omega$ and $\phi\approx70°$, respectively. One can estimate the power for the holding voltage and the special short pulse at 60 kHz, i.e. $P_h=U_h^2\cos\phi/Z\approx0.02$ W and $P_S=U_S^2\cos\phi/Z\approx1.2$ W, where the typical holding voltage is $U_h\approx5$ V and the voltage of the special pulse is $U_S=40$ V. The energy consumed by special DC and AC pulses (that are very short in duration, ~0.3 ms) used to accelerate the switching, is less than 1 mJ. The power consumption of the display of a smart phone is typically ~0.5 W [10]. Thus, the power consumption should not be a problem for the practical application.

Optics EXPRESS

One of the potentially detrimental features of DFLC-based devices is dielectric heating. According to Refs [11,12], it increases the temperature of DFLC by $\Delta T \approx \pi f \varepsilon_0 \varepsilon'' U^2 / hd$, where $\varepsilon_0 = 8.85\times10^{-12}$ F/m is the vacuum permittivity, ε'' is the imaginary part of the permittivity of DFLCs, U is the applied voltage and h is the heat transfer coefficient of the surrounding medium. We measured the real and imaginary parts of permittivity of DP002-026 as $\varepsilon' \approx 8.8$ and $\varepsilon'' \approx 0.03$ at 60 kHz in the planar state at 28°C. With the heat transfer coefficient of the still air being $h = 16\ \mathrm{Wm^{-2}K^{-1}}$ [11,12], one can estimate that the temperature of DP002-026 in a cell with d = 4.5 μm will increase by 0.02°C and 1.1°C for the applied voltages 5 V and 40 V, respectively, at 60 kHz. The heating of electrodes can be estimated as $\Delta T = P / Ah \approx 2^{\circ} C$. Such a heating will not significantly change the dielectric anisotropy of the material and thus would not be detrimental to the shutter performance.

4. Conclusions

We demonstrate polarization-independent fast electro-optical switching of a two-cell shutter based on a dual-frequency nematic doped with a dichroic dye. The demonstrated electro-optic effect may be used in switchers, eyewear, and color filters. The response time is less than 0.3 ms for both transparent-dark and dark-transparent transitions. The power consumption and dielectric heating effects are not significant. The proposed optical scheme of the shutter can be implemented in other geometries. For example, the two homeotropic cells can be replaced by two planar cells with the director fields that are mutually perpendicular. In this geometry, the field-free state is dark. A low frequency field would realign the director in both cell homeotropically and the device would become transparent.

Funding

Valeo, Inc. (France).

Acknowledgment

The authors acknowledge useful discussions with Dr. Kedar Sathaye.

References

1. D.-K. Yang and S.-T. Wu, *Fundamentals of liquid crystal devices,* II ed. (John Wiley & Sons, 2014).
2. A. B. Golovin, S. V. Shiyanovskii, and O. D. Lavrentovich, "Fast switching dual-frequency liquid crystal optical retarder, driven by an amplitude and frequency modulated voltage," Appl. Phys. Lett. **83**(19), 3864–3866 (2003).
3. Y. Yin, M. Gu, A. Golovin, S. Shiyanovskii, and O. Lavrentovich, "Fast switching optical modulator based on dual frequency nematic cell," Mol. Cryst. Liq. Cryst. (Phila. Pa.) **421**(1), 133–144 (2004).
4. M. Mrukiewicz, P. Perkowski, W. Piecek, R. Mazur, O. Chojnowska, and K. Garbat, "Two-step switching in dual-frequency nematic liquid crystal mixtures," J. Appl. Phys. **118**(17), 173104 (2015).
5. X.-W. Lin, W. Hu, X.-K. Hu, X. Liang, Y. Chen, H.-Q. Cui, G. Zhu, J.-N. Li, V. Chigrinov, and Y.-Q. Lu, "Fast response dual-frequency liquid crystal switch with photo-patterned alignments," Opt. Lett. **37**(17), 3627–3629 (2012).
6. W. Duan, P. Chen, B.-Y. Wei, S.-J. Ge, X. Liang, W. Hu, and Y.-Q. Lu, "Fast-response and high-efficiency optical switch based on dual-frequency liquid crystal polarization grating," Opt. Mater. Express **6**(2), 597–602 (2016).
7. G. H. Heilmeier and L. A. Zanoni, "Gust-host interactions in nematic liquid crystals. A new electro-optic effect," Appl. Phys. Lett. **13**(3), 91–92 (1968).
8. K. Y. Han, T. Miyashita, and T. Uchida, "Accurate measurement of the pretilt angle in a liquid crystal cell by an improved crystal rotation method," Mol. Cryst. Liq. Cryst. (Phila. Pa.) **241**(1), 147–157 (1994).
9. A. Ranjkesh, J.-C. Choi, K.-I. Joo, H.-W. Park, M. S. Zakerhamidi, and H.-R. Kim, "Linear dichroism and order parameters of nematics doped with azo dyes," Mol. Cryst. Liq. Cryst. (Phila. Pa.) **647**(1), 107–118 (2017).
10. S. Tarkoma, M. Siekkinen, E. Lagerspetz, and Y. Xiao, *Smartphone Energy Consumption: Modeling and Optimization* (Cambridge University, 2014).
11. Y. Yin, S. V. Shiyanovskii, and O. D. Lavrentovich, "Electric heating effects in nematic liquid crystals," J. Appl. Phys. **100**(2), 024906 (2006).
12. Y.-C. Hsiao and W. Lee, "Lower operation voltage in dual-frequency cholesteric liquid crystals based on the thermodielectric effect," Opt. Express **21**(20), 23927–23933 (2013).

Mail QQ邮箱 mail.qq.com

RE: Reprint Permissions

发件人：copyright<copyright@osa.org>
时 间：2022年9月15日(星期四) 凌晨4:56
收件人：ruicong.zhang<ruicong.zhang@hrtcn.org>; copyright<copyright@osa.org>

Dear Dr. Zhang,

Thank you for contacting Optica Publishing Group.

For the use of figures 3 and 4 from Byeong-Hun Yu, Jae-Won Huh, Ki-Han Kim, and Tae-Hoon Yoon, "Light shutter using dichroic-dye-doped long-pitch cholesteric liquid crystals," Opt. Express 21, 29332-29337 (2013):

> Optica Publishing Group considers your requested use of its copyrighted material to be Fair Use under United States Copyright Law. We request that a complete citation of the original material be included in any publication.

While your publisher should be able to provide additional guidance, we prefer the below citation formats:

> For citations in figure captions:
>
> [Reprinted/Adapted] with permission from [ref #] © The Optical Society. (Please include the full citation in your reference list)
>
> For images without captions:
>
> Journal Vol. #, first page (year published) An example: Biomed. Opt. Express 6, 793 (2015)

Please let me know if you have any questions.

Kind Regards,

Hannah Greenwood

Hannah Greenwood

September 14, 2022

Authorized Agent, Optica Publishing Group

OPTICA PUBLISHING GROUP | Formerly OSA

From: ruicong.zhang <ruicong.zhang@hrtcn.org>
Sent: Monday, September 5, 2022 10:03 PM
To: Optica Publishing Group Copyright <copyright@osa.org>
Subject: Reprint Permissions

Dear Editor,

Hello! We would like to apply for permission to reproduce images from papers published by your publisher, as detailed below.

Title : Light shutter using dichroic-dye-doped long-pitch cholesteric liquid crystals
Author :Byeong-Hun Yu, Jae-Won Huh, Ki-Han Kim, and Tae-Hoon Yoon
Volume, issue, and page numbers: 21, 24,29332-29337 (2013)
Journal : Optics Express
Expected content to be reproduced : Fig.3 and Fig.4

Information on the papers we plan to publish is as follows.

Title :Advanced liquid crystal-based switchable optical devices for light protection application: principles and strategies
Publisher : Springer Nature
Journal : Light: Science & Applications
Estimated publication time : In November 2022
Main author of the article : Ruicong Zhang, Jiajun li, Zhibo Zhang, Jiecai Han, Lei Yang, Tianyu Wang, Jiaqi Zhu

We admire your society's contribution to the Institute of Optics and hope to obtain permission to reprint images.

With thanks!

Ruicong Zhang

Harbin Institute of Technology

ruicong.zhang

ruicong.zhang@hrtcn.org

Light shutter using dichroic-dye-doped long-pitch cholesteric liquid crystals

Byeong-Hun Yu, Jae-Won Huh, Ki-Han Kim, and Tae-Hoon Yoon*

Department of Electronics Engineering, Pusan National University, Busan 609-735, South Korea
*thyoon@pusan.ac.kr

Abstract: We propose a light shutter device using dichroic-dye-doped liquid crystals (LCs) whose Bragg reflection wavelength is set to be infrared by controlling the pitch of cholesteric liquid crystals (ChLCs). A dye-doped long-pitch ChLC cell is switchable between the dark planar state and the transparent homeotropic state. It has the advantages of high transmittance, low operation voltage, and an easy fabrication process relative to previous LC light shutter devices. The proposed light shutter device is expected to achieve high visibility for transparent organic light-emitting diode displays and emerging smart windows, which can be used in airplanes, cars, and other similar applications.

©2013 Optical Society of America

OCIS codes: (230.0230) Optical devices; (230.3720) Liquid-crystal devices.

References and links

1. C. W. Tang and S. A. VanSlyke, "Organic electroluminescent diodes," Appl. Phys. Lett. **51**(12), 913–915 (1987).
2. G. Gu, V. Bulović, P. E. Burrows, S. R. Forrest, and M. E. Thompson, "Transparent organic light emitting devices," Appl. Phys. Lett. **68**(19), 2606–2608 (1996).
3. J. W. Doane, N. A. Vaz, B. G. Wu, and S. Žumer, "Field controlled light scattering from nematic microdroplets," Appl. Phys. Lett. **48**(4), 269–271 (1986).
4. R. A. M. Hikmet, "Electrically induced light scattering from anisotropic gels," J. Appl. Phys. **68**(9), 4406–4412 (1990).
5. D.-K. Yang, J. L. West, L.-C. Chien, and J. W. Doane, "Control of reflectivity and bistability in displays using cholesteric liquid crystals," J. Appl. Phys. **76**(2), 1331–1333 (1994).
6. C. P. Chen, K.-H. Kim, T.-H. Yoon, and J. C. Kim, "A viewing angle switching panel using guest–host liquid crystal," Jpn. J. Appl. Phys. **48**(6), 062401 (2009).
7. H.-J. Jin, K.-H. Kim, H. Jin, J. C. Kim, and T.-H. Yoon, "Dye-doped liquid crystal device switchable between reflective and transmissive modes," J. Inf. Disp. **12**(1), 17–21 (2011).
8. A. Y.-G. Fuh, C.-C. Chen, C.-K. Liu, and K.-T. Cheng, "Polarizer-free, electrically switchable and optically rewritable displays based on dye-doped polymer-dispersed liquid crystals," Opt. Express **17**(9), 7088–7094 (2009).
9. Y.-H. Lin, H.-S. Chen, T.-H. Chiang, C.-H. Wu, and H.-K. Hsu, "A reflective polarizer-free electro-optical switch using dye-doped polymer-stabilized blue phase liquid crystals," Opt. Express **19**(3), 2556–2561 (2011).
10. Y.-H. Lin, J.-M. Yang, Y.-R. Lin, S.-C. Jeng, and C.-C. Liao, "A polarizer-free flexible and reflective electrooptical switch using dye-doped liquid crystal gels," Opt. Express **16**(3), 1777–1785 (2008).
11. G. H. Lee, K. Y. Hwang, J. E. Jang, Y. W. Jin, S. Y. Lee, and J. E. Jung, "Characteristics of color optical shutter with dye-doped polymer network liquid crystal," Opt. Lett. **36**(5), 754–756 (2011).
12. C.-T. Wang and T.-H. Lin, "Bistable reflective polarizer-free optical switch based on dye-doped cholesteric liquid crystal," Opt. Mater. Express **1**(8), 1457–1462 (2011).
13. K.-H. Kim, H.-J. Jin, K.-H. Park, J.-H. Lee, J. C. Kim, and T.-H. Yoon, "Long-pitch cholesteric liquid crystal cell for switchable achromatic reflection," Opt. Express **18**(16), 16745–16750 (2010).
14. K.-H. Kim, H.-J. Jin, D. H. Song, B.-H. Cheong, H.-Y. Choi, S. T. Shin, J. C. Kim, and T.-H. Yoon, "Switching of liquid-crystal devices between reflective and transmissive modes using long-pitch cholesteric liquid crystals," Opt. Lett. **35**(20), 3504–3506 (2010).

1. Introduction

Recently, transparent displays have drawn much attention as next-generation displays. In particular, transparent displays using organic light-emitting diodes (OLEDs) are being studied actively [1, 2]. However, since transparent OLEDs cannot provide the black color, they

#196303 - $15.00 USD Received 23 Aug 2013; accepted 23 Oct 2013; published 20 Nov 2013

exhibit poor visibility characteristics. This inevitable problem can be solved by placing a light shutter at the back of a transparent OLED display. In order to realize a light shutter, several methods have been proposed, including polymer dispersed liquid crystals (PDLC), LC gel, and cholesteric liquid crystal (ChLC) devices [3–5]. However, these devices cannot provide the black color, as they only switch between transparent and opaque states by the scattering effect. Therefore, such methods may not be suitable for achieving high visibility in a transparent OLED display.

In this work, we demonstrate a light shutter device using dye-doped LCs to achieve high visibility for a transparent OLED display. Dye-doped LC devices were developed and attract much attention because of their high transmittance and polarizer-free structure. Because of their dichroism, dye molecules strongly absorb the incident light polarized parallel to their absorption axes and weakly absorb the incident light polarized perpendicular to their absorption axes. Moreover, a dye-doped LC device is convenient for switching because dye molecules are easily aligned by LC molecules [6, 7].

Achieving a good dark state in dye-doped LC devices is not easy while operating in such modes as the vertical alignment (VA) mode and the electrically controlled birefringence (ECB) mode because dye molecules absorb only the light linearly polarized along the rubbing direction in the dark state. A dye-doped twisted nematic (TN) cell also shows a poor dark state in spite of its twisted structure, because it absorbs the light polarized along a specific direction owing to the wave-guiding effect. In order to obtain a dark state independent of the incident light polarization, several methods have been proposed, including the dye-doped PDLC, the dye-doped blue phase LC, and the dye-doped polymer-networked LC [8–12]. However, these methods have some disadvantages such as hysteresis, high operation voltage, low transmittance, and a complicated fabrication process owing to the in-cell monomer structure. Moreover, the addition of the UV curing process for photo-polymerization of a pre-polymer material can be a serious disadvantage in mass production.

The periodic structure of ChLCs in the planar state causes Bragg reflection, where the wavelength of the reflected light is determined by its pitch. Therefore, long-pitch ChLCs reflecting infrared are transparent in both planar and homeotropic states [13, 14]. In this paper, we propose a light shutter device in which dichroic dyes are doped to long-pitch ChLCs whose Bragg reflection wavelength is set to be infrared by controlling the pitch of ChLC. By doping dye molecules, we can achieve the dark planar state and the transparent homeotropic state. In contrast to the TN mode, the wave-guiding effect can be ignored owing to the periodically twisted structure and the small pitch of the ChLCs. The proposed light shutter device has the advantages of high transmittance and an easy fabrication process. A dye-doped long-pitch ChLC cell shows a good dark state regardless of the polarization direction of the incident light owing to the helical structure of the LC mixture in the planar state. Moreover, it is switchable at a much lower operating voltage than seen in previous studies. We expect that the proposed light shutter device can be applied to obtain high visibility for a transparent OLED and to applications such as emerging “smart windows” for airplanes, cars, et cetera.

2. Cell fabrication

Figure 1 shows the configuration of a dye-doped long-pitch ChLC cell. In the planar state, LCs and dye molecules have a periodic structure, as shown in Fig. 1(a). Dye molecules absorb the arbitrarily polarized light because they are twisted along the helical axis perpendicular to the substrate. Bragg reflection by ChLCs in the planar state can be ignored owing to the infrared reflection characteristics. In the homeotropic state, LCs and dye molecules are aligned perpendicularly to the substrate, allowing most of the arbitrarily polarized light to pass through, as shown in Fig. 1(b). The focal-conic state is not used in the proposed light shutter because light scattering by randomly distributed molecular domains results in poor visibility.

#196303 - $15.00 USD Received 23 Aug 2013; accepted 23 Oct 2013; published 20 Nov 2013
(C) 2013 OSA

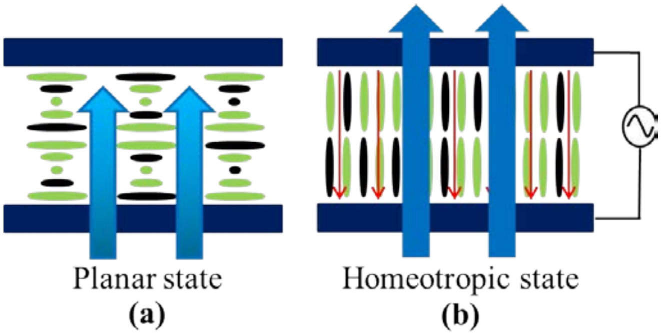

Fig. 1. Configuration of a dye-doped long-pitch ChLC cell, of which the textures are in (a) the planar (dark) and (b) the homeotropic (transparent) state.

To confirm the electro-optic characteristics of the proposed configuration, a dye-doped long-pitch ChLC cell was fabricated. The top and bottom indium-tin-oxide glass substrates are spin-coated with a homogeneous polyimide alignment material (AL16301K, JSR Micro Korea), and the cell-gap is maintained at 10 or 20 μm by using ball-type spacers. Positive nematic LCs (Δn: 0.159, Δε: 13.5) are mixed with a chiral material (S811, Merck). The mixing ratio is chosen so as to reflect infrared light of wavelength 2,000 nm. Two types of dyes, 2 wt% of S-428 (Mitsui) and 1 wt% of AC1 (Nematel), are doped to the LC mixture to obtain the black color. Then the LC mixture is stirred for 24 hours and an ultrasonic wave is applied to it for 3 hours.

3. Experimental results and discussion

The measured transmission spectra of the fabricated dye-doped long-pitch ChLC cells in the planar and homeotropic states are shown in Fig. 2. When the cell-gaps of the fabricated cells are 20 μm, the measured transmittance in the planar state is 3.7%. By applying 50 V, the cells can be switched to the homeotropic state, where measured transmittance is 42.3%. By lowering the cell-gap to 10 μm, the measured transmittance in the homeotropic state is increased to 58.2%. The voltage applied for switching to the homeotropic state is decreased to 35 V. The transmittance in the planar state also increases to 15.6%.

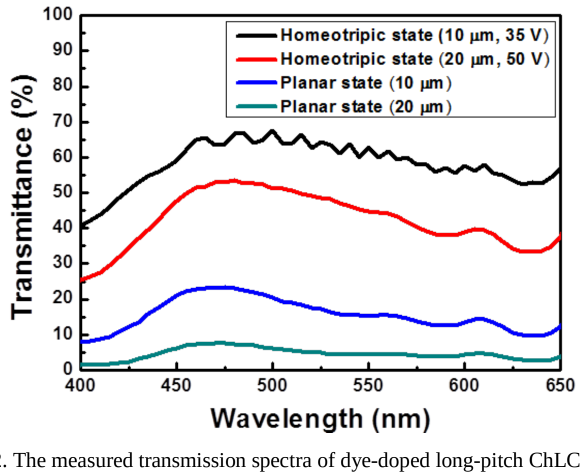

Fig. 2. The measured transmission spectra of dye-doped long-pitch ChLC cells.

We fabricated dye-doped LC cells operating in VA, ECB, and TN modes to confirm the effect of the helical structure of the LC mixture on transmittance in the dark state. Table 1 shows the measured electro-optic characteristics of dye-doped LC cells. Cell-gap (10 μm) and the amount of dyes are identical in all fabricated cells. The measured transmittances in the

#196303 - $15.00 USD Received 23 Aug 2013; accepted 23 Oct 2013; published 20 Nov 2013
(C) 2013 OSA

transparent state are nearly identical in all the fabricated cells due to the same amount of dyes and the equivalent arrangement of LC and dye molecules. However, the transmittance in the dark state of a dye-doped long-pitch ChLC cell is about 50% smaller than that of other dye-doped LC cells. Other dye-doped cells show a poor dark state because they only absorb a specific linearly polarized light. Owing to its helical structure, a dye-doped long-pitch ChLC cell absorbs incident light regardless of polarization direction; thus, it can provide a much better dark state than other LC cells.

Table 1. The measured electro-optic characteristics of dye-doped LC cells.

	VA	ECB	TN	Long-pitch ChLC
Bright state (%)	60.0	55.5	57.4	58.2
Dark state (%)	34.3	30.4	32.4	15.6
Operating voltage (V)	25	20	20	35

The proposed light shutter can absorb arbitrary polarized light because of the weak wave-guiding effect resulting from the small pitch. We fabricated test cells with a fixed amount of dyes and cell-gap to check the dependence of transmittance on pitch in the planar state. The measured transmittances versus the number of pitches in ChLC cells are shown in Fig. 3. The measured transmittances of the fabricated cells are nearly identical in the homeotropic state due to the same amount of dyes being used. However, as the number of pitches is increased, the dark state transmittance decreases. Additionally, the operating voltage increases owing to an increased amount of chiral material.

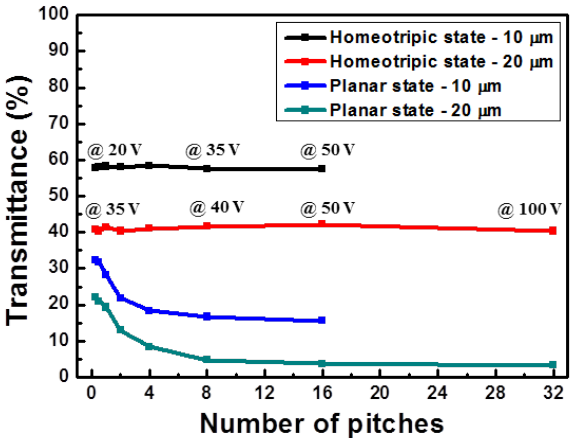

Fig. 3. The measured transmittance versus the number of pitches of dye-doped long-pitch ChLC cells.

We performed numerical calculations using the commercial software Techwiz LCD (Sanayi System Co., Ltd., Korea) to confirm the experimental results. The parameters for the numerical calculations are obtained from the experimental results and the LC parameters. The imaginary parts of ordinary and extraordinary refractive indices of the LC mixture used in the numerical calculation are 0.0012 and 0.014, respectively. Figure 4 shows the dependence of the measured and calculated transmittances in the planar state on the number of pitches. As number of pitches is increased, both measured and calculated dark state transmittances decrease with almost the same slope.

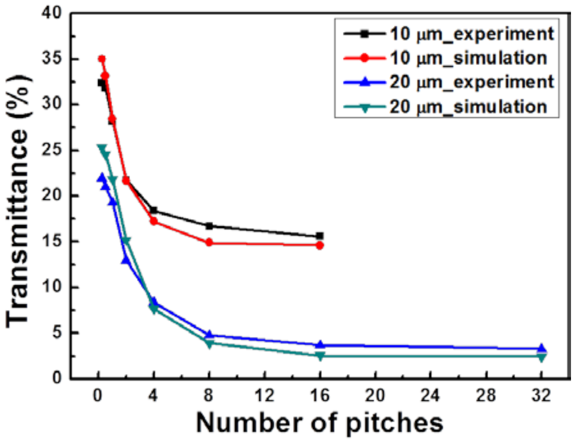

Fig. 4. The measured and calculated transmittances in the planar state vs. the number of pitches.

To investigate the reason for the decrease of transmittance in the planar state, we measured the transmittance of the fabricated 20 μm cells using a polarized light source as shown in Fig. 5. As the number of pitches is increased, the transmittance in the planar state decreases when the polarization of the incident light is perpendicular to the rubbing direction. When the polarization of the incident light is parallel with the rubbing direction, transmittance in the planar state increases slightly. These results show that the decrease of transmittance in the planar state is caused by the decrease of the wave-guiding effect. As the number of pitches is increased, a dye-doped long-pitch ChLC cell shows a much better dark state regardless of the polarization direction of the incident light.

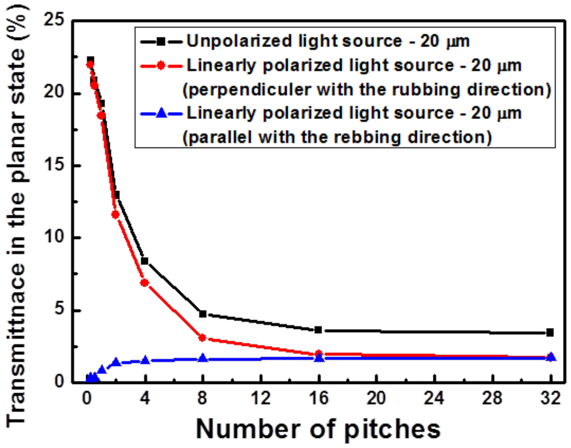

Fig. 5. The transmittance of a dye-doped long-pitch ChLC cell measured by using a linearly polarized light source.

Figure 6 shows photographs of a 20 μm dye-doped long-pitch ChLC cell in the planar and homeotropic states. The cell whose Bragg reflection wavelength is chosen to be 2,000 nm was placed on a backlight unit. In the planar state, the fabricated cell can provide a superior dark state because it absorbs the arbitrarily polarized light from a backlight unit. However, the homeotropic state is transparent because the incident light is allowed to pass through.

#196303 - $15.00 USD Received 23 Aug 2013; accepted 23 Oct 2013; published 20 Nov 2013

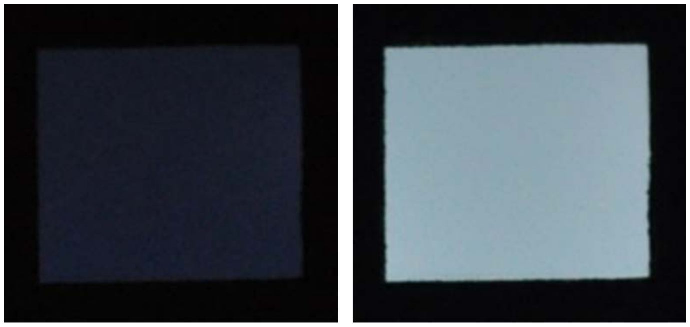

(a) (b)

Fig. 6. Photographs of a dye-doped long-pitch ChLC cell, of which the textures are in (a) the planar (dark) and (b) the homeotropic (transparent) state.

4. Conclusions

In summary, we demonstrated a light shutter device using dye-doped long-pitch ChLCs. It is switchable between the dark planar and transparent homeotropic states. Our experimental results show a high transmittance, a good dark state, and a low operating voltage. The proposed light shutter is suitable to achieve high visibility for a transparent OLED display. We expect that the proposed light shutter is applicable to emerging smart windows, which can be used in various applications.

Acknowledgments

This work was supported by the Global Leading Technology Program of the Office of Strategic R&D Planning (OSP) funded by the Ministry of Trade, Industry & Energy, Republic of Korea (10042412) and the National Research Foundation of Korea(NRF) grant funded by the Korea government (MSIP) (No. 2011-0029198).

111.42.148.36 {ts '2022-09-20 05:40:47'}
