# Supplementary material for: Advanced liquid crystal-based switchable optical devices for light protection applications: principles and strategies
Source: Light Sci Appl. 2023 Jan 3;12:11. doi: 10.1038/s41377-022-01032-y (PMC9807646; doi:10.1038/s41377-022-01032-y)
Supplement: Supplementary file 8 — Fig 8 copyright promotion [file 41377_2022_1032_MOESM8_ESM.pdf]

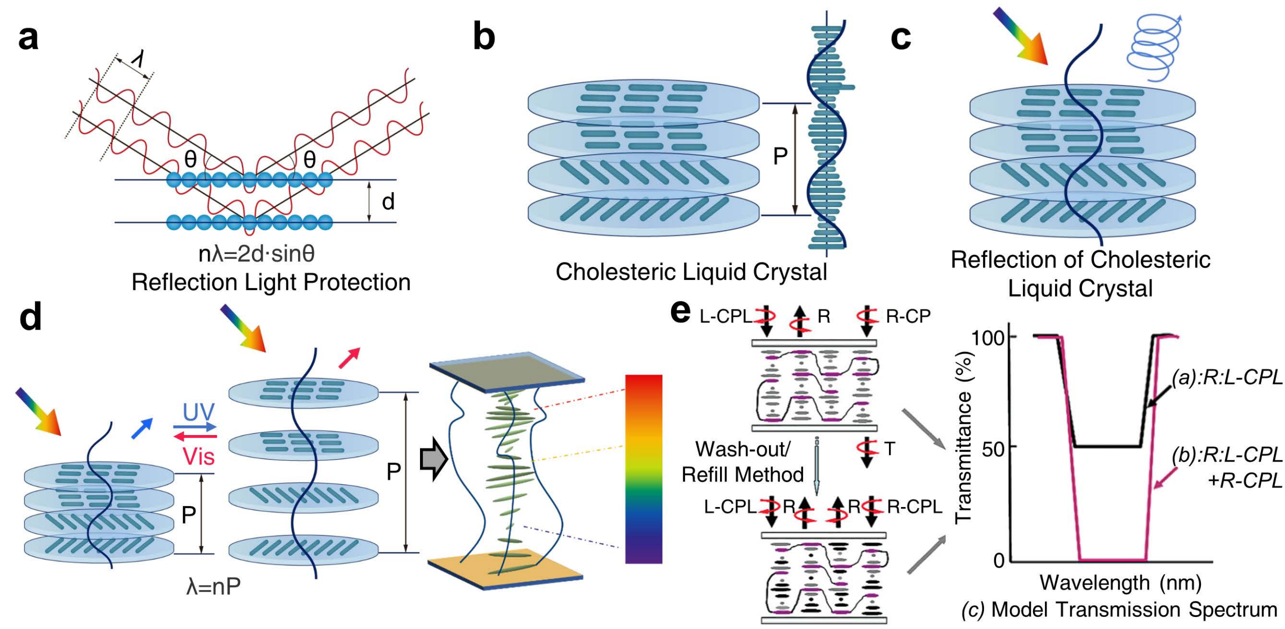
a
λ
θ
θ
d
nλ=2d·sinθ
Reflection Light Protection
b
P
Cholesteric Liquid Crystal
c
Reflection of Cholesteric Liquid Crystal
d
UV
Vis
P
P
λ=nP
e
L-CPL
R
R-CP
Wash-out/ Refill Method
T
L-CPL
R
R
R-CPL
Transmittance (%)
100
50
0
(a):R:L-CPL
(b):R:L-CPL +R-CPL
Wavelength (nm)
(c) Model Transmission Spectrum

RightsLink

Home | Help | Email Support | Ruicong Zhang

Effect of Network Concentration on the Performance of Polymer-Stabilized Cholesteric Liquid Crystals with a Double-Handed Circularly Polarized Light Reflection Band

Author: Jinbao Guo, Huai Yang, Rui Li, et al
Publication: The Journal of Physical Chemistry C
Publisher: American Chemical Society
Date: Sep 1, 2009

Copyright © 2009, American Chemical Society

PERMISSION/LICENSE IS GRANTED FOR YOUR ORDER AT NO CHARGE

This type of permission/license, instead of the standard Terms and Conditions, is sent to you because no fee is being charged for your order. Please note the following:

- Permission is granted for your request in both print and electronic formats, and translations.
- If figures and/or tables were requested, they may be adapted or used in part.
- Please print this page for your records and send a copy of it to your publisher/graduate school.
- Appropriate credit for the requested material should be given as follows: "Reprinted (adapted) with permission from {COMPLETE REFERENCE CITATION}. Copyright {YEAR} American Chemical Society." Insert appropriate information in place of the capitalized words.
- One-time permission is granted only for the use specified in your RightsLink request. No additional uses are granted (such as derivative works or other editions). For any uses, please submit a new request.

If credit is given to another source for the material you requested from RightsLink, permission must be obtained from that source.

BACK CLOSE WINDOW

© 2022 Copyright - All Rights Reserved | Copyright Clearance Center, Inc. | Privacy statement | Data Security and Privacy | For California Residents | Terms and ConditionsComments? We would like to hear from you. E-mail us at customercare@copyright.com

Effect of Network Concentration on the Performance of Polymer-Stabilized Cholesteric Liquid Crystals with a Double-Handed Circularly Polarized Light Reflection Band

Jinbao Guo,[†] Huai Yang,[‡] Rui Li,[†] Nan Ji,[†] Xiaoming Dong,[†] Hao Wu,[†] and Jie Wei*,[†]

College of Materials Science and Engineering, Beijing University of Chemical Technology, Beijing 100029, People's Republic of China, and Department of Materials Physics and Chemistry, School of Materials Science and Engineering, University of Science and Technology Beijing, Beijing 100083, People's Republic of China

Received: April 13, 2009; Revised Manuscript Received: July 31, 2009

Polymer-stabilized cholesteric liquid crystals (PSCLCs) with a double-handed circularly polarized reflection band are fabricated by a wash-out/refill method. By removing the low molar weight LCs from the original PSCLC film, desirable liquid crystals (LCs) can be infiltrated into a prefabricated polymer network. The results showed that the memory of the polymer network controls the resulting material properties. The concentration of the prefabricated polymer network also plays a relevant role in the formation of a single-layer cholesteric LC (Ch-LC) structure that has a clear-cut double-handed circularly polarized reflection band. A light-scattering phenomenon occurring in the system alters the reflection properties of Ch-LCs, which is due to the weak anchoring effect of the network when PSCLC film contains a low network concentration. Both kinds of circularly polarized reflection become more obvious with increase in the network concentration, followed by the strong anchoring effect of the network. The technique developed in this study has great applications in industries that require solid optical functional films and coatings.

Downloaded via HARBIN INST OF TECHNOLOGY on September 19, 2022 at 08:41:28 (UTC).
See https://pubs.acs.org/sharingguidelines for options on how to legitimately share published articles.

1. Introduction

Polymer−liquid crystal dispersions have wide applications in many fields, such as flat-panel displays and switchable optical elements. Therefore, they have attracted continuing interest and had been extensively studied in recent years.[1−6] Polymer-stabilized liquid crystals (PSLCs) are constructed by dispersing a small amount of photoreactive monomers into a liquid crystal (LC) with low molar mass. After ultraviolet (UV) light exposure, a polymer network is formed and interdispersed throughout the LC component. In most cases, the polymer network keeps the order of the LC environment from which it was formed originally.[4−6]

Because of the formation of a macroscopic helical structure, a cholesteric liquid crystal (Ch-LC) exhibits many remarkable optical properties. One of the great properties of the Ch-LC is its selective reflection of circularly polarized incident light.[7,8] Ch-LC with a single pitch selectively reflects light of a wavelength between $\lambda_{min} = Pn_o$ and $\lambda_{max} = Pn_e$. Here, n_o and n_e are the ordinary and extraordinary refractive indices of the locally uniaxial structure, respectively. The bandwidth of the selective reflection spectrum, $\Delta\lambda$, is given by $\Delta\lambda = \lambda_{max} - \lambda_{min} = (n_e - n_o)P = \Delta nP$, where $\Delta n = n_e - n_o$ is the birefringence. Modulating the characteristics of the reflection band (tuning λ_0 in the visible or infrared spectrum, broadening $\Delta\lambda$, or increasing the reflected light flux) is a practical goal that has driven numerous research efforts related to applications, that is, polarizer-free reflective displays (with no backlight requirement), polarizers and color filters, mirrorless lasing, or smart switchable reflective windows for the dynamical control of solar light.[9−30]

As is known to all, circularly polarized light with single handedness is reflected by a Ch-LC structure, which gives the maximum reflection efficiency of 50% at normal incidence for ambient (unpolarized) light. Therefore, Ch-LC films that can reflect both right- and left-circularly polarized light (R-CPL and L-CPL) simultaneously are attracting more and more attention.[25−30] For example, Mitov et al. have obtained a single-layer Ch-LC gel from a photopolymerizable monomer/Ch-LC composite that exceeds the 50% reflectance limit. The Ch-LC used in Mitov's research has characteristics of a thermally induced inversion of the helicity sense. After the composites are cured by UV light when the helix is right-handed, the reflectance turns out to be greater than 50% when measured at the temperature assigned for a cholesteric helix with the same pitch but a left-handed sense.[28,29]

We have reported that a single-layer polymer-stabilized cholesteric liquid crystal (PSCLC) film had been developed by a wash-out/refill method, which can reflect both R-CPL and L-CPL simultaneously.[30] The PSCLC film was achieved by prefabricating the polymer network with a left-handed helical structure and then refilling with the Ch-LCs with a right-handed helical structure into the network. The reflection intensity of the PSCLC film is close to 100% when the pitch lengths of the two opposite helical structures are the same. The results strongly suggested that memory effect brought from the polymer network controls the resulting film properties.

The purpose of this paper, based on the above fabrication method, is to discover the influence of network concentration on the performance of PSCLC film with a double-handed circularly polarized light reflection band. The polymer network in this study is mainly derived from nematic photopolymerizable monomer. The morphology of the polymer network of different systems was investigated in order to understand the relationship between the morphology of the polymer network and the

* To whom correspondence should be addressed. E-mail: weijie-2008@hotmail.com.
[†] Beijing University of Chemical Technology.
[‡] University of Science and Technology Beijing.

10.1021/jp903394r CCC: $40.75 © 2009 American Chemical Society
Published on Web 08/26/2009

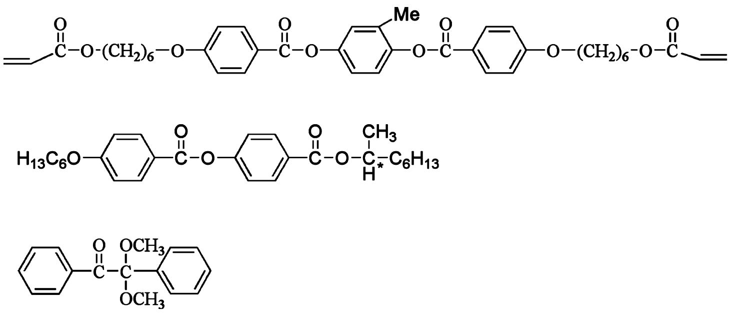

C6M

S811 (R811)

Irgacure 651

Figure 1. Chemical structures of the materials used.

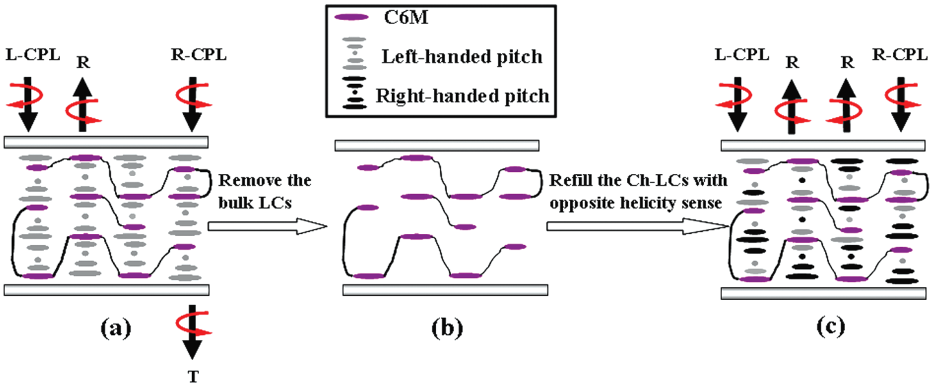

Figure 2. Schematic representation of the method to prepare the PSCLC film: R, reflection; T, transmission.

reflectance properties. Additionally, the memory effect of the polymer network in PSCLCs was further clarified in this paper.

2. Experimental Section

2.1. Materials. In this study, nonreactive LCs, SLC1717 (20 °C, 589 nm, $\Delta n = 0.201$, Slichem Liquid Crystal Material Co., Ltd.); photopolymerizable monomer, C6M; chiral dopants, S811 and R811 (Merck Co. Ltd.); and photoinitiator, 2,2-dimethoxy-2-phenyl-acetophenone (Irgacure 651, TCI Co. Ltd.), were used. Nematic diacrylate monomer, C6M, was synthesized according to the method suggested by D. J. Broer et al.,[31] and another two nematic diacrylate monomers, LC242 and LC1057, were purchased from BASF Co. Ltd. Figure 1 shows the chemical structures of C6M, S811, R811, and Irgacure 651.

2.2. Experimental Cells. To induce a planar orientation of LC molecules, the inner surfaces of indium tin oxide coated (ITO) glass cells were coated with a 3.0 wt % polyvinyl alcohol (PVA) aqueous solution. The deposited film was dried at 80 °C for 30 min and subsequently rubbed with a textile cloth under a pressure of 2.0 g/cm^2 along one direction. PET (polyethylene terephthalate) films with a thickness of 25 μm were used as the cell spacers. The samples were filled into the cells by capillary action.

2.3. Preparation of PSCLCs with a Double-Handed Circularly Polarized Light Reflection Band. The PSCLCs with a double-handed circularly polarized light reflection band were prepared by carrying out the following procedure. At first, the cells containing samples with a left-handed helical structure (LHHS) were irradiated with UV light (365 nm, 1.0 mW/cm^2) for 30 min for polymerization purposes, as shown in Figure 2a. Following that, the cells were immersed in cyclohexane for about 48 h and later in tetrahydrofuran for 20 min to remove the nonreactive LCs. After that, the cells were kept in a vacuum chamber at 60 °C for about 3 h. Thus, the polymer network with a LHHS was obtained as shown in Figure 2b. Finally, the cells containing the polymer network were refilled with samples with a right-handed helical structure (RHHS) by a vacuum filling process, as shown in Figure 2c.

2.4. Measurements. The samples were observed using a polarizing light microscope (POM) (Olympus, BX51). The optical images were recorded using Linksys 2.43 software. The transmission spectra were obtained by using a UV/vis spectrophotometer (Hitachi, U-3010) at normal incidence. The transmittance of a blank cell was normalized to 100%. In general, λ_M and $\Delta\lambda$ are defined as the minimum wavelength of the transmitted light and the bandwidth at half-height of the peak, respectively. The morphology of the polymer network was observed by scanning electron microscopy (SEM) (Hitachi, S-4700). The samples for SEM studies were prepared according to the method described in the previous study.[14]

3. Results and Discussion

To examine the effect of network concentration on the performance of PSCLC film with a double-handed circularly polarized light reflection band, mixtures 1−7 were prepared as listed in Table 1. Herein, the polymer network was formed mainly by photopolymerization of nematic LC diacrylate C6M in the nonreactive LCs. Among all the samples, mixtures 1 and 2 contain 10.0% and 25.0% left-handed chiral dopant S811, respectively, in which the concentration of C6M is 5.0%. To adjust reflection bands of two kinds of circularly polarized light overlap, mixtures 3−5 contain 22.6, 21.5, and 20.3% left-handed chiral dopant S811, respectively, where the concentration of C6M is 10.0, 15.0, and 20.0%, respectively. Mixtures 6 and 7 were added to 10.0% and 25.0% right-handed chiral dopant R811, respectively.

The PSCLCs with a double-handed circularly polarized light reflection band were fabricated as shown in Figure 2. The general idea is that the left-handed polymer network was prefabricated and then the right-handed LCs are infiltrated into the network. According to the above fabrication process, to

TABLE 1: Compositions of Ch-LC Mixtures and Reflection Band of the Cells Obtained

cell[a]	mixture (type)	weight ratio/wt %	λ_M/nm[b] before	λ_M/nm[b] after
	LHHS	SLC-1717/S811/C6M/Irg651		
A (1/6)	1	84.9/10.0/5.0/0.1	1015	1002
B (2/7)	2	69.9/25.0/5.0/0.1	645	620
C (3/7)	3	67.2/22.6/10.0/0.2	658	617
D (4/7)	4	63.2/21.5/15.0/0.3	696	624
E (5/7)	5	59.3/20.3/20.0/0.4	690	602
	RHHS	SLC-1717/R811		
	6	90.0/10.0	955	
	7	75.0/25.0	635	

[a] Cell A (1/6) means that cell A was obtained by infiltrating mixture 6 into the cell containing mixture 1 after polymerization and extraction of nonreactive LCs thereafter. [b] Reflection band before and after UV irradiation.

explain it conveniently, cells containing 5.0% network originated from mixtures 1 and 2 and were refilled with mixtures 6 and 7, which are called cells A and B, respectively. Cells containing 10, 15, and 20% network originated from mixtures 3−5 and were refilled with mixture 7, which are called cells C, D, and E, respectively. As is well-known, the reflection originates from selective reflection, in which one circularly polarized light component with the handedness the same as that of the Ch-LC helix is totally reflected, but the other circularly polarized component is transmitted without reflection. Therefore, the reflectance essentially cannot exceed 50%.[7,8] We show in this study that the film structure is constructed by the permanent solid helical structure of the polymer network and the bulk Ch-LCs with opposite helical structure. Therefore, Ch-LCs films that can reflect both R-CPL and L-CPL simultaneously are expected.

Figure 3a shows the transmission spectra of a cell containing mixture 1 before and after UV irradiation, a cell containing mixture 5, and cell A. It is observed that the cell containing mixture 1 exhibits 50% reflectance before and after UV irradiation (curves 1 and 2). Additionally, we can see that the transmission spectrum of the cell containing mixture 1 after UV irradiation had a slight blue shift compared with that of the cell before UV irradiation. This should be attributed to the reduction of nematic LC concentration from the reactive monomer SLC1717. That is, the cross-linking reaction of C6M in SLC-1717 induced volume shrinkage, thereby resulting in a slight blue shift of the transmission spectrum.[28,29] The cell containing mixture 6 also exhibits 50% reflectance (curve 3). After low molar mass LCs were washed off and refilled with mixture 6, cell A exhibits an unobvious reflection band (curve 4). Furthermore, Figure 3b shows the transmission spectra of a cell containing mixture 2 before and after UV irradiation, a cell containing mixture 7, and cell B. Compared to cell A, cell B also contains 5.0% polymer network, although the reflection band shifts from near IR region to the visible region due to the increase of the concentration of S811. From Figure 3b, we can observe that the spectrum behavior of cell B is also indistinct, which is identical to that of cell A.

Figure 4a shows the transmission spectra of a cell containing mixture 3 before and after UV irradiation, a cell containing mixture 7, and cell C. After UV irradiation, the reflection band of the cell with mixture 3 (which contains 10% C6M) shifts from 660 nm (curve 1) to 620 nm (curve 2) as a result of the formation of the polymer network in the bulk LCs. As mentioned above, this is due to the cross-linking-induced volume

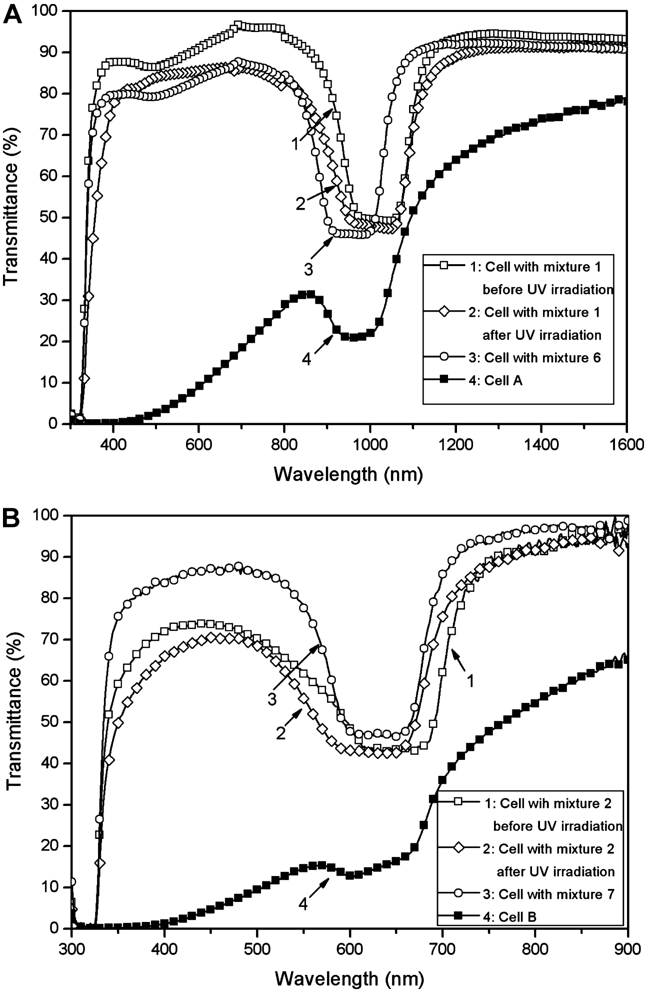

Figure 3. (a) Transmission spectra of a cell containing mixture 1 before and after UV irradiation, a cell containing mixture 6, and cell A. (b) Transmission spectra of a cell containing mixture 2 before and after UV irradiation, a cell containing mixture 7, and cell B.

shrinkage. Moreover, it is notable that the blue shift phenomenon becomes more obvious with increase in C6M concentration, as shown in Table 1 and Figure 4a−c. We then adjusted the concentration of left-handed S811 in mixtures 3−5 to make the two kinds of circularly polarized light reflection bands overlap with each other. For cell C, it can be observed that the reflection band is still indistinct (curve 4) in Figure 4a, which is the same with cells A and B. However, regarding cells D and E, which contain 15% and 20% polymer network, respectively, the spectrum behavior of them is different from that of cells A−C, and the reflection intensity of both cell D and cell E approaches about 100% (curve 4), as shown in Figure 4b,c. This strongly suggested that both R-CPL and L-CPL have been reflected within the same reflection band by cells D and E. The Ch-LC film with a double-handed circularly polarized light reflection band is due to the Ch-LC composite being divided into two distinct environments: the bulk LCs and the strongly network-dominated regions. As shown in Figure 2c, the PSCLC film consists of the polymer network with LHHS and the Ch-LC with RHHS. For the regions farthest from the polymer network, the enclosed LC should behave much the same as in the bulk, leading to Ch structures with a right-handed sense to participate in the reflection of R-CPL. At the closest neighborhood of the polymer network, due to the anchoring effect of the polymer network on the LC molecules, the LC should template the Ch order of the network; thus, the helicity sense of these regions

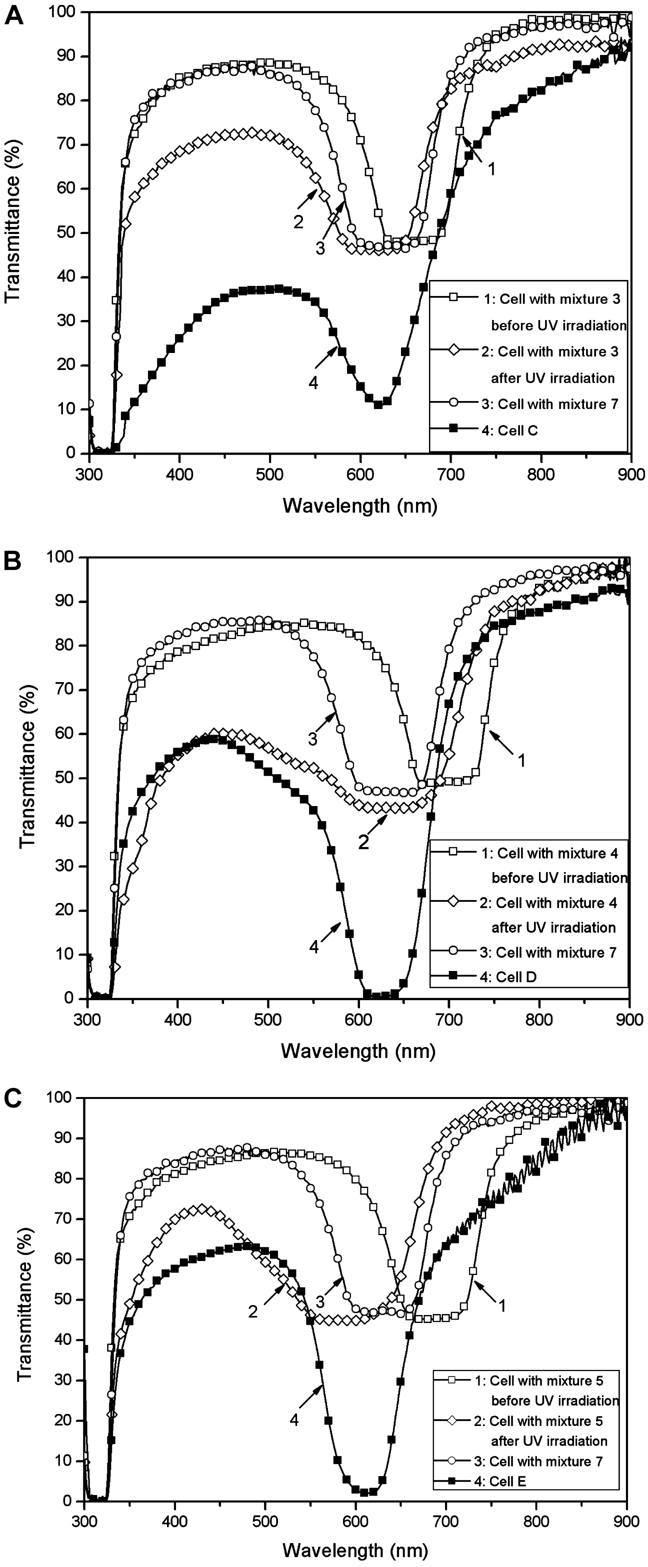

Figure 4. (a) Transmission spectra of a cell containing mixture 3 before and after UV irradiation, a cell containing mixture 7, and cell C. (b) Transmission spectra of a cell containing mixture 4 before and after UV irradiation, a cell containing mixture 7, and cell D. (c) Transmission spectra of a cell containing mixture 5 before and after UV irradiation, a cell containing mixture 7, and cell E.

is left-handed, which participated in the reflection of L-CPL. We confirm that the association of both behaviors within a single-layer PSCLC film made the spectrum obtained exceed the 50% reflectance limit when the pitch lengths of the two opposite helical structures are the same. It has been suggested that the main function of the network is to provide a stable internal memory of the helicity sense.[28,29]

Figure 5a−c shows the POM photographs of the cell containing mixture 4 before and after UV irradiation and after extraction of low molar weight LCs, respectively. It can be seen that the cell containing mixture 4 before and after UV irradiation remains a well-aligned Grandjean planar texture in Figure 5a,b. When the low molar weight LCs were extracted from the cell, it exhibits a different texture behavior from that of the cell before and after UV irradiation. The birefringence phenomenon cannot be observed under POM after extraction of low molar weight LCs, as shown in Figure 5c. This is because there is only the polymer networks with a mass of meshes existing in the system when the low molar weight LCs were extracted from the cell; the networks are incomplete LC structures and do not exhibit the LCs phase, though they were oriented integrally.

The prefabricated LHHS polymer network was refilled with the Ch-LCs with RHHS. Because of the difference in concentration of the network, cells B−E exhibit different texture in Figure 6. Cells A and B exhibited a badly aligned Grandjean planar texture under POM, which is attributed to the weak aligning effect of the polymer network on the refilled Ch-LCs. As shown in Figure 6a,b, Ch-LC molecules have the helical structure on the whole. However, because the helical axes of the domain farthest from the polymer network are oriented randomly, there exists light scattering in the system, which is in accordance with the spectrum characteristics of cells B and C. Cells D and E still remained in an appropriate planar texture when Ch-LCs with RHHS were refilled into the network in Figure 6c,d. This is because of the strong anchoring of the polymer network on LC molecules. However, it must be noted that the planar texture of cell E was influenced by the high C6M concentration, which we believe is due to the limited solubility of C6M in the low molar weigh LCs.

Figure 7 shows SEM photographs of the surface morphology of the cells containing mixtures 2−5 polymerized at room temperature. A smooth polymer network can be observed for mixtures 2−4 containing 5, 10, and 15% C6M (Figure 7a−c). This indicates good solubility of the components within the LCs. It can be confirmed that, if the monomer is highly soluble in LCs, it undergoes a radical chain polymerization, leading to smooth networks.[5,32,33] Additionally, a higher monomer concentration results in a much denser network, as indicated in Figure 7a−c. However, this trend is not significant because the system in our study has a large monomer content, thereby resulting in the difference of the surface morphology of the networks observed by SEM becoming small, which is very similar to that of previous research.[6,32] As regards to mixture 5, which contains 20% C6M, the rice-grain-like network was observed in Figure 7d.[34,35] This behavior can be attributed to the solubility limit of C6M within the LCs. When C6M's concentration is close to the solubility limit, it undergoes precipitation polymerization, leading to rice-grain-like networks.[33]

To further understand the memory effect of the network in PSCLCs, a photopolymerizable mixture 8 with a nematic phase at lower temperature was prepared by mixing C6M, LC242, and LC1057 at a weight ratio of C6M/LC242/LC1057 = 1/1/1, and 2% by the weight of monomers of photoinitiator Irgacure 651 is added. Cell G was then prepared by refilling mixture 8 into cell F, followed by polymerization at 40 °C. In this case, cell F contains mixture 4 and the low molar weight LCs were extracted from it after polymerization. Figure 8 shows the transmission spectra of cells F and G. We see that cell F has no

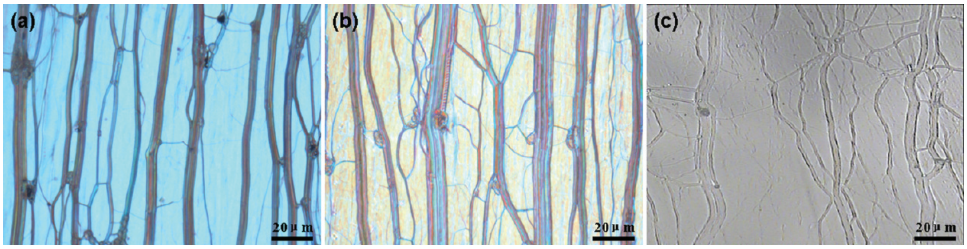

Figure 5. POM photographs of (a) a cell containing mixture 4 before and (b) after UV irradiation and (c) after extraction of low molar weight LCs.

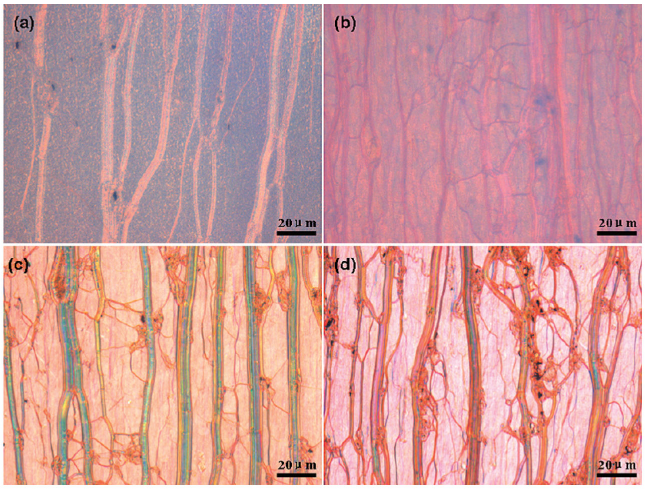

Figure 6. POM photographs of (a) cell B, (b) cell C, (c) cell D, and (d) cell E.

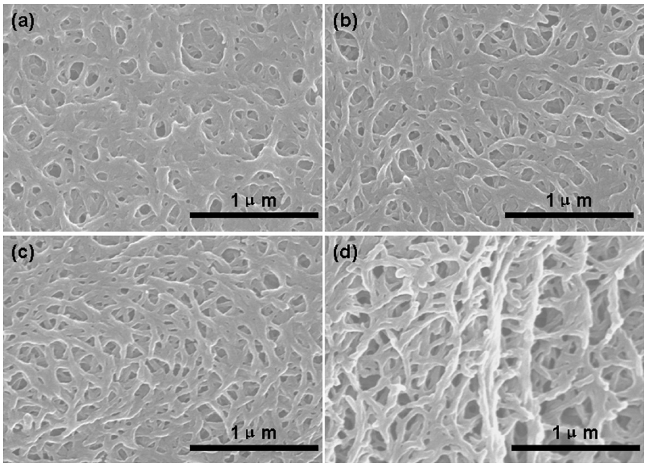

Figure 7. SEM photographs of the surface morphology of the cells containing (a) mixture 2, (b) mixture 3, (c) mixture 4, and (d) mixture 5.

selective reflection of light and keeps a transmission state in the visible region (curve 1), which is attributed to the extraction of low molar weight LCs from the cell. However, for cell G, a reflection band reappeared at about 630 nm in the spectra (curve 2), which is approximate to that of the cell containing mixture 4 after polymerization. From the above discussion, we can confirm further that the polymer network has a characteristic of memorizing the Ch-LC helix structure in PSCLCs.

Figure 9 shows an SEM image of the cross section of cell G. The distribution of the pitch length can be observed in Figure 9. An integrated solid cholesteric structure is formed after infiltration of mixture 8 into cell F and polymerization thereafter, which we confirm is due to the helix-structure memory effect of the polymer network prefabricated in the previous step. The

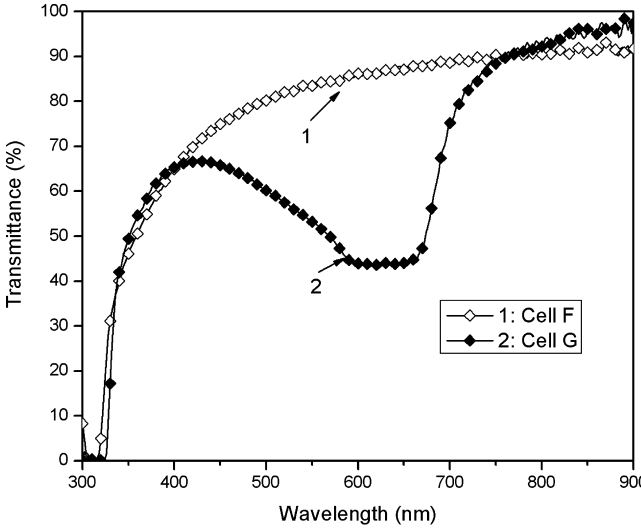

Figure 8. Transmission spectra of cell F and cell G.

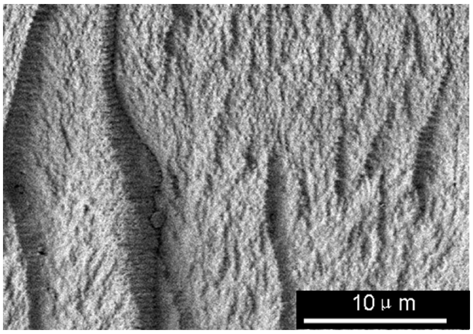

Figure 9. SEM micrograph of the cross section of cell G.

results undoubtedly suggest the transfer of the organization of the mesophase onto the structure of the network,[5] which offers strong evidence for the above explanation.

The images of cell D under a polarizer were shown in Figure 10. The polarizer used here has two windows for both left-handed polarized light and right-handed polarized light, which is prepared by stacking multilayer PVA films on the TAC substrate, followed by dyeing and a uniaxial tension process. It can be seen that cell D exhibits selective reflection of light within both the left-handed polarizer window and the right-handed polarizer window, which indicates that both R-CPL and L-CPL have been reflected by cell D within the same reflection band. From the above discussion, it appears clearly that, due to the wavelength and polarization-selective reflection, the PSCLC film with a double-handed circularly polarized reflection band is applicable both for counterfeit deterrence in banknotes and secure documents and for authentication processes of brand and product protection. Additionally, other potential applications are related to tunable bandpass filters, polarizer-free reflective displays without using a backlight requirement, polarizers or

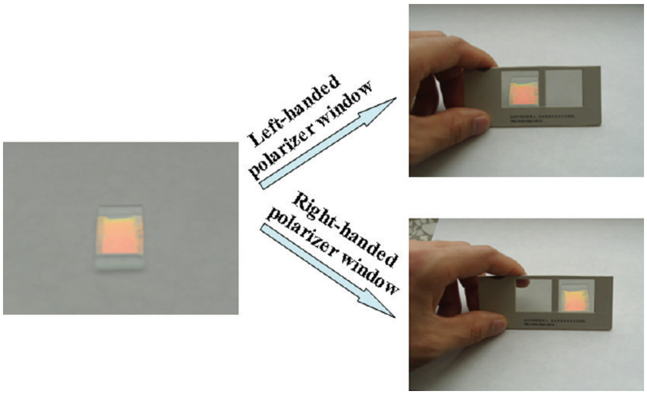

Figure 10. Image photographs of cell D when observed by a polarizer.

color filters, mirrorless lasing, and smart switchable reflective windows for the dynamic control of solar light.

4. Conclusions

In summary, we have fabricated PSCLC film with a double-handed circularly polarized reflection band by a wash-out/refill method. The concentration of the prefabricated polymer network facilitates the formation of a single-layer Ch-LC structure that has a legible double-handed circularly polarized reflection band. The spectrum characteristic is unobvious when the PSCLCs contain a low network concentration. This could be attributed to the weak anchoring effect, followed by a light-scattering phenomenon occurring in the system. However, both kinds of circularly polarized reflection become clear with increments in the network concentration in connection with the strong anchoring effect, as demonstrated by POM and SEM. Additionally, the memory effect of the polymer network in PSCLCs was further emphasized and proved by SEM. A novel opportunity to modulate the reflection over 50% in a single-layer Ch-LC film is offered; this result could be used in many potential applications, including security technologies that make use of patterned, monolithic optical layers, smart reflective windows for solar light management, and reflective polarizer-free displays with higher brightness.

Acknowledgment. This research was supported by the National Natural Science Foundation (Grant No. 50673007).

References and Notes

(1) Mucha, M. *Prog. Polym. Sci.* **2003**, *28*, 837.
(2) Shibaev, P. V.; Kopp, V. I.; Genack, A. Z. *J. Phys. Chem. B* **2003**, *29*, 6961.
(3) Kjellander, B. K. C.; Bulle-Lieuwma, C. W. T.; van IJzendoorn, L. J.; de Jong, A. M.; Niemantsverdriet, J. W.; Broer, D. J. *J. Phys. Chem. C* **2007**, *29*, 10965.
(4) Yang, D.-K.; Chien, L.-C.; Doane, J. W. *Appl. Phys. Lett.* **1992**, *60*, 3120.
(5) Dierking, I. *Adv. Mater.* **2000**, *12*, 167.
(6) Dierking, I.; Kosbar, L. L.; Afzali-Ardakani, A.; Lowe, A. C.; Held, G. A. *J. Appl. Phys.* **1997**, *7*, 3007.
(7) Chilaya, G. In *Chirality in Liquid Crystals*; Springer Series Partially Ordered Systems; Kitzerow, H., Bahr, C., Eds.; Springer: New York, 2001.
(8) de Gennes, P. G.; Prost, J. *The Physics of Liquid Crystals*; Clarendon Press: Oxford, U.K., 1993.
(9) Broer, D. J.; Lub, J.; Mol, G. N. *Nature* **1995**, *378*, 467.
(10) Broer, D. J.; Mol, G. N. *Adv. Mater.* **1999**, *11*, 573.
(11) Hikmet, R. A. M.; Kemperman, H. *Nature* **1998**, *392*, 476.
(12) Binet, C.; Mitov, M.; Mauzac, M. *J. Appl. Phys.* **2001**, *90*, 1730.
(13) Boudet, A.; Binet, C.; Mitov, M.; Bourgrette, C.; Boucher, E. *Eur. Phys. J. E* **2000**, *2*, 247.
(14) Mitov, M.; Nouvet, E.; Dessaud, N. *Eur. Phys. J. E* **2004**, *15*, 413.
(15) Guillard, H.; Sixou, P.; Reboul, L.; Perichaud, A. *Polymer* **2001**, *42*, 9753.
(16) Dyer, D. J.; Schröder, U. P.; Chan, K. P.; Twieg, R. J. *Chem. Mater.* **1997**, *9*, 1665.
(17) Relaix, S.; Bourgerette, C.; Mitov, M. *Appl. Phys. Lett.* **2006**, *89*, 251907.
(18) Fan, B.; Vartak, S.; Eakin, J. N.; Faris, S. M. *Appl. Phys. Lett.* **2008**, *92*, 061101.
(19) Fan, B.; Vartak, S.; Eakin, J. N.; Faris, S. M. *J. Appl. Phys.* **2008**, *104*, 023108.
(20) Yang, H.; Mishima, K.; Matsuyama, K.; Hayashi, K. I.; Kikuchi, H.; Kajiyama, T. *Appl. Phys. Lett.* **2003**, *82*, 2407.
(21) Xiao, J. M.; Zhao, D. Y.; Cao, H.; Yang, H. *Liq. Cryst.* **2007**, *34*, 473.
(22) Bian, Z. Y.; Li, K. X.; Huang, W.; Cao, H.; Zhang, H. Q.; Yang, H. *Appl. Phys. Lett.* **2007**, *91*, 201908.
(23) Guo, J. B.; Sun, J.; Li, K. X.; Cao, H.; Yang, H. *Liq. Cryst.* **2008**, *35*, 87.
(24) Bouligand, Y. In *Liquid Crystalline Order in Polymers*; Blumstein, A., Ed.; Academic Press: New York, 1978.
(25) Song, M. H.; Park, B.; Shin, K.-C.; Ohta, T.; Tsunoda, Y.; Hoshi, H.; Takanishi, Y.; Ishikawa, K.; Watanabe, J.; Nishimura, S.; Toyooka, T.; Zhu, Z.; Swager, T. M.; Takezoe, H. *Adv. Mater.* **2004**, *16*, 779.
(26) Hwang, J.; Song, M. H.; Park, B.; Nishimura, S.; Toyooka, T.; Wu, J. W.; Takanishi, Y.; Ishikawa, K.; Takezoe, H. *Nat. Mater.* **2005**, *4*, 383.
(27) Makow, D. M. *Appl. Opt.* **1980**, *19*, 1274.
(28) Mitov, M.; Dessaud, N. *Nat. Mater.* **2006**, *5*, 361.
(29) Mitov, M.; Dessaud, N. *Liq. Cryst.* **2007**, *2*, 184.
(30) Guo, J. B.; Cao, H.; Wei, J.; Zhang, D. W.; Liu, F.; Pan, G. H.; Zhao, D. Y.; He, W. L.; Yang, H. *Appl. Phys. Lett.* **2008**, *93*, 201901.
(31) Broer, D. J.; Boven, J. G.; Mol, N. *Makromol. Chem.* **1989**, *190*, 2255.
(32) Ma, R. Q.; Yang, D. K. *Phys. Rev. E* **2000**, *61*, 1567.
(33) Dierking, I.; Kosbar, L. L.; Lowe, A. C.; Held, G. A. *Appl. Phys. Lett.* **1997**, *71*, 2454.
(34) Rajaran, C. V.; Hudson, S. D.; Chien, L. C. *Chem. Mater.* **1995**, *7*, 2300.
(35) Yang, D.-K.; Chien, L.-C.; Fung, Y. K. In *Liquid Crystals in Complex Geometries*; Crawford, G. P., Zumer, S., Eds.; Taylor & Francis: London, 1996; pp 103−142.

JP903394R

Abstract

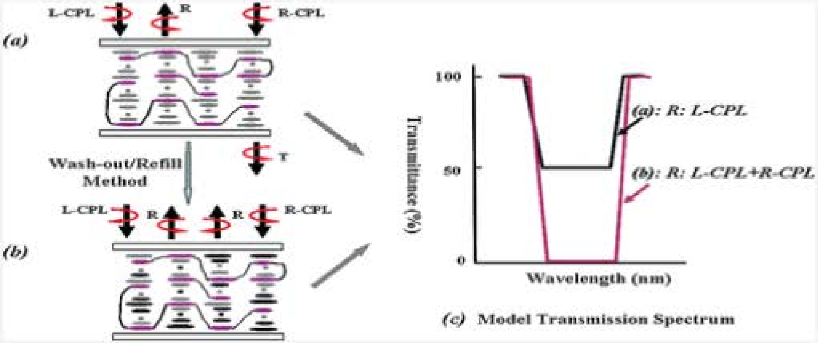

Polymer-stabilized cholesteric liquid crystals (PSCLCs) with a double-handed circularly polarized reflection band are fabricated by a wash-out/refill method. By removing the low molar weight LCs from the original PSCLC film, desirable liquid crystals (LCs) can be infiltrated into a prefabricated polymer network. The results showed that the memory of the polymer network controls the resulting material properties. The concentration of the prefabricated polymer network also plays a relevant role in the formation of a single-layer cholesteric LC (Ch-LC) structure that has a clear-cut double-handed circularly polarized reflection band. A light-scattering phenomenon occurring in the system alters the reflection properties of Ch-LCs, which is due to the weak anchoring effect of the network when PSCLC film contains a low network concentration. Both kinds of circularly polarized reflection become more obvious with increase in the network concentration, followed by the strong anchoring effect of the network. The technique developed in this study has great applications in industries that require solid optical functional films and coatings.
