# Supplementary material for: Advanced liquid crystal-based switchable optical devices for light protection applications: principles and strategies
Source: Light Sci Appl. 2023 Jan 3;12:11. doi: 10.1038/s41377-022-01032-y (PMC9807646; doi:10.1038/s41377-022-01032-y)
Supplement: Supplementary file 9 — Fig 10 copyright promotion [file 41377_2022_1032_MOESM9_ESM.pdf]

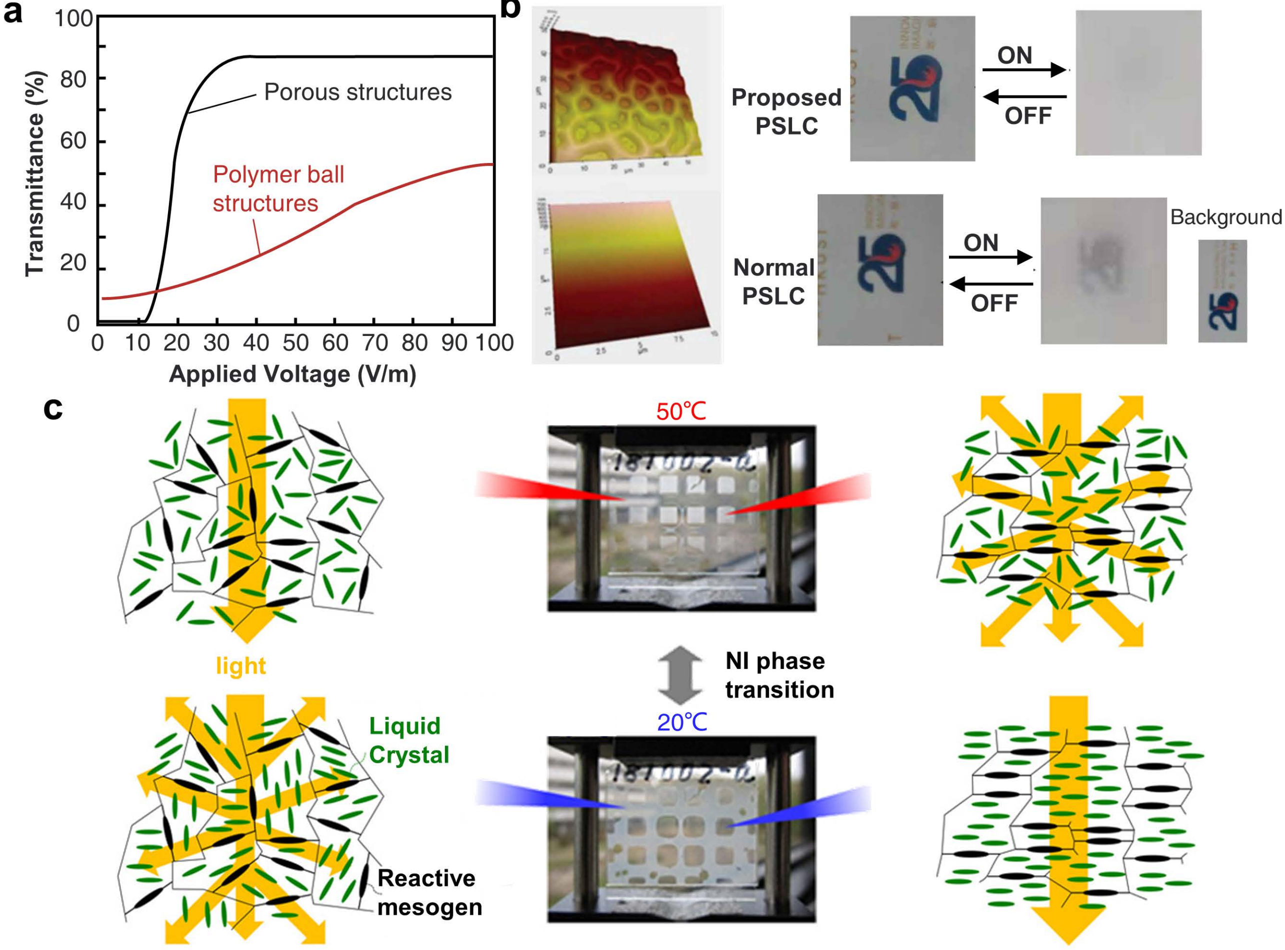

a
Transmittance (%)
Applied Voltage (V/m)
Porous structures
Polymer ball structures
0
20
40
60
80
100
0
10
20
30
40
50
60
70
80
90
100
b
Proposed PSLC
Normal PSLC
ON
OFF
Background
c
50℃
20℃
NI phase transition
light
Liquid Crystal
Reactive mesogen

Permissions Request [ref:_00D0Y35Iji._5007R3P9mUw:ref]

发件人：permissionrequest<permissionrequest@tandf.co.uk>
时　间：2022年10月7日(星期五) 晚上11:08
收件人：ruicong.zhang<ruicong.zhang@hrtcn.org>

Taylor & Francis Group
an informa business

Our Ref: la/tlct/02542226

07/10/2022

Springer Nature c/o Ruicong Zhang

Figure 2 from Yuanyuan Zhan, Han Lu, Mingliang Jin & Guofu Zhou (2020) Electrohydrodynamic instabilities for smart window applications, Liquid Crystals, 47:7, 977-983, DOI: 10.1080/02678292.2019.1692929

Figure 2 & 3 from Cuiling Meng, Man Chun Tseng, Shu Tuen Tang, Chen Xiang Zhao, Sze Yan Yeung & Hoi Sing Kwok (2019) Normally transparent smart window with haze enhancement via inhomogeneous alignment surface, Liquid Crystals, 46:3, 484-491, DOI: 10.1080/02678292.2018.1508764

Thank you for your attached correspondence requesting permission to reproduce the above material in your forthcoming publication entitled 'Advanced liquid crystal-based switchable optical devices for light protection applications: principles and strategies' to be published in Light: Science & Applications, published by Springer Nature.

This usage falls under the provisions of the STM Agreement, we shall be pleased to waive our fees, and to grant you non exclusive world rights in all languages, covering print and e-journal usage of your Work all editions, on the condition that:

1. The original source of publication and Taylor & Francis Ltd, are acknowledged in the caption, including a reference to the Journal's web site: www.tandfonline.com

2. You do not license to any third party permission to reproduce this copyrighted material, in any form, and at any time.

3. This permission does not cover any third party copyrighted work which may appear in the material requested. Please ensure you have checked all original source details for the rights holder.

4. Any alterations/adaptions to the original work must be approved by the original author(s) of the article.

Thank you for your interest in our Journals.

Yours sincerely,

Lee-Ann Anderson | Senior Permissions Executive, Journals
Taylor & Francis Group
4 Park Square, Milton Park, Abingdon, OX14 4RN
Permissions e-mail: journalpermissions@tandf.co.uk
Web: www.tandfonline.com
Tel: +44 (0)20 8052 0659

Taylor & Francis is a trading name of Informa UK Limited,
registered in England under no. 1072954

Disclaimer: T&F publish Open Access articles in our subscription priced journals, please check if the article you are interested in is an OA article and if so, which licence was it published under.

Before printing, think about the environment.

ref:_00D0Y35Iji._5007R3P9mUw:ref

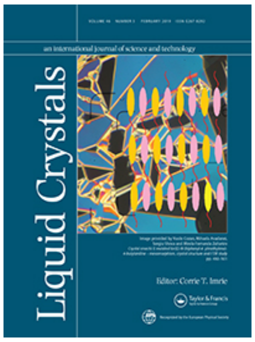

Liquid Crystals

ISSN: 0267-8292 (Print) 1366-5855 (Online) Journal homepage: https://www.tandfonline.com/loi/tlct20

Normally transparent smart window with haze enhancement via inhomogeneous alignment surface

Cuiling Meng, Man Chun Tseng, Shu Tuen Tang, Chen Xiang Zhao, Sze Yan Yeung & Hoi Sing Kwok

To cite this article: Cuiling Meng, Man Chun Tseng, Shu Tuen Tang, Chen Xiang Zhao, Sze Yan Yeung & Hoi Sing Kwok (2019) Normally transparent smart window with haze enhancement via inhomogeneous alignment surface, Liquid Crystals, 46:3, 484-491, DOI: 10.1080/02678292.2018.1508764

To link to this article: https://doi.org/10.1080/02678292.2018.1508764

Published online: 22 Aug 2018.

Submit your article to this journal

Article views: 547

View related articles

View Crossmark data

Citing articles: 23 View citing articles

Full Terms & Conditions of access and use can be found at
https://www.tandfonline.com/action/journalInformation?journalCode=tlct20

LIQUID CRYSTALS
2019, VOL. 46, NO. 3, 484–491
https://doi.org/10.1080/02678292.2018.1508764

Taylor & Francis
Taylor & Francis Group

Check for updates

Normally transparent smart window with haze enhancement via inhomogeneous alignment surface

Cuiling Meng, Man Chun Tseng, Shu Tuen Tang, Chen Xiang Zhao, Sze Yan Yeung and Hoi Sing Kwok

State Key Laboratory on Advanced Displays and Optoelectronics Technologies, Hong Kong University of Science and Technology, Kowloon, Hong Kong, China

ABSTRACT

Smart windows which switch between transparent and scattering states can be used not only as privacy window in buildings and cars, but also as information displays, and motorcycle helmet visors. Here, we have proposed a reverse mode polymer-stabilised liquid crystals (PSLCs) smart window based on inhomogeneous alignment surface, which greatly enhances the haze by 42% when compared to device without inhomogeneity treatment. A 100-cm^2 window that switches between the power-off clear state (~4% haze) and a power-on scattering state (64% haze at 15 V) in less than 3.5 ms is demonstrated. Since no high-temperature treatment (< 80°C) is involved in device fabrication, further development on plastic films is feasible.

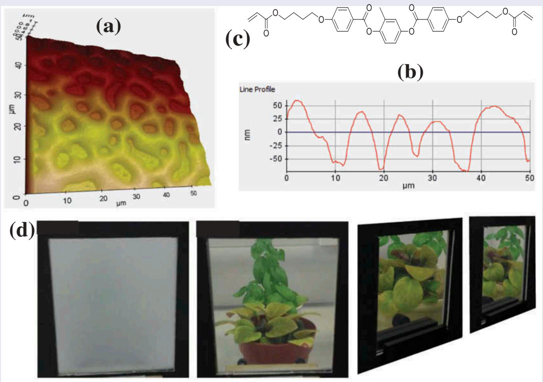

ARTICLE HISTORY
Received 5 May 2018
Accepted 31 July 2018

KEYWORDS
Reactive mesogen; inhomogeneity; scattering; smart window

1. Introduction

There is burgeoning interest in commercialising dynamic windows that enable electronic control of visible light and solar energy [1–5]. Many companies have recently invested a significant amount of resources and effort on dynamic windows based on its potential to increase architectural beauty, occupant comfort and energy efficiency [6–8]. In buildings, energy savings of 20% can be made through smart windows due to reduced lighting, heating and cooling costs [9]. In addition, smart windows look substantially fashionable than simply using a blind, and are more convenient for optimal adjustment since they can be automated with a computer and incorporated into smart houses. Dynamic windows can also be implemented in the sunroofs of vehicles and in switchable sunglasses, which would outperform those using passive photochromic glasses that darken only upon exposure to UV light.

Smart window technologies consist of electrically driven media such as electrochromic (EC) [9,10], suspended particle electrophoresis [11,12], polymer dispersed liquid crystals [2,13] and cholesteric liquid crystals [14–16]. The EC windows switch from a voltage-off clear state to a voltage-on coloured state. The switching time depends on the window size and can be as long as several minutes. While the suspended particle device (SPD) has less than 1% light leakage in the dark state, the switching time lasts for 1 ~ 3 s [12]. Due to the long switching time and light leakage problem, the EC and SPD windows are mainly applied to architectural windows and cannot be used as privacy windows. Thermochromic windows, which switch in response to a temperature change, are unideal due

CONTACT Cuiling Meng cmengab@connect.ust.hk

© 2018 Informa UK Limited, trading as Taylor & Francis Group

to the lack of user control [17]. A research group in Stanford recently proposed a dynamic window based on reversible metal electrodeposition which, despite its wide range of transmission (i.e. 5%-80%), also suffers from slow response (up to 3 min) [3]. Another group recently proposed a new way to create a scattering window by depositing grass-like nanostructure onto the glass and 80 s is needed to switch the window via heat evaporation [18]. As to PDLCs, while an attractive feature is their fast response (i.e. a few milliseconds), they function by a normally scattering mode, making them undesirable for any application in which maintaining a power off window view is important. Some attempts have been made to give self-powered or energy saving smart windows. For example, a luminescent solar concentrator or an absorbing a-Si layer has been integrated into the PDLC cell to realise self-powered switching [19,20]. However, the switching is highly weather-dependent. Ultra-low driving voltage PDLC device via a thiol-ene polymerisation has also been reported [21]. Besides, reserve-mode PDLCs through coating an alignment layer and refractive index matching has also been demonstrated in Ref [22]. But the transparency at power-off state still needs further improvement. An alternative to PDLCs is PSLCs, where a smaller quantity of monomer (< 10%) is embedded in a continuous LC matrix. One of the advantages of PSLCs over PDLCs is the lower driving voltage and ultra-high transparent initial state due to the smaller polymer percentage. However, the relatively weak haze of PSLCs, i.e. 40%-50% [23], significantly limits its application to devices such as light shutters, privacy windows, etc.

For most LC devices, the alignment layer plays a critical role on LC conformation. Polyimide (PI), characterised by its outstanding thermal and electrical properties has been widely applied as the alignment layers to align LC molecules. Usually, the azimuthal LC anchoring direction is determined by mechanical buffing on the PI alignment layer. The homeotropic and homogeneous type PI are used to induce 90° and near zero pretilt angle for vertical alignment mode and in-plane switching mode LC displays respectively [24]. Besides conventional PI, there also exist photoalignment layer that without the need of mechanical buffing. When exposed to linearly polarised light, the photoalignment layer can provide a uniform azimuthal direction to the LC molecules with a very low pretilt angle (i.e. 0.2°) [25]. With photoalignment technique, a complicated aligning pattern for a specific LC device can be easily achieved [26–29]. In addition, by controlling the ratio between planar and vertical PI, alignment layer with pretilt angle between 0° and 90°Can also be obtained [30–32]. Such alignment layer with controllable pretilt angle can find applications on many different LC devices [24,33].

In this paper, an inhomogeneous morphology was embedded on a vertical polyimide alignment layer and it is found that the power-on haze of an initially transparent window can be greatly enhanced. The window can be switched uniformly between transparent (4% haze) and opaque states (64% haze) in less than 3.5 ms at 15 V.

2. Methodology

Figure 1 shows the five-layer PSLC smart window structure. In the centre, there is the polymer-stabilised LC bulk. On both sides of the LC bulk is the inhomogeneous vertical alignment film supported by a conductive substrate (i.e. indium tin oxide, ITO-coated glass). In the voltage-off state, the LC molecules are aligned vertically and the polymer network replicates this alignment. Since the refractive indices of LC molecules are chosen to match that of the polymer, the device appears transparent. When a voltage is applied, the LC molecules with negative dielectric anisotropy tend to reorient and deviate from the vertically aligned position. LC molecules in the vicinity of the polymer strands keep their original orientations, while the bulk molecules reorient to various azimuthal and polar

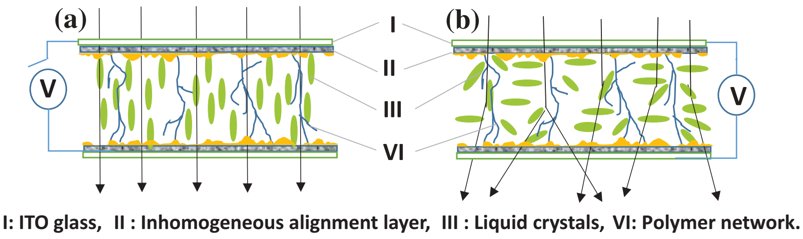

Figure 1. (Colour online) Schematics of the proposed haze-enhanced PSLCs smart window. (a) no voltage applied, PSLCs smart window is in a transparent state; (b) with voltage applied, many randomly aligned domains formed and significant light scattering results.

angles [9]. As a result, many randomly aligned LC domains are formed and a resultant scattering state occurs. The scattering increases and then reaches a maximum as the voltage increases. Under excessive voltage, the scattering may start to drop. When the applied voltage is turned off, the LCs return to their original vertical alignment and the device returns to its transparent state.

Central to the haze-enhanced PSLCs smart windows is the inhomogeneous surface, which was deliberately created via a temperature-induced phase separation method. Specific procedures are described as follows: first, 3.6% SE-4811 vertical polyimide (Nissan Chemical) in N-Methyl-2-pyrrolidone (NMP) solvent was prepared and denoted as sol-I. Second solution termed as sol-II, formed by 5% of reactive mesogen UCL017 (DIC Corp.) doped with photo-initiator, was also prepared. Sol-I and sol-II were then mixed together in 1:25 mass ratio (*MR*). To ensure complete miscibility, the mixture was put on a magnetic stirrer for 30 min. The mixture was then spin coated on a well-cleaned ITO glass substrate, followed by soft-bake at 80°C for 60 s, and then cooled slowly in air afterwards (the cooling rate is found to be around 1°C per second). After 20s ~ 30s' cooling, phase separation starts and the alignment surface gradually evolves from a smooth film to an inhomogeneous surface with a puddle-like morphology (Figure 2(a)). After that, a UV lamp (20 mW/cm^2) was used to polymerise the layer for 5 min so as to form a robust inhomogeneous surface. It is revealed that, after mapped by atomic force microscopy (AFM), the layer has a profile depth of around 110 nm (Figure 2(b)). For comparison, in pure vertical polyimide, which is characterised by a very smooth surface in Figure 2(c). The average profile depth is only 1 nm (Figure 2(d)).

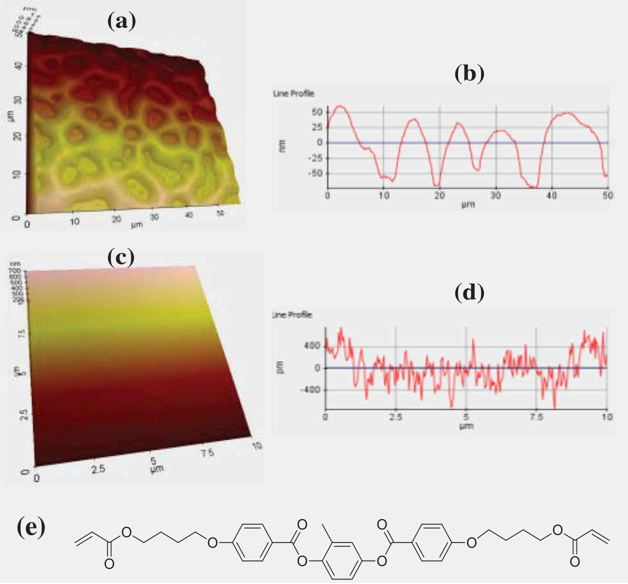

Figure 2. (Colour online) Alignment surface morphologies with (a) and without (c) inhomogeneity treatment, where the average depth is 110 nm (b) and 1 nm (d) respectively, (e) Chemical structure of reactive mesogen UCL017.

3. Experimental results

Samples were then made with substrates with and without surface inhomogeneity. The cell gap was maintained at 5 μm using silicon spacer balls. The silicon balls did not damage the polymerised reactive mesogen due to the latter's robust nature. An LC mixture consisted of 95% negative LC (Δn = 0.1033, ne = 1.5851, no = 1.4818, Δε = -4.5) and 5% reactive mesogen with small amount of photo-initiator was then filled into the empty cells by capillary action under yellow light conditions. The LC mixture was brought into a desired orientation by the surface alignment interaction. Photo-polymerisation was then induced by irradiating the samples using the same UV lamp as before for 5 min. During photo-polymerisation, the order of the nematic phase was templated by the forming polymer network [1]. Afterwards, when we applied a 10 V 1 kHz square-wave to the sample LC cells, very prominent haze enhancement was found in sample with inhomogeneity treatment as shown in Figure 3(b,d).

It is interesting to find that different polyimide to reactive mesogen ratio (*MR)* results in different densities of inhomogeneity. As presented in Figure 4, except for the two extreme recipes 1:0 and 0:1 (Figure 4(a,f)), the remaining ones showed distinct puddle-like

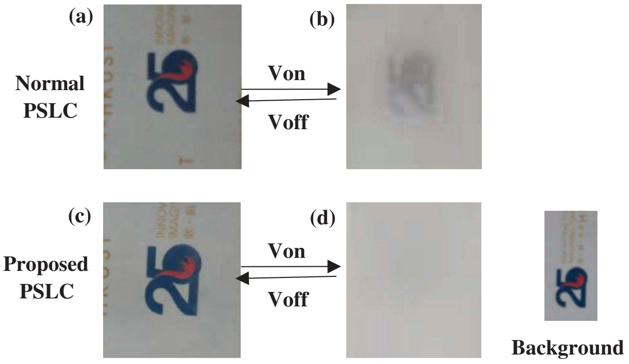

Figure 3. (Colour online) Comparison of the power on haze states of the two different samples. The upper row is the result from smooth vertical alignment sample (a and b) and the lower row is result from inhomogeneous vertical alignment sample (c and d). The background is a printed paper placed under the samples, and the distance between the samples and printed paper is 10 cm.

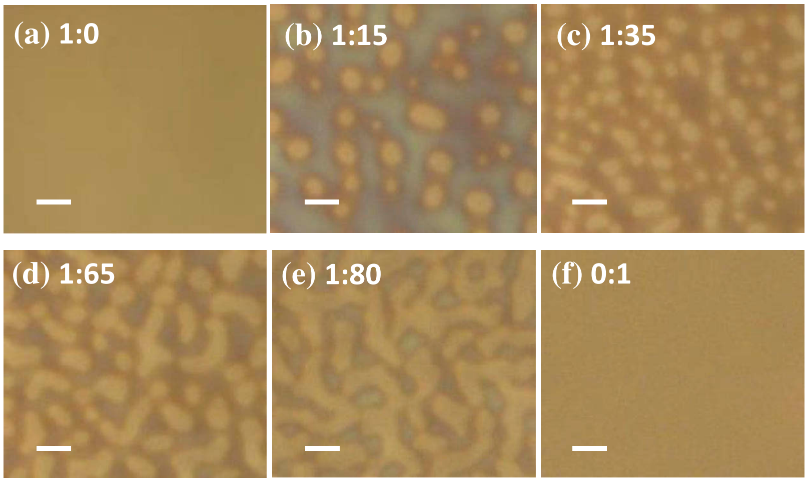

Figure 4. (Colour online) Alignment surface morphologies of surfaces with different VA polyimide to reactive mesogen ratio (*MR*s = 1:0, 1:15, 1:35, 1:65, 1:80 and 0:1). All white scale bars in the insets represent length of 10 μm.

features. The differences among them are the size and density of the puddle-like shape. For example, recipe 1:15 shows rather sparse domains. However, as *MR* increases to 1:35, the domains are distributed with higher density (Figure 4(c)). A further decrease on *MR*s (e.g. 1:65 and 1:80) results in a sparser distribution again but the domain size becomes larger. This is probably due to the recombination of smaller domains as a greater amount of reactive mesogen was added in SE-4811 polyimide. Then, samples with different substrates inhomogeneity (i.e., *MR*s = 1:0, 1:15, 1:25, 1:35, 1:45, 1:65, 1:80 and 0:1) were successively assembled. All samples have the same 5 μm cell gap. The haze of all samples under different driving voltages are measured according to ASTM D1003 [34]. Results are given in Figure 5(a,b).

In Figure 5(a), when *MR* = 1:35 highest haze of up to 64% is achieved at 15 V. Compare to the ordinary PSLC with smooth surface (*MR* = 1:0), an approximate 42% improvement on the haze performance is achieved. At the same time, the clear state of recipe 1:35 still maintained at a very low haze (i.e. 4.3%). Figure 5(b) summarises the power-off and -on hazes of six PSLC devices respectively. Recipe 1:35 shows the widest haze gap between power-on and power-off haze states, up to 60%. Such high contrast is quite acceptable for switchable windows even for transparent display application. Samples made by recipes 1:15, 1:25 and 1:45 also show up to 50% gap between power-on and -off states, which provides a high tolerance during recipe preparation and sample fabrication. It is noticed that the scattering drops a little bit at excessive voltage loading (e.g. >25 V). This is because at excessive voltage more LC molecules near the polymer branches can overcome the constraint of the polymer network and align themselves more homogeneously, thus reach at a state with higher order. Hence, the ability to scatter light will abate to some extent.

Dependence of cell gap on hazy performance was also studied. With the optimised alignment recipe, that is *MR* = 1:35, samples with different cell gaps were made. The measured power-on and -off haze values

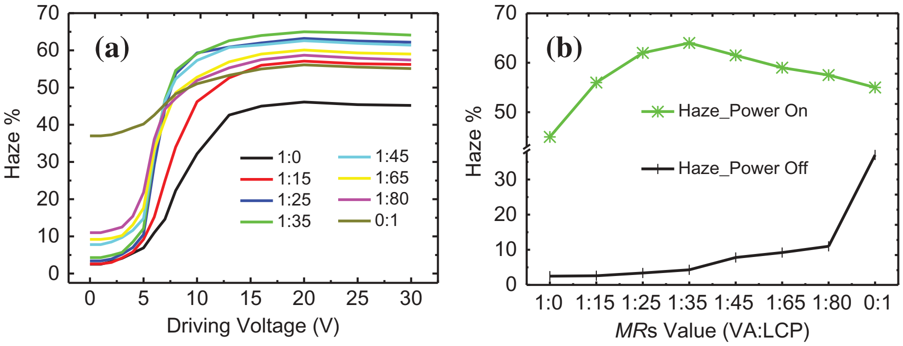

Figure 5. (Colour online) (a) Haze dynamic of different PSLC devices made by different alignment recipe characterised with various surface inhomogeneity; (b) haze levels of different devices at power on and power off states.

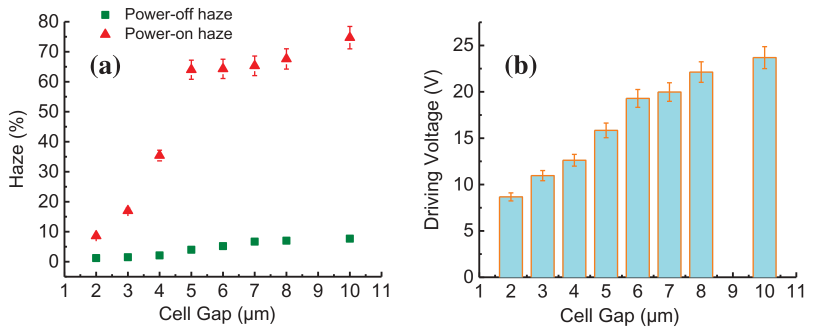

Figure 6. (Colour online) (a) Power-on and -off hazy levels at different cell gaps; (b) driving voltage required for maximised power-on haze at different cell gap cases.

were plotted in Figure 6(a). It is shown that the power-on haze increases sharply from 2 μm to 5 μm and then becomes more gently afterwards. While the power-off transparency gradually decreases as the cell gap increases and this is unfavourable. Although it is expected that larger cell gap will result in higher power on haze, however from Figure 6(b), it also means that the driving voltage is also increased. For example, the driving voltage of a 10 μm cell is 25 V and that of a 5 μm cell is 15 V. Consider the requirements of low driving voltage, high power-on haze and high power-off transparency, a 5 μm device cell gap seems to have a good balance on all these requirements. In addition, thinner cell gap is favourable for fast switching [35].

Figure 7(a) shows the temporal response of a 5 μm cell gap sample. Here, the rising time from clear state to haze state (an interval from 90% transmittance to 10% transmittance) is only 0.55 ms. and the falling or relaxation time, is 2.6 ms. Therefore the total response time is less than 3.5 ms. Such response time is much faster than previously reported SPD and heat-evaporated smart glass [3,4,11,12,18]. The fast rising response is not only benefited from the relatively small

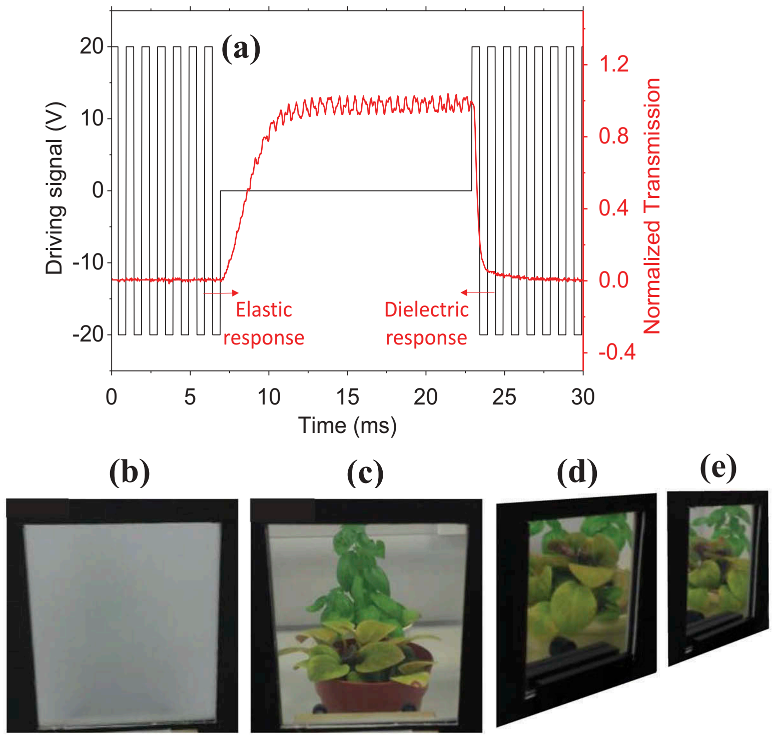

Figure 7. (Colour online) (a) Transmittance dynamic (red curve) of PSLC smart window as a function of driving scheme (black curve) with amplitude of 20 V and frequency of 1 kHz square-wave voltage; (b) and (c) are the ON and OFF state of a 100-cm^2 PSLC smart window, and the plants in the background are held at a distance of 20 cm; optical image of the OFF state through the device at different viewing directions are also shown: (c) normal viewing, (d) 40° from normal and (e) 70° from normal.

cell gap (i.e. 5 μm) but also owns to the effect of the polymer network in LC bulk [36]. At last by applying all the previous described optimised parameters, a 100-cm^2 reverse mode smart window was made and demonstrated in Figure 7(b-e). The proposed PSLC window exhibited a high transparent power off state (Figure 7(c)), a good power on opaque state (Figure 7 (b)) and a wide viewing angle. For instance, when viewed at 40° (Figure 7(d)) or even 70° (Figure 7(e)) from the sample normal, the green plants behind the window device were still clearly visible.

4. Discussion

From the results presented above, it is clear that the introduction of an inhomogeneous alignment surface to the PSLC cell can effectively enhance the power-on scattering or haze while keeping a relatively low power-off haze. The reasons behind great scattering enhancement at power-on state on the samples with inhomogeneous surface have been studied. First of all, the inhomogeneous surface gives small polar and random azimuthal angle to the vertically aligned liquid crystal molecules. Since no rubbing treatment is involved, random azimuthal angles aligning takes place when E-field is applied. Here, random polar angles are due to the effect of reactive mesogen, UCL017. In our study, UCL017 used is a planar-type monomer which favours homogeneous alignment while SE-4811 is a vertical alignment polyimide. The phase separation induced by temperature causes a random distribution of UCL017 and SE-4811 materials (as seen from Figure 4(b–e)). As a result, individual positions would manifest different pretilt angles determined by the local ratio of UCl017 and SE-4811 [37]. On application of voltage, various local LC bulk will tend to align at different pretilt angles θ_i (as presented in Figure 8(a)) so as to form many scattering domains.

Second, it is believed that the presence of the puddle-like micro-structures had promoted the formation of the polymer strands both on the substrate surface and in the LC bulk. And the distribution and structure of the polymer strands formed directly affect the PSLC cell scattering performances. Scanning electron microscope (SEM) pictures of the polymer networks formed showing that there are many polymer-clusters located on the inhomogeneous surface (Figure 8(b)) while there are only little and sparse polymer found on the smooth vertical PI only surface (Figure 8(c)). For SEM samples preparation, PSLC cells were placed in hexane for two days to remove the LCs while keeping the polymer-network structure intact. With a VA PI to reactive mesogen ratio is of 1:35, it has also been

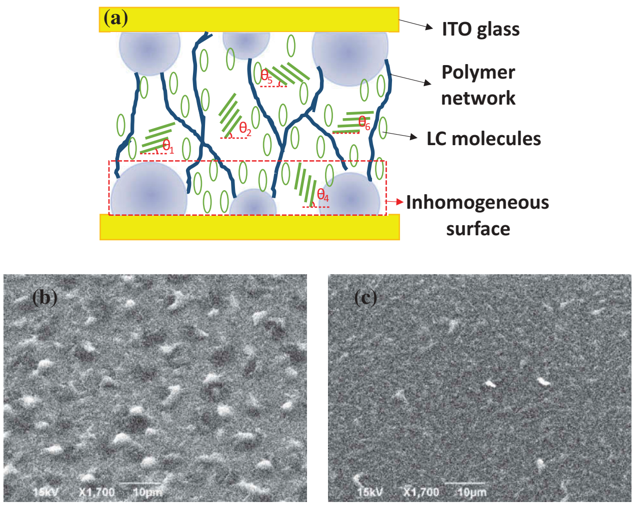

Figure 8. (Colour online) (a) Random pretilt domains schematic at power-on state based on inhomogeneous alignment surface; (b) and (c) top view of polymer network formed in inhomogeneous surface and smooth surface, respectively.

demonstrated that optimised haze performance is achieved (Figure 5(b)). Third, a same type of reactive mesogen is used both in the surface alignment layer and in the LC mixture, it is expected that this greatly strengthens the adhesion of polymer network onto the surface and also makes the formation of polymer strands near the puddles more efficient. This idea is supported by the results in Figure 5(b) where the power-off haze of recipe MR = 0:1 is much higher that MR = 1:0. Here MR = 0:1 represents pure reactive mesogen layer and MR = 1:0 represents pure VA PI layer, both are smooth layers.

5. Conclusion

In conclusion, a reverse mode haze-enhanced PSLCs window device has been developed. The normally clear state is particularly useful for window usage since no electrical power is needed to maintain a window view. Central to this device is the inhomogeneous vertical alignment surface induced by phase separation between a vertical polyimide and planar-type reactive mesogen. It was demonstrated that the surface inhomogeneity greatly helped to enhance the voltage on scattering state (64% haze at 15 V) while maintained a clear state (~4% haze) at power off state. The windows can be switched in less than 3.5 ms and can be operated hundreds of times without any degeneracy trace on switching speed and haze. Taken together, a wealth of potential for our proposed PSLC smart window devices can be realised in applications such as fast switching privacy windows and high performance transparent displays.

Acknowledgements

We are sincerely grateful for the support from Research Grants Council (RGC 614413) and partners State Key Laboratory on Advanced Displays and Optoelectronics Technologies of Hong Kong University of Science and Technology.

Disclosure statement

No potential conflict of interest was reported by the authors. Cuiling MENG and Man Chun Tseng distributed equally to this work.

Funding

This work was supported by the Research Grants Council [614413].

References

[1] Dierking I. Polymer network-stabilized liquid crystals. Adv Mater. 2000;12(3):167–181.
[2] Cupelli D, Nicoletta FP, Manfredi S, et al. Self-adjusting smart windows based on polymer-dispersed liquid crystals. Solar Energy Materials Solar Cells. 2009;93:2008–2012.
[3] Barile CJ, Slotcavage DJ, Hou J, et al. Dynamic windows with neutral color, high contrast, and excellent durability using reversible metal electrodeposition. Joule. 2017;1:133–145.
[4] Lampert CM. Chromogenic smart materials. Materialstoday. 2004;7(3):28–35.
[5] Wang X, Zhang G, Ren H. A large-area optical switch using surface-expandable liquid droplets. J Disp Tech. 2016;12(12): 1565–1569.
[6] Cheng S, Chen C, Lian J. High image quality of transparent display with the proprietary scattering liquid crystal. Inter Disp Manu Conference (IDMC). 2017;1166–1169.
[7] Heo J, Huh J, Yoona T. Sub-millisecond switching of polymer-stabilized liquid crystals with crossed patterned electrodes. AIP Adv. 2015;5:047118.
[8] Stangel L., South S.F. Startup raises $65M for its smart glass-tinting technology. 2017. Available from: http://www.bizjournals.-comsanjosenews20170131south-s-f-startup-raises-65m-forits-smart-lass.html.
[9] Azens A, Granqvist CG. Electrochromic smart windows: energy efficiency and device aspects. J Solid State Electrochem. 2007;7:64–68.
[10] Granqvist CG. Electrochromic materials: out of a niche. News Views Nature Mater. 2006;5:89–90.
[11] Ren H, Xu S, Wu ST. Optical switch based on variable aperture. Opt Lett. 2012;37(9):1421–1423.
[12] Vergaz R, Pena JS, Barrios D, et al. Modelling and electro-optical testing of suspended particle devices. Solar Energy Materials Solar Cells. 2008;92:1483–1487.
[13] Kim Y, Park S, Hong J. Fabrication of flexible polymer dispersed liquid crystal films using conducting polymer thin films as the driving electrodes. Thin Solid Films. 2009;517:3066–3069.
[14] Fuh AY, Shin Z, Yang C, et al. Electrically controllable smart window with greyscale based on polymer-stabilised cholesteric texture films. Liq Cryst. 2016;43(12):1784–1790.
[15] Lu H, Chu Y, Jing S, et al. Characterisation and effect of polymer network deformation in reverse-mode polymer-stabilised cholesteric texture. Liq Cryst. 2016;44(3):437–443.
[16] Chu Y, Yin Z, Sha J, et al. Regulation and control of polymer network deformation in reverse-mode polymer-stabilised cholesteric texture. Liq Cryst. 2016;44(4):688–694.
[17] Alamri SN. The temperature behavior of smart windows under direct solar radiation. Solar Energy Materials Solar Cells. 2009;93:1657–1662.
[18] Haghanifar S, Gao T, Rodriguez T, et al. Ultrahigh-transparency, ultrahigh-haze nanograss glass with fluid-induced switchable haze'. Optica. 2017;4(12):1522–1525.
[19] Mateen F, Oh H, Jung W, et al. Polymer dispersed liquid crystal device with integrated luminescent solar concentrator. Liq Cryst. 2018;45(4):498–506.
[20] Murray J, Ma D, Munday JN. Electrically controllable light trapping for self-powered switchable solar windows. ACS Photonics. 2017;4:1–7.
[21] Shi Z, Shao L, Wang F, et al. Fabrication of dye-doped polymer-dispersed liquid crystals with low driving voltage based on nucleophile-initiated thiol-ene click reaction. Liq Cryst. 2018;45(4):579–585.
[22] Filpo G, Formoso P, Manfredi S, et al. Preparation and characterisation of bifunctional reverse-mode polymer dispersed liquid crystals. Liq Cryst. 2017;44(10):1607–1616.
[23] Tseng MC, Meng CL, Tang ST, et al. Haze free reverse mode liquid crystal light control film with inhomogeneous alignment layer. United States patent, leading intellectual property firm, Beijing, US Appl. No. 62/603,602. 2017.
[24] Jeng SC, Hwang SJ. Controlling the alignment of polyimide for liquid crystal devices. Licensee InTech. 2012;5:88–91.
[25] Meng C, Tseng M, Tang S, et al. Optical rewritable liquid crystal displays without a front polarizer. Opt Lett. 2018;43(4):899–902.
[26] Hu W, Srivastava A, Lin X, et al. Polarization independent liquid crystal gratings based on orthogonal photoalignments. Appl Phys Lett. 2012;100:111116.
[27] Ma L, Li S, Li W, et al. Rationally designed dynamic superstructures enabled by photoaligning cholesteric liquid crystals. Adv Opt Mater. 2015;3:1691–1696.
[28] Tang M, Chen P, Zhang W, et al. Integrated and reconfigurable optical paths based on stacking optical functional films. Opt Express. 2016;24(22):25510–25514.
[29] Ma L, Tang M, Hu W, et al. Smectic layer origami via preprogrammed photoalignment. Adv Mater. 2017;29:1606671–1606677.

[30] Lee S, Kim S, Wu ST. Emerging vertical-alignment liquid-crystal technology associated with surface modification using UV-curable monomer. J SID. 2009;17 (7):551–559.
[31] Yeung FS, Ho JY, Li YW, et al. Variable liquid crystal pretilt angles by nanostructured surfaces. Appl Phys Lett. 2006;88:051910.
[32] Lee CY, Tseng MC, Jacob H, et al. Variable liquid crystal pretilt angle using nano-alignment surfaces. J Soc Inf Disp. 2012;43(1):289–292.
[33] Tseng MC, Fan F, Lee CY, et al. Tunable lens by spatially varying liquid crystal pretilt angles. J Appl Phys. 2011;109:083109.
[34] Yu H, Hsaio C. Comparison of different measurement methods for transmittance haze. Metrologia. 2009;46: S233–S237.
[35] Matsushima T, Okazaki K, Yang Y, et al. New fast response time in-plane switching liquid crystal mode. J SID. 2015.; 43(2): 648–651.
[36] Baek J, Kim K, Kim JC, et al. Fast switching of vertical alignment liquid crystal cells with liquid crystalline polymer networks. Jpn J Appl Phys. 2009;48:056507.
[37] Wan TK, Tsui KC, Kwok HS, et al. Liquid crystal pretilt control by inhomogeneous surfaces. Phys Review E. 2005;72:021711.

RightsLink

Home | Help | Email Support | Ruicong Zhang

Normal- and Reverse-Mode Thermoresponsive Controllability in Optical Attenuation of Polymer Network Liquid Crystals

ACS Publications
Most Trusted. Most Cited. Most Read.

Author: Hiroshi Kakiuchida, Akihiko Matsuyama, Akifumi Ogiwara

Publication: Applied Materials

Publisher: American Chemical Society

Date: May 1, 2019

Copyright © 2019, American Chemical Society

PERMISSION/LICENSE IS GRANTED FOR YOUR ORDER AT NO CHARGE

This type of permission/license, instead of the standard Terms and Conditions, is sent to you because no fee is being charged for your order. Please note the following:

- Permission is granted for your request in both print and electronic formats, and translations.
- If figures and/or tables were requested, they may be adapted or used in part.
- Please print this page for your records and send a copy of it to your publisher/graduate school.
- Appropriate credit for the requested material should be given as follows: "Reprinted (adapted) with permission from {COMPLETE REFERENCE CITATION}. Copyright {YEAR} American Chemical Society." Insert appropriate information in place of the capitalized words.
- One-time permission is granted only for the use specified in your RightsLink request. No additional uses are granted (such as derivative works or other editions). For any uses, please submit a new request.

If credit is given to another source for the material you requested from RightsLink, permission must be obtained from that source.

BACK CLOSE WINDOW

© 2022 Copyright - All Rights Reserved | Copyright Clearance Center, Inc. | Privacy statement | Data Security and Privacy | For California Residents | Terms and ConditionsComments? We would like to hear from you. E-mail us at customercare@copyright.com

ACS APPLIED MATERIALS & INTERFACES

Research Article

Cite This: *ACS Appl. Mater. Interfaces* 2019, 11, 19404−19412

www.acsami.org

Downloaded via HARBIN INST OF TECHNOLOGY on September 19, 2022 at 08:50:49 (UTC).
See https://pubs.acs.org/sharingguidelines for options on how to legitimately share published articles.

Normal- and Reverse-Mode Thermoresponsive Controllability in Optical Attenuation of Polymer Network Liquid Crystals

Hiroshi Kakiuchida,[*,†] Akihiko Matsuyama,[‡] and Akifumi Ogiwara[§]

[†]Structural Materials Research Institute, National Institute of Advanced Industrial Science and Technology, 2266-98 Anagahora, Shimoshidami, Moriyama-ku, Nagoya, Aichi 463-8560, Japan

[‡]Faculty of Computer Science and Systems Engineering, Kyushu Institute of Technology, 680-4 Kawazu, Iizuka, Fukuoka 820-8502, Japan

[§]Department of Electronics Engineering, Kobe City College of Technology, 8-3 Gakuen-higashi, Nishiku, Kobe 651-2194, Japan

Supporting Information

ABSTRACT: A simple nonuniform irradiation method for photopolymerization-induced phase separation (PPIPS) was developed to produce unconventional mesoscale domain structures composed of liquid crystal (LC) and reactive mesogen (RM) phases. The LC/RM phase formations and their molecular orientation ordering through PPIPS were comprehensively investigated as a function of LC/RM molar ratio, curing temperature, and the use of uniform or nonuniform irradiation. Then, two different optical-anisotropic structures that can cause normal- or reverse-mode thermoresponsive light attenuation were formed by nonuniform irradiation at different curing temperatures at the same molar ratios. These two structures consist of mesoscale domains organized with multiaxially orientation-ordered LCs and orientation-disordered RMs for normal-mode thermoresponse and uniaxially orientation-ordered LCs and RMs for reverse-mode thermoresponse. Phase-separation nuclei were generated by nonuniform irradiation at the incipient stage during the PPIPS process under nonuniform irradiation and subsequently coalesced to form mesoscale polymer networks while maintaining their molecular orientation order. This is a promising method to overcome the restraint of structural controllability due to intrinsic material properties and thus to provide unconventional optical and photonic devices, such as thermoresponsive smart windows and thermometric sheets.

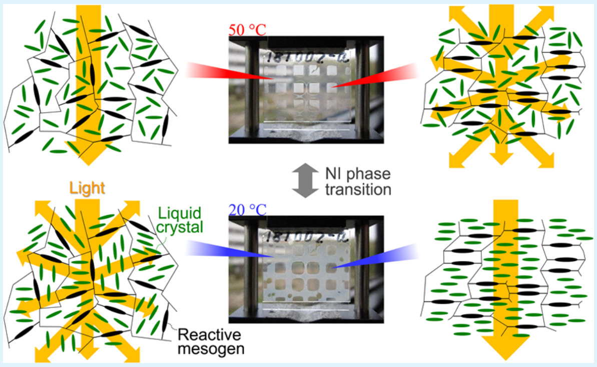

KEYWORDS: *polymer network liquid crystal, reactive mesogen, photopolymerization-induced phase separation, uni-/multiaxial orientation order, nematic-to-isotropic phase transition, thermoresponsive transmittance, smart window, thermometric sheet*

1. INTRODUCTION

Composites of liquid crystals (LCs) and polymers, called polymer network liquid crystals (PNLCs), are often formed through photopolymerization-induced phase separation (PPIPS).[1−3] During PPIPS, monomers are photopolymerized, squeezing LCs out to form polymer-rich domains, and the exuded LCs locally aggregate to form LC(-rich) domains. The textures and formation processes of the LC/polymer phase separation, which are important in soft matter physics to explore novel optical and photonic structures, have been extensively measured and calculated in the points of view of domain morphology, diffusion kinetics, the time-dependent Ginzburg-Landau model and Flory-Huggins theory.[4−7] However, elaborate meso (submicron- to micron-)-scale textures organized by domain shape and molecular orientation are still challenging to design and fabricate for optical and photonic applications because PPIPS processes are strongly affected by the physicochemical properties of the starting materials. Domain shape, or specifically domain size, which is a dominant factor for optical clarity of PNLCs, is mainly influenced by the curing temperature, LC/monomer molar ratio, and monomer functional number.[2,8−10] The larger (smaller) domain size can be achieved by photoexposure at higher (lower) curing temperatures from the raw mixtures of LCs and monomers with larger (smaller) functional numbers.[8,9] On the other hand, the orientation orders of LCs and reactive mesogens (RMs) are larger (smaller) at lower (higher) temperatures using monomers with smaller (larger) functional numbers. According to these intrinsic properties, large domains with a high orientation order are logically impossible for LC/RM systems to have because a high orientation order can be achieved by a low curing temperature for samples consisting of monomers

Received: January 21, 2019
Accepted: May 7, 2019
Published: May 7, 2019

© 2019 American Chemical Society

DOI: 10.1021/acsami.9b01280
ACS Appl. Mater. Interfaces 2019, 11, 19404−19412

with small functional monomers, whereas large domains can be achieved by a high curing temperature for samples consisting of monomers with large functional monomers.

PNLCs possessing mesoscale domains with a high orientation order are useful in modulating light waves by scattering, in response to various extrinsic stimuli such as electric fields, temperature, and light. These multiple stimuli responses are widely applicable to optical and photonic devices, such as smart windows,[11−14] physicochemical sensors,[15] information storage,[16] tunable optical filters and lasers,[17,18] and focusable lenses.[19] To control light propagation efficiently in response to extrinsic stimuli, the mesoscale structures composed of LCs and polymers need to be significantly transformed by involving large refractive-index modulations, such as phase transitions among nematic, homeotropic, cholesteric, and isotropic states. In particular, PNLCs must have mesoscale domains consisting of orientation-ordered or -disordered LCs and RMs for applications to versatile thermoresponsive light diffusers, extending their applications into new industrial, business, healthcare, and domestic fields including thermo-optic devices, switchable windows with valuable uses in energy-saving buildings and automobiles, thermometric sheets for health checks, optical isolators for glare safety, and amenities for comfort.[12−14,20−22]

To overcome the "restriction in mesoscale structural designs of PNLCs", we demonstrate a direct method to control the domain distribution in the PPIPS processes, using nonuniform irradiation. In this method, a light that has an intensity with a spatially nonuniform distribution is irradiated to the sample, and PPIPS progresses with different rates, in response to the irradiation intensity, depending on the location in the samples. Thus, mesoscale domains with various distributions of molecular orientation are produced by employing the nonuniform irradiation method, even though the nature of PPIPS tends to form smaller domains. In this study, three types of phase-separation structures were fabricated, as shown in Scheme 1a−c, and each structure produces transparence (haze) depending on optical (in)homogeneity. As shown in Scheme 1a, if LC molecules are multiaxially ordered in their orientation depending on the randomly oriented polymerized RMs at temperatures (τ) below the nematic-to-isotropic (NI) phase transition temperature (τ_{NI}), the refractive index is mismatched between the domains of the LC phase, and consequently, the PNLC produces light scattering or haze. LCs are transformed from the nematic state to the isotropic state, and the PNLC becomes isotropic or homogenous to produce transparence, when $\tau > \tau_{NI}$. As shown in Scheme 1b, if LC molecules are uniaxially ordered in their orientation along the direction of the orientation of uniaxially polymerized RMs at $\tau < \tau_{NI}$, the refractive index is completely matched for both ordinary and extraordinary components in the whole structure of LC and RM phases and consequently the PNLC produces transparence. When $\tau > \tau_{NI}$, LCs are transformed to the isotropic state with refractive index mismatched to that of the RM phase and consequently the PNLC produces haze. This phase transition does not produce light scattering if the domain size of phase separation is much smaller than the optical wavelength, as shown in Scheme 1c.

2. MATERIALS AND METHODS

We used mixtures of raw materials as shown in Scheme 2. An LC, 4-cyano-4′-hexylbiphenyl, commonly called 6CB (C3154, Tokyo Chemical Industry Co., Ltd.), and a main-chain type of diacrylate

Scheme 1. Three Representative Structures of Phase Separation of LCs and RMs, and Thermoresponsive Switchability in Optical Clarity[a]

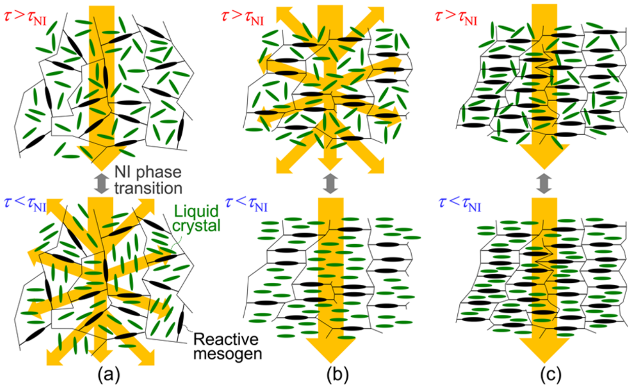

[a](a) Domains of LCs multiaxially ordered in their orientation in randomly oriented polymerized RMs at temperatures (τ) below the NI phase transition temperature (τ_{NI}), and the PNLC is hazy. The LCs are transformed to an isotropic state at $\tau > \tau_{NI}$, and the PNLC becomes transparent. (b) Domains of LCs uniaxially ordered in their orientation along uniaxially oriented polymerized RMs at $\tau < \tau_{NI}$ and the PNLC is transparent. When the LCs are transformed to isotropic state at $\tau > \tau_{NI}$, the PNLC becomes hazy. (c) Structure is the same as (b), but the domain size of phase separation is much smaller than the optical wavelength; thus, the PNLC is transparent, independent of τ.

Scheme 2. Raw Materials for Mixtures

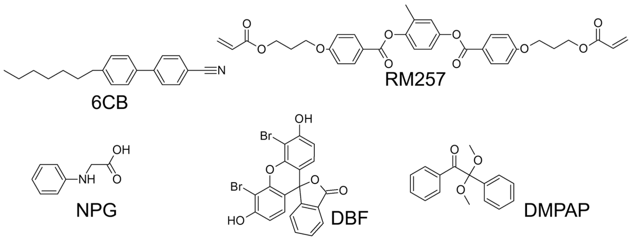

RM, 1,4-bis-[4-(3-acryloyloxypropyloxy)benzoyloxy]-2-methylbenzene (RM257, Merck Corp.), were mixed together. In one experiment, we added a photoinitiator, dibromofluorescein (DBF), and a coinitiator, *N*-phenylglycine (NPG) (Tokyo Chemical Industry Co., Ltd.), to the above mixture and initiated the polymerization of RM257 using a green light source. In the second experiment, we added 2,2-dimethoxy-2-phenylacetophenone (DMPAP) to the above mixture and initiated the polymerization of RM257 using a UV light source. The mixtures were prepared with the molar ratio, *X*, of LCs to the mixture of LCs and RMs ranging from 60 to 95 mol %. DBF and NPG were added to the mixtures at 0.1 and 0.1 wt %, respectively, for PPIPS by the green light source, or DMPAP was added to the mixtures at 1 wt % for PPIPS by the UV light source. Then, the mixtures were stirred at a temperature of 60 °C until they became uniform transparent liquids. 6CB has refractive indices for ordinary and extraordinary rays of n_o = 1.5370 and n_e = 1.6918, respectively, and an NI phase transition temperature of τ_{NI} = 29 °C. RM257 has n_o = 1.5370, n_e = 1.6918, and τ_{NI} = 126 °C. τ_{NI} of the raw mixture of 6CB and RM257 was examined for different mixture ratios, using a differential scanning calorimeter (DSC) (DSC7020, Hitachi High-Tech Science Corp.).

Transparent glass plates with a size of 25 × 20 × 0.7 mm^3 were prepared with a rubbing treatment of polyimide coating on the surfaces (EHC Co., Ltd.) to stabilize horizontally oriented LCs and RMs. Then, the mixtures of LCs and RMs were poured into a gap of 30 μm between a pair of the glasses. Next, these samples were placed on a thermoregulator, and the curing temperature, τ_c, was varied

DOI: 10.1021/acsami.9b01280
ACS Appl. Mater. Interfaces 2019, 11, 19404−19412

between 20 and 60 °C. The samples were cured for 5 min, as shown in Scheme 3a, by photoirradiation using a Nd/YVO$_4$ laser (J150GS, Showa Optronics Co., Ltd.), whose wavelength was 532 nm and intensity was 200 mW/cm^2 on the samples, or using a UV semiconductor laser (TCSQ-03750200, NEOARK Corp.), whose wavelength was 357 nm and intensity was 50 mW/cm^2 on the samples. The intensity distribution of the photoirradiation on the samples was made uniform by direct irradiation or nonuniform by irradiation passing the light through a light diffuser. The light diffuser (DFSQ1-50C02-1500, SIGMAKOKI Co., Ltd.) was located 20 mm before the sample surface to project a nonuniform or speckle pattern onto the samples.[13,14] Post-treatments were the same as those in our previous work,[13,14] as described below. After the photocuring, we irradiated the samples for 5 min, using a lamp with an intensity of 0.8 mW/cm^2 and a wavelength of 365 nm, to polymerize residual RMs.

Scheme 3. Optical Setups for (a) Nonuniform Irradiations and (b) Measurements of Direct Transmittance

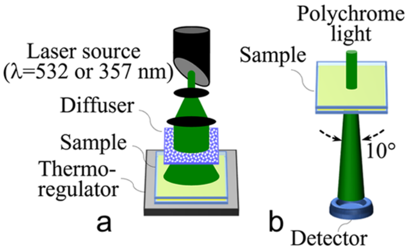

To indicate clarity, we measured direct transmittance, T_d, at a range of temperatures, τ, between 20 and 50 °C and wavelengths, λ, between 200 and 2500 nm, using a spectrophotometer (U4100, Hitachi High-Technologies Corp.). To determine direct transmittance, the samples were irradiated with light at a right angle, and the transmitted light within a divergence angle of 10° was measured, as shown in Scheme 3b. Spatially averaged birefringence of the whole structure of mesoscale phase separation in the PNLCs, Δn, was determined by the measurement of the transmittance spectrum using a spectrophotometer, U4100, with two polarizers at a crossed-Nicols state. The samples were sandwiched by these two polarizers at polarizing azimuths of ±45° from the direction of the rubbing treatment. The direct transmittance at this crossed-Nicols setup repeatedly changes as a function of wavelength if the sample has an optical anisotropy along the rubbing-treatment direction. The wavelengths where the peaks and valleys of the transmittance spectrum are located were analyzed, and the wavelength dispersion of Δn was determined using the four-term Cauchy's equation. A Fourier transform infrared (FTIR) spectrophotometer (Frontier, PerkinElmer Co., Ltd.) was used to examine the LC molecular orientation order and the progress in acrylate polymerization. The absorption area of the peak of 2226 cm^{-1} band was used to examine the orientation order parameter, S, of the LCs that possess cyano groups (−C≡N).[23] The areas were measured separately at polarizations parallel ($A_\parallel$) and perpendicular ($A_\perp$) to the direction of the rubbing treatment, and S was estimated as $(A_\parallel - A_\perp)/(A_\parallel + 2A_\perp)$.[24] The residual monomers were calculated from the absorption area around 1635 cm^{-1}, where this absorption arises from carbon double bonds (−C═C−) in acryloyl groups.[25,26] The infrared absorption areas around 2226 and 1635 cm^{-1} were determined between the spectral curve and the baseline tangent to the curve. The size of the phase-separation domains and the distribution of the optical anisotropy were examined with a polarizing optical microscope (POM) (MT9430, Meiji Techno Co., Ltd.) at the crossed-Nicols state where one polarizer before the sample and the other polarizer behind it were set at polarization azimuths of +45 and −45°, respectively, from the rubbing-treatment direction. The cross section of the polymer phase was observed using a scanning electron microscope (SEM) (S-4300, Hitachi High-Technologies Corp.), after using methanol to rinse away the LC molecules from the cross section.

From the measurements of Δn and S, the molecular orientation of LC and RM can be separately analyzed, and the phase-separation structure and its thermoresponsive behavior can be deduced. As shown in Scheme 1b,c, if LC molecules are uniaxially ordered in their orientation along the uniaxially oriented polymerized RMs at $\tau < \tau_{NI}$, Δn decreases but remains above zero with the rise in τ, whereas S decreases to zero, since the polymerized RMs maintain their uniaxial order even at $\tau > \tau_{NI}$. On the other hand, as shown in Scheme 1a, if LC molecules are multiaxially ordered in randomly oriented polymerized RMs at $\tau < \tau_{NI}$, both Δn and S decrease to zero with the rise in τ.

3. RESULTS AND DISCUSSION

The orientation order of LC molecules aggregated as mesoscale domains changes due to NI phase transition as a function of temperature and, consequently, the clarity thermoresponsively changes between transparence and haze. The direct transmittance (T_d) was measured at different temperatures for samples prepared through uniform irradiation at different curing temperatures and from mixtures with various molar ratios of LCs to the mixture of LCs and RMs (X). T_d values measured at different τ values for two representative samples are shown in Figure 1a,b. Sample I, prepared at a curing temperature (τ_c) of 60 °C, has a normal-mode thermoresponsive transmittance change, that is, from haze to transparence with the rise in τ. Sample II, prepared at a τ_c of 20 °C, has a reverse-mode thermoresponsive transmittance change, that is, from transparence to haze with the rise in τ. The spectra of T_d reversibly change in response to τ, and the magnitude of the change is larger at shorter wavelengths, which typically results from light scattering behavior. Figure 2a shows transmittance−temperature (T_d−τ) plots of these two samples. T_d for all of the samples inflects at approximately 30 °C in τ. This inflection point is close to the NI phase transition temperature (τ_{NI}) of the LC used here. This agreement suggests that the thermoresponsive switchability between transparent and hazy states is due to the NI phase transition behavior in LC molecules in the PNLCs. Figure 2b shows a contour map of the thermoresponsive transmittance change, ΔT_d, as a function of preparation parameters, X and τ_c, where $\Delta T_d = T_d\,(50\ °C) - T_d\,(20\ °C)$. The samples with large X prepared at high τ_c show the normal-mode thermoresponsive transmittance change ($\Delta T_d > 0$), as expressed by the bluish color. Particularly, at an X of 90 mol % and τ_c of 60 °C, ΔT_d was larger than 80%. The samples prepared at low τ_c show the reverse-mode thermoresponsive transmittance change ($\Delta T_d < 0$), as expressed by the reddish color in Figure 2b, although the magnitude of the change,

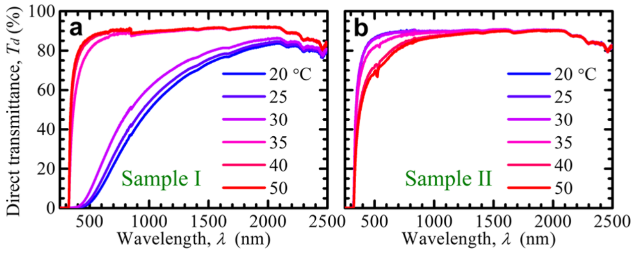

Figure 1. Direct transmittance spectra recorded at temperatures between 20 and 50 °C for (a) sample I and (b) sample II, and the preparation conditions are described in the text.

DOI: 10.1021/acsami.9b01280
ACS Appl. Mater. Interfaces 2019, 11, 19404−19412

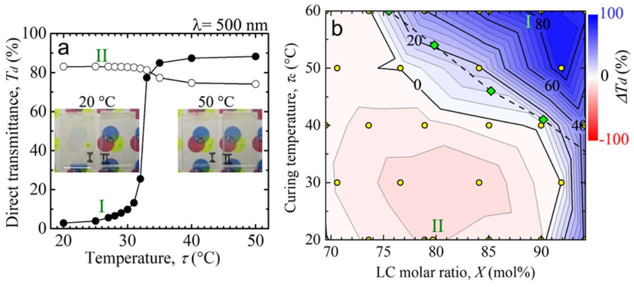

Figure 2. Thermoresponse in direct transmittance for samples obtained by uniform irradiation. (a) Transmittance at a wavelength of 500 nm (T_d), as a function of temperature (τ), for two samples (I and II) obtained at different preparation conditions; the insets are the snapshots of samples I and II at temperatures τ = 20 and 50 °C, placed 20 mm away in front of pictures, where the scale bar indicates 10 mm. (b) Magnitude of thermoresponsive change in the direct transmittance (ΔT_d), as a function of curing temperature (τ_c) and molar ratio of LCs to the mixture of LCs and RMs (X), and the magnitude (ΔT_d) is defined in the text. The green diamonds with broken lines are τ_{NI} of the raw mixtures measured by DSC (Suppl. 1), where τ_{NI} = 65 °C at X = 70.4 mol % and τ_{NI} = 34 °C at X = 95.2 mol %. The yellow circles are the measurement points used to produce the contour lines. The contours were determined simply in the linear interpolations between the measurements. Sample I was prepared at τ_c = 60 °C and X = 90 mol %, and sample II was prepared at τ_c = 20 °C and X = 80 mol %.

|ΔT_d|, is less than 10%, which is small relative to that of the normal-mode thermoresponse (|ΔT_d| ≈ 80%). Green diamonds with broken lines in the figure, which exhibit τ_{NI} of the raw mixtures determined by DSC measurements (Supporting Information Figure S1), suggest that the difference between normal- and reverse-mode thermoresponses is related to the orientation order of the raw materials during PPIPS to fabricate the PNLCs. However, the PNLCs formed through PPIPS at τ_c above τ_{NI} of the raw materials have a large normal-mode thermoresponse, whereas those formed through PPIPS at τ_c below τ_{NI} have a small or zero reverse-mode thermoresponse. Samples with different X were observed at temperatures below and above τ_{NI} by a POM with a crossed-Nicols state, as shown in Figure 3. The micrographs evenly or unevenly change in response to temperature, depending on X and τ_c, and they are not completely dark, except the micrograph taken at τ = 50 °C for the sample prepared at X = 90 mol % and τ_c = 60 °C. The lack of complete darkness indicates that the samples possess optical anisotropy that depolarizes the incident light. Cross sections of samples I and II were observed by SEM, as shown in the same figure. The domains are not exhibited or are too small to be discerned for the samples prepared at lower τ_c, whereas they have a micron scale for the samples prepared at higher τ_c. This τ_c dependence of the domain size can be explained by some established work: the diffusion process of LCs and monomers is more restricted at a lower τ_c and, consequently, the growth of the domains is hindered.[8,27]

Our objective regarding applications is to have a large clarity change in the reverse-mode thermoresponse, but this is hindered by the fact that at low τ_c the LC and RM phases separate with domain sizes too small to produce an intense light scattering. Here, we employed a nonuniform irradiation method using a light diffuser to forcibly control the domain size during PPIPS. Transmittance spectra measured at different temperatures for two representative samples are shown in Figure 4a,b. Normal-mode thermoresponse remained large in sample III, whereas reverse-mode thermoresponse appeared larger in sample IV, by comparison with the results in Figure 1.

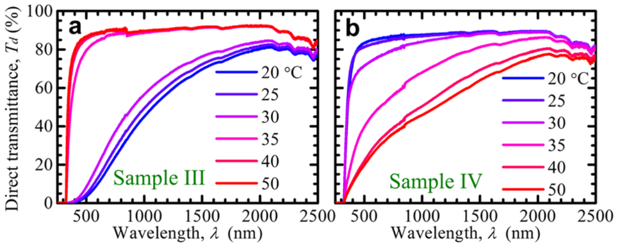

Figure 4. Direct transmittance spectra recorded at temperatures between 20 and 50 °C for (a) sample III and (b) sample IV, and the preparation conditions are described in the text.

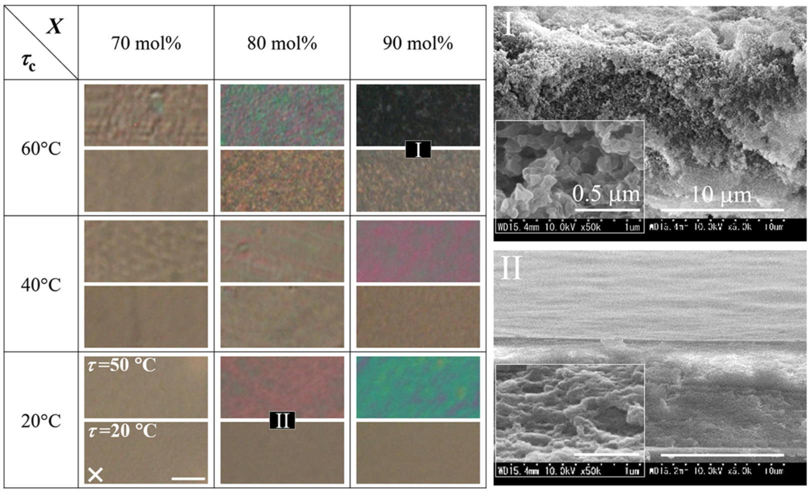

Figure 3. POM images for samples obtained by uniform irradiation. The samples were prepared at different curing temperatures (τ_c) and molar ratios of LCs to the mixture of LCs and RMs (X). The micrographs were taken at temperatures τ = 20 and 50 °C at a crossed-Nicols state, as expressed by a cross in the figure. The scale bar indicates 10 μm. Samples I and II are exhibited as the micrographs of (X = 90 mol %, τ_c = 60 °C) and (X = 80 mol %, τ_c = 20 °C), respectively, and were observed by SEM as shown on the right-hand side.

DOI: 10.1021/acsami.9b01280
ACS Appl. Mater. Interfaces 2019, 11, 19404−19412

Figure 5a,b shows the temperature dependence of T_d and a color contour map of ΔT_d, respectively, for the samples

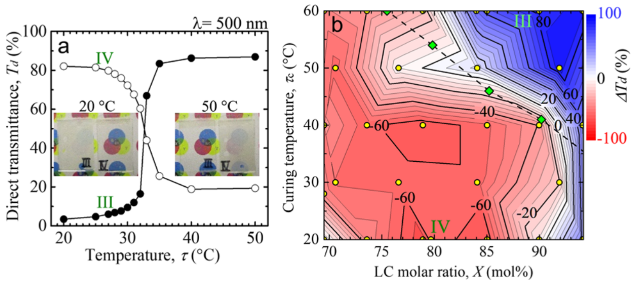

Figure 5. Thermoresponse in direct transmittance for samples obtained by nonuniform irradiation. (a) Transmittance at a wavelength of 500 nm (T_d), as a function of temperature (τ), for two samples (III and IV) obtained at different preparation conditions; the insets are the snapshots of samples III and IV at temperatures τ = 20 and 50 °C, placed 20 mm away in front of pictures, where the scale bar indicates 10 mm. (b) Magnitude of thermoresponsive change in the direct transmittance (ΔT_d), as a function of curing temperature (τ_c) and molar ratio of LCs to the mixture of LCs and RMs (X), and the magnitude (ΔT_d) is defined in the text. The figures are shown in the same form as Figure 2. Sample III was prepared at τ_c = 60 °C and X = 90 mol %, and sample IV was prepared at τ_c = 20 °C and X = 80 mol %.

prepared through nonuniform irradiation in the same styles as those in Figure 2. A large $|\Delta T_d|$ is exhibited in reverse-mode thermoresponse for the samples prepared at low τ_c as well as in normal-mode thermoresponse for the samples prepared at high τ_c. The samples were observed by POM and SEM, as shown in Figure 6. As a comparison with the micrographs in Figure 3, the brightness distribution for most of the samples in Figure 6 unevenly changes in response to temperature. In particular, the micrographs at lower τ_c have a greater tendency to exhibit the brightness change between homogeneous at τ = 20 °C and inhomogeneous at τ = 50 °C. According to the observation by SEM, and POM as well, phase separation with a mesoscale domain size was formed through PPIPS under nonuniform irradiation, even though such phase separation could not be achieved by uniform irradiation.

The process of mesoscale phase separation through PPIPS under nonuniform irradiation was observed by a POM, as shown in Figure 7. The micrographs were taken at τ = 50 °C

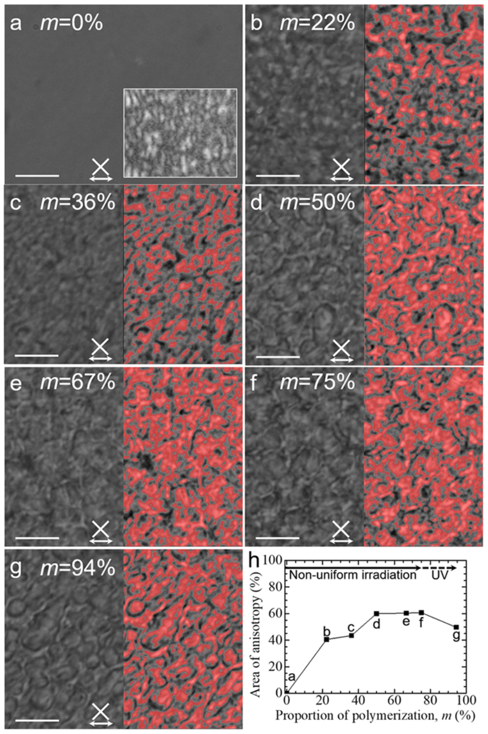

Figure 7. POM images taken at different elapsed times during PPIPS and the progress of anisotropic area as a function of the proportion of polymerization. (a) shows the POM image before nonuniform irradiation. The inset shows the intensity distributions of the nonuniform irradiation. (b), (c), (d), (e), (f), and (g) POM images at proportions of polymerization, m = 22, 36, 50, 67, 75, and 94%, respectively, where the right-half areas of the respective images are the same micrographs after image processing to emphasize areas (red color) that are brighter than the average brightness of the image. (h) Sum of areas that are brighter than the average brightness of the images [red areas in (b)−(g)], as a function of m, where the polymerization was quantified by the decrease in the infrared peak area of the 1615 cm^{-1} band due to C ═C bonds.

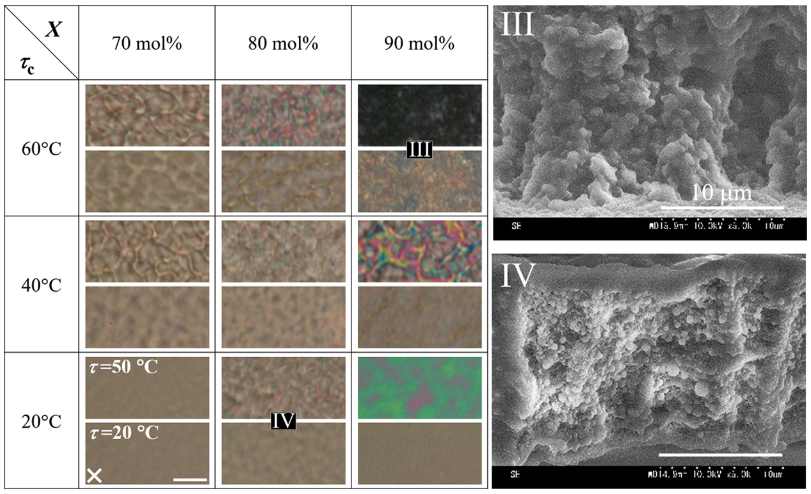

Figure 6. POM and SEM images for samples obtained by nonuniform irradiation. The figures are shown in the same form as Figure 3. Samples III and IV are exhibited as the micrographs of (X = 90 mol %, τ_c = 60 °C) and (X = 80 mol %, τ_c = 20 °C), respectively.

DOI: 10.1021/acsami.9b01280
ACS Appl. Mater. Interfaces 2019, 11, 19404−19412

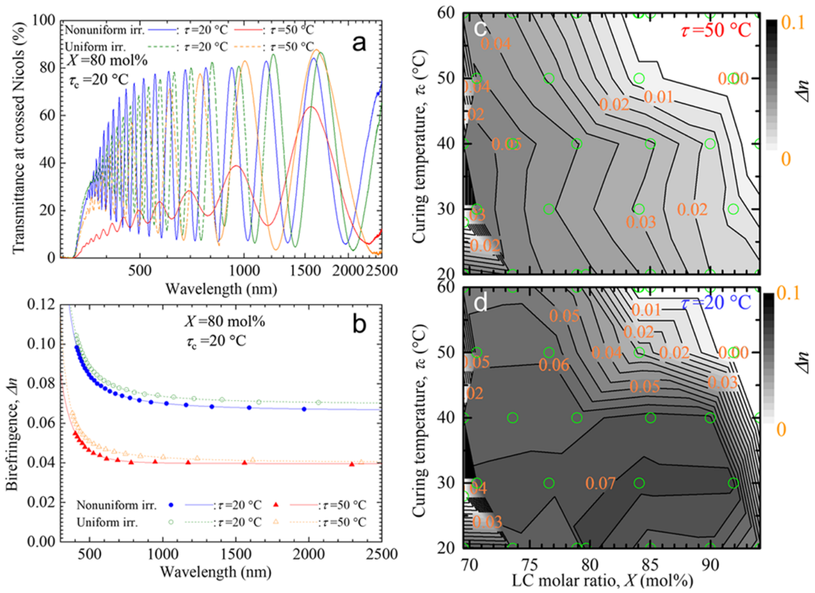

Figure 8. Birefringence (Δn) along the direction of the rubbing treatment estimated by transmittance spectrum. (a) Transmittance spectra recorded in a crossed-Nicols state at temperatures τ = 20 and 50 °C for samples obtained by uniform or nonuniform irradiation (samples II and IV, respectively). (b) Wavelength dispersion of Δn estimated from the wavelengths where peaks and valleys are located in the transmittance spectra in (a), where solid and broken curves are fitted to the four-term Cauchy's equation. The contour maps are Δn at a wavelength of 1000 nm at (c) τ = 50 °C and (d) τ = 20 °C, as a function of the curing temperature (τ_c) and the molar ratio of LCs to the mixture of LCs and RMs (X), where the circles are the measurement points to produce the contour lines. The contours were produced simply in the linear interpolations between the measurements.

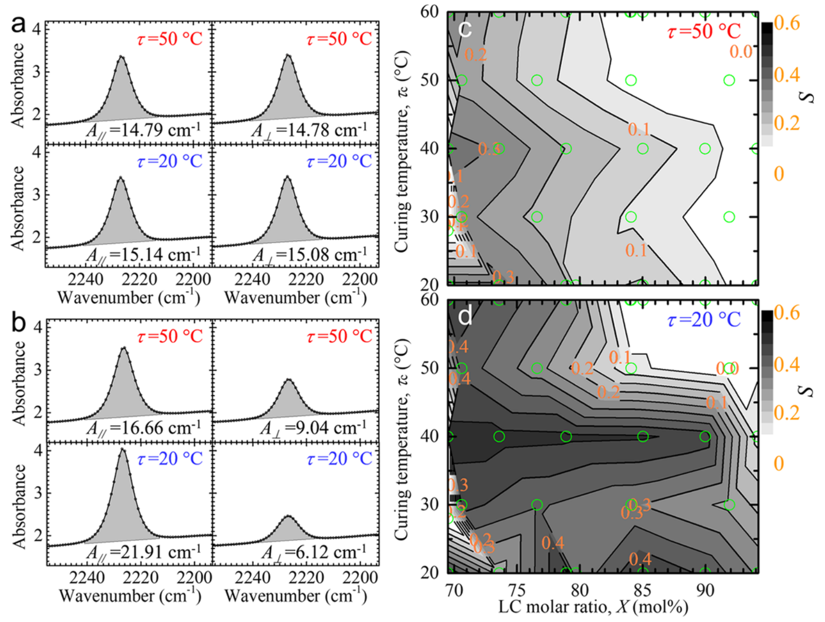

Figure 9. Orientation order parameter (S) of LC molecules along the direction of the rubbing treatment estimated by the polarization of the FTIR absorption spectrum. FTIR absorption was measured at polarizations parallel and perpendicular to the direction of the rubbing treatment at temperatures τ = 20 and 50 °C. (a) and (b) Absorption spectra of sample I (with uniform irradiation at τ_c = 60 °C and X = 90 mol %) and sample IV (with nonuniform irradiation at τ_c = 20 °C and X = 80 mol %), respectively. Absorption areas ($A_{\parallel}$ and $A_{\perp}$) at (c) τ = 50 °C and (d) τ = 20 °C, which were estimated between the spectral curve and the baseline tangent to the curve, as expressed by gray color in Figure 9a,b, are plotted as a function of the curing temperature (τ_c) and the molar ratio of LCs to the mixture of LCs and RMs (X), where the circles are the measurement points to produce the contour lines. The contours were produced simply in the linear interpolations between the measurements.

for different times of PPIPS. Figure 7a is the image of the sample before irradiation, where the inset shows the intensity distribution of speckle patterns projected on the sample by the nonuniform irradiation. The images on the left-hand side in Figure 7b−g are the snapshots during PPIPS. The images on the right-hand side are the same micrographs after image processing, to emphasize areas that are brighter than the average brightness of the image. Optical anisotropy and isotropy are observed as brightness and darkness, respectively. In the micrographs measured at a τ of 50 °C, LCs (τ_{NI} = 29

DOI: 10.1021/acsami.9b01280
ACS Appl. Mater. Interfaces 2019, 11, 19404−19412

°C) appear to be orientation-disordered and exhibit darker regions, whereas RMs (τ_{NI} = 126 °C) appeared to be orientation-ordered and exhibit brighter regions. In the incipient stage of PPIPS (Figure 7b,c), droplet-shape patterns formed, most likely from the speckle patterns. As PPIPS progressed (Figure 7d–f), the patterns transformed from droplet shapes to reticular shapes. Since the progress of the polymerization became inefficient under irradiation with a green laser after about 5 min, the samples were subsequently irradiated by a UV lamp for 5 min to polymerize the residual monomers. This additional uniform UV irradiation successfully promoted PPIPS since the contrast of bright and dark patterns was sharpened (Figure 7g). Figure 7h shows areas of optical anisotropy, as a function of the proportion of polymerization obtained by FTIR measurements. The spatial intensity distribution of the nonuniform irradiation causes mesoscale domain size of incipient dropletlike polymerized RMs without disturbing their orientation order. The domain-shape transformation suggests that the nonuniform irradiation determines the domain size in the incipient stage but does not contribute to the subsequent growth of the domains toward reticular structures.

The transmittance spectra were recorded at the crossed-Nicols state to determine birefringence, Δn. Figure 8a shows the transmittances measured at τ = 20 and 50 °C for sample II (uniform irradiation) and sample IV (nonuniform irradiation). The transmittances periodically change as a function of wavelength because the samples have uniaxial optical anisotropy along the direction of the rubbing treatment. By analyzing the peak and valley locations in the transmittance, the wavelength dispersion of Δn was determined as shown in Figure 8b, where the curves are fitted to Cauchy's equation. The values of Δn were obtained at temperatures of τ = 50 and 20 °C for all of the samples and are plotted as a function of X and τ_c, as shown in Figure 8c,d, respectively. The dependence of Δn on τ is directly connected to the thermoresponsive transmittance change. Δn at τ = 20 °C was relatively larger for the samples prepared at lower τ_c, and Δn at τ = 50 °C was relatively larger for the samples prepared at a lower X. The magnitude of the thermoresponsive change in Δn depends on X and τ_c, that is, there is a larger change at a larger X and at lower τ_c. On the other hand, Δn approaches zero, as X and τ_c increase.

The thermoresponsive change in Δn arises from the NI phase transition of LC phases in the PNLCs, based on the evidence we show. The order parameter of LC molecular orientation, S, is convenient for examining the NI phase transition phenomena. S was determined at τ = 20 and 50 °C by FTIR absorption measurements at around 2225 cm^{-1}. Figure 9a,b shows the absorption spectra of sample I (X = 90 mol %, τ_c = 60 °C, uniform irradiation) and sample IV (X = 80 mol %, τ_c = 20 °C, nonuniform irradiation), respectively, where the measurements were carried out at two polarization azimuths of parallel and perpendicular to the direction of the rubbing treatment at temperatures of τ = 20 and 50 °C. S at τ = 50 and 20 °C is plotted as a function of X and τ_c, in Figure 9c,d, respectively. S was almost the same, independent of whether uniform or nonuniform irradiation was used. When the samples stayed at τ = 20 °C, as shown in Figure 9d, S is high at low τ_c or low X. When τ = 50 °C, as shown in Figure 9c, S decreases from the values at τ = 20 °C and, particularly at large X, it becomes zero.

Combining the results of the POM and SEM images, Δn, and S, as shown in Figures 3, 6, 8, and 9, respectively, we can deduce that the microscopic phase-separation structures produced under nonuniform irradiation are as follows: phase-separation structures that do not produce any haze must be optically homogeneous. The normal-mode thermoresponsive PNLCs (samples I and III in Figures 2a and 5a, respectively), which were fabricated at high τ_c by both uniform and nonuniform irradiation to the samples with large X, possess a polymer network composed of RMs polymerized in random directions (Scheme 1a). In this structure, at τ below τ_{NI}, LCs in different domains are orientation-ordered but with different axes of orientation, although the substrates are rubbing-treated. Structures composed of LC domains possessing such multiaxial orientation orders produce haze since refractive indices are mismatched between the LC domains. When τ rises above τ_{NI}, as LCs become orientation-disordered, their refractive indices converge on an isotropic value that matches that of the polymerized orientation-disordered RMs and consequently the optical clarity switches from haze to transparence. The reverse-mode thermoresponsive PNLCs (sample IV in Figure 5a), which were fabricated by nonuniform irradiation at low τ_c to the samples with X approximately equal to 80 mol %, possess a polymer network composed of RMs polymerized in a uniaxial direction along the rubbing-treatment direction (Scheme 1b). In this structure, at τ below τ_{NI}, LCs are uniaxially orientation-ordered along the same direction as the polymerized RMs over the sample. The refractive indices completely agree between LC and RM phases for every polarization azimuth and, consequently, the PNLCs exhibit transparence. When τ is above τ_{NI}, the LCs become orientation-disordered, whereas the polymerized RMs remain uniaxially orientation-ordered. This structural change brings about the mismatching of the refractive indices between the LC and RM phases and consequently switches the optical clarity from transparence to haze. We can deduce the PNLC structures more deeply from the result of S, as shown in Figure 9. The value of S for the reverse-mode thermoresponsive PNLCs fabricated particularly at lower τ_c at lower X did not reach zero at τ = 50 °C or higher than τ_{NI}. This result indicates that some LC molecules in the reverse-mode thermoresponsive PNLCs are in the nematic state, even at temperatures higher than τ_{NI}, and suggests that there is scope for improvement of the reverse-mode thermoresponsiveness by producing phase-separation structures that can release these immobile LCs.

Since these two different polymerized structures, as shown in Scheme 1a,b, are individually fabricated by only controlling τ_c, the mixture of normal- and reverse-mode thermoresponses can be achieved by superimposed exposures at different τ_c, as shown in the transmittance spectra and snapshots in Figure 10a and a movie (Movie S1, Supporting Information). This technique, where the patterned thermoresponsiveness was achieved by double exposure at different curing temperatures, may allow the production of more elaborate light shutters or sensors with a less complicated fabrication process, such as thermometric sheets for recreation, healthcare, and safety. The reverse-mode thermoresponsive behavior can be fabricated by nonuniform irradiation using a UV laser light source. Figure 10b shows the direct transmittance spectra recorded at temperatures between 20 and 50 °C. Nonuniform irradiation with a UV laser light source can produce reverse-mode thermoresponsive structures, although the fabrication con-

DOI: 10.1021/acsami.9b01280
ACS Appl. Mater. Interfaces 2019, 11, 19404–19412

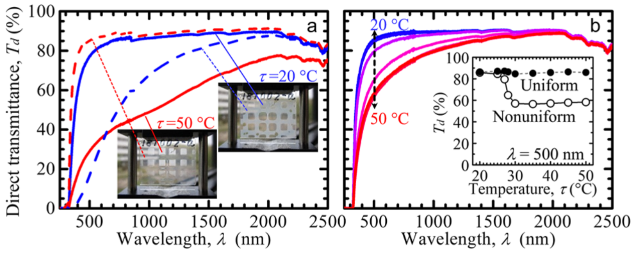

Figure 10. Samples produced by advanced techniques using (a) superposed exposure method or (b) different wavelength light sources. (a) A demonstration-grid-patterned sample including normal- and reverse-mode thermoresponsive switchability in optical clarity, and direct transmittance spectra recorded at 20 and 50 °C. See the thermoresponsive behaviors in Movie S1 in the Supporting Information. Normal-mode thermoresponse, where the optical clarity increases (decreases) as the device warms (cools), can be achieved by the combination of isotropic-polymerized RMs with multiaxial orientation-ordered LCs, as shown in Scheme 1a. Reverse-mode, where the optical clarity decreases (increases) as the device warms (cools), can be achieved by the combination of uniaxially anisotropic-polymerized RMs with uniaxial orientation-ordered LCs, as shown in Scheme 1b. The snapshots in the inset were taken at τ = 20 and 50 °C for the sample prepared by two-step exposures: A sample of X = 85 mol % was nonuniformly irradiated with a photomask at τ_c = 20 °C and then uniformly irradiated without a photomask at τ_c = 60 °C. (b) Direct transmittance spectra of a reverse-mode thermoresponsive PNLC (X = 85 mol %, and 1 mol % of DMPAP) prepared through nonuniform irradiation with a UV laser. The inset shows the temperature dependence of T_d for the samples prepared through uniform and nonuniform irradiations with the UV laser.

ditions are not still optimized, unlike the conditions with a green laser.

4. CONCLUSIONS

Two representative optical-anisotropic structures that can produce normal- and reverse-mode thermoresponsive light attenuations were simply produced through different curing temperatures from the same material recipes. These two structures consist of mesoscale domains organized with multiaxially orientation-ordered LCs and orientation-disordered RMs for a normal-mode thermoresponse and uniaxially orientation-ordered LCs and RMs for a reverse-mode thermoresponse. The nonuniform irradiation method first demonstrated here generates phase-separation nuclei that coalesce to mesoscale domains through PPIPS, even though the nature of the PPIPS tends to form much smaller domains. This is a promising method to overcome the restraint of structural controllability due to intrinsic material properties and thus to provide unconventional optical and photonic devices. Specifically, structures that provide a reverse-mode thermoresponse in light transmittance can be used for smart windows that reduce and increase solar transmission in response to ambient temperature.

■ ASSOCIATED CONTENT

Supporting Information

The Supporting Information is available free of charge on the ACS Publications website at DOI: 10.1021/acsami.9b01280.

DSC measurements of the raw mixtures of LCs and RMs at six different molar ratios between 70 and 95 mol % to determine their τ_{NI} (Figure S1) (PDF)

Thermoresponsive behavior of the optical clarity of a patterned sample that includes normal- and reverse-mode thermoresponses depending on the location of demonstration grid patterns (MP4)

■ AUTHOR INFORMATION

Corresponding Author

*E-mail: h.kakiuchida@aist.go.jp.

ORCID

Hiroshi Kakiuchida: 0000-0002-0084-2545

Notes

The authors declare no competing financial interest.

■ ACKNOWLEDGMENTS

Drs. E. Kobayashi (SAGA-LS) and A. Hozumi (AIST) gave suggestions on our experiments. Dr. Y. Yamada (AIST) gave a hint about smart windows. This work was partly supported by JSPS KAKENHI grant numbers 15K05257 and 17K06408.

■ REFERENCES

(1) Boots, H. M. J.; Kloosterboer, J. G.; Serbutoviez, C.; Touwslager, F. J. Polymerization-Induced Phase Separation. 1. Conversion−Phase Diagrams. *Macromolecules* **1996**, *29*, 7683−7689.

(2) Amundson, K.; Blaaderen, A.; Wiltzius, P. Morphology and Electro-Optic Properties of Polymer-Dispersed Liquid-Crystal Films. *Phys. Rev. E* **1997**, *55*, 1646−1654.

(3) Dai, H.; Chen, L.; Zhang, B.; Si, G.; Liu, Y. J. Optically Isotropic, Electrically Tunable Liquid Crystal Droplet Arrays Formed by Photopolymerization-Induced Phase Separation. *Opt. Lett.* **2015**, *40*, 2723−2726.

(4) Serbutoviez, C.; Kloosterboer, J. G.; Boots, H. M. J.; Touwslager, F. J. Polymerization-Induced Phase Separation. 2. Morphology of Polymer-Dispersed Liquid Crystal Thin Films. *Macromolecules* **1996**, *29*, 7690−7698.

(5) Bowley, C. C.; Crawford, G. P. Diffusion Kinetics of Formation of Holographic Polymer-Dispersed Liquid Crystal Display Materials. *Appl. Phys. Lett.* **2000**, *76*, 2235−2237.

(6) Kyu, T.; Nwabunma, D.; Chiu, H.-W. Theoretical Simulation of Holographic Polymer-Dispersed Liquid-Crystal Films via Pattern Photopolymerization-Induced Phase Separation. *Phys. Rev. E: Stat., Nonlinear, Soft Matter Phys.* **2001**, *63*, No. 061802.

(7) Soulé, E. R.; Abukhdeir, N. M.; Rey, A. D. Thermodynamics, Transition Dynamics, and Texturing in Polymer-Dispersed Liquid Crystals with Mesogens Exhibiting a Direct Isotropic/Smectic-A Transition. *Macromolecules* **2009**, *42*, 9486−9497.

(8) Drzaic, P. S. Phase Separation in the Binodal and Spinodal Regime. *Liquid Crystal Dispersions, Series on Liquid Crystals*; World Scientific Publishing, 1995; Vol. *1*, pp 81−88.

(9) Pogue, R. T.; Natarajan, L. V.; Siwecki, S. A.; Tondiglia, V. P.; Sutherland, R. L.; Bunning, T. J. Monomer Functionality Effects in the Anisotropic Phase Separation of Liquid Crystals. *Polymer* **2000**, *41*, 733−741.

(10) Kakiuchida, H.; Ogiwara, A.; Matsuyama, A. Multiple Bragg Diffractions with Different Wavelengths and Polarizations Composed of Liquid Crystal/Polymer Periodic Phases. *ACS Omega* **2017**, *2*, 6081−6090.

(11) Oh, S.-W.; Baek, J.-M.; Kim, S.-H.; Yoon, T.-H. Optical and Electrical Switching of Cholesteric Liquid Crystals Containing Azo Dye. *RSC Adv.* **2017**, *7*, 19497−19501.

(12) Guo, S.-M.; Liang, X.; Zhang, C.-H.; Chen, M.; Shen, C.; Zhang, L.-Y.; Yuan, X.; He, B.-F.; Yang, H. Preparation of a Thermally Light-Transmittance-Controllable Film from a Coexistent System of Polymer-Dispersed and Polymer-Stabilized Liquid Crystals. *ACS Appl. Mater. Interfaces* **2017**, *9*, 2942−2947.

(13) Kakiuchida, H.; Ogiwara, A. Reverse-Mode Thermoresponsive Light Attenuators Produced by Optical Anisotropic Composites of

Nematic Liquid Crystals and Reactive Mesogens. *Opt. Mater.* **2018**, *78*, 273−278.
(14) Kakiuchida, H.; Ogiwara, A. *Chapter 20 Smart Windows, High Quality Liquid Crystal Displays and Smart Devices Volume 2: Surface Alignment, New Technologies and Smart Device Applications*; Ishihara, S.; Kobayashi, S.; Ukai, Y., Eds.; The Institution of Engineering and Technology, 2019; pp 341−359.
(15) Lai, Y.-T.; Kuo, J.-C.; Yang, Y.-J. Polymer-Dispersed Liquid Crystal Doped with Carbon Nanotubes for Dimethyl Methylphosphonate Vapor-Sensing Application. *Appl. Phys. Lett.* **2013**, *102*, No. 191912.
(16) Jayalakshmi, V.; Hegde, G.; Naira, G. G.; Prasad, S. K. Photo-Controlled Conformation-Assisted Permanent Optical Storage Device Employing a Polymer Network Liquid Crystal. *Phys. Chem. Chem. Phys.* **2009**, *11*, 6450−6454.
(17) Khandelwal, H.; Debije, M. G.; White, T. J.; Schenning, A. P. H. J. Electrically tunable infrared reflector with adjustable bandwidth broadening up to 1100 nm. *J. Mater. Chem. A* **2016**, *4*, 6064−6069.
(18) Inoue, Y.; Yoshida, H.; Inoue, K.; Shiozaki, Y.; Kubo, H.; Fujii, A.; Ozaki, M. Tunable Lasing from a Cholesteric Liquid Crystal Film Embedded with a Liquid Crystal Nanopore Network. *Adv. Mater.* **2011**, *23*, 5498−5501.
(19) Ren, H.; Fan, Y.-H.; Lin, Y.-H.; Wu, S.-T. Tunable-Focus Microlens Arrays Using Nanosized Polymer-Dispersed Liquid Crystal Droplets. *Opt. Commun.* **2005**, *247*, 101−106.
(20) Pranga, M.; Czuprynski, K. L.; Klosowicz, S. J. Polymer-Dispersed Liquid Crystals for Thermosensitive Foils and Paints. *Proc. SPIE* **2000**, *4147*, 394−399.
(21) Kakiuchida, H.; Tazawa, M.; Yoshimura, K.; Ogiwara, A. Thermal Control of Transmittance/Diffraction States of Holographic Structures Composed of Polymer and Liquid Crystal Phases. *Sol. Energy Mater. Sol. Cells* **2010**, *94*, 1747−1752.
(22) Ogiwara, A.; Kakiuchida, H. Thermally Tunable Light Filter Composed of Cholesteric Liquid Crystals with Different Temperature Dependence. *Sol. Energy Mater. Sol. Cells* **2016**, *157*, 250−258.
(23) Noble-Luginbuhl, A. R.; Blanchard, R. M.; Nuzzo, R. G. Surface Effects on the Dynamics of Liquid Crystalline Thin Films Confined in Nanoscale Cavities. *J. Am. Chem. Soc.* **2000**, *122*, 3917−3926.
(24) Ward, I. M. Determination of Molecular Orientation by Spectroscopic Techniques. *Adv. Polym. Sci.* **1985**, *66*, 81−115.
(25) Matsukawa, K.; Matsuura, Y.; Inoue, H.; Hanafusa, K.; Nishioka, N. Development of Photocuring Acrylic/Silica Organic-Inorganic Hybrid for Negative Resists. *J. Photopolym. Sci. Technol.* **2001**, *14*, 181−184.
(26) Colthup, N. B.; Daly, L. H.; Wiberley, S. E. Carobonyl Compounds In *Introduction to Infrared and Raman Spectroscopy*; 3rd ed.; Academic Press, 1995; Chapter 9, pp 289−325.
(27) Mcintyre, W. D.; Soane, D. S. Controlled Phase Separation of Polymer-Liquid Crystal Mixtures for Reversible Optical Data Storage. *Appl. Opt.* **1990**, *29*, 1658−1665.

DOI: 10.1021/acsami.9b01280
ACS Appl. Mater. Interfaces 2019, 11, 19404−19412
