# Supplementary material for: Advanced liquid crystal-based switchable optical devices for light protection applications: principles and strategies
Source: Light Sci Appl. 2023 Jan 3;12:11. doi: 10.1038/s41377-022-01032-y (PMC9807646; doi:10.1038/s41377-022-01032-y)
Supplement: Supplementary file 10 — Fig 11 copyright promotion [file 41377_2022_1032_MOESM10_ESM.pdf]

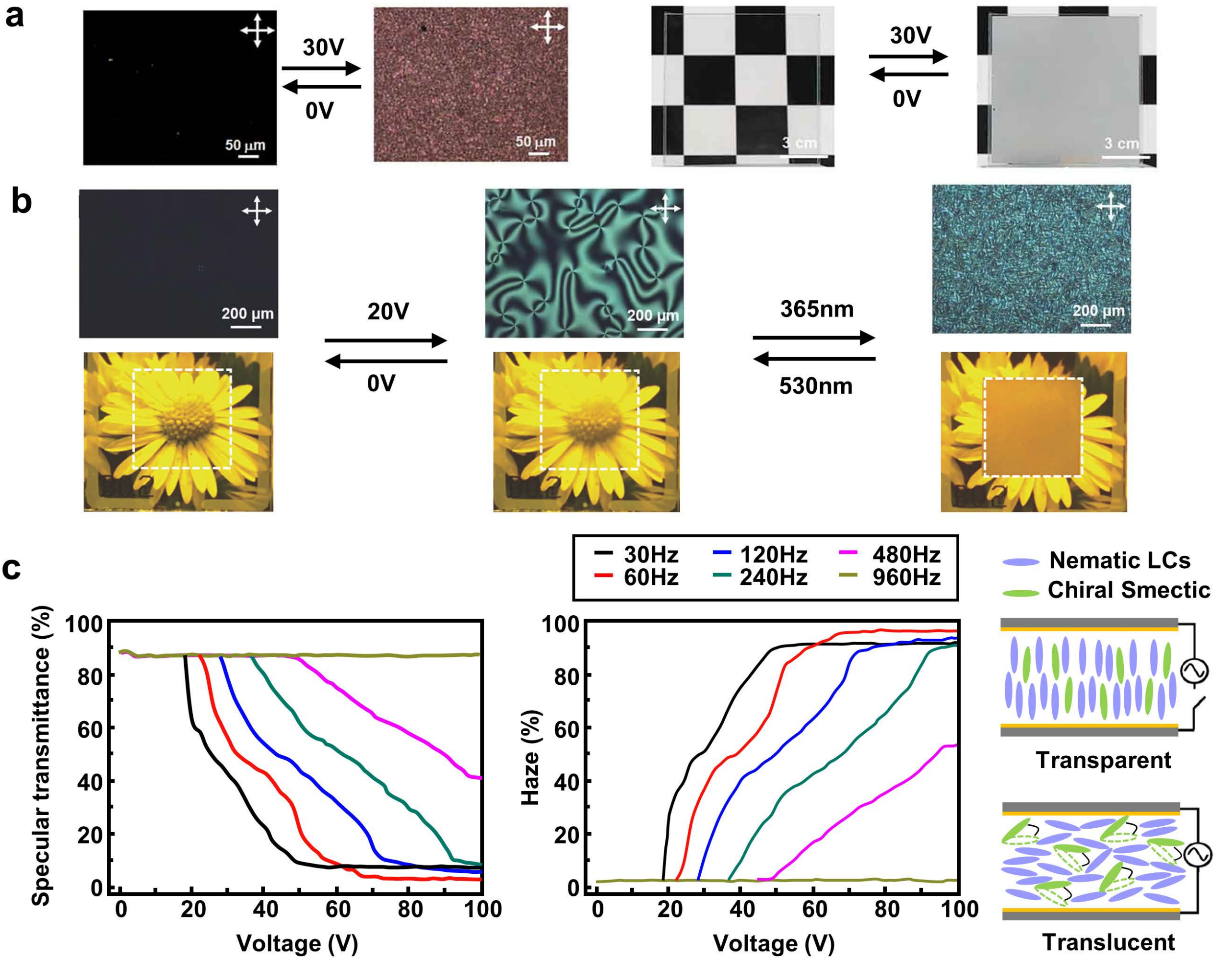

a
30V
0V
50 µm
50 µm
30V
0V
3 cm
3 cm
b
200 µm
20V
0V
200 µm
365nm
530nm
200 µm
c
30Hz
60Hz
120Hz
240Hz
480Hz
960Hz
Specular transmittance (%)
Voltage (V)
Haze (%)
Voltage (V)
Nematic LCs
Chiral Smectic
Transparent
Translucent

QQ邮箱 mail.qq.com

Permissions Request [ref:_00D0Y35Iji._5007R3P9mUw:ref]

发件人：permissionrequest<permissionrequest@tandf.co.uk>
时　间：2022年10月7日(星期五) 晚上11:08
收件人：ruicong.zhang<ruicong.zhang@hrtcn.org>

Taylor & Francis Group
an informa business

Our Ref: la/tlct/02542226

07/10/2022

Springer Nature c/o Ruicong Zhang

Figure 2 from Yuanyuan Zhan, Han Lu, Mingliang Jin & Guofu Zhou (2020) Electrohydrodynamic instabilities for smart window applications, Liquid Crystals, 47:7, 977-983, DOI: 10.1080/02678292.2019.1692929

Figure 2 & 3 from Cuiling Meng, Man Chun Tseng, Shu Tuen Tang, Chen Xiang Zhao, Sze Yan Yeung & Hoi Sing Kwok (2019) Normally transparent smart window with haze enhancement via inhomogeneous alignment surface, Liquid Crystals, 46:3, 484-491, DOI: 10.1080/02678292.2018.1508764

Thank you for your attached correspondence requesting permission to reproduce the above material in your forthcoming publication entitled ‘Advanced liquid crystal-based switchable optical devices for light protection applications: principles and strategies' to be published in Light: Science & Applications, published by Springer Nature.

This usage falls under the provisions of the STM Agreement, we shall be pleased to waive our fees, and to grant you non exclusive world rights in all languages, covering print and e-journal usage of your Work all editions, on the condition that:

1. The original source of publication and Taylor & Francis Ltd, are acknowledged in the caption, including a reference to the Journal’s web site: www.tandfonline.com

2. You do not license to any third party permission to reproduce this copyrighted material, in any form, and at any time.

3. This permission does not cover any third party copyrighted work which may appear in the material requested. Please ensure you have checked all original source details for the rights holder.

4. Any alterations/adaptions to the original work must be approved by the original author(s) of the article.

Thank you for your interest in our Journals.

Yours sincerely,

Lee-Ann Anderson | Senior Permissions Executive, Journals
Taylor & Francis Group
4 Park Square, Milton Park, Abingdon, OX14 4RN
Permissions e-mail: journalpermissions@tandf.co.uk
Web: www.tandfonline.com
Tel: +44 (0)20 8052 0659

Taylor & Francis is a trading name of Informa UK Limited,
registered in England under no. 1072954

Disclaimer: T&F publish Open Access articles in our subscription priced journals, please check if the article you are interested in is an OA article and if so, which licence was it published under.

Before printing, think about the environment.

ref:_00D0Y35Iji._5007R3P9mUw:ref

JOHN WILEY AND SONS LICENSE TERMS AND CONDITIONS

Sep 20, 2022

This Agreement between Harbin Institute of Technology -- Ruicong Zhang ("You") and John Wiley and Sons ("John Wiley and Sons") consists of your license details and the terms and conditions provided by John Wiley and Sons and Copyright Clearance Center.

License Number	5393000613135
License date	Sep 20, 2022
Licensed Content Publisher	John Wiley and Sons
Licensed Content Publication	Advanced Functional Materials
Licensed Content Title	Light-Driven Electrohydrodynamic Instabilities in Liquid Crystals
Licensed Content Author	Danqing Liu, Guofu Zhou, Dirk J. Broer, et al
Licensed Content Date	Apr 6, 2018
Licensed Content Volume	28
Licensed Content Issue	21
Licensed Content Pages	7
Type of Use	Journal/Magazine
Requestor type	University/Academic
Is the reuse sponsored by or associated with a pharmaceutical or medical products company?	no
Format	Print and electronic
Portion	Figure/table
Number of figures/tables	1
Will you be translating?	No
Circulation	200 - 499
Title of new article	Advanced liquid crystal-based switchable optical devices for light protection applications: principles and strategies
Lead author	Ruicong Zhang, Zhibo Zhang, Jiecai Han, Lei Yang, Jiajun li, Zicheng Song Tianyu Wang, Jiaqi Zhu
Title of targeted journal	Light: Science & Applications
Publisher	Springer Nature
Expected publication date	Nov 2022
Portions	Figure 2
Requestor Location	Harbin Institute of Technology<br>No. 92, Xidazhi Street, Nangang District<br><br>Harbin, 150080<br>China<br>Attn: Harbin Institute of Technology
Publisher Tax ID	EU826007151
Total	**0.00 USD**
Terms and Conditions	

TERMS AND CONDITIONS

This copyrighted material is owned by or exclusively licensed to John Wiley & Sons, Inc. or one of its group companies (each a"Wiley Company") or handled on behalf of a society with which a Wiley Company has exclusive publishing rights in relation to a particular work (collectively "WILEY"). By clicking "accept" in connection with completing this licensing transaction, you agree that the following terms and conditions apply to this transaction (along with the billing and payment terms and conditions

established by the Copyright Clearance Center Inc., ("CCC's Billing and Payment terms and conditions"), at the time that you opened your RightsLink account (these are available at any time at http://myaccount.copyright.com).

Terms and Conditions

- The materials you have requested permission to reproduce or reuse (the "Wiley Materials") are protected by copyright.

- You are hereby granted a personal, non-exclusive, non-sub licensable (on a stand-alone basis), non-transferable, worldwide, limited license to reproduce the Wiley Materials for the purpose specified in the licensing process. This license, **and any CONTENT (PDF or image file) purchased as part of your order,** is for a one-time use only and limited to any maximum distribution number specified in the license. The first instance of republication or reuse granted by this license must be completed within two years of the date of the grant of this license (although copies prepared before the end date may be distributed thereafter). The Wiley Materials shall not be used in any other manner or for any other purpose, beyond what is granted in the license. Permission is granted subject to an appropriate acknowledgement given to the author, title of the material/book/journal and the publisher. You shall also duplicate the copyright notice that appears in the Wiley publication in your use of the Wiley Material. Permission is also granted on the understanding that nowhere in the text is a previously published source acknowledged for all or part of this Wiley Material. Any third party content is expressly excluded from this permission.

- With respect to the Wiley Materials, all rights are reserved. Except as expressly granted by the terms of the license, no part of the Wiley Materials may be copied, modified, adapted (except for minor reformatting required by the new Publication), translated, reproduced, transferred or distributed, in any form or by any means, and no derivative works may be made based on the Wiley Materials without the prior permission of the respective copyright owner.**For STM Signatory Publishers clearing permission under the terms of the STM Permissions Guidelines only, the terms of the license are extended to include subsequent editions and for editions in other languages, provided such editions are for the work as a whole in situ and does not involve the separate exploitation of the permitted figures or extracts,** You may not alter, remove or suppress in any manner any copyright, trademark or other notices displayed by the Wiley Materials. You may not license, rent, sell, loan, lease, pledge, offer as security, transfer or assign the Wiley Materials on a stand-alone basis, or any of the rights granted to you hereunder to any other person.

- The Wiley Materials and all of the intellectual property rights therein shall at all times remain the exclusive property of John Wiley & Sons Inc, the Wiley Companies, or their respective licensors, and your interest therein is only that of having possession of and the right to reproduce the Wiley Materials pursuant to Section 2 herein during the continuance of this Agreement. You agree that you own no right, title or interest in or to the Wiley Materials or any of the intellectual property rights therein. You shall have no rights hereunder other than the license as provided for above in Section 2. No right, license or interest to any trademark, trade name, service mark or other branding ("Marks") of WILEY or its licensors is granted hereunder, and you agree that you shall not assert any such right, license or interest with respect thereto

- NEITHER WILEY NOR ITS LICENSORS MAKES ANY WARRANTY OR REPRESENTATION OF ANY KIND TO YOU OR ANY THIRD PARTY, EXPRESS, IMPLIED OR STATUTORY, WITH RESPECT TO THE MATERIALS OR THE ACCURACY OF ANY INFORMATION CONTAINED IN THE MATERIALS, INCLUDING, WITHOUT LIMITATION, ANY IMPLIED WARRANTY OF MERCHANTABILITY, ACCURACY, SATISFACTORY QUALITY, FITNESS FOR A PARTICULAR PURPOSE, USABILITY, INTEGRATION OR NON-INFRINGEMENT AND ALL SUCH WARRANTIES ARE HEREBY EXCLUDED BY WILEY AND ITS LICENSORS AND WAIVED BY YOU.

- WILEY shall have the right to terminate this Agreement immediately upon breach of this Agreement by you.

- You shall indemnify, defend and hold harmless WILEY, its Licensors and their respective directors, officers, agents and employees, from and against any actual or threatened claims, demands, causes of action or proceedings arising from any breach of this Agreement by you.

- IN NO EVENT SHALL WILEY OR ITS LICENSORS BE LIABLE TO YOU OR ANY OTHER PARTY OR ANY OTHER PERSON OR ENTITY FOR ANY SPECIAL, CONSEQUENTIAL, INCIDENTAL, INDIRECT, EXEMPLARY OR PUNITIVE DAMAGES, HOWEVER CAUSED, ARISING OUT OF OR IN CONNECTION WITH THE DOWNLOADING, PROVISIONING, VIEWING OR USE OF THE MATERIALS REGARDLESS OF THE FORM OF ACTION, WHETHER FOR BREACH OF CONTRACT, BREACH OF WARRANTY, TORT, NEGLIGENCE, INFRINGEMENT OR OTHERWISE (INCLUDING, WITHOUT LIMITATION, DAMAGES BASED ON LOSS OF PROFITS, DATA, FILES, USE, BUSINESS OPPORTUNITY OR CLAIMS OF THIRD PARTIES), AND WHETHER OR NOT THE PARTY HAS BEEN ADVISED OF THE POSSIBILITY OF SUCH DAMAGES. THIS LIMITATION SHALL APPLY NOTWITHSTANDING ANY FAILURE OF ESSENTIAL PURPOSE OF ANY LIMITED REMEDY PROVIDED HEREIN.

- Should any provision of this Agreement be held by a court of competent jurisdiction to be illegal, invalid, or unenforceable, that provision shall be deemed amended to achieve as nearly as possible the same economic effect as the original provision, and the legality, validity and enforceability of the remaining provisions of this Agreement shall not

be affected or impaired thereby.

- The failure of either party to enforce any term or condition of this Agreement shall not constitute a waiver of either party's right to enforce each and every term and condition of this Agreement. No breach under this agreement shall be deemed waived or excused by either party unless such waiver or consent is in writing signed by the party granting such waiver or consent. The waiver by or consent of a party to a breach of any provision of this Agreement shall not operate or be construed as a waiver of or consent to any other or subsequent breach by such other party.

- This Agreement may not be assigned (including by operation of law or otherwise) by you without WILEY's prior written consent.

- Any fee required for this permission shall be non-refundable after thirty (30) days from receipt by the CCC.

- These terms and conditions together with CCC's Billing and Payment terms and conditions (which are incorporated herein) form the entire agreement between you and WILEY concerning this licensing transaction and (in the absence of fraud) supersedes all prior agreements and representations of the parties, oral or written. This Agreement may not be amended except in writing signed by both parties. This Agreement shall be binding upon and inure to the benefit of the parties' successors, legal representatives, and authorized assigns.

- In the event of any conflict between your obligations established by these terms and conditions and those established by CCC's Billing and Payment terms and conditions, these terms and conditions shall prevail.

- WILEY expressly reserves all rights not specifically granted in the combination of (i) the license details provided by you and accepted in the course of this licensing transaction, (ii) these terms and conditions and (iii) CCC's Billing and Payment terms and conditions.

- This Agreement will be void if the Type of Use, Format, Circulation, or Requestor Type was misrepresented during the licensing process.

- This Agreement shall be governed by and construed in accordance with the laws of the State of New York, USA, without regards to such state's conflict of law rules. Any legal action, suit or proceeding arising out of or relating to these Terms and Conditions or the breach thereof shall be instituted in a court of competent jurisdiction in New York County in the State of New York in the United States of America and each party hereby consents and submits to the personal jurisdiction of such court, waives any objection to venue in such court and consents to service of process by registered or certified mail, return receipt requested, at the last known address of such party.

WILEY OPEN ACCESS TERMS AND CONDITIONS

Wiley Publishes Open Access Articles in fully Open Access Journals and in Subscription journals offering Online Open. Although most of the fully Open Access journals publish open access articles under the terms of the Creative Commons Attribution (CC BY) License only, the subscription journals and a few of the Open Access Journals offer a choice of Creative Commons Licenses. The license type is clearly identified on the article.

The Creative Commons Attribution License

The Creative Commons Attribution License (CC-BY) allows users to copy, distribute and transmit an article, adapt the article and make commercial use of the article. The CC-BY license permits commercial and non-

Creative Commons Attribution Non-Commercial License

The Creative Commons Attribution Non-Commercial (CC-BY-NC)License permits use, distribution and reproduction in any medium, provided the original work is properly cited and is not used for commercial purposes.(see below)

Creative Commons Attribution-Non-Commercial-NoDerivs License

The Creative Commons Attribution Non-Commercial-NoDerivs License (CC-BY-NC-ND) permits use, distribution and reproduction in any medium, provided the original work is properly cited, is not used for commercial purposes and no modifications or adaptations are made. (see below)

Use by commercial "for-profit" organizations

Use of Wiley Open Access articles for commercial, promotional, or marketing purposes requires further explicit permission from Wiley and will be subject to a fee.

Further details can be found on Wiley Online Library http://olabout.wiley.com/WileyCDA/Section/id-410895.html

Other Terms and Conditions:

v1.10 Last updated September 2015

Questions? customercare@copyright.com or +1-855-239-3415 (toll free in the US) or +1-978-646-2777.

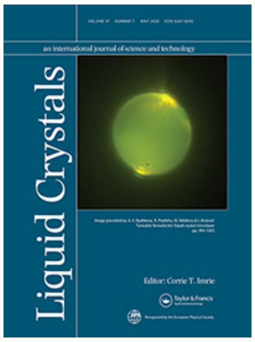

Liquid Crystals

ISSN: (Print) (Online) Journal homepage: https://www.tandfonline.com/loi/tlct20

Electrohydrodynamic instabilities for smart window applications

Yuanyuan Zhan, Han Lu, Mingliang Jin & Guofu Zhou

To cite this article: Yuanyuan Zhan, Han Lu, Mingliang Jin & Guofu Zhou (2020) Electrohydrodynamic instabilities for smart window applications, Liquid Crystals, 47:7, 977-983, DOI: 10.1080/02678292.2019.1692929

To link to this article: https://doi.org/10.1080/02678292.2019.1692929

View supplementary material

Published online: 11 Dec 2019.

Submit your article to this journal

Article views: 505

View related articles

View Crossmark data

Citing articles: 16 View citing articles

Full Terms & Conditions of access and use can be found at
https://www.tandfonline.com/action/journalInformation?journalCode=tlct20

LIQUID CRYSTALS
2020, VOL. 47, NO. 7, 977–983
https://doi.org/10.1080/02678292.2019.1692929

Electrohydrodynamic instabilities for smart window applications

Yuanyuan Zhan[a], Han Lu[a], Mingliang Jin[a,b] and Guofu Zhou[a,b,c]

[a]SCNU-TUE Joint Lab of Device Integrated Responsive Materials (DIRM), National Center for International Research on Green Optoelectronics, South China Normal University, Guangzhou, P. R. China; [b]Guangdong Provincial Key Laboratory of Optical Information Materials and Technology & Institute of Electronic Paper Displays, South China Academy of Advanced Optoelectronics, South China Normal University, Guangzhou, P. R. China; [c]Shenzhen Guohua Optoelectronics Tech. Co. Ltd., Shenzhen, China

ABSTRACT
Electrohydrodynamic instability in liquid crystals is investigated for smart window applications. By comparing different species of ions doped in liquid crystals, the positive charge of the organic part of the salt is the origin of the generation of vortexes and leads to the activation of electrohydrodynamic instability. By replacing conventional electrolytes with zwitterions, compared to electrolyte-doped liquid crystals, zwitterion-doped liquid crystals have a broader range of optimal frequency from 10 Hz to 5 kHz. The devices can be switched between the transparent state and the light scattering state for thousands of times without showing any fatigue. A scattering colour is obtained by incorporating a dichroic dye in zwitterion-doped liquid crystal. A patterned device is designed and shows a localised light scattering and colour effect in the presence of an electric field. This strategy will make electrohydrodynamic instability possible to enrich the applications across smart windows, projection screens and information displays.

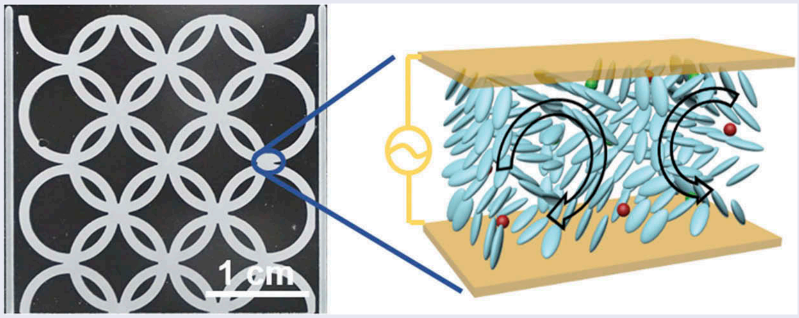

ARTICLE HISTORY
Received 17 July 2019
Accepted 11 November 2019

KEYWORDS
Electrohydrodynamic instability; zwitterion; dichroic dye; smart window; information display

1. Introduction

Electrohydrodynamic instability (EHDI) was first discovered in the 1960s [1]. Since then, it has been drawing much attention from scientists for applications such as liquid crystal (LC) display and beam steering [2,3]. Under a sufficiently high electric field strength, dynamic scattering mode can be induced based on the generation of turbulence originated from ions oscillate between electrodes and leads to light scattering [4]. In the early stage, investigation on EHDI in LC was mainly focused on William Domains, generally driven by a voltage of direct current field based on the injection of ions from electrodes or impurities from the bulk LC [5–7]. With emerging of smart window technology [8–15], light scattering mode is demanding in practice. One option is to reduce electrical conductivity of LC mixture by the generating ions to the host LC and to apply an alternating electric field with a low frequency to oscillate ions to form polydomains [16–18]. For instance, creating zwitterions by illuminating UV can trigger EHDI and therefore generate light scattering effect [19]. This strategy creating a light scattering state by using multiple stimuli is promising in saving energy for smart window applications. Hereby, we thoroughly investigate EHDI by varying species of ions and incorporating dyes in LCs.

2. Experimental section

2.1. Materials

Sorbitan monooleate (J & K Scientific), sodium dodecyl sulphate (Aladdin), decyltrimethylammonium bromide (Sigma-Aldrich), dodecyltrimethylammonium bromide (Sigma-Aldrich), hexadecyl trimethyl

CONTACT Guofu Zhou guofu.zhou@m.scnu.edu.cn
Supplemental data for this article can be accessed here.
© 2019 Informa UK Limited, trading as Taylor & Francis Group

ammonium bromide (Sigma-Aldrich), trimethyloctadecylammonium bromide (Sigma-Aldrich), (Lauryldimethylammonio)acetate (Sigma-Aldrich) and Reichardt's dye (Sigma-Aldrich) were used as ions to dope into a host LC. Negative LCs HNG30400-200 (T_i = 94°C) and dichroic dyes RL013 and non-dichroic dye C1 were purchased from Jiangsu Hecheng Advanced Materials Co., Ltd. Fluorescent dye K160 was purchased from Risk Reactor.

2.2. *Sample preparation*

Commercial cells of 10 μm of cell gap with or without patterns (XGH3030-10, Shenzhen Guohua Optoelectronics Tech. Co., Ltd.) were used for experiments. The samples that used for all the measurements are 3 cm by 3 cm in width and length, unless otherwise specified. The cells were filled with an LC mixture by capillary suction at 110°C. The samples were cooled slowly to 75°C and held for 2 h to eliminate any thermal instability before further cooled down to room temperature. For the comparison of different species of ions in activating EHDI, each sample contains 2.7×10^{-6} mol of ions. Samples only doped with dye was prepared by incorporating 0.3 wt% of the dye in a host LC. Coloured samples that can trigger EHDI were prepared by doping 0.3 wt% of dichroic dye and 0.1 wt% of zwitterionic Reichardt's dye in a host LC.

2.3. *Sample characterisation*

The textures of samples were checked using polarised optical microscopy (POM) (Leica DM2700P) combined with heating and cooling system (Linkam32). Transmission spectra were recorded with a UV/VIS/NIR spectrometer (PerkinElmer 950 and R1 Series, Shanghai Ideaoptics Instrument Co., Ltd). Angle-resolved light scattering spectra were performed by angle-resolved fibre optical spectrometer (R1 series, Shanghai Ideaoptics Instrument Co., Ltd). The alternating electric field with a sine wave was provided by a function generator (AFG1022, Tektronix). The electric signal from the function generator was amplified by a high-voltage linear amplifier (A400, FLC Electronics). The output voltage was measured by an oscilloscope (TBS2000, Tektronix).

3. Results and discussions

We chose a negative LC (Δε = −8.3) with a birefringence of 0.149 (Supporting Information, Table S1 and Figure S1), in the presence of a small amount of cationic electrolyte hexadecyltrimethylammonium bromide (CTAB); initially, on polarised optical microscope, the LC is black which is corresponding to its homeotropic alignment between two electrodes perpendicular to the substrates (Figure 1(a)). By applying an alternating current (AC) field, the birefringence of LC is changed and multi-domains can be observed [19]. This is due to that the long axis of the LC molecule can be reoriented perpendicular to the electric

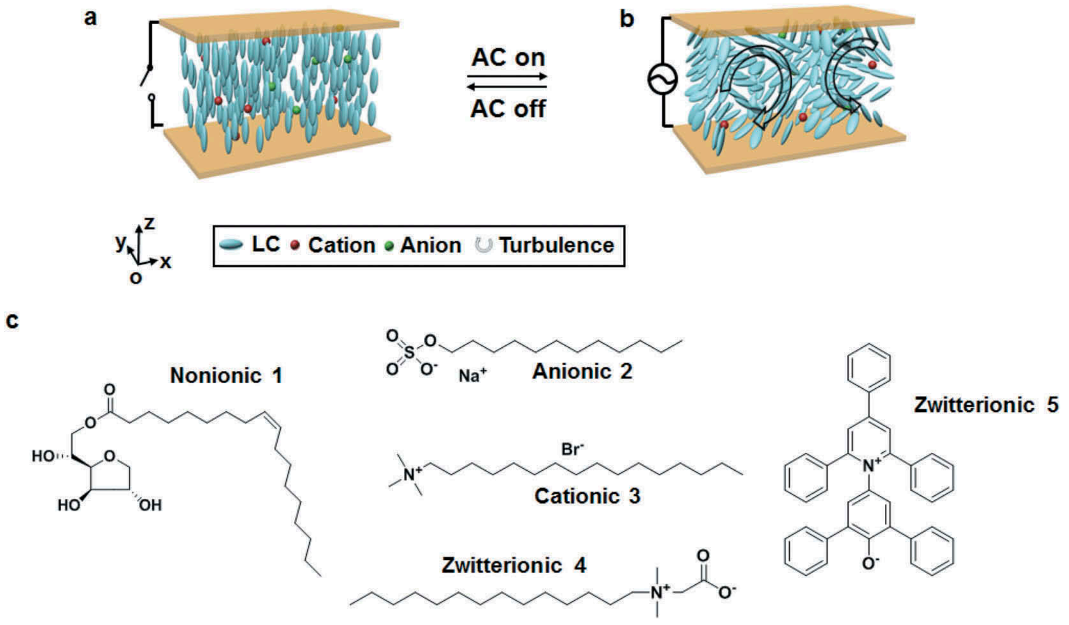

Figure 1. (Colour online) Schematic representation of electrohydrodynamic instability (EHDI) (a) at 0 V and (b) under the application of an alternating electric field. (c) Chemical structures of four different species of ions: the non-ionic **1**, the anionic **2**, the cationic **3** and the zwitterionic **4, 5.**

field line, and at the same time, ions start oscillating between electrodes (Figure 1(b)). The electrical force exerted on the ions can be transferred to the LC by the exchange of momentum. Analogous to thermal-induced Rayleigh–Bernard instability, the LC molecules are set in motion and EHDI is triggered.

We first investigate the influence of species of electrolyte on the activation of EHDI. By comparing three different species of commercial electrolytes, non-ionic molecule **1**, anionic molecule **2** and cationic molecule **3** (Figure 1(c)), only the sample containing cationic electrolyte has a dramatic transmittance decrease during applying the field (Figure 3(a), Supporting Information, Figure S2). This result can be explained by that the positive charge is the main source of movable charge carrier (Supporting Information, Figure S3). The argument is that negative Br^- ions in molecule **3** are barely soluble in the bulk LC mixture, and they also tend to be strongly attracted to opposite charges in the conductive indium tin oxide layers. The positive CTA^+ ions are relatively more soluble in the LC mixture because the N^+ charge is separated by those three methyl groups from the opposite charges and is less attracted. In addition, the aliphatic tails can help the positive CTA^+ ions dissolve more easily in the host LC. On the contrary, anionic molecule **2** can poorly dissolve in the bulk nematic LC though the tiny amount is doped and therefore cannot initiate EHDI, while as expected, the non-ionic molecule **1** cannot activate EHDI due to the lack of charges in the molecule.

The modest solubility of electrolyte in host LCs and accumulation of ions onto electrodes tend to reduce the lifetime of devices. In order to solve those problems, hereby, we propose to replace electrolyte with zwitterion. A zwitterion is a molecule with multi-functional groups of which one possesses a positive and the other possesses a negative electrical charge, and the entire molecule appears electrically neutral [20]. In our previous research, we created zwitterions in the LC upon exposure to UV light, and the effect of EHDI in the presence of zwitterions is comparable to that of LC doped with conventional electrolyte. The principle behind is that zwitterions can oscillate between electrodes following the polarity change of the applied AC electric field and generate chaotic vortices, resulting in macroscopic light scattering. Hereby, we replace the electrolyte with zwitterions (molecules **4, 5**). Transmittance measurement on UV-Vis spectra at 550 nm shows that zwitterion **5** is more efficient in triggering EHDI, and we ascribe this to the larger molecular size of zwitterion **5** which can easily create vortexes when oscillating between electrodes (Figure 2(b), Supporting Information, Figure S4). Therefore, we mainly utilise zwitterion **5** to study EHDI in this work.

The zwitterion **5** shows a maximum absorption around 726 nm in LCs (Supporting Information, Figure S5). To reduce the colour effect originated from the absorption of the zwitterionic dye, we use LC cells with a cell gap of 10 μm. EHDI is characterised on POM and demonstrated macroscopically. When blending a certain amount of the zwitterion with LC, in the absence of electric field, LC shows a homeotropic alignment between electrodes and the sample appears transparent suggesting the zwitterions mixing with LC uniformly (Figure 2(a,c,e)). Transmission spectra show no absorption of the zwitterion dye at field-off state. When the sample is driven by the AC field with stepwise changing the voltage at a frequency of 1 kHz, light scattering occurs. POM image shows that when the voltage birefringence of LC is changed (Figure 2(b)), it leads to a difference of refractive index in each vortex. Those vortexes generate light scattering (Figure 2(d)). Based on ASTM D1003-00, Standard Test Method for Haze and Luminous Transmittance of Transparent Plastics, the haze measured at 30 V_{rms} is 93.68%, indicating that most of the light is scattered at an angle greater than 2.5° from the normal.

When switching on the AC field, the EHDI is developed within 100 ms and followed by the second stage approximately 1 s where the LC molecules speed up to maximum velocity. This result is consistent with the observation that the output current is gradually increasing after switching on the field. Therefore, the rise time of turbulence generation is related to the amount of mobile ions and their velocity. The disappearance of EHDI takes 200 ms (Figure 2(g)). The relaxation of the light scattering is related to reorientation of LC molecules under the influence of the surface anchoring force exerted by the homeotropic alignment layers.

We investigated angle-resolved light scattering by varying an angle of incident light from the normal by measuring the transmitted light intensity as a function of the angle of incidence (Supporting Information, Figure S6). Figure 2(h) shows that at the threshold voltage of 10 V_{rms} activating EHDI, the transmitted intensity decreases with increasing incident angle, which is because the effective path length of the light within the sample increases and therefore more of the light is expected to be scattered with increasing angle. Below the threshold, only negative LC is reoriented by the field and the sample is nearly 100% transparent. However, with further increase in voltage up to 20 V_{rms}, the transmitted intensity shows angular independence, indicating that increase of voltage gives rise to smaller domains with LC molecules dynamically oriented along all directions and a higher velocity of the turbulence.

Next, we compare EHDI in the cationic electrolyte-doped and zwitterion-doped LCs. Transmittance

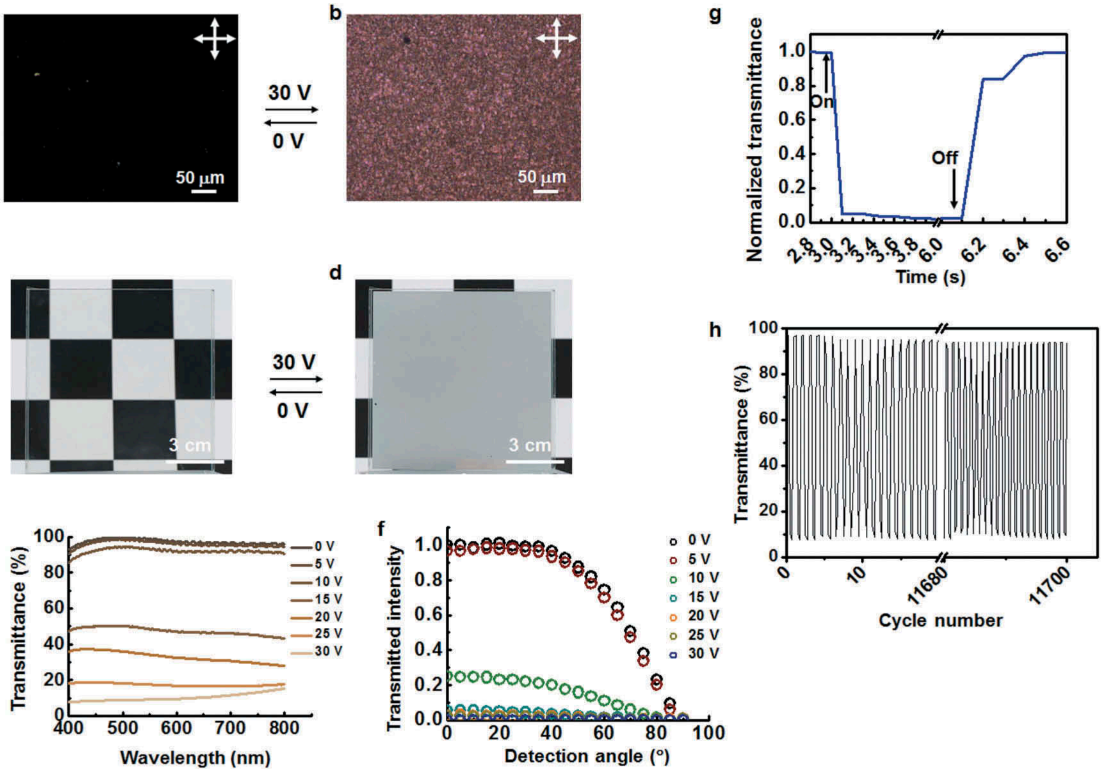

Figure 2. (Colour online) Polarised optical microscope image showing the cell at (a) field-off state and (b) field-on state. Macroscopic images showing the cell switched from (c) the initial transparent state in the absence of the field to (d) the field-on light scattering state. The size of the sample is 9 cm × 9 cm in width and length. The checkboard background is behind the sample with a distance of 10 cm. (e) Transmittance spectra of LC mixture doped with zwitterion at different voltage at 550 nm. (f) Angel-resolved transmitted light intensity measurement. (g) Response time of the window between switch-on and switch-off. The frequency applied is 1 kHz. (h) Thousands of switches between the transparent state and the light scattering state measured on UV/Vis spectroscopy.

measurement indicates that cation–LC and zwitterion–LC show a comparable light scattering effect (Figure 3(a)). The threshold voltage for triggering EHDI in zwitterion–LC is slightly higher, which might be due to the large molecular size of the zwitterion. The current generated from the movement of charge carriers in zwitterion–LC is approximately three times as much as that in cation–LC at a voltage of 30 V_{rms} (Figure 3(b)). This result suggests that less accumulation of the zwitterions on the electrodes takes place. With the measured current, we are able to estimate the power consumption of the window. Following the equation of power $P = V{\cdot}I{\cdot}$, where V is the rms voltage and I is the rms current passing through the window, the window of 9 cm by 9 cm demonstrated in Figure 2(d) consumes 0.649 W of power, and for 24 h, the energy consumed is 0.016 kWh (Supporting Information S7).

The optimal frequency is also investigated by giving the samples a frequency sweep under a sine wave signal generated from function generator. Figure 3(c) shows that the optimal frequency in zwitterion–LC appears to be ranging from 100 Hz to 5 kHz, whereas cationLC has an optimal frequency ranging from 10 to 200 Hz. This difference is originated from the conductivity of the LC mixture which can be regulated by the amount of ions. We proved that by reducing the amount of zwitterions approximately 16 times, the cut-off frequency for EHDI can be lowered and the optimal range can be narrowed (Supporting Information, Figure S8).

Notably, EHDI is dependent on temperature [21]. We investigated the thermal behaviour of EHDI by giving samples heating and cooling treatment (Figure 3(d)). Upon heating up to 85°C where the LC mixture is still nematic, the transmittance increases are shown on both samples. This can be attributed to the decrease in birefringence of LCs and the increase of the conductivity of LC mixture when the temperature increases. Upon cooling, zwitterion–LC shows a reversible transmittance change and can go back to the initial value. Cation–LC shows a transmittance increase during the cooling process. We ascribe this to the consecutive adsorption of ions to the electrodes started from the heating process, and therefore, the amount of movable cations decreases during cooling. At room temperature, zwitterion–LC can be switched more than 11,700 times consecutively since

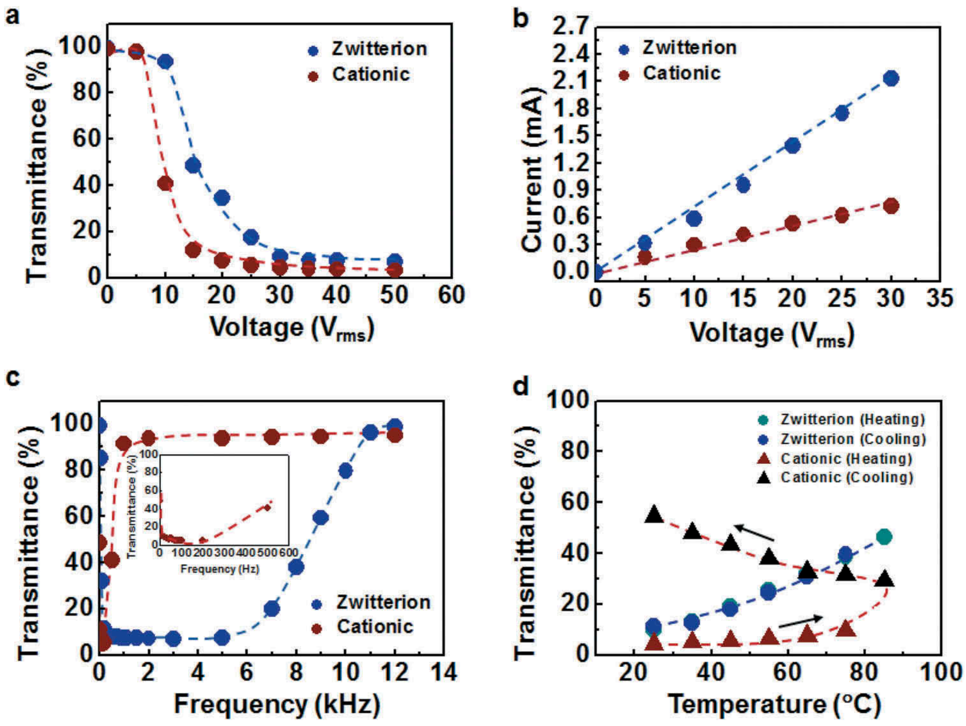

Figure 3. (Colour online) Comparison between cationic electrolyte-doped and zwitterion **5**-doped LCs in EHDI. (a) Light scattering upon the application of voltage at a frequency of 1 kHz. Transmittance was measured at 550 nm. (b) Current passing through the samples. (c) Frequency-dependent transmittance at 30 V_{rms}. (d) Temperature-dependent light scattering upon heating and cooling process, respectively. The sample was applied with a continuous field is 30 V_{rms} at 1 kHz.

during applying the field no heat is generated from the motion of charges. At elevated temperature, the sample can also be switched reversibly without showing any fatigue compared to cation–LC. During the UV-Vis measurement with an applied voltage of 30 V_{rms} for consecutive 25 h, the sample appears to be stable (Supporting Information, Figure S9).

In our previous zwitterionic light-responsive EHDI, the colour effect can only be created when photochromic zwitterionic merocyanine was created under the illumination of UV light. And localised scattering was obtained by placing a mask between the UV source and the sample, which is not convenient for smart window applications [19]. Therefore, we propose to develop a coloured device without extra external stimulus by incorporating dichroic dye into zwitterion–LC. Initially, the dichroic dye can be well aligned with host LC in a homeotropic fashion [22], and therefore, less colour can be exhibited. When addressing the electric field, colour appears based on the molecular orientation of the dichroic dyes. The scattering colour is characterised on UV/Vis spectroscopy. A dye RL013 with a high dichroic ratio (DR) of approximately 12.1 is mixed with LCs containing zwitterion, showing a maximum absorption at 560 nm (Supporting Information, Figure S10). To avoid the absorption band of the dye, light scattering was measured at a wavelength of 700 nm. At the field-off state, the dichroic dye dissolved in LC mixture aligns parallel to the director of LC molecules and shows high transmittance at 560 and 700 nm (Figure 4(a,e)). When applying the AC field, light scattering is induced, and in the meantime, the colour effect is exhibited due to the motion of the dye involved in the vortex (Figure 4(b,e)). Above the optimal frequency, for example, 5 kHz, EHDI steps into dielectric regime and the ions stop oscillating while the dichroic dye within LC can still be reoriented parallel to the electrodes. Therefore, the scattering can be erased while the sample appears purple (Figure 4(c,f)). Transmittance at 560 nm decreases because of the dichroic absorption of the dye, while the transmittance at 700 nm keeps the same due to the inactivation of EHDI (Figure 4(f)). A designed device with patterns is shown in Figure 4(d) (Supporting Information, Figure S11). By patterning conductive indium tin oxide electrodes, scattered colour state with specific patterns can be obtained. Taking a closer look, the scattering can be sharply observed, indicating a high resolution of the device. Another example with a low DR of approximately 3.1 is investigated and demonstrated (Figure 4(h–i)). With decreasing the DR at field-off state, the sample shows a lower transmittance at the

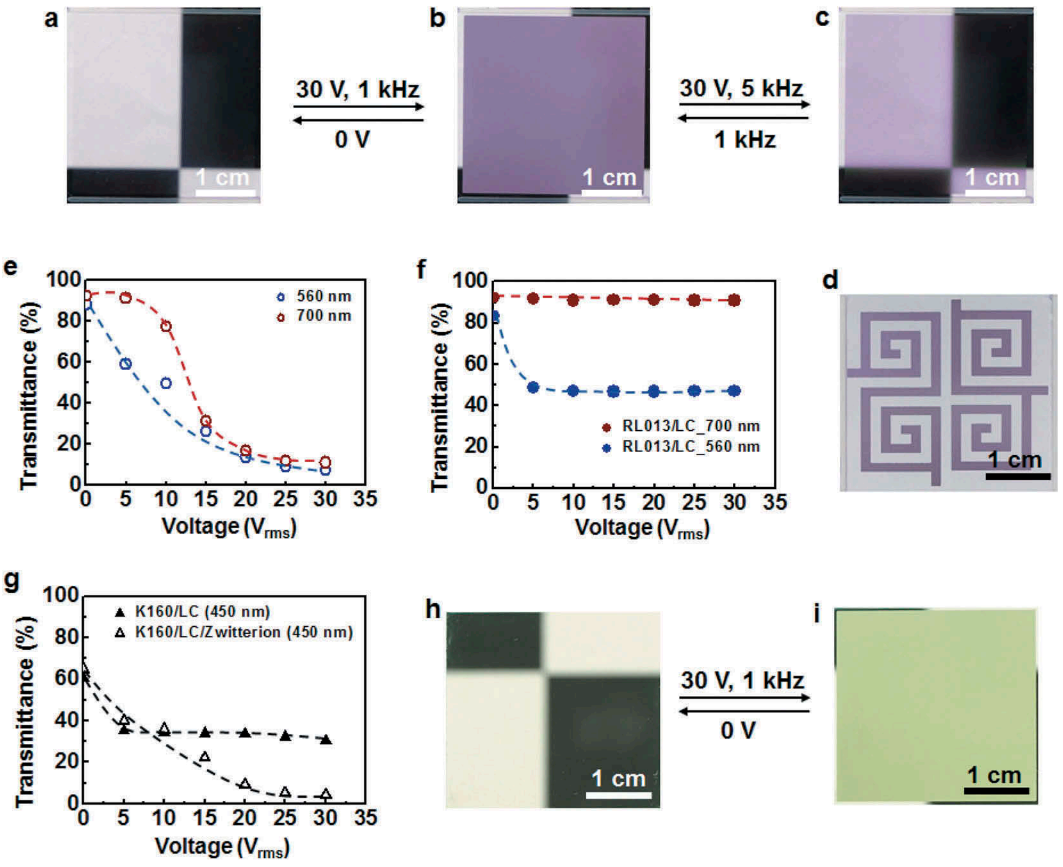

Figure 4. (Colour online) Colour window based on EHDI. Demonstration of the coloured window (a) at off-state, (b) switched to a coloured scattering state in the presence of the AC field of 30 V_{rms} at 1 kHz and (c) adjusted to a coloured state at 5 kHz. (d) Designed device with patterned ITO electrodes switched on to the coloured scattering state by the electricity. (e) Transmittance of the sample containing LC doped with RL013 and zwitterion at a wavelength of 560 and 700 nm, respectively. (f) Transmittance of LCs doped with RL013. (g) Comparison of transmittance change in K160 doped systems. (h, i) Light scattering switched between the transparent state and the scattered colour state.

wavelength of maximum absorption. For instance, K160 shows approximately 25% less absorption than RL013 (Figure 4(g)). In comparison, in dielectric regime, the non-dichroic dye-doped LC shows a small decrease of absorption when increasing the voltage (Supporting Information, Figure S12).

4. Conclusions

In conclusion, by comparing different species of the electrolyte, we have demonstrated that positive charge of the organic part of the salt plays a role in generating turbulence and activating EHDI. By replacing electrolyte with charge neural zwitterion, the optimal frequency of EHDI can be adjusted to the range of 10 Hz to 5 kHz, and the scattering can be reversibly switched by temperature in the presence of the AC field. This result suggests less accumulation of zwitterions on the electrodes, especially at elevated temperatures. We also demonstrated a coloured device by incorporating dichroic dye in zwitterion–LC. The device showed a distinguished contrast of colour between field-off and field-on state. The colour effect in the dielectric regime is dependent on the DR of the dye. By patterning the conductive electrodes, a localised scattering colour has been demonstrated. Based on this thorough investigation on EHDI in ion-doped LC system, we are able to develop devices for smart window applications.

Acknowledgements

We thank Prof. Albert Schenning, Prof. Dirk Broer and Dr Danqing Liu for discussions. The results presented are part of research programs financed by the National Natural Science Foundation of China (51561135014, U1501244), Guangdong Innovative Research Team Program (No. 2013C102), the Program for Changjiang Scholars and Innovative Research Teams in Universities (No. IRT_17R40), Science and Technology Project of Guangdong Province (No. 2018A050501012), Guangdong Provincial Key Laboratory of Optical Information Materials and Technology

(No. 2017B030301007), MOE International Laboratory for Optical Information Technologies and the 111 Project. Additional information is available in the supplementary materials and from the authors.

Disclosure statement

No potential conflict of interest was reported by the authors.

Funding

This work was supported by the National Natural Science Foundation of China [51561135014, U1501244];Guangdong Provincial Key Laboratory of Optical Information Materials and Technology [No. 2017B030301007]; the Program for Changjiang Scholars and Innovative Research Teams in Universities [No. IRT_17R40];Science and Technology Project of Guangdong Province [No. 2018A050501012]; and Guangdong Innovative Research Team Program [No. 2013C102].

References

[1] Williams R. Domains in liquid crystals. J Chem Phys. 1963;39(2):384–388.
[2] Ong HL. Electro-optical properties of guest-host nematic liquid-crystal displays. J Appl Phys. 1988;63(4):1247–1249.
[3] Serak SV, Hrozhyk U, Hwang J, et al. High contrast switching of transmission due to electrohydrodynamic effect in stacked thin systems of liquid crystals. Appl Opt. 2016;55(30):8506–8512.
[4] Heilmeier GH, Zanoni LA, Barton LA. Dynamic scattering: a new electrooptic effect in certain classes of nematic liquid crystals. Proc IEEE. 1968;56(7):1162–1171.
[5] Kai S, Hayashi K, Hidaka Y. Pattern forming instability in homeotropically aligned liquid crystals. J Phys Chem. 1996;100(49):19007–19016.
[6] Funfschilling D, Sammuli B, Dennin M. Patterns of electroconvection in the nematic liquid crystal N4. Phys Rev E. 2003;67(1):016207.
[7] Zhang BR, Kitzerow H. Pattern formation in a nematic liquid crystal mixture with negative anisotropy of the electric conductivity – a long-known system with “Inverse” light scattering revisited. J Phys Chem B. 2016;120(27):6865–6871.
[8] St John WD, Fritz WJ, Lu ZJ, et al. Bragg reflection from cholesteric liquid crystals. Phys Rev E. 1995;51(2):1191–1198.
[9] Yang DK, Chien LC, Doane JW. Cholesteric liquid crystal/polymer dispersion for haze-free light shutters. Appl Phys Lett. 1992;60(25):3102–3104.
[10] Wu ST, Yang DK. Reflective liquid crystal displays. New York, NY: John Wiley & Sons Inc.; 2001.
[11] Hu W, Zhao H, Song L, et al. Electrically controllable selective reflection of chiral nematic liquid crystal/chiral ionic liquid composites. Adv Mater. 2010;22(4):468–472.
[12] Meng CL, Tseng MC, Tang ST, et al. Normally transparent smart window with haze enhancement via inhomogeneous alignment surface. Liq Cryst. 2019;46(3):484–491.
[13] Hu XW, Zeng WJ, Yang WM, et al. Effective electrically tunable infrared reflectors based on polymer stabilised cholesteric liquid crystals. Liq Cryst. 2019;46(2):185–192.
[14] Guo SM, Liang X, Zhang HM, et al. An electrically light-transmittance-controllable film with a low-driving voltage from a coexistent system of polymer-dispersed and polymer-stabilised cholesteric liquid crystals. Liq Cryst. 2018;45(12):1854–1860.
[15] Fuh AYG, Chih SY, Wu ST. Advanced electro-optical smart window based on PSLC using a photoconductive TiOPc electrode. Liq Cryst. 2018;45(6):864–871.
[16] Richter H, Buka A, Rehberg I. Electrohydrodynamic convection in a homeotropically aligned nematic sample. Phys Rev E. 1995;51(6):5886–5890.
[17] Carr EF. Influence of electric fields on the molecular alignment in the liquid crystal p-(anisalamino)-phenyl acetate. Mol Cryst. 1969;7(1):253–268.
[18] Kramer L, Pesch W. Electrohydrodynamic instabilities in nematic liquid crystals. In: Buka A, Kramer L, editors. Pattern formation in liquid crystals. Partially ordered systems. New York (NY): Springer; 1996;221–255.
[19] Zhan Y, Schenning APHJ, Broer DJ, et al. Light-driven electrohydrodynamic instabilities in liquid crystals. Adv Funct Mater. 2018;28(21):1707436.
[20] Reichardt C. Solvatochromism, thermochromism, piezochromism, halochromism, and chiro-solvatochromism of pyridinium N-phenoxide betaine dyes. Chem Soc Rev. 1992;21(3):147–153.
[21] Dvorjetski D, Silberberg Y, Wiener-Avnear E. Temperature dependence of the electrohydrodynamic instability in nematic liquid crystals. Mol Cryst Liq Cryst. 1977;42(4):273–281.
[22] Heilmeier GH, Zanoni LA. Guest-host interactions in nematic liquid crystals. A new electro-optic effect. Appl Phys Lett. 1968;13(3):91–92.

Check for updates

Light-Driven Electrohydrodynamic Instabilities in Liquid Crystals

Yuanyuan Zhan, Albertus P. H. J. Schenning, Dirk J. Broer, Guofu Zhou, and Danqing Liu**

The induction of electrohydrodynamic instabilities in nematic liquid crystals through light illumination are reported. For this purpose, a photochromic spiropyran is added to the liquid crystal mixture. When an electrical field is applied in the absence of UV light, the homeotropic liquid crystal reorients perpendicular to the electrical field driven by its negative dielectric anisotropy. Upon exposure to UV light, the nonionic spiropyran isomerizes to the zwitterionic merocyanine form inducing electrohydrodynamic instabilities which turns the cell from transparent into highly scattering. The reverse isomerization to closed-ring spiropyran form occurs thermally or under visible light, which stops the electrohydrodynamic instabilities and the cell becomes transparent again. It is demonstrated that the photoionic electrohydrodynamic instabilities can be used for light regulation. Local exposure, either to drive the electrohydrodynamics or to remove them enables the formation of colored images.

1. Introduction

Liquid crystals (LCs) are an interesting class of materials, especially for the large optical and dielectric anisotropy which forms the basis for a number of electro-optical effects.[1] Electrically tunable birefringence is used for most liquid crystal displays.[2] For example, patterned LC alignment results in switchable gratings and optical lenses.[3] Light scattering effects, in which transparent and scattering states are modulated through an electric field, are presently the basis for the development of a variety of applications ranging from reflective displays to smart windows.[4] Such devices are most often based on mixtures of polymer and liquid crystals.[5] For example, polymer dispersed liquid crystals (PDLCs) in which the scattering is based on the refractive index mismatch between a phase separated LC constituent and a polymer binder in the field-off state,[6] while in polymer stabilized liquid crystals (PSLCs), the scattering appears in the field-on state and originates from a combination of polydomain formation and a refractive index mismatch between the LC and a liquid crystal polymer additive.[7] The electro-optical performances in both PDLCs and PSLCs strongly rely on the delicate control over the polymerization process and the choice of polymer and liquid crystals is critical.[8,9] Polymer-free scattering devices are therefore appealing because of their simplicity of fabrication.[10]

In this work, we reevaluate an old principle based on electrohydrodynamic instabilities (EHDI) when an LC is subjected to an electric field. EHDI in LCs was discovered and reported already in the 1960s and was initially used to make scattering-based displays.[11,12] EHDI requires the coexistence of two events, the reorientation of aligned LC and the motion of charge carriers under an electric field. Generally, homeotropically aligned LCs with a negative dielectric anisotropy are used that realign to a planar orientation.[13] Charge carriers are often added to increase the conductivity and thereby reduce the switching voltage of the EHDI effect.[14] In the current work, we propose to generate the ionic species by light. Thereto, we use a photochromic dye to create charge carriers by light. Using light as a contactless and remote stimulus would enable us to fabricate dual responsive and patterned addressable colored scattering devices by local exposure.[15,16]

For our experiments, an LC mixture with a negative dielectric anisotropy ($\Delta\varepsilon = -8.3$) was selected. In a first reference experiment, hexadecyltrimethylammonium bromide (CTAB) was added as electrolyte to study EHDI of the LC mixture. The CTAB-doped LC mixture is placed in a cell constructed with two glass plates provided with transparent electrodes and homeotropic alignment layers. The LC is initially homeotropically aligned (**Figure 1**a,c) and shows a high light transmittance (Figure 1e). When applying an external alternating electric (AC) field of 20 V_{rms} across the cell, the initially homeotropically oriented liquid crystals tilt 90° toward a polydomain planar orientation. Meanwhile, the mobile charge carriers start oscillating under the influence of the AC field generating chaotic turbulence (Figure 1b,d,). Consequently, the LC strongly scatters light and completely shields the

Y. Zhan, Prof. A. P. H. J. Schenning, Prof. D. J. Broer, Prof. G. Zhou, Dr. D. Liu
SCNU-TUE Joint Lab of Device Integrated Responsive Materials (DIRM)
National Center for International Research on Green Optoelectronics
South China Normal University
Guangzhou 510006, P. R. China
E-mail: guofu.zhou@m.scnu.edu.cn; d.liu1@tue.nl

Y. Zhan, Prof. A. P. H. J. Schenning, Prof. D. J. Broer, Prof. G. Zhou, Dr. D. Liu
Laboratory of Functional Organic Materials and Devices (SFD)
Department of Chemical Engineering and Chemistry
Eindhoven University of Technology
Groene Loper 5, 5612 AE Eindhoven, The Netherlands

Prof. A. P. H. J. Schenning, Prof. D. J. Broer, Dr. D. Liu
Institute for Complex Molecular Systems (ICMS)
Eindhoven University of Technology
Groene Loper 5, 5612 AE Eindhoven, The Netherlands

Prof. G. Zhou
Shenzhen Guohua Optoelectronics Tech. Co. Ltd.
Shenzhen 518110, China

The ORCID identification number(s) for the author(s) of this article can be found under https://doi.org/10.1002/adfm.201707436.

DOI: 10.1002/adfm.201707436

© 2018 WILEY-VCH Verlag GmbH & Co. KGaA, Weinheim

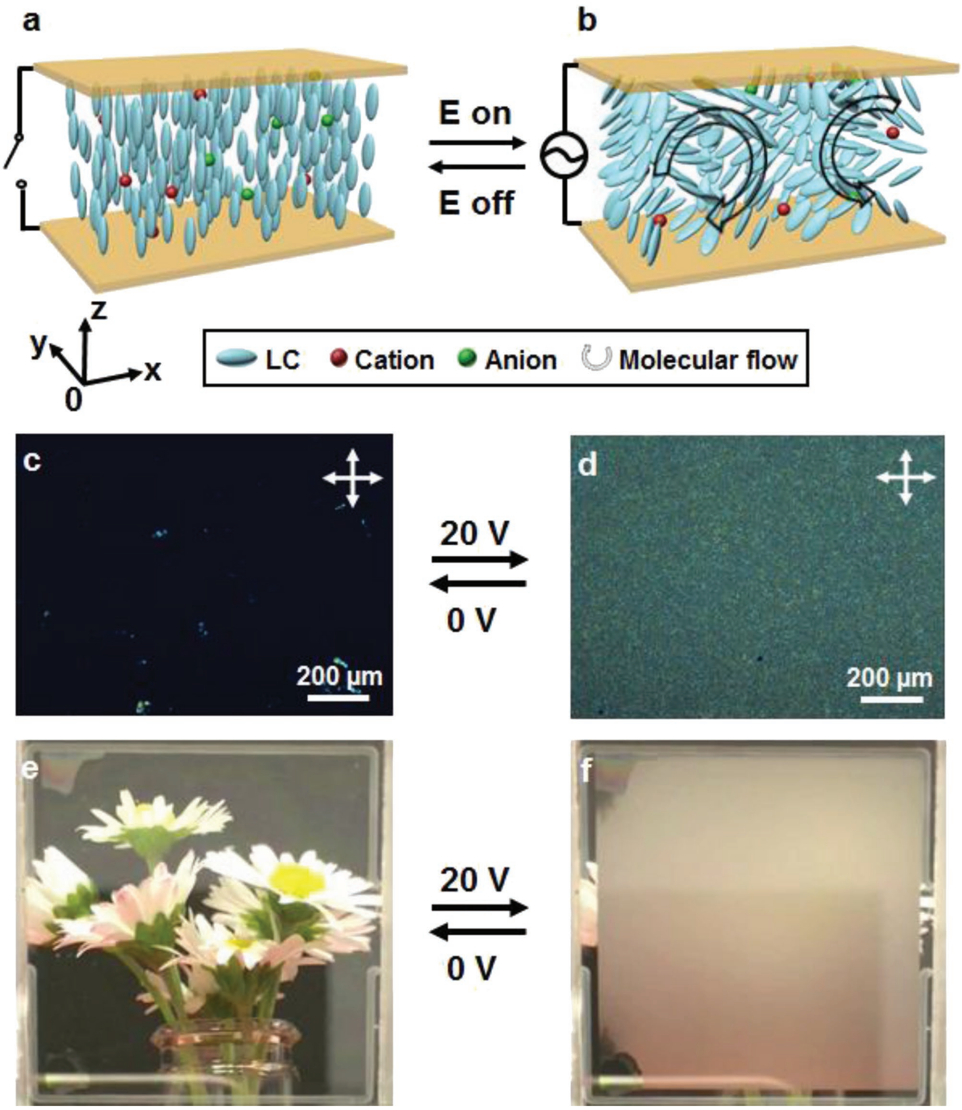

Figure 1. Electrohydrodynamic instability in a CTAB-doped LC mixture. Schematic representation of a) homeotropically aligned LC mixture in a cell at 0 V_{rms} and b) under an alternating electric field of 20 V_{rms} at 50 Hz showing EHDI. Cross-polarized optical microscopy image showing c) initial homeotropic orientated LC at 0 V_{rms} and d) turbulence patterns formed in the LC at 20 V_{rms} and 50 Hz. e) The transparent state at 0 V_{rms} showing the flowers placed behind the cell, and f) the light scattering state under 20 V_{rms} at 50 Hz which hides the flowers. The voltage described in the pictures is the root-mean-square voltage. The cell gap is 10 µm.

background (Figure 1f). The EHDI effect stops when the electric field is switched off and the initial homeotropic LC orientation and highly transparent state reverts immediately within 10 s.

In order to fabricate dual responsive colored scattering devices, we added the photochromic 1′,3′-dihydro-1′,3′,3′-trimethyl-6-nitrospiro[2H-1-benzopyran-2,2′-(2H)-indole], further denoted as the spiropyran, instead of CTAB to the LC mixture. The spiropyran aligns with the LC host (**Figure 2**a,d; Table S1 and Figure S2, Supporting Information).[17] Moreover, it can be switched reversibly by UV light from the nonionogenic ring-closed spiropyran form (SP) to the blue zwitterionic merocyanine open form (MC) (Figure S1, Supporting Information). Based on this, we can reversibly modulate the scattering and color of the LC device by light and electricity. As with the SP-doped sample, when applying an external AC field, SP-doped LC is reoriented perpendicularly to the electric field line resulting in a planar alignment (Figure 2b). Since the alignment layer does not give a preferred reorientation direction, a random polydomain texture is formed which is characterized by point disclinations connected by line disclinations. The director describes a complex rotation on its transition from one domain into the other which becomes visible when observed between cross polarizers in optical microscopy. Since the LC is not homogenously aligned in the mono-domain fashion, minor light scattering appears yet the sample still exhibits highly optical transparence (Figure 2h). When addressed with UV light (λ = 365 nm) at low intensity (18 mW cm^{-2}) for 5 s the nonionogenic closed-ring SP form converts to the ionic open-ring MC form (the MC conversion is

© 2018 WILEY-VCH Verlag GmbH & Co. KGaA, Weinheim

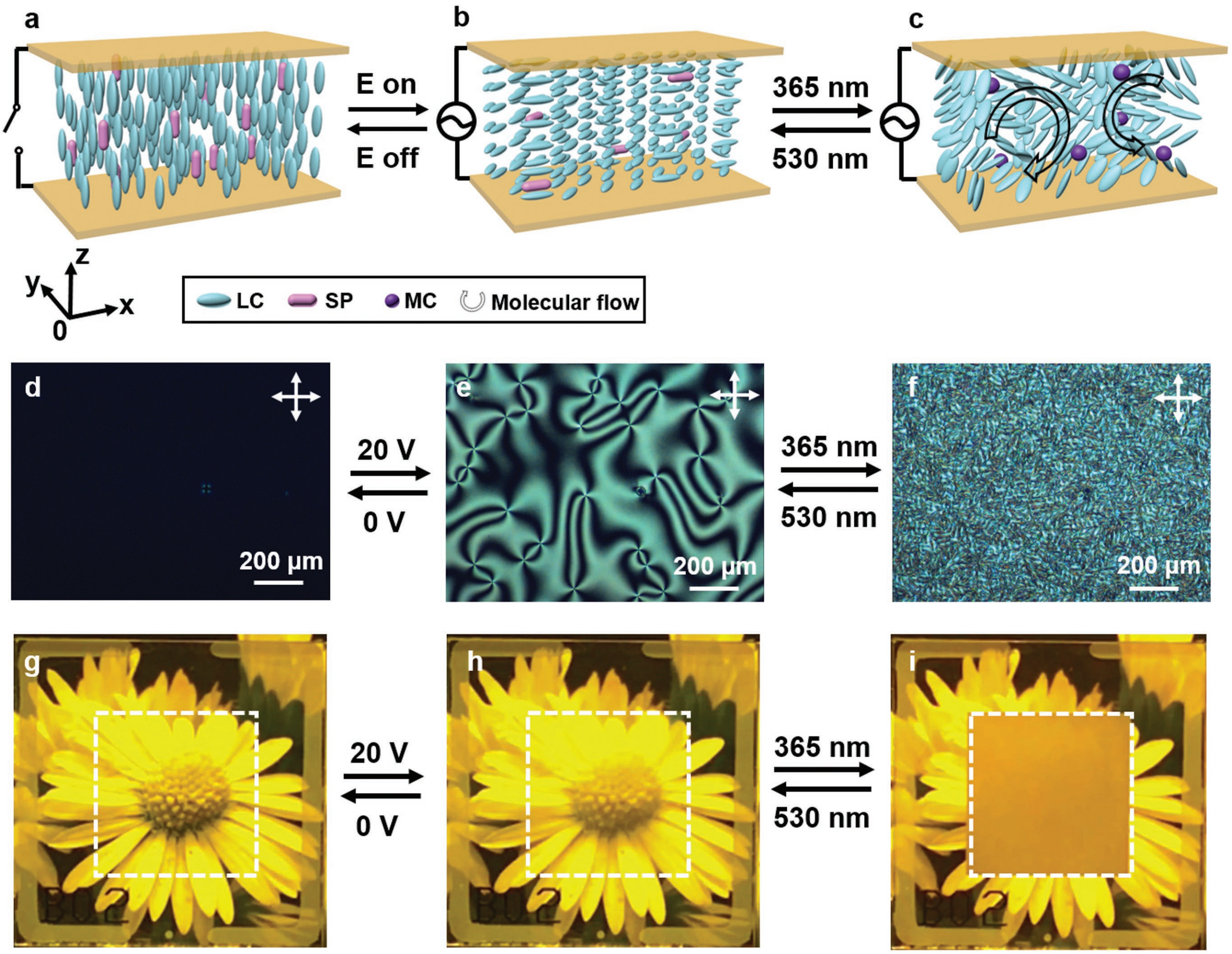

Figure 2. Light-induced EHDI. Schematic illustration of a) initial homeotropically aligned SP-doped LC at 0 V_{rms}. b) LC realigns to random planar polydomain under the AC field of 20 V_{rms} at 50 Hz. c) UV exposure converts SP to MC and initiates EHDI. d–f) The corresponding cross-polarized optical microscopic images of (a)–(c), respectively. Photos show that the sample is g) transparent without electric field, h) slightly scattering under the electric field, and i) highly opaque when illuminated with UV light. The samples are kept in the laboratory with yellow light to avoid MC to SP back reaction. The dotted square highlights the active area of the sample with the dimension of 1 cm × 1 cm. The voltage described in the pictures is root-mean-square voltage. The cell gap is 9 µm.

given in Figure S3, Supporting Information). Consequently, the formed MC oscillates with the electric field which in combination with the realigned LCs triggers the EHDI (Figure 2c,f). Hence, strong light scattering is observed and the scattering cell shields the background (Figure 2i). The light scattered state is also colored correlating to the absorption spectrum of the MC isomer (Figure S1, Supporting Information). The EHDI and the corresponding light scattering stay for 10 min after switching off the UV irradiation (Figure S4, Supporting Information). The transparent state can be formed again by exposing the sample to green light at $\lambda = 530$ nm which stimulates the back isomerization to the SP form. Further switching off the external electric field, the LCs matrix recovers to the original homeotropic alignment.

To analyze the light-induced EHDI principle in more detail, we investigate first the input voltage and the corresponding output current (**Figure 3**a, Figure S5, Supporting Information). Before switching on the EHDI by UV light, a low current of 2 µA is measured at 5 V_{rms} which is comparable with LC mixture without SP.[18] Upon UV exposure, the current increases significantly by a factor of 4 as the evidence of the formation of charge carriers upon the conversion of SP to the MC isomer. Next, we estimate the influence of electric field frequency and strength during UV light exposure.[19] Figure 3b shows the optimal frequency to trigger the EHDI is 50 Hz. Below this value, the MC oscillation increases with frequency and a larger turbulence is generated and retained. Above the optimal frequency, the MC oscillation begins to lag behind the oscillation field polarity. Consequently, EHDI effect decreases and eventually disappears. Next, the scattering is characterized by stepwise changing the AC voltage from 0 to 30 V_{rms} at 50 Hz (Figure 3c). The scattering was measured at a wavelength of 700 nm to avoid the absorption band of MC. Under ambient conditions without UV illumination, the sample exhibits a constant high optical transmission with increasing voltage. Only when voltage

 © 2018 WILEY-VCH Verlag GmbH & Co. KGaA, Weinheim

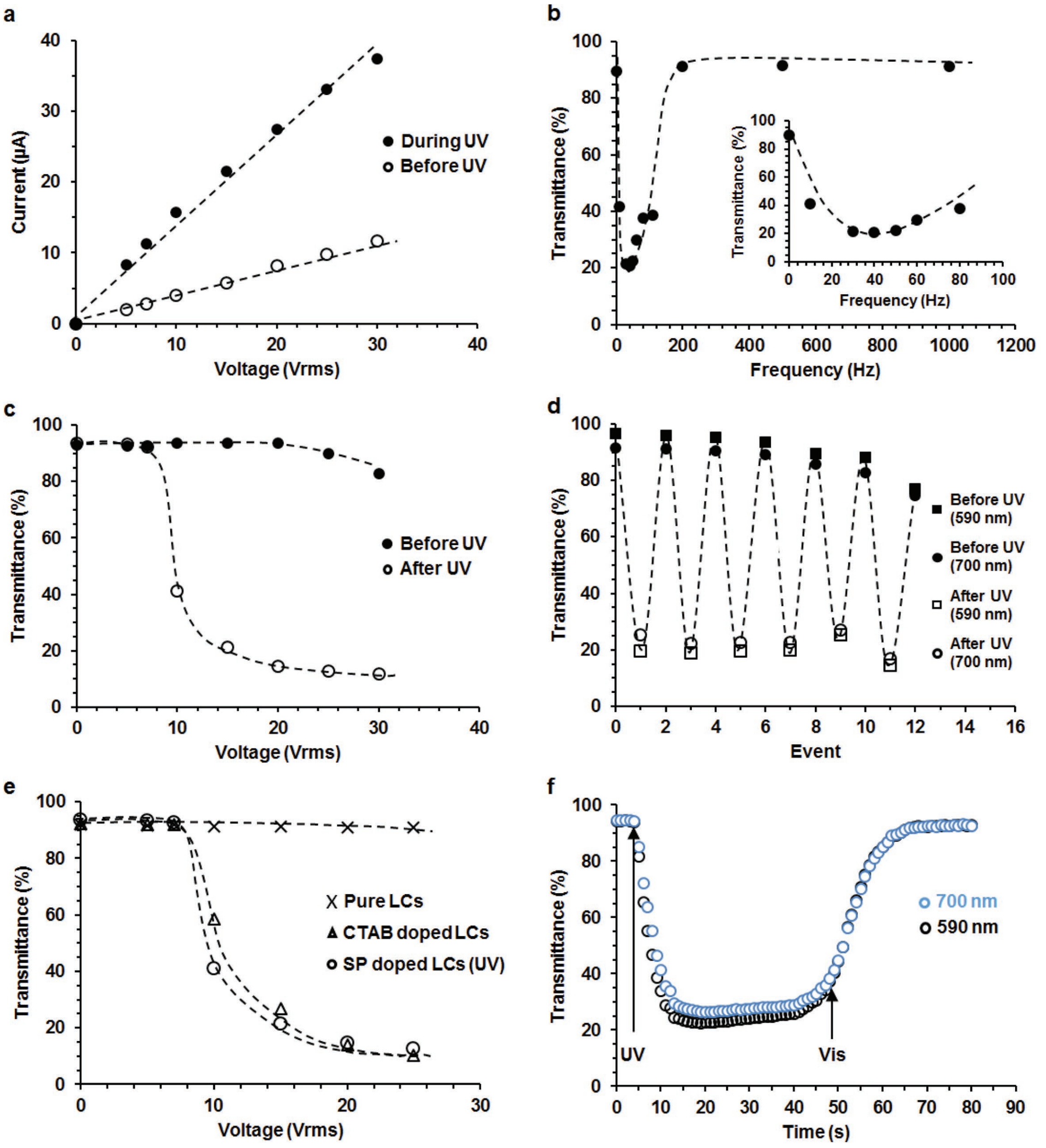

Figure 3. Characterization of light-induced EHDI. a) Current generated in the sample before and after EHDI is initiated. b) Influence of frequency on the light scattering. c) Voltage-dependent transmittance measured before and after UV light irradiation. d) Six switching cycles under the alternating UV and green light irradiation. The applied AC voltage is 20 V_{rms} at 50 Hz. The time interval between every two cycles is 2 min. e) Comparison of the transmittance at 700 nm between samples with pure LC, electrolyte CTAB and SP-doped LC. f) Response kinetics of spiropyran conversion and corresponding scattering under UV and green light exposure. The exposure conditions for all experiments are 18 mW cm^{-2} at 365 nm and 7 mW cm^{-2} at 530 nm. The cell gap is 9 µm.

exceeds 30 V_{rms}, the transmittance of the sample deceases slightly. Upon UV illumination, the transmittance drops dramatically to 16% at 20 V_{rms} as the result of the EHDI effect. Note that in the experiments, the ultraviolet–visible spectra are taken directly after UV illumination.

By alternating the UV and green light exposure, the cell can be switched between the scattering and transparent state (Figure 3d). When measuring the transmittance at 590 nm which is the absorption maximum of MC it can be seen that the transmittance in the scattering state is low while it is high in the transparent state (Figure S6, Supporting Information). This clearly shows that the scattering is caused by the formation of MC while in the transparent state the SP form is present. We further notice that the sample does not completely recover to its initial transmittance after several switching cycles which might be ascribed to a small fraction of MC still present after green light exposure.[20] It should be noted that the sample can fully relax back to the initial state at an elevated temperature. When comparing the EHDI effect between samples with pure LC, electrolyte CTAB-doped LC and SP-containing LC upon

© 2018 WILEY-VCH Verlag GmbH & Co. KGaA, Weinheim

applying an AC field, the pure LC sample as expected does not exhibit EHDI effect (Figure 3e). The light exposed SP–LC and CTAB–LC devices show comparable transmittance change suggesting similar EHDI behavior. The transmittance can also be adjusted by the AC field strength, as seen in Figure 3c,e. Above the threshold voltage, the LC breaks into small domains and moderate light scattering is induced. Further increasing the voltage generates the chaotic turbulence in LCs and results in the maximum light scattering.

In order to quantify the response kinetics, we measured the time-resolved transmittance of SP in the LC and the light-induced scattering by taking ultraviolet–visible spectra at 590 and 700 nm, respectively. Results are given in Figure 3f. It is obvious that the LC light scattering occurs simultaneously with the SP to MC conversion without any phase lag. The back relaxation from MC to SP and the ceasing of light scattering are also synchronized and take 10 min in the dark (Figure S4, Supporting Information). The back reaction can be accelerated to 20 s by exposing the sample to green light. For reference, we performed an identical experiment with SP dissolved in tetrahydrofuran as solvent (Figure S7, Supporting Information). Results indicate that LCs matrix does not influence the isomerization of the photochemical reaction of spiropyran.

In order to demonstrate a light-rewritable patterned scattering device, we performed a mask-wise UV exposure as illustrated in **Figure 4**a–d (Movies S1–S3, Supporting Information). The projected flower pattern gives a conversion from transparent to blue and opaque, which is caused by the absorption of MC isomer and the EHDI-induced light scattering, respectively. The scattering pattern can be erased when addressed with a high frequency voltage, for example, 1 kHz (Figure 3b) or by switching off the electric field. Both methods remove the EHDI-induced light scattering while keeping the light-induced MC pattern (Figure 4e). The entire pattern can be erased by green light converting the colored MC isomer to the SP colorless form. The printed pattern can also be erased locally through focused green light (Figure 4f). Currently, most e-writer tablets provide a global erasing possibility. Based on our technology, we

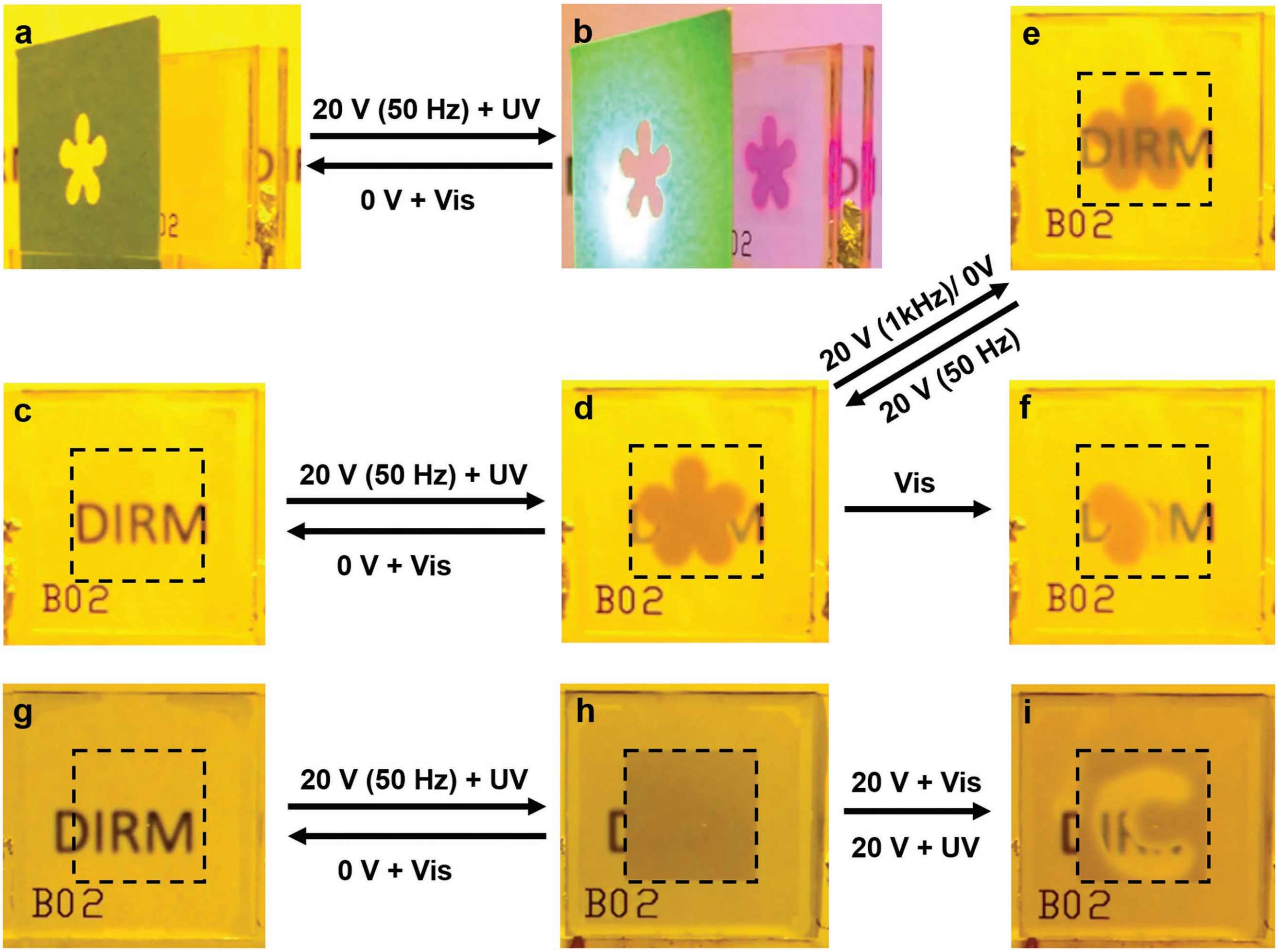

Figure 4. Photos show that EHDI can be locally generated and erased. a,b) The blue opaque flower is generated. c,d) The corresponding front view of (a) and (b), respectively. e) The blossom is partially erased by focused green light. f) The blue opaque flower turns into transparent when addressed with high-frequency voltage or switching off the AC field. g–i) Direct writing on the EHDI background through focused green light. All the samples are kept in the laboratory with yellow light to prevent MC to SP back reaction. The electric active area is highlighted by the dotted square. The voltage described in the pictures is root-mean-square voltage. The cell provides homeotropic alignment and the cell gap is 9 µm.

© 2018 WILEY-VCH Verlag GmbH & Co. KGaA, Weinheim

propose a complementary solution to partially erase the display while keep the desired information on the display as shown in Figure 4f. Besides displaying preprogrammed information, for example, by patterned electrodes or by masked exposure, direct writing is enabled by using a focused light source (Figure 4g–i). In this experiment, the colored EHDI scattering provides the background while the focused green light partially erases EHDI and leaves information on scattering sample.

In conclusion, we have presented a new approach in initiating electrohydrodynamic instabilities in liquid crystals under light illumination. EHDI can be triggered or removed by alternating UV and green light exposure within tens of seconds. We especially explore the light scattering effect originated from EHDI in which the sample can be modulated from nearly 100% transparent state to 16% transmittance. The light scattering from EHDI exhibits a strong shielding effect which is proposed to be useful in smart window applications. Using light to trigger EHDI provides possibilities to localize light scattering by mask-wise exposure or by local writing with a focused beam. We anticipate the use of the effect for an information display or a smart window in which the messages can be written, stored, and erased, either locally or globally. In addition, this technology possesses large potential applications, such as the dye-doped colored windows, segmented triggered window, and e-paper.

2. Experimental Section

Materials: 1′,3′-Dihydro-1′,3′,3′-trimethyl-6-nitrospiro[2H-1-benzopyran-2,2′-(2H)-indole] (spiropyran) was obtained from Sigma-Aldrich. CTAB was purchased from Sigma-Aldrich. LCs with negative dielectric anisotropy were obtained from Jiangsu Hecheng Advanced Materials Co., Ltd. (HNG30400-200, T_{N-I} = 94 °C, $\Delta\varepsilon = -8.3$, $\Delta n = 0.149$). The conventional EHDI mixture contains 0.05 wt% CTAB and 99.95 wt% LCs. The light-induced EHDI mixture contains 0.3 wt% of spiropyran and 99.7 wt% LCs.

Sample Preparation: Commercial cells (S100A090uT80, Instec; and XGH3030-10, Guohua Star) were used for experiments. The cells were filled with LC mixture at by capillary suction at 120 °C. The samples were cooled slowly to 80 °C and held for 2 h to eliminate any thermal instability before further cooled down to room temperature.

Sample Characterization: Samples were checked by optical microscope equipped with crossed polarizers (Nikon Ci Eclipse). Transmittance of the sample was measured by UV–vis–NIR spectrometer (PerkinElmer 750 and Ocean Optics HR2000+). The alternating electric field with a sinusoidal wave function was provided by a function generator (33220A, Agilent). The electric signal from the function generator was amplified through a high-voltage linear amplifier (F20A, FLC Electronics). The output voltage was measured by an oscilloscope (DSOX3032T, Keysight). A LED lamp (M365L2 and M530L3-C2, Thorlabs) was used to provide monochromatic light.

Supporting Information

Supporting Information is available from the Wiley Online Library or from the author.

Acknowledgements

The results presented are part of research programs financed by the National Natural Science Foundation of China (51561135014, U1501244), Guangdong Innovative Research Team Program (No. 2013C102), European Research Commission under ERC Advanced Grant 66999 (VIBRATE), the framework of the 4TU.High-Tech Materials research program "New Horizons in designer materials" (www.4tu.nl/htm), and NWO VENI grant 15135, and additional information is available in the Supporting Information and from the authors.

Conflict of Interest

The authors declare no conflict of interest.

Keywords

electrohydrodynamic instabilities, light scattering, liquid crystals, photochemistry

Received: December 22, 2017
Revised: February 26, 2018
Published online: April 6, 2018

[1] P. G. deGennes, J. Prost, *The Physics of Liquid Crystals*, 2nd ed., Clarendon, Oxford, UK **1993**.
[2] a) W. D. S. John, W. J. Fritz, Z. J. Lu, D. K. Yang, *Phys. Rev. E* **1995**, *51*, 1191; b) D. J. Broer, J. Lub, G. N. Mol, *Nature* **1995**, *378*, 467; c) J. Xiang, Y. N. Li, Q. Li, D. A. Paterson, J. M. D. Storey, C. T. Imrie, O. D. Lavrentovich, *Adv. Mater.* **2015**, *27*, 3014; d) W. Hu, H. Y. Zhao, L. Song, Z. Yang, H. Cao, Z. H. Cheng, Q. Liu, H. Yang, *Adv. Mater.* **2010**, *22*, 468.
[3] a) S. N. Lee, L. C. Chien, S. Sprunt, *Appl. Phys. Lett.* **1998**, *72*, 885; b) D. Q. Liu, D. J. Broer, *Langmuir* **2014**, *30*, 13499; c) P. J. Ackerman, J. van de Lagemaat, I. I. Smalyukh, *Nat. Commun.* **2015**, *6*, 6012; d) C. P. Chiu, T. J. Chiang, J. K. Chen, F. C. Chang, F. H. Ko, C. W. Chu, S. W. Kuo, S. K. Fan, *J. Adhes. Sci. Technol.* **2012**, *26*, 1773.
[4] a) J. Fergason, *SID Int. Symp. Dig. Tech. Pap.* **1985**, *16*, 68; b) R. A. M. Hikmet, *Adv. Mater.* **1992**, *4*, 679; c) R. A. M. Hikmet, H. Kemperman, *Nature* **1998**, *392*, 476; d) F. Liu, J. J. Wang, Z. H. Ge, K. X. Li, H. J. Ding, B. P. Zhang, D. Wang, H. Yang, *J. Mater. Chem. C* **2013**, *1*, 216; e) F. Vicentini, J. L. Cho, L. C. Chien, *Liq. Cryst.* **1998**, *24*, 483; f) K. G. Gutierrez-Cuevas, L. Wang, Z. G. Zheng, H. K. Bisoyi, G. Q. Li, L. S. Tan, R. A. Vaia, Q. Li, *Angew. Chem., Int. Ed.* **2016**, *55*, 13090.
[5] D. K. Yang, L.-C. Chien, *Liquid Crystals in Complex Geometries*, Taylor and Francis, London, UK **1996**.
[6] a) R. A. M. Hikmet, *J. Appl. Phys.* **1990**, *68*, 4406; b) R. A. M. Hikmet, *Liq. Cryst.* **1991**, *9*, 405; c) J. W. Doane, N. A. Vaz, B.-G. Wu, S. Zumer, *Appl. Phys. Lett.* **1986**, *48*, 269.
[7] a) R. A. M. Hikmet, *Liq. Cryst.* **2006**, *33*, 1410; b) I. Dierking, *Adv. Mater.* **2000**, *12*, 167; c) H. Khandelwal, A. P. H. J. Schenning, M. G. Debije, *Adv. Energy Mater.* **2017**, *7*1602209.
[8] Y. K. Fung, D.-K. Yang, Y. Sun, L. C. Chien, S. Zumer, J. W. Doane, *Liq. Cryst.* **1995**, *19*, 797.
[9] a) R. Williams, *J. Chem. Phys.* **1963**, *39*, 384; b) R. Williams, *Nature* **1963**, *199*, 273; c) G. H. Heilmeier, L. A. Zanoni, L. A. Barton, *Appl. Phys. Lett.* **1968**, *13*, 46; d) G. H. Heilmeier, L. A. Zanoni, L. A. Barton, *Proc. IEEE* **1968**, *56*, 1162; e) L. M. Blinov, V. G. Chigrinov, *Electrooptic Effects in Liquid Crystal Materials*, Springer, New York **1996**.
[10] a) J. Sun, R. C. Lan, Y. Z. Gao, M. Wang, W. S. Zhang, L. Wang, L. Y. Zhang, Z. Yang, H. Yang, *Adv. Sci.* **2018**, *5*, 1700613; b) J. Geng, C. Dong, L. P. Zhang, Z. Ma, L. Shi, H. Cao, H. Yang,

 © 2018 WILEY-VCH Verlag GmbH & Co. KGaA, Weinheim

Appl. Phys. Lett. **2006**, *89*, 081130; c) A. Jákli, L.-C. Chien, D. Krüerke, H. Sawade, G. Heppke, *Liq. Cryst.* **2002**, *29*, 377.
[11] a) P. Song, Y. Z. Gao, F. F. Wang, L. Y. Zhang, H. Xie, Z. Yang, H. Yang, *Liq. Cryst.* **2015**, *42*, 390; b) P. Song, H. Cao, F. F. Wang, F. Liu, H. Yang, *Liq. Cryst.* **2012**, *39*, 433; c) Y. Z. Gao, W. H. Yao, J. Sun, H. M. Zhang, Z. D. Wang, L. Wang, D. K. Yang, L. Y. Zhang, H. Yang, *J. Mater. Chem. A* **2015**, *3*, 10738.
[12] a) M. I. Barnik, L. M. Blinov, M. F. Grebenkin, S. A. Pikin, V. G. Chigrinov, *Phys. Lett. A* **1975**, *51*, 175; b) E. Kochowska, S. Németh, G. Pelzl, Á. Buka, *Phys. Rev. E* **2004**, *70*, 011711; c) B. R. Zhang, H. Kitzerow, *J. Phys. Chem. B* **2016**, *120*, 6865; d) S. V. Serak, U. Hrozhyk, J. Hwang, N. V. Tabiryan, D. Steeves, B. R. Kimball, *Appl. Opt.* **2016**, *55*, 8506.
[13] a) S. Kai, K. Hayashi, Y. Hidaka, *J. Phys. Chem.* **1996**, *100*, 19007; b) H. Richter, A. Buka, I. Rehberg, *Phys. Rev. E* **1995**, *51*, 5886; c) Y. Hidaka, J.-H. Huh, K. Hayashi, S. Kai, M. Tribelsky, *Phys. Rev. E* **1997**, *56*, 6256.
[14] E. F. Carr, *Mol. Cryst.* **1969**, *7*, 253.
[15] a) A. Martinez, I. I. Smalyukh, *Opt. Express* **2015**, *23*, 4591; b) M. E. McConney, A. Martinez, V. P. Tondiglia, K. M. Lee, D. Langley, I. I. Smalyukh, T. J. White, *Adv. Mater.* **2013**, *25*, 5880; c) H. Zeng, O. M. Wani, P. Wasylczyk, R. Kaczmarek, A. Priimagi, *Adv. Mater.* **2017**, *29*, 1701814; d) A. H. Gelebart, D. J. Mulder, M. Varga, A. Konya, G. Vantomme, E. W. Meijer, R. L. B. Selinger, D. J. Broer, *Nature* **2017**, *546*, 632.
[16] H. K. Bisoyi, Q. Li, *Chem. Rev.* **2016**, *116*, 15089.
[17] a) R. Klajn, *Chem. Soc. Rev.* **2014**, *43*, 148; b) J. ter Schiphorst, M. van den Broek, T. de Koning, J. N. Murphy, A. P. H. J. Schenning, A. C. C. Esteves, *J. Mater. Chem. A* **2016**, *4*, 8676; c) W. Li, X. R. Wu, H. Qin, Z. Q. Zhao, H. W. Liu, *Adv. Funct. Mater.* **2016**, *26*, 3164; d) J. E. Stumpel, B. Ziolkowski, L. Florea, D. Diamond, D. J. Broer, A. P. H. J. Schenning, *ACS Appl. Mater. Interfaces* **2014**, *6*, 7268; e) M. H. Zhang, X. Hou, J. T. Wang, Y. Tian, X. Fan, J. Zhai, L. Jiang, *Adv. Mater.* **2012**, *24*, 2424.
[18] C. D. Sheraw, L. Zhou, J. R. Huang, D. J. Gundlach, T. N. Jackson, M. G. Kane, I. G. Hill, M. S. Hammond, J. Campi, B. K. Greening, J. Francl, J. West, *Appl. Phys. Lett.* **2002**, *80*, 1088.
[19] M. Urbanski, J. P. F. Lagerwall, *J. Mater. Chem. C* **2016**, *4*, 3485.
[20] T. Seki, K. Ichimura, *J. Phys. Chem.* **1990**, *94*, 3769.

© 2018 WILEY-VCH Verlag GmbH & Co. KGaA, Weinheim
