# Supplementary material for: Advanced liquid crystal-based switchable optical devices for light protection applications: principles and strategies
Source: Light Sci Appl. 2023 Jan 3;12:11. doi: 10.1038/s41377-022-01032-y (PMC9807646; doi:10.1038/s41377-022-01032-y)
Supplement: Supplementary file 13 — Fig 15 copyright promotion [file 41377_2022_1032_MOESM13_ESM.pdf]

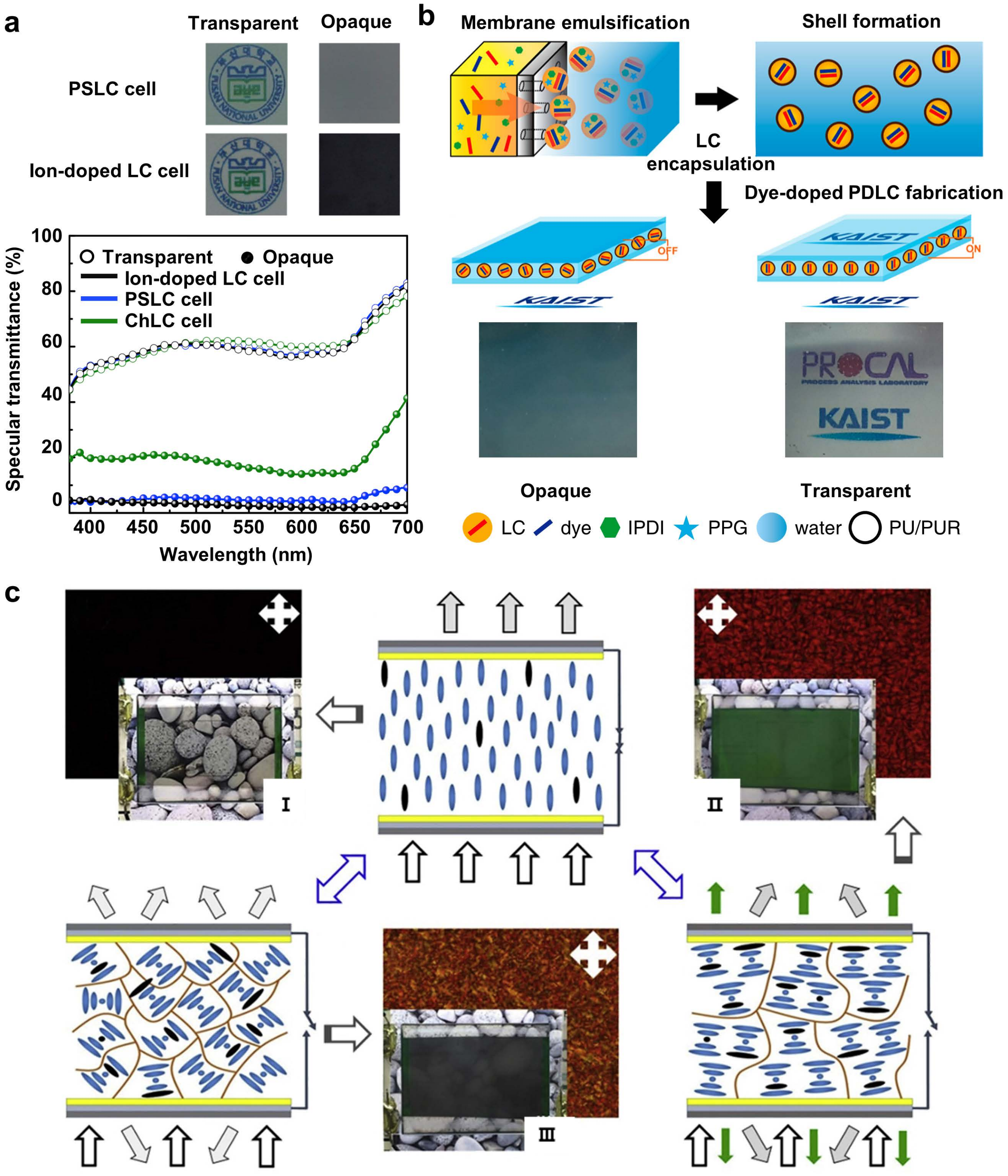
a
Transparent
Opaque
PSLC cell
Ion-doped LC cell
Transparent
Opaque
Ion-doped LC cell
PSLC cell
ChLC cell
Specular transmittance (%)
Wavelength (nm)
b
Membrane emulsification
Shell formation
LC encapsulation
Dye-doped PDLC fabrication
OFF
ON
KAIST
PROCAL
PROCESS ANALYSIS LABORATORY
Opaque
Transparent
LC
dye
IPDI
PPG
water
PU/PUR
c
I
II
III

# ELSEVIER LICENSE TERMS AND CONDITIONS

Sep 19, 2022

This Agreement between Harbin Institute of Technology -- Ruicong Zhang ("You") and Elsevier ("Elsevier") consists of your license details and the terms and conditions provided by Elsevier and Copyright Clearance Center.

| | |
|---|---|
| License Number | 5392410844221 |
| License date | Sep 19, 2022 |
| Licensed Content Publisher | Elsevier |
| Licensed Content Publication | Dyes and Pigments |
| Licensed Content Title | Ion-doped liquid-crystal cell with low opaque-state specular transmittance based on electro-hydrodynamic effect |
| Licensed Content Author | Jae-Won Huh,Jin-Hun Kim,Seung-Won Oh,Seong-Min Ji,Tae-Hoon Yoon |
| Licensed Content Date | Mar 1, 2018 |
| Licensed Content Volume | 150 |
| Licensed Content Issue | n/a |
| Licensed Content Pages | 5 |
| Start Page | 16 |
| End Page | 20 |
| Type of Use | reuse in a journal/magazine |
| Requestor type | academic/educational institute |
| Portion | figures/tables/illustrations |
| Number of figures/tables/illustrations | 3 |
| Format | both print and electronic |
| Are you the author of this Elsevier article? | No |
| Will you be translating? | No |
| Title of new article | Advanced liquid crystal-based switchable optical devices for light protection applications: principles and strategies |
| Lead author | Ruicong Zhang, Zhibo Zhang, Jiecai Han, Lei Yang, Jiajun li, Zicheng Song Tianyu Wang, Jiaqi Zhu |
| Title of targeted journal | Light: Science & Applications |
| Publisher | Springer Nature |
| Expected publication date | Nov 2022 |
| Portions | Figure 5,Figure 6,Figure 7 |
| Requestor Location | Harbin Institute of Technology<br>No. 92, Xidazhi Street, Nangang District<br><br>Harbin, 150080<br>China<br>Attn: Harbin Institute of Technology |
| Publisher Tax ID | GB 494 6272 12 |
| Total | **0.00 USD** |

Terms and Conditions

**INTRODUCTION**

1. The publisher for this copyrighted material is Elsevier. By clicking "accept" in connection with completing this licensing transaction, you agree that the following terms and conditions apply to this transaction (along with the Billing and Payment terms

and conditions established by Copyright Clearance Center, Inc. ("CCC"), at the time that you opened your Rightslink account and that are available at any time at http://myaccount.copyright.com).

**GENERAL TERMS**

2. Elsevier hereby grants you permission to reproduce the aforementioned material subject to the terms and conditions indicated.
3. Acknowledgement: If any part of the material to be used (for example, figures) has appeared in our publication with credit or acknowledgement to another source, permission must also be sought from that source. If such permission is not obtained then that material may not be included in your publication/copies. Suitable acknowledgement to the source must be made, either as a footnote or in a reference list at the end of your publication, as follows:
"Reprinted from Publication title, Vol /edition number, Author(s), Title of article / title of chapter, Pages No., Copyright (Year), with permission from Elsevier [OR APPLICABLE SOCIETY COPYRIGHT OWNER]." Also Lancet special credit - "Reprinted from The Lancet, Vol. number, Author(s), Title of article, Pages No., Copyright (Year), with permission from Elsevier."
4. Reproduction of this material is confined to the purpose and/or media for which permission is hereby given.
5. Altering/Modifying Material: Not Permitted. However figures and illustrations may be altered/adapted minimally to serve your work. Any other abbreviations, additions, deletions and/or any other alterations shall be made only with prior written authorization of Elsevier Ltd. (Please contact Elsevier's permissions helpdesk here). No modifications can be made to any Lancet figures/tables and they must be reproduced in full.
6. If the permission fee for the requested use of our material is waived in this instance, please be advised that your future requests for Elsevier materials may attract a fee.
7. Reservation of Rights: Publisher reserves all rights not specifically granted in the combination of (i) the license details provided by you and accepted in the course of this licensing transaction, (ii) these terms and conditions and (iii) CCC's Billing and Payment terms and conditions.
8. License Contingent Upon Payment: While you may exercise the rights licensed immediately upon issuance of the license at the end of the licensing process for the transaction, provided that you have disclosed complete and accurate details of your proposed use, no license is finally effective unless and until full payment is received from you (either by publisher or by CCC) as provided in CCC's Billing and Payment terms and conditions. If full payment is not received on a timely basis, then any license preliminarily granted shall be deemed automatically revoked and shall be void as if never granted. Further, in the event that you breach any of these terms and conditions or any of CCC's Billing and Payment terms and conditions, the license is automatically revoked and shall be void as if never granted. Use of materials as described in a revoked license, as well as any use of the materials beyond the scope of an unrevoked license, may constitute copyright infringement and publisher reserves the right to take any and all action to protect its copyright in the materials.
9. Warranties: Publisher makes no representations or warranties with respect to the licensed material.
10. Indemnity: You hereby indemnify and agree to hold harmless publisher and CCC, and their respective officers, directors, employees and agents, from and against any and all claims arising out of your use of the licensed material other than as specifically authorized pursuant to this license.
11. No Transfer of License: This license is personal to you and may not be sublicensed, assigned, or transferred by you to any other person without publisher's written permission.
12. No Amendment Except in Writing: This license may not be amended except in a writing signed by both parties (or, in the case of publisher, by CCC on publisher's behalf).
13. Objection to Contrary Terms: Publisher hereby objects to any terms contained in any purchase order, acknowledgment, check endorsement or other writing prepared by you, which terms are inconsistent with these terms and conditions or CCC's Billing and Payment terms and conditions. These terms and conditions, together with CCC's Billing and Payment terms and conditions (which are incorporated herein), comprise the entire agreement between you and publisher (and CCC) concerning this licensing transaction. In the event of any conflict between your obligations established by these terms and conditions and those established by CCC's Billing and Payment terms and conditions, these terms and conditions shall control.
14. Revocation: Elsevier or Copyright Clearance Center may deny the permissions described in this License at their sole discretion, for any reason or no reason, with a full refund payable to you. Notice of such denial will be made using the contact information provided by you. Failure to receive such notice will not alter or invalidate the denial. In no event will Elsevier or Copyright Clearance Center be responsible or liable for any costs, expenses or damage incurred by you as a result of a denial of your permission request, other than a refund of the amount(s) paid by you to Elsevier and/or Copyright Clearance Center for denied permissions.

**LIMITED LICENSE**

The following terms and conditions apply only to specific license types:
15. **Translation**: This permission is granted for non-exclusive world **English** rights only unless your license was granted for translation rights. If you licensed translation rights you may only translate this content into the languages you requested. A professional translator must perform all translations and reproduce the content word for word preserving the integrity of the article.
16. **Posting licensed content on any Website**: The following terms and conditions apply as follows: Licensing material from an Elsevier journal: All content posted to the web site must maintain the copyright information line on the bottom of each image; A hyper-text must be included to the Homepage of the journal from which you are licensing at http://www.sciencedirect.com/science/journal/xxxxx or the Elsevier homepage for books at http://www.elsevier.com; Central Storage: This license does not include permission for a scanned version of the material to be stored in a central repository such as that provided by Heron/XanEdu.
Licensing material from an Elsevier book: A hyper-text link must be included to the Elsevier homepage at http://www.elsevier.com . All content posted to the web site must maintain the copyright information line on the bottom of each image.

**Posting licensed content on Electronic reserve**: In addition to the above the following clauses are applicable: The web site must be password-protected and made available only to bona fide students registered on a relevant course. This permission is granted for 1 year only. You may obtain a new license for future website posting.
17. **For journal authors:** the following clauses are applicable in addition to the above:
**Preprints:**
A preprint is an author's own write-up of research results and analysis, it has not been peer-reviewed, nor has it had any other value added to it by a publisher (such as formatting, copyright, technical enhancement etc.).
Authors can share their preprints anywhere at any time. Preprints should not be added to or enhanced in any way in order to appear more like, or to substitute for, the final versions of articles however authors can update their preprints on arXiv or RePEc with their Accepted Author Manuscript (see below).
If accepted for publication, we encourage authors to link from the preprint to their formal publication via its DOI. Millions of researchers have access to the formal publications on ScienceDirect, and so links will help users to find, access, cite and use the best available version. Please note that Cell Press, The Lancet and some society-owned have different preprint policies. Information on these policies is available on the journal homepage.
**Accepted Author Manuscripts:** An accepted author manuscript is the manuscript of an article that has been accepted for publication and which typically includes author-incorporated changes suggested during submission, peer review and editor-author communications.
Authors can share their accepted author manuscript:

- immediately
  - via their non-commercial person homepage or blog
  - by updating a preprint in arXiv or RePEc with the accepted manuscript
  - via their research institute or institutional repository for internal institutional uses or as part of an invitation-only research collaboration work-group
  - directly by providing copies to their students or to research collaborators for their personal use
  - for private scholarly sharing as part of an invitation-only work group on commercial sites with which Elsevier has an agreement
- After the embargo period
  - via non-commercial hosting platforms such as their institutional repository
  - via commercial sites with which Elsevier has an agreement

In all cases accepted manuscripts should:

- link to the formal publication via its DOI
- bear a CC-BY-NC-ND license - this is easy to do
- if aggregated with other manuscripts, for example in a repository or other site, be shared in alignment with our hosting policy not be added to or enhanced in any way to appear more like, or to substitute for, the published journal article.

**Published journal article (JPA):** A published journal article (PJA) is the definitive final record of published research that appears or will appear in the journal and embodies all value-adding publishing activities including peer review co-ordination, copy-editing, formatting, (if relevant) pagination and online enrichment.
Policies for sharing publishing journal articles differ for subscription and gold open access articles:
**Subscription Articles:** If you are an author, please share a link to your article rather than the full-text. Millions of researchers have access to the formal publications on ScienceDirect, and so links will help your users to find, access, cite, and use the best available version.
Theses and dissertations which contain embedded PJAs as part of the formal submission can be posted publicly by the awarding institution with DOI links back to the formal publications on ScienceDirect.
If you are affiliated with a library that subscribes to ScienceDirect you have additional private sharing rights for others' research accessed under that agreement. This includes use for classroom teaching and internal training at the institution (including use in course packs and courseware programs), and inclusion of the article for grant funding purposes.
**Gold Open Access Articles:** May be shared according to the author-selected end-user license and should contain a CrossMark logo, the end user license, and a DOI link to the formal publication on ScienceDirect.
Please refer to Elsevier's posting policy for further information.
18. **For book authors** the following clauses are applicable in addition to the above: Authors are permitted to place a brief summary of their work online only. You are not allowed to download and post the published electronic version of your chapter, nor may you scan the printed edition to create an electronic version. **Posting to a repository:** Authors are permitted to post a summary of their chapter only in their institution's repository.
19. **Thesis/Dissertation**: If your license is for use in a thesis/dissertation your thesis may be submitted to your institution in either print or electronic form. Should your thesis be published commercially, please reapply for permission. These requirements include permission for the Library and Archives of Canada to supply single copies, on demand, of the complete thesis and include permission for Proquest/UMI to supply single copies, on demand, of the complete thesis. Should your thesis be published commercially, please reapply for permission. Theses and dissertations which contain embedded PJAs as part of the formal submission can be posted publicly by the awarding institution with DOI links back to the formal publications on ScienceDirect.

**Elsevier Open Access Terms and Conditions**
You can publish open access with Elsevier in hundreds of open access journals or in nearly 2000 established subscription journals that support open access publishing. Permitted third party re-use of these open access articles is defined by the author's choice of Creative Commons user license. See our open access license policy for more information.
**Terms & Conditions applicable to all Open Access articles published with Elsevier:**
Any reuse of the article must not represent the author as endorsing the adaptation of the article nor should the article be modified in such a way as to damage the author's honour or reputation. If any changes have been made, such changes must be clearly indicated.
The author(s) must be appropriately credited and we ask that you include the end user license and a DOI link to the formal publication on ScienceDirect.
If any part of the material to be used (for example, figures) has appeared in our publication with credit or acknowledgement to another source it is the responsibility of the user to ensure their reuse complies with the terms and conditions determined by the rights holder.
**Additional Terms & Conditions applicable to each Creative Commons user license:**
**CC BY:** The CC-BY license allows users to copy, to create extracts, abstracts and new works from the Article, to alter and revise the Article and to make commercial use of the Article (including reuse and/or resale of the Article by commercial entities), provided the user gives appropriate credit (with a link to the formal publication through the relevant DOI), provides a link to the license, indicates if changes were made and the licensor is not represented as endorsing the use made of the work. The full details of the license are available at http://creativecommons.org/licenses/by/4.0.
**CC BY NC SA:** The CC BY-NC-SA license allows users to copy, to create extracts, abstracts and new works from the Article, to alter and revise the Article, provided this is not done for commercial purposes, and that the user gives appropriate credit (with a link to the formal publication through the relevant DOI), provides a link to the license, indicates if changes were made and the licensor is not represented as endorsing the use made of the work. Further, any new works must be made available on the same conditions. The full details of the license are available at http://creativecommons.org/licenses/by-nc-sa/4.0.
**CC BY NC ND:** The CC BY-NC-ND license allows users to copy and distribute the Article, provided this is not done for commercial purposes and further does not permit distribution of the Article if it is changed or edited in any way, and provided the user gives appropriate credit (with a link to the formal publication through the relevant DOI), provides a link to the license, and that the licensor is not represented as endorsing the use made of the work. The full details of the license are available at http://creativecommons.org/licenses/by-nc-nd/4.0. Any commercial reuse of Open Access articles published with a CC BY NC SA or CC BY NC ND license requires permission from Elsevier and will be subject to a fee.
Commercial reuse includes:

- Associating advertising with the full text of the Article
- Charging fees for document delivery or access
- Article aggregation
- Systematic distribution via e-mail lists or share buttons

Posting or linking by commercial companies for use by customers of those companies.

20. **Other Conditions**:

v1.10

**Questions? customercare@copyright.com or +1-855-239-3415 (toll free in the US) or +1-978-646-2777.**

Dyes and Pigments 150 (2018) 16–20

Contents lists available at ScienceDirect

Dyes and Pigments

journal homepage: www.elsevier.com/locate/dyepig

# Ion-doped liquid-crystal cell with low opaque-state specular transmittance based on electro-hydrodynamic effect

Jae-Won Huh, Jin-Hun Kim, Seung-Won Oh, Seong-Min Ji, Tae-Hoon Yoon*

*Department of Electronics Engineering, Pusan National University, Busan 46241, South Korea*

ARTICLE INFO

*Keywords:*
Liquid crystals
Light shutter
Dichroic dye
Electro-hydrodynamic effect

ABSTRACT

We demonstrate an ion-doped liquid-crystal (LC) cell that can provide a very low specular transmittance in the opaque state using the electro-hydrodynamic effect. In the opaque state, the LC and dye molecules are oriented randomly in planes parallel to the substrates because of the electrohydrodynamic effect. We found that the total transmittance of an ion-doped LC cell is almost the same as that of a light shutter based on light absorption and its haze value is the same as that of a light shutter based on light scattering. Moreover, an ion-doped LC cell can be fabricated without an ultraviolet curable process. Owing to its excellent opaque state properties, an ion-doped LC cell can be used in see-through display and smart-window applications.

## 1. Introduction

Liquid-crystal (LC) light shutters can be used for various applications, such as light modulators, smart windows, and see-through displays [1–21]. In particular, light shutters, which can be used to control the haze and transmittance simultaneously, have been actively studied for smart-window and see-through display applications [6–16,19]. By simply adding dichroic dye molecules in an LC light shutter based on the light scattering, the light scattering and absorption can be simultaneously controlled.

However, there is a trade-off between the light scattering and absorption. Light scattering relies on the random orientation of LC molecules, whereas light absorption can be maximized by orienting the dye molecules in planes parallel to the substrates. To overcome this trade-off, double-layered devices can be employed [11,16]. One layer is used for light scattering and the other for light absorption. However, these light shutters may suffer from disadvantages, including a low transmittance in the transparent state, a high thickness, and high fabrication costs.

In this study, we demonstrate a single-layered LC light shutter in which LC molecules with negative dielectric anisotropy are oriented randomly in planes parallel to the substrates using the electro-hydrodynamic effect when an electric field is applied between the two substrates. We confirmed that the proposed light shutter exhibits not only almost the same total transmittance as a light shutter based on light absorption, but also the same haze value as a light shutter based on light scattering. Owing to its excellent opaque state and simple structure, we consider the proposed LC light shutter a promising candidate for smart-window and see-through display applications.

## 2. Principle of operation

### 2.1. A light shutter using the electro-hydrodynamic effect

Simply by adding ion dopants in LCs, we can realize a light shutter that is based on light scattering using the electro-hydrodynamic effect [22–24]. For switching the light shutter, the frequency of the applied voltage wave must be carefully chosen because the haze value and operating voltage strongly depend on it.

To confirm the frequency dependence, an LC cell fabricated using ion-doped negative LCs was observed using a polarized optical microscope (POM), as shown in Fig. 1(a). We also measured the total transmittance, specular transmittance, and haze using a haze meter (HM-65W, Murakami Color Research Laboratory) while changing the frequency of the applied voltage wave (direct current (DC), 10 Hz, 100 Hz, 1 kHz, and 10 kHz), as shown in Fig. 1(b).

The specular [diffuse] transmittance $T_s$ [$T_d$] refers to the ratio of the power of the beam that emerges from a cell, which is parallel (within a range of angles of 2.5°) [not parallel] to a beam entering the cell, to the power carried by the beam entering the LC cell. The total transmittance $T_t$ is the sum of the specular transmittance $T_s$ and the diffuse transmittance $T_d$: $T_t = T_s + T_d$. The haze $H$ is the ratio of the diffuse transmittance to the total transmittance: $H = T_d/T_t$.

In the initial transparent state, the POM images exhibit the dark state because of vertically aligned LC molecules. When a voltage wave with a frequency between 0 and 1 kHz is applied, the negative LCs are

* Corresponding author.
*E-mail address:* thyoon@pusan.ac.kr (T.-H. Yoon).

https://doi.org/10.1016/j.dyepig.2017.11.001
Received 7 September 2017; Received in revised form 1 November 2017; Accepted 2 November 2017
Available online 05 November 2017
0143-7208/ © 2017 Elsevier Ltd. All rights reserved.

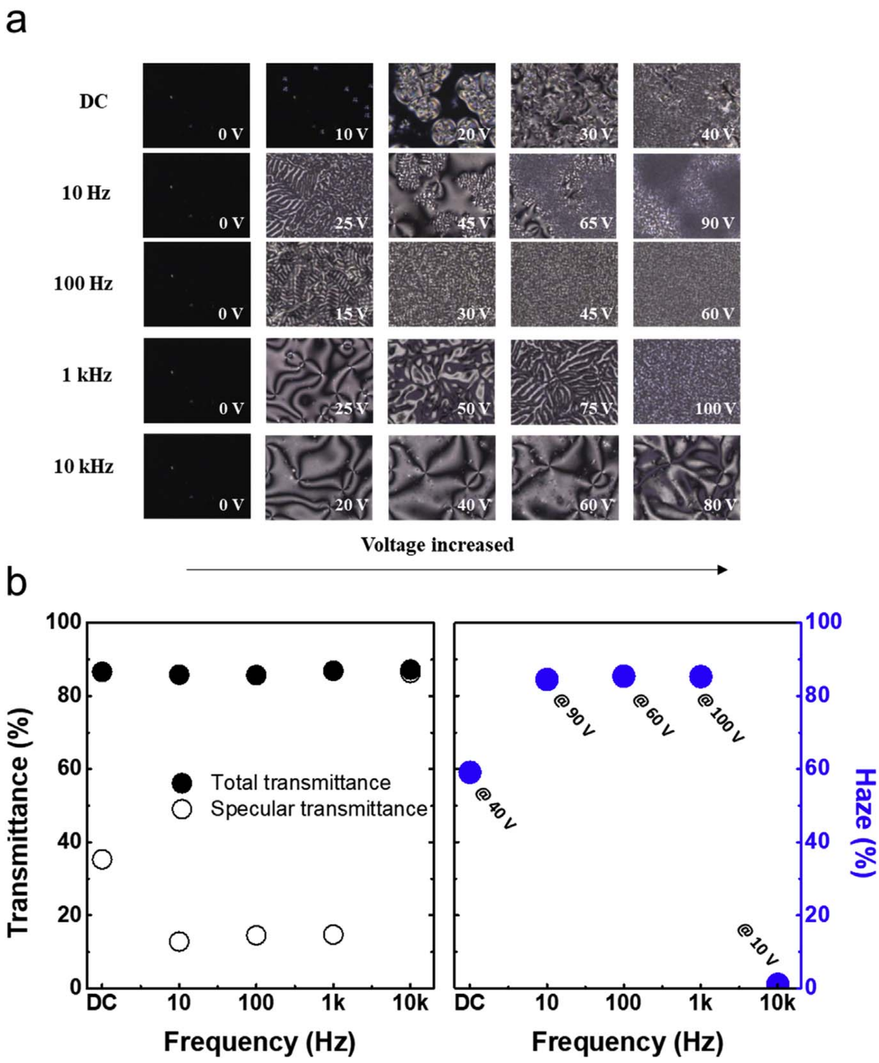


**Fig. 1.** (a) POM images and (b) the measured total transmittance, specular transmittance, haze of an ion-doped LC cell when DC, 10-Hz, 100-Hz, 1-kHz, and 10-kHz voltage waves were applied.

aligned parallel to the substrates. At the same time, the rotation of ions generates turbulence in the cell and POM image shows patterns called the 'Williams domain' [25]. As the applied voltage is increased, the turbulence becomes stronger, and the patterns become small domains which can scatter the incident light strongly. When the frequency of the applied voltage wave was 1 kHz, the cell showed a high haze of 85.3%. The POM image showed dependence on the frequency and amplitude of the applied voltage because the rotation of ions was affected by them [22–24]. When a 10-kHz voltage wave is applied to the cell, negative LCs is aligned parallel to the substrates and distributed randomly. However, the cell showed a low haze of 1.1% because the domains were not small enough for scattering when there was no turbulence. We set the operating frequency as 100 Hz, at which the operating voltage is lowest.

### 2.2. Ion-doped LC cell based on light scattering and absorption

The operating principle of the proposed light shutter is illustrated in Fig. 2. For light absorption by the LC cell, we use dichroic dye molecules because they are easily aligned along the LC alignment direction. When the polarization direction of the incident light is parallel to the absorption axis of the dye molecules, the incident light is strongly absorbed. Conversely, the incident light is weakly absorbed when the direction of polarization is perpendicular to the absorption axis [1,10]. For light scattering via the electro-hydrodynamic effect, we doped the LC mixture with an ionic material (tetra-n-butylammonium bromide, TBAB).

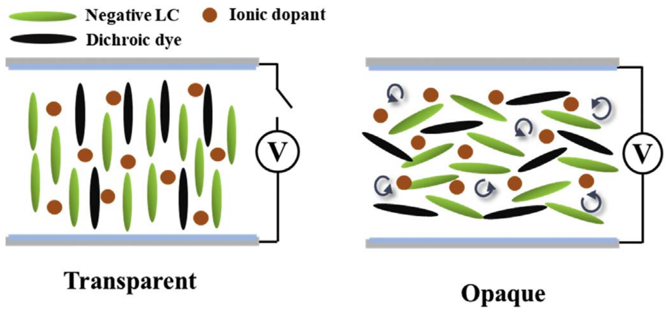


**Fig. 2.** Structure and operation of an ion-doped LC cell in the transparent and opaque states.

In the initial transparent state, most of the incident light passes through the LC cell because the LC and dye molecules are aligned perpendicular to the substrates. When a 100-Hz voltage wave is applied to the LC cell, the negative LC and dye molecules try to orient perpendicular to the vertical electric field. At the same time, rotation of ions caused by the electro-hydrodynamic effect brings about turbulence, which distributes LCs and dye molecules randomly in planes parallel to the substrates. In this state, the incident light is strongly absorbed because most of the dye molecules are oriented parallel to the substrates and scattered strongly by randomly distributed negative LCs with small domains. Using this scheme, an ion-doped LC cell can

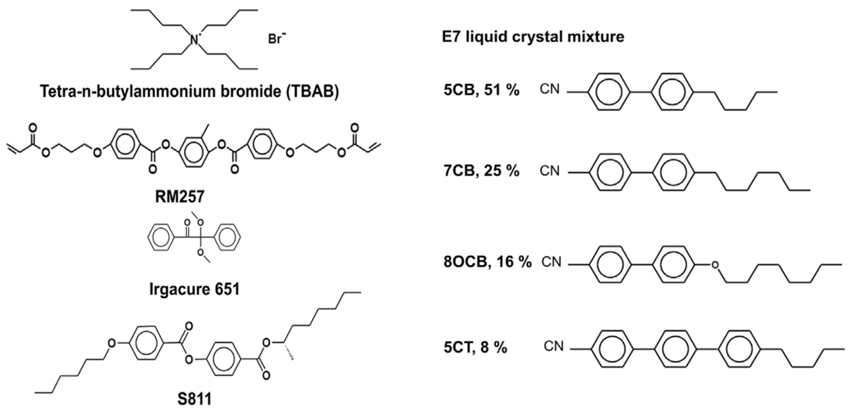


**Fig. 3.** Structure of the ionic material, monomer, photo-initiator, and positive LC mixture used for the fabrication of an ion-doped LC cell.

provide an excellent opaque state without a trade-off between light absorption and scattering.

## 3. Cell fabrication

To investigate the electro-optic characteristics, we fabricated an ion-doped LC cell. We mixed negative LCs (BHR28300-400, Bayi, $\Delta n$: 0.230 and $\Delta\varepsilon$: -9.3) with 0.1 wt% of TBAB (Sigma–Aldrich, the structure is shown in Fig. 3) for the electro-hydrodynamic effect and 1.5 wt% of dichroic dye for light absorption. We used a dichroic dye mixture consisting of X12 (BASF) and S-428 (Mitsui) for a ratio of 7:3. We used the homeotropic alignment layer (SE-5662, Nissan Chemical) and a cell gap of 10 μm for the LC cell.

To compare the transmission characteristics of an ion-doped LC cell with other LC light shutters, we fabricated polymer-stabilized LC (PSLC) and cholesteric LC (ChLC) cells [17,20]. For the same characteristics in the transparent state, we used the same cell gap, dichroic dye mixture, and concentration.

For the PSLC cell, we mixed negative LCs (RTA93000-100, HCCH, $\Delta n$: 0.200 and $\Delta\varepsilon$: -5.5) with 1.6 wt% of ultraviolet (UV) curable monomer (RM257, Merck, the structure is shown in Fig. 3), 0.4 wt% of photo-initiator, (Irgacure 651, BASF, the structure is shown in Fig. 3) and 1.5 wt% of the dichroic dye mixture. We used the same homeotropic alignment layer for the initial transparent state. Then, the LC cell was exposed to UV light of 30 mW/cm$^2$ for 10 min to form the polymer structure.

For the ChLC cell, we mixed positive LCs (E7, Merck, $\Delta n$: 0.237 and $\Delta\varepsilon$: 14.1, the structure is shown in Fig. 3) with 10 wt% of a chiral dopant (S811, Merck, reflection wavelength = 1600 nm, number of pitches: 10) and 1.5 wt% of the dichroic dye mixture. We used a homogeneous alignment layer (PIA-5310, Nissan Chemical) and rubbed in the anti-parallel direction.

## 4. Experimental results and discussion

To evaluate the electro–optic characteristics of the fabricated LC cells, we measured the total transmittance, specular transmittance, and haze using a haze meter (HM-65W, Murakami Color Research Laboratory) while changing the frequency of the applied voltage wave because the electro-hydrodynamic effect strongly depends on the frequency of the applied voltage wave.

Fig. 4 shows the measured total transmittance, specular transmittance, and haze of an ion-doped LC cell. In the initial transparent state, the total transmittance, specular transmittance, and haze values of the ion-doped LC cell were 61.2%, 60.1%, and 0.7%, respectively. When a 100-Hz voltage wave was applied to the cell, the total transmittance and specular transmittance decreased, and haze increased as the amplitude of the applied voltage wave was increased because the negative LC and dye molecules were randomly oriented parallel to the substrates

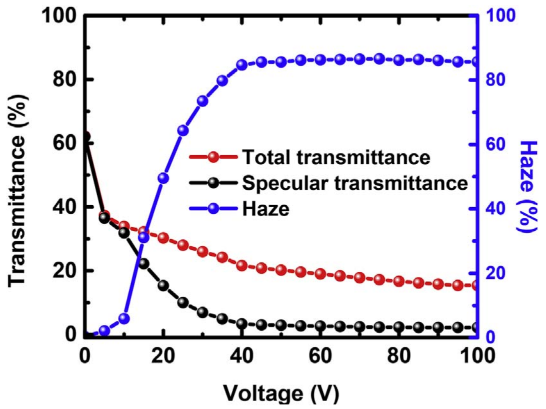


**Fig. 4.** Measured total transmittance, specular transmittance, and haze of an ion-doped LC cell.

by the electro-hydrodynamic effect.

We verified the trade-off between the scattering and absorption in the opaque state by measuring the transmission characteristics of an ion-doped LC cell and other light shutters. The measured specular transmittance can be influenced not only by light absorption but also light scattering, whereas the measured total transmittance can be influenced only by light absorption.

As shown in Fig. 5, the fabricated LC cells exhibited almost the same specular transmittance in the transparent state because we used the same dye mixture and concentration. In the opaque state, the ion-doped LC cell exhibited the lowest specular transmittance among the fabricated LC cells. A ChLC cell in the planar state exhibited a relatively high

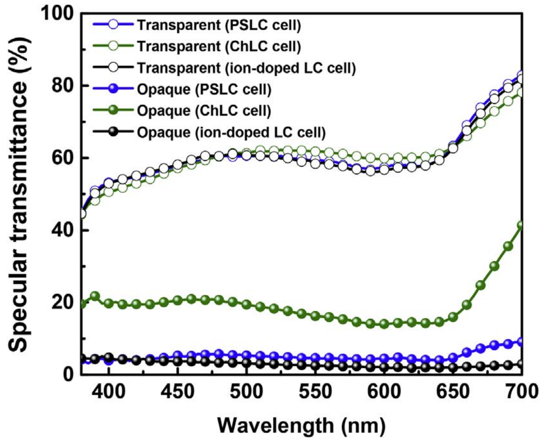


**Fig. 5.** Measured transmission spectra in the transparent and opaque states of ChLC, PSLC, and ion-doped LC cells.

**Table 1**
Total transmittance, specular transmittance, and haze values of ChLC, PSLC, and ion-doped LC cells.

| | Transparent state | | | Opaque state | | |
|---|---|---|---|---|---|---|
| | ChLC cell (homeotropic 45 V) | PSLC cell | Ion-doped LC cell | ChLC cell (planar) | PSLC cell (50 V) | Ion-doped LC cell (40 V) |
| Total transmittance (%) | 60.9 | 62.9 | 61.2 | 18.9 | 31.4 | 20.8 |
| Specular transmittance (%) | 60.5 | 60.8 | 60.1 | 18.7 | 5.3 | 3.0 |
| Haze (%) | 0.7 | 4.3 | 0.7 | 1.1 | 82.7 | 85.6 |

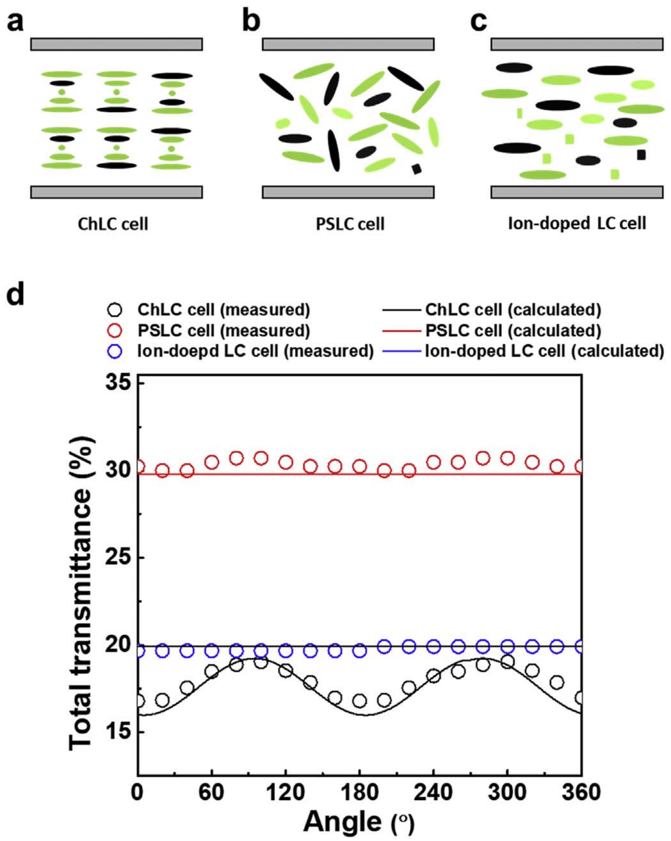


**Fig. 6.** LC and dye distributions in (a) ChLC, (b) PSLC, and (c) ion-doped LC cells assumed for the numerical calculation and (d) the angular dependence of the total transmittance measured or calculated using a polarized light source. The angle 0° is defined as the rubbing direction in a ChLC cell.

specular transmittance because it does not rely on light scattering.

However, the total transmittance of the fabricated cells in the opaque state shows a tendency different from the specular transmittance, as shown in Table 1. Among the fabricated LC cells, the ChLC cell exhibited the lowest total transmittance, whereas the PSLC cell exhibited the highest total transmittance. The total transmittance of an ion-doped LC cell is similar to that of a ChLC cell, whereas the haze value is similar to that of a PSLC cell.

We expect that an ion-doped LC cell strongly absorbs the incident light because of the negative LCs and dye molecules that are aligned parallel to the two substrates and strongly scatters the incident light via the electro-hydrodynamic effect. The operating voltage (40 V) of the ion-doped LC cell was lower than those of the PSLC (50 V) and ChLC (45 V) cells.

To investigate the distributions of the LC and dichroic dye molecules in the opaque state, we measured the total transmittance using a haze meter while rotating the polarization angle of the light source. For comparison, we calculated the distributions of the LC and dye by using the commercial software Techwiz LCD 1D (Sanayi System Company, Ltd.). For numerical calculations, we assumed that all the LC and dye molecules in the ChLC cell were aligned in planes parallel to the substrates, as shown in Fig. 6(a). We assumed that the LC and dye molecules in the PSLC cell were oriented randomly, as shown in Fig. 6(b). We assumed that the LC and dye molecules in the ion-doped LC cell were distributed randomly but that all of them had a tilt angle of 0°, as shown in Fig. 6(c). As shown in Fig. 6(d), the calculated results match the experimental results very well. The angular dependence of the total transmittance in the ChLC cell is caused by the wave-guiding effect [10].

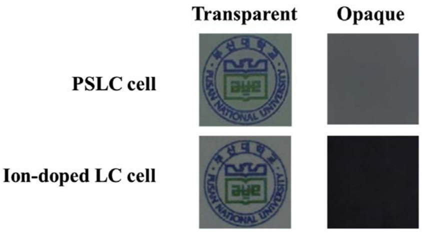


**Fig. 7.** Images of PSLC and ion-doped LC cells placed on a printed paper.

The ChLC cell exhibited the lowest transmittance regardless of the polarization angle because all the LC and dye molecules were aligned parallel to the substrates, and it had a twisted structure in the planar state. The PSLC cell exhibited the highest transmittance among all the fabricated LC cells because most of the LC and dye molecules were not aligned parallel to the substrates. The ion-doped LC cell exhibited a slightly higher transmittance than the ChLC cell because all the LC and dye molecules were not oriented parallel to the substrates.

Fig. 7 shows the images of PSLC and ion-doped LC cells placed on a printed paper. We can identify the printed images clearly in the transparent state of both types of LC cells. In the opaque state, both types of cells could hide objects behind them. However, the ion-doped LC cell exhibited a far darker state than the PSLC cell because of the lower total transmittance.

## 5. Conclusion

We demonstrated an LC cell that can provide a very low transmittance in the opaque state. To realize light absorption and the scattering effect simultaneously, we doped the LC with TBAB and dichroic dye. In the opaque state, an ion-doped LC cell can have a very low total transmittance because most of the dye molecules are oriented nearly parallel to the substrates; thus, we can achieve an excellent opaque state. Moreover, the cell can be fabricated without a UV curing process. We consider the ion-doped LC cell an excellent new candidate for see-through display and smart-window applications.

## Acknowledgement

This work was supported by the National Research Foundation of Korea (NRF) grant funded by the Korean government (MSIP) (No. 2017R1A2A1A0500106).

## References

[1] Heilmeier GH, Zanoni LA. Guest-host interactions in nematic liquid crystals. a new electrooptic effect. Appl Phys Lett 1968;13(3):91–2.
[2] Doane JW, Vaz NA, Wu BG, Žumer S. Field controlled light scattering from nematic microdroplets. Appl Phys Lett 1986;48(4):269–71.
[3] Drzaic PS. Polymer dispersed nematic liquid crystal for large area displays and light valves. J Appl Phys 1986;60(6):2142–8.
[4] Hikmet RAM. Electrically induced light scattering from anisotropic gels. J Appl Phys 1990;68(9):4406–12.
[5] Yang D-K, West JL, Chien L-C, Doane JW. Control of reflectivity and bistability in displays using cholesteric liquid crystals. J Appl Phys 1994;76(2):1331–3.
[6] Lin Y-H, Yang J-M, Lin Y-R, Jeng S-C, Liao C-C. A polarizer-free flexible and reflective electro-optical switch using dye-doped liquid crystal gels. Opt Express 2008;16(3):1777–85.
[7] Fuh Y-G, Chen C-C, Liu C-K, Cheng K-T. Polarizer-free, electrically switchable and optically rewritable displays based on dye-doped polymer-dispersed liquid crystals. Opt Express 2009;17(9):7088–94.
[8] Lee GH, Hwang KY, Jang JE, Jin YW, Lee SY, Jung JE. Characteristics of color optical shutter with dye-doped polymer network liquid crystal. Opt Lett 2011;36(5):754–6.
[9] Wang C-T, Lin T-H. Bistable reflective polarizer-free optical switch based on dye-doped cholesteric liquid crystal. Opt Mater. Express 2011;1(8):1457–62.
[10] Yu B-H, Huh J-W, Kim K-H, Yoon T-H. Light shutter using dichroic-dye-doped long-pitch cholesteric liquid crystals. Opt Express 2013;21(24):29332–7.
[11] Huh J-W, Yu B-H, Heo J, Yoon T-H. Double-layered light shutter using long-pitch cholesteric liquid crystal cells. Appl Opt 2015;54(12):3792–5.
[12] Heo J, Huh J-W, Yoon T-H. Fast-switching initially-transparent liquid crystal light shutter with crossed patterned electrodes. AIP Adv 2015;5(4):047118.
[13] Yu B-H, Huh J-W, Heo J, Yoon T-H. Simultaneous control of haze and transmittance using a dye-doped cholesteric liquid crystal cell. Liq Cryst 2015;42(10):1460–4.
[14] Kim M, Park KJ, Seok S, Ok JM, Jung H-T, Choe J, et al. Fabrication of microcapsules for dye-doped polymer-dispersed liquid crystal-based smart windows. ACS Appl Mater. Interfaces 2015;7(32):17904–9.
[15] Huh J-W, Ji S-M, Heo J, Yu B-H, Yoon T-H. Bistable light shutter using dye-doped cholesteric liquid crystals with crossed patterned electrodes. J Disp Technol 2016;12(8):779–83.
[16] Oh S-W, Baek J-M, Heo J, Yoon T-H. Dye-doped cholesteric liquid crystal light shutter with a polymer-dispersed liquid crystal film. Dyes Pigments 2016;134:36–40.
[17] Moheghi A, Nemati H, Li Y, Li Q, Yang D-K. Bistable salt doped cholesteric liquid crystals light shutter. Opt Mater 2016;52:219–23.
[18] Cheng K-T, Lee P-Y, Qasim MM, Liu C-K, Cheng W-F, Wilkinson TD. Electrically switchable and permanently stable light scattering modes by dynamic fingerprint chiral texture. ACS Appl Mater. Interfaces 2016;8(16):10483–93.
[19] Huh J-W, Yu B-H, Heo J, Ji S-M, Yoon T-H. Technologies for display application of liquid crystal light shutters. Mol Cryst Liq Cryst 2017;644(1):120–9.
[20] Choi T-H, Huh J-W, Woo J-H, Kim J-H, Jo Y-S, Yoon T-H. Switching between transparent and translucent states of a two-dimensional liquid crystal phase grating device with crossed interdigitated electrodes. Opt Express 2017;25(10):11275–82.
[21] Choi T-H, Woo J-H, Baek J-M, Choi Y, Yoon T-H. Fast control of haze value using electrically switchable diffraction in a fringe-field switching liquid crystal device. IEEE Tans Electron Dev 2017;64(8):3213–8.
[22] Heilmeier GH, Zanoniand LA, Barton LA. Dynamic scattering: a new electrooptic effect in certain classes of nematic liquid crystals. IEEE Proc 1968;56(7):1162–71.
[23] Heilmeier GH, Zanoni LA, Barton LA. Further studies of the dynamic scattering mode in nematic liquid crystals. IEEE T. Electron Dev 1970;17(1):22–6.
[24] Wang H, Wang L, Xie H, Li C, Guo S, Wang M, et al. Electrically controllable microstructures and dynamic light scattering properties of liquid crystals with negative dielectric anisotropy. RSC Adv 2015;5:33489–95.
[25] Williams R. Domains in liquid crystals. J Chem Phys 1963;39:384–8.

## Fabrication of Microcapsules for Dye-Doped Polymer-Dispersed Liquid Crystal-Based Smart Windows

**Author:** Mingyun Kim, Kyun Joo Park, Seunghwan Seok, et al
**Publication:** Applied Materials
**Publisher:** American Chemical Society
**Date:** Aug 1, 2015

*Copyright © 2015, American Chemical Society*

### PERMISSION/LICENSE IS GRANTED FOR YOUR ORDER AT NO CHARGE

This type of permission/license, instead of the standard Terms and Conditions, is sent to you because no fee is being charged for your order. Please note the following:

- Permission is granted for your request in both print and electronic formats, and translations.
- If figures and/or tables were requested, they may be adapted or used in part.
- Please print this page for your records and send a copy of it to your publisher/graduate school.
- Appropriate credit for the requested material should be given as follows: "Reprinted (adapted) with permission from {COMPLETE REFERENCE CITATION}. Copyright {YEAR} American Chemical Society." Insert appropriate information in place of the capitalized words.
- One-time permission is granted only for the use specified in your RightsLink request. No additional uses are granted (such as derivative works or other editions). For any uses, please submit a new request.

If credit is given to another source for the material you requested from RightsLink, permission must be obtained from that source.

BACK CLOSE WINDOW

© 2022 Copyright - All Rights Reserved | Copyright Clearance Center, Inc. | Privacy statement | Terms and Conditions
Comments? We would like to hear from you. E-mail us at customercare@copyright.com

ACS APPLIED MATERIALS & INTERFACES
Research Article
www.acsami.org

Downloaded via HARBIN INST OF TECHNOLOGY on September 19, 2022 at 09:22:26 (UTC).
See https://pubs.acs.org/sharingguidelines for options on how to legitimately share published articles.

# Fabrication of Microcapsules for Dye-Doped Polymer-Dispersed Liquid Crystal-Based Smart Windows

Mingyun Kim,[†] Kyun Joo Park,[†] Seunghwan Seok,[†] Jong Min Ok,[†] Hee-Tae Jung,[†] Jaehoon Choe,[‡] and Do Hyun Kim*[,†]

[†]Department of Chemical & Biomolecular Engineering, KAIST, 291, Daehak-ro, Yuseong-gu, Daejeon 305-701, Republic of Korea
[‡]Research Park, LG Chem, 188, Munji-ro, Yuseong-gu, Daejeon 305-738, Republic of Korea

Supporting Information

**ABSTRACT:** A dye-doped polymer-dispersed liquid crystal (PDLC) is an attractive material for application in smart windows. Smart windows using a PDLC can be operated simply and have a high contrast ratio compared to those of other devices that employed photochromic or thermochromic material. However, in conventional dye-doped PDLC methods, dye contamination can cause problems and has a limited degree of commercialization of electric smart windows. Here, we report on an approach to resolve dye-related problems by encapsulating the dye in monodispersed capsules. By encapsulation, a fabricated dye-doped PDLC had a contrast ratio of >120 at 600 nm. This fabrication method of encapsulating the dye in a core−shell structured microcapsule in a dye-doped PDLC device provides a practical platform for dye-doped PDLC-based smart windows.

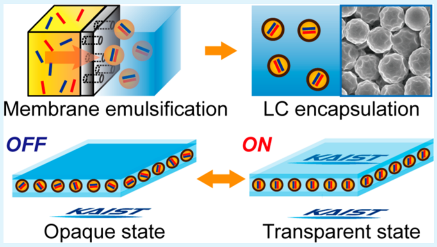


**KEYWORDS:** *smart windows, dye-doped PDLC, polymer-dispersed liquid crystal (PDLC), core−shell structure, polyurethane capsule, optical materials*

## 1. INTRODUCTION

Electro-optical switchable materials have been studied extensively to develop smart glass or smart windows that function through light transmission changes in response to a simple electric charge.[1−6] For smart windows, polymer-dispersed liquid crystal (PDLC) devices have been studied extensively, which can control the transmission of light by an applied voltage.[7−10] For practical use in smart windows, it is necessary to have a distinct transition between an opaque and transparent state. As such, a high contrast ratio (CR) is critical for an improved switching-state yield.

Until now, high switching yields with chromism have been achieved by using dichroic dyes.[11−14] However, the use of the dichroic dye may cause stain contamination from dye residue inside the polymer matrix. This results in the altered absorption of UV light by the dye, causing poor polymerization of the polymer matrix.[11,12,14−16] These dye-related problems can be responsible for a low switching-state yield, a short lifetime, and a high driving voltage for the operation of a dye-doped PDLC device. Thus, it is important to overcome dye-related problems and enhance the CR in the development of a dye-doped PDLC.

A material encapsulation technique using core−shell structured microcapsules may be a good candidate for manufacturing a dye-doped PDLC. Via the adoption of the encapsulation method, LC with a dye can be isolated in microcapsules, resulting in the separation of dyes from the polymer matrix. Furthermore, monodispersed LC-containing capsules can provide enhanced light transmission by preventing the light scattering caused by differently sized LC droplets.[9,17]

Thus far, the microcapsule fabrication technique has shown tremendous improvements by employing various methods such as a layer-by-layer (LbL) method, microfluidic droplet generation, and membrane emulsification.[18−24] Those methods can open new avenues to overcome the limitations of dye-doped PDLCs, which include dye-related issues and additional light scattering due to LC distribution.

In this study, we fabricated uniform-sized microcapsules with LC/dichroic dye cores and polyurethane/polyurea (PU/PUR) shells to create a dye-doped PDLC with a high CR. To prevent dye-related problems and to increase the CR, we fabricated the LC/dye-encapsulating polyurethane particles through integration of membrane emulsification and interfacial polymerization. Membrane emulsification produced monodispersed LC droplets ~4.5 $\mu$m in diameter, and the capsules were directly made through interfacial polymerization on the surface of the liquid core.[25−28] Isolation of LC with the dye by the PU/PUR capsule allowed us to avoid previously reported dye-related issues. Using LC/dye-encapsulated capsules, we demonstrated the switch from transparent and opaque states of a dye-doped PDLC through control of an ac current. We also performed optical evaluations, including light transmittance and CR, to demonstrate its practical application in smart windows.

**Received:** May 23, 2015
**Accepted:** July 20, 2015
**Published:** July 20, 2015

ACS Publications © 2015 American Chemical Society

DOI: 10.1021/acsami.5b04496
ACS Appl. Mater. Interfaces 2015, 7, 17904−17909

## 2. EXPERIMENTAL SECTION

**2.1. Materials.** Isophorone diisocyanate (IPDI), polypropylene glycol (PPG, $M_w$ ~ 2000), diethylenetriamine (DETA), dibutyltin dilaurate (DBTDL), polystyrene (PS) particle (10 $\mu$m), Tween 20, square-shaped indium tin oxide (ITO)-coated glass (surface resistivity of 70−100 Ω/sq, refractive index of 1.517), and polyvinylpyrrolidone (PVP, $M_w$ ~ 10000) were purchased from Sigma-Aldrich. ABIL EM 90 was purchased from Evonik Industries. LC (HPC 21600-000) and Sudan black B (SBB) were obtained from HCCH China and TCI America, respectively. The nematic−isotropic transition temperature of LC was 95 °C, and refractive indices of LC are as follows: $n_o$ = 1.524, $n_e$ = 1.765 at 589 nm, 20 °C. NOA 65 was purchased from Norland Products, and the refractive index of the cured polymer was 1.524. Deionized (DI) water (Human UP900, Human Corp.) was used for all aqueous solutions. All chemicals were used as received without purification.

**2.2. Preparation of Emulsions and Microcapsules.** The dispersed phase consisted of 9.385 g of LC, 3.33 g of IPDI (1.0 M), 1.8 g of PPG (0.06 M), 0.45 g of ABIL EM 90 (3 wt %), and 0.01 g of SBB (0.1 wt %).[29] A mixture of 180 mL of DI water, 0.25% PVP (w/v), and 0.0074% Tween 20 (w/v) was used as the continuous phase. In this study, ABIL EM 90, PVP, and Tween 20 were used as a surfactant to stabilize the emulsions and prevent droplet merging. The microdroplets were generated by using a 1.1 $\mu$m pore SPG membrane device (IMK-40M1, MC Tech) under a pressure of 20.2 kPa using nitrogen gas. In the reservoir, as a dispersed phase, an LC solution was passed through the membrane and a continuous phase was stirred at 175 rpm. The procedures are schematically shown in Figure S1. To make a PU/PUR shell, the generated emulsion was first mixed with 0.03 g of DBTDL in a beaker and then mixed with the aqueous solution of DETA (30.9 g), Tween 20 (0.07 g), PVP (0.3 g), and DI water (90 mL) at 60 °C. Then, the beaker was sealed, and the solution was stirred gently at 60 °C for 5 h. To characterize the generation of the capsule quantitatively, the yield was defined as the weight ratio between products and reactants. The obtained capsules were 13 g on average using the conditions described here. According to the number of functional groups in IPDI (two groups) and DETA (three groups), supposing IPDI and DETA reacted with a 1.5:1 ratio, 1.03 g of DETA reacted with 3.33 g of IPDI in the dispersed phase. Thus, the yield was 81%.

**2.3. Stability Test of Capsules.** To confirm the LC/dye protection performance of the capsules, we dispersed the LC/dye-containing capsules, cracked capsules using a mortar, and naked droplets. Each sample (1 g) was dispersed in DI water (30 mL) for 1 h. Then, 1 mL of the solution was obtained, and samples were separated with a centrifuge. The separated water was analyzed with a Fourier transform infrared spectroscopy microscope (FT-IR, ALPHA, BRUKER). In addition, we dispersed the capsules (0.03 g) in DI water (12 mL) to confirm the stability of capsules depending on time. After mixing, 1 mL of the solution was obtained, and the capsules were separated with a centrifuge. The separated water was analyzed by FT-IR.

**2.4. Fabrication of a Dye-Doped PDLC Device.** To fabricate a dye-doped PDLC device, LC-encapsulating microcapsules, uncured NOA 65, 10 $\mu$m diameter PS particle spacers for a uniform thickness, and two ITO glasses were used. For the uniform dispersion of LC capsules, 0.1 g of PS particles and 0.3 g of LC capsules were mixed in 1 g of DI water. Then, ITO glass was coated with 0.05 g of LC capsules and the PS mixture. After being coated, the glass was dried at room temperature for 12 h to evaporate the water completely. To make a polymer matrix, 0.025 g of NOA 65 was coated on the other ITO glass. Then, two ITO glasses were assembled together so that the glasses were stacked with each other and pressed. Finally, NOA 65 was cured by applying 7.5 J/cm$^2$ of UV irradiation at 365 nm.

**2.5. Electro-optical Property Measurements.** The electro-optical property of the dye-doped PDLC was measured by applied voltage in the range of 0−100 V for a dye-doped PDLC device at 1 kHz using a function generator (33220A, Agilent/HP), a voltage amplifier (F10A, FLC Electronics), and UV−vis spectroscopy (OPTIZEN 3220UV, MECASYS). The function generator can apply 0−10 V in 1 kHz, and the voltage amplifier can amplify the voltage 10 times. UV−vis spectroscopy was used to monitor the transmittance change of the dye-doped PDLC device according to the applied voltage.

**2.6. Instruments.** A field-emission transmission electron microscope (FE-TEM, Tecnai TF30 ST, FEI Co.), a field-emission scanning electron microscope (FE-SEM, S-4800, Hitachi), and a polarized optical microscope (POM, LV-100POL, Nikon) with a charge-coupled device (CCD) camera were used to characterize the morphology of the capsules.

## 3. RESULTS AND DISCUSSION

**3.1. Design of the Overall Experimental System.** All the fabrication procedures are schematically shown in Figure 1.

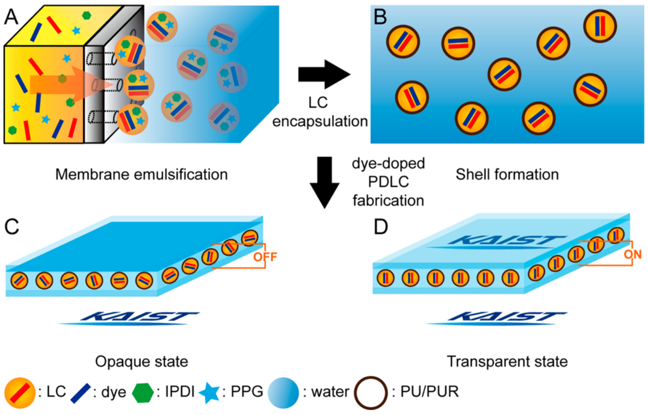


**Figure 1.** Schematic diagrams of the overall experimental system. (A) LC-containing droplet generation by the SPG membrane. (B) PU/PUR shell formation by interfacial polymerization. Electro-optical switching of dye-doped PDLC device in (C) the opaque state and (D) the transparent state by the applied electric field.

To fabricate LC-containing droplets, a hydrophobic dispersed phase (LC, dye, and monomers) was pressurized equally by nitrogen gas through a uniform pore-sized hydrophilic membrane. When the pressure reached a critical value, the LC solution overcomes the capillary pressure of the membrane pore and forms pendant drops on pores. With enough pressure to overcome the interfacial tension of each drop, the drops became sufficiently large and detached by shear force from the agitation of the surrounding water as shown in Figure 1A.[30] The detached droplets were stabilized by surfactants, and the polymerization began. The droplets are shown in Figure S2. The droplets had a diameter of 4.46 ± 0.24 $\mu$m, and the coefficient of variation was 5%. Inside the droplets, monomers reacted with each other and formed a polymer shell at the interface between the LC solution and amine-containing water as shown in Figure 1B. Finally, the fabricated capsules were applied in a dye-doped PDLC device to demonstrate control of light transmission as illustrated in panels C and D of Figure 1. The word "KAIST" was covered with the opaque dye-doped PDLC device at an off state, whereas "KAIST" can be seen when the electric field was applied to the dye-doped PDLC device.

**3.2. Mechanism of PU/PUR Capsule Formation.** To cover the LC and dye, PU/PUR was adopted as a LC-encapsulating polymer shell because of its refractive index (1.5−1.6), good mechanical property, and weatherability.[31−33] For the formation of a PU shell, IPDI and PPG were mixed


DOI: 10.1021/acsami.5b04496
ACS Appl. Mater. Interfaces 2015, 7, 17904−17909

with a LC solution and became components of the droplets. As monomers, IPDI and PPG have an isocyanate (NCO) group and hydroxyl (OH) group, respectively (Figure 2A and reaction

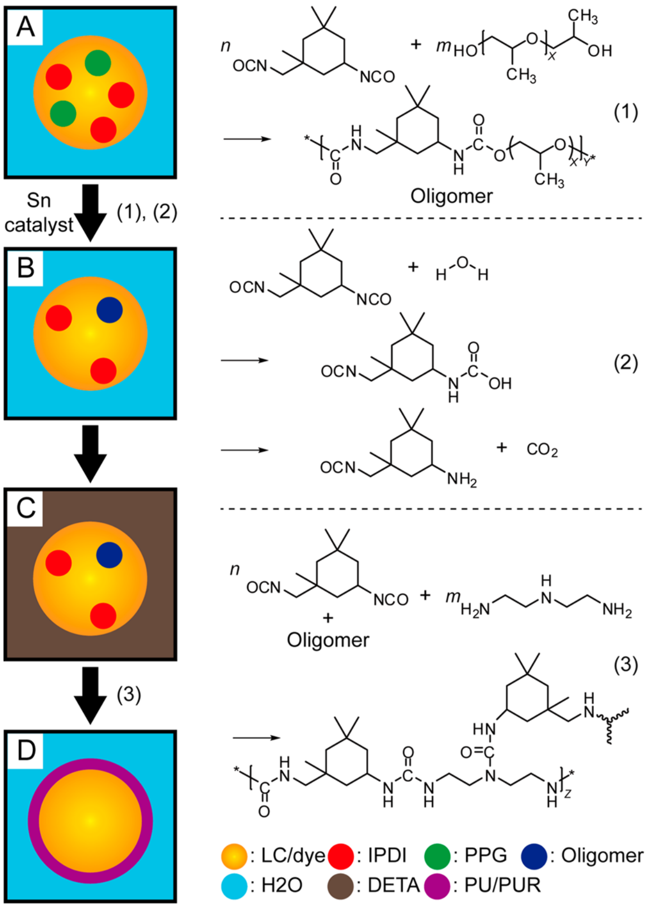


**Figure 2.** Scheme of PU/PUR shell formation. (A) IPDI, PPG, LC, and the dye-containing droplet. (B) Oligomer-synthesized droplet. (C) DETA-added droplet. (D) LC/dye-containing PU/PUR shell microcapsules. During the process, (1) urethane reaction, (2) reaction between IPDI and $H_2O$, and (3) a urea reaction can occur.

1). Here, PPG was not only a reactant but also a nonionic surfactant that improved the stability and size distribution of the emulsion.[34] In an IPDI molecule, two types of NCO groups exist, a primary isocyanatomethyl and a secondary cyclo-aliphatic isocyanate. Between the two NCO groups, the secondary isocyanate group is more reactive than the primary isocyanatomethyl group. In comparison with the secondary isocyanate group, the primary isocyanatomethyl group is less reactive because it is shielded to a greater extent by the adjacent methyl group and $\beta$-carbon.[33]

The reaction between the NCO group and OH group did not occur at the emulsification step (Figure 2A) because of the low activity of IPDI. Hence, the produced emulsion was mixed with a Sn catalyst and heated to activate a NCO group. The catalyst made the carbonyl carbon of the NCO group electron-deficient and reactive. The activated NCO group reacted with the OH group of PPG and produced a urethane oligomer for step growth polymerization (Figure 2B and reaction 1).

In addition to reaction 1 in Figure 2, a side reaction can also occur between the NCO group and surrounding water molecules as shown in reaction 2. The NCO group forms an unstable amino acid group that dissociates into carbon dioxide and an amine end group.[35] The amine group of the resulting reaction 2 participated in the following urea reaction 3 and converted to a urethane group.

After the formation of an oligomer, the remaining IPDI and oligomer reacted with DETA for further polymerization and interconnection of the oligomers by reaction 3 (Figure 2C). The DETA molecule has one reactive secondary amine in the middle and two primary amines at both ends.[31] The NCO group of IPDI and oligomers in a droplet reacted with the amine groups of DETA in the surrounding solution to form a urea group and cross-link the polymer chains on the surface of the droplets. After all of the reactions, LC/dye-containing PU/PUR capsules were obtained as illustrated schematically in Figure 2D.

**3.3. Characterization of Fabricated LC Capsules.** To confirm the proposed mechanism of PU/PUR shell formation, reactants and products were characterized by FT-IR (Figure 3).

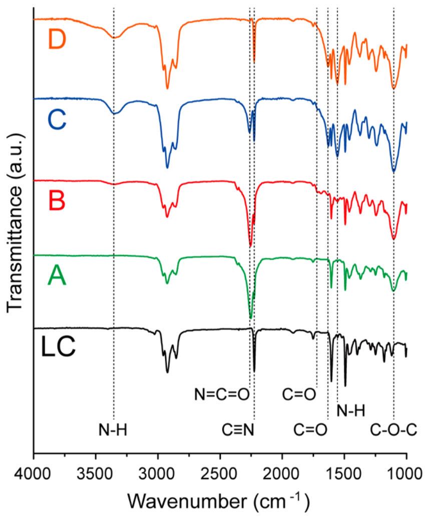


**Figure 3.** FT-IR spectra of the LC (black), (A) the IPDI/PPG/LC/dye mixture, (B) the oligomer-synthesized droplet, (C) the DETA-added droplet, and (D) the LC/dye-containing PU/PUR shell microcapsules.

In the spectra of the LC, the absorption peak located at 2225 $cm^{-1}$ was used to identify the LC. Here, the peak originated from the stretching vibration of nitrile (C≡N) group, and another peak at 2950 $cm^{-1}$ was from the stretching vibration of CH in the alkyl chain. In the spectra of the dispersed phase (IPDI, PPG, and LC) (Figure 3A), the peak of the C≡N group appeared to be due to the LC, and other peaks were also present at 2250 and 1100 $cm^{-1}$, which were attributed to the stretching vibration of the NCO group from the IPDI and ether (C−O−C) group from PPG, respectively. Because of the synthesized urethane (−NHCOO−) group, two more peaks were present at 3340 $cm^{-1}$ for the NH group and 1720 $cm^{-1}$ for the carbonyl (C═O) group as shown in Figure 3B. The intensity of the peak of the NCO group at 2250 $cm^{-1}$ decreased sharply, and the intensity of the peak of the NH group at 3340 $cm^{-1}$ increased with the addition of DETA (Figure 3C). Furthermore, new peaks were observed at 1630 and 1560 $cm^{-1}$, which can be ascribed to the stretching vibration of the C═O group in the urea (−NHCONH−) group and the bending vibration of NH in the urea group. These changes and generation of peaks prove that the NCO group reacted with an added amine (DETA) and produced urea linkages. In the


DOI: 10.1021/acsami.5b04496
ACS Appl. Mater. Interfaces 2015, 7, 17904−17909

spectra of the fabricated capsules (Figure 3D), we were able to observe the disappearance of the peak corresponding to the NCO group at 2250 cm$^{-1}$, the appearance of the peak of the NH group at 3340 cm$^{-1}$, and the peak of the C≡N group of LC at 2225 cm$^{-1}$. The obtained results provide confirmation of the encapsulation of LC in PU/PUR.

**3.4. Characterization of the Morphology of LC Capsules.** The fabricated PU/PUR capsules were characterized to examine their morphology and size distribution as shown in Figure 4 and Figure S3. The capsules had a diameter

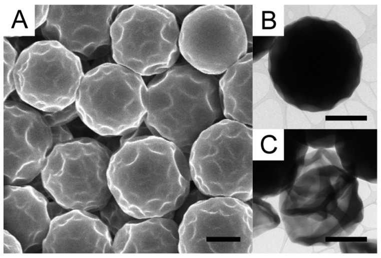


**Figure 4.** (A) SEM image of PU/PUR capsules and TEM images of the capsule (B) with LC and (C) without LC. The scale bar is 2 $\mu$m.

of 4.58 ± 0.19 $\mu$m with a of shell thickness of 100 nm on average, and the coefficient of variation was 4%, which indicates high monodispersity.[36] The monodisperse capsules can prevent additional light scattering caused by interdroplet scattering of different-sized droplets. To check the core−shell structure, sliced capsules were analyzed by scanning electron microscopy (Figure S4), and transmission electron microscopy analysis was also used to observe the inside of the capsules. The fully filled dark shape (Figure 4B) represented that LC was filled in the PU/PUR capsule. The wrinkled transparent image (Figure 4C) showed a case of deflated capsules. The results confirmed a core−shell structure. Filled LC was also investigated by a polarized optical microscope.

To demonstrate the performance of the LC core, polarized light was illuminated to microcapsules as shown in Figure 5.

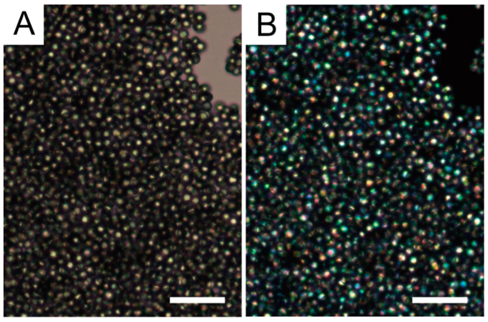


**Figure 5.** (A) Optical microscopic image of microcapsules and (B) polarization microscopic image of microcapsules. The scale bar is 25 $\mu$m.

The LC cores showed shiny colors because of its optical anisotropy, whereas isotropic material like water was shown as black. The LC cores show aligned characteristics in Figure S5. In addition, the nematic−isotropic transition temperature was investigated (Figure S6). The original nematic−isotropic transition temperature of LC was 95 °C. However, the temperature of encapsulated LC with dye became 90 °C.

**3.5. Test of the Stability of Capsules.** Stability tests were performed to confirm the dye-doped LC holding capability of the capsules during the fabrication process (Figure S7). In panel A, the case of encapsulated LC/dye showed no peaks. In the case of cracked capsules, there were several low peaks around 1500 cm$^{-1}$, which differed from those seen in encapsulated LC/dye tests. This result might come from the small amount of leakage of core materials. The naked droplets also showed several little peaks around 1500 cm$^{-1}$; in addition, this case showed LC peaks at 1500 and 1600 cm$^{-1}$. This result was reasonable because the naked droplets were present in forms of emulsion, which means there were many dispersed dye-doped LCs in the water. The results show the dye-doped LC holding capability of capsules. In addition, to confirm the stability of capsules depending on time, the fabricated LC/dye−core capsules were dispersed in DI water for 0 h to 7 days (Figure S7B). The FT-IR spectra showed no peaks even after 7 days. The results showed that a PU/PUR shell successfully held the LC/dye during the fabrication process. Therefore, the proposed LC/dye encapsulation can prevent dye-related problems and enhance the electro-optical properties of the dye-doped PDLC. The capsules were also tested in various organic solvents such as acetone, ethanol, and isopropyl alcohol. In these solvents, the capsules released core materials by dissolving the capsules.

**3.6. Application and Evaluation of a Dye-Doped PDLC Device.** Using the PU/PUR capsules, we assembled a dye-doped PDLC device to demonstrate the applicability of the capsulized LC (Figure 6). The dye-doped PDLC device was

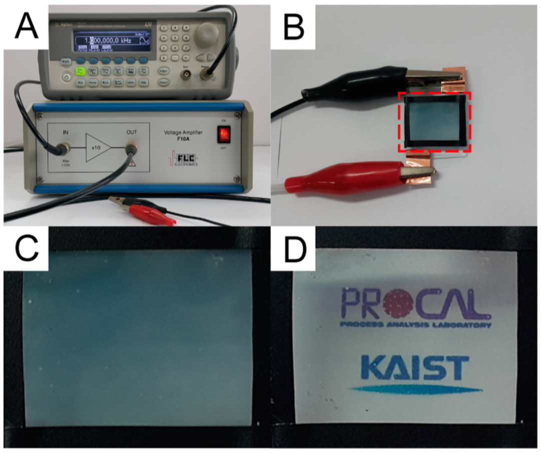


**Figure 6.** Photographs of (A) the voltage supplier and amplifier, (B) the fabricated dye-doped PDLC device, and (C) the off state (opaque) and (D) on state (transparent) of the dye-doped PDLC.

operated by supplying voltage from a function generator and a voltage amplifier (Figure 6A,B). In the absence of an electric field, LC and dye were randomly distributed. This random arrangement brought about mismatched refractive indices between the LC and polymer matrix and caused the opaque state of the dye-doped PDLC (Figure 6C). In comparison, applying an electrical field produced alignment of LC with the dye, which resulted in the refractive index matching with the polymer substrate as shown in Figure 6D.[8] The appearance of the previously hidden words "PROCAL" and "KAIST" indicates that a transparent state was achieved for the dye-doped PDLC.


DOI: 10.1021/acsami.5b04496
ACS Appl. Mater. Interfaces 2015, 7, 17904−17909

We evaluated the electro-optical properties of dye-doped PDLC for practical application. Transmittance, CR, threshold voltage ($V_{th}$), and driving voltage ($V_{on}$) are typical evaluation parameters for dye-doped PDLCs.[10,11,13,29,37] We present the data in Figure 7 and Table 1. Parameters $V_{th}$, $V_{on}$, and CR are generally defined as the voltage at 10 and 90% of the total transmittance change and the ratio between maximal transmittance ($T_{sat}$) and minimal transmittance ($T_0$), respectively. In Figure 7, transmittance increased with the wavelength because of the dispersed LC, which has a high transmittance at a high wavelength. As a result, the highest $T_{sat}$ was measured in dark red light at 700 nm as 90.46. Table 1 reports the properties over a visible wavelength range. In this range, the CR was 121 at 600 nm, corresponding to orange light. This value is sufficiently high compared with those from other studies in Table 2. The enhanced CR would be a result of the synergistic effect of the dye and monodisperse capsules. On the basis of the presented data, we believe that a dye-doped PDLC device using LC/dye capsules is suitable for practical smart windows.

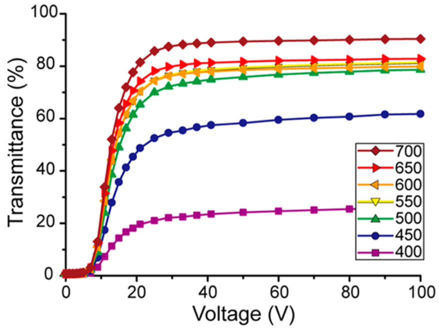


**Figure 7.** Applied voltage (1 kHz) dependence of the transmittance at different wavelengths.

**Table 1. Electro-optical Properties of the Dye-Doped PDLC at Different Wavelengths**

| wavelength (nm) | $T_0$ (%) | $T_{sat}$ (%) | CR | $V_{th}$ (V) | $V_{on}$ (V) |
|---|---|---|---|---|---|
| 700 | 0.83 | 90.46 | 109 | 8.1 | 20.9 |
| 650 | 0.69 | 82.83 | 120 | 8.1 | 21.3 |
| 600 | 0.66 | 79.89 | 121 | 8.2 | 22.4 |
| 550 | 0.68 | 81.39 | 120 | 8.3 | 23.6 |
| 500 | 0.67 | 78.68 | 117 | 8.6 | 26.4 |
| 450 | 0.57 | 61.79 | 108 | 8.7 | 33.5 |
| 400 | 0.45 | 26.04 | 58 | 8.4 | 39.9 |

**Table 2. Data from Other Studies**

| light source | dye | [dye] (wt %) | CR[a] |
|---|---|---|---|
| halogen laser beam, 560 nm | – | 0 | 45–50[10] |
| UV–vis, 600 nm | Sudan black B | 0.1 | 117.28[29] |
| He–Ne laser, 632.8 nm | Disperse red 1 | 0.015 | 114.6[11] |
| | anthraquinone red | 5 | 85.5[37] |
| white light | Hayashibara, azo type (G-series) | 0.2 | ~9[12] |
| | Disperse orange 3 | 0.0625 | 105.88[13] |

[a]The CR values are the maximal values of each study.

## 4. CONCLUSION

In this work, we fabricated microcapsules to isolate LC with a dye from a polymer matrix in dye-doped PDLC to improve transmittance and to prevent problems with dye contamination. The produced LC/dye-containing PU/PUR microcapsules were monodisperse with a diameter of ~4.5 $\mu$m. A mechanism for shell formation was also proposed. Furthermore, the LC/dye holding capacity of capsules without leakage was carefully investigated. To demonstrate the capability of encapsulated LC/dye in capsules as smart windows, we fabricated a dye-doped PDLC device. The device showed a CR higher than 120 at 600 nm and a $T_{sat}$ of 90 at 700 nm. The results for the LC/dye−core/PU/PUR−shell microcapsules and dye-doped PDLC device indicate that LC-encapsulated capsules are promising for application in flexible displays and wearable PDLCs, as well as in improved smart windows.

## ■ ASSOCIATED CONTENT

### Supporting Information

The Supporting Information is available free of charge on the ACS Publications website at DOI: 10.1021/acsami.5b04496.

Schematic diagram of the preparation of the emulsion (Figure S1), optical microscopic image of the emulsion (Figure S2), capsule size distribution (Figure S3), SEM image of torn microcapsules (Figure S4), polarization microscopic images of rotated microcapsules (Figure S5), polarization microscopic images of LC/dye-containing capsules (Figure S6), FT-IR spectra of DI water with LC (green) and dye (red) as well as DI water in which encapsulated LC/dye (blue), cracked capsules (orange), and naked droplets (pink) were soaked and removed and DI water in which the capsules were dispersed for 0 h (green), 1 h (red), 1 day (blue), 4 days (brown), and 7 days (pink) (Figure S7) (PDF)

## ■ AUTHOR INFORMATION

### Corresponding Author

*E-mail: dohyun.kim@kaist.ac.kr. Fax: (+82) 42-350-3910.

### Notes

The authors declare no competing financial interest.

## ■ ACKNOWLEDGMENTS

This research was supported by the Basic Science Research Program through the National Research Foundation of Korea (NRF) funded by the Ministry of Science, ICT & Future Planning (2014R1A5A1009799).

## ■ REFERENCES

(1) Österholm, A. M.; Shen, D. E.; Kerszulis, J. A.; Bulloch, R. H.; Kuepfert, M.; Dyer, A. L.; Reynolds, J. R. Four Shades of Brown: Tuning of Electrochromic Polymer Blends Toward High-Contrast Eyewear. *ACS Appl. Mater. Interfaces* **2015**, *7*, 1413−1421.

(2) Kempe, M. D.; Scruggs, N. R.; Verduzco, R.; Lal, J.; Kornfield, J. A. Self-Assembled Liquid-Crystalline Gels Designed from the Bottom up. *Nat. Mater.* **2004**, *3*, 177−182.

(3) Wang, J.; Zhang, L.; Yu, L.; Jiao, Z.; Xie, H.; Lou, X. W. D.; Sun, X. W. A Bi-Functional Device for Self-Powered Electrochromic Window and Self-Rechargeable Transparent Battery Applications. *Nat. Commun.* **2014**, *5*, 4921.

(4) Runnerstrom, E. L.; Llordés, A.; Lounis, S. D.; Milliron, D. J. Nanostructured Electrochromic Smart Windows: Traditional Materials and NIR-Selective Plasmonic Nanocrystals. *Chem. Commun.* **2014**, *50*, 10555−10572.

(5) Hosseinzadeh Khaligh, H.; Liew, K.; Han, Y.; Abukhdeir, N. M.; Goldthorpe, I. A. Silver Nanowire Transparent Electrodes for Liquid Crystal-Based Smart Windows. *Sol. Energy Mater. Sol. Cells* **2015**, *132*, 337−341.

(6) Lampert, C. M. Large-Area Smart Glass and Integrated Photovoltaics. *Sol. Energy Mater. Sol. Cells* **2003**, *76*, 489−499.

(7) Kumano, N.; Seki, T.; Ishii, M.; Nakamura, H.; Umemura, T.; Takeoka, Y. Multicolor Polymer-Dispersed Liquid Crystals. *Adv. Mater.* **2011**, *23*, 884−888.

(8) West, J. L.; Ondris-Crawford, R. Characterization of Polymer Dispersed Liquid-Crystal Shutters by Ultraviolet/Visible and Infrared Absorption Spectroscopy. *J. Appl. Phys.* **1991**, *70*, 3785−3790.

(9) Ferrari, J. A.; Dalchiele, E. A.; Frins, E. M.; Gentilini, J. A.; Perciante, C. D.; Scherschener, E. Effect of Size Polydispersity in Polymer-Dispersed Liquid-Crystal Films. *J. Appl. Phys.* **2008**, *103*, 084505.

(10) Gao, Y.; Song, P.; Zhang, T.; Yao, W.; Ding, H.; Xiao, J.; Zhu, S.; Cao, H.; Yang, H. Effects of a Triethylamine Catalyst on Curing Time and Electro-Optical Properties of PDLC Films. *RSC Adv.* **2013**, *3*, 23533−23538.

(11) Deshmukh, R. R.; Malik, M. K. Effect of Dichroic Dye on Phase Separation Kinetics and Electro-Optical Characteristics of Polymer Dispersed Liquid Crystals. *J. Phys. Chem. Solids* **2013**, *74*, 215−224.

(12) Jung, J. E.; Lee, G. H.; Jang, J. E.; Hwang, K. Y.; Ahmad, F.; Muhammad, J.; Woo Lee, J.; Jeon, Y. J. Optical Enhancement of Dye-Doped PDLC by Additional Dye-LC Layer Coating. *Opt. Mater.* **2011**, *34*, 256−260.

(13) Kumar, P.; Neeraj; Kang, S.-W.; Lee, S. H.; Raina, K. K. Analysis of Dichroic Dye-Doped Polymer-Dispersed Liquid Crystal Materials for Display Devices. *Thin Solid Films* **2011**, *520*, 457−463.

(14) Eun Jung, J.; Lee, G. H.; Eun Jang, J.; Hwang, K. Y.; Ahmad, F.; Jamil, M.; Jin Woo, L.; Jae Jeon, Y. Optical Property Enhancement of Dye-PDLC Using Active Reflector Structure. *J. Appl. Polym. Sci.* **2012**, *124*, 873−877.

(15) Wu, S. T.; Yang, D. K. *Fundamentals of liquid crystal devices*, 1st ed.; John Wiley and Sons: New York, 2006.

(16) Chen, M.-Y.; Lee, J.-Y. Preparation of Dye-Doped Polymer-Dispersed Liquid Crystals Using Acrylic Monomers, and Enhancement of Contrast by Using THF Solvent as a Cleaning Agent. *J. Chin. Inst. Eng.* **2014**, *37*, 793−798.

(17) Loiko, V. A.; Dick, V. P. Coherent Transmittance of a Polymer Dispersed Liquid Crystal Film in a Strong Field: Effect of Correlation and Polydispersity of Droplets. *Opt. Spectrosc.* **2003**, *94*, 595−599.

(18) Gupta, J. K.; Sivakumar, S.; Caruso, F.; Abbott, N. L. Size-Dependent Ordering of Liquid Crystals Observed in Polymeric Capsules with Micrometer and Smaller Diameters. *Angew. Chem., Int. Ed.* **2009**, *48*, 1652−1655.

(19) Lee, S. S.; Kim, B.; Kim, S. K.; Won, J. C.; Kim, Y. H.; Kim, S. H. Robust Microfluidic Encapsulation of Cholesteric Liquid Crystals Toward Photonic Ink Capsules. *Adv. Mater.* **2015**, *27*, 627−633.

(20) Priest, C.; Quinn, A.; Postma, A.; Zelikin, A. N.; Ralston, J.; Caruso, F. Microfluidic Polymer Multilayer Adsorption on Liquid Crystal Droplets for Microcapsule Synthesis. *Lab Chip* **2008**, *8*, 2182−2187.

(21) Khan, W.; Park, S.-Y. Configuration Change of Liquid Crystal Microdroplets Coated with a Novel Polyacrylic Acid Block Liquid Crystalline Polymer by Protein Adsorption. *Lab Chip* **2012**, *12*, 4553−4559.

(22) Miller, D. S.; Wang, X.; Abbott, N. L. Design of Functional Materials Based on Liquid Crystalline Droplets. *Chem. Mater.* **2014**, *26*, 496−506.

(23) Yow, H. N.; Routh, A. F. Formation of Liquid Core-Polymer Shell Microcapsules. *Soft Matter* **2006**, *2*, 940−949.

(24) Hamlington, B. D.; Steinhaus, B.; Feng, J. J.; Link, D.; Shelley, M. J.; Shen, A. Q. Liquid Crystal Droplet Production in a Microfluidic Device. *Liq. Cryst.* **2007**, *34*, 861−870.

(25) Lee, J.; Hwang, D. R.; Shim, S. E.; Rhym, Y.-M. Controlling Morphology of Polymer Microspheres by Shirasu Porous Glass (SPG) Membrane Emulsification and Subsequent Polymerization: From Solid to Hollow. *Macromol. Res.* **2010**, *18*, 1142−1147.

(26) Vladisavljević, G. T.; Schubert, H. Influence of Process Parameters on Droplet Size Distribution in SPG Membrane Emulsification and Stability of Prepared Emulsion Droplets. *J. Membr. Sci.* **2003**, *225*, 15−23.

(27) Ma, G. H.; Nagai, M.; Omi, S. Study on Preparation and Morphology of Uniform Artificial Polystyrene−Poly(methyl methacrylate) Composite Microspheres by Employing the SPG (Shirasu Porous Glass) Membrane Emulsification Technique. *J. Colloid Interface Sci.* **1999**, *214*, 264−282.

(28) Cao, Z.; Ziener, U. A Versatile Technique to Fabricate Capsules: Miniemulsion. *Curr. Org. Chem.* **2013**, *17*, 30−38.

(29) Ahmad, F.; Jamil, M.; Jeon, Y. J.; Woo, L. J.; Jung, J. E.; Jang, J. E. Investigation of Nonionic Diazo Dye-Doped Polymer Dispersed Liquid Crystal Film. *Bull. Mater. Sci.* **2012**, *35*, 221−231.

(30) Geerken, M. J.; Lammertink, R. G. H.; Wessling, M. Interfacial Aspects of Water Drop Formation at Micro-Engineered Orifices. *J. Colloid Interface Sci.* **2007**, *312*, 460−469.

(31) Jabbari, E.; Khakpour, M. Morphology of and Release Behavior from Porous Polyurethane Microspheres. *Biomaterials* **2000**, *21*, 2073−2079.

(32) Mark, J. E. *Polymer Data Handbook*, 1st ed.; Oxford University Press: Oxford, U.K., 1999.

(33) Lomölder, R.; Plogmann, F.; Speier, P. Selectivity of Isophorone Diisocyanate in the Urethane Reaction Influence of Temperature, Catalysis, and Reaction Partners. *J. Coat. Technol.* **1997**, *69*, 51−57.

(34) Lu, S.; Xing, J.; Zhang, Z.; Jia, G. Preparation and Characterization of Polyurea/Polyurethane Double-Shell Microcapsules Containing Butyl Stearate through Interfacial Polymerization. *J. Appl. Polym. Sci.* **2011**, *121*, 3377−3383.

(35) Xu, J.; Han, H.; Zhang, L.; Zhu, X.; Jiang, X.; Kong, X. Z. Preparation of Highly Uniform and Crosslinked Polyurea Microspheres through Precipitation Copolymerization and Their Property and Structure Characterization. *RSC Adv.* **2014**, *4*, 32134−32141.

(36) Tang, L.; Fan, T. M.; Borst, L. B.; Cheng, J. Synthesis and Biological Response of Size-Specific, Monodisperse Drug-Silica Nanoconjugates. *ACS Nano* **2012**, *6*, 3954−3966.

(37) Yang, K.-J.; Lee, S.-C.; Choi, B.-D. Dye-Doped Polymer Dispersed Liquid Crystal Films for Flexible Displays. *Jpn. J. Appl. Phys.* **2010**, *49*, 05EA05.



DOI: 10.1021/acsami.5b04496
*ACS Appl. Mater. Interfaces* 2015, 7, 17904−17909

# ELSEVIER LICENSE TERMS AND CONDITIONS

Sep 19, 2022

---

This Agreement between Harbin Institute of Technology -- Ruicong Zhang ("You") and Elsevier ("Elsevier") consists of your license details and the terms and conditions provided by Elsevier and Copyright Clearance Center.

| | |
|---|---|
| License Number | 5392411421480 |
| License date | Sep 19, 2022 |
| Licensed Content Publisher | Elsevier |
| Licensed Content Publication | Dyes and Pigments |
| Licensed Content Title | Dichroic-dye-doped short pitch cholesteric liquid crystals for the application of electrically switchable smart windows |
| Licensed Content Author | Vijay Kumar Baliyan,Kwang-Un Jeong,Shin-Woong Kang |
| Licensed Content Date | Jul 1, 2019 |
| Licensed Content Volume | 166 |
| Licensed Content Issue | n/a |
| Licensed Content Pages | 7 |
| Start Page | 403 |
| End Page | 409 |
| Type of Use | reuse in a journal/magazine |
| Requestor type | academic/educational institute |
| Portion | figures/tables/illustrations |
| Number of figures/tables/illustrations | 1 |
| Format | both print and electronic |
| Are you the author of this Elsevier article? | No |
| Will you be translating? | No |
| Title of new article | Advanced liquid crystal-based switchable optical devices for light protection applications: principles and strategies |
| Lead author | Ruicong Zhang, Zhibo Zhang, Jiecai Han, Lei Yang, Jiajun li, Zicheng Song Tianyu Wang, Jiaqi Zhu |
| Title of targeted journal | Light: Science & Applications |
| Publisher | Springer Nature |
| Expected publication date | Nov 2022 |
| Portions | Figure 5 |
| Requestor Location | Harbin Institute of Technology<br>No. 92, Xidazhi Street, Nangang District<br><br><br>Harbin, 150080<br>China<br>Attn: Harbin Institute of Technology |
| Publisher Tax ID | GB 494 6272 12 |
| Total | **0.00 USD** |
| Terms and Conditions | |

**INTRODUCTION**

1. The publisher for this copyrighted material is Elsevier.  By clicking "accept" in connection with completing this licensing transaction, you agree that the following terms and conditions apply to this transaction (along with the Billing and Payment terms

and conditions established by Copyright Clearance Center, Inc. ("CCC"), at the time that you opened your Rightslink account and that are available at any time at http://myaccount.copyright.com).

**GENERAL TERMS**

2. Elsevier hereby grants you permission to reproduce the aforementioned material subject to the terms and conditions indicated.
3. Acknowledgement: If any part of the material to be used (for example, figures) has appeared in our publication with credit or acknowledgement to another source, permission must also be sought from that source. If such permission is not obtained then that material may not be included in your publication/copies. Suitable acknowledgement to the source must be made, either as a footnote or in a reference list at the end of your publication, as follows:
"Reprinted from Publication title, Vol /edition number, Author(s), Title of article / title of chapter, Pages No., Copyright (Year), with permission from Elsevier [OR APPLICABLE SOCIETY COPYRIGHT OWNER]." Also Lancet special credit - "Reprinted from The Lancet, Vol. number, Author(s), Title of article, Pages No., Copyright (Year), with permission from Elsevier."
4. Reproduction of this material is confined to the purpose and/or media for which permission is hereby given.
5. Altering/Modifying Material: Not Permitted. However figures and illustrations may be altered/adapted minimally to serve your work. Any other abbreviations, additions, deletions and/or any other alterations shall be made only with prior written authorization of Elsevier Ltd. (Please contact Elsevier's permissions helpdesk here). No modifications can be made to any Lancet figures/tables and they must be reproduced in full.
6. If the permission fee for the requested use of our material is waived in this instance, please be advised that your future requests for Elsevier materials may attract a fee.
7. Reservation of Rights: Publisher reserves all rights not specifically granted in the combination of (i) the license details provided by you and accepted in the course of this licensing transaction, (ii) these terms and conditions and (iii) CCC's Billing and Payment terms and conditions.
8. License Contingent Upon Payment: While you may exercise the rights licensed immediately upon issuance of the license at the end of the licensing process for the transaction, provided that you have disclosed complete and accurate details of your proposed use, no license is finally effective unless and until full payment is received from you (either by publisher or by CCC) as provided in CCC's Billing and Payment terms and conditions. If full payment is not received on a timely basis, then any license preliminarily granted shall be deemed automatically revoked and shall be void as if never granted. Further, in the event that you breach any of these terms and conditions or any of CCC's Billing and Payment terms and conditions, the license is automatically revoked and shall be void as if never granted. Use of materials as described in a revoked license, as well as any use of the materials beyond the scope of an unrevoked license, may constitute copyright infringement and publisher reserves the right to take any and all action to protect its copyright in the materials.
9. Warranties: Publisher makes no representations or warranties with respect to the licensed material.
10. Indemnity: You hereby indemnify and agree to hold harmless publisher and CCC, and their respective officers, directors, employees and agents, from and against any and all claims arising out of your use of the licensed material other than as specifically authorized pursuant to this license.
11. No Transfer of License: This license is personal to you and may not be sublicensed, assigned, or transferred by you to any other person without publisher's written permission.
12. No Amendment Except in Writing: This license may not be amended except in a writing signed by both parties (or, in the case of publisher, by CCC on publisher's behalf).
13. Objection to Contrary Terms: Publisher hereby objects to any terms contained in any purchase order, acknowledgment, check endorsement or other writing prepared by you, which terms are inconsistent with these terms and conditions or CCC's Billing and Payment terms and conditions. These terms and conditions, together with CCC's Billing and Payment terms and conditions (which are incorporated herein), comprise the entire agreement between you and publisher (and CCC) concerning this licensing transaction. In the event of any conflict between your obligations established by these terms and conditions and those established by CCC's Billing and Payment terms and conditions, these terms and conditions shall control.
14. Revocation: Elsevier or Copyright Clearance Center may deny the permissions described in this License at their sole discretion, for any reason or no reason, with a full refund payable to you. Notice of such denial will be made using the contact information provided by you. Failure to receive such notice will not alter or invalidate the denial. In no event will Elsevier or Copyright Clearance Center be responsible or liable for any costs, expenses or damage incurred by you as a result of a denial of your permission request, other than a refund of the amount(s) paid by you to Elsevier and/or Copyright Clearance Center for denied permissions.

**LIMITED LICENSE**

The following terms and conditions apply only to specific license types:
15. **Translation**: This permission is granted for non-exclusive world **English** rights only unless your license was granted for translation rights. If you licensed translation rights you may only translate this content into the languages you requested. A professional translator must perform all translations and reproduce the content word for word preserving the integrity of the article.
16. **Posting licensed content on any Website**: The following terms and conditions apply as follows: Licensing material from an Elsevier journal: All content posted to the web site must maintain the copyright information line on the bottom of each image; A hyper-text must be included to the Homepage of the journal from which you are licensing at http://www.sciencedirect.com/science/journal/xxxxx or the Elsevier homepage for books at http://www.elsevier.com; Central Storage: This license does not include permission for a scanned version of the material to be stored in a central repository such as that provided by Heron/XanEdu.
Licensing material from an Elsevier book: A hyper-text link must be included to the Elsevier homepage at http://www.elsevier.com . All content posted to the web site must maintain the copyright information line on the bottom of each image.

**Posting licensed content on Electronic reserve**: In addition to the above the following clauses are applicable: The web site must be password-protected and made available only to bona fide students registered on a relevant course. This permission is granted for 1 year only. You may obtain a new license for future website posting.
17. **For journal authors:** the following clauses are applicable in addition to the above:
**Preprints:**
A preprint is an author's own write-up of research results and analysis, it has not been peer-reviewed, nor has it had any other value added to it by a publisher (such as formatting, copyright, technical enhancement etc.).
Authors can share their preprints anywhere at any time. Preprints should not be added to or enhanced in any way in order to appear more like, or to substitute for, the final versions of articles however authors can update their preprints on arXiv or RePEc with their Accepted Author Manuscript (see below).
If accepted for publication, we encourage authors to link from the preprint to their formal publication via its DOI. Millions of researchers have access to the formal publications on ScienceDirect, and so links will help users to find, access, cite and use the best available version. Please note that Cell Press, The Lancet and some society-owned have different preprint policies. Information on these policies is available on the journal homepage.
**Accepted Author Manuscripts:** An accepted author manuscript is the manuscript of an article that has been accepted for publication and which typically includes author-incorporated changes suggested during submission, peer review and editor-author communications.
Authors can share their accepted author manuscript:

- immediately
  - via their non-commercial person homepage or blog
  - by updating a preprint in arXiv or RePEc with the accepted manuscript
  - via their research institute or institutional repository for internal institutional uses or as part of an invitation-only research collaboration work-group
  - directly by providing copies to their students or to research collaborators for their personal use
  - for private scholarly sharing as part of an invitation-only work group on commercial sites with which Elsevier has an agreement
- After the embargo period
  - via non-commercial hosting platforms such as their institutional repository
  - via commercial sites with which Elsevier has an agreement

In all cases accepted manuscripts should:

- link to the formal publication via its DOI
- bear a CC-BY-NC-ND license - this is easy to do
- if aggregated with other manuscripts, for example in a repository or other site, be shared in alignment with our hosting policy not be added to or enhanced in any way to appear more like, or to substitute for, the published journal article.

**Published journal article (JPA):** A published journal article (PJA) is the definitive final record of published research that appears or will appear in the journal and embodies all value-adding publishing activities including peer review co-ordination, copy-editing, formatting, (if relevant) pagination and online enrichment.
Policies for sharing publishing journal articles differ for subscription and gold open access articles:
**Subscription Articles:** If you are an author, please share a link to your article rather than the full-text. Millions of researchers have access to the formal publications on ScienceDirect, and so links will help your users to find, access, cite, and use the best available version.
Theses and dissertations which contain embedded PJAs as part of the formal submission can be posted publicly by the awarding institution with DOI links back to the formal publications on ScienceDirect.
If you are affiliated with a library that subscribes to ScienceDirect you have additional private sharing rights for others' research accessed under that agreement. This includes use for classroom teaching and internal training at the institution (including use in course packs and courseware programs), and inclusion of the article for grant funding purposes.
**Gold Open Access Articles:** May be shared according to the author-selected end-user license and should contain a CrossMark logo, the end user license, and a DOI link to the formal publication on ScienceDirect.
Please refer to Elsevier's posting policy for further information.
18. **For book authors** the following clauses are applicable in addition to the above: Authors are permitted to place a brief summary of their work online only. You are not allowed to download and post the published electronic version of your chapter, nor may you scan the printed edition to create an electronic version. **Posting to a repository:** Authors are permitted to post a summary of their chapter only in their institution's repository.
19. **Thesis/Dissertation**: If your license is for use in a thesis/dissertation your thesis may be submitted to your institution in either print or electronic form. Should your thesis be published commercially, please reapply for permission. These requirements include permission for the Library and Archives of Canada to supply single copies, on demand, of the complete thesis and include permission for Proquest/UMI to supply single copies, on demand, of the complete thesis. Should your thesis be published commercially, please reapply for permission. Theses and dissertations which contain embedded PJAs as part of the formal submission can be posted publicly by the awarding institution with DOI links back to the formal publications on ScienceDirect.

**Elsevier Open Access Terms and Conditions**
You can publish open access with Elsevier in hundreds of open access journals or in nearly 2000 established subscription journals that support open access publishing. Permitted third party re-use of these open access articles is defined by the author's choice of Creative Commons user license. See our open access license policy for more information.
**Terms & Conditions applicable to all Open Access articles published with Elsevier:**
Any reuse of the article must not represent the author as endorsing the adaptation of the article nor should the article be modified in such a way as to damage the author's honour or reputation. If any changes have been made, such changes must be clearly indicated.
The author(s) must be appropriately credited and we ask that you include the end user license and a DOI link to the formal publication on ScienceDirect.
If any part of the material to be used (for example, figures) has appeared in our publication with credit or acknowledgement to another source it is the responsibility of the user to ensure their reuse complies with the terms and conditions determined by the rights holder.
**Additional Terms & Conditions applicable to each Creative Commons user license:**
**CC BY:** The CC-BY license allows users to copy, to create extracts, abstracts and new works from the Article, to alter and revise the Article and to make commercial use of the Article (including reuse and/or resale of the Article by commercial entities), provided the user gives appropriate credit (with a link to the formal publication through the relevant DOI), provides a link to the license, indicates if changes were made and the licensor is not represented as endorsing the use made of the work. The full details of the license are available at http://creativecommons.org/licenses/by/4.0.
**CC BY NC SA:** The CC BY-NC-SA license allows users to copy, to create extracts, abstracts and new works from the Article, to alter and revise the Article, provided this is not done for commercial purposes, and that the user gives appropriate credit (with a link to the formal publication through the relevant DOI), provides a link to the license, indicates if changes were made and the licensor is not represented as endorsing the use made of the work. Further, any new works must be made available on the same conditions. The full details of the license are available at http://creativecommons.org/licenses/by-nc-sa/4.0.
**CC BY NC ND:** The CC BY-NC-ND license allows users to copy and distribute the Article, provided this is not done for commercial purposes and further does not permit distribution of the Article if it is changed or edited in any way, and provided the user gives appropriate credit (with a link to the formal publication through the relevant DOI), provides a link to the license, and that the licensor is not represented as endorsing the use made of the work. The full details of the license are available at http://creativecommons.org/licenses/by-nc-nd/4.0. Any commercial reuse of Open Access articles published with a CC BY NC SA or CC BY NC ND license requires permission from Elsevier and will be subject to a fee.
Commercial reuse includes:

- Associating advertising with the full text of the Article
- Charging fees for document delivery or access
- Article aggregation
- Systematic distribution via e-mail lists or share buttons

Posting or linking by commercial companies for use by customers of those companies.

20. **Other Conditions**:

v1.10

**Questions? customercare@copyright.com or +1-855-239-3415 (toll free in the US) or +1-978-646-2777.**

Dyes and Pigments 166 (2019) 403–409

Contents lists available at ScienceDirect

Dyes and Pigments

journal homepage: www.elsevier.com/locate/dyepig

# Dichroic-dye-doped short pitch cholesteric liquid crystals for the application of electrically switchable smart windows

Vijay Kumar Baliyan[a], Kwang-Un Jeong[b], Shin-Woong Kang[a,*]

[a] Dept. of BIN Convergence Technology, Chonbuk National University, Jeonju, 54896, South Korea
[b] Dept. of Polymer Nano Science and Technology, Chonbuk National University, Jeonju, 54896, South Korea

ARTICLE INFO

*Keywords:*
Cholesteric liquid crystal
Dichroic dye
Smart window
Reflective color
Photonic crystal
Privacy window

ABSTRACT

A short pitch cholesteric liquid crystal (CLC) acts as one-dimensional photonic crystals. It was widely studied for electro-optical switching devices. Although it was proposed for a switchable smart window, its spectral characteristics have critical limitations for window applications. In this report, we present dichroic-dye-doped short pitch CLC films, suitable for switchable window applications. The spectral limitations of a conventional CLC film in the voltage-off screening states were resolved by combining two different approaches of doping dichroic dye and increasing film thickness. Although each adjustment showed insufficient improvements, the implementation of a combined approach resulted in a drastically enhanced shielding performance in the voltage-off state, desirable for privacy windows. The increased film thickness promoted both the scattering focal conic and reflective multi-domain planar configurations for two different voltage-off states. The modified planar configuration caused a specific colored reflection with some turbidities. Furthermore, the dichroic dyes in both states effectively absorbed transmitting and scattered lights, resulting in the dark gray focal conic state and opaque colored reflection in the multi-domain planar state. Such combined efforts brought a great synergetic effect for enhancing a shielding efficiency. As a result, spectral properties of the voltage-off screening states were greatly different from the conventional CLC films, which show a milky white and tinted color, respectively, in a bright environment. The spectral performances of three switchable states were dramatically improved for the application of electrically switchable smart windows. The dichroic-dye-doped CLC films may be usefully adopted for switchable ultraviolet and heat windows if the cholesteric pitch and dichroic dyes are optimized for the corresponding wavelengths of electromagnetic radiation.

## 1. Introduction

Cholesteric liquid crystal (CLC) is one-dimensional (1D) periodic phase of matter with a liquid-like molecular order. It has a great potential for display applications and various electro-optical devices [1–4]. In the CLC phase, LC molecules are associated in a helical fashion, exhibiting one-dimensional periodicity. Due to the periodic helical arrangement of the molecules, the CLC exhibits periodic variation of refractive indices and therefore acts as the 1D photonic crystal. The periodicity (i.e., pitch) can be controlled by the concentration of added chiral components in the mixture. The CLC reveals a unique selective reflection of incident light in the regime of pitch (**P**) ~ refractive index (**n**). Depending on the handedness, the CLCs selectively reflect propagating lights, which meet the wavelength $\boldsymbol{\lambda} = \mathbf{n}{\cdot}\mathbf{P}$. Such selective reflection of CLC films has been adopted for various optical components such as color-filters [5–7], reflective displays [8,9], switchable gratings [10,11], light shutters, lasers [12], broadband reflectors [13–15], and composite films [16,17].

In particular, the electrically switchable bistable CLC was extensively studied for display applications [18,19]. Monochromatic information was effectively displayed on a black background, achieved by painting with the black ink. For a privacy window, however, the conventional CLC films have an intrinsic drawback for shielding transmitting light. Although it shows a good colored reflection with a dark background, it lacks in shielding capability (~16% in visible wavelengths) on bright environments. This is mainly due to the transmitted light (~84% in a visible range) coming from the opposite direction. For a privacy window application, therefore, it is important to curtail the transmitted light intensity for enhancing the screening efficiency.

The dichroic-dye-doped long pitch CLC film was proposed for electrically switchable windows [20–23]. Because the dye molecule with shape anisotropy exhibits dichroic absorptions, uniaxially aligned

* Corresponding author.
*E-mail address:* swkang@jbnu.ac.kr (S.-W. Kang).

https://doi.org/10.1016/j.dyepig.2019.03.045
Received 28 November 2018; Received in revised form 8 March 2019; Accepted 17 March 2019
Available online 19 March 2019
0143-7208/ © 2019 Elsevier Ltd. All rights reserved.

dyes show anisotropic absorption of light, depending on the propagation directions. When the dichroic dye is doped in LCs, the rod-like dye molecules are aligned along the LC director and switchable with LC molecules by applying electric field [22]. By switching CLC between planar and homeotropic states, the dye doped film present transparent and opaque states, respectively. In this case, the shielding state exhibited a dark gray with no color. The contrast between transparent and opaque states is not sufficient enough for effective switchable windows. Recently, emerging technologies were reviewed for the application of energy-efficient smart windows [24]. Each reviewed approach has advantages and disadvantages over other approaches. Currently, not many of them successfully reached to commercial markets. So far, none of them is predominating in the field. Competitiveness of each technology may depends on specific applications, requiring different specialty in their performances.

In this report, we demonstrate the dichroic-dye-doped short pitch CLCs for the electrically switchable smart window. To overcome the intrinsic drawback of conventional CLCs, we attempted to effectively shield a transmitting light by doping dichroic black dyes and by increasing film thickness. Although each adjustment made insufficient progress, the combined efforts resulted in dramatically enhanced screening efficiency. Four different types of CLC cells were fabricated to compare the performances for a switchable privacy window. The switching states of the cells were compared on various backgrounds. The molecular configurations of three switching states were confirmed by polarized optical microscopy. The spectral characteristics were examined by measuring reflectance and transmittance in the visible range. For the dye-doped CLC film with 20.0 μm thickness, both the multi-domain planar and focal conic states showed excellent screening state. They revealed opaque green reflection and dark gray scattering state, respectively. Based on the experimental results, the much enhanced shielding efficiency was explained by schematic illustrations on the molecular configurations of the three switching states, together with the macroscopic cell images.

## 2. Experimental section

### 2.1. Materials

The nematic liquid crystal MLC 15600-100 with dielectric anisotropy ($\varepsilon_{\parallel}$ = 60.6, $\varepsilon_{\perp}$ = 10.4, $\Delta\varepsilon$ = 50.2) and optical birefringence ($n_e$ = 1.6634, $n_o$ = 1.5018, $\Delta n$ = 0.1616) was used as a host LC. The eutectic mixture showed the phase transition from the crystal to namatic and isotropic phase at – 20.0 °C and 90.0 °C, respectively. The chiral dopant R-811 (R-octan-2-yl 4-((4-(hexyloxy)benzoyl)oxy) benzoate), was employed for adjusting the helical pitch of cholesteric mixtures [25]. The measured helical twisting power of R-811 in MLC15600-100 was approximately 12.5/μm. Both were provided by the Merck Performance Materials (Pyongtack, Korea) and used as received. For light absorbing dye, the commercial dichroic dye S-428 (black) was supplied by the Mitsui Fine Chemicals (Tokyo, Japan) and doped to the cholesteric mixtures as received [20,22]. The black dye was a mixture of multiple components from azo- and anthraquinone derivatives. Its dichroic ratios in ZLI-1840 were 9.6 (450 nm), 12.6 (550 nm), and 10.7 (650 nm). The solubility in ZLI 1840 was 5.0 wt% and absorption coefficients in toluene were 38.6, 25.9. 25.5, and 27.6 L/g cm. The absorption maxima in ZLI 1840 (toluene) were 406 nm (399 nm), 525 nm (530 nm), 594 nm (582 nm), and 639 nm (624 nm). The indium-tin-oxide (ITO) coated glass was used as substrates.

### 2.2. Preparation of cholesteric mixture

The color, originated from the selective reflection, could be optimized to red, green or blue by changing the concentration of a chiral dopant. For achieving green reflection at ~550 nm, the cholesteric liquid crystal (CLC) mixture was prepared by adding 23.5 wt% of the R-811 to the host LC. Homogeneous CLC mixture was obtained by stirring in the isotropic phase. By adding 23.5 wt% of the R-811, the nematic to isotropic phase transition temperature ($T_{NI}$) was lowered from 90.0 to 73.0 °C. For the dye-doped cells, the CLC mixture was doped with the dichroic dye S-428 by 1.0 wt%. By mixing the dye, the $T_{NI}$ was further reduced to 70.5 °C (decreased by ~ 2.5 °C).

### 2.3. Cell fabrication

The etched and unetched ITO-coated flat glasses were used as substrates with the alignment-layer coating. The commercial polyimide (PI) solution AL 6514 (JSR Korea) was spin cast and baked at 220 °C for 1 h. Subsequently, the PI-surface was uniaxially rubbed by a rubbing machine. Two facing substrates were assembled to the cell, where the rubbing direction of inner surfaces were aligned antiparallel each other. The cell gap was maintained using ball spacers at 5.0 μm or 20.0 μm. The top and bottom substrates were glued by the sealant. The CLC and dichroic-dye-doped CLC mixtures were loaded into the cells by capillary action in the isotropic temperature and slowly cooled to ambient temperature for characterizations. The switchable active area was either 15 x 15 mm or 42 x 24 mm.

### 2.4. Characterizations

Macroscopic optical properties of the CLC cells were inspected on white and black backgrounds. Microscopic switching states of the CLC cells were examined by polarized optical microscopy (POM), employing Nikon Eclipse LV 100 POL. The POM images were recorded by the Nikon DS-Ri1 CCD camera. Transmissive and reflective spectral characteristics were investigated using the UV–vis spectrophotometer (Jasco V-670, Japan), equipped with a contour integration accessory ARSN-733. Circularly polarized reflectance was measured by employing right or left circular polarizer for an incident beam. The electrical signals for electro-optical switching were produced by the function generator (Agilent 33521A, USA) and amplifier (FLC Electronics A400, Sweden). Square-wave voltage at 1 kHz frequency was applied across the CLC cells with various amplitudes. All characterizations were performed at a room temperature.

## 3. Results and discussion

Fig. 1 shows transmissive and reflective spectral characteristics for a conventional CLC cell with 5.0 μm gap (**Cell-1**). The distinct optical transmission spectra of three switchable states are shown in Fig. 1(a). The voltage-on homeotropic state at 13.0 $V_{pp}$ (black curve, ~85%) was transparent in the entire visible rage while the voltage-off focal conic state (blue curve, ~40%) exhibited semi-transparent state. For the other voltage-off state (red curve, ~70%), however, the transmittance was characterized by the semi-transparent window in a green spectral range, ~57 nm width centered at ~ 536 nm, which was attributed to the selective reflection of a planar cholesteric film. The transmittance differences between on-off switching from the homeotropic to the planar and focal conic state were approximately $\Delta T_p$ ~15 and $\Delta T_f$ ~45, respectively.

It is known that the distinct optical states are originated from molecular configurations of CLC films. Under applied voltage, cholesteric helix is completely unwound and LC molecules are aligned parallel to the electric field (i.e., homeotropic state). The cholesteric helix is uniformly aligned vertical to the substrate in the planar configuration while the cholesteric domains are randomly oriented in the focal conic state. Therefore, the corresponding configurations exhibit selective reflection and scattering of incident light, respectively. The configurations for voltage-off states are determined by the turn-off procedure. The homeotropic state switches to the planar configuration by abruptly removing the applied field to zero. However, it transforms to the focal

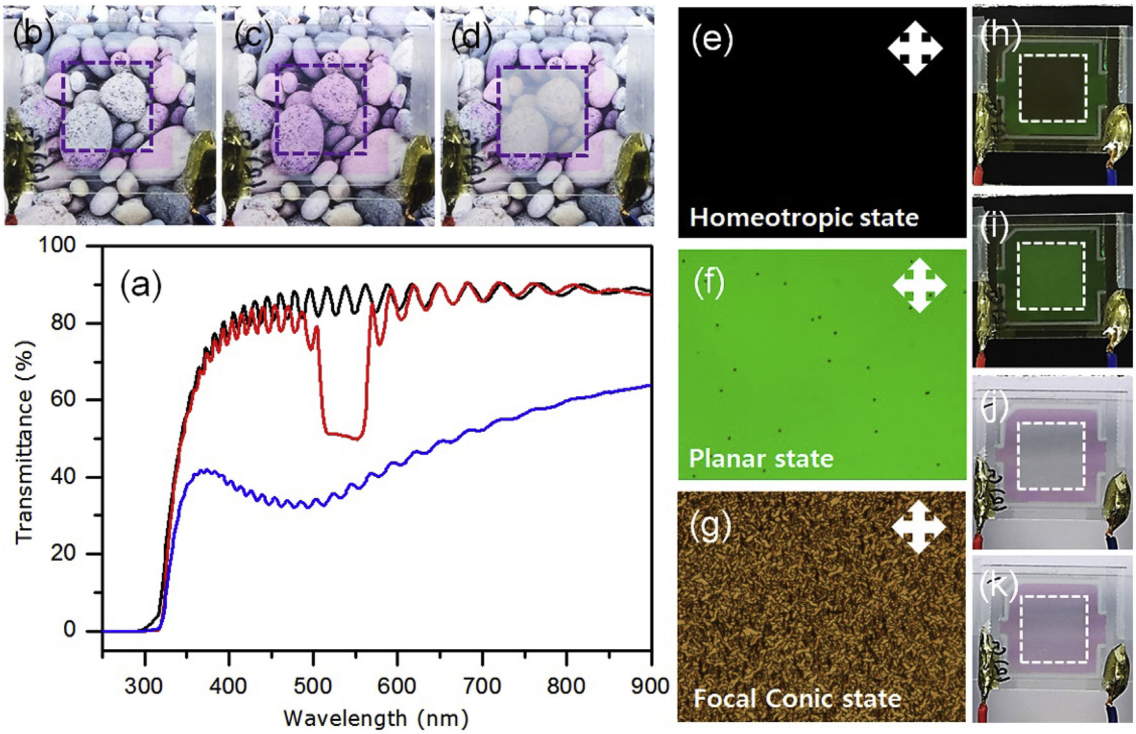


**Fig. 1.** Transmissive and reflective spectral characteristics of the CLC cell (**Cell-1**: 5.0 μm cell gap with no dichroic dye): (a) UV–vis transmission spectra for the homeotropic (black), planar (red), and focal conic (blue) states; (b)/(c)/(d) macroscopic cell images and (e)/(f)/(g) POM images for the transmitting homeotropic, reflective planar, and scattering focal conic states; The cell images of voltage-on homeotropic and voltage-off planar states on (h)/(i) black and ((j)/(k) white backgrounds. The active switching area (15 x 15 mm) was marked by the dotted square. The crossed arrows represent polarizer and analyzer for the POM images. (For interpretation of the references to color in this figure legend, the reader is referred to the Web version of this article.)

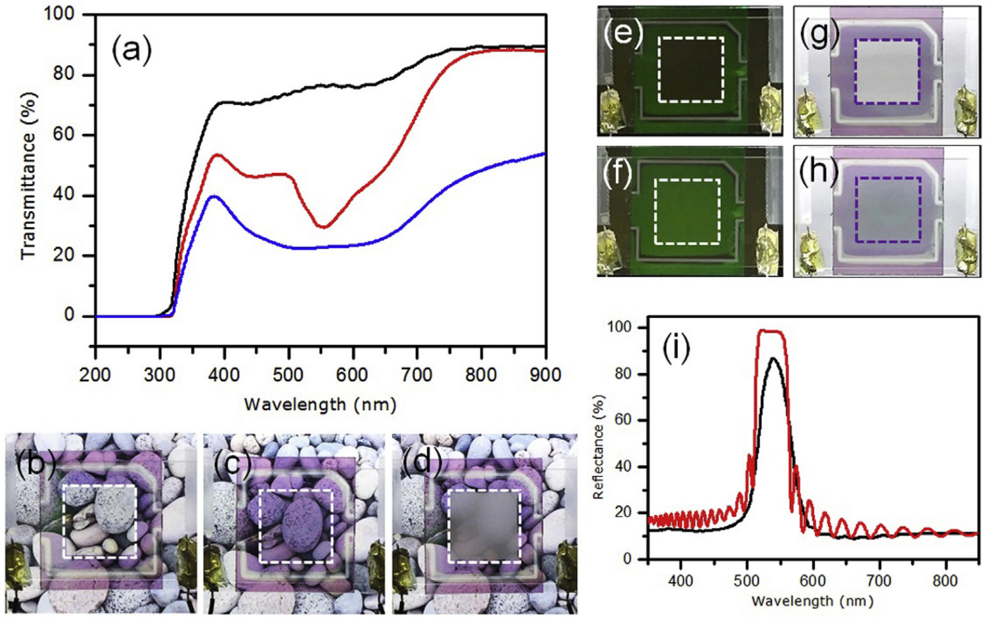


**Fig. 2.** Transmissive and reflective spectral characteristics of the CLC **Cell-2** (5.0 μm cell gap with 1.0 wt% dichroic dye): (a) UV–vis transmission spectra for the homeotropic (black), planar (red), and focal conic (blue) states; (b)/(c)/(d) macroscopic cell images for the transmitting homeotropic, reflective planar, and scattering focal conic states; the cell images of voltage-on homeotropic and voltage-off planar states on (e)/(f) black and ((g)/(h) white backgrounds; (i) circularly polarized reflectance of the cells with (black, **Cell-2**) and without (red, **Cell-1**) dichroic dye. The active switching area (15 x 15 mm) was marked by the dotted square. The applied voltage for black curve in (a), (b), and (g) was 12.0 $V_{pp}$. (For interpretation of the references to color in this figure legend, the reader is referred to the Web version of this article.)

conic configuration if the voltage is gradually decreased. The focal conic state is metastable and slowly transits to the planar states [18].

The macroscopic cell images in Fig. 1(b), 1(c), and 1(d) shows the corresponding optical states. The switchable active area was marked by the dotted square. Such transparent homeotropic, reflective planar, and scattering focal conic states were confirmed by the polarized optical microscopic images as shown in Fig. 1(e), 1(f), and 1(g). These electrically switchable and optically distinct states were usefully employed for information display applications [18].

However, they are unsuitable for switchable window applications due to a poor screening state, especially for a privacy window. As observed in Fig. 1(c) and (d)), the objects are easily seen through the window. Fig. 1(c) appears to be a tinted glass and 1(d) shows milky translucent state. Both states shows insufficient blocking state. In case of Fig. 1(c), a planar reflective state looks effective on the black background as in Fig. 1(i), together with the decent transparent voltage-on state in Fig. 1(h). On the white background, however, the voltage-off planar state exhibits undesirable shielding effect (Fig. 1(k)). This can be

explained by the transmittance curve (red in Fig. 1(a)). The CLC cell is transparent in the visible range, except for the green reflective band near 536 nm. In addition, the CLC film selectively reflects the green wavelengths depending on the handedness of circularly polarized light. Consequently, 50% of green light was reflected and it resulted in transmitting the other 50%. As a result, only ~16% of visible light was screened and thus exhibited a tinted complementary color of green as in Fig. 1(c).

Therefore, the conventional CLC film (**Cell-1**) was not suitable for a switchable window application although it provided an effective electrical switching. It requires much enhanced shielding efficiency. To solve the problem, we took two different approaches. One was adding light absorbing dyes to shield the transmitting light from an opposite side and the other was increasing a thickness of the CLC film.

For the first approach, the same kind of CLC cell (**Cell-2**) was prepared using dichroic-dye-doped cholesteric mixture (1.0 wt% dye S428 and 5.0 μm cell gap). Fig. 2 shows the transmissive and reflective spectral characteristics, observed from the **Cell-2**. Transmittance of the three switchable states was decreased to some extent. However, the voltage-off states still exhibited insufficient shielding for transmitting light. The average transmittance was ~75%, ~45%, and ~30% in the visible range, corresponding to the homeotropic, planar, and focal conic states, respectively. The differences in transmittance were approximately $\Delta T_p$ ~30 and $\Delta T_f$ ~45. The poor shielding states were clearly noticed from the macroscopic photographs as shown in Fig. 2(b), 2(c), and 2(d). Although it showed an excellent transparent state under 12.0 $V_{pp}$, both the turn-off states displayed slightly darker tinted and translucent states. Fig. 2(e)–2(h) demonstrate the origin of deficiency in shielding. As observed in the **Cell-1**, it showed good on- and off-state on the black background while it performed poor on the white background. Fig. 2(i) presents circularly polarized reflectance of the CLC cells with (black curve) and without (red curve) doping dichroic dye. Because the CLC mixture was composed of a left-handed helix, the film selectively reflected left circularly polarized light. The reduced reflectance in the black curve was attributed to the absorption of doped dyes. It was obvious that the poor shielding performance was caused by the transmitted light and doped dichroic dye was not enough to effectively absorb the transmitting light.

For the second approach, we increased the thickness of CLC films to enhance both reflection and scattering. The CLC cell (**Cell-3**) was prepared using cholesteric mixture with no use of dichroic-dye. Simply the cell gap was increased from 5.0 μm to 20.0 μm. Fig. 3 presents the transmissive and reflective characteristics of the **Cell-3**. Compared to the **Cell-1**, the transmittance of three switchable states was decreased simply by increasing a thickness of the CLC film. The average transmittance was ~80%, ~30%, and ~25% in the visible range, corresponding to the homeotropic, planar, and focal conic states, respectively. The transmittance differences between on-off states were $\Delta T_p$ ~50 and $\Delta T_f$ ~55. The cell showed a good transparency for the voltage-on homeotropic state (black curve at 83.0 $V_{pp}$). For the focal conic state, light scattering was significantly increased, resulting in a reduction of transmitted light intensity (blue curve). However, the most significant decrease was observed from the green reflective state (red curve).

The molecular configurations of CLC film were confirmed by POM textures. Fig. 3(b) and (d) correspond to the homeotropic and focal conic states, similar to Fig. 1(e) and (g). For Fig. 3(c), however, the green reflecting planar state showed very distinct texture from the corresponding Fig. 1(f). The cell image for a voltage-off planar state in Fig. 3(f) was also quite different from Fig. 1(c)/2(c) while the voltage-on (83.0 $V_{pp}$) transparent state showed a similar characteristics as observed in Figs. 3(e) and 1(b)/2(b). For the same off-state under different environments, Fig. 3(f) exhibited strong scattering together with slightly colored reflection while Fig. 3(h) presented strong reflection with some degree of scatterings. As seen in Fig. 3(f) and (h), the cell showed milky scattering with a slight magenta, and green reflection with some turbidity. These observations confirmed that the texture in Fig. 3(c) is the mixed state of scattering focal conic and reflecting planar states. Therefore, it resulted in a significantly improved opaque state while maintaining a good transparent state (black and red curves in Fig. 3(a)). Due to better shielding efficiency, compared to the **Cell-1**, the enhanced on-off contrast was also evidenced by the cell images in Fig. 3(e)/3(g)/3(i) and 3(f)/3(h)/3(j).

Although the addition of scattering effect for shielding improved the on-off contrast ($\Delta T_p$ ~ 50), the off-state was still insufficient for a switchable window application due to forward scatterings of transmitting light as seen in Fig. 3(f) and (j) on a bright environment. To solve the problem, the adjustment was made by adding light-absorbing dichroic dyes to the thicker CLC film, which was a combination of the first and second approaches discussed above. The CLC **Cell-4** was fabricated, by doping 1.0 wt% S428 to the CLC mixture, with 20.0 μm cell gap.

Fig. 4 shows a spectral performance of the on-off switching states. The transmittances of three switching states were significantly reduced in the visible range, as shown in Fig. 4(a). The transmitting homeotropic state (80.0 $V_{pp}$) showed ~ 40% transmittance on average. Although dye molecules were aligned parallel to a LC director, the increased film thickness lowered transparency. The transmittance for both planar and focal conic states was reduced to 1–2% in a visible range. The dye-doped CLC film with the increased thickness enhanced a shielding efficiency dramatically. In addition to the thickness, the planar and focal conic configurations promoted the absorption by dye-molecules, lying planar to the cell surface. Both scattering and absorption effectively screened the transmitting light.

The on-off states on a dark background were shown in Fig. 4(b) and (c). The green reflection was more distinct than those for the previous cells, shown in Figs. 1(i), 2(f), and 3(h). Because the dye absorbed backward scatterings of a front light, the reflected color became more vivid as shown in Fig. 4(c). The cell images in Fig. 4(d)/4(e)/4(f), show voltage-on homeotropic, voltage-off planar and focal conic states on a white background. Due to a dichroic absorption of the dyes, the focal conic state exhibited a darker gray, rather than a milky white. The green reflective state with the combined planar and focal conic configurations revealed very effective shielding for a bright environment. Fig. 4(e) demonstrate an excellent colored-opaque state, compared to the previous cells shown in Figs. 1(k), 2(h), and 3(j). Fig. 4(g), 4(h), and 4(i) also shows three switchable states on a casual environment, which correspond to the transmitting homeotropic, reflective planar, and scattering focal conic configurations, respectively. Although both $\Delta T_p$ ~40 and $\Delta T_f$ ~40 were decreased compared to the **Cell-3**, the visibility of on-off switching states was drastically enhanced by adjusting the mixture and film thickness of a short pitch cholesteric liquid crystal.

To comprehend the results, we present schematic illustrations for the molecular configurations and the corresponding optical characteristics of the three switchable states. Fig. 5 compares the dichroic-dye-doped short pitch CLC films with 5.0 μm and 20.0 μm thickness. First, for the thin film CLC, two voltage-off states were switched from the transparent homeotropic state with a good transparency. The black dye S428 was doped to absorb transmitting and scattered lights as illustrated in Fig. 5(a) and (d). The molecular configurations were confirmed by POM images (Fig. 5(b) and (e)). However, the corresponding optical images of the cell showed poor shielding states as shown in Fig. 5(c) and (f). Although the transmittance was slightly decreased from the conventional CLC film with no doped-dyes, it wasn't enough to effectively screen transmitting and scattered lights.

In case of dichroic-dye-doped CLC film with 20 μm thickness, however, it exhibited dramatically enhanced shielding efficiency. Fig. 5(g) corresponds to the molecular configuration of a voltage-on homeotropic state, confirmed by the POM image in Fig. 5(h). Because dichroic dye was aligned parallel to the incident light, the absorption by the dye was minimized. Therefore, the cell showed a good transparent state (Fig. 5(i)). When the applied voltage was gradually turned off, the

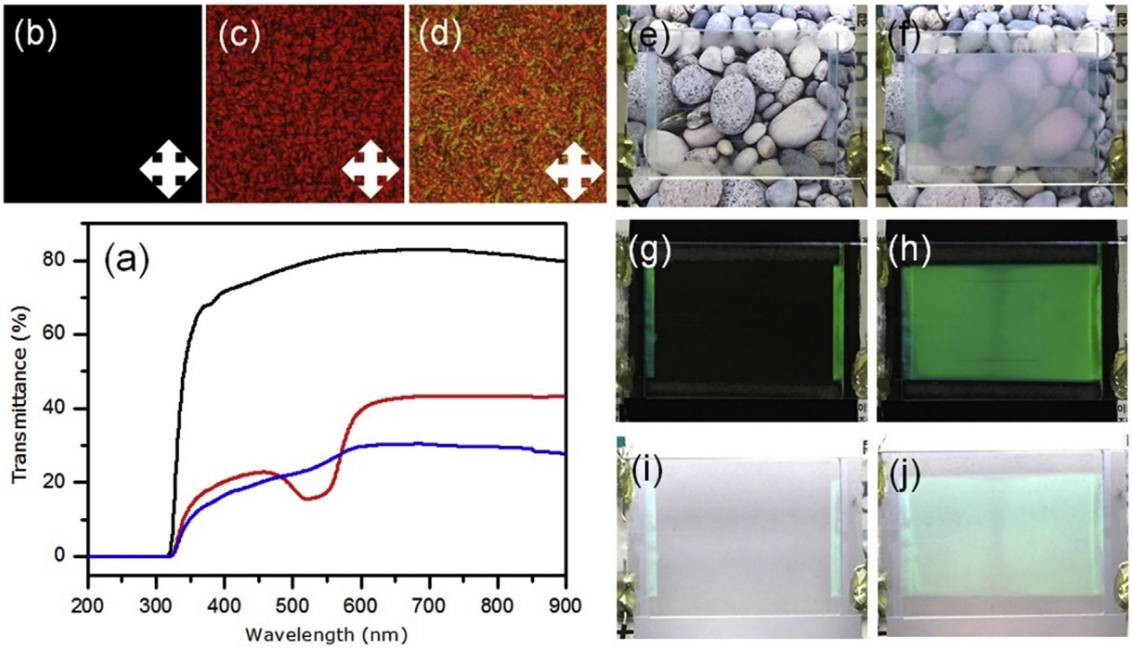


**Fig. 3.** Transmissive and reflective spectral characteristics of the CLC **Cell-3** (20.0 μm cell gap with no dichroic dye): (a) UV–vis transmission spectra for the homeotropic (black), planar (red), and focal conic (blue) states; POM images for the transmitting homeotropic (b), reflective planar (c), and scattering focal conic states (d); (e)/(f) macroscopic cell images of voltage-on homeotropic (83.0 $V_{pp}$) and voltage-off planar states; the cell images of voltage-on homeotropic and voltage-off planar states on (g)/(h) black and (i)/(j) white backgrounds. The active switching area was 42 x 24 mm. The crossed arrows represent polarizer and analyzer for the POM images. The applied voltage for black curve in (a), (b), (e), and (i) was 83.0 $V_{pp}$. (For interpretation of the references to color in this figure legend, the reader is referred to the Web version of this article.)

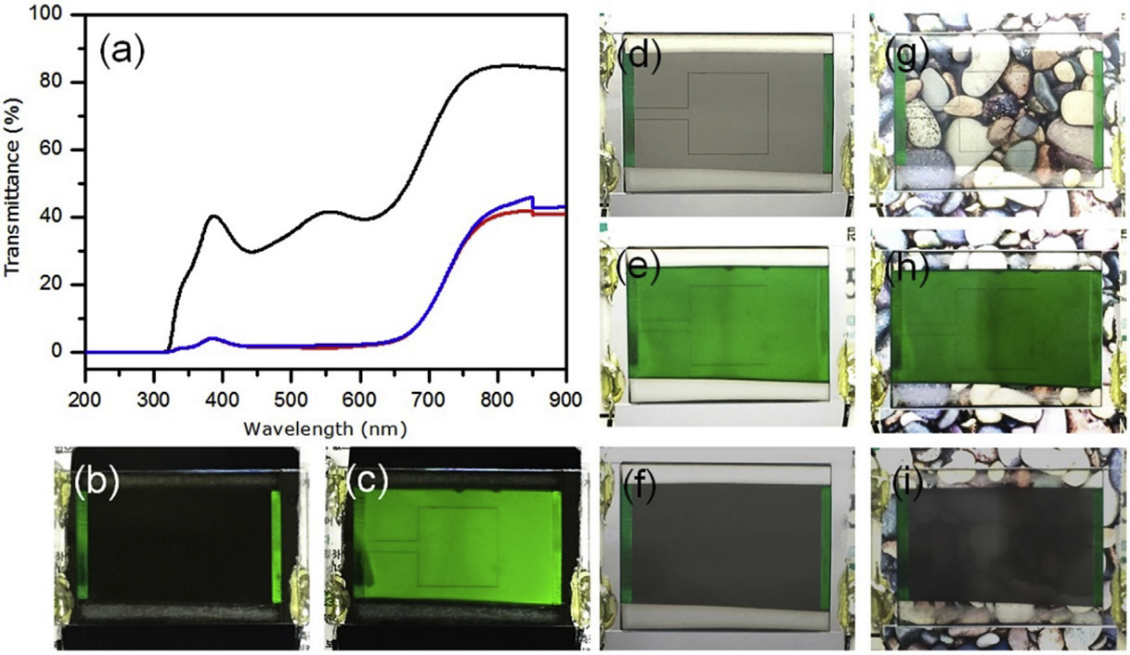


**Fig. 4.** Transmissive and reflective spectral characteristics of the CLC **Cell-4** (20.0 μm cell gap with 1.0 wt% dichroic dye S428): (a) UV–vis transmission spectra for the homeotropic (black), planar (red), and focal conic (blue) states; (b)/(c) macroscopic cell images of voltage-on transparent and voltage-off reflective states on a black background; the cell images of voltage-on homeotropic, voltage-off planar, and voltage-off focal conic states on the (d)/(e)/(f) white background and (g)/(h)/(i) casual circumstance. The active switching area was 42 x 24 mm. The applied voltage for black curve in (a), (b), (d), and (g) was 80.0 $V_{pp}$. (For interpretation of the references to color in this figure legend, the reader is referred to the Web version of this article.)

CLC cell was switched to the scattering focal conic state as illustrated in Fig. 5(j) and confirmed by Fig. 5(k). The randomized cholesteric domains with a larger film thickness increased the scattering of an incident light as denoted by the slanted arrows. In this case, both the forward and backward scattered light were absorbed by the dye molecules. The absorption reduced a scattered light intensity on both directions. Therefore, the CLC cell effectively shielded transmitting light and appeared to be a dark gray as shown in Fig. 5*l*.

When the applied voltage was abruptly turned off, the CLC cell was switched from the homeotropic to the reflective state as presented in

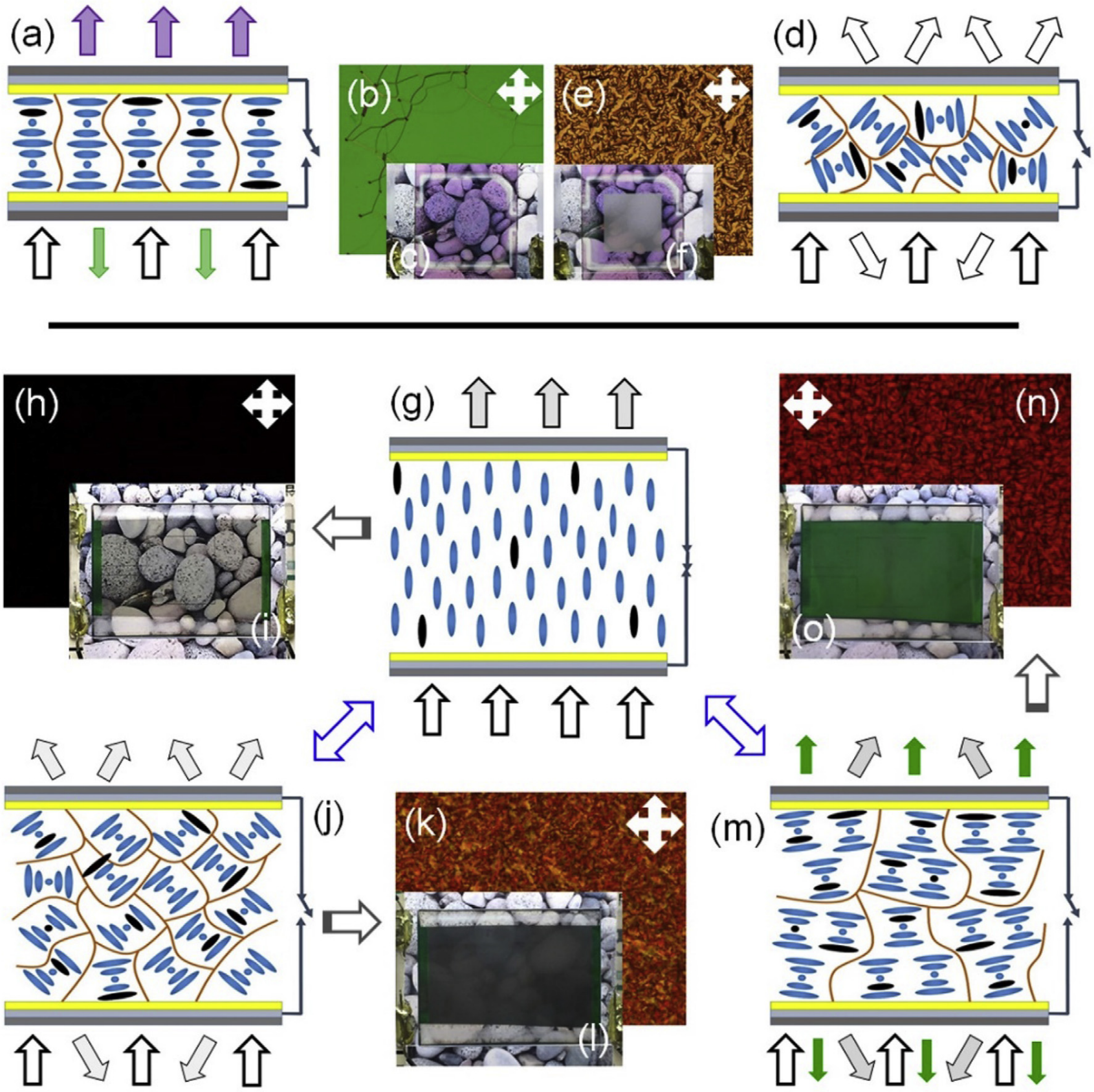


**Fig. 5.** Schematic representation of the molecular configurations and corresponding optical characteristics of the dichroic-dye-doped short pitch cholesteric LCs: (a–f) dichroic-dye-doped CLC film with 5.0 μm cell gap, the voltage-off (a)/(b)/(c) planar and (d)/(e)/(f) focal conic configurations, corresponding POM textures, and cell images; (g–o) dichroic-dye-doped CLC film with 20.0 μm cell gap, (g)/(h)/(i) the voltage-on transmitting homeotropic, (j)/(k)/(l) the voltage-off scattering focal conic, (m)/(n)/(o) the voltage-off planar configurations, and the corresponding microscopic polarized optical and macroscopic optical image of the CLC cell. The blue and black ellipses represent LC and dichroic dye molecules, respectively. The crossed arrows indicate polarizer and analyzer. The double-ended arrow (blue) designates a switching route. The arrows in the cell denote transmitted, scattered, reflected, and incident lights. (For interpretation of the references to color in this figure legend, the reader is referred to the Web version of this article.)

Fig. 5m/5n/5o. In addition to the green reflection, the POM texture (Fig. 5(n)) and the cell image with a high turbidity (Fig. 5(o)) indicated a mixed state of molecular configuration. As depicted in Fig. 5(m), multiple domains of planar aligned CLC selectively reflect 50% of a green light (green arrows) and the domain boundaries scatter a white light (gray arrows). In this case, the dichroic dye plays an important role to absorb both transmitting and scattered lights. It effectively absorbs transmitting light in visible wavelengths. It also absorbs forward and backward scattered lights. As a result, the CLC cell showed a green reflection in both dark and bright environments.

For the dye-doped thick CLC cell, compared to the conventional one, both the voltage-off states showed excellent shielding for a bright environment. The focal conic state in Fig. 5(j) appeared to be a dark gray (Fig. 5*l*), rather than a milky white. The multi-domain planar state in Fig. 5(m) exhibited a greatly improved opaque state with a green reflection. A reflected color can be turned to various colors by optimizing a cholesteric pitch with varied chiral concentrations. In Fig. 5, it was comprehended that the dichroic-dye-doped CLC with an increased film thickness has a great advantage to enhance a shielding efficiency. Both the voltage-off states provided outstanding quality for the blocking state of a switchable smart window.

The dye-doping ratio was optimized by considering absorptions for both the transparent and shielding states. When it was higher than 1.0%, the transparent state became poor. When it was too low, the shielding efficiency wasn't enough for a privacy window application. For the dye-doped mixture, it seemed that dye caused a slight increase in $\Delta\varepsilon$ of the mixture. It was understood by observing a slightly decreased threshold voltage for a homeotropic state of the dye-doped cells. For both thin and thick cells, the threshold voltages were decreased by ~1 and ~3 $V_{pp}$, respectively.

## 4. Conclusions

The short pitch CLC films were investigated for the application of electrically switchable smart windows. The conventional CLC films, used for display applications, showed insufficient optical performance for a switchable window application, mainly due to a poor shielding efficiency. We made two approaches to enhance a shielding performance by doping dichroic dyes and increasing a film thickness. Although each adjustment made insufficient progress, the combined efforts resulted in a great synergetic effect for the enhancement of screening states. The increased film thickness produced not only a strong scattering of the focal conic state but also promoted reflective multi-domain planar state. The modified planar configuration exhibited turbid scattering state with a specific colored reflection. In addition, both transmitting light and backward/forward scattered light were efficiently absorbed in the voltage-off states by doped dichroic dyes while the CLC film showed a good transmitting homeotropic state under applied voltage. As a result, spectral properties of the voltage-off screening states were dramatically improved for the application of electrically switchable window applications. If the cholesteric pitch is tuned for visible wavelengths, it can be usefully employed for a switchable privacy window with a desirable color. It may also be applied for switchable ultraviolet and heat windows when the pitch of CLC and dichroic absorption of dye are adjusted in the ultraviolet and near infrared range, respectively.

## Acknowledgment

This work was supported by the Korea Agency for Infrastructure Technology Advancement (Grant No. 19POQW-B152372-01) and BK21 Plus Program through the National Research Foundation of Korea funded by the Ministry of Education.

## Appendix A. Supplementary data

Supplementary data to this article can be found online at https://doi.org/10.1016/j.dyepig.2019.03.045.

## References

[1] Huang CY, Fu KY, Lo KY, Tsai MS. Bistable transflective cholesteric light shutters. Optic Express 2003;11(6):560–5.
[2] Du F, Lu YQ, Ren HW, Gauza S, Wu ST. Polymer-stabilized cholesteric liquid crystal for polarization-independent variable optical attenuator. Jpn J Appl Phys 2004;43(10):7083–6.
[3] Iwamoto M, Wu C-X, Zhong-can O-Y. Separation of chiral phases by compression: kinetic localization of the enantiomers in a monolayer of racemic amphiphiles viewed as mixing cholesteric liquid crystals. Chem Phys Lett 1998;285(5–6):306–12.
[4] Choi H, Kim J, Nishimura S, Toyooka T, Araoka F, Ishikawa K, Wu JW, Takezoe H. Broadband cavity-mode lasing from dye-doped nematic liquid crystals sandwiched by broadband cholesteric liquid crystal Bragg reflectors. Adv Mater 2010;22(24):2680–4.
[5] Huang Y, Sun Q, Zhang S. Widely tunable optical filter with variable bandwidth based on spatially distributed cholesteric liquid crystal. Opt Eng 2013;52. 044003-1 ~ 5.
[6] Wu ST, Fuh AYG, Ho SJ, Li MS. Bichromatic tuning of reflection bands in integrated CLC reflectors for optical switches, gates, and logic. Appl Phys B 2015;118:379–85.
[7] Fuh AYG, Ho SJ, Wu ST, Li MS. Optical filter with tunable wavelength and bandwidth based on phototunable cholesteric liquid crystals. Appl Opt 2014;53:1658–62.
[8] Yang DK, Doane JW, Yaniv Z, Glasser J. Cholesteric reflective display: drive scheme and contrast. Appl Phys Lett 1994;64:1905–7.
[9] Yang DK, West JL, Chien LC, Doane JW. Control of reflectivity and bistability in displays using cholesteric liquid crystals. J Appl Phys 1994;76:1331–3.
[10] Fuh AYG, Lin CH, Huang CY. Dynamic pattern formation and beam-steering characteristics of cholesteric gratings. Jpn J Appl Phys 2002;41:211–8.
[11] Lin CH, Fuh AYG, Mo TS, Huang CY. Polymer stabilized reflective fingerprint cholesteric texture grating. Jpn J Appl Phys 2002;41:7441–6.
[12] Inoue Y, Yoshida H, Inoue K, Shiozaki Y, Kubo H, Fujii A, Ozaki M. Tunable lasing from a cholesteric liquid crystal film embedded with a liquid crystal nanopore network. Adv Mater 2011;23:5498–501.
[13] Hu JW, Chen TA, Jeng SC. Broadband cholesteric liquid crystal devices with poly (N-vinyl carbazole) microstructures. Liq Cryst 2015;42:52–6.
[14] Guo J, Cao H, Zhang D, Liu F, Pan G, Zhao D, He W, Yang H. Polymer-stabilized liquid crystal films reflecting both right- and left-circularly polarized light. Appl Phys Lett 2008;93. 201901-1 ~ 3.
[15] Zhao Y, Zhang L, He Z, Chen G, Wang D, Zhang H, Yang H. Photoinduced polymer-stabilized chiral nematic liquid crystal films reflecting both right- and left-circularly polarized light. Liq Cryst 2015;42:1120–3.
[16] Kumar R, Raina KK. Electrically modulated fluorescence in optically active polymer stabilised cholesteric liquid crystal shutter. Liq Cryst 2014;41(2):228–33.
[17] Kumar R, Raina KK. Enhanced ordering in polymer stabilized ferroelectric liquid crystal guest–host composites: evidence by polarised fluorescence spectroscopy. Liq Cryst 2014;41(5):694–700.
[18] Wu ST, Yang DK. Reflective liquid crystal displays. Wiley; 2001.
[19] Yang DK, Huang XY, Zhu YM. Bistable cholesteric reflective displays: materials and drive schemes. Annu Rev Mater Sci 1997;27:117–46.
[20] Oh SW, Baek JM, Heo J, Yoon TH. Dye-doped cholesteric liquid crystal light shutter with a polymer dispersed liquid crystal film. Dyes Pigments 2016;134:36–40.
[21] Kim JH, Huh JW, Oh SW, Ji SM, Jo YS, Yu BH, Yoon TH. Bistable switching between homeotropic and focal-conic states in an ion-doped chiral nematic liquid crystal cell. Optics Express 2017;25:29180–8.
[22] Oh SW, Baek JM, Kim SH, Yoon TH. Optical and electrical switching of cholesteric liquid crystals containing azo dye. RSC Adv 2017;7:19497–501.
[23] Yu BH, Huh JW, Heo J, Yoon TH. Simultaneous control of haze and transmittance using a dye-doped cholesteric liquid crystal cell. Liq Cryst 2015;42:1460–4.
[24] Ke Y, Zhou C, Zhou Y, Wang S, Chan SH, Long Y. Emerging thermal-responsive materials and integrated techniques targeting the energy-efficient smart window application. Adv Funct Mater 2018;28(1–18):1800113.
[25] Xing H, Wang X, Xu J, Wei J, Guo J. Polymer network microstructures and electro-optical properties of a pressure-sensitive cholesteric liquid crystal device. RSC Adv 2013;3:17822–8.
