# Supplementary material for: Advanced liquid crystal-based switchable optical devices for light protection applications: principles and strategies
Source: Light Sci Appl. 2023 Jan 3;12:11. doi: 10.1038/s41377-022-01032-y (PMC9807646; doi:10.1038/s41377-022-01032-y)
Supplement: Supplementary file 15 — Fig 17 copyright promotion [file 41377_2022_1032_MOESM15_ESM.pdf]

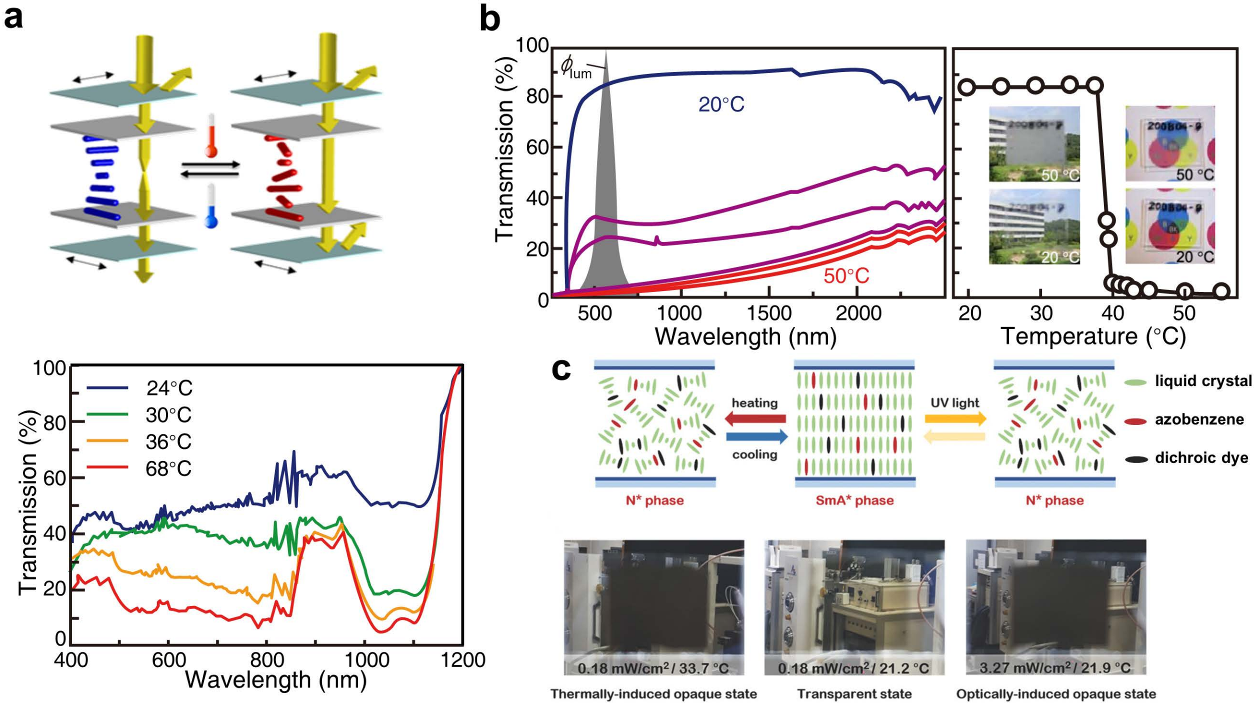

a
24°C
30°C
36°C
68°C
Transmission (%)
Wavelength (nm)
b
ϕ_{lum}
20°C
50°C
Transmission (%)
Wavelength (nm)
Temperature (°C)
50 °C
20 °C
c
heating
cooling
UV light
liquid crystal
azobenzene
dichroic dye
N* phase
SmA* phase
N* phase
0.18 mW/cm² / 33.7 °C
0.18 mW/cm² / 21.2 °C
3.27 mW/cm² / 21.9 °C
Thermally-induced opaque state
Transparent state
Optically-induced opaque state

JOHN WILEY AND SONS LICENSE TERMS AND CONDITIONS

Sep 19, 2022

This Agreement between Harbin Institute of Technology -- Ruicong Zhang ("You") and John Wiley and Sons ("John Wiley and Sons") consists of your license details and the terms and conditions provided by John Wiley and Sons and Copyright Clearance Center.

License Number	5392420711721
License date	Sep 19, 2022
Licensed Content Publisher	John Wiley and Sons
Licensed Content Publication	Journal of Polymer Science
Licensed Content Title	'Smart' light-reflective windows based on temperature responsive twisted nematic liquid crystal polymers
Licensed Content Author	Augustinus J. J. Kragt, Roel C. G. M. Loonen, Dirk J. Broer, et al
Licensed Content Date	Feb 22, 2021
Licensed Content Volume	59
Licensed Content Issue	12
Licensed Content Pages	7
Type of Use	Journal/Magazine
Requestor type	University/Academic
Is the reuse sponsored by or associated with a pharmaceutical or medical products company?	no
Format	Print and electronic
Portion	Figure/table
Number of figures/tables	2
Will you be translating?	No
Circulation	200 - 499
Title of new article	Advanced liquid crystal-based switchable optical devices for light protection applications: principles and strategies
Lead author	Ruicong Zhang, Zhibo Zhang, Jiecai Han, Lei Yang, Jiajun li, Zicheng Song Tianyu Wang, Jiaqi Zhu
Title of targeted journal	Light: Science & Applications
Publisher	Springer Nature
Expected publication date	Nov 2022
Portions	Figure 1, Figure 3
Requestor Location	Harbin Institute of Technology<br>No. 92, Xidazhi Street, Nangang District<br><br>Harbin, 150080<br>China<br>Attn: Harbin Institute of Technology
Publisher Tax ID	EU826007151
Total	**0.00 USD**
Terms and Conditions	

TERMS AND CONDITIONS

This copyrighted material is owned by or exclusively licensed to John Wiley & Sons, Inc. or one of its group companies (each a"Wiley Company") or handled on behalf of a society with which a Wiley Company has exclusive publishing rights in relation to a particular work (collectively "WILEY"). By clicking "accept" in connection with completing this licensing transaction, you agree

that the following terms and conditions apply to this transaction (along with the billing and payment terms and conditions established by the Copyright Clearance Center Inc., ("CCC's Billing and Payment terms and conditions"), at the time that you opened your RightsLink account (these are available at any time at http://myaccount.copyright.com).

Terms and Conditions

- The materials you have requested permission to reproduce or reuse (the "Wiley Materials") are protected by copyright.
- You are hereby granted a personal, non-exclusive, non-sub licensable (on a stand-alone basis), non-transferable, worldwide, limited license to reproduce the Wiley Materials for the purpose specified in the licensing process. This license, **and any CONTENT (PDF or image file) purchased as part of your order,** is for a one-time use only and limited to any maximum distribution number specified in the license. The first instance of republication or reuse granted by this license must be completed within two years of the date of the grant of this license (although copies prepared before the end date may be distributed thereafter). The Wiley Materials shall not be used in any other manner or for any other purpose, beyond what is granted in the license. Permission is granted subject to an appropriate acknowledgement given to the author, title of the material/book/journal and the publisher. You shall also duplicate the copyright notice that appears in the Wiley publication in your use of the Wiley Material. Permission is also granted on the understanding that nowhere in the text is a previously published source acknowledged for all or part of this Wiley Material. Any third party content is expressly excluded from this permission.
- With respect to the Wiley Materials, all rights are reserved. Except as expressly granted by the terms of the license, no part of the Wiley Materials may be copied, modified, adapted (except for minor reformatting required by the new Publication), translated, reproduced, transferred or distributed, in any form or by any means, and no derivative works may be made based on the Wiley Materials without the prior permission of the respective copyright owner.**For STM Signatory Publishers clearing permission under the terms of the STM Permissions Guidelines only, the terms of the license are extended to include subsequent editions and for editions in other languages, provided such editions are for the work as a whole in situ and does not involve the separate exploitation of the permitted figures or extracts,** You may not alter, remove or suppress in any manner any copyright, trademark or other notices displayed by the Wiley Materials. You may not license, rent, sell, loan, lease, pledge, offer as security, transfer or assign the Wiley Materials on a stand-alone basis, or any of the rights granted to you hereunder to any other person.
- The Wiley Materials and all of the intellectual property rights therein shall at all times remain the exclusive property of John Wiley & Sons Inc, the Wiley Companies, or their respective licensors, and your interest therein is only that of having possession of and the right to reproduce the Wiley Materials pursuant to Section 2 herein during the continuance of this Agreement. You agree that you own no right, title or interest in or to the Wiley Materials or any of the intellectual property rights therein. You shall have no rights hereunder other than the license as provided for above in Section 2. No right, license or interest to any trademark, trade name, service mark or other branding ("Marks") of WILEY or its licensors is granted hereunder, and you agree that you shall not assert any such right, license or interest with respect thereto
- NEITHER WILEY NOR ITS LICENSORS MAKES ANY WARRANTY OR REPRESENTATION OF ANY KIND TO YOU OR ANY THIRD PARTY, EXPRESS, IMPLIED OR STATUTORY, WITH RESPECT TO THE MATERIALS OR THE ACCURACY OF ANY INFORMATION CONTAINED IN THE MATERIALS, INCLUDING, WITHOUT LIMITATION, ANY IMPLIED WARRANTY OF MERCHANTABILITY, ACCURACY, SATISFACTORY QUALITY, FITNESS FOR A PARTICULAR PURPOSE, USABILITY, INTEGRATION OR NON-INFRINGEMENT AND ALL SUCH WARRANTIES ARE HEREBY EXCLUDED BY WILEY AND ITS LICENSORS AND WAIVED BY YOU.
- WILEY shall have the right to terminate this Agreement immediately upon breach of this Agreement by you.
- You shall indemnify, defend and hold harmless WILEY, its Licensors and their respective directors, officers, agents and employees, from and against any actual or threatened claims, demands, causes of action or proceedings arising from any breach of this Agreement by you.
- IN NO EVENT SHALL WILEY OR ITS LICENSORS BE LIABLE TO YOU OR ANY OTHER PARTY OR ANY OTHER PERSON OR ENTITY FOR ANY SPECIAL, CONSEQUENTIAL, INCIDENTAL, INDIRECT, EXEMPLARY OR PUNITIVE DAMAGES, HOWEVER CAUSED, ARISING OUT OF OR IN CONNECTION WITH THE DOWNLOADING, PROVISIONING, VIEWING OR USE OF THE MATERIALS REGARDLESS OF THE FORM OF ACTION, WHETHER FOR BREACH OF CONTRACT, BREACH OF WARRANTY, TORT, NEGLIGENCE, INFRINGEMENT OR OTHERWISE (INCLUDING, WITHOUT LIMITATION, DAMAGES BASED ON LOSS OF PROFITS, DATA, FILES, USE, BUSINESS OPPORTUNITY OR CLAIMS OF THIRD PARTIES), AND WHETHER OR NOT THE PARTY HAS BEEN ADVISED OF THE POSSIBILITY OF SUCH DAMAGES. THIS LIMITATION SHALL APPLY NOTWITHSTANDING ANY FAILURE OF ESSENTIAL PURPOSE OF ANY LIMITED REMEDY PROVIDED HEREIN.
- Should any provision of this Agreement be held by a court of competent jurisdiction to be illegal, invalid, or unenforceable, that provision shall be deemed amended to achieve as nearly as possible the same economic effect as the original provision, and the legality, validity and enforceability of the remaining provisions of this Agreement shall not

be affected or impaired thereby.

- The failure of either party to enforce any term or condition of this Agreement shall not constitute a waiver of either party's right to enforce each and every term and condition of this Agreement. No breach under this agreement shall be deemed waived or excused by either party unless such waiver or consent is in writing signed by the party granting such waiver or consent. The waiver by or consent of a party to a breach of any provision of this Agreement shall not operate or be construed as a waiver of or consent to any other or subsequent breach by such other party.

- This Agreement may not be assigned (including by operation of law or otherwise) by you without WILEY's prior written consent.

- Any fee required for this permission shall be non-refundable after thirty (30) days from receipt by the CCC.

- These terms and conditions together with CCC's Billing and Payment terms and conditions (which are incorporated herein) form the entire agreement between you and WILEY concerning this licensing transaction and (in the absence of fraud) supersedes all prior agreements and representations of the parties, oral or written. This Agreement may not be amended except in writing signed by both parties. This Agreement shall be binding upon and inure to the benefit of the parties' successors, legal representatives, and authorized assigns.

- In the event of any conflict between your obligations established by these terms and conditions and those established by CCC's Billing and Payment terms and conditions, these terms and conditions shall prevail.

- WILEY expressly reserves all rights not specifically granted in the combination of (i) the license details provided by you and accepted in the course of this licensing transaction, (ii) these terms and conditions and (iii) CCC's Billing and Payment terms and conditions.

- This Agreement will be void if the Type of Use, Format, Circulation, or Requestor Type was misrepresented during the licensing process.

- This Agreement shall be governed by and construed in accordance with the laws of the State of New York, USA, without regards to such state's conflict of law rules. Any legal action, suit or proceeding arising out of or relating to these Terms and Conditions or the breach thereof shall be instituted in a court of competent jurisdiction in New York County in the State of New York in the United States of America and each party hereby consents and submits to the personal jurisdiction of such court, waives any objection to venue in such court and consents to service of process by registered or certified mail, return receipt requested, at the last known address of such party.

WILEY OPEN ACCESS TERMS AND CONDITIONS
Wiley Publishes Open Access Articles in fully Open Access Journals and in Subscription journals offering Online Open. Although most of the fully Open Access journals publish open access articles under the terms of the Creative Commons Attribution (CC BY) License only, the subscription journals and a few of the Open Access Journals offer a choice of Creative Commons Licenses. The license type is clearly identified on the article.
The Creative Commons Attribution License
The Creative Commons Attribution License (CC-BY) allows users to copy, distribute and transmit an article, adapt the article and make commercial use of the article. The CC-BY license permits commercial and non-
Creative Commons Attribution Non-Commercial License
The Creative Commons Attribution Non-Commercial (CC-BY-NC)License permits use, distribution and reproduction in any medium, provided the original work is properly cited and is not used for commercial purposes.(see below)

Creative Commons Attribution-Non-Commercial-NoDerivs License
The Creative Commons Attribution Non-Commercial-NoDerivs License (CC-BY-NC-ND) permits use, distribution and reproduction in any medium, provided the original work is properly cited, is not used for commercial purposes and no modifications or adaptations are made. (see below)
Use by commercial "for-profit" organizations
Use of Wiley Open Access articles for commercial, promotional, or marketing purposes requires further explicit permission from Wiley and will be subject to a fee.
Further details can be found on Wiley Online Library http://olabout.wiley.com/WileyCDA/Section/id-410895.html

Other Terms and Conditions:

v1.10 Last updated September 2015

Questions? customercare@copyright.com or +1-855-239-3415 (toll free in the US) or +1-978-646-2777.

Received: 7 January 2021 | Revised: 15 January 2021 | Accepted: 15 January 2021
DOI: 10.1002/pol.20210008

RESEARCH ARTICLE

JOURNAL OF POLYMER SCIENCE WILEY

'Smart' light-reflective windows based on temperature responsive twisted nematic liquid crystal polymers

Augustinus J. J. Kragt[1,2,3] | **Roel C. G. M. Loonen**[4] | **Dirk J. Broer**[1,2,5] | **Michael G. Debije**[1] | **Albert P. H. J. Schenning**[1,2,5]

[1]Stimuli-responsive Functional Materials and Devices, Department of Chemical Engineering and Chemistry, Eindhoven University of Technology, Eindhoven, The Netherlands

[2]SCNU-TUE Joint Laboratory of Device Integrated Responsive Materials (DIRM), South China Normal University, Guangzhou Higher Education Mega Center, Guangzhou, China

[3]ClimAd Technology, Nijmegen, The Netherlands

[4]Unit Building Physics and Services, Department of the Built Environment, Eindhoven University of Technology, Eindhoven, Nijmegen, The Netherlands

[5]Institute for Complex Molecular Systems, Eindhoven University of Technology, Eindhoven, The Netherlands

Correspondence
Augustinus J. J. Kragt, Stimuli-responsive Functional Materials and Devices, Department of Chemical Engineering and Chemistry, Eindhoven University of Technology, Den Dolech 2, 5600 MB Eindhoven, The Netherlands.
Email: stijnkragt@climadtechnology.com

Funding information
NWO; Eindhoven University of Technology

Abstract

'Smart' windows which reversibly increase their reflectivity upon heating are attracting considerable attention as devices for maintaining comfortable indoor environmental conditions. In this work, twisted nematic semi-interpenetrating liquid crystal networks which lose their order upon heating are sandwiched between reflective linear polarizers. This 'smart' window reversibly decreases its transmission from about 50–10% over a wavelength range between 400 and 1100 nm upon heating, resulting in the window becoming darker and reflecting more light. This 'smart' window is potentially interesting for energy saving window applications where variation between privacy and visible light transparency states is required.

KEYWORDS

'smart' windows, light reflective devices, liquid crystals, semi-interpenetrating networks, temperature responsive polymers

1 | INTRODUCTION

Given the Western world's primarily indoor lifestyles, there is a rising desire to connect with the outside world via increased fenestration, as windows enhance building (and vehicle) appearances, and allow access of daylight and external views.[1] Access to daylight is an important environmental factor affecting the health and well-being of people in home- and workspace,[2,3] affecting both emotional and motivational states.[4] As a result, the development of windows with additional functionalities, including privacy states or self-regulating solar heat rejection to prevent

This is an open access article under the terms of the Creative Commons Attribution-NonCommercial License, which permits use, distribution and reproduction in any medium, provided the original work is properly cited and is not used for commercial purposes.
© 2021 The Authors. *Journal of Polymer Science* published by Wiley Periodicals LLC.

overheating of room interiors, is an emerging research field.[5–9] In particular, windows which autonomously change their transmission in response to changes in temperature are of special interest for maintaining comfortable indoor climates, while simultaneously reducing the energy loads required for artificial heating and cooling.[10–16]

These switchable windows which can alter their transmissive states in response to temperature have been labeled as 'smart', and most are based on inorganic materials.[12] Thermochromic windows rely on light absorption[17] or scattering,[6,12,18–22] forming 'dark', translucent, or reflective phases to manage the passage of light.

Organic-based thermochromic 'smart' windows have also been reported that switch between reflective and transparent states, scattering and transparent states, or from narrow- to broadband reflecting states. These 'smart' windows are generally based on polymer dispersed liquid crystals (PDLCs) or polymer stabilized liquid crystals (PSLCs) containing low molecular weight LCs able to undergo a re-arrangement upon temperature changes.[23–41]

Our previous reflective LC-based windows utilized a shift in reflective bandwidth upon heating[31] to control light transmission. In this work, we report a novel LC polymer-based 'smart' window which reversibly increases reflectivity upon heating. This reflective 'smart' window relies on a temperature-responsive noncross-linked liquid crystal elastomer (LCE) interpenetrating through a liquid crystalline network (LCN)[42–44] sandwiched between two reflective linear polarizers. The semi-interpenetrating network (semi-IPN) window is effective over a broad wavelength range, including the visible light region (Figure 1(A)). Below the nematic-to-isotropic transition temperature ($T_{N\text{-}I}$) of the twisted nematic LC, the linear polarization (LP) of the light transmitted by the first reflective polarizer is rotated by the LC host and transmitted through the second polarizer, resulting in an in theory 50% overall reflection over the wavelength regime for which the linear polarizers are operative. Above the $T_{N\text{-}I}$, the LC loses order and is unable to rotate the LP light transmitted by the first polarizer, and so the

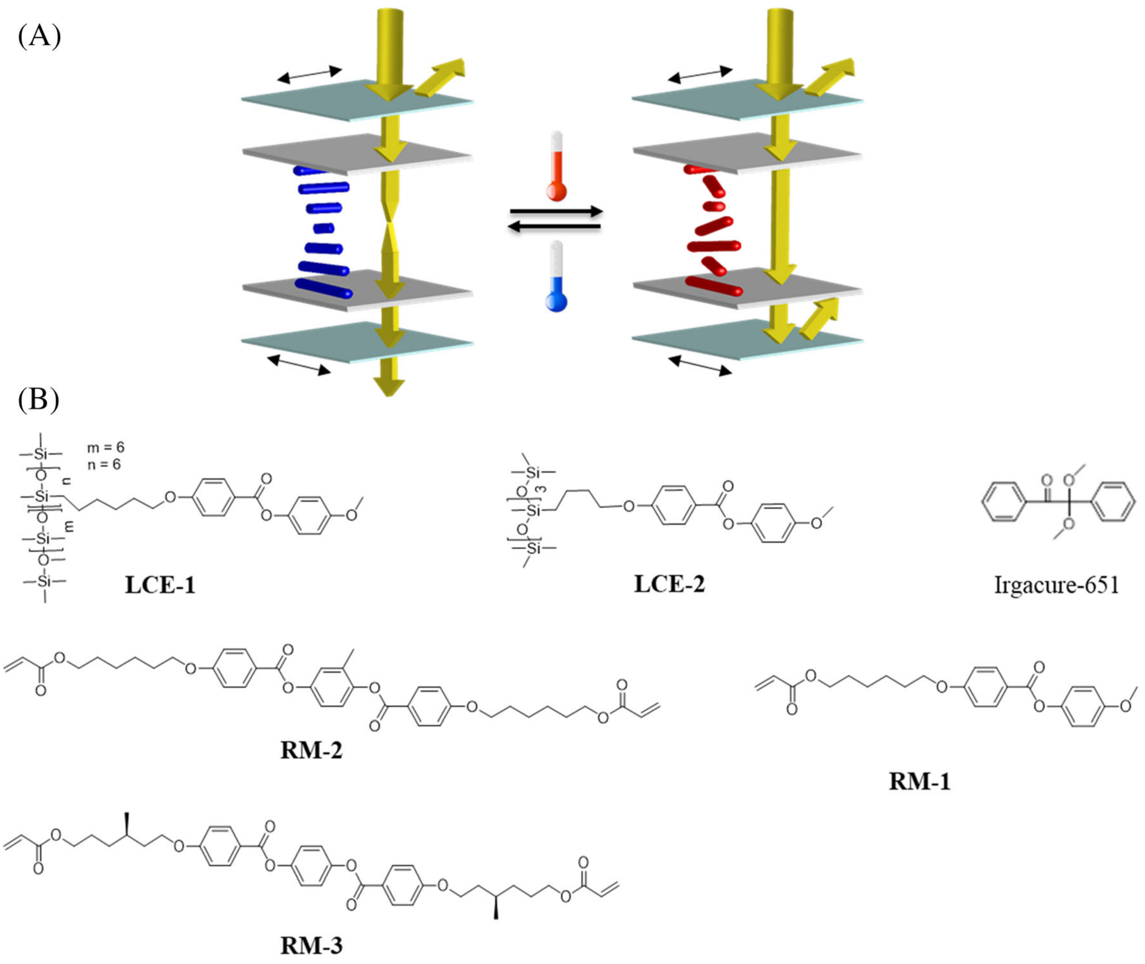

FIGURE 1 (A) Schematic drawing of the reflective 'smart' window. The double headed black arrows indicate the polarization direction of the reflective polarizers and the yellow arrows indicate the path of the traveling light. The LCs are ordered in a twisted nematic fashion below the $T_{N\text{-}I}$ (blue bars) and less ordered above the $T_{N\text{-}I}$ (red bars). (B) Chemical structures of the compounds used. LSc, liquid crystals [Color figure can be viewed at wileyonlinelibrary.com]

remaining light is also reflected by the second polarizer, resulting in higher total reflection over the operative wavelength regime. Thermal simulations show that the energy saving potential of deploying these responsive 'smart' windows as automotive windows could be significant.

2 | EXPERIMENTAL

2.1 | Materials

LCE-1 ((4-methoxyphenyl 4-[hexyloxy]benzoate)siloxane dimethylsiloxane copolymer) and LCE-2 ((4-methoxyphenyl 4-[butoxy]benzoate)siloxane polymer) were purchased from Synthon Chemical GmbH & Co. Achiral monoacrylate RM-1 ((4-methoxyphenyl 4-((6-[acryloyloxy]hexyl)oxy)benzoate), and achiral diacrylate RM-2 (1,4-phenylene bis (4-((6-[acryloyloxy]hexyl)oxy)benzoate)), were purchased from Merck. Chiral diacrylate RM-3 ((R,R)-1,4-di-(6-acryloyloxy-3-methylhexyloxy)benzoyloxy)benzene) was obtained from Philips Research lab. The photoinitiator Irgacure 651 (2,2-dimethoxy-1,2-diphenylethan-1-one) was purchased from Ciba.

2.2 | Methods

2.2.1 | Rubbed polyimide coated glass substrates

Glass slides (3 × 3 cm^2) were cleaned by ultrasonication in ethanol for 30 min and subsequently exposed to a UV-ozone (UVP PR-100) treatment for 20 min. A polyimide (JSR Optmer AL1051) layer was spin coated on these glass substrates by rotating at 1000 rpm for 5 s, followed by 5000 rpm for 40 s using a Karl Suss CT 62 spin coater. After spin coating, the substrates were placed on a hotplate at 100°C for 15 min and subsequently placed in an oven at 180°C for 90 min. The cured polyimide glass substrates were rubbed over a velvet cloth before use.

2.2.2 | Fabrication of reflective 'smart' windows

Twisted nematic LC cells were fabricated by gluing two polyimide glass substrates rubbed in a perpendicular fashion using UV-curable glue (Norland Products Inc UVS 91) containing 6 μm polystyrene beads (Sekisui). The glue was placed on two opposite edges of one substrate and the substrates were pressed together using clamps and cured using a low intensity UV-lamp (4 Philips CLEO 15 W lamps) for 30 min. The LC mixture components were weighed in the desired ratios and subsequently dissolved in dichloromethane (50 wt%). About 40 μl of the solution was placed on a microscope slide on a hotplate (40°C) to evaporate the solvent. The LC mixture was filled in the twisted nematic LC cell overnight in the nematic LC phase by capillary action on a hotplate of about 55°C. After filling, the LC mixture was photopolymerized in 10 min with an EXFO Omnicure S2000 mercury lamp UV light (16 mW/cm^2). Subsequently, two reflective linear polarizers (3 M DBEF films, Figure S1) with adhesive layer on one side were placed following the alignment direction of the twisted nematic LC cell.

2.2.3 | Differential scanning calorimetry

Transition temperatures of the LC mixtures were measured using a TA Instrument DSC Q2000. Measurements consisted of 3 cycles at a rate of 5°C/min.

2.2.4 | UV/Vis/NIR spectrophotometry

Temperature-responsive transmission spectra were measured on a Shimadzu UV-3102 PC UV/Vis/NIR spectrophotometer equipped with an MPC-3100 sample compartment and a Linkam TMS93/LMP93 temperature control stage. Two glass slides were used as baseline measurement. The transmission spectra at room temperature were also measured on a Perkin Elmer Lambda 750 UV/Vis/NIR-spectrophotometer. This spectrum was used to correct for the detector change artifact observed in the temperature-responsive spectra of this 'smart' window. Angular dependent measurements were also performed on the Perkin Elmer spectrophotometer equipped with an ARTA accessory that utilizes a 60 mm integrating sphere mounted on a goniometer with PMT and InGaAs detectors.

3 | RESULTS AND DISCUSSION

3.1 | Preparation of the temperature responsive light-reflective windows

The temperature responsive twisted nematic liquid crystal polymer systems contain a chiral liquid crystalline network (LCN) interpenetrating through a noncrosslinked liquid crystal elastomers (LCE) a and rely on temperature-responsive behavior of the LCE as reported earlier.[42–44] For preparing the first 'smart' window, we filled a 6 μm twisted nematic LC cell with a mixture containing LCE-1 (82.6 wt%), monoacrylate RM-1 (13.2 wt%), diacrylate RM-2 (2.8 wt%) and chiral diacrylate RM-3

(0.4 wt%). The second smart window sample contained LCE-2 (83.6 wt%), monoacrylate RM-1 (13.4 wt%), diacrylate RM-2 (1.6 wt %), chiral diacrylate RM-3 (0.4 wt%). The structures of all the molecules are found in Figure 1 (B). The chiral diacrylate was used to induce the rotation of consecutive LC planes. A photoinitiator (Irgacure-651, 1 wt%) was added to allow photopolymerization of the acrylates to form a network. After photo-polymerization with UV-light, we prepared the 'smart' windows by sandwiching the twisted nematic LC cell between two reflective linear polarizers following the LC director (Figure 1(A)).

3.2 | Characterization of the temperature responsive light-reflective windows

We first characterized the temperature responsive optical device based on the semi-IPN containing LCE-1. Below the T_{N-I} of the twisted nematic LC, LP light transmitted by the first polarizer is rotated by the twisted nematic LC and is transmitted through the second polarizer (the T_{N-I} of the unpolymerized LC mixture is 38°C, Figure S2). As a result, the 'smart' window transmits between 40 and 50% of incoming unpolarized light over the wavelength range for which the polarizers are designed (400–1100 nm, Figure 2(A) and (B)). Note that the irregularities in the transmission spectra can be attributed to imperfections of the reflective linear polarizers and measuring apparatus (Figure S1).

When heated above T_{N-I}, the LCE loses order and the LCN is no longer able to efficiently rotate the LP light transmitted by the first polarizer, and thus encounters the second polarizer with the polarization direction rotated with respect to the polarizer transmission axis, and thus is partially reflected. Because of this, the transmission decreases to approximately 10% over the entire wavelength range upon heating to 69°C (LCE-1, T_{N-I} = 58 °C), thereby reducing transmission through the 'smart' window (Figure S3). The 'smart' window was demonstrated to be reversible over at least 7 cycles of heating and cooling (Figure 2(C)). The transmission of the window is dependent on the incidence angle of the

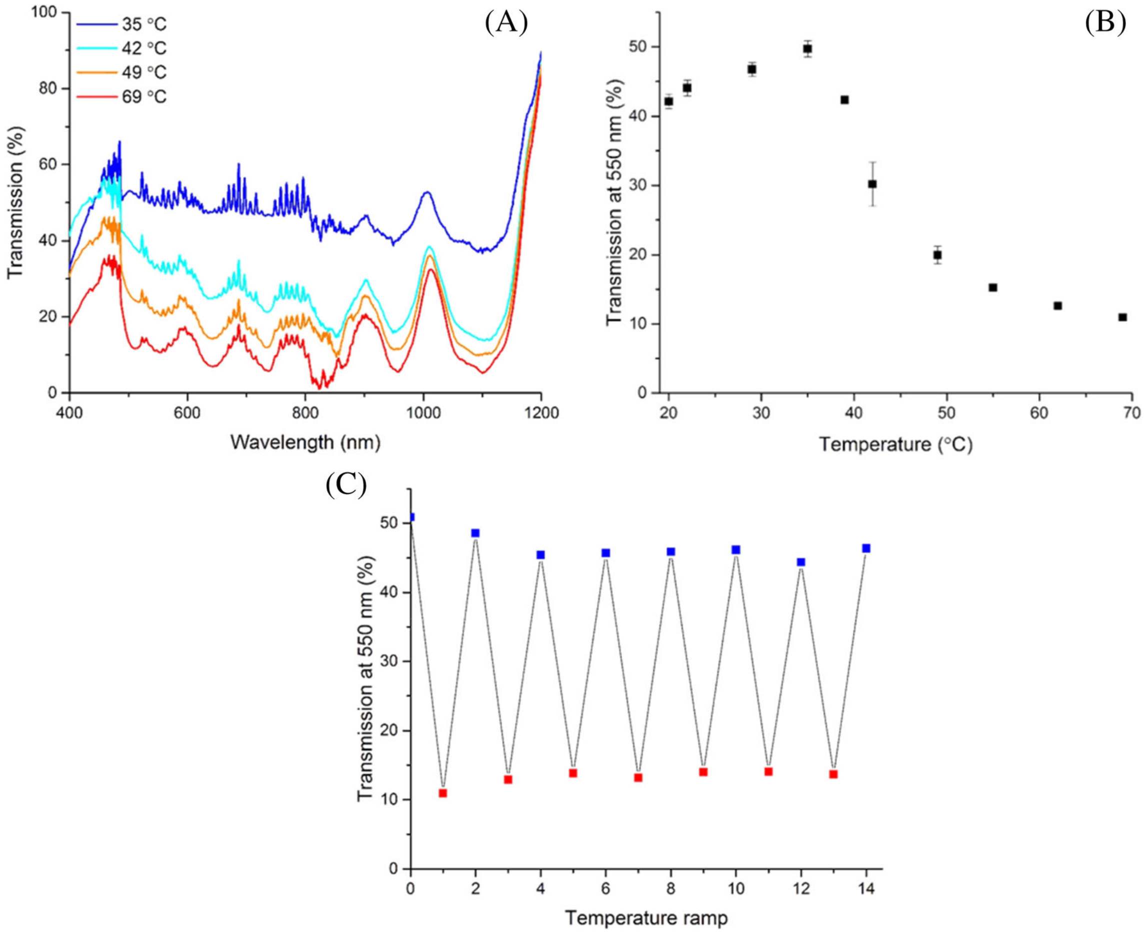

FIGURE 2 (A) Transmission spectra of the 'smart' window containing LCE-1 upon heating. (B) Transmission of the 'smart' window at 550 nm as a function of temperature averaged over one heating and cooling cycle. (C) Transmission of the 'smart' window at 550 nm after consecutive heating (69°C, red squares) and cooling (20°C, blue squares) ramps. LCE, liquid crystal elastomer [Color figure can be viewed at wileyonlinelibrary.com]

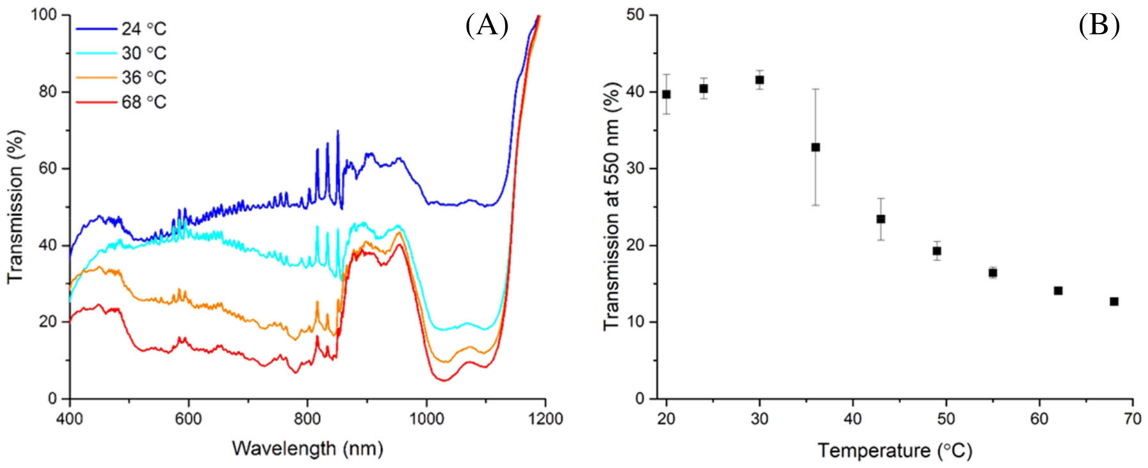

FIGURE 3 (A) Transmission spectra of the 'smart' window containing LCE-2 upon heating. (B) Transmission of the 'smart' window at 550 nm as a function of temperature averaged over one heating and cooling cycle. LCE, liquid crystal elastomer [Color figure can be viewed at wileyonlinelibrary.com]

light with somewhat decreased transmission at larger oblique angles (Figure S4). This is likely the result of generation of elliptical polarization of the light by the LC.

The transition temperature of the 'smart' window can be programmed by using an LCE with a different $T_{N\text{-}I}$, allowing the window response to be tailored to the desires of the occupants and region of the world where the window is deployed. As an example, we produced an additional 'smart' window using an elastomer with a lower transition temperature (LCE-2, $T_{N\text{-}I}$ = 40°C). This resulted in a reduction of the $T_{N\text{-}I}$ of the unpolymerized mixture to 26°C (Figure S5). The device started to decrease its transmission above 30°C, which is 5°C lower compared to the 'smart' window prepared with LCE-1 (Compare Figure 3 with Figure 2).

3.3 | Computational study of overheating reduction in parked vehicles

The type of 'smart' windows we report here can potentially be deployed in a variety of application domains. In particular, the automotive industry appears to be a promising area because the interiors of automobiles may reach very high temperatures in sunny conditions unless steps are taken to reject the incident light.[9] These 'smart' windows could be integrated in automobile rear-, side-, and roof windows to manage the passage of visible and/or IR light for enhanced thermal comfort for the passengers while maintaining passenger safety. Calculations of the visible and solar light transmittance show a clear contrast at various transition temperatures (Figure S6). Simulations were carried out using the software EnergyPlus, with a thermal model of a Tesla model S that was previously validated.[9] Simulation results with the 'smart' window installed on the rear back-, back- and roof windows show that cabin temperatures of the vehicle parked in Amsterdam in June have an average maximum cabin temperature reduction of 3.3 and 6.7°C compared to a standard automotive glass type for external temperatures of 35 and 69°C, respectively (Figure S7). These results indicate a significant potential of these light-reflective 'smart' windows to reduce automotive air-conditioning consumption and increasing the thermal comfort of passengers.

4 | CONCLUSION AND OUTLOOK

We demonstrate the working principle of a novel reflective 'smart' window. The device relies on the reversible decrease of order of a noncross-linked LCE interpenetrated through an LCN upon heating. We sandwiched a twisted nematic semi-IPN network between two reflective linear polarizers, resulting in a reflective 'smart' window which reduces its transmission from about 50–10% over a wavelength range between 400 and 1100 nm. We show that the transition temperature of the 'smart' window can be tuned so that the device can comply to the desires of occupants and different regions of the world.

Thermal simulations of a parked vehicle show that the 'smart' windows can reduce the cabin temperature by 3–7°C when replacing normal automotive glass windows with the 'smart' windows on the rear back-, back- and roof windows of a car, revealing the energy saving potential of this method.

To comply with safety regulations and user demands, future research should focus on controlling the level of transmission change. In addition, the 'smart' windows

show a reflective appearance due to the reflective polarizers used. Absorptive polarizers or a combination of an absorptive and reflective polarizer could be used to reduce this reflective appearance if desired. Furthermore, the scalability of the fabrication method of the 'smart' windows and their durability when exposed to outdoor weather conditions should be investigated to elucidate industrial feasibility.

ACKNOWLEDGMENTS

This research at the Eindhoven University of Technology was funded by the Netherlands Organization of Scientific Research (NWO) and the 'Green Cities 2019' project, which is jointly funded by NWO, through the Merian Fund, and the Chinese Academy of Sciences (CAS). This work was further financially supported by the National Natural Science Foundation of China (Nos. 51561135014, U1501244, 2161101058), the Program for Changjiang Scholars and Innovative Research Teams in Universities (No. IRT13064) and the Guangdong Innovative Research Team Program (No. 2013C102), the Major Science and Technology Projects of Guangdong Province (No. 2015B090913004), the 111 Project, the Collaborative Innovation and Platform for the Construction of special funds of Guangdong Province (No. 2015B050501010), and the SCNU-TUE Joint Lab of Devices Integrated Responsive Materials.

ORCID

Augustinus J. J. Kragt https://orcid.org/0000-0001-9355-6689
Roel C. G. M. Loonen https://orcid.org/0000-0001-6101-1449
Dirk J. Broer https://orcid.org/0000-0001-6136-3276
Michael G. Debije https://orcid.org/0000-0001-8844-1115
Albert P. H. J. Schenning https://orcid.org/0000-0002-3485-1984

REFERENCES

[1] H. Karabay, M. Arıcı, *Energy Build.* **2012**, *45*, 67.
[2] E. Neeman, G. Sweitzer, E. Vine, *Energy Build.* **1984**, *6*, 159.
[3] L. Heschong, R. L. Wright, S. Okura, *J. Illum. Eng. Soc.* **2002**, *31*, 101.
[4] I. Knez, C. Kers, *Environ. Behav.* **2000**, *32*, 817.
[5] M. Casini, *Environ. Eng.* **2014**, *2*, 273.
[6] Y. Ke, C. Zhou, Y. Zhou, S. Wang, S. H. Chan, Y. Long, *Adv. Funct. Mater.* **2018**, *28*, 1800113.
[7] R. C. G. M. Loonen, F. Favoino, J. L. M. Hensen, M. Overend, *J. Build. Perform. Simul.* **2017**, *10*, 205.
[8] A. A. Solovyev, S. V. Rabotkin, N. F. Kovsharov, *Mater. Sci. Semicond. Process.* **2015**, *38*, 373.
[9] V. Soulios, R. C. G. M. Loonen, V. Metavitsiadis, J. L. M. Hensen, *Appl. Energy* **2018**, *231*, 635.
[10] L. Long, H. Ye, *Sci. Rep.* **2014**, *4*, 6427.
[11] M. Kamalisarvestani, R. Saidur, S. Mekhilef, F. S. Javadi, *Renew. Sust. Energ. Rev.* **2013**, *26*, 353.
[12] Y. Wang, E. L. Runnerstrom, D. J. Milliron, *Annu. Rev. Chem. Biomol. Eng.* **2016**, *7*, 283.
[13] V. Costanzo, G. Evola, L. Marletta, *Sol. Energy Mater. Sol. Cells* **2016**, *149*, 110.
[14] S. Hoffmann, E. S. Lee, C. Clavero, *Sol. Energy Mater. Sol. Cells* **2014**, *123*, 65.
[15] A. Raicu, H. R. Wilson, P. Nitz, W. Platzer, V. Wittwer, E. Jahns, *Sol. Energy* **2002**, *72*, 31.
[16] R. C. G. M. Loonen, M. Trčka, D. Cóstola, J. L. M. Hensen, *Renew. Sustain. Energy Rev.* **2013**, *25*, 483.
[17] Y. Cui, Y. Ke, C. Liu, Z. Chen, N. Wang, L. Zhang, Y. Zhou, S. Wang, Y. Gao, Y. Long, *Joule* **2018**, *2*, 1707.
[18] A. Seeboth, R. Ruhmann, O. Mühling, *Materials* **2010**, *3*, 5143.
[19] H. Watanabe, *Sol. Energy Mater. Sol. Cells* **1998**, *54*, 203.
[20] P. Nitz, H. Hartwig, *Sol. Energy* **2005**, *79*, 573.
[21] X. H. Li, C. Liu, S. P. Feng, N. X. Fang, *Joule* **2019**, *3*, 290.
[22] M. J. Serpe, *Nat. News Views* **2019**, *565*, 438.
[23] H. Khandelwal, A. P. H. J. Schenning, M. G. Debije, *Adv. Energy Mater.* **2017**, *7*, 1602209.
[24] C. Lampert, *Sol. Energy Mater. Sol. Cells* **1998**, *52*, 207.
[25] J. Sun, H. Wang, L. Wang, H. Cao, H. Xie, X. Luo, J. Xiao, H. Ding, Z. Yang, H. Yang, *Smart Mater. Struct.* **2014**, *23*, 125038.
[26] W. Zhang, S. Kragt, A. P. H. J. Schenning, L. T. De Haan, G. Zhou, *ACS Omega* **2017**, *2*, 3475.
[27] L. Xiao, H. Cao, J. Sun, H. Wang, D. Wang, Z. Yang, W. He, *Liq. Crystals* **2016**, *43*, 1299.
[28] C. Binet, M. Mitov, M. Mauzac, *J. Appl. Phys.* **2001**, *90*, 1730.
[29] B. W. Hu, H. Zhao, L. Song, Z. Yang, H. Cao, Z. Cheng, *Adv. Mater.* **2010**, *651*, 468.
[30] M. Mitov, E. Nouvet, N. Dessaud, *Eur. Phys. J. E: Soft Matter Biol. Phys.* **2004**, *15*, 413.
[31] H. Khandelwal, G. H. Timmermans, M. G. Debije, A. P. H. J. Schenning, *Chem. Commun.* **2016**, *52*, 10109.
[32] J. Xiang, Y. Li, Q. Li, D. A. Paterson, J. M. D. Storey, C. T. Imrie, O. D. Lavrentovich, *Adv. Mater.* **2015**, *27*, 3014.
[33] H. Khandelwal, R. C. G. M. Loonen, J. L. M. Hensen, M. G. Debije, A. P. H. J. Schenning, *Sci. Rep.* **2015**, *5*, 11773.
[34] M. G. Debije, A. P. H. J. Schenning, *Modul. Mater. Sci. Mater. Eng.* **2016**, 1. https://doi.org/10.1016/B978-0-12-803581-8.04039-X.
[35] X. Liang, C. Guo, M. Chen, S. Guo, L. Zhang, F. Li, S. Guo, H. Yang, *Nanoscale Horizons* **2017**, *2*, 319.
[36] X. Liang, S. Guo, S. Guo, M. Chen, C. Li, Q. Wang, C. Zou, C. Zhang, L. Zhang, H. Yang, *Mater. Horizons* **2017**, *4*, 878.
[37] K. M. Lee, V. P. Tondiglia, M. E. Mcconney, L. V. Natarajan, T. J. Bunning, T. J. White, *ACS Photon* **2014**, *1*, 1033.
[38] C. M. Lampert, *Mater. Today* **2004**, *7*, 28.
[39] F. Liu, J. Wang, Z. Ge, K. Li, H. Ding, B. Zhang, D. Wang, H. Yang, *J. Mater. Chem. C* **2013**, *1*, 216.
[40] R. Guo, K. Li, H. Cao, X. Wu, G. Wang, Z. Cheng, F. Wang, H. Zhang, H. Yang, *Polymer* **2010**, *51*, 5990.
[41] H. Khandelwal, F. Roberz, R. C. G. M. Loonen, J. L. M. Hensen, C. M. W. Bastiaansen, D. J. Broer, M. G. Debije, A. P. H. J. Schenning, *Proc. SPIE* **2014**, *9182*, 91820.
[42] A. J. J. Kragt, D. J. Broer, A. P. H. J. Schenning, *Adv. Funct. Mater.* **2018**, *28*, 1704756.

[43] A. J. J. Kragt, N. C. M. Zuurbier, D. J. Broer, A. P. H. J. Schenning, *ACS Appl. Mater. Interfaces* **2019**, *11*, 28172.
[44] A. J. J. Kragt, I. P. M. Gessel, A. P. H. J. Schenning, D. J. Broer, *Adv. Opt. Mater.* **2019**, *7*, 1901103.

SUPPORTING INFORMATION

Additional supporting information may be found online in the Supporting Information section at the end of this article.

How to cite this article: Kragt AJJ, Loonen RCGM, Broer DJ, Debije MG, Schenning APHJ. 'Smart' light-reflective windows based on temperature responsive twisted nematic liquid crystal polymers. *J Polym Sci*. 2021;59: 1278–1284. https://doi.org/10.1002/pol.20210008

ACS APPLIED MATERIALS & INTERFACES

www.acsami.org Research Article

Thermoresponsive Reflective Scattering of Meso-Scale Phase Separation Structures of Uniaxially Orientation-Ordered Liquid Crystals and Reactive Mesogens

Hiroshi Kakiuchida,* Masayuki Kabata, Takanori Matsuyama, and Akifumi Ogiwara

Cite This: *ACS Appl. Mater. Interfaces* 2021, 13, 41066–41074 Read Online

ACCESS | Metrics & More | Article Recommendations | Supporting Information

ABSTRACT: Polymer network liquid crystals (PNLCs) capable of thermoresponsive change in reflective scattering were fabricated using a self-organization technique called photopolymerization-induced phase separation. These PNLCs exhibit nonscattering states at temperatures τ below the nematic-to-isotropic (NI) phase transition temperature τ_{NI} but reflective scattering states at τ values above τ_{NI}. The magnitude of change of optical clarity is 80% and of solar transmittance is 20% in PNLCs with a thickness of 50 μm. The microscopic structures consist of wavelength- or meso-scale phase separation domains of liquid crystals (LCs) and polymerized reactive mesogens (RMs) in which cyanobiphenyl (CB) groups are thermoresponsively transformed between uniaxially orientation-co-ordered and disordered states. Such thermoresponsive structures were fabricated by employing the CB groups as mesogenic bodies, which were expected to mutually associate due to their physicochemical structures. Cross-linkers stabilized the meso-scale domains and made the PNLCs durable through repeated temperature changes. Polarizing optical microscopy (POM) and scanning electron microscopy showed meso-scale composites that reflectively scatter visible and near-infrared light. POM and Fourier-transform infrared spectroscopy at different temperatures suggest that the orientation order of the CB groups changes in the LC phase in response to temperature but remains ordered in the RM phase. Such a thermoresponsive change in the orientation order produces the switchability in meso-scale nonuniformity and consequently in reflective light scattering. The thermoresponsive PNLCs are not only effective as energy-saving smart windows but also advantageous at stages of manufacture, installation, and operation.

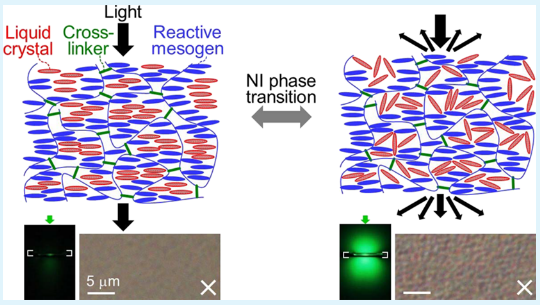

KEYWORDS: *polymer network liquid crystal (PNLC), photopolymerization-induced phase separation (PPIPS), submicrometer domains, nematic-to-isotropic (NI) phase transition, orientation co-order, cyanobiphenyl (CB) group, hemispheric transmittance, thermoresponsive switchable windows*

Downloaded via HARBIN INST OF TECHNOLOGY on September 19, 2022 at 09:39:15 (UTC).
See https://pubs.acs.org/sharingguidelines for options on how to legitimately share published articles.

1. INTRODUCTION

Polymer network liquid crystals (PNLCs), which possess meso- or wavelength-scale phase separation domains of liquid crystals (LCs) and polymers, can efficiently modulate light propagation in response to extrinsic stimuli, namely, temperature, electric fields, light, stress, and so on.[1−6] Given their versatile environmental responsiveness, PNLCs have been studied for various applications such as smart windows,[3,5] physicochemical sensors,[7] information storage,[8,9] tunable optical filters and lasers,[3,10] focusable lenses,[11] and light diffusers.[12,13]

Switchable smart windows can enhance the comfort of occupants while keeping energy consumption low in buildings and vehicles. Such windows can adjust solar transmittance in response to seasonal and daily changes in weather. Transmittance is controlled in various ways, such as by light reflection, absorption, and scattering in response to electric fields, temperature, and light.[6] Each type has different advantages and disadvantages at the manufacture, installation, and operation stages. Electric operation types provide flexible switchability for occupants, but they require intricate electrodes and wiring in fabrication and installation. Thermoresponsive types are readily installed because of their simple structures for assembly, although their response parameters cannot be adjusted after installation. From the viewpoint of optical properties, absorption and reflection-control types can efficiently change light transmission. Recent reports include two-step thermoresponsive control of optical absorption with dichroic dyes and LCs possessing smectic/nematic/isotropic phase transitions.[14] Such multistage changes may be more comfortable than other types. Tunable selective reflectance, which enables us to control near-infrared reflection while

Received: June 4, 2021
Accepted: August 6, 2021
Published: August 24, 2021

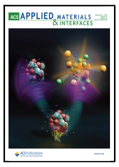

ACS Publications © 2021 American Chemical Society

maintaining high clarity, can be achieved using cholesteric LCs (CLCs), and several extrinsic-stimulative structures of CLCs have been studied, which increase the magnitude of reflectance change in the invisible wavelength range. Nowadays, many thermoresponsive tunable CLCs have been developed.[15] Particularly, a successful CLC, in which helical pitches were nonuniformly stabilized by polymer networks, exhibited a thermoresponsive change in solar transmittance of 17−18% while keeping a visible transmittance of more than 93% independent of temperature,[16] according to our calculations using the thermally tunable selective reflectance spectra. The optical absorption and reflection types are an ideal optical structure for energy-saving switchable windows. However, the sunlight absorbed by the dyes is partly transferred to heat flows into the room, whereas tunability in the infrared selective reflection generated from the CLCs does not yet satisfy practical demands with currently developed materials or structures. On the other hand, scattering-control types are superior in light controllability and ease of fabrication; that is, the structures deflect incident light without substantial absorption and are simple to produce because elaborate design is not necessary.

PNLC nonuniform structures that control light scattering have often been fabricated via a self-organizing process called photopolymerization-induced phase separation (PPIPS).[17−20] Through PPIPS during light exposure, LCs locally segregate from monomers during polymerization, and consequently, an optically nonuniform distribution of LC and polymer domains is formed. Although many studies of light scattering from PNLCs have paid attention only to opaqueness, some research groups are investigating light scattering properties, that is, the dependence of light scattering on angles of incidence and scattering, on optical polarization, and on wavelength.[12,13,21−25] Light diffusers for homogenizing, expanding, and guiding incoming light have been developed with PNLCs for broad applications such as backlit displays, wide-area printing, and smart windows. Light scattering properties of PNLCs have been studied as a function of the size and distribution of LC droplets (domains) and analyzed based on theoretical optics.[21,23,25] Montgomery *et al.* explained hemispheric transmittance/reflectance depending on the droplet diameters by employing Rayleigh−Gans calculations,[21] whereas Barchini *et al.* reproduced diffuse luminous reflectance by numerical simulations of multiple scattering based on the two-flux model of radiative transfer.[23] Zhou *et al.* fabricated light diffusers with 94% haze and high hemispheric (total) transmittance for uniform backlighting.[25] This static light diffusion was achieved by composites of nematic LCs with micrometer-scale polymer balls. Wilson and Eck produced electrically switchable PNLCs and then demonstrated a change of 38% in hemispheric solar transmittance.[22] This was achieved using 110 μm-thick PNLCs with submicrometer- or wavelength-scale domains. Recently, some interesting applications have been reported such as optical homogenizers of oblique-incident light sources,[26,27] anisotropic beam expanders, and polarization-(in)dependent diffusers.[12,13] Switchable privacy windows ideally exhibit complete opacity and transmissive (forward) scattering at hazy states, allowing the view from within buildings and vehicles to be screened without losing brightness. In contrast, energy-saving switchable windows should produce reflective (backward) scattering to block sunlight into rooms during uncomfortably hot daytime hours, although such scattering properties have been challenging to achieve using PNLCs.[28−30] Our purpose is to create thermoresponsive PNLCs whose reflective light scattering changes in response to temperature, that is, zero (or low)-intensity reflective scattering at low temperatures but high-intensity at high temperatures. We develop meso- or wavelength-scale PPIPS structures that transform between optically uniform at low temperatures and nonuniform at high temperatures, to produce thermoresponsive changes in reflective scattering. In specific application, such structures potentially produce effective energy-saving smart windows.

The optical clarity thermoresponsively varies between transparent (nonscattering) and hazy (scattering) states by changing refractive-index nonuniformity formed with LC and RM phases. At low temperatures, LCs and RMs are co-ordered in molecular orientation, and consequently, the PNLC is optically uniform. When the PNLC is heated higher than the nematic-to-isotropic (NI) phase transition temperature, it becomes nonuniform since the LCs become disordered while the RMs remain ordered. According to the Mie scattering theory,[31] the direction and intensity of light scattering primarily depend on (i) the domain size of the nonuniformity and (ii) the magnitude of refractive-index modulation in optical nonuniform structures. The theory indicates that the domains with wavelength-scale or smaller size produce more Rayleigh-like scattering, that is, more backward scattering. Optically nonuniform structures composed of domains small enough to be wavelength-scale were experimentally found out to scatter light more backward than forward,[21,23,24] while those effecting greater refractive-index modulation produce more intense light scattering.[31]

Thermoresponsive PNLCs are preferable because they operate without electrodes and grids for electricity supply. However, they are inferior in the controllability of scattering intensity to electrically operated PNLCs since the molecular orientation of LCs is less ordered in the absence of electric fields. The nonuniform irradiation technique was a successful external method for producing meso-scale nonuniform structures of (i) and (ii) and enabled various thermoresponsive changes in light scattering.[32,33] In the present study, we address this issue via internal material design and use monofunctional reactive mesogens (RMs) possessing cyanobiphenyl (CB) groups as found in the mesogenic body of the LCs, as shown in Scheme 1. The RMs become normal chain polymers accompanying CB branches by polymerization reactions of acryloyl groups via PPIPS. The CB groups in the LCs and RMs may be co-ordered in molecular orientation since they are known to mutually associate due to their strong dipole moment.[34,35] This molecular association is expected to enhance the formation of optical uniformity at temperatures

Scheme 1. Structural Formulae of the Liquid Crystal (LC) and the Reactive Mesogen (RM)[a]

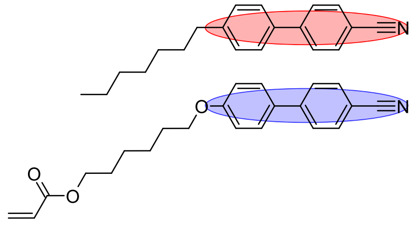

[a]They both possess alkyl chains and a cyanobiphenyl (CB) mesogenic body, as expressed by red and blue ovals.

https://doi.org/10.1021/acsami.1c10377
ACS Appl. Mater. Interfaces 2021, 13, 41066−41074

below the NI phase transition temperature (τ_{NI}), consequently increasing the magnitude of thermoresponsive refractive-index modulation, as described in (ii). Regarding (i), we have two approaches to control the size of phase separation domains in PPIPS. First, an increase in the molar ratio of RMs to LCs was expected to decrease domain size through wriggling and entanglement of the polymer chains, although sufficiently high polymer concentrations might inhibit LC molecular rotation. Second, cross-linkers (CLs) were expected to network RM polymer chains, thus reducing domain size while maintaining the polymer concentration, which may increase reflective scattering based on the theory.[31] Thermoresponsive PNLCs that can be simply fabricated via material design and can efficiently control reflective scattering are a promising candidate for smart windows that effectively saves energy at every stage: manufacture, installation, and operation.

2. EXPERIMENTAL SECTION

UV irradiation was used in PPIPS fabrication of PNLCs from raw mixtures of the LC, RM, CL, and photoinitiator (PI) filling a gap between a pair of transparent glass plates. The LCs possess CB groups and have different values of τ_{NI}. 4-Cyano-4′-heptylbiphenyl (H0812, Tokyo Chemical Industry Co., Ltd. (TCI); τ_{NI} = 42 °C) was used for the whole experiments. The other LCs were used to fabricate the PNLCs with different thermoresponses: 4-cyano-4′-hexylbiphenyl (FR-2321, FUJIFILM Wako Pure Chemical Corp.; τ_{NI} = 29 °C) and two homemade LC mixtures.[36] One LC mixture, which had a τ_{NI} of 52 °C, was prepared by mixing H0812 at 24 wt %, 4-cyano-4′-pentylbiphenyl (C1550; TCI) at 45 wt %, 4-cyano-4′-pentyloxybiphenyl (C1551; TCI) at 10 wt %, 4-cyano-4′-heptyloxybiphenyl (C1606; TCI) at 9 wt %, and 4-cyano-4′-*n*-octyloxybiphenyl (C2618; TCI) at 12 wt %. The other LC mixture, which had a τ_{NI} of 61 °C, was prepared by mixing H0812 at 25 wt %, C1550 at 51 wt %, C2618 at 16 wt %, and 4-cyano-4″-pentyl-*p*-terphenyl (C2910; TCI) at 8 wt %. The RM was 4-[(6-acryloyloxy)hexyloxy]-4′-cyanobiphenyl (Osaka Organic Chemical Industry Ltd. (OOC)), and the CL was an acryloyl bifunctional monomer (LOA-019; OOC). The LC, RM, and CL were mixed at molar ratios of 100:x:y, x and y in mol %. Then, the PI, 2,2-dimethoxy-2-phenylacetophenone (D1702, TCI), was added to the mixture at 1 mol %. In the mixtures, x was varied from 50 to 100 mol %, while y was 0 or 1 mol %. Two types of samples were prepared, that is, without CLs (y = 0 mol %) and with CLs (y = 1 mol %). Each mixture was stirred at 60 °C for an hour, became a transparent liquid, and was defoamed by evacuation. A gap of 6 to 300 μm between a pair of transparent glass plates (25 × 20 × 0.7 mm, EHC Co., Ltd.) was then filled with each mixture. The glass surfaces were coated with polyimide and antiparallelly rubbing-treated to stabilize the orientation order of LC and RM molecules. Each mixture in the glass gap was exposed to UV light for 5 min at an intensity of 1 mW/cm^2 while a temperature of 20 °C was maintained. The light source for developing during PPIPS was a UV LED (MS20LX-365, ARK TECH Corp.), which is luminous at a main wavelength of 365 nm.

Two types of optical transmittances were measured as a function of the wavelength λ from 200 to 2500 nm at various temperatures τ between 20 and 50 °C, using a spectrophotometer (U4100, Hitachi High-Technologies Corp.). The direct transmittance $T_d(\lambda)$ is the intensity ratio of normal transmitted light within a divergence angle of 10° to normal incident light at λ within the optical setup shown in Scheme 2a. The direct luminous transmittance T_{lum}, which is an indicator of optical clarity, was calculated as

$$T_{lum} = \int \phi_{lum}(\lambda) T_d(\lambda) d\lambda / \int \phi_{lum}(\lambda) d\lambda \quad (1)$$

where ϕ_{lum} is the photopic spectral luminous efficiency in units at the wavelength of maximum luminous efficacy.[37] The hemispheric transmittance $T_h(\lambda)$ is the intensity ratio of forward scattered light summed over all angles to normal incident light, as measured using

Scheme 2. Optical Setups to Measure (a) Direct Transmittance T_d and (b) Hemispheric Transmittance T_h[a]

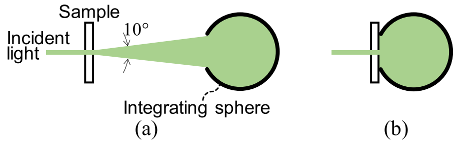

[a] T_d was measured as the transmittance of light within a divergence angle of 10° as determined by the aperture of an integrating sphere. T_h was measured as the transmittance of light passing through the sample detected by an integrating sphere attached to the rear surface of the sample.

the optical setup shown in Scheme 2b. The hemispheric solar transmittance T_{sol}, which is an indicator of the penetration rate of sunlight through windows, was calculated as

$$T_{sol} = \int \phi_{sol}(\lambda) T_h(\lambda) d\lambda / \int \phi_{sol}(\lambda) d\lambda \quad (2)$$

where ϕ_{sol} is the spectral solar irradiance for air mass 1.5 in units of W/m^2/nm.[38]

We examined the orientation order of LC and RM molecules using a Fourier-transform infrared (FTIR) spectrophotometer (Frontier, PerkinElmer Co., Ltd.) with an infrared (IR) polarizer. The IR absorption at a wavenumber around 2226 cm^{-1} was assigned to the molecular vibrational mode of cyano (−C≡N) groups, which are present in both LCs and RMs. The orientation order parameter S along the direction of the rubbing treatment was determined from the area of the absorption peak of the 2226 cm^{-1} band.[39] The areas were measured separately at polarizations parallel ($A_{\parallel}$) and perpendicular ($A_{\perp}$) to the direction of the rubbing treatment at temperatures of 20 and 50 °C, which are below and above the τ_{NI} of 7CB, respectively. S was calculated from these areas as[40]

$$S = (A_{\parallel} - A_{\perp})/(A_{\parallel} + 2A_{\perp}) \quad (3)$$

where $A_{\parallel}$ and $A_{\perp}$ were each determined from the area between the spectral curve and the baseline tangent to the curve.

The meso-scale domains and optical anisotropy at temperatures below and above the τ_{NI} of 7CB were observed using a polarizing optical microscope (POM; MT9430, Meiji Techno Co., Ltd.) at the crossed Nicol state with two polarizers sandwiching the sample, one set at a polarization azimuth of +45° from the rubbing direction and the other set at −45°. The polymer structures were observed using a scanning electron microscope (SEM; S-4300, Hitachi High-Technologies Corp.). The SEM images were obtained after peeling off the glass substrate and rinsing LCs away with methanol.

3. RESULTS AND DISCUSSION

Optical transmittance at various x and y values was measured for temperatures τ below and above τ_{NI}. Figure 1a,b shows direct transmittance T_d as a function of the wavelength λ and of τ, respectively, for a sample without CLs (x = 30 mol % and y = 0 mol %). T_{lum}, which was estimated using eq 1, decreased from 84.8 to 2.1% when τ increased from 20 to 50 °C. As shown in the photograph insets in Figure 1b and a movie (Movie S1, Supporting Information), the sample appearance was transparent at a τ of 20 °C, whereas at a τ of 50 °C, it looked opaque through the window and slightly blurred on the color chart. As shown in Figure 2, hemispheric transmittance T_h thermoresponsively varied, independent of λ. T_{sol}, which was estimated using eq 2, decreased from 88.9 to 84.8% when τ increased from 20 to 50 °C. The magnitude of the thermoresponsive change was smaller for T_{sol} than for T_{lum}, as is typical when such transmittance changes result from light scattering since light tends to scatter forward. As shown in the

https://doi.org/10.1021/acsami.1c10377
ACS Appl. Mater. Interfaces 2021, 13, 41066−41074

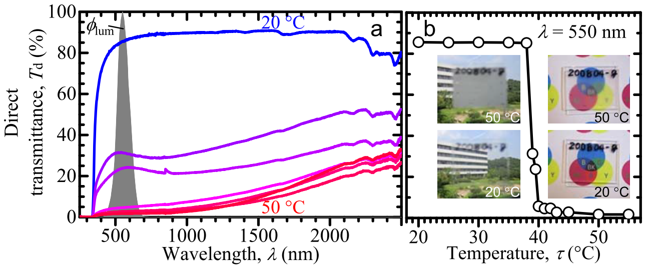

Figure 1. Direct transmittance T_d as a function of (a) wavelength and (b) temperature for a 50 μm-thick sample without CLs. (a) Transmittance spectra for various temperatures between 20 and 50 °C, where the gray highlighted curve indicates photopic spectral luminous efficiency in units at the wavelength of maximum luminous efficacy, ϕ_{lum}.[37] (b) Temperature dependence of T_d at a wavelength of 550 nm around τ_{NI} = 42 °C, with insets showing sample appearances at the window (Movie S1, Supporting Information) and on the color chart at different temperatures. T_{lum} was estimated using eq 1 to change between 84.8% at 20 °C and 2.1% at 50 °C.

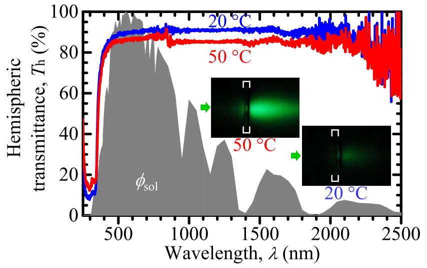

Figure 2. Hemispheric transmittance T_h as a function of the wavelength, for temperatures of 20 and 50 °C, for a 50 μm-thick sample without CLs, where the gray highlighted curve indicates the spectral solar irradiance for air mass 1.5 in units of W/m^2/nm, ϕ_{sol},[38] where it is normalized by the maximum value. The insets show scattering profiles for a green laser originating from the left, as expressed by arrows. T_{sol} was estimated using eq 2 to change between 88.9% at 20 °C and 84.8% at 50 °C.

photograph insets in Figure 2, the sample scattered light more transmissively when $\tau > \tau_{NI}$, whereas little scattering was produced when $\tau < \tau_{NI}$.

Figure 3a,b shows T_d as a function of λ and of τ, respectively, for a sample with CLs (x = 30 mol % and y = 1 mol %). T_{lum} varied from 83.6 to 0.7% when τ increased from 20 to 50 °C. The sample appearances, as shown in the photograph insets in Figure 3b and a movie (Movie S2, Supporting Information), were transparent and hazy at low and high temperatures, respectively. The sample was transparent at a τ of 20 °C, whereas it was translucent through the window and on the color chart at a τ of 50 °C. The reddish view through the window most likely resulted from submicrometer-scale nonuniform domains produced in the PNLC,[31] as explained later with POM and SEM images. As shown in Figure 4, T_h thermoresponsively varied, independently of λ. T_{sol} decreased from 89.1 to 69.6% when τ increased from 20 to 50 °C. The magnitude of the thermoresponsive change in T_{lum} was greater than that for the CL-free sample. As shown in the photograph insets in Figure 4, the sample scattered light more reflectively at $\tau > \tau_{NI}$. Such thermoresponsive switchability of reflective scattering is indispensable for applications to energy-saving smart windows, which can control the penetration of sunlight to the indoors.

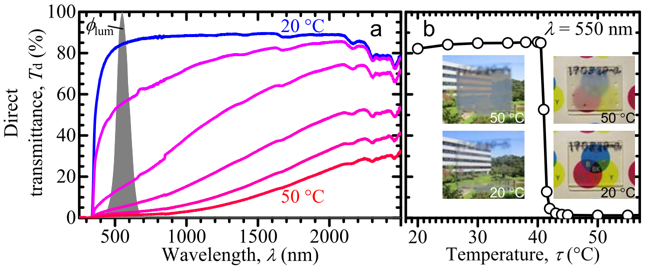

Figure 3. Direct transmittance T_d as a function of (a) wavelength and (b) temperature for a 50 μm-thick sample with CLs. (a) Transmittance spectra for various temperatures between 20 and 50 °C, where the gray highlighted curve indicates photopic spectral luminous efficiency in units at the wavelength of maximum luminous efficacy, ϕ_{lum}.[37] (b) Temperature dependence of T_d at a wavelength of 550 nm around τ_{NI} = 42 °C, with insets showing sample appearances at the window (Movie S2, Supporting Information) and on the color chart at different temperatures. T_{lum} was estimated using eq 1 to change between 83.6% at 20 °C and 0.7% at 50 °C.

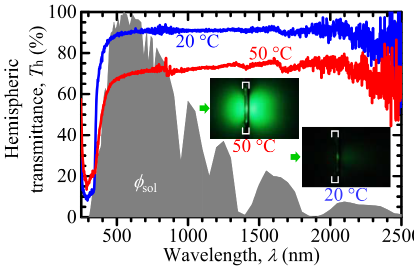

Figure 4. Hemispheric transmittance T_h as a function of the wavelength, at temperatures of 20 and 50 °C, for a 50 μm-thick sample with CLs, where the gray highlighted curve indicates the spectral solar irradiance for air mass 1.5 in units of W/m^2/nm, ϕ_{sol},[38] where it is normalized by the maximum value. The insets show the scattering profiles for a green laser originating from the left, as expressed by arrows. T_{sol} was estimated using eq 2 to change between 89.1% at 20 °C and 69.6% at 50 °C.

T_{lum} and T_{sol} were calculated using eqs 1 and 2 from T_d and T_h for samples without and with CLs and are plotted as a function of x in Figure 5. For samples without CLs (y = 0 mol %), the low-temperature T_{lum} of approximately 90% was as high as the glass substrates, independent of x. T_{lum} decreased with the rise in temperature across τ_{NI} and had a minimum of 5% at x around 30 mol %. This indicates that uniform structures were well-formed with the LCs and RMs at any mixture ratios and efficiently switchable from/to nonuniform structures by temperature change. T_{sol} was approximately 90% at low temperatures, independent of x, and decreased with the rise in temperature across τ_{NI}. The high-temperature T_{sol} had a minimum value of 85% at x around 25 mol %. The results of T_{lum} and T_{sol} suggest that the nonuniform structures formed without CLs produced forward (transmissive) light scattering. For samples with CLs (y = 1 mol %), T_{lum} at low temperatures decreased monotonically with the increase in x and was nearly 40% at an x of 50 mol %. In contrast, T_{lum} at high temperatures decreased from 90 to less than 5% as x increased from 0 to 25 mol % but then increased with a further increase in x. T_{sol} at high temperatures had a minimum value of 75% at x around 25 mol %. The results of T_{lum} and T_{sol} indicate that the nonuniform structures formed with CLs produced more backward (reflective) scattering than those without CLs. To sum up, the LCs and RMs form optically uniform structures

https://doi.org/10.1021/acsami.1c10377
ACS Appl. Mater. Interfaces 2021, 13, 41066−41074

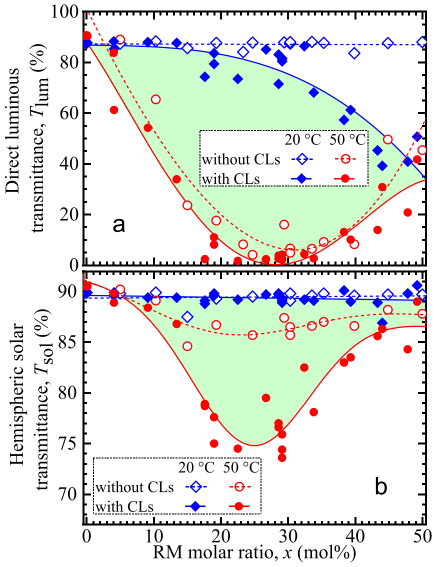

Figure 5. (a) Direct luminous transmittance T_{lum} and (b) hemispheric solar transmittance T_{sol} as a function of the RM molar ratio x for 30 μm-thick samples without CLs (open symbols) and with CLs (filled symbols), where diamonds and circles represent measurements at 20 and 50 °C, respectively. The curves fitted by empirically selected functions, namely, the sum of a linear and a Gaussian function, serve as guides for the eyes. Thermoresponsive changes in transmittance for the samples with CLs are highlighted by the green area between the blue and red solid curves.

and consequently produce excellent transparency at low temperatures. Adding CLs can enhance thermoresponsive switchability between transparent and reflective scattering states, that is, T_{lum} and T_{sol} changed by 80 and 15%, respectively, at x around 25 mol %.

For practical applications to switchable windows, thermoresponsive PNLCs must be high in solar controllability, flexible in temperature dependence, and durable during repetitions of hot and cool states. The thermoresponsive change in T_{sol} was examined at different thicknesses and found to increase monotonically with an increase in thickness, as shown in Supporting Information Figure S1. At a thickness of 300 μm, T_{sol} thermoresponsively changed by 35%, although T_{lum} at the low-temperature transparent state was 40%. Such lower values of T_{lum} for thicker samples result from haziness due to a slight mismatch in the refractive index between the LC and polymer phases. As shown in Supporting Information Figure S2, thermoresponsive switchable temperature of the PNLCs can be flexibly optimized by choosing LCs with different τ_{NI}, depending on the environment around the windows, such as glazing structures, location, climate, and residents' comfort. The durability of the performance during repeated temperature change cycles was examined by 5 min heating and cooling repetitions of a sample with CLs, as shown in Figure 6. The thermoresponse of T_d was unchanged after more than 1000 repetitions for the sample with CLs (Figure 6b), whereas it gradually declined for the sample without CLs (Figure 6a).

The direction and intensity of light scattering are chiefly determined by two structural properties of optical nonuniformity, namely, domain size and refractive-index unevenness, as respectively mentioned in (i) and (ii) in the Introduction. Smaller (or larger) domains can produce more reflective (or transmissive) light scattering.[31] Figures 7 and 8 show POM and SEM images of samples without CLs, respectively, for three different x. The micrographs of POM and SEM show phase separation structures with meso-scale domains whose size depends on x. As shown by POM images at low and high temperatures, samples without CLs transformed between optical uniformity and nonuniformity in response to τ. The domain size of the nonuniform structures decreased from ten to a few micrometers with varying morphology as x increased. Based on the SEM images, at x of 30 and 50 mol %, polymer domains with a (sub)micrometer scale were formed but partly aggregated to produce larger domains. Figures 9 and 10 show POM and SEM images for samples with CLs for three different values of x. The micrographs of POM and SEM show phase separation structures with domains with average sizes smaller than those for samples without CLs. For samples with CLs, as shown by POM images in Figure 9a−c, the microscopic structures at a τ of 20 °C were less uniform at larger x values, causing a decrease in the low-temperature T_{lum} (Figure 5a). The phase separation structures formed with CLs were nonuniform with smaller domains than those without CLs, as shown in Figures 7−10. In particular, at an x of 30 mol %, the nonuniform domains were distributed with a domain size of approximately 1 μm or less, and the CB groups of LC and RM phases were uniformly co-ordered in molecular orientation, as shown in the crossed Nicol POM with polarizers at polarization azimuths of 0 and 90° from the direction of the rubbing treatment (Supporting Information Figure S3). Such small domains may be able to contribute to the production of intense reflective scattering and consequently low T_{sol}; such an interpretation is consistent with the large thermoresponsive changes in T_{sol} at x values between 20 and 30 mol %, as shown in Figure 5b. Analyzing the POM and SEM images, the CLs seemed to network the polymer domains and stabilize the phase separation structures. Particularly, comparing Figure 8b with Figure 10b, the CLs most likely cross-linked the polymer domains effectively to avoid the aggregation between them.

The optical anisotropy observed by POM was caused by the orientation order of CB groups in both LCs and RMs. Figure 11 shows the orientation order parameter, S, of the CB groups, as a function of x. For the raw materials before PPIPS, S was between 0.4 and 0.6 at $\tau < \tau_{NI}$ and zero at $\tau > \tau_{NI}$, irrespective of the samples without or with CLs. The orientation order of the CB groups was influenced by the progress of PPIPS, and this influence was enhanced by the addition of CLs. Without CLs, as shown in Figure 11a, the low-temperature S is the same as for the raw materials, independent of x. On the other hand, the high-temperature S monotonically increased and closely approached the low-temperature value with increasing x. This indicates that polymers disturbed thermal motion of the CB-built LCs, and the number of CB groups immobile in the RMs increased proportionally to x. With CLs, as shown in Figure 11b, the low-temperature S decreased more steeply than without CLs as x increased, whereas the high-temperature S increased less steeply. Comparing dependence of S on x between samples without and with CLs, polymer network formation by CLs most likely disordered the orientation of the CB groups possessed by the RMs rather than the LCs. Combining this idea with the POM images of nonuniform optical anisotropy (Figures 7 and 9), we propose diagrams of the microscopic structures without and with CLs, as shown in Scheme 3.

The reflective scattering intensity at $\tau > \tau_{NI}$ had a maximum when x was about 25 mol % and y was 1 mol %. This was

https://doi.org/10.1021/acsami.1c10377
ACS Appl. Mater. Interfaces 2021, 13, 41066−41074

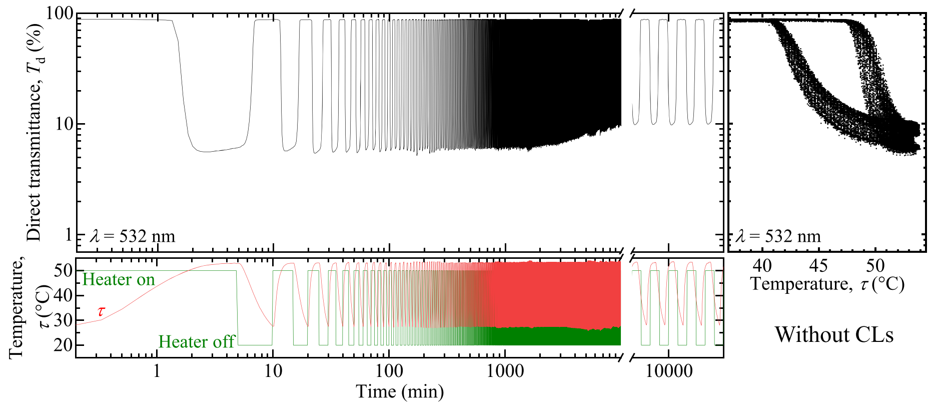

(a)

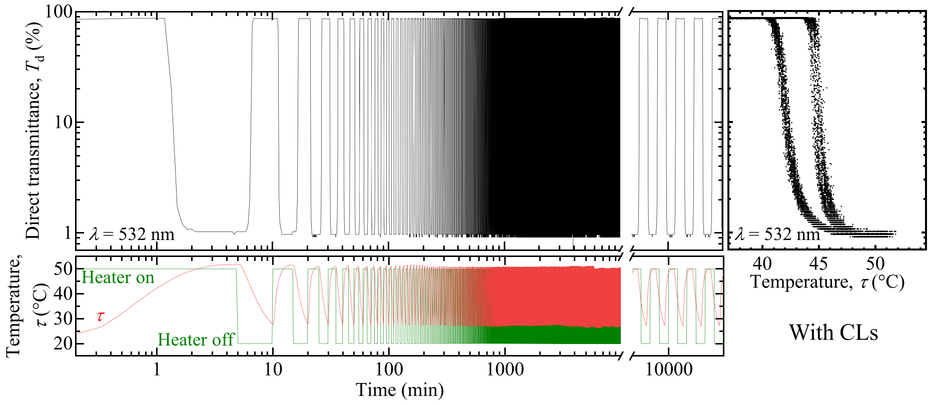

(b)

Figure 6. Direct transmittance (T_d) measured during heating and cooling repetitions for samples of x = 70 mol % (a) without CLs (y = 0 mol %) and (b) with CLs (y = 1 mol %). T_d is plotted as a function of time and temperature (τ). Each cycle consisted of 5 min heating and cooling, as shown by the green line and the red curve. A hysteresis in T_d–τ appeared because there was a difference in temperatures at the measured locations between T_d and τ, which were 1 cm away from each other.

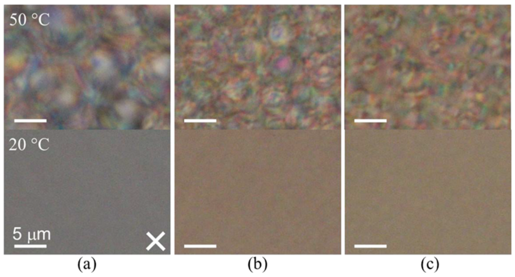

Figure 7. Polarizing optical microscope (POM) images of the samples without CLs observed at temperatures τ of 20 and 50 °C, where the lengths of the scale bars indicate 5 μm. The cross symbol indicates the azimuths of two polarizers in the crossed Nicol state, where the rubbing-treatment direction is horizontal in the images. For these mixtures, cross-linkers (CLs) were absent (i.e., y = 0 mol %), while the reactive mesogen (RM) components x in each mixture were (a) 15, (b) 30, and (c) 50 mol %.

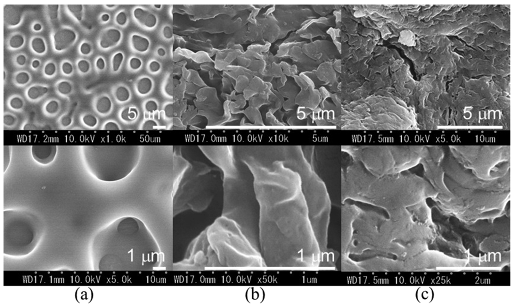

Figure 8. Scanning electron microscope (SEM) images of samples without CLs. The values of x are (a) 15, (b) 30, and (c) 50 mol %. The samples were observed at different magnifications, where the lower micrographs are close-ups of the upper ones. See the scale bars.

achieved by the optical structure (Scheme 3b), that is, first, meso-scale domains of nonuniform structures (Figures 9b and 10b), second, a large thermoresponsive change in the orientation order of LCs, indicated by the green area in Figure 11, and third, polymerized RMs with high orientation-ordered CB groups, indicated by the yellow area in Figure 11. The CLs networked the polymers without decreasing the orientation order and consequently strengthened the meso-scale nonuniform structures against repeating order/disorder transitions of LC molecules, as shown in Figure 6. Note that as shown in Scheme 3a, the PNLCs without CLs satisfied the second and third structural properties but not the first one.

4. CONCLUSIONS

We developed PNLCs whose reflective scattering intensity responds to temperature. The structures consisted of wave-

https://doi.org/10.1021/acsami.1c10377
ACS Appl. Mater. Interfaces 2021, 13, 41066−41074

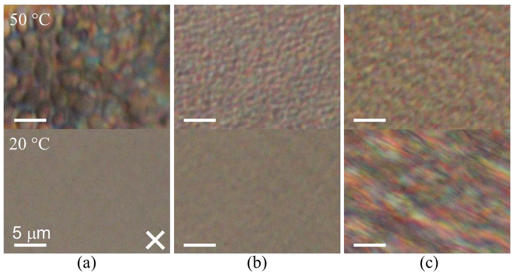

Figure 9. Polarizing optical microscope (POM) images of the samples with CLs observed at temperatures τ of 20 and 50 °C, where the lengths of the scale bars indicate 5 μm. The cross symbol indicates the azimuths of two polarizers in the crossed Nicol state, where the rubbing-treatment direction is horizontal in the images. For these mixtures, the cross-linker (CL) component y was 1 mol %, while the reactive mesogen (RM) components x in each mixture were (a) 16, (b) 30, and (c) 49 mol %.

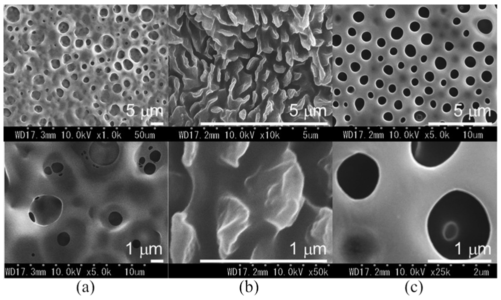

Figure 10. Scanning electron microscope (SEM) images of the samples with CLs. The values of x are (a) 15, (b) 30, and (c) 50 mol %. The samples were observed at different magnifications, where the lower micrographs are close-ups of the upper ones. See the scale bars.

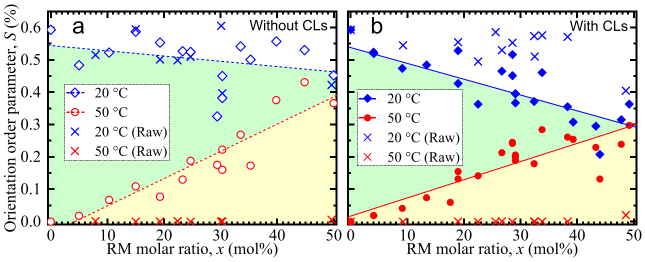

Figure 11. Orientation order parameter S as calculated using eq 3 from the IR absorption areas of −CN groups, as a function of x. The results are for the samples (a) without and (b) with CLs. The data were measured at temperatures τ of 20 and 50 °C, as respectively expressed by blue diamonds and red circles. The blue and red cross symbols in (a) and (b) represent S at τ of 20 and 50 °C, respectively, for the raw mixtures without and with CLs. The S data were fitted by a linear function, as expressed by dashed and solid lines. The values of S at 50 °C and the difference in S between 20 and 50 °C are highlighted by the yellow and green areas, respectively.

length- or meso-scale phase separation domains of LCs and polymerized RMs accompanied by CB groups that were transformed between uniaxially orientation-co-ordered and disordered states at temperatures below and above the NI phase transition temperature, respectively. Such meso-scale anisotropic structures with co-ordered CB groups, which were thermally stabilized by CLs, were formed through PPIPS conducted by ultraviolet exposure. The structures produced thermoresponsive switchability between nonscattering and

Scheme 3. Schematic Structures of Polymer Network Liquid Crystals (PNLCs) (a) without and (b) with Cross-Linkers (CLs)[a]

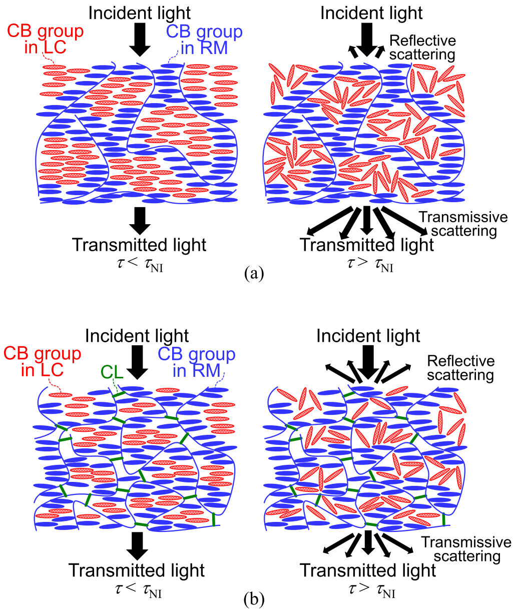

[a]The red and blue ovals express cyanobiphenyl (CB) groups possessed by LCs and RMs, respectively, as shown in Scheme 1. Without CLs, large domains of phase separation are formed by the wriggling and entanglement of reactive mesogen (RM) polymer chains around liquid crystals (LCs). Orientations of CB groups are uniaxially co-ordered in LCs and RMs due to the rubbing treatment at temperatures τ below NI phase transition temperature τ_{NI}. When $\tau > \tau_{NI}$, orientations of CB groups in the LC phase are disordered, whereas that in the RM polymer chains remains ordered. This thermoresponsive transformation produces the change in optical structure between uniformity and nonuniformity and consequently a change in transmissive light scattering. With CLs, the domain size decreases because of cross-linking the RM polymer chains. The orientation order of the CB groups thermoresponsively changes in the same way as that without CLs, although it is less effective than without CLs at $\tau < \tau_{NI}$ because the CLs form networks of RM polymer chains. This thermoresponsive transformation produces the observed change in reflective light scattering.

reflective scattering states, resulting from the existence of thermally mobile and immobile CB groups in the LC and RM phases, respectively. The thermoresponsive PNLCs possess simple structures that are easy to manufacture, install, and operate and have performances of a 19.5% thermoresponsive change in solar transmittance and 89.1% visible transparency at a low-temperature state. The feasibility for practical smart windows was demonstrated by examining solar controllability, flexibility in temperature dependence, and durability against heating and cooling repetitions.

■ ASSOCIATED CONTENT

Supporting Information

The Supporting Information is available free of charge at https://pubs.acs.org/doi/10.1021/acsami.1c10377.

Thickness dependence of direct luminous transmittance (T_{lum}) and hemispheric solar transmittance (T_{sol}) (Figure S1); temperature dependence of direct transmittance (T_d) at a wavelength of 550 nm for the samples with CLs, containing LC materials with different NI

https://doi.org/10.1021/acsami.1c10377
ACS Appl. Mater. Interfaces 2021, 13, 41066−41074

transition temperatures (τ_{NI}) (Figure S2); POM images of a sample with CLs at temperatures of 20 °C in the crossed Nicol states with two polarizers at polarization azimuths of 45 and 0° from the direction of the rubbing treatment (Figure S3) (PDF)

Sample appearance during thermoresponsive behavior of PNLCs without CLs placed at the window (Movie S1) (MP4)

Sample appearance during thermoresponsive behavior of PNLCs with CLs placed at the window (Movie S2) (MP4)

AUTHOR INFORMATION

Corresponding Author

Hiroshi Kakiuchida − *Innovative Functional Materials Research Institute, National Institute of Advanced Industrial Science and Technology, Nagoya 463-8560, Japan;* orcid.org/0000-0002-0084-2545; Phone: +81-52-736-7528; Email: h.kakiuchida@aist.go.jp; Fax: 7405

Authors

Masayuki Kabata − *Osaka Organic Chemical Industry Ltd., Osaka 541-0052, Japan*

Takanori Matsuyama − *Osaka Organic Chemical Industry Ltd., Osaka 541-0052, Japan*

Akifumi Ogiwara − *Department of Electronic Engineering, Kobe City College of Technology, Kobe 651-2194, Japan*

Complete contact information is available at:
https://pubs.acs.org/10.1021/acsami.1c10377

Author Contributions

All authors conceived and planned the experiments. H.K. prepared the samples by the PPIPS process and measured them. The data were examined mainly by H.K. under discussion with all authors. M.K. and T.M. synthesized crucial raw materials. A.O. analyzed the optical properties of the samples. All authors discussed the results and contributed to the final manuscript.

Notes

The authors declare no competing financial interest.

ACKNOWLEDGMENTS

Drs. Y. Yamada and M. Tazawa (AIST) gave a hint about smart windows. Prof. A. Matsuyama (Kyutech) discussed phase separation physics. This work was partly supported by JSPS KAKENHI grant numbers 19K03779 and 20K04632.

REFERENCES

(1) McConney, M. E.; Tondiglia, V. P.; Hurtubise, J. M.; Natarajan, L. V.; White, T. J.; Bunning, T. J. Thermally Induced, Multicolored Hyper-Reflective Cholesteric Liquid Crystals. *Adv. Mater.* **2011**, *23*, 1453−1457.

(2) Guo, S. M.; Liang, X.; Zhang, C. H.; Chen, M.; Shen, C.; Zhang, L. Y.; Yuan, X.; He, B. F.; Yang, H. Preparation of a Thermally Light-Transmittance-Controllable Film from a Coexistent System of Polymer-Dispersed and Polymer-Stabilized Liquid Crystals. *ACS Appl. Mater. Interfaces* **2017**, *9*, 2942−2947.

(3) Khandelwal, H.; Debije, M. G.; White, T. J.; Schenning, A. P. H. J. Electrically Tunable Infrared Reflector with Adjustable Bandwidth Broadening up to 1100 nm. *J. Mater. Chem. A* **2016**, *4*, 6064−6069.

(4) Oh, S.-W.; Baek, J.-M.; Kim, S.-H.; Yoon, T.-H. Optical and Electrical Switching of Cholesteric Liquid Crystals Containing Azo Dye. *RSC Adv.* **2017**, *7*, 19497−19501.

(5) Ke, Y.; Chen, J.; Lin, G.; Wang, S.; Zhou, Y.; Yin, J.; Lee, P. S.; Long, Y. Smart Windows: Electro-, Thermo-, Mechano-, Photo-chromics, and Beyond. *Adv. Energy Mater.* **2019**, *9*, 1902066.

(6) Kakiuchida, H.; Ogiwara, A.; Ishihara, S., Kobayashi, S., Ukai, Y. *Smart Windows, High Quality Liquid Crystal Displays and Smart Devices Volume 2: Surface Alignment, New Technologies and Smart Device Applications*; Institution of Engineering and Technology: 2019, pp. 341−359.

(7) Lai, Y.-T.; Kuo, J.-C.; Yang, Y.-J. Polymer-Dispersed Liquid Crystal Doped with Carbon Nanotubes for Dimethyl Methylphosphonate Vapor-Sensing Application. *Appl. Phys. Lett.* **2013**, *102*, 191912.

(8) Jayalakshmi, V.; Hegde, G.; Nair, G. G.; Prasad, S. K. Photo-Controlled Conformation-Assisted Permanent Optical Storage Device Employing a Polymer Network Liquid Crystal. *Phys. Chem. Chem. Phys.* **2009**, *11*, 6450−6454.

(9) Zheng, Z.; Yao, L.; Xuan, L.; Shen, D. Structural Investigations of Multiple Gratings Recorded in Polymer-Dispersed Liquid Crystals Film by Holography. *Liq. Cryst.* **2011**, *38*, 17−23.

(10) Inoue, Y.; Yoshida, H.; Inoue, K.; Shiozaki, Y.; Kubo, H.; Fujii, A.; Ozaki, M. Tunable Lasing from a Cholesteric Liquid Crystal Film Embedded with a Liquid Crystal Nanopore Network. *Adv. Mater.* **2011**, *23*, 5498−5501.

(11) Ren, H.; Fan, Y.-H.; Lin, Y.-H.; Wu, S.-T. Tunable-Focus Microlens Arrays Using Nanosized Polymer-Dispersed Liquid Crystal Droplets. *Opt. Commun.* **2005**, *247*, 101−106.

(12) Horii, Y.; Shibata, Y.; Ishinabe, T.; Fujikake, H. Polymer Distribution Control of Polymer-Dispersed Liquid Crystals by Uni-Directionally Diffused UV Irradiation Process. *IEICE Trans. Electron.* **2018**, *E101.C*, 857−862.

(13) Ishinabe, T.; Horii, Y.; Shibata, Y.; Fujikake, H. Light Distribution Control of Layer-Structured PDLC Fabricated by Using Micro Lens Structure and Anisotropically Diffused UV Light. *Opt. Express* **2019**, *27*, 13416−13429.

(14) Oh, S.-W.; Kim, S.-H.; Yoon, T.-H. Control of Transmittance by Thermally Induced Phase Transition in Guest−Host Liquid Crystals. *Adv. Sustainable Syst.* **2018**, *2*, 1800066.

(15) Zhang, W.; Froyen, A. A. F.; Schenning, A. P. H. J.; Zhou, G.; Debije, M. G.; de Haan, L. T. Temperature-Responsive Photonic Devices based on Cholesteric Liquid Crystals. *Adv. Photonics Res.* **2021**, *2*, 2100016.

(16) Yang, H.; Mishima, K.; Matsuyama, K.; Hayashi, K.; Kikuchi, H.; Kajiyama, T. Thermally bandwidth-controllable reflective polarizers from (polymer network/liquid crystal/chiral dopant) composites. *Appl. Phys. Lett.* **2003**, *82*, 2407−2409.

(17) Drzaic, P. S. Polymerization-Induce Phase Separation (PIPS). In *Liquid Crystal Dispersions, Series on Liquid Crystals*; World Scientific Publishing: 1995; Vol. *1*, pp. 33−47.

(18) Amundson, K.; van Blaaderen, A.; Wiltzius, P. Morphology and Electro-Optic Properties of Polymer-Dispersed Liquid-Crystal Films. *Phys. Rev. E* **1997**, *55*, 1646−1654.

(19) Pogue, R. T.; Natarajan, L. V.; Siwecki, S. A.; Tondiglia, V. P.; Sutherland, R. L.; Bunning, T. J. Monomer Functionality Effects in the Anisotropic Phase Separation of Liquid Crystals. *Polymer* **2000**, *41*, 733−741.

(20) Wang, H.; Gong, H.; Song, P.; Sun, J.; Guo, S.; Cao, H.; Zhang, L.; Yang, H. Reverse-Mode Polymer Dispersed Liquid Crystal Films Prepared by Patterned Polymer Walls. *Liq. Cryst.* **2015**, *42*, 1320−1328.

(21) Montgomery, G. P., Jr.; West, J. L.; Tamura-Lis, W. Light Scattering from Polymer-Dispersed Liquid Crystal Films: Droplet Size Effects. *J. Appl. Phys.* **1991**, *69*, 1605−1612.

(22) Wilson, H. R.; Eck, W. Transmission Variation Using Scattering/Transparent Switching Films. *Sol. Energy Mater. Sol. Cells* **1993**, *31*, 197−214.

(23) Barchini, R.; Gordon, J. G., II; Hart, M. W. Multiple Light Scattering Model Applied to Reflective Display Materials. *Jpn. J. Appl. Phys.* **1998**, *37*, 6662−6668.

(24) Park, S.; Hong, J. W. Polymer Dispersed Liquid Crystal Film for Variable-Transparency Glazing. *Thin Solid Films* **2009**, *517*, 3183−3186.
(25) Zhou, L.; Han, C.; Zhang, C.; Zhang, L. A Novel Optical Diffuser Based on Polymer Micro-Balls-Filled Nematic Liquid Crystal Composite Film. *RSC Adv.* **2018**, *8*, 40347−40357.
(26) Lee, J.-H.; Lee, J. J.; Lim, Y. J.; Kundu, S.; Kang, S.-W.; Lee, S. H. Enhanced Contrast Ratio and Viewing Angle of Polymer-Stabilized Liquid Crystal via Refractive Index Matching Between Liquid Crystal and Polymer Network. *Opt. Express* **2013**, *21*, 26914−26920.
(27) He, Z.; Yin, K.; Wu, S. T. Passive Polymer-Dispersed Liquid Crystal Enabled Multi-Focal Plane Displays. *Opt. Express* **2020**, *28*, 15294−15299.
(28) Ghosh, A.; Mallick, T. K. Evaluation of Optical Properties and Protection Factors of a PDLC Switchable Glazing for Low Energy Building Integration. *Sol. Energy Mater. Sol. Cells* **2018**, *176*, 391−396.
(29) Jiang, Y.; Shin, Y.; Yang, D.-K. Dual-Mode Switchable Liquid-Crystal Window. *Phys. Rev. Appl.* **2019**, *12*, No. 054037.
(30) Hemaida, A.; Ghosh, A.; Sundaram, S.; Mallick, T. K. Evaluation of Thermal Performance for a Smart Switchable Adaptive Polymer Dispersed Liquid Crystal (PDLC) Glazing. *Sol. Energy* **2020**, *195*, 185−193.
(31) Petty, G. W. *Scattering by Spheres - Mie Theory. A First Course in Atmospheric Radiation*; 2nd ed; Sundog Publishing: 2006, pp. 358−372.
(32) Kakiuchida, H.; Ogiwara, A. Reverse-Mode Thermoresponsive Light Attenuators Produced by Optical Anisotropic Composites of Nematic Liquid Crystals and Reactive Mesogens. *Opt. Mater.* **2018**, *78*, 273−278.
(33) Kakiuchida, H.; Matsuyama, A.; Ogiwara, A. Normal- and Reverse-Mode Thermoresponsive Controllability in Optical Attenuation of Polymer Network Liquid Crystals. *ACS Appl. Mater. Interfaces* **2019**, *11*, 19404−19412.
(34) Ghanadzadeh, A. Dielectric Investigations and Molecular Association in non-Mesogenic and Mesogenic Solutions. *J. Mol. Liq.* **2003**, *102*, 365−377.
(35) Peláez, J.; Wilson, M. Molecular Orientational and Dipolar Correlation in the Liquid Crystal Mixture E7: A Molecular Dynamics Simulation Study at a Fully Atomistic Level. *Phys. Chem. Chem. Phys.* **2007**, *9*, 2968−2975.
(36) Yeh, P.; Gu, C. Table 1.4 Properties of Liquid Crystals in Chapter 1. Preliminaries. In *Optics of Liquid Crystal Displays*; John Wiley & Sons: 2009, p. 23.
(37) *Standard Practice for Calculation of Photometric Transmittance and Reflectance of Materials to Solar Radiation, Annual Book of ASTM Standards*; American Society for Testing and Materials: Easton, 1987, *12*.02, E971−83.
(38) *Solar Energy-Reference Solar Spectral Irradiance at the Ground at Different Receiving Conditions*; ISO9845-1(E), 1992, pp. 1−14.
(39) Noble-Luginbuhl, A. R.; Blanchard, R. M.; Nuzzo, R. G. Surface Effects on the Dynamics of Liquid Crystalline Thin Films Confined in Nanoscale Cavities. *J. Am. Chem. Soc.* **2000**, *122*, 3917−3926.
(40) Ward, I. M. Determination of Molecular Orientation by Spectroscopic Techniques. *Adv. Polym. Sci.* **1985**, *66*, 81−115.

https://doi.org/10.1021/acsami.1c10377
ACS Appl. Mater. Interfaces 2021, 13, 41066−41074

Thermoresponsive Reflective Scattering of Meso-Scale Phase Separation Structures of Uniaxially Orientation-Ordered Liquid Crystals and Reactive Mesogens

Author: Hiroshi Kakiuchida, Masayuki Kabata, Takanori Matsuyama, et al

Publication: Applied Materials

Publisher: American Chemical Society

Date: Sep 1, 2021

Copyright © 2021, American Chemical Society

PERMISSION/LICENSE IS GRANTED FOR YOUR ORDER AT NO CHARGE

This type of permission/license, instead of the standard Terms and Conditions, is sent to you because no fee is being charged for your order. Please note the following:

- Permission is granted for your request in both print and electronic formats, and translations.
- If figures and/or tables were requested, they may be adapted or used in part.
- Please print this page for your records and send a copy of it to your publisher/graduate school.
- Appropriate credit for the requested material should be given as follows: "Reprinted (adapted) with permission from {COMPLETE REFERENCE CITATION}. Copyright {YEAR} American Chemical Society." Insert appropriate information in place of the capitalized words.
- One-time permission is granted only for the use specified in your RightsLink request. No additional uses are granted (such as derivative works or other editions). For any uses, please submit a new request.

If credit is given to another source for the material you requested from RightsLink, permission must be obtained from that source.

BACK CLOSE WINDOW

© 2022 Copyright - All Rights Reserved | Copyright Clearance Center, Inc. | Privacy statement | Terms and Conditions
Comments? We would like to hear from you. E-mail us at customercare@copyright.com

JOHN WILEY AND SONS LICENSE TERMS AND CONDITIONS

Sep 19, 2022

This Agreement between Harbin Institute of Technology -- Ruicong Zhang ("You") and John Wiley and Sons ("John Wiley and Sons") consists of your license details and the terms and conditions provided by John Wiley and Sons and Copyright Clearance Center.

License Number	5392420483733
License date	Sep 19, 2022
Licensed Content Publisher	John Wiley and Sons
Licensed Content Publication	Advanced Sustainable Systems
Licensed Content Title	Optical and Thermal Switching of Liquid Crystals for Self-Shading Windows
Licensed Content Author	Tae-Hoon Yoon, Jong-Min Baek, Sang-Hyeok Kim, et al
Licensed Content Date	Mar 2, 2018
Licensed Content Volume	2
Licensed Content Issue	5
Licensed Content Pages	6
Type of Use	Journal/Magazine
Requestor type	University/Academic
Is the reuse sponsored by or associated with a pharmaceutical or medical products company?	no
Format	Print and electronic
Portion	Figure/table
Number of figures/tables	1
Will you be translating?	No
Circulation	200 - 499
Title of new article	Advanced liquid crystal-based switchable optical devices for light protection applications: principles and strategies
Lead author	Ruicong Zhang, Zhibo Zhang, Jiecai Han, Lei Yang, Jiajun li, Zicheng Song Tianyu Wang, Jiaqi Zhu
Title of targeted journal	Light: Science & Applications
Publisher	Springer Nature
Expected publication date	Nov 2022
Portions	Figure 2
Requestor Location	Harbin Institute of Technology<br>No. 92, Xidazhi Street, Nangang District<br><br>Harbin, 150080<br>China<br>Attn: Harbin Institute of Technology
Publisher Tax ID	EU826007151
Total	**0.00 USD**
Terms and Conditions	

TERMS AND CONDITIONS

This copyrighted material is owned by or exclusively licensed to John Wiley & Sons, Inc. or one of its group companies (each a"Wiley Company") or handled on behalf of a society with which a Wiley Company has exclusive publishing rights in relation to a particular work (collectively "WILEY"). By clicking "accept" in connection with completing this licensing transaction, you agree that the following terms and conditions apply to this transaction (along with the billing and payment terms and conditions

established by the Copyright Clearance Center Inc., ("CCC's Billing and Payment terms and conditions"), at the time that you opened your RightsLink account (these are available at any time at http://myaccount.copyright.com).

Terms and Conditions

- The materials you have requested permission to reproduce or reuse (the "Wiley Materials") are protected by copyright.

- You are hereby granted a personal, non-exclusive, non-sub licensable (on a stand-alone basis), non-transferable, worldwide, limited license to reproduce the Wiley Materials for the purpose specified in the licensing process. This license, **and any CONTENT (PDF or image file) purchased as part of your order,** is for a one-time use only and limited to any maximum distribution number specified in the license. The first instance of republication or reuse granted by this license must be completed within two years of the date of the grant of this license (although copies prepared before the end date may be distributed thereafter). The Wiley Materials shall not be used in any other manner or for any other purpose, beyond what is granted in the license. Permission is granted subject to an appropriate acknowledgement given to the author, title of the material/book/journal and the publisher. You shall also duplicate the copyright notice that appears in the Wiley publication in your use of the Wiley Material. Permission is also granted on the understanding that nowhere in the text is a previously published source acknowledged for all or part of this Wiley Material. Any third party content is expressly excluded from this permission.

- With respect to the Wiley Materials, all rights are reserved. Except as expressly granted by the terms of the license, no part of the Wiley Materials may be copied, modified, adapted (except for minor reformatting required by the new Publication), translated, reproduced, transferred or distributed, in any form or by any means, and no derivative works may be made based on the Wiley Materials without the prior permission of the respective copyright owner.**For STM Signatory Publishers clearing permission under the terms of the STM Permissions Guidelines only, the terms of the license are extended to include subsequent editions and for editions in other languages, provided such editions are for the work as a whole in situ and does not involve the separate exploitation of the permitted figures or extracts,** You may not alter, remove or suppress in any manner any copyright, trademark or other notices displayed by the Wiley Materials. You may not license, rent, sell, loan, lease, pledge, offer as security, transfer or assign the Wiley Materials on a stand-alone basis, or any of the rights granted to you hereunder to any other person.

- The Wiley Materials and all of the intellectual property rights therein shall at all times remain the exclusive property of John Wiley & Sons Inc, the Wiley Companies, or their respective licensors, and your interest therein is only that of having possession of and the right to reproduce the Wiley Materials pursuant to Section 2 herein during the continuance of this Agreement. You agree that you own no right, title or interest in or to the Wiley Materials or any of the intellectual property rights therein. You shall have no rights hereunder other than the license as provided for above in Section 2. No right, license or interest to any trademark, trade name, service mark or other branding ("Marks") of WILEY or its licensors is granted hereunder, and you agree that you shall not assert any such right, license or interest with respect thereto

- NEITHER WILEY NOR ITS LICENSORS MAKES ANY WARRANTY OR REPRESENTATION OF ANY KIND TO YOU OR ANY THIRD PARTY, EXPRESS, IMPLIED OR STATUTORY, WITH RESPECT TO THE MATERIALS OR THE ACCURACY OF ANY INFORMATION CONTAINED IN THE MATERIALS, INCLUDING, WITHOUT LIMITATION, ANY IMPLIED WARRANTY OF MERCHANTABILITY, ACCURACY, SATISFACTORY QUALITY, FITNESS FOR A PARTICULAR PURPOSE, USABILITY, INTEGRATION OR NON-INFRINGEMENT AND ALL SUCH WARRANTIES ARE HEREBY EXCLUDED BY WILEY AND ITS LICENSORS AND WAIVED BY YOU.

- WILEY shall have the right to terminate this Agreement immediately upon breach of this Agreement by you.

- You shall indemnify, defend and hold harmless WILEY, its Licensors and their respective directors, officers, agents and employees, from and against any actual or threatened claims, demands, causes of action or proceedings arising from any breach of this Agreement by you.

- IN NO EVENT SHALL WILEY OR ITS LICENSORS BE LIABLE TO YOU OR ANY OTHER PARTY OR ANY OTHER PERSON OR ENTITY FOR ANY SPECIAL, CONSEQUENTIAL, INCIDENTAL, INDIRECT, EXEMPLARY OR PUNITIVE DAMAGES, HOWEVER CAUSED, ARISING OUT OF OR IN CONNECTION WITH THE DOWNLOADING, PROVISIONING, VIEWING OR USE OF THE MATERIALS REGARDLESS OF THE FORM OF ACTION, WHETHER FOR BREACH OF CONTRACT, BREACH OF WARRANTY, TORT, NEGLIGENCE, INFRINGEMENT OR OTHERWISE (INCLUDING, WITHOUT LIMITATION, DAMAGES BASED ON LOSS OF PROFITS, DATA, FILES, USE, BUSINESS OPPORTUNITY OR CLAIMS OF THIRD PARTIES), AND WHETHER OR NOT THE PARTY HAS BEEN ADVISED OF THE POSSIBILITY OF SUCH DAMAGES. THIS LIMITATION SHALL APPLY NOTWITHSTANDING ANY FAILURE OF ESSENTIAL PURPOSE OF ANY LIMITED REMEDY PROVIDED HEREIN.

- Should any provision of this Agreement be held by a court of competent jurisdiction to be illegal, invalid, or unenforceable, that provision shall be deemed amended to achieve as nearly as possible the same economic effect as the original provision, and the legality, validity and enforceability of the remaining provisions of this Agreement shall not

be affected or impaired thereby.

- The failure of either party to enforce any term or condition of this Agreement shall not constitute a waiver of either party's right to enforce each and every term and condition of this Agreement. No breach under this agreement shall be deemed waived or excused by either party unless such waiver or consent is in writing signed by the party granting such waiver or consent. The waiver by or consent of a party to a breach of any provision of this Agreement shall not operate or be construed as a waiver of or consent to any other or subsequent breach by such other party.

- This Agreement may not be assigned (including by operation of law or otherwise) by you without WILEY's prior written consent.

- Any fee required for this permission shall be non-refundable after thirty (30) days from receipt by the CCC.

- These terms and conditions together with CCC's Billing and Payment terms and conditions (which are incorporated herein) form the entire agreement between you and WILEY concerning this licensing transaction and (in the absence of fraud) supersedes all prior agreements and representations of the parties, oral or written. This Agreement may not be amended except in writing signed by both parties. This Agreement shall be binding upon and inure to the benefit of the parties' successors, legal representatives, and authorized assigns.

- In the event of any conflict between your obligations established by these terms and conditions and those established by CCC's Billing and Payment terms and conditions, these terms and conditions shall prevail.

- WILEY expressly reserves all rights not specifically granted in the combination of (i) the license details provided by you and accepted in the course of this licensing transaction, (ii) these terms and conditions and (iii) CCC's Billing and Payment terms and conditions.

- This Agreement will be void if the Type of Use, Format, Circulation, or Requestor Type was misrepresented during the licensing process.

- This Agreement shall be governed by and construed in accordance with the laws of the State of New York, USA, without regards to such state's conflict of law rules. Any legal action, suit or proceeding arising out of or relating to these Terms and Conditions or the breach thereof shall be instituted in a court of competent jurisdiction in New York County in the State of New York in the United States of America and each party hereby consents and submits to the personal jurisdiction of such court, waives any objection to venue in such court and consents to service of process by registered or certified mail, return receipt requested, at the last known address of such party.

WILEY OPEN ACCESS TERMS AND CONDITIONS
Wiley Publishes Open Access Articles in fully Open Access Journals and in Subscription journals offering Online Open. Although most of the fully Open Access journals publish open access articles under the terms of the Creative Commons Attribution (CC BY) License only, the subscription journals and a few of the Open Access Journals offer a choice of Creative Commons Licenses. The license type is clearly identified on the article.
The Creative Commons Attribution License
The Creative Commons Attribution License (CC-BY) allows users to copy, distribute and transmit an article, adapt the article and make commercial use of the article. The CC-BY license permits commercial and non-
Creative Commons Attribution Non-Commercial License
The Creative Commons Attribution Non-Commercial (CC-BY-NC)License permits use, distribution and reproduction in any medium, provided the original work is properly cited and is not used for commercial purposes.(see below)

Creative Commons Attribution-Non-Commercial-NoDerivs License
The Creative Commons Attribution Non-Commercial-NoDerivs License (CC-BY-NC-ND) permits use, distribution and reproduction in any medium, provided the original work is properly cited, is not used for commercial purposes and no modifications or adaptations are made. (see below)
Use by commercial "for-profit" organizations
Use of Wiley Open Access articles for commercial, promotional, or marketing purposes requires further explicit permission from Wiley and will be subject to a fee.
Further details can be found on Wiley Online Library http://olabout.wiley.com/WileyCDA/Section/id-410895.html

Other Terms and Conditions:

v1.10 Last updated September 2015

Questions? customercare@copyright.com or +1-855-239-3415 (toll free in the US) or +1-978-646-2777.

ADVANCED SUSTAINABLE SYSTEMS

www.advsustainsys.com

Optical and Thermal Switching of Liquid Crystals for Self-Shading Windows

*Seung-Won Oh, Sang-Hyeok Kim, Jong-Min Baek, and Tae-Hoon Yoon**

A liquid crystal light shutter whose transmittance can be controlled by ambient conditions, such as incident solar UV intensity and outdoor temperature, is demonstrated. Self-shading by optical or thermal switching between transparent and opaque states is achieved by liquid crystals doped with push–pull azobenzene and can be used for energy-saving smart windows. The self-shading light shutter is transparent during cool weather or under weak sunlight, which could save the energy used for heating and lighting, and can be switched from transparent to opaque during warm weather or under strong sunlight, saving energy used for cooling.

1. Introduction

Energy saving, especially in buildings, has garnered increasing attention, with a significant focus on windows, since buildings gain and lose a substantial amount of heat and light through them.[1–3] Technologies for light shutters have been widely studied for energy saving because of their ability to control the throughput of sunlight and solar heat into a building with a small applied electric field. Such light shutters can thus provide a comfortable environment for occupants while saving energy for heating, cooling, and artificial lighting.[4–9]

A thermally or optically switchable light shutter has also received attention recently because its configuration can be changed automatically between the transparent and translucent states by ambient light conditions without any externally applied power or signal.[10–18] Recently, we developed liquid crystal (LC) light shutters that are sunlight-switchable between the nematic (N) or chiral nematic (N*) and isotropic (I) phases. Their transmittance can be automatically modulated by ambient conditions using LCs doped with push–pull azobenzene materials.[12,18] However, these devices can be used only for privacy applications because they are initially translucent. Liang et al. also developed an LC light shutter which can be switchable between translucent and transparent states by changing temperature.[16] However, it can be used only for privacy applications because it is initially translucent.

S.-W. Oh, S.-H. Kim, J.-M. Baek, Prof. T.-H. Yoon
Department of Electronics Engineering
Pusan National University
Busan 46241, South Korea
E-mail: thyoon@pusan.ac.kr

The ORCID identification number(s) for the author(s) of this article can be found under https://doi.org/10.1002/adsu.201700164.

DOI: 10.1002/adsu.201700164

In this paper, we demonstrate an LC light shutter whose transmittance can be controlled by ambient conditions, such as incident solar UV intensity and outdoor temperature. Switching between the initial transparent state, obtained by self-aligned LCs in the chiral smectic A (SmA*) phase, and thermally and optically induced opaque states, which can be obtained by self-aligned LCs in the N* phase, can be used for energy-saving windows. The transparent SmA* phase allows saving of energy used for heating and lighting during cool weather or under weak sunlight. The LC cell can be switched from the transparent SmA* phase to the opaque N* phase when the temperature is warm or under strong sunlight, saving energy used for cooling. In addition, such an LC cell can be switched between the transparent and opaque states by applying an electric field without any change of the ambient conditions. Moreover, thanks to self-assembly property of the LC molecules, it can be fabricated easily by filling the LC mixture into an empty cell.

2. Design of a Self-Shading Light Shutter

In our self-shading approach for a light shutter, switching between the transparent and opaque states is achieved by adding a push–pull azobenzene 2-(4-Hydroxyphenylazo)benzoic acid (HABA) to the LC mixture (**Figure 1**a). This azobenzene, which contains two benzene rings with a donor–acceptor, undergoes a transition from *trans*- to *cis*-azobenzene when triggered by UV light, and the opposite transition in the dark.[10,19,20] Once the LCs doped with azobenzene are irradiated by UV light and the trans–cis photo-isomerization takes place, the bent shape of the *cis*-azobenzene introduces molecular disorder in the LC mixture. To support energy-saving applications, we can use the SmA* phase with *trans*-azobenzene as a transparent state and the N* phase with *cis*-azobenzene as an opaque state. The opaque N* phase can be obtained with a homeotropic boundary condition in an LC cell.

Figure 1b depicts the specular transmittances, haze values, and polarized optical microscopy (POM) images of LC cells with the homeotropic boundary condition as functions of the number of pitches. As the number of pitches is increased, the specular transmittance decreases and the haze gradually increases. When the number of pitches is greater than 4, the chiral torque is strong enough to overcome homeotropic surface anchoring, and the focal conic state can be observed so that printed texts can be hidden (Figure S1, Supporting

 © 2018 WILEY-VCH Verlag GmbH & Co. KGaA, Weinheim

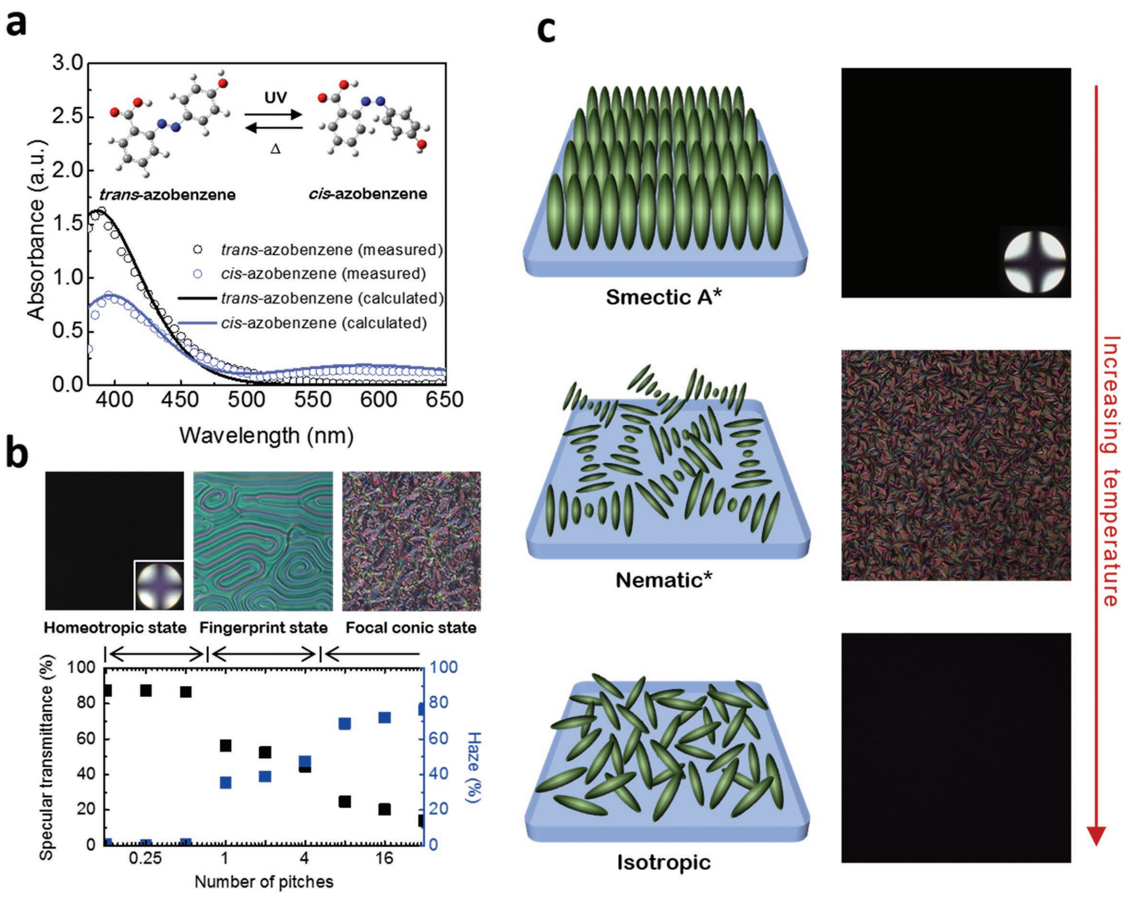

Figure 1. Design of self-aligned LC mixture for a self-shading light shutter. a) The calculated and measured absorption spectra of azobenzene HABA. b) Specular transmittances, haze values, and POM images of LC cells with vertical anchoring at surfaces as functions of the number of pitches. LC cells (10 µm thick) were fabricated using an achiral nematic LC (E7) and a chiral dopant (S811). c) Schematic illustrations and POM images of the self-aligned LC mixture in the SmA*, N*, and I phases.

Information).[18,21,22] In the SmA* phase, LCs are vertically self-aligned, as demonstrated by polarized microscope and conoscopic images in Figure 1c, because of the natural property of LCs in the SmA* phase and the homeotropic boundary condition.[23,24]

Thanks to the self-alignment of LCs, the device can be fabricated simply by injection of the LC mixture into an empty cell without alignment treatments, UV curing, formation of structures, or integration of devices (Figure S2, Supporting Information). Our approach to a self-shading light shutter is shown in **Figure 2**a. The phase of LCs in the fabricated cell can be changed from the SmA* to the N* phase with heating, and vice versa with cooling. Moreover, using UV irradiation, the phase of LCs can be changed from the SmA* to the N* phase by trans–cis photo-isomerization of azobenzene molecules. Once UV irradiation is removed, the LCs rapidly relaxes from the N* phase to the SmA* phase by cis–trans back-isomerization of the azobenzene molecules because the LC mixture contains the push–pull azobenzene which has a fast thermal relaxation property.

To absorb the incident light, we doped LC mixture with the dichroic dye S428 (Mitsui, Japan). In the initial SmA* phase, the LC and dye molecules are aligned perpendicular to the substrates. We use this phase as a transparent state because both light absorption and scattering are minimized. In both thermally and optically induced N* phases, the LC and dye molecules are randomly distributed because of the presence of focal conic domains. We can use both phases as opaque states because light absorption and scattering occur at the same time. Photographs of a light shutter in the thermally induced opaque, transparent, and optically induced opaque states are shown in Figure 2b and the device performance is summarized in **Table 1**. The thermally induced opaque state shows nearly the same total transmittance and haze as the optically induced opaque state.

3. Energy Saving with a Self-Shading Light Shutter

The phase transition of the LCs is affected by the UV intensity. The threshold UV intensity for the SmA*–N* phase transition was ≈2 mW cm^{-2} (**Figure 3**a). The transition from the N* to the SmA* phase can be achieved without any external power or light. Both SmA*–N* and N*–SmA* transitions took place in ≈30 s (Figure S6, Supporting Information). Figure 3b shows the variation in transmittance with temperature of the fabricated

© 2018 WILEY-VCH Verlag GmbH & Co. KGaA, Weinheim

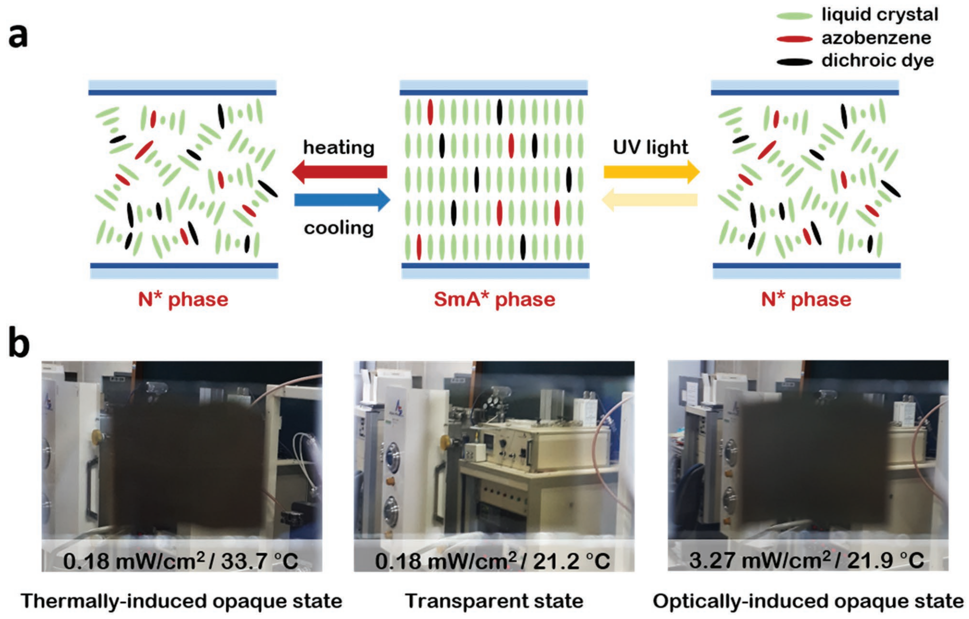

Figure 2. A self-shading light shutter. a) Schematic representation. b) Photographs.

LC cell. The photothermal effect of azobenzene, which can increase the temperature of a window, can be ignored because of a low intensity of 3 mW cm^{-2} in our experiment.[24] Without UV irradiation, the transmittance abruptly decreased at ≈29 °C during the heating process. This temperature is close to the SmA–N transition temperature. With UV irradiation, the transmittance abruptly decreased at ≈19 °C during the heating process. In other words, the phase transition in the LC mixture between the SmA* and N* phases can be controlled by UV light for temperatures ranging from 19 to 29 °C. Although the switching temperature range of the fabricated LC cell is currently rather narrow, we expect that it can be widened by developing new azobenzene materials.

The measured transmission spectra of the fabricated LC cell ranging from 300 to 1100 nm are shown in Figure 3c. The thermally induced opaque state shows nearly the same specular transmittance as the optically induced opaque state. A decrease of the transmittance for shorter wavelengths and an increase of the transmittance for longer wavelengths were observed in all states because of the absorption properties of push–pull azobenzene HABA and the dichroic dye S428 (Figure S4, Supporting Information). The yellowish color in the transparent state can be solved by employing an azobenzene material which is transparent over the entire visible spectra.

A simple illustration of a self-shading window whose light absorption and scattering are dependent on environmental

Table 1. Optical characteristics of the fabricated light shutter.

Phase	T_t [%]	T_s [%]	H [%]
SmA*	64.3	64.0	0.5
Thermally induced N*	23.3	17.1	73.4
Optically induced N*	22.1	16.6	75.1

conditions is shown in **Figure 4**a. The window is transparent at a low temperature or under weak sunlight, saving energy used for heating and lighting. At a high temperature or under strong sunlight, the window can be switched from the transparent to the opaque state, saving energy used for cooling. We should note that the transition between these states was achieved without any external power or signal.

To verify the practical operation of a self-shading window, the fabricated LC cell was attached to an outdoor window for 1 d. At a low UV intensity of 1.22 mW cm^{-2} in the morning, the cell was transparent, and the background was clearly visible. An increased UV intensity of 2.25 mW cm^{-2} resulted in an invisible background image because of the increased opacity of the cell. Under a threshold UV intensity of 1.73 mW cm^{-2} and an outdoor temperature of 30.3 °C, the cell was opaque and blocked the view due to the thermally induced phase change. When both UV intensity and temperature were low because of cloud cover or when there was little sunlight at sunset, the cell rapidly relaxed from the opaque state back to its initial transparent state. This reversible transition of a self-shading window between the transparent and opaque states can be repeated many times without any deterioration in performance (Figure S8, Supporting Information).

To understand the potential impact of a self-shading window on energy demand in a building, we simulated an office building (Tables S1 and S2, Supporting Information). We analyzed these dynamic whole-building performance predictions to determine the effect of a self-shading window on potential primary energy saving for heating, cooling, and artificial lighting. Three different climates were chosen to understand the relationship between the environmental conditions and subsequent energy saving in the buildings when using a self-shading window. The results were compared with reference configurations employing a double-glazed window and the opaque state of a self-shading window as a static dark window.

© 2018 WILEY-VCH Verlag GmbH & Co. KGaA, Weinheim

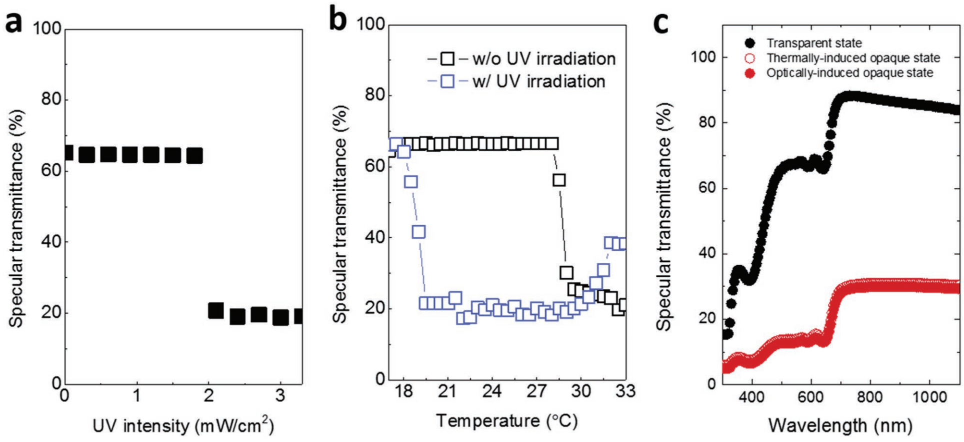

Figure 3. Switching properties of the fabricated light shutter. a) The specular transmittance as a function of the UV intensity. b) Changes in the temperature dependence of the specular transmittance by UV irradiation. c) Transmission spectra.

Our simulations suggest that the impact of a self-shading window in office buildings depends on the local environmental conditions. In San Francisco, which has a warm and sunny climate throughout the year, the application of static dark windows led to saving of cooling energy comparable to that of a double-glazed window. In contrast, in climates dominated by the need for building heating, such as Ottawa, the decrease in warming of the building interior by using static dark windows would lead to an increase in the energy demand required for heating. The results show that windows should be selected considering climatic or seasonal influences to reduce energy consumption because there are trade-offs between heating or lighting and cooling, depending on the solar heat gain and transmittance of windows. On the other hand, the simulation reveals that a self-shading window shows the lowest energy consumption for all areas because it can be switched between the transparent and opaque states by the ambient conditions without any trade-offs. Moreover, because a self-shading window can be switched without applying an electric field, we do not need to account for the energy consumed in operation of the window.

4. Electro-Optic Characteristics

In addition, the light shutter can be switched between the transparent and opaque states without any phase transition by applying an electric field. **Figure 5** shows a schematic illustration of the electrical switching and electro-optic properties of the fabricated light shutter. The switching between transparent and opaque states can be achieved by the electro-hydrodynamic

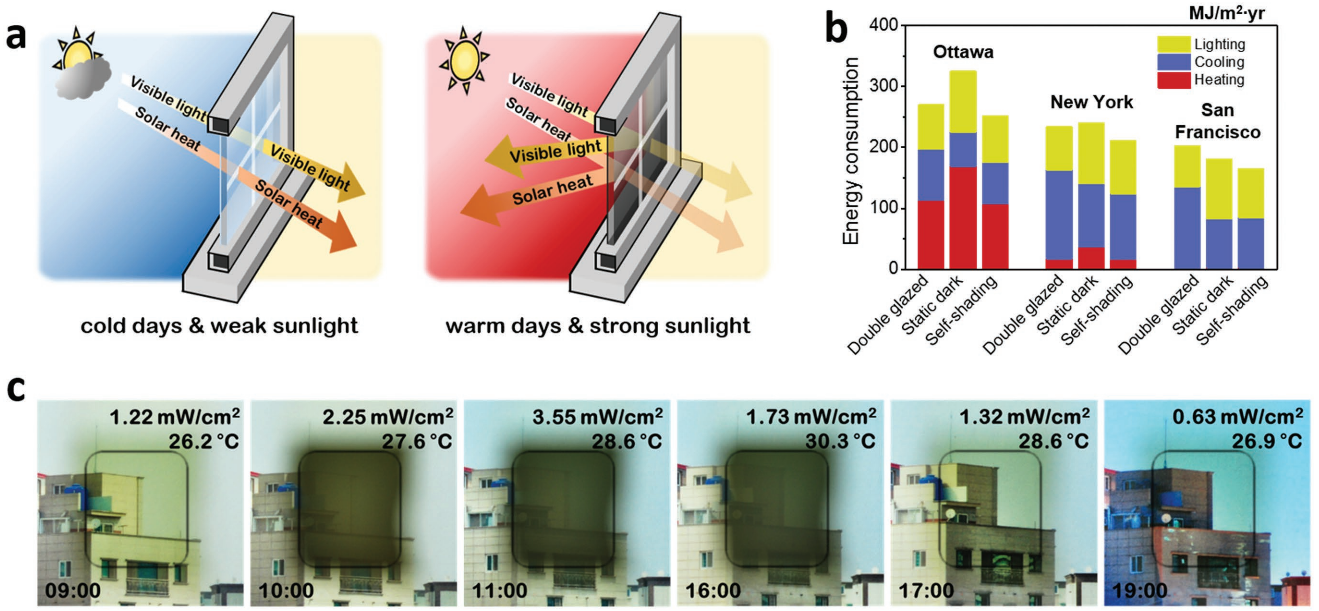

Figure 4. A self-shading window for energy saving. a) Schematic representation of energy saving. b) Comparison of energy consumption for a normal double-glazed window, a static dark window, and a self-shading window for three different climates. c) Photographs of the outdoor switching test.

 © 2018 WILEY-VCH Verlag GmbH & Co. KGaA, Weinheim

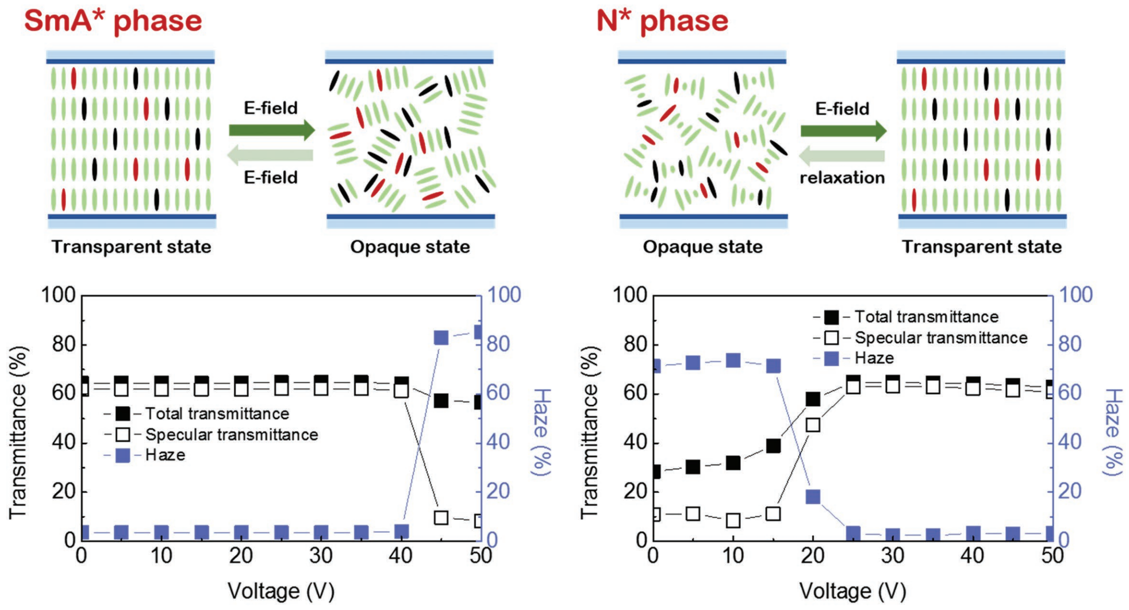

Figure 5. Electrical switching properties of the fabricated light shutter.

effect [dielectric interaction] in the SmA* [N*] phase. In the SmA* phase, the application of a low-frequency electric field of 100 Hz induces an opaque state due to the motion of the ionic material hexadecyltrimethylammonium bromide (HTAB) used for the homeotropic boundary condition, whereas the application of a high-frequency electric field of 1 kHz induces a transparent state.[25–27] The opaque N* phase is switched to the transparent state by vertically orienting the LCs along the direction of the applied 1 kHz electric field.

5. Conclusion

We demonstrated optical and thermal switching of LCs doped with push–pull azobenzene. Self-shading with an LC cell that is switchable by ambient conditions can be used for energy-saving smart windows. During cool weather or under weak sunlight, such windows can save energy used for heating and lighting, and similarly they can save energy used for cooling during warm weather or under strong sunlight. They can also be switched between the transparent and opaque states without any change of the ambient conditions by applying an electric field. Moreover, thanks to their self-assembly property, they can be easily fabricated by filling the LC mixture into an empty cell.

6. Experimental Section

Materials: All chemicals were purchased from commercial suppliers and used without further purification. The LC mixture was composed of achiral LC 8CB (4-cyano-4′-octylbiphenyl), ionic dopant HTAB, chiral dopant S6N (HCCH, China), push–pull azobenzene HABA, and black dichroic dye S428 (Mitsui, Japan). The weight ratios of 8CB/HTAB/S6N/HABA/S428 were 93.68/0.94/2.57/0.94/1.87. We used 8CB (Sigma-Aldrich), exhibiting the SmA* phase at room temperature, doped with a left-handed chiral dopant S6N (HCCH, China) with high helical twisting power to avoid a dramatic decrease of the clearing temperature. The ionic dopant HTAB (Sigma-Aldrich) was used for the homeotropic boundary condition, and push–pull azobenzene HABA (Sigma-Aldrich) was used for the optically induced phase change.

Fabrication of a Self-Shading Light Shutter: The LC mixture was stirred continuously for 24 h at room temperature. An electric field was applied for electrical switching, with the top and bottom substrates containing transparent indium-tin-oxide electrodes. The mixture was filled in a 20 µm thick cell via capillary action at room temperature.

Absorption Spectra of Azobenzene: LC 5CB was used as a solvent for the measurement and calculation of the absorption spectra of the azobenzene HABA. The absorption spectra were measured using a spectrometer (MCPD 3000, Photal) over the wavelength range of 380–780 nm. The spectrum for *cis*-azobenzene was measured under irradiation by unpolarized UV light at 365 nm using a mercury arc lamp (Osram HBO 103 W/2). Calculations were performed using the Gaussview program, supplemented with the standard 6-31G* basis set (referred to as DFT calculations).

Threshold UV Intensity: The effect of UV intensity on the specular transmittance of the fabricated LC cell was carried out at 25 °C using a Mettler FP82 hot-stage and a Mettler FP90 controller. The UV intensity was varied from 0 to 4 mW cm^{-2} with unpolarized UV light at 365 nm using a mercury arc lamp (Osram HBO 103 W/2).

Switching Temperature Range and Phase Transition: The measurements were carried out using a Mettler FP82 hot-stage and a Mettler FP90 controller. To investigate the switching temperature of the fabricated LC cell, we measured the specular transmittance with and without UV irradiation as a function of temperature. The LC cell was exposed to unpolarized UV light at 365 nm with an intensity of 3 mW cm^{-2}.

Scattering and Absorption by the Light Shutter: The total transmittance, specular transmittance, and haze of the fabricated LC cell were measured using a haze meter (HM-65W, Murakami Color Research Laboratory). The specular [diffuse] transmittance T_s [T_d] refers to the ratio of the power of the beam that emerges from a sample cell, which is parallel (within a small 2.5° range) [not parallel] to a beam entering the cell, to the power carried by the beam entering the sample, as shown in Figure S9 in the Supporting Information. The total transmittance T_t is the sum of the specular transmittance T_s and the diffuse transmittance T_d. The haze H can be calculated as the ratio $H = T_d/T_t$.

 © 2018 WILEY-VCH Verlag GmbH & Co. KGaA, Weinheim

Calculation for Energy Saving: The window properties used in the numerical simulations were obtained using the software package WINDOWS7.5 (Table S1, Supporting Information). Using COMFEN5, a computer-based analytical software tool developed by Lawrence Berkeley National Laboratory, we calculated energy consumption in three different climates: (1) Ottawa, Canada, (2) New York, United States, and (3) San Francisco, United States. The operating temperature and UV intensity exposed to the self-shading window were set as 29 °C and 2 mW cm^{-2}, respectively. The parameters describing the building and the energy usage scenarios are listed in detail in Table S2 in the Supporting Information.

Supporting Information

Supporting Information is available from the Wiley Online Library or from the author.

Acknowledgements

This work was supported by the National Research Foundation of Korea (NRF) grant funded by the Korean government (MSIP) (No. 2017R1A2A1A05001067).

Conflict of Interest

The authors declare no conflict of interest.

Keywords

energy saving, liquid crystals, push–pull azobenzene, self-shading, smart window

Received: November 13, 2017
Revised: January 23, 2018
Published online: March 2, 2018

[1] C. E. Ochoa, M. B. C. Aries, E. J. van Loenen, J. L. M. Hensen, *Appl. Energy* **2012**, *95*, 238.
[2] M. P. Gutierrez, L. P. Lee, *Science* **2013**, *341*, 247.
[3] H. Khandelwal, A. P. H. J. Schenning, M. G. Debije, *Adv. Energy Mater.* **2016**, *7*, 1602209.
[4] A. Llordés, G. Garcia, J. Gazquez, D. J. Milliron, *Nature* **2013**, *500*, 323.
[5] Z. Xie, X. Jin, G. Chen, J. Xu, D. Chen, G. Shen, *Chem. Commun.* **2014**, *50*, 608.
[6] C.-C. Wu, J.-C. Liou, C.-C. Diao, *Chem. Commun.* **2015**, *51*, 12625.
[7] H. Shin, S. Seo, C. Park, J. Na, M. Han, E. Kim, *Energy Environ. Sci.* **2016**, *9*, 117.
[8] A. Llordés, Y. Wand, A. Fernadez-Martinez, P. Xiao, T. Lee, A. Poulain, O. Zandi, C. A. S. Cabezas, G. Henkelman, D. J. Milliron, *Nat. Mater.* **2016**, *15*, 1267.
[9] G. Cai, P. Darmawan, X. Cheng, P. S. Lee, *Adv. Energy Mater.* **2017**, *7*, 1602598.
[10] H.-K. Kwon, K.-T. Lee, K. Hur, S. H. Moon, M. M. Quasim, T. D. Wilkinson, J.-Y. Han, H. Ko, I.-K. Han, B. Park, B. K. Min, B.-K. Ju, S. M. Morris, R. H. Friend, D.-H. Ko, *Adv. Energy Mater.* **2015**, *5*, 1401347.
[11] E. Lee, D. Kim, J. Yoon, *ACS Appl. Mater. Interfaces* **2016**, *8*, 26359.
[12] S.-W. Oh, J.-M. Baek, T.-H. Yoon, *Opt. Express* **2016**, *24*, 26575.
[13] K. G. Gutierrez-Cuevas, L. Wang, Z. Zheng, H. K. Bisoyi, G. Li, L.-S. Tan, R. A. Vaia, Q. Li, *Angew. Chem., Int. Ed.* **2016**, *55*, 13090.
[14] H. K. Bisoyi, Q. Li, *Chem. Rev.* **2016**, *116*, 15089.
[15] L. Wang, H. K. Bisoyi, Z. Zheng, K. G. Gutierrez-Cuevas, G. Singh, S. Kumar, T. J. Bunning, Q. Li, *Mater. Today* **2017**, *20*, 230.
[16] X. Liang, S. Guo, M. Chen, C. Li, Q. Wang, C. Zou, C. Zhang, L. Zhang, S. Guo, H. Yang, *Mater. Horiz.* **2017**, *4*, 878.
[17] S. Bogati, R. Basnet, W. Graf, A. Georg, *Sol. Energy Mater. Sol. Cells* **2017**, *166*, 204.
[18] S.-W. Oh, J.-M. Baek, S.-H. Kim, T.-H. Yoon, *RSC Adv.* **2017**, *7*, 19497.
[19] J. García-Amorós, D. Velasco, *Beilstein J. Org. Chem.* **2012**, *8*, 1003.
[20] M. A. Kienzler, A. Reiner, E. Trautman, S. Yoo, D. Trauner, E. Y. Isacoff, *J. Am. Chem. Soc.* **2013**, *135*, 17683.
[21] P. E. Cladis, M. Kléman, *Mol. Cryst. Liq. Cryst.* **1972**, *16*, 1.
[22] P. Oswald, J. Baudry, S. Pirkl, *Phys. Rep.* **2000**, *337*, 67.
[23] I. Dierking, S. T. Lagerwall, *Liq. Cryst.* **1999**, *26*, 83.
[24] K. M. Lee, T. J. White, *Macromolecules* **2012**, *45*, 7163.
[25] D. J. Gardiner, S. M. Morris, H. J. Coles, *Sol. Energy Mater. Sol. Cells* **2009**, *93*, 301.
[26] G. H. Heilmeier, L. A. Zanoni, L. A. Barton, *Appl. Phys. Lett.* **1968**, *13*, 46.
[27] G. H. Heilmeier, L. A. Zanoni, L. A. Barton, *IEEE Trans. Electron Devices* **1970**, *17*, 22.

© 2018 WILEY-VCH Verlag GmbH & Co. KGaA, Weinheim
