# Supplementary material for: Advanced liquid crystal-based switchable optical devices for light protection applications: principles and strategies
Source: Light Sci Appl. 2023 Jan 3;12:11. doi: 10.1038/s41377-022-01032-y (PMC9807646; doi:10.1038/s41377-022-01032-y)
Supplement: Supplementary file 16 — Fig 18 copyright promotion [file 41377_2022_1032_MOESM16_ESM.pdf]

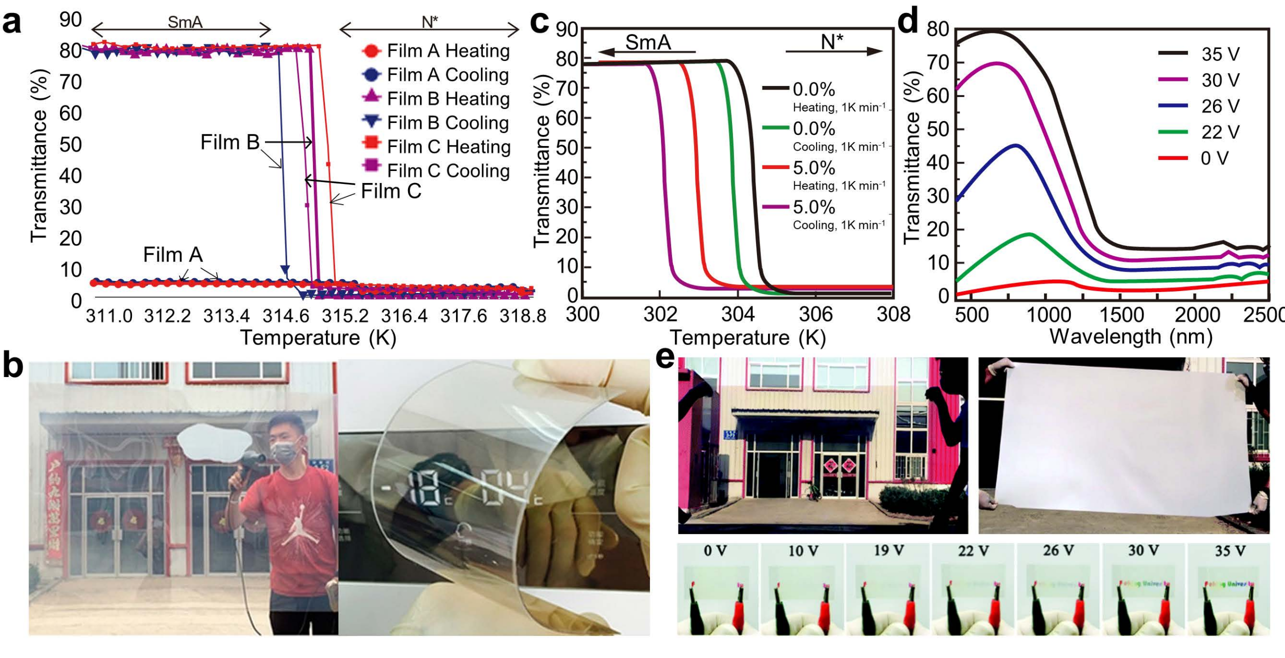

a
SmA
N*
Film A Heating
Film A Cooling
Film B Heating
Film B Cooling
Film C Heating
Film C Cooling
Film B
Film C
Film A
Transmittance (%)
Temperature (K)
311.0 312.2 313.4 314.6 315.2 316.4 317.6 318.8
0 10 20 30 40 50 60 70 80 90
b
c
SmA
N*
0.0% Heating, 1K min-1
0.0% Cooling, 1K min-1
5.0% Heating, 1K min-1
5.0% Cooling, 1K min-1
Transmittance (%)
Temperature (K)
300 302 304 306 308
d
35 V
30 V
26 V
22 V
0 V
Transmittance (%)
Wavelength (nm)
500 1000 1500 2000 2500
e
0 V
10 V
19 V
22 V
26 V
30 V
35 V

ACS APPLIED MATERIALS & INTERFACES

Research Article

www.acsami.org

Preparation of a Thermally Light-Transmittance-Controllable Film from a Coexistent System of Polymer-Dispersed and Polymer-Stabilized Liquid Crystals

Shu-Meng Guo,[†] Xiao Liang,[‡,§] Cui-Hong Zhang,[‡,§] Mei Chen,[‡,§] Chen Shen,[∥] Lan-Ying Zhang,[‡,§] Xiao Yuan,[‡,§] Bao-Feng He,[‡,§] and Huai Yang*[,†,‡,§]

[†]Department of Materials Physics and Chemistry, University of Science and Technology Beijing, Beijing 100083, P. R. China
[‡]Department of Materials Science and Engineering, College of Engineering, Peking University, Beijing 100871, P. R. China
[§]Key Laboratory of Polymer Chemistry and Physics of Ministry of Education, Peking University, Beijing 100871, P. R. China
[∥]Department of Environmental Science and Engineering, Fudan University, Shanghai 200000, P. R. China

Supporting Information

Downloaded via HARBIN INST OF TECHNOLOGY on September 19, 2022 at 10:01:18 (UTC).
See https://pubs.acs.org/sharingguidelines for options on how to legitimately share published articles.

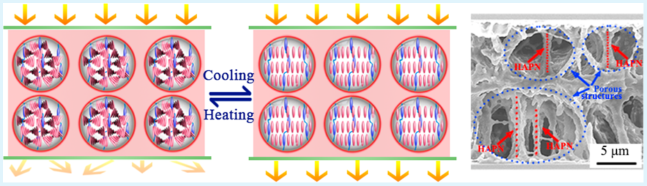

ABSTRACT: Polymer-dispersed liquid crystal (PDLC) and polymer-stabilized liquid crystal (PSLC) systems are the two primary distinct systems in the field of liquid crystal (LC) technology, and they are differentiated by their unique microstructures. Here, we present a novel coexistent system of polymer-dispersed and polymer-stabilized liquid crystals (PD&SLCs), which forms a homeotropically aligned polymer network (HAPN) within the LC droplets after a microphase separation between the LC and polymer matrix and combines the advantages of both the PDLC and PSLC systems. Then, we prepare a novel thermally light-transmittance-controllable (TLTC) film from the PD&SLC system, where the transmittance can be reversibly changed through thermal control from a transparent to a light-scattering state. The film also combines the advantageous features of flexibility and a potential for large-scale manufacturing, and it shows significant promise in future applications from smart windows to temperature sensors.

KEYWORDS: *liquid crystal, polymer-dispersed liquid crystal, polymer-stabilized liquid crystal, coexistent system, thermally light-transmittance-controllable film*

INTRODUCTION

Polymer-dispersed liquid crystal (PDLC) and polymer-stabilized liquid crystal (PSLC) systems with unique characteristics can be obtained by modifying the microstructures of polymer/liquid crystal (LC) composites.[1−10] PDLC systems are prepared by dispersing LC droplets in a polymer matrix. During the polymerization, which may be induced by UV, thermal curing, etc., the solubility of the LC in the polymer decreases, and phase separation occurs, which results in the formation of a microphase separation structure.[11−17] In a typical fabrication of a PDLC film by UV curing, a homogeneous mixture of a LC and nonliquid-crystalline photopolymerizable monomers (NLCMs) is sandwiched between two conductive substrates. A PDLC film exhibits strong light scattering because the optical axes of the LC molecules in the droplets are randomly oriented.[18] However, the light-scattering state can be switched into a transparent state by applying an electric field to the film to homeotropically reorient the directions of the LC with a positive dielectric anisotropy.[19] In a PDLC film, the polymer matrix content is usually higher than 20 wt % and, at times, even higher than 40 wt % to ensure that the mechanical strength of the flexible film is sufficient for large-scale fabrication and long-term stability.[20] On the basis of the above-mentioned characteristics, PDLC films are widely used in window films, displays, microlenses, light shutters, etc.[2,21−25]

A PSLC system, as implied by its name, is a system in which the alignment of LC molecules is stabilized by interactions between the polymer network and the LC molecules. In a typical fabrication of a PSLC device, a uniform mixture of a LC and liquid crystalline photopolymerizable monomers (LCMs) is sandwiched between indium tin oxide (ITO) substrates.[26] The initial orientation of the LC mixture (mixture of LC and LCM) can be easily controlled; in particular, the molecular directions of the LC mixture can be aligned in parallel or homeotropically by simply pretreating the inner surfaces or applying an electric or a magnetic field. During the curing process, cross-linking between the LCM molecules occurs within the LC matrix, which results in the formation of a parallel or homeotropically aligned polymer network that can stabilize the initial orientation of the LC molecules.[27,28] In general, the polymer network content in a PSLC system is usually constrained below 10%; otherwise, switching by the application of an external field becomes difficult, although the

Received: October 20, 2016
Accepted: December 21, 2016
Published: December 21, 2016

 © 2016 American Chemical Society

DOI: 10.1021/acsami.6b13366
ACS Appl. Mater. Interfaces 2017, 9, 2942−2947

mechanical properties might be enhanced to some extent.[26] To our knowledge, the large-scale fabrication of flexible films composed of a PSLC system with such above-mentioned features has not been reported.

As mentioned above, PDLC and PSLC systems have different advantages. PDLC films exhibit only a random orientation of LC molecules, and flexible films with a large area can be easily fabricated. In contrast, PSLC films can match the initial orientation of the LC, and flexible films are difficult to fabricate due to the weak shear strength of the film as a result of the low polymer network content. Here, a novel coexistent system of polymer-dispersed and polymer-stabilized liquid crystals (PD&SLCs), which combines the advantages of PDLC and PSLC systems, is developed. Moreover, using a LC with a smectic A (SmA)-chiral nematic (N*) phase transition, we first report the preparation of a novel thermally light-transmittance-controllable (TLTC) film from the PD&SLC system. The TLTC film can be thermally and reversibly switched from a transparent state to a strong light-scattering state, depending on the contrast between the environment temperature and the phase-transition temperature of the LC; in addition, the large-scale fabrication of the TLTC film is feasible. The TLTC film shows promise in a wide range of applications, such as smart window films and temperature sensors.

■ EXPERIMENTAL METHODS

Materials. Figure 1 shows the chemical structures and some physical parameters of the materials used in the experiments. SLC-1717, S811, and Irgacure 651 were purchased from Shijiazhuang Chengzhi Yonghua Display Materials Co., Ltd., Merck Co., Ltd., and TCI Co., Ltd., respectively. SLC-1717 (Cr-233.0 K-N-365.0 K-I) is a low-molar-mass nematic thermotropic LC, and S811 (Cr-320.0 K) is a left-handed chiral compound with a helical twisting power of 10.1 μm^{-1}. Meanwhile, S811 possesses good chemical stability and the miscibility with most low-molar-mass liquid crystal. The NLCMs, 3,5,5-trimethylhexyl acrylate (TMHA) and butane-1,4-diyl diacrylate (BDDA), were obtained from Alfa Aesar Co., Ltd.. The LCM, 2-methyl-1,4-phenylene bis(4-((6-(acryloyloxy) hexyl) oxy) benzoate) (C6M), and the liquid crystalline compounds, nCB and 8OCB, were synthesized according to the methods proposed by Broer et al.[29] and Gray et al.,[30] respectively. The SmA LC was a mixture of nCB and 8OCB, and the LC with the SmA-N*phase transition (Figures S1 and S2) was a mixture of SmA LC, SLC177, and S811. Glass bead spacers were purchased from Sekisui Chemical Co., Ltd.

Preparation of the Films. Mixtures of Samples A, B, and C, which are listed in Table 1, were first prepared and sandwiched between two substrates of transparent ITO-coated plastic films. The thickness of the films was adjusted to 17.5 μm using glass bead spacers. Then, an electric field square-wave (160.0 V, 0.01 Hz) was applied with a function generator (Tektronix AFG3102) and an RF power amplifier (NF HSA4051) to the sandwiched films of Samples A, B, and C, and the films were cured at approximately 298.2 K for approximately 10.0 min using a UV lamp (PS135, UV Flood, Stockholm Sweden) with an intensity of 0.1 mW/cm^2 (365 nm). The resulting films from Samples A, B, and C are referred to as Films A, B, and C, respectively.

■ MEASUREMENTS

The phase-transition temperatures were investigated by differential scanning calorimetry (DSC, A PerkinElmer DSC8000) at a heating or cooling rate of 10.0 K·min^{-1} under a dry nitrogen purge. The optical textures of the LC with a SmA-N* phase transition were observed using a polarizing optical microscope (POM, Carl Zeiss Axio Vision SE64) equipped with a hot stage (Linkam LK-600PM), which was calibrated to an accuracy of ±0.1 K.

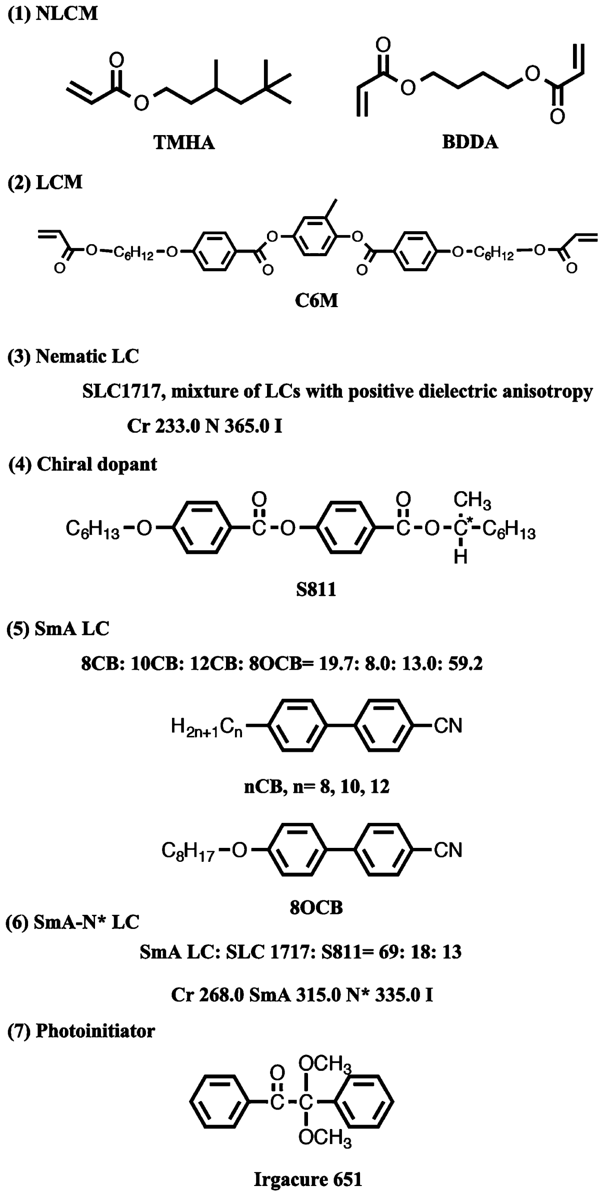

Figure 1. Chemical structures and some physical properties of the materials used: (1) TMHA and BDDA, (2) C6M, (3) SLC1717, (4) S811, and (5) 8CB, 10CB, 12CB, and 8OCB; the compositions of (6) SmA-N* LC and (7) Irgacure 651.

Table 1. Compositions of the Samples Studied[a]

sample	(TMHA + BDDA)/C6M/(SmA-N* LC) [wt %]	after irradiation
A	(16.0 + 4.0)/0/80.0	Film A
B	(0 + 0)/3.0/97.0	Film B
C	(16.0 + 4.0)/3.0/77.0	Film C

[a]In all samples, the weight ratio between the monomers and photoinitiator was 25:1.

The morphologies of the polymer network were observed using a scanning electron microscope (SEM, HITACHI S-4800). For SEM analysis of the polymer network, all films were first dipped in hexane (AR) for approximately 7 days at room temperature to fully extract the LC molecules from the films and then dried in vacuum for approximately 24 h, and finally, thin layers of gold were coated onto the films to eliminate or reduce the buildup of electric charge.

DOI: 10.1021/acsami.6b13366
ACS Appl. Mater. Interfaces 2017, 9, 2942−2947

The thermo-optical spectra were obtained by a UV/vis/NIR spectrophotometer (JASCO V-570) equipped with a hot stage (Linkam LK-600PM), which was calibrated to an accuracy of ±0.1 K. The transmittance of air was normalized as 100.0%. The shear strengths of the films were measured using a universal tensile testing machine (LETRY) at the rate of 10 mm·min^{-1}, and each of the tested films was 10 cm × 4 cm.

RESULTS AND DISCUSSION

Figure 2 shows the SEM photographs of the polymer networks of Films A, B, and C. A porous structure of polymer networks in Film A can be clearly observed from Figure 2a, which is due to polymerization between the NLCM molecules. Although an electric field was applied during the curing process, no homeotropically aligned polymer network (HAPN) had formed within the porous structures because the NLCMs could not be oriented by an electric field. Therefore, the network of Film A is the same as that formed in a typical PDLC system.[31,32] From Figure 2b, it can be observed that the fibers of the network of Film B align perpendicularly to the substrate surfaces. This is attributed to the homeotropic orientation of both the LC and LCM molecules upon the application of an electric field to Film B during the curing process; in addition, HAPNs, which are the same as the polymer networks in a typical PSLC system, were formed from the polymerization of the LCMs.[33] From Figure 2c, the formation of not only the porous structures of the polymer networks such as those in Film A but also the HAPN within the porous structure can be clearly observed in Film C, which presents a coexistence of the structures in Figure 2a,b. This novel polymer network structure, referred to here as the PD&SLC system, has never been previously reported to the best of our knowledge.

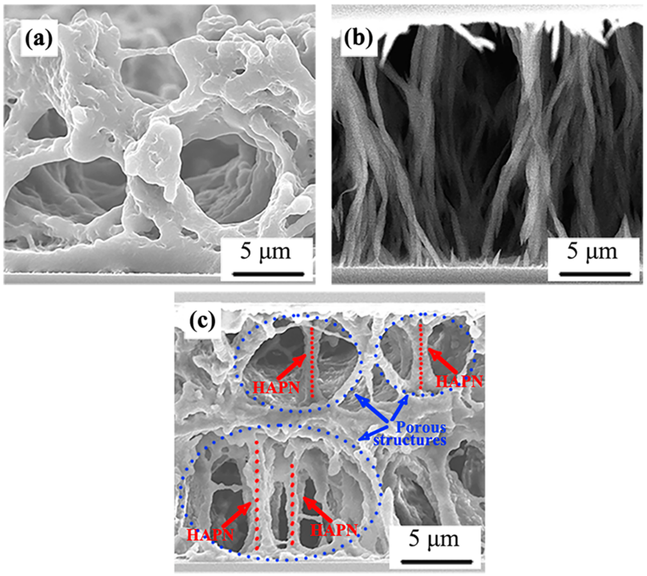

Figure 2. SEM photographs of the polymer networks of the films observed from a side view of the cells. (a) A porous structure of polymer networks of Film A. (b) The HAPN of Film B. (c) A coexistent structure of both the porous polymer networks and the HAPN of Film C.

Figure 3 shows a schematic illustration of the possible formation mechanism of the PD&SLC system. Initially, the NLCM/LCM/LC/photoinitiator mixture of Sample C was a homogeneous isotropic solution under room temperature, and its molecules could not be oriented perpendicularly to the substrate surfaces even with the application of an electric field, as shown in Figure 3a. After irradiation by UV light, cross-linking occurred between the NLCMs, between the LCMs and between the NLCM and LCM molecules in Film C. However, the cross-linking between the NLCM molecules was dominant; i.e., cross-linking primarily occurred between NLCM molecules rather than between LCMs or between NLCM and LCM molecules. This is possibly attributed to the larger flexibility of the polymer fibers formed from NLCMs than those from LCMs and from NLCM and LCM molecules. The radical addition reaction is mainly controlled by the mobility of the molecules, and the mobility of the LCM macromolecules with rigid structures is lower than that from the NLCM macromolecules with flexible segments.[34] Thus, in the initial polymerization stage, a porous structure of polymer networks, as observed in Figure 2a, was formed, and a microphase separation similar to that in a PDLC system occurred, as presented in Figure 3b. Moreover, after irradiation was conducted for approximately 3.0 min, the microstructures of the polymer network in Film C were observed by SEM (Figure S3), and the results indicate that no HAPN was formed in the porous structures. Subsequently, upon the consumption of the NLCM, the domains mainly in Film C containing the LC and LCM molecules increasingly exhibited an N* phase at approximately 298.2 K (Figure 3c), and the molecules of the LC and LCM were perpendicularly oriented by the applied electric field, as observed in Figure 3d. Simultaneously, the HAPN was formed due to the photopolymerization of the LCM, as shown in Figure 3e. Finally, most of the LCM molecules underwent polymerization, and the domains primarily contained the LC, which exhibited a SmA phase at approximately 298.2 K, as shown in Figure 3f. The final structure is a PD&SLC system. In this case, Film C is transparent, as observed in Figure 3i. However, when Film C is heated to a temperature higher than the phase-transition temperature, the N* phase of the LC forms a focal-conic texture (Figure S4c); Figure 3h shows a schematic illustration of the molecular arrangement. Thus, the film exhibits a strong light-scattering state, as shown in Figure 3j, which is due to the competition between the intrinsic spiral structure of the N* phase and the constraining effect of the HAPN.[35] At temperatures lower than the phase-transition temperature of the LC, the molecules of the LC in the SmA phase are homeotropically oriented (Figure S4a), and Film C is transparent again due to the molecular interaction between the HAPN and the LC molecules (Figure 3g). The phase-transition behavior of the liquid crystal encapsulated in the PD&SLC system at temperature changing can be proved by DSC (Figure S5). Our experiments demonstrated that a stable transparent state at room temperature can be maintained for over a year.

Figure 4 shows the temperature dependence of the transmittance of Films A, B, and C, the wavelength dependence of the transmittance of Film C measured at approximately 312.0 and 317.0 K, and the shear strengths of Films A, B, and C at approximately 298.2 K. It can be observed that the transmittance of Film A changes slightly as the temperature increases, as observed in Figure 4a, which is a common characteristic of a PDLC system. However, a sharp change from a transparent to a strong light-scattering state can be observed for Films B and C within a temperature range of approximately 0.3 K when the LC is heated from the SmA to the N* phase,

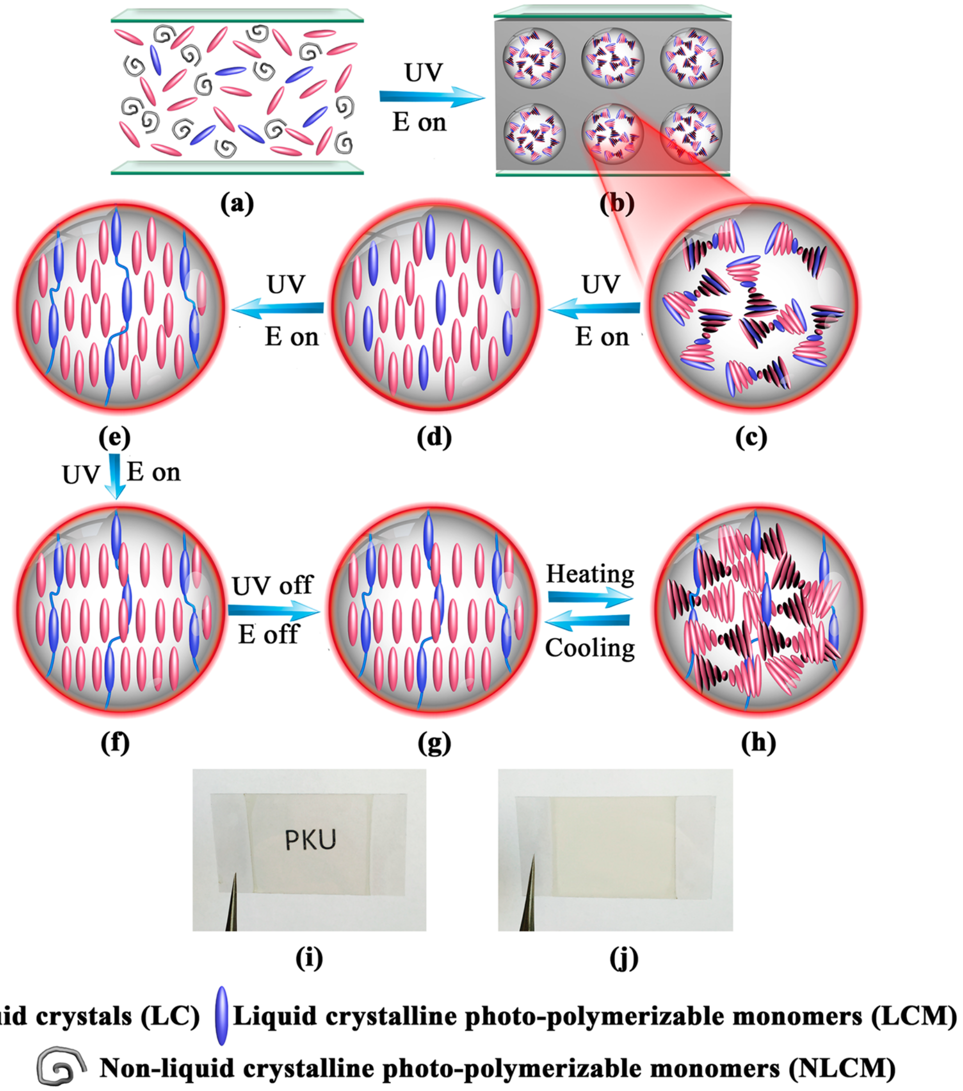

Figure 3. Schematic illustration of the preparation of Film C. (a) The homogeneous isotropic mixture sandwiched between two pieces of plastic sheets. (b) A microphase separation between the liquid crystalline mixture and polymer matrix. (c) The randomly oriented liquid crystalline mixture within a LC droplet. (d) The perpendicularly aligned liquid crystalline mixture. (e) The HAPN formed within a LC droplet. (f) The N* phase gradually turns into the SmA phase upon the consumption of photopolymerizable monomers. (g) The perpendicularly aligned SmA phase within a LC droplet. (h) The focal-conic texture of the heat-induced N* phase within a LC droplet. (i) A photograph of the transparent state of the film at a temperature below the phase-transition temperature of the LC. (j) A photograph of the heat-induced light-scattering state of the film.

and this change is reversible. Figure 4b shows that transmittances of the transparent and light-scattering states of Film C change only a little in the wavelength range from 400 to 800 nm. Although Film B exhibits similar thermal-optical properties as Film C, its maximum shear strength is nearly 1.0 KPa, while the maximum shear strengths of Film A and Film C are approximately 16.5 and 27.5 KPa, respectively, as shown in Figure 4c. The obviously higher shear strength of Film C compared to those of Films A and B and, in particular, the much higher shear strength of Film C compared to that of Film B are due to the absence of the HAPN within the porous polymer network of Film A and the smaller content of the HAPN in Film B.

Figure 5 shows the photos of Film C. The successful large-scale preparation of Film C is shown in Figure 5a. When the film is heated with a hot air blower, the heated region exhibits a light-scattering state. Figure 5b demonstrates the flexibility of Film C. Notably, Film C with a higher shear strength can be prepared more easily than Film B. Due to the lower shear strength between two flexible substrates of Film B, the substrates can be easily separated with even a relatively small force. On the one hand, the large-scale preparation of Film B is difficult; on the other hand, such a large-scale fabrication is of little practical use. Clearly, Film C has the advantages of the strong shear strength of Film A and the similar thermal-optical properties of Film B. Therefore, the PD&SLC system has the advantages of both the excellent large-scale manufacturing of a PDLC system and the convenient control of the orientation of the LC molecules of a PSLC system in its initial state.

■ CONCLUSIONS

In conclusion, a novel PD&SLC system with coexistent structures of the polymer networks of PDLC and PSLC systems has been developed. On the basis of the coexistent structures of the polymer networks, this novel system has both the advantages of the strong shear strength of a PDLC system and the convenient control of the orientation of the LC

DOI: 10.1021/acsami.6b13366
ACS Appl. Mater. Interfaces 2017, 9, 2942−2947

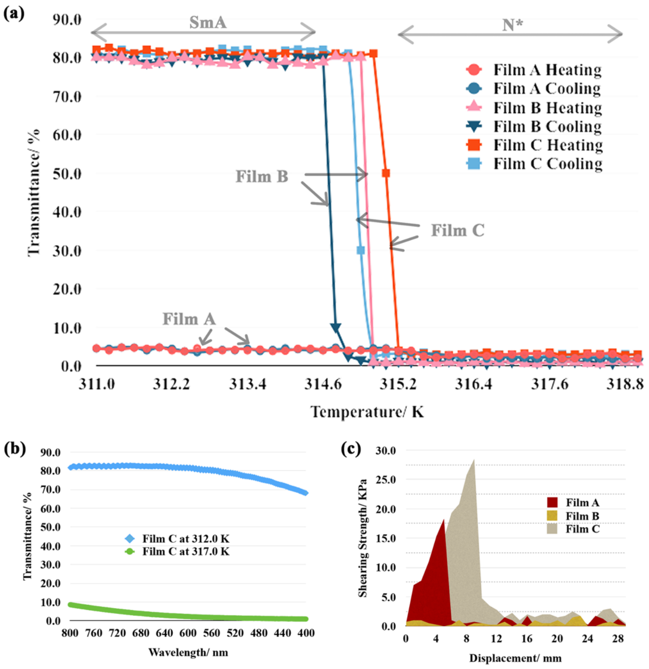

Figure 4. (a) The temperature dependence of the transmittance of Films A, B, and C. (b) The wavelength dependence of the transmittance of Film C measured at approximately 312.0 and 317.0 K. (c) The shear strengths of Films A, B, and C.

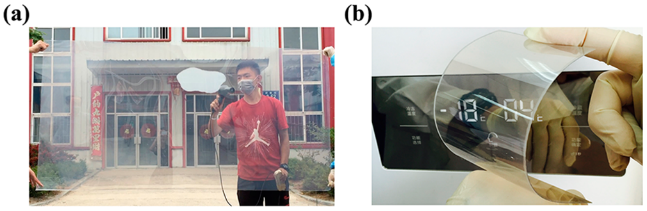

Figure 5. Photographs of Film C: (a) a large-scale film and (b) the flexibility of the film.

molecules of a PSLC system in its initial state. Therefore, this system combines the excellent large-scale manufacturing characteristics of a PDLC system and the thermo-optical properties of a PSLC system. By using a LC with a SmA-N* phase transition, films obtained from this system can reversibly transverse transparent and strong light-scattering states, which accompany the phase transition. This film has great potential applications; for example, it can be used as an energy-saving smart window film upon doping with infrared light absorbers or as a temperature sensor upon doping with dichroic dyes. These applications will be discussed in detail in the near future.

■ ASSOCIATED CONTENT

Supporting Information

The Supporting Information is available free of charge on the ACS Publications website at DOI: 10.1021/acsami.6b13366.

DCS and POM images of pure LC and LC encapsulated in the PD&SLC system and SEM photographs of Sample C cured for approximately 3.0 min (PDF)

Movie of a flexible TLTC film in a large scale from the PD&SLC system (AVI)

■ AUTHOR INFORMATION

Corresponding Author

*E-mail: yanghuai@pku.edu.cn.

ORCID

Shu-Meng Guo: 0000-0002-0466-6382

Author Contributions

The manuscript was written through contributions of all authors. All authors have given approval to the final version of the manuscript.

Notes

The authors declare no competing financial interest.

■ ACKNOWLEDGMENTS

This work was supported by the National Natural Science Foundation of China (NSFC) (Grant Nos. 51573006, 51333001, 51302006, 51303008, and 51573003) and the Major Project of Beijing Science and Technology Program (Grant No. Z151100003315023).

■ ABBREVIATIONS

LC, liquid crystal
PDLC, polymer-dispersed liquid crystal
PSLC, polymer-stabilized liquid crystal
PD&SLCs, a coexistent system of polymer-dispersed and polymer-stabilized liquid crystals
TLTC, thermally light-transmittance-controllable
NLCMs, nonliquid-crystalline photopolymerizable monomers
LCMs, liquid crystalline photopolymerizable monomers
HAPNs, homeotropically aligned polymer networks

■ REFERENCES

(1) Cheng, Z. X.; Wang, T. J.; Li, X.; Zhang, Y. H.; Yu, H. F. NIR-VIS-UV Light-Responsive Actuator Films of Polymer-Dispersed Liquid Crystal/Graphene Oxide Nanocomposites. *ACS Appl. Mater. Interfaces* **2015**, *7*, 27494−27501.

(2) Kumano, N.; Seki, T.; Ishii, M.; Nakamura, H.; Umemura, T.; Takeoka, Y. Multicolor Polymer-Dispersed Liquid Crystals. *Adv. Mater.* **2011**, *23*, 884−888.

(3) Kim, M.; Park, K. J.; Seok, S.; Ok, J. M.; Jung, H. T.; Choe, J.; Kim, D. H. Fabrication of Microcapsules for Dye-Doped Polymer-Dispersed Liquid Crystal-Based Smart Windows. *ACS Appl. Mater. Interfaces* **2015**, *7*, 17904−17909.

(4) Seo, J.; Song, M.; Jeong, J.; Nam, S.; Heo, I.; Park, S. Y.; Kang, I. K.; Lee, J. H.; Kim, H.; Kim, Y. Broadband pH-Sensing Organic Transistors with Polymeric Sensing Layers Featuring Liquid Crystal Microdomains Encapsulated by Di-Block Copolymer Chains. *ACS Appl. Mater. Interfaces* **2016**, *8*, 23862−23867.

(5) Yu, L.; Cheng, Z. X.; Dong, Z. J.; Zhang, Y. H.; Yu, H. F. Photomechanical Response of Polymer-Dispersed Liquid Crystals/Graphene Oxide Nanocomposites. *J. Mater. Chem. C* **2014**, *2*, 8501−8506.

(6) Kikuchi, H.; Yokota, M.; Hisakado, Y.; Yang, H.; Kajiyama, T. Polymer-Stabilized Liquid Crystal Blue Phases. *Nat. Mater.* **2002**, *1*, 64−68.

(7) Broer, D. J.; Lub, J.; Mol, G. N. Wide-Band Reflective Polarizars From Cholesteric Polymer Networks with a Pitch Gradien. *Nature* **1995**, *378*, 467−469.

(8) Yu, L.; Yu, H. F. Light-Powered Tumbler Movement of Graphene Oxide/Polymer Nanocomposites. *ACS Appl. Mater. Interfaces* **2015**, *7*, 3834−3839.

(9) Zheng, Z. G.; Wang, H. F.; Zhu, G.; Lin, X. W.; Li, J. N.; Hu, W.; Cui, H. Q.; Shen, D.; Lu, Y. Q. Low-Temperature-Applicable Polymer-

Stabilized Blue-Phase Liquid Crystal and its Kerr Effect. *J. Soc. Inf. Disp.* **2012**, *20*, 326−332.
(10) Zheng, Z. G.; Wang, C.; Shen, D. Dichroic-Dye-Doped Polymer Stabilized Optically Isotropic Chiral Liquid Crystals. *J. Mater. Chem. C* **2013**, *1*, 6471−6478.
(11) Allouchery, V.; Roussel, F.; Buisine, J. M. Thermodynamic and Electro-Optic Characteristics of UV-Cured Monofunctional Acrylate/Nematic Liquid Crystal Mixtures. *Mol. Cryst. Liq. Cryst. Sci. Technol., Sect. A* **1999**, *329*, 227−237.
(12) Nastal, E.; Zuranska, E.; Mucha, M. Effect of Curing Progress on the Electrooptical and Switching Properties of PDLC System. *J. Appl. Polym. Sci.* **1999**, *71*, 455−463.
(13) Vaz, N. A.; Montgomery, G. P. Refractive-Indexes of Polymer-Dispersed Liquid-Crystal Film Materials-Epoxy Based Systems. *J. Appl. Phys.* **1987**, *62*, 3161−3172.
(14) Smith, G. W.; Vaz, N. A. The Relationship Between Formation Kinetics and Microdroplet Size of Epoxy-Based Polymer-Dispersed Liquid-Crystals. *Liq. Cryst.* **1988**, *3*, 543−571.
(15) Soule, E. R.; Abukhdeir, N. M.; Rey, A. D. Thermodynamics, Transition Dynamics, and Texturing in Polymer-Dispersed Liquid Crystals with Mesogens Exhibiting a Direct Isotropic/Smectic-A Transition. *Macromolecules* **2009**, *42*, 9486−9497.
(16) Kuschel, F.; Hartmann, L.; Bauer, M. Simplified Preparation of Polymer-Encapsulated LC layers by Thermally Induced Phase Separation. *Liq. Cryst.* **2011**, *38*, 325−331.
(17) Srivastava, J. K.; Singh, R. K.; Dhar, R.; Singh, S. Thermal and Morphological Studies of Liquid Crystalline Materials Dispersed in a Polymer Matrix. *Liq. Cryst.* **2011**, *38*, 849−859.
(18) Wu, S. T. Voltage-Biased Liquid Crystal Optical Power Limiters. *Proc. SPIE* **1986**, *684*, 108−112.
(19) Doane, J. W.; Vaz, N. A.; Wu, B. G.; Zumer, S. Field Controlled Light-Scattering from Nematic Microdroplets. *Appl. Phys. Lett.* **1986**, *48*, 269−271.
(20) Zhang, C.; Wang, D.; Cao, H.; Song, P.; Yang, C.; Yang, H.; Hu, G. H. Preparation and Electro-Optical Properties of Polymer Dispersed Liquid Crystal Films with Relatively Low Liquid Crystal Content. *Polym. Adv. Technol.* **2013**, *24*, 453−459.
(21) Cupelli, D.; Pasquale Nicoletta, F.; Manfredi, S.; Vivacqua, M.; Formoso, P.; De Filpo, G.; Chidichimo, G. Self-Adjusting Smart Windows Based on Polymer-Dispersed Liquid Crystals. *Sol. Energy Mater. Sol. Cells* **2009**, *93*, 2008−2012.
(22) Buyuktanir, E. A.; Mitrokhin, M.; Holter, B.; Glushchenko, A.; West, J. L. Flexible Bistable Smectic-A Polymer Dispersed Liquid Crystal Display. *Jpn. J. Appl. Phys.* **2006**, *45*, 4146−4151.
(23) Sheraw, C. D.; Zhou, L.; Huang, J. R.; Gundlach, D. J.; Jackson, T. N.; Kane, M. G.; Hill, I. G.; Hammond, M. S.; Campi, J.; Greening, B. K.; Francl, J.; West, J. Organic Thin-Film Transistor-Driven Polymer-Dispersed Liquid Crystal Displays on Flexible Polymeric Substrates. *Appl. Phys. Lett.* **2002**, *80*, 1088−1090.
(24) Xiong, G. R.; Han, G. Z.; Sun, C.; Xu, H.; Wei, H. M.; Gu, Z. Z. Phototunable Microlens Array Based on Polymer Dispersed Liquid Crystals. *Adv. Funct. Mater.* **2009**, *19*, 1082−1086.
(25) Liu, Y. J.; Ding, X. Y.; Lin, S. C. S.; Shi, J. J.; Chiang, I. K.; Huang, T. J. Surface Acoustic Wave Driven Light Shutters Using Polymer-Dispersed Liquid Crystals. *Adv. Mater.* **2011**, *23*, 1656−1659.
(26) Dierking, I. Polymer Network-Stabilized Liquid Crystals. *Adv. Mater.* **2000**, *12*, 167−181.
(27) Hu, W.; Zhao, H.; Song, L.; Yang, Z.; Cao, H.; Cheng, Z.; Liu, Q.; Yang, H. Electrically Controllable Selective Reflection of Chiral Nematic Liquid Crystal/Chiral Ionic Liquid Composites. *Adv. Mater.* **2010**, *22*, 468−472.
(28) Yang, H.; Kikuchi, H.; Kajiyama, T. Temperature Dependent Light Transmission-Light Scattering Switching of (Homeotropic Liquid Crystalline Polymer Network/Liquid Crystals/Chiral Dopant) Composite Film. *Liq. Cryst.* **2000**, *27*, 1695−1699.
(29) Broer, D. J.; Boven, J.; Mol, G. N.; Challa, G. Insitu Photopolymerization of Oriented Liquid-Crystalline Acrylates 0.3. Oriented Polymer Networks from a Mesogenic Diacrylate. *Makromol. Chem.* **1989**, *190*, 2255−2268.
(30) Gray, G. W.; Harrison, K. J.; Nash, J. A. New Family of Nematic Liquid-Crystals for Displays. *Electron. Lett.* **1973**, *9*, 130−131.
(31) Zhang, W. J.; Lin, J. P.; Yu, T. S.; Lin, S. L.; Yang, D. Z. Effect of Electric Field on Phase Separation of Polymer Dispersed Liquid Crystal. *Eur. Polym. J.* **2003**, *39*, 1635−1640.
(32) Coates, D. Polymer-Dispersed Liquid Crystals. *J. Mater. Chem.* **1995**, *5*, 2063−2072.
(33) Fung, Y. K.; Yang, D. K.; Ying, S.; Chien, L. C.; Zumer, S.; Doane, J. W. Polymer Networks Formed in Liquid-Crystals. *Liq. Cryst.* **1995**, *19*, 797−801.
(34) Wen, X. H.; Zhang, D.; Zhang, L. X. Conformations and Migration Behaviors of Confined Semiflexible Polymers under Poiseuille Flow. *Polymer* **2012**, *53*, 873−880.
(35) Pan, G.; Yu, L.; Zhang, H.; Guo, J.; Guo, R.; Cao, H.; Yang, Z.; Yang, H.; Zhu, S. Effects on Thermo-Optical Properties of the Composition of a Polymer-Stabilised Liquid Crystal with a Smectic A-Chiral Nematic Phase Transition. *Liq. Cryst.* **2008**, *35*, 1151−1160.

DOI: 10.1021/acsami.6b13366
ACS Appl. Mater. Interfaces 2017, 9, 2942−2947

CCC RightsLink®

Home | Help | Live Chat | Ruicong Zhang

ACS Publications
Most Trusted. Most Cited. Most Read.

Preparation of a Thermally Light-Transmittance-Controllable Film from a Coexistent System of Polymer-Dispersed and Polymer-Stabilized Liquid Crystals

Author: Shu-Meng Guo, Xiao Liang, Cui-Hong Zhang, et al
Publication: Applied Materials
Publisher: American Chemical Society
Date: Jan 1, 2017

Copyright © 2017, American Chemical Society

PERMISSION/LICENSE IS GRANTED FOR YOUR ORDER AT NO CHARGE

This type of permission/license, instead of the standard Terms and Conditions, is sent to you because no fee is being charged for your order. Please note the following:

- Permission is granted for your request in both print and electronic formats, and translations.
- If figures and/or tables were requested, they may be adapted or used in part.
- Please print this page for your records and send a copy of it to your publisher/graduate school.
- Appropriate credit for the requested material should be given as follows: "Reprinted (adapted) with permission from {COMPLETE REFERENCE CITATION}. Copyright {YEAR} American Chemical Society." Insert appropriate information in place of the capitalized words.
- One-time permission is granted only for the use specified in your RightsLink request. No additional uses are granted (such as derivative works or other editions). For any uses, please submit a new request.

If credit is given to another source for the material you requested from RightsLink, permission must be obtained from that source.

BACK CLOSE WINDOW

© 2022 Copyright - All Rights Reserved | Copyright Clearance Center, Inc. | Privacy statement | Terms and Conditions
Comments? We would like to hear from you. E-mail us at customercare@copyright.com

Materials Horizons

COMMUNICATION

View Article Online
View Journal | View Issue

Check for updates

Cite this: *Mater. Horiz.*, 2017, 4, 878

Received 11th April 2017,
Accepted 21st June 2017

DOI: 10.1039/c7mh00224f

rsc.li/materials-horizons

Published on 21 June 2017. Downloaded on 9/20/2022 10:26:26 AM.

A temperature and electric field-responsive flexible smart film with full broadband optical modulation†

Xiao Liang,[a] Shumeng Guo,[b] Mei Chen,[a] Chenyue Li,[b] Qian Wang,[c] Cheng Zou,[a] Cuihong Zhang,[a] Lanying Zhang,[a] Shaojun Guo *[ad] and Huai Yang *[a]

This study provides a new procedure for the preparation of a flexible multi-responsive smart film containing tin-doped indium oxide nanocrystals and a phase-separated liquid crystal-polymer. This film can reversibly control the passage of visible light in response to temperature and electric field and also screen near infrared light.

With the rapid increase in energy consumption driven by new buildings and remarkable progress of living standard, great efforts have been devoted towards the development of smart windows with multi-features, including optical modulation, large-scale manufacturing, and energy-saving, to replace the traditional way of sun-shading.[1] Traditionally, the intense visible light on scorching days can be blocked by smart windows based on electro-,[2–4] thermo-,[5–7] gaso-,[8,9] and photochromogenic[10,11] materials that can change their transmittance according to an external stimuli. Among these, the electrochromic materials can achieve the largest projected energy savings by providing the widest range of solar control.[12] However, although these smart materials enhance the internal comfort and increase the energy efficiency to some extent, they can only manipulate a limited range of solar radiation, constricted in the region of visible light. Their transmittance still maintains high levels in the near infrared radiation (NIR) region.[4] Moreover, these chromogenic materials can only respond to one external stimulus, either temperature, electric field or light; this greatly limits their intellectuality.

Conceptual insights

Smart windows, with the abilities of achieving energy-saving and optimizing solar energy utilization, are playing a key role in reducing the overall energy expenditure and increasing comfort levels for people inside buildings. However, the optical modulation of smart windows is usually constricted within a limited waveband, either visible (400–800 nm) or near infrared (NIR, 800–2500 nm) region. Moreover, they require additional energy to maintain the colored or transparent state. Herein, we report a flexible multi-responsive smart film by creating a compatible interface between tin-doped indium oxide (ITO) nanocrystals and polar syrup containing liquid crystals with a smectic A (SmA)/chiral nematic (N*) phase transition and photo-polymerizable monomers. This film has a widest waveband modulation reported to date, covering both visible and NIR regions (400–2500 nm). The transmittance of the as-made smart film can be thermally changed reversibly from highly transparent (78%) to a strong light-scattering (1.5%) state in the visible region, and the light-scattering state of the film can also be electrically regulated. Moreover, more than 85% of the invisible NIR light can be efficiently shielded due to a well-preserved localized surface plasmon resonance from ITO NCs. This study represents a key step forward towards the preparation of optical materials with multi-functional features for applications in energy-saving smart windows.

To enhance the energy efficiency of a smart window, the modulation of NIR irradiation, which carries about 50% of the solar energy,[13] is of equal importance. Compared to these traditional optical materials that can only modulate visible light, degenerately doped semiconductor nanocrystals (NCs), such as tin-doped indium oxide (ITO), are promising candidates as NIR shielding materials because of the combination of their high transparency in the visible region due to their large band gaps (E_g > 3.0 eV) and tunable NIR absorption resulting from localized surface plasmon resonances (LSPRs).[14–16] However, these NIR shielding materials can only selectively block NIR irradiation and cannot modulate the transmittance of visible light. In this regard, to achieve an energy-efficient smart window, the exploration of new multi-responsive smart materials with the broadband optical modulation that can cover the region ranging from visible to NIR light is desirable and attractive. However,

[a] *Department of Materials Science and Engineering, College of Engineering, Peking University, Beijing 100871, People's Republic of China. E-mail: yanghuai@pku.edu.cn*
[b] *Key Laboratory of Polymer Chemistry and Physics of Ministry of Education, Peking University, Beijing 100871, People's Republic of China*
[c] *Key Laboratory of Bio-Inspired Smart Interfacial Science and Technology, Ministry of Education, School of Chemistry and Environment, Beihang University, Beijing 100191, China*
[d] *BIC-ESAT, College of Engineering, Peking University, Beijing 100871, China. E-mail: guosj@pku.edu.cn*

† Electronic supplementary information (ESI) available. See DOI: 10.1039/c7mh00224f

This journal is © The Royal Society of Chemistry 2017

Published on 21 June 2017. Downloaded on 9/20/2022 10:26:26 AM.

achievement of this target is still a great challenge because the interface between visible materials and NIR materials is usually incompatible.

Nowadays, flexible, stretchable, and wearable optical materials have received extensive attention.[17,18] Liquid crystal (LC)/polymer composites, which usually combine the ordered structures and multi-responsive characteristic of LCs with excellent physical and chemical properties of polymers, have gained intensive research interest in recent years.[19–23] They are structurally divided into two different systems: a polymer-dispersed liquid crystal (PDLC) system, where LC droplets are randomly dispersed in a polymer matrix, and a polymer-stabilized liquid crystal (PSLC) system, where the initial orientation of LCs can be stabilized by the polymer network.[24,25] Generally, the polymer content in a PDLC film is usually higher than 40 wt%, endowing flexibility and excellent mechanical strength to the film in practical applications.[26] However, a PDLC film usually exhibits a light scattering state without the use of an electric field because the porous polymer matrix lacks orientation control over LCs,[27] which is not suitable for an energy-efficient smart window for buildings. A PSLC device has an orientated liquid crystalline polymer network to control the orientation of LCs. However, the polymer content in a PSLC device is usually lower than 10 wt%, or otherwise the device cannot be driven by an external stimulus.[25] Thus, a practical flexible and smart film cannot be prepared in a PSLC system due to low polymer content.

Herein, we report a new method for making a smart flexible film with excellent mechanical strength that not only can intelligently tune its transmittance in the visible region in response to both temperature and electric field, but can also shield solar energy in the NIR region. Our material for the smart film is a composite of a phase-separated polymer network with a unique microstructure where ITO NCs and liquid-crystalline droplets with a SmA–N* phase transition are uniformly dispersed. Different from either a PDLC or PSLC system, the film is structurally a co-existence of PDLC and PSLC, in which orientated liquid-crystalline polymer networks (OLPNs) are formed within the porous polymer matrix. The optical modulation of the material in the visible region is realized by spatially varying the refractive index, and the NIR irradiation of solar energy is effectively rejected by the strong LSPR absorption from ITO NCs. Unlike the conventional smart windows, the as-made film has an optical-modulation covering from visible to NIR region (400 to 2500 nm) that makes it a smart material with the widest waveband modulation reported to date. Moreover, the transmittance of the present film in visible light can be changed both thermally and electrically, whereas most smart materials are usually mono-responsive. In addition, the polymeric syrup containing highly compatible inorganic and organic components enables our film to be readily processed on flexible substrates *via* solution-based roll-to-roll production to realize large-scale manufacturing, and the shearing strength of the as-made flexible film is 280 times that of a PSLC film due to high content of the unique polymer structure. Moreover, the phase transition temperature of LCs in our film can be readily tuned from below to above room temperature according to different requirements by adjusting the LC compositions; this makes the film suitable for application in areas with different climates. The as-prepared film shows a great potential application as a smart energy-saving window, and the proposed preparation method enlightens new insights into the engineering of novel flexible hybrid inorganic–organic functional matters.

To obtain these smart materials with a broadband optical modulation, *a prior* challenge is to achieve a necessary homogeneous distribution of ITO NCs in the reactive syrup of LCs and photopolymerizable monomers. The synthesized ITO NCs are typically stabilized by oleylamine and oleic acid, which can be readily dispersed in nonpolar solvents such as hexane, dichloromethane, toluene, *etc.*[12,28,29] However, the LC/monomer mixture is usually viewed as a solvent with an intensive polarity. This drastic change in polarity of the medium will destabilize the dispersion and lead to severe aggregations.[30] Consequently, it not only results in a loss of transmittance in the visible region[31,32] but also detrimentally affects the NIR shielding ability of the NCs due to significant reduction of the surface electron density in large aggregates. Moreover, ITO NCs have high conductivity, and the conductive dopants will cause a severe charging problem while applying an electric field to the film between substrates to reorient the orientations of LCs.

To solve these problems, we proposed a facile strategy (Scheme 1a–c) for the incorporation of nonpolar ITO NCs into the LC/polymer composite by encapsulating the ITO NCs with an insulating silica barrier to form a core/shell structure (Scheme 1b), followed by a methacryloxypropyltrimethoxysilane (MPTMS) surface treatment (Scheme 1c). This coated silica layer can work as a tuning agent for making the interface of ITO NCs and LCs highly compatible due to the following advantages. First, the thin silica layer is optically transparent and does not affect the optical properties of the ITO NCs; second, the silica shells serve as a capping layer, transforming the highly conductive ITO NCs ito insulating ITO NCs. Finally, the hydrophilic silica shells allow the ITO NCs to be well incorporated into polar environments, and the well dispersion can be further enhanced by functionalizing the silica shells with surfactants. The preparation procedure of the multi-responsive smart material with broadband optical modulation is illustrated in Scheme 1d–k. The LCs we used have a SmA–N* phase transition (SmA–N*-LCs) at 304.3 K. A mixture of non-liquid crystalline acrylate monomers (NAMs) and photopolymerizable LCs (PLCs) is used as photopolymerizable monomers. As shown in Scheme 1d, the reactive syrup containing ITO NCs is sandwiched between two plastic transparent substrates. The thickness of the film is regulated to 20 μm using glass bead spacers. Then, the film is irradiated by UV light for a short time to initiate polymerization, which brings phase separation between LCs and the polymer, forming a porous polymer network and LCs droplets (Scheme 1e and f), structurally like a PDLC film. This porous polymer structure provides mechanical strength and also endows flexibility to the film. Then, an electric field is applied to perpendicularly orient the LCs (Scheme 1g). Moreover, a second step of UV polymerization is carried out to complete the cross-linking between PLCs within the LC droplets, forming an

Published on 21 June 2017. Downloaded on 9/20/2022 10:26:26 AM.

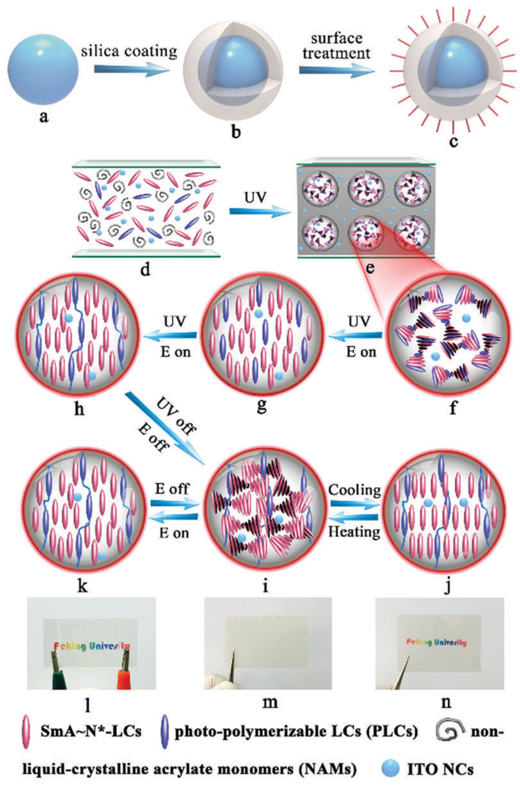

Scheme 1 Schematic of the transfer procedure for ITO NCs (a–c): single ITO NC is encapsulated with a hydrophilic silica barrier followed by MPTMS surface treatment. (d–k) Preparation procedures for the smart film: a homogenous polymeric syrup is sandwiched between two plastic substrates (d), then as a first step, the film is irradiated by UV light to initiate polymerization, forming a phase-separated porous polymer structure and LC droplets (e and f). Then, an electric field is applied to homeotropically align the LCs (g). Moreover, a second step of UV polymerization is carried out to complete the cross-linking between PLCs within LC droplets, forming OLPNs within the porous structure (h). The as-made film can reversibly change between transparent (n) and opaque state (m) in response to temperature. Moreover, the opaque state of the film can be switched to transparent state by applying an electric field (l).

OLPN within the porous structure (Scheme 1h). After this two-step polymerization, a novel coexistent polymer structure of PDLC and PSLC is constructed. After the film is cooled down to room temperature (298 K), lower than the phase transition temperature of SmA–N*-LCs, homeotropically aligned SmA phase is formed in the film, induced by the OLPN (Scheme 1j). The film exhibits a transparent state due to the good matching of the refractive indices between SmA–N*-LCs and polymer network. When the temperature is above the phase transition temperature of SmA–N*-LCs, a focal-conic texture of heat-induced N* phase is formed within LC domains (Scheme 1i). The film exhibits a strong light-scattering state resulting from the mismatch of the refractive indices between SmA–N*-LCs and polymer network. Apart from temperature, the light-scattering state of the film can also be actively switched back to the transparent state by applying a homeotropically oriented electric field, as shown in Scheme 1k, since LCs are inherently electric field-responsive materials. Additionally, due to the strong LSPR absorption of ITO NCs incorporated into the film, the NIR irradiation can also be efficiently blocked.

Typically, the LSPR absorption band of ITO NCs is narrow, which exhibits a symmetry/asymmetry line shape depending on the dopant distribution.[33] For use as an NIR filter, however, a broadband absorption in the NIR region as well as high transmittance in visible region are much more desirable. It is well known that the LSPR wavelengths of ITO NCs are significantly related to the electron density and can be tuned *via* varying the doping dosage. To obtain a broadband absorption in the NIR region, we carefully investigated the LSPR properties of the ITO NCs with different mole percentages of Sn {%Sn = 100% × [Sn]/([Sn] + [In])} by tuning the Sn% during initial feeding. As shown in Fig. 1a, with an increasing Sn% from 1.5% to 8.3%, the final color of the ITO NCs can be varied from light green to dark blue as Sn^{4+} dopants lower the conduction band of the ITO NCs, which are responsible for the variations of LSPRs in the NIR region and produce blue color in the dispersion.[34] X-ray analysis (XRD) of these synthesized NCs, as shown in Fig. 1b, indicates that the crystal structures of all the ITO NCs are in good agreement with the cubic bixbyite structure of In_2O_3. Fig. 1c shows the vis-NIR spectra of ITO NCs with different Sn%. The ITO NCs show the clear LSPR peaks from 1560 nm to >2200 nm and undergo a gradual blue shift with the Sn% increasing from 1.5% to 8.3%.[10] Based on the abovementioned vis-NIR spectra of the ITO NCs, we mixed ITO NCs with different Sn%, and a mixture with a broadband NIR absorption was finally obtained (Fig. 1d).

The transmission electron microscopy (TEM) images of the original nonpolar-solvent-soluble ITO NCs dispersed in hexane and ITO/silica core/shell NCs dispersed in ethanol are shown in Fig. S1 (ESI†) and Fig. 2a, respectively. All the ITO/silica NCs have the uniform size of 34 nm with a majority of one silica shell encapsulating one single ITO NC. The thickness of the

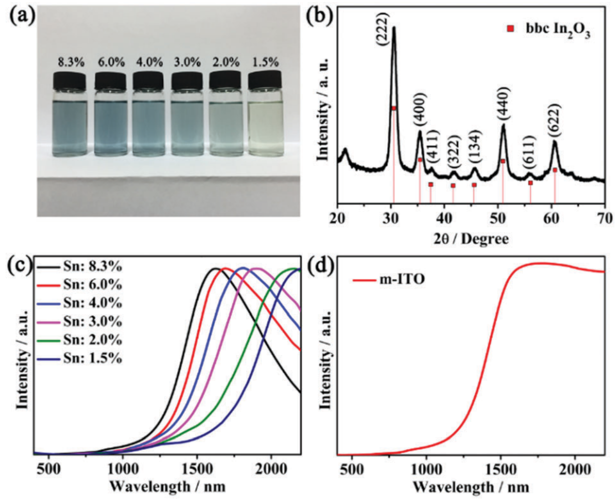

Fig. 1 (a) Images of a series of ITO NCs dispersed in hexane with different Sn compositions. (b) XRD pattern of ITO NCs. (c) Distinctive NIR absorption of ITO NCs with different Sn compositions. (d) A broadband NIR absorption of ITO NCs by mixing ITO NCs with different Sn compositions.

Published on 21 June 2017. Downloaded on 9/20/2022 10:26:26 AM.

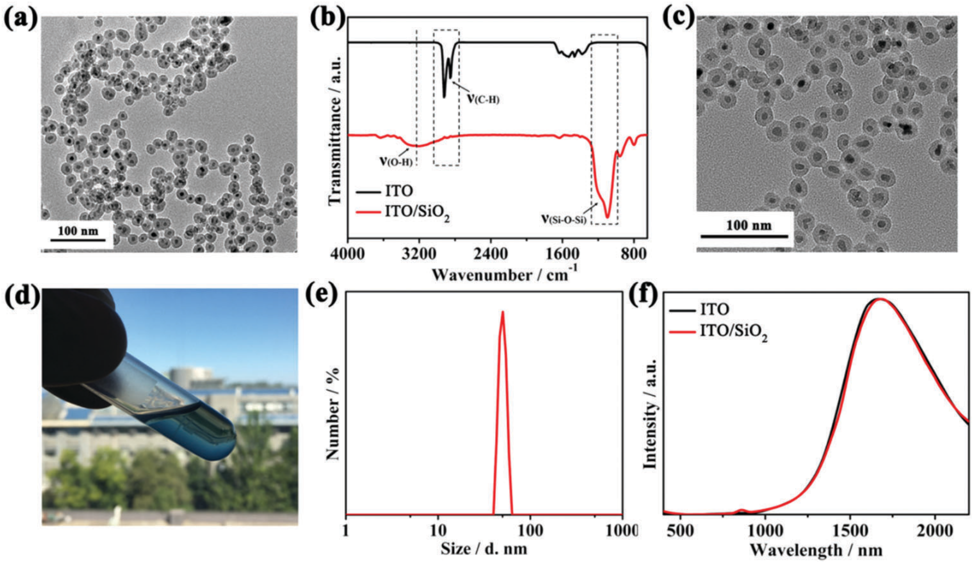

Fig. 2 (a) TEM image of ITO/SiO_2 in ethanol with the shell thickness of 6 nm. (b) FTIR spectra of ITO (black) and ITO/SiO_2 NCs (red). (c) TEM image of MPTMS-functionalized ITO/SiO_2 NCs in ethanol. (d) The image of MPTMS-functionalized ITO/SiO_2 NCs dispersed in the polymeric syrup with a concentration of 5.0 wt%. (e) DLS measurement of MPTMS-functionalized ITO/SiO_2 NCs dispersed in the polymeric syrup with the concentration of 5.0 wt%. (f) The comparison of the absorption spectra of the representative ITO NCs containing 4% Sn before (black) and after silica coating (red).

silica shell can be tuned from 6 nm to 25 nm by varying the amount of the TEOS precursor and the concentration of the ITO NCs used (Fig. S2, ESI†). If not specified, we used ITO/silica (6 nm) for the next characterization and optical application. The XRD pattern of ITO/SiO_2 NCs (Fig. S3, ESI†) shows the characteristic diffraction peaks of ITO NCs. A broad diffraction peak at 20–30 degree was assigned to silica, indicating that the silica shell was amorphous. The Fourier transform infrared (FT-IR) spectra of the original ITO NCs and ITO/silica core/shell NCs are shown in Fig. 2b. Before the silica coating, the vibrational band at 2938 cm^{-1}–2854 cm^{-1} was attributed to $\nu_{(C-H)}$, indicating the existence of organic molecules on the surface of ITO NCs. However, after silica coating, new vibrational bands at 3490 cm^{-1} and 1020 cm^{-1}–1120 cm^{-1} appear and are assigned as the terminal-group of silanol ($\nu_{(O-H)}$) and the representative siloxane network stretching ($\nu_{(Si-O-Si)}$), respectively. These groups not only endow polarity to the ITO/SiO_2 NCs, thus promoting their dispersion in polar solvents, but also facilitate further surface treatments.

The ITO/SiO_2 NCs were further modified with MPTMS at room temperature in ethanol mixed with ammonium hydroxide to adjust the pH value to 9.[35] Fig. 2c shows the TEM image of MPTMS-functionalized ITO/SiO_2 NCs dispersed in ethanol. We found that NCs can still well maintain their morphology without any aggregation. The MPTMS-functionalized NCs were then transferred to the polymeric syrup containing LCs and acrylate monomers. A homogeneous dispersion with blue color was observed (Fig. 2d), suggesting that the interface between MPTMS-functionalized NCs and the polymeric syrup was compatible. Dynamic light scattering (DLS) measurement in the polymeric syrup, as shown in Fig. 2e, shows a hydrodynamic diameter of 51.2 ± 1.6 nm for the MPTMS-functionalized ITO/SiO_2 NCs, larger than the dry size of NCs. This is because the ITO/SiO_2 NCs were MPTMS grafted and charged in the syrup, creating an electrical double layer surrounding the nanoparticles and consequently increasing the colloidal hydrodynamic radius as compared to the actual size. Another key issue of particular concern is that whether or not the silica shell and further treatment will influence the LSPR absorption of ITO NCs in the NIR region. Vis-NIR spectra, as shown in Fig. 2f, indicates that the distinctive absorptions of the representative ITO NCs containing 4% Sn were well-preserved after silica coating and even after further MPTMS treatment.

The smart films containing ITO/SiO_2 NCs were prepared by the stepwise UV polymerization (Scheme 1). Fig. 3a shows the temperature-dependent transmittance of the films with 5.0 wt% ITO/SiO_2 and without NCs at 560 nm. Due to homogenously dispersed ITO/SiO_2 NCs, there is only a slight decrease of ~3% in the transparency for the film with 5.0 wt% ITO/SiO_2 NCs as compared to that for the film without NCs. Moreover, the phase transition temperature for ITO/SiO_2 decreased from 302.5 K to 301.4 K, which could be ascribed to the fact that part of ITO/SiO_2 NCs remained in the LC domains after phase separation. A similarly reversible change from transparent to strong light scattering state (Fig. 3b and c) was observed for both films accompanied by a SmA–N* phase transition. This reversible change of transmittance in visible light can be attributed to the well-designed microstructure of the polymer network in the films, as confirmed by scanning electron microscopy (SEM) (Fig. 3d and e). Note that before SEM characterization, all the prepared films were dipped in hexane for 15 days to fully extract the LC molecules. In Fig. 3d, a porous structure of the polymer matrix can be clearly seen with a thickness of 20 μm. Fig. 3e shows the enlarged SEM image of the polymer network. It can be clearly seen that OLPNs were formed within the porous structure. When the LCs changed from a N* to SmA phase, these OLPNs worked as

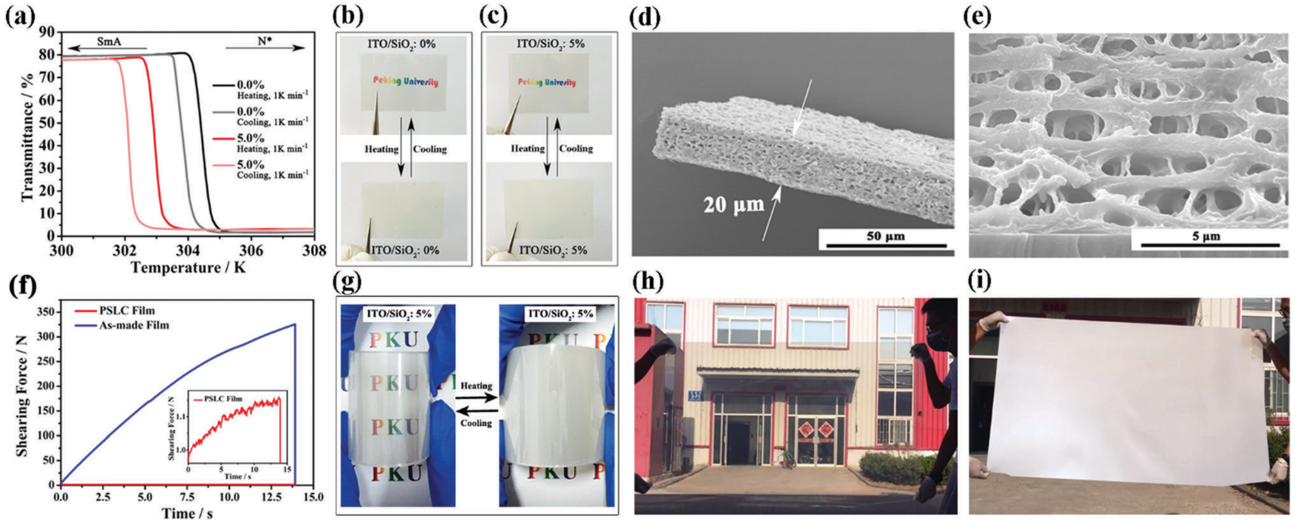

Fig. 3 (a) Temperature dependence of the transmittance of the smart films containing 0% ITO/SiO_2 and 5.0 wt% ITO/SiO_2. Images of the smart films containing (b) 0% ITO/SiO_2 and (c) 5.0 wt% ITO/SiO_2 below and above the phase transition temperature, respectively. SEM images (d) of the polymer network of the smart film containing 5.0 wt% ITO/SiO_2 and (e) the enlarged image of the polymer network from the side view. (f) Shearing force–displacement curves of the as-made film (blue) and the PSLC film (red). Inset: Enlarged shearing force-displacement curve of the PSLC film. Images demonstrating (g) the flexibility of the as-made smart film and (h and i) the large-scale manufacturing of the film.

an alignment agent to induce the SmA phase LCs to be homeotropically oriented; thus, the film exhibited a transparent state. Otherwise, the SmA phase LCs would form a focal-conic texture, and the film would still be light-scattering after phase transition. Moreover, this elaborately designed polymer structure endowed the film with both excellent mechanical strength and flexibility. We measured the shearing forces of the as-made film with 5.0 wt% ITO/SiO_2 and a PSLC film of the same size to compare their mechanical strengths (the SEM image of the polymer network in the PSLC film is shown in Fig. S4, ESI†). As shown in Fig. 3f, the shearing force of the as-made film reached 325.7 N during the shearing process, about 280 times that of the PSLC film. Additionally, the flexibility of the as-prepared film was also tested in Fig. 3g. The excellent thermal-optical properties of the film were well-preserved under bending or folding. Fig. 3h shows the as-made smart film with a size of 1.8 m × 1.1 m for its large-scale fabrication.

The vis-NIR transittance spectra was used to systematically study the optical modulation performance of the films incorporated with different amounts of ITO/SiO_2 NCs in both visible and NIR region at different temperatures. Apparently, the NIR shielding performance of the film was enhanced as more ITO/SiO_2 NCs were incorporated, as shown in Fig. 4a, resulting from their strong LSPRs in the NIR region. The film could reject up to 85% of NIR radiation in its transparent state with 5.0 wt% ITO/SiO_2 incoporated in it. The absorbed NIR irradiation through LSPRs can be effectively transferred into heat and thus facilitate the phase transition of the LCs and reduce the response time upon solar irradiation, as demonstrated in Fig. S5 (ESI†). In comparison, the smart film with the same amount of unmodified ITO NCs as dopants could only shield ~40% of the NIR radiation, and the transmittance in the visible region was more than 20% lower than that of the other films with modified

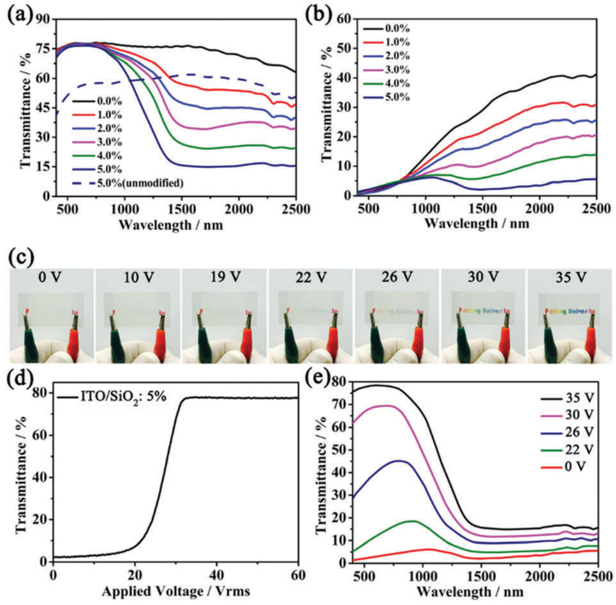

Fig. 4 The vis-NIR transmittance spectra of the films with different concentrations of ITO/SiO_2 measured at 300 K (a) and 308 K (b), respectively. (c) Images of the film containing 5.0 wt% ITO/SiO_2 with various voltages applied. (d) The voltage dependence of the transmittance for the smart film containing 5.0 wt% ITO/SiO_2. (e) Vis-NIR transmittance spectra of the film containing 5.0 wt% ITO/SiO_2 with various voltages applied.

ITO NCs. This decrease in the NIR shielding performance and transparency in the visible region is largely due to the poor dispersibility of the unmodified ITO NCs in the LC/monomer system, leading to the formation of large aggregates. The aggregation, once occurred, not only detrimentally affects the transparent state of the film due to the Rayleigh and Mie scattering, but would also dramatically reduce the surface electron density of the ITO

Published on 21 June 2017. Downloaded on 9/20/2022 10:26:26 AM.

This journal is © The Royal Society of Chemistry 2017

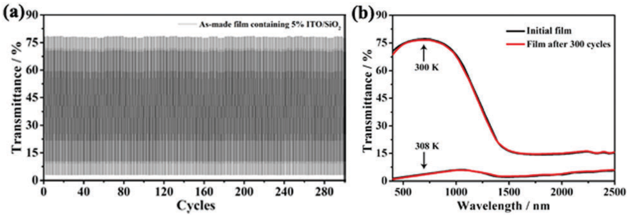

Fig. 5 (a) The transmittance of the film at 550 nm during the heating and cooling cycles. (b) The transmittance spectra of the initial film (black line) and the film after 300 cycles (red line).

particles, which consequently decreases the strength of the LSPRs in the NIR region. Once the environmental temperature exceeds the LCs phase transition temperature, the films would intelligently turn from the transparent to the strong light scattering state. However, this light scattering was less effective in the wavelength longer that 800 nm, as shown in Fig. 4b, due to the fact that LC birefringence decreases as the wavelength increases.[36,37] Incorporation of ITO/SiO_2 into the films resulted in this drawback, and the film could block more that 95% of the solar radiation ranged from 400 nm to 2500 nm in the scattering state.

The electrooptical property of the film was also investigated since LCs are electric field responsive materials. Introduction of unmodified ITO NCs into the film would cause severe electric charge problem while applying an electric field to the film due to the highly conductive property of the ITO NCs. After silica coating, the film with 5 wt% ITO/SiO_2 exhibited excellent electrooptical properties without any charge problem at high temperatures. It could be actively switched from the light scattering to the transparent state under the applied voltage of $\sim$32 V (Fig. 4c) within 183 ms (Fig. S6, ESI†). Fig. 4d shows the voltage dependence of the transmittance of the film. The result reveals that the threshold voltage (V_{th}) and saturation voltage (V_{sat}) of the film are 21.4 V and 32.3 V, respectively. Herein, the V_{th} and V_{sat} are defined as the voltages required for the transmittance to reach 10% and 90%, respectively. These results are comparable with those of most of the current electrically switchable device studies based on an LC/polymer composite.[35,38–40] The vis-NIR spectra of the film (Fig. 4e) indicated that the applied voltage mainly increased the transmittance in the visible region, but the NIR light could still be effectively blocked due to the absorption of ITO/SiO_2 NCs in the film.

Furthermore, to show the stability and reversibility of the as-made smart film, the film containing 5% ITO/SiO_2 NCs was subjected to heating–cooling cycles for 300 times, and the transmittance of the film at 550 nm was monitored during the cycles (Fig. 5a). The results show that the transmittance of transparent and opaque states is almost the same during the cycles. Moreover, the transmittance spectra of the film before and after the cycles were compared (Fig. 5b). The transmittance spectra of the film at 300 K and 308 K after 300 cycles were almost the same as the initial spectra, indicating the long-term stability of the film.

Conclusions

In summary, we present a novel multi-responsive composite film with a broadband optical modulation covering both visible and NIR light. These excellent properties of the film are achieved by creating a compatible SiO_2 tuning layer between nonpolar ITO NCs and polar polymeric syrup containing SmA–N*-LCs and photopolymerizable monomers, followed by formation of elaborately designed coexistent polymer structures in the film with the formation of OLPN in the porous polymer matrix. The transmittance of the film in the visible region can be not only reversibly changed between 78% and 1.5% in response to temperature accompanied by a SmA–N* phase transition, but also actively regulated by applying an electric field to homeotropically align the LCs. Moreover, more than 85% of the NIR irradiation can also be effectively shielded without affecting the visible light transmittance due to the well-preserved LSPR from ITO NCs. In addition, the film possesses excellent flexibility, mechanical strength, and processability because of the coexisting polymer structure. The as-prepared film shows great potential application as a smart energy-saving window, and the proposed strategy enlightens new insights into the engineering of novel flexible hybrid inorganic–organic functional matters.

Acknowledgements

This work was financially supported by the National Natural Science Foundation of China (NSFC) (Grant No. 51333001, 51573006, 51561135014 and 51671003), the Major Project of Beijing Science and Technology Program (Grant No. Z121100006512002), National Basic Research Program of China (No. 2016YFB0100201) and the Sino-American Cooperative Project of Chinese Ministry of Science and Technology (Grant No. 2013DFB50340).

Notes and references

1 D. Ge, E. Lee, L. Yang, Y. Cho, M. Li, D. S. Gianola and S. Yang, *Adv. Mater.*, 2015, **27**, 2489–2495.
2 H. Shin, S. Seo, C. Park, J. Na, M. Han and E. Kim, *Energy Environ. Sci.*, 2016, **9**, 117–122.
3 J. Kim, J. You, B. Kim, T. Park and E. Kim, *Adv. Mater.*, 2011, **23**, 4168–4173.
4 P. M. Beaujuge, S. Ellinger and J. R. Reynolds, *Nat. Mater.*, 2008, **7**, 795–799.
5 J. Zheng, S. Bao and P. Jin, *Nano Energy*, 2015, **11**, 136–145.
6 Y. Gao, S. Wang, L. Kang, Z. Chen, J. Du, X. Liu, H. Luo and M. Kanehira, VO_2–Sb: SnO_2 Composite Thermochromic Smart Glass Foil, *Energy Environ. Sci.*, 2012, **5**, 8234–8237.
7 Y. Gao, C. Cao, L. Dai, H. Luo, M. Kanehira, Y. Ding and Z. L. Wang, *Energy Environ. Sci.*, 2012, **5**, 8708–8715.
8 D. Li, G. Wu, G. Gao, J. Shen and F. Huang, *ACS Appl. Mater. Interfaces*, 2011, **3**, 4573–4579.
9 W. Feng, G. Wu and G. Gao, *J. Mater. Chem. A*, 2014, **2**, 585–590.

Published on 21 June 2017. Downloaded on 9/20/2022 10:26:26 AM.

Published on 21 June 2017. Downloaded on 9/20/2022 10:26:26 AM.

10 F. M. Raymo and M. Tomasulo, *Chem. Soc. Rev.*, 2005, **34**, 327–336.
11 T. Ohko, T. Tatsuma, T. Fujii, K. Naoi, C. Niwa, Y. Kubota and A. Fujishima, *Nat. Mater.*, 2003, **2**, 29–31.
12 G. Garcia, R. Buonsanti, A. Llordes, E. L. Runnerstrom, A. Bergerud and D. J. Milliron, *Adv. Opt. Mater.*, 2013, **1**, 215–220.
13 Y. Sang, Z. Zhao, M. Zhao, P. Hao, Y. Leng and H. Liu, *Adv. Mater.*, 2015, **27**, 363–369.
14 G. Garcia, R. Buonsanti, E. L. Runnerstrom, R. J. Mendelsberg, A. Llordes, A. Anders, T. J. Richardson and D. J. Milliron, *Nano Lett.*, 2011, **11**, 4415–4420.
15 S. Choi, K. M. Nam, B. K. Park, W. S. Seo and J. T. Park, *Chem. Mater.*, 2008, **20**, 2609–2611.
16 A. Calzolari, A. Ruini and A. Catellani, *ACS Photonics*, 2014, **1**, 703–709.
17 F. Xu and Y. Zhu, *Adv. Mater.*, 2012, **24**, 5117–5122.
18 J. Song, S. A. Kulinich, J. Li, Y. Liu and H. Zeng, *Angew. Chem., Int. Ed.*, 2015, **127**, 472–476.
19 A. Ciferri, W. R. Krigbaum and R. B. Meyer, *Polymer Liquid Crystals*, Elsevier, USA, 1982.
20 V. P. Tondiglia, L. V. Natarajan, R. L. Sutherland, D. Tomlin and T. J. Bunning, *Adv. Mater.*, 2002, **14**, 187–191.
21 V. Vorflusev and S. Kumar, *Science*, 1999, **283**, 1903–1905.
22 H. Kikuchi, M. Yokota, Y. Hisakado, H. Yang and T. Kajiyama, *Nat. Mater.*, 2002, **1**, 64–68.
23 N. Kumano, T. Seki, M. Ishii, H. Nakamura, T. Umemura and Y. Takeoka, *Adv. Mater.*, 2011, **23**, 884–888.
24 D. A. Higgins, *Adv. Mater.*, 2000, **12**, 251–264.
25 I. Dierking, *Adv. Mater.*, 2000, **12**, 167–181.
26 C. Zhang, D. Wang, H. Cao, P. Song, C. Yang, H. Yang and G. Hu, *Polym. Adv. Technol.*, 2013, **24**, 453–459.
27 S. T. Wu, Proceedings of the SPIE-The International Society for Optical Engineering, 1986, vol. 684, pp. 108–112.
28 M. Kanehara, H. Koike, T. Yoshinaga and T. Teranishi, *J. Am. Chem. Soc.*, 2009, **131**, 17736–17737.
29 J. Lee, S. Lee, G. Li, M. A. Petruska, D. C. Paine and S. Sun, *J. Am. Chem. Soc.*, 2012, **134**, 13410–13414.
30 S. H. Stelzig, M. Klapper and K. Müllen, *Adv. Mater.*, 2008, **20**, 929–932.
31 J. Wen and G. L. Wilkes, *Chem. Mater.*, 1996, **8**, 1667–1681.
32 E. Manias, A. Touny, L. Wu, K. Strawhecker, B. Lu and T. C. Chung, *Chem. Mater.*, 2001, **13**, 3516–3523.
33 S. D. Lounis, E. L. Runnerstrom, A. Bergerud, D. Nordlund and D. J. Milliron, *J. Am. Chem. Soc.*, 2014, **136**, 7110–7116.
34 A. M. Schimpf, S. D. Lounis, E. L. Runnerstrom, D. J. Milliron and D. R. Gamelin, *J. Am. Chem. Soc.*, 2015, **137**, 518–524.
35 J. D. Busbee, A. T. Yuhl, L. V. Natarajan, V. P. Tongdilia, T. J. Bunning, R. A. Vaia and P. V. Braun, *Adv. Mater.*, 2009, **21**, 3659–3662.
36 S. T. Wu, *Opt. Eng.*, 1987, **26**, 262120.
37 W. Li, L. Yu, W. He, X. Yuan, D. Zhao, W. Huang, H. Cao, Z. Yang and H. Yang, *J. Phys. Chem. C*, 2008, **112**, 13739–13743.
38 W. Hu, H. Zhao, L. Song, Z. Yang, H. Cao, Z. Cheng, Q. Liu and H. Yang, *Adv. Mater.*, 2010, **22**, 468–472.
39 M. Kim, K. J. Park, S. Seok, J. M. Ok, H. T. Jung, J. Choe and D. H. Kim, *ACS Appl. Mater. Interfaces*, 2015, **7**, 17904–17909.
40 R. Baetens, B. P. Jelle and A. Gustavsen, *Sol. Energy Mater. Sol. Cells*, 2010, **94**, 87–105.

This journal is © The Royal Society of Chemistry 2017

CCC Marketplace™

Order Confirmation

Thank you, your order has been placed. An email confirmation has been sent to you. Your order license details and printable licenses will be available within 24 hours. Please access Manage Account for final order details.

This is not an invoice. Please go to manage account to access your order history and invoices.

CUSTOMER INFORMATION

Payment by invoice: You can cancel your order until the invoice is generated by contacting customer service.

Billing Address

Dr. Ruicong Zhang
Harbin Institute of Technology
No. 92, Xidazhi Street, Nangang District
Harbin, 150080
China

+86 15776491006
20b918088@stu.hit.edu.cn

Customer Location

Dr. Ruicong Zhang
Harbin Institute of Technology
No. 92, Xidazhi Street, Nangang District
Harbin, 150080
China

PO Number (optional)

N/A

Payment options

Invoice

PENDING ORDER CONFIRMATION

Confirmation Number: Pending

Order Date: 06-Sep-2022

1. Materials Horizons

0.00 USD

Article: A temperature and electric field-responsive flexible smart film with full broadband optical modulation

**Order License ID**	Pending	**Publisher**	Royal Society of Chemistry
**ISSN**	2051-6355		
**Type of Use**	Republish in a thesis/dissertation	**Portion**	Image/photo/illustration

LICENSED CONTENT

**Publication Title**	Materials Horizons	**Rightsholder**	Royal Society of Chemistry
		**Publication Type**	e-Journal

**Article Title**	A temperature and electric field-responsive flexible smart film with full broadband optical modulation
**Author/Editor**	Royal Society of Chemistry (Great Britain)
**Date**	01/01/2014
**Language**	English
**Country**	United Kingdom of Great Britain and Northern Ireland
**Start Page**	878
**End Page**	884
**Issue**	5
**Volume**	4

REQUEST DETAILS

**Portion Type**	Image/photo/illustration
**Number of images / photos / illustrations**	2
**Format (select all that apply)**	Print
**Who will republish the content?**	Academic institution
**Duration of Use**	Current edition and up to 5 years
**Lifetime Unit Quantity**	Up to 499
**Rights Requested**	Main product
**Distribution**	Worldwide
**Translation**	Original language of publication
**Copies for the disabled?**	No
**Minor editing privileges?**	No
**Incidental promotional use?**	No
**Currency**	USD

NEW WORK DETAILS

**Title**	Advanced liquid crystal-based switchable optical devices for light protection applications: principles and strategies
**Instructor name**	Ruicong Zhang
**Institution name**	Light: Science & Applications
**Expected presentation date**	2022-11-01

ADDITIONAL DETAILS

**Order reference number**	N/A
**The requesting person / organization to appear on the license**	Ruicong Zhang

REUSE CONTENT DETAILS

**Title, description or numeric reference of the portion(s)**	Fig 3 and Fig 4
**Editor of portion(s)**	Liang, Xiao; Guo, Shumeng; Chen, Mei; Li, Chenyue; Wang, Qian; Zou, Cheng; Zhang, Cuihong; Zhang, Lanying; Guo, Shaojun; Yang, Huai
**Volume of serial or monograph**	4
**Title of the article/chapter the portion is from**	A temperature and electric field-responsive flexible smart film with full broadband optical modulation
**Author of portion(s)**	Liang, Xiao; Guo, Shumeng; Chen, Mei; Li, Chenyue; Wang, Qian; Zou, Cheng; Zhang, Cuihong; Zhang, Lanying; Guo, Shaojun; Yang, Huai

**Page or page range of portion**	878-884	**Issue, if republishing an article from a serial**	5
		**Publication date of portion**	2017-09-01

Total Items: 1

Total Due: 0.00 USD

Accepted: Marketplace Order General Terms and Conditions and any applicable Publisher Terms and Conditions
