# Supplementary material for: Advanced liquid crystal-based switchable optical devices for light protection applications: principles and strategies
Source: Light Sci Appl. 2023 Jan 3;12:11. doi: 10.1038/s41377-022-01032-y (PMC9807646; doi:10.1038/s41377-022-01032-y)
Supplement: Supplementary file 17 — Fig 19 copyright promotion [file 41377_2022_1032_MOESM17_ESM.pdf]

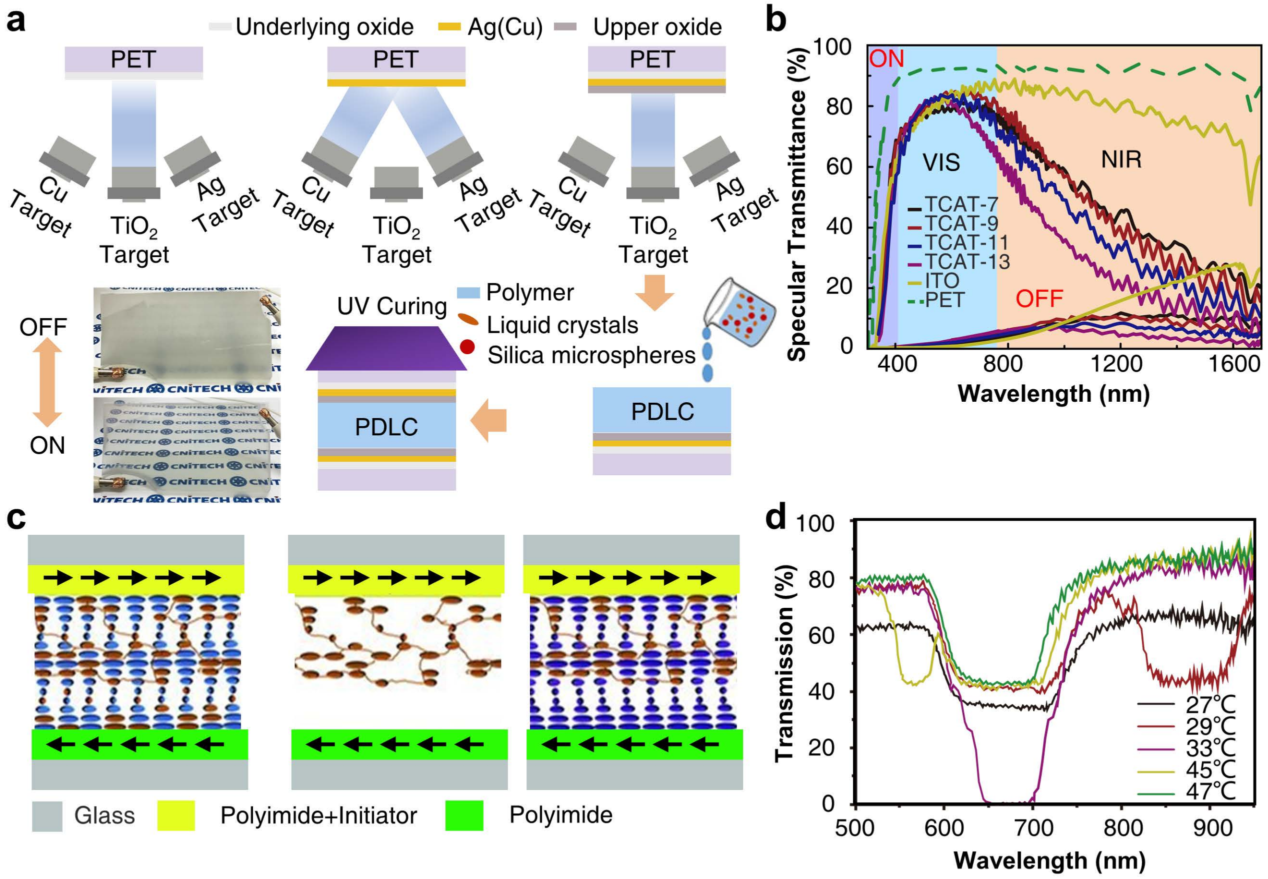
a
Underlying oxide
Ag(Cu)
Upper oxide
PET
Cu Target
TiO2 Target
Ag Target
OFF
ON
UV Curing
Polymer
Liquid crystals
Silica microspheres
PDLC
b
ON
VIS
NIR
OFF
TCAT-7
TCAT-9
TCAT-11
TCAT-13
ITO
PET
Specular Transmittance (%)
Wavelength (nm)
c
Glass
Polyimide+Initiator
Polyimide
d
Transmission (%)
Wavelength (nm)
27°C
29°C
33°C
45°C
47°C

ELSEVIER LICENSE
TERMS AND CONDITIONS

Oct 23, 2022

This Agreement between Harbin Institute of Technology -- Ruicong Zhang ("You") and Elsevier ("Elsevier") consists of your license details and the terms and conditions provided by Elsevier and Copyright Clearance Center.

License Number	5414710080990
License date	Oct 23, 2022
Licensed Content Publisher	Elsevier
Licensed Content Publication	Solar Energy
Licensed Content Title	Simultaneous achievement of high visible transmission and near-infrared heat shielding in flexible liquid crystal-based smart windows via electrode design
Licensed Content Author	Jinhua Huang,Jia Li,Junjun Xu,Zhaozhao Wang,Wei Sheng,Hongjiang Li,Ye Yang,Weijie Song
Licensed Content Date	Aug 1, 2019
Licensed Content Volume	188
Licensed Content Issue	n/a
Licensed Content Pages	8
Start Page	857
End Page	864
Type of Use	reuse in a journal/magazine
Requestor type	academic/educational institute
Portion	figures/tables/illustrations
Number of figures/tables/illustrations	3
Format	both print and electronic
Are you the author of this Elsevier article?	No
Will you be translating?	No
Title of new article	Advanced liquid crystal-based switchable optical devices for light protection applications: principles and strategies
Lead author	Ruicong Zhang, Zhibo Zhang, Jiecai Han, Lei Yang, Jiajun li, Zicheng Song Tianyu Wang, Jiaqi Zhu
Title of targeted journal	Light: Science & Applications
Publisher	Springer Nature
Expected publication date	Nov 2022
Portions	Figure 1,Figure 5 and Figure 6
Requestor Location	Harbin Institute of Technology<br>No. 92, Xidazhi Street, Nangang District<br><br>Harbin, 150080<br>China<br>Attn: Harbin Institute of Technology
Publisher Tax ID	GB 494 6272 12
Total	**0.00 USD**
Terms and Conditions	

INTRODUCTION

1. The publisher for this copyrighted material is Elsevier. By clicking "accept" in connection with completing this licensing transaction, you agree that the following terms and conditions apply to this transaction (along with the Billing and Payment terms and conditions established by Copyright Clearance Center, Inc. ("CCC"), at the time that you opened your Rightslink account and that are available at any time at http://myaccount.copyright.com).

GENERAL TERMS

2. Elsevier hereby grants you permission to reproduce the aforementioned material subject to the terms and conditions indicated.
3. Acknowledgement: If any part of the material to be used (for example, figures) has appeared in our publication with credit or acknowledgement to another source, permission must also be sought from that source. If such permission is not obtained then that material may not be included in your publication/copies. Suitable acknowledgement to the source must be made, either as a footnote or in a reference list at the end of your publication, as follows:
"Reprinted from Publication title, Vol /edition number, Author(s), Title of article / title of chapter, Pages No., Copyright (Year), with permission from Elsevier [OR APPLICABLE SOCIETY COPYRIGHT OWNER]." Also Lancet special credit - "Reprinted from The Lancet, Vol. number, Author(s), Title of article, Pages No., Copyright (Year), with permission from Elsevier."
4. Reproduction of this material is confined to the purpose and/or media for which permission is hereby given.
5. Altering/Modifying Material: Not Permitted. However figures and illustrations may be altered/adapted minimally to serve your work. Any other abbreviations, additions, deletions and/or any other alterations shall be made only with prior written authorization of Elsevier Ltd. (Please contact Elsevier's permissions helpdesk here). No modifications can be made to any Lancet figures/tables and they must be reproduced in full.
6. If the permission fee for the requested use of our material is waived in this instance, please be advised that your future requests for Elsevier materials may attract a fee.
7. Reservation of Rights: Publisher reserves all rights not specifically granted in the combination of (i) the license details provided by you and accepted in the course of this licensing transaction, (ii) these terms and conditions and (iii) CCC's Billing and Payment terms and conditions.
8. License Contingent Upon Payment: While you may exercise the rights licensed immediately upon issuance of the license at the end of the licensing process for the transaction, provided that you have disclosed complete and accurate details of your proposed use, no license is finally effective unless and until full payment is received from you (either by publisher or by CCC) as provided in CCC's Billing and Payment terms and conditions. If full payment is not received on a timely basis, then any license preliminarily granted shall be deemed automatically revoked and shall be void as if never granted. Further, in the event that you breach any of these terms and conditions or any of CCC's Billing and Payment terms and conditions, the license is automatically revoked and shall be void as if never granted. Use of materials as described in a revoked license, as well as any use of the materials beyond the scope of an unrevoked license, may constitute copyright infringement and publisher reserves the right to take any and all action to protect its copyright in the materials.
9. Warranties: Publisher makes no representations or warranties with respect to the licensed material.
10. Indemnity: You hereby indemnify and agree to hold harmless publisher and CCC, and their respective officers, directors, employees and agents, from and against any and all claims arising out of your use of the licensed material other than as specifically authorized pursuant to this license.
11. No Transfer of License: This license is personal to you and may not be sublicensed, assigned, or transferred by you to any other person without publisher's written permission.
12. No Amendment Except in Writing: This license may not be amended except in a writing signed by both parties (or, in the case of publisher, by CCC on publisher's behalf).
13. Objection to Contrary Terms: Publisher hereby objects to any terms contained in any purchase order, acknowledgment, check endorsement or other writing prepared by you, which terms are inconsistent with these terms and conditions or CCC's Billing and Payment terms and conditions. These terms and conditions, together with CCC's Billing and Payment terms and conditions (which are incorporated herein), comprise the entire agreement between you and publisher (and CCC) concerning this licensing transaction. In the event of any conflict between your obligations established by these terms and conditions and those established by CCC's Billing and Payment terms and conditions, these terms and conditions shall control.
14. Revocation: Elsevier or Copyright Clearance Center may deny the permissions described in this License at their sole discretion, for any reason or no reason, with a full refund payable to you. Notice of such denial will be made using the contact information provided by you. Failure to receive such notice will not alter or invalidate the denial. In no event will Elsevier or Copyright Clearance Center be responsible or liable for any costs, expenses or damage incurred by you as a result of a denial of your permission request, other than a refund of the amount(s) paid by you to Elsevier and/or Copyright Clearance Center for denied permissions.

LIMITED LICENSE

The following terms and conditions apply only to specific license types:

15. **Translation**: This permission is granted for non-exclusive world **English** rights only unless your license was granted for translation rights. If you licensed translation rights you may only translate this content into the languages you requested. A professional translator must perform all translations and reproduce the content word for word preserving the integrity of the article.

16. **Posting licensed content on any Website**: The following terms and conditions apply as follows: Licensing material from an Elsevier journal: All content posted to the web site must maintain the copyright information line on the bottom of each image; A hyper-text must be included to the Homepage of the journal from which you are licensing at http://www.sciencedirect.com/science/journal/xxxxx or the Elsevier homepage for books at http://www.elsevier.com; Central Storage: This license does not include permission for a scanned version of the material to be stored in a central repository such as that provided by Heron/XanEdu.

Licensing material from an Elsevier book: A hyper-text link must be included to the Elsevier homepage at http://www.elsevier.com . All content posted to the web site must maintain the copyright information line on the bottom of each image.

Posting licensed content on Electronic reserve: In addition to the above the following clauses are applicable: The web site must be password-protected and made available only to bona fide students registered on a relevant course. This permission is granted for 1 year only. You may obtain a new license for future website posting.

17. **For journal authors:** the following clauses are applicable in addition to the above:

Preprints:

A preprint is an author's own write-up of research results and analysis, it has not been peer-reviewed, nor has it had any other value added to it by a publisher (such as formatting, copyright, technical enhancement etc.).

Authors can share their preprints anywhere at any time. Preprints should not be added to or enhanced in any way in order to appear more like, or to substitute for, the final versions of articles however authors can update their preprints on arXiv or RePEc with their Accepted Author Manuscript (see below).

If accepted for publication, we encourage authors to link from the preprint to their formal publication via its DOI. Millions of researchers have access to the formal publications on ScienceDirect, and so links will help users to find, access, cite and use the best available version. Please note that Cell Press, The Lancet and some society-owned have different preprint policies. Information on these policies is available on the journal homepage.

Accepted Author Manuscripts: An accepted author manuscript is the manuscript of an article that has been accepted for publication and which typically includes author-incorporated changes suggested during submission, peer review and editor-author communications.

Authors can share their accepted author manuscript:

- immediately
  - via their non-commercial person homepage or blog
  - by updating a preprint in arXiv or RePEc with the accepted manuscript
  - via their research institute or institutional repository for internal institutional uses or as part of an invitation-only research collaboration work-group
  - directly by providing copies to their students or to research collaborators for their personal use
  - for private scholarly sharing as part of an invitation-only work group on commercial sites with which Elsevier has an agreement
- After the embargo period
  - via non-commercial hosting platforms such as their institutional repository
  - via commercial sites with which Elsevier has an agreement

In all cases accepted manuscripts should:

- link to the formal publication via its DOI
- bear a CC-BY-NC-ND license - this is easy to do
- if aggregated with other manuscripts, for example in a repository or other site, be shared in alignment with our hosting policy not be added to or enhanced in any way to appear more like, or to substitute for, the published journal article.

Published journal article (JPA): A published journal article (PJA) is the definitive final record of published research that appears or will appear in the journal and embodies all value-adding publishing activities including peer review co-ordination, copy-editing, formatting, (if relevant) pagination and online enrichment.

Policies for sharing publishing journal articles differ for subscription and gold open access articles:

Subscription Articles: If you are an author, please share a link to your article rather than the full-text. Millions of researchers have access to the formal publications on ScienceDirect, and so links will help your users to find, access, cite, and use the best available version.

Theses and dissertations which contain embedded PJAs as part of the formal submission can be posted publicly by the awarding institution with DOI links back to the formal publications on ScienceDirect.

If you are affiliated with a library that subscribes to ScienceDirect you have additional private sharing rights for others' research accessed under that agreement. This includes use for classroom teaching and internal training at the institution (including use in course packs and courseware programs), and inclusion of the article for grant funding purposes.

Gold Open Access Articles: May be shared according to the author-selected end-user license and should contain a CrossMark logo, the end user license, and a DOI link to the formal publication on ScienceDirect.

Please refer to Elsevier's posting policy for further information.

18. **For book authors** the following clauses are applicable in addition to the above: Authors are permitted to place a brief summary of their work online only. You are not allowed to download and post the published electronic version of your chapter, nor may you scan the printed edition to create an electronic version. **Posting to a repository:** Authors are permitted to post a summary of their chapter only in their institution's repository.

19. **Thesis/Dissertation**: If your license is for use in a thesis/dissertation your thesis may be submitted to your institution in either print or electronic form. Should your thesis be published commercially, please reapply for permission. These requirements include permission for the Library and Archives of Canada to supply single copies, on demand, of the complete thesis and include permission for Proquest/UMI to supply single copies, on demand, of the complete thesis. Should your thesis be published commercially, please reapply for permission. Theses and dissertations which contain embedded PJAs as part of the formal submission can be posted publicly by the awarding institution with DOI links back to the formal publications on ScienceDirect.

Elsevier Open Access Terms and Conditions

You can publish open access with Elsevier in hundreds of open access journals or in nearly 2000 established subscription journals that support open access publishing. Permitted third party re-use of these open access articles is defined by the author's choice of Creative Commons user license. See our open access license policy for more information.

Terms & Conditions applicable to all Open Access articles published with Elsevier:

Any reuse of the article must not represent the author as endorsing the adaptation of the article nor should the article be modified in such a way as to damage the author's honour or reputation. If any changes have been made, such changes must be clearly indicated.

The author(s) must be appropriately credited and we ask that you include the end user license and a DOI link to the formal publication on ScienceDirect.

If any part of the material to be used (for example, figures) has appeared in our publication with credit or acknowledgement to another source it is the responsibility of the user to ensure their reuse complies with the terms and conditions determined by the rights holder.

Additional Terms & Conditions applicable to each Creative Commons user license:

CC BY: The CC-BY license allows users to copy, to create extracts, abstracts and new works from the Article, to alter and revise the Article and to make commercial use of the Article (including reuse and/or resale of the Article by commercial entities), provided the user gives appropriate credit (with a link to the formal publication through the relevant DOI), provides a link to the license, indicates if changes were made and the licensor is not represented as endorsing the use made of the work. The full details of the license are available at http://creativecommons.org/licenses/by/4.0.

CC BY NC SA: The CC BY-NC-SA license allows users to copy, to create extracts, abstracts and new works from the Article, to alter and revise the Article, provided this is not done for commercial purposes, and that the user gives appropriate credit (with a link to the formal publication through the relevant DOI), provides a link to the license, indicates if changes were made and the licensor is not represented as endorsing the use made of the work. Further, any new works must be made available on the same conditions. The full details of the license are available at http://creativecommons.org/licenses/by-nc-sa/4.0.

CC BY NC ND: The CC BY-NC-ND license allows users to copy and distribute the Article, provided this is not done for commercial purposes and further does not permit distribution of the Article if it is changed or edited in any way, and provided the user gives appropriate credit (with a link to the formal publication through the relevant DOI), provides a link to the license, and that the licensor is not represented as endorsing the use made of the work. The full details of the license are available at http://creativecommons.org/licenses/by-nc-nd/4.0. Any commercial reuse of Open Access articles published with a CC BY NC SA or CC BY NC ND license requires permission from Elsevier and will be subject to a fee.

Commercial reuse includes:

- Associating advertising with the full text of the Article
- Charging fees for document delivery or access
- Article aggregation
- Systematic distribution via e-mail lists or share buttons

Posting or linking by commercial companies for use by customers of those companies.

20. **Other Conditions**:

v1.10

Questions? customercare@copyright.com or +1-855-239-3415 (toll free in the US) or +1-978-646-2777.

Solar Energy 188 (2019) 857–864

Contents lists available at ScienceDirect

Solar Energy

journal homepage: www.elsevier.com/locate/solener

Simultaneous achievement of high visible transmission and near-infrared heat shielding in flexible liquid crystal-based smart windows via electrode design

Jinhua Huang[a,b], Jia Li[a,*], Junjun Xu[a,c], Zhaozhao Wang[a], Wei Sheng[a], Hongjiang Li[a], Ye Yang[a], Weijie Song[a,d,*]

[a] Ningbo Institute of Material Technology and Engineering, Chinese Academy of Sciences, Ningbo 315201, PR China
[b] University of Chinese Academy of Sciences, Beijing 100049, PR China
[c] Institute of Materials Science, Shanghai University, Shanghai 200072, PR China
[d] Jiangsu Collaborative Innovation Center of Photovoltaic Science and Engineering, Changzhou University, Changzhou 213164, PR China

ARTICLE INFO

Keywords:
Smart window
Polymer dispersed liquid crystals
Ultrathin Ag electrode
Heat shielding

ABSTRACT

Light and heat management are the two vital requirements for smart window towards ideal solar control. However, as a representative polymer dispersed liquid crystal (PDLC) device, little attention has been paid to heat management. Herein, a TiO_2/Ag(Cu)/TiO_2 (TCAT) electrode was designed and optimized to achieve the tunability of transmission in visible range and near-infrared heat shielding simultaneously. The continuous growth of ultrathin Ag films with low percolation threshold was achieved on TiO_2 underlayer by doping a certain amount of Cu. Then the trade-off of PDLC devices' optoelectrical properties was realized. The average ON state specular transmittance of TCAT-based PDLC devices reached 81.9%, 84.9%, 81.9% and 79.9% for Ag(Cu) thicknesses of 7 nm, 9 nm, 11 nm and 13 nm, respectively. In addition, TCAT electrodes exhibited satisfactory ratio of light to solar gain by taking visible transmittance and solar heat gain coefficient into comprehensive consideration. Compared with ITO-based PDLC counterpart, a notable temperature-decreases of 4.6 °C was observed at ON state of TCAT-based device, indicating an excellent heat shielding performance. The proposed TCAT electrode design is promising in smart window application especially for building energy conservation in hot areas or the summer season.

1. Introduction

Building energy conservation is a vital and urgent worldwide issue to alleviate global warming, among which saving energy consumption from window is the top priority. It is reported that energy usage in buildings accounts for about 35% of the total worldwide energy consumption, of which 50% is dissipated from windows (Wang and Zhai, 2018). With the emerging of smart devices, the development of smart window whose light transmission properties could be altered with applied voltage, light or heat provides a good solution to window energy saving (Sabry et al., 2014; Liang et al., 2018; Khandelwal et al., 2017; Wang et al., 2016; Zakirullin and Letuta, 2015). As a representative of smart window, a polymer dispersed liquid crystals (PDLC) device, featured with a liquid crystal layer sandwiched by two parallel transparent electrodes, is considered as a promising electro-optically switchable device. PDLC device regulates the transmittance of visible light by switching between transparency and opacity by applying with external voltage, which plays a role in protecting privacy and replacing physical curtains (Murray et al., 2017; Liang et al., 2017). On the other hand, space heating and cooling are the dominant parts of residential and commercial energy consumption. Near-infrared (NIR) range of solar spectrum carries more than 50% of the solar energy which results in increased heat gain of the buildings. In addition, the heat caused by NIR light reduces the physical comfort of human body, especially in hot areas or the summer season (Alghamedi et al., 2014). In general, the practical application of smart window is faced with the challenge from the tunability of light and heat. It is of great significance to reduce the energy consumption through broadband spectrum controlling for both visible and NIR range via smart window.

Most commonly, the transparent conducting electrodes used for commercially available PDLC devices nowadays are still based on indium-tin oxide (ITO) because of its excellent and stable photoelectric

* Corresponding authors at: Ningbo Institute of Material Technology and Engineering, Chinese Academy of Sciences, Ningbo 315201, PR China.
E-mail addresses: lijia@nimte.ac.cn (J. Li), weijiesong@nimte.ac.cn (W. Song).

https://doi.org/10.1016/j.solener.2019.06.063
Received 28 March 2019; Received in revised form 11 May 2019; Accepted 25 June 2019
Available online 02 July 2019
0038-092X/ © 2019 Published by Elsevier Ltd on behalf of International Solar Energy Society.

characteristics. However, the ITO electrode is criticized for its drawbacks such as the scarcity of raw material, the mechanical brittleness due to its ceramic nature, and limited sheet resistance on flexible substrates, which limit its application (Hosel et al., 2014; Muhsin et al., 2014). Varied alternative electrodes to ITO have been developed and integrated into PDLC devices including graphene (Kim et al., 2016), silver nanowire (Khaligh et al., 2015), conductive polymer (Boussoualem et al., 2010), metal mesh (Qi et al., 2016) and ultrathin Ag film stacks (Kim et al., 2014). Among these new materials, transparent electrodes based on ultrathin Ag film (normally sandwiched by two oxide layers to form a three-layer oxide/metal/oxide structure) are regarded as a promising alternative because of their superior electrical and tunable optical properties. Eun Mi Kim et al. reported a transparent conductive ZnInSnO/Ag/ZnInSnO multilayer films for PDLC smart windows with a 64% transmittance value at ON state (Kim et al., 2014). Our previous work presented an amino-functionalized sub-40 nm Ag/ZnO transparent electrode, and the Ag/ZnO-based flexible PDLC device exhibited a 65% ON-state specular transmittance (Huang et al., 2017). However, the transmittance of ultrathin Ag film-based PDLC smart windows were still lower than that of commercial ITO based devices (above 70%), which was mainly attributed to the refractive index mismatch inside the devices. Furthermore, due to the reflectance nature of metal, the NIR transmittance of the Ag thin films could be tailored by changing its thickness. Referring to the concept of low emissivity glass with the similar Ag stack structure, ultrathin Ag film electrode could reduce the radiative heat transfer through the window, thus regulating the thermal performance of the PDLC devices. The advantage of this ultrathin Ag film stack electrodes in modulating NIR light of PDLCs is worth exploring and optimizing. However, as we know, the thermal management of PDLC devices is rarely studied.

Normally, in the oxide/metal/oxide structure, the optical and electrical properties are mutually restricted and mainly determined by the thickness and surface morphology of the metal layer. To balance the trade-off between these two properties, the continuous growth of ultrathin Ag films with low percolation threshold becomes a prerequisite to achieve low optical and electrical losses by restraining the traditional three-dimension growth mode (Wang et al., 2014; Chang et al., 2016; Kang et al., 2015). Among various methods to enhance the continuous two-dimension growth of Ag thin films, metal additive is considered to be an effective one. Guo et al. systematically studied the ultra-smooth and low threshold growth after adding Al to Ag (Zhang et al., 2017). Our previous work demonstrated that the Cu dopant into Ag films achieved 6-nm continuous Ag-Cu films with a transmittance of 80% at 550 nm and a sheet resistance of 14.1 Ω/sq (Huang et al., 2018). The continuous growth of ultrathin Ag films paves a way for the device application. However, in the specific application of PDLC devices, the optical interactions between electrodes and other functional layers, as well as optoelectronic properties, need to be further investigated.

In this work, inspired by the concept from low emissivity glass, a TiO_2/Ag(Cu)/TiO_2 structure was simulated and realized to achieve high transmittance in visible spectrum and tunable thermal shielding performance in NIR range simultaneously. The low percolation threshold growth with superior electrical and optical properties were realized by doping little Cu into ultrathin Ag films. Flexible PDLC smart windows device based on this electrode exhibited high visible transmittance of over 80% and demonstrated the thermal shielding performance.

2. Experimental details

2.1. Oxide/Ag/oxide electrode deposition

The TiO_2/Ag(Cu)/TiO_2 transparent electrodes with various Ag(Cu) thickness (7 nm, 9 nm, 11 nm and 13 nm, respectively) were deposited on PET substrates (120 μm, Singyes Materials Co. Ltd., Zhuhai) using a multi-gun sputtering system at room temperature. To observe the growth behavior of the ultrathin Ag(Cu) films, TiO_2/Ag/TiO_2 electrodes with the same thickness were prepared as control samples. The sputtering chamber was evacuated to a base pressure of 5×10^{-4} Pa and 0.7 Pa during sputtering. The TiO_2 films were deposited with a radio frequency (RF) power of 200 W from a 3 in. TiO_2 target. The Ag thin films were deposited using a 3 in. Ag target with a direct current (DC) power of 40 W, while the Ag(Cu) thin films were co-sputtered from Ag and Cu targets with DC power of 40 W and 4 W, respectively. The thickness of the metal and TiO_2 thin films were controlled by deposition time.

2.2. PDLC devices fabrication

The as-deposited PET/TiO_2/Ag(Cu)/TiO_2 and precleaned PET/ITO (100 nm, 80 Ω/sq) were used as transparent electrodes for PDLC devices. A mixture of polymer and nematic liquid crystals was purchased from Shenzhen Broadthink Advanced Materials Tech. Co. Ltd.. Silica microspheres with a mean diameter of 20 μm were used as spacers to control the thickness of the PDLC device. The mixture was then spread on the bottom electrode and covered by the top electrode. The sandwich structure was then exposed to UV light with an intensity of 5.2 mW/cm^2 at 365 nm for 10 min of curing. The size of the PDLC devices were about 10×5 cm^2. Antireflection (AR) coating of hybridized hollow silica nanospheres layers on both sides of the PDLC devices were fabricated using a dip-coated method.

2.3. Characterization and measurement

The thickness and optical constant of films were obtained and fitted using a spectroscopic ellipsometer (J. A. Woollam, M2000DI). The optical transmittances were measured using a UV–Vis-NIR spectrophotometer (Agilent, Cary 5000). The surface morphologies were observed using a field emission scanning electron microscope (FE-SEM, Hitachi S-4800). The sheet resistance was measured using a four-point probe system (NAPSON, Cresbox). To evaluate the performance of the fabricated PDLC device, the specular transmittance-voltage curve was measured using the spectroscopic ellipsometer with an alternating voltage potential (0–30 V) applied across the device.

3. Results and discussion

3.1. Electrode design and optical simulation of PDLC devices

The schematic of the fabrication process of the oxide/Ag/oxide electrode based PDLC device is illustrated in Fig. 1a. An oxide underlying layer, an ultrathin Ag(Cu) metal intermediate layer and an oxide upper layer were deposited sequentially on PET substrate to form a laminated oxide/Ag/oxide electrode structure. Then, the polymer dispersed liquid crystal droplets are cured by ultraviolet light and sandwiched between two symmetrical oxide/Ag/oxide electrodes mentioned above to get a PDLC device. Based on the optical property tunability of the oxide/Ag/oxide electrode in both visible and NIR range, as well as the alignment nature of liquid crystal molecules, a model of PDLC smart window was designed to realize photothermal collaborative management. Fig. 1b depicts two modulation modes in TAT electrode-based PDLC devices. At the OFF state (without applied voltage), the PDLC device strongly scatters both the visible and NIR light due to random orientation of liquid crystals in polymer (Liang et al., 2017). At the ON state (with applied voltage), owing to the liquid crystal alignment, passing of visible light is allowed, while NIR light is partly rejected by TAT electrodes because of its infrared reflection nature.

The first requirement for PDLC-based smart window is the high transmittance, which leads to good visual effect at ON state and high contrast ratio between the two switch states. To verify the optical feasibility of oxide/Ag/oxide electrodes in PDLC device regarding high specular transmittance at ON state, the refractive indices matching

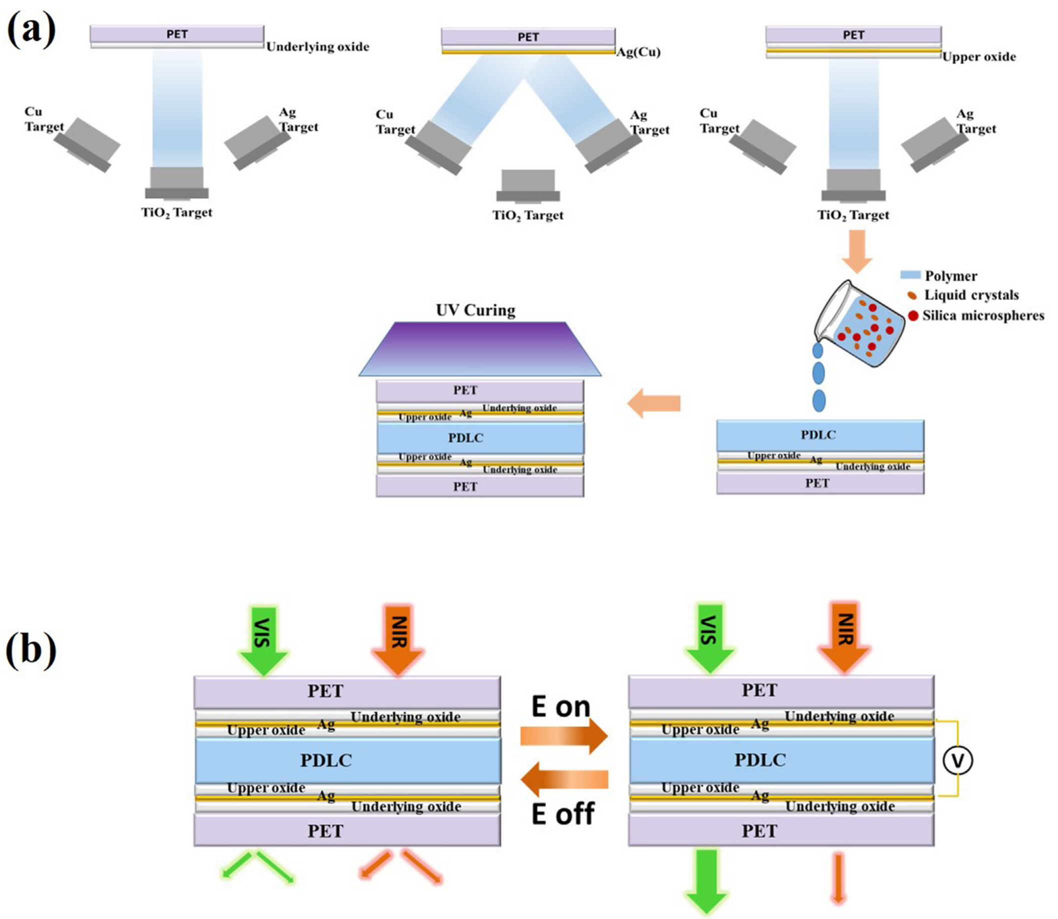

Fig. 1. The schematic of the (a) fabrication process and (b) two optical modulation modes of the oxide/Ag/oxide electrode based PDLC devices.

between oxide/Ag/oxide electrodes and polymer layer was taken into consideration and the transmittance values of the whole PDLC devices were optimized. To begin with, four types of frequently-used metal oxide materials were chosen as alternatives of oxide/Ag/oxide electrode, including TiO_2, ZnO, ITO and Ga doped ZnO (Ga_2O_3 content in the target was 5 wt%, denoted as GZO). Their refractive indices values, together with those of Ag thin film and polymer layer determined experimentally using the spectroscopic ellipsometry, are plotted in Fig. 2a. The transmittance values of the PDLC devices were calculated by the 4 × 4 propagation matrix method using the experimentally derived refractive indices and extinction coefficients (Abdulhalim, 1999). The optimal simulated results of these four-type oxide/Ag/oxide electrode-based devices with the fixed Ag thickness (9 nm) are plotted in Fig. 2b. Here, the reflectance of both PET/Air interfaces was neglected as PET was selected as incident (exit) medium. In order to reduce the interface reflection loss caused by the mismatch of refractive indices among substrate, oxide/Ag/oxide electrode and polymer layers, an effective refractive index value which is close to that of PET substrate and polymer layer (1.5 ~ 1.6) is desired for oxide/Ag/oxide electrode. As described in our previous work, ultrathin Ag films-based multilayers could be approximately converted to a single effective layer using effective-medium theory (EMT) (Huang et al., 2017). The calculated average effective refractive indices were about 1.69, 1.35, 1.14 and 0.96 for corresponding TiO_2, ZnO, ITO and GZO oxide materials, respectively. Among these, device using TiO_2/Ag/TiO_2 (denoted as TAT) electrodes exhibited the highest visible transmittance than others. Therefore, TiO_2/Ag/TiO_2 (TAT) structure was chosen as electrode for PDLC devices in this work.

Fig. 2c demonstrates the dependence of average visible transmittance (400–780 nm) of TAT-based device on the upper and underlying TiO_2 thickness variation with Ag thickness fixed at 9 nm. The maximum transmittance value reached 92.6% when thicknesses of both upper and underlying TiO_2 were about 35 nm. The optimal calculated transmittance and reflectance spectra of TAT-based PDLC devices are shown in Fig. 2d. The simulated transmittance values of the TAT-based PDLC devices were 92.5%, 92.6%, 91.6% and 88.6% for four different Ag film thicknesses of 7 nm, 9 nm, 11 nm and 13 nm, respectively. It demonstrated the feasible application of TAT electrode in PDLC devices. Furthermore, the transmittance and reflectance behavior could be tailored through adjustment of Ag films' thickness.

3.2. Fabrication and performance of transparent electrodes

To achieve the consistency of the experimental results with the simulated ones, the continuous growth behavior of Ag thin films with a low percolation threshold thickness and restrained 3D Vomer-Weber mode is a prerequisite (Yun, 2017). An even worse wetting behavior of Ag thin films on TiO_2 underlayer than that of other frequently-used oxide materials such as ZnO was reported, because of the stronger Ti-O bonding than Zn-O bonding (Campbell, 1997). Our previous work reported an improved growth of ultrathin Ag film with a low threshold thickness of 6 nm by Cu doping. Co-sputtering of Cu during Ag growth was expected to lower the surface diffusion of the Ag atom, thus modify the Ag thin film surface morphology towards two-dimensional plane like, with smaller and denser clusters than those of pure Ag. The atomic concentration of Cu was about 6% in the Ag(Cu) thin films, which was confirmed by X-ray photoelectron spectroscopy analysis (Huang et al., 2018). Herein, the growth behavior of Ag(Cu) thin films sandwiched between double TiO_2 layers were evaluated. Fig. 3 records the SEM micrographs of PET/TiO_2/Ag and PET/TiO_2/Ag(Cu) films as the metal

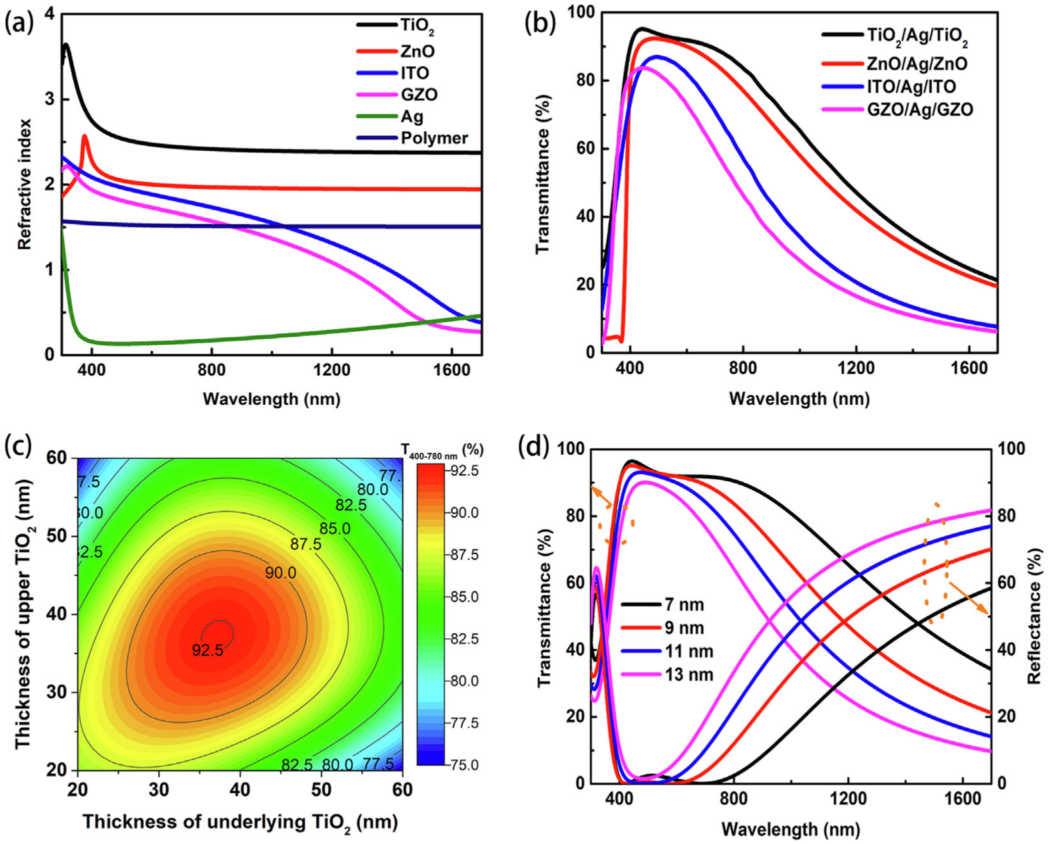

Fig. 2. (a) Refractive index of the materials using in simulations. (b) Optimal simulated transmittances spectra of PDLC devices based on different oxide/Ag/oxide electrodes (the thickness of Ag thin film was set at 9 nm). (c) Simulated average visible transmittance (400–780 nm) of TAT based PDLC device in ON state as a function of thicknesses of upper and underlying TiO_2. The thickness of Ag was 9 nm. (d) Optimal simulated transmittance and reflectance spectra of TAT based PDLC devices with change in the Ag thickness.

thickness increases. Comparing to pure Ag thin films, the Ag(Cu) thin films on TiO_2 showed a more dense and homogeneous morphology at 7 nm and further growth. The result demonstrated a better wetting behavior of Ag thin films on TiO_2 underlayer after doping a small amount of Cu. The better wetting effect and higher nuclei density on TiO_2 underlayer might be attributed to higher surface energy of Cu (1.96 J m^{-2}) than that of Ag (1.2–1.42 J m^{-2}) (Formica et al., 2013).

Fig. 4a compare the optical transmittance of the TAT and TiO_2/Ag(Cu)/TiO_2 (denoted as TCAT) multilayers on PET substrates with varied metal films thicknesses (7 nm, 9 nm, 11 nm and 13 nm, respectively). For TAT multilayers with lower Ag thickness (< 13 nm), broad dips were observed (insert of Fig. 4a), since absorption was related to the localized surface plasmon resonance of discontinuous Ag island morphology (Gu et al., 2014; Peres et al., 2016). However, after a small amount of Cu was doped into Ag films, the transmittances of TCAT multilayers in the whole visible range were improved significantly compared to that of TAT multilayers with the corresponding thickness. It further proved the continuous growth of the Ag(Cu) thin films, which was in accordance with the morphology observation in Fig. 3. The average visible transmittance values for both TAT and TCAT multilayers were calculated and compared with the simulated results in Fig. 4b. For TAT multilayers with lower Ag thickness (< 13 nm), the average visible transmittances were fairly low, deviating far from the simulated data. This could be explained by the absorption and scattering of the discontinuous Ag thin films. Increasing Ag thickness to 13 nm improved the transmittance of TAT, but there still existed a gap between experimental data and simulated one. Comparatively, the average visible transmittances of TCAT electrodes were in better consistence with simulated data, suggesting a continuous morphology of Ag (Cu) mid-layer without unwanted absorption. Fig. 4c plots the measured sheet resistance values of TAT and TCAT electrodes with different Ag thicknesses. Obviously, the sheet resistances of TCAT were much lower than those of TAT with metal thickness < 13 nm. For instance, regarding the metal layer with a thickness of 9 nm, the sheet

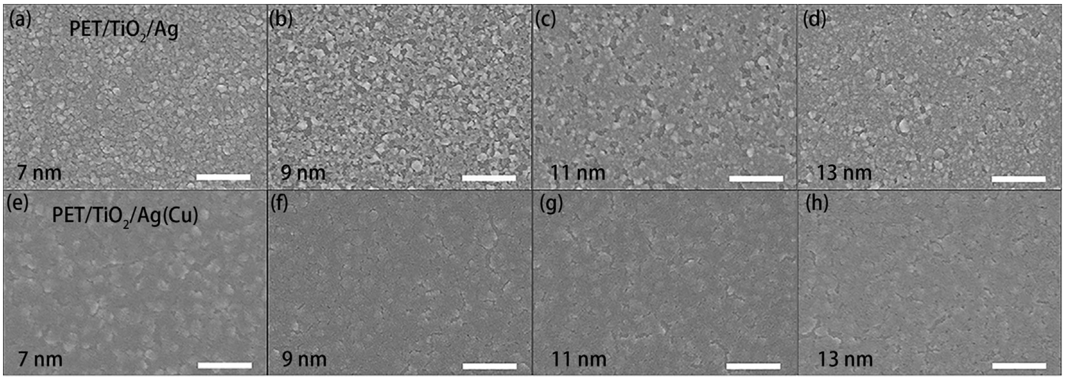

Fig. 3. (a)–(d), (e)–(h) Surface SEM micrographs of PET/TiO_2/Ag and PET/TiO_2/Ag(Cu) films with increasing metal thickness, respectively. The atomic concentration of Cu was around 6% in the obtained Ag(Cu) thin film.

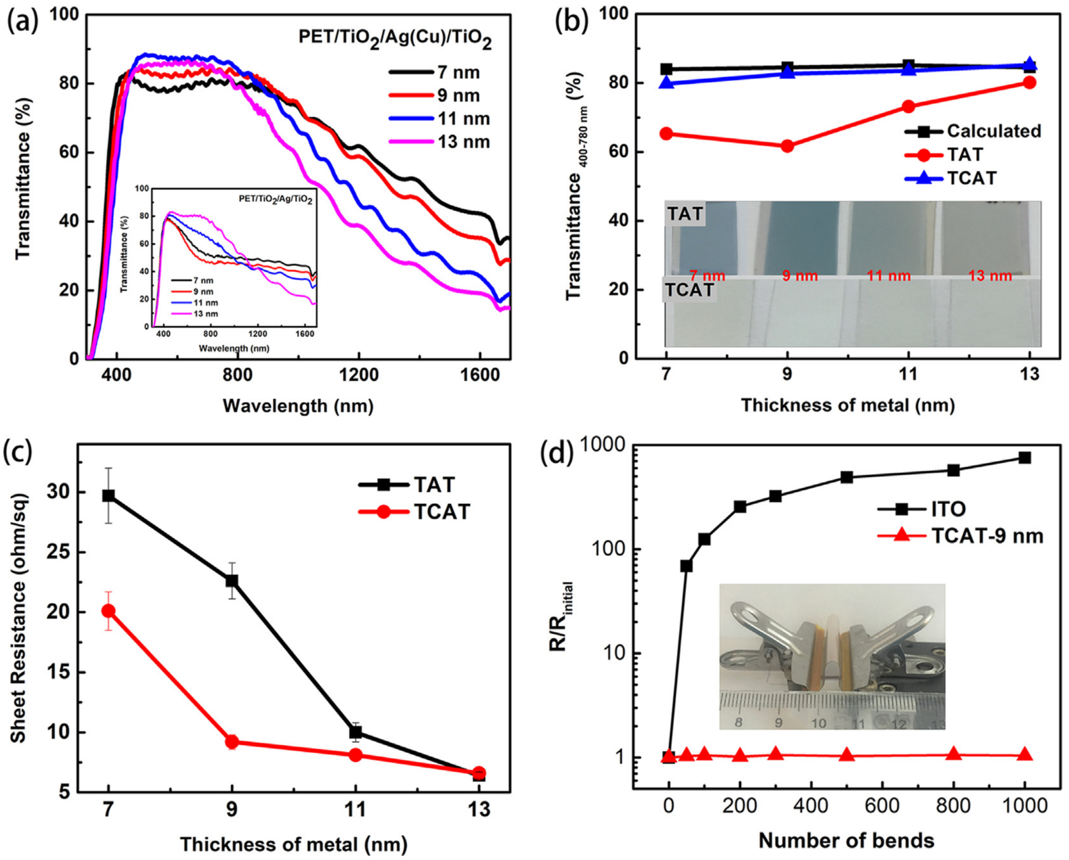

Fig. 4. (a) Transmittance spectra of TAT and TCAT electrodes on PET substrates with different metal thickness. (b) Simulated and measured average visible transmittance of TAT and TCAT electrodes with different metal thickness. Inset showed the photographic images of TAT and TCAT electrodes with different metal thicknesses. (c) Sheet resistance of TAT and TCAT electrodes with different metal thickness. (d) The change of the sheet resistance values of TACT-9 nm electrode and ITO control sample as a function of the number of bends at a fixed bending radius of 2 mm.

resistances of TCAT and TAT electrodes were 9.2 Ω/sq and 22.6 Ω/sq respectively. Fig. 4d evaluates the change of the sheet resistance values of TACT-9 nm electrode and ITO control sample as a function of the number of bends at a fixed bending radius of 2 mm. The change in the sheet resistance of the electrodes could be expressed as R/R_0, where R_0 and R were the sheet resistance values at the initial state and after bending, respectively. As shown in Fig. 4d, no obvious changes in resistance were observed for the TACT-9 nm electrode after 1000 bending cycles. In contrast, the resistance of the commercial ITO increased significantly by about 100 times after only 50 bending cycles.

3.3. Performance of PDLC based smart windows

The as-prepared TCAT transparent electrodes with different metal thicknesses on PET substrates were applied in PDLC devices. Commercially available ITO electrodes were also used for comparison. Fig. 5a exhibits the photographic images of the PDLC device based on TCAT electrodes at the OFF and ON states with an AC voltage of 25 V. The high contrast ratio with good uniformity indicated the feasibility of substituting the traditional ITO electrode by the ultrathin Ag multilayer. Fig. 5b shows the specular transmittance-voltage curves of the TCAT electrode based PDLC devices and ITO-based control sample at the wavelength of 550 nm. All the PDLC devices based on TCAT electrodes, except TCAT-7 nm, displayed comparable performances than that of ITO electrodes. The relatively poor transmittance of TCAT-7 nm based PDLC was mainly attributed to the inferior transmittance of TCAT-7 nm electrodes as described in Fig. 4a. Furthermore, the average transmittance of the device in the visible range was further improved by combined with antireflection coating (ARC) layers on both sides of the PET substrate, which reduced the unwanted reflection at the PET/Air interfaces. In our previous work, transmittance value of the PET or glass substrates was increased by about 5% through fabrication of single-layer SiO_2 ARC coatings with the appropriate refractive index according to the relation of $n = \sqrt{n_{air} n_{substrate}}$ (Zhang et al., 2014). Herein, SiO_2 ARC layers were integrated into the PDLC device structure by dipping coated on both sides of the PET surfaces to further increase the transmittance of the PDLC device. The measured average transmittance of the devices was compared with the calculated ones (Fig. 5c). As expected, the average visible transmittance values for all the TCAT-based PDLC devices were improved obviously, reaching about 81.9% (TCAT-7 nm), 84.9% (TCAT-9 nm), 81.9% (TCAT-11 nm) and 79.9% (TCAT-13 nm), respectively. In addition, the variation of average visible transmittance of TCAT-based PDLCs with Ag(Cu) thickness was in good consistent with the calculated result. However, there still existed a gap between the calculated and measured results, which mainly came from residual reflections at the PET/Air interfaces after a single SiO_2 ARC layer. Fig. 5d plotted the normalized transmittance values of the TCAT-9 nm based PDLC device changing with ON-OFF switching cycles at 550 nm. As can be seen, the device has endured 300 cycles with no evident signs of degradation, which demonstrated the stability of the PDLC device based on the oxide/Ag/oxide electrode.

Table 1 summarizes the key parameters for evaluating the performance and characteristics of TCAT and ITO electrodes, including visible transmittance (T_v), emissivity (ε), heat transfer coefficient (U), solar heat gain coefficient ($SHGC$) and light to solar gain ratio (LSG). T_v is the visible transmittance value of the sample at 550 nm. U indicates the heat loss arisen from indoor and outdoor environment temperature difference. $SHGC$ is the measure of solar thermal energy transmitted

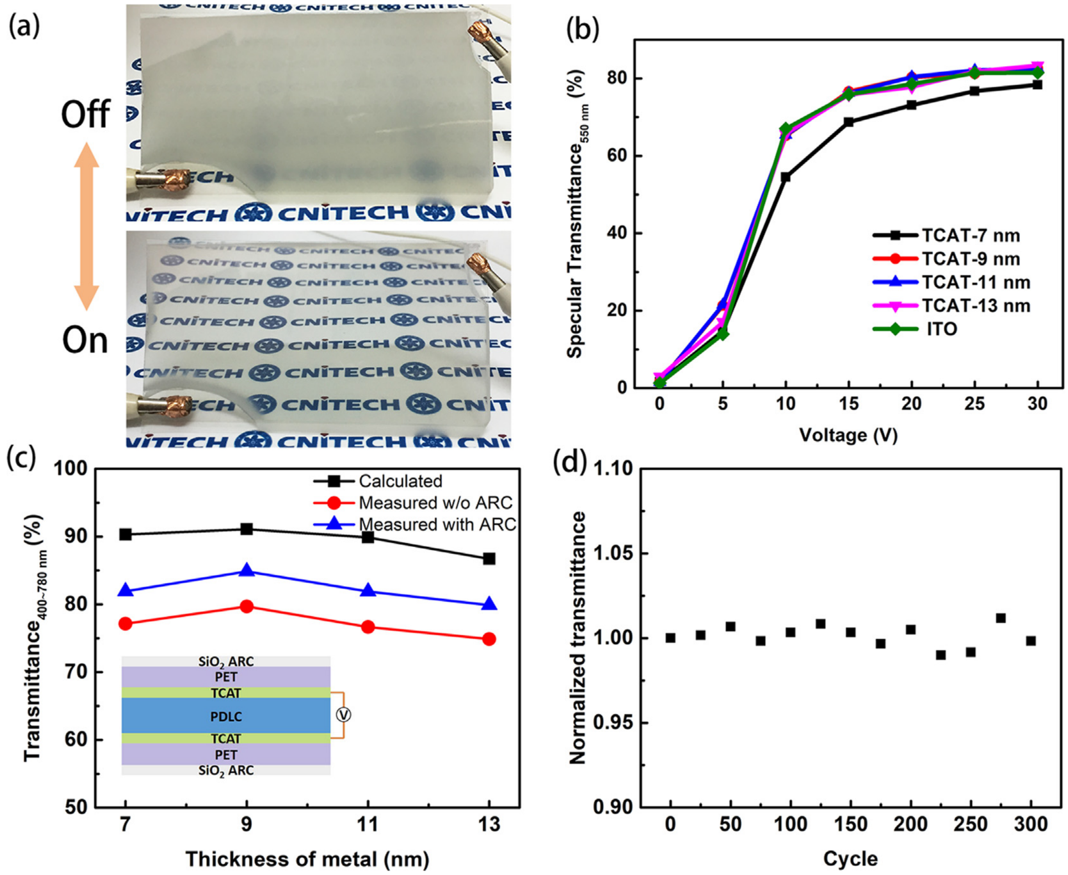

Fig. 5. (a) Photographic images of the PDLC device with the TCAT electrodes at the off state and ON state. (b) Specular transmittance-voltage curves of PDLCs at 550 nm. (c) Calculated and measured average visible transmittance of PDLCs devices with and without SiO_2 AR coatings. (d) Normalized transmittance values of the TCAT-9 nm PDLC device changing with ON-OFF switching cycles at 550 nm.

Table 1
Summary of samples with visible transmittance (***T_v***), emissivity (ε), heat transfer coefficient (U), solar heat gain coefficient (*SHGC*) and light to solar gain ratio (*LSG*).

Sample	T_v (550 nm)	ε	U (W/m^2 K)	*SHGC*	*LSG*
TCAT-7 nm	78.1	0.260	6.22	0.74	1.05
TCAT-9 nm	82.2	0.119	6.19	0.74	1.11
TCAT-11 nm	87.7	0.104	6.18	0.71	1.23
TCAT-13 nm	85.5	0.085	6.18	0.68	1.25
ITO	82.3	0.566	6.28	0.81	1.02

directly or indirectly (absorbed and then transmitted inward) with a range of 0–1, in which higher value means higher solar heat transmitted. The lower the *SHGC* value, the less solar heat transmitted and the greater the shading ability (Arbab and Finley, 2010). The emissivity and sheet resistance are directly proportional (Koubli et al., 2015). The emissivity values of the TCAT electrodes were calculated using the following formula (Loka et al., 2016):

$$\varepsilon = 0.0129 \cdot R_{\blacksquare} - 6.7 \times 10^{-5} R_{\blacksquare}^2 \quad (1)$$

where $R_{\blacksquare}$ is the sheet resistance of the electrode. The U and *SHGC* values were calculated using the Windows5 and Optics5 software. Herein, we defined *LSG* value as follows by considering both the visible transmittance and the heat shielding performance in NIR wavelength range:

$$LSG = \frac{T_v(550\ \text{nm})}{SHGC} \quad (2)$$

High LSG represents the relative efficiency of glazing materials and their ability to transmit daylight while blocking heat gain. As metal thickness increased beyond 9 nm, the TCAT electrodes showed comparative and even higher transmittance value at 550 nm compared with ITO control sample. It was clear that all the TCAT electrodes outperformed ITO sample in terms of ε, U and *SHGC* values due to their high conductivity and reflectance performance in the NIR range. As a result, high-quality TCAT electrodes are characterized by high *LSG* value.

As compared above, the TCAT electrodes based PDLC devices were superior to ITO counterpart regarding heat shielding performance while keeping the comparable visible transmittance in visual effect. Prototype experiments were conducted to verify the heat shielding effect using a halogen lamp. Each device was placed between the lamp and thermocouple for 10 min, and then the temperature reading was recorded (Fig. 6). The surface temperature climbed up to 46 °C after irradiation with only two bare PET substrates. At the OFF state, all the samples exhibited excellent heat shielding performance due to the strong scattering effect of the disordered liquid crystal alignment. At this state, the differences in transmittance between the TCAT and ITO samples were less obvious, which led to a minor temperature difference. However, at the ON state, TCAT-based devices showed stronger heat shielding effect than that ITO-based ones. As shown in the inset of Fig. 6a, the surface temperatures of the ITO and TCAT-13 nm based devices were 39.3 °C and 34.7 °C, respectively.

To better understand this phenomenon, specular transmittance curves of PDLC devices based on TCAT and ITO electrodes at ON and OFF states are plotted in Fig. 6b, respectively. In visible range, the two types of PDLC devices showed the comparable high transmittance value at ON state and close to zero transmittance value at OFF state, fully meeting the requirements of building lighting and privacy protection. However, in NIR range, the transmittance of TCAT electrode based

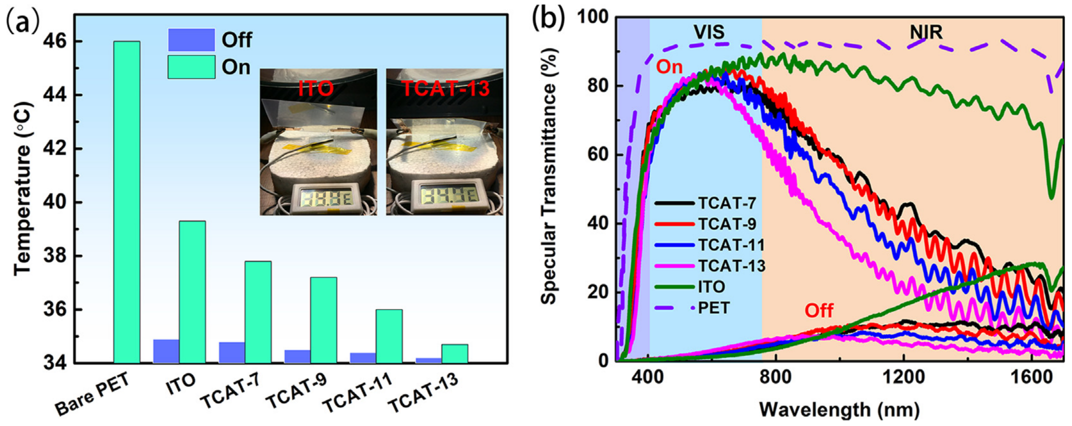

Fig. 6. (a) Heat-shielding performance of PDLCs. Insets showed PDLC devices as heat-shielding windows under halogen lamp irradiation. (b) Specular transmittance curves of PDLC devices in ON and OFF states.

PDLC devices declined obviously due to the high reflectance of the Ag thin film, which contributed to the dramatic decrease of the measured temperature. In general, the smart window made of the TCAT electrode-based PDLC exhibited good shielding performance under visible light at OFF state, and high transmittance, as well as excellent heat shielding performance at ON state. Hence it was best applicable to blocking out the unneeded solar heat and save cooling energy consumption in the summer season or hot areas.

4. Conclusions

In summary, we designed a TiO_2/Ag(Cu)/TiO_2 electrode which was suitable for PDLC smart window device to realize high transmission and heat shielding simultaneously. The optimized thicknesses of top and bottom TiO_2 (around 35 nm) and Ag interlayer were simulated to reach a maximum specular transmittance of the PDLC device in the visible range. A low threshold percolation continuous growth of Ag thin films was realized through doping a small amount of Cu into Ag. Experimentally, at an applied voltage of 30 V, the average specular transmittance of the TiO_2/Ag(Cu)/TiO_2 electrode based PDLC devices with antireflective coatings on both sides reached about 81.9%, 84.9%, 81.9% and 79.9% for Ag thin film thicknesses of 7 nm, 9 nm, 11 nm and 13 nm, respectively. *LSG* values were evaluated by considering the trade-off between the visible transmittance and the heat shielding performance. All the TCAT electrodes outperformed ITO sample in terms of ε, *U*, *SHGC* and *LSG* values. To demonstrate, a notable temperature decrease of 4.6 °C was observed comparing with ITO counterpart, exhibiting an excellent heat shielding performance. The designed PDLC device featuring high transmittance and good heat shielding performance demonstrated a potential application of smart window in summer season or hot areas, contributing to building energy conservation.

Acknowledgements

This work was supported by National Natural Science Foundation of China (61774160), Zhejiang Natural Science Foundation (LY19F040003), International Cooperation Program of Ningbo (2017D10005), Ningbo Natural Science Foundation (2018A610142) and the Program for Ningbo Municipal Science and Technology Innovative Research Team (Grant No. 2016B10005).

References

Abdulhalim, I., 1999. Analytic propagation matrix method for linear optics of arbitrary biaxial layered media. J. Opt. a-Pure Appl. Op. 1 (5), 646–653.
Alghamedi, R., Vasiliev, M., Nur-E-Alam, M., Alameh, K., 2014. Spectrally-selective all-inorganic scattering luminophores for solar energy-harvesting clear glass windows. Sci. Rep. 4, 6632.
Arbab, M., Finley, J.J., 2010. Glass in architecture. Int. J. Appl. Glass Sci. 1 (1), 118–129.
Boussoualem, M., King, R.C.Y., Brun, J.F., Duponchel, B., Ismaili, M., Roussel, F., 2010. Electro-optic and dielectric properties of optical switching devices based on liquid crystal dispersions and driven by conducting polymer [poly(3,4-ethylene dioxythiophene):polystyrene sulfonate (PEDOT:PSS)]-coated electrodes. J. Appl. Phys. 108 (11), 113526.
Campbell, C.T., 1997. Ultrathin metal films and particles on oxide surfaces: structural, electronic and chemisorptive properties. Surf. Sci. Rep. 27 (1–3), 1–111.
Chang, C.Y., Chang, Y.C., Huang, W.K., Liao, W.C., Wang, H., Yeh, C., Tsai, B.C., Huang, Y.C., Tsao, C.S., 2016. Achieving high efficiency and improved stability in large-area ITO-free perovskite solar cells with thiol-functionalized self-assembled monolayers. J. Mater. Chem. A 4 (20), 7903–7913.
Formica, N., Ghosh, D.S., Carrilero, A., Chen, T.L., Simpson, R.E., Pruneri, V., 2013. Ultrastable and atomically smooth ultrathin silver films grown on a copper seed layer. ACS Appl. Mater. Inter. 5 (8), 3048–3053.
Gu, D., Zhang, C., Wu, Y.K., Guo, L.J., 2014. Ultrasmooth and thermally stable silver-based thin films with subnanometer roughness by aluminum doping. ACS Nano. 8 (10), 10343–10351.
Hosel, M., Angmo, D., Sondergaard, R.R., Benatto, G.A.D., Carle, J.E., Jorgensen, M., Krebs, F.C., 2014. High-volume processed, ITO-free superstrates and substrates for roll-to-roll development of organic electronics. Adv. Sci. 1 (1), 1400002.
Huang, J.H., Lu, Y.H., Wu, W.X., Li, J., Zhang, X.P., Zhu, C.T., Yang, Y., Xu, F., Song, W.J., 2017. Amino-functionalized sub-40nm ultrathin Ag/ZnO transparent electrodes for flexible polymer dispersed liquid crystal devices. J. Appl. Phys. 122 (19), 195302.
Huang, J.H., Liu, X.H., Lu, Y.H., Zhou, Y.H., Xu, J.J., Li, J., Wang, H.Q., Fang, J.F., Yang, Y., Wang, W.Y., Tan, R.Q., Song, W.J., 2018. Seed-layer-free growth of ultrathin Ag transparent conductive films imparts flexibility to polymer solar cells. Sol. Energy Mat. Sol. C 184, 73–81.
Kang, H., Jung, S., Jeong, S., Kim, G., Lee, K., 2015. Polymer-metal hybrid transparent electrodes for flexible electronics. Nat. Commun. 6, 6503.
Khaligh, H.H., Liew, K., Han, Y.N., Abukhdeir, N.M., Goldthorpe, I.A., 2015. Silver nanowire transparent electrodes for liquid crystal-based smart windows. Sol. Energy Mat. Sol. C 132, 337–341.
Khandelwal, H., Schenning, A.P.H.J., Debije, M.G., 2017. Infrared regulating smart window based on organic materials. Adv. Energy Mater. 7 (14), 1602209.
Kim, E.M., Choi, I.S., Oh, J.P., Kim, Y.B., Lee, J.H., Choi, Y.S., Cho, J.D., Kim, Y.B., Heo, G.S., 2014. Transparent conductive ZnInSnO-Ag-ZnInSnO multilayer films for polymer dispersed liquid-crystal based smart windows. Jpn. J. Appl. Phys. 53 (9), 095505.
Kim, Y., Kim, K., Kim, K.B., Park, J.Y., Lee, N., Seo, Y., 2016. Flexible polymer dispersed liquid crystal film with graphene transparent electrodes. Curr. Appl. Phys. 16 (3), 409–414.
Koubli, E., Tsakanikas, S., Leftheriotis, G., Syrrokostas, G., Yianoulis, P., 2015. Optical properties and stability of near-optimum WO3/Ag/WO3 multilayers for electrochromic applications. Solid State Ion. 272, 30–38.
Liang, X., Chen, M., Guo, S.M., Zhang, L.Y., Li, F.S., Yang, H., 2017. Dual-band modulation of visible and near-infrared light transmittance in an all-solution-processed hybrid micro -nano composite film. ACS Appl. Mater. Inter. 9 (46), 40810–40819.
Liang, R.Q., Sun, Y.Y., Aburas, M., Wilson, R., Wu, Y.P., 2018. Evaluation of the thermal and optical performance of thermochromic windows for office buildings in China. Energy Build. 176, 216–231.
Loka, C., Park, K.R., Lee, K.S., 2016. SiO2/TiO2/n-Si/Ag(Cr)/TiO2 thin films with superhydrophilicity and low-emissivity. Jpn. J. Appl. Phys. 55 (1), 01AA06.
Muhsin, B., Roesch, R., Gobsch, G., Hoppe, H., 2014. Flexible ITO-free polymer solar cells based on highly conductive PEDOT:PSS and a printed silver grid. Sol. Energy Mat. Sol. C 130, 551–554.
Murray, J., Ma, D.K., Munday, J.N., 2017. Electrically controllable light trapping for self-powered switchable solar windows. ACS Photon. 4 (1), 1–7.
Peres, L., Bou, A., Barakel, D., Torchio, P., 2016. ZnS/Ag/TiO_2 multilayer electrodes with broadband transparency for thin film solar cells. RSC Adv. 6 (66), 61057–61063.

Qi, L.F., Li, J., Zhu, C.T., Yang, Y., Zhao, S.J., Song, W.J., 2016. Realization of a flexible and mechanically robust Ag mesh transparent electrode and its application in a PDLC device. RSC Adv. 6 (16), 13531–13536.
Sabry, M., Eames, P.C., Singh, H., Wu, Y.P., 2014. Smart windows: thermal modelling and evaluation. Sol Energy 103, 200–209.
Wang, Y., Runnerstrom, E.L., Milliron, D.J., 2016. Switchable materials for smart windows. Ann. Rev. Chem. Biomol. 7, 283–304.
Wang, W., Song, M., Bae, T.S., Park, Y.H., Kang, Y.C., Lee, S.G., Kim, S.Y., Kim, D.H., Lee, S., Min, G.H., Lee, G.H., Kang, J.W., Yun, J., 2014. Transparent ultrathin oxygen-doped silver electrodes for flexible organic solar cells. Adv. Funct. Mater. 24 (11), 1551–1561.
Wang, R., Zhai, X., 2018. Handbook of energy systems in green buildings, first ed. Springer, Germany.
Yun, J., 2017. Ultrathin metal films for transparent electrodes of flexible optoelectronic devices. Adv. Funct. Mater. 27 (18), 1606641.
Zakirullin, R.S., Letuta, S.N., 2015. A smart window for angular selective filtering solar radiation. Sol Energy 120, 585–592.
Zhang, C., Kinsey, N., Chen, L., Ji, C.G., Xu, M.J., Ferrera, M., Pan, X.Q., Shalaev, V.M., Boltasseva, A., Guo, L.J., 2017. High-performance doped silver films: overcoming fundamental material limits for nanophotonic applications. Adv. Mater. 29 (19), 1500768.
Zhang, J., Lan, P.J., Li, J., Xu, H., Wang, Q., Zhang, X.P., Zheng, L.R., Lu, Y.H., Dai, N., Song, W.J., 2014. Sol-gel derived near-UV and visible antireflection coatings from hybridized hollow silica nanospheres. J. Sol-Gel Sci. Tech. 71 (2), 267–275.

JOHN WILEY AND SONS LICENSE TERMS AND CONDITIONS

Sep 19, 2022

This Agreement between Harbin Institute of Technology -- Ruicong Zhang ("You") and John Wiley and Sons ("John Wiley and Sons") consists of your license details and the terms and conditions provided by John Wiley and Sons and Copyright Clearance Center.

License Number	5392430199276
License date	Sep 19, 2022
Licensed Content Publisher	John Wiley and Sons
Licensed Content Publication	Advanced Materials
Licensed Content Title	Thermally Induced, Multicolored Hyper-Reflective Cholesteric Liquid Crystals
Licensed Content Author	Timothy J. Bunning, Timothy J. White, Lalgudi V. Natarajan, et al
Licensed Content Date	Feb 2, 2011
Licensed Content Volume	23
Licensed Content Issue	12
Licensed Content Pages	5
Type of Use	Journal/Magazine
Requestor type	University/Academic
Is the reuse sponsored by or associated with a pharmaceutical or medical products company?	no
Format	Print and electronic
Portion	Figure/table
Number of figures/tables	2
Will you be translating?	No
Circulation	200 - 499
Title of new article	Advanced liquid crystal-based switchable optical devices for light protection applications: principles and strategies
Lead author	Ruicong Zhang, Zhibo Zhang, Jiecai Han, Lei Yang, Jiajun li, Zicheng Song Tianyu Wang, Jiaqi Zhu
Title of targeted journal	Light: Science & Applications
Publisher	Springer Nature
Expected publication date	Nov 2022
Portions	Figure 1,Figure 3
Requestor Location	Harbin Institute of Technology<br>No. 92, Xidazhi Street, Nangang District<br><br>Harbin, 150080<br>China<br>Attn: Harbin Institute of Technology
Publisher Tax ID	EU826007151
Total	**0.00 USD**
Terms and Conditions	

TERMS AND CONDITIONS

This copyrighted material is owned by or exclusively licensed to John Wiley & Sons, Inc. or one of its group companies (each a"Wiley Company") or handled on behalf of a society with which a Wiley Company has exclusive publishing rights in relation to a particular work (collectively "WILEY"). By clicking "accept" in connection with completing this licensing transaction, you agree that the following terms and conditions apply to this transaction (along with the billing and payment terms and conditions

established by the Copyright Clearance Center Inc., ("CCC's Billing and Payment terms and conditions"), at the time that you opened your RightsLink account (these are available at any time at http://myaccount.copyright.com).

Terms and Conditions

- The materials you have requested permission to reproduce or reuse (the "Wiley Materials") are protected by copyright.

- You are hereby granted a personal, non-exclusive, non-sub licensable (on a stand-alone basis), non-transferable, worldwide, limited license to reproduce the Wiley Materials for the purpose specified in the licensing process. This license, **and any CONTENT (PDF or image file) purchased as part of your order,** is for a one-time use only and limited to any maximum distribution number specified in the license. The first instance of republication or reuse granted by this license must be completed within two years of the date of the grant of this license (although copies prepared before the end date may be distributed thereafter). The Wiley Materials shall not be used in any other manner or for any other purpose, beyond what is granted in the license. Permission is granted subject to an appropriate acknowledgement given to the author, title of the material/book/journal and the publisher. You shall also duplicate the copyright notice that appears in the Wiley publication in your use of the Wiley Material. Permission is also granted on the understanding that nowhere in the text is a previously published source acknowledged for all or part of this Wiley Material. Any third party content is expressly excluded from this permission.

- With respect to the Wiley Materials, all rights are reserved. Except as expressly granted by the terms of the license, no part of the Wiley Materials may be copied, modified, adapted (except for minor reformatting required by the new Publication), translated, reproduced, transferred or distributed, in any form or by any means, and no derivative works may be made based on the Wiley Materials without the prior permission of the respective copyright owner.**For STM Signatory Publishers clearing permission under the terms of the STM Permissions Guidelines only, the terms of the license are extended to include subsequent editions and for editions in other languages, provided such editions are for the work as a whole in situ and does not involve the separate exploitation of the permitted figures or extracts,** You may not alter, remove or suppress in any manner any copyright, trademark or other notices displayed by the Wiley Materials. You may not license, rent, sell, loan, lease, pledge, offer as security, transfer or assign the Wiley Materials on a stand-alone basis, or any of the rights granted to you hereunder to any other person.

- The Wiley Materials and all of the intellectual property rights therein shall at all times remain the exclusive property of John Wiley & Sons Inc, the Wiley Companies, or their respective licensors, and your interest therein is only that of having possession of and the right to reproduce the Wiley Materials pursuant to Section 2 herein during the continuance of this Agreement. You agree that you own no right, title or interest in or to the Wiley Materials or any of the intellectual property rights therein. You shall have no rights hereunder other than the license as provided for above in Section 2. No right, license or interest to any trademark, trade name, service mark or other branding ("Marks") of WILEY or its licensors is granted hereunder, and you agree that you shall not assert any such right, license or interest with respect thereto

- NEITHER WILEY NOR ITS LICENSORS MAKES ANY WARRANTY OR REPRESENTATION OF ANY KIND TO YOU OR ANY THIRD PARTY, EXPRESS, IMPLIED OR STATUTORY, WITH RESPECT TO THE MATERIALS OR THE ACCURACY OF ANY INFORMATION CONTAINED IN THE MATERIALS, INCLUDING, WITHOUT LIMITATION, ANY IMPLIED WARRANTY OF MERCHANTABILITY, ACCURACY, SATISFACTORY QUALITY, FITNESS FOR A PARTICULAR PURPOSE, USABILITY, INTEGRATION OR NON-INFRINGEMENT AND ALL SUCH WARRANTIES ARE HEREBY EXCLUDED BY WILEY AND ITS LICENSORS AND WAIVED BY YOU.

- WILEY shall have the right to terminate this Agreement immediately upon breach of this Agreement by you.

- You shall indemnify, defend and hold harmless WILEY, its Licensors and their respective directors, officers, agents and employees, from and against any actual or threatened claims, demands, causes of action or proceedings arising from any breach of this Agreement by you.

- IN NO EVENT SHALL WILEY OR ITS LICENSORS BE LIABLE TO YOU OR ANY OTHER PARTY OR ANY OTHER PERSON OR ENTITY FOR ANY SPECIAL, CONSEQUENTIAL, INCIDENTAL, INDIRECT, EXEMPLARY OR PUNITIVE DAMAGES, HOWEVER CAUSED, ARISING OUT OF OR IN CONNECTION WITH THE DOWNLOADING, PROVISIONING, VIEWING OR USE OF THE MATERIALS REGARDLESS OF THE FORM OF ACTION, WHETHER FOR BREACH OF CONTRACT, BREACH OF WARRANTY, TORT, NEGLIGENCE, INFRINGEMENT OR OTHERWISE (INCLUDING, WITHOUT LIMITATION, DAMAGES BASED ON LOSS OF PROFITS, DATA, FILES, USE, BUSINESS OPPORTUNITY OR CLAIMS OF THIRD PARTIES), AND WHETHER OR NOT THE PARTY HAS BEEN ADVISED OF THE POSSIBILITY OF SUCH DAMAGES. THIS LIMITATION SHALL APPLY NOTWITHSTANDING ANY FAILURE OF ESSENTIAL PURPOSE OF ANY LIMITED REMEDY PROVIDED HEREIN.

- Should any provision of this Agreement be held by a court of competent jurisdiction to be illegal, invalid, or unenforceable, that provision shall be deemed amended to achieve as nearly as possible the same economic effect as the original provision, and the legality, validity and enforceability of the remaining provisions of this Agreement shall not

be affected or impaired thereby.

- The failure of either party to enforce any term or condition of this Agreement shall not constitute a waiver of either party's right to enforce each and every term and condition of this Agreement. No breach under this agreement shall be deemed waived or excused by either party unless such waiver or consent is in writing signed by the party granting such waiver or consent. The waiver by or consent of a party to a breach of any provision of this Agreement shall not operate or be construed as a waiver of or consent to any other or subsequent breach by such other party.
- This Agreement may not be assigned (including by operation of law or otherwise) by you without WILEY's prior written consent.
- Any fee required for this permission shall be non-refundable after thirty (30) days from receipt by the CCC.
- These terms and conditions together with CCC's Billing and Payment terms and conditions (which are incorporated herein) form the entire agreement between you and WILEY concerning this licensing transaction and (in the absence of fraud) supersedes all prior agreements and representations of the parties, oral or written. This Agreement may not be amended except in writing signed by both parties. This Agreement shall be binding upon and inure to the benefit of the parties' successors, legal representatives, and authorized assigns.
- In the event of any conflict between your obligations established by these terms and conditions and those established by CCC's Billing and Payment terms and conditions, these terms and conditions shall prevail.
- WILEY expressly reserves all rights not specifically granted in the combination of (i) the license details provided by you and accepted in the course of this licensing transaction, (ii) these terms and conditions and (iii) CCC's Billing and Payment terms and conditions.
- This Agreement will be void if the Type of Use, Format, Circulation, or Requestor Type was misrepresented during the licensing process.
- This Agreement shall be governed by and construed in accordance with the laws of the State of New York, USA, without regards to such state's conflict of law rules. Any legal action, suit or proceeding arising out of or relating to these Terms and Conditions or the breach thereof shall be instituted in a court of competent jurisdiction in New York County in the State of New York in the United States of America and each party hereby consents and submits to the personal jurisdiction of such court, waives any objection to venue in such court and consents to service of process by registered or certified mail, return receipt requested, at the last known address of such party.

WILEY OPEN ACCESS TERMS AND CONDITIONS
Wiley Publishes Open Access Articles in fully Open Access Journals and in Subscription journals offering Online Open. Although most of the fully Open Access journals publish open access articles under the terms of the Creative Commons Attribution (CC BY) License only, the subscription journals and a few of the Open Access Journals offer a choice of Creative Commons Licenses. The license type is clearly identified on the article.
The Creative Commons Attribution License
The Creative Commons Attribution License (CC-BY) allows users to copy, distribute and transmit an article, adapt the article and make commercial use of the article. The CC-BY license permits commercial and non-
Creative Commons Attribution Non-Commercial License
The Creative Commons Attribution Non-Commercial (CC-BY-NC)License permits use, distribution and reproduction in any medium, provided the original work is properly cited and is not used for commercial purposes.(see below)

Creative Commons Attribution-Non-Commercial-NoDerivs License
The Creative Commons Attribution Non-Commercial-NoDerivs License (CC-BY-NC-ND) permits use, distribution and reproduction in any medium, provided the original work is properly cited, is not used for commercial purposes and no modifications or adaptations are made. (see below)
Use by commercial "for-profit" organizations
Use of Wiley Open Access articles for commercial, promotional, or marketing purposes requires further explicit permission from Wiley and will be subject to a fee.
Further details can be found on Wiley Online Library http://olabout.wiley.com/WileyCDA/Section/id-410895.html

Other Terms and Conditions:

v1.10 Last updated September 2015

Questions? customercare@copyright.com or +1-855-239-3415 (toll free in the US) or +1-978-646-2777.

Thermally Induced, Multicolored Hyper-Reflective Cholesteric Liquid Crystals

*Michael E. McConney, Vincent P. Tondiglia, Jennifer M. Hurtubise, Lalgudi V. Natarajan, Timothy J. White, and Timothy J. Bunning**

Cholesteric liquid crystals (CLCs) can exhibit vibrant colors due to a macroscopic helical twist of the molecular director. Selectively reflective materials with central wavelengths ranging from the UV to the infrared can be easily fabricated by mixing a chiral dopant into a nematic liquid crystal host. The central wavelength of the reflection is given by

$$\lambda_0 = \bar{n} p \tag{1}$$

where p is the pitch length of the helical twist of the director and $\bar{n}$ is the average refractive index of the mixture defined as

$$\bar{n} = (n_s + n_o)/2 \tag{2}$$

where n_s and n_o are the extraordinary and ordinary refractive indices, respectively. The maximum reflection of unpolarized light is 50% because a planar aligned cell only reflects circularly polarized light of the same handedness as the helical pitch. Methodologies to overcome this limitation include the stacking of two opposite-handed CLC films or stacking two same-handed CLC films separated by a half waveplate.[1–8] Here a methodology is reported to obtain near 100% reflectivity in a single CLC film (so called hyper-reflectivity) in which surface-bound polymer stabilization is used to spatially segregate disparate regions of opposite handedness in a single cell. Through the use of a thermally tunable CLC mixture, the high contrast condition can be induced with temperature. The versatility of the approach is further demonstrated by creating a cell with two static reflection notches at different wavelengths and thermally inducing high contrast at each notch by varying the temperature.

High-contrast, selectively reflecting materials have a variety of prospective applications in displays and photonics, allowing for on/off control of a portion of the spectrum while passing all other wavelengths. A simple method to attain high contrast reflectors has utilized chiral polymeric materials mixed with opposite-handed CLC mixtures to yield hyper-reflective CLCs (defined here as single-cell CLCs with a reflection greater than 50%). This approach yields similar optical properties as stacking multiple cells of CLCs of opposite handedness but results in devices with reduced complexity, decreased optical loss (Fresnel reflection from multiple glass surfaces), and lower cost. To date, the fabrication of CLCs with a nearly complete bandgap has only been recently reported. In both approaches, a polymer network is formed of one handedness and surrounded by a CLC mixture of the opposite handedness. In the work of Mitov et al. a right-handed polymer structure was formed in the presence of a thermally driven twist inversion compound.[9–12] Upon cooling, the bulk mixture inverts the rotation handedness of the helix and the surrounding CLC mixture becomes left-handed. Thus, regions reflecting both handedness coexist within a single cell. Similarly, Guo et al. formed a helical polymer structure, but removed the non-polymerized components before subsequently refilling with a left-handed CLC.[13–15] In both of these approaches, the region surrounding the right-handed polymer structure forms a right-handed CLC bandgap while the bulk mixture takes on its preferred left-handed form. Matching the position of these reflection notches allows for high contrast. It is important to note that the right-hand/left-hand local heterogeneity is uniform across the cell thickness. We recently reported a methodology to form these high-contrast CLCs through the use of a surface tethered polymer network (STPN), which is fabricated on only one side of the cell using photopolymerization of chiral monomer mixtures, thereby creating a cell with two separate homogenous regions through the thickness.[16] In the work presented here, dynamic high contrast CLC cells wherein the contrast can be controlled through temperature are demonstrated. Cholesteric materials that possess a smectic A*/CLC phase transition just above room temperature are known to have very sensitive temperature dependence of the selective reflection wavelength.[17–19] By choosing a mixture with an opposite handedness than the STPN, application of heat first causes the CLC phase (and a reflection notch) to appear and then to blue shift as the temperature increases. When the reflection wavelength of the right- and left-handed regions are equal, a very high contrast condition occurs.

The process for fabricating the high-contrast CLCs is shown in **Figure 1**. Planar cells were fabricated so that one of the alignment layers was doped with 1 wt% of Irgacure 369 (photoinitiator), as shown schematically in Figure 1a. These cells were correspondingly filled with a right-handed CLC mixture containing the right-handed chiral dopant (R811), achiral nematic liquid crystal (E7), and a right handed chiral monomer (RMM691). The central reflection of this mixture before polymerization is approximately 720 nm. Upon polymerization with UV light the bandwidth of the reflection broadens slightly and the notch

Dr. M. E. McConney, V. P. Tondiglia, J. M. Hurtubise, Dr. L. V. Natarajan, Dr. T. J. White, Dr. T. J. Bunning
Materials and Manufacturing Directorate
Air Force Research Laboratory
Wright-Patterson Air Force Base, OH, 45433, USA
E-mail: Timothy.Bunning@wpafb.af.mil
V. P. Tondiglia, Dr. L. V. Natarajan
Science Applications International Corporation
Dayton, OH, 45431, USA

DOI: 10.1002/adma.201003552

 © 2011 WILEY-VCH Verlag GmbH & Co. KGaA, Weinheim

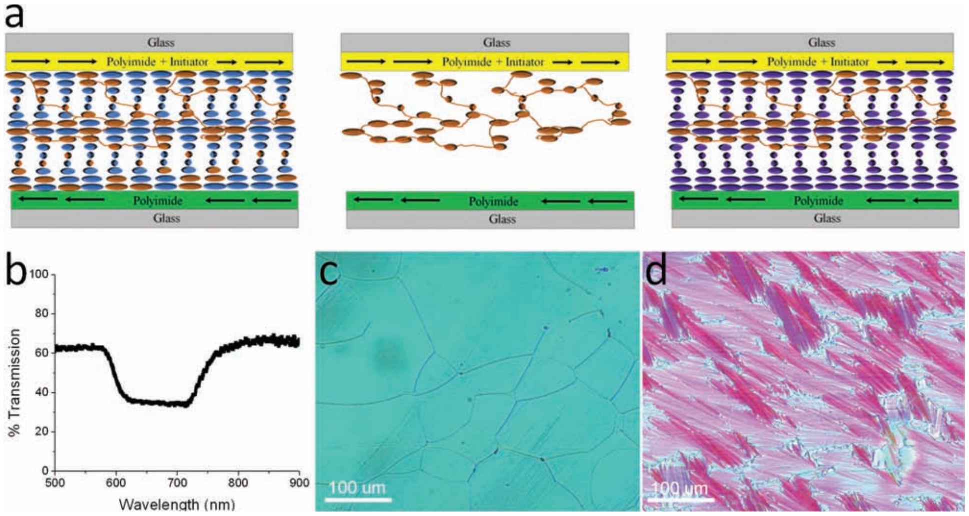

Figure 1. a) Schematic representation of the fabrication process where polymerization from one side of the cell causes growth of a chiral polymer structure from the top side of the cell only. The middle panel indicates that the polymer structure only traverses a fraction of the cell thickness and that when refilled the bottom half of the cell exhibits properties of the filling mixture. b) The unpolarized transmission spectra of the STPN cell refilled with a left-handed SmA∗ mixture at 27 °C. c) The texture of the polymer-rich side of the cell and d) the texture from the back-side of the cell. The images were taken moments apart from the same cell at the same temperature (20 °C). Upon careful observation, the underlying smectic broken fan texture can be seen in (c) and the underlying cholesteric phase can be in (d).

blue-shifts to 670 nm. There are several clear consequences to embedding the photoinitiator in the anchoring polyimide film. The polymer gel is tethered to the surface, presumably through entanglement, but the nature of the attachment is the subject of current studies. The reaction is localized to one side of the cell, which results in a polymer network that traverses only about two-thirds of the cell upon reswelling. While this approach is fairly unique, there are examples of surface-localized polymerization through photoinitiation in the presence of highly absorbing dyes.[20,21] For more information about the surface based initiation, see the Supporting Information.

After polymerization, the liquid crystal (LC) mixture and unpolymerized monomer was leeched from the cell by immersing in cyclohexane for several days followed by vacuum oven drying, leaving only a cell with a surface tethered polymer network composed of a collapsed, right-handed polymer template. This STPN cell was backfilled with a thermally tunable smectic A∗ (SmA∗) mixture that transitions to a left-handed CLC at 27 °C. The SmA∗ → CLC phase transition has been known to yield widely tunable devices, recently reported to be tunable by almost 2000 nm.[17–19] The SmA∗ mixture drawn into the cell was composed of 27% S811 in E7.[22,23]

Figure 1b shows the reflection spectra of the STPN cell at 27 °C after filling with the S811 (27%)/E7 mixture. The presence of the reflection notch (optically confirmed to be right circularly polarized) indicates that the STPN is capable of transforming the bulk smectic structure into the CLC phase. Polarized optical microscopy images from both sides of the cell confirm distinctive mesophases exist on each side of the cell. Figure 1c clearly shows distinct, characteristic oily streaks, typical of a CLC planar (reflective) texture when the samples are imaged with the STPN side on top. As shown in Figure 1d, the characteristic broken fan texture of the SmA∗ phase is observed when the sample is observed from the other side. LC phases induced exclusively through structured materials, while not common, have been reported.[24] It is well-known that polymerization in the presence of a LC solvent has a dramatic effect on the structure of the polymer, as well as the kinetics of the reaction.[25,26] Furthermore, this structured polymer can, in turn, change the properties of the surrounding LC (called polymer stabilized LCs), such as extending phase temperatures, quickening molecular relaxation times, and altering LC electrical switching properties.[27–31] One advantage of our two-pot approach is the ability to decouple the LC-templated polymer from the initial LC solvent, which enables the study of polymer stabilized systems that have large mismatches in the polymer structure and the LC packing structure, such as the case of inducing a CLC in a SmA∗.

The baseline thermal behavior of the LC mixture employed to backfill the STPN in a standard LC cell is shown in **Figure 2**. Large-scale tuning of the selective reflection notch from the

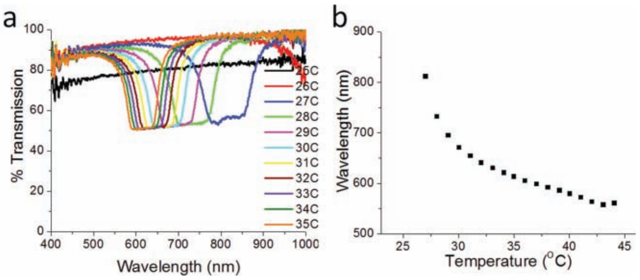

Figure 2. Thermal tuning properties of SmA∗ to CLC transition. a) Unpolarized transmission spectra of bulk mixture without polymer structure when heated from 25 °C to 35 °C. b) The corresponding notch position as a function of temperature.

© 2011 WILEY-VCH Verlag GmbH & Co. KGaA, Weinheim

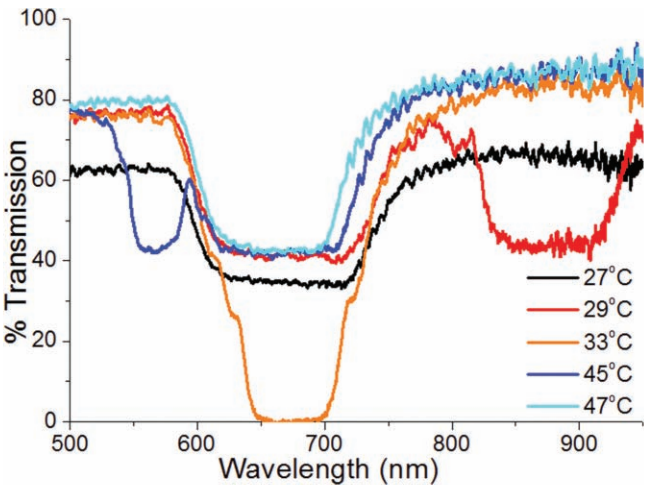

Figure 3. Unpolarized transmission spectra showing thermal tuning of a cell composed of a left-handed SmA*–CLC mixture and STPN that was templated in the presence of a right-handed CLC with a notch at 670 nm.

near-IR (>1000 nm) into the blue (≈450 nm) is observed upon heating. The high sensitivity of the thermal shift is shown in Figure 2b. Circularly polarized spectroscopy confirms that the reflection handedness of the thermally induced CLC phase is left-handed (see Supporting Information). Backfilling of the STPN cell with this mixture at room temperature, while the LC mixture is still in the smectic phase, does however yield a right circularly polarized reflection notch (again confirmed with circularly polarized spectroscopy, see Supporting Information) that matches the periodicity of the STPN scaffold.

Shown in **Figure 3** are the transmission spectra of the STPN cell filled with the S811 (27%)/E7 mixture as a function of temperature. At temperatures up to 27 °C, a single, right circularly polarized notch is evident, resulting from the right-handed STPN. Further heating to 29 °C results in the appearance of a second notch (optically confirmed to be left-handed) that appears due to the SmA* → CLC phase transition of the S811/E7 mixture. Thus, the cell possesses two reflection notches, one centered at 675 nm and the second centered at 880 nm, with the former reflecting only right circularly polarized light and the latter reflecting only left circularly polarized light. Additional heating between 31 °C and 43 °C causes the left-handed 880 nm reflection notch to blue-shift in wavelength until it overlaps with the static reflection band of the STPN. At this temperature, a high-contrast condition exists as the cell possesses reflectivity greater than 99% in a single cell architecture due to the reflection of light of both handedness centered at 675 nm (see Movie 1, Supporting Information). Continued heating subsequently reduces the contrast as the left-handed notch continues to blue shift (shown at 560 nm at 45 °C in Figure 3). Above 46 °C, the S811/E7 mixture transitions to the isotropic phase leaving the right circularly polarized reflection notch induced by the polymer scaffold to persist to more than 100 °C.

The versatility of the STPN approach is furthered demonstrated in a cell fabricated with right-handed STPNs on both sides, as schematically shown in **Figure 4**a. This cell was fabricated by preparing two STPN cells in the same manner as shown in Figure 1a, but with networks exhibiting slightly different selective reflection wavelengths. After draining, (Figure 1a, second frame) the cells were split and the two cells with attached STPNs were put back together to form a LC cell with two STPN regions (Figure 4a, regions depicted with green and blue shading) and an untemplated region in between (Figure 4a, region depicted with red shading). This dual-STPN cell was backfilled with the thermally tunable S811 (27%)/E7 mixture in a manner similar to that described above (see Experimental Section). Two reflection notches are observed at 25 °C, one at 580 nm and the other at 790 nm, and both were confirmed to be right circularly polarized bandgaps using circularly polarized spectroscopy. Upon heating, a left circularly polarized reflection notch appears due to the transition of the S811 (27%)/E7 mixture into the cholesteric phase in the region of the cell between the two STPN areas. Thus, at 28 °C three CLC reflection notches are present, as shown in Figure 4b, including a very broad and shallow left circularly polarized reflection of the S811/E7 mixture centered at 1400 nm and the two right-handed STPN notches at 580 nm and 790 nm. The left-handed reflection is broad and shallow because the notch position shifts dramatically and therefore even at a slow heating rate of

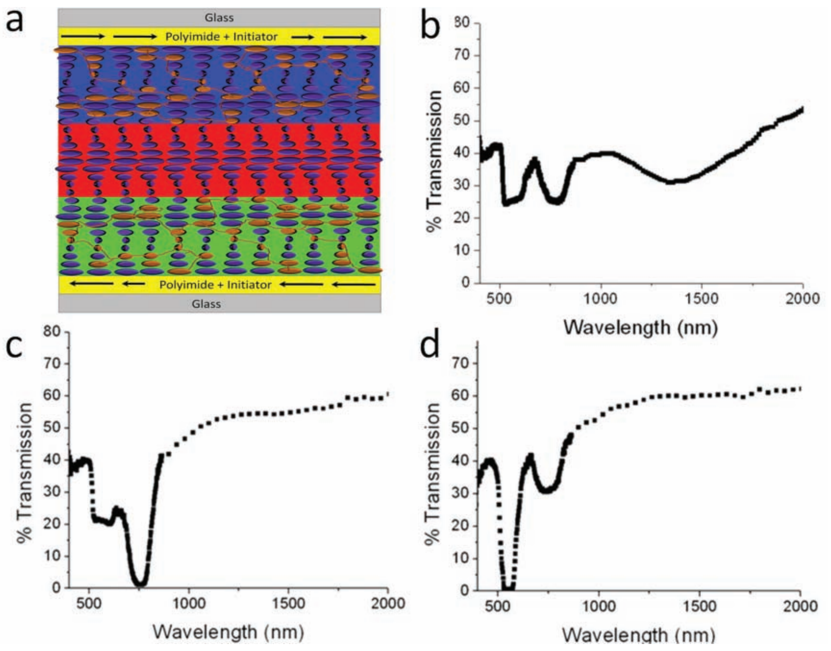

Figure 4. a) A schematic showing the structure of a cell with two STPN surfaces, yielding three possible bandgaps in a single system. The two STPN regions are depicted with green and blue shading and the region of the cell without polymer is depicted with red shading. b–d) The unpolarized transmission spectra at b) 28.0 °C, c) 28.5 °C, and d) 34.5 °C, show the thermal tuning of a left-handed mixture in the presence of two right circularly polarized static notches at 550 nm and 750 nm.

1 °C min^{-1} the mixture is still not completely at equilibrium. Furthermore, the pitch of the CLC is quite long and therefore the reflection depth is expected to be low. Further heating to 33 °C blue-shifts the left-handed notch until it overlaps with the 790 nm notch, inducing high-contrast at this wavelength as shown in Figure 4c. Continued heating to 42 °C induces high contrast at 580 nm as shown in Figure 4d (see Movie 2, Supporting Information). The scatter in the sample is caused by a lack of homogenous anchoring for the bulk LC. Nonetheless, not only do STPN cells allow for the observation of high contrast, they also enable the possibility for dynamic optical materials with three-color capability. Potentially this construct could also be used to enable tunable, multimode lasing.

In conclusion, we have presented a method to fabricate surface tethered polymer networks composed of helical polymer structures. This technique allows for the realization of high-contrast CLCs with spatially segregated reflection through the thickness of the cell (*z*-axis) rather than throughout the cell. Similar to earlier works, the STPN acts as a structurally chiral scaffold that forces the swollen LC mixture to take on the same handedness of the polymeric material and reflects light according to the as-fabricated pitch. Backfilling a STPN cell with a thermally tunable SmA* mixture allows for temperature-induced, nearly complete reflection. Additionally, cells made with STPN on both substrates show the capability for simultaneous three-color reflection and multicolor thermally induced hyper-reflectivity.

Experimental Section

Cell Preparation: A mixture of polyimide solution (8 mL of PI2555 polyimide (HD Microsystems), 32.5 mL of *N*-methyl pyrolidone and 9.1 mL of 1-methoxy-2-propanol) was filtered through a 0.45-μm filter (Pall, Acrodisc PSF syringe filter) onto a glass substrate. The mixture was spin-coated (APH, spin 150) by ramping up to 1500 rpm for 15 s followed by spinning at 3000 rpm for 1 min. The coated glass was placed on the edge of a hot plate (Torrey Pines Scientific, HS30) set to 200 °C (the edge temperature, $T = 50$ °C, measured with surface probe thermometer) for 30 min. An initiator-doped polyimide solution was prepared by mixing in 1% by weight Irgacure 369 (Ciba) to the previously prepared polyimide mixture. A glass substrate was coated with this initiator-doped polyimide solution under identical spin-coating conditions. This coated glass was also baked in the same manner. Then the films were rubbed to achieve a planar oriented anchoring layer. Then the glass pieces were glued together with 30-μm spacers to form an empty cell.

Polymeric Scaffold Preparation: All cells, except the dual STPN cells, were filled with a mixture containing 19.5% chiral monomer RMM691 (Merck), 8.5% chiral dopant R811(Merck), and 3.5% achiral diacrylate RMM257 (Merck), and 68.5% achiral nematic E7(Merck). The cells were exposed to 1.7 mW cm^{-2} UV light (Exfo Omnicure S1000, 300–500 nm) for 30 min, thereby polymerizing the monomer. The side with photoinitiator was facing the UV light. The cells were then immersed in cyclohexane for at least 7 days, until the cell was essentially transparent; the faint color was homogenous and did not change with time. Then the cells were dried under the application of a light vacuum. If a dried cell was broken open, it was apparent by eye that the polymer scaffold was only on the side with the initiator by faint the blue reflection. Dried single-sided STPN cells cells were capillary filled the SmA* mixture. To create the two-sided STPN cells, two 15-μm cells were filled with their monomer mixtures. One cell was filled with a mixture containing 19.8% RMM691, 19.8%R811, 3.7% RMM257, and 56.7% E7. The other cell was filled with 19.2% RMM691, 2.6% R811, 2.6% RMM257, and 75.6% E7. The cells were polymerized, the LC mixture was leeched, and the cells were dried as before. The dried cells were split open and the SmA* mixture was applied to the polymer surface and allowed to swell. After swelling, the excess SmA* mixture was thoroughly removed by gently blowing the mixture off. Then these two glass substrates with STPN were glued together with 30-μm spacers and the cell was subsequently further capillary filled with more of the SmA* mixture to fill the empty remaining gap.

Optical Measurements: Cells were attached to a thermoelectric cooler (TEC) with a thermal grease. The TEC had a hole in it that facilitated the transmission measurements. The TEC temperature was measured with a surface mounted thermistor and temperature was controlled with a 2510 TEC controller (Keithley). Transmission spectra were taken with an USB2000+ vis-NIR spectrometer (Ocean Optics). Measurements were made by passing unpolarized light through the sample and then passing it through a Fresnel rhomb/polarizer. The Fresnel rhomb/polarizer combination broke the light into the left circularly polarized component and the right circularly polarized component. A split fiber was used to send both the right-CPL and left-CPL to the spectrometer. Then the polarized measurements were made by blocking either the right-handed or left-handed channel. The unpolarized measurements were performed both by combining the left-CPL and right-CPL channels of the Fresnel rhomb with the split fiber and also through by-passing the Fresnel rhomb/polarizer to the spectrometer.

Supporting Information

Supporting Information is available from the Wiley Online Library or from the author.

Acknowledgements

The authors wish to thank Dr. Christopher Bailey, Dr. Kyungmin Lee, and Dr. Michael Birnkrant for useful discussions. Funding from the Air Force Office of Scientific Research and the Materials and Manufacturing Directorate of the Air Force Research Laboratory is gratefully acknowledged. MEM gratefully acknowledges the National Research Council for fellowship support.

Received: September 28, 2010
Revised: December 13, 2010
Published online: February 2, 2011

[1] D. M. Makow, *Appl. Opt.* **1980**, *19*, 1274.
[2] D. M. Makow, C. L. Sanders, *Nature* **1978**, *276*, 48.
[3] S. Caveney, *Proc. R. Soc. London, Ser. B* **1971**, *178*, 205.
[4] H. P. Chen, D. Katsis, J. C. Mastrangelo, S. H. Chen, S. D. Jacobs, P. J. Hood, *Adv. Mater.* **2000**, *12*, 1283.
[5] M. H. Song, B. Park, K. C. Shin, T. Ohta, Y. Tsunoda, H. Hoshi, Y. Takanishi, K. Ishikawa, J. Watanabe, S. Nishimura, T. Toyooka, Z. Zhu, T. M. Swager, H. Takezoe, *Adv. Mater.* **2004**, *16*, 779.
[6] M. H. Song, N. Y. Ha, K. Amemiya, B. Park, Y. Takanishi, K. Ishikawa, J. W. Wu, S. Nishimura, T. Toyooka, H. Takezoe, *Adv. Mater.* **2006**, *18*, 193.
[7] M. H. Song, K.-C. Shin, B. Park, Y. Takanishi, K. Ishikawa, J. Watanabe, S. Nishimura, T. Toyooka, Z. Zhu, T. M. Swager, H. Takezoe, *Sci. Technol. Adv. Mat.* **2004**, *5*, 437.
[8] N. Y. Ha, S. M. Jeong, S. Nishimura, H. Takezoe, *Appl. Phys. Lett.* **2010**, *96*, 153301
[9] M. Mitov, N. Dessaud, *Nat. Mater.* **2006**, *5*, 361.
[10] M. Mitov, N. Dessaud, *Liq. Cryst.* **2007**, *34*, 183.
[11] A. C. Tasolamprou, M. Mitov, D. C. Zografopoulos, E. E. Kriezis, *Opt. Commun.* **2009**, *282*, 903.

© 2011 WILEY-VCH Verlag GmbH & Co. KGaA, Weinheim

[12] S. Relaix, M. Mitov, *Liq. Cryst.* **2008**, *35*, 1037.
[13] J. Guo, H. Cao, J. Wei, D. Zhang, F. Liu, G. Pan, D. Zhao, W. He, H. Yang, *Appl. Phys. Lett.* **2008**, *93*, 201901.
[14] J. Guo, H. Yang, R. Li, N. Ji, X. Dong, H. Wu, J. Wei, *J. Phys. Chem. C* **2009**, *113*, 16538.
[15] J. Guo, F. Liu, F. Chen, J. Wei, H. Yang, *Liq. Cryst.* **2010**, *37*, 171.
[16] M. E. McConney, V. P. Tondiglia, J. M. Hurtubise, T. J. White, T. J. Bunning, *Chem. Commun.* **2011**, *47*, 505.
[17] F. Ania, H. Stegemeyer, *Mol. Cryst. Liq. Cryst.* **1985**, *2*, 67.
[18] L. V. Natarajan, J. M. Wofford, V. P. Tondiglia, R. L. Sutherland, H. Koerner, R. A. Vaia, T. J. Bunning, *J. Appl. Phys.* **2008**, *103*, 093107.
[19] T. J. White, M. E. McConney, T. J. Bunning, *J. Mater. Chem.* **2010**, *20*, 9832.
[20] S. Relaix, C. Bourgerette, M. Mitov, *Appl. Phys. Lett.* **2006**, *89*, 251907.
[21] S. Relaix, C. Bourgerette, M. Mitov, *Liq. Cryst.* **2007**, *34*, 1009.
[22] W.-R. Chen, J.-C. Hwang, *Mol. Cryst. Liq. Cryst.* **2004**, *423*, 85.
[23] S.-Y. T. Tzeng, C.-N. Chen, Y. Tzeng, *Liq. Cryst.* **2010**, *37*, 1221.
[24] K. Robbie, D. J. Broer, M. J. Brett, *Nature* **1999**, *399*, 764.
[25] J. D. Clapper, L. Sievens-Figueroa, C. A. Guymon, *Chem. Mater.* **2007**, *20*, 768.
[26] C. A. Guymon, E. N. Hoggan, N. A. Clark, T. P. Rieker, D. M. Walba, C. N. Bowman, *Science* **1997**, *275*, 57.
[27] P. Archer, I. Dierking, *Soft Matter* **2009**, *5*, 835.
[28] H. Kikuchi, M. Yokota, Y. Hisakado, H. Yang, T. Kajiyama, *Nat. Mater.* **2002**, *1*, 64.
[29] T. J. White, R. L. Bricker, L. V. Natarajan, V. P. Tondiglia, L. Green, Q. Li, T. J. Bunning, *Opt. Express* **2010**, *18*, 173.
[30] I. Dierking, *Adv. Mater.* **2000**, *12*, 167.
[31] I. Dierking, *Adv. Funct. Mater.* **2004**, *14*, 883.

© 2011 WILEY-VCH Verlag GmbH & Co. KGaA, Weinheim
