# Supplementary material for: Advanced liquid crystal-based switchable optical devices for light protection applications: principles and strategies
Source: Light Sci Appl. 2023 Jan 3;12:11. doi: 10.1038/s41377-022-01032-y (PMC9807646; doi:10.1038/s41377-022-01032-y)
Supplement: Supplementary file 7 — Fig 7 copyright promotion [file 41377_2022_1032_MOESM7_ESM.pdf]

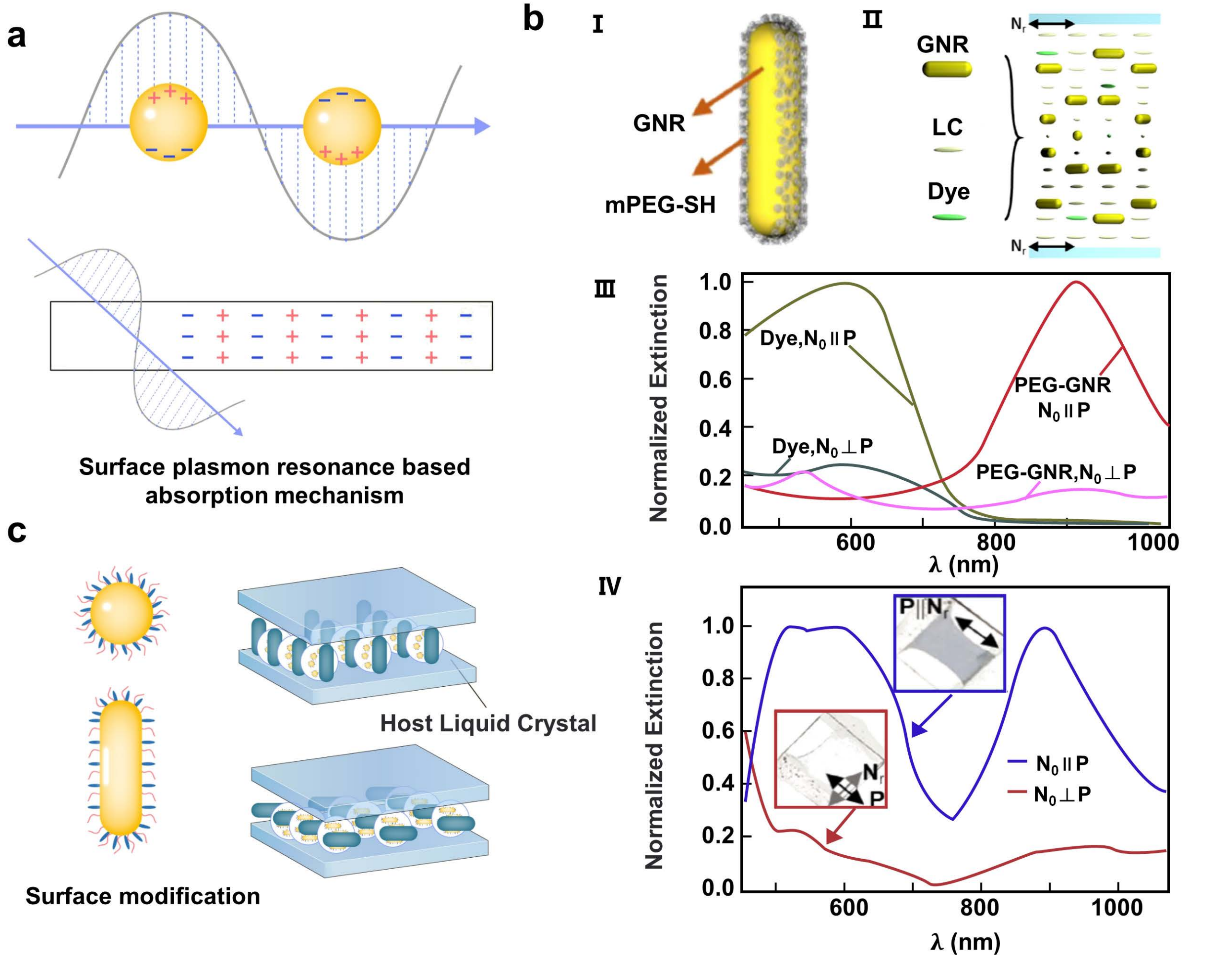
a
Surface plasmon resonance based absorption mechanism
b
I
GNR
mPEG-SH
II
GNR
LC
Dye
N_r
III
Normalized Extinction
Dye,N_0 ∥ P
Dye,N_0 ⊥ P
PEG-GNR N_0 ∥ P
PEG-GNR,N_0 ⊥ P
λ (nm)
IV
Normalized Extinction
P||N_r
N_r
P
N_0 ∥ P
N_0 ⊥ P
λ (nm)
c
Host Liquid Crystal
Surface modification

主　题:	RE: Reprint Permissions
发件人:	"Optica Publishing Group Copyright" <copyright@osa.org> 2022-10-25 22:35:18
收件人:	"张锐聪" <ruicong_zhang@stu.hit.edu.cn>, "Optica Publishing Group Copyright" <copyright@osa.org>

Dear Dr. Zhang,

Thank you for contacting Optica Publishing Group.

For the use of material from Chun-Wei Chen, Alyssa N. Brigeman, Tsung-Jui Ho, and Iam Choon Khoo, "Normally transparent smart window based on electrically induced instability in dielectrically negative cholesteric liquid crystal," Opt. Mater. Express 8, 691-697 (2018):

There are only 5 figures in this article. Please let us know if you would like to reproduce one of these figures.

For the use of figure 2 from Ghadah H. Sheetah, Qingkun Liu, Bohdan Senyuk, Blaise Fleury, and Ivan. I. Smalyukh, "Electric switching of visible and infrared transmission using liquid crystals co-doped with plasmonic gold nanorods and dichroic dyes," Opt. Express 26, 22264-22272 (2018), figure 1 from Bing-Xiang Li, Rui-Lin Xiao, Sathyanarayana Paladugu, Sergij V. Shiyanovskii, and Oleg D. Lavrentovich, "Dye-doped dual-frequency nematic cells as fast-switching polarization-independent shutters," Opt. Express 27, 3861-3866 (2019) and figure 5 from P. Lakshmi Madhuri, Saranya Bhupathi, S. Shuddhodana, Zaher M. A. Judeh, Sheng-Hsiung Yang, Yi Long, and Ibrahim Abdulhalim, "Hybrid vanadium dioxide-liquid crystal tunable non-reciprocal scattering metamaterial smart window for visible and infrared radiation control," Opt. Mater. Express 11, 3023-3037 (2021):

Optica Publishing Group considers your requested use of its copyrighted material to be Fair Use under United States Copyright Law. We request that a complete citation of the original material be included in any publication.

As this article is published under the terms of the Optica Publishing Group Open Access Publishing Agreement, when adapting or otherwise creating a derivative version of the article, users must maintain attribution to the author(s) and the published article's title, journal citation, and DOI. Users should also indicate if changes were made and avoid any implication that the author or Optica Publishing Group endorses the use.

While your publisher should be able to provide additional guidance, we prefer the below citation formats:

For citations in figure captions:

[Reprinted/Adapted] with permission from [ref #] © The Optical Society. (Please include the full citation in your reference list)

For images without captions:

Journal Vol. #, first page (year published) An example: Opt. Express 27, 1164 (2019)

Please let me know if you have any questions.

Kind Regards,

Hannah Greenwood

Hannah Greenwood

October 25, 2022

Authorized Agent, Optica Publishing Group

OPTICA PUBLISHING GROUP | Formerly OSA

From: 张锐聪 <ruicong_zhang@stu.hit.edu.cn>
Sent: Wednesday, October 19, 2022 8:18 AM
To: Optica Publishing Group Copyright <copyright@osa.org>
Subject: Reprint Permissions

ear Editor,

Hello! We would like to apply for permission to reproduce images from papers published by your publisher, as detailed below.

Title : Electric switching of visible and infrared transmission using liquid crystals co-doped with plasmonic gold nanorods and dichroic dyes
Author : Ghadah H. Sheetah, Qingkun Liu, Bohdan Senyuk, Blaise Fleury, and Ivan. I. Smalyukh
Volume, issue, and page numbers: 26, 22264-22272 (2018)
Journal : Optics Express
Expected content to be reproduced : Fig.2(a),   Fig.2(e) and Fig.2(f)

Special request: We hope to allow us to rearrange these pictures, as shown below.
Original picture:

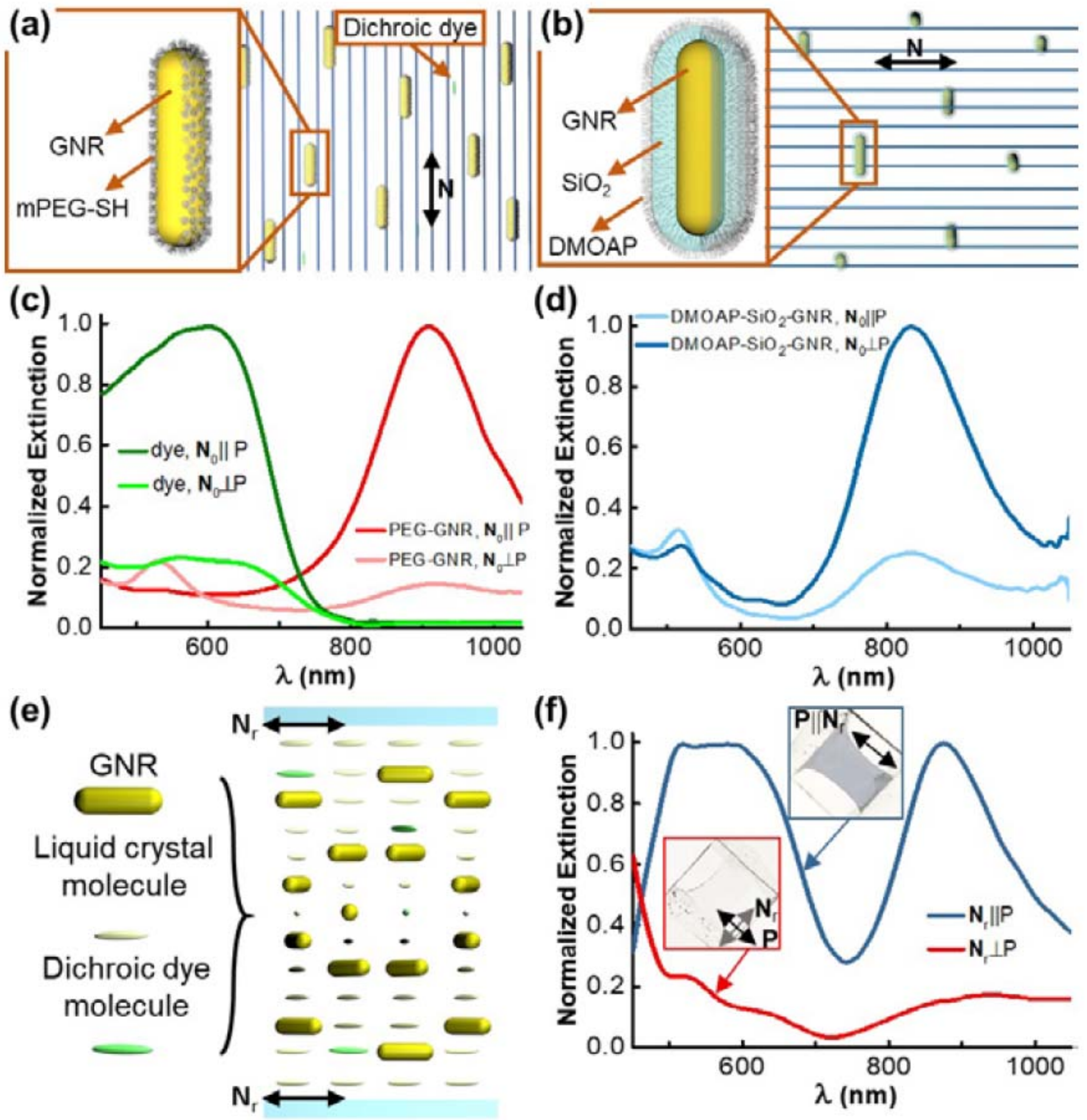

Fig. 2. Unidirectionally aligned and twisted structures of LC doped with GNRs and dichroic dye molecules. (a) Schematic of GNRs capped with mPEG-SH following **N** (blue lines) in a uniformly aligned LC; the inset shows details of surface functionalization of GNRs. (b) Schematic diagram of DMOAP-SiO_2-GNRs that exhibit self-alignment perpendicular to **N**; the inset shows details of silica shells around GNRs and DMOAP surface functionalization. (c,d)

Our contributions:

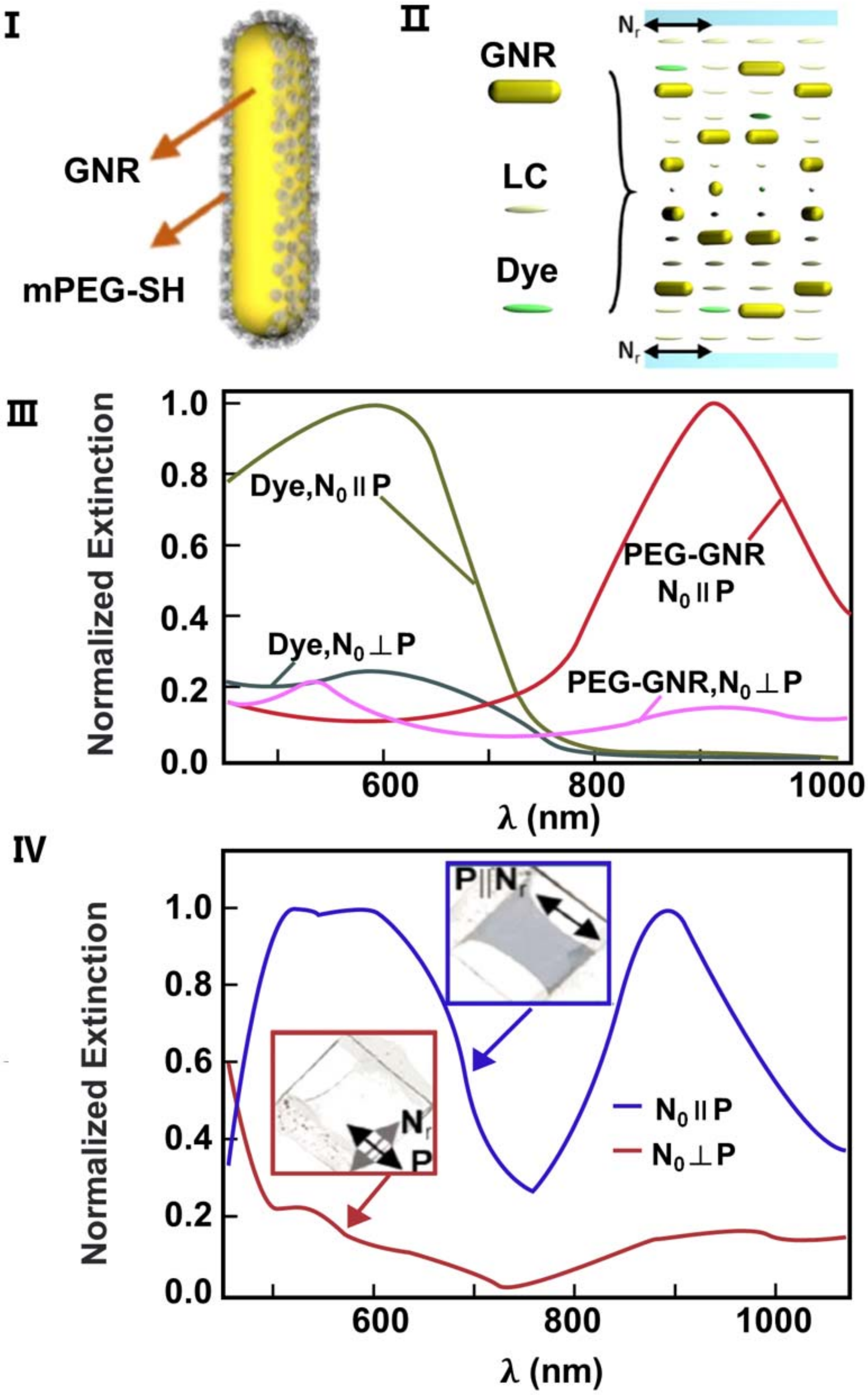

Information on the papers we plan to publish is as follows.
Title : Advanced liquid crystal-based switchable optical devices for light protection applications: principles and strategies
Publisher : Springer Nature
Journal : Light: Science & Applications
Estimated publication time : In November 2022

Main author of the article : Ruicong Zhang, Jiajun li, Zhibo Zhang, Jiecai Han, Lei Yang, Tianyu Wang, Jiaqi Zhu

We admire your society's contribution to the Institute of Optics and hope to obtain permission to reprint images.

With thanks!

Ruicong Zhang

Harbin Institute of Technology

Research Article | Vol. 26, No. 17 | 20 Aug 2018 | OPTICS EXPRESS 22264
Optics EXPRESS

Electric switching of visible and infrared transmission using liquid crystals co-doped with plasmonic gold nanorods and dichroic dyes

GHADAH H. SHEETAH,[1,2] QINGKUN LIU,[1,2] BOHDAN SENYUK,[2] BLAISE FLEURY,[2] AND IVAN. I. SMALYUKH[1,2,3,4,*]

[1]*Materials Science and Engineering Program, University of Colorado, Boulder, Colorado 80309, USA*
[2]*Department of Physics and Soft Materials Research Center, University of Colorado, Boulder, Colorado 80309, USA*
[3]*Department of Electrical, Computer and Energy Engineering and Soft Materials Research Center, University of Colorado, Boulder, Colorado 80309, USA*
[4]*Renewable and Sustainable Energy Institute, National Renewable Energy Laboratory and University of Colorado, Boulder, Colorado 80309, USA*
**ivan.smalyukh@colorado.edu*

Abstract: Smart windows and many other applications require synchronous or alternating facile electric switching of transmitted light intensity in visible and near infrared spectral ranges, but most electrochromic devices suffer from slow, nonuniform switching, high power consumption and limited options for designing spectral characteristics. Here we develop a guest-host mesostructured composite with rod-like dye molecules and plasmonic nanorods spontaneously aligned either parallel or orthogonally to the director of the liquid crystal host. This composite material enables fast, low-voltage electric switching of electromagnetic radiation in visible and infrared ranges, which can be customized depending on the needs of applications, like climate-dependent optimal solar gain control in smart windows.

© 2018 Optical Society of America under the terms of the OSA Open Access Publishing Agreement

1. Introduction

Liquid crystal (LC) devices, such as optical modulators, displays and shutters, often utilize dichroic dye guest molecules to control visible light intensity [1–4]. Typically, dichroic dyes are rod-like molecules with dimensions comparable to the LC molecules. Thus, they tend to align with the LC director **N** (the average orientation of rod-like LC-molecules [5]) and follow **N** as it rotates in response to low-voltage electric fields. Since the absorption of dye molecules depends on their orientation with respect to the light propagation direction and the polarization direction, if this light is linearly polarized [1], switching the LC director allows for tuning the relative fractions of light absorbed by the dye molecules and transmitted through the LC cell. Plasmonic analogs of these guest-host LCs, introduced recently [6–9], extend this principle to rod-like plasmonic nanoparticles with typical dimensions in the range of tens of nanometers. Dichroic dyes [1–4] and plasmonic nanoparticles, such as gold nanorods (GNRs) [6–9], have their advantages and disadvantages when used with LCs and were previously utilized only separately in recent studies or applications. For example, a large variety of inexpensive commercially available dyes and their (often high) solubility concentration limits make them ideal for designing switchable light absorption in the visible spectral range, but not in the infrared [1]. In contrast, plasmonic GNRs with longitudinal surface plasmon resonance (SPR) peaks in the infrared range allow for tuning peak absorption within the near infrared but rarely in the short-wavelength part of the visible spectrum [6–9]. Co-dispersions of plasmonic GNRs were developed recently [10] to demonstrate that short-range or broadband spectral characteristics can be obtained in the switchable plasmonic guest-

#336206 https://doi.org/10.1364/OE.26.022264
Journal © 2018 Received 27 Jun 2018; revised 2 Aug 2018; accepted 2 Aug 2018; published 13 Aug 2018

host composites by mixing nanorods with different aspect ratios and surface treatments within the LC dispersions, though this again was limited in terms of the accessible spectral range.

In this work, we develop a mesostructured LC composite with both molecular dichroic dyes and plasmonic nanoparticles homogeneously distributed within the host medium. We demonstrate that long axes of dye molecules and GNRs can be spontaneously aligned parallel to **N**, as well as that the rod-like nanoparticles can be also oriented orthogonally to **N** by defining perpendicular boundary conditions (BCs) on their surfaces. This allows tuning the transmitted light intensity in both the visible and the near infrared spectral ranges, either in-phase or out-of-phase. Using different sample geometries, nematic and cholesteric LCs with negative or positive dielectric anisotropy, we show that the LC composite's switching times can be varied from millisecond to seconds and transmission of both unpolarized and polarized light can be controlled depending on the needs of applications such as smart windows, optical shutters, modulators, etc.

2. Method and design

GNRs were synthesized following a seed mediated method while using surfactants and pH control to tune their aspect ratio [11,12]. To obtain the longitudinal localized SPR peaks at 780 nm, 815 nm and 1010 nm, the synthesized GNRs were designed to have mean lengths and diameters of 77 × 21 nm, 93 × 23 nm, and 104 × 20 nm, respectively, as determined by transmission electron microscopy (TEM) (Fig. 1(a)-(c)). GNRs with the longitudinal SPR peak at 815 nm were surface-functionalized with thiol-terminated methoxy-poly(ethylene glycol) (mPEG-SH 5kDa, JemKem Technology) [10]. For some GNRs, porous silica shells were grown around the nanoparticles (Fig. 1(a),(b)), followed by further surface-functionalization with dimethyloctadecyl[3-(trimethoxysilyl) propyl] ammonium chloride (DMOAP, ACROS Organics) according to procedures described elsewhere [8–10]. The thickness of the silica shell was controlled to maintain the anisotropic shape of the ensuing nanostructures, as needed for orienting these particles in LCs. The extinction spectra of both silica and PEG capped GNRs in isotropic solvents are shown in Fig. 1(d),(e). To disperse different types of synthesized GNRs in various LCs, we used nematic 4-cyano- 4′-pentylbiphenyl (5CB, Chengzhi Yonghua Display Materials Co., Ltd.) as an intermediate nematic solvent. First, 60 μL of GNRs dispersion in methanol ~0.91 nmol/L was kept in a 0.5 mL centrifuge tube at 90°C for an hour to fully evaporate the solvent. 15 μL of 5CB was then added and kept under sonication for 5 min at 40°C while in the isotropic phase and then vigorously stirred until it cooled down to the nematic phase. The sample was centrifuged at 2000 rpm for 3 min to remove occasional aggregates caused by the nucleation of nematic domains during the phase transition [13], yielding a stable dispersion 0.68 nmol/L of GNRs within 5CB. To obtain dispersions with negative dielectric anisotropy $\Delta\varepsilon$, these 5CB dispersions of GNRs were mixed with a nematic mixture AMLC-0010 (from AlphaMicron Inc.) with $\Delta\varepsilon = -3.7$ and containing dichroic dyes with strong absorption within the visible spectrum. The mixing of 5CB and AMLC-0010 by a ratio of 1:10 (vol. %) yielded a mixture with an estimated $\Delta\varepsilon\approx-2$. The cholesteric pitch p of LC mixtures was maintained within p = 10-60 μm using a left-handed chiral dopant ZLI-811 (from Merck) and measured with the Grandjean-Cano wedge cell method [14]. Alternatively, the dispersion of DMOAP capped silica on GNRs (DMOAP-SiO_2-GNRs) in 5CB was mixed with the AMLC-0010 at ratio 1:1 (vol. %) yielding a nematic mixture with $\Delta\varepsilon\approx4.6$.

We used different configurations of cells with homeotropic (perpendicular) and planar BCs for the LC far-field director $\mathbf{N}_0$. In the former case, polyimide SE1211 (Nissan Chemical Industries, Ltd.) was spin-coated onto the glass plates with transparent indium tin oxide (ITO) electrodes at 3000 rpm for 60 s and baked at 180°C for 60 min. In the latter case, a thin layer of 1 wt% aqueous polyvinyl alcohol (PVA, Sigma-Aldrich) was spin-coated and kept in an oven at 100°C for 1h and then unidirectionally rubbed. The cells were assembled using 30 μm silica spacers and glued at their edges with UV-curable NOA-63 glue (Norland Products,

Optics EXPRESS

Inc.); the cell gap thickness was confirmed by using an optical interference method. We used an Olympus BX-51 polarizing optical microscope (POM) equipped with 10 × , 20 × , and 50 × objectives with numerical aperture 0.3–0.9 along with a CCD camera (GS3-U3-28S5C, from Point Grey Research). The light source was a tungsten-halogen lamp. Extinction and transmittance spectra were recorded using a microscope-mounted spectrometer USB2000-FLG (Ocean Optics) with a broadband (400-1200 nm) polarizer inserted into the optical path after the sample. Electric switching properties, including the threshold voltage and response time, were measured utilizing a photodiode and data acquisition card SCC-68 (National Instruments Co.) controlled by a homemade software [6] written in LabVIEW and a Si amplified photodetector PDA100A (Thorlabs Inc.). The response times, including both the rise time upon applying the voltage and the decay time after removing the voltage, were measured as in our previous studies and are described elsewhere [6–10]. Wavelength-selective studies of switching of dye and GNR orientations utilized optical interference filters such as 700 nm long pass filter (Semrock Inc.) and an infrared filter (Olympus). TEM images were obtained using a FEI Tecnai T12 Spirit.

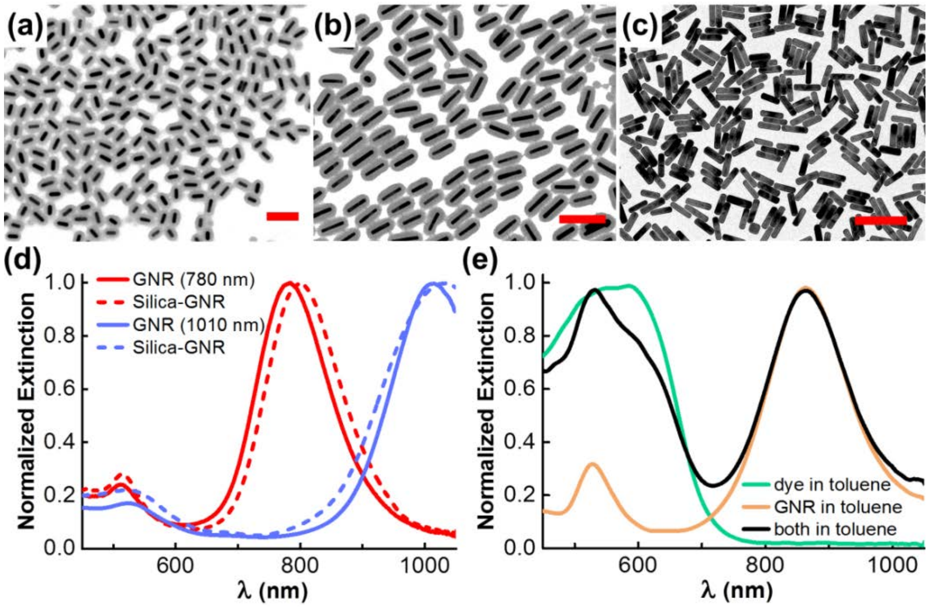

Fig. 1. TEM imaging of GNRs and extinction spectra of dye molecules and nanoparticles in isotropic solvents. (a-c) TEM images of GNRs with different longitudinal SPR peaks: (a) 780 nm, (b) 1010 nm and (c) 815 nm. GNRs shown in (a) and (b) are coated with a silica shell of average thickness of 21 nm and 25 nm, respectively. Scale bars are 200 nm. (d) Normalized by the maximum intensity extinction spectra of GNRs in water before (red and blue solid lines) and after silica capping (dashed corresponding lines). (e) Normalized by the maximum intensity extinction spectra of dye molecules and PEG-capped GNRs in toluene taken separately (green and orange lines) and when dispersed jointly (black line). Note that the extinction peaks of dye and the longitudinal SPR of GNRs are red-shifted due to the high refractive index of toluene.

3. Experiment and Characterization

GNRs treated with PEG promoted tangential BCs on their surfaces, prompting self-alignment of GNRs within the aligned LC parallel to **N** (Fig. 2(a)), while DMOAP-SiO_2-GNRs self-aligned perpendicularly to **N** because of perpendicular BCs (Fig. 2(b)). This self-alignment (Fig. 2(a),(b)) is driven by the minimization of the surface anchoring energy [6,7]. The corresponding polarization-dependent extinction spectra of PEG-GNR and DMOAP-SiO_2-GNRs in uniformly aligned 5CB samples are shown in Fig. 2(c),(d). The rod-like dichroic

dyes follow the LC molecules and **N** because of the induced dipole-dipole (anisotropic van der Waals) interactions [1], similar to that between LC molecules themselves [5,14]. Although the mechanisms of alignment of nanometer-sized rod-like dichroic dye molecules and much larger (20 to 100 nm) nanorods are rather different, the orientational self-ordering of both leads to anisotropic, polarization-dependent optical characteristics (Fig. 2(c),(d)). Orientational self-ordering of different types of GNRs and dye molecules can be quantified by determining the scalar order parameter commonly defined as $S = \langle 3\cos^2\theta\text{-}1\rangle/2$, where the angled brackets denote sample average values and θ is the angle between the long axis of rod-like dye molecules or nanoparticles and **N** [14]. By using samples with the uniformly aligned $\mathbf{N}_0$ defined by the rubbing direction $\mathbf{N}_r$ in planar LC cells, the order parameter $S = (A_{\|}\text{-}A_{\perp})/(A_{\|} + 2A_{\perp})$ of both the dichroic dye and GNRs can be experimentally determined based on the peak absorbance values $A_{\|}$ and $A_{\perp}$ at the linear polarization orientation of the probing light $P\|N_0$ and $P\perp N_0$, respectively [1,14]. The PEG-GNRs and DMOAP-SiO_2-GNRs (Figs. 2(c) and 2(d)) exhibit order parameter values of 0.66 and −0.41, respectively, consistent with their average alignment parallel and perpendicular to $\mathbf{N}_0$. In a similar way (Fig. 2(c)), we determine an order parameter of ≈0.61 for the dye.

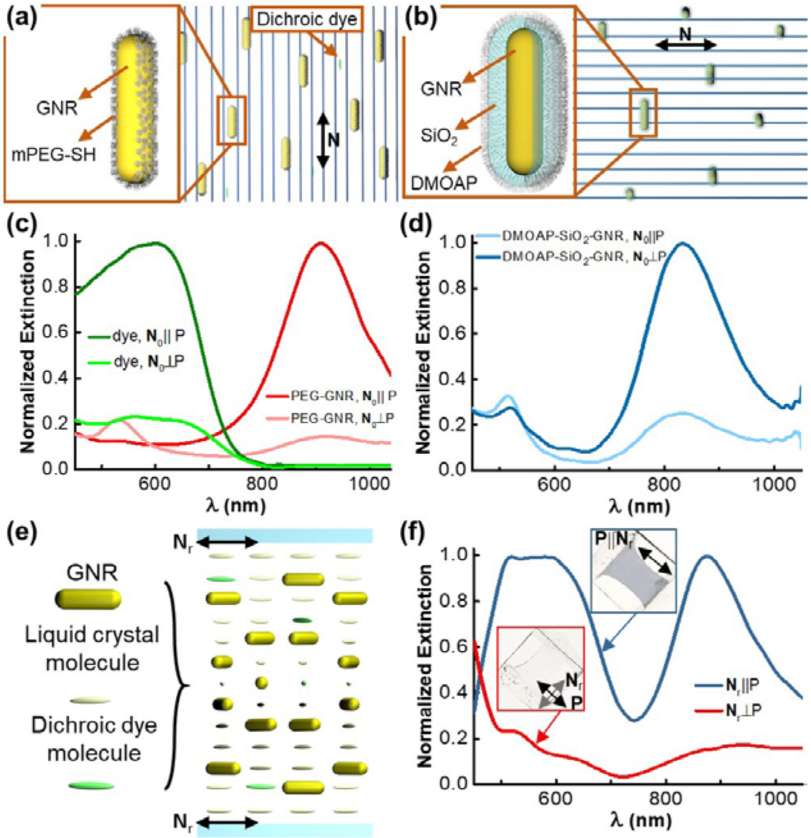

Fig. 2. Unidirectionally aligned and twisted structures of LC doped with GNRs and dichroic dye molecules. (a) Schematic of GNRs capped with mPEG-SH following **N** (blue lines) in a uniformly aligned LC; the inset shows details of surface functionalization of GNRs. (b) Schematic diagram of DMOAP-SiO_2-GNRs that exhibit self-alignment perpendicular to **N**; the inset shows details of silica shells around GNRs and DMOAP surface functionalization. (c,d) Normalized by the maximum intensity extinction spectra of LC with (c) PEG-functionalized and (d) DMOAP-SiO_2-GNRs in a planar cell for linear polarizations of probing light $(\mathbf{P})\|(\mathbf{N})_0$ and $\mathbf{P}\perp\mathbf{N}_0$. (e) Schematic diagram of the cholesteric LC co-doped with dichroic dye and PEG-functionalized GNRs in a planar cell with the alignment of **N** at cell substrates defined by the

rubbing direction $\mathbf{N}_r$. (f) Normalized spectra of cholesteric LC with $\Delta\varepsilon<0$ (1:10 mixture of 5CB and AMLC-0010) co-doped with the dichroic dye and PEG-functionalized GNRs in a planar cell shown in (e); the spectra are obtained for $\mathbf{P}||\mathbf{N}_r$ and $\mathbf{P}\perp\mathbf{N}_r$.

Dye molecules and GNRs align with respect to the local director also in samples with spatially varying **N**, such as the uniformly π-twisted configuration shown in (Fig. 2(e)). In this sample, the dichroic dye molecules and PEG-functionalized GNRs follow the helicoidal **N** in the LC with $\Delta\varepsilon<0$ and p = 60 μm in a planar cell of thickness $d\approx p/2\approx 30$ μm. Following **N**, dye molecules and GNRs twist by π around the helical axis perpendicular to cell substrates. This structure of spatial orientations of dye molecules and nanoparticles is inferred from the extinction spectra of the composite LC (Fig. 2(f)).

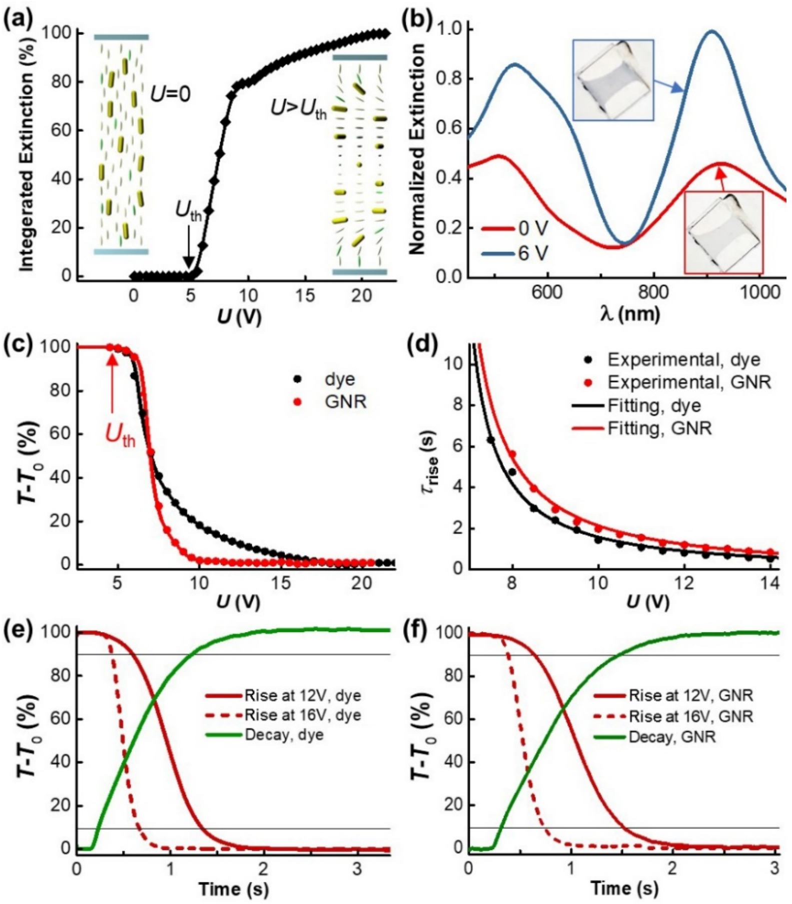

Fig. 3. Electric switching of a cholesteric LC co-doped with GNRs and dichroic dyes in a homeotropic cell. (a) Integrated extinction of light traversing through a homeotropic cholesteric LC cell versus U. The threshold voltage U_{th} is marked by an arrow; cell thickness is $d\approx p/2$ with $p\approx 60$ μm. Schematics in the insets show patterns of orientation of dichroic dye and LC molecules and GNRs within the LC at (left) $U<U_{th}$ and (right) $U>U_{th}$. (b) Normalized by the maximum intensity extinction spectra of the homeotropic cell for linear polarizations with and without field, with insets of photographs of inch-size cells. (c) Voltage dependence of the transmittance of natural white light across the homeotropic cell measured separately utilizing optical filters for both PEG-GNR and the dichroic dye. (d) Voltage-dependent rise times for both dye and GNR in the same sample. (e, f) Rise and decay times for dye and PEG-GNR, respectively, measured based on changes of a relative transmittance (T-T_0), where T_0 is the minimum transmittance of the composite, for the same LC system by using corresponding optical filters.

Due to a large pitch, linear polarizations of visible and near-infrared electromagnetic radiation traversing such LC cell closely follow the rotation of **N** as the Mauguin parameter $\Delta np/(2\lambda)>>1$ [14], where the optical anisotropy of used mixtures was within $\Delta n\approx 0.05\text{-}0.2$ and λ is the wavelength of the incident light. This results in a strong dependence of the extinction spectra on the polarization of the probing light **P** (Fig. 2(f)) with a maximum extinction due to the dye and the longitudinal SPR of PEG-functionalized GNRs at $\mathbf{P}||\mathbf{N}_r$. Under the Mauguin regime of light propagation, the strong polarization dependence of the spectra shown in (Fig. 2(f)) confirms that the dye molecules and the rod-like particles mimic the spatial LC director configurations, as depicted in Fig. 2(e).

A cholesteric LC composite with $\Delta\varepsilon<0$, the same as shown in Fig. 2(f), was also introduced into a homeotropic cell with $d\approx p/2\approx 30$ μm. Despite the cholesteric LC's tendency to twist, strong perpendicular BCs at the substrates of this cell cause unwinding of **N** (a left-side inset in Fig. 3(a)). Application of 1 kHz AC voltage to the transparent ITO electrodes on the confining substrates forces LC molecules to rotate to be orthogonal to the electric field and tangential to the cell substrates. This realignment is due to the dielectric coupling between **N** and electric field and leads to the so-called translationally invariant twisted configuration [15] (a right-side inset in Fig. 3(b)) with the threshold voltage $U_{\text{th}}^2=(\pi^2/|\Delta\varepsilon|)(K_{33}-K_{22}^2d^2q_0^2/[K_{33}\pi^2])$, where K_{22} and K_{33} are the twist and bend Frank elastic constants, respectively, and $q_0 = 2\pi/p$ [16]. The chiral nature of the used cholesteric LC tends to reduce U_{th} of the Freedericksz transition, as compared to the nematic counterpart (for which $q_0 = 0$), so that it is in the range of several volts despite the small value of $\Delta\varepsilon$. The experimentally measured value of $U_{\text{th}}\approx 5$ V (Fig. 3(a)) is consistent with an estimation obtained from the above expression using the sample LC parameters. The extinction spectra (Fig. 3(b)) exhibit a strong dependence on the applied voltage $U>U_{\text{th}}$, revealing that both GNRs and dye molecules (Fig. 3(a)-(c)) in this composite follow the changes of the LC orientation with applied U. Interestingly, the PEG-GNR transmission decay faster than the dye's does (Fig. 3(c)).

To characterize the switching kinetics of our LC composite (Fig. 3(d)), we separately probed the response times of both the dichroic dye and the GNRs using optical filters. Rise and decay times were calculated based on the relative light transmittance changes (Fig. 3(e),(f)) between 90% and 10%, respectively. The rise (τ_{rise}) and decay (τ_{decay}) times of switching **N** are related as $\tau_{\text{rise}}=\tau_{\text{decay}}/[(U/U_{\text{th}})^2-1]$ for pristine LCs [14]. Mechanical coupling of GNRs and **N** through the surface anchoring and direct van der Waals interactions between dye and LC molecules previously led to similar relations in guest-host dichroic [1–4] and plasmonic [6–10] LCs. This is also the case for our guest-host LCs that contain plasmonic GNRs and dichroic dye molecules at the same time (Fig. 3(d)). Reorientation of dichroic dye and **N** is faster than that of PEG-functionalized GNRs, which is expected given that the dye molecules are comparable in dimensions to that of LC molecules while GNRs are much larger and exhibit lagging of rotational response, as also observed previously [9]. The average τ_{decay} values for dichroic dye and PEG-functionalized GNRs are 1.33 s and 1.38 s, respectively, relatively long because of the large cell thickness $d\approx 30$ μm, whereas τ_{rise} is in the range of subseconds at $U>10$V (Fig. 3(d)-(f)). These response times can be shortened further by orders of magnitude through reducing d due to the scaling $\tau_{\text{decay}}\propto d^2$, similar to that of pristine LCs [6].

We also designed the nematic LC composite with $\Delta\varepsilon>0$ doped with dichroic dye and DMOAP-SiO_2-GNRs (Fig. 4). DMOAP imposes homeotropic alignment of LC molecules on the surface of GNR nanostructures, resulting in a novel configuration where GNRs orient in the plane perpendicular to **N** and are able to freely rotate around **N**. In a planar cell at $U>U_{\text{th}}$, **N** reorients to align with the applied field, which is followed by the reorientation of the GNRs and the dye molecules which tend to stay perpendicular and parallel to **N**, respectively (Fig. 4(a)). Extinction spectra of this composite demonstrate strong polarization and U

dependences (Fig. 4(b)), allowing for the effective switching between the visible or near infrared absorbance bands in an alternating manner. Using appropriate optical filters, similar values of U_{th} were obtained by measuring independently the optical responses of the GNRs and dye molecules (Fig. 4(c)). The GNR reorientation is slower than both **N** and dichroic dye molecules' reorientation (Fig. 4(d)). Characterization of τ_{rise} and τ_{decay} are shown in Fig. 4(e),(f), respectively. The response times in this composite are faster as compared to its counterpart with the negative dielectric anisotropy (Fig. 3(d)), owing to a larger absolute value of dielectric anisotropy of the LC host. Further exploration of cell configurations and LC composites with various additives in the forms of organic and inorganic materials may allow pre-designed switching of electromagnetic radiation in visible and infrared ranges, depending on the needs of applications.

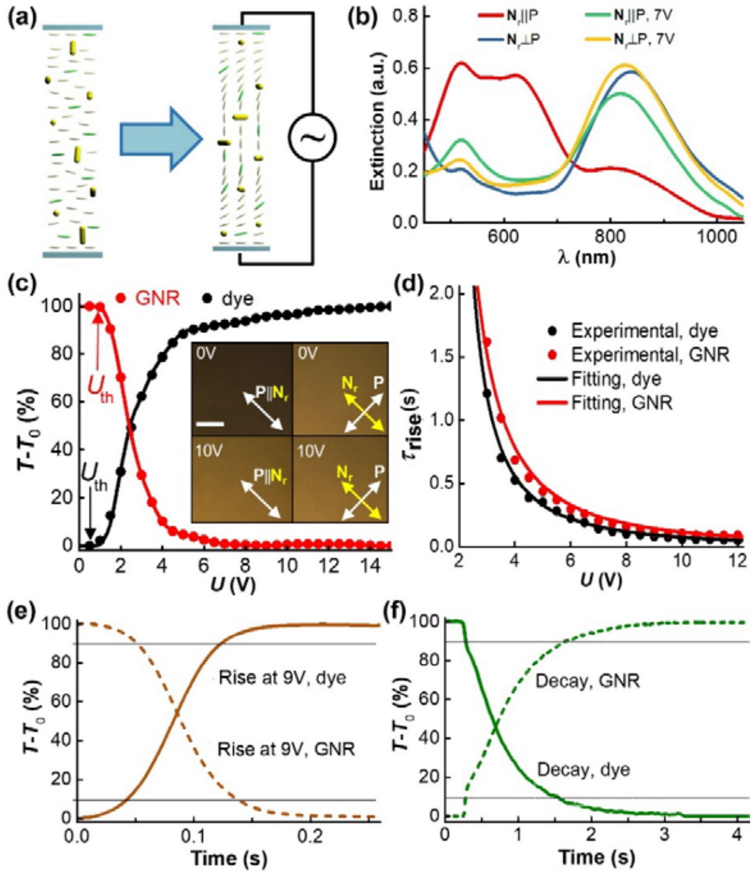

Fig. 4. Nematic LC co-doped with dye and DMOAP-SiO_2-GNRs in a planar cell. (a) Schematics of GNRs (yellow rods) and dichroic dye molecules (green ellipsoids) self-aligning with respect to **N** at (left) $U<U_{th}$ and (right) $U>U_{th}$. (b) Extinction spectra of the planar cell for polarizations $\mathbf{P}||\mathbf{N}_r$ and $\mathbf{P}\perp\mathbf{N}_r$. Switching between the visible-range and the near infrared extinction bands can be done by rotating the cell 90° with respect to **P**, or by applying U. (c) Relative change of transmittance versus U in the spectral ranges of absorption of GNRs and dye obtained using natural white light through the planar cell measured separately utilizing the appropriate optical filters. Insets show micrographs of the planar cell upon rotating **P** from $\mathbf{P}||\mathbf{N}_r$ to $\mathbf{P}\perp\mathbf{N}_r$ and with applying U; the scale bar is 50 µm. (d) Comparison of U-dependent τ_{rise} for dye and GNRs within the same sample. (e,f) Characterization of (e) τ_{rise} and (f) τ_{decay} for dye and GNRs based on relative changes of transmittance (T-T_0) using optical filters to pre-select the respective absorbance bands. Average values of τ_{decay} are 1.37 s and 1.41 s, as determined for the dye and GNRs, respectively.

Optics EXPRESS

4. Discussion and conclusions

To conclude, we designed and implemented mesostructured guest-host LC composites based on co-doping nematics and cholesterics with dichroic dyes and shape-anisotropic plasmonic nanoparticles. Our findings demonstrate that nematic and cholesteric LC hosts with positive or negative dielectric anisotropies and different sample geometries can be used to design guest-host composite systems capable of synchronous (Fig. 3(b)) or alternating (Fig. 4(b)) switching of light transmission in visible and infrared spectral ranges. Relatively short (≤1 s) response times of our guest-host are composites faster than typical responses of solid-state electrochromic devices like the ones utilized in smart windows [17,18]. The response time can be reduced further down to hundreds or even tens of milliseconds by varying gap thickness of the used cells because the LCs are known to respond much faster in thinner cells, as studied before for positive dielectric LCs [6]. Our guest-host mesostructured composites are advantageous compared with other different LC-based composites that are already implemented in smart windows applications. For example, polymer dispersed liquid crystals (PDLC) provide operation modes based on only switching between scattering and clear states. Moreover, the voltage required to switch PDLC is higher by almost one order of magnitude relative to our system, although the response time is faster compared to our system [20,21]. The thermochromic LC systems are driven by higher voltages as well and suffer from nonuniform switching [19]. The dichroic dyes-based conventional guest-host LC displays which show similar response time as our system mainly function in the visible region of the light spectrum, not extending to cover the infrared region of the spectrum [22]. In contrast, we can selectively tune the IR absorbance peak through changing the aspect ratio of the inserted GNRs in these composites to modulate the radiation in many near infrared regions. Although, our examples (Figs. 2(e) and 2(f) and Fig. 3) of switching cholesteric LC configurations exhibit polarization-dependent properties, one can also extend this approach of composite guest-host LCs with both nanoparticles and dyes to the regime of polarization-independent responses when the Mauguin parameter is tuned to be small, $\Delta np/(2\lambda)<1$, which was previously achieved for guest-host LCs utilizing only dichroic dyes [4]. This would be of interest for applications in a new breed of smart windows.

Funding

This study was partially supported by the U.S. Department of Energy, Office of Basic Energy Sciences, Division of Materials Sciences and Engineering, under Award ER46921, contract DE-SC0010305 with the University of Colorado Boulder. G.H.S. also acknowledges support from the King Faisal University.

Acknowledgments

We acknowledge discussions with Haridas Mundoor.

References

1. B. Bahadur, *Handbook of Liquid Crystals* (Wiley-VCH, Weinheim, 1998), 2A.
2. G. H. Heilmeier and L. A. Zanoni, “Guest-host interactions in nematic liquid crystals. A new electro-optic effect,” Appl. Phys. Lett. **13**(3), 91–92 (1968).
3. T. Uchida, H. Seki, C. Shishido, and M. Wada, “Bright dichroic guest–host LCDs without a polarizer,” Proc. Soc. Inf. Disp. **22**(1), 41–46 (1981).
4. B. Taheri, T. Kosa, V. Bodnar, L. Sukhomlinova, L. Su, C. Martincic, J. Chonko, and E.-Y. Park, “Guest-host liquid crystal devices for adaptive window application,” Proc. SPIE 7618. Emerging Liquid Crystal Technologies **V**, 76180W (2010).
5. P. M. Chaikin and T. C. Lubensky, *Principles of condensed matter physics* (Cambridge Univ. Press, 2000).
6. Q. Liu, Y. Yuan, and I. I. Smalyukh, “Electrically and optically tunable plasmonic guest-host liquid crystals with long-range ordered nanoparticles,” Nano Lett. **14**(7), 4071–4077 (2014).
7. Q. Liu, Y. Cui, D. Gardner, X. Li, S. He, and I. I. Smalyukh, “Self-alignment of plasmonic gold nanorods in reconfigurable anisotropic fluids for tunable bulk metamaterial applications,” Nano Lett. **10**(4), 1347–1353 (2010).

Optics EXPRESS

8. L. Jiang, H. Mundoor, Q. Liu, and I. I. Smalyukh, “Electric switching of fluorescence decay in gold-silica-dye nematic nanocolloids mediated by surface plasmons,” ACS Nano **10**(7), 7064–7072 (2016).
9. Y. Zhang, Q. Liu, H. Mundoor, Y. Yuan, and I. I. Smalyukh, “Metal nanoparticle dispersion, alignment, and assembly in nematic liquid crystals for applications in switchable plasmonic color filters and E-polarizers,” ACS Nano **9**(3), 3097–3108 (2015).
10. G. H. Sheetah, Q. Liu, and I. I. Smalyukh, “Self-assembly of predesigned optical materials in nematic codispersions of plasmonic nanorods,” Opt. Lett. **41**(21), 4899–4902 (2016).
11. X. Ye, C. Zheng, J. Chen, Y. Gao, and C. B. Murray, “Using binary surfactant mixtures to simultaneously improve the dimensional tunability and monodispersity in the seeded growth of gold nanorods,” Nano Lett. **13**(2), 765–771 (2013).
12. B. Nikoobakht and M. A. El-Sayed, “Preparation and growth mechanism of gold nanorods (NRs) using seed-mediated growth method,” Chem. Mater. **15**(10), 1957–1962 (2003).
13. D. F. Gardner, J. S. Evans, and I. I. Smalyukh, “Towards reconfigurable optical metamaterials: Colloidal nanoparticle self-assembly and self-alignment in liquid crystals,” Mol. Cryst. Liq. Cryst. **545**(1), 1227–1245 (2011).
14. L. M. Blinov and V. G. Chigrinov, *Electrooptic effects in liquid crystal materials* (Springer-Verlag, 1996).
15. I. I. Smalyukh, B. I. Senyuk, P. Palffy-Muhoray, O. D. Lavrentovich, H. Huang, E. C. Gartland, Jr., V. H. Bodnar, T. Kosa, and B. Taheri, “Electric-field-induced nematic-cholesteric transition and three-dimensional director structures in homeotropic cells,” Phys. Rev. E **72**(6), 061707 (2005).
16. K. A. Crandall, M. R. Fisch, R. G. Petschek, and C. Rosenblatt, “Vanishing Freedericksz transition threshold voltage in a chiral nematic liquid crystal,” Appl. Phys. Lett. **64**(13), 1741–1743 (1994).
17. R. Baetens, B. P. Jelle, and A. Gustavsen, “Properties, requirements and possibilities of smart windows for dynamic daylight and solar energy control in buildings: A state-of-the-art review,” Sol. Energy Mater. Sol. Cells **94**(2), 87–105 (2010).
18. E. L. Runnerstrom, A. Llordés, S. D. Lounis, and D. J. Milliron, “Nanostructured electrochromic smart windows: traditional materials and NIR-selective plasmonic nanocrystals,” Chem. Commun. (Camb.) **50**(73), 10555–10572 (2014).
19. I. Sage, “Thermochromic liquid crystals,” Liq. Cryst. **38**(11–12), 1551–1561 (2011).
20. J. Heo, J.-W. Huh, and T.-H. Yoon, “Fast-switching initially-transparent liquid crystal light shutter with crossed patterned electrodes,” AIP Adv. **5**(4), 047118 (2015).
21. H. Ren and S.-T. Wu, “Anisotropic liquid crystal gels for switchable polarizers and displays,” Appl. Phys. Lett. **81**(8), 1432–1434 (2002).
22. Y.-C. Hsiao, K.-C. Huang, and W. Lee, “Photo-switchable chiral liquid crystal with optical tristability enabled by a photoresponsive azo-chiral dopant,” Opt. Express **25**(3), 2687–2693 (2017).

78.31.211.57 {ts '2022-09-20 04:40:55'}
