# Supplementary material for: Advanced liquid crystal-based switchable optical devices for light protection applications: principles and strategies
Source: Light Sci Appl. 2023 Jan 3;12:11. doi: 10.1038/s41377-022-01032-y (PMC9807646; doi:10.1038/s41377-022-01032-y)
Supplement: Supplementary file 11 — Fig 13 copyright promotion [file 41377_2022_1032_MOESM11_ESM.pdf]

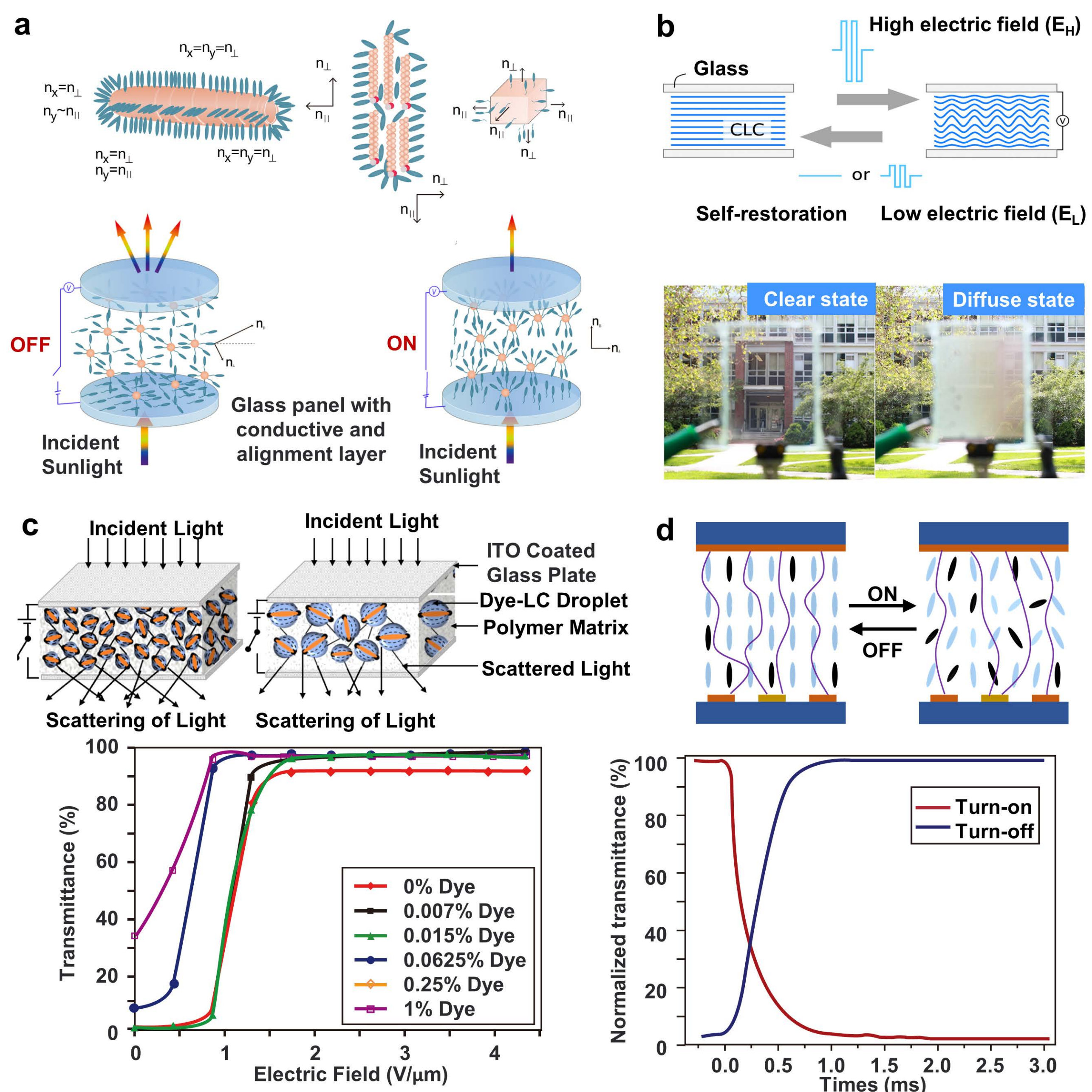

a
$n_x=n_y=n_\perp$
$n_x=n_\perp$
$n_y\sim n_{||}$
$n_x=n_\perp$
$n_y=n_{||}$
$n_x=n_y=n_\perp$
OFF
ON
Glass panel with conductive and alignment layer
Incident Sunlight
Incident Sunlight
b
High electric field (E_H)
Glass
CLC
or
Self-restoration
Low electric field (E_L)
Clear state
Diffuse state
c
Incident Light
Incident Light
ITO Coated Glass Plate
Dye-LC Droplet
Polymer Matrix
Scattered Light
Scattering of Light
Scattering of Light
Transmittance (%)
Electric Field (V/µm)
0% Dye
0.007% Dye
0.015% Dye
0.0625% Dye
0.25% Dye
1% Dye
d
ON
OFF
Normalized transmittance (%)
Times (ms)
Turn-on
Turn-off

RE: Reprint Permissions

发件人: **copyright** <copyright@osa.org>
时 间: 2022年9月27日 (星期二) 下午9 : 35
收件人: ruicong.zhang <ruicong.zhang@hrtcn.org>; copyright <copyright@osa.org>

Dear Dr. Zhang,

Thank you for contacting Optica Publishing Group.

For the use of figure 1 from Chun-Wei Chen, Alyssa N. Brigeman, Tsung-Jui Ho, and Iam Choon Khoo, "Normally transparent smart window based on electrically induced instability in dielectrically negative cholesteric liquid crystal," Opt. Mater. Express 8, 691-697 (2018) and figure 8 from Ibrahim Abdulhalim, P. Lakshmi Madhuri, Mahmud Diab, and Taleb Mokari, "Novel easy to fabricate liquid crystal composite with potential for electrically or thermally controlled transparency windows," Opt. Express 27, 17387-17401 (2019):

> Optica Publishing Group considers your requested use of its copyrighted material to be Fair Use under United States Copyright Law. We request that a complete citation of the original material be included in any publication.
>
> As this article is published under the terms of the Optica Publishing Group Open Access Publishing Agreement, when adapting or otherwise creating a derivative version of the article, users must maintain attribution to the author(s) and the published article's title, journal citation, and DOI. Users should also indicate if changes were made and avoid any implication that the author or Optica Publishing Group endorses the use.

While your publisher should be able to provide additional guidance, we prefer the below citation formats:

> For citations in figure captions:

[Reprinted/Adapted] with permission from [ref #] © The Optical Society. (Please include the full citation in your reference list)

For images without captions:

Journal Vol. #, first page (year published) An example: Opt. Express 27, 1164 (2019)

Please let me know if you have any questions.

Kind Regards,

Hannah Greenwood

Hannah Greenwood

September 27, 2022

Authorized Agent, Optica Publishing Group

OPTICA PUBLISHING GROUP | Formerly OSA

From: ruicong.zhang <ruicong.zhang@hrtcn.org>
Sent: Tuesday, September 20, 2022 9:10 AM
To: Optica Publishing Group Copyright <copyright@osa.org>
Subject: Reprint Permissions

Dear Editor,

Hello! We would like to apply for permission to reproduce images from papers published by your publisher, as detailed below.

Title：Normally transparent smart window based on electrically induced instability in dielectrically negative cholesteric liquid crystal

Author :Chun-Wei Chen, Alyssa N. Brigeman, Tsung-Jui Ho, and Iam Choon Khoo

Volume, issue, and page numbers: 8,3,691-697 (2018)

Journal：Optical Materials Express

Expected content to be reproduced：Fig.1

Information on the papers we plan to publish is as follows.

Title：Advanced liquid crystal-based switchable optical devices for light protection applications: principles and strategies

Publisher：Springer Nature

Journal：Light: Science & Applications

Estimated publication time：In November 2022

Main author of the article：Ruicong Zhang, Zhibo Zhang, Jiecai Han, Lei Yang, Jiajun li,Zicheng Song， Tianyu Wang, Jiaqi Zhu

We admire your society's contribution to the Institute of Optics and hope to obtain permission to reprint images.

With thanks!

Ruicong Zhang

Harbin Institute of Technology

ruicong.zhang

ruicong.zhang@hrtcn.org

快捷回复给所有人...

上一封 下一封

« 返回 | 回复 回复全部 转发 删除 彻底删除 举报 拒收 标记为... 移动到...

Research Article | Vol. 8, No. 3 | 1 Mar 2018 | OPTICAL MATERIALS EXPRESS 691
Optical Materials EXPRESS

Normally transparent smart window based on electrically induced instability in dielectrically negative cholesteric liquid crystal

CHUN-WEI CHEN, ALYSSA N. BRIGEMAN, TSUNG-JUI HO, AND IAM CHOON KHOO*

Department of Electrical Engineering, Pennsylvania State University, University Park, PA 16802, USA
**ick1@psu.edu*

Abstract: We demonstrate a normally transparent smart window based on a cholesteric liquid crystal with negative dielectric anisotropy. The window is electrically switchable between clear and diffuse states. The clear state exhibits a well-aligned planar cholesteric texture and is stable in the absence of an electric field, while the diffuse state is switched on when applying a field higher than the undulation instability threshold. The degree of translucency can be controlled by varying the field strength. When the applied field is removed, the smart window relaxes back to the clear state spontaneously. It is also found that a much faster diffuse–clear process can be stimulated by reducing the field below the instability threshold. The smart window presents itself a promising projector screen for augmented reality applications as it features single stable state, sub-second switching speed, and polymerization-free fabrication.

© 2018 Optical Society of America under the terms of the OSA Open Access Publishing Agreement

OCIS codes: (230.3720) Liquid-crystal devices; (130.4815) Optical switching devices.

References and links

1. T. Sielhorst, M. Feuerstein, and N. Navab, "Advanced Medical Displays: A Literature Review of Augmented Reality," J. Disp. Technol. **4**(4), 451–467 (2008).
2. M. Tönnis, C. Lange, and G. Klinker, "Visual Longitudinal and Lateral Driving Assistance in the Head-Up Display of Cars," in Proceedings of 6th IEEE and ACM International Symposium on Mixed and Augmented Reality (IEEE, 2007), pp. 91–94.
3. H. Kim, X. Wu, J. L. Gabbard, and N. F. Polys, "Exploring head-up augmented reality interfaces for crash warning systems," in Proceedings of the 5th International Conference on Automotive User Interfaces and Interactive Vehicular Applications (ACM, 2013), pp. 224–227.
4. C. M. Lampert, "Large-area smart glass and integrated photovoltaics," Sol. Energy Mater. Sol. Cells **76**(4), 489–499 (2003).
5. Y.-H. Fan, H. Ren, and S.-T. Wu, "Normal-mode anisotropic liquid-crystal gels," Appl. Phys. Lett. **82**(18), 2945–2947 (2003).
6. J. Murray, D. Ma, and J. N. Munday, "Electrically Controllable Light Trapping for Self-Powered Switchable Solar Windows," ACS Photonics **4**(1), 1–7 (2017).
7. Y.-C. Hsiao, C.-Y. Tang, and W. Lee, "Fast-switching bistable cholesteric intensity modulator," Opt. Express **19**(10), 9744–9749 (2011).
8. C.-C. Li, H.-Y. Tseng, T.-W. Pai, Y.-C. Wu, W.-H. Hsu, H.-C. Jau, C.-W. Chen, and T.-H. Lin, "Bistable cholesteric liquid crystal light shutter with multielectrode driving," Appl. Opt. **53**(22), E33–E37 (2014).
9. C.-C. Li, H.-Y. Tseng, H.-C. Liao, H.-M. Chen, T. Hsieh, S.-A. Lin, H.-C. Jau, Y.-C. Wu, Y.-L. Hsu, W.-H. Hsu, and T.-H. Lin, "Enhanced image quality of OLED transparent display by cholesteric liquid crystal back-panel," Opt. Express **25**(23), 29199–29206 (2017).
10. K.-T. Cheng, P.-Y. Lee, M. M. Qasim, C.-K. Liu, W.-F. Cheng, and T. D. Wilkinson, "Electrically Switchable and Permanently Stable Light Scattering Modes by Dynamic Fingerprint Chiral Textures," ACS Appl. Mater. Interfaces **8**(16), 10483–10493 (2016).
11. A. Moheghi, H. Nemati, Y. Li, Q. Li, and D.-K. Yang, "Bistable salt doped cholesteric liquid crystals light shutter," Opt. Mater. **52**, 219–223 (2016).
12. S. V. Serak, U. Hrozhyk, J. Hwang, N. V. Tabiryan, D. Steeves, and B. R. Kimball, "High contrast switching of transmission due to electrohydrodynamic effect in stacked thin systems of liquid crystals," Appl. Opt. **55**(30), 8506–8512 (2016).
13. Y. Suzuki, N. Mizoshita, K. Hanabusa, and T. Kato, "Homeotropically oriented nematic physical gels for electrooptical materials," J. Mater. Chem. **13**(12), 2870–2874 (2003).

#320342 https://doi.org/10.1364/OME.8.000691
Journal © 2018 Received 22 Jan 2018; revised 20 Feb 2018; accepted 20 Feb 2018; published 23 Feb 2018

Optical Materials EXPRESS

14. Y.-H. Fan, H. Ren, X. Liang, Y.-H. Lin, and S.-T. Wu, "Dual-frequency liquid crystal gels with submillisecond response time," Appl. Phys. Lett. **85**(13), 2451–2453 (2004).
15. Y.-H. Lin, H. Ren, S. Gauza, Y.-H. Wu, Y. Zhou, and S.-T. Wu, "High Contrast and Fast Response Polarization-Independent Reflective Display Using a Dye-Doped Dual-Frequency Liquid Crystal Gel," Mol. Cryst. Liq. Cryst. (Phila. Pa.) **453**(1), 371–378 (2006).
16. F. Ahmad, M. Jamil, and Y. J. Jeon, "Current Trends in Studies on Reverse-Mode Polymer Dispersed Liquid-Crystal Films – A Review," Electron. Mater. Lett. **10**(4), 679–692 (2014).
17. H. Khandelwal, R. C. G. M. Loonen, J. L. M. Hensen, A. P. H. J. Schenning, and M. G. Debije, "Application of broadband infrared reflector based on cholesteric liquid crystal polymer bilayer film to windows and its impact on reducing the energy consumption in buildings," J. Mater. Chem. A Mater. Energy Sustain. **2**(35), 14622 (2014).
18. G. Palermo, L. De Sio, and C. Umeton, "Flexible Structures Based on a Short Pitch Cholesteric Liquid Crystals," Mol. Cryst. Liq. Cryst. (Phila. Pa.) **619**(1), 35–41 (2015).
19. H. Khandelwal, A. P. H. J. Schenning, and M. G. Debije, "Infrared Regulating Smart Window Based on Organic Materials," Adv. Energy Mater. **7**(14), 1602209 (2017).
20. I. C. Khoo, C.-W. Chen, T.-J. Ho, and T.-H. Lin, "Femtoseconds-picoseconds nonlinear optics with nearly-mm thick cholesteric liquid crystals," in Proc. SPIE **10125**, E*merging Liquid Crystal Technologies XII*, L.-C. Chien, ed. (2017), 1012507.
21. C.-W. Chen, X. Guo, X. Ni, T.-H. Lin, and I. C. Khoo, "Slowing sub-picosecond laser pulses with 0.55 mm-thick cholesteric liquid crystal," Opt. Mater. Express **7**(6), 2005–2011 (2017).
22. B. I. Senyuk, I. I. Smalyukh, and O. D. Lavrentovich, "Undulations of lamellar liquid crystals in cells with finite surface anchoring near and well above the threshold," Phys. Rev. E Stat. Nonlin. Soft Matter Phys. **74**(1 Pt 1), 011712 (2006).

1. Introduction

Augmented reality (AR) is an emerging technology that projects useful information directly into the user's field of vision, integrated into environmental scenery [1–3]. A bright, multicolored background with fine details can significantly degrade the performance of such see-through displays. To circumvent the crosstalk between the virtual graphics and the background, a projector screen with controllable local translucency is necessary. Existing smart window technologies that enable switching between clear (transparent) and diffuse (translucent) states include suspended particle device (SPD), polymer-dispersed liquid crystal (PDLC, including polymer-network liquid crystal), bistable cholesteric texture (BCT), and dynamic scattering mode (DSM) nematic liquid crystal. However, for safety reasons, normally translucent modes (*e.g.*, SPD [4] and normal-mode PDLC [5,6]) and bistable modes (*e.g.*, BCT [7–11]) are unfavorable for practical use. For instance, in automotive applications, such as AR head-up display [2,3], the driver's visibility can be reduced dramatically if the circuit suddenly fails and turns the whole screen scattering. *Normally transparent* shutters are therefore preferred. In this context, nematic DSM and reverse-mode PDLC are good candidates. DSM [12] exploits the electro-hydrodynamic instabilities in a liquid crystal to create optical scattering, yet, the induced ion movement could establish a permanent inner electric field that leads to performance degradation over time. In a reverse-mode PDLC [13–16], the clear–diffuse switching is driven under the action of a dielectric torque (no ionic mechanism is involved). However, the driving voltage and other properties of the PDLC depend strongly on the morphology of the polymer network, thus less feasible for large-scale fabrication. High angular dependence of the clear-state transparency (which originates from the liquid crystal/polymer index-matching mechanism [16]) is also of serious concern, especially for automotive applications.

Here we report a *normally-transparent* dynamic diffuser based on a newly observed field induced dielectric instability in cholesteric liquid crystal (CLC). Besides exhibiting infrared filtering ability due to the CLC's photonic bandgap and angle-independent clear-state transparency since no heterogeneous optical composite is used [17–20], this field-induced transparent–translucent change can potentially be utilized to construct smart windows with desirable characteristics such as fast and reversible switching (10's–500 ms), fast self-restoration from the diffuse state to the clear state (subsecond–seconds), and polymerization-free fabrication.

Optical Materials EXPRESS

2. Sample preparation

CLCs are liquid crystals of which the molecules are self-assembled into one-dimensional helices. It was formulated by mixing a nematic liquid crystal with negative dielectric anisotropy DNM-C (ordinary index n_e ≈1.59, extraordinary index n_o ≈1.48, and dielectric anisotropy $\Delta\varepsilon = \varepsilon_{//} - \varepsilon_{\perp} \approx -3.6$; same as the nematic host used in [21]) with a trace of a chiral agent R5011 (helical twisting power ≈130 μm^{-1}, from HCCH). The samples were fabricated by filling glass sandwich cells with a dielectrically negative cholesteric liquid crystal via capillary action. The inner surfaces of the glass cells were pre-coated with indium tin oxide to serve as transparent electrodes and poly(vinyl alcohol) films to induce planar alignment. Cells with two different cell gaps, 32 and 45 μm, were used in the following experiments. The reflection band was tuned to the desired near-infrared spectral region (in this case around 800 nm) by controlling the concentration of R5011 in the CLC. The experiments were performed with single-pixel samples; for practical use, the CLC cells will be pixelated so as to achieve local translucency control.

3. Results and discussion

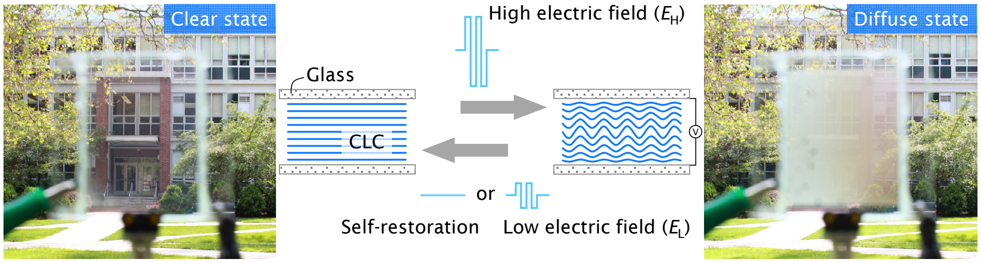

Fig. 1. Schematic and photographs of the proposed CLC smart window (d ≈45 μm).

In the absence of an external field, the helices are aligned along the surface normal by the strong anchoring force of the alignment layer—known as the planar state. The CLC hence acts as a Bragg reflector in the near infrared regime and is highly transparent in the visible [Fig. 1, left panel]. When an alternating-current electric field is applied to the CLC cell, the degree of translucency, characterized by the specular transmission spectra shown in Fig. 2(a), can be either improved or degraded depending on the field strength (E) and frequency (f). The spectra were measured by using a spectrometer (USB4000, Ocean Optics) that has a finite aperture of 5 mm in diameter and was set at 10 cm away from the sample.

In the low applied field regime (*e.g.*, E < 2.8 V/μm at f = 1 kHz), an increase of the electric field strength leads to an overall rise of the transmittance in the visible range and sharpening of the photonic bandgap, *cf.* Fig. 2. This is due to the negative dielectric anisotropy of the CLC; the field-induced dipole moment is perpendicular to the principal molecular axis, and so the electric torque brings the molecules away from the field axis. As revealed by the microscope images in Fig. 3(a), the grain boundaries of the planar domains diminish with increasing field, smoothening the texture.

Optical Materials EXPRESS

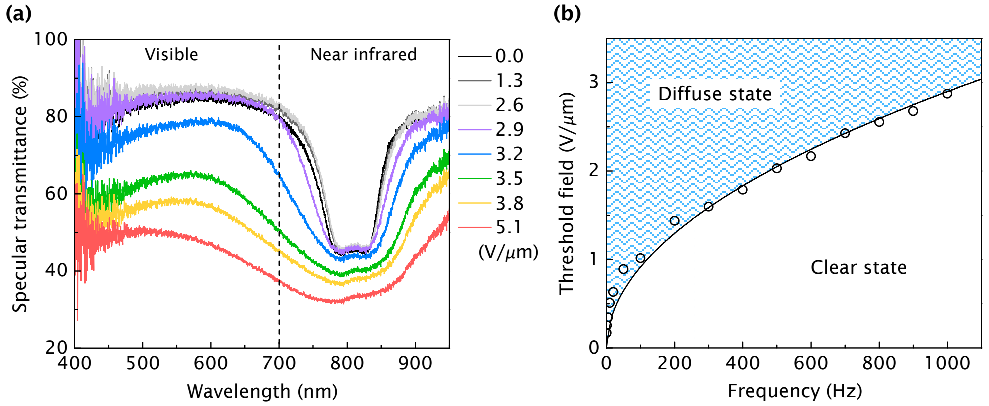

Fig. 2. (a) Specular transmission spectra at different field strengths (d ≈32 μm and f = 1 kHz). (b) Dependence of instability threshold on driving frequency.

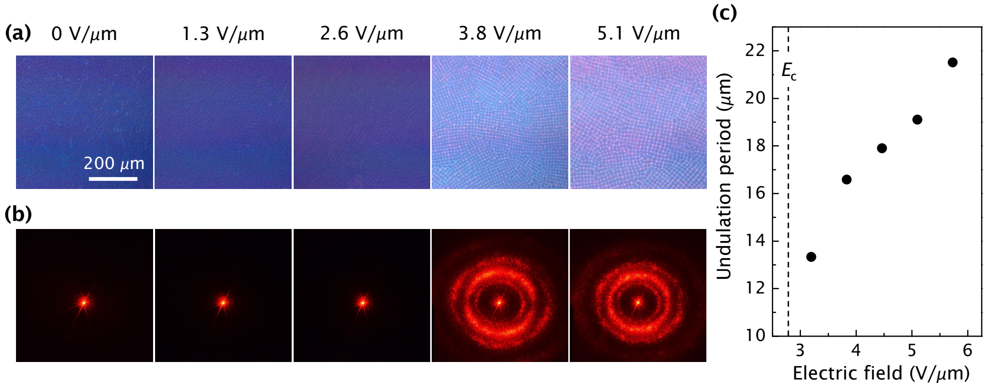

Fig. 3. (a) Microscope images, (b) far-field diffraction patterns, and (c) undulation periods of CLC at different field strengths above the threshold field E_c ≈2.8 V (d ≈32 μm and f = 1 kHz).

In response to fields above a certain threshold [*i.e.*, the solid curve in Fig. 2(b)], the planar state becomes structurally unstable and therefore scatters light. The data points in Fig. 2(b) were collected by probing the specular transmission of the CLC cell with a HeNe laser at λ = 633 nm and recording the field strength at which the transmittance begins to drop. The measured threshold field scales with the square root of driving frequency, suggesting that the observed blurring of the sample was caused by the field-induced instability in the dielectric regime [22]. A planar CLC can be regarded as layers of different director orientations stacked along the helical axis. In a CLC that is experiencing field-induced instability, the associated layers tend to tilt towards the field axis.

The instability result in platelets of two-dimensional (2D) periodic undulations, as a consequence of balancing the induced torque with the confining substrates and the surface anchoring by poly(vinyl alcohol) [Fig. 3(a)]. At wavelengths far from the photonic bandgap (*e.g.*, in the visible regime), each platelet acts as an optical grating with micrometer-scaled periods and thus gives rise to optical diffractions. Figure 3(b) depicts that the diffractions are quasi-omnidirectional (*cf.* the round diffraction patterns appearing at E ≈3.8 and 5.1 V/μm) in that the 2D grating orients differently from one platelet to another. The grating period (Λ) ranges from 13 to 22 μm and increases with the field [Fig. 3(c)]. Given that the cell gap (d) is ~30 μm, the grating operates in the Raman-Nath regime ($\Lambda^2 \gg \lambda \cdot d$), leading to multiple orders of diffraction. The quasi-omnidirectional multi-order diffractions effectively generates spatial crosstalk in the optical image that passes through the CLC cell. Meanwhile, the reflection band becomes broadened. According to the Bragg's law, the bandgap (λ_B) of the CLC blue-

Optical Materials EXPRESS

shifts as the helix tilts away from the viewing direction—$\lambda_B = \bar{n} \cdot p \cdot \cos\theta$, where $\bar{n} \approx (n_e + n_o)/2$ is the average index of the CLC and θ is the angle between the helical axis and the surface normal [21]. In an undulated CLC, the tilt angle of the helix varies in space and so does the magnitude of the blue-shift, *cf.* Figures 1 and 2(a) [22]. The bandgap is expanded toward the visible regime with increasing applied field, indicating the possibility of grayscale control. The platelet (grain) boundaries and inhomogeneous period of undulation also contribute to light diffusion. The translucency can be further improved by increasing the interaction length (increasing the cell gap or stacking cells).

To gain more insight into the switching dynamics, we monitored the specular transmission change of the CLC cell in electric fields above the instability threshold. The HeNe laser was employed as a probe that passed through the cell and arrived at a photodiode (818-SL, Newport; the active diameter is 11.3 mm) which was connected via a power meter (1830-C, Newport) to an oscilloscope (TDS360, Tektronix). The distance between the cell and photodiode was ~0.7 m. Figure 4(a) shows the clear–diffuse switching with a 1 kHz AC field (E_H) of ~3.8 V/μm. The molecules were first aligned by the field, revealed by a sharp rise in specular transmittance (T) from 86 to 89% within 5 ms. At ~65 ms upon the application of the field, the undulation instability became visible and caused a transmittance drop from 89 to 11%. The corresponding fall time was ~40 ms. This was followed by some fluctuations and soon became steady at $T \approx 26\%$. The field was then removed at ~2.49 s. The CLC hence relaxed to the clear state, taking ~525 ms to restore 90% of the initial transmittance (henceforth defined as the rise time). Such a field-off response is in contrast to the CLC smart windows that are driven by the field-induced instability in the conduction regime (usually at low driving frequencies), of which the diffuse state is stable upon the removal of the applied field [10,11].

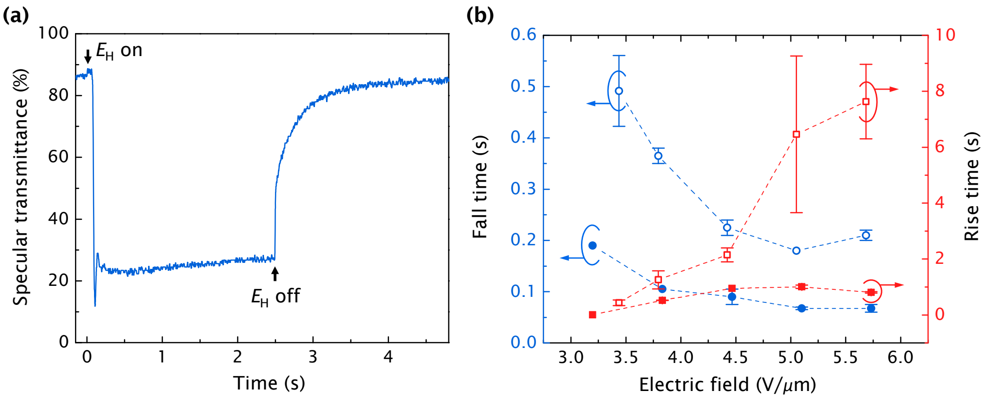

Fig. 4. (a) Time-resolved specular transmittance revealing the clear–diffuse switching and self-restoration processes ($E_H \approx 3.8$ V/μm, $f = 1$ kHz, and $d \approx 32$ μm). (b) Response time for the clear–diffuse (fall time, circles) and diffuse–clear (rise time, squares) at different field strengths (E_H) and thicknesses (filled symbols: 32 μm, open symbols: 45 μm).

As depicted in Fig. 4(b), the response times vary with the driving frequency and cell gap. The fall time is in general one order of magnitude shorter than the (self-restoration) rise time. In the 32 μm-thick cell, the fall time is on the order of 10's ms and drops with increasing field strength. The rise time follows an opposite trend, from 5 ms at 3.2 V/μm to ~1 s at ~5 V/μm. Although increasing the cell gap to 45 μm can improve the translucency, both the deformation and relaxation processes become slower. Adopting the layer stack configuration [12,17,20] or increasing the anchoring force of the surface alignment could prove effective in reducing the response time of the self-restoration process while retaining high translucency in the diffuse state. Note that the spontaneous diffuse–clear transition is the key to circumventing a reduction of visibility caused by unexpected failure of the driver circuit that otherwise turns

Optical Materials EXPRESS

the whole window into a permanent diffuse state. This can be a serious concern when using a normally translucent or bistable window, but not the case for a normally transparent window. Under normal operating conditions, this relatively slow process (compared to the fall time) can be sped up to several 10's ms by applying a small electric field.

Recalling the field strength dependence of specular transmission in Fig. 2, applying an electric field below the instability threshold to a deformed CLC can assist the diffuse–clear restoration by exerting a torque to reorient the LC molecules until they are aligned perpendicular to the field axis. Figure 5 demonstrates the spontaneous and field-induced restoration processes with a 45 μm-thick CLC. When a high field (E_H ≈4.5 V/μm) is applied, the CLC first experiences a transient higher-transmittance clear state (T ≈90%) that lasts for only a few 10's ms and then transitions into a diffuse state (T ≈20%). Switching off the high field brings the CLC back to the stable clear state through the self-relaxation process (induced by the surface anchoring solely). A successive application of E_H drives the sample into the diffuse state again. Subsequently, reducing the field strength to 2.7 V/μm (E_L), the clear state is restored with rise time of only ~70 ms.

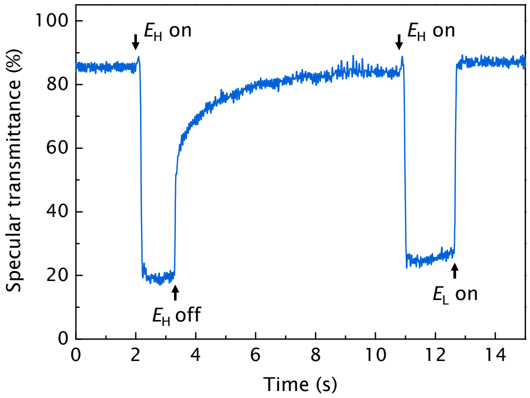

Fig. 5. Time-resolved specular transmittance diagram showing the field-induced clear–diffuse transition, spontaneous restoration, and field-induced restoration process (E_H ≈4.5 V/μm, E_L ≈2.7 V/μm, d ≈45 μm, and f ≈1 kHz).

4. Summary

A normally transparent smart window has been demonstrated, enabled by the electrically induced undulation instability in a CLC with negative dielectric anisotropy. The window is switchable between the clear and diffuse states with controllable translucency by varying the field strength. The clear state, in which the cholesteric helices are uniformly oriented along the window normal, is the only stable state in the absence of an electric field, an important characteristic for safety concerns. By applying an electric field higher than the threshold for the undulation instability to occur, the CLC window is switched to the diffuse state taking 10's–100's ms. When the applied field is removed, the clear state is restored spontaneously with response time of subsecond–seconds depending on the cell gap and field strength. A much faster restoration of < 100 ms can be gained by directly reducing the field strength below the instability threshold instead of switching off the voltage. Featuring single stable state, subsecond-scaled response, and polymerization-free fabrication, the CLC smart window shows great potential for AR applications.

Funding

Pennsylvania State University (W. E. Leonhard Professorship); Air Force Research Laboratory.

Optical Materials EXPRESS

Acknowledgments

The authors are grateful to Tsung-Hsien Lin for providing the nematic material.

45.142.167.203 {ts '2022-09-20 21:45:16'}

ELSEVIER LICENSE
TERMS AND CONDITIONS

Sep 19, 2022

This Agreement between Harbin Institute of Technology -- Ruicong Zhang ("You") and Elsevier ("Elsevier") consists of your license details and the terms and conditions provided by Elsevier and Copyright Clearance Center.

License Number	5392380608516
License date	Sep 19, 2022
Licensed Content Publisher	Elsevier
Licensed Content Publication	Journal of Physics and Chemistry of Solids
Licensed Content Title	Effects of dye doping on electro-optical, thermo-electro-optical and dielectric properties of polymer dispersed liquid crystal films
Licensed Content Author	Anuja Katariya-Jain,R.R. Deshmukh
Licensed Content Date	Jan 1, 2022
Licensed Content Volume	160
Licensed Content Issue	n/a
Licensed Content Pages	1
Start Page	110363
End Page	0
Type of Use	reuse in a journal/magazine
Requestor type	academic/educational institute
Portion	figures/tables/illustrations
Number of figures/tables/illustrations	2
Format	both print and electronic
Are you the author of this Elsevier article?	No
Will you be translating?	No
Title of new article	Advanced liquid crystal-based switchable optical devices for light protection applications: principles and strategies
Lead author	Ruicong Zhang, Zhibo Zhang, Jiecai Han, Lei Yang, Jiajun li, Zicheng Song Tianyu Wang, Jiaqi Zhu
Title of targeted journal	Light: Science & Applications
Publisher	Springer Nature
Expected publication date	Nov 2022
Portions	Figure 9, Figure 10
Requestor Location	Harbin Institute of Technology<br>No. 92, Xidazhi Street, Nangang District<br><br>Harbin, 150080<br>China<br>Attn: Harbin Institute of Technology
Publisher Tax ID	GB 494 6272 12
Total	**0.00 USD**

Terms and Conditions

INTRODUCTION

1. The publisher for this copyrighted material is Elsevier. By clicking "accept" in connection with completing this licensing transaction, you agree that the following terms and conditions apply to this transaction (along with the Billing and Payment terms

and conditions established by Copyright Clearance Center, Inc. ("CCC"), at the time that you opened your Rightslink account and that are available at any time at http://myaccount.copyright.com).

GENERAL TERMS

2. Elsevier hereby grants you permission to reproduce the aforementioned material subject to the terms and conditions indicated.
3. Acknowledgement: If any part of the material to be used (for example, figures) has appeared in our publication with credit or acknowledgement to another source, permission must also be sought from that source. If such permission is not obtained then that material may not be included in your publication/copies. Suitable acknowledgement to the source must be made, either as a footnote or in a reference list at the end of your publication, as follows:
"Reprinted from Publication title, Vol /edition number, Author(s), Title of article / title of chapter, Pages No., Copyright (Year), with permission from Elsevier [OR APPLICABLE SOCIETY COPYRIGHT OWNER]." Also Lancet special credit - "Reprinted from The Lancet, Vol. number, Author(s), Title of article, Pages No., Copyright (Year), with permission from Elsevier."
4. Reproduction of this material is confined to the purpose and/or media for which permission is hereby given.
5. Altering/Modifying Material: Not Permitted. However figures and illustrations may be altered/adapted minimally to serve your work. Any other abbreviations, additions, deletions and/or any other alterations shall be made only with prior written authorization of Elsevier Ltd. (Please contact Elsevier's permissions helpdesk here). No modifications can be made to any Lancet figures/tables and they must be reproduced in full.
6. If the permission fee for the requested use of our material is waived in this instance, please be advised that your future requests for Elsevier materials may attract a fee.
7. Reservation of Rights: Publisher reserves all rights not specifically granted in the combination of (i) the license details provided by you and accepted in the course of this licensing transaction, (ii) these terms and conditions and (iii) CCC's Billing and Payment terms and conditions.
8. License Contingent Upon Payment: While you may exercise the rights licensed immediately upon issuance of the license at the end of the licensing process for the transaction, provided that you have disclosed complete and accurate details of your proposed use, no license is finally effective unless and until full payment is received from you (either by publisher or by CCC) as provided in CCC's Billing and Payment terms and conditions. If full payment is not received on a timely basis, then any license preliminarily granted shall be deemed automatically revoked and shall be void as if never granted. Further, in the event that you breach any of these terms and conditions or any of CCC's Billing and Payment terms and conditions, the license is automatically revoked and shall be void as if never granted. Use of materials as described in a revoked license, as well as any use of the materials beyond the scope of an unrevoked license, may constitute copyright infringement and publisher reserves the right to take any and all action to protect its copyright in the materials.
9. Warranties: Publisher makes no representations or warranties with respect to the licensed material.
10. Indemnity: You hereby indemnify and agree to hold harmless publisher and CCC, and their respective officers, directors, employees and agents, from and against any and all claims arising out of your use of the licensed material other than as specifically authorized pursuant to this license.
11. No Transfer of License: This license is personal to you and may not be sublicensed, assigned, or transferred by you to any other person without publisher's written permission.
12. No Amendment Except in Writing: This license may not be amended except in a writing signed by both parties (or, in the case of publisher, by CCC on publisher's behalf).
13. Objection to Contrary Terms: Publisher hereby objects to any terms contained in any purchase order, acknowledgment, check endorsement or other writing prepared by you, which terms are inconsistent with these terms and conditions or CCC's Billing and Payment terms and conditions. These terms and conditions, together with CCC's Billing and Payment terms and conditions (which are incorporated herein), comprise the entire agreement between you and publisher (and CCC) concerning this licensing transaction. In the event of any conflict between your obligations established by these terms and conditions and those established by CCC's Billing and Payment terms and conditions, these terms and conditions shall control.
14. Revocation: Elsevier or Copyright Clearance Center may deny the permissions described in this License at their sole discretion, for any reason or no reason, with a full refund payable to you. Notice of such denial will be made using the contact information provided by you. Failure to receive such notice will not alter or invalidate the denial. In no event will Elsevier or Copyright Clearance Center be responsible or liable for any costs, expenses or damage incurred by you as a result of a denial of your permission request, other than a refund of the amount(s) paid by you to Elsevier and/or Copyright Clearance Center for denied permissions.

LIMITED LICENSE

The following terms and conditions apply only to specific license types:
15. **Translation**: This permission is granted for non-exclusive world **English** rights only unless your license was granted for translation rights. If you licensed translation rights you may only translate this content into the languages you requested. A professional translator must perform all translations and reproduce the content word for word preserving the integrity of the article.
16. **Posting licensed content on any Website**: The following terms and conditions apply as follows: Licensing material from an Elsevier journal: All content posted to the web site must maintain the copyright information line on the bottom of each image; A hyper-text must be included to the Homepage of the journal from which you are licensing at http://www.sciencedirect.com/science/journal/xxxxx or the Elsevier homepage for books at http://www.elsevier.com; Central Storage: This license does not include permission for a scanned version of the material to be stored in a central repository such as that provided by Heron/XanEdu.
Licensing material from an Elsevier book: A hyper-text link must be included to the Elsevier homepage at http://www.elsevier.com . All content posted to the web site must maintain the copyright information line on the bottom of each image.

Posting licensed content on Electronic reserve: In addition to the above the following clauses are applicable: The web site must be password-protected and made available only to bona fide students registered on a relevant course. This permission is granted for 1 year only. You may obtain a new license for future website posting.
17. **For journal authors:** the following clauses are applicable in addition to the above:
Preprints:
A preprint is an author's own write-up of research results and analysis, it has not been peer-reviewed, nor has it had any other value added to it by a publisher (such as formatting, copyright, technical enhancement etc.).
Authors can share their preprints anywhere at any time. Preprints should not be added to or enhanced in any way in order to appear more like, or to substitute for, the final versions of articles however authors can update their preprints on arXiv or RePEc with their Accepted Author Manuscript (see below).
If accepted for publication, we encourage authors to link from the preprint to their formal publication via its DOI. Millions of researchers have access to the formal publications on ScienceDirect, and so links will help users to find, access, cite and use the best available version. Please note that Cell Press, The Lancet and some society-owned have different preprint policies. Information on these policies is available on the journal homepage.
Accepted Author Manuscripts: An accepted author manuscript is the manuscript of an article that has been accepted for publication and which typically includes author-incorporated changes suggested during submission, peer review and editor-author communications.
Authors can share their accepted author manuscript:

- immediately
    - via their non-commercial person homepage or blog
    - by updating a preprint in arXiv or RePEc with the accepted manuscript
    - via their research institute or institutional repository for internal institutional uses or as part of an invitation-only research collaboration work-group
    - directly by providing copies to their students or to research collaborators for their personal use
    - for private scholarly sharing as part of an invitation-only work group on commercial sites with which Elsevier has an agreement
- After the embargo period
    - via non-commercial hosting platforms such as their institutional repository
    - via commercial sites with which Elsevier has an agreement

In all cases accepted manuscripts should:

- link to the formal publication via its DOI
- bear a CC-BY-NC-ND license - this is easy to do
- if aggregated with other manuscripts, for example in a repository or other site, be shared in alignment with our hosting policy not be added to or enhanced in any way to appear more like, or to substitute for, the published journal article.

Published journal article (JPA): A published journal article (PJA) is the definitive final record of published research that appears or will appear in the journal and embodies all value-adding publishing activities including peer review co-ordination, copy-editing, formatting, (if relevant) pagination and online enrichment.
Policies for sharing publishing journal articles differ for subscription and gold open access articles:
Subscription Articles: If you are an author, please share a link to your article rather than the full-text. Millions of researchers have access to the formal publications on ScienceDirect, and so links will help your users to find, access, cite, and use the best available version.
Theses and dissertations which contain embedded PJAs as part of the formal submission can be posted publicly by the awarding institution with DOI links back to the formal publications on ScienceDirect.
If you are affiliated with a library that subscribes to ScienceDirect you have additional private sharing rights for others' research accessed under that agreement. This includes use for classroom teaching and internal training at the institution (including use in course packs and courseware programs), and inclusion of the article for grant funding purposes.
Gold Open Access Articles: May be shared according to the author-selected end-user license and should contain a CrossMark logo, the end user license, and a DOI link to the formal publication on ScienceDirect.
Please refer to Elsevier's posting policy for further information.
18. **For book authors** the following clauses are applicable in addition to the above: Authors are permitted to place a brief summary of their work online only. You are not allowed to download and post the published electronic version of your chapter, nor may you scan the printed edition to create an electronic version. **Posting to a repository:** Authors are permitted to post a summary of their chapter only in their institution's repository.
19. **Thesis/Dissertation**: If your license is for use in a thesis/dissertation your thesis may be submitted to your institution in either print or electronic form. Should your thesis be published commercially, please reapply for permission. These requirements include permission for the Library and Archives of Canada to supply single copies, on demand, of the complete thesis and include permission for Proquest/UMI to supply single copies, on demand, of the complete thesis. Should your thesis be published commercially, please reapply for permission. Theses and dissertations which contain embedded PJAs as part of the formal submission can be posted publicly by the awarding institution with DOI links back to the formal publications on ScienceDirect.

Elsevier Open Access Terms and Conditions
You can publish open access with Elsevier in hundreds of open access journals or in nearly 2000 established subscription journals that support open access publishing. Permitted third party re-use of these open access articles is defined by the author's choice of Creative Commons user license. See our open access license policy for more information.
Terms & Conditions applicable to all Open Access articles published with Elsevier:
Any reuse of the article must not represent the author as endorsing the adaptation of the article nor should the article be modified in such a way as to damage the author's honour or reputation. If any changes have been made, such changes must be clearly indicated.
The author(s) must be appropriately credited and we ask that you include the end user license and a DOI link to the formal publication on ScienceDirect.
If any part of the material to be used (for example, figures) has appeared in our publication with credit or acknowledgement to another source it is the responsibility of the user to ensure their reuse complies with the terms and conditions determined by the rights holder.
Additional Terms & Conditions applicable to each Creative Commons user license:
CC BY: The CC-BY license allows users to copy, to create extracts, abstracts and new works from the Article, to alter and revise the Article and to make commercial use of the Article (including reuse and/or resale of the Article by commercial entities), provided the user gives appropriate credit (with a link to the formal publication through the relevant DOI), provides a link to the license, indicates if changes were made and the licensor is not represented as endorsing the use made of the work. The full details of the license are available at http://creativecommons.org/licenses/by/4.0.
CC BY NC SA: The CC BY-NC-SA license allows users to copy, to create extracts, abstracts and new works from the Article, to alter and revise the Article, provided this is not done for commercial purposes, and that the user gives appropriate credit (with a link to the formal publication through the relevant DOI), provides a link to the license, indicates if changes were made and the licensor is not represented as endorsing the use made of the work. Further, any new works must be made available on the same conditions. The full details of the license are available at http://creativecommons.org/licenses/by-nc-sa/4.0.
CC BY NC ND: The CC BY-NC-ND license allows users to copy and distribute the Article, provided this is not done for commercial purposes and further does not permit distribution of the Article if it is changed or edited in any way, and provided the user gives appropriate credit (with a link to the formal publication through the relevant DOI), provides a link to the license, and that the licensor is not represented as endorsing the use made of the work. The full details of the license are available at http://creativecommons.org/licenses/by-nc-nd/4.0. Any commercial reuse of Open Access articles published with a CC BY NC SA or CC BY NC ND license requires permission from Elsevier and will be subject to a fee.
Commercial reuse includes:

- Associating advertising with the full text of the Article
- Charging fees for document delivery or access
- Article aggregation
- Systematic distribution via e-mail lists or share buttons

Posting or linking by commercial companies for use by customers of those companies.

20. **Other Conditions**:

v1.10

Questions? customercare@copyright.com or +1-855-239-3415 (toll free in the US) or +1-978-646-2777.

Journal of Physics and Chemistry of Solids 160 (2022) 110363

Contents lists available at ScienceDirect

Journal of Physics and Chemistry of Solids

journal homepage: www.elsevier.com/locate/jpcs

Effects of dye doping on electro-optical, thermo-electro-optical and dielectric properties of polymer dispersed liquid crystal films

Anuja Katariya-Jain, R.R. Deshmukh *

Department of Physics, Institute of Chemical Technology, Matunga, Mumbai, 400019, India

ARTICLE INFO

Keywords:
Polymer dispersed liquid crystal (PDLC)
Phase separation
Dye–doped PDLC (DPDLC)
Electro-optical
Dielectric relaxation spectroscopy (DRS)
Cole–Cole modeling

ABSTRACT

In this study, polymer dispersed liquid crystal (PDLC) films comprising a phase separated low molecular weight thermotropic liquid crystal (LC) and high molecular weight polymer were, prepared using the polymer–induced phase separation method. In order to enhance the optical efficiency of the PDLC films, LC was doped with dichroic azo dye. Various characterization techniques were employed to determine the properties of the components and, the operating principles of the dye–doped PDLC (DPDLC) films. Morphological studies using polarizing optical microscopy and scanning electron microscopy indicated that the LC droplet configuration changed with various dye concentrations and voltages. Electro-optical analysis showed that the performance of the DPDLC film with a low dye concentration (0.007%) was excellent, with a high contrast ratio (241) and transmittance difference ($\Delta T = 98.57\%$), low threshold ($V_{TH} = 0.85$ V/μm) and saturation voltages ($V_{SAT} = 1.45$ V/μm), and a minimum hysteresis effect. The effects of temperature on the electro-optical properties of the DPDLC films were studied. Absorbance analyses were conducted using ultraviolet–visible spectrophotometry to complement the transmittance analyses. Dielectric relaxation spectroscopy was employed to measure relaxation frequency/time of DPDLC films. Debye and Cole – Cole modeling were conducted to understand nature of the relaxation process. The zero values of the distribution parameter (α) for all of the DPDLC composite films confirmed their Debye type relaxation process.

1. Introduction

Polymer dispersed liquid crystals (PDLCs) are thin composite films made from a high molecular weight polymer and low molecular weight thermotropic liquid crystal (LC) using the phase separation method. The LC droplets formed by the phase separation method are mechanically and structurally stabilized by the polymer matrix. The most common applications of PDLC films include flat panel flexible displays with large areas, switchable windows, light valves, high definition spatial light modulators, optical and thermal sensors, strain gauges, color projectors, bistable reflective displays, and optical shutters that operate in normal or reverse modes [1–4].

The birefringence property of LCs is important for the operation of composite films. LCs are optically anisotropic materials with two distinct refractive indexes (RIs). In the absence of an electric field, directors present in micron–sized LC droplets are randomly oriented in the polymer matrix. When light falls on the film, it is scattered and refracted multiple times on the polymer–LC interfaces and the film appears opaque as a result. After the application of suitable electric field, the LC directors are aligned along the direction of the external electric field. Under these conditions, for an LC with positive birefringence ($\Delta n > 0$), the ordinary RI (n_o) of the LC matches with the RI of the polymer matrix (n_p), whereas for an LC with negative birefringence ($\Delta n < 0$), the extra–ordinary RI (n_e) of the LC matches with n_p, thereby leading to the transparent appearance of the film [5–7]. In addition to the optical anisotropy of LC, the excellent performance of PDLC composite films is influenced by many factors, such as the dielectric anisotropy of the LC, polymer-LC interactions, the size, shape, and structure of the LC droplets, and the match/mismatch of the RI between the LC and its host polymer, as well as between different LC droplets. The main role of the polymer matrix is to support the LC droplets and provide flexibility and mechanical strength to the device, but physical interactions with the polymer can affect the formation and configuration of meso-phases. In some cases, the spherical LC droplets may be deformed because of the polymer matrix. Parameters such as the physical properties of the constituents (e.g., conductivity, RI, and viscosity), their proportions, solubility and diffusion of LC in the monomer/pre-polymer, phase

* Corresponding author.,
E-mail addresses: rr.deshmukh@ictmumbai.edu.in, rajedeshmukh@rediffmail.com (R.R. Deshmukh).

https://doi.org/10.1016/j.jpcs.2021.110363
Received 22 January 2021; Received in revised form 29 August 2021; Accepted 30 August 2021
Available online 31 August 2021
0022-3697/© 2021 Elsevier Ltd. All rights reserved.

Table 1
Properties of Materials Used in this Study.

Material Used	Properties
Liquid Crystal HPC850100-100	$n_o = 1.518$, $n_e = 1.768$, $\Delta n = 0.250$, $\epsilon_{II} = 39.9$, $\varepsilon_\perp = 8.1$, $\Delta\varepsilon = 31.8$, $T_{S\text{-}N} < -40\ ^\circ C$, $T_{N\text{-}I} = 73\ ^\circ C$, Viscosity = 65 cSt
Dichroic Dye Disperse Orange-25	Appearance = Orange Solid Powder; Melting Point = 170 °C<br>Maximum Absorbance at Wavelength λ_{max} = ~457 nm
Monomer Unit NOA-65	Viscosity = 1200 cSt, Refractive Index = 1.524, Absorbance = 350–380 nm.

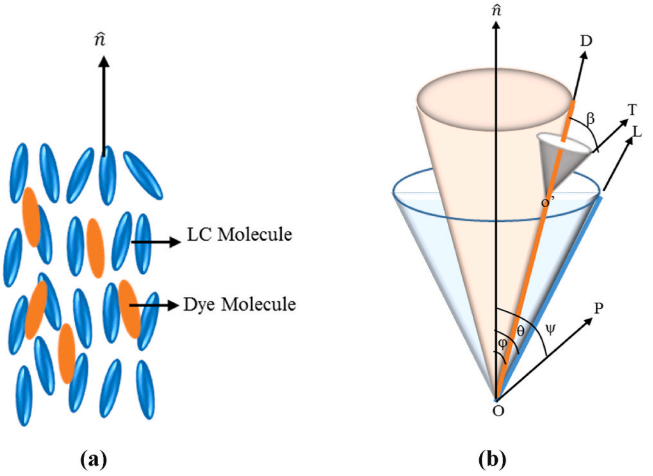

Fig. 1. Dye and LC Molecules with Cylindrically Symmetric Structures. (b) Geometrical Relationships of Dye with its Transition Moment, LC, and Polarized Light.

separation method employed, dopants and temperature determine the morphology of the droplets and electro-optical characteristics of PDLC films [8–15]. The dopant and temperature affect the morphology of the LC droplets and thus the contrast ratio (CR) and transmittance of the composite films [16,17]. Therefore, in the present study, we used a dichroic dye to dope a nematic LC and form polymer-LC composite films, and the properties of the composite systems were determined.

Dichroic dye molecules are long and rigid molecules that exhibit a strong transition moment along one molecular axis, thereby leading to the anisotropic absorption of light, and they tend to align along the director when dissolved in an LC solvent. This phenomenon where guest dye molecules align with the host LC is known as the guest–host interaction. The color intensity of the system can be manipulated by only controlling the LC rotation because dye molecules tend to align with the LC molecules. The two types of dichroic dyes according to the form of dichroism are pleochroic or positive and negative dichroic dyes [18–24]. Pleochroic or positive dichroic dyes are generally used with LCs because they exhibit higher order parameters and dichroic ratios compared with their counterpart negative dichroic dyes. Dyes typically have a narrow absorption spectrum and the wavelength corresponding to the peak is denoted as λ_{max}. The visible color of a dye is attributable to the light reflected by the dye or the complementary color.

The effects of doping an LC material with dye on the morphological, electro-optical, thermo-electro-optical, and dielectric properties of the resulting polymer–LC composite films were investigated in the present study. The responses of the composite films to the different applied electric fields and temperatures were also studied using theoretical and modeling techniques.

2. Experimental and analytical work

2.1. Materials and methods

The performance of any device depends on its properties, and thus the methods and materials which employed for its production. Selecting appropriate materials is very important for the optimum performance of display devices. Determining the different parameters for the constituent materials helps to select appropriate materials for a particular device. Materials are selected based on their individual chemical and physical properties, as well as their compatibility with the other component materials. In this study, a nematic LC, dye, and monomer unit were used to prepare efficient polymer–LC composite films. The chemical and physical properties of these materials are described in the following.

2.1.1. LCs

Various properties must be considered when selecting LCs for optical devices. The LC state occurs only when the LC molecules exhibit an approximately anisotropic form. Therefore, a structurally, optically, and dielectrically anisotropic, thermotropic nematic LC was used in this study. In order to prepare polymer–LC composites, the ordinary RI of the LC (n_o) should match with the RI of the polymer (n_p). The viscosities of both phases must support each other when preparing their homogenous mixture. The nematic–isotropic temperature ($T_{N\text{-}I}$) of the LC should not be excessively high because the properties of the monomer/polymer units may be changed/deformed at high temperatures. After considering all of these factors, commercially available nematic LC HPC850100-100 was obtained from HCCH Jiangsu Hecheng Display Technology Co. Ltd, China. Some important parameters of LC HPC850100-100 for LC devices are presented in Table 1.

2.1.2. Dichroic dye disperse orange 25

When an LC is doped with a small quantity of dichroic dye, the dye molecules tend to align in parallel with the LC molecules on average. However, in actual practice due to fluctuations, the LC molecules and dye molecules both deviate from the director's direction $\hat{n}$ to different extents depending on their molecular structures. Fig. 1(a) and (b) show schematic and geometrical representations of the LC and dye molecules, respectively. Fig. 1(b), clearly demonstrates that the long molecular axes of the LC molecule L, dye molecule D, and electric vector P form angles of θ, ϕ, and ψ, respectively, with the director $\hat{n}$, which are responsible for the order parameters of the dye and LC, as well as the dichroic ratio. Dyes used to dope an LC medium must have a high order parameter ($S_D = \frac{1}{2}\langle 3\cos^2\phi - 1\rangle$), high dichroic ratio ($D = \frac{A_{||}}{A_\perp}$, where $A_{||}$ and $A_\perp$ are the absorbances at $\psi = 0^\circ$ and $\psi = 90^\circ$ respectively), good stability and excellent solubility in the LC and compatibility with the LC but not the polymer matrix [24–27]. The order parameter of a dye is an indication of how well the dye molecules aligns itself with the LC. The dye order parameter is directly proportional to the length of a dye molecule and inversely proportional to its width. The dye order parameter follows the order parameter of the host LC over the entire temperature range, and thus for a color display, the choice of the dichroic mixture depends upon the compatible LC host, which must have a high and sustainable order parameter over the operating temperature range of the device. Doping with dichroic dyes alters the clearing temperature of the mixture by 0.5–5 °C depending upon its compatibility with the host LC.

Photostability is one of the important parameters that need to be considered in LC devices. Many classes of dyes decompose or fade due to photoprocesses. The photostability of azo dyes depends on the wavelength corresponding to the maximum absorption designated as λ_{max}. A dye with high photostability generally improves the stability of the whole system.

The solubility of a dye depends mainly on its structure and the host entity. Asymmetrically substituted dichroic dyes are more soluble in LCs

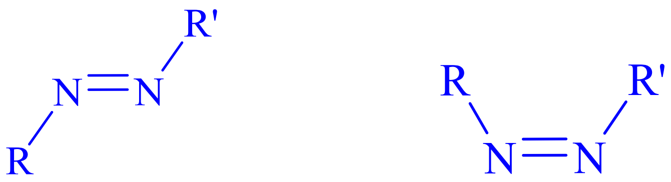

(a) (b)

Fig. 2. General chemical structures of azo dyes: (a) Trans form and (b) cis form.

than their symmetric analogues [28]. The insertion of lateral alkyl substituents groups in dichroic dye molecules improves the solubility of dyes in LCs. A mixture of dichroic dyes with similar absorption spectra usually has solubility greater than that of a single dye. Doping an LC with dye, increases the viscosity of the host (LC), thereby decreasing the working temperature of the device. The RI, elastic constants, and dielectric anisotropy of the dye–LC mixture are basically those of the LC host [19,22,24,25,28–33].

Considering all of these properties, an "azo" dye was selected from the two types of dichroic dyes comprising anthraquinone and azo dyes. An azo dye is a synthetic organic dye that contains a minimum of one azo group (-N=N-) but it may contain two (diazo), three (triazo) or more (polyazo) azo groups as part of the molecular structure. In addition, at least one but usually two aromatic residues are attached to the azo group. Azo dyes occur in the more stable *trans* form shown in Fig. 2(a) rather than the *cis* form depicted in Fig. 2(b). Both nitrogen atoms are sp^2 hybridized so that the carbon nitrogen bond angles are 120°. Azo dyes possess cylindrical shapes similar to LC molecules and they have high extinction coefficients. The charge transfer absorption band excitation occurs in the direction of the molecular axis, which is responsible for their color and they are available in almost all shades [30,32,34]. Fig. 3(a) shows the structural formula for the commercially available dichroic azo dye "disperse orange 25," which we used in this study. This dye was obtained from Sigma Aldrich (Madison, WI, USA). The chemical name for Disperse Orange 25 is 3-[*n-ethyl*-4-(4-nitrophenylazo) phenyl amino] propionitrile. The molecular structure (Fig. 3(a)) and absorption spectrum for the dye (in chloroform) (Fig. 3(b)) are shown in Fig. 3. The physical properties of disperse orange 25 are summarized in Table 1.

2.1.3. Monomer unit NOA-65

In the present study, the PDLC film was prepared with the ultraviolet (UV) curable monomer called NOA-65, which was purchased from Norland Products Inc., Cranbury, USA. NOA-65 is a colorless, optically transparent, chemically stable and photocurable liquid mixture of Mercapto-ester and Acrylate Monomer. It has a broad absorption peak in the range of 350–380 nm. When NOA-65 is exposed to UV light for curing, it transforms into a gel in seconds and then become tough and resilient within minutes. The cured film of this monomer is very flexible, thereby making it a promising candidate for use in flexible PDLC displays. The physical properties of NOA-65 are summarized in Table 1.

2.1.4. Sample cell assembly for Dye–Doped PDLC (DPDLC) films

The following procedure was employed to prepare DPDLC film using disperse Orange 25+ HPC850100-1*00* + *N*OA-65, which we denote as "O*00* N."

1. First, we prepared empty cells using two indium tin oxide (ITO) coated transparent glass plates with resistance of 12 Ω sq. and dimensions of 2.25 cm × 1.5 cm. The conducting sides of the two ITO-coated glass substrates were placed facing each other and the thickness of the sample cell was maintained at 23 μm by inserting polyethylene terephthalate film spacers at the opposite sides of the glass plates. A specially designed jaw was used to hold the assembly and apply equal pressure everywhere on the cell. The cell was then sealed by applying a UV-curable optical adhesive (UV Sealant 91, Norland, USA) along the outer edges. The cell was placed into a UV chamber for the curing process. An image of an empty sample cell is shown in Fig. 4.
2. Next, the dye was dissolved in the LC at various concentrations comprising 0%, 0.007%, 0.015%, 0.0625%, 0.125%, 0.25%, 0.5% and 1%. The different combinations were heated to the nematic isotropic temperature ($T_{N\text{-}I}$) of the LC to ensure the appropriate mixing of the components.
3. These dye–LC mixtures were combined with NOA-65 at an optimized proportion of 40/60 wt/wt%, respectively. The dye + LC and prepolymer mixture was again heated to $T_{N\text{-}I}$ of the LC and stirred continuously to ensure homogenization. The mixture was cooled and then used to fill a prefabricated empty cell at the optimum temperature of 42 °C by capillary action. Filling the cell at a higher temperature caused most of the LC droplets to coalesce and form large droplets, which set within polymer matrix during photopolymerization. The resultant film was transparent even in the OFF state, which reduced the efficiency of the DPDLC film. However, when the empty cell was filled at temperatures below 42 °C, the viscosity of the mixture increased and the flow rate decreased. Thus, the material filled the cell in a heterogenous manner and some air bubbles were trapped within it and this DPDLC film exhibited poor display properties as a consequence.
4. Finally, the sample cell assembly was placed in a UV chamber for 30 min, to allow complete photopolymerization (curing). The distance between the UV source and sample was kept constant at about 5 mm. Light was provided at a wavelength of 365 nm using an 8 W UV lamp tube with a length of 30.3 cm and diameter of 1.6 cm.

2.2. Operating principle of DPDLC composite films

In order to prepare DPDLC films, the polymer-induced phase separation method was employed to obtain well dispersed but phase separated (from polymer matrix) dye-doped LC droplets. The absorption and scattering of light by the DPDLC films could be controlled or tuned by applying a suitable electric field to modulate the LC molecules. The strong transition moment of the dichroic dye molecules was controlled

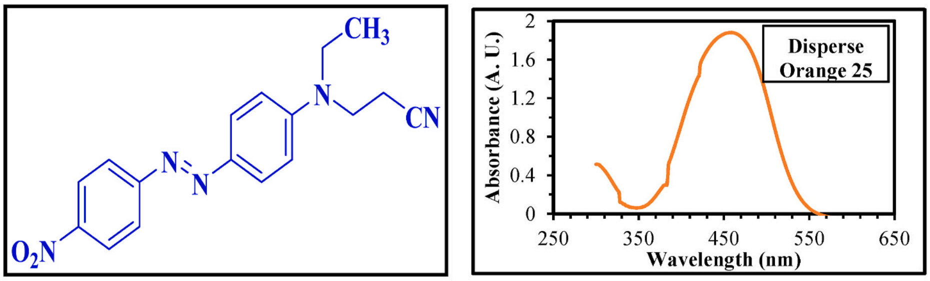

(a) (b)

Fig. 3. (a) Molecular structure, and (b) absorption spectrum of dichroic azo dye disperse orange 25.

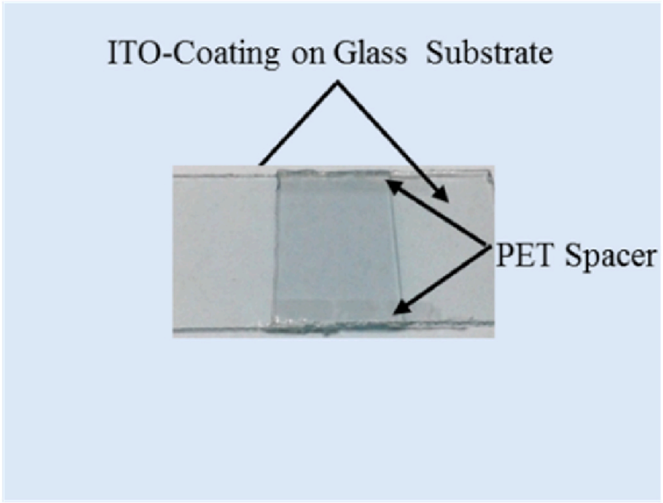

Fig. 4. Empty sample cell assembly.

by the symmetry axis of the LC molecule in the absence or presence of an external electric field [35–38]. In the absence of an electric field in the, "OFF state," the dye molecules were oriented in a random manner with the LC directors. The incident light was scattered from the polymer–LC interfaces to increase the optical path length for light, thereby greatly decreasing the transmission of light. In the presence of an electric field, in the "ON state," the field exerted a large torque on the dipolar dye–LC molecules to align the dye molecules and LC directors in the direction of the field, thereby enhancing the transmission of light. Schematic representations of the random orientation of LC directors in the OFF state and alignment of LC directors in the ON state were provided in previous studies [39–41].

2.3. *Morphological analysis*

Morphological analysis of the polymer–LC composite films was essential for understanding electro-optic effects. Therefore, polarizing optical microscopy (POM) and scanning electron microscopy (SEM) were employed to determine the configuration and orientation of the LC droplets.

2.3.1. *POM*

The POM technique is non-destructive and it provides flexibility for recording *in-situ* images under a changing electric field. POM (Olympus BX53, Singapore) was applied to understand the effects of dye doping on the morphology and configuration of the LC droplets. POM images were captured to investigate the *in-situ* effect of an applied electric field on the configuration of the dye doped LC droplets and their alignment. A charge coupled device camera interfaced with a computer was linked to the POM system to record high quality images. All samples were examined under crossed polarizers using a10 × objective lens.

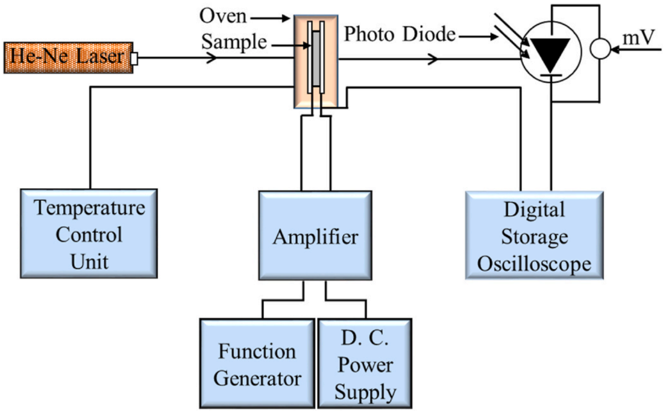

Fig. 5. Experimental Set-up used to determine the Electro-optical and Thermo-Electro-optical Properties of DPDLC Films.

2.3.2. *SEM*

POM has limited magnification power, so SEM (Tescan MIRA 3 Model, USA) images were also captured to characterize the microstructures created in polymer matrix after the removal of LC material. SEM imaging of DPDLC films is a destructive technique. In order to record SEM images, it was necessary to remove the transparent conducting glass electrodes that covered both sides of the composite film. It is well known that the images obtained in the presence of LC material in a polymer matrix exhibit poor contrast because the SEM technique is not capable of distinguishing between the two phases, i.e., LC and polymer. Hence, to improve the contrast, the composite films were placed in methanol for 48 h at room temperature to extract the LC material from the films. Methanol was employed because it is not a solvent for the polymer but it is a solvent for LC. The films were then washed repeatedly around 10 times with methanol to ensure the complete removal of the LC material from polymer network/matrix. The samples were then free of the LC material and only the spongy polymeric matrix remained with voids where the LC droplets were originally located. The samples were then air dried and placed in a vacuum desiccator for 12 h to remove any remaining traces of methanol in the polymer matrix.

2.4. *Electro-optical analysis*

The electro-optical properties of DPDLC films were recorded based on the transmittance and hysteresis as a function of the applied voltage. A monochromatic ($\lambda = 632.8$ nm) coherent beam produced from a 10 mW He–Ne laser source (model 105 P Photochemical Inc., Toronto, ON, Canada) was incident onto the sample to record the transmittance and associated parameters of DPDLC films. An amplified ac electric field at frequencies ranging from 50 Hz to 1 kHz produced by a function generator was applied to the sample cell assembly to evaluate the changes in the optical transmittance as a function of the voltage. The light transmitted through composite films was recorded using a photodiode placed at a distance of 300 mm normal to the sample cells as shown in Fig. 5. The source, sample and photodiode were kept on a common optical axis. The transmittance versus voltage curves were obtained for various frequencies by increasing the voltage in steps of 10 V up to 100 V during the scan up cycle, followed by the scan down cycle with the same steps, where the temperature was constant at 25 °C. After one complete cycle, the DPDLC films were kept in the OFF state (no voltage applied) for 5 min to minimize any memory effect. Among the various frequencies tested, we found that 200 Hz was the optimized frequency (the optimization data are not presented due to space constraints) for our samples [10,42]. The graphs plotting the relationship between transmittance and voltage at different frequencies were reported previously [43].

2.5. *Thermo-electro-optical analysis*

The transmittance of the film changed with the temperature and voltage according to thermo-electro-optical analysis. The changes in optical transmittance at different temperatures were determined by placing each sample in an oven attached to a temperature sensor and display unit. The temperature of the sample was varied from 25 °C to 50 °C. The heating rate was slow and steady to ensure the accuracy of the measurements of the thermo-electro-optical properties. The temperature was increased at a rate of 2 °C/min in the experiment. A schematic illustration of the experimental set-up is shown in Fig. 5.

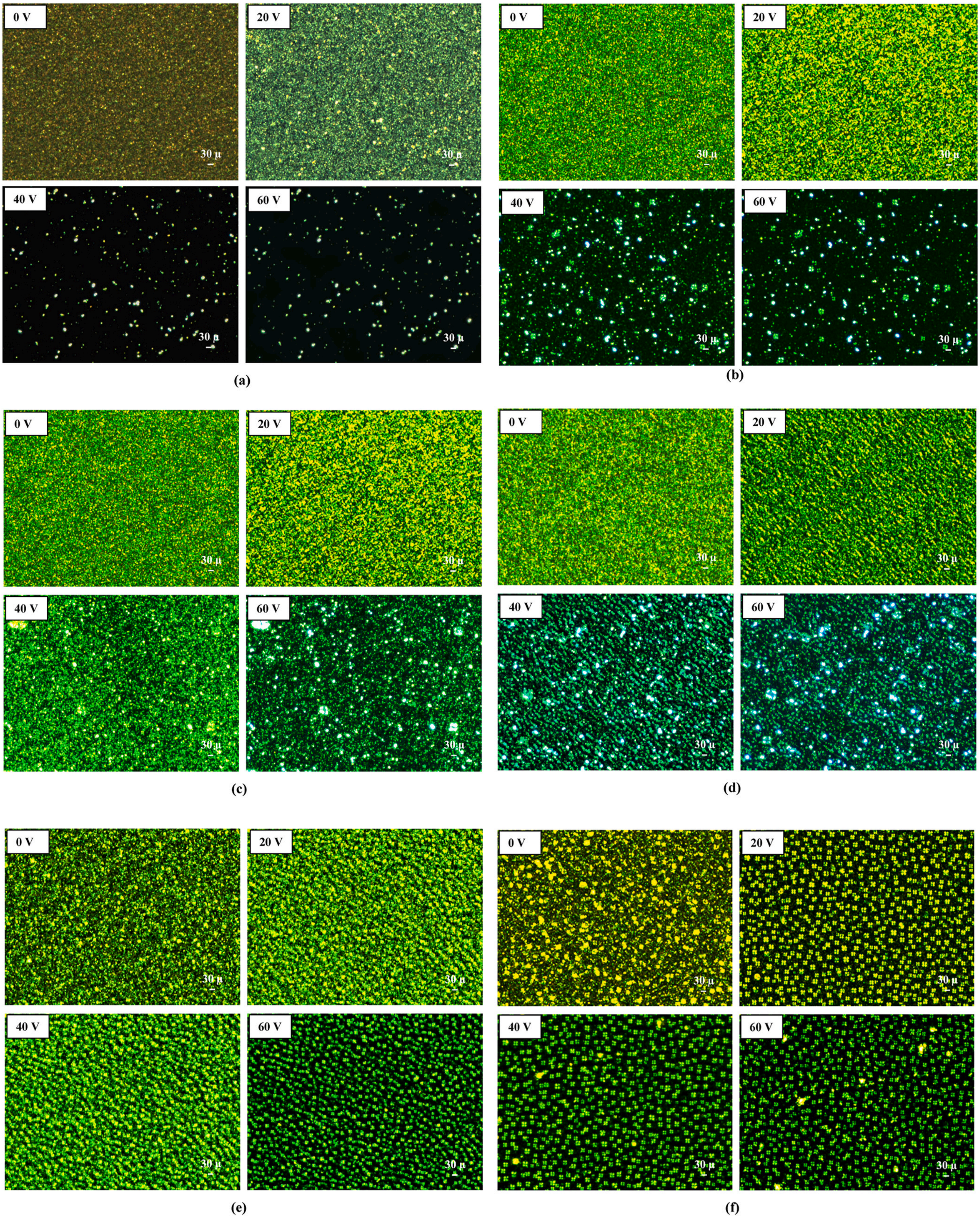

Fig. 6. Droplet structures of (a) 0%, (b) 0.007%, (c) 0.015%, (d) 0.0625%, (e) 0.25%, and (f) 1% O*OO* N DPDLC films at different voltages.

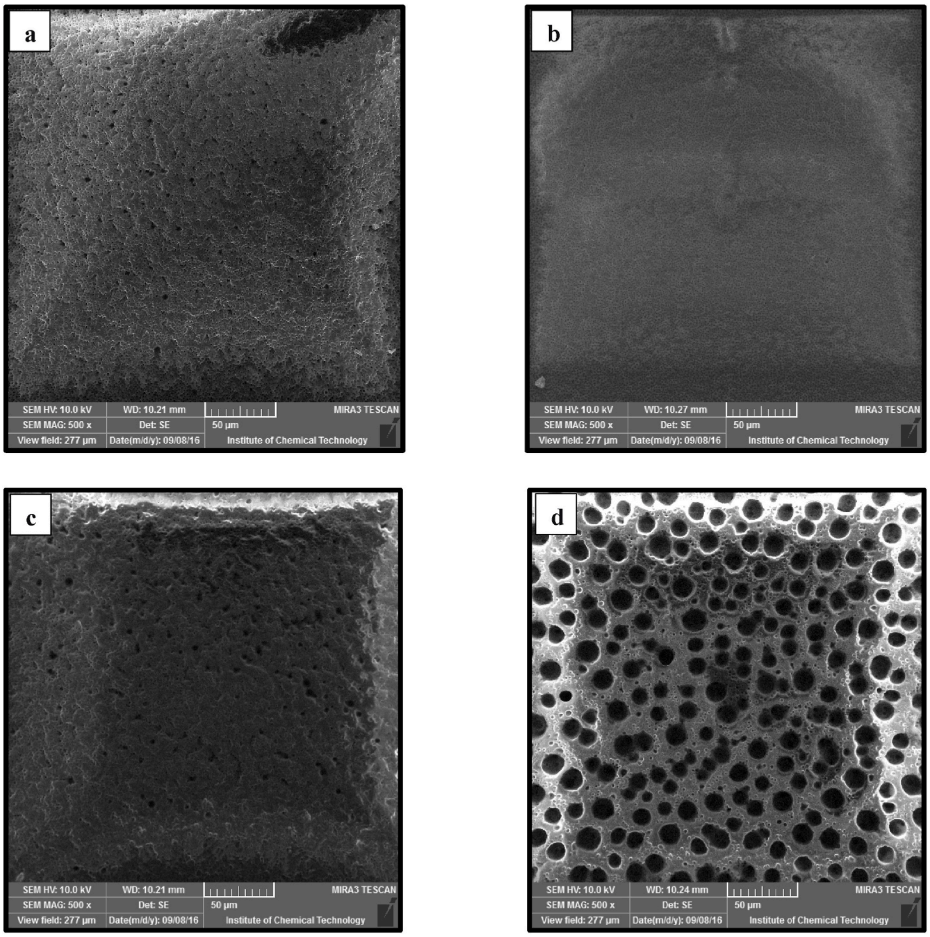

Fig. 7. Cavities formed after removing LC from (a) 0%, (b) 0.007%, (c) 0.0625%, and (d) 1% O*00* N DPDLC films.

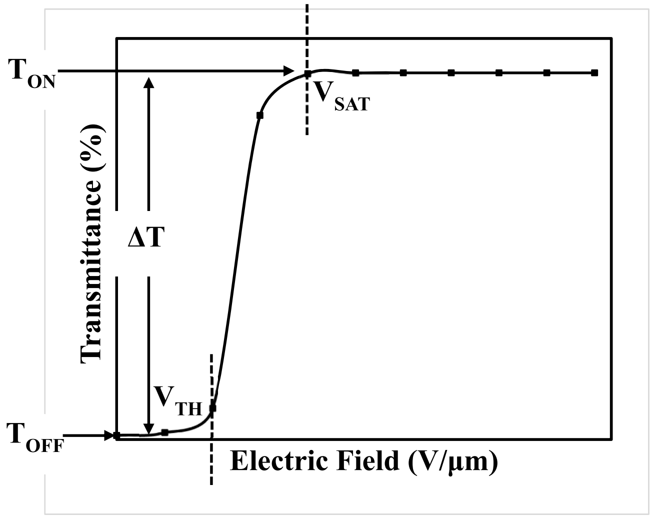

Fig. 8. Schematic representation of various parameters in the Transmittance vs Electric Field Plot.

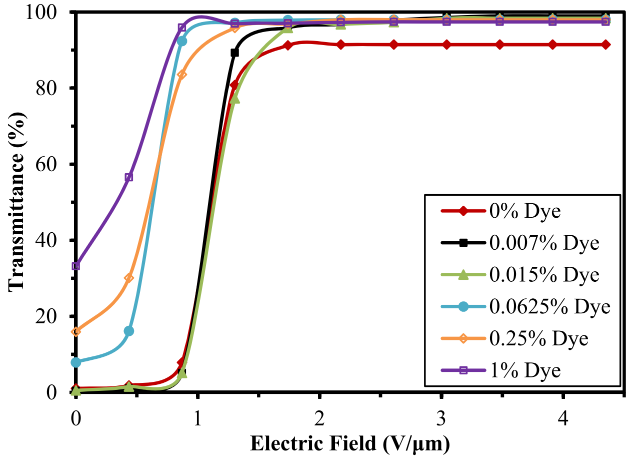

Fig. 9. Transmittance vs Electric Field Curve for O*00* N DPDLC Films with Various Dye Concentrations at a Frequency of 200 Hz and Temperature of 25 °C.

2.6. *Absorption analysis*

The transmittance of the DPDLC composite films was verified based on their absorbance characteristics. The absorbance spectra were recorded for DPDLC composite films in the visible region (400–700 nm) using an UV–Visible (UV–Vis) spectrophotometer (PerkinElmer Lambda 35, Waltham, MA, USA). The effects of an external electric field on the *in-situ* absorbance properties of the composite films were also recorded.

2.7. *Dielectric relaxation spectroscopy (DRS)*

The buildup and decay of charge polarization do not occur instantaneously following the application and removal of the field,

Table 2
Voltage–transmittance data for O*00* N DPDLC films with various dye concentrations.

Dye Conc. (wt %)	T_{OFF} (%)	T_{ON}(%)	ΔT (%)	CR	V_{TH} (V/μm)	Vsat (V/μm)
**0.0**	1.02	91.43	90.41	90	0.88	1.5
0.007	**0.41**	**98.98**	**98.57**	**241**	**0.85**	**1.45**
**0.015**	0.51	98.37	97.86	193	0.85	1.7
**0.0625**	7.86	97.96	90.1	12	0.42	1.0
**0.25**	15.52	97.96	82.44	6	0.4	1.1
**1**	33.16	97.40	64.24	3	0.4	1.0

respectively, but instead they require a finite time interval, which is known as dielectric relaxation. Thus, DRS analysis was conducted to understand the dynamics of the LC droplets confined in the polymer matrix, thereby obtaining information about the interdependent process and intermolecular interactions between the dye–doped LC molecules and polymer–LC interfaces. The dielectric properties of the composite films were determined as a function of the frequency (20 Hz–20 MHz) at various temperature (25 °C–60 °C) under a constant (1 V) bias voltage using a precision impedance analyzer (Wayne Kerr 6500B, 0–40 DCV, UK). This method is based on the fundamental concept of energy storage and subsequent energy loss due to the orientation/reorientation of LC dipoles in a changing electric field [44,45].

3. Results and discussion

3.1. Morphological analysis

POM and SEM instruments were employed to conduct morphological analyses of O*00* N DPDLC composite films.

3.1.1. POM

Fig. 6 (a), 6(b), 6(c), 6(d), 6(e), and 6(f) show the POM images of the 0%, 0.007%, 0.015%, 0.0625%, 0.25% and 1%, O*00* N DPDLC films, respectively, which were observed under crossed polarizers using a 10 × objective lens. Electric field amplitudes of 0 V, 20 V, 40 V, and 60 V were applied to each film to elucidate their electro-optical characteristics.

POM images obtained at the same magnification clearly showed the dispersion, size, and shape of the dye–doped LC droplets distributed in the polymer matrix. Fig. 6(a–f) clearly demonstrate that the droplet size increased as the dye concentration increased from 0% to 1% in the dye–doped LC. It should be noted that the droplet size was less than 30 μm even for the 1% DPDLC film, possibly because the dye molecules absorbed some of the UV radiations provided for photopolymerization and the amount of time available for droplet growth resulted in a greater droplet size. The Stokes equation indicates that the LC droplet size will increase with the viscosity. The viscosity of the dye–LC mixture

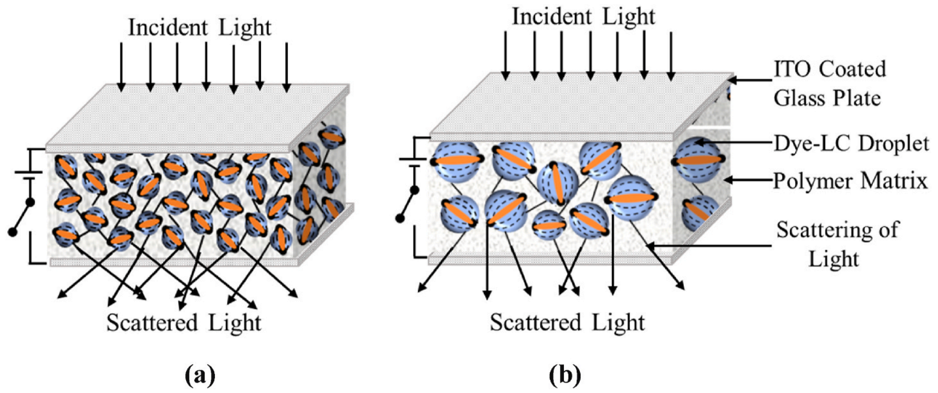

Fig. 10. Scattering from DPDLC Films containing LC Droplets with (a) Small Diameters and (b) Large Diameters.

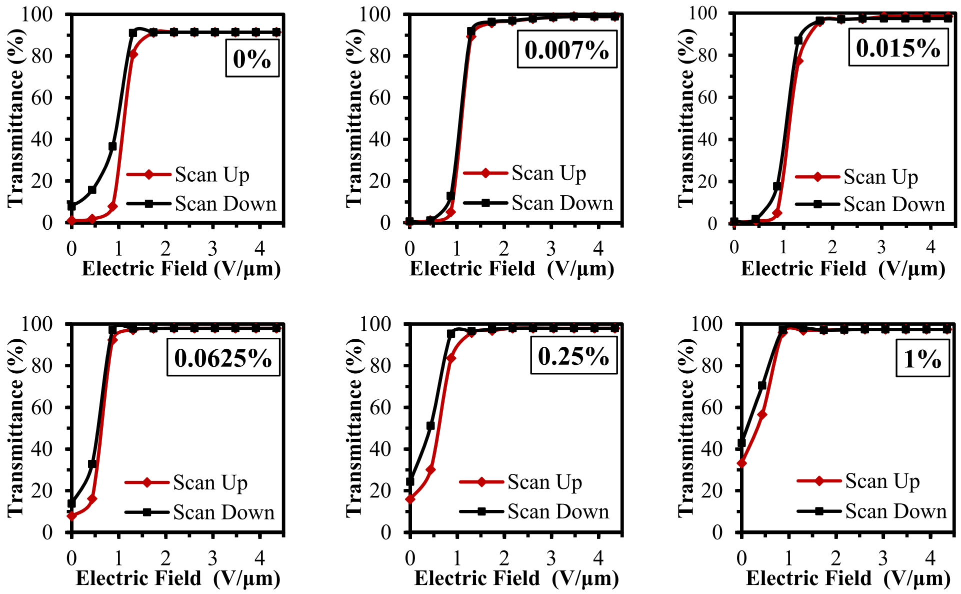

Fig. 11. Hysteresis curves for O*00* N DPDLC films with various dye concentrations.

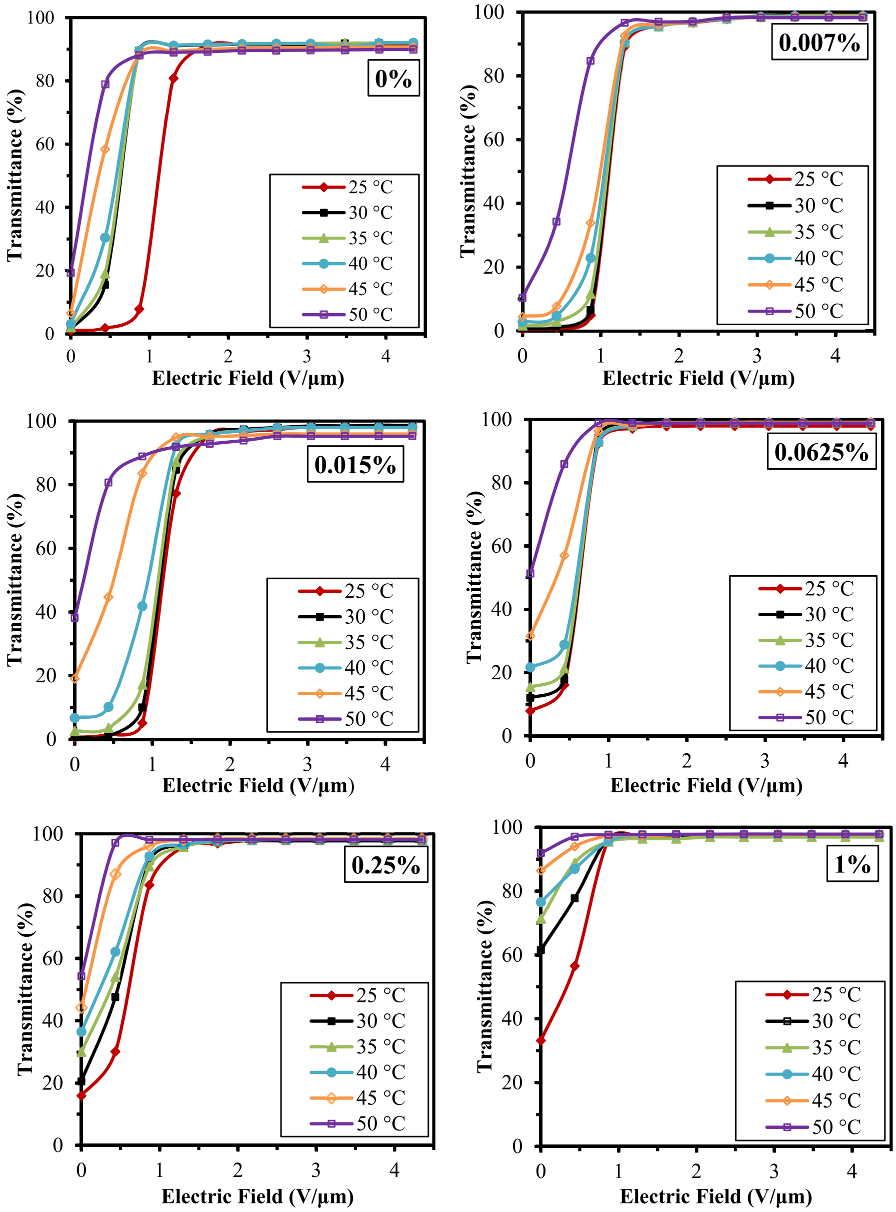

Fig. 12. Transmittance vs Electric Field Curves for O*00* N DPDLC Films with Various Dye Concentrations at Different Temperatures.

increased with the dye concentration, thereby leading to increase in the LC droplet size. The surface area of the dye–LC droplets increased, because the polymer matrix surface anchoring forces on the droplets also increased. After the application of an electric field, the small LC droplets were vertically aligned along the field, thereby leading to a dark appearance according to the POM observations, as depicted in Fig. 6a. As the droplet size increased, under a low electric field (~10 V), a considerable difference was observed in the director configuration of the LC molecules inside the LC droplets and those located at the interface of the polymer–LC droplets because the LC molecules at the interface experienced a higher anchoring energy than those inside LC droplets. When the electric field increased further (≥30 V), the LC molecules at the center were vertically aligned and they appeared dark according to POM observations, whereas the LC molecules at the polymer walls were tangentially aligned along the external electric field to form the arms of a Maltese cross, which are also known as isogyres. The different alignments of the LC molecules in the LC droplets at various voltage resulted in changes in the RI of the droplet and thus the green color of the film varied under POM [44–46,48,49].

3.1.2. SEM

Fig. 7(a), 7(b), 7(c), and 7(d) show SEM images of some representative DPDLC films with dye concentrations of 0%, 0.007%, 0.0625%, and 1%, respectively. Clearly, the SEM micrographs demonstrate that the sizes of the cavities (which originally contained LC droplets) increased from submicron to 10 µm as the dye concentration increased. The sizes of the cavities in the 0% and 0.007% DPDLC films were less than 1 µm, and 1–2 µm for those in the 0.625% DPDLC film. As the dye concentration increased further, cavity size was ~10 µm in the 1% DPDLC film. The polymer boundaries were quite thick because of the high monomer concentration (60% of the total weight).

3.2. Electro-optical properties

The electro-optical properties of O*00* N DPDLC films were studied in terms of the changes in the optical transmittance as a function of the applied electric field. The optimized frequency for the electrical field was 200 Hz at 25 °C. The morphological analyses of DPDLC films suggested that the application of an electric field minimized the free energy

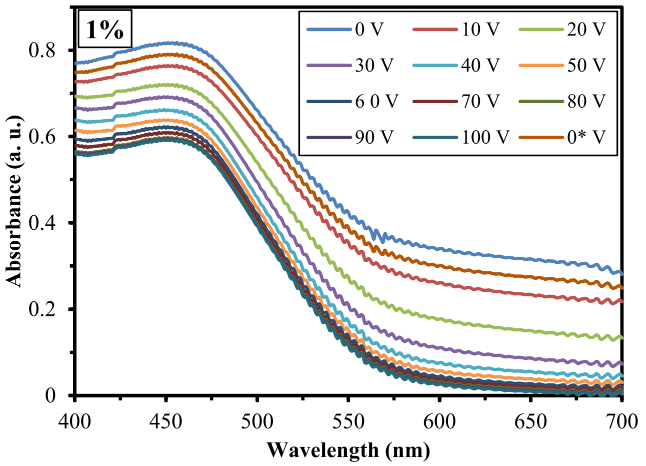

Fig. 13. UV–Vis Absorbance Curve for the 1% O*00* N DPDLC Film at Different Voltages and a Frequency of 200 Hz. (0* V is After withdrawing the Saturation Voltage).

of the LC droplets and the LC directors aligned along the direction of the applied electric field. The small amount of dye doped in the LC was also orientated along with the LC molecules, which improved the nonlinear response of the device [4,5,7,50].The rotation of LC droplet directors and associated dye molecules with the field induced nonlinear changes in the transmittance of the films.

3.2.1. Transmittance analysis

Transmittance analysis was conducted by calculating parameters comprising the OFF-state transmittance (T_{OFF}), ON-state transmittance (T_{ON}), transmittance difference (ΔT), *CR*, threshold voltage (V_{TH}), and saturation voltage (V_{ON}). A schematic plot of the transmittance versus the electric field is shown in Fig. 8 to illustrate these parameters clearly. T_{OFF} is the value of the transmittance at zero voltage. T_{ON} is the maximum transmittance value at a sufficient voltage. The electric field (corresponding voltage) required to activate the cell is the threshold voltage V_{TH}. Equation (3) is the mathematical formula used for calculating V_{TH}, but we calculated it by extrapolating the linear region method rather than with the conventional V_{10} characterization scheme. The voltage corresponding to the maximum transmittance is V_{ON}. The thickness of PDLC/DPDLC films is constant (23 μm in our study), so the terms comprising electric field and applied voltage can be used interchangeably where d is the film thickness, ρ_p and ρ_{LC} are the resistivities of the polymer and LC, respectively, $l = a/b$ is the aspect ratio, k is the elastic constant, and $\Delta\varepsilon$ is the dielectric anisotropy of the LC.

The CR, ΔT, and V_{TH} are calculated using Equations (1)–(3) respectively.

$$CR = \frac{T_{ON}(\%)}{T_{OFF}(\%)} \tag{1}$$

$$\Delta T(\%) = T_{ON} - T_{OFF} \tag{2}$$

$$V_{TH} = \frac{d}{3a}\left[\frac{\rho_p}{\rho_{LC}} + 2\right]\left[\frac{k(l^2-1)}{\Delta\varepsilon}\right]^{\frac{1}{2}}, \tag{3}$$

The dependences of the transmittance on the electric field (voltage) for DPDLC films with dye contents ranging from 0% to 1% are shown in Fig. 9. The transmittance parameters obtained from Fig. 9 are presented in Table 2.

As shown in Fig. 9, the transmission curve shifted toward the lower electric field values as the dye content increased, because the droplet size increased with the dye content and the total number of droplets in the available area of film decreased. Thus, the amount of boundaries/interfaces between the dye–LC droplets and polymer matrix decreased, resulted with less scattering, as shown in Fig. 10. Fig. 10 clearly shows that the incident light scattered more in DPDLC films containing a large number of small LC droplets (Fig. 10a) compared with the DPDLC film containing a low number of large LC droplets (Fig. 10b), and thus less electrical energy was required to overcome the anchoring forces between the LC droplets and polymer matrix in the DPDLC films containing larger droplets. Morphological analyses showed that the LC droplets were very small in the 0%, 0.007% and 0.015% DPDLC films and thus the large numbers of LC droplets present in these films were responsible for the high scattering, i.e., low T_{OFF} state transmittance. The T_{OFF} values for the 0.007% and 0.015% DPDLC films were even lower than that for the 0% DPDLC film because some absorption of light also occurred in the 0.007% and 0.015% DPDLC films in addition to scattering. The dye concentrations in these films were sufficiently small that they did not interrupt the polymerization process under UV light but they could absorb visible light. The droplet size increased as the dye concentration increased and thus the T_{OFF} value also increased. The T_{ON} values were higher for the doped films than the undoped film because the anchoring forces between the LC and polymer were weak due to the larger droplet sizes. The low value of T_{OFF} and high value of T_{ON} resulted in high *CR* and ΔT values. The *CR* and ΔT values were highest for the 0.007% DPDLC film (i.e lowest dye concentration). The electro-optical parameter comprising V_{TH} clearly depends mainly on droplet size according to Equation (3) whereas, all other of the parameters were almost constant. As mentioned above, as the dye concentration increased, the LC droplet size increased and the total number of droplets decreased, so lesser energy was needed to switch the DPDLC film. Therefore, V_{TH} was lower for the doped film than the undoped film. In the 0.25% and 1% DPDLC films, the LC droplets were too large to produce a scattering effect and thus the films were highly transparent even without an applied external field, and they are not suitable for applications. The value of V_{SAT} also decreased as the dye content increased [51]. All of these transmittance parameters are presented in Table 2. According to Fig. 9 and Table 2, the DPDLC film with the lowest dye concentration (0.007%), had lowest T_{OFF}, value and highest T_{ON} value, and thus high *CR* and ΔT values, and small V_{TH} and V_{SAT} values, and thus it was optimum film.

3.2.2. Hysteresis

In order to study hysteresis, the voltage was systematically increased from 0 V to 100 V in the scan up cycle and the transmittance of the DPDLC films increased from T_{OFF} to T_{ON}, whereas the voltage was systematically decreased from 100 V to 0 V in the scan down cycle, and thus the transmittance decreased from T_{ON} to T_{OFF}. The transmittance values were slightly higher in the scan down cycle, and thus the same path was not followed as that in the scan up cycle, and thus hysteresis occurred. A polymer–LC composite film should have a minimum hysteresis value for practical applications. Hysteresis depends on the rate at which the voltage is applied and removed. In the same composite film, the hysteresis value is small for slowly varying voltages (time period of seconds) compared with rapidly varying voltages (time period of microseconds) [23].

The hysteresis behaviors of all the O*00* N composite films with various dye concentrations are shown in Fig. 11, which demonstrates that hysteresis was not detected at higher voltages in the scan down cycle because the LC directors in the droplets persisted in a similar orientation state under the higher electric field. However, when the applied field was lowered further, the LC directors started to reorient and generate a hysteresis effect because the application and removal of the electric field created a significant difference in the alignment/orientation of the LC director's at the polymer–LC interface and inside the dye–doped LC droplets [52]. The orientation/reorientation of the LC directors depended on the LC/polymer compatibility and polarization induced at the polymer–LC and LC–LC interfaces, which determined the distribution of the relaxation time [53]. Hysteresis might have occurred because of the electric charges that remained even after the removal of

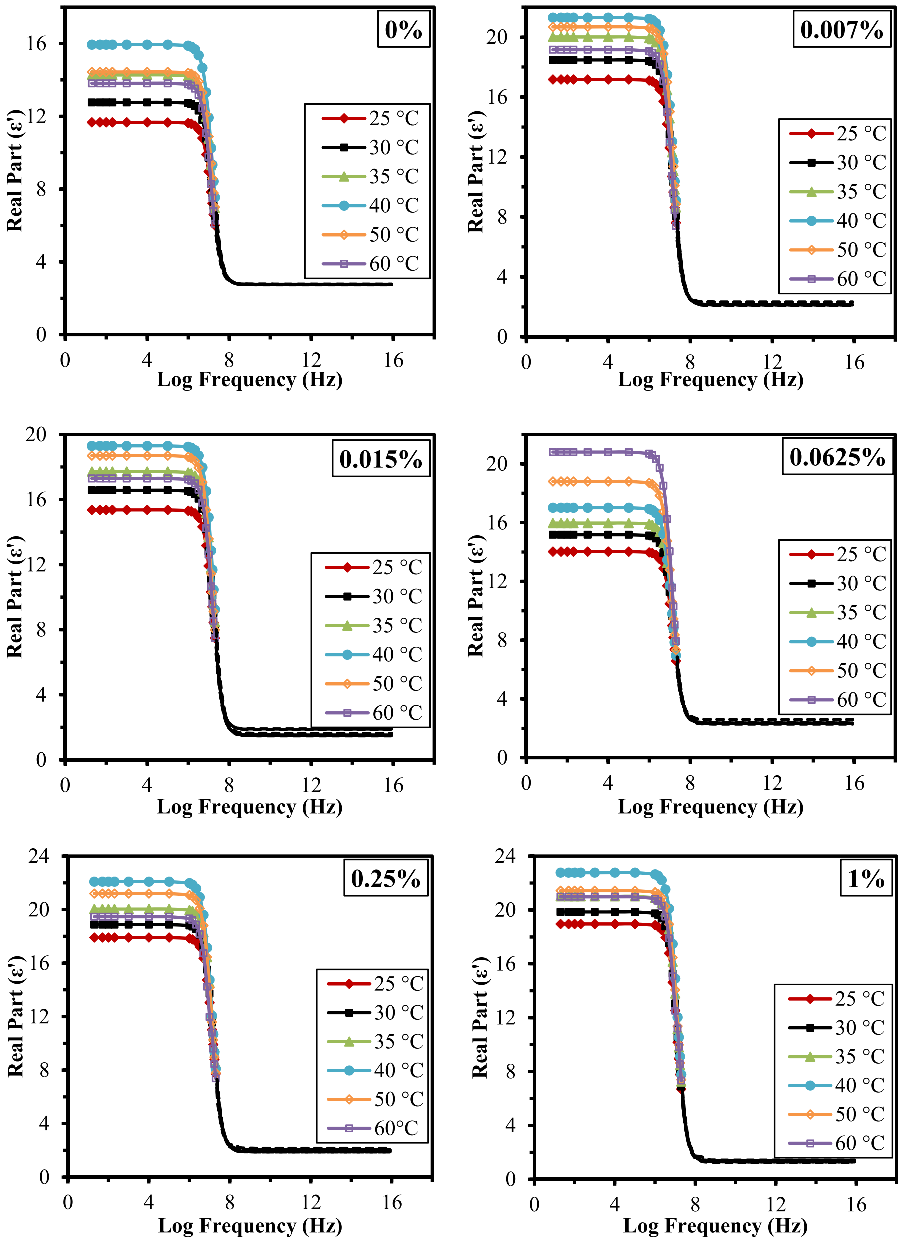

Fig. 14. Frequency dependence of the real part of the complex dielectric constant for O*00* N DPDLC films with various dye concentrations at different temperatures.

the field. These residual electric charges served as a capacitor during the scan-down cycle [42,54]. The depolarization of charges and defect structures within LC droplets may sometimes result in hysteresis. We observed that the 0% DPDLC film exhibited hysteresis, which was reduced after the addition of a small amount of dye (0.007% and 0.015% DPDLC films). We suggest that the azo dye molecules induced a positive torque on the LC droplets to enhance the orientation/reorientation mechanism, thereby reducing the hysteresis effect. Further increasing the dye content increased the amount of defect structures in the LC droplets and greater hysteresis occurred [51,52,55–57].

3.3. Thermo-electro-optical characteristics

Thermotropic LCs are temperature–sensitive materials, and their phase changes as a function of the temperature. RI mis-matching–matching for the polymer and LC is responsible for the opaque-transparent states of PDLC devices and it is also temperature dependent. The birefringence ($\Delta n = n_e - n_o$) should be as high as possible for an efficient PDLC device. The value of Δn decreases as the temperature increases, and thus the value of T_{OFF} increases with the temperature even without an applied field, as shown in Fig. 12. Fig. 12 illustrates the change in the optical transmittance for various O*00* N DPDLC films according to an applied electric field with a frequency of 200 Hz in the temperature range of 25–50 °C [46,58–60]. As the temperature increased, T_{OFF} also increased and the *CR* and ΔT values decreased. The viscosity of LC–polymer matrix decreased as the temperature increased, thereby facilitating the orientation of the LC droplet directors along the direction of the externally applied field at relatively low voltages. Hence, T_{ON} was achieved at lower voltages. The values of V_{TH} and V_{SAT} also decreased as the temperature increased, which can be explained by Equation (3) because V_{TH} is proportional to $\left(\frac{k}{\Delta\varepsilon}\right)^{1/2}$ with $k \sim S^2$ and $\Delta\varepsilon \sim S$, where the S is order parameter of LC. Thus, the decrease in the value of S as the temperature increases leads to decrease in V_{TH} [46,61,62].

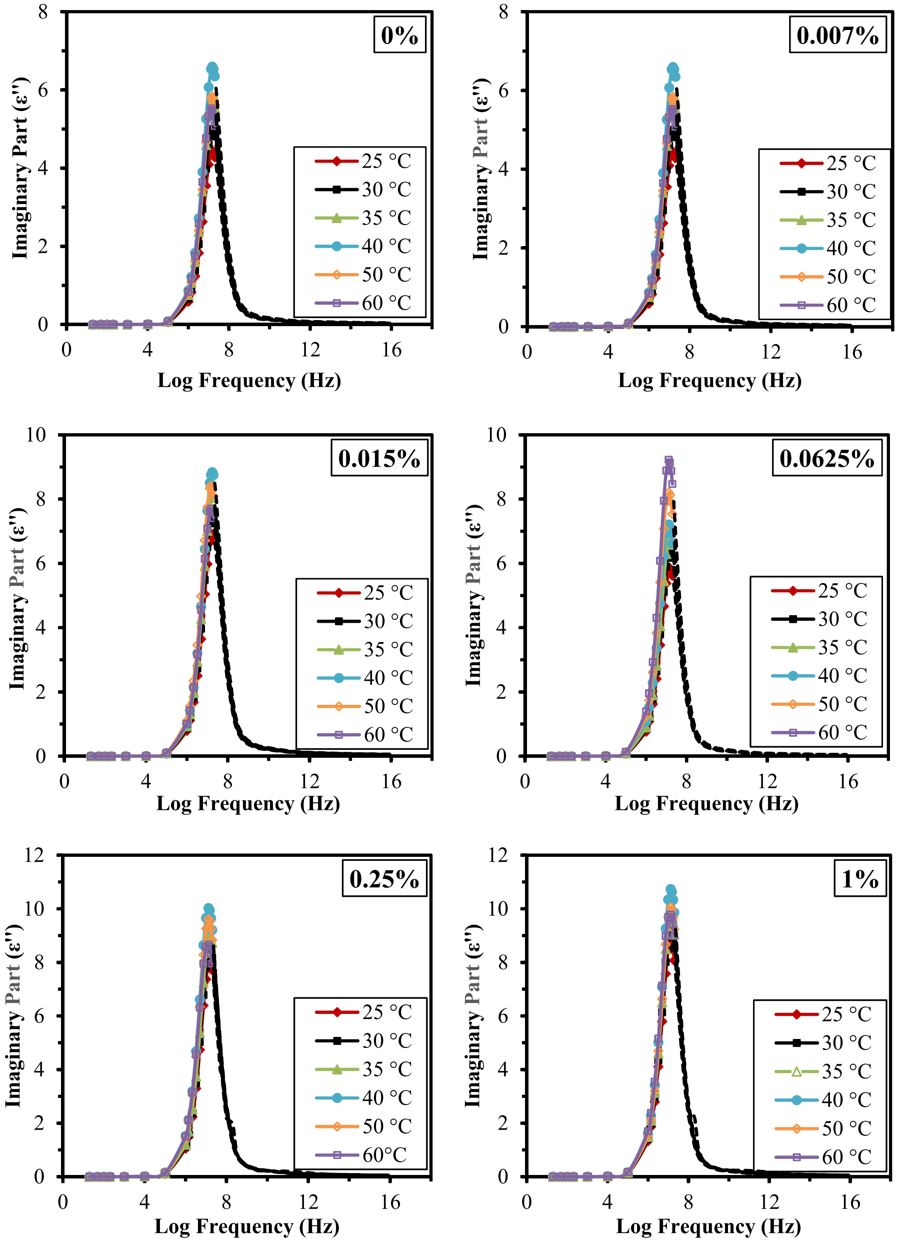

Fig. 15. Frequency dependence of the imaginary part for the complex dielectric constant *O00* N of DPDLC films with various dye concentrations at different temperatures.

3.4. Absorbance characteristics

The absorption characteristics of the DPDLC films were recorded using an UV–Vis spectrophotometer to understand the orientation and dispersion of the dichroic dyes in the LC, and thus the polymer–LC composite film. Fig. 13 shows the absorbance spectra obtained for the 1% DPDLC film over the wavelength range from 400 nm to 700 nm under an applied electric field. At 0 V, the absorption maximum (λ_{max}) for the DPDLC film was observed at ~ 455 nm. The anisotropic absorption of light with a particular wavelength by a dichroic dye molecule depends on the angular velocity of orientation, which then affects the internal dipole–dipole interactions between the nematic LC droplets and excited dichroic dye molecules. These excited dye molecules possess angular momentum. The total angular momentum is conserved by the rotation of LC directors in the direction opposite to the dye molecules [41,63–65].

The LC dispersed dye molecules are randomly oriented with respect to the unpolarized incident light, so almost every vibrating plane of light is absorbed, thereby resulting in higher absorbance in the absence of an electric field (OFF-state). Among the randomly oriented dye molecules (in the OFF-state), the molecules where the long axis is perpendicular to the direction of the propagation of light will absorb relatively more light.

After the application of an electric field the randomly aligned LC molecules and associated dye molecules tend to align in the direction of applied electric field, and thus the dye molecule orients away from its high absorption axis. The weak absorbance under a higher electric field confirmed the higher order alignment of the dye doped LC droplets in the direction of the external electric field, thereby agreeing with the transmittance findings [66]. At higher fields (above 60 V) most of the LC droplets were aligned and a nominal change was observed in the absorption spectra. After removing the electric field, some LC droplets still remained aligned, and thus the plot for 0* V (after withdrawing the saturation voltage) did not coincide with that for 0 V, as shown in

Fig. 13, because the induced interfacial polarization and residual electric charge were not removed even when the field was switched off during the scan down cycle, and thus the relaxation time lagged behind the change in the voltage. These findings are in good agreement with the hysteresis effect described above [43].

3.5. DRS

DPDLC composite films are assumed to be heterogeneous dielectric systems. DRS analysis is a useful technique for understanding the intermolecular interaction between the dye molecules and LC droplets in a polymer matrix with respect to a time–dependent electric field. The sample cell assembly was analogous to a parallel plate capacitor, and thus the following formula was used to calculate the relative dielectric constant [67,68]:

$$\varepsilon_r = \frac{C_p}{C_0} = \frac{C_p \times d}{\varepsilon_0 \times A} \tag{4a}$$

where, C_p is the parallel capacitance, d is the thickness of the composite film, ε_0 is the free space permittivity, with a value of 8.854×10^{-12} F/m, and A is area of the electrode [69,70]. The dielectric permittivity can be expressed as a function of the angular frequency (ω) of an applied electric field. The complex dielectric constant of a DPDLC film can be expressed as:

$$\varepsilon^*(\omega) = \varepsilon'(\omega) - i\varepsilon''(\omega), \tag{4b}$$

where $\varepsilon'(\omega)$ and $\varepsilon''(\omega)$ are real and imaginary parts of the complex dielectric constant, respectively [70–73], which are associated with the stored and dissipated energy. The stored energy is referred to as dispersion whereas the dissipated energy is the absorption of the system. The amount of energy dissipated due to an oscillating field was measured using the loss tangent expression (Equation (5)) [45,71,72].

$$tan\delta = \frac{\varepsilon''(\omega)}{\varepsilon'(\omega)} \tag{5}$$

3.5.1. Debye model

Single relaxation is a characteristic of Debye type relaxation and the time required for this relaxation is known as the Debye relaxation time (τ), which is inversely related to the relaxation frequency (*f*). The dissipation factor is maximized at the relaxation frequency. The relationship between the relaxation frequency *f* and relaxation time τ is as follows.

$$\tau = \frac{1}{\omega} = \frac{1}{2\pi f} \tag{6}$$

The expressions for the dispersion and absorption of a single relaxation as a function of the angular frequency (ω) and relaxation time (τ) are as follows.

$$\varepsilon'(\omega) = \varepsilon_\infty + \frac{(\Delta\varepsilon)}{1+\omega^2\tau^2} \tag{7}$$

$$\varepsilon''(\omega) = \frac{(\Delta\varepsilon)\omega\tau}{1+\omega^2\tau^2}. \tag{8}$$

Debye type relaxation is normally expressed in terms of the complex dielectric constant $\varepsilon^*(\omega)$ of a medium. After substituting the values from Equations (7) and (8) into Equation (4), we obtain:

$$\varepsilon^* = \varepsilon_\infty + \frac{(\Delta\varepsilon)}{1+i\omega\tau}, \tag{9}$$

where $\Delta\varepsilon$ is the dielectric strength of the material, which provides information about the voltage that a material can withstand before breakdown occurs:

$$\Delta\varepsilon = \varepsilon_s - \varepsilon_\infty, \tag{10}$$

where ε_s and ε_∞ are the values of the relative dielectric constant at a frequency of 20 Hz and at the relaxation frequency *f*, respectively, which can be obtained from the experimental relaxation spectrum [73–76]. Graphs plotting the frequency versus the real and imaginary parts of the complex dielectric constants for the DPDLC films with different dye concentrations at different temperatures and a biasing voltage of 1 V are shown in Figs. 14 and 15, respectively. When the applied frequency was less than the relaxation frequency, the dipoles could maintain pace with the alternating field and contribute to the total polarization. At this stage, the dissipated energy (ε'') was proportional to the applied frequency. After further increasing the frequency but still below the relaxation frequency, ε'' continued to increase but ε' decreased because of the phase lag between dipole alignment and the applied field. Above the relaxation frequency, the field alternations were so rapid that the dipoles could not maintain the pace with the alternating field, and thus the orientation polarization ended and both ε' and ε'' dropped off. Figs. 14 and 15 show that the positions (log frequency) of the peaks in the spectrum for ε'' were at the same frequency at which ε' changed. In practice, the single relaxation time mechanism is not sufficient to understand the dynamics of materials, and thus the distribution of the relaxation times concept was introduced [41].

Initially, both the real and imaginary components of complex dielectric constant increased when the temperature increased from 25 °C to 40 °C. The increase in temperature relaxed the intermolecular interactions due to the orientation–reorientation process for molecular dipoles, thereby increasing the complex dielectric constant components. However, further increasing the temperature perturbed the dipoles because thermal agitation became dominant at this stage, and thus the values of the complex dielectric constant components started to decrease [77].

3.5.2. Cole–Cole model

The Cole–Cole equation is a relaxation model used to describe secondary relaxations and it is given by the following equation:

$$\varepsilon^*(\omega) = \varepsilon'(\omega) - i\varepsilon''(\omega) = \varepsilon_\infty + \frac{\Delta\varepsilon}{1+(i\omega\tau)^{1-\alpha}}, \tag{11}$$

where, α is the distribution parameter and τ is the average relaxation time. All of the other terms are the same as those described above. It would be difficult to analyze experimental data based on Figs. 14 and 15, so in order to qualitatively evaluate the distribution of the relaxation time, a Cole–Cole plot was drawn (Fig. 16) in an Argand plane between the real part ε' and imaginary part ε'' of the complex dielectric constant. The values of ε' and ε'' were fitted to form a semicircle, which is a characteristic of relaxation behavior. α can be expressed in terms of the angle ϕ between the arc radius and ε'-axis as shown in Fig. 16, and it is given by Equation (12) [78–80].

The distribution parameter α gives information about the breadth of the relaxation time and it can take a value between 0 and 1in order to describe different spectral shapes. If the center of the semicircle lies on the ε'-axis then the value of the distribution parameter (α) is zero and the system exhibits Debye type relaxation. If the center is below the ε' -axis, then the value of the distribution parameter α is non zero and the system exhibits non-Debye type relaxation. If $\alpha > 0.5$, then there may be more than one relaxation process. When $\alpha = 0$, the Cole–Cole model reduces to the Debye model [74,81]. The semicircular arc shows the mono-dispersive nature of the system. The frequency associated with the highest point of this semi-circular curve (Cole–Cole Plot) is the relaxation frequency *f* of the orientational polarization for LC droplets. Dielectric heating is maximized at the relaxation frequency, which yields the maximum dissipation factor.

To understand the effects of temperature and dye doping on the dielectric strength and relaxation process, Cole–Cole plots (Fig. 17) were

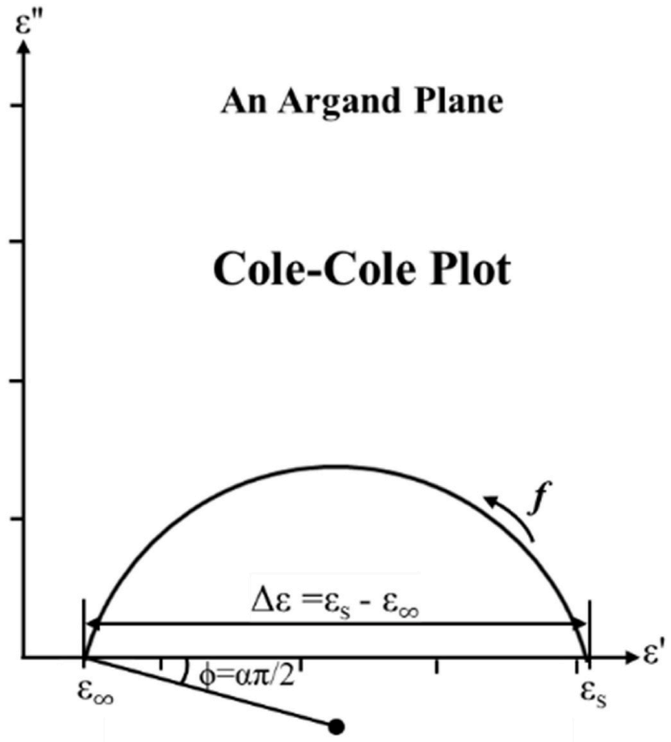

Fig. 16. Schematic representation of various parameters in the Cole–Cole plot.

$$\phi = \frac{\alpha\pi}{2} \quad (12)$$

drawn for O*00* N DPDLC films with various dye concentrations at different temperatures.

After calculating α, according to Fig. 16 and Equation (12), we found that α = 0 for all of the DPDLC films with different dye concentrations and thus exhibited Debye type relaxation, probably due to dipolar rotation around the long molecular axis of the dye–LC droplets. The dielectric strength was also very high for the 0.007% DPDLC film doped with a low amount of dye (with high *CR* and ΔT values, and low V_{TH} and V_{SAT} values), and thus it could withstand for the maximum voltage without breaking down [72,73,82,83]. According to Fig. 17 and Equation (10), we found that the dielectric strength (at 25 °C) was 8.89 for the 0% DPDLC film. After adding a small quantity of dye (0.007%) to the LC, the dielectric strength increased and reached 15.02, possibly because the absorption of some electrical energy by the dye molecules delayed the intrinsic electric breakdown, thereby increasing the value of dielectric strength. The size of LC droplets increased as the dye concentration increased to 0.015% and 0.0625% in the LC, and thus dipole–dipole interactions between the dye and LC molecules became dominant, and space charges tended to accumulate near the poles of the dye–LC system to enhance the local field and decrease the breakdown strength. The dielectric strength value for the 0.015% DPDLC film and 0.0625% DPDLC film were 13.83 and 11.69, respectively. As the dye concentration increased further (0.25% and 1% in LC), the droplet size increased and interfacial polarization (Maxwell–Wagner effect) became important. The depletion layer that formed between the dye–doped LC droplets and polymer matrix was responsible for the increased dielectric strength. The dielectric strength values for the 0.25% DPDLC film and 1% DPDLC film were 16.02 and 17.58, respectively [45,47].

All of the graphs showed that the value of $\Delta\varepsilon$ increased up to 40 °C and then decreased, possibly because the slight increase in temperature reduced the intermolecular interactions to relax the orientation–reorientation process for the dipoles, whereas thermal agitation became dominant at high temperatures to induce randomization in the dipoles. Thermal breakdown occurred at this point and it was responsible for the decrease in the dielectric strength of the composite film. The dielectric parameters for the optimum DPDLC film (0.007% dye doped) at different temperatures are summarized in Table 3.

Table 3 shows that the dielectric strength increased with the temperature up to 40 °C, but the dielectric strength then started to decrease as the temperature increased further. Similar to the 0.007% DPDLC film, the values of α (not shown here) calculated from the Cole–Cole plots drawn for O*00* N DPDLC films with different dye concentration in the temperature range from 25 to 60 °C were also zero, and thus they exhibited Debye type relaxation.

4. Conclusions

In this study, we characterized O*00* N DPDLC composite films synthesized using the photopolymerization technique with morphological, electro-optical, thermo-electro-optical, absorbance, and DRS methods. Morphological analyses showed that the droplet size increased as the dye concentration increased. The application of an electric field significantly affected the configuration of the dye–doped LC droplets which was responsible for the electro-optical properties of the composite films. Electro-optical analyses showed that the DPDLC film with the lowest dye concentration was optimal because it had the minimum T_{OFF} (= 0.41%), maximum T_{ON} (= 98.98%), high ΔT (= 98.57%) and *CR* (= 241) values, and low V_{TH} (= 0.85 V/μm) and V_{SAT} (= 1.45 V/μm) voltages. The high dye concentration degraded the electro-optical properties of the DPDLC film. Thermo-optical analysis demonstrated changes in the optical transmittance due to thermal variations. The electro-optical properties also degraded as the temperature increased. Characterization of the 1% DPDLC film showed that the absorption value decreased as the applied voltage increased, thereby confirming the higher order alignment of the dye–LC system along the direction of the applied electric field. DRS analysis and Cole–Cole plots indicated the presence of Debye type relaxation with zero value for the distribution parameter (α) for all of the O*00* N DPDLC composite films. The dielectric strengths of the DPDLC films increased up to a temperature of 40 °C and then started to decrease. Hence, we conclude that doping LC with a very small amount of dye, significantly improved the properties of the PDLC composite films and they were sensitive to temperature.

Funding

This research was supported by the Department of Science and Technology (DST-WOS-A), India through Grant No. SR/WOS/A/PM-91/2018(G).

Authors' contributions

Dr. Anuja Katariya-Jain: Methodology, Conducting research, Data curation, Investigation, Writing-draft preparation.

Prof, R. R. Deshmukh: Conceptualization, Visualization, Supervision, Project administration, Writing- Reviewing and Editing.

Declaration of competing interest

The authors declare that they have no conflicts of interest that might have influenced the work reported in this paper.

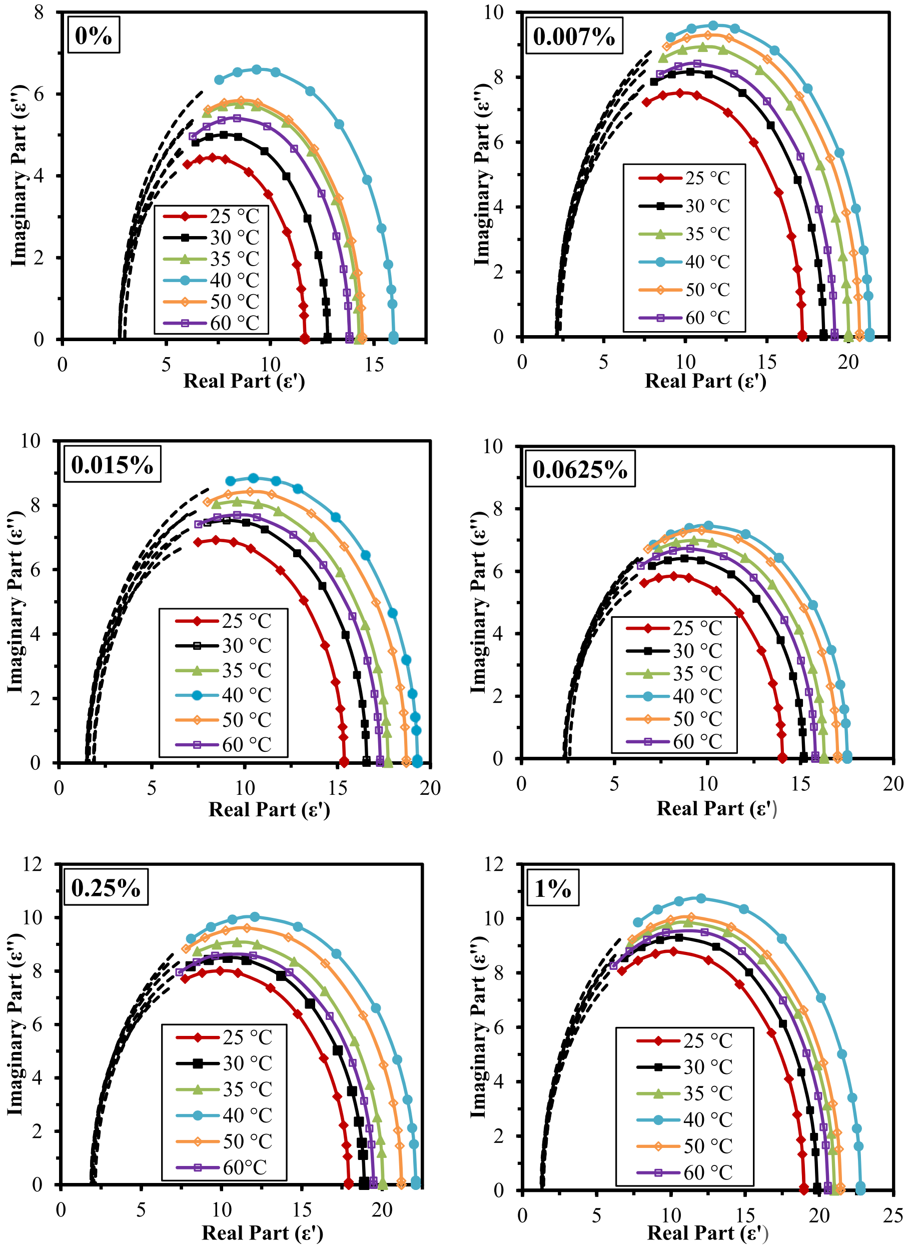

Fig. 17. Cole–Cole plots for O*00* N DPDLC films with various dye concentrations at different temperatures.

Table 3
Fitting parameters for 0.007% O*00* N DPDLC film at different temperatures.

Temperature (°C)	*f (MHz)*	ε_s	ε_∞	$\Delta\varepsilon$	*τ (sec)*	*α*
25	15.1	17.17	2.15	15.02	$1.05\ E^{-8}$	0
30	15.1	18.47	2.14	16.33	$1.05\ E^{-8}$	0
35	15.1	20.00	2.13	17.87	$1.05\ E^{-8}$	0
40	15.1	21.30	2.12	19.18	$1.05\ E^{-8}$	0
50	15.1	20.68	2.09	18.59	$1.05\ E^{-8}$	0
60	13.2	19.15	2.32	16.83	$1.21\ E^{-8}$	0

Acknowledgments

Dr. Anuja Katariya-Jain would like to thank the Department of Science & Technology (DST-WOS-A), India through Grant No. SR/WOS/A/PM-91/2018(G).

References

[1] G.P. Crawford, D. Svensek, S. Zumer, in: H.-S. Kitzerow, C. Bahr (Eds.), Chirality in Liquid Crystals Partially Ordered System, Springer, Newyork, 2001.
[2] Y.J. Jeon, Y. Bingzhu, J.T. Rhee, D.L. Cheung, M. Jamil, Macromol. Theory Simul. 16 (2007) 643.
[3] R. Karapinar, Turk. J. Phys. 22 (1998) 227.
[4] G. Spruce, R.D. Pringle, Electron. Commun. Eng. J. 4 (1992) 91.
[5] J.W. Doane, A. Golemme, J.L. West, J.B. Whitehead, B.-G. Wu, Mol. Cryst. Liq. Cryst. Inc. Nonlinear Opt. 165 (1988) 511.
[6] B. Bahadur (Ed.), Liquid Crystal Apllications and Uses, 1, World Scientific, Singapore, 1990.
[7] H. Stegemeyer (Ed.), Liquid Crystals, Springer, Newyork, n.d., 1994.
[8] R.R. Deshmukh, in: V.K. Thakur, M.R. Kessler (Eds.), Liquid Crystalline Polymers, Springer International Publishing, Cham, 2015, pp. 169–195.
[9] J.A. Ferrari, E.A. Dalchiele, E.M. Frins, J.A. Gentilini, Ć.D. Perciante, E. Scherschener, J. Appl. Phys. 103 (2008).
[10] A.K. Kalkar, V.V. Kunte, Mol. Cryst. Liq. Cryst. Sci. Technol. Sect. A Mol. Cryst. Liq. Cryst. 383 (2002) 1.
[11] F. Liu, H. Cao, Q. Mao, P. Song, H. Yang, Liq. Cryst. 39 (2012) 419.
[12] Y. Lu, J. Wei, Y. Shi, O. Jin, J. Guo, Liq. Cryst. 40 (2013) 581.
[13] R. Miedzinski, J. Ebothe, I. Fuks-Janczarek, I.V. Kityk, A. Majchrowski, R. Weglowski, S.J. Klosowicz, J. Mater. Sci. Mater. Electron. 21 (2010) 659.

[14] M.H. Saeed, S. Zhang, L. Zhou, G. Chen, M. Wang, L. Zhang, D. Yang, H. Yang, J. Mod. Opt. 67 (2020) 682.
[15] J.L. West, R. Ondris-Crawford, M.A. Erdmann, Dichroic Dye Containing Polymer-Dispersed Liquid Crystal Films, 1990, p. 76.
[16] A.K. Misra, B.P. Singh, S. Chandraker, K.K. Pandey, P.K. Tripathi, J.K. Saluja, R. Manohar, Opt. Mater. 93 (2019) 19.
[17] D. Pourmostafa, H. Tajalli, A. Vahedi, K. Milanchian, Opt. Mater. 107 (2020) 110061.
[18] M.E. Abbasov, G.O. Carlisle, J. Mater. Sci. Mater. Electron. 23 (2012) 712.
[19] R.J. Cox, Mol. Cryst. Liq. Cryst. 55 (1979) 1.
[20] S. Imazeki, M. Kaneko, T. Ozawa, A. Mukoh, Mol. Cryst. Liq. Cryst. Inc. Nonlinear Opt. 159 (1988) 219.
[21] G. Pelzl, H. Zaschke, D. Demus, Displays 6 (1985) 141.
[22] H. Seki, T. Uchida, Y. Shibata, Mol. Cryst. Liq. Cryst. 138 (1986) 349.
[23] P.S. Drzaic, Liquid Crystal Dispersions, WORLD SCIENTIFIC, Singapore, 1995.
[24] B. Bahadur (Ed.), Liquid Crystal Applications and Uses-, 3, World Scientific, Singapore, 1992.
[25] B. Bahadur, R.K. Sarna, V.G. Bhide, Mol. Cryst. Liq. Cryst. 75 (1981) 121.
[26] A.C. Lowe, Mol. Cryst. Liq. Cryst. 66 (1981) 295.
[27] T.J. Scheffer, J. Nehring, in: G.J. Sprokel (Ed.), The Physics and Chemistry of Liquid Crystal Devices, Springer, New York, 1980.
[28] F.C. Saunders, K.J. Harrison, E.P. Raynes, D.J. Thompson, IEEE Trans. Electron. Dev. 30 (1983) 499.
[29] B. Bahadur (Ed.), Liquid Crystals Applications and Uses, –2, World Scientific, Singapore, 1991.
[30] J. Cognard, T. Hieu Phan, Mol. Cryst. Liq. Cryst. 68 (1981) 207.
[31] G.W. Gray, Dyes Pigments 3 (1982) 203.
[32] A.V. Ivashchenko, Dichroic Dyes for Liquid Crystal Displays, CRC Press, 2018.
[33] M.G. Pellatt, I.H.C. Roe, J. Constant, Mol. Cryst. Liq. Cryst. 59 (1980) 299.
[34] S. V Serak, A. V Agashkov, V.Y. Reshetnyak, Quant. Electron. 31 (2001) 273.
[35] P.S. Drzaic, Pure Appl. Chem. 68 (1996) 1435.
[36] P. Malik, K.K. Raina, Phys. B Condens. Matter 405 (2010) 161.
[37] S.P. Yadav, K.K. Pandey, A.K. Misra, S. Dixit, R. Manohar, Can. J. Phys. 89 (2011) 661.
[38] D. Bauman, W. Haase, Mol. Cryst. Liq. Cryst. Inc. Nonlinear Opt. 168 (1989) 155.
[39] P. Kumar, K.K. Raina, Curr. Appl. Phys. 7 (2007) 636.
[40] D.-K. Yang, S.T. Wu (Eds.), Fundamentals of Liquid Crystaldevices, Wiley, Chichester, 2006.
[41] A. Katariya Jain, R.R. Deshmukh, in: M. Sasani Ghamsari, I. Carlescu (Eds.), Liquid Crystals and Display Technology, IntechOpen, London, 2020, p. 13.
[42] R.R. Deshmukh, M.K. Malik, J. Appl. Polym. Sci. 108 (2008) 3063.
[43] R.R. Deshmukh, A. Katariya-Jain, Liq. Cryst. 43 (2016) 256.
[44] G.G. Raju, Dielectrics in Electric Fields, second ed., 2016.
[45] V.K. Thakur, M.R. Kessler, in: A. Tiwari, S. Valyukh (Eds.), Advanced Energy Materials, Scrivener Publishing LLC-Wiley, Salem (Massachusetts), 2014, pp. 207–257.
[46] R.R. Deshmukh, A. Katariya Jain, Liq. Cryst. 41 (2014) 960.
[47] G. Önsal, G. Kocakülah, A. Kahyaoğlu, O. Köysal, J. Mol. Liq. 284 (2019) 607.
[48] A. Hadj Sahraoui, S. Delenclos, S. Longuemart, D. Dadarlat, J. Appl. Phys. 110 (2011).
[49] I. Dierking (Ed.), Polymer-Modified Liquid Crystals, Royal Society of Chemistry, Cambridge, 2019.
[50] A. Brignon, J.P. Huignard, Phase Conjugated Laser Optics, John Wiley & Sons, New Jersey, 2004.
[51] R.R. Deshmukh, M.K. Malik, J. Appl. Polym. Sci. 131 (2014) 1.
[52] P.S. Drzaic, Liq. Cryst. 3 (1988) 1543.
[53] A.K. Kalkar, V. V Kunte, S.A. Bhamare, J. Appl. Polym. Sci. 107 (2008) 689.
[54] M.K. Malik, P.G. Bhatia, R.R. Deshmukh, Indian J. Sci. Technol. 5 (2012) 3440.
[55] R.R. Deshmukh, M.K. Malik, J. Phys. Chem. Solid. 74 (2013) 215.
[56] R. H. Reamey, W. Montoya, A. Wong, R. Corporation, C. Drive, and M. Park, 1665, 2 (1992).
[57] P. Kumar, Neeraj, S.W. Kang, S.H. Lee, K.K. Raina, Thin Solid Films 520 (2011) 457.
[58] D. Coates, J. Mater. Chem. 5 (1995) 2063.
[59] M. Dutta Gupta, A. Mukhopadhyay, S.K. Roy, R. Dabrowski, J. Appl. Phys. 113 (2013) 1.
[60] N.A. Vaz, G.P. Montgomery, J. Appl. Phys. 62 (1987) 3161.
[61] P. Malik, K.K. Raina, Opt. Mater. 27 (2004) 613.
[62] R.R. Deshmukh, M.K. Malik, J. Appl. Polym. Sci. 109 (2008) 627.
[63] R. Deshmukh, M. Malik, Liq. Cryst. 40 (2013) 1050.
[64] L. Marrucci, D. Paparo, P. Maddalena, E. Massera, E. Prudnikova, E. Santamato, J. Chem. Phys. 107 (1997) 9783.
[65] I. Jánossy, Phys. Rev. E 49 (1994) 2957.
[66] R. Deshmukh, M. Malik, S. Parab, Adv. Mater. Res. 584 (2012) 79.
[67] A. Katariya Jain, R.R. Deshmukh, Liq. Cryst. 46 (2019) 1191.
[68] G. Kocakülah, M. Yıldırım, O. Köysal, J. Mater. Sci. Mater. Electron. 31 (2020) 2583.
[69] R. Deshmukh, S. Parab, M. Malik, Adv. Mater. Res. 584 (2012) 531.
[70] A.K. Jonscher, Dielectric Relaxation in Solids, Chelsea Dielectric Press, London, 1983.
[71] S.S. Parab, M.K. Malik, R.R. Deshmukh, J. Non-Cryst. Solids 358 (2012) 2713.
[72] P.G. De Gennes, J. Prost, The Physics of Liquid Crystals, Oxford University Press, New York, 1993.
[73] M. Rahman, C.-W. Hsieh, C.-T. Wang, B.-R. Jian, W. Lee, Dyes Pigments 84 (2010) 128.
[74] K.S. Cole, R.H. Cole, J. Chem. Phys. 9 (1941) 341.
[75] V.F. Lvovich, Impedance Spectroscopy Applications to Electrochemical and Dielectric Phenomena, Wiley, New Jersey, 2012.
[76] S. Wrobel, in: W. Hasse, S. Wrobel (Eds.), Relaxation Phenomena Liquid Crystals, Magnetic Systems, Polymers, High-Tc Superconductors, Metallic Glasses, Springer-Verlag, Berlin Heidelberg, 2003.
[77] S.S. Parab, M.K. Malik, P.G. Bhatia, R.R. Deshmukh, J. Mol. Liq. 199 (2014) 287.
[78] S.N. Al-Refaie, Appl. Phys. A Solids Surfaces 52 (1991) 234.
[79] M. Okutan, F. Yakuphanoglu, S.E. San, O. Koysal, Phys. B Condens. Matter 368 (2005) 308.
[80] S.S. Parab, M.K. Malik, R. Dabrowski, R.R. Deshmukh, J. Mol. Liq. 183 (2013) 20.
[81] K.S. Cole, R.H. Cole, J. Chem. Phys. 10 (1942) 98.
[82] S. Özder, M. Okutan, O. Köysal, H. Göktaş, S.E. San, Phys. B Condens. Matter 390 (2007) 101.
[83] R.R. Deshmukh, A.K. Jain, Liq. Cryst. 43 (2016) 436.
