# Supplementary material for: Advanced liquid crystal-based switchable optical devices for light protection applications: principles and strategies
Source: Light Sci Appl. 2023 Jan 3;12:11. doi: 10.1038/s41377-022-01032-y (PMC9807646; doi:10.1038/s41377-022-01032-y)
Supplement: Supplementary file 14 — Fig 16 copyright promotion [file 41377_2022_1032_MOESM14_ESM.pdf]

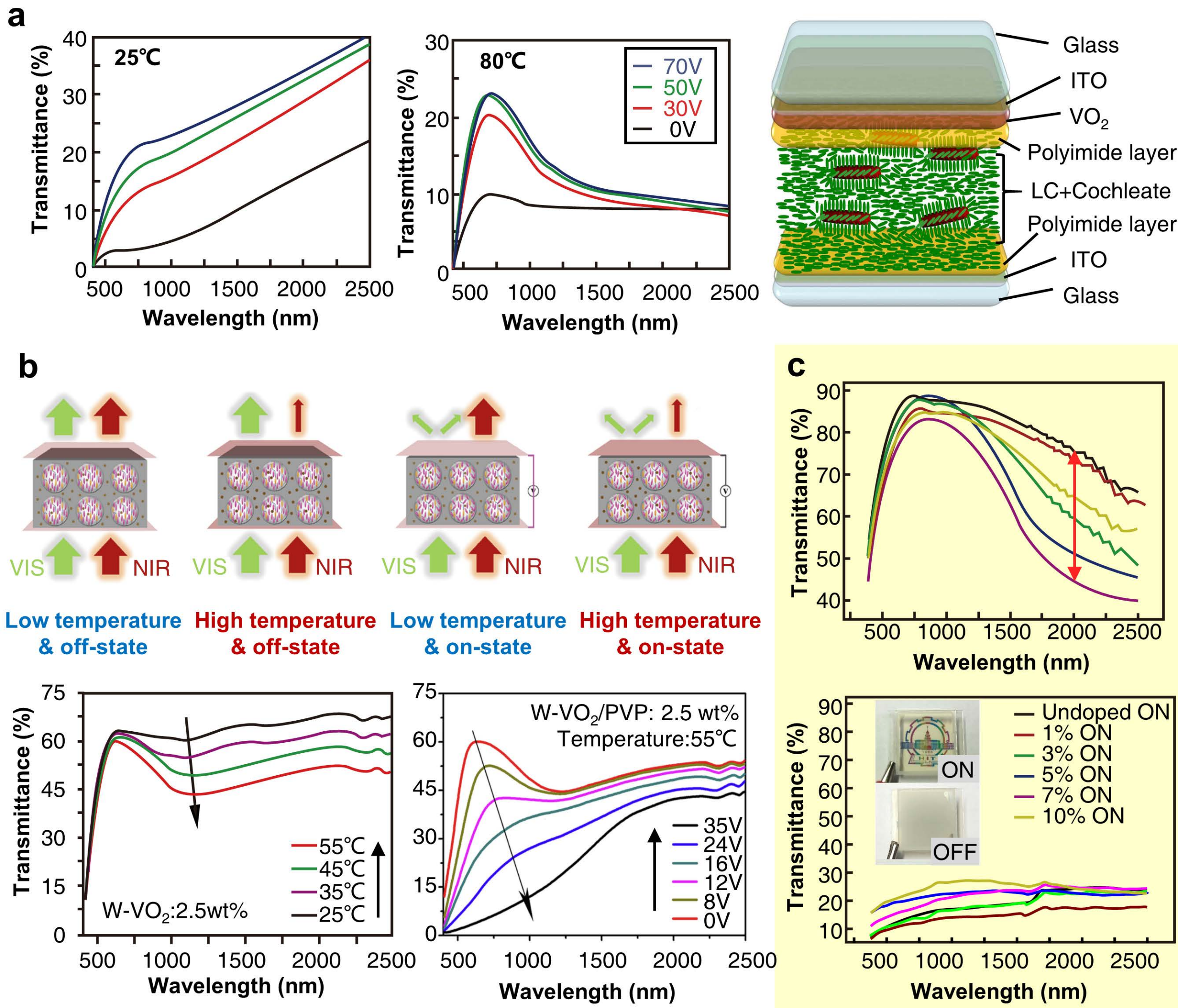
a
25℃
Transmittance (%)
Wavelength (nm)
80℃
70V
50V
30V
0V
Glass
ITO
VO2
Polyimide layer
LC+Cochleate
Polyimide layer
ITO
Glass
b
VIS
NIR
Low temperature & off-state
High temperature & off-state
Low temperature & on-state
High temperature & on-state
55℃
45℃
35℃
25℃
W-VO2:2.5wt%
W-VO2/PVP: 2.5 wt%
Temperature:55℃
35V
24V
16V
12V
8V
0V
c
Undoped ON
1% ON
3% ON
5% ON
7% ON
10% ON
ON
OFF

主　题:	RE: Reprint Permissions	
发件人:	"Optica Publishing Group Copyright" <copyright@osa.org>	2022-9-27 21:26:33
收件人:	"张锐聪" <ruicong_zhang@stu.hit.edu.cn>, "Optica Publishing Group Copyright" <copyright@osa.org>	

Dear Dr. Zhang,

Thank you for contacting Optica Publishing Group.

For the use of figures 1 and 5 from P. Lakshmi Madhuri, Saranya Bhupathi, S. Shuddhodana, Zaher M. A. Judeh, Sheng-Hsiung Yang, Yi Long, and Ibrahim Abdulhalim, "Hybrid vanadium dioxide-liquid crystal tunable non-reciprocal scattering metamaterial smart window for visible and infrared radiation control," Opt. Mater. Express 11, 3023-3037 (2021):

> Optica Publishing Group considers your requested use of its copyrighted material to be Fair Use under United States Copyright Law. We request that a complete citation of the original material be included in any publication.
>
> As this article is published under the terms of the Optica Publishing Group Open Access Publishing Agreement, when adapting or otherwise creating a derivative version of the article, users must maintain attribution to the author(s) and the published article's title, journal citation, and DOI. Users should also indicate if changes were made and avoid any implication that the author or Optica Publishing Group endorses the use.

While your publisher should be able to provide additional guidance, we prefer the below citation formats:

> For citations in figure captions:
>
> [Reprinted/Adapted] with permission from [ref #] © The Optical Society. (Please include the full citation in your reference list)
>
> For images without captions:
>
> Journal Vol. #, first page (year published) An example: Opt. Express 27, 1164 (2019)

Please let me know if you have any questions.

Kind Regards,

Hannah Greenwood

Hannah Greenwood

September 27, 2022

Authorized Agent, Optica Publishing Group

OPTICA PUBLISHING GROUP | Formerly OSA

From: 张锐聪 <ruicong_zhang@stu.hit.edu.cn>
Sent: Tuesday, September 20, 2022 4:37 AM
To: Optica Publishing Group Copyright <copyright@osa.org>
Subject: Reprint Permissions

Dear Editor,

Hello! We would like to apply for permission to reproduce images from papers published by your publisher, as detailed below.

Title : Hybrid vanadium dioxide-liquid crystal tunable non-reciprocal scattering metamaterial smart window for visible and infrared radiation control
Author :P. Lakshmi Madhuri, Saranya Bhupathi, S. Shuddhodana, Zaher M. A. Judeh, Sheng-Hsiung Yang, Yi Long, and Ibrahim Abdulhalim
Volume, issue, and page numbers: 11,9,3023-3037 (2021)
Journal : Optical Materials Express
Expected content to be reproduced : Fig.1 and Fig.5

Information on the papers we plan to publish is as follows.
Title : Advanced liquid crystal-based switchable optical devices for light protection applications: principles and strategies
Publisher : Springer Nature
Journal : Light: Science & Applications
Estimated publication time : In November 2022

Main author of the article : Ruicong Zhang, Jiajun li, Zhibo Zhang, Jiecai Han, Lei Yang, Tianyu Wang, Jiaqi Zhu

We admire your society's contribution to the Institute of Optics and hope to obtain permission to reprint images.

With thanks!

Ruicong Zhang

Harbin Institute of Technology

主　题:	RE: Reprint Permissions
发件人:	"Optica Publishing Group Copyright" <copyright@osa.org> 2022-10-25 22:35:18
收件人:	"张锐聪" <ruicong_zhang@stu.hit.edu.cn>, "Optica Publishing Group Copyright" <copyright@osa.org>

Dear Dr. Zhang,

Thank you for contacting Optica Publishing Group.

For the use of material from Chun-Wei Chen, Alyssa N. Brigeman, Tsung-Jui Ho, and Iam Choon Khoo, "Normally transparent smart window based on electrically induced instability in dielectrically negative cholesteric liquid crystal," Opt. Mater. Express 8, 691-697 (2018):

There are only 5 figures in this article. Please let us know if you would like to reproduce one of these figures.

For the use of figure 2 from Ghadah H. Sheetah, Qingkun Liu, Bohdan Senyuk, Blaise Fleury, and Ivan. I. Smalyukh, "Electric switching of visible and infrared transmission using liquid crystals co-doped with plasmonic gold nanorods and dichroic dyes," Opt. Express 26, 22264-22272 (2018), figure 1 from Bing-Xiang Li, Rui-Lin Xiao, Sathyanarayana Paladugu, Sergij V. Shiyanovskii, and Oleg D. Lavrentovich, "Dye-doped dual-frequency nematic cells as fast-switching polarization-independent shutters," Opt. Express 27, 3861-3866 (2019) and figure 5 from P. Lakshmi Madhuri, Saranya Bhupathi, S. Shuddhodana, Zaher M. A. Judeh, Sheng-Hsiung Yang, Yi Long, and Ibrahim Abdulhalim, "Hybrid vanadium dioxide-liquid crystal tunable non-reciprocal scattering metamaterial smart window for visible and infrared radiation control," Opt. Mater. Express 11, 3023-3037 (2021):

Optica Publishing Group considers your requested use of its copyrighted material to be Fair Use under United States Copyright Law. We request that a complete citation of the original material be included in any publication.

As this article is published under the terms of the Optica Publishing Group Open Access Publishing Agreement, when adapting or otherwise creating a derivative version of the article, users must maintain attribution to the author(s) and the published article's title, journal citation, and DOI. Users should also indicate if changes were made and avoid any implication that the author or Optica Publishing Group endorses the use.

While your publisher should be able to provide additional guidance, we prefer the below citation formats:

For citations in figure captions:

[Reprinted/Adapted] with permission from [ref #] © The Optical Society. (Please include the full citation in your reference list)

For images without captions:

Journal Vol. #, first page (year published) An example: Opt. Express 27, 1164 (2019)

Please let me know if you have any questions.

Kind Regards,

Hannah Greenwood

Hannah Greenwood

October 25, 2022

Authorized Agent, Optica Publishing Group

OPTICA PUBLISHING GROUP | Formerly OSA

From: 张锐聪 <ruicong_zhang@stu.hit.edu.cn>
Sent: Wednesday, October 19, 2022 8:18 AM
To: Optica Publishing Group Copyright <copyright@osa.org>
Subject: Reprint Permissions

ear Editor,

Hello! We would like to apply for permission to reproduce images from papers published by your publisher, as detailed below.

Title : Electric switching of visible and infrared transmission using liquid crystals co-doped with plasmonic gold nanorods and dichroic dyes
Author : Ghadah H. Sheetah, Qingkun Liu, Bohdan Senyuk, Blaise Fleury, and Ivan. I. Smalyukh
Volume, issue, and page numbers: 26, 22264-22272 (2018)
Journal : Optics Express
Expected content to be reproduced : Fig.2(a),   Fig.2(e) and Fig.2(f)

Special request: We hope to allow us to rearrange these pictures, as shown below.
Original picture:

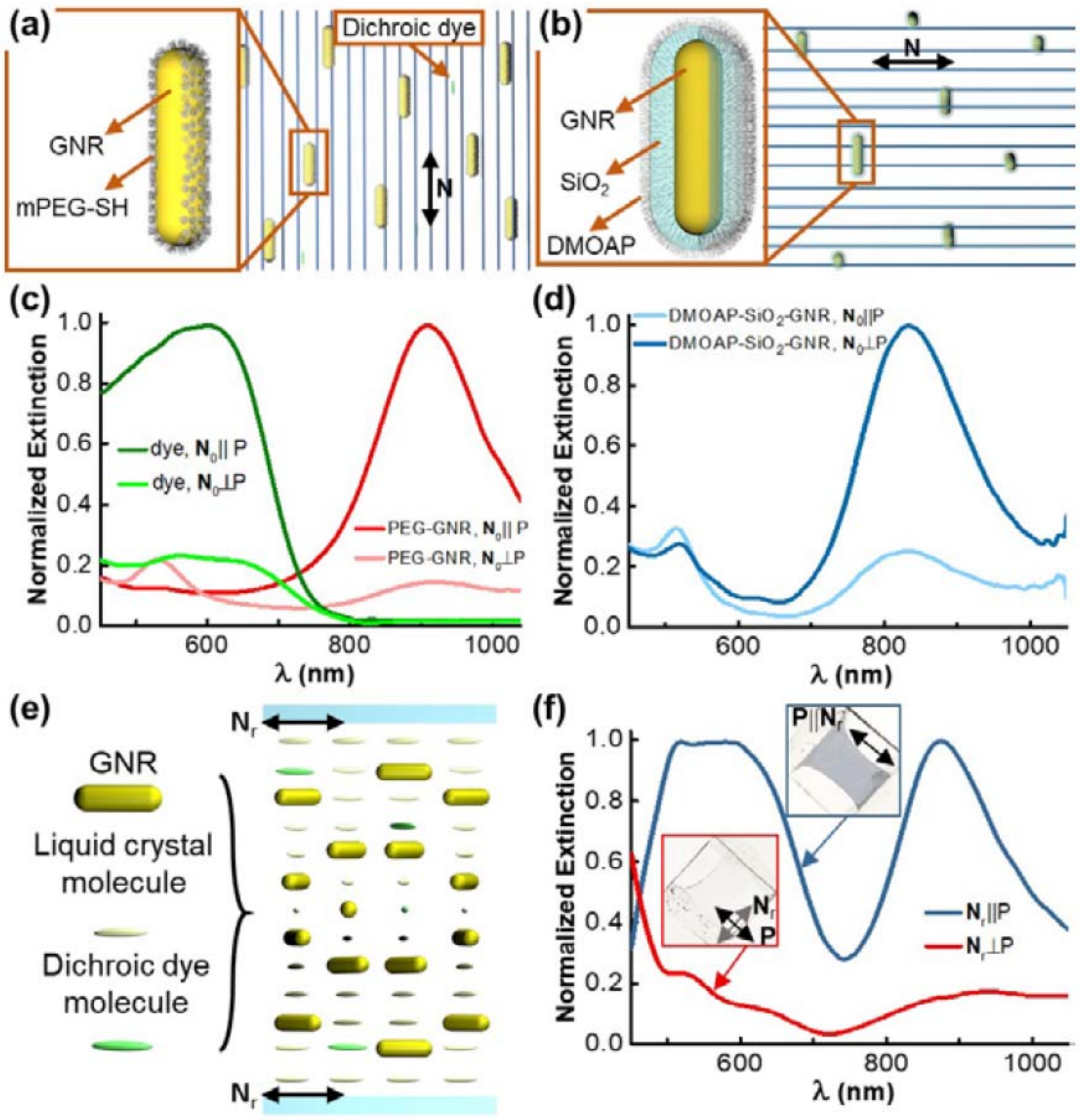

Fig. 2. Unidirectionally aligned and twisted structures of LC doped with GNRs and dichroic dye molecules. (a) Schematic of GNRs capped with mPEG-SH following **N** (blue lines) in a uniformly aligned LC; the inset shows details of surface functionalization of GNRs. (b) Schematic diagram of DMOAP-SiO_2-GNRs that exhibit self-alignment perpendicular to **N**; the inset shows details of silica shells around GNRs and DMOAP surface functionalization. (c,d)

Our contributions:

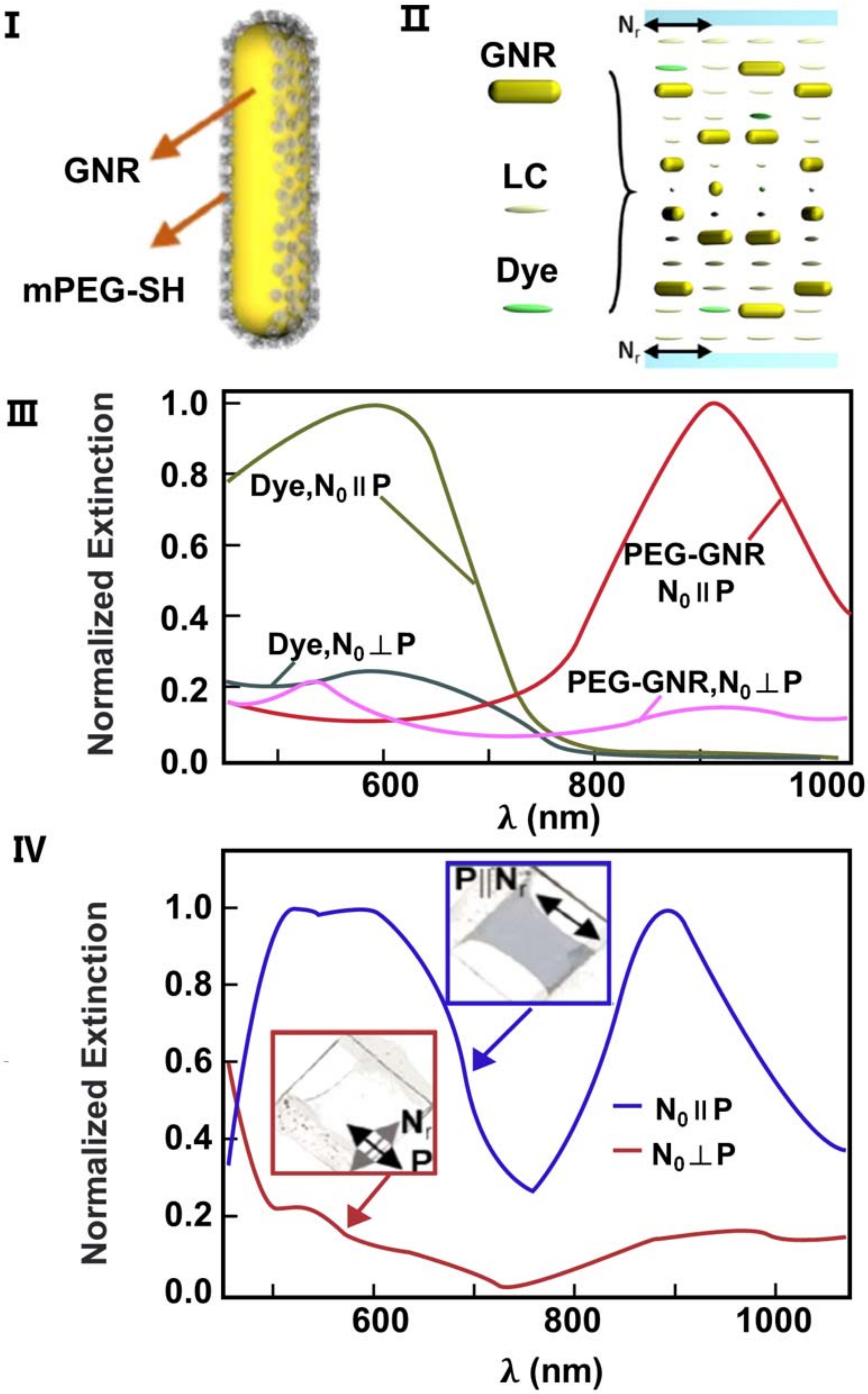

Information on the papers we plan to publish is as follows.
Title : Advanced liquid crystal-based switchable optical devices for light protection applications: principles and strategies
Publisher : Springer Nature
Journal : Light: Science & Applications
Estimated publication time : In November 2022

Main author of the article : Ruicong Zhang, Jiajun li, Zhibo Zhang, Jiecai Han, Lei Yang, Tianyu Wang, Jiaqi Zhu

We admire your society's contribution to the Institute of Optics and hope to obtain permission to reprint images.

With thanks!

Ruicong Zhang

Harbin Institute of Technology

主　题:	Reprint Permissions
发件人:	"张锐聪" <ruicong_zhang@stu.hit.edu.cn> 2022-10-23 17:40:22
收件人:	copyright@osa.org

Dear Editor,

Hello! We would like to apply for permission to reproduce images from papers published by your publisher, as detailed below.

Title : Hybrid vanadium dioxide-liquid crystal tunable non-reciprocal scattering metamaterial smart window for visible and infrared radiation control
Author : Madhuri, P. L., Bhupathi, S., Shuddhodana, S., Judeh, Z. M., Yang, S. H., Long, Y., & Abdulhalim, I.
Volume, issue, and page numbers: 11(9), 3023-3037.(2021)
Journal : Optical Materials Express
Expected content to be reproduced : Fig.5
Special request: We hope to allow us to rearrange these pictures, as shown below.
Original picture:

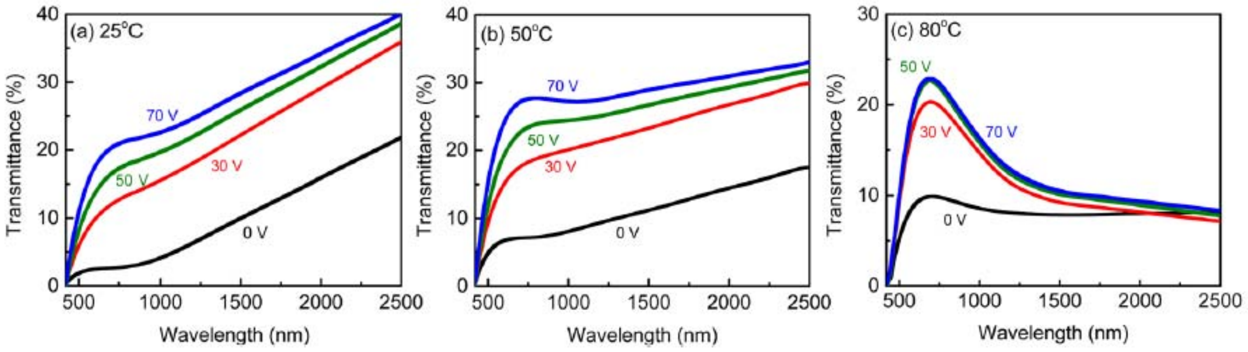

Fig. 5. Transmission spectra of VO_2 based LC-cochleate device plotted as a function of wavelength at three different temperatures a) 25°C b) 50°C and c) 80°C for various voltages.

Our contributions:

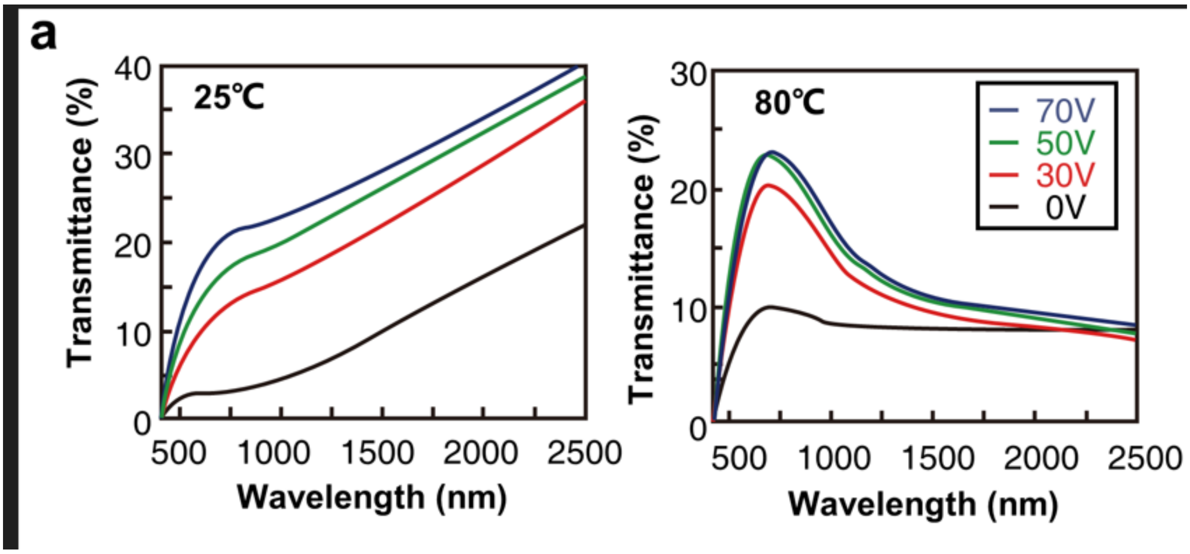

Information on the papers we plan to publish is as follows.
Title : Advanced liquid crystal-based switchable optical devices for light protection applications: principles and strategies
Publisher : Springer Nature
Journal : Light: Science & Applications
Estimated publication time : In November 2022
Main author of the article : Ruicong Zhang, Jiajun li, Zhibo Zhang, Jiecai Han, Lei Yang, Tianyu Wang, Jiaqi Zhu

We admire your society's contribution to the Institute of Optics and hope to obtain permission to reprint images.

With thanks!

Ruicong Zhang

Research Article | Vol. 11, No. 9 / 1 Sep 2021 / Optical Materials Express 3023

Optical Materials EXPRESS

Check for updates

Hybrid vanadium dioxide-liquid crystal tunable non-reciprocal scattering metamaterial smart window for visible and infrared radiation control

P. Lakshmi Madhuri,[1] Saranya Bhupathi,[2] S. Shuddhodana,[3] Zaher M. A. Judeh,[3] Sheng-Hsiung Yang,[4] Yi Long,[2] and Ibrahim Abdulhalim[1,*]

[1] *Department of Electro-Optics and Photonics Engineering and the Ilse Katz Institute for Nanoscale Science and Technology, School of Electrical and Computer Engineering, Ben-Gurion University of the Negev, Beer-Sheva 8410501, Israel*
[2] *School of Materials Science and Engineering, Nanyang Technological University, 639798, Singapore*
[3] *School of Chemical and Biomedical Engineering, Nanyang Technological University, Singapore, 62 Nanyang Drive, N1.2-B1-14, Singapore 637459, Singapore*
[4] *Institute of Lighting and Energy Photonics, National Chiao Tung University, No. 301, Gaofa 3rd Road, Guiren District, Tainan City 71150, Taiwan*
[*] *abdulhlm@bgu.ac.il*

Abstract: Smart windows based on VO_2 can control the infrared radiation entering the building based on the temperature, however, the visible part of the spectrum is not controlled. Liquid crystal (LC) based privacy windows, on the other hand, control the visibility either with temperature or applied voltage, however, the total transparency remains fixed as the scattering is mainly in the forward direction. To be able to control both the visibility and the temperature in the house, here we combine both layers in which the LC layer is made of a composite of nanoporous organic microparticles called Cochleates at small concentrations embedded in the LC matrix, thus acting as a tunable scattering metamaterial. The VO_2-LC interface has less Fresnel reflectivity and therefore higher solar modulation is expected in an optimized window. In addition, being hidden under the LC layer, VO_2 will be protected from oxidation. Electro-optic and thermo-optic properties of the device are investigated including the response time measurements. A non-reciprocity effect is observed showing better performance when the VO_2 layer is facing the outside world, in which the window becomes more transparent from inside than from outside and also showing higher solar modulation. Response time is 1 ms for the rise and 10 ms fall time at 70 V. This approach opens up a new possibility of thermochromic VO_2 and LC-based systems to satisfy the real-life requirements on smart window applications.

© 2021 Optical Society of America under the terms of the OSA Open Access Publishing Agreement

1. Introduction

Windows were invented since humans started to build houses for privacy, safety and to protect themselves from cold or hot weathers. Recent advances in modern technology, however are re-inventing the window by making it smarter to be able to self-control its visibility and heat transfer. With the increasing awareness in reducing the emission of harmful green-house gases that adversely affect our environment there is race for reducing energy consumption in buildings. Buildings are responsible for about 40% of the total energy consumption used for heating, cooling, lightening or air conditioning [1]. Buildings continuously exchange energy through their windows by means of conduction, convection and radiation. Managing this heat exchange through windows is a possible approach to effectively control the building energy consumptions. In summer, solar radiation and the visible light entering buildings should be controlled to reduce the air-conditioning energy consumption. On the contrary, in winters, buildings should allow the

#432784 https://doi.org/10.1364/OME.432784
Journal © 2021 Received 31 May 2021; revised 29 Jun 2021; accepted 30 Jun 2021; published 18 Aug 2021
Corrected: 23 Sep 2021

Optical Materials EXPRESS

inflow of solar radiation to retain the heat [2]. Smart windows which adjust the transmittance of solar irradiation are considered to be the promising candidates in reducing energy consumption of the buildings [3].

One of the effective routes to achieve this goal is by employing smart coatings on building windows that control the solar radiation. In this regard, several smart coatings based on electrochromism [4–6], gasochromism [7,8], thermochromism [9,10] and photochromism [11,12] have been widely investigated for the energy-efficient coating applications. Among them, thermochromic windows have gained enormous interest as they can modulate near-infrared radiation (NIR) from transmissive to opaque in response to the rise in environmental temperature, which does not require extra stimuli, in turn reduces the energy consumption. Thermochromic windows can be operated in two states: a transparent state with a higher solar transmittance and an opaque state with a lower solar transmittance [13].

Vanadium oxide (VO_2) is one such promising material for energy saving applications due to its reversible metal-insulator (MIT) phase transition that occurs at critical transition temperature (Tc) at 68 °C [14] accompanied by the changes in the optical properties [15]. Below Tc, VO_2 is in dielectric monoclinic phase that is transmissive to NIR. Above Tc, VO_2 in its metallic tetragonal phase is reflective for NIR. This feature makes VO_2 a promising material to be employed in the thermochromic smart coatings [16,17].

VO_2 coatings are mostly used as VO_2-based multilayer films [18–20] and VO_2 nanoparticles based flexible foils [21–23]. However, there are several obstacles limiting the applicability of VO_2 smart coatings [24]. (i) Phase-transition temperature (Tc) for pure VO_2 (68 °C) is too high to be applied on building windows, while Tc around 40 °C is acceptable; (ii) Luminous transmittance (T_{lum}) for single layer VO_2 with desirable solar modulation (ΔT_{sol}) is usually less than 40% due to the absorption in the short-wavelength range in both the insulating and metallic states of VO_2, needs to be larger than 50% for daily applications; (iii) VO_2 can easily transform into V_2O_5 phase when exposed to the environment, this phase is not just stable but does not possess the thermochromic property [25]. For practical applications, VO_2 must possess excellent thermochromic performance for several years. Therefore, environmental stability of VO_2 is a great challenge for practical applications as smart coatings. Recently, a unique core-shell structure made of Au/VO_2 was introduced which could reduce the Tc of VO_2 up to 10 °C, explained as a result of internal stress when metal nanoparticles are introduced in the VO_2 matrix, and not due to Plasmonic heating as it was believed before [26]. A machine learning approach to design and optimize VO_2 based nanostructured smart window performance was introduced to simplify the heavy numerical simulations usually required when complicated photonic structures of VO_2 are required to obtain better performance [27]. Improved transmittance in NIR and solar modulation are obtained by incorporating nanostructured VO_2 films, which can be achieved by femtosecond pulsed laser patterning [28] or patterned nano-holes [29]. Apart from their applications in smart windows, such structures are also being explored for their tunable photonic bandgap properties [30].

In parallel to this, there is also a great interest in controlling the visible radiation for privacy and human comfort in particular in places where windows cannot be opened for safety reasons such as in trains, hospitals, hotels and high buildings. Hence privacy windows in which the visibility can be controlled are in demand. Most of the existing solutions are based on voltage controlled light scattering using polymer dispersed LCs [31,32], dye-doped LCs [33,34], particle dispersions in LCs [35] and by employing transparent electrodes [36–38]. However, the scattering is both for the visible and infrared light and usually in the forward direction, meaning that the heat cannot be controlled based on these privacy windows. On the other hand, the smart windows that control the heat do not control the visible light. Hence there is a need for a smarter window that can control both the heat and the visibility. This is the main motivation for this work in which we present the first demonstration of such hybrid smart window showing the two functions by combining

VO_2 layer with a tunable scattering metamaterial layer made of LC-nanoporous microparticles composite which we have shown recently [39,40] to be able to control the transparency by voltage and temperature. The VO_2–LC interface has less Fresnel reflectivity and therefore higher solar modulation is expected in an optimized window. In addition, being hidden under the LC layer, VO_2 will be protected from oxidation.

2. Materials and methods

2.1. VO_2 film deposition on ITO substrate

VO_2 thin films were prepared through physical vapor deposition (PVD). The vanadium source is a 390 cm^2 99.9% purity vanadium target provided by Cemecon. During the deposition, the deposition chamber was evacuated to under 0.5 mPa and maintained at 420 °C throughout the experiment. Argon was pumped into the chamber with a flow rate of 500 sccm to serve as the protective gas, and the substrates were subjected to a DC bias of 120 V. High power impulse magnetron sputtering (HiPIMS) power was then supplied with a pulse frequency of 1500 Hz and a pulse on-time of 200 µs, which resulted in a duty cycle of 30%. After forming a monolayer of vanadium metal, oxygen gas was then introduced into the chamber with a flow rate of 53 sccm. The pressure was kept at 500 mPa during the deposition process [28].

2.2. Characterization

The surface morphology and the topography of VO_2 film on ITO were studied by Scanning Electron Microscopy (SEM, Quanta 200, FEI) and Atomic Force Microscopy (AFM, MFP-3D-Bio, Asylum Research, Oxford Instruments). SEM images were captured at an accelerating voltage of 3 kV, and AFM was operated in the tapping mode. The XPS data were collected using an X-ray photoelectron spectrometer ESCALAB 250 (Termo Scientific, England) ultrahigh vacuum ($1*10^{-9}$ bar) apparatus with an Al Kα X-ray source and a monochromator. The X-ray beam size was 500 µm and survey spectra were recorded with pass energy (PE) 150 eV and high energy resolution spectra were recorded with pass energy (PE) 20 eV. To correct for charging effects, all spectra were calibrated relative to a carbon C 1s peak positioned at 284.8 eV. Processing of the XPS results was carried out using AVANTGE program.

2.3. Sample preparation of LC cell assembly

Cochleates are lipid-based supramolecular tubular structures with a morphology accompanied by tightly packed bilayers [41]. A composite mixture of cochleates with nematic LC, BL036 (a mixture of alkylcyanobiphenyls obtained from Canaan Chem, China) is used for the device fabrication. Measurements are performed on the device made of 12 wt% cochleate in the BL036 [42]. The weighed amount of cochleate and LC is ultrasonicated for 15 mins in the presence of ethanol. The composite was subjected to magnetic stirring for 3 hrs in the presence of heat. The process helps in evaporating the ethanol, aiding to the uniform dispersion of the material.

Schematic illustration of the VO_2 based LC-cochleate device is shown in Fig. 1. Out of the two substrates employed in the LC cell assembly, one was Indium-Tin oxide (ITO) coated SF11 glass (used to enable the light transmission in the range 420–2300 nm) and the other was VO_2 coated ITO glass substrate. These two substrates were spin coated with a photoalignment layer ROP-108, obtained from ROLIC Technologies Ltd.-Switzerland. The substrates were baked at 180 °C for 15 mins. Thereafter, the substrates were treated with a linearly polarized ultraviolet (UV) light of power density of 0.5 mW/cm^2 for 30 mins. Coating of the substrates with the photoalignment layer and further treatment with UV light helps in the uniform alignment of the LC molecules in the device. These two glass substrates were assembled together with a cell gap of 10 µm. The prepared LC-cochleate mixture was then filled into the device in the isotropic phase of LC using capillary action.

Optical Materials EXPRESS

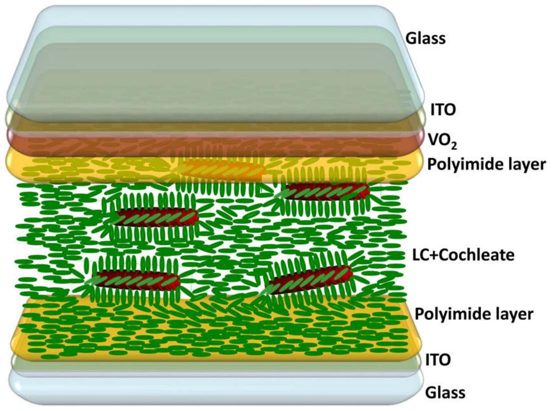

Fig. 1. Schematic illustration of VO_2 based LC- Cochleate device.

Electro-optic measurements on the device were performed with a tungsten-halogen light source (190–2500 nm) and the output response is recorded from the spectrometer for the entire Vis-IR range of the spectrum (StellarNet UV-Vis-NIR spectrometers (Blackcomet-SR) for the range 200–1080 nm and (NIR) for the range 900–2500 nm). Haze-transmittance measurements on the LC device were performed using an integrating sphere (Thorlabs). A bipolar sine wave input AC signal of specific amplitude at 1 kHz frequency from the function generator (GW Instek, AFG-2005) amplified by high-voltage amplifier (Tabor Electronics-9100A) is fed to the device. Instec hot-stage with mk 2000 controller is employed for the temperature dependent measurements.

3. Results and discussion

3.1. Device

The LC device can be operated between scattering and transparent states with the application of external voltage. The device switching for a sine wave input AC signal applied at 1 kHz frequency at different RMS voltages is shown in Fig. 2. As the applied voltage increases, gradual increase in the transparency from opaque state (voltage OFF) to transparent state (voltage ON) is achieved.

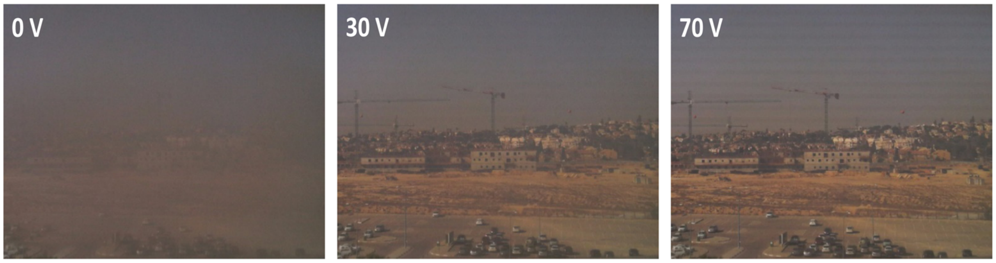

Fig. 2. Images taken through the LC device for a sine wave input AC signal applied at 1 kHz frequency in the scattering state obtained with RMS voltage of 0 V, translucent state at 30 V and the transparent state at 70 V in the absence of polarizers.

It should be mentioned that the voltage drop can be calculated based on the equivalent circuit model where each layer is represented by capacitance and resistor in parallel and the two layers are connected together in series [43]. Based on this the voltage drop is mostly on the LC layer because resistivity of VO_2 is much smaller (kΩ to few hundred kΩ at room temperature and few

Optical Materials EXPRESS

Ω above the MIT temperature) than that of the LC and the capacitance of the VO_2 layer is much larger than that of the LC (dielectric constant of VO_2 around 36 at room temperature and around 6000 above the MIT temperature) [44].

3.2. Microscopy and XPS analysis of VO_2 film

The surface morphology of the VO_2 thin-film is studied from SEM and AFM images shown in Fig. S1 of Supplement 1. Morphology confirms the uniform and dense coating of the film with the film thickness of ∼ 150 nm. In order to verify the contents of the film, XPS surface measurement was performed on the VO_2 film deposited on ITO substrate. The XPS spectra can be resolved into two main peaks in the V $2p_{3/2}$ and V $2p_{1/2}$ regions in Fig. S2. The parameters obtained after profile fitting such as peak position, full width at half-maximum (FWHM) and area are tabulated. The photoelectron peaks for the V $2p_{3/2}$ line in the range from 514–518 eV and for the V $2p_{1/2}$ line from 522–526 eV are the characteristic binding energies of V^{4+} (VO_2 phase) [45,46]. Traces of V_2O_5 are found in the peak fitting of XPS spectra. The higher binding energies for the V $2p_{3/2}$ line in the range from 516–520 eV and for the V $2p_{1/2}$ at 525 eV indicate the presence of V^{5+} (V_2O_5 phase), from the surface oxidation of the film [46,47]. Therefore, the XPS peak fitting confirms the presence of vanadium multiphases such as VO_2 and V_2O_5. This can be a result of the prolonged exposure of the films to air as they were prepared in NTU-Singapore (SHARE, CREATE labs), tested and shipped to BGU-Israel for device assembly.

3.3. Optical microscopy

Polarizing optical microscopic images of the VO_2 based LC-Cochleate device obtained without and in the presence of external AC voltages are shown in Fig. 3. The images are collected with device positioned at 0° (optic axis parallel to one of the polarizers), rotated by 45° with respect to the crossed polarizers, and in the absence of one of the polarizers. The uniform dispersion of the particles in the LC is observed both with and without applied voltage. Some of the LC molecules can get trapped within the hollow cylindrical anisotropic cochleate particles. With the application of voltage, LC molecules transfer their torque to the cochleate particles which in turn orient along the field direction. The system reverts back to the stable state upon withdrawal of the applied voltage, the state governed by the elastic forces. For more detailed description of the tunable scattering metamaterial LC-Chochleate device the reader is referred to our recent work [42].

3.4. Electro-optic measurements

The transmission spectrum of the VO_2 based LC-Cochleate device was measured by a UV−VIS−NIR spectrometer (Stellarnet). The experimental set-up employed for the measurements is shown in Fig. 4. The light beam from the source is collimated using the lens and directed to the device in the hot-stage from the pinhole. The scattered beam from the device gets collected by the collection lens. The lens focuses the beam to the fiber which in turn is fed to the spectrometer and PC.

The transmittance spectra of the VO_2 based LC-cochleate device obtained at three different temperatures of 25 °C, 50 °C and 80 °C for various voltages are shown in Fig. 5(a-c). The transmittance increases with the increase in the applied voltage. As the VO_2 is more transmissive to the light in IR region in its insulating state, at 25 °C and 50 °C the transmittance continues to increase throughout the IR wavelength range (900–2500 nm). The transmittance increases from 5% to 23% at the highest applied voltage of 70 V. At high temperature 80 °C, VO_2 is in its metallic state reflecting most of the IR radiation, hence the profiles show a drop in the overall transmittance of light from the device.

Figure 6(a-c) show the transmittance profiles at fixed AC RMS voltages (0 V, 50 V and 70 V) for three different temperatures, 25 °C, 50 °C and 80 °C. In the absence of external applied

Optical Materials EXPRESS

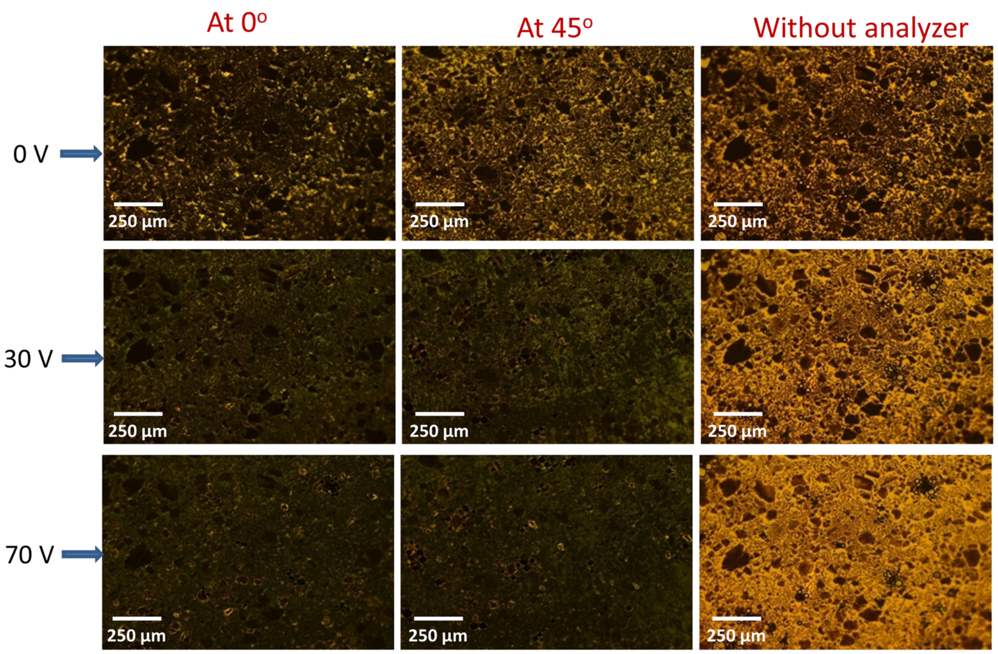

Fig. 3. Polarizing optical microscopy images of the VO_2 based LC-Cochleate device at different voltages. The images are obtained in the transmission mode with the LC cell positioned with optic axis at 0°, and 45° with respect to the polarizer axis between the crossed polarizers.

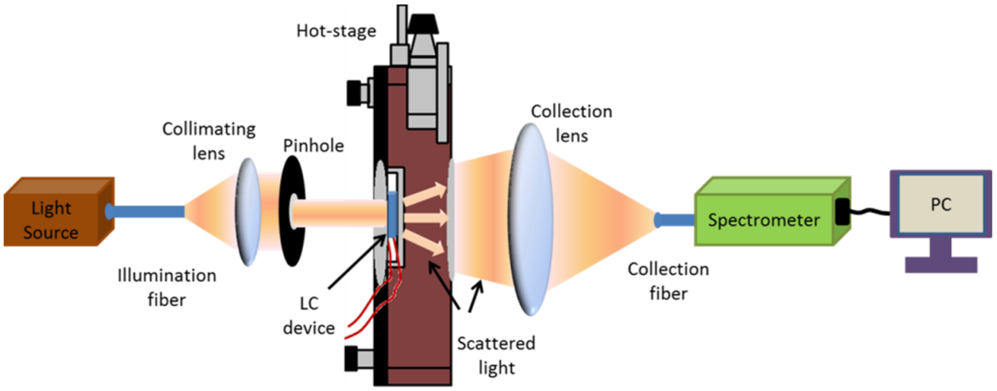

Fig. 4. Schematic of the experimental set-up employed for the transmission measurements.

Optical Materials EXPRESS

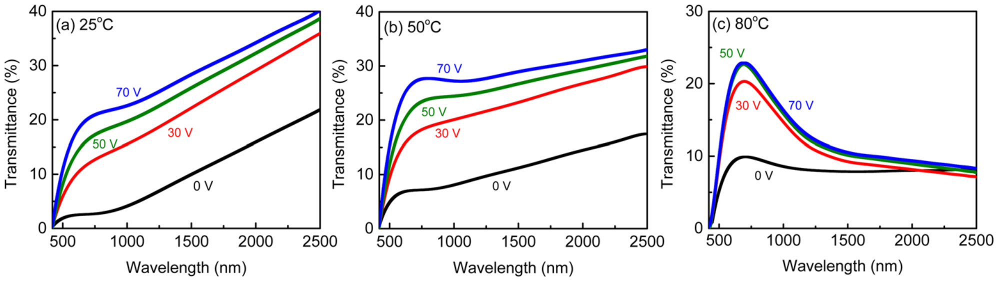

Fig. 5. Transmission spectra of VO_2 based LC-cochleate device plotted as a function of wavelength at three different temperatures a) 25°C b) 50°C and c) 80°C for various voltages.

field, at room temperature the device is in its scattering state owing to the refractive index (RI) mismatch between cochleate and LC molecules, and hence the transmittance is low in the visible region. As the VO_2 is in its insulating state at low temperatures, this allows the transmission of NIR through the device. As the applied voltage increases, the index matching between cochleate and LC molecules increases the transmittance from the device. At 70 V, the device becomes almost transparent, as the cochleates and LC molecules orient along the field direction reducing the scattering in the device. This further increases the transmittance. The profiles at 80 °C, show a reduction in transmittance in NIR region due to the fact that VO_2 is in its metallic state reflecting most of the IR radiation. Transmission spectra of pure VO_2 in the cold (20 °C) and hot (90 °C) states are shown in Fig. 6(d). Pure VO_2 allows up to 40% of transmission of visible light, but the device with LC molecules transmits about 23% of the incoming light. This can be optimized further by choosing more transparent glass than SF11 as well as more transparent ITO layers.

The transmission spectra of VO_2 are calculated from the optical constants obtained from the fit parameters of classical Drude-Lorentz classical oscillator model [48]. The model assumes the complex dielectric constant is given by the dispersion relation:

$$\epsilon(\omega) = \epsilon_\infty - \frac{\omega_n^2}{\omega^2 + i\omega_c\omega} + \sum_{i=1}^{n} \frac{S_i\omega_i^2}{\omega_i^2 - \omega^2 - i\Gamma_i\omega\omega_i} \tag{1}$$

The first term on the right ϵ_∞ represents a contribution to the real part of the dielectric constant from high-frequency electronic transitions. The second term gives the free-electron contribution to the dielectric constant. Here $\omega_n = (4\pi n_c e^2/m^*)^{1/2}$ is the carrier density parameter and is related to the plasma frequency, and $\omega_c = e/\mu_{\text{opt}} m^*$ is the collision frequency, where n_c is the number of conduction electrons and m^* the so-called optical mass of the electrons. The third term, which is a sum over Lorentz classical oscillators, takes into account the contributions to the dielectric constant from lattice vibrations. Herein, S_i, ω_i and Γ_i represent strength, frequency and linewidth respectively, of the ith oscillator. Parameters of optical constants obtained from the fitting in the insulating phase (at 20 °C) and metallic phase (at 90 °C) of VO_2 are tabulated in Table S1.

The integrated values of luminous transmittance (T_{lum}, 380–780 nm), infrared transmittance (T_{NIR}, 780–2500 nm), and solar transmittance (T_{sol}, 380–2500 nm), which are used to quantify the amounts of visible, NIR, and solar energy entering a smart window, can be determined from the following equations [29].

$$T_{\text{lum}} = \frac{\int_{\lambda=380}^{780} \varphi_{lum} T(\lambda) d\lambda}{\int_{\lambda=380}^{780} \varphi_{lum} d\lambda} \tag{2}$$

$$T_{\text{NIR}} = \frac{\int_{\lambda=780}^{2500} AM_{1.5}(\lambda)\ T(\lambda)\ d\lambda}{\int_{\lambda=780}^{2500} AM_{1.5}(\lambda)\ d\lambda} \tag{3}$$

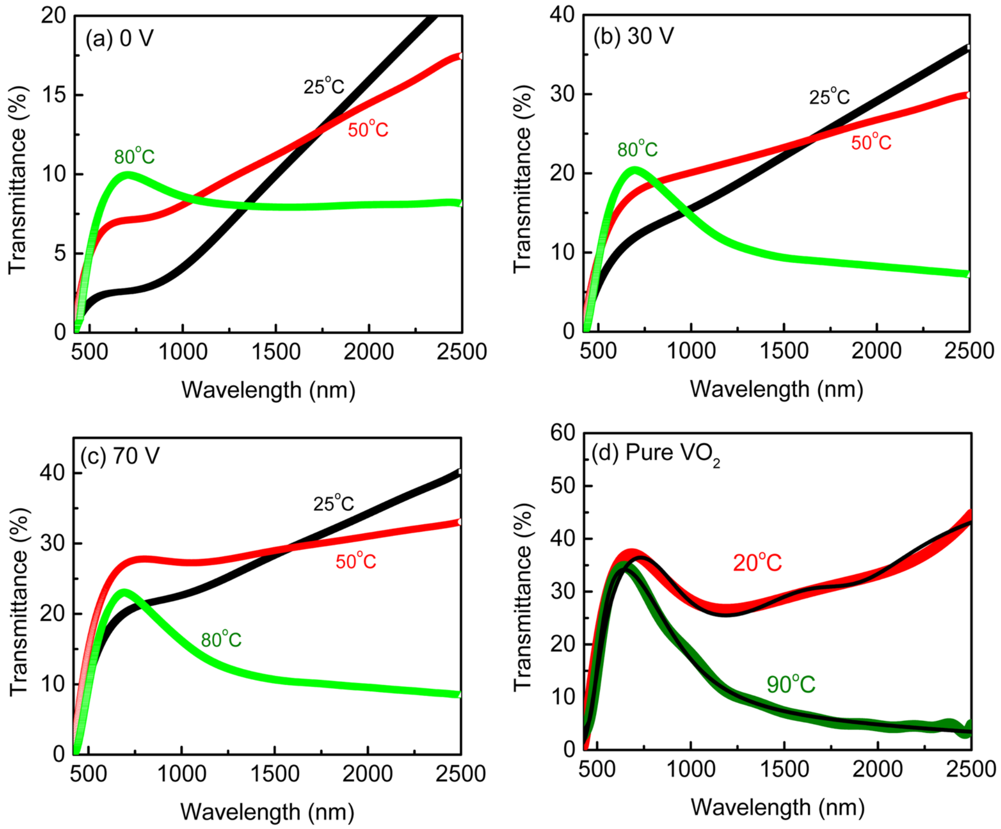

Fig. 6. Transmission spectra of LC device at a) 0 V b) 30 V c) 70 V for three different temperatures and d) Pure VO_2 sample without the LC at 20 °C and 90 °C. The lines through the data points for VO_2 are simulated using the fit parameters obtained from Drude-Lorentz classical oscillator model described below.

$$T_{sol} = \frac{\int_{\lambda=380}^{2500} AM_{1.5}(\lambda)\, T(\lambda)\, d\lambda}{\int_{\lambda=380}^{2500} AM_{1.5}(\lambda)\, d\lambda} \quad (4)$$

The modulation levels of transmitted luminous, NIR, and solar radiation at cold and hot weather conditions can be represented as ΔT_{lum}, ΔT_{NIR} and ΔT_{sol}, respectively, and it can be calculated from the following equations:

$$\Delta T_{lum} = T_{lum(cold)} - T_{lum(hot)} \quad (5)$$

$$\Delta T_{NIR} = T_{NIR(cold)} - T_{NIR(hot)} \quad (6)$$

$$\Delta T_{sol} = T_{sol(cold)} - T_{sol(hot)} \quad (7)$$

$T(\lambda)$ represents the measured spectral transmittance. The photopic luminous efficiency of the human eye (φ_{lum}) [49] and the air mass (AM) 1.5 solar irradiance spectrum distributions (corresponding to the sun standing 37° above the horizon) [50] are used as weighting functions for the wavelength-dependent transmittance coefficients. Cold and hot states stand for the insulating state of VO_2 at 20 °C and the metallic state of VO_2 at 90 °C, respectively.

Thermochromic parameters of bulk VO_2 show T_{lum} (Avg) = 39.7%, ΔT_{NIR} = 8% and ΔT_{sol} = 10.2%, whereas the device with LC-cochleate exhibits slightly lower performance with T_{lum}(Avg) = 5.3%, ΔT_{NIR} = 1.9% and ΔT_{sol} = 3.6% at 0 V, the values improve at 70 V (T_{lum}(Avg) = 17.3%,

Optical Materials EXPRESS

$\Delta T_{NIR} = 10.1\%$ and $\Delta T_{sol} = 4.16\%$). The low values of T_{lum} are attributed to the strong reflection and absorption in the visible region both due to non-optimized glass substrate and ITO layers. Employing antireflection coatings (ARCs) have proved to be one of the effective strategies for enhancing the low T_{lum} without degrading the thermochromic properties of the VO_2 films. Another problem for the low modulation values at 0 V is the fact that the device is scattering in this state and therefore the collected light to the optical fiber is not the whole light transmitted through the device.

The simulated transmittance profiles from the device at 0 V and 70 V for two different temperatures 25 °C and 80 °C are given in Fig. 7. The simulation was performed using 4 × 4 matrix formalism [51]. The measured transmittance values are lower in compared to simulated results for both the temperatures at 0 V. This is due to the fact that the device is in its scattering state without the application of voltage, while the simulation does not take into account the scattering. With VO_2 being in its insulating state at 25 °C it transmits most of the IR radiation causing the rise in transmittance in the infrared region.

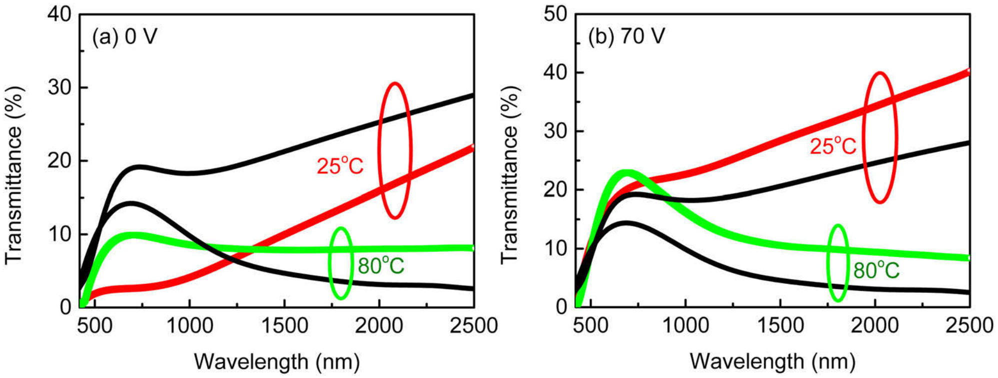

Fig. 7. Measured (red and green curves) and simulated transmittance profiles (black curves) from the device at (a) 0 V and (b) 70 V for two different temperatures 25°C and 80°C.

The transmittance at 80 °C is mainly contributed by two factors: Firstly, the increase in the transmittance observed from the device as the LC-Cochleate composite gets more diluted upon approaching closer to the isotropic phase, which reduces the scattering. Secondly, at this temperature VO_2 being in its metallic state reflects most of the IR radiation, reducing the transmission in the IR region. At 70 V, most of the LC-Cochleate molecules are oriented along the applied field direction leading to increase in the transmittance from the device. The measured transmittance change between the low and high temperature profiles in the visible region were 66% and 26% respectively at 0 V and 70 V. Similarly the change in the IR region is about 71% at 0 V and 89% at 70 V. The simulated transmittance change between 25 °C and 80 °C in the visible region was about 30% for both 0 V and 70 V. The change is about 89% and 92% at 0 V and 70 V respectively in the IR region.

Temperature dependence of transmittance in the wavelength range (900–2500 nm) at different voltages (0 V, 30 V and 70 V) is shown in Fig. 8. Evidently, the transmittance increases with the increase in wavelength for all the voltages below 70 °C. This originates from the fact that VO_2 is in its insulating state below 70 °C, allowing the IR radiation to pass through the device. Above 70 °C, the VO_2 is in its metallic state blocking the IR, lowering the total transmittance from the device. An interesting result observed in Fig. 8 is the fact that there is almost no sensitivity to the wavelength in the IR at the MIT transition, particularly at the scattering state (0 V).

Variation of the total transmittance (TT) is shown in Fig. 9, obtained using an integration sphere in the wavelength range of 420–2500 nm at 25 °C and 80 °C for different voltages (0 V,

Optical Materials EXPRESS

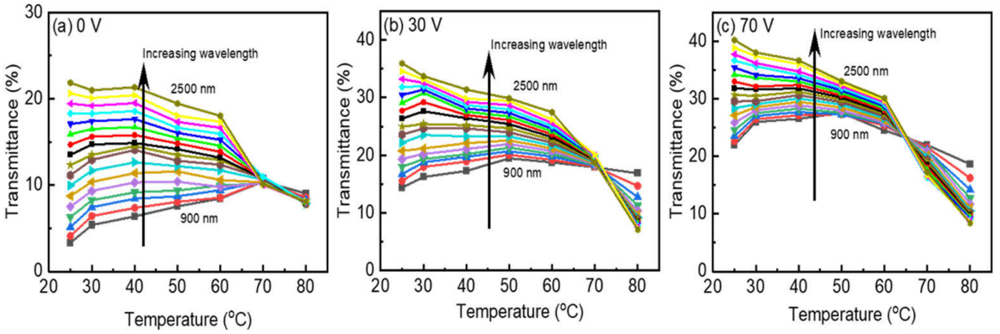

Fig. 8. Transmittance of VO_2 based LC-Cochleate device plotted as a function of temperature at three different RMS voltages a) 0 V b) 30 V and c) 70 V in the wavelength range (900–2500 nm). Transmittance increases with the increase in wavelength.

50 V and 70 V) at two different configurations, depending on which side the output signal is collected. The Fig. 9 (a, b), show the TT collected from the VO_2 side of the device showing that the transmittance increases as the applied voltage increases. At 25 °C the increase in transmittance is about 15% in the visible region and 17% in the IR region for the highest applied voltage 70 V. At 80 °C the change in transmittance is about 61% in the visible region and 8% in the IR region. The reflectance measured with integration sphere for 0 V, 50 V and 70 V at 25 °C and 80 °C for the output collected from LC side and VO_2 side of the device is shown in Fig. S3. The 3 mm diameter beam was collimated with a lens from an optical fiber, then hit the sample on the opposite side of the sphere. The incidence angle is supposed to be zero, however small angles are possible due to misalignment and some divergence of the beam.

Thermotropic parameters calculated from the data obtained using the integration sphere gives more accurate values, with T_{lum} (at 25 °C) = 5.06%, T_{lum} (at 80 °C) = 15.1%, ΔT_{NIR} = 1.6% and ΔT_{sol} = 5.1% at 0 V, the values being T_{lum} (at 25 °C) = 6.7%, T_{lum} (at 80 °C) = 30.3%, ΔT_{NIR} = 0.8% and ΔT_{sol} = 10.7% at 70 V. The solar modulation gets doubled at 70 V. For the output collected from the LC side of the device (see Fig. 9(c, d)) the transmittance at 80 °C almost reduces to half the value of the signal from the VO_2 side. Parameters calculated in this configuration have T_{lum} (at 25 °C) = 5.4%, T_{lum} (at 80 °C) = 12.1%, ΔT_{NIR} = 16% and ΔT_{sol} = 4.8%, at 0 V, while at 70 V, the values being T_{lum} (at 25 °C) = 9.2%, T_{lum} (at 80 °C) = 15.5%, ΔT_{NIR} = 12.4% and ΔT_{sol} = 2.8%. The reason for the nonreciprocal effect is due to two effects: (i) the asymmetric lossy structure, and (ii) the scattering. The losses in the ITO layer and the VO_2 can cause nonreciprocal behavior however this is a small effect as can be seen in Fig. S4 showing simulated reflectance/transmittance at normal incidence for unpolarized light from both sides of the device and different temperatures assuming uniform non-scattering device. The non-reciprocity due to losses and asymmetric structure showing around 5% maximum differences between the two sides. On the other hand in the true measurement (Fig. S3) the differences between the two sides can be as large as 30% depending on the temperature, voltage and wavelength. The scattering has larger effect because of the reflectivity dependence of VO_2 on the incidence angle, as when the light incident first on the VO_2 side, the reflectivity is mainly that of small angles, while the light is incident from the LC side, due to the forward scattering, many large angles hit the VO_2 and therefore changing the reflected and thus transmitted signals. As a privacy window it can be recommended to have the LC side facing the inside of the room so that the window is more transparent for the people inside and less when looking from outside. This orientation also gives the higher ΔT_{sol}, which is better in terms of energy consumption.

Figure 10 shows the schematic of experimental set-up employed in the measurements using integration sphere. The scattered beam from the device enters the integration sphere from the

Optical Materials EXPRESS

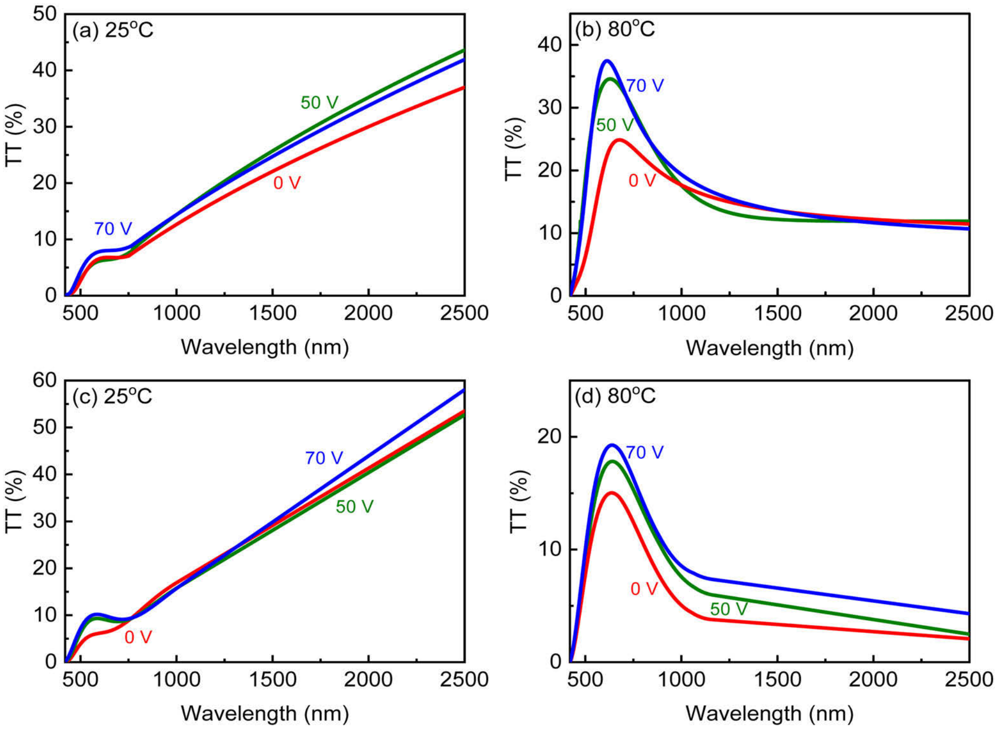

Fig. 9. (a, b) Variation in the total transmittance TT for different voltages 0 V, 50 V and 70 V when the output collected from the VO_2 side at 25 °C and 80 °C respectively. (c, d) TT for the output collected from the LC side at 25 °C and 80 °C respectively.

entrace port. Upon undergoing reflections within the sphere, the signal collected by the detector port is collected by the fiber to the spectrometer.

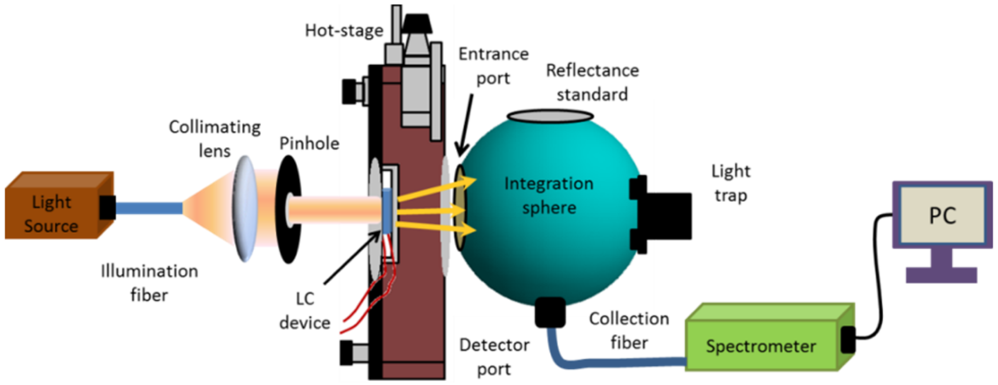

Fig. 10. Schematic of set-up employed for the measurements using integration sphere.

One of the important parameters in determining the performance of switchable devices is the response time. The schematic of experimental set-up employed for the response time measurements is shown in Fig. S5. A low frequency square wave input signal for the amplitude modulation given from the function generator to the amplifier is fed to the LC device for the response time measurements. Rise time and fall times at different voltages for temperatures 25

Optical Materials EXPRESS

°C, 50 °C and 80 °C is shown in Fig. 11(a, b). The rise time is measured between 10% and 90% values during the voltage ON state, while the fall time is measured from 90% and 10% in the voltage OFF state. A decreasing trend is observed in the rise time with the increase in applied voltage. The measured rise and fall times are about 1 ms and 10 ms at 70 V. It is worth mentioning that the fall time at voltages above 10–15 V of 10 ms is much less than the visco-elastic relaxation time. The anchoring of the LC molecules to the walls of Cochleate tubes and the existence of domains prevents the backflow effect and thus responsible for such shorter relaxation times. The LC molecules in the device start to orient above 1 V and then continue to gradually increase without indication of cochleates re-orientation. The only indication of the reorientation of the cochleates is the abrupt reduction in the fall time around 10-15 V which is believed to be result of the formation of cylindrical walls created by cochleates separated by an average distance of around 5 µm (due to the 10% concentration of cochleates with diameter of 500 nm). Since the device thickness is 10 µm, the switching time is expected to reduce by at least factor 4. Additional reductions can be coming from variation in the anchoring conditions. Also worth mentioning that the LC molecules inside the cochleates will be switching at much fast speed because of the small diameter of 500 nm.

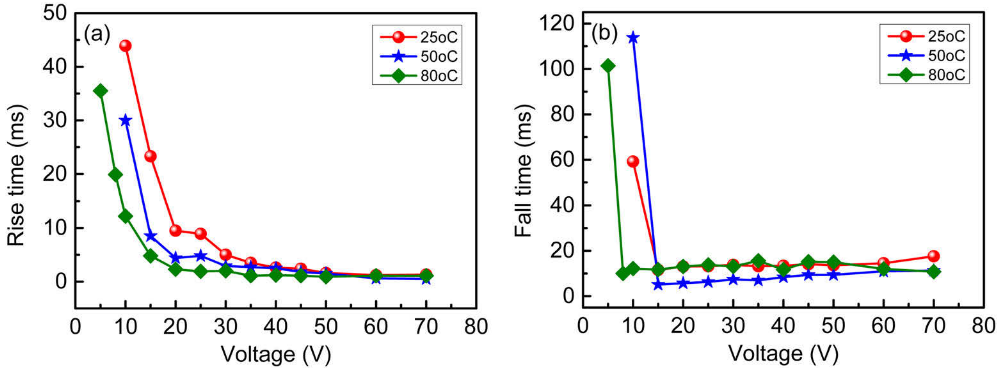

Fig. 11. Device response time to the applied voltage, with a) the rise time and b) fall time measured during the voltage ON and OFF states for different temperatures 25 °C, 50 °C and 80 °C.

4. Conclusions

A true smart window that controls both the infrared light and the visibility is demonstrated based on a hybrid VO_2-LC tunable scattering metamaterial structure. While VO_2 controls the IR transmission as the temperature varies it cannot control the visible light transparency. On the otherhand the LC based layer controls the transparency in the whole spectral range by voltage and temperature however it is based on forward scattering. The combination then is essential in order for the window to act both as privacy window in which the visibility is controlled and to control the heat as an energy saving window. Temperature and voltage dependent transmittance measurements were performed to determine the electro-optic and thermo-optic properties of the device. A non-reciprocity effect is reported in which the electro-optic and thermo-optic properties depend on which side the window is facing the outside world. The reason for the nonreciprocal effect is partially due to the asymmetric lossy structure, however the large effect originates due to the reflectivity dependence of VO_2 on the incidence angle, as when the light incident on the VO_2 side, the reflectivity is mainly that of small angles, while the light is incident from the LC side, due to the forward scattering, many large angles hit the VO_2 and therefore changing the reflected and thus transmitted signals. As a privacy window it can be then recommended to have the LC

Optical Materials EXPRESS

side facing the inside of the room so the window is more transparent for people inside and less when looking from outside. This orientation also gives the higher ΔT_{sol}, which is better in terms of energy consumption. Response time is faster in this system. Further optimization is possible through choosing more transparent glass substrates and ITO coatings, less absorbing LC in the IR, and addition of anti-reflection coatings. Combination of other VO_2 structures that gave better performance can now be combined with LC privacy window to give even better performance. Using fresh VO_2 prepared samples and the device construction immediately before exposing the VO_2 to air will help maintain its thermochromic properties at optimum. The LC layer in this window helps in protecting the VO_2 layer from further oxidation once the device is assembled. In addition the LC-VO_2 interface exhibits less Fresnel reflectivity and this fact is expected to improve the window performance. This device opens up a new possibility of thermochromic VO_2 and LC based systems to satisfy the real-life requirements on smart window applications.

Funding. Ministry of National Infrastructure, Energy and Water Resources; Ministry of Science, Technology and Space; National Research Foundation Singapore.

Acknowledgements. The research is supported partially by The Israel Ministry of National Infrastructure, Energy and Water Resources, and the binational collaboration project supported jointly by the Ministries of Science and Technology of Israel and Taiwan. This research is supported by grants from the National Research Foundation, Prime Minister's Office, Singapore under its Campus of Research Excellence and Technological Enterprise (CREATE) program.

Disclosures. The authors declare no conflict of interest.

Data availability. Data underlying the results presented in this paper are not publicly available at this time but may be obtained from the authors upon reasonable request.

Supplemental document. See Supplement 1 for supporting content.

References

1. A. M. Omer, "Energy, environment and sustainable development," Renewable and Sustainable Energy Reviews **12**(9), 2265–2300 (2008).
2. C. G. Granqvist, "Electrochromics and thermochromics: towards a new paradigm for energy efficient buildings," Materials Today: Proceedings **3**, S2–S11 (2016).
3. H. Kim and S. Yang, "Responsive smart windows from nanoparticle–polymer composites," Adv. Funct. Mater. **30**(2), 1902597 (2020).
4. J. Wang, L. Zhang, L. Yu, Z. Jiao, H. Xie, X. W. Lou, and X. Wei Sun, "A bi-functional device for self-powered electrochromic window and self-rechargeable transparent battery applications," Nat. Commun. **5**(1), 4921 (2014).
5. C. G. Granqvist, "Oxide electrochromics: An introduction to devices and materials," Sol. Energy Mater. Sol. Cells **99**, 1–13 (2012).
6. S. K. Deb, "Opportunities and challenges in science and technology of WO_3 for electrochromic and related applications," Sol. Energy Mater. Sol. Cells **92**(2), 245–258 (2008).
7. V. Wittwer, M. Datz, J. Ell, A. Georg, W. Graf, and G. Walze, "Gasochromic windows," Sol. Energy Mater. Sol. Cells **84**(1-4), 305–314 (2004).
8. W.-L. Jang, Y.-M. Lu, C.-L. Chen, Y.-R. Lu, C.-L. Dong, P.-H. Hsieh, W.-S. Hwang, J.-L. Chen, J.-M. Chen, T.-S. Chan, J.-F. Lee, and W.-C. Chou, "Local geometric and electronic structures of gasochromic VOx films," Phys. Chem. Chem. Phys. **16**(10), 4699 (2014).
9. J. Zheng, S. Bao, and P. Jin, "TiO_2(R)/VO_2(M)/TiO_2(A) multilayer film as smart window: Combination of energy-saving, antifogging and self-cleaning functions," Nano Energy **11**, 136–145 (2015).
10. T. Chang, X. Cao, N. Li, S. Long, X. Gao, L. R. Dedon, G. Sun, H. Luo, and P. Jin, "Facile and low-temperature fabrication of thermochromic Cr_2O_3/VO_2 smart coatings: enhanced solar modulation ability, high luminous transmittance and uv-shielding function," ACS Appl. Mater. Interfaces **9**(31), 26029–26037 (2017).
11. N. Li, Y. Li, G. Sun, Y. Zhou, S. Ji, H. Yao, X. Cao, S. Bao, and P. Jin, "Enhanced photochromic modulation efficiency: a novel plasmonic molybdenum oxide hybrid," Nanoscale **9**(24), 8298–8304 (2017).
12. N. Li, Y. Li, W. Li, S. Ji, and P. Jin, "One-step hydrothermal synthesis of TiO_2 @MoO_3 core–shell nanomaterial: microstructure, growth mechanism, and improved photochromic property," J. Phys. Chem. C **120**(6), 3341–3349 (2016).
13. S. Hoffmann, E. S. Lee, and C. Clavero, "Examination of the technical potential of near-infrared switching thermochromic windows for commercial building applications," Sol. Energy Mater. Sol. Cells **123**, 65–80 (2014).
14. F. J. Morin, "Oxides which show a metal-to-insulator transition at the Neel temperature," Phys. Rev. Lett. **3**(1), 34–36 (1959).
15. M. Taha, S. Walia, T. Ahmed, D. Headland, W. Withayachumnankul, S. Sriram, and M. Bhaskaran, "Insulator–metal transition in substrate-independent VO_2 thin film for phase-change devices," Sci. Rep. **7**(1), 17899 (2017).

Optical Materials EXPRESS

16. Y. Cui, Y. Ke, C. Liu, Z. Chen, N. Wang, L. Zhang, Y. Zhou, S. Wang, Y. Gao, and Y. Long, "Thermochromic VO_2 for energy-efficient smart windows," Joule **2**(9), 1707–1746 (2018).
17. X. P. Zhao, S. A. Mofid, T. Gao, G. Tan, B. P. Jelle, X. B. Yin, and R. G. Yang, "Durability-enhanced vanadium dioxide thermochromic film for smart windows," Materials Today Physics **13**, 100205 (2020).
18. H. Liu, D. Wan, A. Ishaq, L. Chen, B. Guo, S. Shi, H. Luo, and Y. Gao, "Sputtering deposition of sandwich-structured V_2O_5 /Metal (V, W)/ V_2O_5 multilayers for the preparation of high-performance thermally sensitive VO_2 thin films with selectivity of VO_2 (B) and VO_2 (M) polymorph," ACS Appl. Mater. Interfaces **8**(12), 7884–7890 (2016).
19. N. R. Mlyuka, G. A. Niklasson, and C. G. Granqvist, "Thermochromic VO_2 -based multilayer films with enhanced luminous transmittance and solar modulation," phys. stat. sol. (a) **206**(9), 2155–2160 (2009).
20. T. Chang, X. Cao, L. R. Dedon, S. Long, A. Huang, Z. Shao, N. Li, H. Luo, and P. Jin, "Optical design and stability study for ultrahigh-performance and long-lived vanadium dioxide-based thermochromic coatings," Nano Energy **44**, 256–264 (2018).
21. Y. Chen, X. Zeng, J. Zhu, R. Li, H. Yao, X. Cao, S. Ji, and P. Jin, "High performance and enhanced durability of thermochromic films using VO_2 @ZnO core–shell nanoparticles," ACS Appl. Mater. Interfaces **9**(33), 27784–27791 (2017).
22. Y. Ke, X. Wen, D. Zhao, R. Che, Q. Xiong, and Y. Long, "Controllable fabrication of two-dimensional patterned VO_2 nanoparticle, nanodome, and nanonet arrays with tunable temperature-dependent localized surface plasmon resonance," ACS Nano **11**(7), 7542–7551 (2017).
23. M. Li, H. Wu, L. Zhong, H. Wang, Y. Luo, and G. Li, "Active and dynamic infrared switching of VO_2 (M) nanoparticle film on ITO glass," J. Mater. Chem. C **4**(8), 1579–1583 (2016).
24. S.-Y. Li, G. A. Niklasson, and C. G. Granqvist, "Thermochromic fenestration with VO_2-based materials: Three challenges and how they can be met," Thin Solid Films **520**(10), 3823–3828 (2012).
25. Y.-X. Ji, S.-Y. Li, G. A. Niklasson, and C. G. Granqvist, "Durability of thermochromic VO_2 thin films under heating and humidity: Effect of Al oxide top coatings," Thin Solid Films **562**, 568–573 (2014).
26. I. Balin, S. Wang, P. Wang, Y. Long, and I. Abdulhalim, "Enhanced transition-temperature reduction in a half-sphere au/ vo_2 core-shell structure: local plasmonics versus induced stress and percolation effects Igal," Phys. Rev. Appl. **11**(3), 034064 (2019).
27. I. Balin, V. Garmider, Y. Long, and I. Abdulhalim, "Training artificial neural network for optimization of nanostructured VO_2 -based smart window performance," Opt. Express **27**(16), A1030 (2019).
28. S. Bhupathi, S. Wang, M. Abutoama, I. Balin, L. Wang, P. G. Kazansky, Y. Long, and I. Abdulhalim, "Femtosecond laser-induced vanadium oxide metamaterial nanostructures and the study of optical response by experiments and numerical simulations," ACS Appl. Mater. Interfaces **12**(37), 41905–41918 (2020).
29. C. Liu, I. Balin, S. Magdassi, I. Abdulhalim, and Y. Long, "Vanadium dioxide nanogrid films for high transparency smart architectural window applications," Opt. Express **23**(3), A124 (2015).
30. Y. Ke, I. Balin, N. Wang, Q. Lu, A. I. Y. Tok, T. J. White, S. Magdassi, I. Abdulhalim, and Y. Long, "Two-dimensional SiO_2/VO_2 photonic crystals with statically visible and dynamically infrared modulated for smart window deployment," ACS Appl. Mater. Interfaces **8**(48), 33112–33120 (2016).
31. E. P. Pozhidaev, A. V. Kaznacheev, S. I. Torgova, V. V. Kesaev, and V. A. Barbashov, "Polymer dispersed liquid crystals with electrically controlled light scattering in the visible and near-infrared ranges," Opt. Mater. Express **10**(12), 3030 (2020).
32. N. Nasir, H. Hong, M. A. Rehman, S. Kumar, and Y. Seo, "Polymer-dispersed liquid-crystal-based switchable glazing fabricated via vacuum glass coupling," RSC Adv. **10**(53), 32225–32231 (2020).
33. J. R. Talukder, Y.-H. Lee, and S.-T. Wu, "Photo-responsive dye-doped liquid crystals for smart windows," Opt. Express **27**(4), 4480 (2019).
34. H. Sun, Z. Xie, C. Ju, X. Hu, D. Yuan, W. Zhao, L. Shui, and G. Zhou, "Dye-doped electrically smart windows based on polymer-stabilized liquid crystal," Polymers **11**(4), 694 (2019).
35. E. Castellón and D. Levy, "Smart windows based on liquid crystal dispersions," in *Transparent Conductive Materials* (Wiley-VCH Verlag GmbH & Co. KGaA, 2018), pp. 337–365.
36. S.-H. Chung and H. Y. Noh, "Polymer-dispersed liquid crystal devices with graphene electrodes," Opt. Express **23**(25), 32149 (2015).
37. I. C. Kim, T.-H. Kim, S. H. Lee, and B.-S. Kim, "Extremely foldable and highly transparent nanofiber-based electrodes for liquid crystal smart devices," Sci. Rep. **8**(1), 11517 (2018).
38. H. Hosseinzadeh Khaligh, K. Liew, Y. Han, N. M. Abukhdeir, and I. A. Goldthorpe, "Silver nanowire transparent electrodes for liquid crystal-based smart windows," Sol. Energy Mater. Sol. Cells **132**, 337–341 (2015).
39. I. Abdulhalim, P. L. Madhuri, M. Diab, and T. Mokari, "Novel easy to fabricate liquid crystal composite with potential for electrically or thermally controlled transparency windows," Opt. Express **27**(12), 17387 (2019).
40. L. M. Pappu, R. J. Martin-Palma, B. Martín-Adrados, and I. Abdulhalim, "Voltage controlled scattering from porous silicon Mie-particles in liquid crystals," J. Mol. Liq. **281**, 108–116 (2019).
41. . Shuddhodana, P. W. K. Wong and Z. Judeh, "Continuous, high-throughput production of artemisinin-loaded supramolecular cochleates using simple off-the-shelf flow focusing device," Materials Science and Engineering: C **108**, 110410 (2020).

Optical Materials EXPRESS

42. P. L. Madhuri, Shuddhodana, Z. M. A. Judeh, and I. Abdulhalim, "Cochleate-doped liquid crystal as switchable metamaterial window mediated by molecular orientation modified aggregation," Part. Part. Syst. Charact. **37**(5), 2000067 (2020).
43. K. Sayyah, "One-dimensional equivalent circuit simulation of a photodiode-based spatial light modulator," IEEE Trans. Electron Devices **43**(12), 2101–2108 (1996).
44. Z. Yang, C. Ko, V. Balakrishnan, G. Gopalakrishnan, and S. Ramanathan, "Dielectric and carrier transport properties of vanadium dioxide thin films across the phase transition utilizing gated capacitor devices," Phys. Rev. B **82**(20), 205101 (2010).
45. X. Liu, G. Xie, C. Huang, Q. Xu, Y. Zhang, and Y. Luo, "A facile method for preparing VO_2 nanobelts," Mater. Lett. **62**(12-13), 1878–1880 (2008).
46. O. Monfort, T. Roch, L. Satrapinskyy, M. Gregor, T. Plecenik, A. Plecenik, and G. Plesch, "Reduction of V_2O_5 thin films deposited by aqueous sol–gel method to VO_2(B) and investigation of its photocatalytic activity," Appl. Surf. Sci. **322**, 21–27 (2014).
47. Y. Li, J.-L. Kuang, Y. Lu, and W.-B. Cao, "Facile synthesis, characterization of flower-like vanadium pentoxide powders and their photocatalytic behavior," Acta Metall. Sin. (Engl. Lett.) **30**(10), 1017–1026 (2017).
48. H. W. Verleur, A. S. Barker, and C. N. Berglund, "Optical properties of VO_2 between 0.25 and 5 eV," Phys. Rev. **172**(3), 788–798 (1968).
49. T. Smith and J. Guild, "The C.I.E. colorimetric standards and their use," Trans. Opt. Soc. **33**(3), 73–134 (1932).
50. "American Society for Testing and Materials, "ASTM G173-03 reference spectra,"" https://www.nrel.gov/grid/solar-resource/spectra-am1.5.html.
51. I. Abdulhalim, "Analytic propagation matrix method for anisotropic magneto-optic layered media," J. Opt. A: Pure Appl. Opt. **2**(6), 557–564 (2000).

CCC RightsLink®

Home | Help | Email Support | Sign in | Create Account

ACS Publications
Most Trusted. Most Cited. Most Read.

Dual-Band Modulation of Visible and Near-Infrared Light Transmittance in an All-Solution-Processed Hybrid Micro–Nano Composite Film

Author: Xiao Liang, Mei Chen, Shumeng Guo, et al

Publication: Applied Materials

Publisher: American Chemical Society

Date: Nov 1, 2017

Copyright © 2017, American Chemical Society

PERMISSION/LICENSE IS GRANTED FOR YOUR ORDER AT NO CHARGE

This type of permission/license, instead of the standard Terms and Conditions, is sent to you because no fee is being charged for your order. Please note the following:

- Permission is granted for your request in both print and electronic formats, and translations.
- If figures and/or tables were requested, they may be adapted or used in part.
- Please print this page for your records and send a copy of it to your publisher/graduate school.
- Appropriate credit for the requested material should be given as follows: "Reprinted (adapted) with permission from {COMPLETE REFERENCE CITATION}. Copyright {YEAR} American Chemical Society." Insert appropriate information in place of the capitalized words.
- One-time permission is granted only for the use specified in your RightsLink request. No additional uses are granted (such as derivative works or other editions). For any uses, please submit a new request.

If credit is given to another source for the material you requested from RightsLink, permission must be obtained from that source.

BACK CLOSE WINDOW

© 2022 Copyright - All Rights Reserved | Copyright Clearance Center, Inc. | Privacy statement | Terms and Conditions
Comments? We would like to hear from you. E-mail us at customercare@copyright.com

ACS APPLIED MATERIALS & INTERFACES
Research Article
Cite This: ACS Appl. Mater. Interfaces 2017, 9, 40810-40819
www.acsami.org

Dual-Band Modulation of Visible and Near-Infrared Light Transmittance in an All-Solution-Processed Hybrid Micro–Nano Composite Film

Xiao Liang,[†] Mei Chen,[†] Shumeng Guo,[‡] Lanying Zhang,[†] Fasheng Li,[§] and Huai Yang*,[†]

[†]Department of Materials Science and Engineering, College of Engineering, Peking University, Beijing 100871, People's Republic of China

[‡]Department of Materials Physics and Chemistry, School of Materials Science and Engineering, University of Science and Technology Beijing, Beijing 100083, People's Republic of China

[§]Department of Chemistry, Dalian Medical University, Dalian 116044, People's Republic of China

Supporting Information

Downloaded via HARBIN INST OF TECHNOLOGY on September 19, 2022 at 09:13:17 (UTC).
See https://pubs.acs.org/sharingguidelines for options on how to legitimately share published articles.

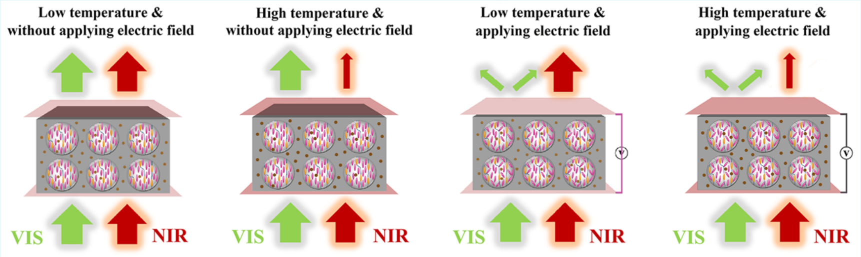

ABSTRACT: Smart windows with controllable visible and near-infrared light transmittance can significantly improve the building's energy efficiency and inhabitant comfort. However, most of the current smart window technology cannot achieve the target of ideal solar control. Herein, we present a novel all-solution-processed hybrid micronano composite smart material that have four optical states to separately modulate the visible and NIR light transmittance through voltage and temperature, respectively. This dual-band optical modulation was achieved by constructing a phase-separated polymer framework, which contains the microsized liquid crystals domains with a negative dielectric constant and tungsten-doped vanadium dioxide (W-VO_2) nanocrystals (NCs). The film with 2.5 wt % W-VO_2 NCs exhibits transparency at normal condition, and the passage of visible light can be reversibly and actively regulated between 60.8% and 1.3% by external applied voltage. Also, the transmittance of NIR light can be reversibly and passively modulated between 59.4% and 41.2% by temperature. Besides, the film also features easy all-solution processability, fast electro-optical (E-O) response time, high mechanical strength, and long-term stability. The as-prepared film provides new opportunities for next-generation smart window technology, and the proposed strategy is conductive to engineering novel hybrid inorganic–organic functional matters.

KEYWORDS: *near-infrared and visible-light transmittance, dual-band control, liquid crystals, tungsten-doped vanadium dioxide nanocrystals, polymer structure*

1. INTRODUCTION

With the continual increase of electricity cost and the global temperature, it becomes more and more difficult to keep a pleasant and environmental-friendly atmosphere in the architectural environment.[1−3] In recent years, people have paid close attention to control the passage of light via transparent glass windows (e.g., in the office building). Meanwhile, multiple techniques have been used to cope with the heat into such spaces as well as the influx of light.[4−9] Compared with the cumbersome physical obstacles (e.g., curtains, blinds, or awnings), smart windows can intelligently tune the transmittance of solar irradiation according to the weather conditions and personal preference. Overall, they are not only much more elegant in aesthetics and convenient in control,[10] but also play a significant role in improving the building's energy efficiency and inhabitant comfort.[11−13]

In general, an ideal smart window should independently control the passage of visible sunlight and the near-infrared (NIR) irradiation into a building[14,15] so that it can be universally applicable across different climate zones and personal preferences. As is well-known, the invisible NIR light carries about half of the solar energy.[2] Thus, it may be more appropriate to employ materials that can passively reject the unwanted NIR light in high temperature periods but intelligently allow the passage of NIR light in a cold weather. Equally, controlling the passage of visible

Received: August 4, 2017
Accepted: November 2, 2017
Published: November 2, 2017

© 2017 American Chemical Society

Scheme 1. Four Optical Modulation Modes Realized in Hybrid Micro−Nano Composite Film[a]

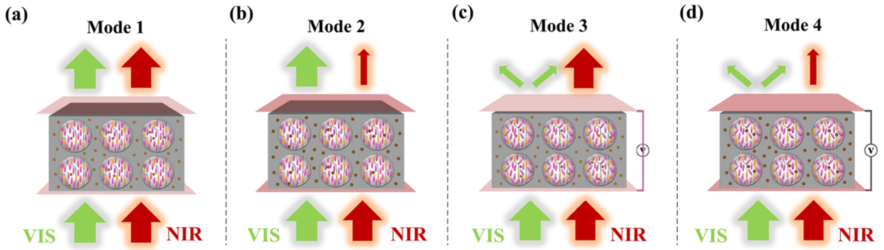

[a](a) Mode 1: the film allows the passage of both visible and NIR light with no voltage applied at low environmental temperature. (b) Mode 2: the film allows the passage of visible light and rejects most NIR light with no voltage applied at high environmental temperature. (c) Mode 3: the film strongly scatters the visible light and allows the passage of NIR light by applying a voltage at low environmental temperature. (d) Mode 4: the film strongly scatters the visible light and rejects the most NIR light by applying a voltage at high environmental temperature.

light actively is also important to block the intensive sunlight on scorching days or to prevent the people outside seeing directly inside the buildings for privacy protection. Apart from this dual-band solar control, other crucial considerations, such as long-term stability, low-cost processability, fast respond time, high mechanical strength, and being transparent at normal condition, should also be considered in the material design for practical application.[16,17]

Nowadays, tremendous efforts have been taken to realize this dual-band modulation of solar irradiation. Approaches based on electro-,[11,18,19] thermo-,[20,21] gaso-[22,23] and photochromogenic[24,25] materials represent promising progress for creating the smart windows with excellent properties. Among them, the electrochromic devices can achieve the largest projected energy savings by offering the widest range of solar control.[26] However, most of the smart windows based on electrochromism can only either selectively block the NIR light or control the passage of NIR and visible light simultaneously.[27,28] Moreover, the visible transmittance in most electrochromic devices still maintains a clear version during the solar modulation,[27] which means that the physical obstacles (e.g., curtains, blinds or awnings) are still required for privacy protection in buildings equipped with these electrochromic windows. Also, the solar regulation in electrochromic devices is realized in an active mode, but sometimes a passive mode (e.g., solar control via temperature) in controlling the invisible NIR light will be much more desirable.

Recent research indicates that hybrid systems for smart windows show much more enhanced performances compared with other approaches.[29] Herein, we promote an all-solution-processed hybrid micronano composite material that have four different optical states in response to temperature and external voltage to separately modulate both the visible and NIR light transmittance. The film possesses a porous polymer morphology and has a thickness of ~30 μm. In this porous framework, microsized nematic liquid crystals (LCs) droplets with a negative dielectric constant and tungsten-doped vanadium dioxide (W-VO_2) nanocrystals (NCs) are homogeneously dispersed in as shown in Scheme 1. Unlike the traditional polymer dispersed liquid crystals (PDLCs) film,[30−32] this material is transparent at normal condition. Moreover, visible light transmittance of the film can be tuned actively and reversibly from transparency to opaqueness by applying a voltage to control the orientations of LCs within the microsized droplets. Also, the modulation of NIR light transmittance is realized passively by the thermochromic behavior of W-VO_2 NCs during the reversible metal-to-insulator transition (MIT) at near room temperature. In this way, four optical modulation modes can be realized in this hybrid film to separately control the passage of both visible (actively) and NIR (passively) light according to different stimuli, as shown from Scheme 1a−d. This hybrid micro−nano material can not only enlighten insights into engineering novel functional organic−inorganic matters, but also provide new opportunities for the next-generation smart window technology.

2. EXPERIMENTAL METHODS

2.1. Materials. Unless specified, the chemicals were purchased from Sigma-Aldrich (St. Louis, MO). E7, used as LCs with a positive dielectric constant (P-LCs), was purchased from Shijiazhuang Yongsheng Huatsing Liquid Crystal Co., Ltd. Photoinitiator, Irgacure 651, was obtained from TCI Co., Ltd. Hydroxypropyl methacrylate (HPMA) and dodecyl methacrylate (LMA) were purchased from Tokyo Chemical Industry Co., Ltd. Polyethylene glycol diacrylate (PEGDA600) was provided by Sartomer Co., Ltd. Liquid-crystalline acrylate monomers (LAMs), C6M, were synthesized according to the methods suggested by Broer et al.[33] and Gray et al.[34] LC-0518#, used as LCs with a negative dielectric constant (N-LCs), were homemade LCs mixture with a negative dielectric constant suggested by the literature.[35] The glass beads and the conducting PET films were purchased from Sekisui Chemical Co., Ltd. and Kangdexin Composite Material Co., Ltd., respectively. The chemical structures and some physical properties of chemicals used were provided in Scheme S1.

2.2. Synthesis of W-VO_2 NCs. The W-VO_2 NCs were synthesized using the thermolysis method.[36,37] First, 9.6 mL of concentrated hydrochloric acid and 1 g of hydrazine hydrogen chloride were alternately introduced into a 7 mL of aqueous suspension containing 5.46 g of vanadium pentoxide under continuous stirring until a blue mixture was obtained. After that, the blue mixture was filtered to obtain the clear vanadyl chloride ($VOCl_2$) solution. Then the $VOCl_2$ solution was dropwise added into a 20 mL of aqueous suspension containing 0.306 g of tungstic acid and 15 g of ammonium bicarbonate NH_4HCO_3 with stirring under nitrogen protection to produce the violet precursor. Next, the precursor was filtered, washed with a saturated NH_4HCO_3 solution for four times, and dried under vacuum in an oven. Finally, the violet precursor was heated in a tube furnace under a flow of nitrogen at 500 °C for 0.5 h to obtain the W-VO_2 NCs.

2.3. PVP Functionalized W-VO_2 NCs. As-made W-VO_2 NCs were functionalized by PVP by the following procedures. Typically, 15 mg of the as-made W-VO_2 NCs was first dispersed into 2 mL of deionized water under continuous ultrasonication for 10 min. In the meantime, 8 mg of PVP was dissolved in 2 mL of deionized water by ultrasonicating the solution for 15 min. Subsequently, the PVP and the colloidal solutions were mixed under continuous stirring for 24 h. Then the W-VO_2 NCs coated with PVP were collected by centrifugation and rinsed with deionized water to remove residual PVP molecules. Finally, the PVP coated W-VO_2 NCs were obtained by repeating the above procedure three times.

2.4. Preparation of Hybrid Smart Films Containing W-VO_2/PVP NCs. A syrup containing P-LCs, E7, isotropic acrylate monomers (IAMs), HPMA, LMA, ethoxylate dimethacrylate (Bis-EMA15), LAMs,

DOI: 10.1021/acsami.7b11582
ACS Appl. Mater. Interfaces 2017, 9, 40810−40819

C6M, photoinitiator, Irgacure 651, and W-VO_2/PVP NCs was prepared and mixed homogeneously. An additional 0.5 wt % of 30 μm nonreactive glass bead spacers (made up of polystyrene) was added to adjust the thickness of the films. The hybrid smart films were prepared according to the procedures in Scheme 2. In detail, the syrup was first sandwiched

Scheme 2. Schematic Depictions of Preparation Procedures for Hybrid Micro−Nano Composite Film[a]

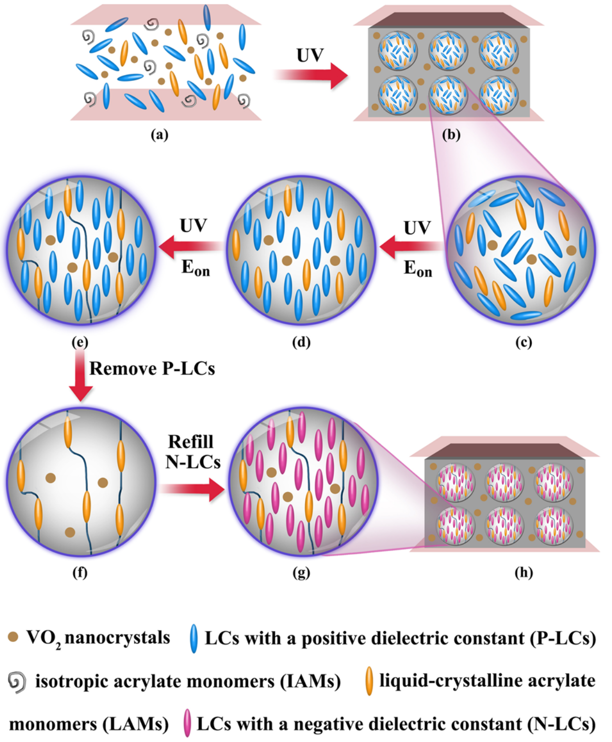

[a](a) Side view of the polymeric syrup containing W-VO_2 before polymerization. (b) Porous structure was formed after a short time of UV irradiation. (c) Enlarged P-LCs domains within the porous structure. (d) Voltage was applied to align the long-axis directions of P-LCs molecules perpendicularly to the substrates. (e) HALPNs were formed after further irradiating the film by UV exposure with the voltage applied simultaneously. (f) P-LCs were extracted by dipping the film into cyclohexane. (g) N-LCs were homeotropically aligned induced by the HALPNs. (h) Final structure of the hybrid micro−nano composite film.

between the two pieces of transparent and conductive surfaces. These conducting surfaces we used were thin layers of tin doped indium oxides (ITO) coated on the inner surfaces of polyester (PET) substrates. After that, the cell was irradiated under UV light (PS135, UV Flood, Stockholm Sweden, 365 nm) with an intensity of 0.5 mw/cm^2 for 90 s at room temperature. Then a voltage (square wave, 50 Hz, 150 V) was applied to oriented the long-axis molecular directions the P-LCs perpendicularly to the surface of the conductive substrate. At the same time, the film was irradiated by UV light again with an intensity of 0.5 mw/cm^2 for an additional 5 min at room temperature to complete the polymerization. Subsequently, the P-LCs were completely removed out of the film by soaking with cyclohexane for 6 days (P-LCs molecules could move from inside film to outside cyclohexane solvent driven by the P-LCs concentration gradient[38,39]). Then, after the film was dried under vacuum for 2 h, N-LCs, LC-0518#, were refilled into the film under vacuum by capillary action and the hybrid smart film with dual-band optical modulation was prepared.

2.5. Measurements. TEM observation was carried out on a Tecnai G2 F30 microscope operating at 200 kV. XRD patterns of W-VO_2 NCs were obtained on a Bruker AXS D8-Advanced diffractometer with Cu Kα radiation (λ = 1.5418 Å). The composition and surface chemistry of W-VO_2 NCs were studied by an energy-dispersive X-ray (EDX) spectrometer attached to a scanning electron microscopy (SEM, HITACHI S-4800) and X-ray photoelectron spectroscopy (XPS, Thermo escalab 250XI), respectively. The zeta potentials of the sample were determined by a Zeta potential analyzer (Zeta PALS BI-90 Plus, Brookhaven Instruments). FT-IR spectra were measured on a BrukerVector-22 FT-IR spectrophotometer using a KBr pellet over a range of 4000−600 cm^{-1}. The phase transition behavior of W-VO_2 NCs was characterized by A PerkinElmer DSC8000 with a mechanical refrigerator under nitrogen protection at a heating and cooling rate of 10 °C/min. The morphologies of the polymer framework after the stepwise polymerization were characterized by scanning electron microscopy (SEM, HITACHI S-4800). To observe the polymer framework, LCs molecules were first extracted by dipping the films in cyclohexane for at least 4 days at ambient temperature. After that, the films were dried under vacuum for about 2 h to eliminate the cyclohexane. At last, thin layers of gold were deposited onto the films to increase conductivity. The vis/NIR spectra and the thermo-optical transmittance were obtained by a UV−vis/NIR spectrophotometer (JASCO V-570) equipped with a hot stage (Linkam LK-600PM) calibrated to an accuracy of ±0.1 K. Electro-optical (E-O) properties of the hybrid smart films were measured by a LC device parameters tester (LCT-5016C, Changchun Liancheng Instrument Co. Ltd., China) equipped with a hot stage (Linkam LK-600PM). A halogen tungsten lamp (550 nm) was applied as the source of incident light, and a photodiode was used to record the transmittances of the as-made films. The distance between the films and the detector was about 300 mm. During the measurements, a voltage (square wave) with a frequency of 50 Hz was applied to change the transmittance of the films. The shearing strength was measured by universal tensile testing machine (LETRY) at the rate of 10 mm min^{-1} and the size of the tested films was 20 mm × 10 mm. The integral luminous transmittance T_{lum} (380−780 nm) and T_{sol} (250−2500 nm) were calculated by eq 1:

$$T_{lum/sol} = \frac{\int \varphi_{lum/sol}(\lambda) T(\lambda)\, d\lambda}{\int \varphi_{lum/sol}(\lambda)\, d\lambda} \quad (1)$$

where φ_{lum} (λ) denotes the standard luminous efficiency function of photopic vision[40] from 380−780 nm, and φ_{sol} (λ) refers to the IR/solar irradiance spectrum distribution for air mass 1.5 (corresponding to the sun standing 37 °C above the horizon with 1.5 atm thickness and the presence of a solar zenith angel of = 48.2).[41] $T(\lambda)$ is the tested spectral transmittance. ΔT_{sol} is determined by $\Delta T_{sol} = T_{sol(1)} - T_{sol(2)}$. Transmittance contrast (TC) is obtained by TC = %$T_{off\text{-}voltage}$ − %$T_{on\text{-}voltage}$.[42]

3. RESULTS AND DISCUSSION

A stepwise UV polymerization strategy promoted in our previous work[17] was applied to prepare the smart film. As shown in Scheme 2, first, a homogeneous polymeric syrup containing LCs with a positive dielectric constant (P-LCs), isotropic acrylate monomers (IAMs), liquid-crystalline acrylate monomers (LAMs), and W-VO_2 NCs was sandwiched between two plastic conductive substrates (Scheme 2a). After that, IAMs and LAMs within the film were partially polymerized through irradiating the film by UV light for ∼90 s, and a preliminary phase-separated P-LCs/polymer structure was formed as shown in Scheme 2b and c. Subsequently, the long-axis molecular directions of P-LCs and LAMs were perpendicularly oriented by applying a voltage of 150 V(Scheme 2d). In the meantime, cross-linking among LAMs was completed by further irradiating the film under UV light, which formed a homeotropically aligned liquid−crystalline polymer network (HALPN) in the microdomains of the porous matrix (Scheme 2e). Next, the film was dipped into cyclohexane to fully extract the P-LCs (Scheme 2f) and then refilled using LCs with a negative dielectric constant (N-LCs) (Scheme 2g) under vacuum by capillary action. Afterward, the smart film with

DOI: 10.1021/acsami.7b11582
ACS Appl. Mater. Interfaces 2017, 9, 40810−40819

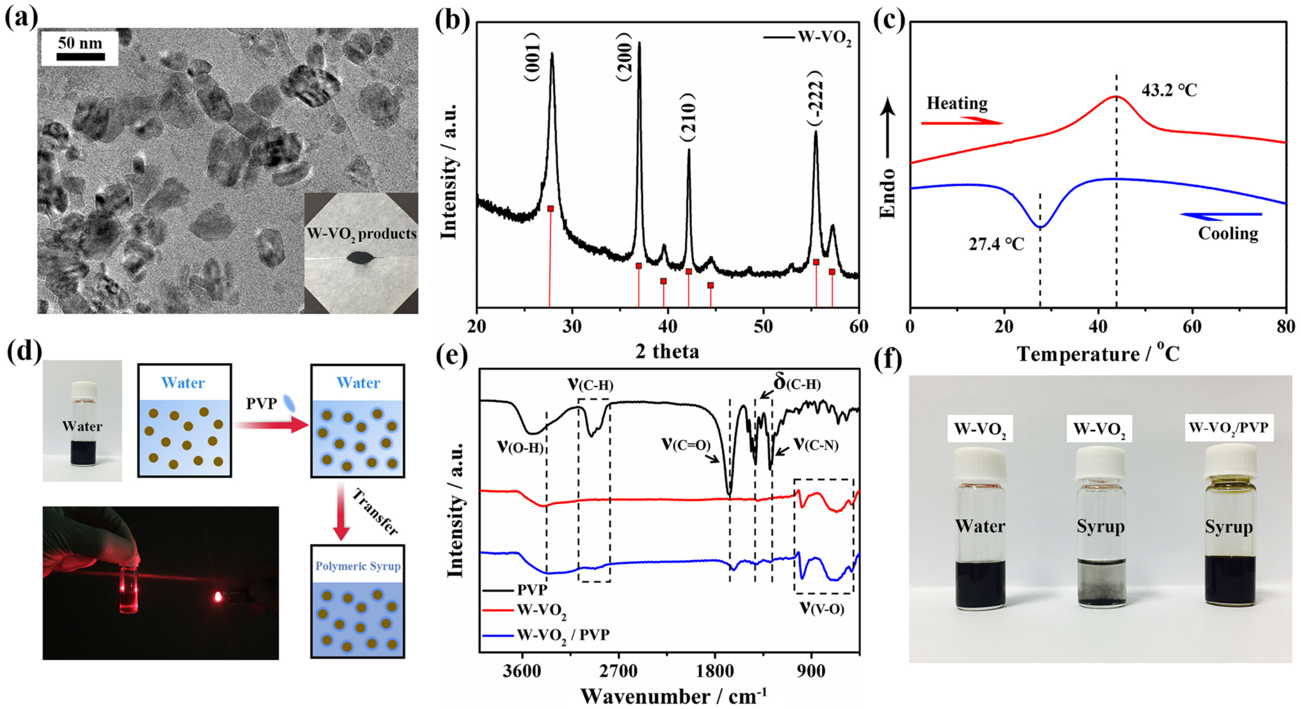

Figure 1. (a) TEM photograph (insert: photograph of the synthesized W-VO_2 products) of the W-VO_2 NCs. (b) XRD pattern and (c) DSC curves of the as-made W-VO_2 NCs. (d) Schematic illustration of the surface modification procedure of the as-made W-VO_2 NCs, and the Tyndall effect of W-VO_2/PVP NCs within the mixture of P-LCs and polymerizable monomers. (e) FT-IR spectra of W-VO_2/PVP NCs (blue curve), W-VO_2 NCs (red curve), and PVP (black curve), respectively. (f) Digital photographs showing the dispersibility of the pristine W-VO_2 NCs dispersed in water and the syrup, respectively, and the PVP functionalized W-VO_2 NCs dispersed in the syrup.

dual-band optical modulation was prepared as shown in Scheme 2h. In this film, the HALPN within the N-LCs microdroplets plays a critical role in inducing the N-LCs to be homeotropically aligned after it was refilled into the film. Because the refractive index (RI) values of the long-molecule-axis of N-LCs and polymer were well-matched, the film displays a transparent appearance at normal condition. Furthermore, through the application of a voltage, these N-LCs tend to be aligned parallel across the direction of the generated electric field, which will thereby generate a spatially varied RI between these microsized N-LCs domains and polymer and turn the film from transparency to strong light-scattering. Thus, by applying different voltages, the visible light transmittance of the film can be actively and dynamically modulated. Moreover, nanocomponents of W-VO_2 within the film undergo a drastic change of NIR optical properties during the phase transition at near room temperature, making the NIR light transmittance of the film thermally switchable in a passive way.

W-VO_2 NCs were synthesized according to the thermolysis method.[36,37] The TEM image in Figure 1a shows the morphology of the as-made W-VO_2 NCs products and the sizes of most of the synthesized NCs is below 50 nm. The X-ray diffraction (XRD) pattern measured at 20 °C in Figure 1b shows the monoclinic structure of VO_2 (M) phase (JCPDS No. 72−0514), without impurity peaks. Besides, differential scanning calorimetry (DSC) measurement in Figure 1c indicates the as-made W-VO_2 NCs have a reduced MIT temperature of ~43.2 °C (heating cycle) compared with the bulk VO_2, 68 °C.[6,20,21] Moreover, the chemical composition of the W-VO_2 NCs products was determined by energy-dispersive X-ray (EDX) analysis and X-ray photoelectron spectroscopy (XPS) measurement. EDX results in Figure S1 show that the elements W, V, and O were all detected, and the composition of the W-VO_2 is shown in Table S1, indicating that the concentration of W was 1.32 atom %. The surface composition of the W-VO_2 products was revealed from XPS results in Figure S2. Four elements, C, W, O, V, were detected from the XPS measurement, which further confirmed the composition of the W-VO_2 products. The carbon peak could be resulted from the surface contamination.[36] These results are consistent with the previous works of W-VO_2 NCs.[36,37,43−45]

Prior to the preparation of the film, achieving a homogeneous distribution of W-VO_2 NCs within the polymeric syrup should be in the first place. However, the as-made W-VO_2 NCs spontaneously form large aggregates (Figure 1f) within the mixture containing acrylate monomers and LCs probably due to the reason that the surface electric double layers of W-VO_2 NCs were compressed after the phase transfer, which will lead to a great loss of transparency and poor mechanical properties.[46] Therefore, rendering a homogeneous dispersion of W-VO_2 NCs within the film is essential for practical applications. The synthesized W-VO_2 NCs were found to be well-dispersed in water (Figure 1d) resulted from the high zeta-potential value of about −43.3 mV as shown in Figure S3. Taking advantage of the good dispersibility of the W-VO_2 NCs in aqueous medium, we promote a facile surface-treatment strategy by functionalizing the as-made W-VO_2 NCs with poly(vinylpyrrolidone) (PVP) in water, as shown in Figure 1d (details are provided in the Experimental Methods). As is well-known, this amphiphilic and nonionic polymer can not only absorb onto a broad range of different materials, including many metal oxides,[47] but also has multiple polar functional groups (e.g., carbanyl and amide). Thus, the coated PVP layer will create a highly compatible interface between the polar syrup containing LCs and W-VO_2 NCs and render a homogeneous W-VO_2 NCs dispersion.

DOI: 10.1021/acsami.7b11582
ACS Appl. Mater. Interfaces 2017, 9, 40810−40819

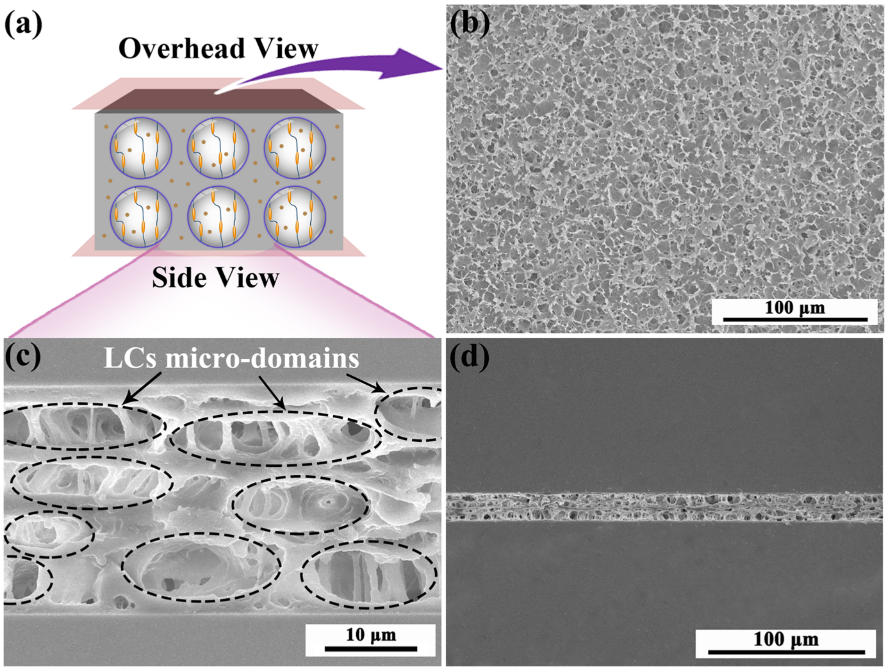

Figure 2. (a) Schematic diagram of the porous polymer framework within the film. SEM observation of the morphology of the polymer framework from (b) the overhead view and (c) the side view. (d) Enlarged version of the SEM photograph of the polymer framework from the side view.

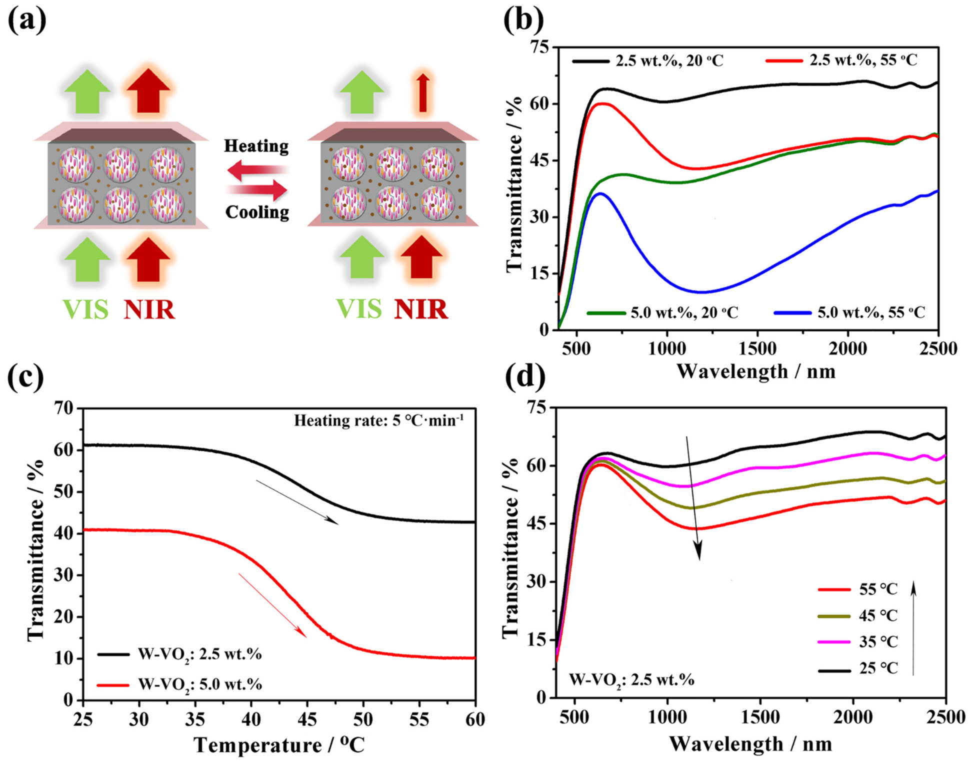

Figure 3. (a) Schematic illustration of the optical behaviors of the as-prepared film upon the heating or cooling the film. (b) Vis/NIR transmittance spectra from 400 nm to ~2500 nm for the as-prepared films with 2.5 and 5.0 wt % W-VO_2 NCs incorporated at 20 and 55 °C, respectively. (c) NIR light transmittance at 1150 nm of the films containing 2.5 and 5.0 wt % during the heating process from 20 to 55 °C (heating rate: 5 °C/min). (d) Dynamic variations of the vis/NIR transmittance spectra from 400 nm to ~2500 nm for the film with 2.5 wt % W-VO_2 NCs incorporated during the heating process from 20 to 55 °C.

The success of the promoted PVP surface treatment was confirmed by FT-IR spectrum, as shown in Figure 1e. In the black curve of PVP, five distinct peaks, ascribed to the stretch vibrational bands of $\nu_{(O-H)}$, $\nu_{(C-H)}$, $\nu_{(C=O)}$, and $\nu_{(C-N)}$ at 3490 cm^{-1}, 2938−2854 cm^{-1}, 1655 cm^{-1}, and 1290 cm^{-1}, and the flexural vibrational bands of $\delta_{(C-H)}$ at around 1423 cm^{-1}, were detected. After modifying the surfaces of W-VO_2 NCs with PVP, the above peaks from PVP could still be detected from the blue

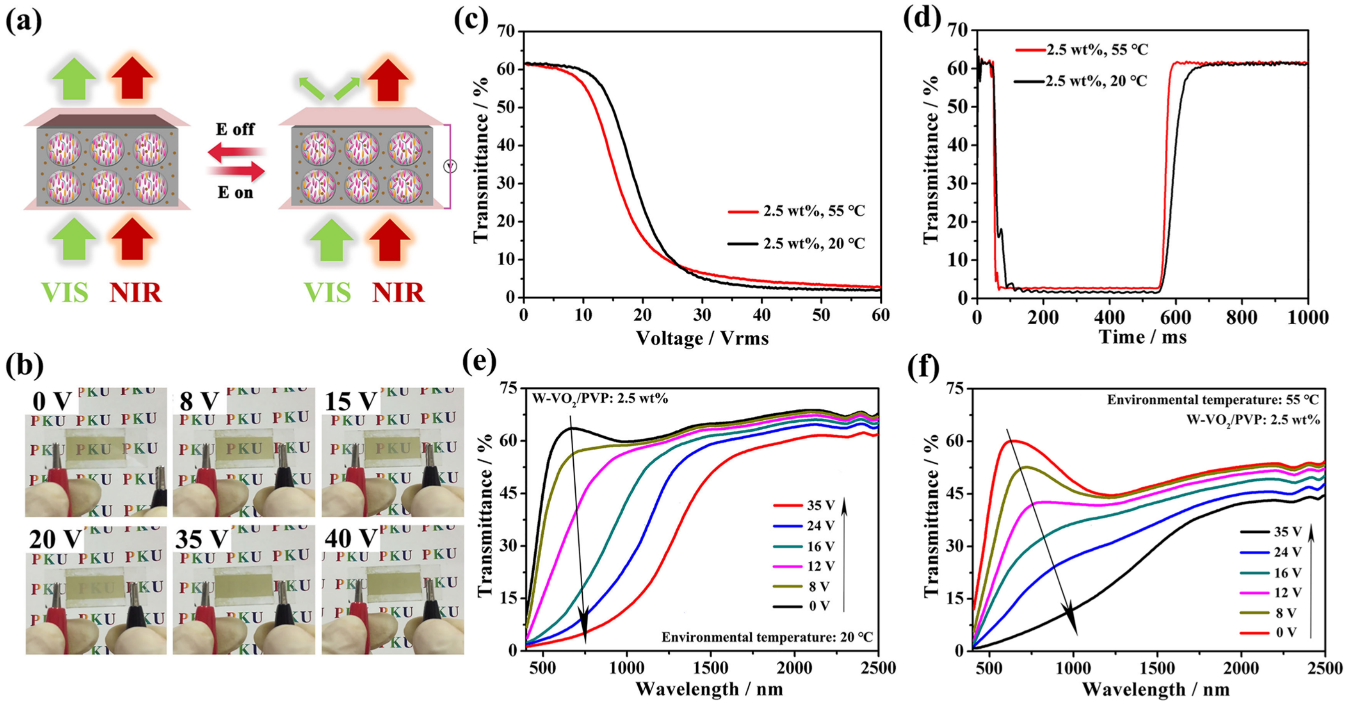

Figure 4. (a) Schematic illustration of the optical behaviors of the as-made film by applying or removing the vltage at low environmental temperature. (b) Digital photographs of the as-prepared films with 2.5 wt % W-VO_2 NCs incorporated at ~20 °C by applying different voltages. (c) Transmittance variations at 550 nm of the as-prepared film with 2.5 wt % W-VO_2 NCs by applying different voltages at 20 and 55 °C, respectively. (d) E-O response of the as-made film containing 2.5 wt % W-VO_2/PVP NCs at 20 and 55 °C, respectively. Vis/NIR transmittance spectra from 400 nm to ~2500 nm of the film with 2.5 wt % W-VO_2 NCs incorporated under the application of various voltages at (e) 20 °C and (f) 55 °C, respectively.

curve in the spectrum. However, the result of pristine W-VO_2 NCs (red curve) does not indicate the characteristic peaks of PVP, demonstrating the success of this PVP surface treatment process. These W-VO_2/PVP NCs can keep stable for at least one-month storage. By contrast, large precipitates could be observed for the pristine W-VO_2 NCs within a half day as shown in Figure 1f.

The as-made hybrid smart films containing W-VO_2 NCs were fabricated according to the stepwise polymerization strategy in Scheme 2. During the fabrication, we first constructed a polymer framework with a unique microstructure by using P-LCs as a template. After fully extracting P-LCs by dipping the film in cyclohexane, we used SEM to characterize the polymer framework within the film. As shown in Figure 2b, a porous microstructure of polymer matrix can be clearly seen from the overhead view. When flexible substrates are used, this porous polymer matrix not only provides excellent adhesive strength to the film, but also plays a key role in sustaining the film thickness and preventing the two substrates from being easily separated apart, as demonstrated in Figure S4. Moreover, from the side view in Figure 2c and d, homeotropically aligned polymer networks can be clearly seen within the P-LCs microdomains. Taking advantages of the polymerization induced phase separation and using P-LCs as a template, we successfully constructed a homogeneous microstructured polymer framework between the two substrates. Furthermore, after N-LCs were refilled into the film, the interactions between "the homeotropically oriented polymer networks" and "the N-LCs molecules" induced perpendicular alignment of the long molecular axis of LCs. Since the ordinary RI of N-LCs and the RI of polymer are well-matched, the as-made film exhibits a transparent state at the normal condition. Besides the phase-separated N-LCs/polymer microstructures between the substrates, as the W-VO_2/PVP NCs were homogeneously dispersed within the composite (Figure 1f), also confirmed by the SEM photograph in Figure S5, we can conclude that the as-made film processes a hybrid phase-separated micro–nanostructure.

Because of the sharp, first-order, and reversible MIT at 43.2 °C as well as the corresponding drastic changes in NIR optical properties of the as-synthesized W-VO_2 NCs, NIR light transmittance of the film can be passively modulated according to the temperature variations. We studied the NIR modulation performance of the as-prepared films with 2.5 and 5.0 wt % W-VO_2 NCs incorporated, respectively. It can be seen in Figure 3a and b that the variation of temperature does not affect the orientations of N-LCs because of the anchoring forces from HALPN. Thus, the film maintains transparency in visible region at no matter higher or lower temperature. However, a drastic change of NIR transmittance was observed at different environmental temperatures, as shown in Figure 3b. Specifically, during the heating process from 20 to 55 °C, the transmittance of NIR light dynamically decreased from 59.4% to 41.2% and 40.6% to 8.1% for the as-made films containing 2.5 and 5.0 wt % W-VO_2 NCs, respectively. Furthermore, this passively controllable NIR transmittance was confirmed by the vis/NIR spectra at different environment temperatures in Figure 3d and Figure S6, indicating an effective thermally switchable NIR light property.

Moreover, the visible light transmittance of the film can also be independently and dynamically regulated by the external voltages as shown in Figure 4a and b. The electro-optical (E-O) behaviors of the film at different temperatures were first investigated, as shown in Figure 4c and d. When the applied voltage increased from 0 to 35 V, the visible light transmittance at 550 nm of the film containing 2.5 wt % W-VO_2/PVP NCs gradually decreased from 60.8% to 1.3%, as shown in Figure 4c. This is because during the increase of applied voltages, the long molecule axis of N-LCs

DOI: 10.1021/acsami.7b11582
ACS Appl. Mater. Interfaces 2017, 9, 40810−40819

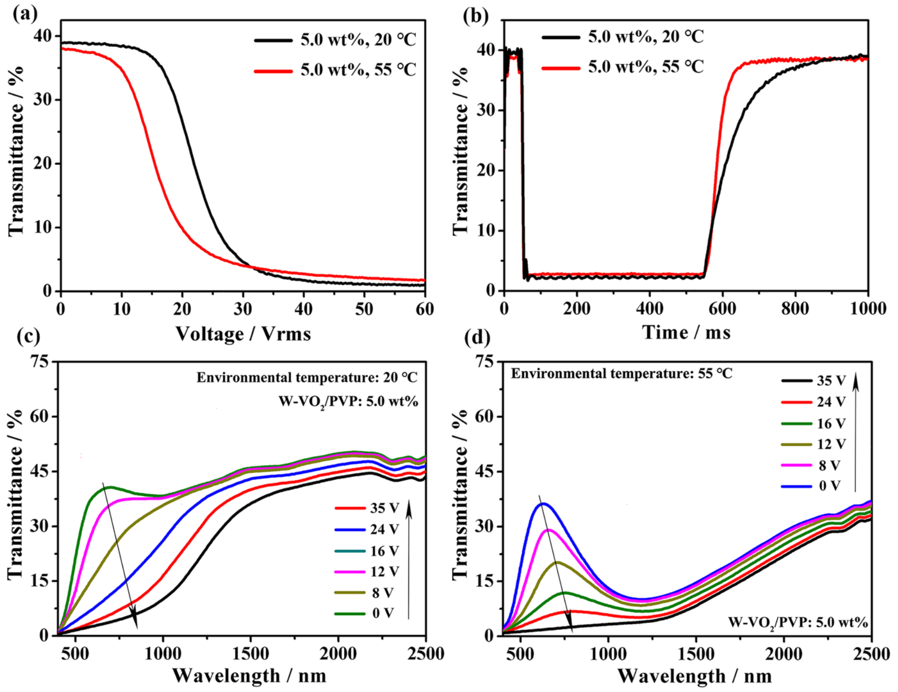

Figure 5. (a) Transmittance variations of the as-prepared film with 5.0 wt % W-VO_2 NCs by applying different voltage at 20 and 55 °C, respectively. (b) E-O response of the as-prepared film with 5.0 wt % W-VO_2/PVP NCs incorporated at 20 and 55 °C, respectively. Vis/NIR transmittance spectra from 400–2500 nm of the film with 5.0 wt % W-VO_2 NCs incorporated under the application of various voltages at (c) 20 °C and (d) 55 °C, respectively.

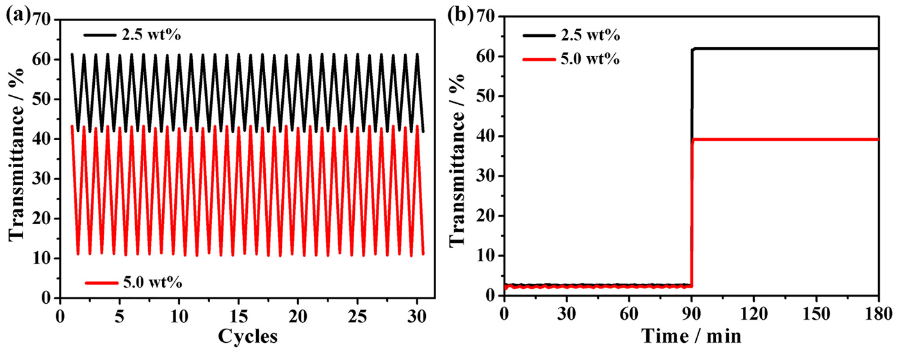

Figure 6. (a) Transmittance of the films containing 2.5 wt % (black) and 5.0 wt % (red) W-VO_2/PVP NCs at 1150 nm during the heating and cooling cycles. (b) Transmittance of the film containing 2.5 wt % (black) and 5.0 wt % (red) W-VO_2/PVP NCs at 550 nm during the voltage-on and voltage-off state, respectively.

tends to align parallelly to the direction of the electric field, generating a spatial variation of IR between the micro-LCs domains and the polymer. This spatially mismatched IR within the film strongly scatters the visible light and turns the film from a transparent to an opaque state. However, when the applied voltage was removed, induced by the molecule interaction from HALPN, the film could rapidly change from opaqueness to transparency within 84 ms as shown in Figure 4d. Also, it is noticeable that the driving and saturated voltages of the film at 55 °C were lower than those at 20 °C, and the response time of the film at 55 °C was faster than that at 20 °C. This improvement of E-O properties at higher temperature could be attributed to the fact that the viscosity of N-LCs was lower at higher temperature, making the N-LCs more easily to be driven by the electric field. Besides, it can be seen from Figure 5a and b that similar E-O properties were also observed from the as-prepared film with 5.0 wt % W-VO_2/PVP NCs incorporated.

Vis/NIR spectra of the films with 2.5 and 5.0 wt % of W-VO_2/PVP NCs incorporated further demonstrated the independent control of visible light in the films, as shown in Figures 4e and f and 5c and d, respectively. Obviously, below the phase transition temperature of W-VO_2 NCs, the visible light transmittance at 400 nm to ~800 nm dynamically decreased with the increase of the applied voltage, while the NIR light transmittance still maintained at relatively high level (Figures 4e and 5c) due to the fact that LC birefringence decreases as the wavelength increases.[48,49] Similarly, as the environmental temperature went above the phase transition temperature of W-VO_2 NCs, the NIR light transmittance in the film drastically decreased due to the reflective effects of W-VO_2 NCs, and the visible light transmittance could be still dynamically controlled by the applied external voltages (Figures 4f and 5d).

Furthermore, we tested the stability and the reversibility of the as-made films. In details, the thermal-optical stability of the films containing 2.5 and 5.0 wt % W-VO_2 NCs was measured by

DOI: 10.1021/acsami.7b11582
ACS Appl. Mater. Interfaces 2017, 9, 40810–40819

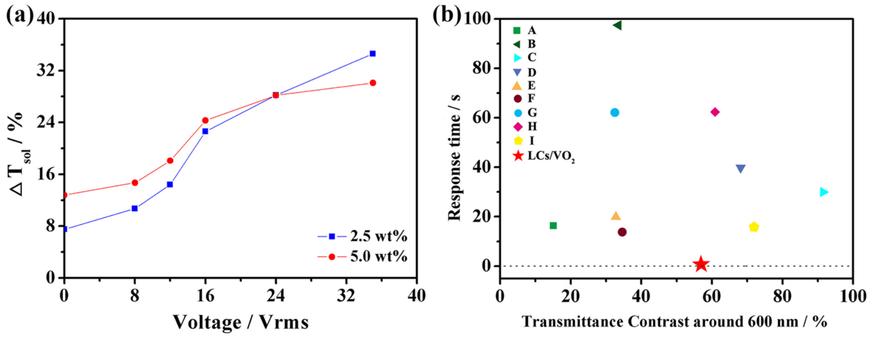

Figure 7. (a) ΔT_{sol} dependence of voltage of the film containing 2.5 wt % W-VO_2/PVP NCs and 5.0 wt % W-VO_2/PVP NCs. (b) Comparison of the response time and transmittance contrast of the as-made film containing 2.5 wt % W-VO_2/PVP NCs with other reported electrochromic materials; (A) PEDOT:PSS,[50] (B) poly(3,4-(2,2-diethyl propylenedioxy) thio-phene and FL dye, Br^- /Br^{3-}.[51] (C) Electrodeposition of WO_3 nanoparticles,[52] (D) hydrogel/transparent electrodes,[53] (E) V_2O_5/polymer composite,[54] (F) poly(aniline-N-butyl-sulfonate)/PEDOT/poly(3-hexylthiophene),[55] (G) PEDOT,[42] (H) poly((2,2-bis(2-ethylhexyloxymethyl)-propylene-1,3-dioxy)-3,4-thiophene-2,5-diyl) and poly(*N*-octadecyl-(propylene-1,3-dioxy)-3,4-pyrrole-2,5-diyl),[56] and (I) inkjet printed WO_3 nanoparticles.[57]

Table 1. Comparison of Optical Performances of As-Made Film with Previously Reported Hybrid Hydrogel/VO_2 Film

sample	ΔT_{sol}	T_{lum}	TC at 600 nm	response type		control mode	
				vis	NIR	vis	NIR
**hydrogel/VO_2**	~35%	82.1%	~45%	temperature		passive	
**LCs/VO_2**	34.6%	57.8%	58.9%	voltage	temperature	active	passive

subjecting the films to the heating−cooling cycles for 30 times, and the transmittances of the films at 1150 nm were recorded during the cycles, as shown in Figure 6a. The results show that the transmittances of the films containing 2.5 and 5.0 wt % W-VO_2 NCs at 20 and 55 °C are almost the same during the cycles, indicating an excellent thermal stability and reversibility. Moreover, to show the E-O stability of the films, we monitored the transmittances of the films at 550 nm during the voltage-on state (34 V) for 90 min and then recorded the transmittances for 90 min after removing the voltage, as shown in Figure 6b. Obviously, the films could maintain the opaqueness and transparency during the voltage-on and voltage-off state, respectively, showing good E-O stability.

Also, we compared the optical performances of the micro/nanohybrid film with standard electro-/thermo-chromic materials, and the results are summarized in Table S2 and Figure 7. If we only consider the thermo-optical properties of the film, the ΔT_{sol} and T_{lum} (20 °C) for the film containing 2.5 and 5.0 wt % W-VO_2/PVP NCs are 7.5%/11.6% and 57.8%/39.7%, respectively. These results are comparable with the current research communities in VO_2-based films as shown in Table S2. Moreover, the ΔT_{sol} of the film can be further enhanced by combining the electro-optical properties of the film. As shown in Figure 7a, by increasing the applied voltage from 0 to 34 V, the ΔT_{sol} for the film containing 2.5 wt % W-VO_2/PVP NCs can be dynamically enhanced from 7.5% to 34.6%, and for the film containing 5.0 wt % W-VO_2/PVP NCs, the ΔT_{sol} can be enhanced from 11.6% to 30.1%. We have also compared the transmittance contrast (TC, around 600 nm) and the response time (RT) of the as-made film with the current electrochromic materials as shown in Figure 7b. The TC for the as-made film is 58.9%, which is acceptable compared with other reported values. Noticeably, the RT for the film is only within 100 ms, which is much faster than the previous electrochromic or hydrogel materials. This is because the optical switching in LCs-based films is realized by the changes of LCs molecules orientations under the application of the voltage,[31] while for the electro- or thermo-chromic materials, it usually involves oxidation−reduction reactions or phase transition processes during the changes of optical properties.

Since the film in this work from a hybrid system enriched the current research community of smart films with separate control ability of both visible and NIR light, we further compared the optical performances of the as-made film with the previously reported hybrid hydrogel/VO_2 material,[29] which also exhibits a dual-band optical modulation ability. As shown in Table 1, the ΔT_{sol} values for both of them are almost the same, ~35% for the hydrogel/VO_2 and 34.6% for the as-made film in this work, respectively. The T_{lum} at 20 °C for the hydrogel/VO_2 is 24.3% higher than our material, which can be probably attributed to the different VO_2 contents in the two materials. However, because of the strong light-scattering effects of LCs in visible light region, the TC of our material is ~14% higher than that of the hydrogel/VO_2 material. Additionally, since hydrogel and VO_2 are both thermochromic materials, the transmittances of visible and NIR light in hydrogel/VO_2 are both passively controlled by environmental temperature, while ours shows a dual-responsive ability: the passage of visible light can be actively controlled by the external applied voltages and the NIR light can be passively controlled by environmental temperature.

4. CONCLUSIONS

In summary, we present a novel all-solution-processed hybrid micronano composite smart material that have four optical modulation modes to separately modulate the visible and NIR light transmittances according to the external applied voltage and environmental temperature, respectively. This dual-band optical modulation of the film was achieved by constructing a phase-separated polymer framework containing microsized N-LCs domains and W-VO_2 NCs. The film with 2.5 wt % W-VO_2 NCs exhibits transparency at normal condition Moreover, the passage of visible light at 550 nm can be reversibly regulated between

DOI: 10.1021/acsami.7b11582
ACS Appl. Mater. Interfaces 2017, 9, 40810−40819

60.8% and 1.3% by actively controlling the directions of N-LCs via external applied voltages. Also, the transmittance of NIR light can be modulated between 59.4% and 41.2% by the thermochromic behavior of W-VO_2 NCs during the MIT at 43.2 °C. Besides, the film also features easy all-solution processability, high mechanical strength, fast E-O response time, and long-term stability. This hybrid micronano material not only enlightens insights into engineering functional organic–inorganic matters, but also provides new opportunities for the next-generation smart window technology.

■ ASSOCIATED CONTENT

Supporting Information

The Supporting Information is available free of charge on the ACS Publications website at DOI: 10.1021/acsami.7b11582.

Chemicals structures and physical properties of some materials used; EDS and XPS results for W-VO_2 NCs; zeta potential of W-VO_2 NCs dispersed in water; shearing strength of as-prepared film with W-VO_2/PVP NCs incorporated; comparison of thermochromic performance of as-made film with previous results of VO_2-based films (PDF)

Movie showing that passage of visible light in film can be actively and dynamically controlled via applying different voltages (AVI)

■ AUTHOR INFORMATION

Corresponding Author

*E-mail: yanghuai@pku.edu.cn.

ORCID

Shumeng Guo: 0000-0002-0466-6382

Huai Yang: 0000-0002-3773-6666

Author Contributions

The manuscript was written through contributions of all authors. All authors have given approval to the final version of the manuscript.

Notes

The authors declare no competing financial interest.

■ ACKNOWLEDGMENTS

We appreciate the financial support from the National Natural Science Foundation of China (NSFC) (Grant Nos. 51573006, 51333001, 51573003, 51372029, and 51561135014).

■ REFERENCES

(1) Bell, L. E. Cooling, Heating, Generating Power, and Recovering Waste Heat with Thermoelectric Systems. *Science* **2008**, *321*, 1457–1461.

(2) Khandelwal, H.; Schenning, A. P. H. J.; Debije, M. G. Infrared Regulating Smart Window Based on Organic Materials. *Adv. Energy Mater.* **2017**, *7*, 1602209.

(3) *Buildings Energy Data Book, section 2.1.5*; U.S. Department of Energy, 2011. http://web.archive.org/web/20130216182348/http://buildingsdatabook.eren.doe.gov/TableView.aspx?table=2.1.5 (accessed July 5, 2017).

(4) Kim, H.; Kim, Y.; Kim, K. S.; Jeong, H. Y.; Jang, A. R.; Han, S. H.; Yoon, D. H.; Suh, K. S.; Shin, H. S.; Kim, T. Y.; Yang, W. S. Flexible Thermochromic Window Based on Hybridized VO_2/Graphene. *ACS Nano* **2013**, *7*, 5769–5776.

(5) Wang, J.; Zhang, L.; Yu, L.; Jiao, Z.; Xie, H.; Lou, X.; Sun, X. A Bi-Functional Device for Self-Powered Electrochromic Window and Self-Rechargeable Transparent Battery Applications. *Nat. Commun.* **2014**, *5*, 4921.

(6) Zheng, J.; Bao, S.; Jin, P. TiO_2(R)/VO_2(M)/TiO_2(A) Multilayer Film as Smart Window: Combination of Energy-Saving, Antifogging and Self-Cleaning Functions. *Nano Energy* **2015**, *11*, 136–145.

(7) Chen, F.; Ren, Y.; Guo, J.; Yan, F. Thermo-and Electro-Dual Responsive Poly (Ionic Liquid) Electrolyte Based Smart Windows. *Chem. Commun.* **2017**, *53*, 1595–1598.

(8) Yu, Z.; Yao, Y.; Yao, J.; Zhang, L.; Chen, Z.; Gao, Y.; et al. Transparent Wood Containing Cs_XWO_3 Nanoparticles for Heat-Shielding Window Applications. *J. Mater. Chem. A* **2017**, *5*, 6019–6024.

(9) Gao, Y.; Yao, W.; Sun, J.; Zhang, H.; Wang, Z.; Wang, L.; Yang, D.; Zhang, L.; Yang, H. A Novel Soft Matter Composite Material for Energy-Saving Smart Windows: From Preparation to Device Application. *J. Mater. Chem. A* **2015**, *3*, 10738–10746.

(10) Ge, D.; Lee, E.; Yang, L.; Cho, Y.; Li, M.; Gianola, D. S.; Yang, S. A Robust Smart Window: Reversibly Switching from High Transparency to Angle-Independent Structural Color Display. *Adv. Mater.* **2015**, *27*, 2489–2495.

(11) Shin, H.; Seo, S.; Park, C.; Na, J.; Han, M.; Kim, E. Energy Saving Electrochromic Windows from Bistable Low-HOMO Level Conjugated Polymers. *Energy Environ. Sci.* **2016**, *9*, 117–122.

(12) DeForest, N.; Shehabi, A.; O'Donnell, J.; Garcia, G.; Greenblatt, J.; Lee, E. S.; Selkowitz, S.; Milliron, D. J. United States Energy and CO_2 Savings Potential from Deployment Of Near-Infrared Electrochromic Window Glazings. *Build. Environ.* **2015**, *89*, 107–117.

(13) Shehabi, A.; DeForest, N.; McNeil, A.; Masanet, E.; Greenblatt, J.; Lee, E. S.; Masson, G.; Helms, B. A.; Milliron, D. J. US Energy Savings Potential from Dynamic Daylighting Control Glazings. *Energy Buildings* **2013**, *66*, 415–423.

(14) Wang, Y.; Runnerstrom, E. L.; Milliron, D. J. Switchable Materials for Smart Windows. *Annu. Rev. Chem. Biomol. Eng.* **2016**, *7*, 283–304.

(15) Runnerstrom, E. L.; Llordés, A.; Lounis, S. D.; Milliron, D. J. Nanostructured Electrochromic Smart Windows: Traditional Materials and NIR-Selective Plasmonic Nanocrystals. *Chem. Commun.* **2014**, *50*, 10555–10572.

(16) Baetens, R.; Jelle, B. P.; Gustavsen, A. Properties, Requirements and Possibilities of Smart Windows for Dynamic Daylight and Solar Energy Control in Buildings: A State-of-the-Art Review. *Sol. Energy Mater. Sol. Cells* **2010**, *94*, 87–105.

(17) Guo, S.; Liang, X.; Zhang, C.; Chen, M.; Shen, C.; Zhang, L.; Yuan, X.; He, B.; Yang, H. Preparation Of a Thermally Light-Transmittance-Controllable Film from a Coexistent System of Polymer-Dispersed and Polymer-Stabilized Liquid Crystals. *ACS Appl. Mater. Interfaces* **2017**, *9*, 2942–2947.

(18) Kim, J.; You, J.; Kim, B.; Park, T.; Kim, E. Solution Processable and Patternable Poly (3, 4-alkylenedioxythiophene)s for Large-Area Electrochromic Films. *Adv. Mater.* **2011**, *23*, 4168–4173.

(19) Beaujuge, P. M.; Ellinger, S.; Reynolds, J. R. The Donor-Acceptor Approach Allows a Black-to-Transmissive Switching Polymeric Electrochrome. *Nat. Mater.* **2008**, *7*, 795.

(20) Gao, Y.; Wang, S.; Kang, L.; Chen, Z.; Du, J.; Liu, X.; Luo, H.; Kanehira, M. VO_2-Sb: SnO_2 Composite Thermochromic Smart Glass Foil. *Energy Environ. Sci.* **2012**, *5*, 8234–8237.

(21) Gao, Y.; Wang, S.; Luo, H.; Dai, L.; Cao, C.; Liu, Y.; Chen, Z.; Kanehira, M. Enhanced Chemical Stability of VO_2 Nanoparticles by the Formation of SiO_2/VO_2 Core/Shell Structures and the Application to Transparent and Flexible VO_2-Based Composite Foils with Excellent Thermochromic Properties for Solar Heat Control. *Energy Environ. Sci.* **2012**, *5*, 6104–6110.

(22) Li, D.; Wu, G.; Gao, G.; Shen, J.; Huang, F. Ultrafast Coloring-Bleaching Performance of Nanoporous WO_3–SiO_2 Gasochromic Films Doped with Pd Catalyst. *ACS Appl. Mater. Interfaces* **2011**, *3*, 4573–4579.

(23) Feng, W.; Wu, G.; Gao, G. Ordered Mesoporous WO_3 Film with Outstanding Gasochromic Properties. *J. Mater. Chem. A* **2014**, *2*, 585–590.

(24) Raymo, F. M.; Tomasulo, M. Electron and Energy Transfer Modulation with Photochromic Switches. *Chem. Soc. Rev.* **2005**, *34*, 327–336.

(25) Ohko, Y.; Tatsuma, T.; Fujii, T.; Naoi, K.; Niwa, C.; Kubota, Y.; Fujishima, A. Multicolour Photochromism of TiO_2 Films Loaded with Silver Nanoparticles. *Nat. Mater.* **2003**, *2*, 29−31.
(26) Garcia, G.; Buonsanti, R.; Llordes, A.; Runnerstrom, E. L.; Bergerud, A.; Milliron, D. J. Near-Infrared Spectrally Selective Plasmonic Electrochromic Thin Films. *Adv. Opt. Mater.* **2013**, *1*, 215−220.
(27) Baetens, R.; Jelle, B. P.; Gustavsen, A. Properties, Requirements and Possibilities of Smart Windows for Dynamic Daylight and Solar Energy Control in Buildings: A State-of-the-Art Review. *Sol. Energy Mater. Sol. Cells* **2010**, *94*, 87−105.
(28) Llordés, A.; Garcia, G.; Gazquez, J.; Milliron, D. J. Tunable near-infrared and visible-light transmittance in nanocrystal-in-glass composites. *Nature* **2013**, *500*, 323.
(29) Zhou, Y.; Cai, Y.; Hu, X.; Long, Y. VO_2/Hydrogel Hybrid Nanothermochromic Material with Ultra-High Solar Modulation and Luminous Transmission. *J. Mater. Chem. A* **2015**, *3*, 1121−1126.
(30) Doane, J. W.; Golemme, A.; West, J. L.; Wu, B. G.; et al. Polymer Dispersed Liquid Crystals for Display Application. *Mol. Cryst. Liq. Cryst.* **1988**, *165*, 511−532.
(31) Higgins, D. A. Probing the Mesoscopic Chemical and Physical Properties of Polymer-Dispersed Liquid Crystals. *Adv. Mater.* **2000**, *12*, 251−264.
(32) Kim, M.; Park, K. J.; Seok, S.; Ok, J. M.; Jung, H. T.; Choe, J.; Kim, D. Fabrication of Microcapsules for Dye-Doped Polymer-Dispersed Liquid Crystal-Based Smart Windows. *ACS Appl. Mater. Interfaces* **2015**, *7*, 17904−17909.
(33) Broer, D. J.; Boven, J.; Mol, G. N.; Challa, G. In-Situ Photopolymerization of Oriented Liquid-Crystalline Acrylates, 3. Oriented Polymer Networks from a Mesogenic Diacrylate. *Makromol. Chem.* **1989**, *190*, 2255−2268.
(34) Gray, G. W.; Harrison, K. J.; Nash, J. A. New Family of Nematic Liquid Crystals for Displays. *Electron. Lett.* **1973**, *9*, 130−131.
(35) Chen, M.; Hu, W.; Liang, X.; Zou, C.; Li, F.; Zhang, L.; Chen, F.; Yang, H. A Facile All-Solution-Processed Surface with High Water Contact Angle and High Water Adhesive Force. *ACS Appl. Mater. Interfaces* **2017**, *9*, 23246−23254.
(36) Peng, Z.; Jiang, W.; Liu, H. Synthesis and Electrical Properties of Tungsten-Doped Vanadium Dioxide Nanopowders by Thermolysis. *J. Phys. Chem. C* **2007**, *111*, 1119−1122.
(37) Peng, Z.; Wang, Y.; Du, Y.; Lu, D.; Sun, D. Phase Transition and IR Properties of Tungsten-Doped Vanadium Dioxide Nanopowders. *J. Alloys Compd.* **2009**, *480*, 537−540.
(38) Zhang, W.; Zhang, L.; Liang, X.; Zhou, L.; Xiao, J.; Yu, L.; Li, F.; Cao, H.; Li, K.; Yang, Z.; Yang, H. Unconventional High-Performance Laser Protection System Based on Dichroic Dye-Doped Cholesteric Liquid Crystals. *Sci. Rep.* **2017**, *7*, 42955.
(39) Guo, J.; Cao, H.; Wei, J.; Zhang, D.; Liu, F.; Pan, G.; Zhao, D.; He, W.; Yang, H. Polymer Stabilized Liquid Crystal Films Reflecting both Right- and Left-Circularly Polarized Light. *Appl. Phys. Lett.* **2008**, *93*, 201901.
(40) Wyszecki, G.; Stiles, W. S. Color Science: Concepts and Methods. *Quantitative Data and Formulae*, 2nd ed.; Wiley: New York, 2000.
(41) Standard, A. S. T. M. *Standard Tables of Reference Solar Spectral Irradiances: Direct Normal and Hemispherical on a 37 Degree Tilted Surface*; American Society for Testing and Materials: Philadelphia, PA, 2012; Vol. *14*.
(42) Tran-Van, F.; Beouch, L.; Vidal, F.; Yammine, P.; Teyssié, D.; Chevrot, C. Self-Supported Semi-Interpenetrating Polymer Networks for New Design of Electrochromic Devices. *Electrochim. Acta* **2008**, *53*, 4336−4343.
(43) Gu, Q.; Falk, A.; Wu, J.; Ouyang, L.; Park, H. Current-Driven Phase Oscillation and Domain-Wall Propagation in $W_xV_{1-x}O_2$ Nanobeams. *Nano Lett.* **2007**, *7*, 363−366.
(44) Chae, B. G.; Kim, H. T.; Yun, S. J. Characteristics of W-and Ti-Doped VO_2 Thin Films Prepared by Sol-Gel Method. *Electrochem. Solid-State Lett.* **2008**, *11*, D53−D55.
(45) Manning, T. D.; Parkin, I. P.; Pemble, M. E.; Sheel, D.; Vernardou, D. Intelligent Window Coatings: Atmospheric Pressure Chemical Vapor Deposition of Tungsten-Doped Vanadium Dioxide. *Chem. Mater.* **2004**, *16*, 744−749.
(46) Stelzig, S. H.; Klapper, M.; Müllen, K. A Simple and Efficient Route to Transparent Nanocomposites. *Adv. Mater.* **2008**, *20*, 929−932.
(47) Graf, C.; Vossen, D. L. J.; Imhof, A.; van Blaaderen, A. A General Method to Coat Colloidal Particles with Silica. *Langmuir* **2003**, *19*, 6693−6700.
(48) Wu, S. T. Infrared Properties of Nematic Liquid Crystals: an Overview. *Opt. Eng.* **1987**, *26*, 262120.
(49) Li, W.; Yu, L.; He, W.; Yuan, X.; Zhao, D.; Huang, W.; Cao, H.; Yang, Z.; Yang, H. Effect of a Photopolymerizable Monomer Containing a Hydrogen Bond on Near-Infrared Radiation Transmittance of Nematic Liquid Crystal/Monomers Composites. *J. Phys. Chem. C* **2008**, *112*, 13739−13743.
(50) Mecerreyes, D.; Marcilla, R.; Ochoteco, E.; Grande, H.; Pomposo, J. A.; Vergaz, R.; Sánchez Pena, J. M. A Simplified All-Polymer Flexible Electrochromic Device. *Electrochim. Acta* **2004**, *49*, 3555−3559.
(51) Hsu, C. Y.; Lee, K. M.; Huang, J. H.; Justin Thomas, K. R.; Lin, J. T.; Ho, K. C. A Novel Photoelectrochromic Device with Dual Application Based on Poly (3, 4-Alkylenedioxythiophene) Thin Film and an Organic Dye. *J. Power Sources* **2008**, *185*, 1505−1508.
(52) Liu, L.; Layani, M.; Yellinek, S.; Kamyshny, A.; Ling, H.; Lee, P. S.; Magdassi, S.; Mandler, D. Nano to Nano" Electrodeposition of WO_3 Crystalline Nanoparticles for Electrochromic Coatings. *J. Mater. Chem. A* **2014**, *2*, 16224−16229.
(53) Zhou, Y.; Layani, M.; Boey, F. Y. C.; Sokolov, I.; Magdassi, S.; Long, Y. Electro-Thermochromic Devices Composed of Self-Assembled Transparent Electrodes and Hydrogels. *Adv. Mater. Technol.* **2016**, *1*, 1600069.
(54) Tritschler, U.; Beck, F.; Schlaad, H.; Colfen, H. Electrochromic Properties of Self-Organized Multifunctional V_2O_5-Polymer Hybrid Films. *J. Mater. Chem. C* **2015**, *3*, 950−954.
(55) Shin, H.; Kim, Y.; Bhuvana, T.; Lee, J.; Yang, X.; Park, C.; Kim, E. Color Combination of Conductive Polymers for Black Electrochromism. *ACS Appl. Mater. Interfaces* **2012**, *4*, 185−191.
(56) Knott, E. P.; Craig, M. R.; Liu, D. Y.; Babiarz, J. E.; Dyer, A. L.; Reynolds, J. R. A Minimally Coloured Dioxypyrrole Polymer as A Counter Electrode Material in Polymeric Electrochromic Window Devices. *J. Mater. Chem.* **2012**, *22*, 4953−4962.
(57) Layani, M.; Darmawan, P.; Foo, W. L.; Liu, L.; Kamyshny, A.; Mandler, D.; Magdassi, S.; Lee, P. S. Nanostructured Electrochromic Films by Inkjet Printing On Large Area and Flexible Transparent Silver Electrodes. *Nanoscale* **2014**, *6*, 4572−4576.

Visible and infrared optical modulation of PSLC smart films doped with ATO nanoparticles

Z. Zhang, R. Zhang, L. Xu, J. Li, L. Yang, Y. Yang, A. Bolshakov and J. Zhu, *Dalton Trans.*, 2021, **50**, 10033 **DOI:** 10.1039/D1DT01575C

To request permission to reproduce material from this article, please go to the Copyright Clearance Center request page.

If you are **an author contributing to an RSC publication, you do not need to request permission** provided correct acknowledgement is given.

If you are **the author of this article, you do not need to request permission to reproduce figures and diagrams** provided correct acknowledgement is given. If you want to reproduce the whole article in a third-party publication (excluding your thesis/dissertation for which permission is not required) please go to the Copyright Clearance Center request page.

Read more about how to correctly acknowledge RSC content.

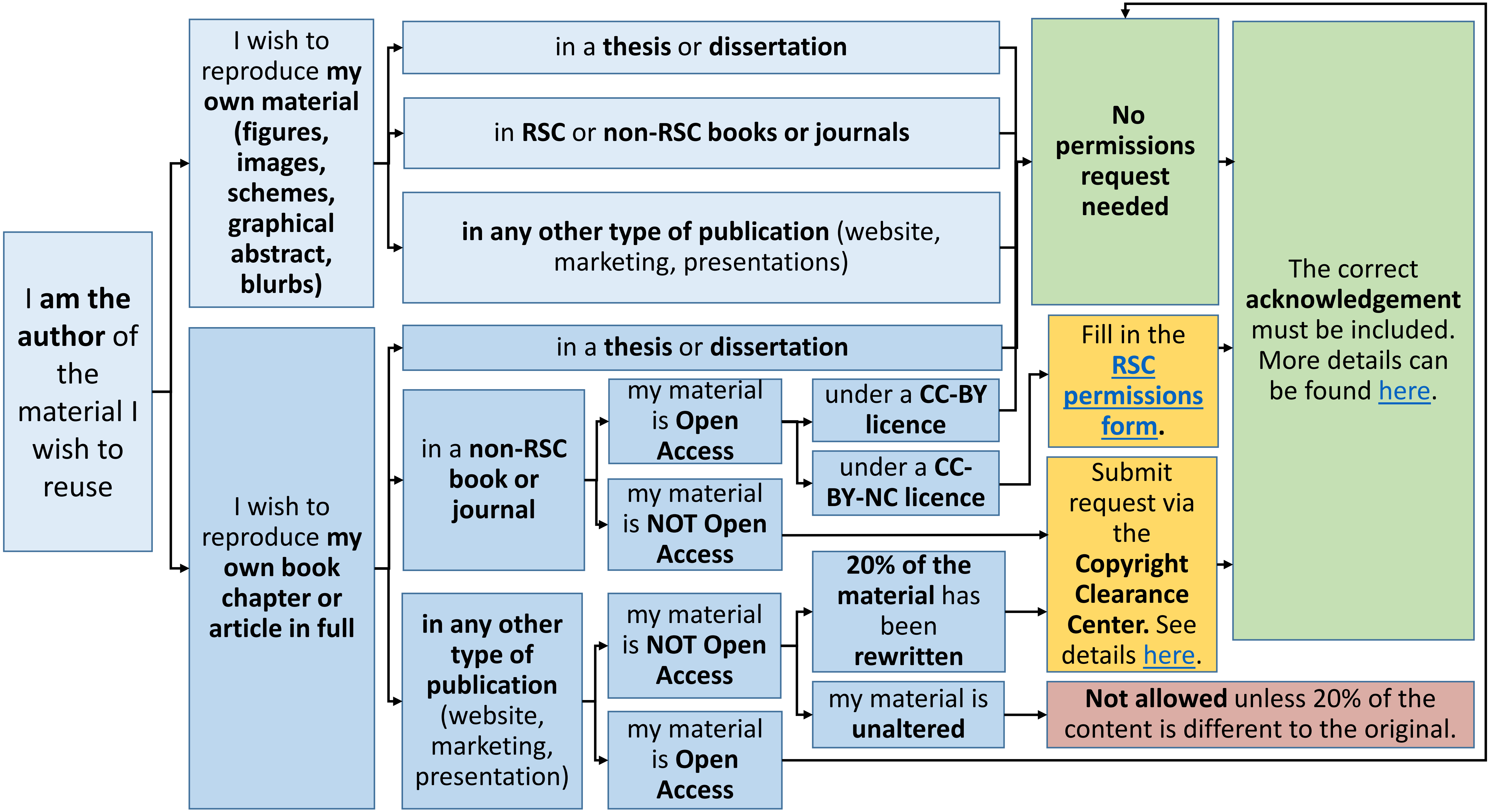
I am the author of the material I wish to reuse
I wish to reproduce my own material (figures, images, schemes, graphical abstract, blurbs)
in a thesis or dissertation
in RSC or non-RSC books or journals
in any other type of publication (website, marketing, presentations)
I wish to reproduce my own book chapter or article in full
in a thesis or dissertation
in a non-RSC book or journal
my material is Open Access
my material is NOT Open Access
under a CC-BY licence
under a CC-BY-NC licence
in any other type of publication (website, marketing, presentation)
my material is NOT Open Access
my material is Open Access
20% of the material has been rewritten
my material is unaltered
No permissions request needed
Fill in the RSC permissions form.
Submit request via the Copyright Clearance Center. See details here.
Not allowed unless 20% of the content is different to the original.
The correct acknowledgement must be included. More details can be found here.

Volume 50
Number 29
7 August 2021
Pages 9971-10312

Dalton Transactions

An international journal of inorganic chemistry

rsc.li/dalton

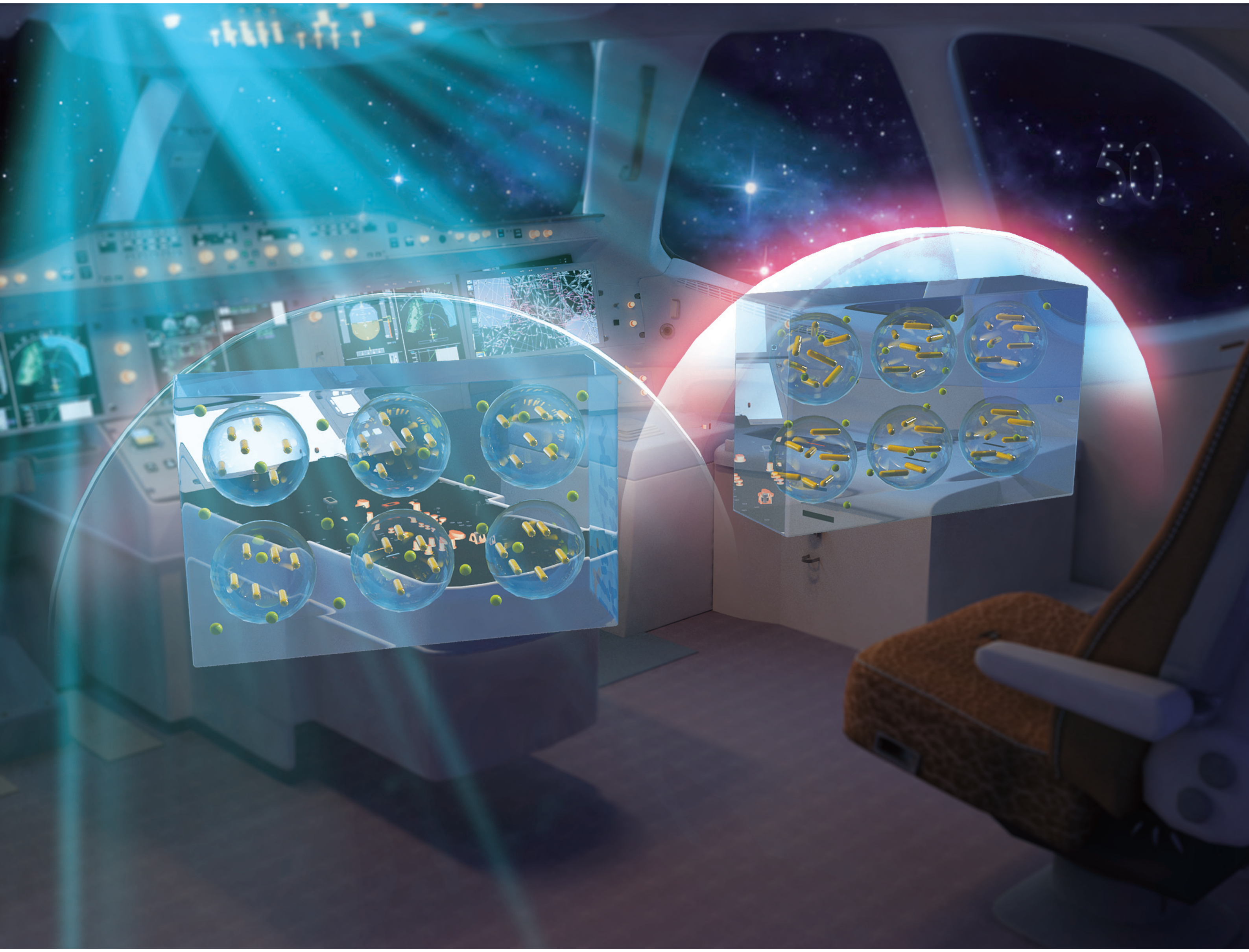

ISSN 1477-9226

ROYAL SOCIETY OF CHEMISTRY

PAPER
Jiaqi Zhu *et al.*
Visible and infrared optical modulation of PSLC smart films doped with ATO nanoparticles

50 Anniversary Volume

Dalton Transactions

PAPER

View Article Online
View Journal | View Issue

Cite this: *Dalton Trans.*, 2021, **50**, 10033

Visible and infrared optical modulation of PSLC smart films doped with ATO nanoparticles†

Zhibo Zhang,[a] Ruicong Zhang,[a] Liangge Xu,[a] Jiajun Li,[a] Lei Yang,[b] Yanan Yang,[c] Andrey Bolshakov[a] and Jiaqi Zhu *[a,d]

Received 15th May 2021,
Accepted 18th June 2021

DOI: 10.1039/d1dt01575c

rsc.li/dalton

In recent years, smart windows have been gradually applied and developed in the fields of environmental protection, energy management, smart camouflage and display. With the continuous upgrading of market demand, smart windows with both visible and infrared band modulation functions are the future development trends. However, the optical modulation of smart windows is usually limited in the visible light band (380–780 nm). In this paper, we report a novel smart window by doping antimony doped tin oxide (ATO) nanoparticles (NPs) into polymer stabilized liquid crystal (PSLC) films. These films have the widest waveband modulation function among those reported so far, covering the visible and infrared regions (380–5500 nm). The transmittance of the as-prepared smart films can be changed reversibly from highly transparent (78.5%) state to a strong light scattering (10%) state in the visible region. In addition, due to the localized surface plasmon resonance of ATO NPs, up to 80.7% of the infrared invisible light can be effectively shielded. The significance of this research is to provide theoretical and technical support for the broadband optical modulation of novel smart windows.

Published on 06 July 2021. Downloaded on 9/19/2022 9:14:43 AM.

Introduction

With increasing attention to environmental protection and energy conservation, great progress has been made in the rational use of energy and the research and development of energy-saving products. In recent years, smart windows have been gradually applied and developed in the fields of environmental protection, energy management, smart camouflage and display.[1–5] The commercial value has also expanded from the single market of buildings to automobiles and airplanes.[6,7] Polymer/liquid crystal is one of the most promising optical modulation materials because of its mature preparation process, fast response, low driving voltage and high contrast.[8–10] Generally, polymer/liquid crystal consists of micron sized birefringent liquid crystal droplets dispersed in other optically transparent and uniform polymer films.[11] The transparent state (ON state) and the scattering state (OFF state) of polymer/liquid crystal are controlled by an external electric field following the matching/mismatching of the refractive index of liquid crystal droplets and the polymer.[12] According to the polymer content, polymer/(liquid crystal) can be classified into polymer dispersed liquid crystal (PDLC, polymer content >20%) and polymer stabilized liquid crystal (PSLC, polymer content <20%).[13] PDLC has a lower OFF state transmittance and better film forming properties, while PSLC has a higher ON state transmittance and a faster response speed, so PSLC is more suitable for smart windows than PDLC.[14] The optical modulation of PSLC is always limited in the visible light band (380–780 nm), however, about 40% of the energy of sunlight comes from infrared light, and some light pollution problems also arise from the infrared band.[15] With the continuous upgrading of intelligent market demand, smart windows with both visible and infrared band modulation functions are the future development trends.[16–18]

Due to the localized surface plasmon resonance (LSPR) characteristics of inorganic semiconductor nanoparticles, (NPs) they have a good shielding effect on ultraviolet (UV) light and infrared light.[19–21] The particle size of the nanoparticles is smaller than the wavelength of the visible band, which cannot basically affect the transmittance of visible light. It is a potential method to improve the modulation of the infrared band by doping inorganic semiconductor nanoparticles into PSLC. Antimony doped tin oxide (ATO) is a kind of n-type semiconductor material formed by replacing Sn with Sb. Because of the good carrier concentration and lower cost compared to

[a]National Key Laboratory of Science and Technology on Advanced Composites in Special Environments, Harbin 150080, P. R. China. E-mail: zhujq@hit.edu.cn
[b]Center of Analysis Measurement, Harbin Institute of Technology, Harbin, 150001, P. R. China
[c]Key Laboratory of Photoelectronic/Electrophotonic Conversion Materials, Key Laboratory of Cluster Science, Ministry of Education of China, School of Chemistry and Chemical Engineering, Beijing Institute of Technology, Beijing 100081, P. R. China
[d]Key Laboratory of Micro-systems and Micro-structures Manufacturing, Ministry of Education, Harbin 150080, P. R. China

†Electronic supplementary information (ESI) available. See DOI: 10.1039/d1dt01575c

This journal is © The Royal Society of Chemistry 2021

Published on 06 July 2021. Downloaded on 9/19/2022 9:14:43 AM.

indium tin oxide (ITO), ATO NPs are widely used in transparent heat insulation coatings.[22–24] Besides, the conductivity of ATO NPs are expected to reduce the driving voltage of liquid crystal molecular orientation, further optimizing the electro-optical properties of PSLC.[25,26] In this paper, ATO NPs were doped into PSLC to develop a novel film, which can be used in smart windows and implement the broadband optical modulation of visible–infrared light.

Experiments and simulation

Materials and preparations

ATO NPs were synthesized by the sol–gel method.[27–29] The ethanol solution of 0.5 mol L^{-1} tin chloride ($SnCl_4$) and 0.5 mol L^{-1} antimony chloride ($SbCl_3$) were mixed at 343.15 K for 30 min. Ammonium hydroxide ($NH_3 \cdot H_2O$, 30%) was added dropwise to the mixture until the pH value of the mixture was 6. The resulting gel was centrifuged, washed several times with deionized water, dried at 353.15 K in a nitrogen environment for 12 h, and finally the dried gel was calcined at 973.15 K for 3 h to obtain ATO NPs. We investigated the PSLC properties of the ATO NPs with different mole ratios of Sb/Sn (Sb/Sn = 3/97, 5/95, 7/93, 10/90, 15/85, and 20/80) by adjusting the proportion of Sb during the initial feeding. The above raw materials were purchased from Sigma Aldrich Co., Llc., China.

GXP-6003 was used as the positive nematic mixed liquid crystal (n_0 = 1.524, n_e = 1.776, T_{NI} = 357.5K, and $\Delta\varepsilon$ = 14.1; Yantai Xianhua Chem-Tech Co., Ltd), and 3,5,5-trimethylhexyl acrylate (TMHA, J&K Scientific Ltd) and polyethylene glycol (600) diacrylate (PEGDA600, Aladdin Co.) were used as UV polymerizable monomers. Benzoin dimethyl ether (Irgacure651, Aladdin Co.) was used as the photoinitiator. A GXP-6003/TMHA/PEDGA600/Irgacure651 mixture was formed with the mass ratio of 80/15/4/1 and it was stirred at 353.15 K until the liquid crystal molecules were transformed into an isotropic state. Then a certain concentration of ATO ethanol dispersion was added to the liquid crystal mixture, and the mass fraction of ATO in the ATO/liquid crystal mixture was 0, 1 wt%, 3 wt%, 5 wt%, 7 wt%, and 10 wt%. The mixture was stirred at 333.15 K until all the ethanol volatilized. The LC cell (North Liquid Crystal Displays Research and Development Center, Co.) with a thickness of 20 μm was placed on a hot stage at 353.15 K, and the above mixture was dropped to the opening side of the LC cell. Capillary action makes the liquid crystal mixture automatically fill the gap of the cell. Finally, the filled cells were placed under the illumination of a UV light source (365 nm, 3.5 mW cm^{-2}) for 20 min to prepare PSLC films.

Measurements

The dispersion characteristics of ATO NPs in ethanol were analyzed using a Talos F200X transmission electron microscope (TEM, FEI Co.), and the elemental distribution of ATO was analyzed by mapping. The particle size distribution of ATO NPs was analyzed using a DynaPro NanoStar dynamic light scattering instrument (DLS, Wyatt Technology Co.). The crystal structure of ATO NPs was characterized by X'PERT X-ray diffraction (XRD, PANalytical Co.). The texture of the ATO-doped PSLC films was characterized using a WMP-6880 polarizing microscope (POM, SHANGHAI WUMO Optical Instrument Co., Ltd). The microstructure of the ATO-doped PSLC films was analyzed using a HELIOS NanoLab 600i field emission scanning electron microscope (FEI Co.). The typical element concentration of ATO in cleaning solution was determined by NexION 300X inductively coupled plasma mass spectrometry (ICP-MS, PerkinElmer Co.). The electro-optical properties of the ATO-doped PSLC films were tested using an LCT-5016 (North Liquid Crystal Displays Research and Development Center, Co.). The transmittance of the ATO-doped PSLC films in the UV-vis-NIR band was measured using a Lambda950 UV-vis-NIR spectrophotometer (PerkinElmer Co.) and the mid-infrared transmittance of the ATO-doped PSLC films was measured with a Fourier transform infrared spectrometer (FTIR, PE Co.). The three-dimensional cutting plane structure of the ATO-doped PSLC films was determined using an Xradia 520 Versa three-dimensional X-ray microscope (XRM, Carl Zeiss Co.).

Simulation methods

ATO semiconductor NPs may optimize the response performance of PSLC due to the LSPR and good conductivity. However, it is difficult to forecast the distribution of ATO NPs in PSLC under general conditions. In order to explore the influence of doping of ATO NPs on the threshold voltage (V_{th}) of PSLC, we established an equivalent circuit model of this system. The dispersion of ATO NPs in various ideal states is shown in Fig. 1a:

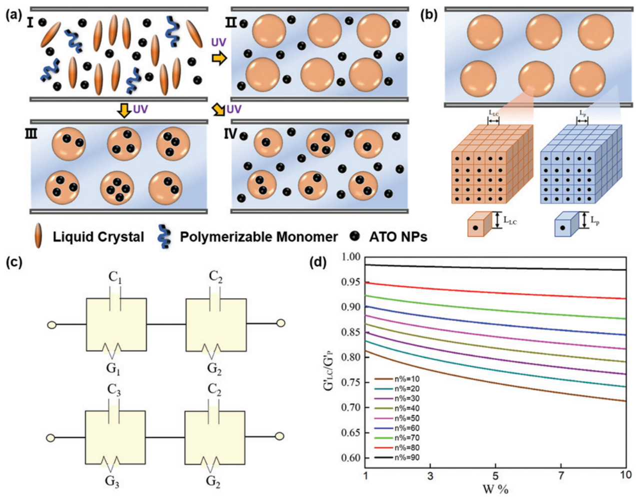

Fig. 1 The electric driving performance of ATO-doped PSLC by simulation. (a) Ideal dispersion model of ATO NPs in PSLC, (I) the mixture of liquid crystal, polymerizable monomer and ATO NPs, (II) ATO NPs concentrated in the polymer network area of PSLC after phase separation, (III) ATO NPs concentrated in the liquid crystal droplets, (IV) ATO NPs evenly distributed in the polymer network area and liquid crystal droplets. (b) Equivalent model of ATO-doped PSLC. (c) Equivalent circuit model, (I) ATO-doped LC and (II) ATO-doped polymer. (d) Variation trend of G'_{LC}/G'_{P} with ATO doping concentrations, where W% is the doping percentage of ATO NPs in PSLC and n% is the doping percentage of ATO NPs in the liquid crystal.

This journal is © The Royal Society of Chemistry 2021

Published on 06 July 2021. Downloaded on 9/19/2022 9:14:43 AM.

I shows the mixture of liquid crystal, polymerizable monomer and nanoparticles without phase separation, and II–IV show that the nanoparticles were only concentrated in the polymer network area, only concentrated in the liquid crystal droplets and evenly distributed in the polymer network area and liquid crystal droplets of PSLC after phase separation. On the basis of the above, we studied the equivalent circuit model of the ATO-doped liquid crystal and ATO-doped polymer, respectively. As shown in Fig. 1b, L_{LC}, L_p and a represent the side lengths of the liquid crystal cube, polymer cube and ATO NP cube, respectively,[30] and each polymer and liquid crystal equivalent cube containing ATO NPs can be represented as the equivalent circuit shown in Fig. 1c, where C_1, C_2 and C_3 are the capacitances of the liquid crystal, ATO NPs and the polymer, respectively, and G_1, G_2 and G_3 are the conductivities of the liquid crystal, ATO NPs and the polymer, respectively. After a series of calculations, we introduced a positive correlation between the equivalent conductance (the equivalent conductivities of the ATO-doped liquid crystal and ATO-doped polymer are G'_{LC} and G'_p, respectively) and V_{th}, judging the trends of V_{th} of PSLC according to the G'_{LC}/G'_p ratio at different ATO doping concentrations. For the detailed calculation process refer to the ESI.†

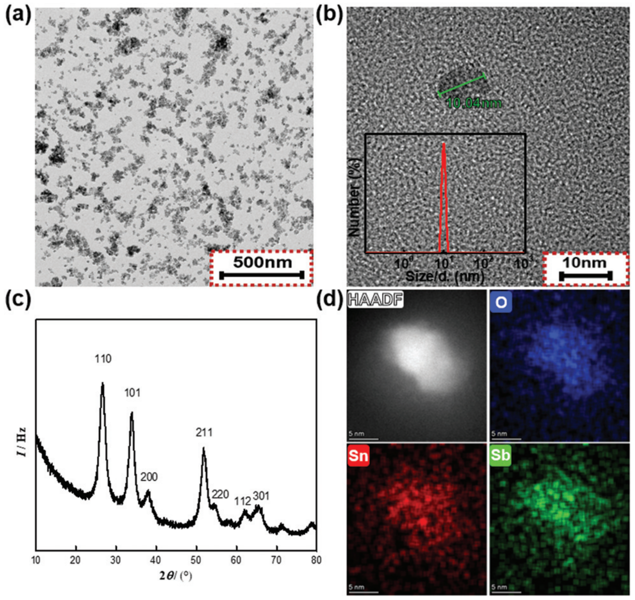

Fig. 2 (a) TEM image of ATO NPs in ethanol. (b) TEM image of the typical size of ATO NPs and DLS measurement of ATO NPs in ethanol with a concentration of 5.0 wt%. (c) XRD spectrum of ATO NPs. (d) EDS-HAADF measurement of ATO NPs (Sn/Sb = 90/10).

Results and discussion

The simulation results of V_{th} optimization by ATO NPs of ATO-doped PSLC are shown in Fig. 1d. It can be seen that the G'_{LC}/G'_p ratio decreases with the increase of doping percentage (W%, from eqn (S3)†) of ATO NPs, and the decrease rate of ATO NPs concentrated in the polymer network area is faster than that in liquid crystal droplets. According to eqn (S2†), since other parameters remain unchanged, the relation $V_{th} \propto G'_{LC}/G'_p$ is deduced. Therefore, we confirm that the addition of ATO NPs significantly reduces the threshold voltage of PSLC. When the doping percentage of ATO NPs reaches 10 wt% in PSLC, and 90% of these ATO NPs being distributed in the polymer network area, the G'_{LC}/G'_p ratio of PSLC decreases most significantly compared to undoped ones, reaching the maximum is 12.4%.

The transmission electron microscopy (TEM) images of the ATO NPs dispersed in ethanol are shown in Fig. 2a and b. It was found that ATO NPs showed no obvious aggregation phenomenon, indicating good dispersion. The average particle size of ATO NPs was 10 nm and dynamic light scattering (DLS) measurement of the ATO NPs showed a hydrodynamic diameter of 10 nm ± 1.2 nm. It was found that ATO NPs prepared by the sol–gel method in this paper had a uniform particle size. The XRD analysis of ATO NPs showed the characteristic diffraction peaks of ATO NPs (Fig. 2c). The as-prepared ATO NPs showed the rutile structure of SnO_2, and the diffraction peaks were broadened, $d[110] = 0.33373$, $d[101] = 0.26393$, and $d[211] = 0.17620$. It was found that Sb does not form second crystalline phases, such as Sb_2O_3 and Sb_2O_5. All the antimony ions enter the SnO_2 lattice instead of tin ions. Therefore, the sol–gel method achieves a better composite doping. Another key issue of particular concern is that whether the ATO NPs synthesized by the sol–gel method can maintain the content and proportion of the Sn and Sb elements. The results of EDS-HAADF measurement (Fig. 2d) and EDS SPEC measurement (Fig. S1†) showed that the content of Sn/Sb in ATO nanoparticles was strictly controlled.

The polarizing microscopic (POM) images of PSLC with different ATO NP doping concentrations are shown in Fig. 3. With the increase of ATO NP doping concentration, the size of liquid crystal droplets in PSLC enlarge, however, the size uniformity of PSLC decreases. This indicates that ATO nanoparticles can enter into the liquid crystal droplets during the phase separation process, thus affecting the segregation of the liquid crystal and polymer molecules. The POM images of PSLC before and after doping with ATO NPs were observed at 50× magnification. It was found that after the voltage reached a certain amplitude, the liquid crystal molecules were aligned along the electric field. A bright state in the case of a parallel polarizer and a dark black state in the case of a vertical polarizer were observed. Taking the 3 wt% ATO doping concentration as the typical doping concentration, it was found that the doped ATO did not affect the orientation of the liquid crystal molecules of PSLC in the ON state.

The PSLC samples were immersed in cyclohexane at room temperature for two days to remove the liquid crystals, and the micromorphology of the polymer network was observed with a scanning electron microscope (SEM). As shown in Fig. 4a–Fig. f, with the increase of ATO NPs doping concentration, the polymer network becomes more and more sparse, the mesh becomes larger and irregularly distributed, and the phenomenon of large mesh nesting small mesh occurs. The reason is that the incorporation of ATO NPs affects the phase separation

Published on 06 July 2021. Downloaded on 9/19/2022 9:14:43 AM.

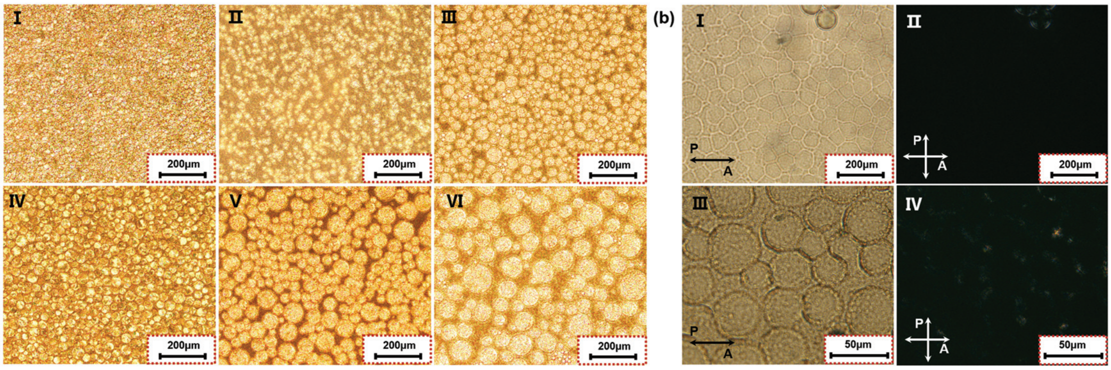

Fig. 3 (a) POM 10× image of PSLC in the OFF state. (I) undoped, (II) 1 wt%, (III) 3 wt%, (IV) 5 wt%, (V) 7 wt%, (VI) 10 wt%. (b) POM 50× image of PSLC. (I) ON state parallel polarization image of undoped, (II) ON state orthogonal polarization image of undoped, (III) ON state parallel polarization image of 3 wt% doped, (IV) ON state orthogonal polarization image of 3 wt% doped.

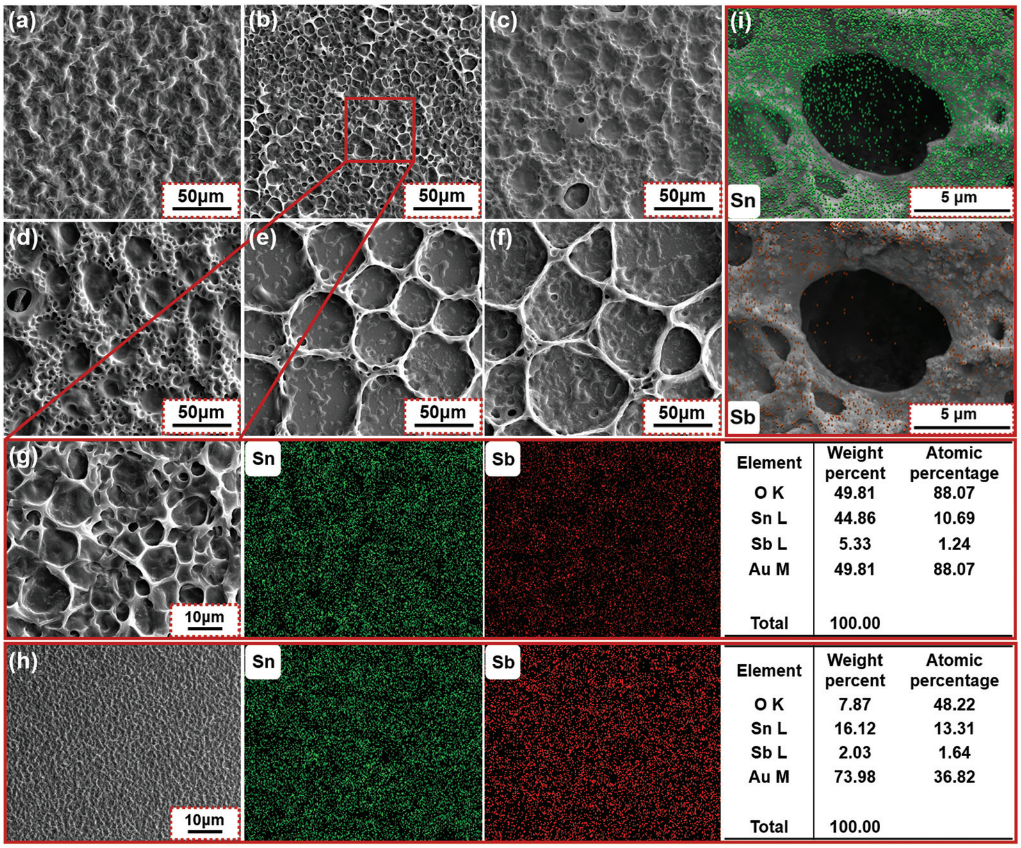

Fig. 4 Microstructure of the polymer network (LC removed) by SEM. (a) Undoped, (b) 1 wt%, (c) 3 wt%, (d) 5 wt%, (e) 7 wt%, (f) 10 wt%. (g) EDS measurement of the polymer network. (h) EDS measurement of ATO-doped PSLC (LC not removed). (i) EDS measurement of the polymer wall around a typical liquid crystal droplet.

rate of PSLC, the liquid crystals and polymerizable monomers have little time to separate. The polymer pores gradually become larger, and the polymer wall of large-size droplets is thickened. The microstructure obtained by SEM is consistent with that obtained with a polarizing microscope. On the other hand, the non-uniform network structure makes the electro-optical performance of PSLC complex, which needs further research. The EDS analysis results are shown in Fig. 4g–i. It can be seen from Fig. 4g that a large number of Sn and Sb elements are distributed in the polymer network. Comparing the percentages of Sn and Sb elements in PSLC before (Fig. 4h) and after the removal of liquid crystals, the percentage of ATO NPs in the polymer network increased significantly after the removal of liquid crystals, and the proportion of ATO NPs in

 This journal is © The Royal Society of Chemistry 2021

Published on 06 July 2021. Downloaded on 9/19/2022 9:14:43 AM.

the polymer network did not decrease with the loss of liquid crystals. In order to eliminate the influence of the deposition of ATO NPs initially in liquid crystal droplets on the EDS results, we took out the PSLC film immersed in cyclohexane and washed it several times with unused cyclohexane. The solution collected after washing was tested by ICP-MS until there were no Sn and Sb elements. So we can infer that ATO NPs were transferred to the polymer network more during the process of phase separation, and the proportion of ATO NPs in the polymer network increased. This may be due to the polymerization of monomers into a network structure with a higher density and viscosity during the process of phase separation, which is easier to bind ATO nanoparticles. Fig. 4i shows the selected polymer wall around a typical liquid crystal droplet, with the enrichment of Sn and Sb, which is consistent with the above conclusion.

Due to the LSPR, ATO NPs have a certain absorption effect on the infrared wavelength, but usually the LSPR absorption band of ATO NPs is very narrow, which shows a symmetric/asymmetric line shape according to the distribution of doped particles.[31] It is an ideal state for a PSLC smart window to maintain high transmittance of visible light and broadband absorption of infrared light at the same time. Since the LSPR wavelength of ATO NPs is closely related to the electron density, it can be adjusted by changing the doping amount of Sb. We prepared ATO NPs with different mole ratios of Sb/Sn (Sb/Sn = 3/97, 5/95, 7/93, 10/90, 15/85, and 20/80) to study the effect of the Sb/Sn doping ratio on the LSPR properties of ATO NPs. Fig. 5a I shows the NIR absorption spectra of ATO NPs with different doping amounts of Sb. ATO NPs showed clear LSPR peaks from 1500 nm to 2500 nm in the NIR region, and experienced a gradual red shift with the increase of the doping amount of Sb from 1% to 10%. Based on the abovementioned NIR absorption spectra of ATO NPs, we mixed ATO with different Sb doping amounts, and finally obtained a mixture with broadband NIR absorption characteristics, as shown in Fig. 5a II. The visible and near-infrared light modulation properties of the PSLC films doped with different concentrations of ATO NPs in the ON state (V = 30 V) and the OFF state (V = 0 V) were systematically studied using a UV-vis-NIR spectrophotometer. Due to the strong absorption characteristics of ATO NPs in the near-infrared region, upon addition of ATO NPs, the near-infrared shielding performance of the PSLC films is enhanced. We define the inhibition rate as

$$\text{Inhibition rate} = (T_{\text{undoped PSLC}} - T_{\text{ATO-doped PSLC}})/T_{\text{undoped PSLC}} \quad (1)$$

where $T_{\text{undoped PSLC}}$ is the transmittance of the undoped PSLC, and $T_{\text{ATO-doped PSLC}}$ is the transmittance of the ATO-doped PSLC. When the mass fraction of ATO NPs is 7 wt%, the maximum inhibition rate of the PSLC films on NIR radiation can reach 38.3% from 1500 nm to 2500 nm in the ON state (Fig. 5b). Through the spectral display in the OFF state, when the concentrations of ATO NPs are 1 wt% and 3 wt%, the overall transmittance decreases. With the increase of the doping concentration, the visible transmittance increases, and the infrared transmittance remains low (Fig. 5c). These results show that the appropriate concentration of doping is conducive to the uniform distribution of the size and position of the liquid crystal droplets, resulting in the improvement of the optical contrast of PSLC. With the increase of the doping concentration of ATO NPs, the size of the liquid crystal droplets increases gradually, while the size uniformity decreases and destroys the contrast of PSLC, which is consistent with the results of the above POM and SEM measurements.

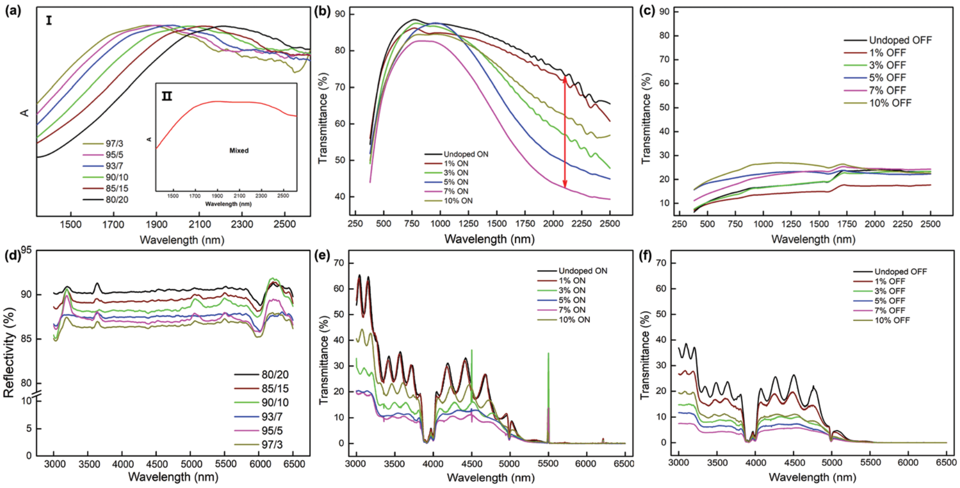

Fig. 5 (a) NIR absorption spectra variations of ATO NPs with different Sb/Sn mole ratios (I) and broadband absorption by mixing ATO NPs with different Sb/Sn mole ratios (II). NIR transmission spectra of PSLC in the ON state (b) and OFF state (c) with different doping concentrations of ATO NPs. (d) Distinctive mid-infrared reflectivity of ATO NPs with different Sb/Sn mole ratios. Mid-infrared transmission spectra of PSLC in the ON state (e) and OFF state (f) with different doping concentrations of ATO NPs.

Published on 06 July 2021. Downloaded on 9/19/2022 9:14:43 AM.

According to Drude–Lorentz free electron theory, with the increase of the light wavelength, the shielding effect of semiconductor nanoparticles will change from absorption to reflection. Fig. 5d shows that the reflectivity of ATO NPs with different doping amounts of Sb is more than 85% in the range of 3000 nm to 6500 nm, showing an excellent reflection effect. This makes the ATO-doped PSLC films with NPs to still maintain a good shielding efficiency in the mid-infrared region of 3000 nm to 6500 nm. Due to the fact that birefringence of LC decreases with increase in wavelength, the effect of scattering in the mid-infrared region is poor, which results in the decrease of the transmittance of PSLC in the OFF state in the mid-infrared region, which is significantly less than those in the visible and near infrared regions. After doping with ATO NPs, the maximum inhibition rate was 62.3% in the ON state, and 76% in the OFF state, when the mass fraction was 7 wt%, as shown in Fig. 5e and f. Combining the efficiencies of NIR and mid infrared inhibition, we concluded that the infrared inhibition rate of the ATO doped PSLC films can reach 48.1% in the ON state and 57.4% in the OFF state. By comparing before and after discoloration, we found that the maximum infrared inhibition rate can reach up to 80.7% (the maximum transmittance curve compared with the minimum transmittance curve). Doping with ATO NPs obviously optimizes the infrared modulation ability of PSLC.

However, by observing the trends of vis-NIR and mid infrared spectra, it can be found that when the doping concentration is further increased (up to 10 wt%), the shielding efficiencies of NIR and mid infrared are significantly reduced, and the visible light transmittance is not effectively improved while the infrared transmittance increases. A decrease in the infrared shielding performance and visible transparency is mainly due to the poor dispersion of excessive ATO NPs in PSLC, which leads to the formation of large aggregates. Once this phenomenon occurs, it will not only have adverse effects on the transparent state of the film due to Rayleigh scattering and Mie scattering,[31] but also significantly reduce the surface electron density of ATO particles, thus reducing the intensity of LSPR in the infrared region.

Fig. 6 shows the photoelectric performance of the PSLC films doped with different concentrations of ATO NPs. With the increase of ATO NPs, the ON state transmittance of PSLC decreases first and then increases, but the OFF state transmittance increases as a whole. This is due to the aggregation of ATO NPs and a decrease in size uniformity of liquid crystal droplets. ATO NPs also affect the distribution of the liquid crystal molecular director to a certain extent, and hinder the alignment consistency of liquid crystal molecules under an electric field. Above all they lead to a decrease in the PSLC scattering intensity, so the optical contrast of PSLC increases first and then decreases. However, the decrease of contrast is acceptable. When the mass fraction of ATO NPs is 3 wt%, the contrast only decreases by 0.1. In the aspect of response performance, V_{th} is according to equation S1. It can be seen from Fig. 4 that most of the ATO NPs are trapped in the polymer matrix instead of diffusing with LC due to an increase in the

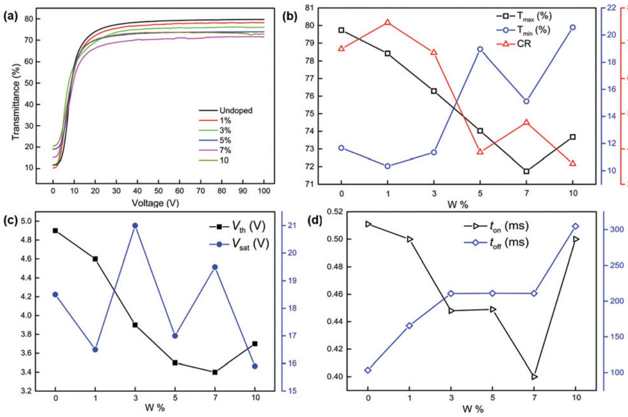

Fig. 6 The photoelectric performance of ATO-doped PSLC with different doping concentrations of ATO NPs. (a) T–V curves of PSLC films. (b) T_{max}, T_{min} and CR of PSLC films. (c) V_{th} and V_{sat} of PSLC films. (d) t_{on} and t_{off} of PSLC films.

medium viscosity during photopolymerization. This increases the conductivity of the polymer matrix because of the good conductivity of semiconductor ATO NPs. In this case, the local field acting on the droplet is given by[32]

$$E_{LC} = E_{appl}\left(\frac{3\sigma_{p+ATO}}{2\sigma_{p+ATO} + \sigma_{LC}}\right) \quad (2)$$

where, we assume an isolated spherical LC droplet in a polymer which is in a uniform electric field, E_{LC} is the field yielded in a single droplet with the application of electrostatic boundary conditions, E_{appl} is the field in the polymer, σ_{p+ATO} is the composite conductivity of the polymer matrix doped with ATO, and σ_{LC} is the conductivity of LC. After calculation, it was found that when $\sigma_{LC} > 0$, E_{LC} increases with the increase of σ_{p+ATO}. An increase in local equivalent voltage leads to lower V_{th} for orientation of the liquid crystal. The remaining small amount of ATO NPs dispersed in LC droplets will increase the dielectric anisotropy ($\Delta\varepsilon$) of LC, which will further reduce the V_{th} value of PSLC. The above results are consistent with the simulation results. The saturation voltage (V_{sat}) explains the degree of difficulty of liquid crystal molecules to achieve a uniform orientation along the external field. The main factors affecting V_{sat} are $\Delta\varepsilon$ and the size of LC droplets, both of which are negatively correlated with V_{sat}. Generally, the trend of V_{sat} is similar to that of V_{th}, but it can be seen from Fig. 1c that with the increase of ATO doping concentration, V_{sat} of PSLC fluctuates up and down. The results show that the size uniformity of liquid crystal droplets is seriously affected by excessive ATO NP doping, and the droplet size becomes the main factor affecting V_{sat}. The response time (t_{on}) and recovery time (t_{off}) of PSLC films are defined as:[33]

$$t_{on} = \frac{\gamma}{\Delta\varepsilon \times E^2 + \dfrac{K(l^2-1)}{a^2}} \quad (3)$$

$$t_{off} = \frac{\gamma a^2}{K(\omega^2-1)} \quad (4)$$

This journal is © The Royal Society of Chemistry 2021

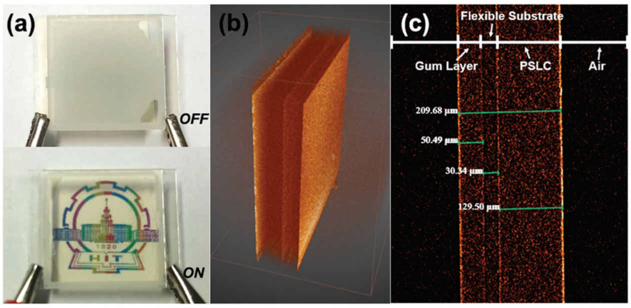

Fig. 7 (a) Digital photographs of the ATO-doped PSLC smart window in the OFF and ON states. (b) Three dimensional XRM image of the ATO-doped PSLC film on a flexible substrate. (c) Cross sectional XRM image of the ATO-doped PSLC film on a flexible substrate.

where γ is the viscosity coefficient of LC, $\Delta\varepsilon$ is the dielectric anisotropy of LC, E is the strength of the applied electric field, a is the semi major axis of the LC droplet, K is the effective elastic constant and ω is the length diameter ratio of the elongated droplet. An increase in $\Delta\varepsilon$ and the droplet size can significantly reduce the response time of PSLC. Meanwhile, an increase in the LC droplet size causes a decrease in interfacial anchoring force to the LC molecule, resulting in an increase of the recovery time.[34,35]

Fig. 7a shows the digital photographs of an ATO-doped PSLC smart window in the OFF and ON states, and the 'HIT' logo can be clearly seen in the ON state. As ATO NPs hardly affect the visible light, the PSLC smart window is still milky white after coloring. We also transferred the film onto a flexible substrate (such as ITO-PET and ITO-PEN) by roll-to-roll coating, which strictly controlled the thickness of the film and the uniformity of the interface between the film and substrates (Fig. 7b and c). We verified this process by the XRM test, which showed that ATO NPs did not destroy the uniformity and film-forming property of PSLC films, providing a new idea for the smart films with infrared modulation.

Conclusions

To sum up, we have presented a novel composite film for smart windows, which has broadband optical modulation function covering both visible light and infrared light. ATO NPs with uniform particle size distribution were prepared by the sol–gel method. After the procedures of dispersion, blending and UV induced phase separation, we doped the PSLC films with ATO NPs. The transmittance of the films can change reversibly between 78.5% and 10% in the visible region, and they can shield up to 80.7% of the infrared radiation without affecting the visible transmittance. ATO NPs also reduce the threshold voltage and optimize the response performance of PSLC. In addition, the films can be transferred onto flexible substrates, which show great potential for applications in the fields of multi curvature smart windows and flexible and wearable electronics. It also provides new ideas for the development of hybrid functional materials and multi-responsive materials.

Conflicts of interest

The authors declare that they have no known competing financial interest or personal relationships that could have appeared to influence the work reported in this paper.

Acknowledgements

This work was supported by the following grants: the National Natural Science Foundation for Distinguished Young Scholars of China (Grant No. 51625201), the Key Project of National Natural Science Foundation of China (Grant No. 52032004), and the Open Fund of Key Laboratory (Grant No. JZX7Y201911SY008601).

References

1 C. Meng, E. Chen, L. Wang, S. Tang, M. Tseng, J. Guo, Y. Ye, Q. F. Yan and H. Kwok, *Opt. Express*, 2019, **27**, 13098–13107.
2 H. N. Kim and S. Yang, *Adv. Funct. Mater.*, 2019, **30**, 1902597.
3 E. Castellón and D. Levy, *Smart Windows Based on Liquid Crystal Dispersions: Materials, Synthesis, Characterization, Applications, Transparent Conductive Materials*, 2018.
4 J. Yang, X. Zhang, X. Zhang, L. Wang, W. Feng and Q. Li, *Adv. Mater.*, 2021, **33**, 2004754.
5 W. Ling, H. K. Bisoyi, Z. Zheng, K. G. Gutierrez-Cuevas and L. Quan, *Mater. Today*, 2017, **20**, 230.
6 L. Gao and B. A. Bryan, *Nature*, 2017, **544**, 217–222.
7 J. Zheng, S. Bao and P. Jin, *Nano Energy*, 2015, **11**, 136–145.
8 C.-Y. Li, X. Wang, X. Liang, J. Sun, C.-X. Li, S.-F. Zhang, L.-Y. Zhang, H.-Q. Zhang and H. Yang, *Crystals*, 2019, **9**, 282.
9 T. Kajiyama, H. Kikuchi and A. Takahara, 1992.
10 N. Kawatsuki and H. Ono, *Chem. Lett.*, 1995, **1995**, 333–334.
11 S. Bronnikov, S. Kostromin and V. Zuev, *J. Macromol. Sci., Part B: Phys.*, 2013, **52**, 1718–1735.
12 I. Dierking, *Adv. Mater.*, 2010, **12**, 167–181.
13 S. Guo, X. Liang, H. Zhang, W. Shen, C. Li, X. Wang, C. Zhang, L. Zhang and H. Yang, *Liq. Cryst.*, 2018, **45**, 1854–1860.
14 G. P. Montgomery, G. W. Smith and N. A. Vaz, *Polymer-Dispersed Liquid Crystal Films, Liquid Crystalline and Mesomorphic Polymers*, 1994.
15 P. M. Beaujuge, S. Ellinger and J. R. Reynolds, *Nat. Mater.*, 2008, **7**, 795–799.
16 H. Khandelwal, A. P. H. J. Schenning and M. G. Debije, *Adv. Energy Mater.*, 2017, **7**, 1602209.
17 P. Jeevanandam, R. S. Mulukutla, M. Phillips, S. Chaudhuri, L. E. Erickson and K. J. Klabunde, *J. Phys. Chem. C*, 2007, **111**, 1912–1918.

Published on 06 July 2021. Downloaded on 9/19/2022 9:14:43 AM.

Published on 06 July 2021. Downloaded on 9/19/2022 9:14:43 AM.

18 L. Wang, K. G. Gutierrez-Cuevas, H. K. Bisoyi, J. Xiang, G. Singh, R. S. Zola, S. Kumar, O. D. Lavrentovich, A. Urbas and Q. Li, *Chem. Commun.*, 2015, **51**, 15039–15042.
19 Q. Tan, G. Yu, Y. Liao, B. Hu and X. Zhang, *Colloid Polym. Sci.*, 2014, **292**, 3233–3241.
20 G. Garcia, R. Buonsanti, E. L. Runnerstrom, R. J. Mendelsberg, A. Llordes, A. Anders, T. J. Richardson and D. J. Milliron, *Nano Lett.*, 2011, **11**, 4415–4420.
21 R. Gao, H. Xiong, R. Li, C. Hou, Y. Li, Q. Zhang and H. Wang, *Adv. Funct. Mater.*, 2018, 817–829.
22 N. Li, Q. Meng and N. Zhang, *Particuology*, 2014, **17**, 49–53.
23 L. Wang, J. Hang, L. Shi, X. Sun and F. Xu, *Mater. Lett.*, 2012, **87**, 35–38.
24 S. I. Choi, K. M. Nam, K. Bo, P. Won and J. T. Park, *Chem. Mater.*, 2008, **20**, 2609–2611.
25 M. Zhang, J. Zheng, K. Gui, K. Wang, C. Guo, X. Wei and S. Zhuang, *Appl. Opt.*, 2013, **52**, 7411–7418.
26 M. Jamil, F. Ahmad, J. T. Rhee and Y. J. Jeon, *Current Science*, 2011, **101**, 1544–1552.
27 D. L. Zhang, Z. B. Deng, L. Y. Chen and J. B. Zhang, *Key Eng. Mater.*, 2007, **336–338**, 750–753.
28 T. D. Senguttuvan and L. K. Malhotra, *Thin Solid Films*, 1996, **289**, 22–28.
29 Y. J. Lin and C. J. Wu, *Surf. Coat. Technol.*, 1997, **88**, 239–247.
30 Y. Sakai, N. Nishida, H. Shiraki, Y. Shiraishi, T. Miyama, N. Toshima and S. Kobayashi, *Mol. Cryst. Liq. Cryst.*, 2011, **441**, 143–152.
31 X. Liang, S. Guo, M. Chen, C. Li, Q. Wang, C. Zou, C. Zhang, L. Zhang, S. Guo and H. Yang, *Mater. Horiz.*, 2017, **4**, 878–884.
32 S. K. Shriyan and A. K Fonte Cc Hio, *Opt. Express*, 2010, **18**, 24842.
33 B.-G. Wu, J. H. Erdmann and J. W. Doane, *Liq. Cryst.*, 1989, **5**, 1453–1465.
34 S. Okushima and T. Kawakatsu, *Phys. Rev. E*, 2017, **96**, 052704.
35 J. Zhou, D. M. Collard, J. O. Park and M. J. Srinivasarao, *J. Am. Chem. Soc.*, 2002, **124**, 9980–9981.

This journal is © The Royal Society of Chemistry 2021
